# The Time Scale of Electronic Resonance in Oxidized DNA as Modulated by Solvent Response: An MD/QM-MM Study

**DOI:** 10.3390/molecules26185497

**Published:** 2021-09-10

**Authors:** Alessandro Landi, Amedeo Capobianco, Andrea Peluso

**Affiliations:** Dipartimento di Chimica e Biologia “A. Zambelli”, Università di Salerno, Via Giovanni Paolo II, 132, I-84084 Fisciano, SA, Italy; alelandi1@unisa.it (A.L.); apeluso@unisa.it (A.P.)

**Keywords:** DNA oxidation, DNA hole transfer, DNA, molecular dynamics, quantum dynamics, electron transfer, charge transfer, quantum coherence

## Abstract

The time needed to establish electronic resonant conditions for charge transfer in oxidized DNA has been evaluated by molecular dynamics simulations followed by QM/MM computations which include counterions and a realistic solvation shell. The solvent response is predicted to take *ca.* 800–1000 ps to bring two guanine sites into resonance, a range of values in reasonable agreement with the estimate previously obtained by a kinetic model able to correctly reproduce the observed yield ratios of oxidative damage for several sequences of oxidized DNA.

## 1. Introduction

Hole transport along one-electron oxidized DNA has been deeply investigated in the last decades [1,2,3,4,5], primarily for its biological role in mutagenesis, carcinogenesis, and ageing [6,7], and also because of its attractiveness for molecular electronics applications [8,9,10,11]. Indeed, owing to its unique redox and self assembling properties, DNA could be potentially used as an active material or a templating agent in the fabrication of biocompatible and biodegradable devices [12,13].

Hole transfer (HT) usually ends up on a guanine (G) site where the oxidative damage is preferentially observed, because guanine is the most prone to oxidation by removal of a single electron among natural occurring nucleobases. Furthermore, consecutive stacked guanines give rise to deeper hole-traps due to their lower hole site energy with respect to a single G [14,15]. DNA tracts made of adjacent adenines (A) are known to act as shuttle sites greatly favoring the hole transport, because adenine possesses a relatively low ionization energy and promotes intense stacking interactions which are able to stabilize the positive charge through the formation of delocalized polarons [16,17,18,19,20,21]. On the opposite, pyrimidine nucleobases (thymine, T, cytosine, C) act as barrier sites for hole transport due to their higher oxidation free energy [22].

Experimental evidence gathered in last years has shown that the dynamics of hole transfer in DNA is largely dependent on the specific sequence of DNA bases, being modulated by the different hole site energies and electronic coupling of nucleobases [15,19,23,24,25]. The kinetics of HT is characterized by two distinct regimes [26,27]: A short-range one with an exponential decay of hole-transfer rates as a function of the donor-acceptor (DA) distance, and a long-range regime, where HT rates exhibit a very weak distance dependence. Those results have usually been rationalized in terms of two different mechanisms: single step coherent hole tunneling (super-exchange or flickering resonance) for short DA distances and incoherent multistep hopping for long-range hole transport [28,29,30,31,32,33].

**Figure 1:**
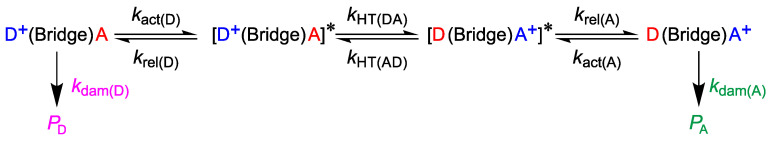
The kinetic scheme for HT in DNA. D${}^{+}$(Bridge)A and D(Bridge)A${}^{+}$ indicate the initial and the final state, giving rise to damaged products $P_{D}$ and $P_{A}$. [D${}^{+}$(Bridge)A]* and [D(Bridge)A${}^{+}$]* denote the ensembles of structures in which the hole donor and acceptor are in vibronic resonance with each other, so that $k_{\mathrm{HT}}\left(\mathrm{DA}\right)=k_{\mathrm{HT}}\left(\mathrm{AD}\right)$; $k_{\mathrm{HT}}$ has been computed by resolving the time dependent Schrödinger equation. $k_{\mathrm{dam}}$’s and $k_{\mathrm{rel}}$’s have been inferred from experimental data; no direct information is available for $k_{\mathrm{act}}$’s.

Recently, we proposed a unifying mechanism of hole transport in DNA, accounting for both short- and long-range regimes [34,35]. Our approach takes into account the manifold of fast coherent elementary electron-transfer processes which occur when the donor, i.e., the site where the hole has been injected and the acceptor, i.e., the site where the charge is eventually localized are brought into vibronic degeneracy by the response of solvent molecules and counterions. In detail, Figure 1, the proposed mechanism consists of four steps: (i) an activation step which brings a donor and the acceptor sites into resonance; (ii) an elementary electron transfer between resonant donor and acceptor groups; (iii) relaxation of non-equilibrium species (including the environment) to their minimum energy configurations; (iv) formation of oxidative damage products. In Figure 1, D${}^{+}$(Bridge)A and D(Bridge)A${}^{+}$ indicate the minimum energy structures with the charge localized on the donor (D) and the acceptor (A) groups; [D${}^{+}$(Bridge)A]* and [D(Bridge)A${}^{+}$]* denote the ensembles of structures in which D and A are in vibronic resonance, and $P_{D}$ and $P_{A}$ are the products of oxidative damage occurring at the D and A sites.

$k_{\mathrm{act}}$’s (step 1 and the reverse of step 3) are the rates for the activation stage that accounts for the time needed to bring [D${}^{+}$(Bridge)A]* and [D(Bridge)A${}^{+}$]* into degeneracy, condition achieved by collisions and environmental motions. An Arrhenius-like dependence with temperature is assumed to hold:

$$k_{\mathrm{act}}\left(\mathrm{DA}\right)=k_{\mathrm{act}}^{0}\left(T\right)\times \exp\left(-\frac{\Delta E_{\mathrm{DA}}}{k_{B}T}\right),$$

where $\Delta E_{\mathrm{DA}}$ is the in situ electronic hole site energy difference between donor and acceptor and $k_{\mathrm{act}}^{0}\left(T\right)$ is the rate constant for bringing into electronic resonance two sites possessing the same hole site energy.

Step 3 and the reverse of step 1 take into account the solvent response to a non-equilibrium charge distribution of the solute; pump-probe experiments in water solutions showed that solvent relaxation occurs in a few tens of femtoseconds, so that we set $k_{\mathrm{rel}}\left(D\right)=k_{\mathrm{rel}}\left(A\right)=10^{13}$ s${}^{-1}$ [36,37]. Because, in actual experiments, the hole is injected at G and the damage is observed at (G)${}_{n}$ tracts, $k_{\mathrm{dam}}$ have been set as the rates of deprotonation of G$\cdot^{+}$ and GG$\cdot^{+}$ radicals, i.e., $1\times 10^{7}$ and $3\times 10^{6}$ s${}^{-1}$, respectively [7,38]. Deprotonation of oxidized purine nucleobases inside DNA has been frequently observed [39,40] and it is commonly accepted as the first step of formation of the products of oxidative damage occurring at G sites [41,42,43].

The $k_{\mathrm{HT}}$’s (step 2) were computed by quantum dynamics simulations of hole transfer considering the DNA sequence in its minimum-energy configuration, inasmuch as solvent configuration does not alter tunneling rates in resonance conditions. Because the hole donor and acceptor sites are in vibronic resonance with each other, $k_{\mathrm{HT}}\left(\mathrm{DA}\right)=k_{\mathrm{HT}}\left(\mathrm{AD}\right)$.

With the above parameters and a proper setting for $k_{\mathrm{act}}$ (see below), the distribution of oxidative damages predicted by the multistep mechanism of Figure 1 was found to be in excellent agreement with experimental observations for both the 5${}^{\prime }$-GGG(T)${}_{n}$G-3${}^{\prime }$ sequences studied by Giese [26] and most of the oligonucleotides investigated by Schuster and coworkers in [44]. Notably, our mechanism allows for reconciling apparently discordant experimental observations. The (A)${}_{4}$ tract in double stranded (ds) DNA is a paradigmatic example. Indeed, (A)${}_{4}$ was found to act as a shuttle site in 5${}^{\prime }$-GGG(T)${}_{4}$G-3${}^{\prime }$ double stranded (ds) DNA where the charge is injected at G-3${}^{\prime }$ and the damage is mostly observed at the GGG site. On the opposite, (A)${}_{4}$ proved to be not effective for the hole transport in 5${}^{\prime }$-${}^{8}$GTGTG(T)${}_{4}$GTGTGG-3${}^{\prime }$ (${}^{8}$G denotes 8-oxo-7,8-dihydroguanine) where the charge initially generated at the G-3${}^{\prime }$ of the GG unit yields a damage mainly localized at the 5${}^{\prime }$-G of the same GG unit, instead of migrating to ${}^{8}$G which is a deeper trap than GG and even GGG. Our mechanism is able to rationalize both experimental facts. The (A)${}_{4}$ tract is *per se* an efficient bridge, however the latter sequence contains several G sites possessing close hole energies, so that interference among probability amplitudes pertaining to indistinguishable hole paths has to be taken into account. In detail, for 5${}^{\prime }$-${}^{8}$GTGTG(T)${}_{4}$GTGTGG-3${}^{\prime }$ several nucleobases can be brought in resonance with the donor site, so that different indistinguishable paths all contribute to hole localization on the observed trap site, mainly the 5${}^{\prime }$-G of the GG tract [35].

A further advantage of the present model is that of accounting for solvent effects, thus providing absolute rates and yields of oxidative damage for hole transfer in solution.

In previous work, $k_{\mathrm{act}}$ was taken as an adjustable parameter in the kinetic model. In order to compare theoretical predictions with the experimental results, the set of ordinary differential equations (ODEs) of the kinetic scheme of Figure 1 was solved to compute the yield ratios of damaged products. Numerical resolutions of the set of ODEs demonstrated that the experimental yield ratios are compatible with the experimental outcomes only with $k_{\mathrm{act}}$ of the order of $10^{10}$ s${}^{-1}$. Unfortunately, that parameter has no experimental counterpart, because no direct information about the time needed to bring two sites of DNA into resonance is available. Hence, a further investigation about the reliability of the inferred value for $k_{\mathrm{act}}$ appears to be a needed task. Interestingly, the resolution of the ODE equations has shown that the optimum value for $k_{\mathrm{act}}$ is almost independent of the nature and the length of the specific DNA sequence, indeed a single value, $k_{\mathrm{act}}=2\times 10^{10}$ s${}^{-1}$ was found to ensure an excellent agreement between predicted and observed damaged product ratios for all the DNA sequences investigated in [35]. That observation suggests that a useful analysis of the timescale of the activation step of HT could be initially limited to a single sequence. Therefore, herein we have carried out a preliminary study based on classical molecular dynamics (MD) simulations and QM/MM computations aimed at estimating the order of magnitude of the time needed to bring the G and GGG sites of double stranded 5${}^{\prime }$-GGGTG-3${}^{\prime }$, the simplest among previously studied DNA sequences, into vibronic resonance.

## 2. Computational Details

The starting geometry of the ds 5${}^{\prime }$-GGGTG-3${}^{\prime }$ DNA nucleotide was generated by using the 3DNA package [45] with the standard parameters of calf thymus B-DNA. The geometry of the 3${}^{\prime }$ terminal G was replaced with the equilibrium geometry of G${}^{+}$, i.e., G in its cationic form. The geometry of G${}^{+}$ was optimized at the B3LYP/6-311++G(d,p) level of theory with the unrestricted formalism. Solvent (water) effects were included via the polarizable continuum model (PCM) [46]. MD simulations for the GGGTG${}^{+}$ sequence were carried out by using the AMBER 18 suite of programs adopting the OL15 force field [47,48]. Explicit water molecules were treated by using the TIP3P force field [49]. An equilibrated box (truncated octahedron, radius = 22 Å) consisting of ≈15,000 water molecules was used, corresponding to a *ca*. 4 mM DNA. Na${}^{+}$ counterions were added to balance the negative charges of the phosphate groups; the positive charge of the DNA double strand was neutralized by adding a Cl${}^{-}$ ion [50]. Each Na${}^{+}$ ion was initially placed in the OPO plane of each phosphate unit, equidistant from formally charged oxygen atoms. The O–Na${}^{+}$ distance was set at 14 Å, in such a way that no solute atom is closer than 10 Å to any box side, see [51]. That distance was chosen after carrying out a few preliminary tests, showing that for lower O–Na${}^{+}$ distances, only the diffusion of Na${}^{+}$ ions in the bulk was observed, without achieving properly converged simulations. That is a subtle point, because although it has now been well ascertained that starting position and mobility of ions are relevant parameters, strongly influencing the function and the structure of nucleic acids, modeling those effects in MD simulations is still a challenging task [52] and has indeed been termed as “one of Achilles heels of atomistic MD simulations of RNA” [53]. Difficulties of simulating ions stem from force-field approximations, use of small sizes of the simulation boxes, and issues with the periodic boundary conditions [54,55,56].

Pending further studies, the ‘addions’ facility of the Amber suite, based on the minimization of the Coulombic potential evaluated on a grid, was adopted to place the Cl${}^{-}$ ion.

The charges used for G${}^{+}$ in MD simulations were obtained by summing the atomic charges internally stored in Amber for guanine [57] to the difference of the atomic RESP charge of G${}^{+}$ with the corresponding ones of G [58]. The particle mesh Ewald (PME) method for the treatment of the long-range electrostatics and the SHAKE algorithm (with an integration time step of 2 fs) for restraining the length of chemical bonds involving hydrogen atoms were used in all simulations [59,60]. A cut-off distance of 24 Å was used for non-bonding interactions. Harmonic restraints (k=50 kcal mol${}^{-1}$/Å${}^{2}$) were imposed to G${}^{+}$-3${}^{\prime }$ residue in order to keep its geometry as close as possible to the starting geometry of G${}^{+}$ during all simulations (*vide infra*).

An initial minimization of the water box and ions was performed by keeping the DNA strand fixed, followed by a further geometry optimization of the whole system. Geometry optimization was followed by an equilibration at constant volume from 0 to 300 K in 20 ps (steps of 2 fs). Longer equilibration times (up to 1 ns) were also considered, but they resulted in no appreciable differences in the subsequent MD simulations. Molecular dynamics were carried out on a 2 ns time scale adopting an NPT ensemble, at 300 K. Because we are not concerned here with the problem of sampling all the configurations, 2 ns appears to be a well suited time, certainly longer with respect to the expected HT time scale. Furthermore, internal base-pair opening events leading to DNA denaturation are estimated to occur in *ca.* 1–10 ms, far longer than time scale of hole transport in oxidized DNA [21,61].

For each production simulation (time step 2 fs), snapshots were retrieved every 0.5 ps by using the PTRAJ module of the Amber package. For each snapshot, two single-point two-layer ONIOM QM/MM calculations [62] have been carried out, in which the globally neutral 5${}^{\prime }$-GGGTG-3${}^{\prime }$ double strand has been considered. In the former ONIOM computation, only the G-3${}^{\prime }$ nucleobase was included in the QM region; in the latter, only the 5${}^{\prime }$-GGG nucleobase step was included in the QM region. Hole site energies of the G-3${}^{\prime }$ and 5${}^{\prime }$-GGG units were estimated in the framework of the vertical approximation, by adopting Koopman’s theorem and evaluating the energy of the Kohn–Sham HOMO of the G and GGG moieties [63].

Dangling bonds due to the breaking of the covalent bonds between the C9 atom of G and the C1${}^{\prime }$ atom of deoxyribose were saturated by hydrogen atoms [64]. The QM layer was treated at the density functional level of theory by using the APFD functional in conjunction with the 6-31G* basis set [65]. The MM layer composed of the remaining nucleobases, the sugar-phosphate backbone, the surrounding ions (Na${}^{+}$ and Cl${}^{-}$), and the water molecules was treated at the AMBER/TIP3P MM level. The electron embedding scheme, in which the electron density of the QM layer is polarized by the MM part was adopted throughout. ONIOM computations were carried out by using the Gaussian 09 package [66].

**Figure 2:**
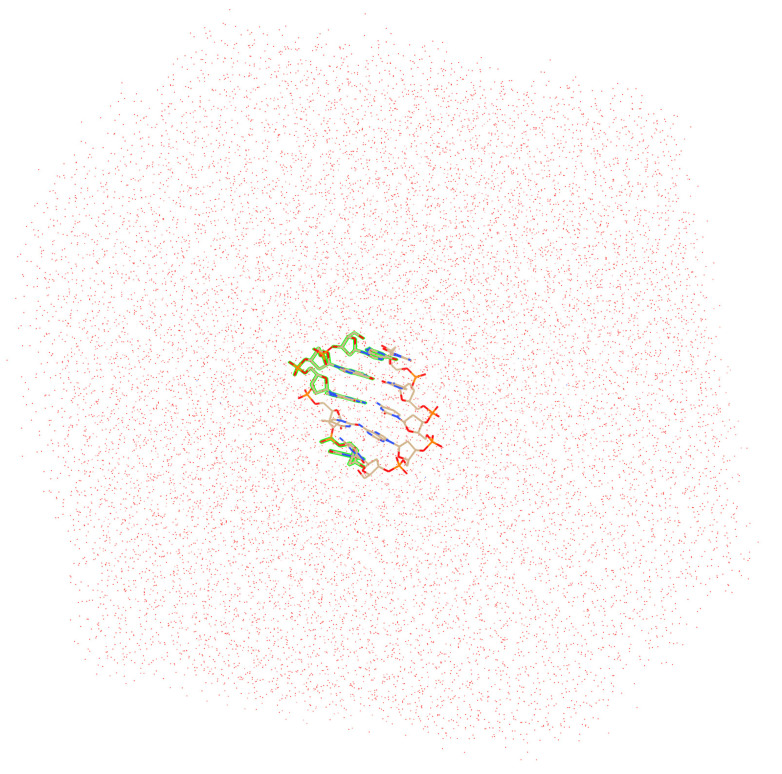
Optimized structure of 5${}^{\prime }$-GGGTG-3${}^{\prime }$ double strand immersed in a box consisting of *ca*. 15,000 water molecules. Hydrogen atoms have been omitted for clarity. Water molecules are depicted as red dots. Guanine nucleobases included in the QM layers of ONIOM computations are highlighted with a green border.

A pictorial representation of the optimized system is given in Figure 2, Cartesian coordinates of the optimized structure are given in the Supplementary Materials.

**Figure 3:**
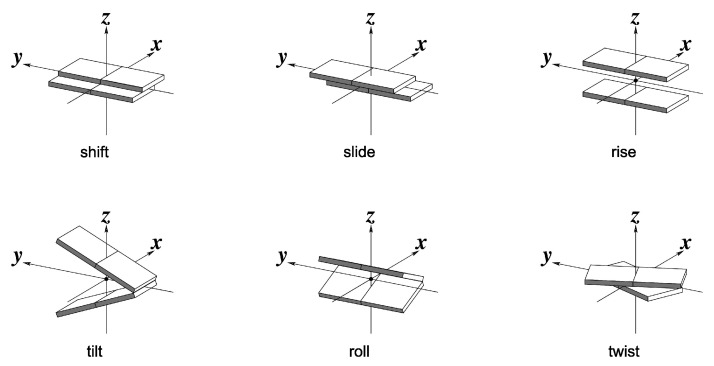
Graphical definitions of rigid-body coordinates used to describe the geometry of sequential base-pair steps.

The conformations of ds-5${}^{\prime }$-GGGTG-3${}^{\prime }$ were analyzed in terms of standard rigid body coordinates (see Figure 3) by using the 3DNA software [45,67,68].

## 3. Preliminary Considerations

Several MD and QM/MM studies dealing with HT in DNA were carried out in the last two decades. However, most simulations were performed on neutral DNA strands and were aimed at inferring the parameters that regulate the charge transfer in oxidized DNA, such as the hole site energies, the hopping integrals and their fluctuations originated by the dielectric environment. Therefore, quite long times were adopted in order to sample the whole conformational space [69,70,71,72]. The finalities of the present work are very different: herein, we are interested at estimating the time needed to disrupt the local solvation pattern around the G${}^{+}$-3${}^{\prime }$ nucleotide, corresponding to the loss of solvation energy of that site, eventually favoring the stabilization of the hole for the 5${}^{\prime }$-GGG${}^{+}$ terminal. To that end, we had to resort to a globally charged DNA strand [51].

Although NMR studies show that proximal waters near sites on the surface DNA are much less mobile than bulk water, with residence times near the phosphate backbone of a few hundred picoseconds [73,74], the dynamical response of water to a change in solute charge distribution is a much faster process, occurring in a few tenths of fs [36,37]. Therefore, starting the simulation with the charge localized on G${}^{+}$ appears to be a realistic choice. That condition, also met by adopting the equilibrium geometry of G${}^{+}$, ensures an optimum hydration pattern for the ionized 5${}^{\prime }$-GGGTG-3${}^{\prime }$ oligonucleotide holding the hole at the G-3${}^{\prime }$ site. It must be further stressed that, herein, we are interested in the activation step (Figure 1), namely the step that brings 5${}^{\prime }$-GGG into resonance with G-3${}^{\prime }$, but with the charge still localized on G. Therefore, the hole must remain localized on the G-3${}^{\prime }$ moiety during the whole simulation. Only when solvent response to such a non-equilibrium charge distribution leads to resonance conditions, HT can take place. As long as the simulation proceeds, the GGG tract, i.e., the thermodynamic site for the hole, can acquire favorable solvation energy. That can been rationalized in the framework of the extended RRKM theory [75] inasmuch as the GGG stack possesses several more vibrational degrees of freedom than a single G. Of course, even G-3${}^{\prime }$ on which the charge is located, could change its nuclear configuration over time, thus contributing to achieve the equalization of the G and GGG hole site levels. However, in order to properly model that effect, a realistic force field for G${}^{+}$ would be needed, ideally a quantum force field, due to the well-known difficulties of molecular mechanics in treating charged species. We have deliberately excluded that contribution because we are not sufficiently confident in the strength of the current classic force field in dealing with molecular ions. Therefore, rather than introducing further ambiguities, we have preferred to keep the geometry of G${}^{+}$-3${}^{\prime }$ restrained during the simulation to further ensure that the charge remain localized at that site.

## 4. Results and Discussion

**Figure 4:**
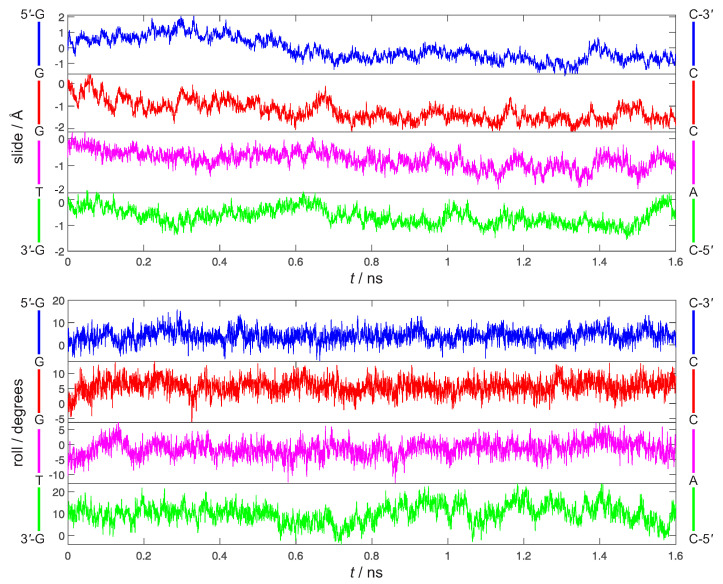
Time evolution of the four slide (Å, **top**) and roll (degrees, **bottom**) coordinates of ds-5${}^{\prime }$-GGGTG-3${}^{\prime }$ during 1.6 ns dynamics simulations in water.

**Figure 5:**
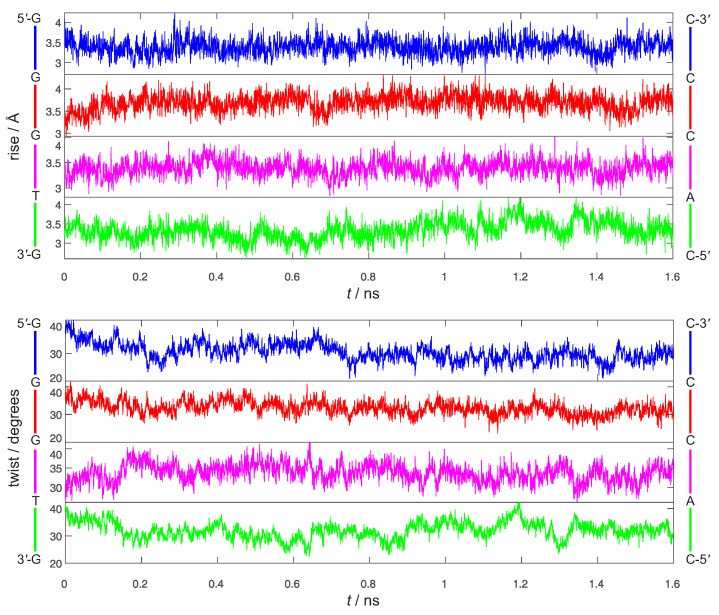
Time evolution of the four rise (Å, **top**) and twist (degrees, **bottom**) coordinates of ds-5${}^{\prime }$-GGGTG-3${}^{\prime }$ during 1.6 ns dynamics simulations in water.

The rigid body coordinates of 5${}^{\prime }$-GGGTG-3${}^{\prime }$ are reported in Figure 4 and Figure 5. Although the geometry of the 3${}^{\prime }$ ending G has been kept restrained to the one of G${}^{+}$, the 5${}^{\prime }$-GGGTG-3${}^{\prime }$ double helix retains an almost regular structure, pretty close to B-DNA during the whole simulation. Indeed, slide and roll values (Figure 4) fall into the domain of standard B-DNA in *ca*. 50% of the simulation time, occasionally approaching the A-DNA zone of Calladine’s diagram [76] for all the steps, but the 5${}^{\prime }$-TG-3${}^{\prime }$:3${}^{\prime }$-AC-5${}^{\prime }$ terminal residue of the oligomer, which exhibits the largest deviation from standard B-DNA, achieving roll values close to 20${}^{\circ }$ for a large part of the simulation. However, this is not definitively new, indeed ending steps of DNA are known to exhibit a larger conformational heterogeneity than the internal positions [77,78]. Furthermore (Figure 5), both rise and twist remain close to their standard values (3.3 Å and 36${}^{\circ }$, respectively), a relevant point inasmuch as it is now well ascertained that electronic couplings for HT are mainly influenced by the fluctuations of the rise and twist coordinates [79,80].

**Figure 6:**
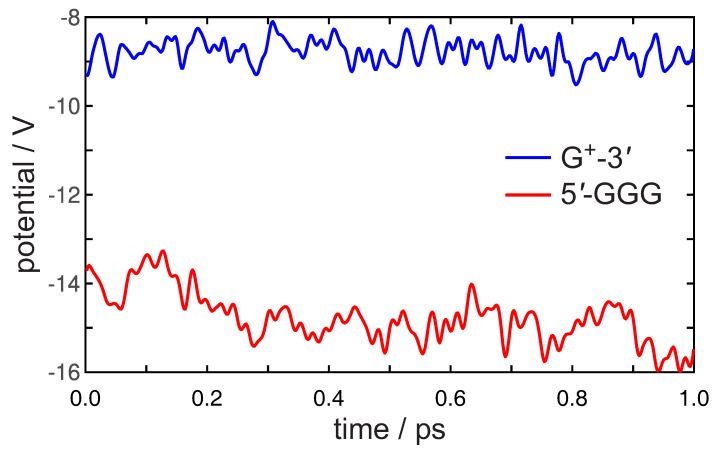
Electrostatic potential fluctuations at the G-3${}^{\prime }$ site (blue line) and 5${}^{\prime }$-GGG nucleobase stack (red line) for the initial 100 fs dynamics for ds-5${}^{\prime }$-GGGTG-3${}^{\prime }$ B-DNA sequence. Averages over 3 simulations are reported. The hole is localized at G-3${}^{\prime }$.

The electrostatic potential evaluated at the mean point of the positively charged G-3${}^{\prime }$ site and the neutral 5${}^{\prime }$-GGG unit is reported as a function of time, up to 1 ps, in Figure 6.

A positive charge is well stabilized on both sites, especially the GGG stack, indeed the averaged potentials amount to −8.78±0.27 V and −14.78±0.57 V for G and GGG, respectively. Notably, the potential at GGG is seen to decrease during the simulation. The large variations of the potential observed at GGG roughly indicate how thermal fluctuations of the environment can comparatively stabilize a hole at that site. The first oscillation of the potential occurs in *ca*. 45 fs for both signals, suggesting the action of DNA vibrations. Indeed, the Fourier analysis of the signals of Figure 6 returns a spectrum of operating frequencies in the range 100 to 3000 cm${}^{-1}$, thus demonstrating that both the DNA vibrational modes and backbone and solvent reorganization influence the electrostatic potential [81].

**Figure 7:**
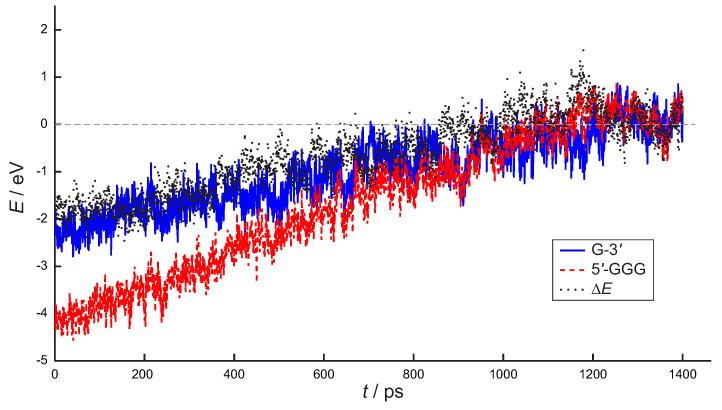
HOMO energy (eV) of 5${}^{\prime }$-GGG nucleobase stack (red dashed line) and G-3${}^{\prime }$ nucleobase (blue line) together with their difference (ΔE, black dots) for the 5${}^{\prime }$-GGGTG-3${}^{\prime }$ double strand immersed in a box consisting of *ca*. 15,000 water molecules and including counterions. Average values over 3 simulations are reported. The hole is initially (t=0) localized on G.

The HOMO energies of the GGG and G moieties retrieved by ONIOM computations (carried out for each snapshot) are plotted in Figure 7 as a function of time, a similar figure, also including statistical errors is provided in the Supplementary Materials.

At the starting time, the vertical ionization potential of G is predicted to lie ≈2 eV below the one of GGG, as expected, because the positive charge has been initially injected on G-3${}^{\prime }$. An almost linear increase in the HOMO energy of both the G and the GGG moieties is predicted for the first 800 ps (ΔE≈0, dotted black curve, Figure 7), with the hole site energy of GGG decreasing faster, in line with the results of Figure 6. Equalization of HOMO levels of G and GGG is found to occur at *ca*. 800 ps and then the G and GGG HOMO levels remain almost degenerate up to 2 ns, i.e., the whole simulation time.

**Figure 8:**
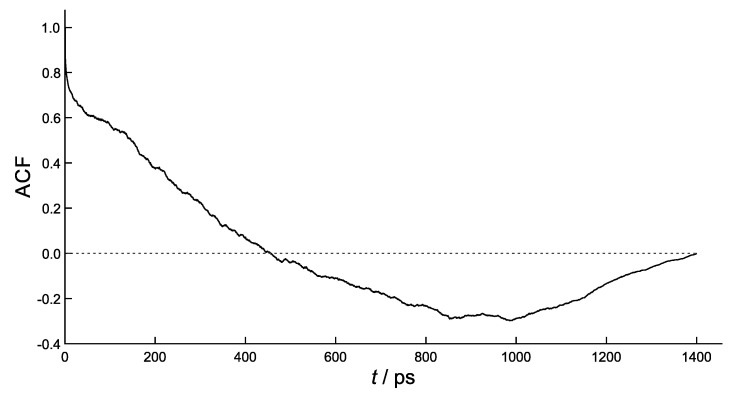
Autocorrelation function $\mathrm{ACF}=\langle \delta E(0)\delta E(t)\rangle /\langle {\left(\delta E\right)}^{2}\rangle$ averaged over 3 simulations.

The autocorrelation function of ΔE(t), defined as

$$\mathrm{ACF}=\frac{\langle \delta E(0)\delta E(t)\rangle }{\langle {\left(\delta E\right)}^{2}\rangle },$$

with δE(t)=ΔE(t)−〈ΔE〉 denoteing the instantaneous fluctuation of ΔE(t) from its equilibrium value, is diagrammed in Figure 8.

This correlation function, that measures the loss of memory of random fluctuations in the hole energy difference, does not exhibit the simple exponential decay typical of linear processes. Although the sampling time (0.5 ps) does not permit the evaluation of the initial decay, it is clearly seen that ACF reverses its direction at *ca*. 450 ps with a half period of *ca*. 900 ps, roughly the same time needed to annihilate ΔE(t). That confirms that the initial state is indeed at equilibrium, thus the half period $\tau_{\mathrm{act}}=900$ ps can safely be taken as the time needed to bring G${}^{+}$-3${}^{\prime }$ and 5${}^{\prime }$-GGG${}^{+}$ into electronic resonance. Present estimate thus appears to be *ca*. one order of magnitude larger than the values, 50–100 ps, inferred in previous work [34,35].

In order to assess the reliability of the present estimate of the activation time, we have computed the $P_{\mathrm{GGG}}/P_{G}$ yield ratios of oxidative damage at GGG and G sites for the 5${}^{\prime }$-GGG(T)${}_{n}$G-3${}^{\prime }$ sequences in which the charge is initially injected on G-3${}^{\prime }$, by using $\tau_{\mathrm{act}}=900$ ps, i.e., $k_{\mathrm{act}}=1/\tau_{\mathrm{act}}=1.1\times 10^{9}$ s${}^{-1}$ in the kinetic scheme (Figure 1), together with the $k_{\mathrm{HT}}$’s predicted by the quantum dynamics simulations of [34]. Current estimates of the oxidative damage yields are reported in Table 1 together with the results of [34] (obtained by adopting $\tau_{\mathrm{act}}=100$ ps) and their experimental counterparts from [26].

The relative yield ratios predicted upon enlarging the size of the ${\left(T\right)}_{n}$ bridge in 5${}^{\prime }$-GGG(T)${}_{n}$G-3${}^{\prime }$ sequences are well reproduced also by using an activation time amounting to *ca*. 1000 ps (Table 1), i.e., one order of magnitude larger than previous estimates. However, absolute yield ratios turn out to be underestimated by a factor of ten. Therefore, it appears that faster kinetics than those presently inferred for the activation step are needed to achieve quantitative predictions. That suggests that the approximations introduced in the theoretical model, in particular the restraints imposed to G${}^{+}$-3${}^{\prime }$, result in a too slow process possibly because of the lack of nuclear degrees of freedom of G${}^{+}$-3${}^{\prime }$ in simulation.

However, one should not put too much reliance to the discrepancy of the different values, Indeed, lacking a reliable force field for oxidized guanine, present preliminary simulations do not allow for the vibrational relaxation of the G-3${}^{\prime }$ moiety occurring before the removal of the positive charge. That, in turn, gives rise to a larger equilibration time. Therefore, the presently estimated activation time should be considered as an upper limit.

## 5. Conclusions

The activation time, i.e., the time needed for bringing the donor (G) and the acceptor site (GGG) into resonance and activating the hole transfer in the 5${}^{\prime }$-GGGTG${}^{+}$-3${}^{\prime }$ double stranded oxidized DNA sequence has been predicted by a theoretical protocol consisting in molecular dynamics simulations including the whole DNA strand, water and counterions at realistic concentration, followed by QM/MM computations. Activation appears to be modulated by both the vibrational modes of DNA and solvent response. The results of this preliminary study are consistent with an activation occurring in ≈0.9 ns. The time constant obtained by present computations is *ca*. one order of magnitude larger than previous estimates, ≈50–100 ps, inferred by a kinetic model capable of reproducing almost quantitatively the observed yield ratios of damaged DNA products, the most significant among observable quantities, at different sites for several diverse oxidized DNA sequences.

Of course, the computational strategy herein developed can be further improved. Work is in progress along that line. 

## Figures and Tables

**Table 1:** $P_{\mathrm{GGG}}/P_{G}$ yield ratios of oxidative damage at GGG and G sites for the oxidized 5${}^{\prime }$-GGG(T)${}_{n}$G-3${}^{\prime }$ DNA sequences predicted by using the kinetic model of Figure 1, together with $\tau_{\mathrm{act}}=900$ ps (first column, present estimate), and $\tau_{\mathrm{act}}=100$ ps (second column, ref [34]). Experimental values ([26]) are reported in the third column.

*n*	$\tau_{\mathrm{act}}=900$ ps	$\tau_{\mathrm{act}}=100$ ps	exper.
1	31	273	250
2	4.2	42	30
3	0.5	5.2	4.0
4	0.35	3.0	3.5
5	0.28	2.5	3.0
6	0.23	2.2	2.6
7	0.20	2.0	2.5

## Supplementary Materials

The following are available online. Cartesian coordinates of the whole system optimized at the AMBER OL15/TIP3P MM level of theory. Figure S1, HOMO energy of 5${}^{\prime }$-GGG stack and G-3${}^{\prime }$ nucleobase as a function of time, also including error bars.
