# Supplementary material for: The Time Scale of Electronic Resonance in Oxidized DNA as Modulated by Solvent Response: An MD/QM-MM Study
Source: Molecules. 2021 Sep 10;26(18):5497. doi: 10.3390/molecules26185497 (PMC8465834; doi:10.3390/molecules26185497)
Supplement: Supplementary file 1 [file molecules-26-05497-s001.zip › molecules-1348680-supplementary.pdf]

The Time Scale of Electronic Resonance in Oxidized DNA as Modulated by Solvent Response: An MD/QM-MM study

– Supplementary Materials –

Alessandro Landi, Amedeo Capobianco*, Andrea Peluso
Dipartimento di Chimica e Biologia "A. Zambelli", Università di Salerno
Via Giovanni Paolo II, I-84084, Fisciano (SA), Italy.
*Email: acapobianco@unisa.it

TABLE OF CONTENTS

- Cartesian coordinates of the whole system at its optimized geometry.
- HOMO energy of 5′-GGG stack and G-3′ nucleobase as a function of time, also including error bars.

Cartesian coordinates (Å) of the whole system optimized at the MM level by using the AMBER OL15 + TIP3P force field.

```
45740
Optimized geometry of the whole system
H       -5.66500      -7.97200       9.40700
O       -4.88400      -7.43600       9.24700
C       -3.59600      -8.07600       9.21000
H       -3.60600      -8.80000       8.39500
H       -3.38200      -8.58800      10.14800
C       -2.51400      -7.04600       8.94700
H       -1.58200      -7.47500       9.31400
O       -2.28400      -6.83400       7.52400
C       -2.27000      -5.43700       7.27700
H       -1.24600      -5.06800       7.33000
N       -2.65100      -5.21500       5.85400
C       -3.90400      -5.17000       5.28500
H       -4.78100      -5.30800       5.90000
N       -3.89700      -4.95300       3.99100
C       -2.54100      -4.84700       3.68200
C       -1.90400      -4.61300       2.43700
O       -2.41300      -4.44900       1.33000
N       -0.50900      -4.57900       2.57300
H        0.03100      -4.41900       1.74600
C        0.17900      -4.74900       3.75800
N        1.51000      -4.68200       3.67900
H        2.06200      -4.53300       2.84700
H        2.02100      -4.81500       4.54000
N       -0.42000      -4.96900       4.92700
C       -1.77300      -5.00500       4.81200
C       -2.78500      -5.64300       9.49000
H       -3.53800      -5.59300      10.27500
C       -3.25800      -4.84300       8.27600
H       -4.33000      -4.65900       8.35700
H       -2.72900      -3.89200       8.23900
O       -1.56000      -5.08400       9.95000
P       -1.56300      -4.21200      11.29300
O       -1.47100      -5.10900      12.46700
O       -2.71000      -3.27900      11.27700
O       -0.19800      -3.39000      11.13700
C        0.89500      -3.98200      10.41100
H        0.49500      -4.80100       9.81400
H        1.65700      -4.36600      11.08900
C        1.52200      -2.95400       9.48900
H        2.53300      -3.30100       9.28100
O        0.87500      -2.90900       8.18500
C        0.60500      -1.55300       7.86600
H        1.43300      -1.14400       7.28700
N       -0.54000      -1.52100       6.91400
C       -1.89100      -1.59200       7.17400
H       -2.24000      -1.68700       8.19200
N       -2.64500      -1.53500       6.10300
C       -1.72800      -1.41800       5.05900
C       -1.94500      -1.31600       3.66000
O       -3.00600      -1.30700       3.04400
N       -0.73400      -1.21500       2.96100
H       -0.78300      -1.13800       1.96600
C        0.52200      -1.21300       3.53800
N        1.55400      -1.10900       2.69700
H        1.51200      -1.04200       1.69000
H        2.47500      -1.11600       3.11000
N        0.72300      -1.31000       4.85000
C       -0.44100      -1.40900       5.54300
```

C	1.47100	-1.50600	9.97600
H	1.29900	-1.38700	11.04500
C	0.31300	-0.87800	9.20200
H	-0.53200	-0.72300	9.87300
H	0.62700	0.08100	8.78900
O	2.67500	-0.85000	9.59700
P	3.35300	0.17900	10.61800
O	4.18800	-0.56400	11.58900
O	2.31600	1.06100	11.19600
O	4.29300	1.02500	9.63700
C	4.82800	0.38800	8.46200
H	4.24200	-0.51200	8.27500
H	5.87400	0.11800	8.60300
C	4.70900	1.31800	7.27000
H	5.44900	0.98500	6.54200
O	3.43300	1.17900	6.58200
C	2.89500	2.47500	6.37300
H	3.19700	2.84100	5.39100
N	1.41500	2.34900	6.26000
C	0.47000	2.26200	7.25900
H	0.77600	2.27900	8.29500
N	-0.76200	2.15900	6.82100
C	-0.62400	2.18100	5.43400
C	-1.60900	2.10300	4.41600
O	-2.82700	1.99800	4.53000
N	-1.03300	2.16200	3.14000
H	-1.64900	2.11300	2.35400
C	0.32000	2.27900	2.88100
N	0.66900	2.31700	1.59300
H	0.05400	2.25800	0.79500
H	1.65700	2.39400	1.39600
N	1.24400	2.35100	3.83700
C	0.70000	2.29600	5.08100
C	4.80000	2.81200	7.58000
H	5.25900	3.05400	8.53800
C	3.35100	3.29800	7.57300
H	3.03100	3.50100	8.59500
H	3.27500	4.20900	6.98000
O	5.49900	3.45900	6.52400
P	6.53200	4.62900	6.87900
O	7.84000	4.04200	7.24200
O	5.92800	5.53700	7.87800
O	6.65100	5.38200	5.47200
C	6.48000	4.62700	4.25800
H	5.98500	3.69000	4.51500
H	7.44000	4.41300	3.78900
C	5.60900	5.39800	3.28600
H	5.82900	5.00700	2.29200
O	4.19100	5.13000	3.47800
C	3.50200	6.37000	3.51600
H	3.14000	6.67100	2.53300
N	2.24500	6.17700	4.29200
C	2.25200	6.21200	5.66200
H	3.16900	6.42500	6.19100
C	1.12400	6.04000	6.36900
C	1.12000	6.07600	7.86900
H	1.47900	7.04700	8.21200
H	0.10600	5.91400	8.23500
H	1.77300	5.29200	8.25300
C	-0.13700	5.81200	5.70400
O	-1.22100	5.64500	6.26700
N	-0.05300	5.79300	4.32600
H	-0.98000	5.62600	3.77800
C	1.09300	5.96700	3.57400
O	1.06800	5.93300	2.35500
C	5.71100	6.92100	3.36700
H	6.60800	7.29600	3.85900
C	4.47700	7.35000	4.16200
H	4.78000	7.66400	5.16000
H	3.98700	8.17900	3.65300
O	5.61100	7.45900	2.05400
P	6.53800	8.70100	1.65100
O	7.87100	8.21000	1.23500
O	6.52600	9.70200	2.74100
O	5.75700	9.28000	0.38100
C	5.00000	8.37600	-0.44400
H	4.83900	7.46000	0.12500
H	5.53500	8.14100	-1.36500
C	3.65700	8.99000	-0.79000
H	3.30900	8.48700	-1.69300
O	2.63900	8.69600	0.20700
C	1.97500	9.90600	0.53900
H	1.09300	10.02000	-0.09000
N	1.41300	9.76400	1.91100
C	2.03700	9.92600	3.12900
H	3.08300	10.19300	3.16800
N	1.25400	9.72800	4.16200
C	0.02400	9.41300	3.58700
C	-1.21700	9.09700	4.20000
O	-1.49000	9.02900	5.39500
N	-2.21100	8.84200	3.24400
H	-3.12300	8.61000	3.57900
C	-2.02800	8.88700	1.87600
N	-3.10300	8.61100	1.13200
H	-4.02400	8.36400	1.46300
H	-2.97500	8.63200	0.13000
N	-0.86500	9.18200	1.30200
C	0.11000	9.43200	2.21500
C	3.63300	10.51400	-0.90400
H	4.60800	10.97800	-1.05500
C	3.04000	10.99200	0.42100
H	3.82500	11.43800	1.03100
H	2.26500	11.73400	0.22700
O	2.74900	10.88300	-1.95500
H	2.66700	11.82800	-2.10300
H	-12.25200	8.56000	5.37000
O	-11.62700	8.37300	4.66500
C	-11.31400	9.41000	3.71600
H	-10.87300	10.24200	4.26500
H	-12.20900	9.75300	3.19700
C	-10.31300	8.89800	2.70000
H	-10.41100	9.53400	1.82100
O	-8.93600	9.08500	3.13400
C	-8.23700	7.86400	2.94300
H	-7.75700	7.87000	1.96400
N	-7.09300	7.83200	3.89600
C	-7.26900	7.45200	5.19100
H	-8.25100	7.16800	5.54100

C	-6.22700	7.42200	6.06000
H	-6.38800	7.11500	7.09300
C	-4.94500	7.80700	5.54800
N	-3.88400	7.80000	6.34100
H	-2.99900	8.07700	5.94300
H	-3.97300	7.52500	7.30800
N	-4.78300	8.17800	4.27100
C	-5.83900	8.20100	3.41800
O	-5.72700	8.53800	2.23400
C	-10.39900	7.40700	2.37600
H	-11.34500	6.93400	2.63700
C	-9.27100	6.76900	3.18600
H	-9.69300	6.21300	4.02300
H	-8.70400	6.08900	2.54900
O	-10.12300	7.21900	0.99300
P	-10.97000	6.13700	0.17200
O	-12.24600	6.74000	-0.27300
O	-11.08100	4.89100	0.96200
O	-10.02300	5.88800	-1.09300
C	-9.18100	6.96000	-1.55700
H	-9.11000	7.69800	-0.75700
H	-9.59800	7.43100	-2.44800
C	-7.79600	6.43200	-1.87300
H	-7.34300	7.14200	-2.56500
O	-6.91800	6.44500	-0.71100
C	-6.28300	5.17900	-0.61700
H	-5.32400	5.14300	-1.13400
N	-5.90300	4.95700	0.80600
C	-6.68300	4.50400	1.83100
H	-7.70600	4.21100	1.64300
N	-6.06200	4.41500	2.96500
C	-4.77300	4.84100	2.67400
C	-3.61900	4.98000	3.46400
N	-3.57900	4.68900	4.77200
H	-2.72000	4.80900	5.28900
H	-4.40800	4.35100	5.24000
N	-2.50500	5.42900	2.85900
C	-2.55100	5.71600	1.55800
H	-1.66700	6.07900	1.05400
N	-3.56300	5.62700	0.72300
C	-4.66600	5.17400	1.35500
C	-7.73200	4.98700	-2.36700
H	-8.67100	4.59200	-2.75400
C	-7.30500	4.17800	-1.14300
H	-8.15400	3.60400	-0.77200
H	-6.50000	3.49700	-1.41900
O	-6.71400	4.88300	-3.35500
P	-6.96800	3.96000	-4.63700
O	-7.72800	4.72100	-5.65400
O	-7.56000	2.67300	-4.21200
O	-5.47300	3.71800	-5.15300
C	-4.47900	4.73300	-4.91800
H	-4.85600	5.39500	-4.13800
H	-4.28600	5.31200	-5.82100
C	-3.18700	4.09600	-4.44800
H	-2.39000	4.80900	-4.66400
O	-3.14400	3.92800	-3.00200
C	-2.72700	2.60200	-2.71700
H	-1.64500	2.58300	-2.58700
N	-3.25000	2.23100	-1.37200
C	-4.52900	1.79300	-1.21400
H	-5.18000	1.71000	-2.07100
C	-5.00600	1.45500	0.01100
H	-6.03200	1.10600	0.11800
C	-4.10300	1.58100	1.11600
N	-4.50100	1.26800	2.34000
H	-3.83400	1.37100	3.09100
H	-5.44300	0.93800	2.50000
N	-2.84500	2.01200	0.94900
C	-2.38800	2.34600	-0.28500
O	-1.23400	2.74800	-0.47900
C	-2.90100	2.69500	-4.99000
H	-3.45700	2.42800	-5.88900
C	-3.29100	1.75300	-3.85200
H	-4.21900	1.24000	-4.10600
H	-2.50100	1.01900	-3.70000
O	-1.50400	2.56800	-5.22300
P	-1.00100	1.77900	-6.52200
O	-1.00500	2.69000	-7.68700
O	-1.77800	0.52800	-6.67200
O	0.50600	1.43000	-6.11600
C	1.21500	2.31900	-5.23300
H	0.48000	2.95100	-4.73600
H	1.91700	2.94500	-5.78500
C	1.97300	1.51900	-4.19100
H	2.77200	2.16500	-3.82400
O	1.16500	1.23500	-3.01400
C	1.29200	-0.14500	-2.70700
H	2.09700	-0.28300	-1.98400
N	0.07400	-0.56600	-1.96000
C	-1.07500	-0.88500	-2.61500
H	-1.11300	-0.83400	-3.69400
C	-2.18500	-1.27000	-1.93500
H	-3.09600	-1.52200	-2.47700
C	-2.08400	-1.32300	-0.50600
N	-3.12900	-1.69000	0.22000
H	-3.01700	-1.71500	1.22300
H	-3.99900	-1.93400	-0.22900
N	-0.94800	-1.00800	0.12800
C	0.15100	-0.62500	-0.57100
O	1.21800	-0.32700	-0.02000
C	2.46900	0.14500	-4.63900
H	2.52800	0.00800	-5.71900
C	1.45800	-0.84100	-4.05400
H	0.83100	-1.23700	-4.85300
H	1.98700	-1.66000	-3.56800
O	3.73800	-0.10300	-4.04500
P	4.86900	-0.83400	-4.91000
O	5.57500	0.16300	-5.74600
O	4.28000	-1.98400	-5.62800
O	5.85100	-1.36300	-3.76100
C	5.94900	-0.62200	-2.53100
H	5.08400	0.03700	-2.46900
H	6.86200	-0.02700	-2.50200
C	5.93500	-1.57300	-1.35100
H	6.39800	-1.04200	-0.51900
O	4.58400	-1.86800	-0.89100
C	4.46000	-3.27300	-0.73900

H	4.69300	-3.54900	0.29000
N	3.02000	-3.62800	-0.88100
C	2.45200	-3.77700	-2.10700
H	3.04700	-3.63800	-2.99700
C	1.14000	-4.10100	-2.23600
H	0.70300	-4.21700	-3.22800
C	0.39300	-4.27700	-1.02600
N	-0.89100	-4.59400	-1.07800
H	-1.38200	-4.70800	-0.20300
H	-1.34900	-4.71300	-1.97000
N	0.96300	-4.12900	0.17700
C	2.27800	-3.80400	0.28400
O	2.83800	-3.65800	1.37700
C	6.54800	-2.95100	-1.60000
H	7.21600	-3.00800	-2.45900
C	5.35000	-3.87400	-1.82300
H	5.28700	-4.14100	-2.87800
H	5.47100	-4.77800	-1.22700
O	7.22900	-3.37100	-0.42300
H	7.64900	-4.23200	-0.48400
Na	-11.48400	-3.85900	22.17100
Na	1.44500	1.49800	25.16200
Na	13.14800	7.64800	19.68500
Na	21.84600	-3.09000	6.01500
Na	-24.00000	0.08900	3.41500
Na	-19.68100	-0.98900	-10.18500
Na	-7.66300	-5.24900	-19.34400
Na	6.72600	-6.73300	-20.04800
Cl	-4.48600	-6.38700	-22.13600
O	6.73300	-10.02400	45.75800
H	6.08200	-9.43900	45.37200
H	7.52800	-9.49400	45.81800
O	8.21100	-12.33500	46.29500
H	7.57600	-12.26200	47.00700
H	7.88100	-11.74600	45.61700
O	2.43900	-5.15700	49.32600
H	2.36100	-4.20600	49.40500
H	1.83500	-5.50300	49.98300
O	10.37600	-17.45500	46.50500
H	11.28800	-17.22300	46.32900
H	9.86700	-16.87200	45.94200
O	4.71200	-9.79900	48.04600
H	4.57700	-8.95400	48.47500
H	5.25300	-9.59700	47.28300
O	8.02700	-16.28500	45.08300
H	7.51100	-15.51500	45.32200
H	7.75400	-16.95900	45.70700
O	13.00000	-13.53200	47.01900
H	12.22300	-13.87100	46.57400
H	13.67900	-13.52700	46.34400
O	4.40500	-14.43500	41.56200
H	3.49800	-14.66600	41.76100
H	4.90200	-15.23200	41.75000
O	7.31200	-5.72600	44.48200
H	6.63800	-5.42200	45.09000
H	7.69900	-4.92300	44.13200
O	10.81600	-10.72300	49.85400
H	10.36200	-11.56400	49.91000
H	10.18500	-10.08700	50.19100
O	9.25900	-8.88600	50.78900
H	9.55200	-8.32500	50.07000
H	9.76900	-8.59300	51.54400
O	5.28200	-12.79300	43.42300
H	4.84400	-13.51000	42.96400
H	6.02300	-12.56600	42.86200
O	13.39900	-14.51700	49.48100
H	13.26100	-14.32700	48.55300
H	13.04300	-13.75600	49.93800
O	5.51100	-5.55800	46.69700
H	5.68600	-6.02900	47.51100
H	4.82400	-4.93100	46.92500
O	9.40300	-13.93500	43.01700
H	8.78600	-14.66000	42.92000
H	8.93400	-13.17400	42.67300
O	4.37900	-7.36400	49.38500
H	3.59600	-6.82100	49.47900
H	5.07800	-6.84600	49.78400
O	10.22700	-10.61100	47.13600
H	10.50200	-10.72500	48.04500
H	9.35700	-11.00900	47.09700
O	6.86200	-6.36200	50.77900
H	7.04400	-7.18000	51.24200
H	7.68500	-6.14500	50.34200
O	7.19600	-7.43500	48.04100
H	7.72500	-7.94600	47.42800
H	7.45900	-7.75100	48.90500
O	10.29000	-7.46400	48.65600
H	10.46400	-8.02900	47.90400
H	10.78300	-6.66400	48.47400
O	11.27800	-8.14700	45.75600
H	11.97300	-7.52600	45.97500
H	11.62000	-8.99700	46.03100
O	13.17700	-10.69700	47.28500
H	13.70200	-10.35800	46.55900
H	13.08900	-11.63200	47.10100
O	5.36900	-8.04100	44.66300
H	4.79700	-7.42200	44.21000
H	5.94200	-7.49100	45.19700
O	8.58900	-7.75700	45.79400
H	9.52200	-7.82300	45.99700
H	8.50100	-6.92100	45.33600
O	11.21800	-8.44100	42.75500
H	11.13900	-8.77500	43.64800
H	12.03900	-7.94800	42.75400
O	5.75400	-16.70500	42.00500
H	6.70000	-16.83400	42.07400
H	5.37500	-17.44300	42.48200
O	3.93800	-18.78000	41.94000
H	3.03100	-19.07600	42.01700
H	4.21600	-19.07700	41.07400
O	8.78800	-11.12400	42.37600
H	9.49200	-10.91900	42.99100
H	8.06400	-10.55600	42.64000
O	10.69700	-10.68500	44.42500
H	11.53100	-11.11700	44.24300
H	10.53500	-10.85700	45.35300
O	10.35100	-14.18200	45.79100
H	10.29600	-14.34800	44.85000

H	9.72600	-13.47500	45.94700
O	13.83500	-1.44700	43.78900
H	14.08800	-1.96900	43.02700
H	12.89700	-1.61000	43.89100
O	13.55500	0.45600	45.99100
H	13.43800	-0.22000	46.65900
H	13.66500	-0.03100	45.17400
O	15.53800	-5.05400	44.69200
H	15.70600	-4.18600	45.05900
H	15.34800	-4.89100	43.76800
O	15.51700	-2.59800	45.67700
H	15.39700	-2.15300	46.51500
H	14.82200	-2.25000	45.11800
O	12.15800	-4.23200	44.93900
H	11.32200	-3.89400	45.26000
H	12.01900	-4.36700	44.00200
O	14.13300	-9.38800	49.64700
H	13.48000	-9.70300	49.02200
H	14.09100	-10.01000	50.37300
O	15.24500	0.89400	41.56200
H	14.33700	0.66300	41.76100
H	15.74200	0.09800	41.75000
O	6.99200	-1.28400	50.06600
H	6.79500	-1.87700	49.34100
H	6.43700	-1.58900	50.78400
O	16.12100	2.53600	43.42300
H	15.68300	1.82000	42.96400
H	16.86200	2.76400	42.86200
O	9.48700	-1.75800	47.75000
H	8.55700	-1.81900	47.96700
H	9.58300	-2.28000	46.95400
O	9.40500	-5.23200	50.33800
H	9.66600	-4.31800	50.22900
H	10.22700	-5.70000	50.48800
O	20.24200	1.39400	43.01700
H	19.62500	0.66900	42.92000
H	19.77400	2.15500	42.67300
O	12.03200	-4.38700	42.13900
H	11.15500	-4.50800	41.77500
H	12.50800	-5.17800	41.88700
O	6.76000	-2.79300	47.58000
H	7.05500	-3.70200	47.52700
H	5.85900	-2.81400	47.25800
O	8.48900	-5.11100	47.70400
H	8.16000	-5.99200	47.52100
H	8.84300	-5.16700	48.59200
O	11.60300	-4.82400	48.07100
H	11.94200	-4.29600	47.34900
H	12.05000	-4.48600	48.84700
O	13.59000	-6.32700	46.27100
H	14.31000	-5.94900	45.76700
H	12.96600	-5.60800	46.37400
O	17.73300	-5.62200	47.20700
H	17.94900	-4.74000	46.90600
H	17.25200	-6.01400	46.47700
O	13.73600	-6.79200	49.10100
H	13.85800	-7.72000	49.30000
H	13.44600	-6.77800	48.18800
O	9.73400	-3.44700	45.69700
H	9.04100	-3.37300	45.04100
H	9.48900	-4.21100	46.21900
O	16.59300	-1.37600	42.00500
H	17.53900	-1.50500	42.07400
H	16.21500	-2.11400	42.48200
O	14.77700	-3.45100	41.94000
H	13.87100	-3.74700	42.01700
H	15.05500	-3.74800	41.07400
O	16.39400	-8.90700	48.09500
H	15.58400	-9.25200	48.47000
H	17.08200	-9.21600	48.68400
O	13.54700	-6.59200	42.08200
H	14.40900	-6.26600	42.34100
H	13.73200	-7.37900	41.56900
O	14.50000	-9.35200	45.37000
H	14.48800	-8.40800	45.52600
H	15.39300	-9.53500	45.07600
O	6.73300	-33.01800	32.48300
H	6.08200	-32.43300	32.09600
H	7.52800	-32.48700	32.54200
O	8.21100	-35.32900	33.02000
H	7.57600	-35.25600	33.73200
H	7.88100	-34.74000	32.34200
O	2.43900	-28.15100	36.05000
H	2.36100	-27.20000	36.12900
H	1.83500	-28.49600	36.70800
O	10.37600	-40.44900	33.22900
H	11.28800	-40.21600	33.05400
H	9.86700	-39.86500	32.66700
O	4.71200	-32.79300	34.77100
H	4.57700	-31.94800	35.19900
H	5.25300	-32.59100	34.00700
O	8.02700	-39.27900	31.80800
H	7.51100	-38.50800	32.04700
H	7.75400	-39.95200	32.43100
O	13.00000	-36.52600	33.74300
H	12.22300	-36.86500	33.29900
H	13.67900	-36.52000	33.06900
O	4.40500	-37.42900	28.28700
H	3.49800	-37.66000	28.48600
H	4.90200	-38.22500	28.47400
O	7.31200	-28.71900	31.20600
H	6.63800	-28.41600	31.81500
H	7.69900	-27.91600	30.85700
O	10.81600	-33.71700	36.57800
H	10.36200	-34.55800	36.63500
H	10.18500	-33.08100	36.91600
O	9.25900	-31.88000	37.51300
H	9.55200	-31.31900	36.79400
H	9.76900	-31.58700	38.26900
O	5.28200	-35.78700	30.14800
H	4.84400	-36.50300	29.68800
H	6.02300	-35.55900	29.58600
O	13.39900	-37.51100	36.20600
H	13.26100	-37.32100	35.27800
H	13.04300	-36.74900	36.66200
O	5.51100	-28.55100	33.42100
H	5.68600	-29.02300	34.23600
H	4.82400	-27.92500	33.64900

O	9.40300	-36.92900	29.74100
H	8.78600	-37.65400	29.64400
H	8.93400	-36.16800	29.39800
O	4.37900	-30.35700	36.10900
H	3.59600	-29.81500	36.20300
H	5.07800	-29.84000	36.50900
O	10.22700	-33.60500	33.86000
H	10.50200	-33.71900	34.77000
H	9.35700	-34.00300	33.82200
O	6.86200	-29.35600	37.50400
H	7.04400	-30.17400	37.96600
H	7.68500	-29.13900	37.06600
O	7.19600	-30.42900	34.76500
H	7.72500	-30.94000	34.15200
H	7.45900	-30.74500	35.63000
O	10.29000	-30.45700	35.38100
H	10.46400	-31.02300	34.62800
H	10.78300	-29.65700	35.19900
O	11.27800	-31.14100	32.48000
H	11.97300	-30.52000	32.69900
H	11.62000	-31.99100	32.75600
O	13.17700	-33.69100	34.00900
H	13.70200	-33.35200	33.28400
H	13.08900	-34.62600	33.82500
O	5.36900	-31.03500	31.38800
H	4.79700	-30.41600	30.93500
H	5.94200	-30.48500	31.92200
O	8.58900	-30.75100	32.51900
H	9.52200	-30.81600	32.72200
H	8.50100	-29.91500	32.06000
O	11.21800	-31.43400	29.47900
H	11.13900	-31.76800	30.37300
H	12.03900	-30.94200	29.47800
O	5.75400	-39.69900	28.72900
H	6.70000	-39.82800	28.79800
H	5.37500	-40.43700	29.20600
O	3.93800	-41.77400	28.66500
H	3.03100	-42.07000	28.74100
H	4.21600	-42.07100	27.79800
O	8.78800	-34.11800	29.10000
H	9.49200	-33.91300	29.71600
H	8.06400	-33.55000	29.36500
O	10.69700	-33.67900	31.15000
H	11.53100	-34.11100	30.96800
H	10.53500	-33.85100	32.07800
O	10.35100	-37.17600	32.51600
H	10.29600	-37.34200	31.57400
H	9.72600	-36.46800	32.67200
O	16.13400	-23.21000	41.18200
H	15.79600	-23.32500	40.29400
H	17.00300	-23.61000	41.16200
O	11.99300	-21.35600	37.17100
H	11.42900	-21.44700	36.40300
H	11.87800	-22.17600	37.65200
O	9.53900	-19.86100	40.01000
H	10.33100	-20.20200	39.59400
H	9.77900	-19.75100	40.93000
O	17.57300	-17.68900	32.48300
H	16.92200	-17.10400	32.09600
H	18.36700	-17.15800	32.54200
O	11.49800	-20.60000	32.88100
H	12.27100	-21.14700	32.74300
H	11.85100	-19.73600	33.09300
O	13.33700	-19.27200	46.08800
H	13.04600	-18.36800	45.97300
H	13.97300	-19.23100	46.80300
O	14.26200	-21.89500	42.67800
H	14.85900	-22.11900	41.96400
H	14.32000	-22.63600	43.28100
O	13.83500	-24.44100	30.51300
H	14.08800	-24.96300	29.75200
H	12.89700	-24.60400	30.61600
O	18.87900	-25.43700	38.32100
H	19.66600	-25.85500	37.97000
H	19.14300	-24.53300	38.48900
O	19.05000	-20.00000	33.02000
H	18.41500	-19.92600	33.73200
H	18.72000	-19.41100	32.34200
O	13.55500	-22.53800	32.71600
H	13.43800	-23.21300	33.38400
H	13.66500	-23.02500	31.89900
O	15.89800	-23.07700	36.42000
H	15.53400	-23.60400	37.13200
H	16.75400	-23.46700	36.24700
O	15.53800	-28.04800	31.41700
H	15.70600	-27.18000	31.78400
H	15.34800	-27.88500	30.49300
O	18.29600	-19.58600	43.08300
H	17.99600	-18.68400	42.97500
H	17.95100	-19.85300	43.93500
O	11.74700	-23.13500	39.11700
H	10.82200	-23.26700	39.32500
H	11.99600	-22.35500	39.61400
O	15.51700	-25.59200	32.40100
H	15.39700	-25.14600	33.24000
H	14.82200	-25.24400	31.84300
O	9.10800	-23.49200	39.95100
H	9.23700	-23.23800	40.86500
H	8.42800	-22.89900	39.63200
O	13.27800	-12.82200	36.05000
H	13.20000	-11.87100	36.12900
H	12.67400	-13.16700	36.70800
O	21.21600	-25.12000	33.22900
H	22.12800	-24.88700	33.05400
H	20.70700	-24.53600	32.66700
O	19.82300	-20.13700	40.62100
H	19.19400	-19.97300	39.91800
H	19.43500	-19.72300	41.39200
O	23.70200	-19.20200	37.97500
H	23.01700	-18.82200	37.42500
H	23.78600	-18.59200	38.70800
O	15.37500	-20.40000	37.88600
H	15.83900	-20.12100	37.09600
H	15.11100	-21.30200	37.70300
O	12.26800	-20.85300	40.82400
H	12.83800	-20.42400	40.18500
H	12.85900	-21.13500	41.52200
O	14.31200	-24.27800	34.69500

H	15.01500	-23.82600	35.16200
H	13.98000	-24.91900	35.32300
O	8.05500	-26.01900	39.99600
H	7.26500	-25.76400	39.52000
H	8.54600	-25.20400	40.10100
O	10.00600	-25.65500	36.95500
H	9.20900	-25.54300	37.47300
H	9.77900	-25.31600	36.08900
O	15.83700	-19.13000	44.90300
H	15.90100	-18.66800	45.73900
H	15.12800	-19.76100	45.03100
O	15.55200	-17.46400	34.77100
H	15.41600	-16.61800	35.19900
H	16.09300	-17.26200	34.00700
O	9.00500	-19.94600	35.34400
H	9.51700	-19.15200	35.49400
H	9.65700	-20.64300	35.27700
O	10.81400	-16.82900	37.42600
H	10.99100	-15.90800	37.23800
H	11.61000	-17.14400	37.85500
O	11.93700	-15.31200	43.42700
H	12.48800	-15.44000	42.65500
H	11.09800	-15.01200	43.07600
O	6.97000	-21.61700	36.12000
H	7.80700	-21.20700	35.90300
H	7.14700	-22.55700	36.09000
O	18.86600	-23.95000	31.80800
H	18.35100	-23.17900	32.04700
H	18.59400	-24.62300	32.43100
O	16.45100	-29.10500	36.86600
H	15.59500	-29.49400	36.68700
H	16.86300	-29.02500	36.00600
O	23.83900	-21.19600	33.74300
H	23.06200	-21.53500	33.29900
H	24.51900	-21.19100	33.06900
O	18.65500	-26.07700	33.63400
H	18.35000	-25.76200	34.48500
H	19.60800	-25.99900	33.67700
O	17.09900	-19.91800	35.28300
H	16.57100	-20.65300	34.97100
H	16.59800	-19.14000	35.03700
O	11.83100	-29.25600	37.64400
H	12.40600	-29.47800	36.91100
H	12.41800	-29.18700	38.39700
O	20.88900	-23.36900	38.45000
H	21.40200	-22.73600	38.95200
H	21.53900	-23.96800	38.08500
O	12.15800	-27.22600	31.66300
H	11.32200	-26.88800	31.98400
H	12.01900	-27.36100	30.72600
O	14.13300	-32.38200	36.37100
H	13.48000	-32.69700	35.74700
H	14.09100	-33.00400	37.09700
O	15.24500	-22.10000	28.28700
H	14.33700	-22.33100	28.48600
H	15.74200	-22.89600	28.47400
O	18.15100	-13.39000	31.20600
H	17.47800	-13.08700	31.81500
H	18.53800	-12.58700	30.85700
O	17.36700	-25.89400	40.69600
H	16.44900	-25.67200	40.53800
H	17.78300	-25.81900	39.83700
O	18.15100	-24.49500	35.82600
H	18.89800	-23.98200	35.51700
H	18.42000	-24.81600	36.68700
O	16.42300	-21.99900	32.80900
H	15.51500	-22.28300	32.90600
H	16.45300	-21.57900	31.94900
O	21.65500	-18.38800	36.57800
H	21.20200	-19.22900	36.63500
H	21.02500	-17.75200	36.91600
O	20.09900	-16.55000	37.51300
H	20.39100	-15.99000	36.79400
H	20.60800	-16.25700	38.26900
O	6.99200	-24.27800	36.79100
H	6.79500	-24.87100	36.06600
H	6.43700	-24.58300	37.50800
O	14.97200	-24.38600	38.91000
H	15.02700	-25.33800	38.83400
H	14.16100	-24.23000	39.39400
O	16.12100	-20.45800	30.14800
H	15.68300	-21.17400	29.68800
H	16.86200	-20.23000	29.58600
O	12.91100	-25.76800	36.66900
H	12.09800	-25.46900	37.07600
H	13.40700	-26.16300	37.38700
O	15.06200	-15.77200	38.60300
H	15.02000	-15.54600	37.67300
H	15.97800	-16.00600	38.75100
O	24.23800	-22.18200	36.20600
H	24.10000	-21.99200	35.27800
H	23.88200	-21.42000	36.66200
O	13.17900	-18.89600	38.39900
H	13.97300	-19.39100	38.19700
H	12.46800	-19.42400	38.03600
O	17.99800	-16.51400	42.75000
H	18.05200	-15.94500	43.51800
H	17.13000	-16.34400	42.38500
O	9.48700	-24.75200	34.47500
H	8.55700	-24.81300	34.69200
H	9.58300	-25.27400	33.67800
O	9.40500	-28.22600	37.06300
H	9.66600	-27.31200	36.95300
H	10.22700	-28.69400	37.21200
O	15.75600	-18.32300	42.07900
H	15.61700	-18.69100	42.95100
H	14.96700	-17.80800	41.90700
O	16.35000	-13.22200	33.42100
H	16.52600	-13.69300	34.23600
H	15.66400	-12.59600	33.64900
O	17.30600	-18.94500	39.46700
H	16.97100	-18.98500	40.36300
H	16.80700	-19.61000	38.99400
O	20.24200	-21.59900	29.74100
H	19.62500	-22.32500	29.64400
H	19.77400	-20.83800	29.39800
O	10.91700	-10.07300	40.19500
H	11.15600	-9.44100	40.87300

H	10.07400	-10.42400	40.48200
O	12.03200	-27.38100	28.86300
H	11.15500	-27.50200	28.49900
H	12.50800	-28.17200	28.61100
O	3.55200	-22.59300	38.95700
H	4.23900	-22.90700	38.36900
H	3.39700	-21.68900	38.68400
O	15.42900	-14.26500	42.59900
H	15.35600	-13.46000	43.11100
H	14.98700	-14.06600	41.77400
O	15.21800	-15.02800	36.10900
H	14.43500	-14.48600	36.20300
H	15.91800	-14.51100	36.50900
O	17.88900	-14.36700	44.75500
H	18.60200	-13.73200	44.68300
H	17.39800	-14.27000	43.93900
O	19.61100	-20.46200	36.32700
H	18.67700	-20.34100	36.15700
H	19.78900	-21.36400	36.06300
O	15.11200	-27.07700	38.48000
H	14.74100	-27.63000	39.16800
H	15.60700	-27.68100	37.92800
O	12.94000	-16.61700	45.57600
H	13.64100	-15.98400	45.72900
H	12.47000	-16.27600	44.81500
O	21.06600	-18.27500	33.86000
H	21.34200	-18.39000	34.77000
H	20.19600	-18.67400	33.82200
O	17.70100	-14.02700	37.50400
H	17.88400	-14.84500	37.96600
H	18.52500	-13.81000	37.06600
O	18.03600	-15.10000	34.76500
H	18.56400	-15.61100	34.15200
H	18.29800	-15.41600	35.63000
O	8.53700	-17.08400	42.50900
H	9.12300	-17.82900	42.64100
H	8.34000	-16.77300	43.39200
O	11.39100	-14.04800	37.52500
H	10.72200	-13.56400	37.04100
H	11.25200	-13.80200	38.43900
O	15.53200	-12.01400	44.12300
H	16.08300	-11.85100	44.88800
H	15.87200	-11.42400	43.45100
O	6.76000	-25.78700	34.30500
H	7.05500	-26.69600	34.25100
H	5.85900	-25.80800	33.98300
O	18.66800	-14.06600	41.11000
H	18.54400	-14.86700	40.60100
H	19.32600	-13.57100	40.62100
O	10.28900	-27.71100	40.28700
H	9.56800	-27.09700	40.14800
H	11.07900	-27.17500	40.20900
O	20.73200	-16.26500	42.73800
H	19.78500	-16.38400	42.66500
H	21.08000	-17.15300	42.82000
O	8.48900	-28.10500	34.42800
H	8.16000	-28.98500	34.24600
H	8.84300	-28.16000	35.31600
O	17.73700	-16.47000	38.93800
H	17.58900	-17.39800	39.11900
H	18.56200	-16.44800	38.45400
O	11.60300	-27.81800	34.79600
H	11.94200	-27.28900	34.07400
H	12.05000	-27.48000	35.57200
O	21.34000	-15.64700	40.02400
H	22.26500	-15.85300	39.88700
H	21.17200	-15.89500	40.93300
O	13.59000	-29.32000	32.99500
H	14.31000	-28.94200	32.49200
H	12.96600	-28.60200	33.09900
O	17.73300	-28.61600	33.93100
H	17.94900	-27.73300	33.63000
H	17.25200	-29.00700	33.20200
O	12.47300	-25.65700	40.50800
H	12.32700	-25.34300	41.40100
H	11.99500	-25.03900	39.95500
O	14.33200	-28.66700	40.55600
H	14.31800	-29.56300	40.22000
H	15.05000	-28.66000	41.19000
O	21.13000	-15.12800	35.38100
H	21.30300	-15.69400	34.62800
H	21.62200	-14.32800	35.19900
O	13.73600	-29.78600	35.82500
H	13.85800	-30.71400	36.02400
H	13.44600	-29.77200	34.91300
O	22.11700	-15.81200	32.48000
H	22.81200	-15.19100	32.69900
H	22.45900	-16.66200	32.75600
O	24.01600	-18.36200	34.00900
H	24.54100	-18.02300	33.28400
H	23.92800	-19.29700	33.82500
O	15.67800	-14.44800	46.13800
H	15.98700	-14.69300	47.01000
H	16.47600	-14.24400	45.65000
O	15.70000	-12.29400	38.36900
H	15.26100	-12.78700	39.06200
H	16.45100	-12.83700	38.12800
O	8.17900	-16.10300	38.37100
H	9.00500	-16.40300	37.99100
H	7.82900	-16.87300	38.82000
O	13.14800	-16.75100	40.44100
H	12.52600	-17.41400	40.14100
H	13.81800	-16.72500	39.75800
O	6.38000	-18.18500	39.33000
H	5.54400	-18.61200	39.14800
H	6.35300	-17.99100	40.26700
O	12.70900	-18.20000	33.91700
H	12.85800	-17.51700	33.26300
H	13.51600	-18.22000	34.43200
O	10.46400	-22.17400	34.92300
H	10.21900	-23.07700	35.12100
H	10.63400	-22.17200	33.98100
O	9.73400	-26.44100	32.42100
H	9.04100	-26.36600	31.76500
H	9.48900	-27.20500	32.94400
O	16.20900	-15.70500	31.38800
H	15.63600	-15.08600	30.93500
H	16.78200	-15.15500	31.92200

O	19.42800	-15.42200	32.51900
H	20.36100	-15.48700	32.72200
H	19.34100	-14.58600	32.06000
O	22.05700	-16.10500	29.47900
H	21.97800	-16.43900	30.37300
H	22.87800	-15.61300	29.47800
O	16.59300	-24.36900	28.72900
H	17.53900	-24.49900	28.79800
H	16.21500	-25.10800	29.20600
O	14.77700	-26.44400	28.66500
H	13.87100	-26.74100	28.74100
H	15.05500	-26.74100	27.79800
O	5.69500	-25.12400	39.11500
H	5.37600	-25.97700	38.82200
H	5.14900	-24.90900	39.87100
O	16.39400	-31.90100	34.81900
H	15.58400	-32.24600	35.19500
H	17.08200	-32.21000	35.40800
O	13.54700	-29.58600	28.80600
H	14.40900	-29.26000	29.06600
H	13.73200	-30.37300	28.29300
O	5.56400	-21.50300	41.08400
H	4.87000	-21.91800	40.57100
H	6.31100	-21.46100	40.48800
O	7.47800	-21.57200	38.96100
H	7.45500	-21.47300	38.00900
H	7.78800	-20.72500	39.28200
O	10.13700	-19.51400	43.07500
H	11.09100	-19.54600	43.00800
H	9.93800	-19.97500	43.89000
O	20.48300	-28.55300	35.00500
H	19.59600	-28.26800	34.78700
H	20.89900	-28.71300	34.15800
O	8.49200	-13.97200	40.09200
H	8.35700	-14.73200	39.52600
H	8.07500	-13.24800	39.62500
O	19.62700	-18.78800	29.10000
H	20.33100	-18.58300	29.71600
H	18.90400	-18.22100	29.36500
O	20.45700	-23.03700	35.22000
H	20.40100	-22.58000	34.38100
H	21.01000	-23.79800	35.04300
O	11.64800	-13.07500	40.30400
H	11.53200	-12.13100	40.20200
H	10.82800	-13.37800	40.69300
O	21.53600	-18.35000	31.15000
H	22.37100	-18.78200	30.96800
H	21.37500	-18.52200	32.07800
O	21.19100	-21.84700	32.51600
H	21.13600	-22.01200	31.57400
H	20.56500	-21.13900	32.67200
O	14.50000	-32.34600	32.09400
H	14.48800	-31.40200	32.25100
H	15.39300	-32.52900	31.80000
O	12.67400	-12.42200	43.52000
H	13.57700	-12.39000	43.83700
H	12.44800	-13.35200	43.53500
O	14.42900	-13.66900	40.18100
H	13.47900	-13.66900	40.06700
H	14.73300	-14.41600	39.66400
O	26.97400	-7.88000	41.18200
H	26.63500	-7.99600	40.29400
H	27.84200	-8.28100	41.16200
O	22.83200	-6.02700	37.17100
H	22.26900	-6.11800	36.40300
H	22.71700	-6.84700	37.65200
O	20.37900	-4.53200	40.01000
H	21.17100	-4.87300	39.59400
H	20.61800	-4.42200	40.93000
O	28.41200	-2.36000	32.48300
H	27.76100	-1.77400	32.09600
H	29.20700	-1.82900	32.54200
O	22.33800	-5.27100	32.88100
H	23.11100	-5.81800	32.74300
H	22.69000	-4.40600	33.09300
O	25.10200	-6.56500	42.67800
H	25.69800	-6.79000	41.96400
H	25.15900	-7.30700	43.28100
O	24.67400	-9.11200	30.51300
H	24.92700	-9.63300	29.75200
H	23.73700	-9.27500	30.61600
O	29.71900	-10.10800	38.32100
H	30.50500	-10.52600	37.97000
H	29.98300	-9.20300	38.48900
O	29.89000	-4.67100	33.02000
H	29.25400	-4.59700	33.73200
H	29.55900	-4.08100	32.34200
O	24.39400	-7.20900	32.71600
H	24.27800	-7.88400	33.38400
H	24.50400	-7.69500	31.89900
O	26.73700	-7.74800	36.42000
H	26.37300	-8.27500	37.13200
H	27.59400	-8.13800	36.24700
O	26.37800	-12.71900	31.41700
H	26.54500	-11.85100	31.78400
H	26.18700	-12.55600	30.49300
O	22.58600	-7.80600	39.11700
H	21.66200	-7.93800	39.32500
H	22.83500	-7.02600	39.61400
O	26.35700	-10.26300	32.40100
H	26.23700	-9.81700	33.24000
H	25.66200	-9.91400	31.84300
O	19.94700	-8.16200	39.95100
H	20.07700	-7.90900	40.86500
H	19.26700	-7.57000	39.63200
O	24.11700	2.50700	36.05000
H	24.04000	3.45800	36.12900
H	23.51300	2.16200	36.70800
O	32.05500	-9.79100	33.22900
H	32.96700	-9.55800	33.05400
H	31.54600	-9.20700	32.66700
O	30.66200	-4.80800	40.62100
H	30.03300	-4.64400	39.91800
H	30.27500	-4.39400	41.39200
O	34.54100	-3.87300	37.97500
H	33.85600	-3.49300	37.42500
H	34.62500	-3.26300	38.70800
O	26.21500	-5.07100	37.88600

H	26.67900	-4.79200	37.09600
H	25.95100	-5.97200	37.70300
O	23.10700	-5.52400	40.82400
H	23.67700	-5.09500	40.18500
H	23.69800	-5.80500	41.52200
O	25.15200	-8.94800	34.69500
H	25.85400	-8.49600	35.16200
H	24.82000	-9.59000	35.32300
O	18.89400	-10.69000	39.99600
H	18.10400	-10.43400	39.52000
H	19.38500	-9.87500	40.10100
O	20.84600	-10.32600	36.95500
H	20.04900	-10.21400	37.47300
H	20.61800	-9.98700	36.08900
O	17.17800	-10.43700	42.42800
H	17.42900	-9.55200	42.69400
H	17.71400	-10.61700	41.65500
O	26.39100	-2.13400	34.77100
H	26.25600	-1.28900	35.19900
H	26.93200	-1.93200	34.00700
O	19.84400	-4.61700	35.34400
H	20.35700	-3.82300	35.49400
H	20.49600	-5.31400	35.27700
O	21.65400	-1.50000	37.42600
H	21.83000	-0.57800	37.23800
H	22.44900	-1.81400	37.85500
O	17.80900	-6.28800	36.12000
H	18.64600	-5.87800	35.90300
H	17.98700	-7.22800	36.09000
O	29.70500	-8.62000	31.80800
H	29.19000	-7.85000	32.04700
H	29.43300	-9.29400	32.43100
O	27.29000	-13.77500	36.86600
H	26.43400	-14.16500	36.68700
H	27.70300	-13.69600	36.00600
O	34.67900	-5.86700	33.74300
H	33.90200	-6.20600	33.29900
H	35.35800	-5.86200	33.06900
O	29.49400	-10.74800	33.63400
H	29.18900	-10.43300	34.48500
H	30.44700	-10.67000	33.67700
O	27.93900	-4.58900	35.28300
H	27.41100	-5.32300	34.97100
H	27.43700	-3.81100	35.03700
O	22.67100	-13.92700	37.64400
H	23.24500	-14.14900	36.91100
H	23.25800	-13.85800	38.39700
O	31.72900	-8.03900	38.45000
H	32.24200	-7.40600	38.95200
H	32.37900	-8.63900	38.08500
O	22.99800	-11.89600	31.66300
H	22.16100	-11.55900	31.98400
H	22.85800	-12.03100	30.72600
O	24.97300	-17.05300	36.37100
H	24.31900	-17.36800	35.74700
H	24.93100	-17.67500	37.09700
O	26.08400	-6.77100	28.28700
H	25.17700	-7.00200	28.48600
H	26.58100	-7.56700	28.47400
O	28.99100	1.93900	31.20600
H	28.31700	2.24200	31.81500
H	29.37700	2.74200	30.85700
O	28.20700	-10.56400	40.69600
H	27.28900	-10.34300	40.53800
H	28.62300	-10.49000	39.83700
O	28.99000	-9.16500	35.82600
H	29.73700	-8.65300	35.51700
H	29.25900	-9.48700	36.68700
O	27.26300	-6.66900	32.80900
H	26.35400	-6.95400	32.90600
H	27.29200	-6.25000	31.94900
O	32.49500	-3.05900	36.57800
H	32.04100	-3.90000	36.63500
H	31.86400	-2.42300	36.91600
O	30.93800	-1.22100	37.51300
H	31.23000	-0.66100	36.79400
H	31.44800	-0.92800	38.26900
O	17.83200	-8.94900	36.79100
H	17.63400	-9.54200	36.06600
H	17.27600	-9.25400	37.50800
O	25.81100	-9.05700	38.91000
H	25.86600	-10.00900	38.83400
H	25.00000	-8.90100	39.39400
O	26.96000	-5.12900	30.14800
H	26.52200	-5.84500	29.68800
H	27.70100	-4.90100	29.58600
O	23.75000	-10.43900	36.66900
H	22.93700	-10.13900	37.07600
H	24.24600	-10.83300	37.38700
O	25.90100	-0.44300	38.60300
H	25.85900	-0.21700	37.67300
H	26.81800	-0.67700	38.75100
O	35.07800	-6.85300	36.20600
H	34.93900	-6.66300	35.27800
H	34.72100	-6.09100	36.66200
O	23.24200	-12.86700	42.28500
H	23.88200	-13.00800	41.58800
H	22.41000	-13.15400	41.90900
O	24.01800	-3.56700	38.39900
H	24.81200	-4.06200	38.19700
H	23.30700	-4.09500	38.03600
O	20.32600	-9.42300	34.47500
H	19.39600	-9.48400	34.69200
H	20.42200	-9.94500	33.67800
O	20.24500	-12.89700	37.06300
H	20.50500	-11.98200	36.95300
H	21.06600	-13.36500	37.21200
O	21.71700	-11.48300	44.23200
H	22.39000	-11.69200	43.58400
H	20.89000	-11.68100	43.79300
O	26.59500	-2.99400	42.07900
H	26.45600	-3.36200	42.95100
H	25.80700	-2.47900	41.90700
O	27.19000	2.10700	33.42100
H	27.36500	1.63600	34.23600
H	26.50300	2.73400	33.64900
O	28.14600	-3.61600	39.46700
H	27.81100	-3.65600	40.36300

H	27.64600	-4.28100	38.99400
O	22.12000	-8.20900	44.99300
H	21.98200	-8.94100	45.59400
H	23.03000	-8.29800	44.71100
O	31.08100	-6.27000	29.74100
H	30.46400	-6.99500	29.64400
H	30.61300	-5.50900	29.39800
O	22.87100	-12.05200	28.86300
H	21.99400	-12.17300	28.49900
H	23.34700	-12.84300	28.61100
O	14.39200	-7.26400	38.95700
H	15.07900	-7.57800	38.36900
H	14.23600	-6.36000	38.68400
O	19.19500	-12.37500	43.38600
H	19.11100	-12.98200	42.65100
H	18.37300	-11.88400	43.38700
O	26.05800	0.30100	36.10900
H	25.27400	0.84300	36.20300
H	26.75700	0.81800	36.50900
O	30.45000	-5.13200	36.32700
H	29.51600	-5.01200	36.15700
H	30.62800	-6.03500	36.06300
O	25.95100	-11.74800	38.48000
H	25.58100	-12.30100	39.16800
H	26.44700	-12.35200	37.92800
O	31.90500	-2.94600	33.86000
H	32.18100	-3.06100	34.77000
H	31.03600	-3.34400	33.82200
O	28.54100	1.30200	37.50400
H	28.72300	0.48500	37.96600
H	29.36400	1.51900	37.06600
O	28.87500	0.22900	34.76500
H	29.40400	-0.28200	34.15200
H	29.13800	-0.08700	35.63000
O	19.37700	-1.75400	42.50900
H	19.96200	-2.50000	42.64100
H	19.17900	-1.44400	43.39200
O	22.23000	1.28100	37.52500
H	21.56100	1.76500	37.04100
H	22.09200	1.52800	38.43900
O	13.96800	-9.18400	41.22900
H	14.00700	-9.71700	42.02300
H	13.46200	-9.71000	40.61000
O	17.59900	-10.45700	34.30500
H	17.89500	-11.36600	34.25100
H	16.69800	-10.47900	33.98300
O	21.12900	-12.38200	40.28700
H	20.40700	-11.76800	40.14800
H	21.91800	-11.84600	40.20900
O	19.32800	-12.77600	34.42800
H	18.99900	-13.65600	34.24600
H	19.68200	-12.83100	35.31600
O	28.57600	-1.14100	38.93800
H	28.42800	-2.06900	39.11900
H	29.40100	-1.11900	38.45400
O	22.44200	-12.48900	34.79600
H	22.78200	-11.96000	34.07400
H	22.88900	-12.15100	35.57200
O	24.42900	-13.99100	32.99500
H	25.15000	-13.61300	32.49200
H	23.80600	-13.27200	33.09900
O	28.57200	-13.28700	33.93100
H	28.78800	-12.40400	33.63000
H	28.09200	-13.67800	33.20200
O	23.31200	-10.32800	40.50800
H	23.16600	-10.01400	41.40100
H	22.83500	-9.71000	39.95500
O	25.17200	-13.33800	40.55600
H	25.15800	-14.23400	40.22000
H	25.88900	-13.33100	41.19000
O	31.96900	0.20100	35.38100
H	32.14300	-0.36400	34.62800
H	32.46200	1.00100	35.19900
O	17.78700	-7.78800	43.44700
H	17.24200	-7.01200	43.57900
H	18.66500	-7.44100	43.28900
O	24.57500	-14.45600	35.82500
H	24.69700	-15.38500	36.02400
H	24.28600	-14.44300	34.91300
O	32.95600	-0.48200	32.48000
H	33.65200	0.13800	32.69900
H	33.29900	-1.33300	32.75600
O	18.57300	-8.66100	46.34000
H	17.83100	-8.51200	46.92600
H	18.25200	-8.40000	45.47700
O	34.85600	-3.03200	34.00900
H	35.38100	-2.69400	33.28400
H	34.76800	-3.96800	33.82500
O	26.54000	3.03500	38.36900
H	26.10000	2.54200	39.06200
H	27.29000	2.49200	38.12800
O	19.01900	-0.77400	38.37100
H	19.84500	-1.07400	37.99100
H	18.66800	-1.54300	38.82000
O	23.98700	-1.42100	40.44100
H	23.36500	-2.08400	40.14100
H	24.65700	-1.39500	39.75800
O	17.22000	-2.85500	39.33000
H	16.38300	-3.28300	39.14800
H	17.19200	-2.66200	40.26700
O	23.54800	-2.87100	33.91700
H	23.69700	-2.18800	33.26300
H	24.35500	-2.89100	34.43200
O	21.30400	-6.84500	34.92300
H	21.05800	-7.74800	35.12100
H	21.47300	-6.84300	33.98100
O	20.57300	-11.11200	32.42100
H	19.88000	-11.03700	31.76500
H	20.32800	-11.87600	32.94400
O	27.04800	-0.37600	31.38800
H	26.47500	0.24300	30.93500
H	27.62100	0.17400	31.92200
O	30.26800	-0.09300	32.51900
H	31.20100	-0.15800	32.72200
H	30.18000	0.74300	32.06000
O	32.89600	-0.77600	29.47900
H	32.81800	-1.11000	30.37300
H	33.71700	-0.28400	29.47800

O	27.43300	-9.04000	28.72900
H	28.37800	-9.17000	28.79800
H	27.05400	-9.77900	29.20600
O	25.61700	-11.11500	28.66500
H	24.71000	-11.41200	28.74100
H	25.89400	-11.41200	27.79800
O	16.53500	-9.79500	39.11500
H	16.21500	-10.64800	38.82200
H	15.98800	-9.58000	39.87100
O	20.31300	-7.07800	42.83200
H	20.53100	-6.14700	42.85100
H	20.72500	-7.43700	43.61700
O	27.23300	-16.57100	34.81900
H	26.42300	-16.91700	35.19500
H	27.92200	-16.88100	35.40800
O	24.38700	-14.25600	28.80600
H	25.24900	-13.93100	29.06600
H	24.57200	-15.04300	28.29300
O	16.40300	-6.17400	41.08400
H	15.71000	-6.58900	40.57100
H	17.15100	-6.13200	40.48800
O	18.31700	-6.24200	38.96100
H	18.29400	-6.14400	38.00900
H	18.62700	-5.39600	39.28200
O	20.97600	-4.18500	43.07500
H	21.93000	-4.21600	43.00800
H	20.77700	-4.64600	43.89000
O	21.56600	-5.61500	45.64300
H	22.38500	-5.12000	45.66600
H	21.84000	-6.53100	45.61400
O	31.32300	-13.22400	35.00500
H	30.43500	-12.93900	34.78700
H	31.73800	-13.38400	34.15800
O	19.33100	1.35700	40.09200
H	19.19600	0.59700	39.52600
H	18.91400	2.08100	39.62500
O	30.46700	-3.45900	29.10000
H	31.17000	-3.25400	29.71600
H	29.74300	-2.89100	29.36500
O	31.29700	-7.70800	35.22000
H	31.24000	-7.25100	34.38100
H	31.84900	-8.46900	35.04300
O	22.48800	2.25400	40.30400
H	22.37100	3.19900	40.20200
H	21.66700	1.95100	40.69300
O	32.37500	-3.02100	31.15000
H	33.21000	-3.45300	30.96800
H	32.21400	-3.19300	32.07800
O	32.03000	-6.51800	32.51600
H	31.97500	-6.68300	31.57400
H	31.40500	-5.81000	32.67200
O	16.81900	-11.28100	46.34200
H	17.46500	-11.79100	46.83000
H	16.61100	-10.54400	46.91600
O	25.34000	-17.01700	32.09400
H	25.32800	-16.07300	32.25100
H	26.23200	-17.20000	31.80000
O	25.26900	1.66000	40.18100
H	24.31800	1.66000	40.06700
H	25.57200	0.91300	39.66400
O	35.51400	6.21800	30.51300
H	35.76600	5.69600	29.75200
H	34.57600	6.05400	30.61600
O	35.23300	8.12000	32.71600
H	35.11700	7.44500	33.38400
H	35.34400	7.63400	31.89900
O	37.21700	2.61000	31.41700
H	37.38400	3.47800	31.78400
H	37.02700	2.77300	30.49300
O	37.19600	5.06600	32.40100
H	37.07600	5.51200	33.24000
H	36.50100	5.41500	31.84300
O	33.83700	3.43300	31.66300
H	33.00100	3.77000	31.98400
H	33.69700	3.29800	30.72600
O	35.81200	-1.72300	36.37100
H	35.15900	-2.03900	35.74700
H	35.77000	-2.34600	37.09700
O	36.92300	8.55800	28.28700
H	36.01600	8.32700	28.48600
H	37.42000	7.76200	28.47400
O	28.67100	6.38000	36.79100
H	28.47300	5.78700	36.06600
H	28.11600	6.07600	37.50800
O	37.80000	10.20100	30.14800
H	37.36200	9.48400	29.68800
H	38.54100	10.42800	29.58600
O	31.16600	5.90700	34.47500
H	30.23500	5.84500	34.69200
H	31.26100	5.38400	33.67800
O	31.08400	2.43200	37.06300
H	31.34500	3.34700	36.95300
H	31.90600	1.96500	37.21200
O	41.92100	9.05900	29.74100
H	41.30400	8.33400	29.64400
H	41.45300	9.82000	29.39800
O	33.71100	3.27800	28.86300
H	32.83400	3.15600	28.49900
H	34.18600	2.48600	28.61100
O	28.43900	4.87200	34.30500
H	28.73400	3.96300	34.25100
H	27.53700	4.85100	33.98300
O	30.16700	2.55300	34.42800
H	29.83900	1.67300	34.24600
H	30.52100	2.49800	35.31600
O	33.28200	2.84000	34.79600
H	33.62100	3.36900	34.07400
H	33.72900	3.17900	35.57200
O	35.26800	1.33800	32.99500
H	35.98900	1.71600	32.49200
H	34.64500	2.05700	33.09900
O	39.41100	2.04200	33.93100
H	39.62800	2.92500	33.63000
H	38.93100	1.65100	33.20200
O	35.41500	0.87300	35.82500
H	35.53600	-0.05600	36.02400
H	35.12500	0.88700	34.91300
O	31.41300	4.21700	32.42100

H	30.71900	4.29200	31.76500
H	31.16800	3.45400	32.94400
O	38.27200	6.28900	28.72900
H	39.21800	6.16000	28.79800
H	37.89300	5.55000	29.20600
O	36.45600	4.21400	28.66500
H	35.54900	3.91700	28.74100
H	36.73400	3.91700	27.79800
O	38.07200	-1.24200	34.81900
H	37.26300	-1.58800	35.19500
H	38.76100	-1.55200	35.40800
O	35.22600	1.07300	28.80600
H	36.08800	1.39800	29.06600
H	35.41100	0.28600	28.29300
O	36.17900	-1.68800	32.09400
H	36.16700	-0.74300	32.25100
H	37.07100	-1.87100	31.80000
O	13.27800	-35.81600	22.77500
H	13.20000	-34.86500	22.85400
H	12.67400	-36.16100	23.43200
O	10.81400	-39.82300	24.15000
H	10.99100	-38.90100	23.96200
H	11.61000	-40.13700	24.58000
O	11.93700	-38.30500	30.15100
H	12.48800	-38.43400	29.37900
H	11.09800	-38.00600	29.80100
O	18.15100	-36.38400	17.93100
H	17.47800	-36.08100	18.53900
H	18.53800	-35.58100	17.58100
O	15.06200	-38.76600	25.32700
H	15.02000	-38.54000	24.39800
H	15.97800	-39.00000	25.47600
O	16.35000	-36.21600	20.14600
H	16.52600	-36.68700	20.96000
H	15.66400	-35.58900	20.37400
O	10.91700	-33.06700	26.92000
H	11.15600	-32.43500	27.59700
H	10.07400	-33.41800	27.20600
O	15.42900	-37.25900	29.32400
H	15.35600	-36.45400	29.83600
H	14.98700	-37.06000	28.49900
O	15.21800	-38.02200	22.83400
H	14.43500	-37.48000	22.92800
H	15.91800	-37.50500	23.23300
O	17.88900	-37.36000	31.48000
H	18.60200	-36.72600	31.40700
H	17.39800	-37.26400	30.66400
O	12.94000	-39.61100	32.30000
H	13.64100	-38.97700	32.45300
H	12.47000	-39.27000	31.54000
O	17.70100	-37.02100	24.22800
H	17.88400	-37.83800	24.69100
H	18.52500	-36.80400	23.79100
O	18.03600	-38.09400	21.49000
H	18.56400	-38.60500	20.87700
H	18.29800	-38.40900	22.35400
O	8.53700	-40.07700	29.23300
H	9.12300	-40.82300	29.36600
H	8.34000	-39.76700	30.11700
O	11.39100	-37.04200	24.24900
H	10.72200	-36.55800	23.76600
H	11.25200	-36.79500	25.16400
O	15.53200	-35.00800	30.84800
H	16.08300	-34.84500	31.61300
H	15.87200	-34.41700	30.17600
O	18.66800	-37.06000	27.83400
H	18.54400	-37.86100	27.32500
H	19.32600	-36.56500	27.34500
O	15.67800	-37.44200	32.86300
H	15.98700	-37.68700	33.73500
H	16.47600	-37.23700	32.37400
O	15.70000	-35.28800	25.09300
H	15.26100	-35.78100	25.78600
H	16.45100	-35.83100	24.85200
O	8.17900	-39.09700	25.09600
H	9.00500	-39.39700	24.71500
H	7.82900	-39.86600	25.54500
O	13.14800	-39.74400	27.16500
H	12.52600	-40.40700	26.86600
H	13.81800	-39.71800	26.48300
O	6.38000	-41.17800	26.05400
H	5.54400	-41.60600	25.87300
H	6.35300	-40.98500	26.99200
O	16.20900	-38.69900	18.11200
H	15.63600	-38.08000	17.65900
H	16.78200	-38.14900	18.64600
O	8.49200	-36.96600	26.81700
H	8.35700	-37.72600	26.25100
H	8.07500	-36.24200	26.35000
O	11.64800	-36.06900	27.02900
H	11.53200	-35.12400	26.92700
H	10.82800	-36.37200	27.41700
O	12.67400	-35.41600	30.24400
H	13.57700	-35.38400	30.56100
H	12.44800	-36.34600	30.25900
O	14.42900	-36.66300	26.90500
H	13.47900	-36.66300	26.79200
H	14.73300	-37.41000	26.38900
O	26.97400	-30.87400	27.90600
H	26.63500	-30.99000	27.01800
H	27.84200	-31.27500	27.88600
O	22.83200	-29.02100	23.89600
H	22.26900	-29.11200	23.12700
H	22.71700	-29.84100	24.37600
O	20.37900	-27.52500	26.73400
H	21.17100	-27.86600	26.31900
H	20.61800	-27.41600	27.65400
O	28.41200	-25.35400	19.20700
H	27.76100	-24.76800	18.82100
H	29.20700	-24.82300	19.26700
O	22.33800	-28.26500	19.60600
H	23.11100	-28.81200	19.46700
H	22.69000	-27.40000	19.81700
O	24.17600	-26.93700	32.81300
H	23.88500	-26.03200	32.69700
H	24.81200	-26.89500	33.52700
O	25.10200	-29.55900	29.40300
H	25.69800	-29.78400	28.68900

H	25.15900	-30.30100	30.00500
O	24.67400	-32.10500	17.23800
H	24.92700	-32.62700	16.47600
H	23.73700	-32.26900	17.34100
O	29.71900	-33.10200	25.04500
H	30.50500	-33.52000	24.69400
H	29.98300	-32.19700	25.21300
O	29.89000	-27.66400	19.74400
H	29.25400	-27.59100	20.45600
H	29.55900	-27.07500	19.06600
O	24.39400	-30.20300	19.44000
H	24.27800	-30.87800	20.10800
H	24.50400	-30.68900	18.62300
O	26.73700	-30.74200	23.14500
H	26.37300	-31.26800	23.85600
H	27.59400	-31.13200	22.97200
O	26.37800	-35.71300	18.14100
H	26.54500	-34.84400	18.50800
H	26.18700	-35.55000	17.21700
O	29.13500	-27.25100	29.80800
H	28.83500	-26.34800	29.69900
H	28.79000	-27.51800	30.66000
O	22.58600	-30.79900	25.84100
H	21.66200	-30.93200	26.04900
H	22.83500	-30.02000	26.33800
O	26.35700	-33.25700	19.12600
H	26.23700	-32.81100	19.96400
H	25.66200	-32.90800	18.56700
O	19.94700	-31.15600	26.67600
H	20.07700	-30.90300	27.59000
H	19.26700	-30.56300	26.35600
O	24.11700	-20.48600	22.77500
H	24.04000	-19.53600	22.85400
H	23.51300	-20.83200	23.43200
O	32.05500	-32.78500	19.95400
H	32.96700	-32.55200	19.77900
H	31.54600	-32.20100	19.39100
O	30.66200	-27.80200	27.34500
H	30.03300	-27.63700	26.64300
H	30.27500	-27.38800	28.11600
O	34.54100	-26.86700	24.70000
H	33.85600	-26.48700	24.14900
H	34.62500	-26.25700	25.43300
O	26.21500	-28.06500	24.61000
H	26.67900	-27.78600	23.82100
H	25.95100	-28.96600	24.42700
O	23.10700	-28.51700	27.54800
H	23.67700	-28.08900	26.91000
H	23.69800	-28.79900	28.24700
O	25.15200	-31.94200	21.41900
H	25.85400	-31.49000	21.88700
H	24.82000	-32.58400	22.04700
O	18.89400	-33.68400	26.72100
H	18.10400	-33.42800	26.24500
H	19.38500	-32.86900	26.82600
O	20.84600	-33.31900	23.67900
H	20.04900	-33.20800	24.19800
H	20.61800	-32.98100	22.81300
O	26.67600	-26.79500	31.62700
H	26.74100	-26.33300	32.46300
H	25.96700	-27.42500	31.75600
O	17.17800	-33.43100	29.15200
H	17.42900	-32.54600	29.41800
H	17.71400	-33.61000	28.38000
O	26.39100	-25.12800	21.49500
H	26.25600	-24.28300	21.92400
H	26.93200	-24.92600	20.73200
O	19.84400	-27.61100	22.06800
H	20.35700	-26.81600	22.21900
H	20.49600	-28.30800	22.00100
O	21.65400	-24.49400	24.15000
H	21.83000	-23.57200	23.96200
H	22.44900	-24.80800	24.58000
O	22.77700	-22.97600	30.15100
H	23.32700	-23.10500	29.37900
H	21.93800	-22.67700	29.80100
O	17.80900	-29.28200	22.84400
H	18.64600	-28.87200	22.62700
H	17.98700	-30.22200	22.81400
O	29.70500	-31.61400	18.53200
H	29.19000	-30.84400	18.77100
H	29.43300	-32.28800	19.15600
O	34.67900	-28.86100	20.46800
H	33.90200	-29.20000	20.02300
H	35.35800	-28.85600	19.79300
O	29.49400	-33.74200	20.35800
H	29.18900	-33.42700	21.20900
H	30.44700	-33.66300	20.40100
O	27.93900	-27.58300	22.00800
H	27.41100	-28.31700	21.69500
H	27.43700	-26.80500	21.76200
O	22.67100	-36.92100	24.36800
H	23.24500	-37.14300	23.63600
H	23.25800	-36.85200	25.12100
O	31.72900	-31.03300	25.17500
H	32.24200	-30.40000	25.67700
H	32.37900	-31.63300	24.80900
O	22.99800	-34.89000	18.38800
H	22.16100	-34.55300	18.70900
H	22.85800	-35.02500	17.45100
O	26.08400	-29.76500	15.01100
H	25.17700	-29.99600	15.21000
H	26.58100	-30.56100	15.19900
O	28.99100	-21.05500	17.93100
H	28.31700	-20.75200	18.53900
H	29.37700	-20.25200	17.58100
O	28.20700	-33.55800	27.42100
H	27.28900	-33.33700	27.26300
H	28.62300	-33.48400	26.56200
O	28.99000	-32.15900	22.55100
H	29.73700	-31.64700	22.24200
H	29.25900	-32.48100	23.41100
O	27.26300	-29.66300	19.53400
H	26.35400	-29.94700	19.63100
H	27.29200	-29.24300	18.67400
O	32.49500	-26.05300	23.30300
H	32.04100	-26.89300	23.35900
H	31.86400	-25.41600	23.64000

O	30.93800	-24.21500	24.23800
H	31.23000	-23.65400	23.51900
H	31.44800	-23.92200	24.99300
O	17.83200	-31.94300	23.51500
H	17.63400	-32.53600	22.79000
H	17.27600	-32.24700	24.23300
O	25.81100	-32.05000	25.63500
H	25.86600	-33.00300	25.55900
H	25.00000	-31.89400	26.11900
O	26.96000	-28.12200	16.87200
H	26.52200	-28.83900	16.41300
H	27.70100	-27.89500	16.31100
O	23.75000	-33.43300	23.39400
H	22.93700	-33.13300	23.80000
H	24.24600	-33.82700	24.11100
O	25.90100	-23.43700	25.32700
H	25.85900	-23.21100	24.39800
H	26.81800	-23.67100	25.47600
O	35.07800	-29.84700	22.93000
H	34.93900	-29.65600	22.00200
H	34.72100	-29.08500	23.38700
O	23.24200	-35.86100	29.01000
H	23.88200	-36.00200	28.31200
H	22.41000	-36.14700	28.63400
O	24.01800	-26.56100	25.12300
H	24.81200	-27.05600	24.92200
H	23.30700	-27.08900	24.76000
O	28.83700	-24.17900	29.47400
H	28.89100	-23.61000	30.24200
H	27.96900	-24.00900	29.11000
O	20.32600	-32.41600	21.19900
H	19.39600	-32.47800	21.41600
H	20.42200	-32.93900	20.40300
O	20.24500	-35.89100	23.78700
H	20.50500	-34.97600	23.67800
H	21.06600	-36.35800	23.93700
O	21.71700	-34.47700	30.95700
H	22.39000	-34.68500	30.30800
H	20.89000	-34.67500	30.51700
O	26.59500	-25.98800	28.80300
H	26.45600	-26.35600	29.67600
H	25.80700	-25.47300	28.63100
O	27.19000	-20.88700	20.14600
H	27.36500	-21.35800	20.96000
H	26.50300	-20.26000	20.37400
O	28.14600	-26.61000	26.19200
H	27.81100	-26.64900	27.08800
H	27.64600	-27.27500	25.71900
O	22.12000	-31.20300	31.71700
H	21.98200	-31.93500	32.31800
H	23.03000	-31.29200	31.43600
O	31.08100	-29.26400	16.46600
H	30.46400	-29.98900	16.36900
H	30.61300	-28.50300	16.12200
O	21.75600	-17.73800	26.92000
H	21.99600	-17.10600	27.59700
H	20.91300	-18.08900	27.20600
O	22.87100	-35.04500	15.58800
H	21.99400	-35.16700	15.22400
H	23.34700	-35.83700	15.33600
O	14.39200	-30.25800	25.68200
H	15.07900	-30.57200	25.09400
H	14.23600	-29.35400	25.40900
O	19.19500	-35.36900	30.11000
H	19.11100	-35.97600	29.37500
H	18.37300	-34.87800	30.11100
O	26.26900	-21.93000	29.32400
H	26.19600	-21.12400	29.83600
H	25.82600	-21.73100	28.49900
O	26.05800	-22.69300	22.83400
H	25.27400	-22.15100	22.92800
H	26.75700	-22.17500	23.23300
O	28.72900	-22.03100	31.48000
H	29.44100	-21.39700	31.40700
H	28.23800	-21.93500	30.66400
O	30.45000	-28.12600	23.05100
H	29.51600	-28.00600	22.88200
H	30.62800	-29.02900	22.78700
O	25.95100	-34.74200	25.20500
H	25.58100	-35.29400	25.89300
H	26.44700	-35.34600	24.65200
O	23.78000	-24.28200	32.30000
H	24.48100	-23.64800	32.45300
H	23.30900	-23.94000	31.54000
O	31.90500	-25.94000	20.58500
H	32.18100	-26.05400	21.49400
H	31.03600	-26.33800	20.54600
O	28.54100	-21.69100	24.22800
H	28.72300	-22.50900	24.69100
H	29.36400	-21.47500	23.79100
O	28.87500	-22.76500	21.49000
H	29.40400	-23.27500	20.87700
H	29.13800	-23.08000	22.35400
O	19.37700	-24.74800	29.23300
H	19.96200	-25.49400	29.36600
H	19.17900	-24.43800	30.11700
O	22.23000	-21.71300	24.24900
H	21.56100	-21.22800	23.76600
H	22.09200	-21.46600	25.16400
O	13.96800	-32.17800	27.95400
H	14.00700	-32.71100	28.74700
H	13.46200	-32.70400	27.33500
O	26.37100	-19.67900	30.84800
H	26.92300	-19.51600	31.61300
H	26.71200	-19.08800	30.17600
O	17.59900	-33.45100	21.02900
H	17.89500	-34.36000	20.97600
H	16.69800	-33.47200	20.70700
O	29.50800	-21.73000	27.83400
H	29.38300	-22.53200	27.32500
H	30.16600	-21.23600	27.34500
O	21.12900	-35.37600	27.01200
H	20.40700	-34.76200	26.87300
H	21.91800	-34.84000	26.93300
O	31.57100	-23.93000	29.46200
H	30.62400	-24.04900	29.39000
H	31.92000	-24.81700	29.54500
O	19.32800	-35.77000	21.15300

H	18.99900	-36.65000	20.97000
H	19.68200	-35.82500	22.04100
O	28.57600	-24.13400	25.66200
H	28.42800	-25.06300	25.84300
H	29.40100	-24.11200	25.17800
O	22.44200	-35.48200	21.52100
H	22.78200	-34.95400	20.79800
H	22.88900	-35.14400	22.29600
O	32.17900	-23.31100	26.74900
H	33.10400	-23.51700	26.61100
H	32.01100	-23.56000	27.65800
O	24.42900	-36.98500	19.72000
H	25.15000	-36.60700	19.21600
H	23.80600	-36.26600	19.82300
O	23.31200	-33.32200	27.23300
H	23.16600	-33.00800	28.12500
H	22.83500	-32.70300	26.68000
O	31.96900	-22.79300	22.10500
H	32.14300	-23.35800	21.35300
H	32.46200	-21.99300	21.92300
O	17.78700	-30.78100	30.17200
H	17.24200	-30.00500	30.30400
H	18.66500	-30.43400	30.01300
O	32.95600	-23.47600	19.20500
H	33.65200	-22.85600	19.42400
H	33.29900	-24.32700	19.48000
O	18.57300	-31.65400	33.06500
H	17.83100	-31.50600	33.65100
H	18.25200	-31.39400	32.20100
O	34.85600	-26.02600	20.73400
H	35.38100	-25.68800	20.00900
H	34.76800	-26.96200	20.55000
O	26.51800	-22.11300	32.86300
H	26.82600	-22.35800	33.73500
H	27.31500	-21.90800	32.37400
O	26.54000	-19.95900	25.09300
H	26.10000	-20.45100	25.78600
H	27.29000	-20.50200	24.85200
O	19.01900	-23.76800	25.09600
H	19.84500	-24.06800	24.71500
H	18.66800	-24.53700	25.54500
O	23.98700	-24.41500	27.16500
H	23.36500	-25.07800	26.86600
H	24.65700	-24.38900	26.48300
O	17.22000	-25.84900	26.05400
H	16.38300	-26.27700	25.87300
H	17.19200	-25.65600	26.99200
O	23.54800	-25.86500	20.64100
H	23.69700	-25.18100	19.98800
H	24.35500	-25.88400	21.15600
O	21.30400	-29.83800	21.64700
H	21.05800	-30.74200	21.84500
H	21.47300	-29.83700	20.70500
O	20.57300	-34.10500	19.14600
H	19.88000	-34.03100	18.49000
H	20.32800	-34.86900	19.66800
O	27.04800	-23.37000	18.11200
H	26.47500	-22.75100	17.65900
H	27.62100	-22.82000	18.64600
O	30.26800	-23.08600	19.24300
H	31.20100	-23.15200	19.44700
H	30.18000	-22.25100	18.78500
O	32.89600	-23.77000	16.20400
H	32.81800	-24.10400	17.09700
H	33.71700	-23.27800	16.20300
O	27.43300	-32.03400	15.45400
H	28.37800	-32.16300	15.52300
H	27.05400	-32.77200	15.93100
O	25.61700	-34.10900	15.38900
H	24.71000	-34.40600	15.46600
H	25.89400	-34.40600	14.52300
O	16.53500	-32.78800	25.83900
H	16.21500	-33.64200	25.54700
H	15.98800	-32.57300	26.59500
O	20.31300	-30.07200	29.55600
H	20.53100	-29.14000	29.57500
H	20.72500	-30.43100	30.34200
O	16.40300	-29.16800	27.80900
H	15.71000	-29.58300	27.29600
H	17.15100	-29.12600	27.21200
O	18.31700	-29.23600	25.68500
H	18.29400	-29.13700	24.73300
H	18.62700	-28.39000	26.00700
O	20.97600	-27.17900	29.80000
H	21.93000	-27.21000	29.73300
H	20.77700	-27.64000	30.61500
O	21.56600	-28.60800	32.36700
H	22.38500	-28.11400	32.39100
H	21.84000	-29.52500	32.33900
O	19.33100	-21.63700	26.81700
H	19.19600	-22.39700	26.25100
H	18.91400	-20.91300	26.35000
O	30.46700	-26.45300	15.82500
H	31.17000	-26.24800	16.44000
H	29.74300	-25.88500	16.09000
O	31.29700	-30.70200	21.94500
H	31.24000	-30.24500	21.10600
H	31.84900	-31.46300	21.76700
O	22.48800	-20.74000	27.02900
H	22.37100	-19.79500	26.92700
H	21.66700	-21.04200	27.41700
O	32.37500	-26.01500	17.87400
H	33.21000	-26.44700	17.69200
H	32.21400	-26.18600	18.80200
O	32.03000	-29.51100	19.24000
H	31.97500	-29.67700	18.29900
H	31.40500	-28.80400	19.39600
O	16.81900	-34.27500	33.06600
H	17.46500	-34.78500	33.55500
H	16.61100	-33.53800	33.64100
O	23.51300	-20.08700	30.24400
H	24.41600	-20.05500	30.56100
H	23.28700	-21.01700	30.25900
O	25.26900	-21.33400	26.90500
H	24.31800	-21.33400	26.79200
H	25.57200	-22.08000	26.38900
O	37.81300	-15.54500	27.90600
H	37.47500	-15.66100	27.01800

H	38.68200	-15.94600	27.88600
O	33.67200	-13.69100	23.89600
H	33.10800	-13.78200	23.12700
H	33.55700	-14.51100	24.37600
O	31.21800	-12.19600	26.73400
H	32.01000	-12.53700	26.31900
H	31.45800	-12.08700	27.65400
O	39.25200	-10.02400	19.20700
H	38.60000	-9.43900	18.82100
H	40.04600	-9.49400	19.26700
O	33.17700	-12.93500	19.60600
H	33.95000	-13.48300	19.46700
H	33.53000	-12.07100	19.81700
O	35.01600	-11.60800	32.81300
H	34.72500	-10.70300	32.69700
H	35.65100	-11.56600	33.52700
O	35.94100	-14.23000	29.40300
H	36.53800	-14.45400	28.68900
H	35.99900	-14.97200	30.00500
O	35.51400	-16.77600	17.23800
H	35.76600	-17.29800	16.47600
H	34.57600	-16.94000	17.34100
O	40.55800	-17.77300	25.04500
H	41.34400	-18.19100	24.69400
H	40.82200	-16.86800	25.21300
O	40.72900	-12.33500	19.74400
H	40.09400	-12.26200	20.45600
H	40.39900	-11.74600	19.06600
O	35.23300	-14.87300	19.44000
H	35.11700	-15.54900	20.10800
H	35.34400	-15.36000	18.62300
O	37.57700	-15.41200	23.14500
H	37.21200	-15.93900	23.85600
H	38.43300	-15.80200	22.97200
O	37.21700	-20.38300	18.14100
H	37.38400	-19.51500	18.50800
H	37.02700	-20.22100	17.21700
O	39.97500	-11.92200	29.80800
H	39.67500	-11.01900	29.69900
H	39.63000	-12.18800	30.66000
O	33.42600	-15.47000	25.84100
H	32.50100	-15.60300	26.04900
H	33.67400	-14.69100	26.33800
O	37.19600	-17.92800	19.12600
H	37.07600	-17.48200	19.96400
H	36.50100	-17.57900	18.56700
O	30.78700	-15.82700	26.67600
H	30.91600	-15.57300	27.59000
H	30.10600	-15.23400	26.35600
O	34.95700	-5.15700	22.77500
H	34.87900	-4.20600	22.85400
H	34.35300	-5.50300	23.43200
O	41.50100	-12.47300	27.34500
H	40.87200	-12.30800	26.64300
H	41.11400	-12.05900	28.11600
O	37.05400	-12.73500	24.61000
H	37.51800	-12.45700	23.82100
H	36.79000	-13.63700	24.42700
O	33.94700	-13.18800	27.54800
H	34.51700	-12.76000	26.91000
H	34.53700	-13.47000	28.24700
O	35.99100	-16.61300	21.41900
H	36.69400	-16.16100	21.88700
H	35.65900	-17.25500	22.04700
O	29.73400	-18.35500	26.72100
H	28.94400	-18.09900	26.24500
H	30.22500	-17.54000	26.82600
O	31.68500	-17.99000	23.67900
H	30.88800	-17.87800	24.19800
H	31.45800	-17.65200	22.81300
O	37.51600	-11.46600	31.62700
H	37.58000	-11.00400	32.46300
H	36.80700	-12.09600	31.75600
O	28.01700	-18.10200	29.15200
H	28.26800	-17.21700	29.41800
H	28.55300	-18.28100	28.38000
O	37.23000	-9.79900	21.49500
H	37.09500	-8.95400	21.92400
H	37.77100	-9.59700	20.73200
O	30.68300	-12.28100	22.06800
H	31.19600	-11.48700	22.21900
H	31.33600	-12.97900	22.00100
O	32.49300	-9.16500	24.15000
H	32.67000	-8.24300	23.96200
H	33.28900	-9.47900	24.58000
O	33.61600	-7.64700	30.15100
H	34.16700	-7.77500	29.37900
H	32.77700	-7.34800	29.80100
O	28.64800	-13.95300	22.84400
H	29.48600	-13.54300	22.62700
H	28.82600	-14.89300	22.81400
O	40.54500	-16.28500	18.53200
H	40.02900	-15.51500	18.77100
H	40.27300	-16.95900	19.15600
O	38.12900	-21.44000	23.59100
H	37.27400	-21.82900	23.41200
H	38.54200	-21.36100	22.73100
O	40.33300	-18.41200	20.35800
H	40.02800	-18.09800	21.20900
H	41.28600	-18.33400	20.40100
O	38.77800	-12.25300	22.00800
H	38.25000	-12.98800	21.69500
H	38.27700	-11.47600	21.76200
O	33.51000	-21.59100	24.36800
H	34.08400	-21.81400	23.63600
H	34.09700	-21.52200	25.12100
O	33.83700	-19.56100	18.38800
H	33.00100	-19.22300	18.70900
H	33.69700	-19.69600	17.45100
O	35.81200	-24.71700	23.09600
H	35.15900	-25.03200	22.47200
H	35.77000	-25.34000	23.82200
O	36.92300	-14.43500	15.01100
H	36.01600	-14.66600	15.21000
H	37.42000	-15.23200	15.19900
O	39.83000	-5.72600	17.93100
H	39.15600	-5.42200	18.53900
H	40.21700	-4.92300	17.58100

O	39.04600	-18.22900	27.42100
H	38.12800	-18.00800	27.26300
H	39.46200	-18.15400	26.56200
O	39.82900	-16.83000	22.55100
H	40.57700	-16.31800	22.24200
H	40.09900	-17.15200	23.41100
O	38.10200	-14.33400	19.53400
H	37.19300	-14.61800	19.63100
H	38.13200	-13.91400	18.67400
O	41.77700	-8.88600	24.23800
H	42.07000	-8.32500	23.51900
H	42.28700	-8.59300	24.99300
O	28.67100	-16.61400	23.51500
H	28.47300	-17.20700	22.79000
H	28.11600	-16.91800	24.23300
O	36.65100	-16.72100	25.63500
H	36.70500	-17.67400	25.55900
H	35.84000	-16.56500	26.11900
O	37.80000	-12.79300	16.87200
H	37.36200	-13.51000	16.41300
H	38.54100	-12.56600	16.31100
O	34.59000	-18.10300	23.39400
H	33.77700	-17.80400	23.80000
H	35.08600	-18.49800	24.11100
O	36.74100	-8.10800	25.32700
H	36.69800	-7.88200	24.39800
H	37.65700	-8.34100	25.47600
O	34.08100	-20.53200	29.01000
H	34.72100	-20.67300	28.31200
H	33.24900	-20.81800	28.63400
O	34.85700	-11.23100	25.12300
H	35.65100	-11.72600	24.92200
H	34.14600	-11.76000	24.76000
O	39.67700	-8.85000	29.47400
H	39.73000	-8.28100	30.24200
H	38.80800	-8.67900	29.11000
O	31.16600	-17.08700	21.19900
H	30.23500	-17.14900	21.41600
H	31.26100	-17.61000	20.40300
O	31.08400	-20.56200	23.78700
H	31.34500	-19.64700	23.67800
H	31.90600	-21.02900	23.93700
O	32.55700	-19.14800	30.95700
H	33.22900	-19.35600	30.30800
H	31.73000	-19.34600	30.51700
O	37.43400	-10.65900	28.80300
H	37.29600	-11.02700	29.67600
H	36.64600	-10.14400	28.63100
O	38.02900	-5.55800	20.14600
H	38.20400	-6.02900	20.96000
H	37.34300	-4.93100	20.37400
O	38.98500	-11.28000	26.19200
H	38.65000	-11.32000	27.08800
H	38.48500	-11.94600	25.71900
O	32.95900	-15.87400	31.71700
H	32.82100	-16.60600	32.31800
H	33.87000	-15.96300	31.43600
O	41.92100	-13.93500	16.46600
H	41.30400	-14.66000	16.36900
H	41.45300	-13.17400	16.12200
O	32.59500	-2.40900	26.92000
H	32.83500	-1.77700	27.59700
H	31.75200	-2.76000	27.20600
O	33.71100	-19.71600	15.58800
H	32.83400	-19.83700	15.22400
H	34.18600	-20.50800	15.33600
O	25.23100	-14.92800	25.68200
H	25.91800	-15.24200	25.09400
H	25.07600	-14.02400	25.40900
O	30.03400	-20.03900	30.11000
H	29.95000	-20.64700	29.37500
H	29.21300	-19.54900	30.11100
O	37.10800	-6.60100	29.32400
H	37.03500	-5.79500	29.83600
H	36.66600	-6.40200	28.49900
O	36.89700	-7.36400	22.83400
H	36.11400	-6.82100	22.92800
H	37.59600	-6.84600	23.23300
O	39.56800	-6.70200	31.48000
H	40.28100	-6.06700	31.40700
H	39.07700	-6.60600	30.66400
O	41.29000	-12.79700	23.05100
H	40.35500	-12.67700	22.88200
H	41.46700	-13.70000	22.78700
O	36.79000	-19.41200	25.20500
H	36.42000	-19.96500	25.89300
H	37.28600	-20.01700	24.65200
O	34.61900	-8.95300	32.30000
H	35.32000	-8.31900	32.45300
H	34.14900	-8.61100	31.54000
O	42.74500	-10.61100	20.58500
H	43.02100	-10.72500	21.49400
H	41.87500	-11.00900	20.54600
O	39.38000	-6.36200	24.22800
H	39.56200	-7.18000	24.69100
H	40.20300	-6.14500	23.79100
O	39.71400	-7.43500	21.49000
H	40.24300	-7.94600	20.87700
H	39.97700	-7.75100	22.35400
O	30.21600	-9.41900	29.23300
H	30.80100	-10.16500	29.36600
H	30.01800	-9.10900	30.11700
O	33.07000	-6.38400	24.24900
H	32.40100	-5.89900	23.76600
H	32.93100	-6.13700	25.16400
O	24.80800	-16.84800	27.95400
H	24.84600	-17.38200	28.74700
H	24.30200	-17.37500	27.33500
O	37.21000	-4.34900	30.84800
H	37.76200	-4.18600	31.61300
H	37.55100	-3.75900	30.17600
O	28.43900	-18.12200	21.02900
H	28.73400	-19.03100	20.97600
H	27.53700	-18.14300	20.70700
O	40.34700	-6.40100	27.83400
H	40.22200	-7.20200	27.32500
H	41.00500	-5.90700	27.34500
O	31.96800	-20.04600	27.01200

H	31.24700	-19.43300	26.87300
H	32.75700	-19.51100	26.93300
O	42.41100	-8.60000	29.46200
H	41.46400	-8.72000	29.39000
H	42.75900	-9.48800	29.54500
O	30.16700	-20.44100	21.15300
H	29.83900	-21.32100	20.97000
H	30.52100	-20.49600	22.04100
O	39.41500	-8.80500	25.66200
H	39.26800	-9.73300	25.84300
H	40.24100	-8.78300	25.17800
O	33.28200	-20.15300	21.52100
H	33.62100	-19.62500	20.79800
H	33.72900	-19.81500	22.29600
O	35.26800	-21.65600	19.72000
H	35.98900	-21.27800	19.21600
H	34.64500	-20.93700	19.82300
O	39.41100	-20.95100	20.65600
H	39.62800	-20.06900	20.35500
H	38.93100	-21.34300	19.92600
O	34.15100	-17.99300	27.23300
H	34.00600	-17.67900	28.12500
H	33.67400	-17.37400	26.68000
O	36.01100	-21.00300	27.28100
H	35.99700	-21.89800	26.94400
H	36.72800	-20.99500	27.91500
O	28.62600	-15.45200	30.17200
H	28.08100	-14.67600	30.30400
H	29.50400	-15.10500	30.01300
O	35.41500	-22.12100	22.55000
H	35.53600	-23.04900	22.74900
H	35.12500	-22.10700	21.63700
O	29.41300	-16.32500	33.06500
H	28.67100	-16.17700	33.65100
H	29.09200	-16.06400	32.20100
O	37.35700	-6.78400	32.86300
H	37.66600	-7.02900	33.73500
H	38.15400	-6.57900	32.37400
O	37.37900	-4.63000	25.09300
H	36.93900	-5.12200	25.78600
H	38.13000	-5.17300	24.85200
O	29.85800	-8.43900	25.09600
H	30.68400	-8.73800	24.71500
H	29.50800	-9.20800	25.54500
O	34.82600	-9.08600	27.16500
H	34.20400	-9.74900	26.86600
H	35.49700	-9.06000	26.48300
O	28.05900	-10.52000	26.05400
H	27.22200	-10.94800	25.87300
H	28.03100	-10.32700	26.99200
O	34.38800	-10.53600	20.64100
H	34.53700	-9.85200	19.98800
H	35.19400	-10.55500	21.15600
O	32.14300	-14.50900	21.64700
H	31.89800	-15.41300	21.84500
H	32.31300	-14.50700	20.70500
O	31.41300	-18.77600	19.14600
H	30.71900	-18.70200	18.49000
H	31.16800	-19.54000	19.66800
O	37.88700	-8.04100	18.11200
H	37.31500	-7.42200	17.65900
H	38.46100	-7.49100	18.64600
O	41.10700	-7.75700	19.24300
H	42.04000	-7.82300	19.44700
H	41.01900	-6.92100	18.78500
O	38.27200	-16.70500	15.45400
H	39.21800	-16.83400	15.52300
H	37.89300	-17.44300	15.93100
O	36.45600	-18.78000	15.38900
H	35.54900	-19.07600	15.46600
H	36.73400	-19.07700	14.52300
O	27.37400	-17.45900	25.83900
H	27.05400	-18.31300	25.54700
H	26.82700	-17.24400	26.59500
O	31.15200	-14.74300	29.55600
H	31.37100	-13.81100	29.57500
H	31.56500	-15.10200	30.34200
O	38.07200	-24.23600	21.54400
H	37.26300	-24.58100	21.91900
H	38.76100	-24.54500	22.13300
O	35.22600	-21.92100	15.53100
H	36.08800	-21.59600	15.79000
H	35.41100	-22.70800	15.01800
O	27.24300	-13.83900	27.80900
H	26.54900	-14.25300	27.29600
H	27.99000	-13.79700	27.21200
O	29.15700	-13.90700	25.68500
H	29.13400	-13.80800	24.73300
H	29.46700	-13.06000	26.00700
O	31.81500	-11.84900	29.80000
H	32.77000	-11.88100	29.73300
H	31.61700	-12.31000	30.61500
O	32.40500	-13.27900	32.36700
H	33.22500	-12.78500	32.39100
H	32.67900	-14.19600	32.33900
O	42.16200	-20.88800	21.73000
H	41.27400	-20.60300	21.51200
H	42.57700	-21.04900	20.88300
O	30.17000	-6.30700	26.81700
H	30.03600	-7.06700	26.25100
H	29.75400	-5.58300	26.35000
O	41.30600	-11.12400	15.82500
H	42.01000	-10.91900	16.44000
H	40.58200	-10.55600	16.09000
O	42.13600	-15.37300	21.94500
H	42.07900	-14.91600	21.10600
H	42.68900	-16.13400	21.76700
O	33.32700	-5.41000	27.02900
H	33.21000	-4.46600	26.92700
H	32.50600	-5.71300	27.41700
O	42.86900	-14.18200	19.24000
H	42.81400	-14.34800	18.29900
H	42.24400	-13.47500	19.39600
O	27.65900	-18.94500	33.06600
H	28.30500	-19.45600	33.55500
H	27.45000	-18.20900	33.64100
O	36.17900	-24.68100	18.81900
H	36.16700	-23.73700	18.97500

H	37.07100	-24.86400	18.52500
O	34.35300	-4.75800	30.24400
H	35.25500	-4.72500	30.56100
H	34.12600	-5.68800	30.25900
O	36.10800	-6.00500	26.90500
H	35.15800	-6.00400	26.79200
H	36.41100	-6.75100	26.38900
O	42.05700	3.13300	26.73400
H	42.85000	2.79200	26.31900
H	42.29700	3.24200	27.65400
O	41.62600	-0.49800	26.67600
H	41.75500	-0.24400	27.59000
H	40.94600	0.09500	26.35600
O	40.57300	-3.02600	26.72100
H	39.78300	-2.77000	26.24500
H	41.06400	-2.21100	26.82600
O	42.52500	-2.66100	23.67900
H	41.72800	-2.54900	24.19800
H	42.29700	-2.32200	22.81300
O	38.85700	-2.77200	29.15200
H	39.10800	-1.88800	29.41800
H	39.39300	-2.95200	28.38000
O	41.52300	3.04800	22.06800
H	42.03500	3.84200	22.21900
H	42.17500	2.35000	22.00100
O	39.48800	1.37700	22.84400
H	40.32500	1.78600	22.62700
H	39.66600	0.43700	22.81400
O	39.51000	-1.28400	23.51500
H	39.31300	-1.87700	22.79000
H	38.95500	-1.58900	24.23300
O	42.00500	-1.75800	21.19900
H	41.07500	-1.81900	21.41600
H	42.10100	-2.28000	20.40300
O	41.92300	-5.23200	23.78700
H	42.18400	-4.31800	23.67800
H	42.74500	-5.70000	23.93700
O	36.07000	0.40100	25.68200
H	36.75700	0.08700	25.09400
H	35.91500	1.30500	25.40900
O	40.87400	-4.71000	30.11000
H	40.79000	-5.31800	29.37500
H	40.05200	-4.21900	30.11100
O	41.05500	5.91000	29.23300
H	41.64100	5.16500	29.36600
H	40.85800	6.22100	30.11700
O	35.64700	-1.51900	27.95400
H	35.68600	-2.05300	28.74700
H	35.14100	-2.04600	27.33500
O	39.27800	-2.79300	21.02900
H	39.57300	-3.70200	20.97600
H	38.37700	-2.81400	20.70700
O	41.00700	-5.11100	21.15300
H	40.67800	-5.99200	20.97000
H	41.36100	-5.16700	22.04100
O	39.46500	-0.12300	30.17200
H	38.92100	0.65300	30.30400
H	40.34300	0.22400	30.01300
O	40.25200	-0.99600	33.06500
H	39.51000	-0.84800	33.65100
H	39.93100	-0.73500	32.20100
O	40.69800	6.89100	25.09600
H	41.52300	6.59100	24.71500
H	40.34700	6.12100	25.54500
O	38.89800	4.80900	26.05400
H	38.06200	4.38100	25.87300
H	38.87100	5.00200	26.99200
O	42.25200	-3.44700	19.14600
H	41.55900	-3.37300	18.49000
H	42.00700	-4.21100	19.66800
O	38.21300	-2.13000	25.83900
H	37.89400	-2.98300	25.54700
H	37.66700	-1.91500	26.59500
O	41.99100	0.58600	29.55600
H	42.21000	1.51800	29.57500
H	42.40400	0.22700	30.34200
O	38.08200	1.49000	27.80900
H	37.38900	1.07600	27.29600
H	38.83000	1.53200	27.21200
O	39.99600	1.42200	25.68500
H	39.97300	1.52100	24.73300
H	40.30600	2.26900	26.00700
O	41.01000	9.02200	26.81700
H	40.87500	8.26200	26.25100
H	40.59300	9.74600	26.35000
O	38.49800	-3.61600	33.06600
H	39.14400	-4.12600	33.55500
H	38.29000	-2.87900	33.64100
O	34.95700	-28.15100	9.49900
H	34.87900	-27.20000	9.57800
H	34.35300	-28.49600	10.15700
O	32.49300	-32.15900	10.87500
H	32.67000	-31.23700	10.68700
H	33.28900	-32.47300	11.30400
O	33.61600	-30.64100	16.87600
H	34.16700	-30.76900	16.10400
H	32.77700	-30.34100	16.52500
O	39.83000	-28.71900	4.65500
H	39.15600	-28.41600	5.26400
H	40.21700	-27.91600	4.30600
O	36.74100	-31.10100	12.05200
H	36.69800	-30.87500	11.12200
H	37.65700	-31.33500	12.20000
O	38.02900	-28.55100	6.87000
H	38.20400	-29.02300	7.68500
H	37.34300	-27.92500	7.09800
O	32.59500	-25.40200	13.64400
H	32.83500	-24.77100	14.32200
H	31.75200	-25.75400	13.93100
O	37.10800	-29.59500	16.04800
H	37.03500	-28.78900	16.56000
H	36.66600	-29.39500	15.22300
O	36.89700	-30.35700	9.55900
H	36.11400	-29.81500	9.65200
H	37.59600	-29.84000	9.95800
O	39.56800	-29.69600	18.20400
H	40.28100	-29.06100	18.13200
H	39.07700	-29.59900	17.38800

O	34.61900	-31.94600	19.02500
H	35.32000	-31.31300	19.17800
H	34.14900	-31.60500	18.26400
O	39.38000	-29.35600	10.95300
H	39.56200	-30.17400	11.41500
H	40.20300	-29.13900	10.51500
O	39.71400	-30.42900	8.21400
H	40.24300	-30.94000	7.60100
H	39.97700	-30.74500	9.07900
O	30.21600	-32.41300	15.95800
H	30.80100	-33.15800	16.09100
H	30.01800	-32.10200	16.84100
O	33.07000	-29.37800	10.97400
H	32.40100	-28.89300	10.49000
H	32.93100	-29.13100	11.88800
O	37.21000	-27.34300	17.57200
H	37.76200	-27.18000	18.33700
H	37.55100	-26.75300	16.90000
O	40.34700	-29.39500	14.55900
H	40.22200	-30.19600	14.05000
H	41.00500	-28.90000	14.07000
O	37.35700	-29.77700	19.58700
H	37.66600	-30.02300	20.45900
H	38.15400	-29.57300	19.09900
O	37.37900	-27.62400	11.81800
H	36.93900	-28.11600	12.51100
H	38.13000	-28.16600	11.57700
O	29.85800	-31.43200	11.82000
H	30.68400	-31.73200	11.44000
H	29.50800	-32.20200	12.26900
O	34.82600	-32.08000	13.89000
H	34.20400	-32.74300	13.59000
H	35.49700	-32.05400	13.20700
O	28.05900	-33.51400	12.77900
H	27.22200	-33.94200	12.59700
H	28.03100	-33.32100	13.71600
O	37.88700	-31.03500	4.83700
H	37.31500	-30.41600	4.38400
H	38.46100	-30.48500	5.37100
O	30.17000	-29.30100	13.54200
H	30.03600	-30.06100	12.97500
H	29.75400	-28.57700	13.07400
O	33.32700	-28.40400	13.75300
H	33.21000	-27.46000	13.65100
H	32.50600	-28.70700	14.14200
O	34.35300	-27.75100	16.96900
H	35.25500	-27.71900	17.28600
H	34.12600	-28.68100	16.98400
O	36.10800	-28.99800	13.63000
H	35.15800	-28.99800	13.51600
H	36.41100	-29.74500	13.11400
O	42.05700	-19.86100	13.45900
H	42.85000	-20.20200	13.04300
H	42.29700	-19.75100	14.37900
O	41.62600	-23.49200	13.40000
H	41.75500	-23.23800	14.31400
H	40.94600	-22.89900	13.08100
O	40.57300	-26.01900	13.44500
H	39.78300	-25.76400	12.96900
H	41.06400	-25.20400	13.55100
O	42.52500	-25.65500	10.40400
H	41.72800	-25.54300	10.92200
H	42.29700	-25.31600	9.53800
O	38.85700	-25.76600	15.87700
H	39.10800	-24.88200	16.14300
H	39.39300	-25.94600	15.10400
O	41.52300	-19.94600	8.79300
H	42.03500	-19.15200	8.94300
H	42.17500	-20.64300	8.72600
O	39.48800	-21.61700	9.56900
H	40.32500	-21.20700	9.35200
H	39.66600	-22.55700	9.53900
O	39.51000	-24.27800	10.24000
H	39.31300	-24.87100	9.51500
H	38.95500	-24.58300	10.95700
O	42.00500	-24.75200	7.92400
H	41.07500	-24.81300	8.14100
H	42.10100	-25.27400	7.12700
O	41.92300	-28.22600	10.51200
H	42.18400	-27.31200	10.40300
H	42.74500	-28.69400	10.66100
O	36.07000	-22.59300	12.40600
H	36.75700	-22.90700	11.81900
H	35.91500	-21.68900	12.13300
O	40.87400	-27.70400	16.83500
H	40.79000	-28.31100	16.10000
H	40.05200	-27.21300	16.83600
O	41.05500	-17.08400	15.95800
H	41.64100	-17.82900	16.09100
H	40.85800	-16.77300	16.84100
O	35.64700	-24.51300	14.67800
H	35.68600	-25.04700	15.47200
H	35.14100	-25.03900	14.05900
O	39.27800	-25.78700	7.75400
H	39.57300	-26.69600	7.70000
H	38.37700	-25.80800	7.43200
O	41.00700	-28.10500	7.87800
H	40.67800	-28.98500	7.69500
H	41.36100	-28.16000	8.76500
O	39.46500	-23.11700	16.89600
H	38.92100	-22.34100	17.02800
H	40.34300	-22.77000	16.73800
O	40.25200	-23.99000	19.78900
H	39.51000	-23.84200	20.37600
H	39.93100	-23.72900	18.92600
O	40.69800	-16.10300	11.82000
H	41.52300	-16.40300	11.44000
H	40.34700	-16.87300	12.26900
O	38.89800	-18.18500	12.77900
H	38.06200	-18.61200	12.59700
H	38.87100	-17.99100	13.71600
O	42.25200	-26.44100	5.87000
H	41.55900	-26.36600	5.21400
H	42.00700	-27.20500	6.39300
O	38.21300	-25.12400	12.56400
H	37.89400	-25.97700	12.27100
H	37.66700	-24.90900	13.32000
O	41.99100	-22.40700	16.28100

H	42.21000	-21.47600	16.30000
H	42.40400	-22.76700	17.06600
O	38.08200	-21.50300	14.53300
H	37.38900	-21.91800	14.02000
H	38.83000	-21.46100	13.93700
O	39.99600	-21.57200	12.41000
H	39.97300	-21.47300	11.45800
H	40.30600	-20.72500	12.73200
O	41.01000	-13.97200	13.54200
H	40.87500	-14.73200	12.97500
H	40.59300	-13.24800	13.07400
O	38.49800	-26.61000	19.79100
H	39.14400	-27.12000	20.28000
H	38.29000	-25.87300	20.36500
O	-14.94500	5.30500	45.75800
H	-15.59700	5.89000	45.37200
H	-14.15100	5.83500	45.81800
O	-13.46800	2.99400	46.29500
H	-14.10300	3.06700	47.00700
H	-13.79800	3.58300	45.61700
O	-11.30200	-2.12600	46.50500
H	-10.39100	-1.89300	46.32900
H	-11.81100	-1.54300	45.94200
O	-16.96600	5.53000	48.04600
H	-17.10200	6.37500	48.47500
H	-16.42500	5.73200	47.28300
O	-13.65200	-0.95600	45.08300
H	-14.16700	-0.18500	45.32200
H	-13.92400	-1.62900	45.70700
O	-8.67900	1.79700	47.01900
H	-9.45600	1.45800	46.57400
H	-8.00000	1.80300	46.34400
O	-17.27400	0.89400	41.56200
H	-18.18100	0.66300	41.76100
H	-16.77600	0.09800	41.75000
O	-14.36700	9.60400	44.48200
H	-15.04100	9.90700	45.09000
H	-13.98000	10.40700	44.13200
O	-10.86300	4.60600	49.85400
H	-11.31700	3.76500	49.91000
H	-11.49300	5.24200	50.19100
O	-12.42000	6.44300	50.78900
H	-12.12700	7.00400	50.07000
H	-11.91000	6.73600	51.54400
O	-16.39700	2.53600	43.42300
H	-16.83500	1.82000	42.96400
H	-15.65600	2.76400	42.86200
O	-8.28000	0.81200	49.48100
H	-8.41800	1.00200	48.55300
H	-8.63600	1.57400	49.93800
O	-16.16800	9.77200	46.69700
H	-15.99200	9.30000	47.51100
H	-16.85400	10.39800	46.92500
O	-12.27600	1.39400	43.01700
H	-12.89300	0.66900	42.92000
H	-12.74400	2.15500	42.67300
O	-17.30000	7.96600	49.38500
H	-18.08300	8.50800	49.47900
H	-16.60000	8.48300	49.78400
O	-11.45200	4.71800	47.13600
H	-11.17600	4.60400	48.04500
H	-12.32200	4.32000	47.09700
O	-14.81700	8.96700	50.77900
H	-14.63500	8.14900	51.24200
H	-13.99400	9.18400	50.34200
O	-14.48300	7.89400	48.04100
H	-13.95400	7.38300	47.42800
H	-14.22000	7.57800	48.90500
O	-11.38900	7.86600	48.65600
H	-11.21500	7.30000	47.90400
H	-10.89600	8.66600	48.47400
O	-5.53500	0.62900	50.27500
H	-5.09200	-0.12000	49.87700
H	-6.41200	0.62000	49.89300
O	-10.40100	7.18200	45.75600
H	-9.70600	7.80300	45.97500
H	-10.05900	6.33200	46.03100
O	-8.50200	4.63200	47.28500
H	-7.97700	4.97100	46.55900
H	-8.59000	3.69700	47.10100
O	-16.31000	7.28800	44.66300
H	-16.88200	7.90700	44.21000
H	-15.73600	7.83800	45.19700
O	-13.09000	7.57200	45.79400
H	-12.15700	7.50700	45.99700
H	-13.17800	8.40800	45.33600
O	-10.46100	6.88900	42.75500
H	-10.54000	6.55500	43.64800
H	-9.64000	7.38100	42.75400
O	-15.92500	-1.37600	42.00500
H	-14.97900	-1.50500	42.07400
H	-16.30300	-2.11400	42.48200
O	-17.74100	-3.45100	41.94000
H	-18.64700	-3.74700	42.01700
H	-17.46300	-3.74800	41.07400
O	-12.89100	4.20500	42.37600
H	-12.18700	4.41000	42.99100
H	-13.61400	4.77300	42.64000
O	-10.98200	4.64400	44.42500
H	-10.14700	4.21200	44.24300
H	-11.14400	4.47200	45.35300
O	-11.32700	1.14700	45.79100
H	-11.38300	0.98100	44.85000
H	-11.95300	1.85500	45.94700
O	-7.84400	13.88200	43.78900
H	-7.59100	13.36000	43.02700
H	-8.78100	13.71900	43.89100
O	-8.12400	15.78500	45.99100
H	-8.24100	15.11000	46.65900
H	-8.01400	15.29800	45.17400
O	-6.14000	10.27500	44.69200
H	-5.97300	11.14300	45.05900
H	-6.33100	10.43800	43.76800
O	-6.16200	12.73100	45.67700
H	-6.28200	13.17700	46.51500
H	-6.85600	13.07900	45.11800
O	-9.52100	11.09700	44.93900
H	-10.35700	11.43500	45.26000

H	-9.66000	10.96200	44.00200
O	-7.54500	5.94100	49.64700
H	-8.19900	5.62600	49.02200
H	-7.58700	5.31900	50.37300
O	-6.43400	16.22300	41.56200
H	-7.34100	15.99200	41.76100
H	-5.93700	15.42700	41.75000
O	-5.55800	17.86500	43.42300
H	-5.99600	17.14900	42.96400
H	-4.81700	18.09300	42.86200
O	-12.19200	13.57100	47.75000
H	-13.12200	13.51000	47.96700
H	-12.09600	13.04900	46.95400
O	-12.27300	10.09700	50.33800
H	-12.01300	11.01100	50.22900
H	-11.45200	9.62900	50.48800
O	-1.43700	16.72400	43.01700
H	-2.05400	15.99800	42.92000
H	-1.90500	17.48500	42.67300
O	-9.64700	10.94200	42.13900
H	-10.52400	10.82100	41.77500
H	-9.17100	10.15100	41.88700
O	-14.91900	12.53600	47.58000
H	-14.62400	11.62700	47.52700
H	-15.82000	12.51500	47.25800
O	-13.19000	10.21800	47.70400
H	-13.51900	9.33800	47.52100
H	-12.83600	10.16300	48.59200
O	-10.07600	10.50500	48.07100
H	-9.73600	11.03400	47.34900
H	-9.62900	10.84300	48.84700
O	-8.08900	9.00300	46.27100
H	-7.36800	9.38100	45.76700
H	-8.71300	9.72100	46.37400
O	-3.94600	9.70700	47.20700
H	-3.73000	10.59000	46.90600
H	-4.42700	9.31600	46.47700
O	-7.94300	8.53700	49.10100
H	-7.82100	7.60900	49.30000
H	-8.23300	8.55100	48.18800
O	-11.94500	11.88200	45.69700
H	-12.63800	11.95700	45.04100
H	-12.19000	11.11800	46.21900
O	-5.08600	13.95400	42.00500
H	-4.14000	13.82400	42.07400
H	-5.46400	13.21500	42.48200
O	-6.90100	11.87900	41.94000
H	-7.80800	11.58200	42.01700
H	-6.62400	11.58200	41.07400
O	-5.28500	6.42200	48.09500
H	-6.09500	6.07700	48.47000
H	-4.59700	6.11300	48.68400
O	-3.35600	5.50000	49.71700
H	-2.80300	5.92000	50.37600
H	-3.11900	4.57400	49.76000
O	-8.13100	8.73700	42.08200
H	-7.26900	9.06300	42.34100
H	-7.94600	7.95000	41.56900
O	-7.17800	5.97700	45.37000
H	-7.19100	6.92100	45.52600
H	-6.28600	5.79400	45.07600
O	-3.36300	2.57100	49.62400
H	-4.13000	2.01700	49.77000
H	-2.95200	2.64300	50.48600
O	-14.94500	-17.68900	32.48300
H	-15.59700	-17.10400	32.09600
H	-14.15100	-17.15800	32.54200
O	-13.46800	-20.00000	33.02000
H	-14.10300	-19.92600	33.73200
H	-13.79800	-19.41100	32.34200
O	-19.24000	-12.82200	36.05000
H	-19.31800	-11.87100	36.12900
H	-19.84400	-13.16700	36.70800
O	-11.30200	-25.12000	33.22900
H	-10.39100	-24.88700	33.05400
H	-11.81100	-24.53600	32.66700
O	-16.96600	-17.46400	34.77100
H	-17.10200	-16.61800	35.19900
H	-16.42500	-17.26200	34.00700
O	-13.65200	-23.95000	31.80800
H	-14.16700	-23.17900	32.04700
H	-13.92400	-24.62300	32.43100
O	-8.67900	-21.19600	33.74300
H	-9.45600	-21.53500	33.29900
H	-8.00000	-21.19100	33.06900
O	-17.27400	-22.10000	28.28700
H	-18.18100	-22.33100	28.48600
H	-16.77600	-22.89600	28.47400
O	-14.36700	-13.39000	31.20600
H	-15.04100	-13.08700	31.81500
H	-13.98000	-12.58700	30.85700
O	-10.86300	-18.38800	36.57800
H	-11.31700	-19.22900	36.63500
H	-11.49300	-17.75200	36.91600
O	-12.42000	-16.55000	37.51300
H	-12.12700	-15.99000	36.79400
H	-11.91000	-16.25700	38.26900
O	-16.39700	-20.45800	30.14800
H	-16.83500	-21.17400	29.68800
H	-15.65600	-20.23000	29.58600
O	-8.28000	-22.18200	36.20600
H	-8.41800	-21.99200	35.27800
H	-8.63600	-21.42000	36.66200
O	-16.16800	-13.22200	33.42100
H	-15.99200	-13.69300	34.23600
H	-16.85400	-12.59600	33.64900
O	-12.27600	-21.59900	29.74100
H	-12.89300	-22.32500	29.64400
H	-12.74400	-20.83800	29.39800
O	-17.30000	-15.02800	36.10900
H	-18.08300	-14.48600	36.20300
H	-16.60000	-14.51100	36.50900
O	-11.45200	-18.27500	33.86000
H	-11.17600	-18.39000	34.77000
H	-12.32200	-18.67400	33.82200
O	-14.81700	-14.02700	37.50400
H	-14.63500	-14.84500	37.96600
H	-13.99400	-13.81000	37.06600

O	-14.48300	-15.10000	34.76500
H	-13.95400	-15.61100	34.15200
H	-14.22000	-15.41600	35.63000
O	-11.38900	-15.12800	35.38100
H	-11.21500	-15.69400	34.62800
H	-10.89600	-14.32800	35.19900
O	-5.53500	-22.36500	37.00000
H	-5.09200	-23.11400	36.60200
H	-6.41200	-22.37400	36.61700
O	-10.40100	-15.81200	32.48000
H	-9.70600	-15.19100	32.69900
H	-10.05900	-16.66200	32.75600
O	-8.50200	-18.36200	34.00900
H	-7.97700	-18.02300	33.28400
H	-8.59000	-19.29700	33.82500
O	-16.31000	-15.70500	31.38800
H	-16.88200	-15.08600	30.93500
H	-15.73600	-15.15500	31.92200
O	-13.09000	-15.42200	32.51900
H	-12.15700	-15.48700	32.72200
H	-13.17800	-14.58600	32.06000
O	-10.46100	-16.10500	29.47900
H	-10.54000	-16.43900	30.37300
H	-9.64000	-15.61300	29.47800
O	-15.92500	-24.36900	28.72900
H	-14.97900	-24.49900	28.79800
H	-16.30300	-25.10800	29.20600
O	-17.74100	-26.44400	28.66500
H	-18.64700	-26.74100	28.74100
H	-17.46300	-26.74100	27.79800
O	-12.89100	-18.78800	29.10000
H	-12.18700	-18.58300	29.71600
H	-13.61400	-18.22100	29.36500
O	-10.98200	-18.35000	31.15000
H	-10.14700	-18.78200	30.96800
H	-11.14400	-18.52200	32.07800
O	-11.32700	-21.84700	32.51600
H	-11.38300	-22.01200	31.57400
H	-11.95300	-21.13900	32.67200
O	-2.35400	-7.83300	41.24500
H	-1.71600	-7.95200	40.54200
H	-2.47100	-6.88500	41.30400
O	-5.54500	-7.88000	41.18200
H	-5.88300	-7.99600	40.29400
H	-4.67600	-8.28100	41.16200
O	-9.68600	-6.02700	37.17100
H	-10.24900	-6.11800	36.40300
H	-9.80100	-6.84700	37.65200
O	-12.13900	-4.53200	40.01000
H	-11.34700	-4.87300	39.59400
H	-11.90000	-4.42200	40.93000
O	-4.10600	-2.36000	32.48300
H	-4.75700	-1.77400	32.09600
H	-3.31200	-1.82900	32.54200
O	-10.18000	-5.27100	32.88100
H	-9.40700	-5.81800	32.74300
H	-9.82800	-4.40600	33.09300
O	-8.34200	-3.94300	46.08800
H	-8.63300	-3.03900	45.97300
H	-7.70600	-3.90200	46.80300
O	-7.41600	-6.56500	42.67800
H	-6.82000	-6.79000	41.96400
H	-7.35900	-7.30700	43.28100
O	-7.84400	-9.11200	30.51300
H	-7.59100	-9.63300	29.75200
H	-8.78100	-9.27500	30.61600
O	-2.79900	-10.10800	38.32100
H	-2.01300	-10.52600	37.97000
H	-2.53600	-9.20300	38.48900
O	-2.62800	-4.67100	33.02000
H	-3.26400	-4.59700	33.73200
H	-2.95900	-4.08100	32.34200
O	2.60100	-10.17000	39.22400
H	3.33400	-10.74100	39.45800
H	1.84500	-10.55800	39.66500
O	-8.12400	-7.20900	32.71600
H	-8.24100	-7.88400	33.38400
H	-8.01400	-7.69500	31.89900
O	-5.78100	-7.74800	36.42000
H	-6.14500	-8.27500	37.13200
H	-4.92400	-8.13800	36.24700
O	-6.14000	-12.71900	31.41700
H	-5.97300	-11.85100	31.78400
H	-6.33100	-12.55600	30.49300
O	-3.38300	-4.25700	43.08300
H	-3.68300	-3.35500	42.97500
H	-3.72800	-4.52400	43.93500
O	-9.93200	-7.80600	39.11700
H	-10.85700	-7.93800	39.32500
H	-9.68300	-7.02600	39.61400
O	0.01700	-11.73900	40.76300
H	-0.89200	-11.80700	40.46900
H	-0.03400	-11.26200	41.59100
O	-6.16200	-10.26300	32.40100
H	-6.28200	-9.81700	33.24000
H	-6.85600	-9.91400	31.84300
O	-12.57100	-8.16200	39.95100
H	-12.44200	-7.90900	40.86500
H	-13.25100	-7.57000	39.63200
O	-8.40100	2.50700	36.05000
H	-8.47800	3.45800	36.12900
H	-9.00500	2.16200	36.70800
O	1.88300	-13.53700	42.54100
H	1.24000	-13.24400	41.89500
H	1.37800	-13.65200	43.34600
O	-0.46300	-9.79100	33.22900
H	0.44900	-9.55800	33.05400
H	-0.97200	-9.20700	32.66700
O	-2.17900	-5.36800	46.86500
H	-1.77100	-5.81000	46.12100
H	-2.90100	-5.94300	47.11900
O	-1.85600	-4.80800	40.62100
H	-2.48500	-4.64400	39.91800
H	-2.24300	-4.39400	41.39200
O	2.02300	-3.87300	37.97500
H	1.33800	-3.49300	37.42500
H	2.10700	-3.26300	38.70800
O	-0.72200	-6.22700	44.57500

H	-0.51000	-5.58400	43.89800
H	-0.76900	-7.06100	44.10700
O	-3.11900	-13.61500	41.47800
H	-3.98100	-13.31700	41.77000
H	-3.09300	-13.39800	40.54600
O	-6.30300	-5.07100	37.88600
H	-5.83900	-4.79200	37.09600
H	-6.56800	-5.97200	37.70300
O	-9.41100	-5.52400	40.82400
H	-8.84100	-5.09500	40.18500
H	-8.82000	-5.80500	41.52200
O	-7.36600	-8.94800	34.69500
H	-6.66400	-8.49600	35.16200
H	-7.69800	-9.59000	35.32300
O	-13.62400	-10.69000	39.99600
H	-14.41400	-10.43400	39.52000
H	-13.13300	-9.87500	40.10100
O	0.39300	-3.35000	42.74700
H	1.21400	-3.75200	42.46500
H	0.38900	-3.45300	43.69800
O	-11.67200	-10.32600	36.95500
H	-12.46900	-10.21400	37.47300
H	-11.90000	-9.98700	36.08900
O	-5.84200	-3.80100	44.90300
H	-5.77700	-3.33900	45.73900
H	-6.55100	-4.43100	45.03100
O	8.48300	-7.00600	39.09500
H	7.73000	-7.57500	38.94000
H	8.61800	-7.03900	40.04200
O	-6.12700	-2.13400	34.77100
H	-6.26200	-1.28900	35.19900
H	-5.58600	-1.93200	34.00700
O	-12.67400	-4.61700	35.34400
H	-12.16200	-3.82300	35.49400
H	-12.02200	-5.31400	35.27700
O	-10.86500	-1.50000	37.42600
H	-10.68800	-0.57800	37.23800
H	-10.06900	-1.81400	37.85500
O	-4.50000	-1.51200	48.90100
H	-4.94400	-2.29800	48.58300
H	-3.83800	-1.32400	48.23500
O	-9.74100	0.01800	43.42700
H	-9.19100	-0.11100	42.65500
H	-10.58000	0.31700	43.07600
O	-2.40200	-1.23200	46.92900
H	-2.42000	-0.41100	46.43700
H	-1.48000	-1.35600	47.15400
O	-14.70900	-6.28800	36.12000
H	-13.87200	-5.87800	35.90300
H	-14.53100	-7.22800	36.09000
O	-2.81300	-8.62000	31.80800
H	-3.32800	-7.85000	32.04700
H	-3.08500	-9.29400	32.43100
O	-5.22800	-13.77500	36.86600
H	-6.08400	-14.16500	36.68700
H	-4.81500	-13.69600	36.00600
O	-3.69800	-16.24100	41.26600
H	-4.20200	-16.27800	40.45200
H	-3.40500	-15.33200	41.32500
O	2.16100	-5.86700	33.74300
H	1.38400	-6.20600	33.29900
H	2.84000	-5.86200	33.06900
O	-3.02400	-10.74800	33.63400
H	-3.32900	-10.43300	34.48500
H	-2.07100	-10.67000	33.67700
O	-4.57900	-4.58900	35.28300
H	-5.10700	-5.32300	34.97100
H	-5.08100	-3.81100	35.03700
O	0.40000	-6.39400	40.40000
H	-0.25800	-5.70400	40.48800
H	1.20400	-6.00400	40.74300
O	-9.84800	-13.92700	37.64400
H	-9.27300	-14.14900	36.91100
H	-9.26000	-13.85800	38.39700
O	-0.79000	-8.03900	38.45000
H	-0.27600	-7.40600	38.95200
H	-0.14000	-8.63900	38.08500
O	-9.52100	-11.89600	31.66300
H	-10.35700	-11.55900	31.98400
H	-9.66000	-12.03100	30.72600
O	-7.54500	-17.05300	36.37100
H	-8.19900	-17.36800	35.74700
H	-7.58700	-17.67500	37.09700
O	-6.43400	-6.77100	28.28700
H	-7.34100	-7.00200	28.48600
H	-5.93700	-7.56700	28.47400
O	-3.52700	1.93900	31.20600
H	-4.20100	2.24200	31.81500
H	-3.14100	2.74200	30.85700
O	-4.31200	-10.56400	40.69600
H	-5.22900	-10.34300	40.53800
H	-3.89600	-10.49000	39.83700
O	-3.52800	-9.16500	35.82600
H	-2.78100	-8.65300	35.51700
H	-3.25900	-9.48700	36.68700
O	-5.25500	-6.66900	32.80900
H	-6.16400	-6.95400	32.90600
H	-5.22600	-6.25000	31.94900
O	-0.02300	-3.05900	36.57800
H	-0.47700	-3.90000	36.63500
H	-0.65400	-2.42300	36.91600
O	-1.08000	-12.13000	37.80800
H	-1.81800	-12.64200	38.13900
H	-0.82700	-12.57000	36.99700
O	-1.58000	-1.22100	37.51300
H	-1.28800	-0.66100	36.79400
H	-1.07000	-0.92800	38.26900
O	-0.97900	-19.35200	38.40200
H	-0.67300	-20.21200	38.11500
H	-0.25400	-18.76000	38.20200
O	-14.68700	-8.94900	36.79100
H	-14.88400	-9.54200	36.06600
H	-15.24200	-9.25400	37.50800
O	-6.70700	-9.05700	38.91000
H	-6.65200	-10.00900	38.83400
H	-7.51800	-8.90100	39.39400
O	-5.55800	-5.12900	30.14800
H	-5.99600	-5.84500	29.68800

H	-4.81700	-4.90100	29.58600
O	-8.76800	-10.43900	36.66900
H	-9.58100	-10.13900	37.07600
H	-8.27200	-10.83300	37.38700
O	-6.61700	-0.44300	38.60300
H	-6.65900	-0.21700	37.67300
H	-5.70100	-0.67700	38.75100
O	2.56000	-6.85300	36.20600
H	2.42100	-6.66300	35.27800
H	2.20300	-6.09100	36.66200
O	2.58600	-4.78500	41.21700
H	3.02000	-4.87800	42.06500
H	3.30000	-4.80900	40.57900
O	-8.50000	-3.56700	38.39900
H	-7.70600	-4.06200	38.19700
H	-9.21100	-4.09500	38.03600
O	-3.68100	-1.18500	42.75000
H	-3.62700	-0.61600	43.51800
H	-4.54900	-1.01500	42.38500
O	-12.19200	-9.42300	34.47500
H	-13.12200	-9.48400	34.69200
H	-12.09600	-9.94500	33.67800
O	-12.27300	-12.89700	37.06300
H	-12.01300	-11.98200	36.95300
H	-11.45200	-13.36500	37.21200
O	3.44800	-4.74300	43.75000
H	3.64200	-3.83800	43.99600
H	3.34800	-5.20200	44.58400
O	-1.49000	-4.59200	49.32400
H	-1.64200	-4.82100	48.40700
H	-1.50000	-3.63500	49.33700
O	-4.72200	-7.01900	44.30900
H	-3.79300	-6.86800	44.13600
H	-5.14300	-6.93100	43.45400
O	-5.92300	-2.99400	42.07900
H	-6.06200	-3.36200	42.95100
H	-6.71200	-2.47900	41.90700
O	-5.32800	2.10700	33.42100
H	-5.15300	1.63600	34.23600
H	-6.01500	2.73400	33.64900
O	-4.37300	-3.61600	39.46700
H	-4.70800	-3.65600	40.36300
H	-4.87200	-4.28100	38.99400
O	-1.43700	-6.27000	29.74100
H	-2.05400	-6.99500	29.64400
H	-1.90500	-5.50900	29.39800
O	0.21900	-1.56000	47.40200
H	0.16000	-2.24400	46.73500
H	0.62100	-0.81800	46.95000
O	-10.76200	5.25600	40.19500
H	-10.52200	5.88800	40.87300
H	-11.60500	4.90500	40.48200
O	-9.64700	-12.05200	28.86300
H	-10.52400	-12.17300	28.49900
H	-9.17100	-12.84300	28.61100
O	0.22100	-3.59600	45.63300
H	-0.66700	-3.93400	45.75600
H	0.79000	-4.32800	45.87000
O	-18.12700	-7.26400	38.95700
H	-17.43900	-7.57800	38.36900
H	-18.28200	-6.36000	38.68400
O	-6.24900	1.06400	42.59900
H	-6.32200	1.86900	43.11100
H	-6.69200	1.26300	41.77400
O	-6.46000	0.30100	36.10900
H	-7.24400	0.84300	36.20300
H	-5.76100	0.81800	36.50900
O	-3.15100	-13.89000	38.75300
H	-2.65400	-14.64300	38.43300
H	-3.92200	-13.85300	38.18700
O	4.70200	-8.18500	40.39500
H	4.69100	-7.30300	40.02400
H	3.83400	-8.53800	40.20000
O	-3.79000	0.96300	44.75500
H	-3.07700	1.59700	44.68300
H	-4.28100	1.05900	43.93900
O	-2.06800	-5.13200	36.32700
H	-3.00200	-5.01200	36.15700
H	-1.89000	-6.03500	36.06300
O	-6.56700	-11.74800	38.48000
H	-6.93700	-12.30100	39.16800
H	-6.07100	-12.35200	37.92800
O	1.32000	-9.29100	36.86200
H	1.78600	-8.45800	36.78800
H	1.77000	-9.75600	37.56600
O	-8.73800	-1.28800	45.57600
H	-8.03700	-0.65400	45.72900
H	-9.20900	-0.94700	44.81500
O	-0.61300	-2.94600	33.86000
H	-0.33700	-3.06100	34.77000
H	-1.48200	-3.34400	33.82200
O	-0.70900	-16.92300	40.90100
H	-1.62300	-16.69200	41.06900
H	-0.28000	-16.83900	41.75200
O	-0.93800	-8.73400	43.40100
H	-1.59100	-8.48600	42.74600
H	-0.56000	-9.54700	43.06700
O	-3.97700	1.30200	37.50400
H	-3.79500	0.48500	37.96600
H	-3.15400	1.51900	37.06600
O	-3.64300	0.22900	34.76500
H	-3.11400	-0.28200	34.15200
H	-3.38000	-0.08700	35.63000
O	-13.14200	-1.75400	42.50900
H	-12.55600	-2.50000	42.64100
H	-13.33900	-1.44400	43.39200
O	-10.28800	1.28100	37.52500
H	-10.95700	1.76500	37.04100
H	-10.42600	1.52800	38.43900
O	-6.14700	3.31500	44.12300
H	-5.59500	3.47800	44.88800
H	-5.80700	3.90600	43.45100
O	-1.29600	-1.85900	49.97800
H	-0.75200	-1.24600	49.48400
H	-1.87700	-1.30100	50.49500
O	-14.91900	-10.45700	34.30500
H	-14.62400	-11.36600	34.25100
H	-15.82000	-10.47900	33.98300

O	-3.01000	1.26300	41.11000
H	-3.13500	0.46200	40.60100
H	-2.35200	1.75800	40.62100
O	-11.38900	-12.38200	40.28700
H	-12.11100	-11.76800	40.14800
H	-10.60000	-11.84600	40.20900
O	-0.94700	-0.93600	42.73800
H	-1.89400	-1.05500	42.66500
H	-0.59900	-1.82400	42.82000
O	-13.19000	-12.77600	34.42800
H	-13.51900	-13.65600	34.24600
H	-12.83600	-12.83100	35.31600
O	-3.94200	-1.14100	38.93800
H	-4.09000	-2.06900	39.11900
H	-3.11700	-1.11900	38.45400
O	-10.07600	-12.48900	34.79600
H	-9.73600	-11.96000	34.07400
H	-9.62900	-12.15100	35.57200
O	-0.33900	-0.31800	40.02400
H	0.58600	-0.52300	39.88700
H	-0.50700	-0.56600	40.93300
O	-8.08900	-13.99100	32.99500
H	-7.36800	-13.61300	32.49200
H	-8.71300	-13.27200	33.09900
O	-3.94600	-13.28700	33.93100
H	-3.73000	-12.40400	33.63000
H	-4.42700	-13.67800	33.20200
O	-9.20600	-10.32800	40.50800
H	-9.35200	-10.01400	41.40100
H	-9.68400	-9.71000	39.95500
O	-7.34600	-13.33800	40.55600
H	-7.36000	-14.23400	40.22000
H	-6.62900	-13.33100	41.19000
O	-0.54900	0.20100	35.38100
H	-0.37600	-0.36400	34.62800
H	-0.05600	1.00100	35.19900
O	-7.94300	-14.45600	35.82500
H	-7.82100	-15.38500	36.02400
H	-8.23300	-14.44300	34.91300
O	-5.07400	-16.77100	38.96200
H	-5.05100	-16.70700	38.00700
H	-4.65700	-17.61100	39.15600
O	5.30500	-7.03500	37.00000
H	5.74800	-7.78500	36.60200
H	4.42700	-7.04400	36.61700
O	0.43800	-0.48200	32.48000
H	1.13300	0.13800	32.69900
H	0.78100	-1.33300	32.75600
O	2.33700	-3.03200	34.00900
H	2.86300	-2.69400	33.28400
H	2.25000	-3.96800	33.82500
O	-6.00100	0.88100	46.13800
H	-5.69200	0.63600	47.01000
H	-5.20300	1.08600	45.65000
O	-5.97900	3.03500	38.36900
H	-6.41800	2.54200	39.06200
H	-5.22800	2.49200	38.12800
O	-13.49900	-0.77400	38.37100
H	-12.67300	-1.07400	37.99100
H	-13.85000	-1.54300	38.82000
O	-8.53100	-1.42100	40.44100
H	-9.15300	-2.08400	40.14100
H	-7.86100	-1.39500	39.75800
O	-15.29800	-2.85500	39.33000
H	-16.13500	-3.28300	39.14800
H	-15.32600	-2.66200	40.26700
O	2.02700	-2.39900	49.31400
H	1.23600	-2.41700	48.77500
H	1.79000	-1.86000	50.06900
O	2.14700	-5.55000	46.62700
H	2.19600	-5.71500	47.56900
H	2.03800	-6.41700	46.23700
O	-8.97000	-2.87100	33.91700
H	-8.82100	-2.18800	33.26300
H	-8.16300	-2.89100	34.43200
O	3.64800	-3.13000	46.87200
H	3.35700	-2.76700	47.70800
H	3.03600	-3.84400	46.69300
O	4.84800	-5.56500	39.39200
H	5.68300	-5.12600	39.55600
H	4.98400	-6.04100	38.57300
O	1.89700	-8.00700	45.34000
H	2.37300	-7.81400	44.53200
H	0.97400	-7.95100	45.09400
O	-11.21500	-6.84500	34.92300
H	-11.46000	-7.74800	35.12100
H	-11.04500	-6.84300	33.98100
O	-11.94500	-11.11200	32.42100
H	-12.63800	-11.03700	31.76500
H	-12.19000	-11.87600	32.94400
O	-5.47000	-0.37600	31.38800
H	-6.04300	0.24300	30.93500
H	-4.89700	0.17400	31.92200
O	0.59700	-11.13000	43.57300
H	1.54900	-11.03100	43.59400
H	0.42800	-11.92300	44.08200
O	-2.25100	-0.09300	32.51900
H	-1.31700	-0.15800	32.72200
H	-2.33800	0.74300	32.06000
O	0.37800	-0.77600	29.47900
H	0.30000	-1.11000	30.37300
H	1.19900	-0.28400	29.47800
O	3.07600	-7.91000	42.68700
H	3.04400	-8.84800	42.87300
H	3.68600	-7.83100	41.95300
O	-5.08600	-9.04000	28.72900
H	-4.14000	-9.17000	28.79800
H	-5.46400	-9.77900	29.20600
O	3.22600	-11.11900	42.73300
H	2.70900	-11.90300	42.54600
H	4.11000	-11.44700	42.89700
O	0.88700	-17.37500	38.69200
H	0.68900	-17.68300	39.57600
H	1.49700	-16.64800	38.82200
O	8.89400	-6.84800	41.82700
H	9.62400	-7.11000	42.38800
H	8.28400	-7.58500	41.86800
O	6.97500	-8.70700	42.42500

H	6.24900	-8.84200	41.81600
H	6.56000	-8.40000	43.23000
O	-6.90100	-11.11500	28.66500
H	-7.80800	-11.41200	28.74100
H	-6.62400	-11.41200	27.79800
O	-15.98300	-9.79500	39.11500
H	-16.30300	-10.64800	38.82200
H	-16.53000	-9.58000	39.87100
O	-5.28500	-16.57100	34.81900
H	-6.09500	-16.91700	35.19500
H	-4.59700	-16.88100	35.40800
O	-3.35600	-17.49400	36.44200
H	-2.80300	-17.07400	37.10100
H	-3.11900	-18.42000	36.48400
O	-8.13100	-14.25600	28.80600
H	-7.26900	-13.93100	29.06600
H	-7.94600	-15.04300	28.29300
O	-16.11500	-6.17400	41.08400
H	-16.80800	-6.58900	40.57100
H	-15.36700	-6.13200	40.48800
O	0.02200	-15.38400	35.95000
H	-0.55800	-14.72500	35.56700
H	-0.24500	-16.20600	35.53800
O	-1.52800	-15.98300	38.15200
H	-1.02800	-15.87100	37.34300
H	-0.88400	-16.28700	38.79200
O	-14.20100	-6.24200	38.96100
H	-14.22400	-6.14400	38.00900
H	-13.89100	-5.39600	39.28200
O	-11.54200	-4.18500	43.07500
H	-10.58800	-4.21600	43.00800
H	-11.74100	-4.64600	43.89000
O	-1.19600	-13.22400	35.00500
H	-2.08300	-12.93900	34.78700
H	-0.78000	-13.38400	34.15800
O	2.15600	-13.19900	38.72100
H	2.93200	-12.70900	38.99300
H	1.43100	-12.77300	39.17800
O	4.59300	-12.48600	39.77800
H	5.18800	-12.72700	39.06700
H	4.66600	-13.20500	40.40500
O	-13.18700	1.35700	40.09200
H	-13.32200	0.59700	39.52600
H	-13.60400	2.08100	39.62500
O	-2.05100	-3.45900	29.10000
H	-1.34800	-3.25400	29.71600
H	-2.77500	-2.89100	29.36500
O	-1.22200	-7.70800	35.22000
H	-1.27800	-7.25100	34.38100
H	-0.66900	-8.46900	35.04300
O	2.58900	-11.91900	36.04500
H	2.33300	-12.32000	36.87600
H	1.96400	-11.20500	35.92000
O	6.90000	-9.18000	38.55200
H	6.34000	-9.11000	39.32600
H	6.29500	-9.35500	37.83200
O	-10.03000	2.25400	40.30400
H	-10.14700	3.19900	40.20200
H	-10.85100	1.95100	40.69300
O	-0.14300	-3.02100	31.15000
H	0.69200	-3.45300	30.96800
H	-0.30400	-3.19300	32.07800
O	-0.48800	-6.51800	32.51600
H	-0.54300	-6.68300	31.57400
H	-1.11300	-5.81000	32.67200
O	-7.17800	-17.01700	32.09400
H	-7.19100	-16.07300	32.25100
H	-6.28600	-17.20000	31.80000
O	-9.00500	2.90700	43.52000
H	-8.10200	2.93900	43.83700
H	-9.23100	1.97700	43.53500
O	-7.24900	1.66000	40.18100
H	-8.20000	1.66000	40.06700
H	-6.94600	0.91300	39.66400
O	-3.36300	-20.42300	36.34900
H	-4.13000	-20.97700	36.49400
H	-2.95200	-20.35000	37.21000
O	8.48600	7.49600	41.24500
H	9.12400	7.37700	40.54200
H	8.36800	8.44400	41.30400
O	5.29500	7.44900	41.18200
H	4.95700	7.33300	40.29400
H	6.16400	7.04800	41.16200
O	1.15300	9.30200	37.17100
H	0.59000	9.21100	36.40300
H	1.03900	8.48200	37.65200
O	-1.30000	10.79700	40.01000
H	-0.50800	10.45700	39.59400
H	-1.06000	10.90700	40.93000
O	6.73300	12.96900	32.48300
H	6.08200	13.55500	32.09600
H	7.52800	13.50000	32.54200
O	0.65900	10.05800	32.88100
H	1.43200	9.51100	32.74300
H	1.01200	10.92300	33.09300
O	3.42300	8.76400	42.67800
H	4.01900	8.53900	41.96400
H	3.48100	8.02200	43.28100
O	2.99600	6.21800	30.51300
H	3.24800	5.69600	29.75200
H	2.05800	6.05400	30.61600
O	8.04000	5.22100	38.32100
H	8.82600	4.80300	37.97000
H	8.30400	6.12600	38.48900
O	8.21100	10.65900	33.02000
H	7.57600	10.73200	33.73200
H	7.88100	11.24800	32.34200
O	13.44100	5.15900	39.22400
H	14.17300	4.58900	39.45800
H	12.68500	4.77100	39.66500
O	2.71500	8.12000	32.71600
H	2.59900	7.44500	33.38400
H	2.82500	7.63400	31.89900
O	5.05800	7.58100	36.42000
H	4.69400	7.05500	37.13200
H	5.91500	7.19100	36.24700
O	10.96300	-1.12900	43.68400
H	10.63200	-0.26000	43.46000

H	10.36800	-1.44500	44.36300
O	4.69900	2.61000	31.41700
H	4.86600	3.47800	31.78400
H	4.50900	2.77300	30.49300
O	0.90800	7.52400	39.11700
H	-0.01700	7.39100	39.32500
H	1.15600	8.30300	39.61400
O	10.85600	3.59000	40.76300
H	9.94800	3.52200	40.46900
H	10.80500	4.06800	41.59100
O	4.67800	5.06600	32.40100
H	4.55800	5.51200	33.24000
H	3.98300	5.41500	31.84300
O	-1.73100	7.16700	39.95100
H	-1.60200	7.42000	40.86500
H	-2.41200	7.76000	39.63200
O	2.43900	17.83700	36.05000
H	2.36100	18.78700	36.12900
H	1.83500	17.49100	36.70800
O	12.72200	1.79200	42.54100
H	12.08000	2.08500	41.89500
H	12.21800	1.67700	43.34600
O	10.37600	5.53800	33.22900
H	11.28800	5.77100	33.05400
H	9.86700	6.12200	32.66700
O	5.01400	4.89800	44.23900
H	5.86000	5.32200	44.38200
H	4.95300	4.24100	44.93300
O	8.98300	10.52100	40.62100
H	8.35400	10.68600	39.91800
H	8.59600	10.93500	41.39200
O	12.86200	11.45600	37.97500
H	12.17700	11.83600	37.42500
H	12.94700	12.06600	38.70800
O	8.16300	2.55900	45.89700
H	7.92000	3.31300	45.36000
H	8.43900	2.93700	46.73200
O	7.72000	1.71400	41.47800
H	6.85800	2.01200	41.77000
H	7.74600	1.93100	40.54600
O	4.53600	10.25800	37.88600
H	5.00000	10.53700	37.09600
H	4.27200	9.35700	37.70300
O	1.42900	9.80600	40.82400
H	1.99900	10.23400	40.18500
H	2.01900	9.52400	41.52200
O	3.47300	6.38100	34.69500
H	4.17500	6.83300	35.16200
H	3.14100	5.73900	35.32300
O	-2.78400	4.63900	39.99600
H	-3.57500	4.89500	39.52000
H	-2.29300	5.45400	40.10100
O	-0.83300	5.00400	36.95500
H	-1.63000	5.11500	37.47300
H	-1.06000	5.34200	36.08900
O	-4.50100	4.89200	42.42800
H	-4.25000	5.77700	42.69400
H	-3.96500	4.71300	41.65500
O	4.71200	13.19500	34.77100
H	4.57700	14.04000	35.19900
H	5.25300	13.39700	34.00700
O	1.19900	-0.72600	51.43200
H	0.80600	-1.27900	52.10700
H	2.01600	-0.41500	51.82300
O	-1.83500	10.71200	35.34400
H	-1.32200	11.50700	35.49400
H	-1.18200	10.01500	35.27700
O	-0.02500	13.82900	37.42600
H	0.15100	14.75100	37.23800
H	0.77000	13.51500	37.85500
O	4.11600	0.69000	49.02400
H	4.85700	1.29000	48.94500
H	4.34800	-0.05200	48.46600
O	-0.74000	5.87500	47.30200
H	-0.85500	4.97400	47.60300
H	-1.59600	6.12200	46.95100
O	8.06200	-3.28200	43.30100
H	7.71800	-2.39200	43.22500
H	8.43000	-3.47200	42.43800
O	-3.87000	9.04100	36.12000
H	-3.03200	9.45100	35.90300
H	-3.69200	8.10100	36.09000
O	8.02700	6.70900	31.80800
H	7.51100	7.47900	32.04700
H	7.75400	6.03500	32.43100
O	5.61100	1.55400	36.86600
H	4.75600	1.16400	36.68700
H	6.02400	1.63300	36.00600
O	7.14100	-0.91200	41.26600
H	6.63800	-0.94900	40.45200
H	7.43400	-0.00200	41.32500
O	3.38600	-0.82800	40.27200
H	4.25100	-1.02400	39.91300
H	3.45300	-1.05700	41.19900
O	13.00000	9.46200	33.74300
H	12.22300	9.12300	33.29900
H	13.67900	9.46700	33.06900
O	7.81500	4.58100	33.63400
H	7.51000	4.89600	34.48500
H	8.76800	4.66000	33.67700
O	6.26000	10.74000	35.28300
H	5.73200	10.00600	34.97100
H	5.75900	11.51800	35.03700
O	11.23900	8.93500	40.40000
H	10.58200	9.62500	40.48800
H	12.04300	9.32500	40.74300
O	0.99200	1.40200	37.64400
H	1.56600	1.18000	36.91100
H	1.57900	1.47100	38.39700
O	10.05000	7.29000	38.45000
H	10.56300	7.92300	38.95200
H	10.70000	6.69000	38.08500
O	1.31900	3.43300	31.66300
H	0.48200	3.77000	31.98400
H	1.17900	3.29800	30.72600
O	3.29400	-1.72300	36.37100
H	2.64100	-2.03900	35.74700
H	3.25200	-2.34600	37.09700

O	4.40500	8.55800	28.28700
H	3.49800	8.32700	28.48600
H	4.90200	7.76200	28.47400
O	7.31200	17.26800	31.20600
H	6.63800	17.57100	31.81500
H	7.69900	18.07100	30.85700
O	6.52800	4.76500	40.69600
H	5.61000	4.98600	40.53800
H	6.94400	4.83900	39.83700
O	7.31100	6.16400	35.82600
H	8.05900	6.67600	35.51700
H	7.58000	5.84200	36.68700
O	5.58400	8.66000	32.80900
H	4.67500	8.37600	32.90600
H	5.61400	9.08000	31.94900
O	10.81600	12.27000	36.57800
H	10.36200	11.42900	36.63500
H	10.18500	12.90700	36.91600
O	9.75900	3.20000	37.80800
H	9.02100	2.68700	38.13900
H	10.01200	2.76000	36.99700
O	9.25900	14.10800	37.51300
H	9.55200	14.66900	36.79400
H	9.76900	14.40100	38.26900
O	9.86000	-4.02300	38.40200
H	10.16600	-4.88300	38.11500
H	10.58600	-3.43100	38.20200
O	-3.84700	6.38000	36.79100
H	-4.04500	5.78700	36.06600
H	-4.40300	6.07600	37.50800
O	4.13200	6.27300	38.91000
H	4.18700	5.32000	38.83400
H	3.32200	6.42900	39.39400
O	5.28200	10.20100	30.14800
H	4.84400	9.48400	29.68800
H	6.02300	10.42800	29.58600
O	2.07200	4.89000	36.66900
H	1.25900	5.19000	37.07600
H	2.56800	4.49600	37.38700
O	4.22300	14.88600	38.60300
H	4.18000	15.11200	37.67300
H	5.13900	14.65200	38.75100
O	13.39900	8.47600	36.20600
H	13.26100	8.66600	35.27800
H	13.04300	9.23800	36.66200
O	1.56300	2.46200	42.28500
H	2.20300	2.32100	41.58800
H	0.73100	2.17600	41.90900
O	2.33900	11.76200	38.39900
H	3.13300	11.26700	38.19700
H	1.62800	11.23400	38.03600
O	-1.35300	5.90700	34.47500
H	-2.28300	5.84500	34.69200
H	-1.25700	5.38400	33.67800
O	-1.43400	2.43200	37.06300
H	-1.17300	3.34700	36.95300
H	-0.61200	1.96500	37.21200
O	0.03800	3.84600	44.23200
H	0.71100	3.63800	43.58400
H	-0.78900	3.64800	43.79300
O	5.94100	0.47200	46.24900
H	5.29400	1.17100	46.15500
H	6.71100	0.90600	46.61700
O	6.11700	8.31000	44.30900
H	7.04700	8.46100	44.13600
H	5.69700	8.39900	43.45400
O	4.91600	12.33500	42.07900
H	4.77700	11.96700	42.95100
H	4.12800	12.85000	41.90700
O	5.51100	17.43600	33.42100
H	5.68600	16.96500	34.23600
H	4.82400	18.06300	33.64900
O	6.46700	11.71300	39.46700
H	6.13200	11.67400	40.36300
H	5.96700	11.04800	38.99400
O	0.44100	7.12000	44.99300
H	0.30300	6.38800	45.59400
H	1.35200	7.03100	44.71100
O	9.40300	9.05900	29.74100
H	8.78600	8.33400	29.64400
H	8.93400	9.82000	29.39800
O	3.06900	6.83200	44.48100
H	3.77900	6.19200	44.44000
H	3.02600	7.08300	45.40400
O	1.78200	3.81900	46.30100
H	1.24600	3.36800	46.95300
H	1.19400	3.95300	45.55800
O	1.19300	3.27800	28.86300
H	0.31600	3.15600	28.49900
H	1.66800	2.48600	28.61100
O	-7.28700	8.06500	38.95700
H	-6.60000	7.75100	38.36900
H	-7.44200	8.96900	38.68400
O	-2.48400	2.95400	43.38600
H	-2.56800	2.34700	42.65100
H	-3.30500	3.44500	43.38700
O	4.37900	15.63000	36.10900
H	3.59600	16.17200	36.20300
H	5.07800	16.14800	36.50900
O	7.68900	1.44000	38.75300
H	8.18600	0.68700	38.43300
H	6.91700	1.47600	38.18700
O	15.54200	7.14400	40.39500
H	15.53100	8.02600	40.02400
H	14.67300	6.79100	40.20000
O	8.77200	10.19700	36.32700
H	7.83700	10.31700	36.15700
H	8.94900	9.29400	36.06300
O	4.27200	3.58100	38.48000
H	3.90200	3.02900	39.16800
H	4.76800	2.97700	37.92800
O	12.15900	6.03900	36.86200
H	12.62500	6.87100	36.78800
H	12.61000	5.57300	37.56600
O	10.22700	12.38300	33.86000
H	10.50200	12.26900	34.77000
H	9.35700	11.98500	33.82200
O	10.13100	-1.59300	40.90100

H	9.21700	-1.36300	41.06900
H	10.56000	-1.51000	41.75200
O	9.90100	6.59600	43.40100
H	9.24800	6.84300	42.74600
H	10.27900	5.78200	43.06700
O	6.86200	16.63200	37.50400
H	7.04400	15.81400	37.96600
H	7.68500	16.84800	37.06600
O	7.19600	15.55800	34.76500
H	7.72500	15.04800	34.15200
H	7.45900	15.24300	35.63000
O	-2.30200	13.57500	42.50900
H	-1.71700	12.82900	42.64100
H	-2.50000	13.88500	43.39200
O	0.55200	16.61000	37.52500
H	-0.11700	17.09500	37.04100
H	0.41300	16.85700	38.43900
O	-7.71000	6.14500	41.22900
H	-7.67200	5.61200	42.02300
H	-8.21600	5.61900	40.61000
O	-0.33100	0.99200	44.72500
H	-0.26700	1.85300	44.31300
H	-0.39500	0.37900	43.99300
O	-0.85600	3.06400	48.37300
H	-0.22900	2.68200	48.98600
H	-1.71300	2.84600	48.73800
O	-4.08000	4.87200	34.30500
H	-3.78400	3.96300	34.25100
H	-4.98100	4.85100	33.98300
O	-0.55000	2.94700	40.28700
H	-1.27100	3.56100	40.14800
H	0.23900	3.48300	40.20900
O	2.25300	0.28300	46.56100
H	2.77600	0.52900	47.32400
H	2.03400	1.11500	46.14200
O	-2.35100	2.55300	34.42800
H	-2.67900	1.67300	34.24600
H	-1.99700	2.49800	35.31600
O	6.89700	14.18900	38.93800
H	6.75000	13.26000	39.11900
H	7.72300	14.21100	38.45400
O	0.76300	2.84000	34.79600
H	1.10300	3.36900	34.07400
H	1.21100	3.17900	35.57200
O	3.45800	1.22400	44.24600
H	3.11700	0.42500	44.64800
H	2.75200	1.52400	43.67400
O	4.10900	-1.69100	42.69900
H	4.93200	-2.16500	42.57700
H	4.35300	-0.91200	43.19900
O	2.75000	1.33800	32.99500
H	3.47100	1.71600	32.49200
H	2.12700	2.05700	33.09900
O	6.89300	2.04200	33.93100
H	7.11000	2.92500	33.63000
H	6.41300	1.65100	33.20200
O	1.63300	5.00100	40.50800
H	1.48800	5.31500	41.40100
H	1.15600	5.62000	39.95500
O	3.49300	1.99100	40.55600
H	3.47900	1.09500	40.22000
H	4.21000	1.99800	41.19000
O	5.34600	2.42500	42.60200
H	5.08700	3.34700	42.60000
H	4.81400	2.02800	43.29200
O	10.29000	15.53000	35.38100
H	10.46400	14.96500	34.62800
H	10.78300	16.33000	35.19900
O	-3.89200	7.54200	43.44700
H	-4.43700	8.31800	43.57900
H	-3.01400	7.88900	43.28900
O	2.89700	0.87300	35.82500
H	3.01800	-0.05600	36.02400
H	2.60700	0.88700	34.91300
O	5.76600	-1.44200	38.96200
H	5.78800	-1.37800	38.00700
H	6.18300	-2.28100	39.15600
O	16.14400	8.29400	37.00000
H	16.58700	7.54400	36.60200
H	15.26700	8.28500	36.61700
O	11.27800	14.84700	32.48000
H	11.97300	15.46700	32.69900
H	11.62000	13.99600	32.75600
O	-3.10600	6.66900	46.34000
H	-3.84700	6.81700	46.92600
H	-3.42600	6.92900	45.47700
O	13.17700	12.29700	34.00900
H	13.70200	12.63500	33.28400
H	13.08900	11.36100	33.82500
O	4.86100	18.36400	38.36900
H	4.42100	17.87200	39.06200
H	5.61200	17.82100	38.12800
O	-2.66000	14.55500	38.37100
H	-1.83400	14.25500	37.99100
H	-3.01000	13.78600	38.82000
O	2.30800	13.90800	40.44100
H	1.68600	13.24500	40.14100
H	2.97900	13.93400	39.75800
O	-4.45900	12.47400	39.33000
H	-5.29600	12.04600	39.14800
H	-4.48700	12.66700	40.26700
O	2.38100	2.38600	50.03400
H	2.43000	3.26200	49.65200
H	2.92300	1.84400	49.46100
O	1.87000	12.45800	33.91700
H	2.01800	13.14200	33.26300
H	2.67600	12.43900	34.43200
O	4.61100	3.13900	46.78900
H	3.74100	3.43200	46.51700
H	4.84700	3.72300	47.50900
O	15.68700	9.76400	39.39200
H	16.52200	10.20300	39.55600
H	15.82300	9.28800	38.57300
O	-0.37500	8.48500	34.92300
H	-0.62100	7.58100	35.12100
H	-0.20600	8.48600	33.98100
O	7.31500	-0.64300	44.30500
H	7.83300	0.09300	43.98000

H	6.96000	-0.33700	45.14000
O	-1.10600	4.21700	32.42100
H	-1.79900	4.29200	31.76500
H	-1.35000	3.45400	32.94400
O	7.56600	-4.22300	39.82500
H	7.84600	-4.89100	40.45100
H	8.35100	-4.03000	39.31300
O	5.36900	14.95300	31.38800
H	4.79700	15.57200	30.93500
H	5.94200	15.50300	31.92200
O	11.43700	4.19900	43.57300
H	12.38800	4.29900	43.59400
H	11.26700	3.40700	44.08200
O	9.45800	1.28400	43.75800
H	8.86300	1.56500	43.06300
H	9.17300	1.77400	44.53000
O	8.58900	15.23700	32.51900
H	9.52200	15.17100	32.72200
H	8.50100	16.07200	32.06000
O	11.21800	14.55300	29.47900
H	11.13900	14.21900	30.37300
H	12.03900	15.04500	29.47800
O	13.91500	7.41900	42.68700
H	13.88300	6.48100	42.87300
H	14.52600	7.49800	41.95300
O	5.75400	6.28900	28.72900
H	6.70000	6.16000	28.79800
H	5.37500	5.55000	29.20600
O	14.06500	4.21100	42.73300
H	13.54900	3.42600	42.54600
H	14.94900	3.88200	42.89700
O	11.72600	-2.04500	38.69200
H	11.52800	-2.35400	39.57600
H	12.33600	-1.31900	38.82200
O	3.93800	4.21400	28.66500
H	3.03100	3.91700	28.74100
H	4.21600	3.91700	27.79800
O	-5.14400	5.53500	39.11500
H	-5.46400	4.68100	38.82200
H	-5.69100	5.75000	39.87100
O	-1.36600	8.25100	42.83200
H	-1.14700	9.18300	42.85100
H	-0.95400	7.89200	43.61700
O	5.55400	-1.24200	34.81900
H	4.74400	-1.58800	35.19500
H	6.24300	-1.55200	35.40800
O	7.48300	-2.16400	36.44200
H	8.03600	-1.74500	37.10100
H	7.72000	-3.09100	36.48400
O	2.70800	1.07300	28.80600
H	3.57000	1.39800	29.06600
H	2.89300	0.28600	28.29300
O	-5.27500	9.15500	41.08400
H	-5.96900	8.74000	40.57100
H	-4.52800	9.19700	40.48800
O	10.86100	-0.05400	35.95000
H	10.28100	0.60400	35.56700
H	10.59500	-0.87700	35.53800
O	9.31200	-0.65400	38.15200
H	9.81200	-0.54200	37.34300
H	9.95500	-0.95800	38.79200
O	-3.36200	9.08700	38.96100
H	-3.38500	9.18600	38.00900
H	-3.05200	9.93300	39.28200
O	-0.70300	11.14400	43.07500
H	0.25100	11.11300	43.00800
H	-0.90200	10.68300	43.89000
O	-0.11300	9.71500	45.64300
H	0.70600	10.20900	45.66600
H	0.16100	8.79800	45.61400
O	9.64400	2.10600	35.00500
H	8.75600	2.39100	34.78700
H	10.05900	1.94500	34.15800
O	12.99500	2.13000	38.72100
H	13.77100	2.62000	38.99300
H	12.27000	2.55600	39.17800
O	15.43200	2.84300	39.77800
H	16.02700	2.60300	39.06700
H	15.50500	2.12400	40.40500
O	-2.34800	16.68600	40.09200
H	-2.48200	15.92600	39.52600
H	-2.76500	17.41000	39.62500
O	8.78800	11.87000	29.10000
H	9.49200	12.07500	29.71600
H	8.06400	12.43800	29.36500
O	9.61800	7.62100	35.22000
H	9.56100	8.07800	34.38100
H	10.17100	6.86000	35.04300
O	13.42800	3.41000	36.04500
H	13.17200	3.01000	36.87600
H	12.80400	4.12400	35.92000
O	17.74000	6.15000	38.55200
H	17.18000	6.21900	39.32600
H	17.13400	5.97400	37.83200
O	0.80900	17.58300	40.30400
H	0.69200	18.52800	40.20200
H	-0.01200	17.28100	40.69300
O	10.69700	12.30800	31.15000
H	11.53100	11.87600	30.96800
H	10.53500	12.13700	32.07800
O	10.35100	8.81200	32.51600
H	10.29600	8.64600	31.57400
H	9.72600	9.51900	32.67200
O	-4.85900	4.04800	46.34200
H	-4.21400	3.53800	46.83000
H	-5.06800	4.78500	46.91600
O	3.66100	-1.68800	32.09400
H	3.64900	-0.74300	32.25100
H	4.55300	-1.87100	31.80000
O	3.59000	16.98900	40.18100
H	2.63900	16.98900	40.06700
H	3.89300	16.24200	39.66400
O	7.47600	-5.09300	36.34900
H	6.70900	-5.64800	36.49400
H	7.88700	-5.02100	37.21000
O	-0.52000	1.19500	50.43900
H	0.34300	0.83900	50.65000
H	-1.09300	0.85600	51.12700

O	13.83500	21.54700	30.51300
H	14.08800	21.02500	29.75200
H	12.89700	21.38300	30.61600
O	13.55500	23.45000	32.71600
H	13.43800	22.77400	33.38400
H	13.66500	22.96300	31.89900
O	15.53800	17.94000	31.41700
H	15.70600	18.80800	31.78400
H	15.34800	18.10200	30.49300
O	15.51700	20.39500	32.40100
H	15.39700	20.84100	33.24000
H	14.82200	20.74400	31.84300
O	12.15800	18.76200	31.66300
H	11.32200	19.10000	31.98400
H	12.01900	18.62700	30.72600
O	14.13300	13.60600	36.37100
H	13.48000	13.29100	35.74700
H	14.09100	12.98300	37.09700
O	15.24500	23.88800	28.28700
H	14.33700	23.65700	28.48600
H	15.74200	23.09100	28.47400
O	6.99200	21.70900	36.79100
H	6.79500	21.11600	36.06600
H	6.43700	21.40500	37.50800
O	16.12100	25.53000	30.14800
H	15.68300	24.81300	29.68800
H	16.86200	25.75700	29.58600
O	9.48700	21.23600	34.47500
H	8.55700	21.17400	34.69200
H	9.58300	20.71300	33.67800
O	9.40500	17.76100	37.06300
H	9.66600	18.67600	36.95300
H	10.22700	17.29400	37.21200
O	20.24200	24.38800	29.74100
H	19.62500	23.66300	29.64400
H	19.77400	25.14900	29.39800
O	12.03200	18.60700	28.86300
H	11.15500	18.48600	28.49900
H	12.50800	17.81500	28.61100
O	6.76000	20.20100	34.30500
H	7.05500	19.29200	34.25100
H	5.85900	20.18000	33.98300
O	8.48900	17.88200	34.42800
H	8.16000	17.00200	34.24600
H	8.84300	17.82700	35.31600
O	11.60300	18.17000	34.79600
H	11.94200	18.69800	34.07400
H	12.05000	18.50800	35.57200
O	13.59000	16.66700	32.99500
H	14.31000	17.04500	32.49200
H	12.96600	17.38600	33.09900
O	17.73300	17.37200	33.93100
H	17.94900	18.25400	33.63000
H	17.25200	16.98000	33.20200
O	13.73600	16.20200	35.82500
H	13.85800	15.27400	36.02400
H	13.44600	16.21600	34.91300
O	9.73400	19.54700	32.42100
H	9.04100	19.62100	31.76500
H	9.48900	18.78300	32.94400
O	16.59300	21.61800	28.72900
H	17.53900	21.48900	28.79800
H	16.21500	20.88000	29.20600
O	14.77700	19.54300	28.66500
H	13.87100	19.24700	28.74100
H	15.05500	19.24600	27.79800
O	16.39400	14.08700	34.81900
H	15.58400	13.74200	35.19500
H	17.08200	13.77800	35.40800
O	18.32300	13.16500	36.44200
H	18.87600	13.58400	37.10100
H	18.56000	12.23800	36.48400
O	13.54700	16.40200	28.80600
H	14.40900	16.72700	29.06600
H	13.73200	15.61500	28.29300
O	14.50000	13.64200	32.09400
H	14.48800	14.58600	32.25100
H	15.39300	13.45900	31.80000
O	18.31600	10.23600	36.34900
H	17.54900	9.68200	36.49400
H	18.72700	10.30800	37.21000
O	-14.94500	-40.68300	19.20700
H	-15.59700	-40.09700	18.82100
H	-14.15100	-40.15200	19.26700
O	-13.46800	-42.99400	19.74400
H	-14.10300	-42.92000	20.45600
H	-13.79800	-42.40400	19.06600
O	-19.24000	-35.81600	22.77500
H	-19.31800	-34.86500	22.85400
H	-19.84400	-36.16100	23.43200
O	-11.30200	-48.11400	19.95400
H	-10.39100	-47.88100	19.77900
H	-11.81100	-47.53000	19.39100
O	-16.96600	-40.45700	21.49500
H	-17.10200	-39.61200	21.92400
H	-16.42500	-40.25500	20.73200
O	-13.65200	-46.94300	18.53200
H	-14.16700	-46.17300	18.77100
H	-13.92400	-47.61700	19.15600
O	-8.67900	-44.19000	20.46800
H	-9.45600	-44.52900	20.02300
H	-8.00000	-44.18500	19.79300
O	-17.27400	-45.09400	15.01100
H	-18.18100	-45.32500	15.21000
H	-16.77600	-45.89000	15.19900
O	-14.36700	-36.38400	17.93100
H	-15.04100	-36.08100	18.53900
H	-13.98000	-35.58100	17.58100
O	-10.86300	-41.38200	23.30300
H	-11.31700	-42.22300	23.35900
H	-11.49300	-40.74600	23.64000
O	-12.42000	-39.54400	24.23800
H	-12.12700	-38.98400	23.51900
H	-11.91000	-39.25100	24.99300
O	-16.39700	-43.45200	16.87200
H	-16.83500	-44.16800	16.41300
H	-15.65600	-43.22400	16.31100
O	-8.28000	-45.17600	22.93000

H	-8.41800	-44.98600	22.00200
H	-8.63600	-44.41400	23.38700
O	-16.16800	-36.21600	20.14600
H	-15.99200	-36.68700	20.96000
H	-16.85400	-35.58900	20.37400
O	-12.27600	-44.59300	16.46600
H	-12.89300	-45.31800	16.36900
H	-12.74400	-43.83200	16.12200
O	-17.30000	-38.02200	22.83400
H	-18.08300	-37.48000	22.92800
H	-16.60000	-37.50500	23.23300
O	-11.45200	-41.26900	20.58500
H	-11.17600	-41.38400	21.49400
H	-12.32200	-41.66700	20.54600
O	-14.81700	-37.02100	24.22800
H	-14.63500	-37.83800	24.69100
H	-13.99400	-36.80400	23.79100
O	-14.48300	-38.09400	21.49000
H	-13.95400	-38.60500	20.87700
H	-14.22000	-38.40900	22.35400
O	-11.38900	-38.12200	22.10500
H	-11.21500	-38.68700	21.35300
H	-10.89600	-37.32200	21.92300
O	-5.53500	-45.35800	23.72400
H	-5.09200	-46.10800	23.32600
H	-6.41200	-45.36700	23.34200
O	-10.40100	-38.80500	19.20500
H	-9.70600	-38.18500	19.42400
H	-10.05900	-39.65600	19.48000
O	-8.50200	-41.35500	20.73400
H	-7.97700	-41.01700	20.00900
H	-8.59000	-42.29100	20.55000
O	-16.31000	-38.69900	18.11200
H	-16.88200	-38.08000	17.65900
H	-15.73600	-38.14900	18.64600
O	-13.09000	-38.41600	19.24300
H	-12.15700	-38.48100	19.44700
H	-13.17800	-37.58000	18.78500
O	-10.46100	-39.09900	16.20400
H	-10.54000	-39.43300	17.09700
H	-9.64000	-38.60700	16.20300
O	-15.92500	-47.36300	15.45400
H	-14.97900	-47.49300	15.52300
H	-16.30300	-48.10200	15.93100
O	-12.89100	-41.78200	15.82500
H	-12.18700	-41.57700	16.44000
H	-13.61400	-41.21400	16.09000
O	-10.98200	-41.34400	17.87400
H	-10.14700	-41.77600	17.69200
H	-11.14400	-41.51600	18.80200
O	-11.32700	-44.84100	19.24000
H	-11.38300	-45.00600	18.29900
H	-11.95300	-44.13300	19.39600
O	-2.35400	-30.82700	27.97000
H	-1.71600	-30.94600	27.26600
H	-2.47100	-29.87900	28.02900
O	-5.54500	-30.87400	27.90600
H	-5.88300	-30.99000	27.01800
H	-4.67600	-31.27500	27.88600
O	-9.68600	-29.02100	23.89600
H	-10.24900	-29.11200	23.12700
H	-9.80100	-29.84100	24.37600
O	-12.13900	-27.52500	26.73400
H	-11.34700	-27.86600	26.31900
H	-11.90000	-27.41600	27.65400
O	-4.10600	-25.35400	19.20700
H	-4.75700	-24.76800	18.82100
H	-3.31200	-24.82300	19.26700
O	-10.18000	-28.26500	19.60600
H	-9.40700	-28.81200	19.46700
H	-9.82800	-27.40000	19.81700
O	-8.34200	-26.93700	32.81300
H	-8.63300	-26.03200	32.69700
H	-7.70600	-26.89500	33.52700
O	-7.41600	-29.55900	29.40300
H	-6.82000	-29.78400	28.68900
H	-7.35900	-30.30100	30.00500
O	-7.84400	-32.10500	17.23800
H	-7.59100	-32.62700	16.47600
H	-8.78100	-32.26900	17.34100
O	-2.79900	-33.10200	25.04500
H	-2.01300	-33.52000	24.69400
H	-2.53600	-32.19700	25.21300
O	-2.62800	-27.66400	19.74400
H	-3.26400	-27.59100	20.45600
H	-2.95900	-27.07500	19.06600
O	2.60100	-33.16400	25.94900
H	3.33400	-33.73400	26.18200
H	1.84500	-33.55200	26.38900
O	-8.12400	-30.20300	19.44000
H	-8.24100	-30.87800	20.10800
H	-8.01400	-30.68900	18.62300
O	-5.78100	-30.74200	23.14500
H	-6.14500	-31.26800	23.85600
H	-4.92400	-31.13200	22.97200
O	-6.14000	-35.71300	18.14100
H	-5.97300	-34.84400	18.50800
H	-6.33100	-35.55000	17.21700
O	-3.38300	-27.25100	29.80800
H	-3.68300	-26.34800	29.69900
H	-3.72800	-27.51800	30.66000
O	-9.93200	-30.79900	25.84100
H	-10.85700	-30.93200	26.04900
H	-9.68300	-30.02000	26.33800
O	0.01700	-34.73300	27.48700
H	-0.89200	-34.80100	27.19300
H	-0.03400	-34.25500	28.31600
O	-6.16200	-33.25700	19.12600
H	-6.28200	-32.81100	19.96400
H	-6.85600	-32.90800	18.56700
O	-12.57100	-31.15600	26.67600
H	-12.44200	-30.90300	27.59000
H	-13.25100	-30.56300	26.35600
O	-8.40100	-20.48600	22.77500
H	-8.47800	-19.53600	22.85400
H	-9.00500	-20.83200	23.43200
O	1.88300	-36.53100	29.26500
H	1.24000	-36.23800	28.61900

H	1.37800	-36.64600	30.07100
O	-0.46300	-32.78500	19.95400
H	0.44900	-32.55200	19.77900
H	-0.97200	-32.20100	19.39100
O	-2.17900	-28.36200	33.59000
H	-1.77100	-28.80400	32.84500
H	-2.90100	-28.93700	33.84300
O	-1.85600	-27.80200	27.34500
H	-2.48500	-27.63700	26.64300
H	-2.24300	-27.38800	28.11600
O	2.02300	-26.86700	24.70000
H	1.33800	-26.48700	24.14900
H	2.10700	-26.25700	25.43300
O	-0.72200	-29.22100	31.29900
H	-0.51000	-28.57700	30.62300
H	-0.76900	-30.05400	30.83100
O	-3.11900	-36.60900	28.20300
H	-3.98100	-36.31100	28.49400
H	-3.09300	-36.39200	27.27100
O	-6.30300	-28.06500	24.61000
H	-5.83900	-27.78600	23.82100
H	-6.56800	-28.96600	24.42700
O	-9.41100	-28.51700	27.54800
H	-8.84100	-28.08900	26.91000
H	-8.82000	-28.79900	28.24700
O	-7.36600	-31.94200	21.41900
H	-6.66400	-31.49000	21.88600
H	-7.69800	-32.58400	22.04700
O	-13.62400	-33.68400	26.72100
H	-14.41400	-33.42800	26.24500
H	-13.13300	-32.86900	26.82600
O	0.39300	-26.34300	29.47100
H	1.21400	-26.74500	29.19000
H	0.38900	-26.44700	30.42300
O	-11.67200	-33.31900	23.67900
H	-12.46900	-33.20800	24.19800
H	-11.90000	-32.98100	22.81300
O	-5.84200	-26.79500	31.62700
H	-5.77700	-26.33300	32.46300
H	-6.55100	-27.42500	31.75600
O	8.48300	-30.00000	25.82000
H	7.73000	-30.56900	25.66400
H	8.61800	-30.03300	26.76700
O	-6.12700	-25.12800	21.49500
H	-6.26200	-24.28300	21.92400
H	-5.58600	-24.92600	20.73200
O	-12.67400	-27.61100	22.06800
H	-12.16200	-26.81600	22.21900
H	-12.02200	-28.30800	22.00100
O	-10.86500	-24.49400	24.15000
H	-10.68800	-23.57200	23.96200
H	-10.06900	-24.80800	24.58000
O	-4.50000	-24.50600	35.62500
H	-4.94400	-25.29200	35.30700
H	-3.83800	-24.31800	34.96000
O	-9.74100	-22.97600	30.15100
H	-9.19100	-23.10500	29.37900
H	-10.58000	-22.67700	29.80100
O	-2.40200	-24.22600	33.65300
H	-2.42000	-23.40500	33.16200
H	-1.48000	-24.35000	33.87900
O	-14.70900	-29.28200	22.84400
H	-13.87200	-28.87200	22.62700
H	-14.53100	-30.22200	22.81400
O	-2.81300	-31.61400	18.53200
H	-3.32800	-30.84400	18.77100
H	-3.08500	-32.28800	19.15600
O	-5.22800	-36.76900	23.59100
H	-6.08400	-37.15900	23.41200
H	-4.81500	-36.69000	22.73100
O	-3.69800	-39.23500	27.99000
H	-4.20200	-39.27200	27.17700
H	-3.40500	-38.32500	28.05000
O	2.16100	-28.86100	20.46800
H	1.38400	-29.20000	20.02300
H	2.84000	-28.85600	19.79300
O	-3.02400	-33.74200	20.35800
H	-3.32900	-33.42700	21.20900
H	-2.07100	-33.66300	20.40100
O	-4.57900	-27.58300	22.00800
H	-5.10700	-28.31700	21.69500
H	-5.08100	-26.80500	21.76200
O	0.40000	-29.38800	27.12400
H	-0.25800	-28.69800	27.21200
H	1.20400	-28.99800	27.46800
O	-9.84800	-36.92100	24.36800
H	-9.27300	-37.14300	23.63600
H	-9.26000	-36.85200	25.12100
O	-0.79000	-31.03300	25.17500
H	-0.27600	-30.40000	25.67700
H	-0.14000	-31.63300	24.80900
O	-9.52100	-34.89000	18.38800
H	-10.35700	-34.55300	18.70900
H	-9.66000	-35.02500	17.45100
O	-7.54500	-40.04600	23.09600
H	-8.19900	-40.36200	22.47200
H	-7.58700	-40.66900	23.82200
O	-6.43400	-29.76500	15.01100
H	-7.34100	-29.99600	15.21000
H	-5.93700	-30.56100	15.19900
O	-3.52700	-21.05500	17.93100
H	-4.20100	-20.75200	18.53900
H	-3.14100	-20.25200	17.58100
O	-4.31200	-33.55800	27.42100
H	-5.22900	-33.33700	27.26300
H	-3.89600	-33.48400	26.56200
O	-3.52800	-32.15900	22.55100
H	-2.78100	-31.64700	22.24200
H	-3.25900	-32.48100	23.41100
O	-5.25500	-29.66300	19.53400
H	-6.16400	-29.94700	19.63100
H	-5.22600	-29.24300	18.67400
O	-0.02300	-26.05300	23.30300
H	-0.47700	-26.89300	23.35900
H	-0.65400	-25.41600	23.64000
O	-1.08000	-35.12300	24.53300
H	-1.81800	-35.63600	24.86400
H	-0.82700	-35.56300	23.72100

O	-1.58000	-24.21500	24.23800
H	-1.28800	-23.65400	23.51900
H	-1.07000	-23.92200	24.99300
O	-0.97900	-42.34600	25.12700
H	-0.67300	-43.20600	24.84000
H	-0.25400	-41.75400	24.92700
O	-14.68700	-31.94300	23.51500
H	-14.88400	-32.53600	22.79000
H	-15.24200	-32.24700	24.23300
O	-6.70700	-32.05000	25.63500
H	-6.65200	-33.00300	25.55900
H	-7.51800	-31.89400	26.11900
O	-5.55800	-28.12200	16.87200
H	-5.99600	-28.83900	16.41300
H	-4.81700	-27.89500	16.31100
O	-8.76800	-33.43300	23.39400
H	-9.58100	-33.13300	23.80000
H	-8.27200	-33.82700	24.11100
O	-6.61700	-23.43700	25.32700
H	-6.65900	-23.21100	24.39800
H	-5.70100	-23.67100	25.47600
O	2.56000	-29.84700	22.93000
H	2.42100	-29.65600	22.00200
H	2.20300	-29.08500	23.38700
O	2.58600	-27.77900	27.94100
H	3.02000	-27.87100	28.78900
H	3.30000	-27.80300	27.30300
O	-8.50000	-26.56100	25.12300
H	-7.70600	-27.05600	24.92200
H	-9.21100	-27.08900	24.76000
O	-3.68100	-24.17900	29.47400
H	-3.62700	-23.61000	30.24200
H	-4.54900	-24.00900	29.11000
O	-12.19200	-32.41600	21.19900
H	-13.12200	-32.47800	21.41600
H	-12.09600	-32.93900	20.40300
O	-12.27300	-35.89100	23.78700
H	-12.01300	-34.97600	23.67800
H	-11.45200	-36.35800	23.93700
O	3.44800	-27.73700	30.47400
H	3.64200	-26.83200	30.72000
H	3.34800	-28.19600	31.30800
O	-1.49000	-27.58600	36.04900
H	-1.64200	-27.81500	35.13200
H	-1.50000	-26.62900	36.06200
O	-4.72200	-30.01300	31.03300
H	-3.79300	-29.86200	30.86000
H	-5.14300	-29.92400	30.17800
O	-5.92300	-25.98800	28.80300
H	-6.06200	-26.35600	29.67600
H	-6.71200	-25.47300	28.63100
O	-5.32800	-20.88700	20.14600
H	-5.15300	-21.35800	20.96000
H	-6.01500	-20.26000	20.37400
O	-4.37300	-26.61000	26.19200
H	-4.70800	-26.64900	27.08800
H	-4.87200	-27.27500	25.71900
O	-1.43700	-29.26400	16.46600
H	-2.05400	-29.98900	16.36900
H	-1.90500	-28.50300	16.12200
O	0.21900	-24.55400	34.12600
H	0.16000	-25.23800	33.45900
H	0.62100	-23.81200	33.67400
O	-10.76200	-17.73800	26.92000
H	-10.52200	-17.10600	27.59700
H	-11.60500	-18.08900	27.20600
O	-9.64700	-35.04500	15.58800
H	-10.52400	-35.16700	15.22400
H	-9.17100	-35.83700	15.33600
O	0.22100	-26.59000	32.35800
H	-0.66700	-26.92700	32.48000
H	0.79000	-27.32200	32.59400
O	-18.12700	-30.25800	25.68200
H	-17.43900	-30.57200	25.09400
H	-18.28200	-29.35400	25.40900
O	-6.24900	-21.93000	29.32400
H	-6.32200	-21.12400	29.83600
H	-6.69200	-21.73100	28.49900
O	-6.46000	-22.69300	22.83400
H	-7.24400	-22.15100	22.92800
H	-5.76100	-22.17500	23.23300
O	-3.15100	-36.88300	25.47700
H	-2.65400	-37.63600	25.15800
H	-3.92200	-36.84700	24.91200
O	4.70200	-31.17900	27.11900
H	4.69100	-30.29700	26.74900
H	3.83400	-31.53200	26.92500
O	-3.79000	-22.03100	31.48000
H	-3.07700	-21.39700	31.40700
H	-4.28100	-21.93500	30.66400
O	-2.06800	-28.12600	23.05100
H	-3.00200	-28.00600	22.88200
H	-1.89000	-29.02900	22.78700
O	-6.56700	-34.74200	25.20500
H	-6.93700	-35.29400	25.89300
H	-6.07100	-35.34600	24.65200
O	1.32000	-32.28400	23.58600
H	1.78600	-31.45200	23.51200
H	1.77000	-32.75000	24.29100
O	-8.73800	-24.28200	32.30000
H	-8.03700	-23.64800	32.45300
H	-9.20900	-23.94000	31.54000
O	-0.61300	-25.94000	20.58500
H	-0.33700	-26.05400	21.49400
H	-1.48200	-26.33800	20.54600
O	-0.70900	-39.91600	27.62500
H	-1.62300	-39.68600	27.79400
H	-0.28000	-39.83200	28.47700
O	-0.93800	-31.72700	30.12500
H	-1.59100	-31.48000	29.47100
H	-0.56000	-32.54100	29.79200
O	-3.97700	-21.69100	24.22800
H	-3.79500	-22.50900	24.69100
H	-3.15400	-21.47500	23.79100
O	-3.64300	-22.76500	21.49000
H	-3.11400	-23.27500	20.87700
H	-3.38000	-23.08000	22.35400
O	-13.14200	-24.74800	29.23300

H	-12.55600	-25.49400	29.36600
H	-13.33900	-24.43800	30.11700
O	-10.28800	-21.71300	24.24900
H	-10.95700	-21.22800	23.76600
H	-10.42600	-21.46600	25.16400
O	-6.14700	-19.67900	30.84800
H	-5.59500	-19.51600	31.61300
H	-5.80700	-19.08800	30.17600
O	-1.29600	-24.85300	36.70300
H	-0.75200	-24.24000	36.20800
H	-1.87700	-24.29500	37.21900
O	-14.91900	-33.45100	21.02900
H	-14.62400	-34.36000	20.97600
H	-15.82000	-33.47200	20.70700
O	-3.01000	-21.73000	27.83400
H	-3.13500	-22.53200	27.32500
H	-2.35200	-21.23600	27.34500
O	-11.38900	-35.37600	27.01200
H	-12.11100	-34.76200	26.87300
H	-10.60000	-34.84000	26.93300
O	-0.94700	-23.93000	29.46200
H	-1.89400	-24.04900	29.39000
H	-0.59900	-24.81700	29.54500
O	-13.19000	-35.77000	21.15300
H	-13.51900	-36.65000	20.97000
H	-12.83600	-35.82500	22.04100
O	-3.94200	-24.13400	25.66200
H	-4.09000	-25.06300	25.84300
H	-3.11700	-24.11200	25.17800
O	-10.07600	-35.48200	21.52100
H	-9.73600	-34.95400	20.79800
H	-9.62900	-35.14400	22.29600
O	-0.33900	-23.31100	26.74900
H	0.58600	-23.51700	26.61100
H	-0.50700	-23.56000	27.65800
O	-8.08900	-36.98500	19.72000
H	-7.36800	-36.60700	19.21600
H	-8.71300	-36.26600	19.82300
O	-3.94600	-36.28100	20.65600
H	-3.73000	-35.39800	20.35500
H	-4.42700	-36.67200	19.92600
O	-9.20600	-33.32200	27.23300
H	-9.35200	-33.00800	28.12500
H	-9.68400	-32.70300	26.68000
O	-7.34600	-36.33200	27.28100
H	-7.36000	-37.22800	26.94400
H	-6.62900	-36.32500	27.91500
O	-0.54900	-22.79300	22.10500
H	-0.37600	-23.35800	21.35300
H	-0.05600	-21.99300	21.92300
O	-7.94300	-37.45000	22.55000
H	-7.82100	-38.37900	22.74900
H	-8.23300	-37.43600	21.63700
O	-5.07400	-39.76500	25.68600
H	-5.05100	-39.70100	24.73100
H	-4.65700	-40.60400	25.88000
O	5.30500	-30.02900	23.72400
H	5.74800	-30.77900	23.32600
H	4.42700	-30.03800	23.34200
O	0.43800	-23.47600	19.20500
H	1.13300	-22.85600	19.42400
H	0.78100	-24.32700	19.48000
O	2.33700	-26.02600	20.73400
H	2.86300	-25.68800	20.00900
H	2.25000	-26.96200	20.55000
O	-6.00100	-22.11300	32.86300
H	-5.69200	-22.35800	33.73500
H	-5.20300	-21.90800	32.37400
O	-5.97900	-19.95900	25.09300
H	-6.41800	-20.45100	25.78600
H	-5.22800	-20.50200	24.85200
O	-13.49900	-23.76800	25.09600
H	-12.67300	-24.06800	24.71500
H	-13.85000	-24.53700	25.54500
O	-8.53100	-24.41500	27.16500
H	-9.15300	-25.07800	26.86600
H	-7.86100	-24.38900	26.48300
O	-15.29800	-25.84900	26.05400
H	-16.13500	-26.27700	25.87300
H	-15.32600	-25.65600	26.99200
O	2.02700	-25.39300	36.03900
H	1.23600	-25.41100	35.49900
H	1.79000	-24.85400	36.79400
O	2.14700	-28.54400	33.35200
H	2.19600	-28.70900	34.29400
H	2.03800	-29.41100	32.96200
O	-8.97000	-25.86500	20.64100
H	-8.82100	-25.18100	19.98800
H	-8.16300	-25.88400	21.15600
O	3.64800	-26.12400	33.59600
H	3.35700	-25.76100	34.43300
H	3.03600	-26.83700	33.41800
O	4.84800	-28.55900	26.11600
H	5.68300	-28.12000	26.28000
H	4.98400	-29.03500	25.29700
O	1.89700	-31.00100	32.06400
H	2.37300	-30.80800	31.25600
H	0.97400	-30.94500	31.81900
O	-11.21500	-29.83800	21.64700
H	-11.46000	-30.74200	21.84500
H	-11.04500	-29.83700	20.70500
O	-11.94500	-34.10500	19.14600
H	-12.63800	-34.03100	18.49000
H	-12.19000	-34.86900	19.66800
O	-5.47000	-23.37000	18.11200
H	-6.04300	-22.75100	17.65900
H	-4.89700	-22.82000	18.64600
O	0.59700	-34.12400	30.29700
H	1.54900	-34.02400	30.31800
H	0.42800	-34.91600	30.80700
O	-2.25100	-23.08600	19.24300
H	-1.31700	-23.15200	19.44700
H	-2.33800	-22.25100	18.78500
O	0.37800	-23.77000	16.20400
H	0.30000	-24.10400	17.09700
H	1.19900	-23.27800	16.20300
O	3.07600	-30.90400	29.41100
H	3.04400	-31.84200	29.59700

H	3.68600	-30.82500	28.67800
O	-5.08600	-32.03400	15.45400
H	-4.14000	-32.16300	15.52300
H	-5.46400	-32.77200	15.93100
O	3.22600	-34.11200	29.45700
H	2.70900	-34.89700	29.27000
H	4.11000	-34.44100	29.62200
O	0.88700	-40.36800	25.41700
H	0.68900	-40.67700	26.30100
H	1.49700	-39.64200	25.54600
O	8.89400	-29.84200	28.55200
H	9.62400	-30.10300	29.11300
H	8.28400	-30.57900	28.59200
O	6.97500	-31.70100	29.14900
H	6.24900	-31.83600	28.54100
H	6.56000	-31.39300	29.95500
O	-6.90100	-34.10900	15.38900
H	-7.80800	-34.40600	15.46600
H	-6.62400	-34.40600	14.52300
O	-15.98300	-32.78800	25.83900
H	-16.30300	-33.64200	25.54700
H	-16.53000	-32.57300	26.59500
O	-5.28500	-39.56500	21.54400
H	-6.09500	-39.91100	21.91900
H	-4.59700	-39.87500	22.13300
O	-3.35600	-40.48700	23.16600
H	-2.80300	-40.06800	23.82500
H	-3.11900	-41.41400	23.20900
O	-8.13100	-37.25000	15.53100
H	-7.26900	-36.92500	15.79000
H	-7.94600	-38.03700	15.01800
O	-16.11500	-29.16800	27.80900
H	-16.80800	-29.58300	27.29600
H	-15.36700	-29.12600	27.21200
O	0.02200	-38.37700	22.67400
H	-0.55800	-37.71900	22.29200
H	-0.24500	-39.20000	22.26300
O	-1.52800	-38.97700	24.87600
H	-1.02800	-38.86500	24.06800
H	-0.88400	-39.28100	25.51600
O	-14.20100	-29.23600	25.68500
H	-14.22400	-29.13700	24.73300
H	-13.89100	-28.39000	26.00700
O	-11.54200	-27.17900	29.80000
H	-10.58800	-27.21000	29.73300
H	-11.74100	-27.64000	30.61500
O	-1.19600	-36.21700	21.73000
H	-2.08300	-35.93200	21.51200
H	-0.78000	-36.37800	20.88300
O	2.15600	-36.19300	25.44500
H	2.93200	-35.70300	25.71800
H	1.43100	-35.76700	25.90200
O	4.59300	-35.48000	26.50300
H	5.18800	-35.72000	25.79200
H	4.66600	-36.19900	27.13000
O	-13.18700	-21.63700	26.81700
H	-13.32200	-22.39700	26.25100
H	-13.60400	-20.91300	26.35000
O	-2.05100	-26.45300	15.82500
H	-1.34800	-26.24800	16.44000
H	-2.77500	-25.88500	16.09000
O	-1.22200	-30.70200	21.94500
H	-1.27800	-30.24500	21.10600
H	-0.66900	-31.46300	21.76700
O	2.58900	-34.91300	22.77000
H	2.33300	-35.31300	23.60100
H	1.96400	-34.19900	22.64500
O	6.90000	-32.17300	25.27700
H	6.34000	-32.10400	26.05000
H	6.29500	-32.34900	24.55700
O	-10.03000	-20.74000	27.02900
H	-10.14700	-19.79500	26.92700
H	-10.85100	-21.04200	27.41700
O	-0.14300	-26.01500	17.87400
H	0.69200	-26.44700	17.69200
H	-0.30400	-26.18600	18.80200
O	-0.48800	-29.51100	19.24000
H	-0.54300	-29.67700	18.29900
H	-1.11300	-28.80400	19.39600
O	-7.17800	-40.01100	18.81900
H	-7.19100	-39.06600	18.97500
H	-6.28600	-40.19400	18.52500
O	-9.00500	-20.08700	30.24400
H	-8.10200	-20.05500	30.56100
H	-9.23100	-21.01700	30.25900
O	-7.24900	-21.33400	26.90500
H	-8.20000	-21.33400	26.79200
H	-6.94600	-22.08000	26.38900
O	-3.36300	-43.41600	23.07300
H	-4.13000	-43.97100	23.21900
H	-2.95200	-43.34400	23.93500
O	8.48600	-15.49800	27.97000
H	9.12400	-15.61700	27.26600
H	8.36800	-14.55000	28.02900
O	5.29500	-15.54500	27.90600
H	4.95700	-15.66100	27.01800
H	6.16400	-15.94600	27.88600
O	1.15300	-13.69100	23.89600
H	0.59000	-13.78200	23.12700
H	1.03900	-14.51100	24.37600
O	5.77700	-14.39100	33.86500
H	5.76600	-14.78600	32.99300
H	5.93900	-15.12500	34.45800
O	-1.30000	-12.19600	26.73400
H	-0.50800	-12.53700	26.31900
H	-1.06000	-12.08700	27.65400
O	6.73300	-10.02400	19.20700
H	6.08200	-9.43900	18.82100
H	7.52800	-9.49400	19.26700
O	0.65900	-12.93500	19.60600
H	1.43200	-13.48300	19.46700
H	1.01200	-12.07100	19.81700
O	2.49700	-11.60800	32.81300
H	2.20600	-10.70300	32.69700
H	3.13300	-11.56600	33.52700
O	3.42300	-14.23000	29.40300
H	4.01900	-14.45400	28.68900
H	3.48100	-14.97200	30.00500

O	2.99600	-16.77600	17.23800
H	3.24800	-17.29800	16.47600
H	2.05800	-16.94000	17.34100
O	8.04000	-17.77300	25.04500
H	8.82600	-18.19100	24.69400
H	8.30400	-16.86800	25.21300
O	8.21100	-12.33500	19.74400
H	7.57600	-12.26200	20.45600
H	7.88100	-11.74600	19.06600
O	13.44100	-17.83500	25.94900
H	14.17300	-18.40500	26.18200
H	12.68500	-18.22300	26.38900
O	2.71500	-14.87300	19.44000
H	2.59900	-15.54900	20.10800
H	2.82500	-15.36000	18.62300
O	5.05800	-15.41200	23.14500
H	4.69400	-15.93900	23.85600
H	5.91500	-15.80200	22.97200
O	10.96300	-24.12300	30.40800
H	10.63200	-23.25300	30.18400
H	10.36800	-24.43900	31.08700
O	4.69900	-20.38300	18.14100
H	4.86600	-19.51500	18.50800
H	4.50900	-20.22100	17.21700
O	7.45600	-11.92200	29.80800
H	7.15700	-11.01900	29.69900
H	7.11200	-12.18800	30.66000
O	0.90800	-15.47000	25.84100
H	-0.01700	-15.60300	26.04900
H	1.15600	-14.69100	26.33800
O	10.85600	-19.40300	27.48700
H	9.94800	-19.47200	27.19300
H	10.80500	-18.92600	28.31600
O	4.67800	-17.92800	19.12600
H	4.55800	-17.48200	19.96400
H	3.98300	-17.57900	18.56700
O	-1.73100	-15.82700	26.67600
H	-1.60200	-15.57300	27.59000
H	-2.41200	-15.23400	26.35600
O	2.43900	-5.15700	22.77500
H	2.36100	-4.20600	22.85400
H	1.83500	-5.50300	23.43200
O	4.93000	-17.98400	35.43100
H	4.15400	-17.42500	35.42100
H	5.56900	-17.49900	35.95200
O	12.72200	-21.20200	29.26500
H	12.08000	-20.90800	28.61900
H	12.21800	-21.31600	30.07100
O	10.37600	-17.45500	19.95400
H	11.28800	-17.22300	19.77900
H	9.86700	-16.87200	19.39100
O	3.18500	-15.17600	34.99300
H	3.92600	-14.63700	34.71600
H	2.47900	-14.54900	35.15000
O	8.66000	-13.03300	33.59000
H	9.06900	-13.47500	32.84500
H	7.93800	-13.60800	33.84300
O	5.01400	-18.09600	30.96300
H	5.86000	-17.67200	31.10700
H	4.95300	-18.75200	31.65700
O	8.98300	-12.47300	27.34500
H	8.35400	-12.30800	26.64300
H	8.59600	-12.05900	28.11600
O	12.86200	-11.53700	24.70000
H	12.17700	-11.15700	24.14900
H	12.94700	-10.92800	25.43300
O	8.16300	-20.43500	32.62100
H	7.92000	-19.68100	32.08500
H	8.43900	-20.05700	33.45600
O	10.11700	-13.89100	31.29900
H	10.32900	-13.24800	30.62300
H	10.07000	-14.72500	30.83100
O	7.72000	-21.28000	28.20300
H	6.85800	-20.98100	28.49400
H	7.74600	-21.06300	27.27100
O	4.53600	-12.73500	24.61000
H	5.00000	-12.45700	23.82100
H	4.27200	-13.63700	24.42700
O	1.42900	-13.18800	27.54800
H	1.99900	-12.76000	26.91000
H	2.01900	-13.47000	28.24700
O	3.47300	-16.61300	21.41900
H	4.17500	-16.16100	21.88600
H	3.14100	-17.25500	22.04700
O	1.92100	-18.02200	35.79400
H	1.29100	-17.34000	36.02800
H	1.81500	-18.13400	34.85000
O	-2.78400	-18.35500	26.72100
H	-3.57500	-18.09900	26.24500
H	-2.29300	-17.54000	26.82600
O	11.23200	-11.01400	29.47100
H	12.05400	-11.41600	29.19000
H	11.22800	-11.11700	30.42300
O	-0.83300	-17.99000	23.67900
H	-1.63000	-17.87800	24.19800
H	-1.06000	-17.65200	22.81300
O	4.99800	-11.46600	31.62700
H	5.06200	-11.00400	32.46300
H	4.28900	-12.09600	31.75600
O	-4.50100	-18.10200	29.15200
H	-4.25000	-17.21700	29.41800
H	-3.96500	-18.28100	28.38000
O	19.32300	-14.67000	25.82000
H	18.56900	-15.24000	25.66400
H	19.45700	-14.70400	26.76700
O	4.71200	-9.79900	21.49500
H	4.57700	-8.95400	21.92400
H	5.25300	-9.59700	20.73200
O	1.19900	-23.72000	38.15700
H	0.80600	-24.27300	38.83200
H	2.01600	-23.40900	38.54700
O	-1.83500	-12.28100	22.06800
H	-1.32200	-11.48700	22.21900
H	-1.18200	-12.97900	22.00100
O	-0.02500	-9.16500	24.15000
H	0.15100	-8.24300	23.96200
H	0.77000	-9.47900	24.58000
O	4.11600	-22.30400	35.74900

H	4.85700	-21.70300	35.66900
H	4.34800	-23.04600	35.19100
O	-0.74000	-17.11800	34.02700
H	-0.85500	-18.02000	34.32700
H	-1.59600	-16.87200	33.67500
O	6.33900	-9.17700	35.62500
H	5.89600	-9.96300	35.30700
H	7.00100	-8.98900	34.96000
O	1.09800	-7.64700	30.15100
H	1.64900	-7.77500	29.37900
H	0.25900	-7.34800	29.80100
O	8.43700	-8.89600	33.65300
H	8.41900	-8.07500	33.16200
H	9.35900	-9.02100	33.87900
O	4.89500	-11.38200	34.49600
H	5.42700	-12.14300	34.26600
H	4.36600	-11.67500	35.23800
O	8.06200	-26.27600	30.02500
H	7.71800	-25.38600	29.94900
H	8.43000	-26.46600	29.16200
O	-3.87000	-13.95300	22.84400
H	-3.03200	-13.54300	22.62700
H	-3.69200	-14.89300	22.81400
O	8.02700	-16.28500	18.53200
H	7.51100	-15.51500	18.77100
H	7.75400	-16.95900	19.15600
O	5.61100	-21.44000	23.59100
H	4.75600	-21.82900	23.41200
H	6.02400	-21.36100	22.73100
O	7.14100	-23.90500	27.99000
H	6.63800	-23.94200	27.17700
H	7.43400	-22.99600	28.05000
O	3.38600	-23.82200	26.99700
H	4.25100	-24.01700	26.63700
H	3.45300	-24.05100	27.92400
O	13.00000	-13.53200	20.46800
H	12.22300	-13.87100	20.02300
H	13.67900	-13.52700	19.79300
O	7.81500	-18.41200	20.35800
H	7.51000	-18.09800	21.20900
H	8.76800	-18.33400	20.40100
O	6.26000	-12.25300	22.00800
H	5.73200	-12.98800	21.69500
H	5.75900	-11.47600	21.76200
O	11.23900	-14.05800	27.12400
H	10.58200	-13.36900	27.21200
H	12.04300	-13.66900	27.46800
O	0.99200	-21.59100	24.36800
H	1.56600	-21.81400	23.63600
H	1.57900	-21.52200	25.12100
O	10.05000	-15.70400	25.17500
H	10.56300	-15.07100	25.67700
H	10.70000	-16.30400	24.80900
O	1.31900	-19.56100	18.38800
H	0.48200	-19.22300	18.70900
H	1.17900	-19.69600	17.45100
O	3.29400	-24.71700	23.09600
H	2.64100	-25.03200	22.47200
H	3.25200	-25.34000	23.82200
O	4.40500	-14.43500	15.01100
H	3.49800	-14.66600	15.21000
H	4.90200	-15.23200	15.19900
O	7.31200	-5.72600	17.93100
H	6.63800	-5.42200	18.53900
H	7.69900	-4.92300	17.58100
O	6.52800	-18.22900	27.42100
H	5.61000	-18.00800	27.26300
H	6.94400	-18.15400	26.56200
O	7.31100	-16.83000	22.55100
H	8.05900	-16.31800	22.24200
H	7.58000	-17.15200	23.41100
O	5.58400	-14.33400	19.53400
H	4.67500	-14.61800	19.63100
H	5.61400	-13.91400	18.67400
O	10.81600	-10.72300	23.30300
H	10.36200	-11.56400	23.35900
H	10.18500	-10.08700	23.64000
O	9.75900	-19.79400	24.53300
H	9.02100	-20.30600	24.86400
H	10.01200	-20.23400	23.72100
O	9.25900	-8.88600	24.23800
H	9.55200	-8.32500	23.51900
H	9.76900	-8.59300	24.99300
O	9.86000	-27.01700	25.12700
H	10.16600	-27.87700	24.84000
H	10.58600	-26.42500	24.92700
O	-3.84700	-16.61400	23.51500
H	-4.04500	-17.20700	22.79000
H	-4.40300	-16.91800	24.23300
O	4.13200	-16.72100	25.63500
H	4.18700	-17.67400	25.55900
H	3.32200	-16.56500	26.11900
O	5.28200	-12.79300	16.87200
H	4.84400	-13.51000	16.41300
H	6.02300	-12.56600	16.31100
O	2.07200	-18.10300	23.39400
H	1.25900	-17.80400	23.80000
H	2.56800	-18.49800	24.11100
O	4.22300	-8.10800	25.32700
H	4.18000	-7.88200	24.39800
H	5.13900	-8.34100	25.47600
O	7.66900	-17.63900	32.22500
H	8.02400	-17.41200	33.08500
H	8.31600	-17.30700	31.60300
O	13.39900	-14.51700	22.93000
H	13.26100	-14.32700	22.00200
H	13.04300	-13.75600	23.38700
O	1.56300	-20.53200	29.01000
H	2.20300	-20.67300	28.31200
H	0.73100	-20.81800	28.63400
O	13.42600	-12.45000	27.94100
H	13.86000	-12.54200	28.78900
H	14.13900	-12.47400	27.30300
O	2.33900	-11.23100	25.12300
H	3.13300	-11.72600	24.92200
H	1.62800	-11.76000	24.76000
O	7.15900	-8.85000	29.47400
H	7.21200	-8.28100	30.24200

H	6.29000	-8.67900	29.11000
O	-1.35300	-17.08700	21.19900
H	-2.28300	-17.14900	21.41600
H	-1.25700	-17.61000	20.40300
O	-1.43400	-20.56200	23.78700
H	-1.17300	-19.64700	23.67800
H	-0.61200	-21.02900	23.93700
O	0.03800	-19.14800	30.95700
H	0.71100	-19.35600	30.30800
H	-0.78900	-19.34600	30.51700
O	14.28700	-12.40700	30.47400
H	14.48100	-11.50300	30.72000
H	14.18700	-12.86700	31.30800
O	5.94100	-22.52200	32.97400
H	5.29400	-21.82300	32.88000
H	6.71100	-22.08800	33.34100
O	9.34900	-12.25700	36.04900
H	9.19700	-12.48600	35.13200
H	9.33900	-11.30000	36.06200
O	6.11700	-14.68400	31.03300
H	7.04700	-14.53200	30.86000
H	5.69700	-14.59500	30.17800
O	4.91600	-10.65900	28.80300
H	4.77700	-11.02700	29.67600
H	4.12800	-10.14400	28.63100
O	5.51100	-5.55800	20.14600
H	5.68600	-6.02900	20.96000
H	4.82400	-4.93100	20.37400
O	6.46700	-11.28000	26.19200
H	6.13200	-11.32000	27.08800
H	5.96700	-11.94600	25.71900
O	0.44100	-15.87400	31.71700
H	0.30300	-16.60600	32.31800
H	1.35200	-15.96300	31.43600
O	9.40300	-13.93500	16.46600
H	8.78600	-14.66000	16.36900
H	8.93400	-13.17400	16.12200
O	3.06900	-16.16200	31.20600
H	3.77900	-16.80200	31.16400
H	3.02600	-15.91000	32.12800
O	11.05800	-9.22500	34.12600
H	10.99900	-9.90900	33.45900
H	11.46000	-8.48300	33.67400
O	1.78200	-19.17500	33.02500
H	1.24600	-19.62600	33.67700
H	1.19400	-19.04000	32.28200
O	0.07700	-2.40900	26.92000
H	0.31700	-1.77700	27.59700
H	-0.76600	-2.76000	27.20600
O	1.19300	-19.71600	15.58800
H	0.31600	-19.83700	15.22400
H	1.66800	-20.50800	15.33600
O	11.06000	-11.26100	32.35800
H	10.17300	-11.59800	32.48000
H	11.63000	-11.99300	32.59400
O	-7.28700	-14.92800	25.68200
H	-6.60000	-15.24200	25.09400
H	-7.44200	-14.02400	25.40900
O	-2.48400	-20.03900	30.11000
H	-2.56800	-20.64700	29.37500
H	-3.30500	-19.54900	30.11100
O	4.59000	-6.60100	29.32400
H	4.51700	-5.79500	29.83600
H	4.14800	-6.40200	28.49900
O	4.37900	-7.36400	22.83400
H	3.59600	-6.82100	22.92800
H	5.07800	-6.84600	23.23300
O	7.68900	-21.55400	25.47700
H	8.18600	-22.30700	25.15800
H	6.91700	-21.51800	24.91200
O	15.54200	-15.85000	27.11900
H	15.53100	-14.96800	26.74900
H	14.67300	-16.20300	26.92500
O	7.05000	-6.70200	31.48000
H	7.76300	-6.06700	31.40700
H	6.55900	-6.60600	30.66400
O	8.77200	-12.79700	23.05100
H	7.83700	-12.67700	22.88200
H	8.94900	-13.70000	22.78700
O	4.27200	-19.41200	25.20500
H	3.90200	-19.96500	25.89300
H	4.76800	-20.01700	24.65200
O	12.15900	-16.95500	23.58600
H	12.62500	-16.12200	23.51200
H	12.61000	-17.42100	24.29100
O	2.10100	-8.95300	32.30000
H	2.80200	-8.31900	32.45300
H	1.63100	-8.61100	31.54000
O	10.22700	-10.61100	20.58500
H	10.50200	-10.72500	21.49400
H	9.35700	-11.00900	20.54600
O	10.13100	-24.58700	27.62500
H	9.21700	-24.35700	27.79400
H	10.56000	-24.50300	28.47700
O	9.90100	-16.39800	30.12500
H	9.24800	-16.15100	29.47100
H	10.27900	-17.21200	29.79200
O	6.86200	-6.36200	24.22800
H	7.04400	-7.18000	24.69100
H	7.68500	-6.14500	23.79100
O	7.19600	-7.43500	21.49000
H	7.72500	-7.94600	20.87700
H	7.45900	-7.75100	22.35400
O	5.95000	-15.65600	36.61200
H	5.27400	-15.75700	37.28200
H	6.77300	-15.81200	37.07600
O	-2.30200	-9.41900	29.23300
H	-1.71700	-10.16500	29.36600
H	-2.50000	-9.10900	30.11700
O	0.55200	-6.38400	24.24900
H	-0.11700	-5.89900	23.76600
H	0.41300	-6.13700	25.16400
O	-7.71000	-16.84800	27.95400
H	-7.67200	-17.38200	28.74700
H	-8.21600	-17.37500	27.33500
O	4.69200	-4.34900	30.84800
H	5.24400	-4.18600	31.61300
H	5.03300	-3.75900	30.17600

O	-0.33100	-22.00200	31.45000
H	-0.26700	-21.14000	31.03800
H	-0.39500	-22.61500	30.71700
O	-0.85600	-19.93000	35.09700
H	-0.22900	-20.31200	35.71100
H	-1.71300	-20.14800	35.46300
O	9.54300	-9.52300	36.70300
H	10.08700	-8.91000	36.20800
H	8.96200	-8.96500	37.21900
O	-4.08000	-18.12200	21.02900
H	-3.78400	-19.03100	20.97600
H	-4.98100	-18.14300	20.70700
O	7.82900	-6.40100	27.83400
H	7.70400	-7.20200	27.32500
H	8.48700	-5.90700	27.34500
O	-0.55000	-20.04600	27.01200
H	-1.27100	-19.43300	26.87300
H	0.23900	-19.51100	26.93300
O	9.89300	-8.60000	29.46200
H	8.94600	-8.72000	29.39000
H	10.24100	-9.48800	29.54500
O	2.25300	-22.71100	33.28600
H	2.77600	-22.46500	34.04900
H	2.03400	-21.87900	32.86600
O	-2.35100	-20.44100	21.15300
H	-2.67900	-21.32100	20.97000
H	-1.99700	-20.49600	22.04100
O	6.89700	-8.80500	25.66200
H	6.75000	-9.73300	25.84300
H	7.72300	-8.78300	25.17800
O	0.76300	-20.15300	21.52100
H	1.10300	-19.62500	20.79800
H	1.21100	-19.81500	22.29600
O	10.50100	-7.98200	26.74900
H	11.42500	-8.18800	26.61100
H	10.33200	-8.23100	27.65800
O	3.45800	-21.77000	30.97100
H	3.11700	-22.56900	31.37300
H	2.75200	-21.46900	30.39900
O	4.10900	-24.68500	29.42400
H	4.93200	-25.15900	29.30100
H	4.35300	-23.90600	29.92300
O	2.75000	-21.65600	19.72000
H	3.47100	-21.27800	19.21600
H	2.12700	-20.93700	19.82300
O	6.89300	-20.95100	20.65600
H	7.11000	-20.06900	20.35500
H	6.41300	-21.34300	19.92600
O	1.63300	-17.99300	27.23300
H	1.48800	-17.67900	28.12500
H	1.15600	-17.37400	26.68000
O	3.49300	-21.00300	27.28100
H	3.47900	-21.89800	26.94400
H	4.21000	-20.99500	27.91500
O	5.34600	-20.56900	29.32600
H	5.08700	-19.64700	29.32500
H	4.81400	-20.96600	30.01600
O	10.29000	-7.46400	22.10500
H	10.46400	-8.02900	21.35300
H	10.78300	-6.66400	21.92300
O	-3.89200	-15.45200	30.17200
H	-4.43700	-14.67600	30.30400
H	-3.01400	-15.10500	30.01300
O	2.89700	-22.12100	22.55000
H	3.01800	-23.04900	22.74900
H	2.60700	-22.10700	21.63700
O	5.76600	-24.43600	25.68600
H	5.78800	-24.37200	24.73100
H	6.18300	-25.27500	25.88000
O	16.14400	-14.70000	23.72400
H	16.58700	-15.44900	23.32600
H	15.26700	-14.70900	23.34200
O	11.27800	-8.14700	19.20500
H	11.97300	-7.52600	19.42400
H	11.62000	-8.99700	19.48000
O	-3.10600	-16.32500	33.06500
H	-3.84700	-16.17700	33.65100
H	-3.42600	-16.06400	32.20100
O	13.17700	-10.69700	20.73400
H	13.70200	-10.35800	20.00900
H	13.08900	-11.63200	20.55000
O	4.83900	-6.78400	32.86300
H	5.14800	-7.02900	33.73500
H	5.63600	-6.57900	32.37400
O	4.86100	-4.63000	25.09300
H	4.42100	-5.12200	25.78600
H	5.61200	-5.17300	24.85200
O	-2.66000	-8.43900	25.09600
H	-1.83400	-8.73800	24.71500
H	-3.01000	-9.20800	25.54500
O	2.30800	-9.08600	27.16500
H	1.68600	-9.74900	26.86600
H	2.97900	-9.06000	26.48300
O	-4.45900	-10.52000	26.05400
H	-5.29600	-10.94800	25.87300
H	-4.48700	-10.32700	26.99200
O	2.38100	-20.60800	36.75900
H	2.43000	-19.73200	36.37600
H	2.92300	-21.15000	36.18600
O	12.86600	-10.06400	36.03900
H	12.07600	-10.08100	35.49900
H	12.63000	-9.52500	36.79400
O	12.98600	-13.21500	33.35200
H	13.03600	-13.38000	34.29400
H	12.87700	-14.08200	32.96200
O	1.87000	-10.53600	20.64100
H	2.01800	-9.85200	19.98800
H	2.67600	-10.55500	21.15600
O	10.27600	-17.23100	34.66800
H	11.17700	-17.37100	34.37600
H	10.35100	-17.06600	35.60800
O	4.61100	-19.85500	33.51300
H	3.74100	-19.56100	33.24200
H	4.84700	-19.27100	34.23400
O	14.48800	-10.79500	33.59600
H	14.19600	-10.43200	34.43300
H	13.87500	-11.50800	33.41800
O	15.68700	-13.23000	26.11600

H	16.52200	-12.79100	26.28000
H	15.82300	-13.70600	25.29700
O	12.73700	-15.67200	32.06400
H	13.21200	-15.47900	31.25600
H	11.81300	-15.61600	31.81900
O	-0.37500	-14.50900	21.64700
H	-0.62100	-15.41300	21.84500
H	-0.20600	-14.50700	20.70500
O	7.31500	-23.63700	31.03000
H	7.83300	-22.90100	30.70400
H	6.96000	-23.33000	31.86400
O	-1.10600	-18.77600	19.14600
H	-1.79900	-18.70200	18.49000
H	-1.35000	-19.54000	19.66800
O	7.56600	-27.21700	26.55000
H	7.84600	-27.88400	27.17600
H	8.35100	-27.02400	26.03700
O	5.36900	-8.04100	18.11200
H	4.79700	-7.42200	17.65900
H	5.94200	-7.49100	18.64600
O	11.43700	-18.79500	30.29700
H	12.38800	-18.69500	30.31800
H	11.26700	-19.58700	30.80700
O	9.45800	-21.70900	30.48300
H	8.86300	-21.42900	29.78800
H	9.17300	-21.22000	31.25500
O	8.58900	-7.75700	19.24300
H	9.52200	-7.82300	19.44700
H	8.50100	-6.92100	18.78500
O	11.21800	-8.44100	16.20400
H	11.13900	-8.77500	17.09700
H	12.03900	-7.94800	16.20300
O	13.91500	-15.57400	29.41100
H	13.88300	-16.51300	29.59700
H	14.52600	-15.49600	28.67800
O	5.75400	-16.70500	15.45400
H	6.70000	-16.83400	15.52300
H	5.37500	-17.44300	15.93100
O	14.06500	-18.78300	29.45700
H	13.54900	-19.56700	29.27000
H	14.94900	-19.11100	29.62200
O	11.72600	-25.03900	25.41700
H	11.52800	-25.34800	26.30100
H	12.33600	-24.31300	25.54600
O	19.73300	-14.51300	28.55200
H	20.46300	-14.77400	29.11300
H	19.12400	-15.25000	28.59200
O	17.81500	-16.37200	29.14900
H	17.08800	-16.50600	28.54100
H	17.39900	-16.06400	29.95500
O	5.63800	-12.65300	36.91400
H	6.55800	-12.47500	37.10800
H	5.60300	-13.59700	36.76200
O	3.93800	-18.78000	15.38900
H	3.03100	-19.07600	15.46600
H	4.21600	-19.07700	14.52300
O	-5.14400	-17.45900	25.83900
H	-5.46400	-18.31300	25.54700
H	-5.69100	-17.24400	26.59500
O	-1.36600	-14.74300	29.55600
H	-1.14700	-13.81100	29.57500
H	-0.95400	-15.10200	30.34200
O	5.55400	-24.23600	21.54400
H	4.74400	-24.58100	21.91900
H	6.24300	-24.54500	22.13300
O	7.48300	-25.15800	23.16600
H	8.03600	-24.73900	23.82500
H	7.72000	-26.08500	23.20900
O	3.50700	-19.17000	38.94700
H	3.40600	-18.22000	38.87800
H	3.28900	-19.49800	38.07400
O	2.70800	-21.92100	15.53100
H	3.57000	-21.59600	15.79000
H	2.89300	-22.70800	15.01800
O	-5.27500	-13.83900	27.80900
H	-5.96900	-14.25300	27.29600
H	-4.52800	-13.79700	27.21200
O	10.86100	-23.04800	22.67400
H	10.28100	-22.39000	22.29200
H	10.59500	-23.87000	22.26300
O	9.31200	-23.64800	24.87600
H	9.81200	-23.53600	24.06800
H	9.95500	-23.95200	25.51600
O	3.37000	-15.74000	37.76300
H	2.88700	-14.94000	37.97000
H	3.45600	-15.72900	36.81000
O	-3.36200	-13.90700	25.68500
H	-3.38500	-13.80800	24.73300
H	-3.05200	-13.06000	26.00700
O	-0.70300	-11.84900	29.80000
H	0.25100	-11.88100	29.73300
H	-0.90200	-12.31000	30.61500
O	-0.11300	-13.27900	32.36700
H	0.70600	-12.78500	32.39100
H	0.16100	-14.19600	32.33900
O	9.64400	-20.88800	21.73000
H	8.75600	-20.60300	21.51200
H	10.05900	-21.04900	20.88300
O	12.99500	-20.86400	25.44500
H	13.77100	-20.37400	25.71800
H	12.27000	-20.43800	25.90200
O	15.43200	-20.15100	26.50300
H	16.02700	-20.39100	25.79200
H	15.50500	-20.87000	27.13000
O	-2.34800	-6.30700	26.81700
H	-2.48200	-7.06700	26.25100
H	-2.76500	-5.58300	26.35000
O	8.78800	-11.12400	15.82500
H	9.49200	-10.91900	16.44000
H	8.06400	-10.55600	16.09000
O	9.61800	-15.37300	21.94500
H	9.56100	-14.91600	21.10600
H	10.17100	-16.13400	21.76700
O	13.42800	-19.58400	22.77000
H	13.17200	-19.98400	23.60100
H	12.80400	-18.86900	22.64500
O	17.74000	-16.84400	25.27700
H	17.18000	-16.77500	26.05000

H	17.13400	-17.02000	24.55700
O	0.80900	-5.41000	27.02900
H	0.69200	-4.46600	26.92700
H	-0.01200	-5.71300	27.41700
O	10.69700	-10.68500	17.87400
H	11.53100	-11.11700	17.69200
H	10.53500	-10.85700	18.80200
O	10.35100	-14.18200	19.24000
H	10.29600	-14.34800	18.29900
H	9.72600	-13.47500	19.39600
O	-4.85900	-18.94500	33.06600
H	-4.21400	-19.45600	33.55500
H	-5.06800	-18.20900	33.64100
O	7.66600	-11.87700	38.56500
H	8.53500	-11.70300	38.20400
H	7.18700	-11.05800	38.44300
O	3.66100	-24.68100	18.81900
H	3.64900	-23.73700	18.97500
H	4.55300	-24.86400	18.52500
O	1.83500	-4.75800	30.24400
H	2.73700	-4.72500	30.56100
H	1.60800	-5.68800	30.25900
O	3.59000	-6.00500	26.90500
H	2.63900	-6.00400	26.79200
H	3.89300	-6.75100	26.38900
O	7.47600	-28.08700	23.07300
H	6.70900	-28.64100	23.21900
H	7.88700	-28.01500	23.93500
O	-0.52000	-21.79900	37.16400
H	0.34300	-22.15500	37.37400
H	-1.09300	-22.13700	37.85100
O	19.32500	-0.16900	27.97000
H	19.96300	-0.28800	27.26600
H	19.20700	0.78000	28.02900
O	16.13400	-0.21600	27.90600
H	15.79600	-0.33200	27.01800
H	17.00300	-0.61700	27.88600
O	11.99300	1.63800	23.89600
H	11.42900	1.54700	23.12700
H	11.87800	0.81800	24.37600
O	16.61600	0.93800	33.86500
H	16.60500	0.54300	32.99300
H	16.77800	0.20400	34.45800
O	9.53900	3.13300	26.73400
H	10.33100	2.79200	26.31900
H	9.77900	3.24200	27.65400
O	17.57300	5.30500	19.20700
H	16.92200	5.89000	18.82100
H	18.36700	5.83500	19.26700
O	11.49800	2.39400	19.60600
H	12.27100	1.84700	19.46700
H	11.85100	3.25800	19.81700
O	13.33700	3.72100	32.81300
H	13.04600	4.62600	32.69700
H	13.97300	3.76300	33.52700
O	14.26200	1.09900	29.40300
H	14.85900	0.87500	28.68900
H	14.32000	0.35800	30.00500
O	13.83500	-1.44700	17.23800
H	14.08800	-1.96900	16.47600
H	12.89700	-1.61000	17.34100
O	18.87900	-2.44300	25.04500
H	19.66600	-2.86100	24.69400
H	19.14300	-1.53900	25.21300
O	19.05000	2.99400	19.74400
H	18.41500	3.06700	20.45600
H	18.72000	3.58300	19.06600
O	24.28000	-2.50600	25.94900
H	25.01300	-3.07600	26.18200
H	23.52400	-2.89400	26.38900
O	13.55500	0.45600	19.44000
H	13.43800	-0.22000	20.10800
H	13.66500	-0.03100	18.62300
O	15.89800	-0.08300	23.14500
H	15.53400	-0.61000	23.85600
H	16.75400	-0.47300	22.97200
O	21.80300	-8.79400	30.40800
H	21.47100	-7.92400	30.18400
H	21.20700	-9.11000	31.08700
O	15.53800	-5.05400	18.14100
H	15.70600	-4.18600	18.50800
H	15.34800	-4.89100	17.21700
O	18.29600	3.40800	29.80800
H	17.99600	4.31000	29.69900
H	17.95100	3.14100	30.66000
O	11.74700	-0.14100	25.84100
H	10.82200	-0.27300	26.04900
H	11.99600	0.63800	26.33800
O	21.69600	-4.07400	27.48700
H	20.78700	-4.14300	27.19300
H	21.64400	-3.59700	28.31600
O	15.51700	-2.59800	19.12600
H	15.39700	-2.15300	19.96400
H	14.82200	-2.25000	18.56700
O	9.10800	-0.49800	26.67600
H	9.23700	-0.24400	27.59000
H	8.42800	0.09500	26.35600
O	13.27800	10.17200	22.77500
H	13.20000	11.12300	22.85400
H	12.67400	9.82700	23.43200
O	15.77000	-2.65500	35.43100
H	14.99300	-2.09600	35.42100
H	16.40900	-2.17000	35.95200
O	23.56200	-5.87300	29.26500
H	22.91900	-5.57900	28.61900
H	23.05700	-5.98700	30.07100
O	21.21600	-2.12600	19.95400
H	22.12800	-1.89300	19.77900
H	20.70700	-1.54300	19.39100
O	14.02400	0.15300	34.99300
H	14.76600	0.69200	34.71600
H	13.31800	0.78000	35.15000
O	19.50000	2.29600	33.59000
H	19.90800	1.85400	32.84500
H	18.77800	1.72200	33.84300
O	15.85300	-2.76600	30.96300
H	16.69900	-2.34300	31.10700
H	15.79300	-3.42300	31.65700

O	19.82300	2.85600	27.34500
H	19.19400	3.02100	26.64300
H	19.43500	3.27100	28.11600
O	23.70200	3.79200	24.70000
H	23.01700	4.17200	24.14900
H	23.78600	4.40100	25.43300
O	19.00300	-5.10600	32.62100
H	18.75900	-4.35200	32.08500
H	19.27900	-4.72800	33.45600
O	20.95700	1.43800	31.29900
H	21.16800	2.08100	30.62300
H	20.91000	0.60400	30.83100
O	18.55900	-5.95000	28.20300
H	17.69800	-5.65200	28.49400
H	18.58600	-5.73300	27.27100
O	15.37500	2.59400	24.61000
H	15.83900	2.87300	23.82100
H	15.11100	1.69200	24.42700
O	12.26800	2.14100	27.54800
H	12.83800	2.57000	26.91000
H	12.85900	1.85900	28.24700
O	14.31200	-1.28400	21.41900
H	15.01500	-0.83200	21.88600
H	13.98000	-1.92600	22.04700
O	12.76000	-2.69300	35.79400
H	12.13000	-2.01100	36.02800
H	12.65400	-2.80400	34.85000
O	8.05500	-3.02600	26.72100
H	7.26500	-2.77000	26.24500
H	8.54600	-2.21100	26.82600
O	22.07100	4.31500	29.47100
H	22.89300	3.91300	29.19000
H	22.06800	4.21200	30.42300
O	10.00600	-2.66100	23.67900
H	9.20900	-2.54900	24.19800
H	9.77900	-2.32200	22.81300
O	15.83700	3.86300	31.62700
H	15.90100	4.32500	32.46300
H	15.12800	3.23300	31.75600
O	6.33900	-2.77200	29.15200
H	6.58900	-1.88800	29.41800
H	6.87500	-2.95200	28.38000
O	30.16200	0.65900	25.82000
H	29.40800	0.09000	25.66400
H	30.29700	0.62500	26.76700
O	15.55200	5.53000	21.49500
H	15.41600	6.37500	21.92400
H	16.09300	5.73200	20.73200
O	12.03800	-8.39100	38.15700
H	11.64500	-8.94400	38.83200
H	12.85500	-8.08000	38.54700
O	9.00500	3.04800	22.06800
H	9.51700	3.84200	22.21900
H	9.65700	2.35000	22.00100
O	10.81400	6.16400	24.15000
H	10.99100	7.08600	23.96200
H	11.61000	5.85000	24.58000
O	14.95500	-6.97500	35.74900
H	15.69600	-6.37400	35.66900
H	15.18700	-7.71700	35.19100
O	10.09900	-1.78900	34.02700
H	9.98500	-2.69100	34.32700
H	9.24400	-1.54300	33.67500
O	17.17900	6.15300	35.62500
H	16.73500	5.36600	35.30700
H	17.84000	6.34000	34.96000
O	11.93700	7.68200	30.15100
H	12.48800	7.55400	29.37900
H	11.09800	7.98200	29.80100
O	19.27700	6.43300	33.65300
H	19.25900	7.25400	33.16200
H	20.19900	6.30800	33.87900
O	15.73400	3.94700	34.49600
H	16.26700	3.18600	34.26600
H	15.20500	3.65500	35.23800
O	18.90100	-10.94700	30.02500
H	18.55700	-10.05700	29.94900
H	19.26900	-11.13700	29.16200
O	6.97000	1.37700	22.84400
H	7.80700	1.78600	22.62700
H	7.14700	0.43700	22.81400
O	18.86600	-0.95600	18.53200
H	18.35100	-0.18500	18.77100
H	18.59400	-1.62900	19.15600
O	16.45100	-6.11100	23.59100
H	15.59500	-6.50000	23.41200
H	16.86300	-6.03200	22.73100
O	17.98100	-8.57600	27.99000
H	17.47700	-8.61300	27.17700
H	18.27400	-7.66700	28.05000
O	14.22500	-8.49200	26.99700
H	15.09000	-8.68800	26.63700
H	14.29300	-8.72100	27.92400
O	23.83900	1.79700	20.46800
H	23.06200	1.45800	20.02300
H	24.51900	1.80300	19.79300
O	18.65500	-3.08300	20.35800
H	18.35000	-2.76900	21.20900
H	19.60800	-3.00500	20.40100
O	17.09900	3.07600	22.00800
H	16.57100	2.34100	21.69500
H	16.59800	3.85300	21.76200
O	22.07900	1.27100	27.12400
H	21.42100	1.96100	27.21200
H	22.88300	1.66000	27.46800
O	11.83100	-6.26200	24.36800
H	12.40600	-6.48500	23.63600
H	12.41800	-6.19300	25.12100
O	20.88900	-0.37500	25.17500
H	21.40200	0.25800	25.67700
H	21.53900	-0.97500	24.80900
O	12.15800	-4.23200	18.38800
H	11.32200	-3.89400	18.70900
H	12.01900	-4.36700	17.45100
O	14.13300	-9.38800	23.09600
H	13.48000	-9.70300	22.47200
H	14.09100	-10.01000	23.82200
O	15.24500	0.89400	15.01100

H	14.33700	0.66300	15.21000
H	15.74200	0.09800	15.19900
O	18.15100	9.60400	17.93100
H	17.47800	9.90700	18.53900
H	18.53800	10.40700	17.58100
O	17.36700	-2.90000	27.42100
H	16.44900	-2.67800	27.26300
H	17.78300	-2.82500	26.56200
O	18.15100	-1.50100	22.55100
H	18.89800	-0.98900	22.24200
H	18.42000	-1.82200	23.41100
O	16.42300	0.99500	19.53400
H	15.51500	0.71100	19.63100
H	16.45300	1.41500	18.67400
O	21.65500	4.60600	23.30300
H	21.20200	3.76500	23.35900
H	21.02500	5.24200	23.64000
O	20.59900	-4.46500	24.53300
H	19.86100	-4.97700	24.86400
H	20.85100	-4.90500	23.72100
O	20.09900	6.44300	24.23800
H	20.39100	7.00400	23.51900
H	20.60800	6.73600	24.99300
O	20.70000	-11.68700	25.12700
H	21.00600	-12.54800	24.84000
H	21.42500	-11.09600	24.92700
O	6.99200	-1.28400	23.51500
H	6.79500	-1.87700	22.79000
H	6.43700	-1.58900	24.23300
O	14.97200	-1.39200	25.63500
H	15.02700	-2.34500	25.55900
H	14.16100	-1.23600	26.11900
O	16.12100	2.53600	16.87200
H	15.68300	1.82000	16.41300
H	16.86200	2.76400	16.31100
O	12.91100	-2.77400	23.39400
H	12.09800	-2.47500	23.80000
H	13.40700	-3.16900	24.11100
O	15.06200	7.22100	25.32700
H	15.02000	7.44800	24.39800
H	15.97800	6.98800	25.47600
O	18.50900	-2.31000	32.22500
H	18.86300	-2.08300	33.08500
H	19.15600	-1.97800	31.60300
O	24.23800	0.81200	22.93000
H	24.10000	1.00200	22.00200
H	23.88200	1.57400	23.38700
O	12.40300	-5.20200	29.01000
H	13.04200	-5.34400	28.31200
H	11.57000	-5.48900	28.63400
O	24.26500	2.87900	27.94100
H	24.69900	2.78700	28.78900
H	24.97800	2.85500	27.30300
O	13.17900	4.09800	25.12300
H	13.97300	3.60300	24.92200
H	12.46800	3.57000	24.76000
O	17.99800	6.48000	29.47400
H	18.05200	7.04900	30.24200
H	17.13000	6.65000	29.11000
O	9.48700	-1.75800	21.19900
H	8.55700	-1.81900	21.41600
H	9.58300	-2.28000	20.40300
O	9.40500	-5.23200	23.78700
H	9.66600	-4.31800	23.67800
H	10.22700	-5.70000	23.93700
O	10.87800	-3.81900	30.95700
H	11.55000	-4.02700	30.30800
H	10.05100	-4.01600	30.51700
O	25.12700	2.92200	30.47400
H	25.32000	3.82600	30.72000
H	25.02700	2.46200	31.30800
O	16.78000	-7.19200	32.97400
H	16.13300	-6.49300	32.88000
H	17.55100	-6.75900	33.34100
O	20.18800	3.07200	36.04900
H	20.03700	2.84300	35.13200
H	20.17900	4.02900	36.06200
O	16.95700	0.64600	31.03300
H	17.88600	0.79700	30.86000
H	16.53600	0.73400	30.17800
O	15.75600	4.67100	28.80300
H	15.61700	4.30300	29.67600
H	14.96700	5.18500	28.63100
O	16.35000	9.77200	20.14600
H	16.52600	9.30000	20.96000
H	15.66400	10.39800	20.37400
O	17.30600	4.04900	26.19200
H	16.97100	4.00900	27.08800
H	16.80700	3.38300	25.71900
O	11.28100	-0.54400	31.71700
H	11.14300	-1.27600	32.31800
H	12.19100	-0.63300	31.43600
O	20.24200	1.39400	16.46600
H	19.62500	0.66900	16.36900
H	19.77400	2.15500	16.12200
O	13.90900	-0.83200	31.20600
H	14.61900	-1.47300	31.16400
H	13.86600	-0.58100	32.12800
O	21.89800	6.10400	34.12600
H	21.83900	5.42000	33.45900
H	22.29900	6.84600	33.67400
O	12.62200	-3.84600	33.02500
H	12.08500	-4.29700	33.67700
H	12.03300	-3.71100	32.28200
O	10.91700	12.92100	26.92000
H	11.15600	13.55200	27.59700
H	10.07400	12.56900	27.20600
O	12.03200	-4.38700	15.58800
H	11.15500	-4.50800	15.22400
H	12.50800	-5.17800	15.33600
O	21.89900	4.06800	32.35800
H	21.01200	3.73100	32.48000
H	22.46900	3.33700	32.59400
O	8.35600	-4.71000	30.11000
H	8.27100	-5.31800	29.37500
H	7.53400	-4.21900	30.11100
O	15.42900	8.72800	29.32400
H	15.35600	9.53400	29.83600

H	14.98700	8.92800	28.49900
O	15.21800	7.96600	22.83400
H	14.43500	8.50800	22.92800
H	15.91800	8.48300	23.23300
O	18.52800	-6.22500	25.47700
H	19.02500	-6.97800	25.15800
H	17.75700	-6.18800	24.91200
O	26.38100	-0.52100	27.11900
H	26.37000	0.36200	26.74900
H	25.51300	-0.87400	26.92500
O	17.88900	8.62700	31.48000
H	18.60200	9.26200	31.40700
H	17.39800	8.72400	30.66400
O	19.61100	2.53200	23.05100
H	18.67700	2.65200	22.88200
H	19.78900	1.63000	22.78700
O	15.11200	-4.08300	25.20500
H	14.74100	-4.63600	25.89300
H	15.60700	-4.68700	24.65200
O	22.99800	-1.62600	23.58600
H	23.46400	-0.79300	23.51200
H	23.44900	-2.09200	24.29100
O	12.94000	6.37700	32.30000
H	13.64100	7.01000	32.45300
H	12.47000	6.71800	31.54000
O	21.06600	4.71800	20.58500
H	21.34200	4.60400	21.49400
H	20.19600	4.32000	20.54600
O	20.97000	-9.25800	27.62500
H	20.05600	-9.02800	27.79400
H	21.39900	-9.17400	28.47700
O	20.74100	-1.06900	30.12500
H	20.08800	-0.82100	29.47100
H	21.11900	-1.88300	29.79200
O	17.70100	8.96700	24.22800
H	17.88400	8.14900	24.69100
H	18.52500	9.18400	23.79100
O	18.03600	7.89400	21.49000
H	18.56400	7.38300	20.87700
H	18.29800	7.57800	22.35400
O	16.78900	-0.32700	36.61200
H	16.11300	-0.42800	37.28200
H	17.61200	-0.48300	37.07600
O	8.53700	5.91000	29.23300
H	9.12300	5.16500	29.36600
H	8.34000	6.22100	30.11700
O	11.39100	8.94500	24.24900
H	10.72200	9.43000	23.76600
H	11.25200	9.19200	25.16400
O	3.12900	-1.51900	27.95400
H	3.16700	-2.05300	28.74700
H	2.62300	-2.04600	27.33500
O	15.53200	10.98000	30.84800
H	16.08300	11.14300	31.61300
H	15.87200	11.57000	30.17600
O	10.50800	-6.67300	31.45000
H	10.57200	-5.81100	31.03800
H	10.44500	-7.28500	30.71700
O	9.98300	-4.60000	35.09700
H	10.61000	-4.98300	35.71100
H	9.12600	-4.81900	35.46300
O	20.38300	5.80600	36.70300
H	20.92700	6.41900	36.20800
H	19.80100	6.36400	37.21900
O	6.76000	-2.79300	21.02900
H	7.05500	-3.70200	20.97600
H	5.85900	-2.81400	20.70700
O	18.66800	8.92800	27.83400
H	18.54400	8.12700	27.32500
H	19.32600	9.42300	27.34500
O	10.28900	-4.71700	27.01200
H	9.56800	-4.10400	26.87300
H	11.07900	-4.18100	26.93300
O	20.73200	6.72900	29.46200
H	19.78500	6.60900	29.39000
H	21.08000	5.84100	29.54500
O	13.09200	-7.38200	33.28600
H	13.61500	-7.13600	34.04900
H	12.87300	-6.55000	32.86600
O	8.48900	-5.11100	21.15300
H	8.16000	-5.99200	20.97000
H	8.84300	-5.16700	22.04100
O	17.73700	6.52400	25.66200
H	17.58900	5.59600	25.84300
H	18.56200	6.54600	25.17800
O	11.60300	-4.82400	21.52100
H	11.94200	-4.29600	20.79800
H	12.05000	-4.48600	22.29600
O	21.34000	7.34700	26.74900
H	22.26500	7.14100	26.61100
H	21.17200	7.09900	27.65800
O	14.29700	-6.44000	30.97100
H	13.95700	-7.24000	31.37300
H	13.59100	-6.14000	30.39900
O	14.94900	-9.35500	29.42400
H	15.77100	-9.82900	29.30100
H	15.19300	-8.57600	29.92300
O	13.59000	-6.32700	19.72000
H	14.31000	-5.94900	19.21600
H	12.96600	-5.60800	19.82300
O	17.73300	-5.62200	20.65600
H	17.94900	-4.74000	20.35500
H	17.25200	-6.01400	19.92600
O	12.47300	-2.66400	27.23300
H	12.32700	-2.35000	28.12500
H	11.99500	-2.04500	26.68000
O	14.33200	-5.67300	27.28100
H	14.31800	-6.56900	26.94400
H	15.05000	-5.66600	27.91500
O	16.18500	-5.24000	29.32600
H	15.92700	-4.31800	29.32500
H	15.65300	-5.63700	30.01600
O	21.13000	7.86600	22.10500
H	21.30300	7.30000	21.35300
H	21.62200	8.66600	21.92300
O	6.94700	-0.12300	30.17200
H	6.40300	0.65300	30.30400
H	7.82500	0.22400	30.01300

O	13.73600	-6.79200	22.55000
H	13.85800	-7.72000	22.74900
H	13.44600	-6.77800	21.63700
O	16.60500	-9.10600	25.68600
H	16.62700	-9.04200	24.73100
H	17.02200	-9.94600	25.88000
O	26.98300	0.62900	23.72400
H	27.42700	-0.12000	23.32600
H	26.10600	0.62000	23.34200
O	22.11700	7.18200	19.20500
H	22.81200	7.80300	19.42400
H	22.45900	6.33200	19.48000
O	7.73400	-0.99600	33.06500
H	6.99200	-0.84800	33.65100
H	7.41300	-0.73500	32.20100
O	24.01600	4.63200	20.73400
H	24.54100	4.97100	20.00900
H	23.92800	3.69700	20.55000
O	15.67800	8.54600	32.86300
H	15.98700	8.30000	33.73500
H	16.47600	8.75000	32.37400
O	15.70000	10.69900	25.09300
H	15.26100	10.20700	25.78600
H	16.45100	10.15700	24.85200
O	8.17900	6.89100	25.09600
H	9.00500	6.59100	24.71500
H	7.82900	6.12100	25.54500
O	13.14800	6.24300	27.16500
H	12.52600	5.58000	26.86600
H	13.81800	6.26900	26.48300
O	6.38000	4.80900	26.05400
H	5.54400	4.38100	25.87300
H	6.35300	5.00200	26.99200
O	13.22000	-5.27900	36.75900
H	13.26900	-4.40200	36.37600
H	13.76200	-5.82100	36.18600
O	23.70600	5.26500	36.03900
H	22.91500	5.24800	35.49900
H	23.46900	5.80400	36.79400
O	23.82500	2.11500	33.35200
H	23.87500	1.95000	34.29400
H	23.71700	1.24700	32.96200
O	21.11600	-1.90200	34.66800
H	22.01700	-2.04200	34.37600
H	21.19100	-1.73700	35.60800
O	15.45000	-4.52600	33.51300
H	14.58000	-4.23200	33.24200
H	15.68600	-3.94100	34.23400
O	25.32700	4.53400	33.59600
H	25.03600	4.89700	34.43300
H	24.71400	3.82100	33.41800
O	26.52700	2.10000	26.11600
H	27.36100	2.53900	26.28000
H	26.66300	1.62400	25.29700
O	23.57600	-0.34200	32.06400
H	24.05100	-0.15000	31.25600
H	22.65200	-0.28600	31.81900
O	10.46400	0.82000	21.64700
H	10.21900	-0.08400	21.84500
H	10.63400	0.82200	20.70500
O	18.15400	-8.30800	31.03000
H	18.67200	-7.57100	30.70400
H	17.79900	-8.00100	31.86400
O	9.73400	-3.44700	19.14600
H	9.04100	-3.37300	18.49000
H	9.48900	-4.21100	19.66800
O	18.40500	-11.88800	26.55000
H	18.68500	-12.55500	27.17600
H	19.19000	-11.69500	26.03700
O	22.27600	-3.46600	30.29700
H	23.22800	-3.36600	30.31800
H	22.10600	-4.25800	30.80700
O	20.29800	-6.38000	30.48300
H	19.70200	-6.10000	29.78800
H	20.01200	-5.89100	31.25500
O	19.42800	7.57200	19.24300
H	20.36100	7.50700	19.44700
H	19.34100	8.40800	18.78500
O	22.05700	6.88900	16.20400
H	21.97800	6.55500	17.09700
H	22.87800	7.38100	16.20300
O	24.75500	-0.24500	29.41100
H	24.72300	-1.18400	29.59700
H	25.36500	-0.16700	28.67800
O	16.59300	-1.37600	15.45400
H	17.53900	-1.50500	15.52300
H	16.21500	-2.11400	15.93100
O	24.90400	-3.45400	29.45700
H	24.38800	-4.23800	29.27000
H	25.78800	-3.78200	29.62200
O	22.56500	-9.71000	25.41700
H	22.36800	-10.01800	26.30100
H	23.17500	-8.98400	25.54600
O	30.57300	0.81600	28.55200
H	31.30300	0.55500	29.11300
H	29.96300	0.07900	28.59200
O	28.65400	-1.04300	29.14900
H	27.92700	-1.17700	28.54100
H	28.23900	-0.73500	29.95500
O	16.47700	2.67600	36.91400
H	17.39700	2.85400	37.10800
H	16.44200	1.73200	36.76200
O	14.77700	-3.45100	15.38900
H	13.87100	-3.74700	15.46600
H	15.05500	-3.74800	14.52300
O	5.69500	-2.13000	25.83900
H	5.37600	-2.98300	25.54700
H	5.14900	-1.91500	26.59500
O	9.47300	0.58600	29.55600
H	9.69200	1.51800	29.57500
H	9.88600	0.22700	30.34200
O	16.39400	-8.90700	21.54400
H	15.58400	-9.25200	21.91900
H	17.08200	-9.21600	22.13300
O	18.32300	-9.82900	23.16600
H	18.87600	-9.41000	23.82500
H	18.56000	-10.75500	23.20900
O	14.34600	-3.84000	38.94700

H	14.24600	-2.89100	38.87800
H	14.12800	-4.16900	38.07400
O	13.54700	-6.59200	15.53100
H	14.40900	-6.26600	15.79000
H	13.73200	-7.37900	15.01800
O	5.56400	1.49000	27.80900
H	4.87000	1.07600	27.29600
H	6.31100	1.53200	27.21200
O	21.70100	-7.71900	22.67400
H	21.12100	-7.06100	22.29200
H	21.43400	-8.54100	22.26300
O	20.15100	-8.31900	24.87600
H	20.65100	-8.20700	24.06800
H	20.79500	-8.62200	25.51600
O	14.20900	-0.41100	37.76300
H	13.72600	0.38900	37.97000
H	14.29500	-0.40000	36.81000
O	7.47800	1.42200	25.68500
H	7.45500	1.52100	24.73300
H	7.78800	2.26900	26.00700
O	10.13700	3.48000	29.80000
H	11.09100	3.44800	29.73300
H	9.93800	3.01900	30.61500
O	10.72600	2.05000	32.36700
H	11.54600	2.54400	32.39100
H	11.00000	1.13300	32.33900
O	20.48300	-5.55900	21.73000
H	19.59600	-5.27400	21.51200
H	20.89900	-5.72000	20.88300
O	23.83500	-5.53400	25.44500
H	24.61100	-5.04500	25.71800
H	23.10900	-5.10900	25.90200
O	26.27200	-4.82200	26.50300
H	26.86600	-5.06200	25.79200
H	26.34400	-5.54100	27.13000
O	8.49200	9.02200	26.81700
H	8.35700	8.26200	26.25100
H	8.07500	9.74600	26.35000
O	19.62700	4.20500	15.82500
H	20.33100	4.41000	16.44000
H	18.90400	4.77300	16.09000
O	20.45700	-0.04400	21.94500
H	20.40100	0.41300	21.10600
H	21.01000	-0.80400	21.76700
O	24.26800	-4.25500	22.77000
H	24.01100	-4.65500	23.60100
H	23.64300	-3.54000	22.64500
O	28.57900	-1.51500	25.27700
H	28.01900	-1.44600	26.05000
H	27.97300	-1.69100	24.55700
O	11.64800	9.91900	27.02900
H	11.53200	10.86300	26.92700
H	10.82800	9.61600	27.41700
O	21.53600	4.64400	17.87400
H	22.37100	4.21200	17.69200
H	21.37500	4.47200	18.80200
O	21.19100	1.14700	19.24000
H	21.13600	0.98100	18.29900
H	20.56500	1.85500	19.39600
O	5.98000	-3.61600	33.06600
H	6.62600	-4.12600	33.55500
H	5.77200	-2.87900	33.64100
O	18.50500	3.45200	38.56500
H	19.37400	3.62600	38.20400
H	18.02600	4.27100	38.44300
O	14.50000	-9.35200	18.81900
H	14.48800	-8.40800	18.97500
H	15.39300	-9.53500	18.52500
O	12.67400	10.57200	30.24400
H	13.57700	10.60400	30.56100
H	12.44800	9.64200	30.25900
O	14.42900	9.32500	26.90500
H	13.47900	9.32500	26.79200
H	14.73300	8.57800	26.38900
O	18.31600	-12.75800	23.07300
H	17.54900	-13.31200	23.21900
H	18.72700	-12.68600	23.93500
O	10.32000	-6.47000	37.16400
H	11.18300	-6.82600	37.37400
H	9.74600	-6.80800	37.85100
O	30.16500	15.16100	27.97000
H	30.80200	15.04100	27.26600
H	30.04700	16.10900	28.02900
O	26.97400	15.11300	27.90600
H	26.63500	14.99800	27.01800
H	27.84200	14.71300	27.88600
O	22.83200	16.96700	23.89600
H	22.26900	16.87600	23.12700
H	22.71700	16.14700	24.37600
O	20.37900	18.46200	26.73400
H	21.17100	18.12100	26.31900
H	20.61800	18.57200	27.65400
O	28.41200	20.63400	19.20700
H	27.76100	21.21900	18.82100
H	29.20700	21.16500	19.26700
O	22.33800	17.72300	19.60600
H	23.11100	17.17600	19.46700
H	22.69000	18.58700	19.81700
O	25.10200	16.42800	29.40300
H	25.69800	16.20400	28.68900
H	25.15900	15.68700	30.00500
O	24.67400	13.88200	17.23800
H	24.92700	13.36000	16.47600
H	23.73700	13.71900	17.34100
O	29.71900	12.88600	25.04500
H	30.50500	12.46800	24.69400
H	29.98300	13.79000	25.21300
O	29.89000	18.32300	19.74400
H	29.25400	18.39700	20.45600
H	29.55900	18.91200	19.06600
O	35.12000	12.82300	25.94900
H	35.85200	12.25300	26.18200
H	34.36300	12.43600	26.38900
O	24.39400	15.78500	19.44000
H	24.27800	15.11000	20.10800
H	24.50400	15.29800	18.62300
O	26.73700	15.24600	23.14500
H	26.37300	14.71900	23.85600

H	27.59400	14.85600	22.97200
O	32.64200	6.53500	30.40800
H	32.31000	7.40500	30.18400
H	32.04700	6.21900	31.08700
O	26.37800	10.27500	18.14100
H	26.54500	11.14300	18.50800
H	26.18700	10.43800	17.21700
O	22.58600	15.18800	25.84100
H	21.66200	15.05600	26.04900
H	22.83500	15.96800	26.33800
O	32.53500	11.25500	27.48700
H	31.62700	11.18700	27.19300
H	32.48400	11.73200	28.31600
O	26.35700	12.73100	19.12600
H	26.23700	13.17700	19.96400
H	25.66200	13.07900	18.56700
O	19.94700	14.83100	26.67600
H	20.07700	15.08500	27.59000
H	19.26700	15.42400	26.35600
O	24.11700	25.50100	22.77500
H	24.04000	26.45200	22.85400
H	23.51300	25.15600	23.43200
O	34.40100	9.45700	29.26500
H	33.75900	9.75000	28.61900
H	33.89700	9.34200	30.07100
O	32.05500	13.20300	19.95400
H	32.96700	13.43600	19.77900
H	31.54600	13.78700	19.39100
O	26.69200	12.56300	30.96300
H	27.53800	12.98700	31.10700
H	26.63200	11.90600	31.65700
O	30.66200	18.18600	27.34500
H	30.03300	18.35000	26.64300
H	30.27500	18.60000	28.11600
O	34.54100	19.12100	24.70000
H	33.85600	19.50100	24.14900
H	34.62500	19.73100	25.43300
O	29.84200	10.22300	32.62100
H	29.59800	10.97700	32.08500
H	30.11800	10.60200	33.45600
O	29.39900	9.37900	28.20300
H	28.53700	9.67700	28.49400
H	29.42500	9.59600	27.27100
O	26.21500	17.92300	24.61000
H	26.67900	18.20200	23.82100
H	25.95100	17.02100	24.42700
O	23.10700	17.47000	27.54800
H	23.67700	17.89900	26.91000
H	23.69800	17.18800	28.24700
O	25.15200	14.04500	21.41900
H	25.85400	14.49700	21.88600
H	24.82000	13.40400	22.04700
O	18.89400	12.30400	26.72100
H	18.10400	12.55900	26.24500
H	19.38500	13.11800	26.82600
O	20.84600	12.66800	23.67900
H	20.04900	12.78000	24.19800
H	20.61800	13.00700	22.81300
O	17.17800	12.55700	29.15200
H	17.42900	13.44100	29.41800
H	17.71400	12.37700	28.38000
O	26.39100	20.85900	21.49500
H	26.25600	21.70500	21.92400
H	26.93200	21.06100	20.73200
O	22.87800	6.93900	38.15700
H	22.48500	6.38500	38.83200
H	23.69400	7.25000	38.54700
O	19.84400	18.37700	22.06800
H	20.35700	19.17100	22.21900
H	20.49600	17.68000	22.00100
O	21.65400	21.49400	24.15000
H	21.83000	22.41500	23.96200
H	22.44900	21.17900	24.58000
O	25.79500	8.35400	35.74900
H	26.53600	8.95500	35.66900
H	26.02600	7.61200	35.19100
O	20.93900	13.54000	34.02700
H	20.82400	12.63900	34.32700
H	20.08300	13.78600	33.67500
O	29.74000	4.38200	30.02500
H	29.39700	5.27300	29.94900
H	30.10800	4.19300	29.16200
O	17.80900	16.70600	22.84400
H	18.64600	17.11600	22.62700
H	17.98700	15.76600	22.81400
O	29.70500	14.37300	18.53200
H	29.19000	15.14400	18.77100
H	29.43300	13.70000	19.15600
O	27.29000	9.21800	23.59100
H	26.43400	8.82900	23.41200
H	27.70300	9.29700	22.73100
O	28.82000	6.75300	27.99000
H	28.31700	6.71600	27.17700
H	29.11300	7.66200	28.05000
O	25.06400	6.83700	26.99700
H	25.93000	6.64100	26.63700
H	25.13200	6.60800	27.92400
O	34.67900	17.12700	20.46800
H	33.90200	16.78800	20.02300
H	35.35800	17.13200	19.79300
O	29.49400	12.24600	20.35800
H	29.18900	12.56100	21.20900
H	30.44700	12.32400	20.40100
O	27.93900	18.40500	22.00800
H	27.41100	17.67000	21.69500
H	27.43700	19.18300	21.76200
O	32.91800	16.60000	27.12400
H	32.26000	17.29000	27.21200
H	33.72200	16.99000	27.46800
O	22.67100	9.06700	24.36800
H	23.24500	8.84500	23.63600
H	23.25800	9.13600	25.12100
O	31.72900	14.95400	25.17500
H	32.24200	15.58700	25.67700
H	32.37900	14.35500	24.80900
O	22.99800	11.09700	18.38800
H	22.16100	11.43500	18.70900
H	22.85800	10.96200	17.45100

O	24.97300	5.94100	23.09600
H	24.31900	5.62600	22.47200
H	24.93100	5.31900	23.82200
O	26.08400	16.22300	15.01100
H	25.17700	15.99200	15.21000
H	26.58100	15.42700	15.19900
O	28.99100	24.93300	17.93100
H	28.31700	25.23600	18.53900
H	29.37700	25.73600	17.58100
O	28.20700	12.42900	27.42100
H	27.28900	12.65100	27.26300
H	28.62300	12.50400	26.56200
O	28.99000	13.82800	22.55100
H	29.73700	14.34100	22.24200
H	29.25900	13.50700	23.41100
O	27.26300	16.32400	19.53400
H	26.35400	16.04000	19.63100
H	27.29200	16.74400	18.67400
O	32.49500	19.93500	23.30300
H	32.04100	19.09400	23.35900
H	31.86400	20.57100	23.64000
O	31.43800	10.86400	24.53300
H	30.70000	10.35200	24.86400
H	31.69100	10.42400	23.72100
O	30.93800	21.77300	24.23800
H	31.23000	22.33300	23.51900
H	31.44800	22.06500	24.99300
O	31.53900	3.64200	25.12700
H	31.84500	2.78200	24.84000
H	32.26400	4.23400	24.92700
O	17.83200	14.04500	23.51500
H	17.63400	13.45200	22.79000
H	17.27600	13.74000	24.23300
O	25.81100	13.93700	25.63500
H	25.86600	12.98500	25.55900
H	25.00000	14.09300	26.11900
O	26.96000	17.86500	16.87200
H	26.52200	17.14900	16.41300
H	27.70100	18.09300	16.31100
O	23.75000	12.55500	23.39400
H	22.93700	12.85400	23.80000
H	24.24600	12.16000	24.11100
O	25.90100	22.55100	25.32700
H	25.85900	22.77700	24.39800
H	26.81800	22.31700	25.47600
O	35.07800	16.14100	22.93000
H	34.93900	16.33100	22.00200
H	34.72100	16.90300	23.38700
O	23.24200	10.12700	29.01000
H	23.88200	9.98600	28.31200
H	22.41000	9.84000	28.63400
O	24.01800	19.42700	25.12300
H	24.81200	18.93200	24.92200
H	23.30700	18.89900	24.76000
O	20.32600	13.57100	21.19900
H	19.39600	13.51000	21.41600
H	20.42200	13.04900	20.40300
O	20.24500	10.09700	23.78700
H	20.50500	11.01100	23.67800
H	21.06600	9.62900	23.93700
O	21.71700	11.51100	30.95700
H	22.39000	11.30200	30.30800
H	20.89000	11.31300	30.51700
O	27.62000	8.13700	32.97400
H	26.97300	8.83600	32.88000
H	28.39000	8.57000	33.34100
O	27.79600	15.97500	31.03300
H	28.72500	16.12600	30.86000
H	27.37500	16.06300	30.17800
O	26.59500	20.00000	28.80300
H	26.45600	19.63200	29.67600
H	25.80700	20.51500	28.63100
O	27.19000	25.10100	20.14600
H	27.36500	24.63000	20.96000
H	26.50300	25.72700	20.37400
O	28.14600	19.37800	26.19200
H	27.81100	19.33800	27.08800
H	27.64600	18.71300	25.71900
O	22.12000	14.78500	31.71700
H	21.98200	14.05300	32.31800
H	23.03000	14.69600	31.43600
O	31.08100	16.72400	16.46600
H	30.46400	15.99800	16.36900
H	30.61300	17.48500	16.12200
O	24.74800	14.49700	31.20600
H	25.45800	13.85600	31.16400
H	24.70500	14.74800	32.12800
O	23.46100	11.48300	33.02500
H	22.92500	11.03200	33.67700
H	22.87300	11.61800	32.28200
O	22.87100	10.94200	15.58800
H	21.99400	10.82100	15.22400
H	23.34700	10.15100	15.33600
O	14.39200	15.73000	25.68200
H	15.07900	15.41600	25.09400
H	14.23600	16.63400	25.40900
O	19.19500	10.61900	30.11000
H	19.11100	10.01100	29.37500
H	18.37300	11.11000	30.11100
O	26.05800	23.29500	22.83400
H	25.27400	23.83700	22.92800
H	26.75700	23.81200	23.23300
O	29.36700	9.10400	25.47700
H	29.86400	8.35100	25.15800
H	28.59600	9.14100	24.91200
O	37.22000	14.80800	27.11900
H	37.20900	15.69100	26.74900
H	36.35200	14.45500	26.92500
O	30.45000	17.86100	23.05100
H	29.51600	17.98200	22.88200
H	30.62800	16.95900	22.78700
O	25.95100	11.24600	25.20500
H	25.58100	10.69300	25.89300
H	26.44700	10.64200	24.65200
O	33.83800	13.70300	23.58600
H	34.30400	14.53600	23.51200
H	34.28800	13.23800	24.29100
O	31.90500	20.04800	20.58500

H	32.18100	19.93300	21.49400
H	31.03600	19.64900	20.54600
O	31.80900	6.07100	27.62500
H	30.89600	6.30200	27.79400
H	32.23900	6.15500	28.47700
O	31.58000	14.26000	30.12500
H	30.92700	14.50800	29.47100
H	31.95800	13.44700	29.79200
O	28.54100	24.29600	24.22800
H	28.72300	23.47800	24.69100
H	29.36400	24.51300	23.79100
O	28.87500	23.22300	21.49000
H	29.40400	22.71200	20.87700
H	29.13800	22.90700	22.35400
O	19.37700	21.23900	29.23300
H	19.96200	20.49400	29.36600
H	19.17900	21.55000	30.11700
O	22.23000	24.27500	24.24900
H	21.56100	24.75900	23.76600
H	22.09200	24.52100	25.16400
O	13.96800	13.81000	27.95400
H	14.00700	13.27600	28.74700
H	13.46200	13.28300	27.33500
O	21.34700	8.65600	31.45000
H	21.41200	9.51800	31.03800
H	21.28400	8.04400	30.71700
O	20.82300	10.72900	35.09700
H	21.45000	10.34600	35.71100
H	19.96500	10.51000	35.46300
O	17.59900	12.53600	21.02900
H	17.89500	11.62700	20.97600
H	16.69800	12.51500	20.70700
O	21.12900	10.61200	27.01200
H	20.40700	11.22600	26.87300
H	21.91800	11.14800	26.93300
O	23.93100	7.94700	33.28600
H	24.45500	8.19400	34.04900
H	23.71200	8.77900	32.86600
O	19.32800	10.21800	21.15300
H	18.99900	9.33800	20.97000
H	19.68200	10.16300	22.04100
O	28.57600	21.85300	25.66200
H	28.42800	20.92500	25.84300
H	29.40100	21.87500	25.17800
O	22.44200	10.50500	21.52100
H	22.78200	11.03400	20.79800
H	22.88900	10.84300	22.29600
O	25.13700	8.88900	30.97100
H	24.79600	8.09000	31.37300
H	24.43000	9.18900	30.39900
O	25.78800	5.97400	29.42400
H	26.61100	5.50000	29.30100
H	26.03200	6.75300	29.92300
O	24.42900	9.00300	19.72000
H	25.15000	9.38100	19.21600
H	23.80600	9.72100	19.82300
O	28.57200	9.70700	20.65600
H	28.78800	10.59000	20.35500
H	28.09200	9.31600	19.92600
O	23.31200	12.66600	27.23300
H	23.16600	12.98000	28.12500
H	22.83500	13.28400	26.68000
O	25.17200	9.65600	27.28100
H	25.15800	8.76000	26.94400
H	25.88900	9.66300	27.91500
O	27.02400	10.09000	29.32600
H	26.76600	11.01100	29.32500
H	26.49300	9.69300	30.01600
O	31.96900	23.19500	22.10500
H	32.14300	22.62900	21.35300
H	32.46200	23.99500	21.92300
O	17.78700	15.20600	30.17200
H	17.24200	15.98200	30.30400
H	18.66500	15.55300	30.01300
O	24.57500	8.53700	22.55000
H	24.69700	7.60900	22.74900
H	24.28600	8.55100	21.63700
O	27.44400	6.22300	25.68600
H	27.46700	6.28700	24.73100
H	27.86100	5.38300	25.88000
O	37.82300	15.95800	23.72400
H	38.26600	15.20900	23.32600
H	36.94500	15.94900	23.34200
O	32.95600	22.51100	19.20500
H	33.65200	23.13200	19.42400
H	33.29900	21.66100	19.48000
O	18.57300	14.33300	33.06500
H	17.83100	14.48100	33.65100
H	18.25200	14.59400	32.20100
O	34.85600	19.96100	20.73400
H	35.38100	20.30000	20.00900
H	34.76800	19.02600	20.55000
O	26.54000	26.02900	25.09300
H	26.10000	25.53600	25.78600
H	27.29000	25.48600	24.85200
O	19.01900	22.22000	25.09600
H	19.84500	21.92000	24.71500
H	18.66800	21.45000	25.54500
O	23.98700	21.57200	27.16500
H	23.36500	20.90900	26.86600
H	24.65700	21.59800	26.48300
O	17.22000	20.13800	26.05400
H	16.38300	19.71100	25.87300
H	17.19200	20.33200	26.99200
O	24.05900	10.05100	36.75900
H	24.10800	10.92700	36.37600
H	24.60200	9.50800	36.18600
O	23.54800	20.12300	20.64100
H	23.69700	20.80600	19.98800
H	24.35500	20.10300	21.15600
O	26.28900	10.80400	33.51300
H	25.42000	11.09700	33.24200
H	26.52500	11.38800	34.23400
O	37.36600	17.42900	26.11600
H	38.20100	17.86800	26.28000
H	37.50200	16.95300	25.29700
O	21.30400	16.14900	21.64700
H	21.05800	15.24500	21.84500

H	21.47300	16.15100	20.70500
O	28.99300	7.02200	31.03000
H	29.51100	7.75800	30.70400
H	28.63900	7.32800	31.86400
O	20.57300	11.88200	19.14600
H	19.88000	11.95700	18.49000
H	20.32800	11.11800	19.66800
O	29.24500	3.44200	26.55000
H	29.52500	2.77400	27.17600
H	30.03000	3.63500	26.03700
O	27.04800	22.61800	18.11200
H	26.47500	23.23700	17.65900
H	27.62100	23.16700	18.64600
O	33.11500	11.86300	30.29700
H	34.06700	11.96300	30.31800
H	32.94600	11.07100	30.80700
O	31.13700	8.94900	30.48300
H	30.54200	9.22900	29.78800
H	30.85200	9.43800	31.25500
O	30.26800	22.90100	19.24300
H	31.20100	22.83600	19.44700
H	30.18000	23.73700	18.78500
O	32.89600	22.21800	16.20400
H	32.81800	21.88400	17.09700
H	33.71700	22.71000	16.20300
O	35.59400	15.08400	29.41100
H	35.56200	14.14600	29.59700
H	36.20400	15.16200	28.67800
O	27.43300	13.95400	15.45400
H	28.37800	13.82400	15.52300
H	27.05400	13.21500	15.93100
O	35.74400	11.87500	29.45700
H	35.22800	11.09100	29.27000
H	36.62800	11.54700	29.62200
O	33.40500	5.61900	25.41700
H	33.20700	5.31100	26.30100
H	34.01500	6.34500	25.54600
O	25.61700	11.87900	15.38900
H	24.71000	11.58200	15.46600
H	25.89400	11.58200	14.52300
O	16.53500	13.19900	25.83900
H	16.21500	12.34600	25.54700
H	15.98800	13.41400	26.59500
O	20.31300	15.91600	29.55600
H	20.53100	16.84700	29.57500
H	20.72500	15.55600	30.34200
O	27.23300	6.42200	21.54400
H	26.42300	6.07700	21.91900
H	27.92200	6.11300	22.13300
O	29.16200	5.50000	23.16600
H	29.71500	5.92000	23.82500
H	29.39900	4.57400	23.20900
O	24.38700	8.73700	15.53100
H	25.24900	9.06300	15.79000
H	24.57200	7.95000	15.01800
O	16.40300	16.82000	27.80900
H	15.71000	16.40500	27.29600
H	17.15100	16.86200	27.21200
O	32.54000	7.61000	22.67400
H	31.96000	8.26900	22.29200
H	32.27400	6.78800	22.26300
O	30.99000	7.01100	24.87600
H	31.49000	7.12200	24.06800
H	31.63400	6.70700	25.51600
O	18.31700	16.75100	25.68500
H	18.29400	16.85000	24.73300
H	18.62700	17.59800	26.00700
O	20.97600	18.80900	29.80000
H	21.93000	18.77700	29.73300
H	20.77700	18.34800	30.61500
O	21.56600	17.37900	32.36700
H	22.38500	17.87400	32.39100
H	21.84000	16.46200	32.33900
O	31.32300	9.77000	21.73000
H	30.43500	10.05500	21.51200
H	31.73800	9.61000	20.88300
O	34.67400	9.79500	25.44500
H	35.45000	10.28400	25.71800
H	33.94900	10.22100	25.90200
O	37.11100	10.50800	26.50300
H	37.70600	10.26700	25.79200
H	37.18400	9.78800	27.13000
O	19.33100	24.35100	26.81700
H	19.19600	23.59100	26.25100
H	18.91400	25.07500	26.35000
O	30.46700	19.53500	15.82500
H	31.17000	19.74000	16.44000
H	29.74300	20.10200	16.09000
O	31.29700	15.28600	21.94500
H	31.24000	15.74200	21.10600
H	31.84900	14.52500	21.76700
O	35.10700	11.07500	22.77000
H	34.85100	10.67400	23.60100
H	34.48200	11.78900	22.64500
O	39.41800	13.81400	25.27700
H	38.85800	13.88400	26.05000
H	38.81300	13.63800	24.55700
O	22.48800	25.24800	27.02900
H	22.37100	26.19200	26.92700
H	21.66700	24.94500	27.41700
O	32.37500	19.97300	17.87400
H	33.21000	19.54100	17.69200
H	32.21400	19.80100	18.80200
O	32.03000	16.47600	19.24000
H	31.97500	16.31100	18.29900
H	31.40500	17.18400	19.39600
O	16.81900	11.71300	33.06600
H	17.46500	11.20300	33.55500
H	16.61100	12.45000	33.64100
O	25.34000	5.97700	18.81900
H	25.32800	6.92100	18.97500
H	26.23200	5.79400	18.52500
O	25.26900	24.65400	26.90500
H	24.31800	24.65400	26.79200
H	25.57200	23.90700	26.38900
O	29.15500	2.57100	23.07300
H	28.38800	2.01700	23.21900
H	29.56600	2.64300	23.93500

O	21.15900	8.86000	37.16400
H	22.02200	8.50300	37.37400
H	20.58600	8.52100	37.85100
O	35.51400	29.21100	17.23800
H	35.76600	28.69000	16.47600
H	34.57600	29.04800	17.34100
O	35.23300	31.11400	19.44000
H	35.11700	30.43900	20.10800
H	35.34400	30.62700	18.62300
O	37.21700	25.60400	18.14100
H	37.38400	26.47200	18.50800
H	37.02700	25.76700	17.21700
O	37.19600	28.06000	19.12600
H	37.07600	28.50600	19.96400
H	36.50100	28.40900	18.56700
O	33.83700	26.42700	18.38800
H	33.00100	26.76400	18.70900
H	33.69700	26.29200	17.45100
O	35.81200	21.27000	23.09600
H	35.15900	20.95500	22.47200
H	35.77000	20.64800	23.82200
O	36.92300	31.55200	15.01100
H	36.01600	31.32100	15.21000
H	37.42000	30.75600	15.19900
O	28.67100	29.37400	23.51500
H	28.47300	28.78100	22.79000
H	28.11600	29.06900	24.23300
O	37.80000	33.19400	16.87200
H	37.36200	32.47800	16.41300
H	38.54100	33.42200	16.31100
O	31.16600	28.90000	21.19900
H	30.23500	28.83900	21.41600
H	31.26100	28.37800	20.40300
O	31.08400	25.42600	23.78700
H	31.34500	26.34000	23.67800
H	31.90600	24.95800	23.93700
O	33.71100	26.27100	15.58800
H	32.83400	26.15000	15.22400
H	34.18600	25.48000	15.33600
O	28.43900	27.86600	21.02900
H	28.73400	26.95700	20.97600
H	27.53700	27.84400	20.70700
O	30.16700	25.54700	21.15300
H	29.83900	24.66700	20.97000
H	30.52100	25.49200	22.04100
O	33.28200	25.83400	21.52100
H	33.62100	26.36300	20.79800
H	33.72900	26.17200	22.29600
O	35.26800	24.33200	19.72000
H	35.98900	24.71000	19.21600
H	34.64500	25.05100	19.82300
O	39.41100	25.03600	20.65600
H	39.62800	25.91900	20.35500
H	38.93100	24.64500	19.92600
O	35.41500	23.86700	22.55000
H	35.53600	22.93800	22.74900
H	35.12500	23.88000	21.63700
O	31.41300	27.21100	19.14600
H	30.71900	27.28600	18.49000
H	31.16800	26.44700	19.66800
O	38.27200	29.28300	15.45400
H	39.21800	29.15300	15.52300
H	37.89300	28.54400	15.93100
O	36.45600	27.20800	15.38900
H	35.54900	26.91100	15.46600
H	36.73400	26.91100	14.52300
O	38.07200	21.75200	21.54400
H	37.26300	21.40600	21.91900
H	38.76100	21.44200	22.13300
O	40.00200	20.82900	23.16600
H	40.55500	21.24900	23.82500
H	40.23900	19.90300	23.20900
O	35.22600	24.06700	15.53100
H	36.08800	24.39200	15.79000
H	35.41100	23.28000	15.01800
O	36.17900	21.30600	18.81900
H	36.16700	22.25000	18.97500
H	37.07100	21.12300	18.52500
O	39.99400	17.90000	23.07300
H	39.22800	17.34600	23.21900
H	40.40500	17.97300	23.93500
O	-8.40100	-43.48000	9.49900
H	-8.47800	-42.52900	9.57800
H	-9.00500	-43.82600	10.15700
O	-10.86500	-47.48800	10.87500
H	-10.68800	-46.56600	10.68700
H	-10.06900	-47.80200	11.30400
O	-9.74100	-45.97000	16.87600
H	-9.19100	-46.09800	16.10400
H	-10.58000	-45.67100	16.52500
O	-3.52700	-44.04900	4.65500
H	-4.20100	-43.74500	5.26400
H	-3.14100	-43.24600	4.30600
O	-6.61700	-46.43100	12.05200
H	-6.65900	-46.20500	11.12200
H	-5.70100	-46.66400	12.20000
O	-5.32800	-43.88100	6.87000
H	-5.15300	-44.35200	7.68500
H	-6.01500	-43.25400	7.09800
O	-10.76200	-40.73200	13.64400
H	-10.52200	-40.10000	14.32200
H	-11.60500	-41.08300	13.93100
O	-6.24900	-44.92400	16.04800
H	-6.32200	-44.11800	16.56000
H	-6.69200	-44.72500	15.22300
O	-6.46000	-45.68700	9.55900
H	-7.24400	-45.14400	9.65200
H	-5.76100	-45.16900	9.95800
O	-3.79000	-45.02500	18.20400
H	-3.07700	-44.39000	18.13200
H	-4.28100	-44.92900	17.38800
O	-8.73800	-47.27600	19.02500
H	-8.03700	-46.64200	19.17800
H	-9.20900	-46.93400	18.26400
O	-3.97700	-44.68500	10.95300
H	-3.79500	-45.50300	11.41500
H	-3.15400	-44.46800	10.51500
O	-3.64300	-45.75800	8.21400

H	-3.11400	-46.26900	7.60100
H	-3.38000	-46.07400	9.07900
O	-13.14200	-47.74200	15.95800
H	-12.55600	-48.48800	16.09100
H	-13.33900	-47.43200	16.84100
O	-10.28800	-44.70700	10.97400
H	-10.95700	-44.22200	10.49000
H	-10.42600	-44.46000	11.88800
O	-6.14700	-42.67200	17.57200
H	-5.59500	-42.50900	18.33700
H	-5.80700	-42.08200	16.90000
O	-3.01000	-44.72400	14.55900
H	-3.13500	-45.52500	14.05000
H	-2.35200	-44.23000	14.07000
O	-6.00100	-45.10700	19.58700
H	-5.69200	-45.35200	20.45900
H	-5.20300	-44.90200	19.09900
O	-5.97900	-42.95300	11.81800
H	-6.41800	-43.44500	12.51100
H	-5.22800	-43.49600	11.57700
O	-13.49900	-46.76200	11.82000
H	-12.67300	-47.06100	11.44000
H	-13.85000	-47.53100	12.26900
O	-8.53100	-47.40900	13.89000
H	-9.15300	-48.07200	13.59000
H	-7.86100	-47.38300	13.20700
O	-15.29800	-48.84300	12.77900
H	-16.13500	-49.27100	12.59700
H	-15.32600	-48.65000	13.71600
O	-5.47000	-46.36400	4.83700
H	-6.04300	-45.74500	4.38400
H	-4.89700	-45.81400	5.37100
O	-13.18700	-44.63000	13.54200
H	-13.32200	-45.39000	12.97500
H	-13.60400	-43.90600	13.07400
O	-10.03000	-43.73300	13.75300
H	-10.14700	-42.78900	13.65100
H	-10.85100	-44.03600	14.14200
O	-9.00500	-43.08100	16.96900
H	-8.10200	-43.04800	17.28600
H	-9.23100	-44.01100	16.98400
O	-7.24900	-44.32800	13.63000
H	-8.20000	-44.32700	13.51600
H	-6.94600	-45.07400	13.11400
O	8.48600	-38.49100	14.69500
H	9.12400	-38.61100	13.99100
H	8.36800	-37.54300	14.75300
O	5.29500	-38.53900	14.63100
H	4.95700	-38.65500	13.74300
H	6.16400	-38.94000	14.61100
O	1.15300	-36.68500	10.62000
H	0.59000	-36.77600	9.85200
H	1.03900	-37.50500	11.10100
O	5.77700	-37.38500	20.58900
H	5.76600	-37.78000	19.71700
H	5.93900	-38.11900	21.18300
O	-1.30000	-35.19000	13.45900
H	-0.50800	-35.53100	13.04300
H	-1.06000	-35.08100	14.37900
O	6.73300	-33.01800	5.93200
H	6.08200	-32.43300	5.54500
H	7.52800	-32.48700	5.99100
O	0.65900	-35.92900	6.33100
H	1.43200	-36.47600	6.19200
H	1.01200	-35.06500	6.54200
O	2.49700	-34.60200	19.53700
H	2.20600	-33.69700	19.42200
H	3.13300	-34.56000	20.25200
O	3.42300	-37.22400	16.12700
H	4.01900	-37.44800	15.41300
H	3.48100	-37.96500	16.73000
O	2.99600	-39.77000	3.96200
H	3.24800	-40.29200	3.20100
H	2.05800	-39.93300	4.06500
O	8.04000	-40.76600	11.77000
H	8.82600	-41.18400	11.41900
H	8.30400	-39.86200	11.93800
O	8.21100	-35.32900	6.46900
H	7.57600	-35.25600	7.18100
H	7.88100	-34.74000	5.79100
O	2.71500	-37.86700	6.16500
H	2.59900	-38.54300	6.83300
H	2.82500	-38.35400	5.34800
O	5.05800	-38.40600	9.86900
H	4.69400	-38.93300	10.58100
H	5.91500	-38.79600	9.69600
O	4.69900	-43.37700	4.86600
H	4.86600	-42.50900	5.23300
H	4.50900	-43.21400	3.94200
O	7.45600	-34.91500	16.53200
H	7.15700	-34.01300	16.42400
H	7.11200	-35.18200	17.38400
O	0.90800	-38.46400	12.56600
H	-0.01700	-38.59600	12.77400
H	1.15600	-37.68500	13.06300
O	4.67800	-40.92100	5.85000
H	4.55800	-40.47600	6.68900
H	3.98300	-40.57300	5.29200
O	-1.73100	-38.82100	13.40000
H	-1.60200	-38.56700	14.31400
H	-2.41200	-38.22800	13.08100
O	2.43900	-28.15100	9.49900
H	2.36100	-27.20000	9.57800
H	1.83500	-28.49600	10.15700
O	4.93000	-40.97800	22.15500
H	4.15400	-40.41900	22.14600
H	5.56900	-40.49300	22.67700
O	10.37600	-40.44900	6.67800
H	11.28800	-40.21600	6.50300
H	9.86700	-39.86500	6.11600
O	3.18500	-38.17000	21.71700
H	3.92600	-37.63100	21.44100
H	2.47900	-37.54300	21.87400
O	8.66000	-36.02700	20.31400
H	9.06900	-36.46800	19.57000
H	7.93800	-36.60100	20.56800
O	5.01400	-41.08900	17.68800
H	5.86000	-40.66600	17.83100

H	4.95300	-41.74600	18.38200
O	8.98300	-35.46700	14.07000
H	8.35400	-35.30200	13.36700
H	8.59600	-35.05200	14.84100
O	12.86200	-34.53100	11.42400
H	12.17700	-34.15100	10.87400
H	12.94700	-33.92200	12.15700
O	10.11700	-36.88500	18.02400
H	10.32900	-36.24200	17.34700
H	10.07000	-37.71900	17.55600
O	4.53600	-35.72900	11.33500
H	5.00000	-35.45000	10.54500
H	4.27200	-36.63100	11.15200
O	1.42900	-36.18200	14.27300
H	1.99900	-35.75300	13.63400
H	2.01900	-36.46400	14.97100
O	3.47300	-39.60700	8.14400
H	4.17500	-39.15500	8.61100
H	3.14100	-40.24800	8.77200
O	1.92100	-41.01600	22.51900
H	1.29100	-40.33400	22.75200
H	1.81500	-41.12700	21.57400
O	-2.78400	-41.34900	13.44500
H	-3.57500	-41.09300	12.96900
H	-2.29300	-40.53400	13.55100
O	11.23200	-34.00800	16.19600
H	12.05400	-34.41000	15.91400
H	11.22800	-34.11100	17.14700
O	-0.83300	-40.98400	10.40400
H	-1.63000	-40.87200	10.92200
H	-1.06000	-40.64500	9.53800
O	4.99800	-34.46000	18.35200
H	5.06200	-33.99800	19.18800
H	4.28900	-35.09000	18.48000
O	-4.50100	-41.09500	15.87700
H	-4.25000	-40.21100	16.14300
H	-3.96500	-41.27500	15.10400
O	19.32300	-37.66400	12.54400
H	18.56900	-38.23300	12.38900
H	19.45700	-37.69800	13.49100
O	4.71200	-32.79300	8.22000
H	4.57700	-31.94800	8.64800
H	5.25300	-32.59100	7.45600
O	-1.83500	-35.27500	8.79300
H	-1.32200	-34.48100	8.94300
H	-1.18200	-35.97300	8.72600
O	-0.02500	-32.15900	10.87500
H	0.15100	-31.23700	10.68700
H	0.77000	-32.47300	11.30400
O	-0.74000	-40.11200	20.75100
H	-0.85500	-41.01400	21.05200
H	-1.59600	-39.86600	20.40000
O	6.33900	-32.17000	22.35000
H	5.89600	-32.95700	22.03200
H	7.00100	-31.98200	21.68400
O	1.09800	-30.64100	16.87600
H	1.64900	-30.76900	16.10400
H	0.25900	-30.34100	16.52500
O	8.43700	-31.89000	20.37800
H	8.41900	-31.06900	19.88600
H	9.35900	-32.01500	20.60300
O	4.89500	-34.37600	21.22000
H	5.42700	-35.13700	20.99000
H	4.36600	-34.66800	21.96300
O	-3.87000	-36.94600	9.56900
H	-3.03200	-36.53700	9.35200
H	-3.69200	-37.88600	9.53900
O	8.02700	-39.27900	5.25700
H	7.51100	-38.50800	5.49600
H	7.75400	-39.95200	5.88000
O	13.00000	-36.52600	7.19200
H	12.22300	-36.86500	6.74800
H	13.67900	-36.52000	6.51800
O	7.81500	-41.40600	7.08300
H	7.51000	-41.09200	7.93400
H	8.76800	-41.32800	7.12600
O	6.26000	-35.24700	8.73200
H	5.73200	-35.98200	8.42000
H	5.75900	-34.47000	8.48600
O	11.23900	-37.05200	13.84900
H	10.58200	-36.36200	13.93700
H	12.04300	-36.66300	14.19200
O	0.99200	-44.58500	11.09300
H	1.56600	-44.80800	10.36000
H	1.57900	-44.51600	11.84600
O	10.05000	-38.69800	11.89900
H	10.56300	-38.06500	12.40200
H	10.70000	-39.29800	11.53400
O	1.31900	-42.55500	5.11300
H	0.48200	-42.21700	5.43300
H	1.17900	-42.69000	4.17500
O	4.40500	-37.42900	1.73600
H	3.49800	-37.66000	1.93500
H	4.90200	-38.22500	1.92300
O	7.31200	-28.71900	4.65500
H	6.63800	-28.41600	5.26400
H	7.69900	-27.91600	4.30600
O	6.52800	-41.22300	14.14500
H	5.61000	-41.00100	13.98700
H	6.94400	-41.14800	13.28600
O	7.31100	-39.82400	9.27500
H	8.05900	-39.31200	8.96600
H	7.58000	-40.14500	10.13600
O	5.58400	-37.32800	6.25800
H	4.67500	-37.61200	6.35500
H	5.61400	-36.90800	5.39800
O	10.81600	-33.71700	10.02700
H	10.36200	-34.55800	10.08400
H	10.18500	-33.08100	10.36500
O	9.25900	-31.88000	10.96200
H	9.55200	-31.31900	10.24300
H	9.76900	-31.58700	11.71800
O	-3.84700	-39.60700	10.24000
H	-4.04500	-40.20000	9.51500
H	-4.40300	-39.91200	10.95700
O	4.13200	-39.71500	12.35900
H	4.18700	-40.66800	12.28300
H	3.32200	-39.55900	12.84300

O	5.28200	-35.78700	3.59700
H	4.84400	-36.50300	3.13800
H	6.02300	-35.55900	3.03500
O	2.07200	-41.09700	10.11800
H	1.25900	-40.79800	10.52500
H	2.56800	-41.49200	10.83600
O	4.22300	-31.10100	12.05200
H	4.18000	-30.87500	11.12200
H	5.13900	-31.33500	12.20000
O	7.66900	-40.63300	18.95000
H	8.02400	-40.40600	19.80900
H	8.31600	-40.30100	18.32700
O	13.39900	-37.51100	9.65500
H	13.26100	-37.32100	8.72700
H	13.04300	-36.74900	10.11100
O	1.56300	-43.52500	15.73400
H	2.20300	-43.66700	15.03700
H	0.73100	-43.81200	15.35800
O	13.42600	-35.44400	14.66600
H	13.86000	-35.53600	15.51400
H	14.13900	-35.46800	14.02800
O	2.33900	-34.22500	11.84800
H	3.13300	-34.72000	11.64600
H	1.62800	-34.75300	11.48500
O	7.15900	-31.84300	16.19900
H	7.21200	-31.27400	16.96700
H	6.29000	-31.67300	15.83400
O	-1.35300	-40.08100	7.92400
H	-2.28300	-40.14200	8.14100
H	-1.25700	-40.60300	7.12700
O	-1.43400	-43.55500	10.51200
H	-1.17300	-42.64100	10.40300
H	-0.61200	-44.02300	10.66100
O	0.03800	-42.14200	17.68100
H	0.71100	-42.35000	17.03300
H	-0.78900	-42.33900	17.24200
O	14.28700	-35.40100	17.19900
H	14.48100	-34.49700	17.44500
H	14.18700	-35.86000	18.03300
O	9.34900	-35.25100	22.77300
H	9.19700	-35.47900	21.85600
H	9.33900	-34.29300	22.78600
O	6.11700	-37.67700	17.75800
H	7.04700	-37.52600	17.58500
H	5.69700	-37.58900	16.90300
O	4.91600	-33.65200	15.52800
H	4.77700	-34.02000	16.40000
H	4.12800	-33.13800	15.35600
O	5.51100	-28.55100	6.87000
H	5.68600	-29.02300	7.68500
H	4.82400	-27.92500	7.09800
O	6.46700	-34.27400	12.91600
H	6.13200	-34.31400	13.81200
H	5.96700	-34.94000	12.44300
O	0.44100	-38.86700	18.44200
H	0.30300	-39.59900	19.04300
H	1.35200	-38.95600	18.16000
O	9.40300	-36.92900	3.19000
H	8.78600	-37.65400	3.09300
H	8.93400	-36.16800	2.84700
O	3.06900	-39.15500	17.93000
H	3.77900	-39.79600	17.88900
H	3.02600	-38.90400	18.85300
O	11.05800	-32.21900	20.85100
H	10.99900	-32.90300	20.18400
H	11.46000	-31.47700	20.39900
O	1.78200	-42.16900	19.75000
H	1.24600	-42.62000	20.40200
H	1.19400	-42.03400	19.00700
O	0.07700	-25.40200	13.64400
H	0.31700	-24.77100	14.32200
H	-0.76600	-25.75400	13.93100
O	1.19300	-42.71000	2.31200
H	0.31600	-42.83100	1.94900
H	1.66800	-43.50100	2.06000
O	11.06000	-34.25500	19.08200
H	10.17300	-34.59200	19.20500
H	11.63000	-34.98600	19.31900
O	-7.28700	-37.92200	12.40600
H	-6.60000	-38.23600	11.81900
H	-7.44200	-37.01800	12.13300
O	-2.48400	-43.03300	16.83500
H	-2.56800	-43.64100	16.10000
H	-3.30500	-42.54200	16.83600
O	4.59000	-29.59500	16.04800
H	4.51700	-28.78900	16.56000
H	4.14800	-29.39500	15.22300
O	4.37900	-30.35700	9.55900
H	3.59600	-29.81500	9.65200
H	5.07800	-29.84000	9.95800
O	15.54200	-38.84400	13.84400
H	15.53100	-37.96100	13.47300
H	14.67300	-39.19700	13.64900
O	7.05000	-29.69600	18.20400
H	7.76300	-29.06100	18.13200
H	6.55900	-29.59900	17.38800
O	8.77200	-35.79100	9.77600
H	7.83700	-35.67100	9.60600
H	8.94900	-36.69300	9.51200
O	4.27200	-42.40600	11.92900
H	3.90200	-42.95900	12.61700
H	4.76800	-43.01000	11.37700
O	12.15900	-39.94900	10.31100
H	12.62500	-39.11600	10.23700
H	12.61000	-40.41400	11.01500
O	2.10100	-31.94600	19.02500
H	2.80200	-31.31300	19.17800
H	1.63100	-31.60500	18.26400
O	10.22700	-33.60500	7.30900
H	10.50200	-33.71900	8.21900
H	9.35700	-34.00300	7.27100
O	9.90100	-39.39200	16.85000
H	9.24800	-39.14400	16.19500
H	10.27900	-40.20600	16.51600
O	6.86200	-29.35600	10.95300
H	7.04400	-30.17400	11.41500
H	7.68500	-29.13900	10.51500
O	7.19600	-30.42900	8.21400

H	7.72500	-30.94000	7.60100
H	7.45900	-30.74500	9.07900
O	5.95000	-38.65000	23.33600
H	5.27400	-38.75100	24.00700
H	6.77300	-38.80600	23.80000
O	-2.30200	-32.41300	15.95800
H	-1.71700	-33.15800	16.09100
H	-2.50000	-32.10200	16.84100
O	0.55200	-29.37800	10.97400
H	-0.11700	-28.89300	10.49000
H	0.41300	-29.13100	11.88800
O	-7.71000	-39.84200	14.67800
H	-7.67200	-40.37600	15.47200
H	-8.21600	-40.36900	14.05900
O	4.69200	-27.34300	17.57200
H	5.24400	-27.18000	18.33700
H	5.03300	-26.75300	16.90000
O	-0.33100	-44.99600	18.17400
H	-0.26700	-44.13400	17.76200
H	-0.39500	-45.60800	17.44200
O	-0.85600	-42.92300	21.82200
H	-0.22900	-43.30600	22.43600
H	-1.71300	-43.14200	22.18700
O	9.54300	-32.51700	23.42700
H	10.08700	-31.90400	22.93300
H	8.96200	-31.95900	23.94400
O	-4.08000	-41.11600	7.75400
H	-3.78400	-42.02500	7.70000
H	-4.98100	-41.13700	7.43200
O	7.82900	-29.39500	14.55900
H	7.70400	-30.19600	14.05000
H	8.48700	-28.90000	14.07000
O	-0.55000	-43.04000	13.73600
H	-1.27100	-42.42700	13.59700
H	0.23900	-42.50400	13.65800
O	9.89300	-31.59400	16.18700
H	8.94600	-31.71400	16.11400
H	10.24100	-32.48200	16.26900
O	-2.35100	-43.43400	7.87800
H	-2.67900	-44.31500	7.69500
H	-1.99700	-43.49000	8.76500
O	6.89700	-31.79900	12.38700
H	6.75000	-32.72700	12.56800
H	7.72300	-31.77700	11.90300
O	0.76300	-43.14700	8.24500
H	1.10300	-42.61900	7.52300
H	1.21100	-42.80900	9.02100
O	10.50100	-30.97600	13.47400
H	11.42500	-31.18200	13.33600
H	10.33200	-31.22400	14.38200
O	2.75000	-44.65000	6.44400
H	3.47100	-44.27200	5.94100
H	2.12700	-43.93100	6.54800
O	1.63300	-40.98700	13.95700
H	1.48800	-40.67300	14.85000
H	1.15600	-40.36800	13.40400
O	10.29000	-30.45700	8.83000
H	10.46400	-31.02300	8.07700
H	10.78300	-29.65700	8.64800
O	-3.89200	-38.44600	16.89600
H	-4.43700	-37.67000	17.02800
H	-3.01400	-38.09900	16.73800
O	16.14400	-37.69400	10.44900
H	16.58700	-38.44300	10.05100
H	15.26700	-37.70300	10.06600
O	11.27800	-31.14100	5.92900
H	11.97300	-30.52000	6.14800
H	11.62000	-31.99100	6.20500
O	-3.10600	-39.31900	19.78900
H	-3.84700	-39.17100	20.37600
H	-3.42600	-39.05800	18.92600
O	13.17700	-33.69100	7.45800
H	13.70200	-33.35200	6.73300
H	13.08900	-34.62600	7.27400
O	4.83900	-29.77700	19.58700
H	5.14800	-30.02300	20.45900
H	5.63600	-29.57300	19.09900
O	4.86100	-27.62400	11.81800
H	4.42100	-28.11600	12.51100
H	5.61200	-28.16600	11.57700
O	-2.66000	-31.43200	11.82000
H	-1.83400	-31.73200	11.44000
H	-3.01000	-32.20200	12.26900
O	2.30800	-32.08000	13.89000
H	1.68600	-32.74300	13.59000
H	2.97900	-32.05400	13.20700
O	-4.45900	-33.51400	12.77900
H	-5.29600	-33.94200	12.59700
H	-4.48700	-33.32100	13.71600
O	2.38100	-43.60200	23.48300
H	2.43000	-42.72500	23.10100
H	2.92300	-44.14400	22.91000
O	12.86600	-33.05800	22.76300
H	12.07600	-33.07500	22.22400
H	12.63000	-32.51900	23.51800
O	12.98600	-36.20800	20.07700
H	13.03600	-36.37300	21.01800
H	12.87700	-37.07600	19.68600
O	1.87000	-33.52900	7.36600
H	2.01800	-32.84600	6.71200
H	2.67600	-33.54900	7.88100
O	10.27600	-40.22500	21.39200
H	11.17700	-40.36500	21.10100
H	10.35100	-40.06000	22.33200
O	4.61100	-42.84900	20.23800
H	3.74100	-42.55500	19.96600
H	4.84700	-42.26400	20.95900
O	14.48800	-33.78900	20.32100
H	14.19600	-33.42600	21.15700
H	13.87500	-34.50200	20.14200
O	15.68700	-36.22300	12.84100
H	16.52200	-35.78400	13.00500
H	15.82300	-36.69900	12.02200
O	12.73700	-38.66500	18.78900
H	13.21200	-38.47300	17.98100
H	11.81300	-38.60900	18.54300
O	-0.37500	-37.50300	8.37200
H	-0.62100	-38.40700	8.57000

H	-0.20600	-37.50100	7.43000
O	-1.10600	-41.77000	5.87000
H	-1.79900	-41.69600	5.21400
H	-1.35000	-42.53400	6.39300
O	5.36900	-31.03500	4.83700
H	4.79700	-30.41600	4.38400
H	5.94200	-30.48500	5.37100
O	8.58900	-30.75100	5.96800
H	9.52200	-30.81600	6.17100
H	8.50100	-29.91500	5.50900
O	11.21800	-31.43400	2.92800
H	11.13900	-31.76800	3.82200
H	12.03900	-30.94200	2.92700
O	13.91500	-38.56800	16.13600
H	13.88300	-39.50700	16.32200
H	14.52600	-38.49000	15.40200
O	5.75400	-39.69900	2.17900
H	6.70000	-39.82800	2.24700
H	5.37500	-40.43700	2.65500
O	19.73300	-37.50700	15.27600
H	20.46300	-37.76800	15.83700
H	19.12400	-38.24400	15.31700
O	5.63800	-35.64700	23.63900
H	6.55800	-35.46900	23.83300
H	5.60300	-36.59100	23.48700
O	3.93800	-41.77400	2.11400
H	3.03100	-42.07000	2.19000
H	4.21600	-42.07100	1.24700
O	-5.14400	-40.45300	12.56400
H	-5.46400	-41.30600	12.27100
H	-5.69100	-40.23800	13.32000
O	-1.36600	-37.73700	16.28100
H	-1.14700	-36.80500	16.30000
H	-0.95400	-38.09600	17.06600
O	3.50700	-42.16300	25.67100
H	3.40600	-41.21400	25.60200
H	3.28900	-42.49200	24.79900
O	-5.27500	-36.83300	14.53300
H	-5.96900	-37.24700	14.02000
H	-4.52800	-36.79100	13.93700
O	3.37000	-38.73400	24.48800
H	2.88700	-37.93400	24.69400
H	3.45600	-38.72300	23.53400
O	-3.36200	-36.90100	12.41000
H	-3.38500	-36.80200	11.45800
H	-3.05200	-36.05400	12.73200
O	-0.70300	-34.84300	16.52400
H	0.25100	-34.87500	16.45700
H	-0.90200	-35.30400	17.34000
O	-0.11300	-36.27300	19.09200
H	0.70600	-35.77900	19.11500
H	0.16100	-37.19000	19.06300
O	-2.34800	-29.30100	13.54200
H	-2.48200	-30.06100	12.97500
H	-2.76500	-28.57700	13.07400
O	8.78800	-34.11800	2.54900
H	9.49200	-33.91300	3.16500
H	8.06400	-33.55000	2.81400
O	9.61800	-38.36700	8.66900
H	9.56100	-37.91000	7.83000
H	10.17100	-39.12700	8.49200
O	0.80900	-28.40400	13.75300
H	0.69200	-27.46000	13.65100
H	-0.01200	-28.70700	14.14200
O	10.69700	-33.67900	4.59900
H	11.53100	-34.11100	4.41700
H	10.53500	-33.85100	5.52700
O	10.35100	-37.17600	5.96500
H	10.29600	-37.34200	5.02300
H	9.72600	-36.46800	6.12100
O	-4.85900	-41.93900	19.79100
H	-4.21400	-42.44900	20.28000
H	-5.06800	-41.20200	20.36500
O	7.66600	-34.87100	25.29000
H	8.53500	-34.69700	24.92900
H	7.18700	-34.05200	25.16800
O	1.83500	-27.75100	16.96900
H	2.73700	-27.71900	17.28600
H	1.60800	-28.68100	16.98400
O	3.59000	-28.99800	13.63000
H	2.63900	-28.99800	13.51600
H	3.89300	-29.74500	13.11400
O	-0.52000	-44.79200	23.88800
H	0.34300	-45.14900	24.09900
H	-1.09300	-45.13100	24.57600
O	19.32500	-23.16200	14.69500
H	19.96300	-23.28200	13.99100
H	19.20700	-22.21400	14.75300
O	16.13400	-23.21000	14.63100
H	15.79600	-23.32500	13.74300
H	17.00300	-23.61000	14.61100
O	11.99300	-21.35600	10.62000
H	11.42900	-21.44700	9.85200
H	11.87800	-22.17600	11.10100
O	16.61600	-22.05600	20.58900
H	16.60500	-22.45100	19.71700
H	16.77800	-22.78900	21.18300
O	9.53900	-19.86100	13.45900
H	10.33100	-20.20200	13.04300
H	9.77900	-19.75100	14.37900
O	17.57300	-17.68900	5.93200
H	16.92200	-17.10400	5.54500
H	18.36700	-17.15800	5.99100
O	11.49800	-20.60000	6.33100
H	12.27100	-21.14700	6.19200
H	11.85100	-19.73600	6.54200
O	13.33700	-19.27200	19.53700
H	13.04600	-18.36800	19.42200
H	13.97300	-19.23100	20.25200
O	14.26200	-21.89500	16.12700
H	14.85900	-22.11900	15.41300
H	14.32000	-22.63600	16.73000
O	13.83500	-24.44100	3.96200
H	14.08800	-24.96300	3.20100
H	12.89700	-24.60400	4.06500
O	18.87900	-25.43700	11.77000
H	19.66600	-25.85500	11.41900
H	19.14300	-24.53300	11.93800

O	19.05000	-20.00000	6.46900
H	18.41500	-19.92600	7.18100
H	18.72000	-19.41100	5.79100
O	24.28000	-25.50000	12.67300
H	25.01300	-26.07000	12.90700
H	23.52400	-25.88700	13.11400
O	13.55500	-22.53800	6.16500
H	13.43800	-23.21300	6.83300
H	13.66500	-23.02500	5.34800
O	15.89800	-23.07700	9.86900
H	15.53400	-23.60400	10.58100
H	16.75400	-23.46700	9.69600
O	21.80300	-31.78700	17.13300
H	21.47100	-30.91800	16.90900
H	21.20700	-32.10400	17.81200
O	15.53800	-28.04800	4.86600
H	15.70600	-27.18000	5.23300
H	15.34800	-27.88500	3.94200
O	18.29600	-19.58600	16.53200
H	17.99600	-18.68400	16.42400
H	17.95100	-19.85300	17.38400
O	11.74700	-23.13500	12.56600
H	10.82200	-23.26700	12.77400
H	11.99600	-22.35500	13.06300
O	21.69600	-27.06800	14.21200
H	20.78700	-27.13600	13.91800
H	21.64400	-26.59100	15.04000
O	15.51700	-25.59200	5.85000
H	15.39700	-25.14600	6.68900
H	14.82200	-25.24400	5.29200
O	9.10800	-23.49200	13.40000
H	9.23700	-23.23800	14.31400
H	8.42800	-22.89900	13.08100
O	13.27800	-12.82200	9.49900
H	13.20000	-11.87100	9.57800
H	12.67400	-13.16700	10.15700
O	15.77000	-25.64900	22.15500
H	14.99300	-25.09000	22.14600
H	16.40900	-25.16300	22.67700
O	23.56200	-28.86600	15.99000
H	22.91900	-28.57300	15.34400
H	23.05700	-28.98100	16.79500
O	21.21600	-25.12000	6.67800
H	22.12800	-24.88700	6.50300
H	20.70700	-24.53600	6.11600
O	14.02400	-22.84000	21.71700
H	14.76600	-22.30200	21.44100
H	13.31800	-22.21400	21.87400
O	19.50000	-20.69800	20.31400
H	19.90800	-21.13900	19.57000
H	18.77800	-21.27200	20.56800
O	15.85300	-25.76000	17.68800
H	16.69900	-25.33600	17.83100
H	15.79300	-26.41700	18.38200
O	19.82300	-20.13700	14.07000
H	19.19400	-19.97300	13.36700
H	19.43500	-19.72300	14.84100
O	23.70200	-19.20200	11.42400
H	23.01700	-18.82200	10.87400
H	23.78600	-18.59200	12.15700
O	19.00300	-28.10000	19.34600
H	18.75900	-27.34600	18.80900
H	19.27900	-27.72100	20.18100
O	20.95700	-21.55600	18.02400
H	21.16800	-20.91300	17.34700
H	20.91000	-22.39000	17.55600
O	18.55900	-28.94400	14.92700
H	17.69800	-28.64600	15.21900
H	18.58600	-28.72700	13.99500
O	15.37500	-20.40000	11.33500
H	15.83900	-20.12100	10.54500
H	15.11100	-21.30200	11.15200
O	12.26800	-20.85300	14.27300
H	12.83800	-20.42400	13.63400
H	12.85900	-21.13500	14.97100
O	14.31200	-24.27800	8.14400
H	15.01500	-23.82600	8.61100
H	13.98000	-24.91900	8.77200
O	12.76000	-25.68600	22.51900
H	12.13000	-25.00500	22.75200
H	12.65400	-25.79800	21.57400
O	8.05500	-26.01900	13.44500
H	7.26500	-25.76400	12.96900
H	8.54600	-25.20400	13.55100
O	22.07100	-18.67900	16.19600
H	22.89300	-19.08100	15.91400
H	22.06800	-18.78200	17.14700
O	10.00600	-25.65500	10.40400
H	9.20900	-25.54300	10.92200
H	9.77900	-25.31600	9.53800
O	15.83700	-19.13000	18.35200
H	15.90100	-18.66800	19.18800
H	15.12800	-19.76100	18.48000
O	6.33900	-25.76600	15.87700
H	6.58900	-24.88200	16.14300
H	6.87500	-25.94600	15.10400
O	30.16200	-22.33500	12.54400
H	29.40800	-22.90400	12.38900
H	30.29700	-22.36900	13.49100
O	15.55200	-17.46400	8.22000
H	15.41600	-16.61800	8.64800
H	16.09300	-17.26200	7.45600
O	12.03800	-31.38400	24.88100
H	11.64500	-31.93800	25.55600
H	12.85500	-31.07300	25.27200
O	9.00500	-19.94600	8.79300
H	9.51700	-19.15200	8.94300
H	9.65700	-20.64300	8.72600
O	10.81400	-16.82900	10.87500
H	10.99100	-15.90800	10.68700
H	11.61000	-17.14400	11.30400
O	14.95500	-29.96900	22.47300
H	15.69600	-29.36800	22.39400
H	15.18700	-30.71100	21.91500
O	10.09900	-24.78300	20.75100
H	9.98500	-25.68400	21.05200
H	9.24400	-24.53700	20.40000
O	17.17900	-16.84100	22.35000

H	16.73500	-17.62800	22.03200
H	17.84000	-16.65300	21.68400
O	11.93700	-15.31200	16.87600
H	12.48800	-15.44000	16.10400
H	11.09800	-15.01200	16.52500
O	19.27700	-16.56100	20.37800
H	19.25900	-15.74000	19.88600
H	20.19900	-16.68600	20.60300
O	15.73400	-19.04700	21.22000
H	16.26700	-19.80800	20.99000
H	15.20500	-19.33900	21.96300
O	18.90100	-33.94100	16.75000
H	18.55700	-33.05000	16.67400
H	19.26900	-34.13000	15.88700
O	6.97000	-21.61700	9.56900
H	7.80700	-21.20700	9.35200
H	7.14700	-22.55700	9.53900
O	18.86600	-23.95000	5.25700
H	18.35100	-23.17900	5.49600
H	18.59400	-24.62300	5.88000
O	16.45100	-29.10500	10.31600
H	15.59500	-29.49400	10.13600
H	16.86300	-29.02500	9.45500
O	17.98100	-31.57000	14.71500
H	17.47700	-31.60700	13.90100
H	18.27400	-30.66100	14.77400
O	14.22500	-31.48600	13.72100
H	15.09000	-31.68200	13.36200
H	14.29300	-31.71500	14.64800
O	23.83900	-21.19600	7.19200
H	23.06200	-21.53500	6.74800
H	24.51900	-21.19100	6.51800
O	18.65500	-26.07700	7.08300
H	18.35000	-25.76200	7.93400
H	19.60800	-25.99900	7.12600
O	17.09900	-19.91800	8.73200
H	16.57100	-20.65300	8.42000
H	16.59800	-19.14000	8.48600
O	22.07900	-21.72300	13.84900
H	21.42100	-21.03300	13.93700
H	22.88300	-21.33300	14.19200
O	11.83100	-29.25600	11.09300
H	12.40600	-29.47800	10.36000
H	12.41800	-29.18700	11.84600
O	20.88900	-23.36900	11.89900
H	21.40200	-22.73600	12.40200
H	21.53900	-23.96800	11.53400
O	12.15800	-27.22600	5.11300
H	11.32200	-26.88800	5.43300
H	12.01900	-27.36100	4.17500
O	14.13300	-32.38200	9.82000
H	13.48000	-32.69700	9.19600
H	14.09100	-33.00400	10.54600
O	15.24500	-22.10000	1.73600
H	14.33700	-22.33100	1.93500
H	15.74200	-22.89600	1.92300
O	18.15100	-13.39000	4.65500
H	17.47800	-13.08700	5.26400
H	18.53800	-12.58700	4.30600
O	17.36700	-25.89400	14.14500
H	16.44900	-25.67200	13.98700
H	17.78300	-25.81900	13.28600
O	18.15100	-24.49500	9.27500
H	18.89800	-23.98200	8.96600
H	18.42000	-24.81600	10.13600
O	16.42300	-21.99900	6.25800
H	15.51500	-22.28300	6.35500
H	16.45300	-21.57900	5.39800
O	21.65500	-18.38800	10.02700
H	21.20200	-19.22900	10.08400
H	21.02500	-17.75200	10.36500
O	20.59900	-27.45900	11.25700
H	19.86100	-27.97100	11.58800
H	20.85100	-27.89900	10.44600
O	20.09900	-16.55000	10.96200
H	20.39100	-15.99000	10.24300
H	20.60800	-16.25700	11.71800
O	20.70000	-34.68100	11.85200
H	21.00600	-35.54100	11.56400
H	21.42500	-34.08900	11.65100
O	6.99200	-24.27800	10.24000
H	6.79500	-24.87100	9.51500
H	6.43700	-24.58300	10.95700
O	14.97200	-24.38600	12.35900
H	15.02700	-25.33800	12.28300
H	14.16100	-24.23000	12.84300
O	16.12100	-20.45800	3.59700
H	15.68300	-21.17400	3.13800
H	16.86200	-20.23000	3.03500
O	12.91100	-25.76800	10.11800
H	12.09800	-25.46900	10.52500
H	13.40700	-26.16300	10.83600
O	15.06200	-15.77200	12.05200
H	15.02000	-15.54600	11.12200
H	15.97800	-16.00600	12.20000
O	18.50900	-25.30400	18.95000
H	18.86300	-25.07700	19.80900
H	19.15600	-24.97200	18.32700
O	24.23800	-22.18200	9.65500
H	24.10000	-21.99200	8.72700
H	23.88200	-21.42000	10.11100
O	12.40300	-28.19600	15.73400
H	13.04200	-28.33700	15.03700
H	11.57000	-28.48300	15.35800
O	24.26500	-20.11400	14.66600
H	24.69900	-20.20700	15.51400
H	24.97800	-20.13900	14.02800
O	13.17900	-18.89600	11.84800
H	13.97300	-19.39100	11.64600
H	12.46800	-19.42400	11.48500
O	17.99800	-16.51400	16.19900
H	18.05200	-15.94500	16.96700
H	17.13000	-16.34400	15.83400
O	9.48700	-24.75200	7.92400
H	8.55700	-24.81300	8.14100
H	9.58300	-25.27400	7.12700
O	9.40500	-28.22600	10.51200
H	9.66600	-27.31200	10.40300

H	10.22700	-28.69400	10.66100
O	10.87800	-26.81200	17.68100
H	11.55000	-27.02100	17.03300
H	10.05100	-27.01000	17.24200
O	25.12700	-20.07200	17.19900
H	25.32000	-19.16700	17.44500
H	25.02700	-20.53100	18.03300
O	16.78000	-30.18600	19.69800
H	16.13300	-29.48700	19.60400
H	17.55100	-29.75300	20.06600
O	20.18800	-19.92100	22.77300
H	20.03700	-20.15000	21.85600
H	20.17900	-18.96400	22.78600
O	16.95700	-22.34800	17.75800
H	17.88600	-22.19700	17.58500
H	16.53600	-22.26000	16.90300
O	15.75600	-18.32300	15.52800
H	15.61700	-18.69100	16.40000
H	14.96700	-17.80800	15.35600
O	16.35000	-13.22200	6.87000
H	16.52600	-13.69300	7.68500
H	15.66400	-12.59600	7.09800
O	17.30600	-18.94500	12.91600
H	16.97100	-18.98500	13.81200
H	16.80700	-19.61000	12.44300
O	11.28100	-23.53800	18.44200
H	11.14300	-24.27000	19.04300
H	12.19100	-23.62700	18.16000
O	20.24200	-21.59900	3.19000
H	19.62500	-22.32500	3.09300
H	19.77400	-20.83800	2.84700
O	13.90900	-23.82600	17.93000
H	14.61900	-24.46700	17.88900
H	13.86600	-23.57500	18.85300
O	21.89800	-16.88900	20.85100
H	21.83900	-17.57400	20.18400
H	22.29900	-16.14800	20.39900
O	12.62200	-26.84000	19.75000
H	12.08500	-27.29100	20.40200
H	12.03300	-26.70500	19.00700
O	10.91700	-10.07300	13.64400
H	11.15600	-9.44100	14.32200
H	10.07400	-10.42400	13.93100
O	12.03200	-27.38100	2.31200
H	11.15500	-27.50200	1.94900
H	12.50800	-28.17200	2.06000
O	21.89900	-18.92500	19.08200
H	21.01200	-19.26300	19.20500
H	22.46900	-19.65700	19.31900
O	3.55200	-22.59300	12.40600
H	4.23900	-22.90700	11.81900
H	3.39700	-21.68900	12.13300
O	8.35600	-27.70400	16.83500
H	8.27100	-28.31100	16.10000
H	7.53400	-27.21300	16.83600
O	15.42900	-14.26500	16.04800
H	15.35600	-13.46000	16.56000
H	14.98700	-14.06600	15.22300
O	15.21800	-15.02800	9.55900
H	14.43500	-14.48600	9.65200
H	15.91800	-14.51100	9.95800
O	18.52800	-29.21900	12.20200
H	19.02500	-29.97200	11.88200
H	17.75700	-29.18200	11.63600
O	26.38100	-23.51500	13.84400
H	26.37000	-22.63200	13.47300
H	25.51300	-23.86800	13.64900
O	17.88900	-14.36700	18.20400
H	18.60200	-13.73200	18.13200
H	17.39800	-14.27000	17.38800
O	19.61100	-20.46200	9.77600
H	18.67700	-20.34100	9.60600
H	19.78900	-21.36400	9.51200
O	15.11200	-27.07700	11.92900
H	14.74100	-27.63000	12.61700
H	15.60700	-27.68100	11.37700
O	22.99800	-24.62000	10.31100
H	23.46400	-23.78700	10.23700
H	23.44900	-25.08500	11.01500
O	12.94000	-16.61700	19.02500
H	13.64100	-15.98400	19.17800
H	12.47000	-16.27600	18.26400
O	21.06600	-18.27500	7.30900
H	21.34200	-18.39000	8.21900
H	20.19600	-18.67400	7.27100
O	20.97000	-32.25200	14.35000
H	20.05600	-32.02100	14.51800
H	21.39900	-32.16800	15.20100
O	20.74100	-24.06300	16.85000
H	20.08800	-23.81500	16.19500
H	21.11900	-24.87600	16.51600
O	17.70100	-14.02700	10.95300
H	17.88400	-14.84500	11.41500
H	18.52500	-13.81000	10.51500
O	18.03600	-15.10000	8.21400
H	18.56400	-15.61100	7.60100
H	18.29800	-15.41600	9.07900
O	16.78900	-23.32100	23.33600
H	16.11300	-23.42100	24.00700
H	17.61200	-23.47700	23.80000
O	8.53700	-17.08400	15.95800
H	9.12300	-17.82900	16.09100
H	8.34000	-16.77300	16.84100
O	11.39100	-14.04800	10.97400
H	10.72200	-13.56400	10.49000
H	11.25200	-13.80200	11.88800
O	3.12900	-24.51300	14.67800
H	3.16700	-25.04700	15.47200
H	2.62300	-25.03900	14.05900
O	15.53200	-12.01400	17.57200
H	16.08300	-11.85100	18.33700
H	15.87200	-11.42400	16.90000
O	10.50800	-29.66700	18.17400
H	10.57200	-28.80500	17.76200
H	10.44500	-30.27900	17.44200
O	9.98300	-27.59400	21.82200
H	10.61000	-27.97700	22.43600
H	9.12600	-27.81300	22.18700

O	20.38300	-17.18800	23.42700
H	20.92700	-16.57500	22.93300
H	19.80100	-16.63000	23.94400
O	6.76000	-25.78700	7.75400
H	7.05500	-26.69600	7.70000
H	5.85900	-25.80800	7.43200
O	18.66800	-14.06600	14.55900
H	18.54400	-14.86700	14.05000
H	19.32600	-13.57100	14.07000
O	10.28900	-27.71100	13.73600
H	9.56800	-27.09700	13.59700
H	11.07900	-27.17500	13.65800
O	20.73200	-16.26500	16.18700
H	19.78500	-16.38400	16.11400
H	21.08000	-17.15300	16.26900
O	13.09200	-30.37600	20.01000
H	13.61500	-30.12900	20.77300
H	12.87300	-29.54400	19.59100
O	8.48900	-28.10500	7.87800
H	8.16000	-28.98500	7.69500
H	8.84300	-28.16000	8.76500
O	17.73700	-16.47000	12.38700
H	17.58900	-17.39800	12.56800
H	18.56200	-16.44800	11.90300
O	11.60300	-27.81800	8.24500
H	11.94200	-27.28900	7.52300
H	12.05000	-27.48000	9.02100
O	21.34000	-15.64700	13.47400
H	22.26500	-15.85300	13.33600
H	21.17200	-15.89500	14.38200
O	14.29700	-29.43400	17.69500
H	13.95700	-30.23300	18.09700
H	13.59100	-29.13400	17.12300
O	14.94900	-32.34900	16.14800
H	15.77100	-32.82300	16.02600
H	15.19300	-31.57000	16.64800
O	13.59000	-29.32000	6.44400
H	14.31000	-28.94200	5.94100
H	12.96600	-28.60200	6.54800
O	17.73300	-28.61600	7.38000
H	17.94900	-27.73300	7.07900
H	17.25200	-29.00700	6.65100
O	12.47300	-25.65700	13.95700
H	12.32700	-25.34300	14.85000
H	11.99500	-25.03900	13.40400
O	14.33200	-28.66700	14.00500
H	14.31800	-29.56300	13.66900
H	15.05000	-28.66000	14.63900
O	16.18500	-28.23300	16.05100
H	15.92700	-27.31200	16.04900
H	15.65300	-28.63000	16.74100
O	21.13000	-15.12800	8.83000
H	21.30300	-15.69400	8.07700
H	21.62200	-14.32800	8.64800
O	6.94700	-23.11700	16.89600
H	6.40300	-22.34100	17.02800
H	7.82500	-22.77000	16.73800
O	13.73600	-29.78600	9.27400
H	13.85800	-30.71400	9.47300
H	13.44600	-29.77200	8.36200
O	16.60500	-32.10000	12.41100
H	16.62700	-32.03600	11.45600
H	17.02200	-32.94000	12.60500
O	26.98300	-22.36500	10.44900
H	27.42700	-23.11400	10.05100
H	26.10600	-22.37400	10.06600
O	22.11700	-15.81200	5.92900
H	22.81200	-15.19100	6.14800
H	22.45900	-16.66200	6.20500
O	7.73400	-23.99000	19.78900
H	6.99200	-23.84200	20.37600
H	7.41300	-23.72900	18.92600
O	24.01600	-18.36200	7.45800
H	24.54100	-18.02300	6.73300
H	23.92800	-19.29700	7.27400
O	15.67800	-14.44800	19.58700
H	15.98700	-14.69300	20.45900
H	16.47600	-14.24400	19.09900
O	15.70000	-12.29400	11.81800
H	15.26100	-12.78700	12.51100
H	16.45100	-12.83700	11.57700
O	8.17900	-16.10300	11.82000
H	9.00500	-16.40300	11.44000
H	7.82900	-16.87300	12.26900
O	13.14800	-16.75100	13.89000
H	12.52600	-17.41400	13.59000
H	13.81800	-16.72500	13.20700
O	6.38000	-18.18500	12.77900
H	5.54400	-18.61200	12.59700
H	6.35300	-17.99100	13.71600
O	13.22000	-28.27200	23.48300
H	13.26900	-27.39600	23.10100
H	13.76200	-28.81500	22.91000
O	23.70600	-17.72800	22.76300
H	22.91500	-17.74600	22.22400
H	23.46900	-17.19000	23.51800
O	23.82500	-20.87900	20.07700
H	23.87500	-21.04400	21.01800
H	23.71700	-21.74600	19.68600
O	12.70900	-18.20000	7.36600
H	12.85800	-17.51700	6.71200
H	13.51600	-18.22000	7.88100
O	21.11600	-24.89600	21.39200
H	22.01700	-25.03500	21.10100
H	21.19100	-24.73100	22.33200
O	15.45000	-27.51900	20.23800
H	14.58000	-27.22600	19.96600
H	15.68600	-26.93500	20.95900
O	25.32700	-18.45900	20.32100
H	25.03600	-18.09600	21.15700
H	24.71400	-19.17300	20.14200
O	26.52700	-20.89400	12.84100
H	27.36100	-20.45500	13.00500
H	26.66300	-21.37000	12.02200
O	23.57600	-23.33600	18.78900
H	24.05100	-23.14300	17.98100
H	22.65200	-23.28000	18.54300
O	10.46400	-22.17400	8.37200

H	10.21900	-23.07700	8.57000
H	10.63400	-22.17200	7.43000
O	18.15400	-31.30100	17.75400
H	18.67200	-30.56500	17.42900
H	17.79900	-30.99500	18.58900
O	9.73400	-26.44100	5.87000
H	9.04100	-26.36600	5.21400
H	9.48900	-27.20500	6.39300
O	18.40500	-34.88100	13.27400
H	18.68500	-35.54900	13.90000
H	19.19000	-34.68800	12.76200
O	16.20900	-15.70500	4.83700
H	15.63600	-15.08600	4.38400
H	16.78200	-15.15500	5.37100
O	22.27600	-26.46000	17.02200
H	23.22800	-26.36000	17.04300
H	22.10600	-27.25200	17.53100
O	20.29800	-29.37400	17.20800
H	19.70200	-29.09400	16.51200
H	20.01200	-28.88500	17.97900
O	19.42800	-15.42200	5.96800
H	20.36100	-15.48700	6.17100
H	19.34100	-14.58600	5.50900
O	22.05700	-16.10500	2.92800
H	21.97800	-16.43900	3.82200
H	22.87800	-15.61300	2.92700
O	24.75500	-23.23900	16.13600
H	24.72300	-24.17700	16.32200
H	25.36500	-23.16100	15.40200
O	16.59300	-24.36900	2.17900
H	17.53900	-24.49900	2.24700
H	16.21500	-25.10800	2.65500
O	24.90400	-26.44800	16.18200
H	24.38800	-27.23200	15.99500
H	25.78800	-26.77600	16.34600
O	22.56500	-32.70400	12.14100
H	22.36800	-33.01200	13.02600
H	23.17500	-31.97800	12.27100
O	30.57300	-22.17700	15.27600
H	31.30300	-22.43900	15.83700
H	29.96300	-22.91400	15.31700
O	28.65400	-24.03700	15.87400
H	27.92700	-24.17100	15.26500
H	28.23900	-23.72900	16.68000
O	16.47700	-20.31800	23.63900
H	17.39700	-20.14000	23.83300
H	16.44200	-21.26200	23.48700
O	14.77700	-26.44400	2.11400
H	13.87100	-26.74100	2.19000
H	15.05500	-26.74100	1.24700
O	5.69500	-25.12400	12.56400
H	5.37600	-25.97700	12.27100
H	5.14900	-24.90900	13.32000
O	9.47300	-22.40700	16.28100
H	9.69200	-21.47600	16.30000
H	9.88600	-22.76700	17.06600
O	16.39400	-31.90100	8.26800
H	15.58400	-32.24600	8.64400
H	17.08200	-32.21000	8.85700
O	18.32300	-32.82300	9.89100
H	18.87600	-32.40300	10.55000
H	18.56000	-33.74900	9.93300
O	14.34600	-26.83400	25.67100
H	14.24600	-25.88500	25.60200
H	14.12800	-27.16200	24.79900
O	13.54700	-29.58600	2.25500
H	14.40900	-29.26000	2.51500
H	13.73200	-30.37300	1.74300
O	5.56400	-21.50300	14.53300
H	4.87000	-21.91800	14.02000
H	6.31100	-21.46100	13.93700
O	21.70100	-30.71300	9.39900
H	21.12100	-30.05400	9.01700
H	21.43400	-31.53500	8.98700
O	20.15100	-31.31200	11.60100
H	20.65100	-31.20100	10.79200
H	20.79500	-31.61600	12.24100
O	14.20900	-23.40400	24.48800
H	13.72600	-22.60400	24.69400
H	14.29500	-23.39400	23.53400
O	7.47800	-21.57200	12.41000
H	7.45500	-21.47300	11.45800
H	7.78800	-20.72500	12.73200
O	10.13700	-19.51400	16.52400
H	11.09100	-19.54600	16.45700
H	9.93800	-19.97500	17.34000
O	10.72600	-20.94400	19.09200
H	11.54600	-20.44900	19.11500
H	11.00000	-21.86100	19.06300
O	20.48300	-28.55300	8.45400
H	19.59600	-28.26800	8.23600
H	20.89900	-28.71300	7.60700
O	23.83500	-28.52800	12.17000
H	24.61100	-28.03900	12.44200
H	23.10900	-28.10200	12.62700
O	26.27200	-27.81500	13.22700
H	26.86600	-28.05600	12.51700
H	26.34400	-28.53500	13.85500
O	8.49200	-13.97200	13.54200
H	8.35700	-14.73200	12.97500
H	8.07500	-13.24800	13.07400
O	19.62700	-18.78800	2.54900
H	20.33100	-18.58300	3.16500
H	18.90400	-18.22100	2.81400
O	20.45700	-23.03700	8.66900
H	20.40100	-22.58000	7.83000
H	21.01000	-23.79800	8.49200
O	24.26800	-27.24800	9.49400
H	24.01100	-27.64900	10.32500
H	23.64300	-26.53400	9.36900
O	28.57900	-24.50900	12.00200
H	28.01900	-24.43900	12.77500
H	27.97300	-24.68500	11.28100
O	11.64800	-13.07500	13.75300
H	11.53200	-12.13100	13.65100
H	10.82800	-13.37800	14.14200
O	21.53600	-18.35000	4.59900
H	22.37100	-18.78200	4.41700

H	21.37500	-18.52200	5.52700
O	21.19100	-21.84700	5.96500
H	21.13600	-22.01200	5.02300
H	20.56500	-21.13900	6.12100
O	5.98000	-26.61000	19.79100
H	6.62600	-27.12000	20.28000
H	5.77200	-25.87300	20.36500
O	18.50500	-19.54200	25.29000
H	19.37400	-19.36800	24.92900
H	18.02600	-18.72300	25.16800
O	14.50000	-32.34600	5.54300
H	14.48800	-31.40200	5.70000
H	15.39300	-32.52900	5.24900
O	12.67400	-12.42200	16.96900
H	13.57700	-12.39000	17.28600
H	12.44800	-13.35200	16.98400
O	14.42900	-13.66900	13.63000
H	13.47900	-13.66900	13.51600
H	14.73300	-14.41600	13.11400
O	18.31600	-35.75200	9.79800
H	17.54900	-36.30600	9.94300
H	18.72700	-35.68000	10.65900
O	10.32000	-29.46300	23.88800
H	11.18300	-29.82000	24.09900
H	9.74600	-29.80200	24.57600
O	30.16500	-7.83300	14.69500
H	30.80200	-7.95200	13.99100
H	30.04700	-6.88500	14.75300
O	26.97400	-7.88000	14.63100
H	26.63500	-7.99600	13.74300
H	27.84200	-8.28100	14.61100
O	22.83200	-6.02700	10.62000
H	22.26900	-6.11800	9.85200
H	22.71700	-6.84700	11.10100
O	27.45500	-6.72700	20.58900
H	27.44400	-7.12200	19.71700
H	27.61800	-7.46000	21.18300
O	20.37900	-4.53200	13.45900
H	21.17100	-4.87300	13.04300
H	20.61800	-4.42200	14.37900
O	28.41200	-2.36000	5.93200
H	27.76100	-1.77400	5.54500
H	29.20700	-1.82900	5.99100
O	24.17600	-3.94300	19.53700
H	23.88500	-3.03900	19.42200
H	24.81200	-3.90200	20.25200
O	25.10200	-6.56500	16.12700
H	25.69800	-6.79000	15.41300
H	25.15900	-7.30700	16.73000
O	24.67400	-9.11200	3.96200
H	24.92700	-9.63300	3.20100
H	23.73700	-9.27500	4.06500
O	29.71900	-10.10800	11.77000
H	30.50500	-10.52600	11.41900
H	29.98300	-9.20300	11.93800
O	29.89000	-4.67100	6.46900
H	29.25400	-4.59700	7.18100
H	29.55900	-4.08100	5.79100
O	35.12000	-10.17000	12.67300
H	35.85200	-10.74100	12.90700
H	34.36300	-10.55800	13.11400
O	24.39400	-7.20900	6.16500
H	24.27800	-7.88400	6.83300
H	24.50400	-7.69500	5.34800
O	26.73700	-7.74800	9.86900
H	26.37300	-8.27500	10.58100
H	27.59400	-8.13800	9.69600
O	32.64200	-16.45800	17.13300
H	32.31000	-15.58900	16.90900
H	32.04700	-16.77500	17.81200
O	26.37800	-12.71900	4.86600
H	26.54500	-11.85100	5.23300
H	26.18700	-12.55600	3.94200
O	29.13500	-4.25700	16.53200
H	28.83500	-3.35500	16.42400
H	28.79000	-4.52400	17.38400
O	22.58600	-7.80600	12.56600
H	21.66200	-7.93800	12.77400
H	22.83500	-7.02600	13.06300
O	32.53500	-11.73900	14.21200
H	31.62700	-11.80700	13.91800
H	32.48400	-11.26200	15.04000
O	26.35700	-10.26300	5.85000
H	26.23700	-9.81700	6.68900
H	25.66200	-9.91400	5.29200
O	19.94700	-8.16200	13.40000
H	20.07700	-7.90900	14.31400
H	19.26700	-7.57000	13.08100
O	24.11700	2.50700	9.49900
H	24.04000	3.45800	9.57800
H	23.51300	2.16200	10.15700
O	26.60900	-10.32000	22.15500
H	25.83200	-9.76000	22.14600
H	27.24800	-9.83400	22.67700
O	34.40100	-13.53700	15.99000
H	33.75900	-13.24400	15.34400
H	33.89700	-13.65200	16.79500
O	32.05500	-9.79100	6.67800
H	32.96700	-9.55800	6.50300
H	31.54600	-9.20700	6.11600
O	24.86400	-7.51100	21.71700
H	25.60500	-6.97300	21.44100
H	24.15700	-6.88500	21.87400
O	30.33900	-5.36800	20.31400
H	30.74800	-5.81000	19.57000
H	29.61700	-5.94300	20.56800
O	26.69200	-10.43100	17.68800
H	27.53800	-10.00700	17.83100
H	26.63200	-11.08800	18.38200
O	30.66200	-4.80800	14.07000
H	30.03300	-4.64400	13.36700
H	30.27500	-4.39400	14.84100
O	34.54100	-3.87300	11.42400
H	33.85600	-3.49300	10.87400
H	34.62500	-3.26300	12.15700
O	29.84200	-12.77100	19.34600
H	29.59800	-12.01600	18.80900
H	30.11800	-12.39200	20.18100

O	31.79600	-6.22700	18.02400
H	32.00800	-5.58400	17.34700
H	31.74900	-7.06100	17.55600
O	29.39900	-13.61500	14.92700
H	28.53700	-13.31700	15.21900
H	29.42500	-13.39800	13.99500
O	26.21500	-5.07100	11.33500
H	26.67900	-4.79200	10.54500
H	25.95100	-5.97200	11.15200
O	23.10700	-5.52400	14.27300
H	23.67700	-5.09500	13.63400
H	23.69800	-5.80500	14.97100
O	25.15200	-8.94800	8.14400
H	25.85400	-8.49600	8.61100
H	24.82000	-9.59000	8.77200
O	23.60000	-10.35700	22.51900
H	22.97000	-9.67600	22.75200
H	23.49300	-10.46900	21.57400
O	18.89400	-10.69000	13.44500
H	18.10400	-10.43400	12.96900
H	19.38500	-9.87500	13.55100
O	32.91100	-3.35000	16.19600
H	33.73200	-3.75200	15.91400
H	32.90700	-3.45300	17.14700
O	20.84600	-10.32600	10.40400
H	20.04900	-10.21400	10.92200
H	20.61800	-9.98700	9.53800
O	26.67600	-3.80100	18.35200
H	26.74100	-3.33900	19.18800
H	25.96700	-4.43100	18.48000
O	17.17800	-10.43700	15.87700
H	17.42900	-9.55200	16.14300
H	17.71400	-10.61700	15.10400
O	41.00100	-7.00600	12.54400
H	40.24800	-7.57500	12.38900
H	41.13600	-7.03900	13.49100
O	26.39100	-2.13400	8.22000
H	26.25600	-1.28900	8.64800
H	26.93200	-1.93200	7.45600
O	22.87800	-16.05500	24.88100
H	22.48500	-16.60900	25.55600
H	23.69400	-15.74400	25.27200
O	19.84400	-4.61700	8.79300
H	20.35700	-3.82300	8.94300
H	20.49600	-5.31400	8.72600
O	21.65400	-1.50000	10.87500
H	21.83000	-0.57800	10.68700
H	22.44900	-1.81400	11.30400
O	25.79500	-14.63900	22.47300
H	26.53600	-14.03900	22.39400
H	26.02600	-15.38200	21.91500
O	20.93900	-9.45400	20.75100
H	20.82400	-10.35500	21.05200
H	20.08300	-9.20700	20.40000
O	28.01800	-1.51200	22.35000
H	27.57400	-2.29800	22.03200
H	28.68000	-1.32400	21.68400
O	22.77700	0.01800	16.87600
H	23.32700	-0.11100	16.10400
H	21.93800	0.31700	16.52500
O	30.11600	-1.23200	20.37800
H	30.09800	-0.41100	19.88600
H	31.03800	-1.35600	20.60300
O	26.57300	-3.71700	21.22000
H	27.10600	-4.47900	20.99000
H	26.04500	-4.01000	21.96300
O	29.74000	-18.61100	16.75000
H	29.39700	-17.72100	16.67400
H	30.10800	-18.80100	15.88700
O	17.80900	-6.28800	9.56900
H	18.64600	-5.87800	9.35200
H	17.98700	-7.22800	9.53900
O	29.70500	-8.62000	5.25700
H	29.19000	-7.85000	5.49600
H	29.43300	-9.29400	5.88000
O	27.29000	-13.77500	10.31600
H	26.43400	-14.16500	10.13600
H	27.70300	-13.69600	9.45500
O	28.82000	-16.24100	14.71500
H	28.31700	-16.27800	13.90100
H	29.11300	-15.33200	14.77400
O	25.06400	-16.15700	13.72100
H	25.93000	-16.35300	13.36200
H	25.13200	-16.38600	14.64800
O	34.67900	-5.86700	7.19200
H	33.90200	-6.20600	6.74800
H	35.35800	-5.86200	6.51800
O	29.49400	-10.74800	7.08300
H	29.18900	-10.43300	7.93400
H	30.44700	-10.67000	7.12600
O	27.93900	-4.58900	8.73200
H	27.41100	-5.32300	8.42000
H	27.43700	-3.81100	8.48600
O	32.91800	-6.39400	13.84900
H	32.26000	-5.70400	13.93700
H	33.72200	-6.00400	14.19200
O	22.67100	-13.92700	11.09300
H	23.24500	-14.14900	10.36000
H	23.25800	-13.85800	11.84600
O	31.72900	-8.03900	11.89900
H	32.24200	-7.40600	12.40200
H	32.37900	-8.63900	11.53400
O	22.99800	-11.89600	5.11300
H	22.16100	-11.55900	5.43300
H	22.85800	-12.03100	4.17500
O	24.97300	-17.05300	9.82000
H	24.31900	-17.36800	9.19600
H	24.93100	-17.67500	10.54600
O	26.08400	-6.77100	1.73600
H	25.17700	-7.00200	1.93500
H	26.58100	-7.56700	1.92300
O	28.99100	1.93900	4.65500
H	28.31700	2.24200	5.26400
H	29.37700	2.74200	4.30600
O	28.20700	-10.56400	14.14500
H	27.28900	-10.34300	13.98700
H	28.62300	-10.49000	13.28600
O	28.99000	-9.16500	9.27500

H	29.73700	-8.65300	8.96600
H	29.25900	-9.48700	10.13600
O	27.26300	-6.66900	6.25800
H	26.35400	-6.95400	6.35500
H	27.29200	-6.25000	5.39800
O	32.49500	-3.05900	10.02700
H	32.04100	-3.90000	10.08400
H	31.86400	-2.42300	10.36500
O	31.43800	-12.13000	11.25700
H	30.70000	-12.64200	11.58800
H	31.69100	-12.57000	10.44600
O	30.93800	-1.22100	10.96200
H	31.23000	-0.66100	10.24300
H	31.44800	-0.92800	11.71800
O	31.53900	-19.35200	11.85200
H	31.84500	-20.21200	11.56400
H	32.26400	-18.76000	11.65100
O	17.83200	-8.94900	10.24000
H	17.63400	-9.54200	9.51500
H	17.27600	-9.25400	10.95700
O	25.81100	-9.05700	12.35900
H	25.86600	-10.00900	12.28300
H	25.00000	-8.90100	12.84300
O	26.96000	-5.12900	3.59700
H	26.52200	-5.84500	3.13800
H	27.70100	-4.90100	3.03500
O	23.75000	-10.43900	10.11800
H	22.93700	-10.13900	10.52500
H	24.24600	-10.83300	10.83600
O	25.90100	-0.44300	12.05200
H	25.85900	-0.21700	11.12200
H	26.81800	-0.67700	12.20000
O	29.34800	-9.97500	18.95000
H	29.70300	-9.74800	19.80900
H	29.99500	-9.64300	18.32700
O	35.07800	-6.85300	9.65500
H	34.93900	-6.66300	8.72700
H	34.72100	-6.09100	10.11100
O	23.24200	-12.86700	15.73400
H	23.88200	-13.00800	15.03700
H	22.41000	-13.15400	15.35800
O	35.10500	-4.78500	14.66600
H	35.53800	-4.87800	15.51400
H	35.81800	-4.80900	14.02800
O	24.01800	-3.56700	11.84800
H	24.81200	-4.06200	11.64600
H	23.30700	-4.09500	11.48500
O	28.83700	-1.18500	16.19900
H	28.89100	-0.61600	16.96700
H	27.96900	-1.01500	15.83400
O	20.32600	-9.42300	7.92400
H	19.39600	-9.48400	8.14100
H	20.42200	-9.94500	7.12700
O	20.24500	-12.89700	10.51200
H	20.50500	-11.98200	10.40300
H	21.06600	-13.36500	10.66100
O	21.71700	-11.48300	17.68100
H	22.39000	-11.69200	17.03300
H	20.89000	-11.68100	17.24200
O	35.96600	-4.74300	17.19900
H	36.16000	-3.83800	17.44500
H	35.86600	-5.20200	18.03300
O	27.62000	-14.85700	19.69800
H	26.97300	-14.15800	19.60400
H	28.39000	-14.42300	20.06600
O	31.02800	-4.59200	22.77300
H	30.87600	-4.82100	21.85600
H	31.01800	-3.63500	22.78600
O	27.79600	-7.01900	17.75800
H	28.72500	-6.86800	17.58500
H	27.37500	-6.93100	16.90300
O	26.59500	-2.99400	15.52800
H	26.45600	-3.36200	16.40000
H	25.80700	-2.47900	15.35600
O	27.19000	2.10700	6.87000
H	27.36500	1.63600	7.68500
H	26.50300	2.73400	7.09800
O	28.14600	-3.61600	12.91600
H	27.81100	-3.65600	13.81200
H	27.64600	-4.28100	12.44300
O	22.12000	-8.20900	18.44200
H	21.98200	-8.94100	19.04300
H	23.03000	-8.29800	18.16000
O	31.08100	-6.27000	3.19000
H	30.46400	-6.99500	3.09300
H	30.61300	-5.50900	2.84700
O	24.74800	-8.49700	17.93000
H	25.45800	-9.13800	17.88900
H	24.70500	-8.24600	18.85300
O	32.73700	-1.56000	20.85100
H	32.67800	-2.24400	20.18400
H	33.13900	-0.81800	20.39900
O	23.46100	-11.51000	19.75000
H	22.92500	-11.96100	20.40200
H	22.87300	-11.37600	19.00700
O	21.75600	5.25600	13.64400
H	21.99600	5.88800	14.32200
H	20.91300	4.90500	13.93100
O	22.87100	-12.05200	2.31200
H	21.99400	-12.17300	1.94900
H	23.34700	-12.84300	2.06000
O	32.73900	-3.59600	19.08200
H	31.85100	-3.93400	19.20500
H	33.30800	-4.32800	19.31900
O	14.39200	-7.26400	12.40600
H	15.07900	-7.57800	11.81900
H	14.23600	-6.36000	12.13300
O	19.19500	-12.37500	16.83500
H	19.11100	-12.98200	16.10000
H	18.37300	-11.88400	16.83600
O	26.26900	1.06400	16.04800
H	26.19600	1.86900	16.56000
H	25.82600	1.26300	15.22300
O	26.05800	0.30100	9.55900
H	25.27400	0.84300	9.65200
H	26.75700	0.81800	9.95800
O	29.36700	-13.89000	12.20200
H	29.86400	-14.64300	11.88200

H	28.59600	-13.85300	11.63600
O	37.22000	-8.18500	13.84400
H	37.20900	-7.30300	13.47300
H	36.35200	-8.53800	13.64900
O	28.72900	0.96300	18.20400
H	29.44100	1.59700	18.13200
H	28.23800	1.05900	17.38800
O	30.45000	-5.13200	9.77600
H	29.51600	-5.01200	9.60600
H	30.62800	-6.03500	9.51200
O	25.95100	-11.74800	11.92900
H	25.58100	-12.30100	12.61700
H	26.44700	-12.35200	11.37700
O	33.83800	-9.29100	10.31100
H	34.30400	-8.45800	10.23700
H	34.28800	-9.75600	11.01500
O	23.78000	-1.28800	19.02500
H	24.48100	-0.65400	19.17800
H	23.30900	-0.94700	18.26400
O	31.90500	-2.94600	7.30900
H	32.18100	-3.06100	8.21900
H	31.03600	-3.34400	7.27100
O	31.80900	-16.92300	14.35000
H	30.89600	-16.69200	14.51800
H	32.23900	-16.83900	15.20100
O	31.58000	-8.73400	16.85000
H	30.92700	-8.48600	16.19500
H	31.95800	-9.54700	16.51600
O	28.54100	1.30200	10.95300
H	28.72300	0.48500	11.41500
H	29.36400	1.51900	10.51500
O	28.87500	0.22900	8.21400
H	29.40400	-0.28200	7.60100
H	29.13800	-0.08700	9.07900
O	27.62900	-7.99200	23.33600
H	26.95300	-8.09200	24.00700
H	28.45100	-8.14800	23.80000
O	19.37700	-1.75400	15.95800
H	19.96200	-2.50000	16.09100
H	19.17900	-1.44400	16.84100
O	22.23000	1.28100	10.97400
H	21.56100	1.76500	10.49000
H	22.09200	1.52800	11.88800
O	13.96800	-9.18400	14.67800
H	14.00700	-9.71700	15.47200
H	13.46200	-9.71000	14.05900
O	26.37100	3.31500	17.57200
H	26.92300	3.47800	18.33700
H	26.71200	3.90600	16.90000
O	21.34700	-14.33700	18.17400
H	21.41200	-13.47600	17.76200
H	21.28400	-14.95000	17.44200
O	20.82300	-12.26500	21.82200
H	21.45000	-12.64700	22.43600
H	19.96500	-12.48400	22.18700
O	31.22200	-1.85900	23.42700
H	31.76600	-1.24600	22.93300
H	30.64100	-1.30100	23.94400
O	17.59900	-10.45700	7.75400
H	17.89500	-11.36600	7.70000
H	16.69800	-10.47900	7.43200
O	29.50800	1.26300	14.55900
H	29.38300	0.46200	14.05000
H	30.16600	1.75800	14.07000
O	21.12900	-12.38200	13.73600
H	20.40700	-11.76800	13.59700
H	21.91800	-11.84600	13.65800
O	31.57100	-0.93600	16.18700
H	30.62400	-1.05500	16.11400
H	31.92000	-1.82400	16.26900
O	23.93100	-15.04600	20.01000
H	24.45500	-14.80000	20.77300
H	23.71200	-14.21400	19.59100
O	19.32800	-12.77600	7.87800
H	18.99900	-13.65600	7.69500
H	19.68200	-12.83100	8.76500
O	28.57600	-1.14100	12.38700
H	28.42800	-2.06900	12.56800
H	29.40100	-1.11900	11.90300
O	22.44200	-12.48900	8.24500
H	22.78200	-11.96000	7.52300
H	22.88900	-12.15100	9.02100
O	32.17900	-0.31800	13.47400
H	33.10400	-0.52300	13.33600
H	32.01100	-0.56600	14.38200
O	25.13700	-14.10500	17.69500
H	24.79600	-14.90400	18.09700
H	24.43000	-13.80500	17.12300
O	25.78800	-17.02000	16.14800
H	26.61100	-17.49400	16.02600
H	26.03200	-16.24100	16.64800
O	24.42900	-13.99100	6.44400
H	25.15000	-13.61300	5.94100
H	23.80600	-13.27200	6.54800
O	28.57200	-13.28700	7.38000
H	28.78800	-12.40400	7.07900
H	28.09200	-13.67800	6.65100
O	23.31200	-10.32800	13.95700
H	23.16600	-10.01400	14.85000
H	22.83500	-9.71000	13.40400
O	25.17200	-13.33800	14.00500
H	25.15800	-14.23400	13.66900
H	25.88900	-13.33100	14.63900
O	27.02400	-12.90400	16.05100
H	26.76600	-11.98200	16.04900
H	26.49300	-13.30100	16.74100
O	31.96900	0.20100	8.83000
H	32.14300	-0.36400	8.07700
H	32.46200	1.00100	8.64800
O	17.78700	-7.78800	16.89600
H	17.24200	-7.01200	17.02800
H	18.66500	-7.44100	16.73800
O	24.57500	-14.45600	9.27400
H	24.69700	-15.38500	9.47300
H	24.28600	-14.44300	8.36200
O	27.44400	-16.77100	12.41100
H	27.46700	-16.70700	11.45600
H	27.86100	-17.61100	12.60500

O	37.82300	-7.03500	10.44900
H	38.26600	-7.78500	10.05100
H	36.94500	-7.04400	10.06600
O	32.95600	-0.48200	5.92900
H	33.65200	0.13800	6.14800
H	33.29900	-1.33300	6.20500
O	18.57300	-8.66100	19.78900
H	17.83100	-8.51200	20.37600
H	18.25200	-8.40000	18.92600
O	34.85600	-3.03200	7.45800
H	35.38100	-2.69400	6.73300
H	34.76800	-3.96800	7.27400
O	26.51800	0.88100	19.58700
H	26.82600	0.63600	20.45900
H	27.31500	1.08600	19.09900
O	26.54000	3.03500	11.81800
H	26.10000	2.54200	12.51100
H	27.29000	2.49200	11.57700
O	19.01900	-0.77400	11.82000
H	19.84500	-1.07400	11.44000
H	18.66800	-1.54300	12.26900
O	23.98700	-1.42100	13.89000
H	23.36500	-2.08400	13.59000
H	24.65700	-1.39500	13.20700
O	17.22000	-2.85500	12.77900
H	16.38300	-3.28300	12.59700
H	17.19200	-2.66200	13.71600
O	24.05900	-12.94300	23.48300
H	24.10800	-12.06700	23.10100
H	24.60200	-13.48600	22.91000
O	34.54500	-2.39900	22.76300
H	33.75500	-2.41700	22.22400
H	34.30800	-1.86000	23.51800
O	34.66500	-5.55000	20.07700
H	34.71500	-5.71500	21.01800
H	34.55600	-6.41700	19.68600
O	31.95500	-9.56700	21.39200
H	32.85600	-9.70600	21.10100
H	32.03000	-9.40100	22.33200
O	26.28900	-12.19000	20.23800
H	25.42000	-11.89700	19.96600
H	26.52500	-11.60600	20.95900
O	36.16700	-3.13000	20.32100
H	35.87500	-2.76700	21.15700
H	35.55400	-3.84400	20.14200
O	37.36600	-5.56500	12.84100
H	38.20100	-5.12600	13.00500
H	37.50200	-6.04100	12.02200
O	34.41500	-8.00700	18.78900
H	34.89100	-7.81400	17.98100
H	33.49200	-7.95100	18.54300
O	21.30400	-6.84500	8.37200
H	21.05800	-7.74800	8.57000
H	21.47300	-6.84300	7.43000
O	28.99300	-15.97200	17.75400
H	29.51100	-15.23600	17.42900
H	28.63900	-15.66600	18.58900
O	20.57300	-11.11200	5.87000
H	19.88000	-11.03700	5.21400
H	20.32800	-11.87600	6.39300
O	29.24500	-19.55200	13.27400
H	29.52500	-20.22000	13.90000
H	30.03000	-19.35900	12.76200
O	27.04800	-0.37600	4.83700
H	26.47500	0.24300	4.38400
H	27.62100	0.17400	5.37100
O	33.11500	-11.13000	17.02200
H	34.06700	-11.03100	17.04300
H	32.94600	-11.92300	17.53100
O	31.13700	-14.04500	17.20800
H	30.54200	-13.76400	16.51200
H	30.85200	-13.55500	17.97900
O	30.26800	-0.09300	5.96800
H	31.20100	-0.15800	6.17100
H	30.18000	0.74300	5.50900
O	32.89600	-0.77600	2.92800
H	32.81800	-1.11000	3.82200
H	33.71700	-0.28400	2.92700
O	35.59400	-7.91000	16.13600
H	35.56200	-8.84800	16.32200
H	36.20400	-7.83100	15.40200
O	27.43300	-9.04000	2.17900
H	28.37800	-9.17000	2.24700
H	27.05400	-9.77900	2.65500
O	35.74400	-11.11900	16.18200
H	35.22800	-11.90300	15.99500
H	36.62800	-11.44700	16.34600
O	33.40500	-17.37500	12.14100
H	33.20700	-17.68300	13.02600
H	34.01500	-16.64800	12.27100
O	41.41200	-6.84800	15.27600
H	42.14200	-7.11000	15.83700
H	40.80300	-7.58500	15.31700
O	39.49300	-8.70700	15.87400
H	38.76700	-8.84200	15.26500
H	39.07800	-8.40000	16.68000
O	27.31600	-4.98800	23.63900
H	28.23700	-4.81100	23.83300
H	27.28100	-5.93300	23.48700
O	25.61700	-11.11500	2.11400
H	24.71000	-11.41200	2.19000
H	25.89400	-11.41200	1.24700
O	16.53500	-9.79500	12.56400
H	16.21500	-10.64800	12.27100
H	15.98800	-9.58000	13.32000
O	20.31300	-7.07800	16.28100
H	20.53100	-6.14700	16.30000
H	20.72500	-7.43700	17.06600
O	27.23300	-16.57100	8.26800
H	26.42300	-16.91700	8.64400
H	27.92200	-16.88100	8.85700
O	29.16200	-17.49400	9.89100
H	29.71500	-17.07400	10.55000
H	29.39900	-18.42000	9.93300
O	25.18500	-11.50500	25.67100
H	25.08500	-10.55600	25.60200
H	24.96800	-11.83300	24.79900
O	24.38700	-14.25600	2.25500

H	25.24900	-13.93100	2.51500
H	24.57200	-15.04300	1.74300
O	16.40300	-6.17400	14.53300
H	15.71000	-6.58900	14.02000
H	17.15100	-6.13200	13.93700
O	32.54000	-15.38400	9.39900
H	31.96000	-14.72500	9.01700
H	32.27400	-16.20600	8.98700
O	30.99000	-15.98300	11.60100
H	31.49000	-15.87100	10.79200
H	31.63400	-16.28700	12.24100
O	25.04900	-8.07500	24.48800
H	24.56500	-7.27500	24.69400
H	25.13500	-8.06400	23.53400
O	18.31700	-6.24200	12.41000
H	18.29400	-6.14400	11.45800
H	18.62700	-5.39600	12.73200
O	20.97600	-4.18500	16.52400
H	21.93000	-4.21600	16.45700
H	20.77700	-4.64600	17.34000
O	21.56600	-5.61500	19.09200
H	22.38500	-5.12000	19.11500
H	21.84000	-6.53100	19.06300
O	31.32300	-13.22400	8.45400
H	30.43500	-12.93900	8.23600
H	31.73800	-13.38400	7.60700
O	34.67400	-13.19900	12.17000
H	35.45000	-12.70900	12.44200
H	33.94900	-12.77300	12.62700
O	37.11100	-12.48600	13.22700
H	37.70600	-12.72700	12.51700
H	37.18400	-13.20500	13.85500
O	19.33100	1.35700	13.54200
H	19.19600	0.59700	12.97500
H	18.91400	2.08100	13.07400
O	30.46700	-3.45900	2.54900
H	31.17000	-3.25400	3.16500
H	29.74300	-2.89100	2.81400
O	31.29700	-7.70800	8.66900
H	31.24000	-7.25100	7.83000
H	31.84900	-8.46900	8.49200
O	35.10700	-11.91900	9.49400
H	34.85100	-12.32000	10.32500
H	34.48200	-11.20500	9.36900
O	39.41800	-9.18000	12.00200
H	38.85800	-9.11000	12.77500
H	38.81300	-9.35500	11.28100
O	22.48800	2.25400	13.75300
H	22.37100	3.19900	13.65100
H	21.66700	1.95100	14.14200
O	32.37500	-3.02100	4.59900
H	33.21000	-3.45300	4.41700
H	32.21400	-3.19300	5.52700
O	32.03000	-6.51800	5.96500
H	31.97500	-6.68300	5.02300
H	31.40500	-5.81000	6.12100
O	16.81900	-11.28100	19.79100
H	17.46500	-11.79100	20.28000
H	16.61100	-10.54400	20.36500
O	29.34500	-4.21300	25.29000
H	30.21400	-4.03900	24.92900
H	28.86500	-3.39300	25.16800
O	25.34000	-17.01700	5.54300
H	25.32800	-16.07300	5.70000
H	26.23200	-17.20000	5.24900
O	23.51300	2.90700	16.96900
H	24.41600	2.93900	17.28600
H	23.28700	1.97700	16.98400
O	25.26900	1.66000	13.63000
H	24.31800	1.66000	13.51600
H	25.57200	0.91300	13.11400
O	29.15500	-20.42300	9.79800
H	28.38800	-20.97700	9.94300
H	29.56600	-20.35000	10.65900
O	21.15900	-14.13400	23.88800
H	22.02200	-14.49000	24.09900
H	20.58600	-14.47300	24.57600
O	41.00400	7.49600	14.69500
H	41.64200	7.37700	13.99100
H	40.88600	8.44400	14.75300
O	37.81300	7.44900	14.63100
H	37.47500	7.33300	13.74300
H	38.68200	7.04800	14.61100
O	33.67200	9.30200	10.62000
H	33.10800	9.21100	9.85200
H	33.55700	8.48200	11.10100
O	38.29500	8.60200	20.58900
H	38.28400	8.20800	19.71700
H	38.45700	7.86900	21.18300
O	31.21800	10.79700	13.45900
H	32.01000	10.45700	13.04300
H	31.45800	10.90700	14.37900
O	39.25200	12.96900	5.93200
H	38.60000	13.55500	5.54500
H	40.04600	13.50000	5.99100
O	33.17700	10.05800	6.33100
H	33.95000	9.51100	6.19200
H	33.53000	10.92300	6.54200
O	35.01600	11.38600	19.53700
H	34.72500	12.29100	19.42200
H	35.65100	11.42800	20.25200
O	35.94100	8.76400	16.12700
H	36.53800	8.53900	15.41300
H	35.99900	8.02200	16.73000
O	35.51400	6.21800	3.96200
H	35.76600	5.69600	3.20100
H	34.57600	6.05400	4.06500
O	40.55800	5.22100	11.77000
H	41.34400	4.80300	11.41900
H	40.82200	6.12600	11.93800
O	40.72900	10.65900	6.46900
H	40.09400	10.73200	7.18100
H	40.39900	11.24800	5.79100
O	35.23300	8.12000	6.16500
H	35.11700	7.44500	6.83300
H	35.34400	7.63400	5.34800
O	37.57700	7.58100	9.86900
H	37.21200	7.05500	10.58100

H	38.43300	7.19100	9.69600
O	37.21700	2.61000	4.86600
H	37.38400	3.47800	5.23300
H	37.02700	2.77300	3.94200
O	39.97500	11.07200	16.53200
H	39.67500	11.97500	16.42400
H	39.63000	10.80500	17.38400
O	33.42600	7.52400	12.56600
H	32.50100	7.39100	12.77400
H	33.67400	8.30300	13.06300
O	37.19600	5.06600	5.85000
H	37.07600	5.51200	6.68900
H	36.50100	5.41500	5.29200
O	30.78700	7.16700	13.40000
H	30.91600	7.42000	14.31400
H	30.10600	7.76000	13.08100
O	34.95700	17.83700	9.49900
H	34.87900	18.78700	9.57800
H	34.35300	17.49100	10.15700
O	37.44800	5.00900	22.15500
H	36.67200	5.56900	22.14600
H	38.08700	5.49500	22.67700
O	35.70300	7.81800	21.71700
H	36.44400	8.35600	21.44100
H	34.99700	8.44500	21.87400
O	41.17900	9.96100	20.31400
H	41.58700	9.51900	19.57000
H	40.45600	9.38600	20.56800
O	37.53200	4.89800	17.68800
H	38.37800	5.32200	17.83100
H	37.47200	4.24100	18.38200
O	41.50100	10.52100	14.07000
H	40.87200	10.68600	13.36700
H	41.11400	10.93500	14.84100
O	40.68200	2.55900	19.34600
H	40.43800	3.31300	18.80900
H	40.95800	2.93700	20.18100
O	42.63500	9.10200	18.02400
H	42.84700	9.74600	17.34700
H	42.58800	8.26900	17.55600
O	40.23800	1.71400	14.92700
H	39.37700	2.01200	15.21900
H	40.26400	1.93100	13.99500
O	37.05400	10.25800	11.33500
H	37.51800	10.53700	10.54500
H	36.79000	9.35700	11.15200
O	33.94700	9.80600	14.27300
H	34.51700	10.23400	13.63400
H	34.53700	9.52400	14.97100
O	35.99100	6.38100	8.14400
H	36.69400	6.83300	8.61100
H	35.65900	5.73900	8.77200
O	34.43900	4.97200	22.51900
H	33.80900	5.65400	22.75200
H	34.33300	4.86000	21.57400
O	29.73400	4.63900	13.44500
H	28.94400	4.89500	12.96900
H	30.22500	5.45400	13.55100
O	31.68500	5.00400	10.40400
H	30.88800	5.11500	10.92200
H	31.45800	5.34200	9.53800
O	37.51600	11.52800	18.35200
H	37.58000	11.99000	19.18800
H	36.80700	10.89800	18.48000
O	28.01700	4.89200	15.87700
H	28.26800	5.77700	16.14300
H	28.55300	4.71300	15.10400
O	37.23000	13.19500	8.22000
H	37.09500	14.04000	8.64800
H	37.77100	13.39700	7.45600
O	33.71700	-0.72600	24.88100
H	33.32400	-1.27900	25.55600
H	34.53400	-0.41500	25.27200
O	30.68300	10.71200	8.79300
H	31.19600	11.50700	8.94300
H	31.33600	10.01500	8.72600
O	32.49300	13.82900	10.87500
H	32.67000	14.75100	10.68700
H	33.28900	13.51500	11.30400
O	36.63400	0.69000	22.47300
H	37.37500	1.29000	22.39400
H	36.86600	-0.05200	21.91500
O	31.77800	5.87500	20.75100
H	31.66400	4.97400	21.05200
H	30.92300	6.12200	20.40000
O	38.85700	13.81700	22.35000
H	38.41400	13.03100	22.03200
H	39.51900	14.00500	21.68400
O	33.61600	15.34700	16.87600
H	34.16700	15.21800	16.10400
H	32.77700	15.64600	16.52500
O	40.95500	14.09700	20.37800
H	40.93700	14.91800	19.88600
H	41.87700	13.97300	20.60300
O	37.41300	11.61200	21.22000
H	37.94600	10.85100	20.99000
H	36.88400	11.31900	21.96300
O	40.58000	-3.28200	16.75000
H	40.23600	-2.39200	16.67400
H	40.94800	-3.47200	15.88700
O	28.64800	9.04100	9.56900
H	29.48600	9.45100	9.35200
H	28.82600	8.10100	9.53900
O	40.54500	6.70900	5.25700
H	40.02900	7.47900	5.49600
H	40.27300	6.03500	5.88000
O	38.12900	1.55400	10.31600
H	37.27400	1.16400	10.13600
H	38.54200	1.63300	9.45500
O	39.65900	-0.91200	14.71500
H	39.15600	-0.94900	13.90100
H	39.95200	-0.00200	14.77400
O	35.90400	-0.82800	13.72100
H	36.76900	-1.02400	13.36200
H	35.97100	-1.05700	14.64800
O	40.33300	4.58100	7.08300
H	40.02800	4.89600	7.93400
H	41.28600	4.66000	7.12600

O	38.77800	10.74000	8.73200
H	38.25000	10.00600	8.42000
H	38.27700	11.51800	8.48600
O	33.51000	1.40200	11.09300
H	34.08400	1.18000	10.36000
H	34.09700	1.47100	11.84600
O	33.83700	3.43300	5.11300
H	33.00100	3.77000	5.43300
H	33.69700	3.29800	4.17500
O	35.81200	-1.72300	9.82000
H	35.15900	-2.03900	9.19600
H	35.77000	-2.34600	10.54600
O	36.92300	8.55800	1.73600
H	36.01600	8.32700	1.93500
H	37.42000	7.76200	1.92300
O	39.83000	17.26800	4.65500
H	39.15600	17.57100	5.26400
H	40.21700	18.07100	4.30600
O	39.04600	4.76500	14.14500
H	38.12800	4.98600	13.98700
H	39.46200	4.83900	13.28600
O	39.82900	6.16400	9.27500
H	40.57700	6.67600	8.96600
H	40.09900	5.84200	10.13600
O	38.10200	8.66000	6.25800
H	37.19300	8.37600	6.35500
H	38.13200	9.08000	5.39800
O	42.27700	3.20000	11.25700
H	41.54000	2.68700	11.58800
H	42.53000	2.76000	10.44600
O	41.77700	14.10800	10.96200
H	42.07000	14.66900	10.24300
H	42.28700	14.40100	11.71800
O	28.67100	6.38000	10.24000
H	28.47300	5.78700	9.51500
H	28.11600	6.07600	10.95700
O	36.65100	6.27300	12.35900
H	36.70500	5.32000	12.28300
H	35.84000	6.42900	12.84300
O	37.80000	10.20100	3.59700
H	37.36200	9.48400	3.13800
H	38.54100	10.42800	3.03500
O	34.59000	4.89000	10.11800
H	33.77700	5.19000	10.52500
H	35.08600	4.49600	10.83600
O	36.74100	14.88600	12.05200
H	36.69800	15.11200	11.12200
H	37.65700	14.65200	12.20000
O	40.18700	5.35400	18.95000
H	40.54200	5.58200	19.80900
H	40.83400	5.68600	18.32700
O	34.08100	2.46200	15.73400
H	34.72100	2.32100	15.03700
H	33.24900	2.17600	15.35800
O	34.85700	11.76200	11.84800
H	35.65100	11.26700	11.64600
H	34.14600	11.23400	11.48500
O	39.67700	14.14400	16.19900
H	39.73000	14.71300	16.96700
H	38.80800	14.31400	15.83400
O	31.16600	5.90700	7.92400
H	30.23500	5.84500	8.14100
H	31.26100	5.38400	7.12700
O	31.08400	2.43200	10.51200
H	31.34500	3.34700	10.40300
H	31.90600	1.96500	10.66100
O	32.55700	3.84600	17.68100
H	33.22900	3.63800	17.03300
H	31.73000	3.64800	17.24200
O	38.45900	0.47200	19.69800
H	37.81200	1.17100	19.60400
H	39.22900	0.90600	20.06600
O	41.86700	10.73700	22.77300
H	41.71600	10.50800	21.85600
H	41.85800	11.69400	22.78600
O	38.63600	8.31000	17.75800
H	39.56500	8.46100	17.58500
H	38.21500	8.39900	16.90300
O	37.43400	12.33500	15.52800
H	37.29600	11.96700	16.40000
H	36.64600	12.85000	15.35600
O	38.02900	17.43600	6.87000
H	38.20400	16.96500	7.68500
H	37.34300	18.06300	7.09800
O	38.98500	11.71300	12.91600
H	38.65000	11.67400	13.81200
H	38.48500	11.04800	12.44300
O	32.95900	7.12000	18.44200
H	32.82100	6.38800	19.04300
H	33.87000	7.03100	18.16000
O	41.92100	9.05900	3.19000
H	41.30400	8.33400	3.09300
H	41.45300	9.82000	2.84700
O	35.58800	6.83200	17.93000
H	36.29800	6.19200	17.88900
H	35.54500	7.08300	18.85300
O	34.30000	3.81900	19.75000
H	33.76400	3.36800	20.40200
H	33.71200	3.95300	19.00700
O	32.59500	20.58500	13.64400
H	32.83500	21.21700	14.32200
H	31.75200	20.23400	13.93100
O	33.71100	3.27800	2.31200
H	32.83400	3.15600	1.94900
H	34.18600	2.48600	2.06000
O	25.23100	8.06500	12.40600
H	25.91800	7.75100	11.81900
H	25.07600	8.96900	12.13300
O	30.03400	2.95400	16.83500
H	29.95000	2.34700	16.10000
H	29.21300	3.44500	16.83600
O	37.10800	16.39300	16.04800
H	37.03500	17.19900	16.56000
H	36.66600	16.59200	15.22300
O	36.89700	15.63000	9.55900
H	36.11400	16.17200	9.65200
H	37.59600	16.14800	9.95800
O	40.20700	1.44000	12.20200

H	40.70400	0.68700	11.88200
H	39.43500	1.47600	11.63600
O	39.56800	16.29200	18.20400
H	40.28100	16.92600	18.13200
H	39.07700	16.38800	17.38800
O	41.29000	10.19700	9.77600
H	40.35500	10.31700	9.60600
H	41.46700	9.29400	9.51200
O	36.79000	3.58100	11.92900
H	36.42000	3.02900	12.61700
H	37.28600	2.97700	11.37700
O	34.61900	14.04100	19.02500
H	35.32000	14.67500	19.17800
H	34.14900	14.38300	18.26400
O	42.74500	12.38300	7.30900
H	43.02100	12.26900	8.21900
H	41.87500	11.98500	7.27100
O	42.41900	6.59600	16.85000
H	41.76600	6.84300	16.19500
H	42.79700	5.78200	16.51600
O	39.38000	16.63200	10.95300
H	39.56200	15.81400	11.41500
H	40.20300	16.84800	10.51500
O	39.71400	15.55800	8.21400
H	40.24300	15.04800	7.60100
H	39.97700	15.24300	9.07900
O	38.46800	7.33800	23.33600
H	37.79200	7.23700	24.00700
H	39.29100	7.18200	23.80000
O	30.21600	13.57500	15.95800
H	30.80100	12.82900	16.09100
H	30.01800	13.88500	16.84100
O	33.07000	16.61000	10.97400
H	32.40100	17.09500	10.49000
H	32.93100	16.85700	11.88800
O	24.80800	6.14500	14.67800
H	24.84600	5.61200	15.47200
H	24.30200	5.61900	14.05900
O	37.21000	18.64400	17.57200
H	37.76200	18.80700	18.33700
H	37.55100	19.23500	16.90000
O	32.18700	0.99200	18.17400
H	32.25100	1.85300	17.76200
H	32.12400	0.37900	17.44200
O	31.66200	3.06400	21.82200
H	32.28900	2.68200	22.43600
H	30.80500	2.84600	22.18700
O	42.06100	13.47000	23.42700
H	42.60500	14.08300	22.93300
H	41.48000	14.02800	23.94400
O	28.43900	4.87200	7.75400
H	28.73400	3.96300	7.70000
H	27.53700	4.85100	7.43200
O	40.34700	16.59300	14.55900
H	40.22200	15.79100	14.05000
H	41.00500	17.08700	14.07000
O	31.96800	2.94700	13.73600
H	31.24700	3.56100	13.59700
H	32.75700	3.48300	13.65800
O	42.41100	14.39300	16.18700
H	41.46400	14.27400	16.11400
H	42.75900	13.50600	16.26900
O	34.77100	0.28300	20.01000
H	35.29400	0.52900	20.77300
H	34.55200	1.11500	19.59100
O	30.16700	2.55300	7.87800
H	29.83900	1.67300	7.69500
H	30.52100	2.49800	8.76500
O	39.41500	14.18900	12.38700
H	39.26800	13.26000	12.56800
H	40.24100	14.21100	11.90300
O	33.28200	2.84000	8.24500
H	33.62100	3.36900	7.52300
H	33.72900	3.17900	9.02100
O	35.97600	1.22400	17.69500
H	35.63600	0.42500	18.09700
H	35.27000	1.52400	17.12300
O	36.62800	-1.69100	16.14800
H	37.45000	-2.16500	16.02600
H	36.87100	-0.91200	16.64800
O	35.26800	1.33800	6.44400
H	35.98900	1.71600	5.94100
H	34.64500	2.05700	6.54800
O	39.41100	2.04200	7.38000
H	39.62800	2.92500	7.07900
H	38.93100	1.65100	6.65100
O	34.15100	5.00100	13.95700
H	34.00600	5.31500	14.85000
H	33.67400	5.62000	13.40400
O	36.01100	1.99100	14.00500
H	35.99700	1.09500	13.66900
H	36.72800	1.99800	14.63900
O	37.86400	2.42500	16.05100
H	37.60500	3.34700	16.04900
H	37.33200	2.02800	16.74100
O	28.62600	7.54200	16.89600
H	28.08100	8.31800	17.02800
H	29.50400	7.88900	16.73800
O	35.41500	0.87300	9.27400
H	35.53600	-0.05600	9.47300
H	35.12500	0.88700	8.36200
O	38.28400	-1.44200	12.41100
H	38.30600	-1.37800	11.45600
H	38.70100	-2.28100	12.60500
O	29.41300	6.66900	19.78900
H	28.67100	6.81700	20.37600
H	29.09200	6.92900	18.92600
O	37.35700	16.21000	19.58700
H	37.66600	15.96500	20.45900
H	38.15400	16.41500	19.09900
O	37.37900	18.36400	11.81800
H	36.93900	17.87200	12.51100
H	38.13000	17.82100	11.57700
O	29.85800	14.55500	11.82000
H	30.68400	14.25500	11.44000
H	29.50800	13.78600	12.26900
O	34.82600	13.90800	13.89000
H	34.20400	13.24500	13.59000

H	35.49700	13.93400	13.20700
O	28.05900	12.47400	12.77900
H	27.22200	12.04600	12.59700
H	28.03100	12.66700	13.71600
O	34.89900	2.38600	23.48300
H	34.94800	3.26200	23.10100
H	35.44100	1.84400	22.91000
O	34.38800	12.45800	7.36600
H	34.53700	13.14200	6.71200
H	35.19400	12.43900	7.88100
O	37.12900	3.13900	20.23800
H	36.25900	3.43200	19.96600
H	37.36500	3.72300	20.95900
O	32.14300	8.48500	8.37200
H	31.89800	7.58100	8.57000
H	32.31300	8.48600	7.43000
O	39.83300	-0.64300	17.75400
H	40.35100	0.09300	17.42900
H	39.47800	-0.33700	18.58900
O	31.41300	4.21700	5.87000
H	30.71900	4.29200	5.21400
H	31.16800	3.45400	6.39300
O	40.08400	-4.22300	13.27400
H	40.36400	-4.89100	13.90000
H	40.86900	-4.03000	12.76200
O	37.88700	14.95300	4.83700
H	37.31500	15.57200	4.38400
H	38.46100	15.50300	5.37100
O	41.97600	1.28400	17.20800
H	41.38100	1.56500	16.51200
H	41.69100	1.77400	17.97900
O	41.10700	15.23700	5.96800
H	42.04000	15.17100	6.17100
H	41.01900	16.07200	5.50900
O	38.27200	6.28900	2.17900
H	39.21800	6.16000	2.24700
H	37.89300	5.55000	2.65500
O	38.15600	10.34100	23.63900
H	39.07600	10.51900	23.83300
H	38.12100	9.39600	23.48700
O	36.45600	4.21400	2.11400
H	35.54900	3.91700	2.19000
H	36.73400	3.91700	1.24700
O	27.37400	5.53500	12.56400
H	27.05400	4.68100	12.27100
H	26.82700	5.75000	13.32000
O	31.15200	8.25100	16.28100
H	31.37100	9.18300	16.30000
H	31.56500	7.89200	17.06600
O	38.07200	-1.24200	8.26800
H	37.26300	-1.58800	8.64400
H	38.76100	-1.55200	8.85700
O	40.00200	-2.16400	9.89100
H	40.55500	-1.74500	10.55000
H	40.23900	-3.09100	9.93300
O	36.02500	3.82400	25.67100
H	35.92400	4.77400	25.60200
H	35.80700	3.49600	24.79900
O	35.22600	1.07300	2.25500
H	36.08800	1.39800	2.51500
H	35.41100	0.28600	1.74300
O	27.24300	9.15500	14.53300
H	26.54900	8.74000	14.02000
H	27.99000	9.19700	13.93700
O	41.83000	-0.65400	11.60100
H	42.33000	-0.54200	10.79200
H	42.47300	-0.95800	12.24100
O	35.88800	7.25400	24.48800
H	35.40500	8.05400	24.69400
H	35.97400	7.26500	23.53400
O	29.15700	9.08700	12.41000
H	29.13400	9.18600	11.45800
H	29.46700	9.93300	12.73200
O	31.81500	11.14400	16.52400
H	32.77000	11.11300	16.45700
H	31.61700	10.68300	17.34000
O	32.40500	9.71500	19.09200
H	33.22500	10.20900	19.11500
H	32.67900	8.79800	19.06300
O	42.16200	2.10600	8.45400
H	41.27400	2.39100	8.23600
H	42.57700	1.94500	7.60700
O	30.17000	16.68600	13.54200
H	30.03600	15.92600	12.97500
H	29.75400	17.41000	13.07400
O	41.30600	11.87000	2.54900
H	42.01000	12.07500	3.16500
H	40.58200	12.43800	2.81400
O	42.13600	7.62100	8.66900
H	42.07900	8.07800	7.83000
H	42.68900	6.86000	8.49200
O	33.32700	17.58300	13.75300
H	33.21000	18.52800	13.65100
H	32.50600	17.28100	14.14200
O	42.86900	8.81200	5.96500
H	42.81400	8.64600	5.02300
H	42.24400	9.51900	6.12100
O	27.65900	4.04800	19.79100
H	28.30500	3.53800	20.28000
H	27.45000	4.78500	20.36500
O	40.18400	11.11600	25.29000
H	41.05300	11.29000	24.92900
H	39.70500	11.93600	25.16800
O	36.17900	-1.68800	5.54300
H	36.16700	-0.74300	5.70000
H	37.07100	-1.87100	5.24900
O	34.35300	18.23600	16.96900
H	35.25500	18.26800	17.28600
H	34.12600	17.30600	16.98400
O	36.10800	16.98900	13.63000
H	35.15800	16.98900	13.51600
H	36.41100	16.24200	13.11400
O	39.99400	-5.09300	9.79800
H	39.22800	-5.64800	9.94300
H	40.40500	-5.02100	10.65900
O	31.99800	1.19500	23.88800
H	32.86200	0.83900	24.09900
H	31.42500	0.85600	24.57600

O	42.05700	26.12700	13.45900
H	42.85000	25.78600	13.04300
H	42.29700	26.23600	14.37900
O	41.62600	22.49600	13.40000
H	41.75500	22.75000	14.31400
H	40.94600	23.08900	13.08100
O	40.57300	19.96800	13.44500
H	39.78300	20.22400	12.96900
H	41.06400	20.78300	13.55100
O	42.52500	20.33300	10.40400
H	41.72800	20.44500	10.92200
H	42.29700	20.67100	9.53800
O	38.85700	20.22100	15.87700
H	39.10800	21.10600	16.14300
H	39.39300	20.04200	15.10400
O	41.52300	26.04200	8.79300
H	42.03500	26.83600	8.94300
H	42.17500	25.34400	8.72600
O	42.61800	21.20500	20.75100
H	42.50300	20.30300	21.05200
H	41.76200	21.45100	20.40000
O	39.48800	24.37000	9.56900
H	40.32500	24.78000	9.35200
H	39.66600	23.43000	9.53900
O	39.51000	21.70900	10.24000
H	39.31300	21.11600	9.51500
H	38.95500	21.40500	10.95700
O	42.00500	21.23600	7.92400
H	41.07500	21.17400	8.14100
H	42.10100	20.71300	7.12700
O	41.92300	17.76100	10.51200
H	42.18400	18.67600	10.40300
H	42.74500	17.29400	10.66100
O	36.07000	23.39400	12.40600
H	36.75700	23.08100	11.81900
H	35.91500	24.29900	12.13300
O	40.87400	18.28400	16.83500
H	40.79000	17.67600	16.10000
H	40.05200	18.77400	16.83600
O	41.05500	28.90400	15.95800
H	41.64100	28.15800	16.09100
H	40.85800	29.21400	16.84100
O	35.64700	21.47500	14.67800
H	35.68600	20.94100	15.47200
H	35.14100	20.94800	14.05900
O	39.27800	20.20100	7.75400
H	39.57300	19.29200	7.70000
H	38.37700	20.18000	7.43200
O	41.00700	17.88200	7.87800
H	40.67800	17.00200	7.69500
H	41.36100	17.82700	8.76500
O	39.46500	22.87100	16.89600
H	38.92100	23.64700	17.02800
H	40.34300	23.21800	16.73800
O	40.25200	21.99800	19.78900
H	39.51000	22.14600	20.37600
H	39.93100	22.25900	18.92600
O	40.69800	29.88400	11.82000
H	41.52300	29.58500	11.44000
H	40.34700	29.11500	12.26900
O	38.89800	27.80300	12.77900
H	38.06200	27.37500	12.59700
H	38.87100	27.99600	13.71600
O	42.25200	19.54700	5.87000
H	41.55900	19.62100	5.21400
H	42.00700	18.78300	6.39300
O	38.21300	20.86400	12.56400
H	37.89400	20.01000	12.27100
H	37.66700	21.07900	13.32000
O	41.99100	23.58000	16.28100
H	42.21000	24.51200	16.30000
H	42.40400	23.22100	17.06600
O	38.08200	24.48400	14.53300
H	37.38900	24.07000	14.02000
H	38.83000	24.52600	13.93700
O	39.99600	24.41600	12.41000
H	39.97300	24.51500	11.45800
H	40.30600	25.26200	12.73200
O	38.49800	19.37800	19.79100
H	39.14400	18.86700	20.28000
H	38.29000	20.11400	20.36500
O	13.27800	-35.81600	-3.77600
H	13.20000	-34.86500	-3.69700
H	12.67400	-36.16100	-3.11900
O	10.81400	-39.82300	-2.40100
H	10.99100	-38.90100	-2.58800
H	11.61000	-40.13700	-1.97100
O	11.93700	-38.30500	3.60000
H	12.48800	-38.43400	2.82800
H	11.09800	-38.00600	3.25000
O	18.15100	-36.38400	-8.62000
H	17.47800	-36.08100	-8.01200
H	18.53800	-35.58100	-8.97000
O	15.06200	-38.76600	-1.22400
H	15.02000	-38.54000	-2.15300
H	15.97800	-39.00000	-1.07500
O	16.35000	-36.21600	-6.40500
H	16.52600	-36.68700	-5.59100
H	15.66400	-35.58900	-6.17700
O	10.91700	-33.06700	0.36900
H	11.15600	-32.43500	1.04700
H	10.07400	-33.41800	0.65500
O	15.42900	-37.25900	2.77300
H	15.35600	-36.45400	3.28500
H	14.98700	-37.06000	1.94800
O	15.21800	-38.02200	-3.71700
H	14.43500	-37.48000	-3.62300
H	15.91800	-37.50500	-3.31700
O	17.88900	-37.36000	4.92900
H	18.60200	-36.72600	4.85600
H	17.39800	-37.26400	4.11300
O	12.94000	-39.61100	5.74900
H	13.64100	-38.97700	5.90200
H	12.47000	-39.27000	4.98900
O	17.70100	-37.02100	-2.32300
H	17.88400	-37.83800	-1.86000
H	18.52500	-36.80400	-2.76000
O	18.03600	-38.09400	-5.06100

H	18.56400	-38.60500	-5.67400
H	18.29800	-38.40900	-4.19700
O	8.53700	-40.07700	2.68200
H	9.12300	-40.82300	2.81500
H	8.34000	-39.76700	3.56600
O	11.39100	-37.04200	-2.30100
H	10.72200	-36.55800	-2.78500
H	11.25200	-36.79500	-1.38700
O	15.53200	-35.00800	4.29700
H	16.08300	-34.84500	5.06200
H	15.87200	-34.41700	3.62500
O	18.66800	-37.06000	1.28300
H	18.54400	-37.86100	0.77400
H	19.32600	-36.56500	0.79500
O	15.67800	-37.44200	6.31200
H	15.98700	-37.68700	7.18400
H	16.47600	-37.23700	5.82400
O	15.70000	-35.28800	-1.45800
H	15.26100	-35.78100	-0.76500
H	16.45100	-35.83100	-1.69900
O	8.17900	-39.09700	-1.45500
H	9.00500	-39.39700	-1.83500
H	7.82900	-39.86600	-1.00600
O	13.14800	-39.74400	0.61400
H	12.52600	-40.40700	0.31500
H	13.81800	-39.71800	-0.06800
O	6.38000	-41.17800	-0.49600
H	5.54400	-41.60600	-0.67800
H	6.35300	-40.98500	0.44100
O	16.20900	-38.69900	-8.43900
H	15.63600	-38.08000	-8.89200
H	16.78200	-38.14900	-7.90500
O	8.49200	-36.96600	0.26600
H	8.35700	-37.72600	-0.30000
H	8.07500	-36.24200	-0.20100
O	11.64800	-36.06900	0.47800
H	11.53200	-35.12400	0.37600
H	10.82800	-36.37200	0.86600
O	12.67400	-35.41600	3.69300
H	13.57700	-35.38400	4.01000
H	12.44800	-36.34600	3.70800
O	14.42900	-36.66300	0.35400
H	13.47900	-36.66300	0.24100
H	14.73300	-37.41000	-0.16200
O	30.16500	-30.82700	1.41900
H	30.80200	-30.94600	0.71500
H	30.04700	-29.87900	1.47800
O	26.97400	-30.87400	1.35500
H	26.63500	-30.99000	0.46700
H	27.84200	-31.27500	1.33500
O	22.83200	-29.02100	-2.65500
H	22.26900	-29.11200	-3.42300
H	22.71700	-29.84100	-2.17500
O	27.45500	-29.72100	7.31400
H	27.44400	-30.11500	6.44200
H	27.61800	-30.45400	7.90700
O	20.37900	-27.52500	0.18300
H	21.17100	-27.86600	-0.23200
H	20.61800	-27.41600	1.10400
O	28.41200	-25.35400	-7.34400
H	27.76100	-24.76800	-7.73000
H	29.20700	-24.82300	-7.28400
O	22.33800	-28.26500	-6.94500
H	23.11100	-28.81200	-7.08400
H	22.69000	-27.40000	-6.73300
O	24.17600	-26.93700	6.26200
H	23.88500	-26.03200	6.14600
H	24.81200	-26.89500	6.97600
O	25.10200	-29.55900	2.85200
H	25.69800	-29.78400	2.13800
H	25.15900	-30.30100	3.45400
O	24.67400	-32.10500	-9.31300
H	24.92700	-32.62700	-10.07500
H	23.73700	-32.26900	-9.21000
O	29.71900	-33.10200	-1.50600
H	30.50500	-33.52000	-1.85700
H	29.98300	-32.19700	-1.33700
O	29.89000	-27.66400	-6.80700
H	29.25400	-27.59100	-6.09500
H	29.55900	-27.07500	-7.48500
O	24.39400	-30.20300	-7.11100
H	24.27800	-30.87800	-6.44300
H	24.50400	-30.68900	-7.92700
O	26.73700	-30.74200	-3.40600
H	26.37300	-31.26800	-2.69500
H	27.59400	-31.13200	-3.57900
O	26.37800	-35.71300	-8.41000
H	26.54500	-34.84400	-8.04300
H	26.18700	-35.55000	-9.33400
O	29.13500	-27.25100	3.25700
H	28.83500	-26.34800	3.14800
H	28.79000	-27.51800	4.10900
O	22.58600	-30.79900	-0.71000
H	21.66200	-30.93200	-0.50200
H	22.83500	-30.02000	-0.21300
O	26.35700	-33.25700	-7.42500
H	26.23700	-32.81100	-6.58700
H	25.66200	-32.90800	-7.98400
O	19.94700	-31.15600	0.12500
H	20.07700	-30.90300	1.03900
H	19.26700	-30.56300	-0.19500
O	24.11700	-20.48600	-3.77600
H	24.04000	-19.53600	-3.69700
H	23.51300	-20.83200	-3.11900
O	26.60900	-33.31400	8.88000
H	25.83200	-32.75400	8.87000
H	27.24800	-32.82800	9.40100
O	32.05500	-32.78500	-6.59700
H	32.96700	-32.55200	-6.77200
H	31.54600	-32.20100	-7.16000
O	24.86400	-30.50500	8.44200
H	25.60500	-29.96700	8.16500
H	24.15700	-29.87800	8.59900
O	30.33900	-28.36200	7.03900
H	30.74800	-28.80400	6.29400
H	29.61700	-28.93700	7.29200
O	26.69200	-33.42500	4.41200
H	27.53800	-33.00100	4.55600

H	26.63200	-34.08200	5.10600
O	30.66200	-27.80200	0.79400
H	30.03300	-27.63700	0.09200
H	30.27500	-27.38800	1.56500
O	34.54100	-26.86700	-1.85100
H	33.85600	-26.48700	-2.40200
H	34.62500	-26.25700	-1.11800
O	31.79600	-29.22100	4.74800
H	32.00800	-28.57700	4.07200
H	31.74900	-30.05400	4.28000
O	26.21500	-28.06500	-1.94100
H	26.67900	-27.78600	-2.73000
H	25.95100	-28.96600	-2.12300
O	23.10700	-28.51700	0.99700
H	23.67700	-28.08900	0.35900
H	23.69800	-28.79900	1.69600
O	25.15200	-31.94200	-5.13200
H	25.85400	-31.49000	-4.66400
H	24.82000	-32.58400	-4.50400
O	23.60000	-33.35100	9.24300
H	22.97000	-32.66900	9.47700
H	23.49300	-33.46300	8.29900
O	18.89400	-33.68400	0.17000
H	18.10400	-33.42800	-0.30600
H	19.38500	-32.86900	0.27500
O	32.91100	-26.34300	2.92000
H	33.73200	-26.74500	2.63900
H	32.90700	-26.44700	3.87200
O	20.84600	-33.31900	-2.87200
H	20.04900	-33.20800	-2.35300
H	20.61800	-32.98100	-3.73800
O	26.67600	-26.79500	5.07600
H	26.74100	-26.33300	5.91200
H	25.96700	-27.42500	5.20500
O	17.17800	-33.43100	2.60100
H	17.42900	-32.54600	2.86700
H	17.71400	-33.61000	1.82900
O	41.00100	-30.00000	-0.73100
H	40.24800	-30.56900	-0.88700
H	41.13600	-30.03300	0.21600
O	26.39100	-25.12800	-5.05600
H	26.25600	-24.28300	-4.62700
H	26.93200	-24.92600	-5.81900
O	19.84400	-27.61100	-4.48300
H	20.35700	-26.81600	-4.33200
H	20.49600	-28.30800	-4.54900
O	21.65400	-24.49400	-2.40100
H	21.83000	-23.57200	-2.58800
H	22.44900	-24.80800	-1.97100
O	20.93900	-32.44800	7.47600
H	20.82400	-33.34900	7.77600
H	20.08300	-32.20100	7.12400
O	28.01800	-24.50600	9.07400
H	27.57400	-25.29200	8.75700
H	28.68000	-24.31800	8.40900
O	22.77700	-22.97600	3.60000
H	23.32700	-23.10500	2.82800
H	21.93800	-22.67700	3.25000
O	30.11600	-24.22600	7.10300
H	30.09800	-23.40500	6.61100
H	31.03800	-24.35000	7.32800
O	26.57300	-26.71100	7.94500
H	27.10600	-27.47200	7.71500
H	26.04500	-27.00400	8.68700
O	17.80900	-29.28200	-3.70700
H	18.64600	-28.87200	-3.92400
H	17.98700	-30.22200	-3.73700
O	29.70500	-31.61400	-8.01800
H	29.19000	-30.84400	-7.78000
H	29.43300	-32.28800	-7.39500
O	34.67900	-28.86100	-6.08300
H	33.90200	-29.20000	-6.52800
H	35.35800	-28.85600	-6.75800
O	29.49400	-33.74200	-6.19300
H	29.18900	-33.42700	-5.34100
H	30.44700	-33.66300	-6.15000
O	27.93900	-27.58300	-4.54300
H	27.41100	-28.31700	-4.85600
H	27.43700	-26.80500	-4.78900
O	32.91800	-29.38800	0.57300
H	32.26000	-28.69800	0.66200
H	33.72200	-28.99800	0.91700
O	22.67100	-36.92100	-2.18300
H	23.24500	-37.14300	-2.91500
H	23.25800	-36.85200	-1.43000
O	31.72900	-31.03300	-1.37600
H	32.24200	-30.40000	-0.87400
H	32.37900	-31.63300	-1.74200
O	22.99800	-34.89000	-8.16300
H	22.16100	-34.55300	-7.84200
H	22.85800	-35.02500	-9.10000
O	26.08400	-29.76500	-11.54000
H	25.17700	-29.99600	-11.34100
H	26.58100	-30.56100	-11.35200
O	28.99100	-21.05500	-8.62000
H	28.31700	-20.75200	-8.01200
H	29.37700	-20.25200	-8.97000
O	28.20700	-33.55800	0.87000
H	27.28900	-33.33700	0.71200
H	28.62300	-33.48400	0.01100
O	28.99000	-32.15900	-4.00000
H	29.73700	-31.64700	-4.30900
H	29.25900	-32.48100	-3.14000
O	27.26300	-29.66300	-7.01700
H	26.35400	-29.94700	-6.92000
H	27.29200	-29.24300	-7.87700
O	32.49500	-26.05300	-3.24800
H	32.04100	-26.89300	-3.19200
H	31.86400	-25.41600	-2.91100
O	30.93800	-24.21500	-2.31300
H	31.23000	-23.65400	-3.03200
H	31.44800	-23.92200	-1.55800
O	17.83200	-31.94300	-3.03600
H	17.63400	-32.53600	-3.76100
H	17.27600	-32.24700	-2.31800
O	25.81100	-32.05000	-0.91600
H	25.86600	-33.00300	-0.99200
H	25.00000	-31.89400	-0.43200

O	26.96000	-28.12200	-9.67900
H	26.52200	-28.83900	-10.13800
H	27.70100	-27.89500	-10.24000
O	23.75000	-33.43300	-3.15700
H	22.93700	-33.13300	-2.75100
H	24.24600	-33.82700	-2.44000
O	25.90100	-23.43700	-1.22400
H	25.85900	-23.21100	-2.15300
H	26.81800	-23.67100	-1.07500
O	29.34800	-32.96800	5.67400
H	29.70300	-32.74100	6.53400
H	29.99500	-32.63700	5.05200
O	35.07800	-29.84700	-3.62100
H	34.93900	-29.65600	-4.54900
H	34.72100	-29.08500	-3.16400
O	23.24200	-35.86100	2.45900
H	23.88200	-36.00200	1.76100
H	22.41000	-36.14700	2.08300
O	35.10500	-27.77900	1.39000
H	35.53800	-27.87100	2.23900
H	35.81800	-27.80300	0.75200
O	24.01800	-26.56100	-1.42800
H	24.81200	-27.05600	-1.62900
H	23.30700	-27.08900	-1.79100
O	28.83700	-24.17900	2.92400
H	28.89100	-23.61000	3.69100
H	27.96900	-24.00900	2.55900
O	20.32600	-32.41600	-5.35200
H	19.39600	-32.47800	-5.13500
H	20.42200	-32.93900	-6.14800
O	20.24500	-35.89100	-2.76400
H	20.50500	-34.97600	-2.87300
H	21.06600	-36.35800	-2.61400
O	21.71700	-34.47700	4.40600
H	22.39000	-34.68500	3.75700
H	20.89000	-34.67500	3.96700
O	35.96600	-27.73700	3.92400
H	36.16000	-26.83200	4.16900
H	35.86600	-28.19600	4.75700
O	31.02800	-27.58600	9.49800
H	30.87600	-27.81500	8.58100
H	31.01800	-26.62900	9.51100
O	27.79600	-30.01300	4.48200
H	28.72500	-29.86200	4.30900
H	27.37500	-29.92400	3.62700
O	26.59500	-25.98800	2.25200
H	26.45600	-26.35600	3.12500
H	25.80700	-25.47300	2.08100
O	27.19000	-20.88700	-6.40500
H	27.36500	-21.35800	-5.59100
H	26.50300	-20.26000	-6.17700
O	28.14600	-26.61000	-0.35900
H	27.81100	-26.64900	0.53700
H	27.64600	-27.27500	-0.83200
O	22.12000	-31.20300	5.16600
H	21.98200	-31.93500	5.76700
H	23.03000	-31.29200	4.88500
O	31.08100	-29.26400	-10.08500
H	30.46400	-29.98900	-10.18200
H	30.61300	-28.50300	-10.42900
O	24.74800	-31.49100	4.65500
H	25.45800	-32.13100	4.61300
H	24.70500	-31.23900	5.57700
O	32.73700	-24.55400	7.57500
H	32.67800	-25.23800	6.90800
H	33.13900	-23.81200	7.12300
O	23.46100	-34.50400	6.47400
H	22.92500	-34.95500	7.12600
H	22.87300	-34.37000	5.73100
O	21.75600	-17.73800	0.36900
H	21.99600	-17.10600	1.04700
H	20.91300	-18.08900	0.65500
O	22.87100	-35.04500	-10.96300
H	21.99400	-35.16700	-11.32700
H	23.34700	-35.83700	-11.21500
O	32.73900	-26.59000	5.80700
H	31.85100	-26.92700	5.92900
H	33.30800	-27.32200	6.04400
O	14.39200	-30.25800	-0.86900
H	15.07900	-30.57200	-1.45700
H	14.23600	-29.35400	-1.14200
O	19.19500	-35.36900	3.55900
H	19.11100	-35.97600	2.82500
H	18.37300	-34.87800	3.56000
O	26.26900	-21.93000	2.77300
H	26.19600	-21.12400	3.28500
H	25.82600	-21.73100	1.94800
O	26.05800	-22.69300	-3.71700
H	25.27400	-22.15100	-3.62300
H	26.75700	-22.17500	-3.31700
O	37.22000	-31.17900	0.56800
H	37.20900	-30.29700	0.19800
H	36.35200	-31.53200	0.37400
O	28.72900	-22.03100	4.92900
H	29.44100	-21.39700	4.85600
H	28.23800	-21.93500	4.11300
O	30.45000	-28.12600	-3.50000
H	29.51600	-28.00600	-3.66900
H	30.62800	-29.02900	-3.76400
O	25.95100	-34.74200	-1.34600
H	25.58100	-35.29400	-0.65800
H	26.44700	-35.34600	-1.89900
O	33.83800	-32.28400	-2.96500
H	34.30400	-31.45200	-3.03900
H	34.28800	-32.75000	-2.26000
O	23.78000	-24.28200	5.74900
H	24.48100	-23.64800	5.90200
H	23.30900	-23.94000	4.98900
O	31.90500	-25.94000	-5.96600
H	32.18100	-26.05400	-5.05700
H	31.03600	-26.33800	-6.00500
O	31.58000	-31.72700	3.57400
H	30.92700	-31.48000	2.92000
H	31.95800	-32.54100	3.24100
O	28.54100	-21.69100	-2.32300
H	28.72300	-22.50900	-1.86000
H	29.36400	-21.47500	-2.76000
O	28.87500	-22.76500	-5.06100

H	29.40400	-23.27500	-5.67400
H	29.13800	-23.08000	-4.19700
O	27.62900	-30.98500	10.06100
H	26.95300	-31.08600	10.73100
H	28.45100	-31.14100	10.52500
O	19.37700	-24.74800	2.68200
H	19.96200	-25.49400	2.81500
H	19.17900	-24.43800	3.56600
O	22.23000	-21.71300	-2.30100
H	21.56100	-21.22800	-2.78500
H	22.09200	-21.46600	-1.38700
O	13.96800	-32.17800	1.40300
H	14.00700	-32.71100	2.19600
H	13.46200	-32.70400	0.78400
O	26.37100	-19.67900	4.29700
H	26.92300	-19.51600	5.06200
H	26.71200	-19.08800	3.62500
O	21.34700	-37.33100	4.89900
H	21.41200	-36.47000	4.48700
H	21.28400	-37.94400	4.16600
O	20.82300	-35.25900	8.54600
H	21.45000	-35.64100	9.16000
H	19.96500	-35.47700	8.91200
O	31.22200	-24.85300	10.15200
H	31.76600	-24.24000	9.65700
H	30.64100	-24.29500	10.66800
O	17.59900	-33.45100	-5.52200
H	17.89500	-34.36000	-5.57500
H	16.69800	-33.47200	-5.84400
O	29.50800	-21.73000	1.28300
H	29.38300	-22.53200	0.77400
H	30.16600	-21.23600	0.79500
O	21.12900	-35.37600	0.46100
H	20.40700	-34.76200	0.32200
H	21.91800	-34.84000	0.38300
O	31.57100	-23.93000	2.91100
H	30.62400	-24.04900	2.83900
H	31.92000	-24.81700	2.99400
O	19.32800	-35.77000	-5.39800
H	18.99900	-36.65000	-5.58000
H	19.68200	-35.82500	-4.51000
O	28.57600	-24.13400	-0.88800
H	28.42800	-25.06300	-0.70800
H	29.40100	-24.11200	-1.37300
O	22.44200	-35.48200	-5.03000
H	22.78200	-34.95400	-5.75300
H	22.88900	-35.14400	-4.25500
O	32.17900	-23.31100	0.19800
H	33.10400	-23.51700	0.06000
H	32.01100	-23.56000	1.10700
O	24.42900	-36.98500	-6.83100
H	25.15000	-36.60700	-7.33500
H	23.80600	-36.26600	-6.72800
O	23.31200	-33.32200	0.68200
H	23.16600	-33.00800	1.57400
H	22.83500	-32.70300	0.12900
O	31.96900	-22.79300	-4.44600
H	32.14300	-23.35800	-5.19800
H	32.46200	-21.99300	-4.62800
O	17.78700	-30.78100	3.62100
H	17.24200	-30.00500	3.75300
H	18.66500	-30.43400	3.46300
O	37.82300	-30.02900	-2.82700
H	38.26600	-30.77900	-3.22500
H	36.94500	-30.03800	-3.20900
O	32.95600	-23.47600	-7.34600
H	33.65200	-22.85600	-7.12700
H	33.29900	-24.32700	-7.07100
O	18.57300	-31.65400	6.51400
H	17.83100	-31.50600	7.10000
H	18.25200	-31.39400	5.65000
O	34.85600	-26.02600	-5.81700
H	35.38100	-25.68800	-6.54200
H	34.76800	-26.96200	-6.00100
O	26.51800	-22.11300	6.31200
H	26.82600	-22.35800	7.18400
H	27.31500	-21.90800	5.82400
O	26.54000	-19.95900	-1.45800
H	26.10000	-20.45100	-0.76500
H	27.29000	-20.50200	-1.69900
O	19.01900	-23.76800	-1.45500
H	19.84500	-24.06800	-1.83500
H	18.66800	-24.53700	-1.00600
O	23.98700	-24.41500	0.61400
H	23.36500	-25.07800	0.31500
H	24.65700	-24.38900	-0.06800
O	17.22000	-25.84900	-0.49600
H	16.38300	-26.27700	-0.67800
H	17.19200	-25.65600	0.44100
O	24.05900	-35.93700	10.20800
H	24.10800	-35.06100	9.82500
H	24.60200	-36.47900	9.63500
O	34.54500	-25.39300	9.48800
H	33.75500	-25.41100	8.94800
H	34.30800	-24.85400	10.24300
O	34.66500	-28.54400	6.80100
H	34.71500	-28.70900	7.74300
H	34.55600	-29.41100	6.41100
O	23.54800	-25.86500	-5.91000
H	23.69700	-25.18100	-6.56300
H	24.35500	-25.88400	-5.39500
O	31.95500	-32.56100	8.11700
H	32.85600	-32.70000	7.82500
H	32.03000	-32.39500	9.05700
O	26.28900	-35.18400	6.96200
H	25.42000	-34.89100	6.69100
H	26.52500	-34.60000	7.68300
O	36.16700	-26.12400	7.04500
H	35.87500	-25.76100	7.88200
H	35.55400	-26.83700	6.86700
O	37.36600	-28.55900	-0.43400
H	38.20100	-28.12000	-0.27100
H	37.50200	-29.03500	-1.25400
O	34.41500	-31.00100	5.51300
H	34.89100	-30.80800	4.70500
H	33.49200	-30.94500	5.26800
O	21.30400	-29.83800	-4.90400
H	21.05800	-30.74200	-4.70600

H	21.47300	-29.83700	-5.84600
O	20.57300	-34.10500	-7.40500
H	19.88000	-34.03100	-8.06100
H	20.32800	-34.86900	-6.88300
O	27.04800	-23.37000	-8.43900
H	26.47500	-22.75100	-8.89200
H	27.62100	-22.82000	-7.90500
O	30.26800	-23.08600	-7.30800
H	31.20100	-23.15200	-7.10400
H	30.18000	-22.25100	-7.76600
O	32.89600	-23.77000	-10.34700
H	32.81800	-24.10400	-9.45400
H	33.71700	-23.27800	-10.34800
O	35.59400	-30.90400	2.86000
H	35.56200	-31.84200	3.04600
H	36.20400	-30.82500	2.12700
O	27.43300	-32.03400	-11.09700
H	28.37800	-32.16300	-11.02800
H	27.05400	-32.77200	-10.62000
O	41.41200	-29.84200	2.00100
H	42.14200	-30.10300	2.56200
H	40.80300	-30.57900	2.04100
O	27.31600	-27.98200	10.36300
H	28.23700	-27.80400	10.55700
H	27.28100	-28.92700	10.21100
O	25.61700	-34.10900	-11.16100
H	24.71000	-34.40600	-11.08500
H	25.89400	-34.40600	-12.02800
O	16.53500	-32.78800	-0.71200
H	16.21500	-33.64200	-1.00400
H	15.98800	-32.57300	0.04400
O	20.31300	-30.07200	3.00500
H	20.53100	-29.14000	3.02400
H	20.72500	-30.43100	3.79100
O	25.18500	-34.49900	12.39600
H	25.08500	-33.54900	12.32700
H	24.96800	-34.82700	11.52300
O	16.40300	-29.16800	1.25800
H	15.71000	-29.58300	0.74500
H	17.15100	-29.12600	0.66100
O	25.04900	-31.06900	11.21200
H	24.56500	-30.26900	11.41900
H	25.13500	-31.05800	10.25900
O	18.31700	-29.23600	-0.86600
H	18.29400	-29.13700	-1.81700
H	18.62700	-28.39000	-0.54400
O	20.97600	-27.17900	3.24900
H	21.93000	-27.21000	3.18200
H	20.77700	-27.64000	4.06400
O	21.56600	-28.60800	5.81600
H	22.38500	-28.11400	5.84000
H	21.84000	-29.52500	5.78800
O	19.33100	-21.63700	0.26600
H	19.19600	-22.39700	-0.30000
H	18.91400	-20.91300	-0.20100
O	30.46700	-26.45300	-10.72600
H	31.17000	-26.24800	-10.11100
H	29.74300	-25.88500	-10.46100
O	31.29700	-30.70200	-4.60600
H	31.24000	-30.24500	-5.44500
H	31.84900	-31.46300	-4.78400
O	22.48800	-20.74000	0.47800
H	22.37100	-19.79500	0.37600
H	21.66700	-21.04200	0.86600
O	32.37500	-26.01500	-8.67600
H	33.21000	-26.44700	-8.85900
H	32.21400	-26.18600	-7.74900
O	32.03000	-29.51100	-7.31100
H	31.97500	-29.67700	-8.25200
H	31.40500	-28.80400	-7.15500
O	16.81900	-34.27500	6.51500
H	17.46500	-34.78500	7.00400
H	16.61100	-33.53800	7.09000
O	29.34500	-27.20700	12.01500
H	30.21400	-27.03300	11.65300
H	28.86500	-26.38700	11.89200
O	23.51300	-20.08700	3.69300
H	24.41600	-20.05500	4.01000
H	23.28700	-21.01700	3.70800
O	25.26900	-21.33400	0.35400
H	24.31800	-21.33400	0.24100
H	25.57200	-22.08000	-0.16200
O	21.15900	-37.12800	10.61300
H	22.02200	-37.48400	10.82300
H	20.58600	-37.46700	11.30000
O	41.00400	-15.49800	1.41900
H	41.64200	-15.61700	0.71500
H	40.88600	-14.55000	1.47800
O	37.81300	-15.54500	1.35500
H	37.47500	-15.66100	0.46700
H	38.68200	-15.94600	1.33500
O	33.67200	-13.69100	-2.65500
H	33.10800	-13.78200	-3.42300
H	33.55700	-14.51100	-2.17500
O	38.29500	-14.39100	7.31400
H	38.28400	-14.78600	6.44200
H	38.45700	-15.12500	7.90700
O	31.21800	-12.19600	0.18300
H	32.01000	-12.53700	-0.23200
H	31.45800	-12.08700	1.10400
O	39.25200	-10.02400	-7.34400
H	38.60000	-9.43900	-7.73000
H	40.04600	-9.49400	-7.28400
O	33.17700	-12.93500	-6.94500
H	33.95000	-13.48300	-7.08400
H	33.53000	-12.07100	-6.73300
O	35.01600	-11.60800	6.26200
H	34.72500	-10.70300	6.14600
H	35.65100	-11.56600	6.97600
O	35.94100	-14.23000	2.85200
H	36.53800	-14.45400	2.13800
H	35.99900	-14.97200	3.45400
O	35.51400	-16.77600	-9.31300
H	35.76600	-17.29800	-10.07500
H	34.57600	-16.94000	-9.21000
O	40.55800	-17.77300	-1.50600
H	41.34400	-18.19100	-1.85700
H	40.82200	-16.86800	-1.33700

O	40.72900	-12.33500	-6.80700
H	40.09400	-12.26200	-6.09500
H	40.39900	-11.74600	-7.48500
O	35.23300	-14.87300	-7.11100
H	35.11700	-15.54900	-6.44300
H	35.34400	-15.36000	-7.92700
O	37.57700	-15.41200	-3.40600
H	37.21200	-15.93900	-2.69500
H	38.43300	-15.80200	-3.57900
O	37.21700	-20.38300	-8.41000
H	37.38400	-19.51500	-8.04300
H	37.02700	-20.22100	-9.33400
O	39.97500	-11.92200	3.25700
H	39.67500	-11.01900	3.14800
H	39.63000	-12.18800	4.10900
O	33.42600	-15.47000	-0.71000
H	32.50100	-15.60300	-0.50200
H	33.67400	-14.69100	-0.21300
O	37.19600	-17.92800	-7.42500
H	37.07600	-17.48200	-6.58700
H	36.50100	-17.57900	-7.98400
O	30.78700	-15.82700	0.12500
H	30.91600	-15.57300	1.03900
H	30.10600	-15.23400	-0.19500
O	34.95700	-5.15700	-3.77600
H	34.87900	-4.20600	-3.69700
H	34.35300	-5.50300	-3.11900
O	37.44800	-17.98400	8.88000
H	36.67200	-17.42500	8.87000
H	38.08700	-17.49900	9.40100
O	35.70300	-15.17600	8.44200
H	36.44400	-14.63700	8.16500
H	34.99700	-14.54900	8.59900
O	41.17900	-13.03300	7.03900
H	41.58700	-13.47500	6.29400
H	40.45600	-13.60800	7.29200
O	37.53200	-18.09600	4.41200
H	38.37800	-17.67200	4.55600
H	37.47200	-18.75200	5.10600
O	41.50100	-12.47300	0.79400
H	40.87200	-12.30800	0.09200
H	41.11400	-12.05900	1.56500
O	40.68200	-20.43500	6.07000
H	40.43800	-19.68100	5.53400
H	40.95800	-20.05700	6.90500
O	42.63500	-13.89100	4.74800
H	42.84700	-13.24800	4.07200
H	42.58800	-14.72500	4.28000
O	40.23800	-21.28000	1.65200
H	39.37700	-20.98100	1.94400
H	40.26400	-21.06300	0.72000
O	37.05400	-12.73500	-1.94100
H	37.51800	-12.45700	-2.73000
H	36.79000	-13.63700	-2.12300
O	33.94700	-13.18800	0.99700
H	34.51700	-12.76000	0.35900
H	34.53700	-13.47000	1.69600
O	35.99100	-16.61300	-5.13200
H	36.69400	-16.16100	-4.66400
H	35.65900	-17.25500	-4.50400
O	34.43900	-18.02200	9.24300
H	33.80900	-17.34000	9.47700
H	34.33300	-18.13400	8.29900
O	29.73400	-18.35500	0.17000
H	28.94400	-18.09900	-0.30600
H	30.22500	-17.54000	0.27500
O	31.68500	-17.99000	-2.87200
H	30.88800	-17.87800	-2.35300
H	31.45800	-17.65200	-3.73800
O	37.51600	-11.46600	5.07600
H	37.58000	-11.00400	5.91200
H	36.80700	-12.09600	5.20500
O	28.01700	-18.10200	2.60100
H	28.26800	-17.21700	2.86700
H	28.55300	-18.28100	1.82900
O	37.23000	-9.79900	-5.05600
H	37.09500	-8.95400	-4.62700
H	37.77100	-9.59700	-5.81900
O	33.71700	-23.72000	11.60600
H	33.32400	-24.27300	12.28100
H	34.53400	-23.40900	11.99600
O	30.68300	-12.28100	-4.48300
H	31.19600	-11.48700	-4.33200
H	31.33600	-12.97900	-4.54900
O	32.49300	-9.16500	-2.40100
H	32.67000	-8.24300	-2.58800
H	33.28900	-9.47900	-1.97100
O	36.63400	-22.30400	9.19800
H	37.37500	-21.70300	9.11800
H	36.86600	-23.04600	8.64000
O	31.77800	-17.11800	7.47600
H	31.66400	-18.02000	7.77600
H	30.92300	-16.87200	7.12400
O	38.85700	-9.17700	9.07400
H	38.41400	-9.96300	8.75700
H	39.51900	-8.98900	8.40900
O	33.61600	-7.64700	3.60000
H	34.16700	-7.77500	2.82800
H	32.77700	-7.34800	3.25000
O	40.95500	-8.89600	7.10300
H	40.93700	-8.07500	6.61100
H	41.87700	-9.02100	7.32800
O	37.41300	-11.38200	7.94500
H	37.94600	-12.14300	7.71500
H	36.88400	-11.67500	8.68700
O	40.58000	-26.27600	3.47400
H	40.23600	-25.38600	3.39800
H	40.94800	-26.46600	2.61100
O	28.64800	-13.95300	-3.70700
H	29.48600	-13.54300	-3.92400
H	28.82600	-14.89300	-3.73700
O	40.54500	-16.28500	-8.01800
H	40.02900	-15.51500	-7.78000
H	40.27300	-16.95900	-7.39500
O	38.12900	-21.44000	-2.96000
H	37.27400	-21.82900	-3.13900
H	38.54200	-21.36100	-3.82000
O	39.65900	-23.90500	1.43900

H	39.15600	-23.94200	0.62600
H	39.95200	-22.99600	1.49900
O	35.90400	-23.82200	0.44600
H	36.76900	-24.01700	0.08600
H	35.97100	-24.05100	1.37300
O	40.33300	-18.41200	-6.19300
H	40.02800	-18.09800	-5.34100
H	41.28600	-18.33400	-6.15000
O	38.77800	-12.25300	-4.54300
H	38.25000	-12.98800	-4.85600
H	38.27700	-11.47600	-4.78900
O	33.51000	-21.59100	-2.18300
H	34.08400	-21.81400	-2.91500
H	34.09700	-21.52200	-1.43000
O	33.83700	-19.56100	-8.16300
H	33.00100	-19.22300	-7.84200
H	33.69700	-19.69600	-9.10000
O	35.81200	-24.71700	-3.45500
H	35.15900	-25.03200	-4.07900
H	35.77000	-25.34000	-2.72900
O	36.92300	-14.43500	-11.54000
H	36.01600	-14.66600	-11.34100
H	37.42000	-15.23200	-11.35200
O	39.83000	-5.72600	-8.62000
H	39.15600	-5.42200	-8.01200
H	40.21700	-4.92300	-8.97000
O	39.04600	-18.22900	0.87000
H	38.12800	-18.00800	0.71200
H	39.46200	-18.15400	0.01100
O	39.82900	-16.83000	-4.00000
H	40.57700	-16.31800	-4.30900
H	40.09900	-17.15200	-3.14000
O	38.10200	-14.33400	-7.01700
H	37.19300	-14.61800	-6.92000
H	38.13200	-13.91400	-7.87700
O	42.27700	-19.79400	-2.01800
H	41.54000	-20.30600	-1.68700
H	42.53000	-20.23400	-2.83000
O	41.77700	-8.88600	-2.31300
H	42.07000	-8.32500	-3.03200
H	42.28700	-8.59300	-1.55800
O	28.67100	-16.61400	-3.03600
H	28.47300	-17.20700	-3.76100
H	28.11600	-16.91800	-2.31800
O	36.65100	-16.72100	-0.91600
H	36.70500	-17.67400	-0.99200
H	35.84000	-16.56500	-0.43200
O	37.80000	-12.79300	-9.67900
H	37.36200	-13.51000	-10.13800
H	38.54100	-12.56600	-10.24000
O	34.59000	-18.10300	-3.15700
H	33.77700	-17.80400	-2.75100
H	35.08600	-18.49800	-2.44000
O	36.74100	-8.10800	-1.22400
H	36.69800	-7.88200	-2.15300
H	37.65700	-8.34100	-1.07500
O	40.18700	-17.63900	5.67400
H	40.54200	-17.41200	6.53400
H	40.83400	-17.30700	5.05200
O	34.08100	-20.53200	2.45900
H	34.72100	-20.67300	1.76100
H	33.24900	-20.81800	2.08300
O	34.85700	-11.23100	-1.42800
H	35.65100	-11.72600	-1.62900
H	34.14600	-11.76000	-1.79100
O	39.67700	-8.85000	2.92400
H	39.73000	-8.28100	3.69100
H	38.80800	-8.67900	2.55900
O	31.16600	-17.08700	-5.35200
H	30.23500	-17.14900	-5.13500
H	31.26100	-17.61000	-6.14800
O	31.08400	-20.56200	-2.76400
H	31.34500	-19.64700	-2.87300
H	31.90600	-21.02900	-2.61400
O	32.55700	-19.14800	4.40600
H	33.22900	-19.35600	3.75700
H	31.73000	-19.34600	3.96700
O	38.45900	-22.52200	6.42300
H	37.81200	-21.82300	6.32900
H	39.22900	-22.08800	6.79000
O	41.86700	-12.25700	9.49800
H	41.71600	-12.48600	8.58100
H	41.85800	-11.30000	9.51100
O	38.63600	-14.68400	4.48200
H	39.56500	-14.53200	4.30900
H	38.21500	-14.59500	3.62700
O	37.43400	-10.65900	2.25200
H	37.29600	-11.02700	3.12500
H	36.64600	-10.14400	2.08100
O	38.02900	-5.55800	-6.40500
H	38.20400	-6.02900	-5.59100
H	37.34300	-4.93100	-6.17700
O	38.98500	-11.28000	-0.35900
H	38.65000	-11.32000	0.53700
H	38.48500	-11.94600	-0.83200
O	32.95900	-15.87400	5.16600
H	32.82100	-16.60600	5.76700
H	33.87000	-15.96300	4.88500
O	41.92100	-13.93500	-10.08500
H	41.30400	-14.66000	-10.18200
H	41.45300	-13.17400	-10.42900
O	35.58800	-16.16200	4.65500
H	36.29800	-16.80200	4.61300
H	35.54500	-15.91000	5.57700
O	34.30000	-19.17500	6.47400
H	33.76400	-19.62600	7.12600
H	33.71200	-19.04000	5.73100
O	32.59500	-2.40900	0.36900
H	32.83500	-1.77700	1.04700
H	31.75200	-2.76000	0.65500
O	33.71100	-19.71600	-10.96300
H	32.83400	-19.83700	-11.32700
H	34.18600	-20.50800	-11.21500
O	25.23100	-14.92800	-0.86900
H	25.91800	-15.24200	-1.45700
H	25.07600	-14.02400	-1.14200
O	30.03400	-20.03900	3.55900
H	29.95000	-20.64700	2.82500

H	29.21300	-19.54900	3.56000
O	37.10800	-6.60100	2.77300
H	37.03500	-5.79500	3.28500
H	36.66600	-6.40200	1.94800
O	36.89700	-7.36400	-3.71700
H	36.11400	-6.82100	-3.62300
H	37.59600	-6.84600	-3.31700
O	40.20700	-21.55400	-1.07400
H	40.70400	-22.30700	-1.39300
H	39.43500	-21.51800	-1.63900
O	39.56800	-6.70200	4.92900
H	40.28100	-6.06700	4.85600
H	39.07700	-6.60600	4.11300
O	41.29000	-12.79700	-3.50000
H	40.35500	-12.67700	-3.66900
H	41.46700	-13.70000	-3.76400
O	36.79000	-19.41200	-1.34600
H	36.42000	-19.96500	-0.65800
H	37.28600	-20.01700	-1.89900
O	34.61900	-8.95300	5.74900
H	35.32000	-8.31900	5.90200
H	34.14900	-8.61100	4.98900
O	42.74500	-10.61100	-5.96600
H	43.02100	-10.72500	-5.05700
H	41.87500	-11.00900	-6.00500
O	42.41900	-16.39800	3.57400
H	41.76600	-16.15100	2.92000
H	42.79700	-17.21200	3.24100
O	39.38000	-6.36200	-2.32300
H	39.56200	-7.18000	-1.86000
H	40.20300	-6.14500	-2.76000
O	39.71400	-7.43500	-5.06100
H	40.24300	-7.94600	-5.67400
H	39.97700	-7.75100	-4.19700
O	38.46800	-15.65600	10.06100
H	37.79200	-15.75700	10.73100
H	39.29100	-15.81200	10.52500
O	30.21600	-9.41900	2.68200
H	30.80100	-10.16500	2.81500
H	30.01800	-9.10900	3.56600
O	33.07000	-6.38400	-2.30100
H	32.40100	-5.89900	-2.78500
H	32.93100	-6.13700	-1.38700
O	24.80800	-16.84800	1.40300
H	24.84600	-17.38200	2.19600
H	24.30200	-17.37500	0.78400
O	37.21000	-4.34900	4.29700
H	37.76200	-4.18600	5.06200
H	37.55100	-3.75900	3.62500
O	32.18700	-22.00200	4.89900
H	32.25100	-21.14000	4.48700
H	32.12400	-22.61500	4.16600
O	31.66200	-19.93000	8.54600
H	32.28900	-20.31200	9.16000
H	30.80500	-20.14800	8.91200
O	42.06100	-9.52300	10.15200
H	42.60500	-8.91000	9.65700
H	41.48000	-8.96500	10.66800
O	28.43900	-18.12200	-5.52200
H	28.73400	-19.03100	-5.57500
H	27.53700	-18.14300	-5.84400
O	40.34700	-6.40100	1.28300
H	40.22200	-7.20200	0.77400
H	41.00500	-5.90700	0.79500
O	31.96800	-20.04600	0.46100
H	31.24700	-19.43300	0.32200
H	32.75700	-19.51100	0.38300
O	42.41100	-8.60000	2.91100
H	41.46400	-8.72000	2.83900
H	42.75900	-9.48800	2.99400
O	34.77100	-22.71100	6.73500
H	35.29400	-22.46500	7.49800
H	34.55200	-21.87900	6.31500
O	30.16700	-20.44100	-5.39800
H	29.83900	-21.32100	-5.58000
H	30.52100	-20.49600	-4.51000
O	39.41500	-8.80500	-0.88800
H	39.26800	-9.73300	-0.70800
H	40.24100	-8.78300	-1.37300
O	33.28200	-20.15300	-5.03000
H	33.62100	-19.62500	-5.75300
H	33.72900	-19.81500	-4.25500
O	35.97600	-21.77000	4.42000
H	35.63600	-22.56900	4.82200
H	35.27000	-21.46900	3.84800
O	36.62800	-24.68500	2.87300
H	37.45000	-25.15900	2.75100
H	36.87100	-23.90600	3.37300
O	35.26800	-21.65600	-6.83100
H	35.98900	-21.27800	-7.33500
H	34.64500	-20.93700	-6.72800
O	39.41100	-20.95100	-5.89500
H	39.62800	-20.06900	-6.19600
H	38.93100	-21.34300	-6.62500
O	34.15100	-17.99300	0.68200
H	34.00600	-17.67900	1.57400
H	33.67400	-17.37400	0.12900
O	36.01100	-21.00300	0.73000
H	35.99700	-21.89800	0.39300
H	36.72800	-20.99500	1.36400
O	37.86400	-20.56900	2.77500
H	37.60500	-19.64700	2.77400
H	37.33200	-20.96600	3.46500
O	28.62600	-15.45200	3.62100
H	28.08100	-14.67600	3.75300
H	29.50400	-15.10500	3.46300
O	35.41500	-22.12100	-4.00100
H	35.53600	-23.04900	-3.80200
H	35.12500	-22.10700	-4.91300
O	38.28400	-24.43600	-0.86500
H	38.30600	-24.37200	-1.81900
H	38.70100	-25.27500	-0.67100
O	29.41300	-16.32500	6.51400
H	28.67100	-16.17700	7.10000
H	29.09200	-16.06400	5.65000
O	37.35700	-6.78400	6.31200
H	37.66600	-7.02900	7.18400
H	38.15400	-6.57900	5.82400

O	37.37900	-4.63000	-1.45800
H	36.93900	-5.12200	-0.76500
H	38.13000	-5.17300	-1.69900
O	29.85800	-8.43900	-1.45500
H	30.68400	-8.73800	-1.83500
H	29.50800	-9.20800	-1.00600
O	34.82600	-9.08600	0.61400
H	34.20400	-9.74900	0.31500
H	35.49700	-9.06000	-0.06800
O	28.05900	-10.52000	-0.49600
H	27.22200	-10.94800	-0.67800
H	28.03100	-10.32700	0.44100
O	34.89900	-20.60800	10.20800
H	34.94800	-19.73200	9.82500
H	35.44100	-21.15000	9.63500
O	34.38800	-10.53600	-5.91000
H	34.53700	-9.85200	-6.56300
H	35.19400	-10.55500	-5.39500
O	37.12900	-19.85500	6.96200
H	36.25900	-19.56100	6.69100
H	37.36500	-19.27100	7.68300
O	32.14300	-14.50900	-4.90400
H	31.89800	-15.41300	-4.70600
H	32.31300	-14.50700	-5.84600
O	39.83300	-23.63700	4.47900
H	40.35100	-22.90100	4.15300
H	39.47800	-23.33000	5.31400
O	31.41300	-18.77600	-7.40500
H	30.71900	-18.70200	-8.06100
H	31.16800	-19.54000	-6.88300
O	40.08400	-27.21700	-0.00100
H	40.36400	-27.88400	0.62500
H	40.86900	-27.02400	-0.51400
O	37.88700	-8.04100	-8.43900
H	37.31500	-7.42200	-8.89200
H	38.46100	-7.49100	-7.90500
O	41.97600	-21.70900	3.93200
H	41.38100	-21.42900	3.23700
H	41.69100	-21.22000	4.70400
O	41.10700	-7.75700	-7.30800
H	42.04000	-7.82300	-7.10400
H	41.01900	-6.92100	-7.76600
O	38.27200	-16.70500	-11.09700
H	39.21800	-16.83400	-11.02800
H	37.89300	-17.44300	-10.62000
O	38.15600	-12.65300	10.36300
H	39.07600	-12.47500	10.55700
H	38.12100	-13.59700	10.21100
O	36.45600	-18.78000	-11.16100
H	35.54900	-19.07600	-11.08500
H	36.73400	-19.07700	-12.02800
O	27.37400	-17.45900	-0.71200
H	27.05400	-18.31300	-1.00400
H	26.82700	-17.24400	0.04400
O	31.15200	-14.74300	3.00500
H	31.37100	-13.81100	3.02400
H	31.56500	-15.10200	3.79100
O	38.07200	-24.23600	-5.00700
H	37.26300	-24.58100	-4.63200
H	38.76100	-24.54500	-4.41800
O	40.00200	-25.15800	-3.38500
H	40.55500	-24.73900	-2.72600
H	40.23900	-26.08500	-3.34200
O	36.02500	-19.17000	12.39600
H	35.92400	-18.22000	12.32700
H	35.80700	-19.49800	11.52300
O	35.22600	-21.92100	-11.02000
H	36.08800	-21.59600	-10.76100
H	35.41100	-22.70800	-11.53300
O	27.24300	-13.83900	1.25800
H	26.54900	-14.25300	0.74500
H	27.99000	-13.79700	0.66100
O	41.83000	-23.64800	-1.67500
H	42.33000	-23.53600	-2.48300
H	42.47300	-23.95200	-1.03500
O	35.88800	-15.74000	11.21200
H	35.40500	-14.94000	11.41900
H	35.97400	-15.72900	10.25900
O	29.15700	-13.90700	-0.86600
H	29.13400	-13.80800	-1.81700
H	29.46700	-13.06000	-0.54400
O	31.81500	-11.84900	3.24900
H	32.77000	-11.88100	3.18200
H	31.61700	-12.31000	4.06400
O	32.40500	-13.27900	5.81600
H	33.22500	-12.78500	5.84000
H	32.67900	-14.19600	5.78800
O	42.16200	-20.88800	-4.82100
H	41.27400	-20.60300	-5.03900
H	42.57700	-21.04900	-5.66800
O	30.17000	-6.30700	0.26600
H	30.03600	-7.06700	-0.30000
H	29.75400	-5.58300	-0.20100
O	41.30600	-11.12400	-10.72600
H	42.01000	-10.91900	-10.11100
H	40.58200	-10.55600	-10.46100
O	42.13600	-15.37300	-4.60600
H	42.07900	-14.91600	-5.44500
H	42.68900	-16.13400	-4.78400
O	33.32700	-5.41000	0.47800
H	33.21000	-4.46600	0.37600
H	32.50600	-5.71300	0.86600
O	42.86900	-14.18200	-7.31100
H	42.81400	-14.34800	-8.25200
H	42.24400	-13.47500	-7.15500
O	27.65900	-18.94500	6.51500
H	28.30500	-19.45600	7.00400
H	27.45000	-18.20900	7.09000
O	40.18400	-11.87700	12.01500
H	41.05300	-11.70300	11.65300
H	39.70500	-11.05800	11.89200
O	36.17900	-24.68100	-7.73200
H	36.16700	-23.73700	-7.57500
H	37.07100	-24.86400	-8.02600
O	34.35300	-4.75800	3.69300
H	35.25500	-4.72500	4.01000
H	34.12600	-5.68800	3.70800
O	36.10800	-6.00500	0.35400

H	35.15800	-6.00400	0.24100
H	36.41100	-6.75100	-0.16200
O	39.99400	-28.08700	-3.47800
H	39.22800	-28.64100	-3.33200
H	40.40500	-28.01500	-2.61600
O	31.99800	-21.79900	10.61300
H	32.86200	-22.15500	10.82300
H	31.42500	-22.13700	11.30000
O	42.05700	3.13300	0.18300
H	42.85000	2.79200	-0.23200
H	42.29700	3.24200	1.10400
O	41.62600	-0.49800	0.12500
H	41.75500	-0.24400	1.03900
H	40.94600	0.09500	-0.19500
O	40.57300	-3.02600	0.17000
H	39.78300	-2.77000	-0.30600
H	41.06400	-2.21100	0.27500
O	42.52500	-2.66100	-2.87200
H	41.72800	-2.54900	-2.35300
H	42.29700	-2.32200	-3.73800
O	38.85700	-2.77200	2.60100
H	39.10800	-1.88800	2.86700
H	39.39300	-2.95200	1.82900
O	41.52300	3.04800	-4.48300
H	42.03500	3.84200	-4.33200
H	42.17500	2.35000	-4.54900
O	42.61800	-1.78900	7.47600
H	42.50300	-2.69100	7.77600
H	41.76200	-1.54300	7.12400
O	39.48800	1.37700	-3.70700
H	40.32500	1.78600	-3.92400
H	39.66600	0.43700	-3.73700
O	39.51000	-1.28400	-3.03600
H	39.31300	-1.87700	-3.76100
H	38.95500	-1.58900	-2.31800
O	42.00500	-1.75800	-5.35200
H	41.07500	-1.81900	-5.13500
H	42.10100	-2.28000	-6.14800
O	41.92300	-5.23200	-2.76400
H	42.18400	-4.31800	-2.87300
H	42.74500	-5.70000	-2.61400
O	36.07000	0.40100	-0.86900
H	36.75700	0.08700	-1.45700
H	35.91500	1.30500	-1.14200
O	40.87400	-4.71000	3.55900
H	40.79000	-5.31800	2.82500
H	40.05200	-4.21900	3.56000
O	41.05500	5.91000	2.68200
H	41.64100	5.16500	2.81500
H	40.85800	6.22100	3.56600
O	35.64700	-1.51900	1.40300
H	35.68600	-2.05300	2.19600
H	35.14100	-2.04600	0.78400
O	39.27800	-2.79300	-5.52200
H	39.57300	-3.70200	-5.57500
H	38.37700	-2.81400	-5.84400
O	41.00700	-5.11100	-5.39800
H	40.67800	-5.99200	-5.58000
H	41.36100	-5.16700	-4.51000
O	39.46500	-0.12300	3.62100
H	38.92100	0.65300	3.75300
H	40.34300	0.22400	3.46300
O	40.25200	-0.99600	6.51400
H	39.51000	-0.84800	7.10000
H	39.93100	-0.73500	5.65000
O	40.69800	6.89100	-1.45500
H	41.52300	6.59100	-1.83500
H	40.34700	6.12100	-1.00600
O	38.89800	4.80900	-0.49600
H	38.06200	4.38100	-0.67800
H	38.87100	5.00200	0.44100
O	42.25200	-3.44700	-7.40500
H	41.55900	-3.37300	-8.06100
H	42.00700	-4.21100	-6.88300
O	38.21300	-2.13000	-0.71200
H	37.89400	-2.98300	-1.00400
H	37.66700	-1.91500	0.04400
O	41.99100	0.58600	3.00500
H	42.21000	1.51800	3.02400
H	42.40400	0.22700	3.79100
O	38.08200	1.49000	1.25800
H	37.38900	1.07600	0.74500
H	38.83000	1.53200	0.66100
O	39.99600	1.42200	-0.86600
H	39.97300	1.52100	-1.81700
H	40.30600	2.26900	-0.54400
O	41.01000	9.02200	0.26600
H	40.87500	8.26200	-0.30000
H	40.59300	9.74600	-0.20100
O	38.49800	-3.61600	6.51500
H	39.14400	-4.12600	7.00400
H	38.29000	-2.87900	7.09000
O	34.95700	-28.15100	-17.05200
H	34.87900	-27.20000	-16.97300
H	34.35300	-28.49600	-16.39400
O	32.49300	-32.15900	-15.67600
H	32.67000	-31.23700	-15.86400
H	33.28900	-32.47300	-15.24700
O	33.61600	-30.64100	-9.67500
H	34.16700	-30.76900	-10.44700
H	32.77700	-30.34100	-10.02500
O	36.74100	-31.10100	-14.49900
H	36.69800	-30.87500	-15.42800
H	37.65700	-31.33500	-14.35100
O	38.02900	-28.55100	-19.68100
H	38.20400	-29.02300	-18.86600
H	37.34300	-27.92500	-19.45300
O	32.59500	-25.40200	-12.90700
H	32.83500	-24.77100	-12.22900
H	31.75200	-25.75400	-12.62000
O	37.10800	-29.59500	-10.50300
H	37.03500	-28.78900	-9.99100
H	36.66600	-29.39500	-11.32800
O	36.89700	-30.35700	-16.99200
H	36.11400	-29.81500	-16.89900
H	37.59600	-29.84000	-16.59300
O	39.56800	-29.69600	-8.34700
H	40.28100	-29.06100	-8.41900

H	39.07700	-29.59900	-9.16300
O	34.61900	-31.94600	-7.52600
H	35.32000	-31.31300	-7.37300
H	34.14900	-31.60500	-8.28700
O	39.38000	-29.35600	-15.59800
H	39.56200	-30.17400	-15.13500
H	40.20300	-29.13900	-16.03600
O	39.71400	-30.42900	-18.33700
H	40.24300	-30.94000	-18.95000
H	39.97700	-30.74500	-17.47200
O	30.21600	-32.41300	-10.59300
H	30.80100	-33.15800	-10.46000
H	30.01800	-32.10200	-9.71000
O	33.07000	-29.37800	-15.57700
H	32.40100	-28.89300	-16.06100
H	32.93100	-29.13100	-14.66300
O	37.21000	-27.34300	-8.97900
H	37.76200	-27.18000	-8.21400
H	37.55100	-26.75300	-9.65100
O	40.34700	-29.39500	-11.99200
H	40.22200	-30.19600	-12.50100
H	41.00500	-28.90000	-12.48100
O	37.35700	-29.77700	-6.96400
H	37.66600	-30.02300	-6.09200
H	38.15400	-29.57300	-7.45200
O	37.37900	-27.62400	-14.73300
H	36.93900	-28.11600	-14.04000
H	38.13000	-28.16600	-14.97400
O	29.85800	-31.43200	-14.73100
H	30.68400	-31.73200	-15.11100
H	29.50800	-32.20200	-14.28200
O	34.82600	-32.08000	-12.66100
H	34.20400	-32.74300	-12.96100
H	35.49700	-32.05400	-13.34400
O	28.05900	-33.51400	-13.77200
H	27.22200	-33.94200	-13.95400
H	28.03100	-33.32100	-12.83500
O	30.17000	-29.30100	-13.00900
H	30.03600	-30.06100	-13.57600
H	29.75400	-28.57700	-13.47700
O	33.32700	-28.40400	-12.79800
H	33.21000	-27.46000	-12.90000
H	32.50600	-28.70700	-12.40900
O	34.35300	-27.75100	-9.58200
H	35.25500	-27.71900	-9.26500
H	34.12600	-28.68100	-9.56700
O	36.10800	-28.99800	-12.92100
H	35.15800	-28.99800	-13.03500
H	36.41100	-29.74500	-13.43700
O	42.05700	-19.86100	-13.09200
H	42.85000	-20.20200	-13.50800
H	42.29700	-19.75100	-12.17200
O	41.62600	-23.49200	-13.15100
H	41.75500	-23.23800	-12.23700
H	40.94600	-22.89900	-13.47000
O	40.57300	-26.01900	-13.10600
H	39.78300	-25.76400	-13.58200
H	41.06400	-25.20400	-13.00000
O	42.52500	-25.65500	-16.14700
H	41.72800	-25.54300	-15.62900
H	42.29700	-25.31600	-17.01300
O	38.85700	-25.76600	-10.67400
H	39.10800	-24.88200	-10.40800
H	39.39300	-25.94600	-11.44700
O	41.52300	-19.94600	-17.75800
H	42.03500	-19.15200	-17.60800
H	42.17500	-20.64300	-17.82500
O	42.61800	-24.78300	-5.80000
H	42.50300	-25.68400	-5.49900
H	41.76200	-24.53700	-6.15100
O	39.48800	-21.61700	-16.98200
H	40.32500	-21.20700	-17.19900
H	39.66600	-22.55700	-17.01200
O	39.51000	-24.27800	-16.31100
H	39.31300	-24.87100	-17.03600
H	38.95500	-24.58300	-15.59400
O	42.00500	-24.75200	-18.62700
H	41.07500	-24.81300	-18.41000
H	42.10100	-25.27400	-19.42400
O	41.92300	-28.22600	-16.03900
H	42.18400	-27.31200	-16.14800
H	42.74500	-28.69400	-15.89000
O	36.07000	-22.59300	-14.14400
H	36.75700	-22.90700	-14.73200
H	35.91500	-21.68900	-14.41800
O	40.87400	-27.70400	-9.71600
H	40.79000	-28.31100	-10.45100
H	40.05200	-27.21300	-9.71500
O	41.05500	-17.08400	-10.59300
H	41.64100	-17.82900	-10.46000
H	40.85800	-16.77300	-9.71000
O	35.64700	-24.51300	-11.87300
H	35.68600	-25.04700	-11.07900
H	35.14100	-25.03900	-12.49200
O	39.27800	-25.78700	-18.79700
H	39.57300	-26.69600	-18.85100
H	38.37700	-25.80800	-19.11900
O	41.00700	-28.10500	-18.67300
H	40.67800	-28.98500	-18.85600
H	41.36100	-28.16000	-17.78600
O	39.46500	-23.11700	-9.65500
H	38.92100	-22.34100	-9.52300
H	40.34300	-22.77000	-9.81300
O	40.25200	-23.99000	-6.76200
H	39.51000	-23.84200	-6.17500
H	39.93100	-23.72900	-7.62500
O	40.69800	-16.10300	-14.73100
H	41.52300	-16.40300	-15.11100
H	40.34700	-16.87300	-14.28200
O	38.89800	-18.18500	-13.77200
H	38.06200	-18.61200	-13.95400
H	38.87100	-17.99100	-12.83500
O	38.21300	-25.12400	-13.98700
H	37.89400	-25.97700	-14.28000
H	37.66700	-24.90900	-13.23100
O	41.99100	-22.40700	-10.27000
H	42.21000	-21.47600	-10.25100
H	42.40400	-22.76700	-9.48500

O	38.08200	-21.50300	-12.01800
H	37.38900	-21.91800	-12.53100
H	38.83000	-21.46100	-12.61400
O	39.99600	-21.57200	-14.14100
H	39.97300	-21.47300	-15.09300
H	40.30600	-20.72500	-13.81900
O	41.01000	-13.97200	-13.00900
H	40.87500	-14.73200	-13.57600
H	40.59300	-13.24800	-13.47700
O	38.49800	-26.61000	-6.76000
H	39.14400	-27.12000	-6.27100
H	38.29000	-25.87300	-6.18600
O	-36.62400	-2.36000	32.48300
H	-37.27500	-1.77400	32.09600
H	-35.83000	-1.82900	32.54200
O	-35.14700	-4.67100	33.02000
H	-35.78200	-4.59700	33.73200
H	-35.47700	-4.08100	32.34200
O	-32.98100	-9.79100	33.22900
H	-32.06900	-9.55800	33.05400
H	-33.49000	-9.20700	32.66700
O	-38.64500	-2.13400	34.77100
H	-38.78100	-1.28900	35.19900
H	-38.10400	-1.93200	34.00700
O	-35.33100	-8.62000	31.80800
H	-35.84600	-7.85000	32.04700
H	-35.60300	-9.29400	32.43100
O	-30.35700	-5.86700	33.74300
H	-31.13400	-6.20600	33.29900
H	-29.67800	-5.86200	33.06900
O	-38.95200	-6.77100	28.28700
H	-39.86000	-7.00200	28.48600
H	-38.45500	-7.56700	28.47400
O	-36.04600	1.93900	31.20600
H	-36.71900	2.24200	31.81500
H	-35.65900	2.74200	30.85700
O	-32.54200	-3.05900	36.57800
H	-32.99500	-3.90000	36.63500
H	-33.17200	-2.42300	36.91600
O	-34.09800	-1.22100	37.51300
H	-33.80600	-0.66100	36.79400
H	-33.58800	-0.92800	38.26900
O	-38.07600	-5.12900	30.14800
H	-38.51400	-5.84500	29.68800
H	-37.33500	-4.90100	29.58600
O	-29.95900	-6.85300	36.20600
H	-30.09700	-6.66300	35.27800
H	-30.31500	-6.09100	36.66200
O	-37.84600	2.10700	33.42100
H	-37.67100	1.63600	34.23600
H	-38.53300	2.73400	33.64900
O	-33.95500	-6.27000	29.74100
H	-34.57200	-6.99500	29.64400
H	-34.42300	-5.50900	29.39800
O	-38.97900	0.30100	36.10900
H	-39.76200	0.84300	36.20300
H	-38.27900	0.81800	36.50900
O	-33.13100	-2.94600	33.86000
H	-32.85500	-3.06100	34.77000
H	-34.00100	-3.34400	33.82200
O	-36.49600	1.30200	37.50400
H	-36.31300	0.48500	37.96600
H	-35.67200	1.51900	37.06600
O	-36.16100	0.22900	34.76500
H	-35.63300	-0.28200	34.15200
H	-35.89900	-0.08700	35.63000
O	-33.06700	0.20100	35.38100
H	-32.89400	-0.36400	34.62800
H	-32.57400	1.00100	35.19900
O	-27.21300	-7.03500	37.00000
H	-26.77000	-7.78500	36.60200
H	-28.09100	-7.04400	36.61700
O	-32.08000	-0.48200	32.48000
H	-31.38500	0.13800	32.69900
H	-31.73800	-1.33300	32.75600
O	-30.18100	-3.03200	34.00900
H	-29.65600	-2.69400	33.28400
H	-30.26800	-3.96800	33.82500
O	-37.98800	-0.37600	31.38800
H	-38.56100	0.24300	30.93500
H	-37.41500	0.17400	31.92200
O	-34.76900	-0.09300	32.51900
H	-33.83600	-0.15800	32.72200
H	-34.85600	0.74300	32.06000
O	-32.14000	-0.77600	29.47900
H	-32.21800	-1.11000	30.37300
H	-31.31900	-0.28400	29.47800
O	-37.60400	-9.04000	28.72900
H	-36.65800	-9.17000	28.79800
H	-37.98200	-9.77900	29.20600
O	-39.41900	-11.11500	28.66500
H	-40.32600	-11.41200	28.74100
H	-39.14200	-11.41200	27.79800
O	-34.56900	-3.45900	29.10000
H	-33.86600	-3.25400	29.71600
H	-35.29300	-2.89100	29.36500
O	-32.66100	-3.02100	31.15000
H	-31.82600	-3.45300	30.96800
H	-32.82200	-3.19300	32.07800
O	-33.00600	-6.51800	32.51600
H	-33.06100	-6.68300	31.57400
H	-33.63200	-5.81000	32.67200
O	-24.03200	7.49600	41.24500
H	-23.39400	7.37700	40.54200
H	-24.15000	8.44400	41.30400
O	-27.22300	7.44900	41.18200
H	-27.56200	7.33300	40.29400
H	-26.35400	7.04800	41.16200
O	-31.36500	9.30200	37.17100
H	-31.92800	9.21100	36.40300
H	-31.47900	8.48200	37.65200
O	-25.78500	12.96900	32.48300
H	-26.43600	13.55500	32.09600
H	-24.99000	13.50000	32.54200
O	-31.85900	10.05800	32.88100
H	-31.08600	9.51100	32.74300
H	-31.50700	10.92300	33.09300
O	-29.52300	6.21800	30.51300

H	-29.27000	5.69600	29.75200
H	-30.46000	6.05400	30.61600
O	-24.47800	5.22100	38.32100
H	-23.69200	4.80300	37.97000
H	-24.21400	6.12600	38.48900
O	-24.30700	10.65900	33.02000
H	-24.94300	10.73200	33.73200
H	-24.63700	11.24800	32.34200
O	-19.07700	5.15900	39.22400
H	-18.34500	4.58900	39.45800
H	-19.83300	4.77100	39.66500
O	-29.80300	8.12000	32.71600
H	-29.91900	7.44500	33.38400
H	-29.69300	7.63400	31.89900
O	-27.46000	7.58100	36.42000
H	-27.82400	7.05500	37.13200
H	-26.60300	7.19100	36.24700
O	-27.81900	2.61000	31.41700
H	-27.65200	3.47800	31.78400
H	-28.00900	2.77300	30.49300
O	-21.66200	3.59000	40.76300
H	-22.57000	3.52200	40.46900
H	-21.71300	4.06800	41.59100
O	-27.84000	5.06600	32.40100
H	-27.96000	5.51200	33.24000
H	-28.53500	5.41500	31.84300
O	-19.79600	1.79200	42.54100
H	-20.43800	2.08500	41.89500
H	-20.30000	1.67700	43.34600
O	-22.14200	5.53800	33.22900
H	-21.23000	5.77100	33.05400
H	-22.65100	6.12200	32.66700
O	-23.53500	10.52100	40.62100
H	-24.16400	10.68600	39.91800
H	-23.92200	10.93500	41.39200
O	-19.65600	11.45600	37.97500
H	-20.34100	11.83600	37.42500
H	-19.57200	12.06600	38.70800
O	-22.40100	9.10200	44.57500
H	-22.18900	9.74600	43.89800
H	-22.44800	8.26900	44.10700
O	-24.79800	1.71400	41.47800
H	-25.66000	2.01200	41.77000
H	-24.77200	1.93100	40.54600
O	-27.98200	10.25800	37.88600
H	-27.51800	10.53700	37.09600
H	-28.24600	9.35700	37.70300
O	-29.04500	6.38100	34.69500
H	-28.34300	6.83300	35.16200
H	-29.37700	5.73900	35.32300
O	-21.28600	11.98000	42.74700
H	-20.46400	11.57800	42.46500
H	-21.29000	11.87600	43.69800
O	-33.35100	5.00400	36.95500
H	-34.14800	5.11500	37.47300
H	-33.57900	5.34200	36.08900
O	-13.19600	8.32300	39.09500
H	-13.94900	7.75400	38.94000
H	-13.06100	8.29000	40.04200
O	-27.80600	13.19500	34.77100
H	-27.94100	14.04000	35.19900
H	-27.26500	13.39700	34.00700
O	-34.35300	10.71200	35.34400
H	-33.84000	11.50700	35.49400
H	-33.70100	10.01500	35.27700
O	-24.49100	6.70900	31.80800
H	-25.00700	7.47900	32.04700
H	-24.76400	6.03500	32.43100
O	-26.90700	1.55400	36.86600
H	-27.76300	1.16400	36.68700
H	-26.49400	1.63300	36.00600
O	-25.37700	-0.91200	41.26600
H	-25.88000	-0.94900	40.45200
H	-25.08400	-0.00200	41.32500
O	-19.51800	9.46200	33.74300
H	-20.29500	9.12300	33.29900
H	-18.83900	9.46700	33.06900
O	-24.70300	4.58100	33.63400
H	-25.00800	4.89600	34.48500
H	-23.75000	4.66000	33.67700
O	-26.25800	10.74000	35.28300
H	-26.78600	10.00600	34.97100
H	-26.75900	11.51800	35.03700
O	-21.27900	8.93500	40.40000
H	-21.93700	9.62500	40.48800
H	-20.47500	9.32500	40.74300
O	-31.52600	1.40200	37.64400
H	-30.95200	1.18000	36.91100
H	-30.93900	1.47100	38.39700
O	-22.46800	7.29000	38.45000
H	-21.95500	7.92300	38.95200
H	-21.81800	6.69000	38.08500
O	-31.19900	3.43300	31.66300
H	-32.03600	3.77000	31.98400
H	-31.33900	3.29800	30.72600
O	-29.22400	-1.72300	36.37100
H	-29.87800	-2.03900	35.74700
H	-29.26600	-2.34600	37.09700
O	-28.11300	8.55800	28.28700
H	-29.02000	8.32700	28.48600
H	-27.61600	7.76200	28.47400
O	-25.20600	17.26800	31.20600
H	-25.88000	17.57100	31.81500
H	-24.82000	18.07100	30.85700
O	-25.99000	4.76500	40.69600
H	-26.90800	4.98600	40.53800
H	-25.57400	4.83900	39.83700
O	-25.20700	6.16400	35.82600
H	-24.46000	6.67600	35.51700
H	-24.93800	5.84200	36.68700
O	-26.93400	8.66000	32.80900
H	-27.84300	8.37600	32.90600
H	-26.90500	9.08000	31.94900
O	-21.70200	12.27000	36.57800
H	-22.15600	11.42900	36.63500
H	-22.33300	12.90700	36.91600
O	-22.75900	3.20000	37.80800
H	-23.49700	2.68700	38.13900

H	-22.50600	2.76000	36.99700
O	-23.25900	14.10800	37.51300
H	-22.96700	14.66900	36.79400
H	-22.74900	14.40100	38.26900
O	-22.65800	-4.02300	38.40200
H	-22.35200	-4.88300	38.11500
H	-21.93200	-3.43100	38.20200
O	-28.38600	6.27300	38.91000
H	-28.33100	5.32000	38.83400
H	-29.19700	6.42900	39.39400
O	-27.23700	10.20100	30.14800
H	-27.67500	9.48400	29.68800
H	-26.49500	10.42800	29.58600
O	-30.44600	4.89000	36.66900
H	-31.26000	5.19000	37.07600
H	-29.95000	4.49600	37.38700
O	-19.11900	8.47600	36.20600
H	-19.25700	8.66600	35.27800
H	-19.47600	9.23800	36.66200
O	-19.09200	10.54400	41.21700
H	-18.65900	10.45200	42.06500
H	-18.37900	10.52000	40.57900
O	-30.17900	11.76200	38.39900
H	-29.38500	11.26700	38.19700
H	-30.89000	11.23400	38.03600
O	-33.87100	5.90700	34.47500
H	-34.80100	5.84500	34.69200
H	-33.77500	5.38400	33.67800
O	-33.95200	2.43200	37.06300
H	-33.69100	3.34700	36.95300
H	-33.13000	1.96500	37.21200
O	-18.23100	10.58600	43.75000
H	-18.03700	11.49100	43.99600
H	-18.33100	10.12700	44.58400
O	-27.00700	17.43600	33.42100
H	-26.83200	16.96500	34.23600
H	-27.69400	18.06300	33.64900
O	-26.05100	11.71300	39.46700
H	-26.38600	11.67400	40.36300
H	-26.55100	11.04800	38.99400
O	-23.11500	9.05900	29.74100
H	-23.73200	8.33400	29.64400
H	-23.58400	9.82000	29.39800
O	-31.32600	3.27800	28.86300
H	-32.20300	3.15600	28.49900
H	-30.85000	2.48600	28.61100
O	-28.13900	15.63000	36.10900
H	-28.92200	16.17200	36.20300
H	-27.44000	16.14800	36.50900
O	-24.82900	1.44000	38.75300
H	-24.33300	0.68700	38.43300
H	-25.60100	1.47600	38.18700
O	-16.97700	7.14400	40.39500
H	-16.98800	8.02600	40.02400
H	-17.84500	6.79100	40.20000
O	-23.74700	10.19700	36.32700
H	-24.68100	10.31700	36.15700
H	-23.56900	9.29400	36.06300
O	-28.24600	3.58100	38.48000
H	-28.61600	3.02900	39.16800
H	-27.75000	2.97700	37.92800
O	-20.35900	6.03900	36.86200
H	-19.89300	6.87100	36.78800
H	-19.90800	5.57300	37.56600
O	-22.29200	12.38300	33.86000
H	-22.01600	12.26900	34.77000
H	-23.16100	11.98500	33.82200
O	-22.38800	-1.59300	40.90100
H	-23.30100	-1.36300	41.06900
H	-21.95800	-1.51000	41.75200
O	-22.61700	6.59600	43.40100
H	-23.27000	6.84300	42.74600
H	-22.23900	5.78200	43.06700
O	-25.65600	16.63200	37.50400
H	-25.47400	15.81400	37.96600
H	-24.83300	16.84800	37.06600
O	-25.32200	15.55800	34.76500
H	-24.79300	15.04800	34.15200
H	-25.05900	15.24300	35.63000
O	-36.59800	4.87200	34.30500
H	-36.30200	3.96300	34.25100
H	-37.49900	4.85100	33.98300
O	-33.06800	2.94700	40.28700
H	-33.79000	3.56100	40.14800
H	-32.27900	3.48300	40.20900
O	-34.86900	2.55300	34.42800
H	-35.19800	1.67300	34.24600
H	-34.51500	2.49800	35.31600
O	-25.62100	14.18900	38.93800
H	-25.76800	13.26000	39.11900
H	-24.79600	14.21100	38.45400
O	-31.75500	2.84000	34.79600
H	-31.41500	3.36900	34.07400
H	-31.30700	3.17900	35.57200
O	-22.01800	15.01200	40.02400
H	-21.09300	14.80600	39.88700
H	-22.18600	14.76300	40.93300
O	-29.76800	1.33800	32.99500
H	-29.04700	1.71600	32.49200
H	-30.39100	2.05700	33.09900
O	-25.62500	2.04200	33.93100
H	-25.40800	2.92500	33.63000
H	-26.10500	1.65100	33.20200
O	-30.88500	5.00100	40.50800
H	-31.03000	5.31500	41.40100
H	-31.36200	5.62000	39.95500
O	-29.02500	1.99100	40.55600
H	-29.03900	1.09500	40.22000
H	-28.30800	1.99800	41.19000
O	-22.22800	15.53000	35.38100
H	-22.05400	14.96500	34.62800
H	-21.73500	16.33000	35.19900
O	-29.62200	0.87300	35.82500
H	-29.50000	-0.05600	36.02400
H	-29.91100	0.88700	34.91300
O	-26.75200	-1.44200	38.96200
H	-26.73000	-1.37800	38.00700
H	-26.33600	-2.28100	39.15600

O	-16.37400	8.29400	37.00000
H	-15.93100	7.54400	36.60200
H	-17.25100	8.28500	36.61700
O	-21.24000	14.84700	32.48000
H	-20.54500	15.46700	32.69900
H	-20.89800	13.99600	32.75600
O	-19.34100	12.29700	34.00900
H	-18.81600	12.63500	33.28400
H	-19.42900	11.36100	33.82500
O	-19.53200	9.77900	46.62700
H	-19.48200	9.61400	47.56900
H	-19.64100	8.91200	46.23700
O	-30.64900	12.45800	33.91700
H	-30.50000	13.14200	33.26300
H	-29.84200	12.43900	34.43200
O	-18.03000	12.19900	46.87200
H	-18.32200	12.56200	47.70800
H	-18.64300	11.48600	46.69300
O	-16.83100	9.76400	39.39200
H	-15.99600	10.20300	39.55600
H	-16.69500	9.28800	38.57300
O	-19.78200	7.32200	45.34000
H	-19.30600	7.51500	44.53200
H	-20.70500	7.37800	45.09400
O	-32.89300	8.48500	34.92300
H	-33.13900	7.58100	35.12100
H	-32.72400	8.48600	33.98100
O	-33.62400	4.21700	32.42100
H	-34.31700	4.29200	31.76500
H	-33.86900	3.45400	32.94400
O	-27.14900	14.95300	31.38800
H	-27.72200	15.57200	30.93500
H	-26.57600	15.50300	31.92200
O	-21.08100	4.19900	43.57300
H	-20.13000	4.29900	43.59400
H	-21.25100	3.40700	44.08200
O	-23.92900	15.23700	32.51900
H	-22.99600	15.17100	32.72200
H	-24.01700	16.07200	32.06000
O	-21.30000	14.55300	29.47900
H	-21.37900	14.21900	30.37300
H	-20.47900	15.04500	29.47800
O	-18.60300	7.41900	42.68700
H	-18.63500	6.48100	42.87300
H	-17.99300	7.49800	41.95300
O	-26.76400	6.28900	28.72900
H	-25.81800	6.16000	28.79800
H	-27.14300	5.55000	29.20600
O	-18.45300	4.21100	42.73300
H	-18.96900	3.42600	42.54600
H	-17.56900	3.88200	42.89700
O	-20.79200	-2.04500	38.69200
H	-20.99000	-2.35400	39.57600
H	-20.18200	-1.31900	38.82200
O	-12.78500	8.48100	41.82700
H	-12.05500	8.22000	42.38800
H	-13.39400	7.74400	41.86800
O	-14.70300	6.62200	42.42500
H	-15.43000	6.48700	41.81600
H	-15.11900	6.92900	43.23000
O	-28.58000	4.21400	28.66500
H	-29.48700	3.91700	28.74100
H	-28.30200	3.91700	27.79800
O	-26.96400	-1.24200	34.81900
H	-27.77400	-1.58800	35.19500
H	-26.27500	-1.55200	35.40800
O	-25.03500	-2.16400	36.44200
H	-24.48200	-1.74500	37.10100
H	-24.79800	-3.09100	36.48400
O	-29.81000	1.07300	28.80600
H	-28.94800	1.39800	29.06600
H	-29.62500	0.28600	28.29300
O	-21.65700	-0.05400	35.95000
H	-22.23700	0.60400	35.56700
H	-21.92300	-0.87700	35.53800
O	-23.20700	-0.65400	38.15200
H	-22.70700	-0.54200	37.34300
H	-22.56300	-0.95800	38.79200
O	-22.87400	2.10600	35.00500
H	-23.76200	2.39100	34.78700
H	-22.45900	1.94500	34.15800
O	-19.52300	2.13000	38.72100
H	-18.74700	2.62000	38.99300
H	-20.24800	2.55600	39.17800
O	-17.08600	2.84300	39.77800
H	-16.49100	2.60300	39.06700
H	-17.01300	2.12400	40.40500
O	-23.73000	11.87000	29.10000
H	-23.02600	12.07500	29.71600
H	-24.45400	12.43800	29.36500
O	-22.90000	7.62100	35.22000
H	-22.95700	8.07800	34.38100
H	-22.34700	6.86000	35.04300
O	-19.09000	3.41000	36.04500
H	-19.34600	3.01000	36.87600
H	-19.71500	4.12400	35.92000
O	-14.77900	6.15000	38.55200
H	-15.33900	6.21900	39.32600
H	-15.38400	5.97400	37.83200
O	-21.82100	12.30800	31.15000
H	-20.98700	11.87600	30.96800
H	-21.98300	12.13700	32.07800
O	-22.16700	8.81200	32.51600
H	-22.22200	8.64600	31.57400
H	-22.79200	9.51900	32.67200
O	-28.85700	-1.68800	32.09400
H	-28.86900	-0.74300	32.25100
H	-27.96500	-1.87100	31.80000
O	-25.04200	-5.09300	36.34900
H	-25.80900	-5.64800	36.49400
H	-24.63100	-5.02100	37.21000
O	-13.19300	22.82500	41.24500
H	-12.55500	22.70600	40.54200
H	-13.31100	23.77300	41.30400
O	-16.38400	22.77800	41.18200
H	-16.72200	22.66200	40.29400
H	-15.51500	22.37700	41.16200
O	-20.52500	24.63200	37.17100

H	-21.08900	24.54100	36.40300
H	-20.64000	23.81200	37.65200
O	-14.94500	28.29900	32.48300
H	-15.59700	28.88400	32.09600
H	-14.15100	28.82900	32.54200
O	-21.02000	25.38800	32.88100
H	-20.24700	24.84000	32.74300
H	-20.66700	26.25200	33.09300
O	-18.68300	21.54700	30.51300
H	-18.43100	21.02500	29.75200
H	-19.62100	21.38300	30.61600
O	-13.63900	20.55000	38.32100
H	-12.85300	20.13200	37.97000
H	-13.37500	21.45500	38.48900
O	-13.46800	25.98800	33.02000
H	-14.10300	26.06100	33.73200
H	-13.79800	26.57700	32.34200
O	-8.23800	20.48800	39.22400
H	-7.50500	19.91800	39.45800
H	-8.99400	20.10000	39.66500
O	-18.96400	23.45000	32.71600
H	-19.08000	22.77400	33.38400
H	-18.85300	22.96300	31.89900
O	-16.62000	22.91100	36.42000
H	-16.98500	22.38400	37.13200
H	-15.76400	22.52100	36.24700
O	-10.71500	14.20000	43.68400
H	-11.04700	15.07000	43.46000
H	-11.31100	13.88400	44.36300
O	-16.98000	17.94000	31.41700
H	-16.81200	18.80800	31.78400
H	-17.17000	18.10200	30.49300
O	-10.82300	18.92000	40.76300
H	-11.73100	18.85100	40.46900
H	-10.87400	19.39700	41.59100
O	-17.00100	20.39500	32.40100
H	-17.12100	20.84100	33.24000
H	-17.69600	20.74400	31.84300
O	-8.95600	17.12100	42.54100
H	-9.59900	17.41500	41.89500
H	-9.46100	17.00700	43.34600
O	-11.30200	20.86800	33.22900
H	-10.39100	21.10000	33.05400
H	-11.81100	21.45100	32.66700
O	-16.66500	20.22700	44.23900
H	-15.81900	20.65100	44.38200
H	-16.72500	19.57100	44.93300
O	-12.69500	25.85000	40.62100
H	-13.32500	26.01500	39.91800
H	-13.08300	26.26400	41.39200
O	-8.81700	26.78600	37.97500
H	-9.50100	27.16500	37.42500
H	-8.73200	27.39500	38.70800
O	-13.51500	17.88800	45.89700
H	-13.75900	18.64200	45.36000
H	-13.23900	18.26600	46.73200
O	-13.95900	17.04300	41.47800
H	-14.82000	17.34100	41.77000
H	-13.93300	17.26000	40.54600
O	-17.14300	25.58800	37.88600
H	-16.67900	25.86600	37.09600
H	-17.40700	24.68600	37.70300
O	-18.20600	21.71000	34.69500
H	-17.50300	22.16200	35.16200
H	-18.53800	21.06800	35.32300
O	-22.51200	20.33300	36.95500
H	-23.30900	20.44500	37.47300
H	-22.73900	20.67100	36.08900
O	-16.96600	28.52400	34.77100
H	-17.10200	29.36900	35.19900
H	-16.42500	28.72600	34.00700
O	-23.51300	26.04200	35.34400
H	-23.00100	26.83600	35.49400
H	-22.86100	25.34400	35.27700
O	-13.61700	12.04700	43.30100
H	-13.96100	12.93700	43.22500
H	-13.24900	11.85700	42.43800
O	-13.65200	22.03800	31.80800
H	-14.16700	22.80800	32.04700
H	-13.92400	21.36400	32.43100
O	-16.06700	16.88300	36.86600
H	-16.92300	16.49400	36.68700
H	-15.65500	16.96200	36.00600
O	-14.53700	14.41800	41.26600
H	-15.04100	14.38100	40.45200
H	-14.24400	15.32700	41.32500
O	-18.29300	14.50100	40.27200
H	-17.42800	14.30600	39.91300
H	-18.22500	14.27200	41.19900
O	-8.67900	24.79100	33.74300
H	-9.45600	24.45200	33.29900
H	-8.00000	24.79600	33.06900
O	-13.86300	19.91100	33.63400
H	-14.16800	20.22500	34.48500
H	-12.91000	19.98900	33.67700
O	-15.41900	26.07000	35.28300
H	-15.94700	25.33500	34.97100
H	-15.92000	26.84700	35.03700
O	-10.44000	24.26500	40.40000
H	-11.09700	24.95400	40.48800
H	-9.63600	24.65400	40.74300
O	-20.68700	16.73200	37.64400
H	-20.11300	16.50900	36.91100
H	-20.10000	16.80000	38.39700
O	-11.62900	22.61900	38.45000
H	-11.11600	23.25200	38.95200
H	-10.97900	22.01900	38.08500
O	-20.36000	18.76200	31.66300
H	-21.19600	19.10000	31.98400
H	-20.50000	18.62700	30.72600
O	-18.38500	13.60600	36.37100
H	-19.03800	13.29100	35.74700
H	-18.42700	12.98300	37.09700
O	-17.27400	23.88800	28.28700
H	-18.18100	23.65700	28.48600
H	-16.77600	23.09100	28.47400
O	-14.36700	32.59700	31.20600
H	-15.04100	32.90100	31.81500

H	-13.98000	33.40000	30.85700
O	-15.15100	20.09400	40.69600
H	-16.06900	20.31500	40.53800
H	-14.73500	20.16900	39.83700
O	-14.36700	21.49300	35.82600
H	-13.62000	22.00500	35.51700
H	-14.09800	21.17100	36.68700
O	-16.09500	23.98900	32.80900
H	-17.00400	23.70500	32.90600
H	-16.06500	24.40900	31.94900
O	-10.86300	27.60000	36.57800
H	-11.31700	26.75900	36.63500
H	-11.49300	28.23600	36.91600
O	-11.92000	18.52900	37.80800
H	-12.65700	18.01700	38.13900
H	-11.66700	18.08900	36.99700
O	-12.42000	29.43700	37.51300
H	-12.12700	29.99800	36.79400
H	-11.91000	29.73000	38.26900
O	-11.81800	11.30600	38.40200
H	-11.51200	10.44600	38.11500
H	-11.09300	11.89800	38.20200
O	-17.54600	21.60200	38.91000
H	-17.49100	20.64900	38.83400
H	-18.35700	21.75800	39.39400
O	-16.39700	25.53000	30.14800
H	-16.83500	24.81300	29.68800
H	-15.65600	25.75700	29.58600
O	-19.60700	20.21900	36.66900
H	-20.42000	20.51900	37.07600
H	-19.11100	19.82500	37.38700
O	-8.28000	23.80600	36.20600
H	-8.41800	23.99600	35.27800
H	-8.63600	24.56700	36.66200
O	-20.11600	17.79100	42.28500
H	-19.47600	17.65000	41.58800
H	-20.94800	17.50500	41.90900
O	-19.34000	27.09200	38.39900
H	-18.54600	26.59700	38.19700
H	-20.05000	26.56300	38.03600
O	-23.03100	21.23600	34.47500
H	-23.96200	21.17400	34.69200
H	-22.93600	20.71300	33.67800
O	-23.11300	17.76100	37.06300
H	-22.85200	18.67600	36.95300
H	-22.29100	17.29400	37.21200
O	-15.73800	15.80100	46.24900
H	-16.38500	16.50000	46.15500
H	-14.96800	16.23500	46.61700
O	-16.16800	32.76500	33.42100
H	-15.99200	32.29400	34.23600
H	-16.85400	33.39200	33.64900
O	-15.21200	27.04300	39.46700
H	-15.54700	27.00300	40.36300
H	-15.71200	26.37700	38.99400
O	-12.27600	24.38800	29.74100
H	-12.89300	23.66300	29.64400
H	-12.74400	25.14900	29.39800
O	-20.48600	18.60700	28.86300
H	-21.36300	18.48600	28.49900
H	-20.01000	17.81500	28.61100
O	-17.30000	30.95900	36.10900
H	-18.08300	31.50200	36.20300
H	-16.60000	31.47700	36.50900
O	-13.99000	16.76900	38.75300
H	-13.49300	16.01600	38.43300
H	-14.76100	16.80500	38.18700
O	-6.13700	22.47300	40.39500
H	-6.14800	23.35500	40.02400
H	-7.00500	22.12000	40.20000
O	-12.90700	25.52600	36.32700
H	-13.84100	25.64600	36.15700
H	-12.72900	24.62300	36.06300
O	-17.40700	18.91100	38.48000
H	-17.77700	18.35800	39.16800
H	-16.91100	18.30600	37.92800
O	-9.52000	21.36800	36.86200
H	-9.05400	22.20100	36.78800
H	-9.06900	20.90200	37.56600
O	-11.45200	27.71200	33.86000
H	-11.17600	27.59800	34.77000
H	-12.32200	27.31400	33.82200
O	-11.54800	13.73600	40.90100
H	-12.46200	13.96600	41.06900
H	-11.11900	13.82000	41.75200
O	-11.77800	21.92500	43.40100
H	-12.43000	22.17200	42.74600
H	-11.40000	21.11100	43.06700
O	-14.81700	31.96100	37.50400
H	-14.63500	31.14300	37.96600
H	-13.99400	32.17800	37.06600
O	-14.48300	30.88800	34.76500
H	-13.95400	30.37700	34.15200
H	-14.22000	30.57200	35.63000
O	-25.75800	20.20100	34.30500
H	-25.46300	19.29200	34.25100
H	-26.65900	20.18000	33.98300
O	-22.22900	18.27700	40.28700
H	-22.95000	18.89000	40.14800
H	-21.44000	18.81200	40.20900
O	-24.03000	17.88200	34.42800
H	-24.35800	17.00200	34.24600
H	-23.67600	17.82700	35.31600
O	-14.78200	29.51800	38.93800
H	-14.92900	28.59000	39.11900
H	-13.95600	29.54000	38.45400
O	-20.91500	18.17000	34.79600
H	-20.57600	18.69800	34.07400
H	-20.46800	18.50800	35.57200
O	-18.22100	16.55300	44.24600
H	-18.56100	15.75400	44.64800
H	-18.92700	16.85400	43.67400
O	-17.56900	13.63800	42.69900
H	-16.74700	13.16400	42.57700
H	-17.32600	14.41700	43.19900
O	-18.92900	16.66700	32.99500
H	-18.20800	17.04500	32.49200
H	-19.55200	17.38600	33.09900

O	-14.78500	17.37200	33.93100
H	-14.56900	18.25400	33.63000
H	-15.26600	16.98000	33.20200
O	-20.04500	20.33000	40.50800
H	-20.19100	20.64400	41.40100
H	-20.52300	20.94900	39.95500
O	-18.18600	17.32000	40.55600
H	-18.20000	16.42500	40.22000
H	-17.46900	17.32800	41.19000
O	-16.33300	17.75400	42.60200
H	-16.59100	18.67600	42.60000
H	-16.86500	17.35700	43.29200
O	-11.38900	30.85900	35.38100
H	-11.21500	30.29400	34.62800
H	-10.89600	31.65900	35.19900
O	-18.78200	16.20200	35.82500
H	-18.66100	15.27400	36.02400
H	-19.07200	16.21600	34.91300
O	-15.91300	13.88700	38.96200
H	-15.89100	13.95100	38.00700
H	-15.49600	13.04800	39.15600
O	-5.53500	23.62300	37.00000
H	-5.09200	22.87400	36.60200
H	-6.41200	23.61400	36.61700
O	-10.40100	30.17600	32.48000
H	-9.70600	30.79700	32.69900
H	-10.05900	29.32600	32.75600
O	-8.50200	27.62600	34.00900
H	-7.97700	27.96500	33.28400
H	-8.59000	26.69100	33.82500
O	-19.80900	27.78700	33.91700
H	-19.66000	28.47100	33.26300
H	-19.00200	27.76800	34.43200
O	-5.99100	25.09300	39.39200
H	-5.15700	25.53200	39.55600
H	-5.85500	24.61700	38.57300
O	-22.05400	23.81400	34.92300
H	-22.29900	22.91000	35.12100
H	-21.88400	23.81600	33.98100
O	-14.36400	14.68600	44.30500
H	-13.84600	15.42200	43.98000
H	-14.71900	14.99300	45.14000
O	-22.78400	19.54700	32.42100
H	-23.47700	19.62100	31.76500
H	-23.02900	18.78300	32.94400
O	-14.11300	11.10600	39.82500
H	-13.83300	10.43900	40.45100
H	-13.32800	11.29900	39.31300
O	-16.31000	30.28200	31.38800
H	-16.88200	30.90100	30.93500
H	-15.73600	30.83200	31.92200
O	-10.24200	19.52800	43.57300
H	-9.29000	19.62800	43.59400
H	-10.41200	18.73600	44.08200
O	-12.22100	16.61400	43.75800
H	-12.81600	16.89400	43.06300
H	-12.50600	17.10300	44.53000
O	-13.09000	30.56600	32.51900
H	-12.15700	30.50000	32.72200
H	-13.17800	31.40100	32.06000
O	-10.46100	29.88200	29.47900
H	-10.54000	29.54800	30.37300
H	-9.64000	30.37500	29.47800
O	-7.76300	22.74900	42.68700
H	-7.79600	21.81000	42.87300
H	-7.15300	22.82700	41.95300
O	-15.92500	21.61800	28.72900
H	-14.97900	21.48900	28.79800
H	-16.30300	20.88000	29.20600
O	-7.61400	19.54000	42.73300
H	-8.13000	18.75600	42.54600
H	-6.73000	19.21200	42.89700
O	-9.95300	13.28400	38.69200
H	-10.15000	12.97500	39.57600
H	-9.34300	14.01000	38.82200
O	-17.74100	19.54300	28.66500
H	-18.64700	19.24700	28.74100
H	-17.46300	19.24600	27.79800
O	-16.12400	14.08700	34.81900
H	-16.93400	13.74200	35.19500
H	-15.43600	13.77800	35.40800
O	-14.19500	13.16500	36.44200
H	-13.64200	13.58400	37.10100
H	-13.95800	12.23800	36.48400
O	-18.97100	16.40200	28.80600
H	-18.10900	16.72700	29.06600
H	-18.78600	15.61500	28.29300
O	-10.81700	15.27500	35.95000
H	-11.39800	15.93300	35.56700
H	-11.08400	14.45300	35.53800
O	-12.36700	14.67500	38.15200
H	-11.86700	14.78700	37.34300
H	-11.72400	14.37100	38.79200
O	-12.03500	17.43500	35.00500
H	-12.92200	17.72000	34.78700
H	-11.62000	17.27400	34.15800
O	-8.68400	17.45900	38.72100
H	-7.90800	17.94900	38.99300
H	-9.40900	17.88500	39.17800
O	-6.24600	18.17200	39.77800
H	-5.65200	17.93200	39.06700
H	-6.17400	17.45300	40.40500
O	-12.89100	27.19900	29.10000
H	-12.18700	27.40400	29.71600
H	-13.61400	27.76700	29.36500
O	-12.06100	22.95000	35.22000
H	-12.11700	23.40700	34.38100
H	-11.50800	22.18900	35.04300
O	-8.25000	18.73900	36.04500
H	-8.50700	18.33900	36.87600
H	-8.87500	19.45400	35.92000
O	-3.93900	21.47900	38.55200
H	-4.49900	21.54800	39.32600
H	-4.54500	21.30300	37.83200
O	-10.98200	27.63700	31.15000
H	-10.14700	27.20600	30.96800
H	-11.14400	27.46600	32.07800
O	-11.32700	24.14100	32.51600

H	-11.38300	23.97500	31.57400
H	-11.95300	24.84800	32.67200
O	-18.01800	13.64200	32.09400
H	-18.03000	14.58600	32.25100
H	-17.12500	13.45900	31.80000
O	-14.20300	10.23600	36.34900
H	-14.96900	9.68200	36.49400
H	-13.79200	10.30800	37.21000
O	-7.84400	36.87600	30.51300
H	-7.59100	36.35400	29.75200
H	-8.78100	36.71300	30.61600
O	-8.12400	38.77900	32.71600
H	-8.24100	38.10300	33.38400
H	-8.01400	38.29200	31.89900
O	-6.14000	33.26900	31.41700
H	-5.97300	34.13700	31.78400
H	-6.33100	33.43200	30.49300
O	-6.16200	35.72500	32.40100
H	-6.28200	36.17000	33.24000
H	-6.85600	36.07300	31.84300
O	-9.52100	34.09100	31.66300
H	-10.35700	34.42900	31.98400
H	-9.66000	33.95600	30.72600
O	-7.54500	28.93500	36.37100
H	-8.19900	28.62000	35.74700
H	-7.58700	28.31300	37.09700
O	-6.43400	39.21700	28.28700
H	-7.34100	38.98600	28.48600
H	-5.93700	38.42100	28.47400
O	-5.55800	40.85900	30.14800
H	-5.99600	40.14300	29.68800
H	-4.81700	41.08700	29.58600
O	-12.19200	36.56500	34.47500
H	-13.12200	36.50400	34.69200
H	-12.09600	36.04300	33.67800
O	-12.27300	33.09100	37.06300
H	-12.01300	34.00500	36.95300
H	-11.45200	32.62300	37.21200
O	-1.43700	39.71700	29.74100
H	-2.05400	38.99200	29.64400
H	-1.90500	40.47800	29.39800
O	-9.64700	33.93600	28.86300
H	-10.52400	33.81500	28.49900
H	-9.17100	33.14500	28.61100
O	-14.91900	35.53000	34.30500
H	-14.62400	34.62100	34.25100
H	-15.82000	35.50900	33.98300
O	-13.19000	33.21200	34.42800
H	-13.51900	32.33100	34.24600
H	-12.83600	33.15600	35.31600
O	-10.07600	33.49900	34.79600
H	-9.73600	34.02700	34.07400
H	-9.62900	33.83700	35.57200
O	-8.08900	31.99600	32.99500
H	-7.36800	32.37400	32.49200
H	-8.71300	32.71500	33.09900
O	-3.94600	32.70100	33.93100
H	-3.73000	33.58300	33.63000
H	-4.42700	32.30900	33.20200
O	-7.94300	31.53100	35.82500
H	-7.82100	30.60300	36.02400
H	-8.23300	31.54500	34.91300
O	-11.94500	34.87600	32.42100
H	-12.63800	34.95000	31.76500
H	-12.19000	34.11200	32.94400
O	-5.08600	36.94700	28.72900
H	-4.14000	36.81800	28.79800
H	-5.46400	36.20900	29.20600
O	-6.90100	34.87200	28.66500
H	-7.80800	34.57600	28.74100
H	-6.62400	34.57500	27.79800
O	-5.28500	29.41600	34.81900
H	-6.09500	29.07100	35.19500
H	-4.59700	29.10700	35.40800
O	-3.35600	28.49400	36.44200
H	-2.80300	28.91300	37.10100
H	-3.11900	27.56700	36.48400
O	-8.13100	31.73100	28.80600
H	-7.26900	32.05700	29.06600
H	-7.94600	30.94400	28.29300
O	-7.17800	28.97100	32.09400
H	-7.19100	29.91500	32.25100
H	-6.28600	28.78800	31.80000
O	-3.36300	25.56500	36.34900
H	-4.13000	25.01100	36.49400
H	-2.95200	25.63700	37.21000
O	-36.62400	-25.35400	19.20700
H	-37.27500	-24.76800	18.82100
H	-35.83000	-24.82300	19.26700
O	-35.14700	-27.66400	19.74400
H	-35.78200	-27.59100	20.45600
H	-35.47700	-27.07500	19.06600
O	-40.91900	-20.48600	22.77500
H	-40.99700	-19.53600	22.85400
H	-41.52300	-20.83200	23.43200
O	-32.98100	-32.78500	19.95400
H	-32.06900	-32.55200	19.77900
H	-33.49000	-32.20100	19.39100
O	-38.64500	-25.12800	21.49500
H	-38.78100	-24.28300	21.92400
H	-38.10400	-24.92600	20.73200
O	-35.33100	-31.61400	18.53200
H	-35.84600	-30.84400	18.77100
H	-35.60300	-32.28800	19.15600
O	-30.35700	-28.86100	20.46800
H	-31.13400	-29.20000	20.02300
H	-29.67800	-28.85600	19.79300
O	-38.95200	-29.76500	15.01100
H	-39.86000	-29.99600	15.21000
H	-38.45500	-30.56100	15.19900
O	-36.04600	-21.05500	17.93100
H	-36.71900	-20.75200	18.53900
H	-35.65900	-20.25200	17.58100
O	-32.54200	-26.05300	23.30300
H	-32.99500	-26.89300	23.35900
H	-33.17200	-25.41600	23.64000
O	-34.09800	-24.21500	24.23800
H	-33.80600	-23.65400	23.51900

H	-33.58800	-23.92200	24.99300
O	-38.07600	-28.12200	16.87200
H	-38.51400	-28.83900	16.41300
H	-37.33500	-27.89500	16.31100
O	-29.95900	-29.84700	22.93000
H	-30.09700	-29.65600	22.00200
H	-30.31500	-29.08500	23.38700
O	-37.84600	-20.88700	20.14600
H	-37.67100	-21.35800	20.96000
H	-38.53300	-20.26000	20.37400
O	-33.95500	-29.26400	16.46600
H	-34.57200	-29.98900	16.36900
H	-34.42300	-28.50300	16.12200
O	-38.97900	-22.69300	22.83400
H	-39.76200	-22.15100	22.92800
H	-38.27900	-22.17500	23.23300
O	-33.13100	-25.94000	20.58500
H	-32.85500	-26.05400	21.49400
H	-34.00100	-26.33800	20.54600
O	-36.49600	-21.69100	24.22800
H	-36.31300	-22.50900	24.69100
H	-35.67200	-21.47500	23.79100
O	-36.16100	-22.76500	21.49000
H	-35.63300	-23.27500	20.87700
H	-35.89900	-23.08000	22.35400
O	-33.06700	-22.79300	22.10500
H	-32.89400	-23.35800	21.35300
H	-32.57400	-21.99300	21.92300
O	-27.21300	-30.02900	23.72400
H	-26.77000	-30.77900	23.32600
H	-28.09100	-30.03800	23.34200
O	-32.08000	-23.47600	19.20500
H	-31.38500	-22.85600	19.42400
H	-31.73800	-24.32700	19.48000
O	-30.18100	-26.02600	20.73400
H	-29.65600	-25.68800	20.00900
H	-30.26800	-26.96200	20.55000
O	-37.98800	-23.37000	18.11200
H	-38.56100	-22.75100	17.65900
H	-37.41500	-22.82000	18.64600
O	-34.76900	-23.08600	19.24300
H	-33.83600	-23.15200	19.44700
H	-34.85600	-22.25100	18.78500
O	-32.14000	-23.77000	16.20400
H	-32.21800	-24.10400	17.09700
H	-31.31900	-23.27800	16.20300
O	-37.60400	-32.03400	15.45400
H	-36.65800	-32.16300	15.52300
H	-37.98200	-32.77200	15.93100
O	-34.56900	-26.45300	15.82500
H	-33.86600	-26.24800	16.44000
H	-35.29300	-25.88500	16.09000
O	-32.66100	-26.01500	17.87400
H	-31.82600	-26.44700	17.69200
H	-32.82200	-26.18600	18.80200
O	-33.00600	-29.51100	19.24000
H	-33.06100	-29.67700	18.29900
H	-33.63200	-28.80400	19.39600
O	-24.03200	-15.49800	27.97000
H	-23.39400	-15.61700	27.26600
H	-24.15000	-14.55000	28.02900
O	-27.22300	-15.54500	27.90600
H	-27.56200	-15.66100	27.01800
H	-26.35400	-15.94600	27.88600
O	-31.36500	-13.69100	23.89600
H	-31.92800	-13.78200	23.12700
H	-31.47900	-14.51100	24.37600
O	-33.81800	-12.19600	26.73400
H	-33.02600	-12.53700	26.31900
H	-33.57900	-12.08700	27.65400
O	-25.78500	-10.02400	19.20700
H	-26.43600	-9.43900	18.82100
H	-24.99000	-9.49400	19.26700
O	-31.85900	-12.93500	19.60600
H	-31.08600	-13.48300	19.46700
H	-31.50700	-12.07100	19.81700
O	-30.02100	-11.60800	32.81300
H	-30.31200	-10.70300	32.69700
H	-29.38500	-11.56600	33.52700
O	-29.09500	-14.23000	29.40300
H	-28.49900	-14.45400	28.68900
H	-29.03800	-14.97200	30.00500
O	-29.52300	-16.77600	17.23800
H	-29.27000	-17.29800	16.47600
H	-30.46000	-16.94000	17.34100
O	-24.47800	-17.77300	25.04500
H	-23.69200	-18.19100	24.69400
H	-24.21400	-16.86800	25.21300
O	-24.30700	-12.33500	19.74400
H	-24.94300	-12.26200	20.45600
H	-24.63700	-11.74600	19.06600
O	-19.07700	-17.83500	25.94900
H	-18.34500	-18.40500	26.18200
H	-19.83300	-18.22300	26.38900
O	-29.80300	-14.87300	19.44000
H	-29.91900	-15.54900	20.10800
H	-29.69300	-15.36000	18.62300
O	-27.46000	-15.41200	23.14500
H	-27.82400	-15.93900	23.85600
H	-26.60300	-15.80200	22.97200
O	-27.81900	-20.38300	18.14100
H	-27.65200	-19.51500	18.50800
H	-28.00900	-20.22100	17.21700
O	-25.06200	-11.92200	29.80800
H	-25.36200	-11.01900	29.69900
H	-25.40700	-12.18800	30.66000
O	-31.61000	-15.47000	25.84100
H	-32.53500	-15.60300	26.04900
H	-31.36200	-14.69100	26.33800
O	-21.66200	-19.40300	27.48700
H	-22.57000	-19.47200	27.19300
H	-21.71300	-18.92600	28.31600
O	-27.84000	-17.92800	19.12600
H	-27.96000	-17.48200	19.96400
H	-28.53500	-17.57900	18.56700
O	-34.25000	-15.82700	26.67600
H	-34.12000	-15.57300	27.59000
H	-34.93000	-15.23400	26.35600

O	-30.08000	-5.15700	22.77500
H	-30.15700	-4.20600	22.85400
H	-30.68300	-5.50300	23.43200
O	-19.79600	-21.20200	29.26500
H	-20.43800	-20.90800	28.61900
H	-20.30000	-21.31600	30.07100
O	-22.14200	-17.45500	19.95400
H	-21.23000	-17.22300	19.77900
H	-22.65100	-16.87200	19.39100
O	-23.85800	-13.03300	33.59000
H	-23.44900	-13.47500	32.84500
H	-24.58000	-13.60800	33.84300
O	-23.53500	-12.47300	27.34500
H	-24.16400	-12.30800	26.64300
H	-23.92200	-12.05900	28.11600
O	-19.65600	-11.53700	24.70000
H	-20.34100	-11.15700	24.14900
H	-19.57200	-10.92800	25.43300
O	-22.40100	-13.89100	31.29900
H	-22.18900	-13.24800	30.62300
H	-22.44800	-14.72500	30.83100
O	-24.79800	-21.28000	28.20300
H	-25.66000	-20.98100	28.49400
H	-24.77200	-21.06300	27.27100
O	-27.98200	-12.73500	24.61000
H	-27.51800	-12.45700	23.82100
H	-28.24600	-13.63700	24.42700
O	-31.09000	-13.18800	27.54800
H	-30.51900	-12.76000	26.91000
H	-30.49900	-13.47000	28.24700
O	-29.04500	-16.61300	21.41900
H	-28.34300	-16.16100	21.88600
H	-29.37700	-17.25500	22.04700
O	-35.30300	-18.35500	26.72100
H	-36.09300	-18.09900	26.24500
H	-34.81100	-17.54000	26.82600
O	-21.28600	-11.01400	29.47100
H	-20.46400	-11.41600	29.19000
H	-21.29000	-11.11700	30.42300
O	-33.35100	-17.99000	23.67900
H	-34.14800	-17.87800	24.19800
H	-33.57900	-17.65200	22.81300
O	-27.52100	-11.46600	31.62700
H	-27.45600	-11.00400	32.46300
H	-28.22900	-12.09600	31.75600
O	-13.19600	-14.67000	25.82000
H	-13.94900	-15.24000	25.66400
H	-13.06100	-14.70400	26.76700
O	-27.80600	-9.79900	21.49500
H	-27.94100	-8.95400	21.92400
H	-27.26500	-9.59700	20.73200
O	-34.35300	-12.28100	22.06800
H	-33.84000	-11.48700	22.21900
H	-33.70100	-12.97900	22.00100
O	-32.54300	-9.16500	24.15000
H	-32.36700	-8.24300	23.96200
H	-31.74800	-9.47900	24.58000
O	-26.17900	-9.17700	35.62500
H	-26.62300	-9.96300	35.30700
H	-25.51700	-8.98900	34.96000
O	-31.42000	-7.64700	30.15100
H	-30.86900	-7.77500	29.37900
H	-32.25900	-7.34800	29.80100
O	-24.08100	-8.89600	33.65300
H	-24.09900	-8.07500	33.16200
H	-23.15900	-9.02100	33.87900
O	-36.38800	-13.95300	22.84400
H	-35.55000	-13.54300	22.62700
H	-36.21000	-14.89300	22.81400
O	-24.49100	-16.28500	18.53200
H	-25.00700	-15.51500	18.77100
H	-24.76400	-16.95900	19.15600
O	-26.90700	-21.44000	23.59100
H	-27.76300	-21.82900	23.41200
H	-26.49400	-21.36100	22.73100
O	-25.37700	-23.90500	27.99000
H	-25.88000	-23.94200	27.17700
H	-25.08400	-22.99600	28.05000
O	-19.51800	-13.53200	20.46800
H	-20.29500	-13.87100	20.02300
H	-18.83900	-13.52700	19.79300
O	-24.70300	-18.41200	20.35800
H	-25.00800	-18.09800	21.20900
H	-23.75000	-18.33400	20.40100
O	-26.25800	-12.25300	22.00800
H	-26.78600	-12.98800	21.69500
H	-26.75900	-11.47600	21.76200
O	-21.27900	-14.05800	27.12400
H	-21.93700	-13.36900	27.21200
H	-20.47500	-13.66900	27.46800
O	-31.52600	-21.59100	24.36800
H	-30.95200	-21.81400	23.63600
H	-30.93900	-21.52200	25.12100
O	-22.46800	-15.70400	25.17500
H	-21.95500	-15.07100	25.67700
H	-21.81800	-16.30400	24.80900
O	-31.19900	-19.56100	18.38800
H	-32.03600	-19.22300	18.70900
H	-31.33900	-19.69600	17.45100
O	-29.22400	-24.71700	23.09600
H	-29.87800	-25.03200	22.47200
H	-29.26600	-25.34000	23.82200
O	-28.11300	-14.43500	15.01100
H	-29.02000	-14.66600	15.21000
H	-27.61600	-15.23200	15.19900
O	-25.20600	-5.72600	17.93100
H	-25.88000	-5.42200	18.53900
H	-24.82000	-4.92300	17.58100
O	-25.99000	-18.22900	27.42100
H	-26.90800	-18.00800	27.26300
H	-25.57400	-18.15400	26.56200
O	-25.20700	-16.83000	22.55100
H	-24.46000	-16.31800	22.24200
H	-24.93800	-17.15200	23.41100
O	-26.93400	-14.33400	19.53400
H	-27.84300	-14.61800	19.63100
H	-26.90500	-13.91400	18.67400
O	-21.70200	-10.72300	23.30300

H	-22.15600	-11.56400	23.35900
H	-22.33300	-10.08700	23.64000
O	-22.75900	-19.79400	24.53300
H	-23.49700	-20.30600	24.86400
H	-22.50600	-20.23400	23.72100
O	-23.25900	-8.88600	24.23800
H	-22.96700	-8.32500	23.51900
H	-22.74900	-8.59300	24.99300
O	-22.65800	-27.01700	25.12700
H	-22.35200	-27.87700	24.84000
H	-21.93200	-26.42500	24.92700
O	-36.36500	-16.61400	23.51500
H	-36.56300	-17.20700	22.79000
H	-36.92100	-16.91800	24.23300
O	-28.38600	-16.72100	25.63500
H	-28.33100	-17.67400	25.55900
H	-29.19700	-16.56500	26.11900
O	-27.23700	-12.79300	16.87200
H	-27.67500	-13.51000	16.41300
H	-26.49500	-12.56600	16.31100
O	-30.44600	-18.10300	23.39400
H	-31.26000	-17.80400	23.80000
H	-29.95000	-18.49800	24.11100
O	-28.29600	-8.10800	25.32700
H	-28.33800	-7.88200	24.39800
H	-27.37900	-8.34100	25.47600
O	-19.11900	-14.51700	22.93000
H	-19.25700	-14.32700	22.00200
H	-19.47600	-13.75600	23.38700
O	-19.09200	-12.45000	27.94100
H	-18.65900	-12.54200	28.78900
H	-18.37900	-12.47400	27.30300
O	-30.17900	-11.23100	25.12300
H	-29.38500	-11.72600	24.92200
H	-30.89000	-11.76000	24.76000
O	-25.35900	-8.85000	29.47400
H	-25.30600	-8.28100	30.24200
H	-26.22800	-8.67900	29.11000
O	-33.87100	-17.08700	21.19900
H	-34.80100	-17.14900	21.41600
H	-33.77500	-17.61000	20.40300
O	-33.95200	-20.56200	23.78700
H	-33.69100	-19.64700	23.67800
H	-33.13000	-21.02900	23.93700
O	-18.23100	-12.40700	30.47400
H	-18.03700	-11.50300	30.72000
H	-18.33100	-12.86700	31.30800
O	-23.16900	-12.25700	36.04900
H	-23.32100	-12.48600	35.13200
H	-23.17900	-11.30000	36.06200
O	-26.40100	-14.68400	31.03300
H	-25.47100	-14.53200	30.86000
H	-26.82200	-14.59500	30.17800
O	-27.60200	-10.65900	28.80300
H	-27.74100	-11.02700	29.67600
H	-28.39000	-10.14400	28.63100
O	-27.00700	-5.55800	20.14600
H	-26.83200	-6.02900	20.96000
H	-27.69400	-4.93100	20.37400
O	-26.05100	-11.28000	26.19200
H	-26.38600	-11.32000	27.08800
H	-26.55100	-11.94600	25.71900
O	-23.11500	-13.93500	16.46600
H	-23.73200	-14.66000	16.36900
H	-23.58400	-13.17400	16.12200
O	-21.46000	-9.22500	34.12600
H	-21.51900	-9.90900	33.45900
H	-21.05800	-8.48300	33.67400
O	-32.44100	-2.40900	26.92000
H	-32.20100	-1.77700	27.59700
H	-33.28400	-2.76000	27.20600
O	-31.32600	-19.71600	15.58800
H	-32.20300	-19.83700	15.22400
H	-30.85000	-20.50800	15.33600
O	-21.45800	-11.26100	32.35800
H	-22.34600	-11.59800	32.48000
H	-20.88800	-11.99300	32.59400
O	-39.80500	-14.92800	25.68200
H	-39.11800	-15.24200	25.09400
H	-39.96100	-14.02400	25.40900
O	-27.92800	-6.60100	29.32400
H	-28.00100	-5.79500	29.83600
H	-28.37000	-6.40200	28.49900
O	-28.13900	-7.36400	22.83400
H	-28.92200	-6.82100	22.92800
H	-27.44000	-6.84600	23.23300
O	-24.82900	-21.55400	25.47700
H	-24.33300	-22.30700	25.15800
H	-25.60100	-21.51800	24.91200
O	-16.97700	-15.85000	27.11900
H	-16.98800	-14.96800	26.74900
H	-17.84500	-16.20300	26.92500
O	-25.46800	-6.70200	31.48000
H	-24.75500	-6.06700	31.40700
H	-25.95900	-6.60600	30.66400
O	-23.74700	-12.79700	23.05100
H	-24.68100	-12.67700	22.88200
H	-23.56900	-13.70000	22.78700
O	-28.24600	-19.41200	25.20500
H	-28.61600	-19.96500	25.89300
H	-27.75000	-20.01700	24.65200
O	-20.35900	-16.95500	23.58600
H	-19.89300	-16.12200	23.51200
H	-19.90800	-17.42100	24.29100
O	-30.41700	-8.95300	32.30000
H	-29.71600	-8.31900	32.45300
H	-30.88800	-8.61100	31.54000
O	-22.29200	-10.61100	20.58500
H	-22.01600	-10.72500	21.49400
H	-23.16100	-11.00900	20.54600
O	-22.38800	-24.58700	27.62500
H	-23.30100	-24.35700	27.79400
H	-21.95800	-24.50300	28.47700
O	-22.61700	-16.39800	30.12500
H	-23.27000	-16.15100	29.47100
H	-22.23900	-17.21200	29.79200
O	-25.65600	-6.36200	24.22800
H	-25.47400	-7.18000	24.69100

H	-24.83300	-6.14500	23.79100
O	-25.32200	-7.43500	21.49000
H	-24.79300	-7.94600	20.87700
H	-25.05900	-7.75100	22.35400
O	-34.82000	-9.41900	29.23300
H	-34.23500	-10.16500	29.36600
H	-35.01800	-9.10900	30.11700
O	-31.96600	-6.38400	24.24900
H	-32.63500	-5.89900	23.76600
H	-32.10500	-6.13700	25.16400
O	-27.82600	-4.34900	30.84800
H	-27.27400	-4.18600	31.61300
H	-27.48500	-3.75900	30.17600
O	-22.97500	-9.52300	36.70300
H	-22.43100	-8.91000	36.20800
H	-23.55600	-8.96500	37.21900
O	-36.59800	-18.12200	21.02900
H	-36.30200	-19.03100	20.97600
H	-37.49900	-18.14300	20.70700
O	-24.68900	-6.40100	27.83400
H	-24.81400	-7.20200	27.32500
H	-24.03100	-5.90700	27.34500
O	-33.06800	-20.04600	27.01200
H	-33.79000	-19.43300	26.87300
H	-32.27900	-19.51100	26.93300
O	-22.62600	-8.60000	29.46200
H	-23.57300	-8.72000	29.39000
H	-22.27700	-9.48800	29.54500
O	-34.86900	-20.44100	21.15300
H	-35.19800	-21.32100	20.97000
H	-34.51500	-20.49600	22.04100
O	-25.62100	-8.80500	25.66200
H	-25.76800	-9.73300	25.84300
H	-24.79600	-8.78300	25.17800
O	-31.75500	-20.15300	21.52100
H	-31.41500	-19.62500	20.79800
H	-31.30700	-19.81500	22.29600
O	-22.01800	-7.98200	26.74900
H	-21.09300	-8.18800	26.61100
H	-22.18600	-8.23100	27.65800
O	-29.76800	-21.65600	19.72000
H	-29.04700	-21.27800	19.21600
H	-30.39100	-20.93700	19.82300
O	-25.62500	-20.95100	20.65600
H	-25.40800	-20.06900	20.35500
H	-26.10500	-21.34300	19.92600
O	-30.88500	-17.99300	27.23300
H	-31.03000	-17.67900	28.12500
H	-31.36200	-17.37400	26.68000
O	-29.02500	-21.00300	27.28100
H	-29.03900	-21.89800	26.94400
H	-28.30800	-20.99500	27.91500
O	-22.22800	-7.46400	22.10500
H	-22.05400	-8.02900	21.35300
H	-21.73500	-6.66400	21.92300
O	-29.62200	-22.12100	22.55000
H	-29.50000	-23.04900	22.74900
H	-29.91100	-22.10700	21.63700
O	-26.75200	-24.43600	25.68600
H	-26.73000	-24.37200	24.73100
H	-26.33600	-25.27500	25.88000
O	-16.37400	-14.70000	23.72400
H	-15.93100	-15.44900	23.32600
H	-17.25100	-14.70900	23.34200
O	-21.24000	-8.14700	19.20500
H	-20.54500	-7.52600	19.42400
H	-20.89800	-8.99700	19.48000
O	-19.34100	-10.69700	20.73400
H	-18.81600	-10.35800	20.00900
H	-19.42900	-11.63200	20.55000
O	-27.67900	-6.78400	32.86300
H	-27.37100	-7.02900	33.73500
H	-26.88200	-6.57900	32.37400
O	-27.65700	-4.63000	25.09300
H	-28.09700	-5.12200	25.78600
H	-26.90600	-5.17300	24.85200
O	-35.17800	-8.43900	25.09600
H	-34.35200	-8.73800	24.71500
H	-35.52900	-9.20800	25.54500
O	-30.21000	-9.08600	27.16500
H	-30.83200	-9.74900	26.86600
H	-29.53900	-9.06000	26.48300
O	-36.97700	-10.52000	26.05400
H	-37.81400	-10.94800	25.87300
H	-37.00500	-10.32700	26.99200
O	-19.65200	-10.06400	36.03900
H	-20.44200	-10.08100	35.49900
H	-19.88900	-9.52500	36.79400
O	-19.53200	-13.21500	33.35200
H	-19.48200	-13.38000	34.29400
H	-19.64100	-14.08200	32.96200
O	-30.64900	-10.53600	20.64100
H	-30.50000	-9.85200	19.98800
H	-29.84200	-10.55500	21.15600
O	-18.03000	-10.79500	33.59600
H	-18.32200	-10.43200	34.43300
H	-18.64300	-11.50800	33.41800
O	-16.83100	-13.23000	26.11600
H	-15.99600	-12.79100	26.28000
H	-16.69500	-13.70600	25.29700
O	-19.78200	-15.67200	32.06400
H	-19.30600	-15.47900	31.25600
H	-20.70500	-15.61600	31.81900
O	-32.89300	-14.50900	21.64700
H	-33.13900	-15.41300	21.84500
H	-32.72400	-14.50700	20.70500
O	-33.62400	-18.77600	19.14600
H	-34.31700	-18.70200	18.49000
H	-33.86900	-19.54000	19.66800
O	-27.14900	-8.04100	18.11200
H	-27.72200	-7.42200	17.65900
H	-26.57600	-7.49100	18.64600
O	-21.08100	-18.79500	30.29700
H	-20.13000	-18.69500	30.31800
H	-21.25100	-19.58700	30.80700
O	-23.92900	-7.75700	19.24300
H	-22.99600	-7.82300	19.44700
H	-24.01700	-6.92100	18.78500

O	-21.30000	-8.44100	16.20400
H	-21.37900	-8.77500	17.09700
H	-20.47900	-7.94800	16.20300
O	-18.60300	-15.57400	29.41100
H	-18.63500	-16.51300	29.59700
H	-17.99300	-15.49600	28.67800
O	-26.76400	-16.70500	15.45400
H	-25.81800	-16.83400	15.52300
H	-27.14300	-17.44300	15.93100
O	-18.45300	-18.78300	29.45700
H	-18.96900	-19.56700	29.27000
H	-17.56900	-19.11100	29.62200
O	-20.79200	-25.03900	25.41700
H	-20.99000	-25.34800	26.30100
H	-20.18200	-24.31300	25.54600
O	-12.78500	-14.51300	28.55200
H	-12.05500	-14.77400	29.11300
H	-13.39400	-15.25000	28.59200
O	-14.70300	-16.37200	29.14900
H	-15.43000	-16.50600	28.54100
H	-15.11900	-16.06400	29.95500
O	-28.58000	-18.78000	15.38900
H	-29.48700	-19.07600	15.46600
H	-28.30200	-19.07700	14.52300
O	-37.66200	-17.45900	25.83900
H	-37.98200	-18.31300	25.54700
H	-38.20900	-17.24400	26.59500
O	-26.96400	-24.23600	21.54400
H	-27.77400	-24.58100	21.91900
H	-26.27500	-24.54500	22.13300
O	-25.03500	-25.15800	23.16600
H	-24.48200	-24.73900	23.82500
H	-24.79800	-26.08500	23.20900
O	-29.81000	-21.92100	15.53100
H	-28.94800	-21.59600	15.79000
H	-29.62500	-22.70800	15.01800
O	-37.79300	-13.83900	27.80900
H	-38.48700	-14.25300	27.29600
H	-37.04600	-13.79700	27.21200
O	-21.65700	-23.04800	22.67400
H	-22.23700	-22.39000	22.29200
H	-21.92300	-23.87000	22.26300
O	-23.20700	-23.64800	24.87600
H	-22.70700	-23.53600	24.06800
H	-22.56300	-23.95200	25.51600
O	-35.88000	-13.90700	25.68500
H	-35.90300	-13.80800	24.73300
H	-35.57000	-13.06000	26.00700
O	-33.22100	-11.84900	29.80000
H	-32.26700	-11.88100	29.73300
H	-33.42000	-12.31000	30.61500
O	-22.87400	-20.88800	21.73000
H	-23.76200	-20.60300	21.51200
H	-22.45900	-21.04900	20.88300
O	-19.52300	-20.86400	25.44500
H	-18.74700	-20.37400	25.71800
H	-20.24800	-20.43800	25.90200
O	-17.08600	-20.15100	26.50300
H	-16.49100	-20.39100	25.79200
H	-17.01300	-20.87000	27.13000
O	-34.86600	-6.30700	26.81700
H	-35.00100	-7.06700	26.25100
H	-35.28300	-5.58300	26.35000
O	-23.73000	-11.12400	15.82500
H	-23.02600	-10.91900	16.44000
H	-24.45400	-10.55600	16.09000
O	-22.90000	-15.37300	21.94500
H	-22.95700	-14.91600	21.10600
H	-22.34700	-16.13400	21.76700
O	-19.09000	-19.58400	22.77000
H	-19.34600	-19.98400	23.60100
H	-19.71500	-18.86900	22.64500
O	-14.77900	-16.84400	25.27700
H	-15.33900	-16.77500	26.05000
H	-15.38400	-17.02000	24.55700
O	-31.70900	-5.41000	27.02900
H	-31.82600	-4.46600	26.92700
H	-32.53000	-5.71300	27.41700
O	-21.82100	-10.68500	17.87400
H	-20.98700	-11.11700	17.69200
H	-21.98300	-10.85700	18.80200
O	-22.16700	-14.18200	19.24000
H	-22.22200	-14.34800	18.29900
H	-22.79200	-13.47500	19.39600
O	-28.85700	-24.68100	18.81900
H	-28.86900	-23.73700	18.97500
H	-27.96500	-24.86400	18.52500
O	-30.68400	-4.75800	30.24400
H	-29.78100	-4.72500	30.56100
H	-30.91000	-5.68800	30.25900
O	-28.92800	-6.00500	26.90500
H	-29.87900	-6.00400	26.79200
H	-28.62500	-6.75100	26.38900
O	-25.04200	-28.08700	23.07300
H	-25.80900	-28.64100	23.21900
H	-24.63100	-28.01500	23.93500
O	-13.19300	-0.16900	27.97000
H	-12.55500	-0.28800	27.26600
H	-13.31100	0.78000	28.02900
O	-16.38400	-0.21600	27.90600
H	-16.72200	-0.33200	27.01800
H	-15.51500	-0.61700	27.88600
O	-20.52500	1.63800	23.89600
H	-21.08900	1.54700	23.12700
H	-20.64000	0.81800	24.37600
O	-15.90200	0.93800	33.86500
H	-15.91300	0.54300	32.99300
H	-15.74000	0.20400	34.45800
O	-22.97900	3.13300	26.73400
H	-22.18700	2.79200	26.31900
H	-22.73900	3.24200	27.65400
O	-14.94500	5.30500	19.20700
H	-15.59700	5.89000	18.82100
H	-14.15100	5.83500	19.26700
O	-21.02000	2.39400	19.60600
H	-20.24700	1.84700	19.46700
H	-20.66700	3.25800	19.81700
O	-19.18100	3.72100	32.81300

H	-19.47200	4.62600	32.69700
H	-18.54600	3.76300	33.52700
O	-18.25600	1.09900	29.40300
H	-17.65900	0.87500	28.68900
H	-18.19800	0.35800	30.00500
O	-18.68300	-1.44700	17.23800
H	-18.43100	-1.96900	16.47600
H	-19.62100	-1.61000	17.34100
O	-13.63900	-2.44300	25.04500
H	-12.85300	-2.86100	24.69400
H	-13.37500	-1.53900	25.21300
O	-13.46800	2.99400	19.74400
H	-14.10300	3.06700	20.45600
H	-13.79800	3.58300	19.06600
O	-8.23800	-2.50600	25.94900
H	-7.50500	-3.07600	26.18200
H	-8.99400	-2.89400	26.38900
O	-18.96400	0.45600	19.44000
H	-19.08000	-0.22000	20.10800
H	-18.85300	-0.03100	18.62300
O	-16.62000	-0.08300	23.14500
H	-16.98500	-0.61000	23.85600
H	-15.76400	-0.47300	22.97200
O	-10.71500	-8.79400	30.40800
H	-11.04700	-7.92400	30.18400
H	-11.31100	-9.11000	31.08700
O	-16.98000	-5.05400	18.14100
H	-16.81200	-4.18600	18.50800
H	-17.17000	-4.89100	17.21700
O	-14.22200	3.40800	29.80800
H	-14.52200	4.31000	29.69900
H	-14.56700	3.14100	30.66000
O	-20.77100	-0.14100	25.84100
H	-21.69600	-0.27300	26.04900
H	-20.52300	0.63800	26.33800
O	-10.82300	-4.07400	27.48700
H	-11.73100	-4.14300	27.19300
H	-10.87400	-3.59700	28.31600
O	-17.00100	-2.59800	19.12600
H	-17.12100	-2.15300	19.96400
H	-17.69600	-2.25000	18.56700
O	-23.41000	-0.49800	26.67600
H	-23.28100	-0.24400	27.59000
H	-24.09000	0.09500	26.35600
O	-19.24000	10.17200	22.77500
H	-19.31800	11.12300	22.85400
H	-19.84400	9.82700	23.43200
O	-16.74800	-2.65500	35.43100
H	-17.52500	-2.09600	35.42100
H	-16.10900	-2.17000	35.95200
O	-8.95600	-5.87300	29.26500
H	-9.59900	-5.57900	28.61900
H	-9.46100	-5.98700	30.07100
O	-18.49400	0.15300	34.99300
H	-17.75200	0.69200	34.71600
H	-19.20000	0.78000	35.15000
O	-13.01800	2.29600	33.59000
H	-12.61000	1.85400	32.84500
H	-13.74100	1.72200	33.84300
O	-16.66500	-2.76600	30.96300
H	-15.81900	-2.34300	31.10700
H	-16.72500	-3.42300	31.65700
O	-12.69500	2.85600	27.34500
H	-13.32500	3.02100	26.64300
H	-13.08300	3.27100	28.11600
O	-8.81700	3.79200	24.70000
H	-9.50100	4.17200	24.14900
H	-8.73200	4.40100	25.43300
O	-13.51500	-5.10600	32.62100
H	-13.75900	-4.35200	32.08500
H	-13.23900	-4.72800	33.45600
O	-11.56100	1.43800	31.29900
H	-11.35000	2.08100	30.62300
H	-11.60800	0.60400	30.83100
O	-13.95900	-5.95000	28.20300
H	-14.82000	-5.65200	28.49400
H	-13.93300	-5.73300	27.27100
O	-17.14300	2.59400	24.61000
H	-16.67900	2.87300	23.82100
H	-17.40700	1.69200	24.42700
O	-20.25000	2.14100	27.54800
H	-19.68000	2.57000	26.91000
H	-19.65900	1.85900	28.24700
O	-18.20600	-1.28400	21.41900
H	-17.50300	-0.83200	21.88600
H	-18.53800	-1.92600	22.04700
O	-19.75800	-2.69300	35.79400
H	-20.38800	-2.01100	36.02800
H	-19.86400	-2.80400	34.85000
O	-24.46300	-3.02600	26.72100
H	-25.25300	-2.77000	26.24500
H	-23.97200	-2.21100	26.82600
O	-10.44700	4.31500	29.47100
H	-9.62500	3.91300	29.19000
H	-10.45100	4.21200	30.42300
O	-22.51200	-2.66100	23.67900
H	-23.30900	-2.54900	24.19800
H	-22.73900	-2.32200	22.81300
O	-16.68100	3.86300	31.62700
H	-16.61700	4.32500	32.46300
H	-17.39000	3.23300	31.75600
O	-26.17900	-2.77200	29.15200
H	-25.92900	-1.88800	29.41800
H	-25.64300	-2.95200	28.38000
O	-2.35600	0.65900	25.82000
H	-3.11000	0.09000	25.66400
H	-2.22100	0.62500	26.76700
O	-16.96600	5.53000	21.49500
H	-17.10200	6.37500	21.92400
H	-16.42500	5.73200	20.73200
O	-20.48000	-8.39100	38.15700
H	-20.87300	-8.94400	38.83200
H	-19.66300	-8.08000	38.54700
O	-23.51300	3.04800	22.06800
H	-23.00100	3.84200	22.21900
H	-22.86100	2.35000	22.00100
O	-21.70400	6.16400	24.15000
H	-21.52700	7.08600	23.96200

H	-20.90800	5.85000	24.58000
O	-17.56300	-6.97500	35.74900
H	-16.82200	-6.37400	35.66900
H	-17.33100	-7.71700	35.19100
O	-22.41900	-1.78900	34.02700
H	-22.53300	-2.69100	34.32700
H	-23.27400	-1.54300	33.67500
O	-15.34000	6.15300	35.62500
H	-15.78300	5.36600	35.30700
H	-14.67800	6.34000	34.96000
O	-20.58100	7.68200	30.15100
H	-20.03000	7.55400	29.37900
H	-21.42000	7.98200	29.80100
O	-13.24100	6.43300	33.65300
H	-13.26000	7.25400	33.16200
H	-12.31900	6.30800	33.87900
O	-16.78400	3.94700	34.49600
H	-16.25100	3.18600	34.26600
H	-17.31300	3.65500	35.23800
O	-13.61700	-10.94700	30.02500
H	-13.96100	-10.05700	29.94900
H	-13.24900	-11.13700	29.16200
O	-25.54900	1.37700	22.84400
H	-24.71100	1.78600	22.62700
H	-25.37100	0.43700	22.81400
O	-13.65200	-0.95600	18.53200
H	-14.16700	-0.18500	18.77100
H	-13.92400	-1.62900	19.15600
O	-16.06700	-6.11100	23.59100
H	-16.92300	-6.50000	23.41200
H	-15.65500	-6.03200	22.73100
O	-14.53700	-8.57600	27.99000
H	-15.04100	-8.61300	27.17700
H	-14.24400	-7.66700	28.05000
O	-18.29300	-8.49200	26.99700
H	-17.42800	-8.68800	26.63700
H	-18.22500	-8.72100	27.92400
O	-8.67900	1.79700	20.46800
H	-9.45600	1.45800	20.02300
H	-8.00000	1.80300	19.79300
O	-15.41900	3.07600	22.00800
H	-15.94700	2.34100	21.69500
H	-15.92000	3.85300	21.76200
O	-10.44000	1.27100	27.12400
H	-11.09700	1.96100	27.21200
H	-9.63600	1.66000	27.46800
O	-20.68700	-6.26200	24.36800
H	-20.11300	-6.48500	23.63600
H	-20.10000	-6.19300	25.12100
O	-11.62900	-0.37500	25.17500
H	-11.11600	0.25800	25.67700
H	-10.97900	-0.97500	24.80900
O	-20.36000	-4.23200	18.38800
H	-21.19600	-3.89400	18.70900
H	-20.50000	-4.36700	17.45100
O	-18.38500	-9.38800	23.09600
H	-19.03800	-9.70300	22.47200
H	-18.42700	-10.01000	23.82200
O	-17.27400	0.89400	15.01100
H	-18.18100	0.66300	15.21000
H	-16.77600	0.09800	15.19900
O	-14.36700	9.60400	17.93100
H	-15.04100	9.90700	18.53900
H	-13.98000	10.40700	17.58100
O	-15.15100	-2.90000	27.42100
H	-16.06900	-2.67800	27.26300
H	-14.73500	-2.82500	26.56200
O	-14.36700	-1.50100	22.55100
H	-13.62000	-0.98900	22.24200
H	-14.09800	-1.82200	23.41100
O	-16.09500	0.99500	19.53400
H	-17.00400	0.71100	19.63100
H	-16.06500	1.41500	18.67400
O	-10.86300	4.60600	23.30300
H	-11.31700	3.76500	23.35900
H	-11.49300	5.24200	23.64000
O	-12.42000	6.44300	24.23800
H	-12.12700	7.00400	23.51900
H	-11.91000	6.73600	24.99300
O	-11.81800	-11.68700	25.12700
H	-11.51200	-12.54800	24.84000
H	-11.09300	-11.09600	24.92700
O	-25.52600	-1.28400	23.51500
H	-25.72300	-1.87700	22.79000
H	-26.08100	-1.58900	24.23300
O	-17.54600	-1.39200	25.63500
H	-17.49100	-2.34500	25.55900
H	-18.35700	-1.23600	26.11900
O	-16.39700	2.53600	16.87200
H	-16.83500	1.82000	16.41300
H	-15.65600	2.76400	16.31100
O	-19.60700	-2.77400	23.39400
H	-20.42000	-2.47500	23.80000
H	-19.11100	-3.16900	24.11100
O	-17.45600	7.22100	25.32700
H	-17.49900	7.44800	24.39800
H	-16.54000	6.98800	25.47600
O	-14.01000	-2.31000	32.22500
H	-13.65500	-2.08300	33.08500
H	-13.36200	-1.97800	31.60300
O	-8.28000	0.81200	22.93000
H	-8.41800	1.00200	22.00200
H	-8.63600	1.57400	23.38700
O	-20.11600	-5.20200	29.01000
H	-19.47600	-5.34400	28.31200
H	-20.94800	-5.48900	28.63400
O	-8.25300	2.87900	27.94100
H	-7.81900	2.78700	28.78900
H	-7.54000	2.85500	27.30300
O	-19.34000	4.09800	25.12300
H	-18.54600	3.60300	24.92200
H	-20.05000	3.57000	24.76000
O	-14.52000	6.48000	29.47400
H	-14.46600	7.04900	30.24200
H	-15.38900	6.65000	29.11000
O	-23.03100	-1.75800	21.19900
H	-23.96200	-1.81900	21.41600
H	-22.93600	-2.28000	20.40300

O	-23.11300	-5.23200	23.78700
H	-22.85200	-4.31800	23.67800
H	-22.29100	-5.70000	23.93700
O	-21.64000	-3.81900	30.95700
H	-20.96800	-4.02700	30.30800
H	-22.46700	-4.01600	30.51700
O	-7.39100	2.92200	30.47400
H	-7.19800	3.82600	30.72000
H	-7.49100	2.46200	31.30800
O	-15.73800	-7.19200	32.97400
H	-16.38500	-6.49300	32.88000
H	-14.96800	-6.75900	33.34100
O	-12.33000	3.07200	36.04900
H	-12.48100	2.84300	35.13200
H	-12.33900	4.02900	36.06200
O	-15.56100	0.64600	31.03300
H	-14.63200	0.79700	30.86000
H	-15.98200	0.73400	30.17800
O	-16.76200	4.67100	28.80300
H	-16.90100	4.30300	29.67600
H	-17.55100	5.18500	28.63100
O	-16.16800	9.77200	20.14600
H	-15.99200	9.30000	20.96000
H	-16.85400	10.39800	20.37400
O	-15.21200	4.04900	26.19200
H	-15.54700	4.00900	27.08800
H	-15.71200	3.38300	25.71900
O	-21.23800	-0.54400	31.71700
H	-21.37600	-1.27600	32.31800
H	-20.32700	-0.63300	31.43600
O	-12.27600	1.39400	16.46600
H	-12.89300	0.66900	16.36900
H	-12.74400	2.15500	16.12200
O	-18.60900	-0.83200	31.20600
H	-17.89900	-1.47300	31.16400
H	-18.65200	-0.58100	32.12800
O	-10.62100	6.10400	34.12600
H	-10.67900	5.42000	33.45900
H	-10.21900	6.84600	33.67400
O	-19.89600	-3.84600	33.02500
H	-20.43300	-4.29700	33.67700
H	-20.48500	-3.71100	32.28200
O	-21.60200	12.92100	26.92000
H	-21.36200	13.55200	27.59700
H	-22.44500	12.56900	27.20600
O	-20.48600	-4.38700	15.58800
H	-21.36300	-4.50800	15.22400
H	-20.01000	-5.17800	15.33600
O	-10.61900	4.06800	32.35800
H	-11.50600	3.73100	32.48000
H	-10.04900	3.33700	32.59400
O	-28.96600	0.40100	25.68200
H	-28.27900	0.08700	25.09400
H	-29.12100	1.30500	25.40900
O	-24.16200	-4.71000	30.11000
H	-24.24700	-5.31800	29.37500
H	-24.98400	-4.21900	30.11100
O	-17.08900	8.72800	29.32400
H	-17.16200	9.53400	29.83600
H	-17.53100	8.92800	28.49900
O	-17.30000	7.96600	22.83400
H	-18.08300	8.50800	22.92800
H	-16.60000	8.48300	23.23300
O	-13.99000	-6.22500	25.47700
H	-13.49300	-6.97800	25.15800
H	-14.76100	-6.18800	24.91200
O	-6.13700	-0.52100	27.11900
H	-6.14800	0.36200	26.74900
H	-7.00500	-0.87400	26.92500
O	-14.62900	8.62700	31.48000
H	-13.91600	9.26200	31.40700
H	-15.12000	8.72400	30.66400
O	-12.90700	2.53200	23.05100
H	-13.84100	2.65200	22.88200
H	-12.72900	1.63000	22.78700
O	-17.40700	-4.08300	25.20500
H	-17.77700	-4.63600	25.89300
H	-16.91100	-4.68700	24.65200
O	-19.57800	6.37700	32.30000
H	-18.87700	7.01000	32.45300
H	-20.04800	6.71800	31.54000
O	-11.45200	4.71800	20.58500
H	-11.17600	4.60400	21.49400
H	-12.32200	4.32000	20.54600
O	-11.54800	-9.25800	27.62500
H	-12.46200	-9.02800	27.79400
H	-11.11900	-9.17400	28.47700
O	-11.77800	-1.06900	30.12500
H	-12.43000	-0.82100	29.47100
H	-11.40000	-1.88300	29.79200
O	-14.81700	8.96700	24.22800
H	-14.63500	8.14900	24.69100
H	-13.99400	9.18400	23.79100
O	-14.48300	7.89400	21.49000
H	-13.95400	7.38300	20.87700
H	-14.22000	7.57800	22.35400
O	-15.72900	-0.32700	36.61200
H	-16.40500	-0.42800	37.28200
H	-14.90600	-0.48300	37.07600
O	-23.98100	5.91000	29.23300
H	-23.39600	5.16500	29.36600
H	-24.17800	6.22100	30.11700
O	-21.12700	8.94500	24.24900
H	-21.79600	9.43000	23.76600
H	-21.26600	9.19200	25.16400
O	-29.38900	-1.51900	27.95400
H	-29.35100	-2.05300	28.74700
H	-29.89500	-2.04600	27.33500
O	-16.98700	10.98000	30.84800
H	-16.43500	11.14300	31.61300
H	-16.64600	11.57000	30.17600
O	-22.01000	-6.67300	31.45000
H	-21.94600	-5.81100	31.03800
H	-22.07300	-7.28500	30.71700
O	-22.53500	-4.60000	35.09700
H	-21.90800	-4.98300	35.71100
H	-23.39200	-4.81900	35.46300
O	-12.13500	5.80600	36.70300

H	-11.59100	6.41900	36.20800
H	-12.71700	6.36400	37.21900
O	-25.75800	-2.79300	21.02900
H	-25.46300	-3.70200	20.97600
H	-26.65900	-2.81400	20.70700
O	-13.85000	8.92800	27.83400
H	-13.97500	8.12700	27.32500
H	-13.19200	9.42300	27.34500
O	-22.22900	-4.71700	27.01200
H	-22.95000	-4.10400	26.87300
H	-21.44000	-4.18100	26.93300
O	-11.78600	6.72900	29.46200
H	-12.73300	6.60900	29.39000
H	-11.43800	5.84100	29.54500
O	-19.42600	-7.38200	33.28600
H	-18.90300	-7.13600	34.04900
H	-19.64500	-6.55000	32.86600
O	-24.03000	-5.11100	21.15300
H	-24.35800	-5.99200	20.97000
H	-23.67600	-5.16700	22.04100
O	-14.78200	6.52400	25.66200
H	-14.92900	5.59600	25.84300
H	-13.95600	6.54600	25.17800
O	-20.91500	-4.82400	21.52100
H	-20.57600	-4.29600	20.79800
H	-20.46800	-4.48600	22.29600
O	-11.17800	7.34700	26.74900
H	-10.25300	7.14100	26.61100
H	-11.34700	7.09900	27.65800
O	-18.22100	-6.44000	30.97100
H	-18.56100	-7.24000	31.37300
H	-18.92700	-6.14000	30.39900
O	-17.56900	-9.35500	29.42400
H	-16.74700	-9.82900	29.30100
H	-17.32600	-8.57600	29.92300
O	-18.92900	-6.32700	19.72000
H	-18.20800	-5.94900	19.21600
H	-19.55200	-5.60800	19.82300
O	-14.78500	-5.62200	20.65600
H	-14.56900	-4.74000	20.35500
H	-15.26600	-6.01400	19.92600
O	-20.04500	-2.66400	27.23300
H	-20.19100	-2.35000	28.12500
H	-20.52300	-2.04500	26.68000
O	-18.18600	-5.67300	27.28100
H	-18.20000	-6.56900	26.94400
H	-17.46900	-5.66600	27.91500
O	-16.33300	-5.24000	29.32600
H	-16.59100	-4.31800	29.32500
H	-16.86500	-5.63700	30.01600
O	-11.38900	7.86600	22.10500
H	-11.21500	7.30000	21.35300
H	-10.89600	8.66600	21.92300
O	-25.57100	-0.12300	30.17200
H	-26.11600	0.65300	30.30400
H	-24.69300	0.22400	30.01300
O	-18.78200	-6.79200	22.55000
H	-18.66100	-7.72000	22.74900
H	-19.07200	-6.77800	21.63700
O	-15.91300	-9.10600	25.68600
H	-15.89100	-9.04200	24.73100
H	-15.49600	-9.94600	25.88000
O	-5.53500	0.62900	23.72400
H	-5.09200	-0.12000	23.32600
H	-6.41200	0.62000	23.34200
O	-10.40100	7.18200	19.20500
H	-9.70600	7.80300	19.42400
H	-10.05900	6.33200	19.48000
O	-24.78400	-0.99600	33.06500
H	-25.52600	-0.84800	33.65100
H	-25.10500	-0.73500	32.20100
O	-8.50200	4.63200	20.73400
H	-7.97700	4.97100	20.00900
H	-8.59000	3.69700	20.55000
O	-16.84000	8.54600	32.86300
H	-16.53100	8.30000	33.73500
H	-16.04200	8.75000	32.37400
O	-16.81800	10.69900	25.09300
H	-17.25700	10.20700	25.78600
H	-16.06700	10.15700	24.85200
O	-24.33900	6.89100	25.09600
H	-23.51300	6.59100	24.71500
H	-24.68900	6.12100	25.54500
O	-19.37000	6.24300	27.16500
H	-19.99200	5.58000	26.86600
H	-18.70000	6.26900	26.48300
O	-26.13800	4.80900	26.05400
H	-26.97500	4.38100	25.87300
H	-26.16500	5.00200	26.99200
O	-19.29800	-5.27900	36.75900
H	-19.24900	-4.40200	36.37600
H	-18.75600	-5.82100	36.18600
O	-8.81300	5.26500	36.03900
H	-9.60300	5.24800	35.49900
H	-9.04900	5.80400	36.79400
O	-8.69300	2.11500	33.35200
H	-8.64300	1.95000	34.29400
H	-8.80100	1.24700	32.96200
O	-19.80900	4.79400	20.64100
H	-19.66000	5.47700	19.98800
H	-19.00200	4.77400	21.15600
O	-11.40200	-1.90200	34.66800
H	-10.50100	-2.04200	34.37600
H	-11.32800	-1.73700	35.60800
O	-17.06800	-4.52600	33.51300
H	-17.93800	-4.23200	33.24200
H	-16.83200	-3.94100	34.23400
O	-7.19100	4.53400	33.59600
H	-7.48200	4.89700	34.43300
H	-7.80400	3.82100	33.41800
O	-5.99100	2.10000	26.11600
H	-5.15700	2.53900	26.28000
H	-5.85500	1.62400	25.29700
O	-8.94200	-0.34200	32.06400
H	-8.46700	-0.15000	31.25600
H	-9.86600	-0.28600	31.81900
O	-22.05400	0.82000	21.64700
H	-22.29900	-0.08400	21.84500

H	-21.88400	0.82200	20.70500
O	-14.36400	-8.30800	31.03000
H	-13.84600	-7.57100	30.70400
H	-14.71900	-8.00100	31.86400
O	-22.78400	-3.44700	19.14600
H	-23.47700	-3.37300	18.49000
H	-23.02900	-4.21100	19.66800
O	-14.11300	-11.88800	26.55000
H	-13.83300	-12.55500	27.17600
H	-13.32800	-11.69500	26.03700
O	-16.31000	7.28800	18.11200
H	-16.88200	7.90700	17.65900
H	-15.73600	7.83800	18.64600
O	-10.24200	-3.46600	30.29700
H	-9.29000	-3.36600	30.31800
H	-10.41200	-4.25800	30.80700
O	-12.22100	-6.38000	30.48300
H	-12.81600	-6.10000	29.78800
H	-12.50600	-5.89100	31.25500
O	-13.09000	7.57200	19.24300
H	-12.15700	7.50700	19.44700
H	-13.17800	8.40800	18.78500
O	-10.46100	6.88900	16.20400
H	-10.54000	6.55500	17.09700
H	-9.64000	7.38100	16.20300
O	-7.76300	-0.24500	29.41100
H	-7.79600	-1.18400	29.59700
H	-7.15300	-0.16700	28.67800
O	-15.92500	-1.37600	15.45400
H	-14.97900	-1.50500	15.52300
H	-16.30300	-2.11400	15.93100
O	-7.61400	-3.45400	29.45700
H	-8.13000	-4.23800	29.27000
H	-6.73000	-3.78200	29.62200
O	-9.95300	-9.71000	25.41700
H	-10.15000	-10.01800	26.30100
H	-9.34300	-8.98400	25.54600
O	-1.94600	0.81600	28.55200
H	-1.21500	0.55500	29.11300
H	-2.55500	0.07900	28.59200
O	-3.86400	-1.04300	29.14900
H	-4.59100	-1.17700	28.54100
H	-4.27900	-0.73500	29.95500
O	-16.04100	2.67600	36.91400
H	-15.12100	2.85400	37.10800
H	-16.07600	1.73200	36.76200
O	-17.74100	-3.45100	15.38900
H	-18.64700	-3.74700	15.46600
H	-17.46300	-3.74800	14.52300
O	-26.82300	-2.13000	25.83900
H	-27.14300	-2.98300	25.54700
H	-27.36900	-1.91500	26.59500
O	-23.04500	0.58600	29.55600
H	-22.82600	1.51800	29.57500
H	-22.63200	0.22700	30.34200
O	-16.12400	-8.90700	21.54400
H	-16.93400	-9.25200	21.91900
H	-15.43600	-9.21600	22.13300
O	-14.19500	-9.82900	23.16600
H	-13.64200	-9.41000	23.82500
H	-13.95800	-10.75500	23.20900
O	-18.17200	-3.84000	38.94700
H	-18.27200	-2.89100	38.87800
H	-18.39000	-4.16900	38.07400
O	-18.97100	-6.59200	15.53100
H	-18.10900	-6.26600	15.79000
H	-18.78600	-7.37900	15.01800
O	-26.95400	1.49000	27.80900
H	-27.64800	1.07600	27.29600
H	-26.20700	1.53200	27.21200
O	-10.81700	-7.71900	22.67400
H	-11.39800	-7.06100	22.29200
H	-11.08400	-8.54100	22.26300
O	-12.36700	-8.31900	24.87600
H	-11.86700	-8.20700	24.06800
H	-11.72400	-8.62200	25.51600
O	-18.30900	-0.41100	37.76300
H	-18.79200	0.38900	37.97000
H	-18.22300	-0.40000	36.81000
O	-25.04000	1.42200	25.68500
H	-25.06300	1.52100	24.73300
H	-24.73000	2.26900	26.00700
O	-22.38200	3.48000	29.80000
H	-21.42700	3.44800	29.73300
H	-22.58000	3.01900	30.61500
O	-21.79200	2.05000	32.36700
H	-20.97200	2.54400	32.39100
H	-21.51800	1.13300	32.33900
O	-8.68400	-5.53400	25.44500
H	-7.90800	-5.04500	25.71800
H	-9.40900	-5.10900	25.90200
O	-6.24600	-4.82200	26.50300
H	-5.65200	-5.06200	25.79200
H	-6.17400	-5.54100	27.13000
O	-24.02700	9.02200	26.81700
H	-24.16100	8.26200	26.25100
H	-24.44300	9.74600	26.35000
O	-12.89100	4.20500	15.82500
H	-12.18700	4.41000	16.44000
H	-13.61400	4.77300	16.09000
O	-12.06100	-0.04400	21.94500
H	-12.11700	0.41300	21.10600
H	-11.50800	-0.80400	21.76700
O	-3.93900	-1.51500	25.27700
H	-4.49900	-1.44600	26.05000
H	-4.54500	-1.69100	24.55700
O	-20.87000	9.91900	27.02900
H	-20.98600	10.86300	26.92700
H	-21.69000	9.61600	27.41700
O	-10.98200	4.64400	17.87400
H	-10.14700	4.21200	17.69200
H	-11.14400	4.47200	18.80200
O	-11.32700	1.14700	19.24000
H	-11.38300	0.98100	18.29900
H	-11.95300	1.85500	19.39600
O	-26.53800	-3.61600	33.06600
H	-25.89200	-4.12600	33.55500
H	-26.74600	-2.87900	33.64100

O	-14.01300	3.45200	38.56500
H	-13.14400	3.62600	38.20400
H	-14.49200	4.27100	38.44300
O	-18.01800	-9.35200	18.81900
H	-18.03000	-8.40800	18.97500
H	-17.12500	-9.53500	18.52500
O	-19.84400	10.57200	30.24400
H	-18.94100	10.60400	30.56100
H	-20.07000	9.64200	30.25900
O	-18.08900	9.32500	26.90500
H	-19.03900	9.32500	26.79200
H	-17.78600	8.57800	26.38900
O	-14.20300	-12.75800	23.07300
H	-14.96900	-13.31200	23.21900
H	-13.79200	-12.68600	23.93500
O	-22.19800	-6.47000	37.16400
H	-21.33500	-6.82600	37.37400
H	-22.77200	-6.80800	37.85100
O	-2.35400	15.16100	27.97000
H	-1.71600	15.04100	27.26600
H	-2.47100	16.10900	28.02900
O	-5.54500	15.11300	27.90600
H	-5.88300	14.99800	27.01800
H	-4.67600	14.71300	27.88600
O	-9.68600	16.96700	23.89600
H	-10.24900	16.87600	23.12700
H	-9.80100	16.14700	24.37600
O	-5.06300	16.26700	33.86500
H	-5.07400	15.87200	32.99300
H	-4.90100	15.53400	34.45800
O	-12.13900	18.46200	26.73400
H	-11.34700	18.12100	26.31900
H	-11.90000	18.57200	27.65400
O	-4.10600	20.63400	19.20700
H	-4.75700	21.21900	18.82100
H	-3.31200	21.16500	19.26700
O	-10.18000	17.72300	19.60600
H	-9.40700	17.17600	19.46700
H	-9.82800	18.58700	19.81700
O	-8.34200	19.05100	32.81300
H	-8.63300	19.95500	32.69700
H	-7.70600	19.09200	33.52700
O	-7.41600	16.42800	29.40300
H	-6.82000	16.20400	28.68900
H	-7.35900	15.68700	30.00500
O	-7.84400	13.88200	17.23800
H	-7.59100	13.36000	16.47600
H	-8.78100	13.71900	17.34100
O	-2.79900	12.88600	25.04500
H	-2.01300	12.46800	24.69400
H	-2.53600	13.79000	25.21300
O	-2.62800	18.32300	19.74400
H	-3.26400	18.39700	20.45600
H	-2.95900	18.91200	19.06600
O	2.60100	12.82300	25.94900
H	3.33400	12.25300	26.18200
H	1.84500	12.43600	26.38900
O	-8.12400	15.78500	19.44000
H	-8.24100	15.11000	20.10800
H	-8.01400	15.29800	18.62300
O	-5.78100	15.24600	23.14500
H	-6.14500	14.71900	23.85600
H	-4.92400	14.85600	22.97200
O	0.12400	6.53500	30.40800
H	-0.20800	7.40500	30.18400
H	-0.47200	6.21900	31.08700
O	-6.14000	10.27500	18.14100
H	-5.97300	11.14300	18.50800
H	-6.33100	10.43800	17.21700
O	-3.38300	18.73700	29.80800
H	-3.68300	19.63900	29.69900
H	-3.72800	18.47000	30.66000
O	-9.93200	15.18800	25.84100
H	-10.85700	15.05600	26.04900
H	-9.68300	15.96800	26.33800
O	0.01700	11.25500	27.48700
H	-0.89200	11.18700	27.19300
H	-0.03400	11.73200	28.31600
O	-6.16200	12.73100	19.12600
H	-6.28200	13.17700	19.96400
H	-6.85600	13.07900	18.56700
O	-12.57100	14.83100	26.67600
H	-12.44200	15.08500	27.59000
H	-13.25100	15.42400	26.35600
O	-8.40100	25.50100	22.77500
H	-8.47800	26.45200	22.85400
H	-9.00500	25.15600	23.43200
O	-5.90900	12.67400	35.43100
H	-6.68600	13.23300	35.42100
H	-5.27000	13.16000	35.95200
O	1.88300	9.45700	29.26500
H	1.24000	9.75000	28.61900
H	1.37800	9.34200	30.07100
O	-0.46300	13.20300	19.95400
H	0.44900	13.43600	19.77900
H	-0.97200	13.78700	19.39100
O	-7.65400	15.48300	34.99300
H	-6.91300	16.02100	34.71600
H	-8.36100	16.10900	35.15000
O	-2.17900	17.62500	33.59000
H	-1.77100	17.18400	32.84500
H	-2.90100	17.05100	33.84300
O	-5.82600	12.56300	30.96300
H	-4.98000	12.98700	31.10700
H	-5.88600	11.90600	31.65700
O	-1.85600	18.18600	27.34500
H	-2.48500	18.35000	26.64300
H	-2.24300	18.60000	28.11600
O	2.02300	19.12100	24.70000
H	1.33800	19.50100	24.14900
H	2.10700	19.73100	25.43300
O	-2.67600	10.22300	32.62100
H	-2.92000	10.97700	32.08500
H	-2.40000	10.60200	33.45600
O	-0.72200	16.76700	31.29900
H	-0.51000	17.41000	30.62300
H	-0.76900	15.93300	30.83100
O	-3.11900	9.37900	28.20300

H	-3.98100	9.67700	28.49400
H	-3.09300	9.59600	27.27100
O	-6.30300	17.92300	24.61000
H	-5.83900	18.20200	23.82100
H	-6.56800	17.02100	24.42700
O	-9.41100	17.47000	27.54800
H	-8.84100	17.89900	26.91000
H	-8.82000	17.18800	28.24700
O	-7.36600	14.04500	21.41900
H	-6.66400	14.49700	21.88600
H	-7.69800	13.40400	22.04700
O	-8.91800	12.63600	35.79400
H	-9.54800	13.31800	36.02800
H	-9.02500	12.52500	34.85000
O	-13.62400	12.30400	26.72100
H	-14.41400	12.55900	26.24500
H	-13.13300	13.11800	26.82600
O	0.39300	19.64400	29.47100
H	1.21400	19.24200	29.19000
H	0.38900	19.54100	30.42300
O	-11.67200	12.66800	23.67900
H	-12.46900	12.78000	24.19800
H	-11.90000	13.00700	22.81300
O	-5.84200	19.19300	31.62700
H	-5.77700	19.65500	32.46300
H	-6.55100	18.56200	31.75600
O	-15.34000	12.55700	29.15200
H	-15.08900	13.44100	29.41800
H	-14.80400	12.37700	28.38000
O	8.48300	15.98800	25.82000
H	7.73000	15.41900	25.66400
H	8.61800	15.95400	26.76700
O	-6.12700	20.85900	21.49500
H	-6.26200	21.70500	21.92400
H	-5.58600	21.06100	20.73200
O	-9.64000	6.93900	38.15700
H	-10.03400	6.38500	38.83200
H	-8.82400	7.25000	38.54700
O	-12.67400	18.37700	22.06800
H	-12.16200	19.17100	22.21900
H	-12.02200	17.68000	22.00100
O	-10.86500	21.49400	24.15000
H	-10.68800	22.41500	23.96200
H	-10.06900	21.17900	24.58000
O	-6.72300	8.35400	35.74900
H	-5.98300	8.95500	35.66900
H	-6.49200	7.61200	35.19100
O	-11.57900	13.54000	34.02700
H	-11.69400	12.63900	34.32700
H	-12.43500	13.78600	33.67500
O	-4.50000	21.48200	35.62500
H	-4.94400	20.69500	35.30700
H	-3.83800	21.67000	34.96000
O	-9.74100	23.01100	30.15100
H	-9.19100	22.88300	29.37900
H	-10.58000	23.31100	29.80100
O	-2.40200	21.76200	33.65300
H	-2.42000	22.58300	33.16200
H	-1.48000	21.63700	33.87900
O	-5.94500	19.27600	34.49600
H	-5.41200	18.51500	34.26600
H	-6.47300	18.98400	35.23800
O	-2.77800	4.38200	30.02500
H	-3.12100	5.27300	29.94900
H	-2.41000	4.19300	29.16200
O	-14.70900	16.70600	22.84400
H	-13.87200	17.11600	22.62700
H	-14.53100	15.76600	22.81400
O	-2.81300	14.37300	18.53200
H	-3.32800	15.14400	18.77100
H	-3.08500	13.70000	19.15600
O	-5.22800	9.21800	23.59100
H	-6.08400	8.82900	23.41200
H	-4.81500	9.29700	22.73100
O	-3.69800	6.75300	27.99000
H	-4.20200	6.71600	27.17700
H	-3.40500	7.66200	28.05000
O	-7.45400	6.83700	26.99700
H	-6.58900	6.64100	26.63700
H	-7.38600	6.60800	27.92400
O	2.16100	17.12700	20.46800
H	1.38400	16.78800	20.02300
H	2.84000	17.13200	19.79300
O	-3.02400	12.24600	20.35800
H	-3.32900	12.56100	21.20900
H	-2.07100	12.32400	20.40100
O	-4.57900	18.40500	22.00800
H	-5.10700	17.67000	21.69500
H	-5.08100	19.18300	21.76200
O	0.40000	16.60000	27.12400
H	-0.25800	17.29000	27.21200
H	1.20400	16.99000	27.46800
O	-9.84800	9.06700	24.36800
H	-9.27300	8.84500	23.63600
H	-9.26000	9.13600	25.12100
O	-0.79000	14.95400	25.17500
H	-0.27600	15.58700	25.67700
H	-0.14000	14.35500	24.80900
O	-9.52100	11.09700	18.38800
H	-10.35700	11.43500	18.70900
H	-9.66000	10.96200	17.45100
O	-7.54500	5.94100	23.09600
H	-8.19900	5.62600	22.47200
H	-7.58700	5.31900	23.82200
O	-6.43400	16.22300	15.01100
H	-7.34100	15.99200	15.21000
H	-5.93700	15.42700	15.19900
O	-3.52700	24.93300	17.93100
H	-4.20100	25.23600	18.53900
H	-3.14100	25.73600	17.58100
O	-4.31200	12.42900	27.42100
H	-5.22900	12.65100	27.26300
H	-3.89600	12.50400	26.56200
O	-3.52800	13.82800	22.55100
H	-2.78100	14.34100	22.24200
H	-3.25900	13.50700	23.41100
O	-5.25500	16.32400	19.53400
H	-6.16400	16.04000	19.63100

H	-5.22600	16.74400	18.67400
O	-0.02300	19.93500	23.30300
H	-0.47700	19.09400	23.35900
H	-0.65400	20.57100	23.64000
O	-1.08000	10.86400	24.53300
H	-1.81800	10.35200	24.86400
H	-0.82700	10.42400	23.72100
O	-1.58000	21.77300	24.23800
H	-1.28800	22.33300	23.51900
H	-1.07000	22.06500	24.99300
O	-14.68700	14.04500	23.51500
H	-14.88400	13.45200	22.79000
H	-15.24200	13.74000	24.23300
O	-6.70700	13.93700	25.63500
H	-6.65200	12.98500	25.55900
H	-7.51800	14.09300	26.11900
O	-5.55800	17.86500	16.87200
H	-5.99600	17.14900	16.41300
H	-4.81700	18.09300	16.31100
O	-8.76800	12.55500	23.39400
H	-9.58100	12.85400	23.80000
H	-8.27200	12.16000	24.11100
O	-6.61700	22.55100	25.32700
H	-6.65900	22.77700	24.39800
H	-5.70100	22.31700	25.47600
O	-3.17000	13.01900	32.22500
H	-2.81500	13.24600	33.08500
H	-2.52300	13.35100	31.60300
O	2.56000	16.14100	22.93000
H	2.42100	16.33100	22.00200
H	2.20300	16.90300	23.38700
O	-9.27600	10.12700	29.01000
H	-8.63600	9.98600	28.31200
H	-10.10800	9.84000	28.63400
O	2.58600	18.20900	27.94100
H	3.02000	18.11600	28.78900
H	3.30000	18.18400	27.30300
O	-8.50000	19.42700	25.12300
H	-7.70600	18.93200	24.92200
H	-9.21100	18.89900	24.76000
O	-3.68100	21.80900	29.47400
H	-3.62700	22.37800	30.24200
H	-4.54900	21.97900	29.11000
O	-12.19200	13.57100	21.19900
H	-13.12200	13.51000	21.41600
H	-12.09600	13.04900	20.40300
O	-12.27300	10.09700	23.78700
H	-12.01300	11.01100	23.67800
H	-11.45200	9.62900	23.93700
O	-10.80100	11.51100	30.95700
H	-10.12900	11.30200	30.30800
H	-11.62800	11.31300	30.51700
O	3.44800	18.25100	30.47400
H	3.64200	19.15600	30.72000
H	3.34800	17.79200	31.30800
O	-4.89800	8.13700	32.97400
H	-5.54600	8.83600	32.88000
H	-4.12800	8.57000	33.34100
O	-1.49000	18.40200	36.04900
H	-1.64200	18.17300	35.13200
H	-1.50000	19.35900	36.06200
O	-4.72200	15.97500	31.03300
H	-3.79300	16.12600	30.86000
H	-5.14300	16.06300	30.17800
O	-5.92300	20.00000	28.80300
H	-6.06200	19.63200	29.67600
H	-6.71200	20.51500	28.63100
O	-5.32800	25.10100	20.14600
H	-5.15300	24.63000	20.96000
H	-6.01500	25.72700	20.37400
O	-4.37300	19.37800	26.19200
H	-4.70800	19.33800	27.08800
H	-4.87200	18.71300	25.71900
O	-10.39800	14.78500	31.71700
H	-10.53600	14.05300	32.31800
H	-9.48800	14.69600	31.43600
O	-1.43700	16.72400	16.46600
H	-2.05400	15.99800	16.36900
H	-1.90500	17.48500	16.12200
O	-7.77000	14.49700	31.20600
H	-7.06000	13.85600	31.16400
H	-7.81300	14.74800	32.12800
O	0.21900	21.43400	34.12600
H	0.16000	20.74900	33.45900
H	0.62100	22.17500	33.67400
O	-9.05700	11.48300	33.02500
H	-9.59300	11.03200	33.67700
H	-9.64500	11.61800	32.28200
O	-10.76200	28.25000	26.92000
H	-10.52200	28.88200	27.59700
H	-11.60500	27.89900	27.20600
O	-9.64700	10.94200	15.58800
H	-10.52400	10.82100	15.22400
H	-9.17100	10.15100	15.33600
O	0.22100	19.39700	32.35800
H	-0.66700	19.06000	32.48000
H	0.79000	18.66600	32.59400
O	-18.12700	15.73000	25.68200
H	-17.43900	15.41600	25.09400
H	-18.28200	16.63400	25.40900
O	-13.32300	10.61900	30.11000
H	-13.40700	10.01100	29.37500
H	-14.14500	11.11000	30.11100
O	-6.24900	24.05800	29.32400
H	-6.32200	24.86300	29.83600
H	-6.69200	24.25700	28.49900
O	-6.46000	23.29500	22.83400
H	-7.24400	23.83700	22.92800
H	-5.76100	23.81200	23.23300
O	-3.15100	9.10400	25.47700
H	-2.65400	8.35100	25.15800
H	-3.92200	9.14100	24.91200
O	4.70200	14.80800	27.11900
H	4.69100	15.69100	26.74900
H	3.83400	14.45500	26.92500
O	-3.79000	23.95600	31.48000
H	-3.07700	24.59100	31.40700
H	-4.28100	24.05300	30.66400

O	-2.06800	17.86100	23.05100
H	-3.00200	17.98200	22.88200
H	-1.89000	16.95900	22.78700
O	-6.56700	11.24600	25.20500
H	-6.93700	10.69300	25.89300
H	-6.07100	10.64200	24.65200
O	1.32000	13.70300	23.58600
H	1.78600	14.53600	23.51200
H	1.77000	13.23800	24.29100
O	-8.73800	21.70600	32.30000
H	-8.03700	22.33900	32.45300
H	-9.20900	22.04700	31.54000
O	-0.61300	20.04800	20.58500
H	-0.33700	19.93300	21.49400
H	-1.48200	19.64900	20.54600
O	-0.70900	6.07100	27.62500
H	-1.62300	6.30200	27.79400
H	-0.28000	6.15500	28.47700
O	-0.93800	14.26000	30.12500
H	-1.59100	14.50800	29.47100
H	-0.56000	13.44700	29.79200
O	-3.97700	24.29600	24.22800
H	-3.79500	23.47800	24.69100
H	-3.15400	24.51300	23.79100
O	-3.64300	23.22300	21.49000
H	-3.11400	22.71200	20.87700
H	-3.38000	22.90700	22.35400
O	-4.88900	15.00200	36.61200
H	-5.56500	14.90200	37.28200
H	-4.06700	14.84600	37.07600
O	-13.14200	21.23900	29.23300
H	-12.55600	20.49400	29.36600
H	-13.33900	21.55000	30.11700
O	-10.28800	24.27500	24.24900
H	-10.95700	24.75900	23.76600
H	-10.42600	24.52100	25.16400
O	-18.55000	13.81000	27.95400
H	-18.51100	13.27600	28.74700
H	-19.05600	13.28300	27.33500
O	-6.14700	26.30900	30.84800
H	-5.59500	26.47200	31.61300
H	-5.80700	26.89900	30.17600
O	-11.17100	8.65600	31.45000
H	-11.10700	9.51800	31.03800
H	-11.23400	8.04400	30.71700
O	-11.69500	10.72900	35.09700
H	-11.06800	10.34600	35.71100
H	-12.55300	10.51000	35.46300
O	-1.29600	21.13500	36.70300
H	-0.75200	21.74800	36.20800
H	-1.87700	21.69300	37.21900
O	-14.91900	12.53600	21.02900
H	-14.62400	11.62700	20.97600
H	-15.82000	12.51500	20.70700
O	-3.01000	24.25700	27.83400
H	-3.13500	23.45600	27.32500
H	-2.35200	24.75200	27.34500
O	-11.38900	10.61200	27.01200
H	-12.11100	11.22600	26.87300
H	-10.60000	11.14800	26.93300
O	-0.94700	22.05800	29.46200
H	-1.89400	21.93900	29.39000
H	-0.59900	21.17000	29.54500
O	-8.58700	7.94700	33.28600
H	-8.06300	8.19400	34.04900
H	-8.80600	8.77900	32.86600
O	-13.19000	10.21800	21.15300
H	-13.51900	9.33800	20.97000
H	-12.83600	10.16300	22.04100
O	-3.94200	21.85300	25.66200
H	-4.09000	20.92500	25.84300
H	-3.11700	21.87500	25.17800
O	-10.07600	10.50500	21.52100
H	-9.73600	11.03400	20.79800
H	-9.62900	10.84300	22.29600
O	-0.33900	22.67600	26.74900
H	0.58600	22.47000	26.61100
H	-0.50700	22.42800	27.65800
O	-7.38200	8.88900	30.97100
H	-7.72200	8.09000	31.37300
H	-8.08800	9.18900	30.39900
O	-6.73000	5.97400	29.42400
H	-5.90700	5.50000	29.30100
H	-6.48600	6.75300	29.92300
O	-8.08900	9.00300	19.72000
H	-7.36800	9.38100	19.21600
H	-8.71300	9.72100	19.82300
O	-3.94600	9.70700	20.65600
H	-3.73000	10.59000	20.35500
H	-4.42700	9.31600	19.92600
O	-9.20600	12.66600	27.23300
H	-9.35200	12.98000	28.12500
H	-9.68400	13.28400	26.68000
O	-7.34600	9.65600	27.28100
H	-7.36000	8.76000	26.94400
H	-6.62900	9.66300	27.91500
O	-5.49400	10.09000	29.32600
H	-5.75200	11.01100	29.32500
H	-6.02500	9.69300	30.01600
O	-0.54900	23.19500	22.10500
H	-0.37600	22.62900	21.35300
H	-0.05600	23.99500	21.92300
O	-14.73200	15.20600	30.17200
H	-15.27600	15.98200	30.30400
H	-13.85400	15.55300	30.01300
O	-7.94300	8.53700	22.55000
H	-7.82100	7.60900	22.74900
H	-8.23300	8.55100	21.63700
O	-5.07400	6.22300	25.68600
H	-5.05100	6.28700	24.73100
H	-4.65700	5.38300	25.88000
O	5.30500	15.95800	23.72400
H	5.74800	15.20900	23.32600
H	4.42700	15.94900	23.34200
O	0.43800	22.51100	19.20500
H	1.13300	23.13200	19.42400
H	0.78100	21.66100	19.48000
O	-13.94500	14.33300	33.06500

H	-14.68700	14.48100	33.65100
H	-14.26600	14.59400	32.20100
O	2.33700	19.96100	20.73400
H	2.86300	20.30000	20.00900
H	2.25000	19.02600	20.55000
O	-6.00100	23.87500	32.86300
H	-5.69200	23.63000	33.73500
H	-5.20300	24.07900	32.37400
O	-5.97900	26.02900	25.09300
H	-6.41800	25.53600	25.78600
H	-5.22800	25.48600	24.85200
O	-13.49900	22.22000	25.09600
H	-12.67300	21.92000	24.71500
H	-13.85000	21.45000	25.54500
O	-8.53100	21.57200	27.16500
H	-9.15300	20.90900	26.86600
H	-7.86100	21.59800	26.48300
O	-15.29800	20.13800	26.05400
H	-16.13500	19.71100	25.87300
H	-15.32600	20.33200	26.99200
O	-8.45900	10.05100	36.75900
H	-8.41000	10.92700	36.37600
H	-7.91600	9.50800	36.18600
O	2.02700	20.59500	36.03900
H	1.23600	20.57700	35.49900
H	1.79000	21.13300	36.79400
O	2.14700	17.44400	33.35200
H	2.19600	17.27900	34.29400
H	2.03800	16.57700	32.96200
O	-8.97000	20.12300	20.64100
H	-8.82100	20.80600	19.98800
H	-8.16300	20.10300	21.15600
O	-0.56300	13.42700	34.66800
H	0.33800	13.28800	34.37600
H	-0.48800	13.59200	35.60800
O	-6.22900	10.80400	33.51300
H	-7.09800	11.09700	33.24200
H	-5.99300	11.38800	34.23400
O	3.64800	19.86400	33.59600
H	3.35700	20.22700	34.43300
H	3.03600	19.15000	33.41800
O	4.84800	17.42900	26.11600
H	5.68300	17.86800	26.28000
H	4.98400	16.95300	25.29700
O	1.89700	14.98700	32.06400
H	2.37300	15.18000	31.25600
H	0.97400	15.04300	31.81900
O	-11.21500	16.14900	21.64700
H	-11.46000	15.24500	21.84500
H	-11.04500	16.15100	20.70500
O	-3.52500	7.02200	31.03000
H	-3.00700	7.75800	30.70400
H	-3.87900	7.32800	31.86400
O	-11.94500	11.88200	19.14600
H	-12.63800	11.95700	18.49000
H	-12.19000	11.11800	19.66800
O	-3.27400	3.44200	26.55000
H	-2.99300	2.77400	27.17600
H	-2.48900	3.63500	26.03700
O	-5.47000	22.61800	18.11200
H	-6.04300	23.23700	17.65900
H	-4.89700	23.16700	18.64600
O	0.59700	11.86300	30.29700
H	1.54900	11.96300	30.31800
H	0.42800	11.07100	30.80700
O	-1.38100	8.94900	30.48300
H	-1.97600	9.22900	29.78800
H	-1.66600	9.43800	31.25500
O	-2.25100	22.90100	19.24300
H	-1.31700	22.83600	19.44700
H	-2.33800	23.73700	18.78500
O	0.37800	22.21800	16.20400
H	0.30000	21.88400	17.09700
H	1.19900	22.71000	16.20300
O	3.07600	15.08400	29.41100
H	3.04400	14.14600	29.59700
H	3.68600	15.16200	28.67800
O	-5.08600	13.95400	15.45400
H	-4.14000	13.82400	15.52300
H	-5.46400	13.21500	15.93100
O	3.22600	11.87500	29.45700
H	2.70900	11.09100	29.27000
H	4.11000	11.54700	29.62200
O	0.88700	5.61900	25.41700
H	0.68900	5.31100	26.30100
H	1.49700	6.34500	25.54600
O	8.89400	16.14600	28.55200
H	9.62400	15.88400	29.11300
H	8.28400	15.40800	28.59200
O	6.97500	14.28600	29.14900
H	6.24900	14.15200	28.54100
H	6.56000	14.59400	29.95500
O	-5.20200	18.00500	36.91400
H	-4.28100	18.18300	37.10800
H	-5.23700	17.06100	36.76200
O	-6.90100	11.87900	15.38900
H	-7.80800	11.58200	15.46600
H	-6.62400	11.58200	14.52300
O	-15.98300	13.19900	25.83900
H	-16.30300	12.34600	25.54700
H	-16.53000	13.41400	26.59500
O	-12.20500	15.91600	29.55600
H	-11.98700	16.84700	29.57500
H	-11.79300	15.55600	30.34200
O	-5.28500	6.42200	21.54400
H	-6.09500	6.07700	21.91900
H	-4.59700	6.11300	22.13300
O	-3.35600	5.50000	23.16600
H	-2.80300	5.92000	23.82500
H	-3.11900	4.57400	23.20900
O	-7.33300	11.48900	38.94700
H	-7.43300	12.43800	38.87800
H	-7.55100	11.16100	38.07400
O	-8.13100	8.73700	15.53100
H	-7.26900	9.06300	15.79000
H	-7.94600	7.95000	15.01800
O	-16.11500	16.82000	27.80900
H	-16.80800	16.40500	27.29600

H	-15.36700	16.86200	27.21200
O	0.02200	7.61000	22.67400
H	-0.55800	8.26900	22.29200
H	-0.24500	6.78800	22.26300
O	-1.52800	7.01100	24.87600
H	-1.02800	7.12200	24.06800
H	-0.88400	6.70700	25.51600
O	-7.47000	14.91900	37.76300
H	-7.95300	15.71900	37.97000
H	-7.38300	14.92900	36.81000
O	-14.20100	16.75100	25.68500
H	-14.22400	16.85000	24.73300
H	-13.89100	17.59800	26.00700
O	-11.54200	18.80900	29.80000
H	-10.58800	18.77700	29.73300
H	-11.74100	18.34800	30.61500
O	-10.95200	17.37900	32.36700
H	-10.13300	17.87400	32.39100
H	-10.67900	16.46200	32.33900
O	-1.19600	9.77000	21.73000
H	-2.08300	10.05500	21.51200
H	-0.78000	9.61000	20.88300
O	2.15600	9.79500	25.44500
H	2.93200	10.28400	25.71800
H	1.43100	10.22100	25.90200
O	4.59300	10.50800	26.50300
H	5.18800	10.26700	25.79200
H	4.66600	9.78800	27.13000
O	-13.18700	24.35100	26.81700
H	-13.32200	23.59100	26.25100
H	-13.60400	25.07500	26.35000
O	-2.05100	19.53500	15.82500
H	-1.34800	19.74000	16.44000
H	-2.77500	20.10200	16.09000
O	-1.22200	15.28600	21.94500
H	-1.27800	15.74200	21.10600
H	-0.66900	14.52500	21.76700
O	2.58900	11.07500	22.77000
H	2.33300	10.67400	23.60100
H	1.96400	11.78900	22.64500
O	6.90000	13.81400	25.27700
H	6.34000	13.88400	26.05000
H	6.29500	13.63800	24.55700
O	-10.03000	25.24800	27.02900
H	-10.14700	26.19200	26.92700
H	-10.85100	24.94500	27.41700
O	-0.14300	19.97300	17.87400
H	0.69200	19.54100	17.69200
H	-0.30400	19.80100	18.80200
O	-0.48800	16.47600	19.24000
H	-0.54300	16.31100	18.29900
H	-1.11300	17.18400	19.39600
O	-15.69900	11.71300	33.06600
H	-15.05300	11.20300	33.55500
H	-15.90700	12.45000	33.64100
O	-3.17300	18.78100	38.56500
H	-2.30400	18.95500	38.20400
H	-3.65300	19.60000	38.44300
O	-7.17800	5.97700	18.81900
H	-7.19100	6.92100	18.97500
H	-6.28600	5.79400	18.52500
O	-9.00500	25.90100	30.24400
H	-8.10200	25.93300	30.56100
H	-9.23100	24.97100	30.25900
O	-7.24900	24.65400	26.90500
H	-8.20000	24.65400	26.79200
H	-6.94600	23.90700	26.38900
O	-3.36300	2.57100	23.07300
H	-4.13000	2.01700	23.21900
H	-2.95200	2.64300	23.93500
O	-11.35900	8.86000	37.16400
H	-10.49600	8.50300	37.37400
H	-11.93300	8.52100	37.85100
O	8.48600	30.49000	27.97000
H	9.12400	30.37100	27.26600
H	8.36800	31.43800	28.02900
O	5.29500	30.44300	27.90600
H	4.95700	30.32700	27.01800
H	6.16400	30.04200	27.88600
O	1.15300	32.29600	23.89600
H	0.59000	32.20500	23.12700
H	1.03900	31.47600	24.37600
O	-1.30000	33.79100	26.73400
H	-0.50800	33.45000	26.31900
H	-1.06000	33.90100	27.65400
O	6.73300	35.96300	19.20700
H	6.08200	36.54900	18.82100
H	7.52800	36.49400	19.26700
O	0.65900	33.05200	19.60600
H	1.43200	32.50500	19.46700
H	1.01200	33.91700	19.81700
O	3.42300	31.75700	29.40300
H	4.01900	31.53300	28.68900
H	3.48100	31.01600	30.00500
O	2.99600	29.21100	17.23800
H	3.24800	28.69000	16.47600
H	2.05800	29.04800	17.34100
O	8.04000	28.21500	25.04500
H	8.82600	27.79700	24.69400
H	8.30400	29.12000	25.21300
O	8.21100	33.65200	19.74400
H	7.57600	33.72600	20.45600
H	7.88100	34.24200	19.06600
O	13.44100	28.15300	25.94900
H	14.17300	27.58200	26.18200
H	12.68500	27.76500	26.38900
O	2.71500	31.11400	19.44000
H	2.59900	30.43900	20.10800
H	2.82500	30.62700	18.62300
O	5.05800	30.57500	23.14500
H	4.69400	30.04800	23.85600
H	5.91500	30.18500	22.97200
O	10.96300	21.86500	30.40800
H	10.63200	22.73400	30.18400
H	10.36800	21.54800	31.08700
O	4.69900	25.60400	18.14100
H	4.86600	26.47200	18.50800
H	4.50900	25.76700	17.21700

O	0.90800	30.51700	25.84100
H	-0.01700	30.38500	26.04900
H	1.15600	31.29700	26.33800
O	10.85600	26.58400	27.48700
H	9.94800	26.51600	27.19300
H	10.80500	27.06100	28.31600
O	4.67800	28.06000	19.12600
H	4.55800	28.50600	19.96400
H	3.98300	28.40900	18.56700
O	-1.73100	30.16100	26.67600
H	-1.60200	30.41400	27.59000
H	-2.41200	30.75300	26.35600
O	2.43900	40.83000	22.77500
H	2.36100	41.78100	22.85400
H	1.83500	40.48500	23.43200
O	12.72200	24.78600	29.26500
H	12.08000	25.07900	28.61900
H	12.21800	24.67100	30.07100
O	10.37600	28.53200	19.95400
H	11.28800	28.76500	19.77900
H	9.86700	29.11600	19.39100
O	5.01400	27.89200	30.96300
H	5.86000	28.31600	31.10700
H	4.95300	27.23500	31.65700
O	8.98300	33.51500	27.34500
H	8.35400	33.67900	26.64300
H	8.59600	33.92900	28.11600
O	12.86200	34.45000	24.70000
H	12.17700	34.83000	24.14900
H	12.94700	35.06000	25.43300
O	8.16300	25.55200	32.62100
H	7.92000	26.30700	32.08500
H	8.43900	25.93100	33.45600
O	7.72000	24.70800	28.20300
H	6.85800	25.00600	28.49400
H	7.74600	24.92500	27.27100
O	4.53600	33.25200	24.61000
H	5.00000	33.53100	23.82100
H	4.27200	32.35100	24.42700
O	1.42900	32.79900	27.54800
H	1.99900	33.22800	26.91000
H	2.01900	32.51800	28.24700
O	3.47300	29.37400	21.41900
H	4.17500	29.82700	21.88600
H	3.14100	28.73300	22.04700
O	-2.78400	27.63300	26.72100
H	-3.57500	27.88900	26.24500
H	-2.29300	28.44800	26.82600
O	-0.83300	27.99700	23.67900
H	-1.63000	28.10900	24.19800
H	-1.06000	28.33600	22.81300
O	-4.50100	27.88600	29.15200
H	-4.25000	28.77100	29.41800
H	-3.96500	27.70600	28.38000
O	4.71200	36.18900	21.49500
H	4.57700	37.03400	21.92400
H	5.25300	36.39100	20.73200
O	1.19900	22.26800	38.15700
H	0.80600	21.71400	38.83200
H	2.01600	22.57900	38.54700
O	-1.83500	33.70600	22.06800
H	-1.32200	34.50000	22.21900
H	-1.18200	33.00900	22.00100
O	-0.02500	36.82300	24.15000
H	0.15100	37.74500	23.96200
H	0.77000	36.50900	24.58000
O	4.11600	23.68400	35.74900
H	4.85700	24.28400	35.66900
H	4.34800	22.94100	35.19100
O	-0.74000	28.86900	34.02700
H	-0.85500	27.96800	34.32700
H	-1.59600	29.11600	33.67500
O	8.06200	19.71200	30.02500
H	7.71800	20.60200	29.94900
H	8.43000	19.52200	29.16200
O	-3.87000	32.03500	22.84400
H	-3.03200	32.44500	22.62700
H	-3.69200	31.09500	22.81400
O	8.02700	29.70300	18.53200
H	7.51100	30.47300	18.77100
H	7.75400	29.02900	19.15600
O	5.61100	24.54700	23.59100
H	4.75600	24.15800	23.41200
H	6.02400	24.62700	22.73100
O	7.14100	22.08200	27.99000
H	6.63800	22.04500	27.17700
H	7.43400	22.99100	28.05000
O	3.38600	22.16600	26.99700
H	4.25100	21.97000	26.63700
H	3.45300	21.93700	27.92400
O	13.00000	32.45600	20.46800
H	12.22300	32.11700	20.02300
H	13.67900	32.46100	19.79300
O	7.81500	27.57500	20.35800
H	7.51000	27.89000	21.20900
H	8.76800	27.65300	20.40100
O	6.26000	33.73400	22.00800
H	5.73200	33.00000	21.69500
H	5.75900	34.51200	21.76200
O	11.23900	31.92900	27.12400
H	10.58200	32.61900	27.21200
H	12.04300	32.31900	27.46800
O	0.99200	24.39600	24.36800
H	1.56600	24.17400	23.63600
H	1.57900	24.46500	25.12100
O	10.05000	30.28400	25.17500
H	10.56300	30.91700	25.67700
H	10.70000	29.68400	24.80900
O	1.31900	26.42700	18.38800
H	0.48200	26.76400	18.70900
H	1.17900	26.29200	17.45100
O	3.29400	21.27000	23.09600
H	2.64100	20.95500	22.47200
H	3.25200	20.64800	23.82200
O	4.40500	31.55200	15.01100
H	3.49800	31.32100	15.21000
H	4.90200	30.75600	15.19900
O	7.31200	40.26200	17.93100

H	6.63800	40.56500	18.53900
H	7.69900	41.06500	17.58100
O	6.52800	27.75900	27.42100
H	5.61000	27.98000	27.26300
H	6.94400	27.83300	26.56200
O	7.31100	29.15800	22.55100
H	8.05900	29.67000	22.24200
H	7.58000	28.83600	23.41100
O	5.58400	31.65400	19.53400
H	4.67500	31.36900	19.63100
H	5.61400	32.07300	18.67400
O	10.81600	35.26400	23.30300
H	10.36200	34.42300	23.35900
H	10.18500	35.90000	23.64000
O	9.75900	26.19300	24.53300
H	9.02100	25.68100	24.86400
H	10.01200	25.75300	23.72100
O	9.25900	37.10200	24.23800
H	9.55200	37.66200	23.51900
H	9.76900	37.39500	24.99300
O	9.86000	18.97100	25.12700
H	10.16600	18.11100	24.84000
H	10.58600	19.56300	24.92700
O	-3.84700	29.37400	23.51500
H	-4.04500	28.78100	22.79000
H	-4.40300	29.06900	24.23300
O	4.13200	29.26600	25.63500
H	4.18700	28.31400	25.55900
H	3.32200	29.42200	26.11900
O	5.28200	33.19400	16.87200
H	4.84400	32.47800	16.41300
H	6.02300	33.42200	16.31100
O	2.07200	27.88400	23.39400
H	1.25900	28.18400	23.80000
H	2.56800	27.49000	24.11100
O	4.22300	37.88000	25.32700
H	4.18000	38.10600	24.39800
H	5.13900	37.64600	25.47600
O	13.39900	31.47000	22.93000
H	13.26100	31.66000	22.00200
H	13.04300	32.23200	23.38700
O	1.56300	25.45600	29.01000
H	2.20300	25.31500	28.31200
H	0.73100	25.16900	28.63400
O	2.33900	34.75600	25.12300
H	3.13300	34.26100	24.92200
H	1.62800	34.22800	24.76000
O	-1.35300	28.90000	21.19900
H	-2.28300	28.83900	21.41600
H	-1.25700	28.37800	20.40300
O	-1.43400	25.42600	23.78700
H	-1.17300	26.34000	23.67800
H	-0.61200	24.95800	23.93700
O	0.03800	26.84000	30.95700
H	0.71100	26.63100	30.30800
H	-0.78900	26.64200	30.51700
O	5.94100	23.46600	32.97400
H	5.29400	24.16500	32.88000
H	6.71100	23.90000	33.34100
O	6.11700	31.30400	31.03300
H	7.04700	31.45500	30.86000
H	5.69700	31.39200	30.17800
O	4.91600	35.32900	28.80300
H	4.77700	34.96100	29.67600
H	4.12800	35.84400	28.63100
O	5.51100	40.43000	20.14600
H	5.68600	39.95900	20.96000
H	4.82400	41.05700	20.37400
O	6.46700	34.70700	26.19200
H	6.13200	34.66700	27.08800
H	5.96700	34.04200	25.71900
O	0.44100	30.11400	31.71700
H	0.30300	29.38200	32.31800
H	1.35200	30.02500	31.43600
O	9.40300	32.05300	16.46600
H	8.78600	31.32800	16.36900
H	8.93400	32.81400	16.12200
O	3.06900	29.82600	31.20600
H	3.77900	29.18500	31.16400
H	3.02600	30.07700	32.12800
O	1.78200	26.81300	33.02500
H	1.24600	26.36200	33.67700
H	1.19400	26.94700	32.28200
O	1.19300	26.27100	15.58800
H	0.31600	26.15000	15.22400
H	1.66800	25.48000	15.33600
O	-7.28700	31.05900	25.68200
H	-6.60000	30.74500	25.09400
H	-7.44200	31.96300	25.40900
O	-2.48400	25.94800	30.11000
H	-2.56800	25.34100	29.37500
H	-3.30500	26.43900	30.11100
O	4.37900	38.62400	22.83400
H	3.59600	39.16600	22.92800
H	5.07800	39.14100	23.23300
O	7.68900	24.43300	25.47700
H	8.18600	23.68000	25.15800
H	6.91700	24.47000	24.91200
O	15.54200	30.13800	27.11900
H	15.53100	31.02000	26.74900
H	14.67300	29.78500	26.92500
O	8.77200	33.19100	23.05100
H	7.83700	33.31100	22.88200
H	8.94900	32.28800	22.78700
O	4.27200	26.57500	25.20500
H	3.90200	26.02200	25.89300
H	4.76800	25.97100	24.65200
O	12.15900	29.03200	23.58600
H	12.62500	29.86500	23.51200
H	12.61000	28.56700	24.29100
O	10.22700	35.37700	20.58500
H	10.50200	35.26200	21.49400
H	9.35700	34.97900	20.54600
O	10.13100	21.40000	27.62500
H	9.21700	21.63100	27.79400
H	10.56000	21.48400	28.47700
O	9.90100	29.58900	30.12500
H	9.24800	29.83700	29.47100

H	10.27900	28.77600	29.79200
O	6.86200	39.62500	24.22800
H	7.04400	38.80800	24.69100
H	7.68500	39.84200	23.79100
O	7.19600	38.55200	21.49000
H	7.72500	38.04100	20.87700
H	7.45900	38.23600	22.35400
O	-2.30200	36.56900	29.23300
H	-1.71700	35.82300	29.36600
H	-2.50000	36.87900	30.11700
O	0.55200	39.60400	24.24900
H	-0.11700	40.08800	23.76600
H	0.41300	39.85100	25.16400
O	-7.71000	29.13900	27.95400
H	-7.67200	28.60600	28.74700
H	-8.21600	28.61300	27.33500
O	-0.33100	23.98600	31.45000
H	-0.26700	24.84700	31.03800
H	-0.39500	23.37300	30.71700
O	-0.85600	26.05800	35.09700
H	-0.22900	25.67600	35.71100
H	-1.71300	25.83900	35.46300
O	-4.08000	27.86600	21.02900
H	-3.78400	26.95700	20.97600
H	-4.98100	27.84400	20.70700
O	-0.55000	25.94100	27.01200
H	-1.27100	26.55500	26.87300
H	0.23900	26.47700	26.93300
O	2.25300	23.27700	33.28600
H	2.77600	23.52300	34.04900
H	2.03400	24.10900	32.86600
O	-2.35100	25.54700	21.15300
H	-2.67900	24.66700	20.97000
H	-1.99700	25.49200	22.04100
O	6.89700	37.18200	25.66200
H	6.75000	36.25400	25.84300
H	7.72300	37.20400	25.17800
O	0.76300	25.83400	21.52100
H	1.10300	26.36300	20.79800
H	1.21100	26.17200	22.29600
O	3.45800	24.21800	30.97100
H	3.11700	23.41900	31.37300
H	2.75200	24.51800	30.39900
O	4.10900	21.30300	29.42400
H	4.93200	20.82900	29.30100
H	4.35300	22.08200	29.92300
O	2.75000	24.33200	19.72000
H	3.47100	24.71000	19.21600
H	2.12700	25.05100	19.82300
O	6.89300	25.03600	20.65600
H	7.11000	25.91900	20.35500
H	6.41300	24.64500	19.92600
O	1.63300	27.99500	27.23300
H	1.48800	28.30900	28.12500
H	1.15600	28.61300	26.68000
O	3.49300	24.98500	27.28100
H	3.47900	24.08900	26.94400
H	4.21000	24.99200	27.91500
O	5.34600	25.41900	29.32600
H	5.08700	26.34100	29.32500
H	4.81400	25.02200	30.01600
O	10.29000	38.52400	22.10500
H	10.46400	37.95900	21.35300
H	10.78300	39.32400	21.92300
O	-3.89200	30.53500	30.17200
H	-4.43700	31.31100	30.30400
H	-3.01400	30.88200	30.01300
O	2.89700	23.86700	22.55000
H	3.01800	22.93800	22.74900
H	2.60700	23.88000	21.63700
O	5.76600	21.55200	25.68600
H	5.78800	21.61600	24.73100
H	6.18300	20.71200	25.88000
O	16.14400	31.28800	23.72400
H	16.58700	30.53800	23.32600
H	15.26700	31.27900	23.34200
O	11.27800	37.84100	19.20500
H	11.97300	38.46100	19.42400
H	11.62000	36.99000	19.48000
O	-3.10600	29.66200	33.06500
H	-3.84700	29.81100	33.65100
H	-3.42600	29.92300	32.20100
O	13.17700	35.29100	20.73400
H	13.70200	35.62900	20.00900
H	13.08900	34.35500	20.55000
O	4.86100	41.35800	25.09300
H	4.42100	40.86500	25.78600
H	5.61200	40.81500	24.85200
O	-2.66000	37.54900	25.09600
H	-1.83400	37.24900	24.71500
H	-3.01000	36.78000	25.54500
O	2.30800	36.90200	27.16500
H	1.68600	36.23900	26.86600
H	2.97900	36.92800	26.48300
O	-4.45900	35.46800	26.05400
H	-5.29600	35.04000	25.87300
H	-4.48700	35.66100	26.99200
O	2.38100	25.38000	36.75900
H	2.43000	26.25600	36.37600
H	2.92300	24.83700	36.18600
O	1.87000	35.45200	20.64100
H	2.01800	36.13500	19.98800
H	2.67600	35.43200	21.15600
O	4.61100	26.13300	33.51300
H	3.74100	26.42600	33.24200
H	4.84700	26.71700	34.23400
O	15.68700	32.75800	26.11600
H	16.52200	33.19700	26.28000
H	15.82300	32.28200	25.29700
O	-0.37500	31.47800	21.64700
H	-0.62100	30.57500	21.84500
H	-0.20600	31.48000	20.70500
O	7.31500	22.35100	31.03000
H	7.83300	23.08700	30.70400
H	6.96000	22.65700	31.86400
O	-1.10600	27.21100	19.14600
H	-1.79900	27.28600	18.49000
H	-1.35000	26.44700	19.66800

O	7.56600	18.77100	26.55000
H	7.84600	18.10300	27.17600
H	8.35100	18.96400	26.03700
O	5.36900	37.94700	18.11200
H	4.79700	38.56600	17.65900
H	5.94200	38.49700	18.64600
O	11.43700	27.19300	30.29700
H	12.38800	27.29200	30.31800
H	11.26700	26.40000	30.80700
O	9.45800	24.27800	30.48300
H	8.86300	24.55900	29.78800
H	9.17300	24.76800	31.25500
O	8.58900	38.23000	19.24300
H	9.52200	38.16500	19.44700
H	8.50100	39.06600	18.78500
O	11.21800	37.54700	16.20400
H	11.13900	37.21300	17.09700
H	12.03900	38.03900	16.20300
O	13.91500	30.41300	29.41100
H	13.88300	29.47500	29.59700
H	14.52600	30.49200	28.67800
O	5.75400	29.28300	15.45400
H	6.70000	29.15300	15.52300
H	5.37500	28.54400	15.93100
O	14.06500	27.20400	29.45700
H	13.54900	26.42000	29.27000
H	14.94900	26.87600	29.62200
O	11.72600	20.94800	25.41700
H	11.52800	20.64000	26.30100
H	12.33600	21.67500	25.54600
O	3.93800	27.20800	15.38900
H	3.03100	26.91100	15.46600
H	4.21600	26.91100	14.52300
O	-5.14400	28.52800	25.83900
H	-5.46400	27.67500	25.54700
H	-5.69100	28.74300	26.59500
O	-1.36600	31.24500	29.55600
H	-1.14700	32.17600	29.57500
H	-0.95400	30.88500	30.34200
O	5.55400	21.75200	21.54400
H	4.74400	21.40600	21.91900
H	6.24300	21.44200	22.13300
O	7.48300	20.82900	23.16600
H	8.03600	21.24900	23.82500
H	7.72000	19.90300	23.20900
O	2.70800	24.06700	15.53100
H	3.57000	24.39200	15.79000
H	2.89300	23.28000	15.01800
O	-5.27500	32.14900	27.80900
H	-5.96900	31.73400	27.29600
H	-4.52800	32.19100	27.21200
O	10.86100	22.93900	22.67400
H	10.28100	23.59800	22.29200
H	10.59500	22.11700	22.26300
O	9.31200	22.34000	24.87600
H	9.81200	22.45200	24.06800
H	9.95500	22.03600	25.51600
O	-3.36200	32.08100	25.68500
H	-3.38500	32.17900	24.73300
H	-3.05200	32.92700	26.00700
O	-0.70300	34.13800	29.80000
H	0.25100	34.10700	29.73300
H	-0.90200	33.67700	30.61500
O	-0.11300	32.70800	32.36700
H	0.70600	33.20300	32.39100
H	0.16100	31.79100	32.33900
O	9.64400	25.09900	21.73000
H	8.75600	25.38400	21.51200
H	10.05900	24.93900	20.88300
O	12.99500	25.12400	25.44500
H	13.77100	25.61400	25.71800
H	12.27000	25.55000	25.90200
O	15.43200	25.83700	26.50300
H	16.02700	25.59600	25.79200
H	15.50500	25.11700	27.13000
O	-2.34800	39.68000	26.81700
H	-2.48200	38.92000	26.25100
H	-2.76500	40.40400	26.35000
O	8.78800	34.86400	15.82500
H	9.49200	35.06900	16.44000
H	8.06400	35.43200	16.09000
O	9.61800	30.61500	21.94500
H	9.56100	31.07200	21.10600
H	10.17100	29.85400	21.76700
O	13.42800	26.40400	22.77000
H	13.17200	26.00300	23.60100
H	12.80400	27.11800	22.64500
O	17.74000	29.14300	25.27700
H	17.18000	29.21300	26.05000
H	17.13400	28.96800	24.55700
O	0.80900	40.57700	27.02900
H	0.69200	41.52200	26.92700
H	-0.01200	40.27400	27.41700
O	10.69700	35.30200	17.87400
H	11.53100	34.87000	17.69200
H	10.53500	35.13000	18.80200
O	10.35100	31.80500	19.24000
H	10.29600	31.64000	18.29900
H	9.72600	32.51300	19.39600
O	-4.85900	27.04200	33.06600
H	-4.21400	26.53200	33.55500
H	-5.06800	27.77900	33.64100
O	3.66100	21.30600	18.81900
H	3.64900	22.25000	18.97500
H	4.55300	21.12300	18.52500
O	3.59000	39.98300	26.90500
H	2.63900	39.98300	26.79200
H	3.89300	39.23600	26.38900
O	7.47600	17.90000	23.07300
H	6.70900	17.34600	23.21900
H	7.88700	17.97300	23.93500
O	-0.52000	24.18900	37.16400
H	0.34300	23.83300	37.37400
H	-1.09300	23.85000	37.85100
O	13.83500	44.54100	17.23800
H	14.08800	44.01900	16.47600
H	12.89700	44.37700	17.34100
O	13.55500	46.44300	19.44000

H	13.43800	45.76800	20.10800
H	13.66500	45.95700	18.62300
O	15.53800	40.93300	18.14100
H	15.70600	41.80100	18.50800
H	15.34800	41.09600	17.21700
O	15.51700	43.38900	19.12600
H	15.39700	43.83500	19.96400
H	14.82200	43.73800	18.56700
O	12.15800	41.75600	18.38800
H	11.32200	42.09300	18.70900
H	12.01900	41.62100	17.45100
O	14.13300	36.60000	23.09600
H	13.48000	36.28400	22.47200
H	14.09100	35.97700	23.82200
O	15.24500	46.88100	15.01100
H	14.33700	46.65000	15.21000
H	15.74200	46.08500	15.19900
O	6.99200	44.70300	23.51500
H	6.79500	44.11000	22.79000
H	6.43700	44.39800	24.23300
O	16.12100	48.52400	16.87200
H	15.68300	47.80700	16.41300
H	16.86200	48.75100	16.31100
O	9.48700	44.23000	21.19900
H	8.55700	44.16800	21.41600
H	9.58300	43.70700	20.40300
O	9.40500	40.75500	23.78700
H	9.66600	41.67000	23.67800
H	10.22700	40.28800	23.93700
O	12.03200	41.60100	15.58800
H	11.15500	41.47900	15.22400
H	12.50800	40.80900	15.33600
O	6.76000	43.19500	21.02900
H	7.05500	42.28600	20.97600
H	5.85900	43.17400	20.70700
O	8.48900	40.87600	21.15300
H	8.16000	39.99600	20.97000
H	8.84300	40.82100	22.04100
O	11.60300	41.16300	21.52100
H	11.94200	41.69200	20.79800
H	12.05000	41.50200	22.29600
O	13.59000	39.66100	19.72000
H	14.31000	40.03900	19.21600
H	12.96600	40.38000	19.82300
O	17.73300	40.36500	20.65600
H	17.94900	41.24800	20.35500
H	17.25200	39.97400	19.92600
O	13.73600	39.19600	22.55000
H	13.85800	38.26700	22.74900
H	13.44600	39.21000	21.63700
O	9.73400	42.54000	19.14600
H	9.04100	42.61500	18.49000
H	9.48900	41.77700	19.66800
O	16.59300	44.61200	15.45400
H	17.53900	44.48300	15.52300
H	16.21500	43.87300	15.93100
O	14.77700	42.53700	15.38900
H	13.87100	42.24000	15.46600
H	15.05500	42.24000	14.52300
O	16.39400	37.08100	21.54400
H	15.58400	36.73500	21.91900
H	17.08200	36.77100	22.13300
O	18.32300	36.15900	23.16600
H	18.87600	36.57800	23.82500
H	18.56000	35.23200	23.20900
O	13.54700	39.39600	15.53100
H	14.40900	39.72100	15.79000
H	13.73200	38.60900	15.01800
O	14.50000	36.63500	18.81900
H	14.48800	37.57900	18.97500
H	15.39300	36.45200	18.52500
O	18.31600	33.23000	23.07300
H	17.54900	32.67500	23.21900
H	18.72700	33.30200	23.93500
O	-24.03200	-38.49100	14.69500
H	-23.39400	-38.61100	13.99100
H	-24.15000	-37.54300	14.75300
O	-27.22300	-38.53900	14.63100
H	-27.56200	-38.65500	13.74300
H	-26.35400	-38.94000	14.61100
O	-31.36500	-36.68500	10.62000
H	-31.92800	-36.77600	9.85200
H	-31.47900	-37.50500	11.10100
O	-33.81800	-35.19000	13.45900
H	-33.02600	-35.53100	13.04300
H	-33.57900	-35.08100	14.37900
O	-25.78500	-33.01800	5.93200
H	-26.43600	-32.43300	5.54500
H	-24.99000	-32.48700	5.99100
O	-31.85900	-35.92900	6.33100
H	-31.08600	-36.47600	6.19200
H	-31.50700	-35.06500	6.54200
O	-30.02100	-34.60200	19.53700
H	-30.31200	-33.69700	19.42200
H	-29.38500	-34.56000	20.25200
O	-29.09500	-37.22400	16.12700
H	-28.49900	-37.44800	15.41300
H	-29.03800	-37.96500	16.73000
O	-24.47800	-40.76600	11.77000
H	-23.69200	-41.18400	11.41900
H	-24.21400	-39.86200	11.93800
O	-24.30700	-35.32900	6.46900
H	-24.94300	-35.25600	7.18100
H	-24.63700	-34.74000	5.79100
O	-19.07700	-40.82900	12.67300
H	-18.34500	-41.39900	12.90700
H	-19.83300	-41.21700	13.11400
O	-29.80300	-37.86700	6.16500
H	-29.91900	-38.54300	6.83300
H	-29.69300	-38.35400	5.34800
O	-27.46000	-38.40600	9.86900
H	-27.82400	-38.93300	10.58100
H	-26.60300	-38.79600	9.69600
O	-25.06200	-34.91500	16.53200
H	-25.36200	-34.01300	16.42400
H	-25.40700	-35.18200	17.38400
O	-21.66200	-42.39700	14.21200
H	-22.57000	-42.46600	13.91800

H	-21.71300	-41.92000	15.04000
O	-30.08000	-28.15100	9.49900
H	-30.15700	-27.20000	9.57800
H	-30.68300	-28.49600	10.15700
O	-19.79600	-44.19600	15.99000
H	-20.43800	-43.90200	15.34400
H	-20.30000	-44.31000	16.79500
O	-22.14200	-40.44900	6.67800
H	-21.23000	-40.21600	6.50300
H	-22.65100	-39.86500	6.11600
O	-23.85800	-36.02700	20.31400
H	-23.44900	-36.46800	19.57000
H	-24.58000	-36.60100	20.56800
O	-23.53500	-35.46700	14.07000
H	-24.16400	-35.30200	13.36700
H	-23.92200	-35.05200	14.84100
O	-19.65600	-34.53100	11.42400
H	-20.34100	-34.15100	10.87400
H	-19.57200	-33.92200	12.15700
O	-22.40100	-36.88500	18.02400
H	-22.18900	-36.24200	17.34700
H	-22.44800	-37.71900	17.55600
O	-27.98200	-35.72900	11.33500
H	-27.51800	-35.45000	10.54500
H	-28.24600	-36.63100	11.15200
O	-31.09000	-36.18200	14.27300
H	-30.51900	-35.75300	13.63400
H	-30.49900	-36.46400	14.97100
O	-21.28600	-34.00800	16.19600
H	-20.46400	-34.41000	15.91400
H	-21.29000	-34.11100	17.14700
O	-27.52100	-34.46000	18.35200
H	-27.45600	-33.99800	19.18800
H	-28.22900	-35.09000	18.48000
O	-13.19600	-37.66400	12.54400
H	-13.94900	-38.23300	12.38900
H	-13.06100	-37.69800	13.49100
O	-27.80600	-32.79300	8.22000
H	-27.94100	-31.94800	8.64800
H	-27.26500	-32.59100	7.45600
O	-34.35300	-35.27500	8.79300
H	-33.84000	-34.48100	8.94300
H	-33.70100	-35.97300	8.72600
O	-32.54300	-32.15900	10.87500
H	-32.36700	-31.23700	10.68700
H	-31.74800	-32.47300	11.30400
O	-26.17900	-32.17000	22.35000
H	-26.62300	-32.95700	22.03200
H	-25.51700	-31.98200	21.68400
O	-31.42000	-30.64100	16.87600
H	-30.86900	-30.76900	16.10400
H	-32.25900	-30.34100	16.52500
O	-24.08100	-31.89000	20.37800
H	-24.09900	-31.06900	19.88600
H	-23.15900	-32.01500	20.60300
O	-24.49100	-39.27900	5.25700
H	-25.00700	-38.50800	5.49600
H	-24.76400	-39.95200	5.88000
O	-19.51800	-36.52600	7.19200
H	-20.29500	-36.86500	6.74800
H	-18.83900	-36.52000	6.51800
O	-24.70300	-41.40600	7.08300
H	-25.00800	-41.09200	7.93400
H	-23.75000	-41.32800	7.12600
O	-26.25800	-35.24700	8.73200
H	-26.78600	-35.98200	8.42000
H	-26.75900	-34.47000	8.48600
O	-21.27900	-37.05200	13.84900
H	-21.93700	-36.36200	13.93700
H	-20.47500	-36.66300	14.19200
O	-22.46800	-38.69800	11.89900
H	-21.95500	-38.06500	12.40200
H	-21.81800	-39.29800	11.53400
O	-28.11300	-37.42900	1.73600
H	-29.02000	-37.66000	1.93500
H	-27.61600	-38.22500	1.92300
O	-25.20600	-28.71900	4.65500
H	-25.88000	-28.41600	5.26400
H	-24.82000	-27.91600	4.30600
O	-25.99000	-41.22300	14.14500
H	-26.90800	-41.00100	13.98700
H	-25.57400	-41.14800	13.28600
O	-25.20700	-39.82400	9.27500
H	-24.46000	-39.31200	8.96600
H	-24.93800	-40.14500	10.13600
O	-26.93400	-37.32800	6.25800
H	-27.84300	-37.61200	6.35500
H	-26.90500	-36.90800	5.39800
O	-21.70200	-33.71700	10.02700
H	-22.15600	-34.55800	10.08400
H	-22.33300	-33.08100	10.36500
O	-22.75900	-42.78800	11.25700
H	-23.49700	-43.30000	11.58800
H	-22.50600	-43.22800	10.44600
O	-23.25900	-31.88000	10.96200
H	-22.96700	-31.31900	10.24300
H	-22.74900	-31.58700	11.71800
O	-28.38600	-39.71500	12.35900
H	-28.33100	-40.66800	12.28300
H	-29.19700	-39.55900	12.84300
O	-27.23700	-35.78700	3.59700
H	-27.67500	-36.50300	3.13800
H	-26.49500	-35.55900	3.03500
O	-28.29600	-31.10100	12.05200
H	-28.33800	-30.87500	11.12200
H	-27.37900	-31.33500	12.20000
O	-19.11900	-37.51100	9.65500
H	-19.25700	-37.32100	8.72700
H	-19.47600	-36.74900	10.11100
O	-19.09200	-35.44400	14.66600
H	-18.65900	-35.53600	15.51400
H	-18.37900	-35.46800	14.02800
O	-30.17900	-34.22500	11.84800
H	-29.38500	-34.72000	11.64600
H	-30.89000	-34.75300	11.48500
O	-25.35900	-31.84300	16.19900
H	-25.30600	-31.27400	16.96700
H	-26.22800	-31.67300	15.83400

O	-18.23100	-35.40100	17.19900
H	-18.03700	-34.49700	17.44500
H	-18.33100	-35.86000	18.03300
O	-23.16900	-35.25100	22.77300
H	-23.32100	-35.47900	21.85600
H	-23.17900	-34.29300	22.78600
O	-26.40100	-37.67700	17.75800
H	-25.47100	-37.52600	17.58500
H	-26.82200	-37.58900	16.90300
O	-27.60200	-33.65200	15.52800
H	-27.74100	-34.02000	16.40000
H	-28.39000	-33.13800	15.35600
O	-27.00700	-28.55100	6.87000
H	-26.83200	-29.02300	7.68500
H	-27.69400	-27.92500	7.09800
O	-26.05100	-34.27400	12.91600
H	-26.38600	-34.31400	13.81200
H	-26.55100	-34.94000	12.44300
O	-23.11500	-36.92900	3.19000
H	-23.73200	-37.65400	3.09300
H	-23.58400	-36.16800	2.84700
O	-21.46000	-32.21900	20.85100
H	-21.51900	-32.90300	20.18400
H	-21.05800	-31.47700	20.39900
O	-32.44100	-25.40200	13.64400
H	-32.20100	-24.77100	14.32200
H	-33.28400	-25.75400	13.93100
O	-21.45800	-34.25500	19.08200
H	-22.34600	-34.59200	19.20500
H	-20.88800	-34.98600	19.31900
O	-27.92800	-29.59500	16.04800
H	-28.00100	-28.78900	16.56000
H	-28.37000	-29.39500	15.22300
O	-28.13900	-30.35700	9.55900
H	-28.92200	-29.81500	9.65200
H	-27.44000	-29.84000	9.95800
O	-16.97700	-38.84400	13.84400
H	-16.98800	-37.96100	13.47300
H	-17.84500	-39.19700	13.64900
O	-25.46800	-29.69600	18.20400
H	-24.75500	-29.06100	18.13200
H	-25.95900	-29.59900	17.38800
O	-23.74700	-35.79100	9.77600
H	-24.68100	-35.67100	9.60600
H	-23.56900	-36.69300	9.51200
O	-20.35900	-39.94900	10.31100
H	-19.89300	-39.11600	10.23700
H	-19.90800	-40.41400	11.01500
O	-30.41700	-31.94600	19.02500
H	-29.71600	-31.31300	19.17800
H	-30.88800	-31.60500	18.26400
O	-22.29200	-33.60500	7.30900
H	-22.01600	-33.71900	8.21900
H	-23.16100	-34.00300	7.27100
O	-22.61700	-39.39200	16.85000
H	-23.27000	-39.14400	16.19500
H	-22.23900	-40.20600	16.51600
O	-25.65600	-29.35600	10.95300
H	-25.47400	-30.17400	11.41500
H	-24.83300	-29.13900	10.51500
O	-25.32200	-30.42900	8.21400
H	-24.79300	-30.94000	7.60100
H	-25.05900	-30.74500	9.07900
O	-34.82000	-32.41300	15.95800
H	-34.23500	-33.15800	16.09100
H	-35.01800	-32.10200	16.84100
O	-31.96600	-29.37800	10.97400
H	-32.63500	-28.89300	10.49000
H	-32.10500	-29.13100	11.88800
O	-27.82600	-27.34300	17.57200
H	-27.27400	-27.18000	18.33700
H	-27.48500	-26.75300	16.90000
O	-22.97500	-32.51700	23.42700
H	-22.43100	-31.90400	22.93300
H	-23.55600	-31.95900	23.94400
O	-24.68900	-29.39500	14.55900
H	-24.81400	-30.19600	14.05000
H	-24.03100	-28.90000	14.07000
O	-22.62600	-31.59400	16.18700
H	-23.57300	-31.71400	16.11400
H	-22.27700	-32.48200	16.26900
O	-25.62100	-31.79900	12.38700
H	-25.76800	-32.72700	12.56800
H	-24.79600	-31.77700	11.90300
O	-22.01800	-30.97600	13.47400
H	-21.09300	-31.18200	13.33600
H	-22.18600	-31.22400	14.38200
O	-22.22800	-30.45700	8.83000
H	-22.05400	-31.02300	8.07700
H	-21.73500	-29.65700	8.64800
O	-16.37400	-37.69400	10.44900
H	-15.93100	-38.44300	10.05100
H	-17.25100	-37.70300	10.06600
O	-21.24000	-31.14100	5.92900
H	-20.54500	-30.52000	6.14800
H	-20.89800	-31.99100	6.20500
O	-19.34100	-33.69100	7.45800
H	-18.81600	-33.35200	6.73300
H	-19.42900	-34.62600	7.27400
O	-27.67900	-29.77700	19.58700
H	-27.37100	-30.02300	20.45900
H	-26.88200	-29.57300	19.09900
O	-27.65700	-27.62400	11.81800
H	-28.09700	-28.11600	12.51100
H	-26.90600	-28.16600	11.57700
O	-35.17800	-31.43200	11.82000
H	-34.35200	-31.73200	11.44000
H	-35.52900	-32.20200	12.26900
O	-30.21000	-32.08000	13.89000
H	-30.83200	-32.74300	13.59000
H	-29.53900	-32.05400	13.20700
O	-36.97700	-33.51400	12.77900
H	-37.81400	-33.94200	12.59700
H	-37.00500	-33.32100	13.71600
O	-19.65200	-33.05800	22.76300
H	-20.44200	-33.07500	22.22400
H	-19.88900	-32.51900	23.51800
O	-19.53200	-36.20800	20.07700

H	-19.48200	-36.37300	21.01800
H	-19.64100	-37.07600	19.68600
O	-30.64900	-33.52900	7.36600
H	-30.50000	-32.84600	6.71200
H	-29.84200	-33.54900	7.88100
O	-18.03000	-33.78900	20.32100
H	-18.32200	-33.42600	21.15700
H	-18.64300	-34.50200	20.14200
O	-16.83100	-36.22300	12.84100
H	-15.99600	-35.78400	13.00500
H	-16.69500	-36.69900	12.02200
O	-19.78200	-38.66500	18.78900
H	-19.30600	-38.47300	17.98100
H	-20.70500	-38.60900	18.54300
O	-27.14900	-31.03500	4.83700
H	-27.72200	-30.41600	4.38400
H	-26.57600	-30.48500	5.37100
O	-21.08100	-41.78900	17.02200
H	-20.13000	-41.68900	17.04300
H	-21.25100	-42.58100	17.53100
O	-23.92900	-30.75100	5.96800
H	-22.99600	-30.81600	6.17100
H	-24.01700	-29.91500	5.50900
O	-21.30000	-31.43400	2.92800
H	-21.37900	-31.76800	3.82200
H	-20.47900	-30.94200	2.92700
O	-18.60300	-38.56800	16.13600
H	-18.63500	-39.50700	16.32200
H	-17.99300	-38.49000	15.40200
O	-26.76400	-39.69900	2.17900
H	-25.81800	-39.82800	2.24700
H	-27.14300	-40.43700	2.65500
O	-18.45300	-41.77700	16.18200
H	-18.96900	-42.56100	15.99500
H	-17.56900	-42.10500	16.34600
O	-12.78500	-37.50700	15.27600
H	-12.05500	-37.76800	15.83700
H	-13.39400	-38.24400	15.31700
O	-14.70300	-39.36600	15.87400
H	-15.43000	-39.50000	15.26500
H	-15.11900	-39.05800	16.68000
O	-33.22100	-34.84300	16.52400
H	-32.26700	-34.87500	16.45700
H	-33.42000	-35.30400	17.34000
O	-22.87400	-43.88200	8.45400
H	-23.76200	-43.59700	8.23600
H	-22.45900	-44.04300	7.60700
O	-19.52300	-43.85700	12.17000
H	-18.74700	-43.36800	12.44200
H	-20.24800	-43.43200	12.62700
O	-17.08600	-43.14500	13.22700
H	-16.49100	-43.38500	12.51700
H	-17.01300	-43.86400	13.85500
O	-34.86600	-29.30100	13.54200
H	-35.00100	-30.06100	12.97500
H	-35.28300	-28.57700	13.07400
O	-23.73000	-34.11800	2.54900
H	-23.02600	-33.91300	3.16500
H	-24.45400	-33.55000	2.81400
O	-22.90000	-38.36700	8.66900
H	-22.95700	-37.91000	7.83000
H	-22.34700	-39.12700	8.49200
O	-19.09000	-42.57800	9.49400
H	-19.34600	-42.97800	10.32500
H	-19.71500	-41.86300	9.36900
O	-14.77900	-39.83800	12.00200
H	-15.33900	-39.76900	12.77500
H	-15.38400	-40.01400	11.28100
O	-31.70900	-28.40400	13.75300
H	-31.82600	-27.46000	13.65100
H	-32.53000	-28.70700	14.14200
O	-21.82100	-33.67900	4.59900
H	-20.98700	-34.11100	4.41700
H	-21.98300	-33.85100	5.52700
O	-22.16700	-37.17600	5.96500
H	-22.22200	-37.34200	5.02300
H	-22.79200	-36.46800	6.12100
O	-30.68400	-27.75100	16.96900
H	-29.78100	-27.71900	17.28600
H	-30.91000	-28.68100	16.98400
O	-28.92800	-28.99800	13.63000
H	-29.87900	-28.99800	13.51600
H	-28.62500	-29.74500	13.11400
O	-13.19300	-23.16200	14.69500
H	-12.55500	-23.28200	13.99100
H	-13.31100	-22.21400	14.75300
O	-16.38400	-23.21000	14.63100
H	-16.72200	-23.32500	13.74300
H	-15.51500	-23.61000	14.61100
O	-20.52500	-21.35600	10.62000
H	-21.08900	-21.44700	9.85200
H	-20.64000	-22.17600	11.10100
O	-15.90200	-22.05600	20.58900
H	-15.91300	-22.45100	19.71700
H	-15.74000	-22.78900	21.18300
O	-22.97900	-19.86100	13.45900
H	-22.18700	-20.20200	13.04300
H	-22.73900	-19.75100	14.37900
O	-14.94500	-17.68900	5.93200
H	-15.59700	-17.10400	5.54500
H	-14.15100	-17.15800	5.99100
O	-21.02000	-20.60000	6.33100
H	-20.24700	-21.14700	6.19200
H	-20.66700	-19.73600	6.54200
O	-19.18100	-19.27200	19.53700
H	-19.47200	-18.36800	19.42200
H	-18.54600	-19.23100	20.25200
O	-18.25600	-21.89500	16.12700
H	-17.65900	-22.11900	15.41300
H	-18.19800	-22.63600	16.73000
O	-18.68300	-24.44100	3.96200
H	-18.43100	-24.96300	3.20100
H	-19.62100	-24.60400	4.06500
O	-13.63900	-25.43700	11.77000
H	-12.85300	-25.85500	11.41900
H	-13.37500	-24.53300	11.93800
O	-13.46800	-20.00000	6.46900
H	-14.10300	-19.92600	7.18100

H	-13.79800	-19.41100	5.79100
O	-8.23800	-25.50000	12.67300
H	-7.50500	-26.07000	12.90700
H	-8.99400	-25.88700	13.11400
O	-18.96400	-22.53800	6.16500
H	-19.08000	-23.21300	6.83300
H	-18.85300	-23.02500	5.34800
O	-16.62000	-23.07700	9.86900
H	-16.98500	-23.60400	10.58100
H	-15.76400	-23.46700	9.69600
O	-10.71500	-31.78700	17.13300
H	-11.04700	-30.91800	16.90900
H	-11.31100	-32.10400	17.81200
O	-16.98000	-28.04800	4.86600
H	-16.81200	-27.18000	5.23300
H	-17.17000	-27.88500	3.94200
O	-14.22200	-19.58600	16.53200
H	-14.52200	-18.68400	16.42400
H	-14.56700	-19.85300	17.38400
O	-20.77100	-23.13500	12.56600
H	-21.69600	-23.26700	12.77400
H	-20.52300	-22.35500	13.06300
O	-10.82300	-27.06800	14.21200
H	-11.73100	-27.13600	13.91800
H	-10.87400	-26.59100	15.04000
O	-17.00100	-25.59200	5.85000
H	-17.12100	-25.14600	6.68900
H	-17.69600	-25.24400	5.29200
O	-23.41000	-23.49200	13.40000
H	-23.28100	-23.23800	14.31400
H	-24.09000	-22.89900	13.08100
O	-19.24000	-12.82200	9.49900
H	-19.31800	-11.87100	9.57800
H	-19.84400	-13.16700	10.15700
O	-16.74800	-25.64900	22.15500
H	-17.52500	-25.09000	22.14600
H	-16.10900	-25.16300	22.67700
O	-8.95600	-28.86600	15.99000
H	-9.59900	-28.57300	15.34400
H	-9.46100	-28.98100	16.79500
O	-11.30200	-25.12000	6.67800
H	-10.39100	-24.88700	6.50300
H	-11.81100	-24.53600	6.11600
O	-18.49400	-22.84000	21.71700
H	-17.75200	-22.30200	21.44100
H	-19.20000	-22.21400	21.87400
O	-13.01800	-20.69800	20.31400
H	-12.61000	-21.13900	19.57000
H	-13.74100	-21.27200	20.56800
O	-16.66500	-25.76000	17.68800
H	-15.81900	-25.33600	17.83100
H	-16.72500	-26.41700	18.38200
O	-12.69500	-20.13700	14.07000
H	-13.32500	-19.97300	13.36700
H	-13.08300	-19.72300	14.84100
O	-8.81700	-19.20200	11.42400
H	-9.50100	-18.82200	10.87400
H	-8.73200	-18.59200	12.15700
O	-13.51500	-28.10000	19.34600
H	-13.75900	-27.34600	18.80900
H	-13.23900	-27.72100	20.18100
O	-11.56100	-21.55600	18.02400
H	-11.35000	-20.91300	17.34700
H	-11.60800	-22.39000	17.55600
O	-13.95900	-28.94400	14.92700
H	-14.82000	-28.64600	15.21900
H	-13.93300	-28.72700	13.99500
O	-17.14300	-20.40000	11.33500
H	-16.67900	-20.12100	10.54500
H	-17.40700	-21.30200	11.15200
O	-20.25000	-20.85300	14.27300
H	-19.68000	-20.42400	13.63400
H	-19.65900	-21.13500	14.97100
O	-18.20600	-24.27800	8.14400
H	-17.50300	-23.82600	8.61100
H	-18.53800	-24.91900	8.77200
O	-19.75800	-25.68600	22.51900
H	-20.38800	-25.00500	22.75200
H	-19.86400	-25.79800	21.57400
O	-24.46300	-26.01900	13.44500
H	-25.25300	-25.76400	12.96900
H	-23.97200	-25.20400	13.55100
O	-10.44700	-18.67900	16.19600
H	-9.62500	-19.08100	15.91400
H	-10.45100	-18.78200	17.14700
O	-22.51200	-25.65500	10.40400
H	-23.30900	-25.54300	10.92200
H	-22.73900	-25.31600	9.53800
O	-16.68100	-19.13000	18.35200
H	-16.61700	-18.66800	19.18800
H	-17.39000	-19.76100	18.48000
O	-26.17900	-25.76600	15.87700
H	-25.92900	-24.88200	16.14300
H	-25.64300	-25.94600	15.10400
O	-2.35600	-22.33500	12.54400
H	-3.11000	-22.90400	12.38900
H	-2.22100	-22.36900	13.49100
O	-16.96600	-17.46400	8.22000
H	-17.10200	-16.61800	8.64800
H	-16.42500	-17.26200	7.45600
O	-20.48000	-31.38400	24.88100
H	-20.87300	-31.93800	25.55600
H	-19.66300	-31.07300	25.27200
O	-23.51300	-19.94600	8.79300
H	-23.00100	-19.15200	8.94300
H	-22.86100	-20.64300	8.72600
O	-21.70400	-16.82900	10.87500
H	-21.52700	-15.90800	10.68700
H	-20.90800	-17.14400	11.30400
O	-17.56300	-29.96900	22.47300
H	-16.82200	-29.36800	22.39400
H	-17.33100	-30.71100	21.91500
O	-22.41900	-24.78300	20.75100
H	-22.53300	-25.68400	21.05200
H	-23.27400	-24.53700	20.40000
O	-15.34000	-16.84100	22.35000
H	-15.78300	-17.62800	22.03200
H	-14.67800	-16.65300	21.68400

O	-20.58100	-15.31200	16.87600
H	-20.03000	-15.44000	16.10400
H	-21.42000	-15.01200	16.52500
O	-13.24100	-16.56100	20.37800
H	-13.26000	-15.74000	19.88600
H	-12.31900	-16.68600	20.60300
O	-16.78400	-19.04700	21.22000
H	-16.25100	-19.80800	20.99000
H	-17.31300	-19.33900	21.96300
O	-13.61700	-33.94100	16.75000
H	-13.96100	-33.05000	16.67400
H	-13.24900	-34.13000	15.88700
O	-25.54900	-21.61700	9.56900
H	-24.71100	-21.20700	9.35200
H	-25.37100	-22.55700	9.53900
O	-13.65200	-23.95000	5.25700
H	-14.16700	-23.17900	5.49600
H	-13.92400	-24.62300	5.88000
O	-16.06700	-29.10500	10.31600
H	-16.92300	-29.49400	10.13600
H	-15.65500	-29.02500	9.45500
O	-14.53700	-31.57000	14.71500
H	-15.04100	-31.60700	13.90100
H	-14.24400	-30.66100	14.77400
O	-18.29300	-31.48600	13.72100
H	-17.42800	-31.68200	13.36200
H	-18.22500	-31.71500	14.64800
O	-8.67900	-21.19600	7.19200
H	-9.45600	-21.53500	6.74800
H	-8.00000	-21.19100	6.51800
O	-13.86300	-26.07700	7.08300
H	-14.16800	-25.76200	7.93400
H	-12.91000	-25.99900	7.12600
O	-15.41900	-19.91800	8.73200
H	-15.94700	-20.65300	8.42000
H	-15.92000	-19.14000	8.48600
O	-10.44000	-21.72300	13.84900
H	-11.09700	-21.03300	13.93700
H	-9.63600	-21.33300	14.19200
O	-20.68700	-29.25600	11.09300
H	-20.11300	-29.47800	10.36000
H	-20.10000	-29.18700	11.84600
O	-11.62900	-23.36900	11.89900
H	-11.11600	-22.73600	12.40200
H	-10.97900	-23.96800	11.53400
O	-20.36000	-27.22600	5.11300
H	-21.19600	-26.88800	5.43300
H	-20.50000	-27.36100	4.17500
O	-18.38500	-32.38200	9.82000
H	-19.03800	-32.69700	9.19600
H	-18.42700	-33.00400	10.54600
O	-17.27400	-22.10000	1.73600
H	-18.18100	-22.33100	1.93500
H	-16.77600	-22.89600	1.92300
O	-14.36700	-13.39000	4.65500
H	-15.04100	-13.08700	5.26400
H	-13.98000	-12.58700	4.30600
O	-15.15100	-25.89400	14.14500
H	-16.06900	-25.67200	13.98700
H	-14.73500	-25.81900	13.28600
O	-14.36700	-24.49500	9.27500
H	-13.62000	-23.98200	8.96600
H	-14.09800	-24.81600	10.13600
O	-16.09500	-21.99900	6.25800
H	-17.00400	-22.28300	6.35500
H	-16.06500	-21.57900	5.39800
O	-10.86300	-18.38800	10.02700
H	-11.31700	-19.22900	10.08400
H	-11.49300	-17.75200	10.36500
O	-11.92000	-27.45900	11.25700
H	-12.65700	-27.97100	11.58800
H	-11.66700	-27.89900	10.44600
O	-12.42000	-16.55000	10.96200
H	-12.12700	-15.99000	10.24300
H	-11.91000	-16.25700	11.71800
O	-11.81800	-34.68100	11.85200
H	-11.51200	-35.54100	11.56400
H	-11.09300	-34.08900	11.65100
O	-25.52600	-24.27800	10.24000
H	-25.72300	-24.87100	9.51500
H	-26.08100	-24.58300	10.95700
O	-17.54600	-24.38600	12.35900
H	-17.49100	-25.33800	12.28300
H	-18.35700	-24.23000	12.84300
O	-16.39700	-20.45800	3.59700
H	-16.83500	-21.17400	3.13800
H	-15.65600	-20.23000	3.03500
O	-19.60700	-25.76800	10.11800
H	-20.42000	-25.46900	10.52500
H	-19.11100	-26.16300	10.83600
O	-17.45600	-15.77200	12.05200
H	-17.49900	-15.54600	11.12200
H	-16.54000	-16.00600	12.20000
O	-14.01000	-25.30400	18.95000
H	-13.65500	-25.07700	19.80900
H	-13.36200	-24.97200	18.32700
O	-8.28000	-22.18200	9.65500
H	-8.41800	-21.99200	8.72700
H	-8.63600	-21.42000	10.11100
O	-20.11600	-28.19600	15.73400
H	-19.47600	-28.33700	15.03700
H	-20.94800	-28.48300	15.35800
O	-8.25300	-20.11400	14.66600
H	-7.81900	-20.20700	15.51400
H	-7.54000	-20.13900	14.02800
O	-19.34000	-18.89600	11.84800
H	-18.54600	-19.39100	11.64600
H	-20.05000	-19.42400	11.48500
O	-14.52000	-16.51400	16.19900
H	-14.46600	-15.94500	16.96700
H	-15.38900	-16.34400	15.83400
O	-23.03100	-24.75200	7.92400
H	-23.96200	-24.81300	8.14100
H	-22.93600	-25.27400	7.12700
O	-23.11300	-28.22600	10.51200
H	-22.85200	-27.31200	10.40300
H	-22.29100	-28.69400	10.66100
O	-21.64000	-26.81200	17.68100

H	-20.96800	-27.02100	17.03300
H	-22.46700	-27.01000	17.24200
O	-7.39100	-20.07200	17.19900
H	-7.19800	-19.16700	17.44500
H	-7.49100	-20.53100	18.03300
O	-15.73800	-30.18600	19.69800
H	-16.38500	-29.48700	19.60400
H	-14.96800	-29.75300	20.06600
O	-12.33000	-19.92100	22.77300
H	-12.48100	-20.15000	21.85600
H	-12.33900	-18.96400	22.78600
O	-15.56100	-22.34800	17.75800
H	-14.63200	-22.19700	17.58500
H	-15.98200	-22.26000	16.90300
O	-16.76200	-18.32300	15.52800
H	-16.90100	-18.69100	16.40000
H	-17.55100	-17.80800	15.35600
O	-16.16800	-13.22200	6.87000
H	-15.99200	-13.69300	7.68500
H	-16.85400	-12.59600	7.09800
O	-15.21200	-18.94500	12.91600
H	-15.54700	-18.98500	13.81200
H	-15.71200	-19.61000	12.44300
O	-21.23800	-23.53800	18.44200
H	-21.37600	-24.27000	19.04300
H	-20.32700	-23.62700	18.16000
O	-12.27600	-21.59900	3.19000
H	-12.89300	-22.32500	3.09300
H	-12.74400	-20.83800	2.84700
O	-18.60900	-23.82600	17.93000
H	-17.89900	-24.46700	17.88900
H	-18.65200	-23.57500	18.85300
O	-10.62100	-16.88900	20.85100
H	-10.67900	-17.57400	20.18400
H	-10.21900	-16.14800	20.39900
O	-19.89600	-26.84000	19.75000
H	-20.43300	-27.29100	20.40200
H	-20.48500	-26.70500	19.00700
O	-21.60200	-10.07300	13.64400
H	-21.36200	-9.44100	14.32200
H	-22.44500	-10.42400	13.93100
O	-20.48600	-27.38100	2.31200
H	-21.36300	-27.50200	1.94900
H	-20.01000	-28.17200	2.06000
O	-10.61900	-18.92500	19.08200
H	-11.50600	-19.26300	19.20500
H	-10.04900	-19.65700	19.31900
O	-28.96600	-22.59300	12.40600
H	-28.27900	-22.90700	11.81900
H	-29.12100	-21.68900	12.13300
O	-24.16200	-27.70400	16.83500
H	-24.24700	-28.31100	16.10000
H	-24.98400	-27.21300	16.83600
O	-17.08900	-14.26500	16.04800
H	-17.16200	-13.46000	16.56000
H	-17.53100	-14.06600	15.22300
O	-17.30000	-15.02800	9.55900
H	-18.08300	-14.48600	9.65200
H	-16.60000	-14.51100	9.95800
O	-13.99000	-29.21900	12.20200
H	-13.49300	-29.97200	11.88200
H	-14.76100	-29.18200	11.63600
O	-6.13700	-23.51500	13.84400
H	-6.14800	-22.63200	13.47300
H	-7.00500	-23.86800	13.64900
O	-14.62900	-14.36700	18.20400
H	-13.91600	-13.73200	18.13200
H	-15.12000	-14.27000	17.38800
O	-12.90700	-20.46200	9.77600
H	-13.84100	-20.34100	9.60600
H	-12.72900	-21.36400	9.51200
O	-17.40700	-27.07700	11.92900
H	-17.77700	-27.63000	12.61700
H	-16.91100	-27.68100	11.37700
O	-9.52000	-24.62000	10.31100
H	-9.05400	-23.78700	10.23700
H	-9.06900	-25.08500	11.01500
O	-19.57800	-16.61700	19.02500
H	-18.87700	-15.98400	19.17800
H	-20.04800	-16.27600	18.26400
O	-11.45200	-18.27500	7.30900
H	-11.17600	-18.39000	8.21900
H	-12.32200	-18.67400	7.27100
O	-11.54800	-32.25200	14.35000
H	-12.46200	-32.02100	14.51800
H	-11.11900	-32.16800	15.20100
O	-11.77800	-24.06300	16.85000
H	-12.43000	-23.81500	16.19500
H	-11.40000	-24.87600	16.51600
O	-14.81700	-14.02700	10.95300
H	-14.63500	-14.84500	11.41500
H	-13.99400	-13.81000	10.51500
O	-14.48300	-15.10000	8.21400
H	-13.95400	-15.61100	7.60100
H	-14.22000	-15.41600	9.07900
O	-15.72900	-23.32100	23.33600
H	-16.40500	-23.42100	24.00700
H	-14.90600	-23.47700	23.80000
O	-23.98100	-17.08400	15.95800
H	-23.39600	-17.82900	16.09100
H	-24.17800	-16.77300	16.84100
O	-21.12700	-14.04800	10.97400
H	-21.79600	-13.56400	10.49000
H	-21.26600	-13.80200	11.88800
O	-29.38900	-24.51300	14.67800
H	-29.35100	-25.04700	15.47200
H	-29.89500	-25.03900	14.05900
O	-16.98700	-12.01400	17.57200
H	-16.43500	-11.85100	18.33700
H	-16.64600	-11.42400	16.90000
O	-22.01000	-29.66700	18.17400
H	-21.94600	-28.80500	17.76200
H	-22.07300	-30.27900	17.44200
O	-22.53500	-27.59400	21.82200
H	-21.90800	-27.97700	22.43600
H	-23.39200	-27.81300	22.18700
O	-12.13500	-17.18800	23.42700
H	-11.59100	-16.57500	22.93300

H	-12.71700	-16.63000	23.94400
O	-25.75800	-25.78700	7.75400
H	-25.46300	-26.69600	7.70000
H	-26.65900	-25.80800	7.43200
O	-13.85000	-14.06600	14.55900
H	-13.97500	-14.86700	14.05000
H	-13.19200	-13.57100	14.07000
O	-22.22900	-27.71100	13.73600
H	-22.95000	-27.09700	13.59700
H	-21.44000	-27.17500	13.65800
O	-11.78600	-16.26500	16.18700
H	-12.73300	-16.38400	16.11400
H	-11.43800	-17.15300	16.26900
O	-19.42600	-30.37600	20.01000
H	-18.90300	-30.12900	20.77300
H	-19.64500	-29.54400	19.59100
O	-24.03000	-28.10500	7.87800
H	-24.35800	-28.98500	7.69500
H	-23.67600	-28.16000	8.76500
O	-14.78200	-16.47000	12.38700
H	-14.92900	-17.39800	12.56800
H	-13.95600	-16.44800	11.90300
O	-20.91500	-27.81800	8.24500
H	-20.57600	-27.28900	7.52300
H	-20.46800	-27.48000	9.02100
O	-11.17800	-15.64700	13.47400
H	-10.25300	-15.85300	13.33600
H	-11.34700	-15.89500	14.38200
O	-18.22100	-29.43400	17.69500
H	-18.56100	-30.23300	18.09700
H	-18.92700	-29.13400	17.12300
O	-17.56900	-32.34900	16.14800
H	-16.74700	-32.82300	16.02600
H	-17.32600	-31.57000	16.64800
O	-18.92900	-29.32000	6.44400
H	-18.20800	-28.94200	5.94100
H	-19.55200	-28.60200	6.54800
O	-14.78500	-28.61600	7.38000
H	-14.56900	-27.73300	7.07900
H	-15.26600	-29.00700	6.65100
O	-20.04500	-25.65700	13.95700
H	-20.19100	-25.34300	14.85000
H	-20.52300	-25.03900	13.40400
O	-18.18600	-28.66700	14.00500
H	-18.20000	-29.56300	13.66900
H	-17.46900	-28.66000	14.63900
O	-16.33300	-28.23300	16.05100
H	-16.59100	-27.31200	16.04900
H	-16.86500	-28.63000	16.74100
O	-11.38900	-15.12800	8.83000
H	-11.21500	-15.69400	8.07700
H	-10.89600	-14.32800	8.64800
O	-25.57100	-23.11700	16.89600
H	-26.11600	-22.34100	17.02800
H	-24.69300	-22.77000	16.73800
O	-18.78200	-29.78600	9.27400
H	-18.66100	-30.71400	9.47300
H	-19.07200	-29.77200	8.36200
O	-15.91300	-32.10000	12.41100
H	-15.89100	-32.03600	11.45600
H	-15.49600	-32.94000	12.60500
O	-5.53500	-22.36500	10.44900
H	-5.09200	-23.11400	10.05100
H	-6.41200	-22.37400	10.06600
O	-10.40100	-15.81200	5.92900
H	-9.70600	-15.19100	6.14800
H	-10.05900	-16.66200	6.20500
O	-24.78400	-23.99000	19.78900
H	-25.52600	-23.84200	20.37600
H	-25.10500	-23.72900	18.92600
O	-8.50200	-18.36200	7.45800
H	-7.97700	-18.02300	6.73300
H	-8.59000	-19.29700	7.27400
O	-16.84000	-14.44800	19.58700
H	-16.53100	-14.69300	20.45900
H	-16.04200	-14.24400	19.09900
O	-16.81800	-12.29400	11.81800
H	-17.25700	-12.78700	12.51100
H	-16.06700	-12.83700	11.57700
O	-24.33900	-16.10300	11.82000
H	-23.51300	-16.40300	11.44000
H	-24.68900	-16.87300	12.26900
O	-19.37000	-16.75100	13.89000
H	-19.99200	-17.41400	13.59000
H	-18.70000	-16.72500	13.20700
O	-26.13800	-18.18500	12.77900
H	-26.97500	-18.61200	12.59700
H	-26.16500	-17.99100	13.71600
O	-19.29800	-28.27200	23.48300
H	-19.24900	-27.39600	23.10100
H	-18.75600	-28.81500	22.91000
O	-8.81300	-17.72800	22.76300
H	-9.60300	-17.74600	22.22400
H	-9.04900	-17.19000	23.51800
O	-8.69300	-20.87900	20.07700
H	-8.64300	-21.04400	21.01800
H	-8.80100	-21.74600	19.68600
O	-19.80900	-18.20000	7.36600
H	-19.66000	-17.51700	6.71200
H	-19.00200	-18.22000	7.88100
O	-11.40200	-24.89600	21.39200
H	-10.50100	-25.03500	21.10100
H	-11.32800	-24.73100	22.33200
O	-17.06800	-27.51900	20.23800
H	-17.93800	-27.22600	19.96600
H	-16.83200	-26.93500	20.95900
O	-7.19100	-18.45900	20.32100
H	-7.48200	-18.09600	21.15700
H	-7.80400	-19.17300	20.14200
O	-5.99100	-20.89400	12.84100
H	-5.15700	-20.45500	13.00500
H	-5.85500	-21.37000	12.02200
O	-8.94200	-23.33600	18.78900
H	-8.46700	-23.14300	17.98100
H	-9.86600	-23.28000	18.54300
O	-22.05400	-22.17400	8.37200
H	-22.29900	-23.07700	8.57000
H	-21.88400	-22.17200	7.43000

O	-14.36400	-31.30100	17.75400
H	-13.84600	-30.56500	17.42900
H	-14.71900	-30.99500	18.58900
O	-22.78400	-26.44100	5.87000
H	-23.47700	-26.36600	5.21400
H	-23.02900	-27.20500	6.39300
O	-14.11300	-34.88100	13.27400
H	-13.83300	-35.54900	13.90000
H	-13.32800	-34.68800	12.76200
O	-16.31000	-15.70500	4.83700
H	-16.88200	-15.08600	4.38400
H	-15.73600	-15.15500	5.37100
O	-10.24200	-26.46000	17.02200
H	-9.29000	-26.36000	17.04300
H	-10.41200	-27.25200	17.53100
O	-12.22100	-29.37400	17.20800
H	-12.81600	-29.09400	16.51200
H	-12.50600	-28.88500	17.97900
O	-13.09000	-15.42200	5.96800
H	-12.15700	-15.48700	6.17100
H	-13.17800	-14.58600	5.50900
O	-10.46100	-16.10500	2.92800
H	-10.54000	-16.43900	3.82200
H	-9.64000	-15.61300	2.92700
O	-7.76300	-23.23900	16.13600
H	-7.79600	-24.17700	16.32200
H	-7.15300	-23.16100	15.40200
O	-15.92500	-24.36900	2.17900
H	-14.97900	-24.49900	2.24700
H	-16.30300	-25.10800	2.65500
O	-7.61400	-26.44800	16.18200
H	-8.13000	-27.23200	15.99500
H	-6.73000	-26.77600	16.34600
O	-9.95300	-32.70400	12.14100
H	-10.15000	-33.01200	13.02600
H	-9.34300	-31.97800	12.27100
O	-1.94600	-22.17700	15.27600
H	-1.21500	-22.43900	15.83700
H	-2.55500	-22.91400	15.31700
O	-3.86400	-24.03700	15.87400
H	-4.59100	-24.17100	15.26500
H	-4.27900	-23.72900	16.68000
O	-16.04100	-20.31800	23.63900
H	-15.12100	-20.14000	23.83300
H	-16.07600	-21.26200	23.48700
O	-17.74100	-26.44400	2.11400
H	-18.64700	-26.74100	2.19000
H	-17.46300	-26.74100	1.24700
O	-26.82300	-25.12400	12.56400
H	-27.14300	-25.97700	12.27100
H	-27.36900	-24.90900	13.32000
O	-23.04500	-22.40700	16.28100
H	-22.82600	-21.47600	16.30000
H	-22.63200	-22.76700	17.06600
O	-16.12400	-31.90100	8.26800
H	-16.93400	-32.24600	8.64400
H	-15.43600	-32.21000	8.85700
O	-14.19500	-32.82300	9.89100
H	-13.64200	-32.40300	10.55000
H	-13.95800	-33.74900	9.93300
O	-18.17200	-26.83400	25.67100
H	-18.27200	-25.88500	25.60200
H	-18.39000	-27.16200	24.79900
O	-18.97100	-29.58600	2.25500
H	-18.10900	-29.26000	2.51500
H	-18.78600	-30.37300	1.74300
O	-26.95400	-21.50300	14.53300
H	-27.64800	-21.91800	14.02000
H	-26.20700	-21.46100	13.93700
O	-10.81700	-30.71300	9.39900
H	-11.39800	-30.05400	9.01700
H	-11.08400	-31.53500	8.98700
O	-12.36700	-31.31200	11.60100
H	-11.86700	-31.20100	10.79200
H	-11.72400	-31.61600	12.24100
O	-18.30900	-23.40400	24.48800
H	-18.79200	-22.60400	24.69400
H	-18.22300	-23.39400	23.53400
O	-25.04000	-21.57200	12.41000
H	-25.06300	-21.47300	11.45800
H	-24.73000	-20.72500	12.73200
O	-22.38200	-19.51400	16.52400
H	-21.42700	-19.54600	16.45700
H	-22.58000	-19.97500	17.34000
O	-21.79200	-20.94400	19.09200
H	-20.97200	-20.44900	19.11500
H	-21.51800	-21.86100	19.06300
O	-12.03500	-28.55300	8.45400
H	-12.92200	-28.26800	8.23600
H	-11.62000	-28.71300	7.60700
O	-8.68400	-28.52800	12.17000
H	-7.90800	-28.03900	12.44200
H	-9.40900	-28.10200	12.62700
O	-6.24600	-27.81500	13.22700
H	-5.65200	-28.05600	12.51700
H	-6.17400	-28.53500	13.85500
O	-24.02700	-13.97200	13.54200
H	-24.16100	-14.73200	12.97500
H	-24.44300	-13.24800	13.07400
O	-12.89100	-18.78800	2.54900
H	-12.18700	-18.58300	3.16500
H	-13.61400	-18.22100	2.81400
O	-12.06100	-23.03700	8.66900
H	-12.11700	-22.58000	7.83000
H	-11.50800	-23.79800	8.49200
O	-8.25000	-27.24800	9.49400
H	-8.50700	-27.64900	10.32500
H	-8.87500	-26.53400	9.36900
O	-3.93900	-24.50900	12.00200
H	-4.49900	-24.43900	12.77500
H	-4.54500	-24.68500	11.28100
O	-20.87000	-13.07500	13.75300
H	-20.98600	-12.13100	13.65100
H	-21.69000	-13.37800	14.14200
O	-10.98200	-18.35000	4.59900
H	-10.14700	-18.78200	4.41700
H	-11.14400	-18.52200	5.52700
O	-11.32700	-21.84700	5.96500

H	-11.38300	-22.01200	5.02300
H	-11.95300	-21.13900	6.12100
O	-26.53800	-26.61000	19.79100
H	-25.89200	-27.12000	20.28000
H	-26.74600	-25.87300	20.36500
O	-14.01300	-19.54200	25.29000
H	-13.14400	-19.36800	24.92900
H	-14.49200	-18.72300	25.16800
O	-18.01800	-32.34600	5.54300
H	-18.03000	-31.40200	5.70000
H	-17.12500	-32.52900	5.24900
O	-19.84400	-12.42200	16.96900
H	-18.94100	-12.39000	17.28600
H	-20.07000	-13.35200	16.98400
O	-18.08900	-13.66900	13.63000
H	-19.03900	-13.66900	13.51600
H	-17.78600	-14.41600	13.11400
O	-14.20300	-35.75200	9.79800
H	-14.96900	-36.30600	9.94300
H	-13.79200	-35.68000	10.65900
O	-22.19800	-29.46300	23.88800
H	-21.33500	-29.82000	24.09900
H	-22.77200	-29.80200	24.57600
O	-2.35400	-7.83300	14.69500
H	-1.71600	-7.95200	13.99100
H	-2.47100	-6.88500	14.75300
O	-5.54500	-7.88000	14.63100
H	-5.88300	-7.99600	13.74300
H	-4.67600	-8.28100	14.61100
O	-9.68600	-6.02700	10.62000
H	-10.24900	-6.11800	9.85200
H	-9.80100	-6.84700	11.10100
O	-5.06300	-6.72700	20.58900
H	-5.07400	-7.12200	19.71700
H	-4.90100	-7.46000	21.18300
O	-12.13900	-4.53200	13.45900
H	-11.34700	-4.87300	13.04300
H	-11.90000	-4.42200	14.37900
O	-10.18000	-5.27100	6.33100
H	-9.40700	-5.81800	6.19200
H	-9.82800	-4.40600	6.54200
O	-8.34200	-3.94300	19.53700
H	-8.63300	-3.03900	19.42200
H	-7.70600	-3.90200	20.25200
O	-7.41600	-6.56500	16.12700
H	-6.82000	-6.79000	15.41300
H	-7.35900	-7.30700	16.73000
O	-7.84400	-9.11200	3.96200
H	-7.59100	-9.63300	3.20100
H	-8.78100	-9.27500	4.06500
O	2.60100	-10.17000	12.67300
H	3.33400	-10.74100	12.90700
H	1.84500	-10.55800	13.11400
O	-8.12400	-7.20900	6.16500
H	-8.24100	-7.88400	6.83300
H	-8.01400	-7.69500	5.34800
O	0.12400	-16.45800	17.13300
H	-0.20800	-15.58900	16.90900
H	-0.47200	-16.77500	17.81200
O	-6.14000	-12.71900	4.86600
H	-5.97300	-11.85100	5.23300
H	-6.33100	-12.55600	3.94200
O	-3.38300	-4.25700	16.53200
H	-3.68300	-3.35500	16.42400
H	-3.72800	-4.52400	17.38400
O	-9.93200	-7.80600	12.56600
H	-10.85700	-7.93800	12.77400
H	-9.68300	-7.02600	13.06300
O	0.01700	-11.73900	14.21200
H	-0.89200	-11.80700	13.91800
H	-0.03400	-11.26200	15.04000
O	-6.16200	-10.26300	5.85000
H	-6.28200	-9.81700	6.68900
H	-6.85600	-9.91400	5.29200
O	-12.57100	-8.16200	13.40000
H	-12.44200	-7.90900	14.31400
H	-13.25100	-7.57000	13.08100
O	-8.40100	2.50700	9.49900
H	-8.47800	3.45800	9.57800
H	-9.00500	2.16200	10.15700
O	-5.90900	-10.32000	22.15500
H	-6.68600	-9.76000	22.14600
H	-5.27000	-9.83400	22.67700
O	1.88300	-13.53700	15.99000
H	1.24000	-13.24400	15.34400
H	1.37800	-13.65200	16.79500
O	-0.46300	-9.79100	6.67800
H	0.44900	-9.55800	6.50300
H	-0.97200	-9.20700	6.11600
O	-7.65400	-7.51100	21.71700
H	-6.91300	-6.97300	21.44100
H	-8.36100	-6.88500	21.87400
O	-2.17900	-5.36800	20.31400
H	-1.77100	-5.81000	19.57000
H	-2.90100	-5.94300	20.56800
O	-5.82600	-10.43100	17.68800
H	-4.98000	-10.00700	17.83100
H	-5.88600	-11.08800	18.38200
O	-2.67600	-12.77100	19.34600
H	-2.92000	-12.01600	18.80900
H	-2.40000	-12.39200	20.18100
O	-0.72200	-6.22700	18.02400
H	-0.51000	-5.58400	17.34700
H	-0.76900	-7.06100	17.55600
O	-3.11900	-13.61500	14.92700
H	-3.98100	-13.31700	15.21900
H	-3.09300	-13.39800	13.99500
O	-9.41100	-5.52400	14.27300
H	-8.84100	-5.09500	13.63400
H	-8.82000	-5.80500	14.97100
O	-8.91800	-10.35700	22.51900
H	-9.54800	-9.67600	22.75200
H	-9.02500	-10.46900	21.57400
O	-13.62400	-10.69000	13.44500
H	-14.41400	-10.43400	12.96900
H	-13.13300	-9.87500	13.55100
O	0.39300	-3.35000	16.19600
H	1.21400	-3.75200	15.91400

H	0.38900	-3.45300	17.14700
O	-11.67200	-10.32600	10.40400
H	-12.46900	-10.21400	10.92200
H	-11.90000	-9.98700	9.53800
O	-5.84200	-3.80100	18.35200
H	-5.77700	-3.33900	19.18800
H	-6.55100	-4.43100	18.48000
O	-15.34000	-10.43700	15.87700
H	-15.08900	-9.55200	16.14300
H	-14.80400	-10.61700	15.10400
O	8.48300	-7.00600	12.54400
H	7.73000	-7.57500	12.38900
H	8.61800	-7.03900	13.49100
O	-9.64000	-16.05500	24.88100
H	-10.03400	-16.60900	25.55600
H	-8.82400	-15.74400	25.27200
O	-12.67400	-4.61700	8.79300
H	-12.16200	-3.82300	8.94300
H	-12.02200	-5.31400	8.72600
O	-10.86500	-1.50000	10.87500
H	-10.68800	-0.57800	10.68700
H	-10.06900	-1.81400	11.30400
O	-6.72300	-14.63900	22.47300
H	-5.98300	-14.03900	22.39400
H	-6.49200	-15.38200	21.91500
O	-11.57900	-9.45400	20.75100
H	-11.69400	-10.35500	21.05200
H	-12.43500	-9.20700	20.40000
O	-4.50000	-1.51200	22.35000
H	-4.94400	-2.29800	22.03200
H	-3.83800	-1.32400	21.68400
O	-9.74100	0.01800	16.87600
H	-9.19100	-0.11100	16.10400
H	-10.58000	0.31700	16.52500
O	-2.40200	-1.23200	20.37800
H	-2.42000	-0.41100	19.88600
H	-1.48000	-1.35600	20.60300
O	-5.94500	-3.71700	21.22000
H	-5.41200	-4.47900	20.99000
H	-6.47300	-4.01000	21.96300
O	-2.77800	-18.61100	16.75000
H	-3.12100	-17.72100	16.67400
H	-2.41000	-18.80100	15.88700
O	-14.70900	-6.28800	9.56900
H	-13.87200	-5.87800	9.35200
H	-14.53100	-7.22800	9.53900
O	-5.22800	-13.77500	10.31600
H	-6.08400	-14.16500	10.13600
H	-4.81500	-13.69600	9.45500
O	-3.69800	-16.24100	14.71500
H	-4.20200	-16.27800	13.90100
H	-3.40500	-15.33200	14.77400
O	-7.45400	-16.15700	13.72100
H	-6.58900	-16.35300	13.36200
H	-7.38600	-16.38600	14.64800
O	-9.84800	-13.92700	11.09300
H	-9.27300	-14.14900	10.36000
H	-9.26000	-13.85800	11.84600
O	-9.52100	-11.89600	5.11300
H	-10.35700	-11.55900	5.43300
H	-9.66000	-12.03100	4.17500
O	-7.54500	-17.05300	9.82000
H	-8.19900	-17.36800	9.19600
H	-7.58700	-17.67500	10.54600
O	-6.43400	-6.77100	1.73600
H	-7.34100	-7.00200	1.93500
H	-5.93700	-7.56700	1.92300
O	-4.31200	-10.56400	14.14500
H	-5.22900	-10.34300	13.98700
H	-3.89600	-10.49000	13.28600
O	-1.08000	-12.13000	11.25700
H	-1.81800	-12.64200	11.58800
H	-0.82700	-12.57000	10.44600
O	-0.97900	-19.35200	11.85200
H	-0.67300	-20.21200	11.56400
H	-0.25400	-18.76000	11.65100
O	-14.68700	-8.94900	10.24000
H	-14.88400	-9.54200	9.51500
H	-15.24200	-9.25400	10.95700
O	-6.70700	-9.05700	12.35900
H	-6.65200	-10.00900	12.28300
H	-7.51800	-8.90100	12.84300
O	-8.76800	-10.43900	10.11800
H	-9.58100	-10.13900	10.52500
H	-8.27200	-10.83300	10.83600
O	-6.61700	-0.44300	12.05200
H	-6.65900	-0.21700	11.12200
H	-5.70100	-0.67700	12.20000
O	-3.17000	-9.97500	18.95000
H	-2.81500	-9.74800	19.80900
H	-2.52300	-9.64300	18.32700
O	-9.27600	-12.86700	15.73400
H	-8.63600	-13.00800	15.03700
H	-10.10800	-13.15400	15.35800
O	2.58600	-4.78500	14.66600
H	3.02000	-4.87800	15.51400
H	3.30000	-4.80900	14.02800
O	-8.50000	-3.56700	11.84800
H	-7.70600	-4.06200	11.64600
H	-9.21100	-4.09500	11.48500
O	-3.68100	-1.18500	16.19900
H	-3.62700	-0.61600	16.96700
H	-4.54900	-1.01500	15.83400
O	-12.19200	-9.42300	7.92400
H	-13.12200	-9.48400	8.14100
H	-12.09600	-9.94500	7.12700
O	-12.27300	-12.89700	10.51200
H	-12.01300	-11.98200	10.40300
H	-11.45200	-13.36500	10.66100
O	-10.80100	-11.48300	17.68100
H	-10.12900	-11.69200	17.03300
H	-11.62800	-11.68100	17.24200
O	3.44800	-4.74300	17.19900
H	3.64200	-3.83800	17.44500
H	3.34800	-5.20200	18.03300
O	-4.89800	-14.85700	19.69800
H	-5.54600	-14.15800	19.60400
H	-4.12800	-14.42300	20.06600

O	-1.49000	-4.59200	22.77300
H	-1.64200	-4.82100	21.85600
H	-1.50000	-3.63500	22.78600
O	-4.72200	-7.01900	17.75800
H	-3.79300	-6.86800	17.58500
H	-5.14300	-6.93100	16.90300
O	-5.92300	-2.99400	15.52800
H	-6.06200	-3.36200	16.40000
H	-6.71200	-2.47900	15.35600
O	-10.39800	-8.20900	18.44200
H	-10.53600	-8.94100	19.04300
H	-9.48800	-8.29800	18.16000
O	-7.77000	-8.49700	17.93000
H	-7.06000	-9.13800	17.88900
H	-7.81300	-8.24600	18.85300
O	0.21900	-1.56000	20.85100
H	0.16000	-2.24400	20.18400
H	0.62100	-0.81800	20.39900
O	-9.05700	-11.51000	19.75000
H	-9.59300	-11.96100	20.40200
H	-9.64500	-11.37600	19.00700
O	-10.76200	5.25600	13.64400
H	-10.52200	5.88800	14.32200
H	-11.60500	4.90500	13.93100
O	-9.64700	-12.05200	2.31200
H	-10.52400	-12.17300	1.94900
H	-9.17100	-12.84300	2.06000
O	0.22100	-3.59600	19.08200
H	-0.66700	-3.93400	19.20500
H	0.79000	-4.32800	19.31900
O	-18.12700	-7.26400	12.40600
H	-17.43900	-7.57800	11.81900
H	-18.28200	-6.36000	12.13300
O	-13.32300	-12.37500	16.83500
H	-13.40700	-12.98200	16.10000
H	-14.14500	-11.88400	16.83600
O	-6.24900	1.06400	16.04800
H	-6.32200	1.86900	16.56000
H	-6.69200	1.26300	15.22300
O	-6.46000	0.30100	9.55900
H	-7.24400	0.84300	9.65200
H	-5.76100	0.81800	9.95800
O	-3.15100	-13.89000	12.20200
H	-2.65400	-14.64300	11.88200
H	-3.92200	-13.85300	11.63600
O	4.70200	-8.18500	13.84400
H	4.69100	-7.30300	13.47300
H	3.83400	-8.53800	13.64900
O	-3.79000	0.96300	18.20400
H	-3.07700	1.59700	18.13200
H	-4.28100	1.05900	17.38800
O	-6.56700	-11.74800	11.92900
H	-6.93700	-12.30100	12.61700
H	-6.07100	-12.35200	11.37700
O	1.32000	-9.29100	10.31100
H	1.78600	-8.45800	10.23700
H	1.77000	-9.75600	11.01500
O	-8.73800	-1.28800	19.02500
H	-8.03700	-0.65400	19.17800
H	-9.20900	-0.94700	18.26400
O	-0.70900	-16.92300	14.35000
H	-1.62300	-16.69200	14.51800
H	-0.28000	-16.83900	15.20100
O	-0.93800	-8.73400	16.85000
H	-1.59100	-8.48600	16.19500
H	-0.56000	-9.54700	16.51600
O	-3.97700	1.30200	10.95300
H	-3.79500	0.48500	11.41500
H	-3.15400	1.51900	10.51500
O	-4.88900	-7.99200	23.33600
H	-5.56500	-8.09200	24.00700
H	-4.06700	-8.14800	23.80000
O	-13.14200	-1.75400	15.95800
H	-12.55600	-2.50000	16.09100
H	-13.33900	-1.44400	16.84100
O	-10.28800	1.28100	10.97400
H	-10.95700	1.76500	10.49000
H	-10.42600	1.52800	11.88800
O	-18.55000	-9.18400	14.67800
H	-18.51100	-9.71700	15.47200
H	-19.05600	-9.71000	14.05900
O	-6.14700	3.31500	17.57200
H	-5.59500	3.47800	18.33700
H	-5.80700	3.90600	16.90000
O	-11.17100	-14.33700	18.17400
H	-11.10700	-13.47600	17.76200
H	-11.23400	-14.95000	17.44200
O	-11.69500	-12.26500	21.82200
H	-11.06800	-12.64700	22.43600
H	-12.55300	-12.48400	22.18700
O	-1.29600	-1.85900	23.42700
H	-0.75200	-1.24600	22.93300
H	-1.87700	-1.30100	23.94400
O	-14.91900	-10.45700	7.75400
H	-14.62400	-11.36600	7.70000
H	-15.82000	-10.47900	7.43200
O	-3.01000	1.26300	14.55900
H	-3.13500	0.46200	14.05000
H	-2.35200	1.75800	14.07000
O	-11.38900	-12.38200	13.73600
H	-12.11100	-11.76800	13.59700
H	-10.60000	-11.84600	13.65800
O	-0.94700	-0.93600	16.18700
H	-1.89400	-1.05500	16.11400
H	-0.59900	-1.82400	16.26900
O	-8.58700	-15.04600	20.01000
H	-8.06300	-14.80000	20.77300
H	-8.80600	-14.21400	19.59100
O	-13.19000	-12.77600	7.87800
H	-13.51900	-13.65600	7.69500
H	-12.83600	-12.83100	8.76500
O	-10.07600	-12.48900	8.24500
H	-9.73600	-11.96000	7.52300
H	-9.62900	-12.15100	9.02100
O	-7.38200	-14.10500	17.69500
H	-7.72200	-14.90400	18.09700
H	-8.08800	-13.80500	17.12300
O	-6.73000	-17.02000	16.14800

H	-5.90700	-17.49400	16.02600
H	-6.48600	-16.24100	16.64800
O	-8.08900	-13.99100	6.44400
H	-7.36800	-13.61300	5.94100
H	-8.71300	-13.27200	6.54800
O	-3.94600	-13.28700	7.38000
H	-3.73000	-12.40400	7.07900
H	-4.42700	-13.67800	6.65100
O	-9.20600	-10.32800	13.95700
H	-9.35200	-10.01400	14.85000
H	-9.68400	-9.71000	13.40400
O	-7.34600	-13.33800	14.00500
H	-7.36000	-14.23400	13.66900
H	-6.62900	-13.33100	14.63900
O	-5.49400	-12.90400	16.05100
H	-5.75200	-11.98200	16.04900
H	-6.02500	-13.30100	16.74100
O	-14.73200	-7.78800	16.89600
H	-15.27600	-7.01200	17.02800
H	-13.85400	-7.44100	16.73800
O	-7.94300	-14.45600	9.27400
H	-7.82100	-15.38500	9.47300
H	-8.23300	-14.44300	8.36200
O	-5.07400	-16.77100	12.41100
H	-5.05100	-16.70700	11.45600
H	-4.65700	-17.61100	12.60500
O	5.30500	-7.03500	10.44900
H	5.74800	-7.78500	10.05100
H	4.42700	-7.04400	10.06600
O	-13.94500	-8.66100	19.78900
H	-14.68700	-8.51200	20.37600
H	-14.26600	-8.40000	18.92600
O	-6.00100	0.88100	19.58700
H	-5.69200	0.63600	20.45900
H	-5.20300	1.08600	19.09900
O	-5.97900	3.03500	11.81800
H	-6.41800	2.54200	12.51100
H	-5.22800	2.49200	11.57700
O	-13.49900	-0.77400	11.82000
H	-12.67300	-1.07400	11.44000
H	-13.85000	-1.54300	12.26900
O	-8.53100	-1.42100	13.89000
H	-9.15300	-2.08400	13.59000
H	-7.86100	-1.39500	13.20700
O	-15.29800	-2.85500	12.77900
H	-16.13500	-3.28300	12.59700
H	-15.32600	-2.66200	13.71600
O	-8.45900	-12.94300	23.48300
H	-8.41000	-12.06700	23.10100
H	-7.91600	-13.48600	22.91000
O	2.02700	-2.39900	22.76300
H	1.23600	-2.41700	22.22400
H	1.79000	-1.86000	23.51800
O	2.14700	-5.55000	20.07700
H	2.19600	-5.71500	21.01800
H	2.03800	-6.41700	19.68600
O	-8.97000	-2.87100	7.36600
H	-8.82100	-2.18800	6.71200
H	-8.16300	-2.89100	7.88100
O	-0.56300	-9.56700	21.39200
H	0.33800	-9.70600	21.10100
H	-0.48800	-9.40100	22.33200
O	-6.22900	-12.19000	20.23800
H	-7.09800	-11.89700	19.96600
H	-5.99300	-11.60600	20.95900
O	3.64800	-3.13000	20.32100
H	3.35700	-2.76700	21.15700
H	3.03600	-3.84400	20.14200
O	4.84800	-5.56500	12.84100
H	5.68300	-5.12600	13.00500
H	4.98400	-6.04100	12.02200
O	1.89700	-8.00700	18.78900
H	2.37300	-7.81400	17.98100
H	0.97400	-7.95100	18.54300
O	-11.21500	-6.84500	8.37200
H	-11.46000	-7.74800	8.57000
H	-11.04500	-6.84300	7.43000
O	-3.52500	-15.97200	17.75400
H	-3.00700	-15.23600	17.42900
H	-3.87900	-15.66600	18.58900
O	-11.94500	-11.11200	5.87000
H	-12.63800	-11.03700	5.21400
H	-12.19000	-11.87600	6.39300
O	-3.27400	-19.55200	13.27400
H	-2.99300	-20.22000	13.90000
H	-2.48900	-19.35900	12.76200
O	0.59700	-11.13000	17.02200
H	1.54900	-11.03100	17.04300
H	0.42800	-11.92300	17.53100
O	-1.38100	-14.04500	17.20800
H	-1.97600	-13.76400	16.51200
H	-1.66600	-13.55500	17.97900
O	3.07600	-7.91000	16.13600
H	3.04400	-8.84800	16.32200
H	3.68600	-7.83100	15.40200
O	-5.08600	-9.04000	2.17900
H	-4.14000	-9.17000	2.24700
H	-5.46400	-9.77900	2.65500
O	3.22600	-11.11900	16.18200
H	2.70900	-11.90300	15.99500
H	4.11000	-11.44700	16.34600
O	0.88700	-17.37500	12.14100
H	0.68900	-17.68300	13.02600
H	1.49700	-16.64800	12.27100
O	8.89400	-6.84800	15.27600
H	9.62400	-7.11000	15.83700
H	8.28400	-7.58500	15.31700
O	6.97500	-8.70700	15.87400
H	6.24900	-8.84200	15.26500
H	6.56000	-8.40000	16.68000
O	-5.20200	-4.98800	23.63900
H	-4.28100	-4.81100	23.83300
H	-5.23700	-5.93300	23.48700
O	-6.90100	-11.11500	2.11400
H	-7.80800	-11.41200	2.19000
H	-6.62400	-11.41200	1.24700
O	-15.98300	-9.79500	12.56400
H	-16.30300	-10.64800	12.27100

H	-16.53000	-9.58000	13.32000
O	-12.20500	-7.07800	16.28100
H	-11.98700	-6.14700	16.30000
H	-11.79300	-7.43700	17.06600
O	-5.28500	-16.57100	8.26800
H	-6.09500	-16.91700	8.64400
H	-4.59700	-16.88100	8.85700
O	-3.35600	-17.49400	9.89100
H	-2.80300	-17.07400	10.55000
H	-3.11900	-18.42000	9.93300
O	-7.33300	-11.50500	25.67100
H	-7.43300	-10.55600	25.60200
H	-7.55100	-11.83300	24.79900
O	-8.13100	-14.25600	2.25500
H	-7.26900	-13.93100	2.51500
H	-7.94600	-15.04300	1.74300
O	-16.11500	-6.17400	14.53300
H	-16.80800	-6.58900	14.02000
H	-15.36700	-6.13200	13.93700
O	0.02200	-15.38400	9.39900
H	-0.55800	-14.72500	9.01700
H	-0.24500	-16.20600	8.98700
O	-1.52800	-15.98300	11.60100
H	-1.02800	-15.87100	10.79200
H	-0.88400	-16.28700	12.24100
O	-7.47000	-8.07500	24.48800
H	-7.95300	-7.27500	24.69400
H	-7.38300	-8.06400	23.53400
O	-14.20100	-6.24200	12.41000
H	-14.22400	-6.14400	11.45800
H	-13.89100	-5.39600	12.73200
O	-11.54200	-4.18500	16.52400
H	-10.58800	-4.21600	16.45700
H	-11.74100	-4.64600	17.34000
O	-1.19600	-13.22400	8.45400
H	-2.08300	-12.93900	8.23600
H	-0.78000	-13.38400	7.60700
O	2.15600	-13.19900	12.17000
H	2.93200	-12.70900	12.44200
H	1.43100	-12.77300	12.62700
O	4.59300	-12.48600	13.22700
H	5.18800	-12.72700	12.51700
H	4.66600	-13.20500	13.85500
O	-13.18700	1.35700	13.54200
H	-13.32200	0.59700	12.97500
H	-13.60400	2.08100	13.07400
O	2.58900	-11.91900	9.49400
H	2.33300	-12.32000	10.32500
H	1.96400	-11.20500	9.36900
O	6.90000	-9.18000	12.00200
H	6.34000	-9.11000	12.77500
H	6.29500	-9.35500	11.28100
O	-10.03000	2.25400	13.75300
H	-10.14700	3.19900	13.65100
H	-10.85100	1.95100	14.14200
O	-15.69900	-11.28100	19.79100
H	-15.05300	-11.79100	20.28000
H	-15.90700	-10.54400	20.36500
O	-3.17300	-4.21300	25.29000
H	-2.30400	-4.03900	24.92900
H	-3.65300	-3.39300	25.16800
O	-7.17800	-17.01700	5.54300
H	-7.19100	-16.07300	5.70000
H	-6.28600	-17.20000	5.24900
O	-9.00500	2.90700	16.96900
H	-8.10200	2.93900	17.28600
H	-9.23100	1.97700	16.98400
O	-7.24900	1.66000	13.63000
H	-8.20000	1.66000	13.51600
H	-6.94600	0.91300	13.11400
O	-3.36300	-20.42300	9.79800
H	-4.13000	-20.97700	9.94300
H	-2.95200	-20.35000	10.65900
O	-11.35900	-14.13400	23.88800
H	-10.49600	-14.49000	24.09900
H	-11.93300	-14.47300	24.57600
O	8.48600	7.49600	14.69500
H	9.12400	7.37700	13.99100
H	8.36800	8.44400	14.75300
O	5.29500	7.44900	14.63100
H	4.95700	7.33300	13.74300
H	6.16400	7.04800	14.61100
O	1.15300	9.30200	10.62000
H	0.59000	9.21100	9.85200
H	1.03900	8.48200	11.10100
O	5.77700	8.60200	20.58900
H	5.76600	8.20800	19.71700
H	5.93900	7.86900	21.18300
O	-1.30000	10.79700	13.45900
H	-0.50800	10.45700	13.04300
H	-1.06000	10.90700	14.37900
O	6.73300	12.96900	5.93200
H	6.08200	13.55500	5.54500
H	7.52800	13.50000	5.99100
O	2.49700	11.38600	19.53700
H	2.20600	12.29100	19.42200
H	3.13300	11.42800	20.25200
O	3.42300	8.76400	16.12700
H	4.01900	8.53900	15.41300
H	3.48100	8.02200	16.73000
O	8.04000	5.22100	11.77000
H	8.82600	4.80300	11.41900
H	8.30400	6.12600	11.93800
O	8.21100	10.65900	6.46900
H	7.57600	10.73200	7.18100
H	7.88100	11.24800	5.79100
O	13.44100	5.15900	12.67300
H	14.17300	4.58900	12.90700
H	12.68500	4.77100	13.11400
O	10.96300	-1.12900	17.13300
H	10.63200	-0.26000	16.90900
H	10.36800	-1.44500	17.81200
O	7.45600	11.07200	16.53200
H	7.15700	11.97500	16.42400
H	7.11200	10.80500	17.38400
O	0.90800	7.52400	12.56600
H	-0.01700	7.39100	12.77400
H	1.15600	8.30300	13.06300

O	10.85600	3.59000	14.21200
H	9.94800	3.52200	13.91800
H	10.80500	4.06800	15.04000
O	-1.73100	7.16700	13.40000
H	-1.60200	7.42000	14.31400
H	-2.41200	7.76000	13.08100
O	2.43900	17.83700	9.49900
H	2.36100	18.78700	9.57800
H	1.83500	17.49100	10.15700
O	4.93000	5.00900	22.15500
H	4.15400	5.56900	22.14600
H	5.56900	5.49500	22.67700
O	12.72200	1.79200	15.99000
H	12.08000	2.08500	15.34400
H	12.21800	1.67700	16.79500
O	3.18500	7.81800	21.71700
H	3.92600	8.35600	21.44100
H	2.47900	8.44500	21.87400
O	8.66000	9.96100	20.31400
H	9.06900	9.51900	19.57000
H	7.93800	9.38600	20.56800
O	5.01400	4.89800	17.68800
H	5.86000	5.32200	17.83100
H	4.95300	4.24100	18.38200
O	8.98300	10.52100	14.07000
H	8.35400	10.68600	13.36700
H	8.59600	10.93500	14.84100
O	12.86200	11.45600	11.42400
H	12.17700	11.83600	10.87400
H	12.94700	12.06600	12.15700
O	8.16300	2.55900	19.34600
H	7.92000	3.31300	18.80900
H	8.43900	2.93700	20.18100
O	10.11700	9.10200	18.02400
H	10.32900	9.74600	17.34700
H	10.07000	8.26900	17.55600
O	7.72000	1.71400	14.92700
H	6.85800	2.01200	15.21900
H	7.74600	1.93100	13.99500
O	4.53600	10.25800	11.33500
H	5.00000	10.53700	10.54500
H	4.27200	9.35700	11.15200
O	1.42900	9.80600	14.27300
H	1.99900	10.23400	13.63400
H	2.01900	9.52400	14.97100
O	1.92100	4.97200	22.51900
H	1.29100	5.65400	22.75200
H	1.81500	4.86000	21.57400
O	-2.78400	4.63900	13.44500
H	-3.57500	4.89500	12.96900
H	-2.29300	5.45400	13.55100
O	11.23200	11.98000	16.19600
H	12.05400	11.57800	15.91400
H	11.22800	11.87600	17.14700
O	4.99800	11.52800	18.35200
H	5.06200	11.99000	19.18800
H	4.28900	10.89800	18.48000
O	-4.50100	4.89200	15.87700
H	-4.25000	5.77700	16.14300
H	-3.96500	4.71300	15.10400
O	19.32300	8.32300	12.54400
H	18.56900	7.75400	12.38900
H	19.45700	8.29000	13.49100
O	4.71200	13.19500	8.22000
H	4.57700	14.04000	8.64800
H	5.25300	13.39700	7.45600
O	-1.83500	10.71200	8.79300
H	-1.32200	11.50700	8.94300
H	-1.18200	10.01500	8.72600
O	-0.02500	13.82900	10.87500
H	0.15100	14.75100	10.68700
H	0.77000	13.51500	11.30400
O	4.11600	0.69000	22.47300
H	4.85700	1.29000	22.39400
H	4.34800	-0.05200	21.91500
O	-0.74000	5.87500	20.75100
H	-0.85500	4.97400	21.05200
H	-1.59600	6.12200	20.40000
O	6.33900	13.81700	22.35000
H	5.89600	13.03100	22.03200
H	7.00100	14.00500	21.68400
O	1.09800	15.34700	16.87600
H	1.64900	15.21800	16.10400
H	0.25900	15.64600	16.52500
O	8.43700	14.09700	20.37800
H	8.41900	14.91800	19.88600
H	9.35900	13.97300	20.60300
O	4.89500	11.61200	21.22000
H	5.42700	10.85100	20.99000
H	4.36600	11.31900	21.96300
O	8.06200	-3.28200	16.75000
H	7.71800	-2.39200	16.67400
H	8.43000	-3.47200	15.88700
O	7.14100	-0.91200	14.71500
H	6.63800	-0.94900	13.90100
H	7.43400	-0.00200	14.77400
O	13.00000	9.46200	7.19200
H	12.22300	9.12300	6.74800
H	13.67900	9.46700	6.51800
O	6.26000	10.74000	8.73200
H	5.73200	10.00600	8.42000
H	5.75900	11.51800	8.48600
O	11.23900	8.93500	13.84900
H	10.58200	9.62500	13.93700
H	12.04300	9.32500	14.19200
O	10.05000	7.29000	11.89900
H	10.56300	7.92300	12.40200
H	10.70000	6.69000	11.53400
O	7.31200	17.26800	4.65500
H	6.63800	17.57100	5.26400
H	7.69900	18.07100	4.30600
O	6.52800	4.76500	14.14500
H	5.61000	4.98600	13.98700
H	6.94400	4.83900	13.28600
O	10.81600	12.27000	10.02700
H	10.36200	11.42900	10.08400
H	10.18500	12.90700	10.36500
O	9.75900	3.20000	11.25700

H	9.02100	2.68700	11.58800
H	10.01200	2.76000	10.44600
O	9.25900	14.10800	10.96200
H	9.55200	14.66900	10.24300
H	9.76900	14.40100	11.71800
O	9.86000	-4.02300	11.85200
H	10.16600	-4.88300	11.56400
H	10.58600	-3.43100	11.65100
O	-3.84700	6.38000	10.24000
H	-4.04500	5.78700	9.51500
H	-4.40300	6.07600	10.95700
O	4.13200	6.27300	12.35900
H	4.18700	5.32000	12.28300
H	3.32200	6.42900	12.84300
O	4.22300	14.88600	12.05200
H	4.18000	15.11200	11.12200
H	5.13900	14.65200	12.20000
O	7.66900	5.35400	18.95000
H	8.02400	5.58200	19.80900
H	8.31600	5.68600	18.32700
O	13.39900	8.47600	9.65500
H	13.26100	8.66600	8.72700
H	13.04300	9.23800	10.11100
O	1.56300	2.46200	15.73400
H	2.20300	2.32100	15.03700
H	0.73100	2.17600	15.35800
O	13.42600	10.54400	14.66600
H	13.86000	10.45200	15.51400
H	14.13900	10.52000	14.02800
O	2.33900	11.76200	11.84800
H	3.13300	11.26700	11.64600
H	1.62800	11.23400	11.48500
O	7.15900	14.14400	16.19900
H	7.21200	14.71300	16.96700
H	6.29000	14.31400	15.83400
O	-1.43400	2.43200	10.51200
H	-1.17300	3.34700	10.40300
H	-0.61200	1.96500	10.66100
O	0.03800	3.84600	17.68100
H	0.71100	3.63800	17.03300
H	-0.78900	3.64800	17.24200
O	14.28700	10.58600	17.19900
H	14.48100	11.49100	17.44500
H	14.18700	10.12700	18.03300
O	5.94100	0.47200	19.69800
H	5.29400	1.17100	19.60400
H	6.71100	0.90600	20.06600
O	9.34900	10.73700	22.77300
H	9.19700	10.50800	21.85600
H	9.33900	11.69400	22.78600
O	6.11700	8.31000	17.75800
H	7.04700	8.46100	17.58500
H	5.69700	8.39900	16.90300
O	4.91600	12.33500	15.52800
H	4.77700	11.96700	16.40000
H	4.12800	12.85000	15.35600
O	5.51100	17.43600	6.87000
H	5.68600	16.96500	7.68500
H	4.82400	18.06300	7.09800
O	6.46700	11.71300	12.91600
H	6.13200	11.67400	13.81200
H	5.96700	11.04800	12.44300
O	0.44100	7.12000	18.44200
H	0.30300	6.38800	19.04300
H	1.35200	7.03100	18.16000
O	3.06900	6.83200	17.93000
H	3.77900	6.19200	17.88900
H	3.02600	7.08300	18.85300
O	11.05800	13.76900	20.85100
H	10.99900	13.08500	20.18400
H	11.46000	14.51100	20.39900
O	1.78200	3.81900	19.75000
H	1.24600	3.36800	20.40200
H	1.19400	3.95300	19.00700
O	0.07700	20.58500	13.64400
H	0.31700	21.21700	14.32200
H	-0.76600	20.23400	13.93100
O	11.06000	11.73300	19.08200
H	10.17300	11.39500	19.20500
H	11.63000	11.00100	19.31900
O	-7.28700	8.06500	12.40600
H	-6.60000	7.75100	11.81900
H	-7.44200	8.96900	12.13300
O	-2.48400	2.95400	16.83500
H	-2.56800	2.34700	16.10000
H	-3.30500	3.44500	16.83600
O	4.59000	16.39300	16.04800
H	4.51700	17.19900	16.56000
H	4.14800	16.59200	15.22300
O	4.37900	15.63000	9.55900
H	3.59600	16.17200	9.65200
H	5.07800	16.14800	9.95800
O	7.68900	1.44000	12.20200
H	8.18600	0.68700	11.88200
H	6.91700	1.47600	11.63600
O	15.54200	7.14400	13.84400
H	15.53100	8.02600	13.47300
H	14.67300	6.79100	13.64900
O	7.05000	16.29200	18.20400
H	7.76300	16.92600	18.13200
H	6.55900	16.38800	17.38800
O	8.77200	10.19700	9.77600
H	7.83700	10.31700	9.60600
H	8.94900	9.29400	9.51200
O	12.15900	6.03900	10.31100
H	12.62500	6.87100	10.23700
H	12.61000	5.57300	11.01500
O	2.10100	14.04100	19.02500
H	2.80200	14.67500	19.17800
H	1.63100	14.38300	18.26400
O	10.22700	12.38300	7.30900
H	10.50200	12.26900	8.21900
H	9.35700	11.98500	7.27100
O	10.13100	-1.59300	14.35000
H	9.21700	-1.36300	14.51800
H	10.56000	-1.51000	15.20100
O	9.90100	6.59600	16.85000
H	9.24800	6.84300	16.19500

H	10.27900	5.78200	16.51600
O	6.86200	16.63200	10.95300
H	7.04400	15.81400	11.41500
H	7.68500	16.84800	10.51500
O	7.19600	15.55800	8.21400
H	7.72500	15.04800	7.60100
H	7.45900	15.24300	9.07900
O	5.95000	7.33800	23.33600
H	5.27400	7.23700	24.00700
H	6.77300	7.18200	23.80000
O	-2.30200	13.57500	15.95800
H	-1.71700	12.82900	16.09100
H	-2.50000	13.88500	16.84100
O	0.55200	16.61000	10.97400
H	-0.11700	17.09500	10.49000
H	0.41300	16.85700	11.88800
O	-7.71000	6.14500	14.67800
H	-7.67200	5.61200	15.47200
H	-8.21600	5.61900	14.05900
O	4.69200	18.64400	17.57200
H	5.24400	18.80700	18.33700
H	5.03300	19.23500	16.90000
O	-0.33100	0.99200	18.17400
H	-0.26700	1.85300	17.76200
H	-0.39500	0.37900	17.44200
O	-0.85600	3.06400	21.82200
H	-0.22900	2.68200	22.43600
H	-1.71300	2.84600	22.18700
O	9.54300	13.47000	23.42700
H	10.08700	14.08300	22.93300
H	8.96200	14.02800	23.94400
O	7.82900	16.59300	14.55900
H	7.70400	15.79100	14.05000
H	8.48700	17.08700	14.07000
O	-0.55000	2.94700	13.73600
H	-1.27100	3.56100	13.59700
H	0.23900	3.48300	13.65800
O	9.89300	14.39300	16.18700
H	8.94600	14.27400	16.11400
H	10.24100	13.50600	16.26900
O	2.25300	0.28300	20.01000
H	2.77600	0.52900	20.77300
H	2.03400	1.11500	19.59100
O	6.89700	14.18900	12.38700
H	6.75000	13.26000	12.56800
H	7.72300	14.21100	11.90300
O	10.50100	15.01200	13.47400
H	11.42500	14.80600	13.33600
H	10.33200	14.76300	14.38200
O	3.45800	1.22400	17.69500
H	3.11700	0.42500	18.09700
H	2.75200	1.52400	17.12300
O	4.10900	-1.69100	16.14800
H	4.93200	-2.16500	16.02600
H	4.35300	-0.91200	16.64800
O	1.63300	5.00100	13.95700
H	1.48800	5.31500	14.85000
H	1.15600	5.62000	13.40400
O	5.34600	2.42500	16.05100
H	5.08700	3.34700	16.04900
H	4.81400	2.02800	16.74100
O	10.29000	15.53000	8.83000
H	10.46400	14.96500	8.07700
H	10.78300	16.33000	8.64800
O	-3.89200	7.54200	16.89600
H	-4.43700	8.31800	17.02800
H	-3.01400	7.88900	16.73800
O	16.14400	8.29400	10.44900
H	16.58700	7.54400	10.05100
H	15.26700	8.28500	10.06600
O	11.27800	14.84700	5.92900
H	11.97300	15.46700	6.14800
H	11.62000	13.99600	6.20500
O	-3.10600	6.66900	19.78900
H	-3.84700	6.81700	20.37600
H	-3.42600	6.92900	18.92600
O	13.17700	12.29700	7.45800
H	13.70200	12.63500	6.73300
H	13.08900	11.36100	7.27400
O	4.83900	16.21000	19.58700
H	5.14800	15.96500	20.45900
H	5.63600	16.41500	19.09900
O	4.86100	18.36400	11.81800
H	4.42100	17.87200	12.51100
H	5.61200	17.82100	11.57700
O	-2.66000	14.55500	11.82000
H	-1.83400	14.25500	11.44000
H	-3.01000	13.78600	12.26900
O	2.30800	13.90800	13.89000
H	1.68600	13.24500	13.59000
H	2.97900	13.93400	13.20700
O	-4.45900	12.47400	12.77900
H	-5.29600	12.04600	12.59700
H	-4.48700	12.66700	13.71600
O	12.86600	12.93000	22.76300
H	12.07600	12.91200	22.22400
H	12.63000	13.46900	23.51800
O	1.87000	12.45800	7.36600
H	2.01800	13.14200	6.71200
H	2.67600	12.43900	7.88100
O	10.27600	5.76200	21.39200
H	11.17700	5.62300	21.10100
H	10.35100	5.92800	22.33200
O	4.61100	3.13900	20.23800
H	3.74100	3.43200	19.96600
H	4.84700	3.72300	20.95900
O	14.48800	12.19900	20.32100
H	14.19600	12.56200	21.15700
H	13.87500	11.48600	20.14200
O	15.68700	9.76400	12.84100
H	16.52200	10.20300	13.00500
H	15.82300	9.28800	12.02200
O	7.31500	-0.64300	17.75400
H	7.83300	0.09300	17.42900
H	6.96000	-0.33700	18.58900
O	7.56600	-4.22300	13.27400
H	7.84600	-4.89100	13.90000
H	8.35100	-4.03000	12.76200

O	5.36900	14.95300	4.83700
H	4.79700	15.57200	4.38400
H	5.94200	15.50300	5.37100
O	11.43700	4.19900	17.02200
H	12.38800	4.29900	17.04300
H	11.26700	3.40700	17.53100
O	9.45800	1.28400	17.20800
H	8.86300	1.56500	16.51200
H	9.17300	1.77400	17.97900
O	8.58900	15.23700	5.96800
H	9.52200	15.17100	6.17100
H	8.50100	16.07200	5.50900
O	11.21800	14.55300	2.92800
H	11.13900	14.21900	3.82200
H	12.03900	15.04500	2.92700
O	13.91500	7.41900	16.13600
H	13.88300	6.48100	16.32200
H	14.52600	7.49800	15.40200
O	14.06500	4.21100	16.18200
H	13.54900	3.42600	15.99500
H	14.94900	3.88200	16.34600
O	11.72600	-2.04500	12.14100
H	11.52800	-2.35400	13.02600
H	12.33600	-1.31900	12.27100
O	19.73300	8.48100	15.27600
H	20.46300	8.22000	15.83700
H	19.12400	7.74400	15.31700
O	17.81500	6.62200	15.87400
H	17.08800	6.48700	15.26500
H	17.39900	6.92900	16.68000
O	5.63800	10.34100	23.63900
H	6.55800	10.51900	23.83300
H	5.60300	9.39600	23.48700
O	-5.14400	5.53500	12.56400
H	-5.46400	4.68100	12.27100
H	-5.69100	5.75000	13.32000
O	-1.36600	8.25100	16.28100
H	-1.14700	9.18300	16.30000
H	-0.95400	7.89200	17.06600
O	-5.27500	9.15500	14.53300
H	-5.96900	8.74000	14.02000
H	-4.52800	9.19700	13.93700
O	10.86100	-0.05400	9.39900
H	10.28100	0.60400	9.01700
H	10.59500	-0.87700	8.98700
O	9.31200	-0.65400	11.60100
H	9.81200	-0.54200	10.79200
H	9.95500	-0.95800	12.24100
O	3.37000	7.25400	24.48800
H	2.88700	8.05400	24.69400
H	3.45600	7.26500	23.53400
O	-3.36200	9.08700	12.41000
H	-3.38500	9.18600	11.45800
H	-3.05200	9.93300	12.73200
O	-0.70300	11.14400	16.52400
H	0.25100	11.11300	16.45700
H	-0.90200	10.68300	17.34000
O	-0.11300	9.71500	19.09200
H	0.70600	10.20900	19.11500
H	0.16100	8.79800	19.06300
O	12.99500	2.13000	12.17000
H	13.77100	2.62000	12.44200
H	12.27000	2.55600	12.62700
O	15.43200	2.84300	13.22700
H	16.02700	2.60300	12.51700
H	15.50500	2.12400	13.85500
O	-2.34800	16.68600	13.54200
H	-2.48200	15.92600	12.97500
H	-2.76500	17.41000	13.07400
O	9.61800	7.62100	8.66900
H	9.56100	8.07800	7.83000
H	10.17100	6.86000	8.49200
O	13.42800	3.41000	9.49400
H	13.17200	3.01000	10.32500
H	12.80400	4.12400	9.36900
O	17.74000	6.15000	12.00200
H	17.18000	6.21900	12.77500
H	17.13400	5.97400	11.28100
O	0.80900	17.58300	13.75300
H	0.69200	18.52800	13.65100
H	-0.01200	17.28100	14.14200
O	10.69700	12.30800	4.59900
H	11.53100	11.87600	4.41700
H	10.53500	12.13700	5.52700
O	10.35100	8.81200	5.96500
H	10.29600	8.64600	5.02300
H	9.72600	9.51900	6.12100
O	-4.85900	4.04800	19.79100
H	-4.21400	3.53800	20.28000
H	-5.06800	4.78500	20.36500
O	7.66600	11.11600	25.29000
H	8.53500	11.29000	24.92900
H	7.18700	11.93600	25.16800
O	1.83500	18.23600	16.96900
H	2.73700	18.26800	17.28600
H	1.60800	17.30600	16.98400
O	3.59000	16.98900	13.63000
H	2.63900	16.98900	13.51600
H	3.89300	16.24200	13.11400
O	7.47600	-5.09300	9.79800
H	6.70900	-5.64800	9.94300
H	7.88700	-5.02100	10.65900
O	19.32500	22.82500	14.69500
H	19.96300	22.70600	13.99100
H	19.20700	23.77300	14.75300
O	16.13400	22.77800	14.63100
H	15.79600	22.66200	13.74300
H	17.00300	22.37700	14.61100
O	11.99300	24.63200	10.62000
H	11.42900	24.54100	9.85200
H	11.87800	23.81200	11.10100
O	16.61600	23.93100	20.58900
H	16.60500	23.53700	19.71700
H	16.77800	23.19800	21.18300
O	9.53900	26.12700	13.45900
H	10.33100	25.78600	13.04300
H	9.77900	26.23600	14.37900
O	17.57300	28.29900	5.93200

H	16.92200	28.88400	5.54500
H	18.36700	28.82900	5.99100
O	11.49800	25.38800	6.33100
H	12.27100	24.84000	6.19200
H	11.85100	26.25200	6.54200
O	13.33700	26.71500	19.53700
H	13.04600	27.62000	19.42200
H	13.97300	26.75700	20.25200
O	14.26200	24.09300	16.12700
H	14.85900	23.86800	15.41300
H	14.32000	23.35100	16.73000
O	13.83500	21.54700	3.96200
H	14.08800	21.02500	3.20100
H	12.89700	21.38300	4.06500
O	18.87900	20.55000	11.77000
H	19.66600	20.13200	11.41900
H	19.14300	21.45500	11.93800
O	19.05000	25.98800	6.46900
H	18.41500	26.06100	7.18100
H	18.72000	26.57700	5.79100
O	24.28000	20.48800	12.67300
H	25.01300	19.91800	12.90700
H	23.52400	20.10000	13.11400
O	13.55500	23.45000	6.16500
H	13.43800	22.77400	6.83300
H	13.66500	22.96300	5.34800
O	15.89800	22.91100	9.86900
H	15.53400	22.38400	10.58100
H	16.75400	22.52100	9.69600
O	21.80300	14.20000	17.13300
H	21.47100	15.07000	16.90900
H	21.20700	13.88400	17.81200
O	15.53800	17.94000	4.86600
H	15.70600	18.80800	5.23300
H	15.34800	18.10200	3.94200
O	18.29600	26.40100	16.53200
H	17.99600	27.30400	16.42400
H	17.95100	26.13500	17.38400
O	11.74700	22.85300	12.56600
H	10.82200	22.72000	12.77400
H	11.99600	23.63200	13.06300
O	21.69600	18.92000	14.21200
H	20.78700	18.85100	13.91800
H	21.64400	19.39700	15.04000
O	15.51700	20.39500	5.85000
H	15.39700	20.84100	6.68900
H	14.82200	20.74400	5.29200
O	9.10800	22.49600	13.40000
H	9.23700	22.75000	14.31400
H	8.42800	23.08900	13.08100
O	13.27800	33.16600	9.49900
H	13.20000	34.11700	9.57800
H	12.67400	32.82000	10.15700
O	15.77000	20.33900	22.15500
H	14.99300	20.89800	22.14600
H	16.40900	20.82400	22.67700
O	23.56200	17.12100	15.99000
H	22.91900	17.41500	15.34400
H	23.05700	17.00700	16.79500
O	21.21600	20.86800	6.67800
H	22.12800	21.10000	6.50300
H	20.70700	21.45100	6.11600
O	14.02400	23.14700	21.71700
H	14.76600	23.68600	21.44100
H	13.31800	23.77400	21.87400
O	19.50000	25.29000	20.31400
H	19.90800	24.84800	19.57000
H	18.77800	24.71500	20.56800
O	15.85300	20.22700	17.68800
H	16.69900	20.65100	17.83100
H	15.79300	19.57100	18.38200
O	19.82300	25.85000	14.07000
H	19.19400	26.01500	13.36700
H	19.43500	26.26400	14.84100
O	23.70200	26.78600	11.42400
H	23.01700	27.16500	10.87400
H	23.78600	27.39500	12.15700
O	19.00300	17.88800	19.34600
H	18.75900	18.64200	18.80900
H	19.27900	18.26600	20.18100
O	20.95700	24.43200	18.02400
H	21.16800	25.07500	17.34700
H	20.91000	23.59800	17.55600
O	18.55900	17.04300	14.92700
H	17.69800	17.34100	15.21900
H	18.58600	17.26000	13.99500
O	15.37500	25.58800	11.33500
H	15.83900	25.86600	10.54500
H	15.11100	24.68600	11.15200
O	12.26800	25.13500	14.27300
H	12.83800	25.56300	13.63400
H	12.85900	24.85300	14.97100
O	14.31200	21.71000	8.14400
H	15.01500	22.16200	8.61100
H	13.98000	21.06800	8.77200
O	12.76000	20.30100	22.51900
H	12.13000	20.98300	22.75200
H	12.65400	20.18900	21.57400
O	8.05500	19.96800	13.44500
H	7.26500	20.22400	12.96900
H	8.54600	20.78300	13.55100
O	22.07100	27.30900	16.19600
H	22.89300	26.90700	15.91400
H	22.06800	27.20600	17.14700
O	10.00600	20.33300	10.40400
H	9.20900	20.44500	10.92200
H	9.77900	20.67100	9.53800
O	15.83700	26.85700	18.35200
H	15.90100	27.31900	19.18800
H	15.12800	26.22700	18.48000
O	6.33900	20.22100	15.87700
H	6.58900	21.10600	16.14300
H	6.87500	20.04200	15.10400
O	30.16200	23.65300	12.54400
H	29.40800	23.08300	12.38900
H	30.29700	23.61900	13.49100
O	15.55200	28.52400	8.22000
H	15.41600	29.36900	8.64800

H	16.09300	28.72600	7.45600
O	12.03800	14.60300	24.88100
H	11.64500	14.05000	25.55600
H	12.85500	14.91400	25.27200
O	9.00500	26.04200	8.79300
H	9.51700	26.83600	8.94300
H	9.65700	25.34400	8.72600
O	10.81400	29.15800	10.87500
H	10.99100	30.08000	10.68700
H	11.61000	28.84400	11.30400
O	14.95500	16.01900	22.47300
H	15.69600	16.62000	22.39400
H	15.18700	15.27700	21.91500
O	10.09900	21.20500	20.75100
H	9.98500	20.30300	21.05200
H	9.24400	21.45100	20.40000
O	17.17900	29.14600	22.35000
H	16.73500	28.36000	22.03200
H	17.84000	29.33400	21.68400
O	11.93700	30.67600	16.87600
H	12.48800	30.54800	16.10400
H	11.09800	30.97500	16.52500
O	19.27700	29.42700	20.37800
H	19.25900	30.24800	19.88600
H	20.19900	29.30200	20.60300
O	15.73400	26.94100	21.22000
H	16.26700	26.18000	20.99000
H	15.20500	26.64800	21.96300
O	18.90100	12.04700	16.75000
H	18.55700	12.93700	16.67400
H	19.26900	11.85700	15.88700
O	6.97000	24.37000	9.56900
H	7.80700	24.78000	9.35200
H	7.14700	23.43000	9.53900
O	18.86600	22.03800	5.25700
H	18.35100	22.80800	5.49600
H	18.59400	21.36400	5.88000
O	16.45100	16.88300	10.31600
H	15.59500	16.49400	10.13600
H	16.86300	16.96200	9.45500
O	17.98100	14.41800	14.71500
H	17.47700	14.38100	13.90100
H	18.27400	15.32700	14.77400
O	14.22500	14.50100	13.72100
H	15.09000	14.30600	13.36200
H	14.29300	14.27200	14.64800
O	23.83900	24.79100	7.19200
H	23.06200	24.45200	6.74800
H	24.51900	24.79600	6.51800
O	18.65500	19.91100	7.08300
H	18.35000	20.22500	7.93400
H	19.60800	19.98900	7.12600
O	17.09900	26.07000	8.73200
H	16.57100	25.33500	8.42000
H	16.59800	26.84700	8.48600
O	22.07900	24.26500	13.84900
H	21.42100	24.95400	13.93700
H	22.88300	24.65400	14.19200
O	11.83100	16.73200	11.09300
H	12.40600	16.50900	10.36000
H	12.41800	16.80000	11.84600
O	20.88900	22.61900	11.89900
H	21.40200	23.25200	12.40200
H	21.53900	22.01900	11.53400
O	12.15800	18.76200	5.11300
H	11.32200	19.10000	5.43300
H	12.01900	18.62700	4.17500
O	14.13300	13.60600	9.82000
H	13.48000	13.29100	9.19600
H	14.09100	12.98300	10.54600
O	15.24500	23.88800	1.73600
H	14.33700	23.65700	1.93500
H	15.74200	23.09100	1.92300
O	18.15100	32.59700	4.65500
H	17.47800	32.90100	5.26400
H	18.53800	33.40000	4.30600
O	17.36700	20.09400	14.14500
H	16.44900	20.31500	13.98700
H	17.78300	20.16900	13.28600
O	18.15100	21.49300	9.27500
H	18.89800	22.00500	8.96600
H	18.42000	21.17100	10.13600
O	16.42300	23.98900	6.25800
H	15.51500	23.70500	6.35500
H	16.45300	24.40900	5.39800
O	21.65500	27.60000	10.02700
H	21.20200	26.75900	10.08400
H	21.02500	28.23600	10.36500
O	20.59900	18.52900	11.25700
H	19.86100	18.01700	11.58800
H	20.85100	18.08900	10.44600
O	20.09900	29.43700	10.96200
H	20.39100	29.99800	10.24300
H	20.60800	29.73000	11.71800
O	20.70000	11.30600	11.85200
H	21.00600	10.44600	11.56400
H	21.42500	11.89800	11.65100
O	6.99200	21.70900	10.24000
H	6.79500	21.11600	9.51500
H	6.43700	21.40500	10.95700
O	14.97200	21.60200	12.35900
H	15.02700	20.64900	12.28300
H	14.16100	21.75800	12.84300
O	16.12100	25.53000	3.59700
H	15.68300	24.81300	3.13800
H	16.86200	25.75700	3.03500
O	12.91100	20.21900	10.11800
H	12.09800	20.51900	10.52500
H	13.40700	19.82500	10.83600
O	15.06200	30.21500	12.05200
H	15.02000	30.44100	11.12200
H	15.97800	29.98200	12.20000
O	18.50900	20.68400	18.95000
H	18.86300	20.91100	19.80900
H	19.15600	21.01600	18.32700
O	24.23800	23.80600	9.65500
H	24.10000	23.99600	8.72700
H	23.88200	24.56700	10.11100

O	12.40300	17.79100	15.73400
H	13.04200	17.65000	15.03700
H	11.57000	17.50500	15.35800
O	24.26500	25.87300	14.66600
H	24.69900	25.78100	15.51400
H	24.97800	25.84900	14.02800
O	13.17900	27.09200	11.84800
H	13.97300	26.59700	11.64600
H	12.46800	26.56300	11.48500
O	17.99800	29.47300	16.19900
H	18.05200	30.04200	16.96700
H	17.13000	29.64400	15.83400
O	9.48700	21.23600	7.92400
H	8.55700	21.17400	8.14100
H	9.58300	20.71300	7.12700
O	9.40500	17.76100	10.51200
H	9.66600	18.67600	10.40300
H	10.22700	17.29400	10.66100
O	10.87800	19.17500	17.68100
H	11.55000	18.96700	17.03300
H	10.05100	18.97700	17.24200
O	25.12700	25.91600	17.19900
H	25.32000	26.82000	17.44500
H	25.02700	25.45600	18.03300
O	16.78000	15.80100	19.69800
H	16.13300	16.50000	19.60400
H	17.55100	16.23500	20.06600
O	20.18800	26.06600	22.77300
H	20.03700	25.83700	21.85600
H	20.17900	27.02300	22.78600
O	16.95700	23.63900	17.75800
H	17.88600	23.79100	17.58500
H	16.53600	23.72800	16.90300
O	15.75600	27.66400	15.52800
H	15.61700	27.29600	16.40000
H	14.96700	28.17900	15.35600
O	16.35000	32.76500	6.87000
H	16.52600	32.29400	7.68500
H	15.66400	33.39200	7.09800
O	17.30600	27.04300	12.91600
H	16.97100	27.00300	13.81200
H	16.80700	26.37700	12.44300
O	11.28100	22.44900	18.44200
H	11.14300	21.71700	19.04300
H	12.19100	22.36000	18.16000
O	20.24200	24.38800	3.19000
H	19.62500	23.66300	3.09300
H	19.77400	25.14900	2.84700
O	13.90900	22.16100	17.93000
H	14.61900	21.52100	17.88900
H	13.86600	22.41300	18.85300
O	21.89800	29.09800	20.85100
H	21.83900	28.41400	20.18400
H	22.29900	29.84000	20.39900
O	12.62200	19.14800	19.75000
H	12.08500	18.69700	20.40200
H	12.03300	19.28300	19.00700
O	10.91700	35.91400	13.64400
H	11.15600	36.54600	14.32200
H	10.07400	35.56300	13.93100
O	12.03200	18.60700	2.31200
H	11.15500	18.48600	1.94900
H	12.50800	17.81500	2.06000
O	21.89900	27.06200	19.08200
H	21.01200	26.72500	19.20500
H	22.46900	26.33000	19.31900
O	3.55200	23.39400	12.40600
H	4.23900	23.08100	11.81900
H	3.39700	24.29900	12.13300
O	8.35600	18.28400	16.83500
H	8.27100	17.67600	16.10000
H	7.53400	18.77400	16.83600
O	15.42900	31.72200	16.04800
H	15.35600	32.52800	16.56000
H	14.98700	31.92100	15.22300
O	15.21800	30.95900	9.55900
H	14.43500	31.50200	9.65200
H	15.91800	31.47700	9.95800
O	18.52800	16.76900	12.20200
H	19.02500	16.01600	11.88200
H	17.75700	16.80500	11.63600
O	26.38100	22.47300	13.84400
H	26.37000	23.35500	13.47300
H	25.51300	22.12000	13.64900
O	17.88900	31.62100	18.20400
H	18.60200	32.25600	18.13200
H	17.39800	31.71700	17.38800
O	19.61100	25.52600	9.77600
H	18.67700	25.64600	9.60600
H	19.78900	24.62300	9.51200
O	15.11200	18.91100	11.92900
H	14.74100	18.35800	12.61700
H	15.60700	18.30600	11.37700
O	22.99800	21.36800	10.31100
H	23.46400	22.20100	10.23700
H	23.44900	20.90200	11.01500
O	12.94000	29.37000	19.02500
H	13.64100	30.00400	19.17800
H	12.47000	29.71200	18.26400
O	21.06600	27.71200	7.30900
H	21.34200	27.59800	8.21900
H	20.19600	27.31400	7.27100
O	20.97000	13.73600	14.35000
H	20.05600	13.96600	14.51800
H	21.39900	13.82000	15.20100
O	20.74100	21.92500	16.85000
H	20.08800	22.17200	16.19500
H	21.11900	21.11100	16.51600
O	17.70100	31.96100	10.95300
H	17.88400	31.14300	11.41500
H	18.52500	32.17800	10.51500
O	18.03600	30.88800	8.21400
H	18.56400	30.37700	7.60100
H	18.29800	30.57200	9.07900
O	16.78900	22.66700	23.33600
H	16.11300	22.56600	24.00700
H	17.61200	22.51100	23.80000
O	8.53700	28.90400	15.95800

H	9.12300	28.15800	16.09100
H	8.34000	29.21400	16.84100
O	11.39100	31.93900	10.97400
H	10.72200	32.42400	10.49000
H	11.25200	32.18600	11.88800
O	3.12900	21.47500	14.67800
H	3.16700	20.94100	15.47200
H	2.62300	20.94800	14.05900
O	15.53200	33.97300	17.57200
H	16.08300	34.13700	18.33700
H	15.87200	34.56400	16.90000
O	10.50800	16.32100	18.17400
H	10.57200	17.18200	17.76200
H	10.44500	15.70800	17.44200
O	9.98300	18.39300	21.82200
H	10.61000	18.01100	22.43600
H	9.12600	18.17500	22.18700
O	20.38300	28.80000	23.42700
H	20.92700	29.41300	22.93300
H	19.80100	29.35800	23.94400
O	6.76000	20.20100	7.75400
H	7.05500	19.29200	7.70000
H	5.85900	20.18000	7.43200
O	18.66800	31.92200	14.55900
H	18.54400	31.12100	14.05000
H	19.32600	32.41600	14.07000
O	10.28900	18.27700	13.73600
H	9.56800	18.89000	13.59700
H	11.07900	18.81200	13.65800
O	20.73200	29.72300	16.18700
H	19.78500	29.60300	16.11400
H	21.08000	28.83500	16.26900
O	13.09200	15.61200	20.01000
H	13.61500	15.85800	20.77300
H	12.87300	16.44400	19.59100
O	8.48900	17.88200	7.87800
H	8.16000	17.00200	7.69500
H	8.84300	17.82700	8.76500
O	17.73700	29.51800	12.38700
H	17.58900	28.59000	12.56800
H	18.56200	29.54000	11.90300
O	11.60300	18.17000	8.24500
H	11.94200	18.69800	7.52300
H	12.05000	18.50800	9.02100
O	21.34000	30.34100	13.47400
H	22.26500	30.13500	13.33600
H	21.17200	30.09200	14.38200
O	14.29700	16.55300	17.69500
H	13.95700	15.75400	18.09700
H	13.59100	16.85400	17.12300
O	14.94900	13.63800	16.14800
H	15.77100	13.16400	16.02600
H	15.19300	14.41700	16.64800
O	13.59000	16.66700	6.44400
H	14.31000	17.04500	5.94100
H	12.96600	17.38600	6.54800
O	17.73300	17.37200	7.38000
H	17.94900	18.25400	7.07900
H	17.25200	16.98000	6.65100
O	12.47300	20.33000	13.95700
H	12.32700	20.64400	14.85000
H	11.99500	20.94900	13.40400
O	14.33200	17.32000	14.00500
H	14.31800	16.42500	13.66900
H	15.05000	17.32800	14.63900
O	16.18500	17.75400	16.05100
H	15.92700	18.67600	16.04900
H	15.65300	17.35700	16.74100
O	21.13000	30.85900	8.83000
H	21.30300	30.29400	8.07700
H	21.62200	31.65900	8.64800
O	6.94700	22.87100	16.89600
H	6.40300	23.64700	17.02800
H	7.82500	23.21800	16.73800
O	13.73600	16.20200	9.27400
H	13.85800	15.27400	9.47300
H	13.44600	16.21600	8.36200
O	16.60500	13.88700	12.41100
H	16.62700	13.95100	11.45600
H	17.02200	13.04800	12.60500
O	26.98300	23.62300	10.44900
H	27.42700	22.87400	10.05100
H	26.10600	23.61400	10.06600
O	22.11700	30.17600	5.92900
H	22.81200	30.79700	6.14800
H	22.45900	29.32600	6.20500
O	7.73400	21.99800	19.78900
H	6.99200	22.14600	20.37600
H	7.41300	22.25900	18.92600
O	24.01600	27.62600	7.45800
H	24.54100	27.96500	6.73300
H	23.92800	26.69100	7.27400
O	15.67800	31.53900	19.58700
H	15.98700	31.29400	20.45900
H	16.47600	31.74400	19.09900
O	15.70000	33.69300	11.81800
H	15.26100	33.20100	12.51100
H	16.45100	33.15000	11.57700
O	8.17900	29.88400	11.82000
H	9.00500	29.58500	11.44000
H	7.82900	29.11500	12.26900
O	13.14800	29.23700	13.89000
H	12.52600	28.57400	13.59000
H	13.81800	29.26300	13.20700
O	6.38000	27.80300	12.77900
H	5.54400	27.37500	12.59700
H	6.35300	27.99600	13.71600
O	13.22000	17.71500	23.48300
H	13.26900	18.59100	23.10100
H	13.76200	17.17300	22.91000
O	23.70600	28.25900	22.76300
H	22.91500	28.24200	22.22400
H	23.46900	28.79800	23.51800
O	23.82500	25.10800	20.07700
H	23.87500	24.94300	21.01800
H	23.71700	24.24100	19.68600
O	12.70900	27.78700	7.36600
H	12.85800	28.47100	6.71200

H	13.51600	27.76800	7.88100
O	21.11600	21.09200	21.39200
H	22.01700	20.95200	21.10100
H	21.19100	21.25700	22.33200
O	15.45000	18.46800	20.23800
H	14.58000	18.76200	19.96600
H	15.68600	19.05200	20.95900
O	25.32700	27.52800	20.32100
H	25.03600	27.89100	21.15700
H	24.71400	26.81500	20.14200
O	26.52700	25.09300	12.84100
H	27.36100	25.53200	13.00500
H	26.66300	24.61700	12.02200
O	23.57600	22.65100	18.78900
H	24.05100	22.84400	17.98100
H	22.65200	22.70700	18.54300
O	10.46400	23.81400	8.37200
H	10.21900	22.91000	8.57000
H	10.63400	23.81600	7.43000
O	18.15400	14.68600	17.75400
H	18.67200	15.42200	17.42900
H	17.79900	14.99300	18.58900
O	9.73400	19.54700	5.87000
H	9.04100	19.62100	5.21400
H	9.48900	18.78300	6.39300
O	18.40500	11.10600	13.27400
H	18.68500	10.43900	13.90000
H	19.19000	11.29900	12.76200
O	16.20900	30.28200	4.83700
H	15.63600	30.90100	4.38400
H	16.78200	30.83200	5.37100
O	22.27600	19.52800	17.02200
H	23.22800	19.62800	17.04300
H	22.10600	18.73600	17.53100
O	20.29800	16.61400	17.20800
H	19.70200	16.89400	16.51200
H	20.01200	17.10300	17.97900
O	19.42800	30.56600	5.96800
H	20.36100	30.50000	6.17100
H	19.34100	31.40100	5.50900
O	22.05700	29.88200	2.92800
H	21.97800	29.54800	3.82200
H	22.87800	30.37500	2.92700
O	24.75500	22.74900	16.13600
H	24.72300	21.81000	16.32200
H	25.36500	22.82700	15.40200
O	16.59300	21.61800	2.17900
H	17.53900	21.48900	2.24700
H	16.21500	20.88000	2.65500
O	24.90400	19.54000	16.18200
H	24.38800	18.75600	15.99500
H	25.78800	19.21200	16.34600
O	22.56500	13.28400	12.14100
H	22.36800	12.97500	13.02600
H	23.17500	14.01000	12.27100
O	30.57300	23.81000	15.27600
H	31.30300	23.54900	15.83700
H	29.96300	23.07300	15.31700
O	28.65400	21.95100	15.87400
H	27.92700	21.81700	15.26500
H	28.23900	22.25900	16.68000
O	16.47700	25.67000	23.63900
H	17.39700	25.84800	23.83300
H	16.44200	24.72600	23.48700
O	14.77700	19.54300	2.11400
H	13.87100	19.24700	2.19000
H	15.05500	19.24600	1.24700
O	5.69500	20.86400	12.56400
H	5.37600	20.01000	12.27100
H	5.14900	21.07900	13.32000
O	9.47300	23.58000	16.28100
H	9.69200	24.51200	16.30000
H	9.88600	23.22100	17.06600
O	16.39400	14.08700	8.26800
H	15.58400	13.74200	8.64400
H	17.08200	13.77800	8.85700
O	18.32300	13.16500	9.89100
H	18.87600	13.58400	10.55000
H	18.56000	12.23800	9.93300
O	14.34600	19.15300	25.67100
H	14.24600	20.10300	25.60200
H	14.12800	18.82500	24.79900
O	13.54700	16.40200	2.25500
H	14.40900	16.72700	2.51500
H	13.73200	15.61500	1.74300
O	5.56400	24.48400	14.53300
H	4.87000	24.07000	14.02000
H	6.31100	24.52600	13.93700
O	21.70100	15.27500	9.39900
H	21.12100	15.93300	9.01700
H	21.43400	14.45300	8.98700
O	20.15100	14.67500	11.60100
H	20.65100	14.78700	10.79200
H	20.79500	14.37100	12.24100
O	14.20900	22.58300	24.48800
H	13.72600	23.38300	24.69400
H	14.29500	22.59400	23.53400
O	7.47800	24.41600	12.41000
H	7.45500	24.51500	11.45800
H	7.78800	25.26200	12.73200
O	10.13700	26.47400	16.52400
H	11.09100	26.44200	16.45700
H	9.93800	26.01300	17.34000
O	10.72600	25.04400	19.09200
H	11.54600	25.53800	19.11500
H	11.00000	24.12700	19.06300
O	20.48300	17.43500	8.45400
H	19.59600	17.72000	8.23600
H	20.89900	17.27400	7.60700
O	23.83500	17.45900	12.17000
H	24.61100	17.94900	12.44200
H	23.10900	17.88500	12.62700
O	26.27200	18.17200	13.22700
H	26.86600	17.93200	12.51700
H	26.34400	17.45300	13.85500
O	8.49200	32.01600	13.54200
H	8.35700	31.25600	12.97500
H	8.07500	32.74000	13.07400

O	19.62700	27.19900	2.54900
H	20.33100	27.40400	3.16500
H	18.90400	27.76700	2.81400
O	20.45700	22.95000	8.66900
H	20.40100	23.40700	7.83000
H	21.01000	22.18900	8.49200
O	24.26800	18.73900	9.49400
H	24.01100	18.33900	10.32500
H	23.64300	19.45400	9.36900
O	28.57900	21.47900	12.00200
H	28.01900	21.54800	12.77500
H	27.97300	21.30300	11.28100
O	11.64800	32.91200	13.75300
H	11.53200	33.85700	13.65100
H	10.82800	32.61000	14.14200
O	21.53600	27.63700	4.59900
H	22.37100	27.20600	4.41700
H	21.37500	27.46600	5.52700
O	21.19100	24.14100	5.96500
H	21.13600	23.97500	5.02300
H	20.56500	24.84800	6.12100
O	5.98000	19.37800	19.79100
H	6.62600	18.86700	20.28000
H	5.77200	20.11400	20.36500
O	18.50500	26.44600	25.29000
H	19.37400	26.62000	24.92900
H	18.02600	27.26500	25.16800
O	14.50000	13.64200	5.54300
H	14.48800	14.58600	5.70000
H	15.39300	13.45900	5.24900
O	12.67400	33.56500	16.96900
H	13.57700	33.59800	17.28600
H	12.44800	32.63500	16.98400
O	14.42900	32.31800	13.63000
H	13.47900	32.31900	13.51600
H	14.73300	31.57200	13.11400
O	18.31600	10.23600	9.79800
H	17.54900	9.68200	9.94300
H	18.72700	10.30800	10.65900
O	10.32000	16.52400	23.88800
H	11.18300	16.16800	24.09900
H	9.74600	16.18600	24.57600
O	30.16500	38.15400	14.69500
H	30.80200	38.03500	13.99100
H	30.04700	39.10300	14.75300
O	26.97400	38.10700	14.63100
H	26.63500	37.99100	13.74300
H	27.84200	37.70600	14.61100
O	22.83200	39.96100	10.62000
H	22.26900	39.87000	9.85200
H	22.71700	39.14100	11.10100
O	20.37900	41.45600	13.45900
H	21.17100	41.11500	13.04300
H	20.61800	41.56500	14.37900
O	22.33800	40.71700	6.33100
H	23.11100	40.17000	6.19200
H	22.69000	41.58100	6.54200
O	25.10200	39.42200	16.12700
H	25.69800	39.19800	15.41300
H	25.15900	38.68100	16.73000
O	24.67400	36.87600	3.96200
H	24.92700	36.35400	3.20100
H	23.73700	36.71300	4.06500
O	29.71900	35.88000	11.77000
H	30.50500	35.46200	11.41900
H	29.98300	36.78400	11.93800
O	35.12000	35.81700	12.67300
H	35.85200	35.24700	12.90700
H	34.36300	35.42900	13.11400
O	24.39400	38.77900	6.16500
H	24.27800	38.10300	6.83300
H	24.50400	38.29200	5.34800
O	26.73700	38.24000	9.86900
H	26.37300	37.71300	10.58100
H	27.59400	37.85000	9.69600
O	32.64200	29.52900	17.13300
H	32.31000	30.39900	16.90900
H	32.04700	29.21300	17.81200
O	26.37800	33.26900	4.86600
H	26.54500	34.13700	5.23300
H	26.18700	33.43200	3.94200
O	22.58600	38.18200	12.56600
H	21.66200	38.05000	12.77400
H	22.83500	38.96100	13.06300
O	32.53500	34.24900	14.21200
H	31.62700	34.18000	13.91800
H	32.48400	34.72600	15.04000
O	26.35700	35.72500	5.85000
H	26.23700	36.17000	6.68900
H	25.66200	36.07300	5.29200
O	19.94700	37.82500	13.40000
H	20.07700	38.07900	14.31400
H	19.26700	38.41800	13.08100
O	34.40100	32.45000	15.99000
H	33.75900	32.74400	15.34400
H	33.89700	32.33600	16.79500
O	32.05500	36.19700	6.67800
H	32.96700	36.42900	6.50300
H	31.54600	36.78000	6.11600
O	26.69200	35.55600	17.68800
H	27.53800	35.98000	17.83100
H	26.63200	34.90000	18.38200
O	29.84200	33.21700	19.34600
H	29.59800	33.97100	18.80900
H	30.11800	33.59500	20.18100
O	29.39900	32.37300	14.92700
H	28.53700	32.67100	15.21900
H	29.42500	32.59000	13.99500
O	26.21500	40.91700	11.33500
H	26.67900	41.19600	10.54500
H	25.95100	40.01500	11.15200
O	23.10700	40.46400	14.27300
H	23.67700	40.89300	13.63400
H	23.69800	40.18200	14.97100
O	25.15200	37.03900	8.14400
H	25.85400	37.49100	8.61100
H	24.82000	36.39700	8.77200
O	18.89400	35.29700	13.44500

H	18.10400	35.55300	12.96900
H	19.38500	36.11200	13.55100
O	20.84600	35.66200	10.40400
H	20.04900	35.77400	10.92200
H	20.61800	36.00100	9.53800
O	17.17800	35.55000	15.87700
H	17.42900	36.43500	16.14300
H	17.71400	35.37100	15.10400
O	22.87800	29.93200	24.88100
H	22.48500	29.37900	25.55600
H	23.69400	30.24300	25.27200
O	19.84400	41.37100	8.79300
H	20.35700	42.16500	8.94300
H	20.49600	40.67300	8.72600
O	25.79500	31.34800	22.47300
H	26.53600	31.94900	22.39400
H	26.02600	30.60600	21.91500
O	20.93900	36.53400	20.75100
H	20.82400	35.63200	21.05200
H	20.08300	36.78000	20.40000
O	29.74000	27.37600	16.75000
H	29.39700	28.26600	16.67400
H	30.10800	27.18600	15.88700
O	17.80900	39.70000	9.56900
H	18.64600	40.10900	9.35200
H	17.98700	38.76000	9.53900
O	29.70500	37.36700	5.25700
H	29.19000	38.13800	5.49600
H	29.43300	36.69400	5.88000
O	27.29000	32.21200	10.31600
H	26.43400	31.82300	10.13600
H	27.70300	32.29100	9.45500
O	28.82000	29.74700	14.71500
H	28.31700	29.71000	13.90100
H	29.11300	30.65600	14.77400
O	25.06400	29.83100	13.72100
H	25.93000	29.63500	13.36200
H	25.13200	29.60200	14.64800
O	29.49400	35.24000	7.08300
H	29.18900	35.55400	7.93400
H	30.44700	35.31800	7.12600
O	22.67100	32.06100	11.09300
H	23.24500	31.83800	10.36000
H	23.25800	32.13000	11.84600
O	22.99800	34.09100	5.11300
H	22.16100	34.42900	5.43300
H	22.85800	33.95600	4.17500
O	24.97300	28.93500	9.82000
H	24.31900	28.62000	9.19600
H	24.93100	28.31300	10.54600
O	26.08400	39.21700	1.73600
H	25.17700	38.98600	1.93500
H	26.58100	38.42100	1.92300
O	28.20700	35.42300	14.14500
H	27.28900	35.64500	13.98700
H	28.62300	35.49800	13.28600
O	28.99000	36.82200	9.27500
H	29.73700	37.33400	8.96600
H	29.25900	36.50100	10.13600
O	27.26300	39.31800	6.25800
H	26.35400	39.03400	6.35500
H	27.29200	39.73800	5.39800
O	31.43800	33.85800	11.25700
H	30.70000	33.34600	11.58800
H	31.69100	33.41800	10.44600
O	31.53900	26.63600	11.85200
H	31.84500	25.77500	11.56400
H	32.26400	27.22700	11.65100
O	17.83200	37.03900	10.24000
H	17.63400	36.44600	9.51500
H	17.27600	36.73400	10.95700
O	25.81100	36.93100	12.35900
H	25.86600	35.97800	12.28300
H	25.00000	37.08700	12.84300
O	26.96000	40.85900	3.59700
H	26.52200	40.14300	3.13800
H	27.70100	41.08700	3.03500
O	23.75000	35.54900	10.11800
H	22.93700	35.84800	10.52500
H	24.24600	35.15400	10.83600
O	23.24200	33.12100	15.73400
H	23.88200	32.97900	15.03700
H	22.41000	32.83400	15.35800
O	24.01800	42.42100	11.84800
H	24.81200	41.92600	11.64600
H	23.30700	41.89300	11.48500
O	20.32600	36.56500	7.92400
H	19.39600	36.50400	8.14100
H	20.42200	36.04300	7.12700
O	20.24500	33.09100	10.51200
H	20.50500	34.00500	10.40300
H	21.06600	32.62300	10.66100
O	21.71700	34.50400	17.68100
H	22.39000	34.29600	17.03300
H	20.89000	34.30700	17.24200
O	27.62000	31.13100	19.69800
H	26.97300	31.83000	19.60400
H	28.39000	31.56400	20.06600
O	27.79600	38.96900	17.75800
H	28.72500	39.12000	17.58500
H	27.37500	39.05700	16.90300
O	22.12000	37.77900	18.44200
H	21.98200	37.04700	19.04300
H	23.03000	37.68900	18.16000
O	24.74800	37.49100	17.93000
H	25.45800	36.85000	17.88900
H	24.70500	37.74200	18.85300
O	23.46100	34.47700	19.75000
H	22.92500	34.02600	20.40200
H	22.87300	34.61200	19.00700
O	22.87100	33.93600	2.31200
H	21.99400	33.81500	1.94900
H	23.34700	33.14500	2.06000
O	14.39200	38.72400	12.40600
H	15.07900	38.41000	11.81900
H	14.23600	39.62800	12.13300
O	19.19500	33.61300	16.83500
H	19.11100	33.00500	16.10000

H	18.37300	34.10400	16.83600
O	29.36700	32.09800	12.20200
H	29.86400	31.34500	11.88200
H	28.59600	32.13400	11.63600
O	25.95100	34.24000	11.92900
H	25.58100	33.68700	12.61700
H	26.44700	33.63600	11.37700
O	31.80900	29.06500	14.35000
H	30.89600	29.29500	14.51800
H	32.23900	29.14900	15.20100
O	31.58000	37.25400	16.85000
H	30.92700	37.50200	16.19500
H	31.95800	36.44000	16.51600
O	19.37700	44.23300	15.95800
H	19.96200	43.48800	16.09100
H	19.17900	44.54400	16.84100
O	13.96800	36.80400	14.67800
H	14.00700	36.27000	15.47200
H	13.46200	36.27700	14.05900
O	21.34700	31.65000	18.17400
H	21.41200	32.51200	17.76200
H	21.28400	31.03800	17.44200
O	20.82300	33.72300	21.82200
H	21.45000	33.34000	22.43600
H	19.96500	33.50400	22.18700
O	17.59900	35.53000	7.75400
H	17.89500	34.62100	7.70000
H	16.69800	35.50900	7.43200
O	21.12900	33.60600	13.73600
H	20.40700	34.21900	13.59700
H	21.91800	34.14200	13.65800
O	23.93100	30.94100	20.01000
H	24.45500	31.18700	20.77300
H	23.71200	31.77300	19.59100
O	19.32800	33.21200	7.87800
H	18.99900	32.33100	7.69500
H	19.68200	33.15600	8.76500
O	22.44200	33.49900	8.24500
H	22.78200	34.02700	7.52300
H	22.88900	33.83700	9.02100
O	25.13700	31.88300	17.69500
H	24.79600	31.08300	18.09700
H	24.43000	32.18300	17.12300
O	25.78800	28.96800	16.14800
H	26.61100	28.49400	16.02600
H	26.03200	29.74700	16.64800
O	24.42900	31.99600	6.44400
H	25.15000	32.37400	5.94100
H	23.80600	32.71500	6.54800
O	28.57200	32.70100	7.38000
H	28.78800	33.58300	7.07900
H	28.09200	32.30900	6.65100
O	23.31200	35.65900	13.95700
H	23.16600	35.97300	14.85000
H	22.83500	36.27800	13.40400
O	25.17200	32.65000	14.00500
H	25.15800	31.75400	13.66900
H	25.88900	32.65700	14.63900
O	27.02400	33.08300	16.05100
H	26.76600	34.00500	16.04900
H	26.49300	32.68600	16.74100
O	17.78700	38.20000	16.89600
H	17.24200	38.97600	17.02800
H	18.66500	38.54700	16.73800
O	24.57500	31.53100	9.27400
H	24.69700	30.60300	9.47300
H	24.28600	31.54500	8.36200
O	27.44400	29.21700	12.41100
H	27.46700	29.28100	11.45600
H	27.86100	28.37700	12.60500
O	18.57300	37.32700	19.78900
H	17.83100	37.47500	20.37600
H	18.25200	37.58800	18.92600
O	19.01900	45.21300	11.82000
H	19.84500	44.91400	11.44000
H	18.66800	44.44400	12.26900
O	17.22000	43.13200	12.77900
H	16.38300	42.70400	12.59700
H	17.19200	43.32500	13.71600
O	24.05900	33.04400	23.48300
H	24.10800	33.92100	23.10100
H	24.60200	32.50200	22.91000
O	23.54800	43.11700	7.36600
H	23.69700	43.80000	6.71200
H	24.35500	43.09700	7.88100
O	26.28900	33.79700	20.23800
H	25.42000	34.09100	19.96600
H	26.52500	34.38100	20.95900
O	21.30400	39.14300	8.37200
H	21.05800	38.23900	8.57000
H	21.47300	39.14500	7.43000
O	28.99300	30.01500	17.75400
H	29.51100	30.75200	17.42900
H	28.63900	30.32200	18.58900
O	20.57300	34.87600	5.87000
H	19.88000	34.95000	5.21400
H	20.32800	34.11200	6.39300
O	29.24500	26.43500	13.27400
H	29.52500	25.76800	13.90000
H	30.03000	26.62800	12.76200
O	33.11500	34.85700	17.02200
H	34.06700	34.95700	17.04300
H	32.94600	34.06500	17.53100
O	31.13700	31.94300	17.20800
H	30.54200	32.22300	16.51200
H	30.85200	32.43200	17.97900
O	27.43300	36.94700	2.17900
H	28.37800	36.81800	2.24700
H	27.05400	36.20900	2.65500
O	35.74400	34.86900	16.18200
H	35.22800	34.08500	15.99500
H	36.62800	34.54100	16.34600
O	33.40500	28.61300	12.14100
H	33.20700	28.30500	13.02600
H	34.01500	29.33900	12.27100
O	25.61700	34.87200	2.11400
H	24.71000	34.57600	2.19000
H	25.89400	34.57500	1.24700

O	16.53500	36.19300	12.56400
H	16.21500	35.34000	12.27100
H	15.98800	36.40800	13.32000
O	20.31300	38.90900	16.28100
H	20.53100	39.84100	16.30000
H	20.72500	38.55000	17.06600
O	27.23300	29.41600	8.26800
H	26.42300	29.07100	8.64400
H	27.92200	29.10700	8.85700
O	29.16200	28.49400	9.89100
H	29.71500	28.91300	10.55000
H	29.39900	27.56700	9.93300
O	24.38700	31.73100	2.25500
H	25.24900	32.05700	2.51500
H	24.57200	30.94400	1.74300
O	16.40300	39.81300	14.53300
H	15.71000	39.39900	14.02000
H	17.15100	39.85500	13.93700
O	32.54000	30.60400	9.39900
H	31.96000	31.26200	9.01700
H	32.27400	29.78200	8.98700
O	30.99000	30.00400	11.60100
H	31.49000	30.11600	10.79200
H	31.63400	29.70100	12.24100
O	18.31700	39.74500	12.41000
H	18.29400	39.84400	11.45800
H	18.62700	40.59200	12.73200
O	20.97600	41.80300	16.52400
H	21.93000	41.77100	16.45700
H	20.77700	41.34200	17.34000
O	21.56600	40.37300	19.09200
H	22.38500	40.86700	19.11500
H	21.84000	39.45600	19.06300
O	31.32300	32.76400	8.45400
H	30.43500	33.04900	8.23600
H	31.73800	32.60300	7.60700
O	34.67400	32.78900	12.17000
H	35.45000	33.27800	12.44200
H	33.94900	33.21400	12.62700
O	37.11100	33.50100	13.22700
H	37.70600	33.26100	12.51700
H	37.18400	32.78200	13.85500
O	35.10700	34.06800	9.49400
H	34.85100	33.66800	10.32500
H	34.48200	34.78300	9.36900
O	16.81900	34.70700	19.79100
H	17.46500	34.19700	20.28000
H	16.61100	35.44400	20.36500
O	25.34000	28.97100	5.54300
H	25.32800	29.91500	5.70000
H	26.23200	28.78800	5.24900
O	29.15500	25.56500	9.79800
H	28.38800	25.01100	9.94300
H	29.56600	25.63700	10.65900
O	21.15900	31.85300	23.88800
H	22.02200	31.49700	24.09900
H	20.58600	31.51500	24.57600
O	-13.19300	-46.15600	1.41900
H	-12.55500	-46.27500	0.71500
H	-13.31100	-45.20800	1.47800
O	-16.38400	-46.20300	1.35500
H	-16.72200	-46.31900	0.46700
H	-15.51500	-46.60400	1.33500
O	-20.52500	-44.35000	-2.65500
H	-21.08900	-44.44100	-3.42300
H	-20.64000	-45.17000	-2.17500
O	-15.90200	-45.05000	7.31400
H	-15.91300	-45.44500	6.44200
H	-15.74000	-45.78300	7.90700
O	-22.97900	-42.85500	0.18300
H	-22.18700	-43.19600	-0.23200
H	-22.73900	-42.74500	1.10400
O	-14.94500	-40.68300	-7.34400
H	-15.59700	-40.09700	-7.73000
H	-14.15100	-40.15200	-7.28400
O	-21.02000	-43.59400	-6.94500
H	-20.24700	-44.14100	-7.08400
H	-20.66700	-42.72900	-6.73300
O	-19.18100	-42.26600	6.26200
H	-19.47200	-41.36200	6.14600
H	-18.54600	-42.22500	6.97600
O	-18.25600	-44.88800	2.85200
H	-17.65900	-45.11300	2.13800
H	-18.19800	-45.63000	3.45400
O	-13.63900	-48.43100	-1.50600
H	-12.85300	-48.84900	-1.85700
H	-13.37500	-47.52600	-1.33700
O	-13.46800	-42.99400	-6.80700
H	-14.10300	-42.92000	-6.09500
H	-13.79800	-42.40400	-7.48500
O	-18.96400	-45.53200	-7.11100
H	-19.08000	-46.20700	-6.44300
H	-18.85300	-46.01800	-7.92700
O	-16.62000	-46.07100	-3.40600
H	-16.98500	-46.59800	-2.69500
H	-15.76400	-46.46100	-3.57900
O	-14.22200	-42.58000	3.25700
H	-14.52200	-41.67800	3.14800
H	-14.56700	-42.84700	4.10900
O	-19.24000	-35.81600	-3.77600
H	-19.31800	-34.86500	-3.69700
H	-19.84400	-36.16100	-3.11900
O	-16.74800	-48.64300	8.88000
H	-17.52500	-48.08300	8.87000
H	-16.10900	-48.15700	9.40100
O	-11.30200	-48.11400	-6.59700
H	-10.39100	-47.88100	-6.77200
H	-11.81100	-47.53000	-7.16000
O	-18.49400	-45.83400	8.44200
H	-17.75200	-45.29600	8.16500
H	-19.20000	-45.20800	8.59900
O	-13.01800	-43.69100	7.03900
H	-12.61000	-44.13300	6.29400
H	-13.74100	-44.26600	7.29200
O	-12.69500	-43.13100	0.79400
H	-13.32500	-42.96700	0.09200
H	-13.08300	-42.71700	1.56500
O	-8.81700	-42.19600	-1.85100

H	-9.50100	-41.81600	-2.40200
H	-8.73200	-41.58600	-1.11800
O	-11.56100	-44.55000	4.74800
H	-11.35000	-43.90700	4.07200
H	-11.60800	-45.38400	4.28000
O	-17.14300	-43.39400	-1.94100
H	-16.67900	-43.11500	-2.73000
H	-17.40700	-44.29500	-2.12300
O	-20.25000	-43.84700	0.99700
H	-19.68000	-43.41800	0.35900
H	-19.65900	-44.12800	1.69600
O	-10.44700	-41.67300	2.92000
H	-9.62500	-42.07500	2.63900
H	-10.45100	-41.77600	3.87200
O	-16.68100	-42.12400	5.07600
H	-16.61700	-41.66200	5.91200
H	-17.39000	-42.75400	5.20500
O	-2.35600	-45.32900	-0.73100
H	-3.11000	-45.89800	-0.88700
H	-2.22100	-45.36200	0.21600
O	-16.96600	-40.45700	-5.05600
H	-17.10200	-39.61200	-4.62700
H	-16.42500	-40.25500	-5.81900
O	-23.51300	-42.94000	-4.48300
H	-23.00100	-42.14500	-4.33200
H	-22.86100	-43.63700	-4.54900
O	-21.70400	-39.82300	-2.40100
H	-21.52700	-38.90100	-2.58800
H	-20.90800	-40.13700	-1.97100
O	-15.34000	-39.83500	9.07400
H	-15.78300	-40.62100	8.75700
H	-14.67800	-39.64700	8.40900
O	-20.58100	-38.30500	3.60000
H	-20.03000	-38.43400	2.82800
H	-21.42000	-38.00600	3.25000
O	-13.24100	-39.55500	7.10300
H	-13.26000	-38.73400	6.61100
H	-12.31900	-39.67900	7.32800
O	-16.78400	-42.04000	7.94500
H	-16.25100	-42.80100	7.71500
H	-17.31300	-42.33300	8.68700
O	-13.65200	-46.94300	-8.01800
H	-14.16700	-46.17300	-7.78000
H	-13.92400	-47.61700	-7.39500
O	-8.67900	-44.19000	-6.08300
H	-9.45600	-44.52900	-6.52800
H	-8.00000	-44.18500	-6.75800
O	-13.86300	-49.07100	-6.19300
H	-14.16800	-48.75600	-5.34100
H	-12.91000	-48.99300	-6.15000
O	-15.41900	-42.91200	-4.54300
H	-15.94700	-43.64600	-4.85600
H	-15.92000	-42.13400	-4.78900
O	-10.44000	-44.71700	0.57300
H	-11.09700	-44.02700	0.66200
H	-9.63600	-44.32700	0.91700
O	-11.62900	-46.36200	-1.37600
H	-11.11600	-45.72900	-0.87400
H	-10.97900	-46.96200	-1.74200
O	-17.27400	-45.09400	-11.54000
H	-18.18100	-45.32500	-11.34100
H	-16.77600	-45.89000	-11.35200
O	-14.36700	-36.38400	-8.62000
H	-15.04100	-36.08100	-8.01200
H	-13.98000	-35.58100	-8.97000
O	-15.15100	-48.88700	0.87000
H	-16.06900	-48.66600	0.71200
H	-14.73500	-48.81300	0.01100
O	-14.36700	-47.48800	-4.00000
H	-13.62000	-46.97600	-4.30900
H	-14.09800	-47.81000	-3.14000
O	-16.09500	-44.99200	-7.01700
H	-17.00400	-45.27700	-6.92000
H	-16.06500	-44.57300	-7.87700
O	-10.86300	-41.38200	-3.24800
H	-11.31700	-42.22300	-3.19200
H	-11.49300	-40.74600	-2.91100
O	-12.42000	-39.54400	-2.31300
H	-12.12700	-38.98400	-3.03200
H	-11.91000	-39.25100	-1.55800
O	-17.54600	-47.38000	-0.91600
H	-17.49100	-48.33200	-0.99200
H	-18.35700	-47.22400	-0.43200
O	-16.39700	-43.45200	-9.67900
H	-16.83500	-44.16800	-10.13800
H	-15.65600	-43.22400	-10.24000
O	-17.45600	-38.76600	-1.22400
H	-17.49900	-38.54000	-2.15300
H	-16.54000	-39.00000	-1.07500
O	-14.01000	-48.29800	5.67400
H	-13.65500	-48.07100	6.53400
H	-13.36200	-47.96600	5.05200
O	-8.28000	-45.17600	-3.62100
H	-8.41800	-44.98600	-4.54900
H	-8.63600	-44.41400	-3.16400
O	-8.25300	-43.10800	1.39000
H	-7.81900	-43.20100	2.23900
H	-7.54000	-43.13200	0.75200
O	-19.34000	-41.89000	-1.42800
H	-18.54600	-42.38500	-1.62900
H	-20.05000	-42.41800	-1.79100
O	-14.52000	-39.50800	2.92400
H	-14.46600	-38.93900	3.69100
H	-15.38900	-39.33800	2.55900
O	-7.39100	-43.06600	3.92400
H	-7.19800	-42.16100	4.16900
H	-7.49100	-43.52500	4.75700
O	-12.33000	-42.91500	9.49800
H	-12.48100	-43.14400	8.58100
H	-12.33900	-41.95800	9.51100
O	-15.56100	-45.34200	4.48200
H	-14.63200	-45.19100	4.30900
H	-15.98200	-45.25400	3.62700
O	-16.76200	-41.31700	2.25200
H	-16.90100	-41.68500	3.12500
H	-17.55100	-40.80200	2.08100
O	-16.16800	-36.21600	-6.40500
H	-15.99200	-36.68700	-5.59100

H	-16.85400	-35.58900	-6.17700
O	-15.21200	-41.93900	-0.35900
H	-15.54700	-41.97900	0.53700
H	-15.71200	-42.60400	-0.83200
O	-12.27600	-44.59300	-10.08500
H	-12.89300	-45.31800	-10.18200
H	-12.74400	-43.83200	-10.42900
O	-18.60900	-46.82000	4.65500
H	-17.89900	-47.46100	4.61300
H	-18.65200	-46.56900	5.57700
O	-10.62100	-39.88300	7.57500
H	-10.67900	-40.56700	6.90800
H	-10.21900	-39.14100	7.12300
O	-21.60200	-33.06700	0.36900
H	-21.36200	-32.43500	1.04700
H	-22.44500	-33.41800	0.65500
O	-10.61900	-41.91900	5.80700
H	-11.50600	-42.25700	5.92900
H	-10.04900	-42.65100	6.04400
O	-17.08900	-37.25900	2.77300
H	-17.16200	-36.45400	3.28500
H	-17.53100	-37.06000	1.94800
O	-17.30000	-38.02200	-3.71700
H	-18.08300	-37.48000	-3.62300
H	-16.60000	-37.50500	-3.31700
O	-6.13700	-46.50800	0.56800
H	-6.14800	-45.62600	0.19800
H	-7.00500	-46.86100	0.37400
O	-14.62900	-37.36000	4.92900
H	-13.91600	-36.72600	4.85600
H	-15.12000	-37.26400	4.11300
O	-12.90700	-43.45500	-3.50000
H	-13.84100	-43.33500	-3.66900
H	-12.72900	-44.35800	-3.76400
O	-9.52000	-47.61400	-2.96500
H	-9.05400	-46.78100	-3.03900
H	-9.06900	-48.07900	-2.26000
O	-19.57800	-39.61100	5.74900
H	-18.87700	-38.97700	5.90200
H	-20.04800	-39.27000	4.98900
O	-11.45200	-41.26900	-5.96600
H	-11.17600	-41.38400	-5.05700
H	-12.32200	-41.66700	-6.00500
O	-11.77800	-47.05700	3.57400
H	-12.43000	-46.80900	2.92000
H	-11.40000	-47.87000	3.24100
O	-14.81700	-37.02100	-2.32300
H	-14.63500	-37.83800	-1.86000
H	-13.99400	-36.80400	-2.76000
O	-14.48300	-38.09400	-5.06100
H	-13.95400	-38.60500	-5.67400
H	-14.22000	-38.40900	-4.19700
O	-15.72900	-46.31500	10.06100
H	-16.40500	-46.41500	10.73100
H	-14.90600	-46.47100	10.52500
O	-23.98100	-40.07700	2.68200
H	-23.39600	-40.82300	2.81500
H	-24.17800	-39.76700	3.56600
O	-21.12700	-37.04200	-2.30100
H	-21.79600	-36.55800	-2.78500
H	-21.26600	-36.79500	-1.38700
O	-16.98700	-35.00800	4.29700
H	-16.43500	-34.84500	5.06200
H	-16.64600	-34.41700	3.62500
O	-12.13500	-40.18200	10.15200
H	-11.59100	-39.56900	9.65700
H	-12.71700	-39.62400	10.66800
O	-13.85000	-37.06000	1.28300
H	-13.97500	-37.86100	0.77400
H	-13.19200	-36.56500	0.79500
O	-11.78600	-39.25900	2.91100
H	-12.73300	-39.37800	2.83900
H	-11.43800	-40.14700	2.99400
O	-14.78200	-39.46300	-0.88800
H	-14.92900	-40.39200	-0.70800
H	-13.95600	-39.44100	-1.37300
O	-11.17800	-38.64100	0.19800
H	-10.25300	-38.84600	0.06000
H	-11.34700	-38.88900	1.10700
O	-11.38900	-38.12200	-4.44600
H	-11.21500	-38.68700	-5.19800
H	-10.89600	-37.32200	-4.62800
O	-5.53500	-45.35800	-2.82700
H	-5.09200	-46.10800	-3.22500
H	-6.41200	-45.36700	-3.20900
O	-10.40100	-38.80500	-7.34600
H	-9.70600	-38.18500	-7.12700
H	-10.05900	-39.65600	-7.07100
O	-8.50200	-41.35500	-5.81700
H	-7.97700	-41.01700	-6.54200
H	-8.59000	-42.29100	-6.00100
O	-16.84000	-37.44200	6.31200
H	-16.53100	-37.68700	7.18400
H	-16.04200	-37.23700	5.82400
O	-16.81800	-35.28800	-1.45800
H	-17.25700	-35.78100	-0.76500
H	-16.06700	-35.83100	-1.69900
O	-24.33900	-39.09700	-1.45500
H	-23.51300	-39.39700	-1.83500
H	-24.68900	-39.86600	-1.00600
O	-19.37000	-39.74400	0.61400
H	-19.99200	-40.40700	0.31500
H	-18.70000	-39.71800	-0.06800
O	-26.13800	-41.17800	-0.49600
H	-26.97500	-41.60600	-0.67800
H	-26.16500	-40.98500	0.44100
O	-8.81300	-40.72200	9.48800
H	-9.60300	-40.74000	8.94800
H	-9.04900	-40.18300	10.24300
O	-8.69300	-43.87300	6.80100
H	-8.64300	-44.03800	7.74300
H	-8.80100	-44.74000	6.41100
O	-19.80900	-41.19400	-5.91000
H	-19.66000	-40.51000	-6.56300
H	-19.00200	-41.21400	-5.39500
O	-11.40200	-47.89000	8.11700
H	-10.50100	-48.02900	7.82500
H	-11.32800	-47.72400	9.05700

O	-7.19100	-41.45300	7.04500
H	-7.48200	-41.09000	7.88200
H	-7.80400	-42.16600	6.86700
O	-5.99100	-43.88800	-0.43400
H	-5.15700	-43.44900	-0.27100
H	-5.85500	-44.36400	-1.25400
O	-8.94200	-46.33000	5.51300
H	-8.46700	-46.13700	4.70500
H	-9.86600	-46.27400	5.26800
O	-16.31000	-38.69900	-8.43900
H	-16.88200	-38.08000	-8.89200
H	-15.73600	-38.14900	-7.90500
O	-13.09000	-38.41600	-7.30800
H	-12.15700	-38.48100	-7.10400
H	-13.17800	-37.58000	-7.76600
O	-10.46100	-39.09900	-10.34700
H	-10.54000	-39.43300	-9.45400
H	-9.64000	-38.60700	-10.34800
O	-7.76300	-46.23300	2.86000
H	-7.79600	-47.17100	3.04600
H	-7.15300	-46.15400	2.12700
O	-15.92500	-47.36300	-11.09700
H	-14.97900	-47.49300	-11.02800
H	-16.30300	-48.10200	-10.62000
O	-1.94600	-45.17100	2.00100
H	-1.21500	-45.43300	2.56200
H	-2.55500	-45.90800	2.04100
O	-16.04100	-43.31100	10.36300
H	-15.12100	-43.13400	10.55700
H	-16.07600	-44.25600	10.21100
O	-18.30900	-46.39800	11.21200
H	-18.79200	-45.59800	11.41900
H	-18.22300	-46.38700	10.25900
O	-22.38200	-42.50800	3.24900
H	-21.42700	-42.53900	3.18200
H	-22.58000	-42.96900	4.06400
O	-21.79200	-43.93800	5.81600
H	-20.97200	-43.44300	5.84000
H	-21.51800	-44.85400	5.78800
O	-24.02700	-36.96600	0.26600
H	-24.16100	-37.72600	-0.30000
H	-24.44300	-36.24200	-0.20100
O	-12.89100	-41.78200	-10.72600
H	-12.18700	-41.57700	-10.11100
H	-13.61400	-41.21400	-10.46100
O	-12.06100	-46.03100	-4.60600
H	-12.11700	-45.57400	-5.44500
H	-11.50800	-46.79200	-4.78400
O	-20.87000	-36.06900	0.47800
H	-20.98600	-35.12400	0.37600
H	-21.69000	-36.37200	0.86600
O	-10.98200	-41.34400	-8.67600
H	-10.14700	-41.77600	-8.85900
H	-11.14400	-41.51600	-7.74900
O	-11.32700	-44.84100	-7.31100
H	-11.38300	-45.00600	-8.25200
H	-11.95300	-44.13300	-7.15500
O	-14.01300	-42.53600	12.01500
H	-13.14400	-42.36200	11.65300
H	-14.49200	-41.71600	11.89200
O	-19.84400	-35.41600	3.69300
H	-18.94100	-35.38400	4.01000
H	-20.07000	-36.34600	3.70800
O	-18.08900	-36.66300	0.35400
H	-19.03900	-36.66300	0.24100
H	-17.78600	-37.41000	-0.16200
O	-2.35400	-30.82700	1.41900
H	-1.71600	-30.94600	0.71500
H	-2.47100	-29.87900	1.47800
O	-5.54500	-30.87400	1.35500
H	-5.88300	-30.99000	0.46700
H	-4.67600	-31.27500	1.33500
O	-9.68600	-29.02100	-2.65500
H	-10.24900	-29.11200	-3.42300
H	-9.80100	-29.84100	-2.17500
O	-5.06300	-29.72100	7.31400
H	-5.07400	-30.11500	6.44200
H	-4.90100	-30.45400	7.90700
O	-12.13900	-27.52500	0.18300
H	-11.34700	-27.86600	-0.23200
H	-11.90000	-27.41600	1.10400
O	-4.10600	-25.35400	-7.34400
H	-4.75700	-24.76800	-7.73000
H	-3.31200	-24.82300	-7.28400
O	-10.18000	-28.26500	-6.94500
H	-9.40700	-28.81200	-7.08400
H	-9.82800	-27.40000	-6.73300
O	-8.34200	-26.93700	6.26200
H	-8.63300	-26.03200	6.14600
H	-7.70600	-26.89500	6.97600
O	-7.41600	-29.55900	2.85200
H	-6.82000	-29.78400	2.13800
H	-7.35900	-30.30100	3.45400
O	-7.84400	-32.10500	-9.31300
H	-7.59100	-32.62700	-10.07500
H	-8.78100	-32.26900	-9.21000
O	-2.79900	-33.10200	-1.50600
H	-2.01300	-33.52000	-1.85700
H	-2.53600	-32.19700	-1.33700
O	-2.62800	-27.66400	-6.80700
H	-3.26400	-27.59100	-6.09500
H	-2.95900	-27.07500	-7.48500
O	2.60100	-33.16400	-0.60200
H	3.33400	-33.73400	-0.36900
H	1.84500	-33.55200	-0.16200
O	-8.12400	-30.20300	-7.11100
H	-8.24100	-30.87800	-6.44300
H	-8.01400	-30.68900	-7.92700
O	-5.78100	-30.74200	-3.40600
H	-6.14500	-31.26800	-2.69500
H	-4.92400	-31.13200	-3.57900
O	0.12400	-39.45200	3.85700
H	-0.20800	-38.58300	3.63300
H	-0.47200	-39.76800	4.53700
O	-6.14000	-35.71300	-8.41000
H	-5.97300	-34.84400	-8.04300
H	-6.33100	-35.55000	-9.33400
O	-3.38300	-27.25100	3.25700

H	-3.68300	-26.34800	3.14800
H	-3.72800	-27.51800	4.10900
O	-9.93200	-30.79900	-0.71000
H	-10.85700	-30.93200	-0.50200
H	-9.68300	-30.02000	-0.21300
O	0.01700	-34.73300	0.93600
H	-0.89200	-34.80100	0.64200
H	-0.03400	-34.25500	1.76500
O	-6.16200	-33.25700	-7.42500
H	-6.28200	-32.81100	-6.58700
H	-6.85600	-32.90800	-7.98400
O	-12.57100	-31.15600	0.12500
H	-12.44200	-30.90300	1.03900
H	-13.25100	-30.56300	-0.19500
O	-8.40100	-20.48600	-3.77600
H	-8.47800	-19.53600	-3.69700
H	-9.00500	-20.83200	-3.11900
O	-5.90900	-33.31400	8.88000
H	-6.68600	-32.75400	8.87000
H	-5.27000	-32.82800	9.40100
O	1.88300	-36.53100	2.71400
H	1.24000	-36.23800	2.06800
H	1.37800	-36.64600	3.52000
O	-0.46300	-32.78500	-6.59700
H	0.44900	-32.55200	-6.77200
H	-0.97200	-32.20100	-7.16000
O	-7.65400	-30.50500	8.44200
H	-6.91300	-29.96700	8.16500
H	-8.36100	-29.87800	8.59900
O	-2.17900	-28.36200	7.03900
H	-1.77100	-28.80400	6.29400
H	-2.90100	-28.93700	7.29200
O	-5.82600	-33.42500	4.41200
H	-4.98000	-33.00100	4.55600
H	-5.88600	-34.08200	5.10600
O	-1.85600	-27.80200	0.79400
H	-2.48500	-27.63700	0.09200
H	-2.24300	-27.38800	1.56500
O	2.02300	-26.86700	-1.85100
H	1.33800	-26.48700	-2.40200
H	2.10700	-26.25700	-1.11800
O	-2.67600	-35.76400	6.07000
H	-2.92000	-35.01000	5.53400
H	-2.40000	-35.38600	6.90500
O	-0.72200	-29.22100	4.74800
H	-0.51000	-28.57700	4.07200
H	-0.76900	-30.05400	4.28000
O	-3.11900	-36.60900	1.65200
H	-3.98100	-36.31100	1.94400
H	-3.09300	-36.39200	0.72000
O	-6.30300	-28.06500	-1.94100
H	-5.83900	-27.78600	-2.73000
H	-6.56800	-28.96600	-2.12300
O	-9.41100	-28.51700	0.99700
H	-8.84100	-28.08900	0.35900
H	-8.82000	-28.79900	1.69600
O	-7.36600	-31.94200	-5.13200
H	-6.66400	-31.49000	-4.66400
H	-7.69800	-32.58400	-4.50400
O	-8.91800	-33.35100	9.24300
H	-9.54800	-32.66900	9.47700
H	-9.02500	-33.46300	8.29900
O	-13.62400	-33.68400	0.17000
H	-14.41400	-33.42800	-0.30600
H	-13.13300	-32.86900	0.27500
O	0.39300	-26.34300	2.92000
H	1.21400	-26.74500	2.63900
H	0.38900	-26.44700	3.87200
O	-11.67200	-33.31900	-2.87200
H	-12.46900	-33.20800	-2.35300
H	-11.90000	-32.98100	-3.73800
O	-5.84200	-26.79500	5.07600
H	-5.77700	-26.33300	5.91200
H	-6.55100	-27.42500	5.20500
O	-15.34000	-33.43100	2.60100
H	-15.08900	-32.54600	2.86700
H	-14.80400	-33.61000	1.82900
O	8.48300	-30.00000	-0.73100
H	7.73000	-30.56900	-0.88700
H	8.61800	-30.03300	0.21600
O	-6.12700	-25.12800	-5.05600
H	-6.26200	-24.28300	-4.62700
H	-5.58600	-24.92600	-5.81900
O	-9.64000	-39.04900	11.60600
H	-10.03400	-39.60200	12.28100
H	-8.82400	-38.73800	11.99600
O	-12.67400	-27.61100	-4.48300
H	-12.16200	-26.81600	-4.33200
H	-12.02200	-28.30800	-4.54900
O	-10.86500	-24.49400	-2.40100
H	-10.68800	-23.57200	-2.58800
H	-10.06900	-24.80800	-1.97100
O	-6.72300	-37.63300	9.19800
H	-5.98300	-37.03300	9.11800
H	-6.49200	-38.37500	8.64000
O	-11.57900	-32.44800	7.47600
H	-11.69400	-33.34900	7.77600
H	-12.43500	-32.20100	7.12400
O	-4.50000	-24.50600	9.07400
H	-4.94400	-25.29200	8.75700
H	-3.83800	-24.31800	8.40900
O	-9.74100	-22.97600	3.60000
H	-9.19100	-23.10500	2.82800
H	-10.58000	-22.67700	3.25000
O	-2.40200	-24.22600	7.10300
H	-2.42000	-23.40500	6.61100
H	-1.48000	-24.35000	7.32800
O	-5.94500	-26.71100	7.94500
H	-5.41200	-27.47200	7.71500
H	-6.47300	-27.00400	8.68700
O	-2.77800	-41.60500	3.47400
H	-3.12100	-40.71500	3.39800
H	-2.41000	-41.79500	2.61100
O	-14.70900	-29.28200	-3.70700
H	-13.87200	-28.87200	-3.92400
H	-14.53100	-30.22200	-3.73700
O	-2.81300	-31.61400	-8.01800
H	-3.32800	-30.84400	-7.78000

H	-3.08500	-32.28800	-7.39500
O	-5.22800	-36.76900	-2.96000
H	-6.08400	-37.15900	-3.13900
H	-4.81500	-36.69000	-3.82000
O	-3.69800	-39.23500	1.43900
H	-4.20200	-39.27200	0.62600
H	-3.40500	-38.32500	1.49900
O	-7.45400	-39.15100	0.44600
H	-6.58900	-39.34600	0.08600
H	-7.38600	-39.38000	1.37300
O	2.16100	-28.86100	-6.08300
H	1.38400	-29.20000	-6.52800
H	2.84000	-28.85600	-6.75800
O	-3.02400	-33.74200	-6.19300
H	-3.32900	-33.42700	-5.34100
H	-2.07100	-33.66300	-6.15000
O	-4.57900	-27.58300	-4.54300
H	-5.10700	-28.31700	-4.85600
H	-5.08100	-26.80500	-4.78900
O	0.40000	-29.38800	0.57300
H	-0.25800	-28.69800	0.66200
H	1.20400	-28.99800	0.91700
O	-9.84800	-36.92100	-2.18300
H	-9.27300	-37.14300	-2.91500
H	-9.26000	-36.85200	-1.43000
O	-0.79000	-31.03300	-1.37600
H	-0.27600	-30.40000	-0.87400
H	-0.14000	-31.63300	-1.74200
O	-9.52100	-34.89000	-8.16300
H	-10.35700	-34.55300	-7.84200
H	-9.66000	-35.02500	-9.10000
O	-7.54500	-40.04600	-3.45500
H	-8.19900	-40.36200	-4.07900
H	-7.58700	-40.66900	-2.72900
O	-6.43400	-29.76500	-11.54000
H	-7.34100	-29.99600	-11.34100
H	-5.93700	-30.56100	-11.35200
O	-3.52700	-21.05500	-8.62000
H	-4.20100	-20.75200	-8.01200
H	-3.14100	-20.25200	-8.97000
O	-4.31200	-33.55800	0.87000
H	-5.22900	-33.33700	0.71200
H	-3.89600	-33.48400	0.01100
O	-3.52800	-32.15900	-4.00000
H	-2.78100	-31.64700	-4.30900
H	-3.25900	-32.48100	-3.14000
O	-5.25500	-29.66300	-7.01700
H	-6.16400	-29.94700	-6.92000
H	-5.22600	-29.24300	-7.87700
O	-0.02300	-26.05300	-3.24800
H	-0.47700	-26.89300	-3.19200
H	-0.65400	-25.41600	-2.91100
O	-1.08000	-35.12300	-2.01800
H	-1.81800	-35.63600	-1.68700
H	-0.82700	-35.56300	-2.83000
O	-1.58000	-24.21500	-2.31300
H	-1.28800	-23.65400	-3.03200
H	-1.07000	-23.92200	-1.55800
O	-0.97900	-42.34600	-1.42400
H	-0.67300	-43.20600	-1.71100
H	-0.25400	-41.75400	-1.62400
O	-14.68700	-31.94300	-3.03600
H	-14.88400	-32.53600	-3.76100
H	-15.24200	-32.24700	-2.31800
O	-6.70700	-32.05000	-0.91600
H	-6.65200	-33.00300	-0.99200
H	-7.51800	-31.89400	-0.43200
O	-5.55800	-28.12200	-9.67900
H	-5.99600	-28.83900	-10.13800
H	-4.81700	-27.89500	-10.24000
O	-8.76800	-33.43300	-3.15700
H	-9.58100	-33.13300	-2.75100
H	-8.27200	-33.82700	-2.44000
O	-6.61700	-23.43700	-1.22400
H	-6.65900	-23.21100	-2.15300
H	-5.70100	-23.67100	-1.07500
O	-3.17000	-32.96800	5.67400
H	-2.81500	-32.74100	6.53400
H	-2.52300	-32.63700	5.05200
O	2.56000	-29.84700	-3.62100
H	2.42100	-29.65600	-4.54900
H	2.20300	-29.08500	-3.16400
O	-9.27600	-35.86100	2.45900
H	-8.63600	-36.00200	1.76100
H	-10.10800	-36.14700	2.08300
O	2.58600	-27.77900	1.39000
H	3.02000	-27.87100	2.23900
H	3.30000	-27.80300	0.75200
O	-8.50000	-26.56100	-1.42800
H	-7.70600	-27.05600	-1.62900
H	-9.21100	-27.08900	-1.79100
O	-3.68100	-24.17900	2.92400
H	-3.62700	-23.61000	3.69100
H	-4.54900	-24.00900	2.55900
O	-12.19200	-32.41600	-5.35200
H	-13.12200	-32.47800	-5.13500
H	-12.09600	-32.93900	-6.14800
O	-12.27300	-35.89100	-2.76400
H	-12.01300	-34.97600	-2.87300
H	-11.45200	-36.35800	-2.61400
O	-10.80100	-34.47700	4.40600
H	-10.12900	-34.68500	3.75700
H	-11.62800	-34.67500	3.96700
O	3.44800	-27.73700	3.92400
H	3.64200	-26.83200	4.16900
H	3.34800	-28.19600	4.75700
O	-4.89800	-37.85100	6.42300
H	-5.54600	-37.15200	6.32900
H	-4.12800	-37.41700	6.79000
O	-1.49000	-27.58600	9.49800
H	-1.64200	-27.81500	8.58100
H	-1.50000	-26.62900	9.51100
O	-4.72200	-30.01300	4.48200
H	-3.79300	-29.86200	4.30900
H	-5.14300	-29.92400	3.62700
O	-5.92300	-25.98800	2.25200
H	-6.06200	-26.35600	3.12500
H	-6.71200	-25.47300	2.08100

O	-5.32800	-20.88700	-6.40500
H	-5.15300	-21.35800	-5.59100
H	-6.01500	-20.26000	-6.17700
O	-4.37300	-26.61000	-0.35900
H	-4.70800	-26.64900	0.53700
H	-4.87200	-27.27500	-0.83200
O	-10.39800	-31.20300	5.16600
H	-10.53600	-31.93500	5.76700
H	-9.48800	-31.29200	4.88500
O	-1.43700	-29.26400	-10.08500
H	-2.05400	-29.98900	-10.18200
H	-1.90500	-28.50300	-10.42900
O	-7.77000	-31.49100	4.65500
H	-7.06000	-32.13100	4.61300
H	-7.81300	-31.23900	5.57700
O	0.21900	-24.55400	7.57500
H	0.16000	-25.23800	6.90800
H	0.62100	-23.81200	7.12300
O	-9.05700	-34.50400	6.47400
H	-9.59300	-34.95500	7.12600
H	-9.64500	-34.37000	5.73100
O	-10.76200	-17.73800	0.36900
H	-10.52200	-17.10600	1.04700
H	-11.60500	-18.08900	0.65500
O	-9.64700	-35.04500	-10.96300
H	-10.52400	-35.16700	-11.32700
H	-9.17100	-35.83700	-11.21500
O	0.22100	-26.59000	5.80700
H	-0.66700	-26.92700	5.92900
H	0.79000	-27.32200	6.04400
O	-18.12700	-30.25800	-0.86900
H	-17.43900	-30.57200	-1.45700
H	-18.28200	-29.35400	-1.14200
O	-13.32300	-35.36900	3.55900
H	-13.40700	-35.97600	2.82500
H	-14.14500	-34.87800	3.56000
O	-6.24900	-21.93000	2.77300
H	-6.32200	-21.12400	3.28500
H	-6.69200	-21.73100	1.94800
O	-6.46000	-22.69300	-3.71700
H	-7.24400	-22.15100	-3.62300
H	-5.76100	-22.17500	-3.31700
O	-3.15100	-36.88300	-1.07400
H	-2.65400	-37.63600	-1.39300
H	-3.92200	-36.84700	-1.63900
O	4.70200	-31.17900	0.56800
H	4.69100	-30.29700	0.19800
H	3.83400	-31.53200	0.37400
O	-3.79000	-22.03100	4.92900
H	-3.07700	-21.39700	4.85600
H	-4.28100	-21.93500	4.11300
O	-2.06800	-28.12600	-3.50000
H	-3.00200	-28.00600	-3.66900
H	-1.89000	-29.02900	-3.76400
O	-6.56700	-34.74200	-1.34600
H	-6.93700	-35.29400	-0.65800
H	-6.07100	-35.34600	-1.89900
O	1.32000	-32.28400	-2.96500
H	1.78600	-31.45200	-3.03900
H	1.77000	-32.75000	-2.26000
O	-8.73800	-24.28200	5.74900
H	-8.03700	-23.64800	5.90200
H	-9.20900	-23.94000	4.98900
O	-0.61300	-25.94000	-5.96600
H	-0.33700	-26.05400	-5.05700
H	-1.48200	-26.33800	-6.00500
O	-0.70900	-39.91600	1.07400
H	-1.62300	-39.68600	1.24300
H	-0.28000	-39.83200	1.92600
O	-0.93800	-31.72700	3.57400
H	-1.59100	-31.48000	2.92000
H	-0.56000	-32.54100	3.24100
O	-3.97700	-21.69100	-2.32300
H	-3.79500	-22.50900	-1.86000
H	-3.15400	-21.47500	-2.76000
O	-3.64300	-22.76500	-5.06100
H	-3.11400	-23.27500	-5.67400
H	-3.38000	-23.08000	-4.19700
O	-4.88900	-30.98500	10.06100
H	-5.56500	-31.08600	10.73100
H	-4.06700	-31.14100	10.52500
O	-13.14200	-24.74800	2.68200
H	-12.55600	-25.49400	2.81500
H	-13.33900	-24.43800	3.56600
O	-10.28800	-21.71300	-2.30100
H	-10.95700	-21.22800	-2.78500
H	-10.42600	-21.46600	-1.38700
O	-18.55000	-32.17800	1.40300
H	-18.51100	-32.71100	2.19600
H	-19.05600	-32.70400	0.78400
O	-6.14700	-19.67900	4.29700
H	-5.59500	-19.51600	5.06200
H	-5.80700	-19.08800	3.62500
O	-11.17100	-37.33100	4.89900
H	-11.10700	-36.47000	4.48700
H	-11.23400	-37.94400	4.16600
O	-11.69500	-35.25900	8.54600
H	-11.06800	-35.64100	9.16000
H	-12.55300	-35.47700	8.91200
O	-1.29600	-24.85300	10.15200
H	-0.75200	-24.24000	9.65700
H	-1.87700	-24.29500	10.66800
O	-14.91900	-33.45100	-5.52200
H	-14.62400	-34.36000	-5.57500
H	-15.82000	-33.47200	-5.84400
O	-3.01000	-21.73000	1.28300
H	-3.13500	-22.53200	0.77400
H	-2.35200	-21.23600	0.79500
O	-11.38900	-35.37600	0.46100
H	-12.11100	-34.76200	0.32200
H	-10.60000	-34.84000	0.38300
O	-0.94700	-23.93000	2.91100
H	-1.89400	-24.04900	2.83900
H	-0.59900	-24.81700	2.99400
O	-8.58700	-38.04000	6.73500
H	-8.06300	-37.79400	7.49800
H	-8.80600	-37.20800	6.31500
O	-13.19000	-35.77000	-5.39800

H	-13.51900	-36.65000	-5.58000
H	-12.83600	-35.82500	-4.51000
O	-3.94200	-24.13400	-0.88800
H	-4.09000	-25.06300	-0.70800
H	-3.11700	-24.11200	-1.37300
O	-10.07600	-35.48200	-5.03000
H	-9.73600	-34.95400	-5.75300
H	-9.62900	-35.14400	-4.25500
O	-0.33900	-23.31100	0.19800
H	0.58600	-23.51700	0.06000
H	-0.50700	-23.56000	1.10700
O	-7.38200	-37.09900	4.42000
H	-7.72200	-37.89800	4.82200
H	-8.08800	-36.79900	3.84800
O	-6.73000	-40.01400	2.87300
H	-5.90700	-40.48800	2.75100
H	-6.48600	-39.23500	3.37300
O	-8.08900	-36.98500	-6.83100
H	-7.36800	-36.60700	-7.33500
H	-8.71300	-36.26600	-6.72800
O	-3.94600	-36.28100	-5.89500
H	-3.73000	-35.39800	-6.19600
H	-4.42700	-36.67200	-6.62500
O	-9.20600	-33.32200	0.68200
H	-9.35200	-33.00800	1.57400
H	-9.68400	-32.70300	0.12900
O	-7.34600	-36.33200	0.73000
H	-7.36000	-37.22800	0.39300
H	-6.62900	-36.32500	1.36400
O	-5.49400	-35.89800	2.77500
H	-5.75200	-34.97600	2.77400
H	-6.02500	-36.29500	3.46500
O	-0.54900	-22.79300	-4.44600
H	-0.37600	-23.35800	-5.19800
H	-0.05600	-21.99300	-4.62800
O	-14.73200	-30.78100	3.62100
H	-15.27600	-30.00500	3.75300
H	-13.85400	-30.43400	3.46300
O	-7.94300	-37.45000	-4.00100
H	-7.82100	-38.37900	-3.80200
H	-8.23300	-37.43600	-4.91300
O	-5.07400	-39.76500	-0.86500
H	-5.05100	-39.70100	-1.81900
H	-4.65700	-40.60400	-0.67100
O	5.30500	-30.02900	-2.82700
H	5.74800	-30.77900	-3.22500
H	4.42700	-30.03800	-3.20900
O	0.43800	-23.47600	-7.34600
H	1.13300	-22.85600	-7.12700
H	0.78100	-24.32700	-7.07100
O	-13.94500	-31.65400	6.51400
H	-14.68700	-31.50600	7.10000
H	-14.26600	-31.39400	5.65000
O	2.33700	-26.02600	-5.81700
H	2.86300	-25.68800	-6.54200
H	2.25000	-26.96200	-6.00100
O	-6.00100	-22.11300	6.31200
H	-5.69200	-22.35800	7.18400
H	-5.20300	-21.90800	5.82400
O	-5.97900	-19.95900	-1.45800
H	-6.41800	-20.45100	-0.76500
H	-5.22800	-20.50200	-1.69900
O	-13.49900	-23.76800	-1.45500
H	-12.67300	-24.06800	-1.83500
H	-13.85000	-24.53700	-1.00600
O	-8.53100	-24.41500	0.61400
H	-9.15300	-25.07800	0.31500
H	-7.86100	-24.38900	-0.06800
O	-15.29800	-25.84900	-0.49600
H	-16.13500	-26.27700	-0.67800
H	-15.32600	-25.65600	0.44100
O	-8.45900	-35.93700	10.20800
H	-8.41000	-35.06100	9.82500
H	-7.91600	-36.47900	9.63500
O	2.02700	-25.39300	9.48800
H	1.23600	-25.41100	8.94800
H	1.79000	-24.85400	10.24300
O	2.14700	-28.54400	6.80100
H	2.19600	-28.70900	7.74300
H	2.03800	-29.41100	6.41100
O	-8.97000	-25.86500	-5.91000
H	-8.82100	-25.18100	-6.56300
H	-8.16300	-25.88400	-5.39500
O	-0.56300	-32.56100	8.11700
H	0.33800	-32.70000	7.82500
H	-0.48800	-32.39500	9.05700
O	-6.22900	-35.18400	6.96200
H	-7.09800	-34.89100	6.69100
H	-5.99300	-34.60000	7.68300
O	3.64800	-26.12400	7.04500
H	3.35700	-25.76100	7.88200
H	3.03600	-26.83700	6.86700
O	4.84800	-28.55900	-0.43400
H	5.68300	-28.12000	-0.27100
H	4.98400	-29.03500	-1.25400
O	1.89700	-31.00100	5.51300
H	2.37300	-30.80800	4.70500
H	0.97400	-30.94500	5.26800
O	-11.21500	-29.83800	-4.90400
H	-11.46000	-30.74200	-4.70600
H	-11.04500	-29.83700	-5.84600
O	-3.52500	-38.96600	4.47900
H	-3.00700	-38.23000	4.15300
H	-3.87900	-38.66000	5.31400
O	-11.94500	-34.10500	-7.40500
H	-12.63800	-34.03100	-8.06100
H	-12.19000	-34.86900	-6.88300
O	-3.27400	-42.54600	-0.00100
H	-2.99300	-43.21400	0.62500
H	-2.48900	-42.35300	-0.51400
O	-5.47000	-23.37000	-8.43900
H	-6.04300	-22.75100	-8.89200
H	-4.89700	-22.82000	-7.90500
O	0.59700	-34.12400	3.74600
H	1.54900	-34.02400	3.76700
H	0.42800	-34.91600	4.25600
O	-1.38100	-37.03900	3.93200
H	-1.97600	-36.75800	3.23700

H	-1.66600	-36.54900	4.70400
O	-2.25100	-23.08600	-7.30800
H	-1.31700	-23.15200	-7.10400
H	-2.33800	-22.25100	-7.76600
O	0.37800	-23.77000	-10.34700
H	0.30000	-24.10400	-9.45400
H	1.19900	-23.27800	-10.34800
O	3.07600	-30.90400	2.86000
H	3.04400	-31.84200	3.04600
H	3.68600	-30.82500	2.12700
O	-5.08600	-32.03400	-11.09700
H	-4.14000	-32.16300	-11.02800
H	-5.46400	-32.77200	-10.62000
O	3.22600	-34.11200	2.90600
H	2.70900	-34.89700	2.71900
H	4.11000	-34.44100	3.07100
O	0.88700	-40.36800	-1.13400
H	0.68900	-40.67700	-0.25000
H	1.49700	-39.64200	-1.00400
O	8.89400	-29.84200	2.00100
H	9.62400	-30.10300	2.56200
H	8.28400	-30.57900	2.04100
O	6.97500	-31.70100	2.59800
H	6.24900	-31.83600	1.99000
H	6.56000	-31.39300	3.40400
O	-5.20200	-27.98200	10.36300
H	-4.28100	-27.80400	10.55700
H	-5.23700	-28.92700	10.21100
O	-6.90100	-34.10900	-11.16100
H	-7.80800	-34.40600	-11.08500
H	-6.62400	-34.40600	-12.02800
O	-15.98300	-32.78800	-0.71200
H	-16.30300	-33.64200	-1.00400
H	-16.53000	-32.57300	0.04400
O	-12.20500	-30.07200	3.00500
H	-11.98700	-29.14000	3.02400
H	-11.79300	-30.43100	3.79100
O	-5.28500	-39.56500	-5.00700
H	-6.09500	-39.91100	-4.63200
H	-4.59700	-39.87500	-4.41800
O	-3.35600	-40.48700	-3.38500
H	-2.80300	-40.06800	-2.72600
H	-3.11900	-41.41400	-3.34200
O	-7.33300	-34.49900	12.39600
H	-7.43300	-33.54900	12.32700
H	-7.55100	-34.82700	11.52300
O	-8.13100	-37.25000	-11.02000
H	-7.26900	-36.92500	-10.76100
H	-7.94600	-38.03700	-11.53300
O	-16.11500	-29.16800	1.25800
H	-16.80800	-29.58300	0.74500
H	-15.36700	-29.12600	0.66100
O	0.02200	-38.37700	-3.87700
H	-0.55800	-37.71900	-4.25900
H	-0.24500	-39.20000	-4.28800
O	-1.52800	-38.97700	-1.67500
H	-1.02800	-38.86500	-2.48300
H	-0.88400	-39.28100	-1.03500
O	-7.47000	-31.06900	11.21200
H	-7.95300	-30.26900	11.41900
H	-7.38300	-31.05800	10.25900
O	-14.20100	-29.23600	-0.86600
H	-14.22400	-29.13700	-1.81700
H	-13.89100	-28.39000	-0.54400
O	-11.54200	-27.17900	3.24900
H	-10.58800	-27.21000	3.18200
H	-11.74100	-27.64000	4.06400
O	-10.95200	-28.60800	5.81600
H	-10.13300	-28.11400	5.84000
H	-10.67900	-29.52500	5.78800
O	-1.19600	-36.21700	-4.82100
H	-2.08300	-35.93200	-5.03900
H	-0.78000	-36.37800	-5.66800
O	2.15600	-36.19300	-1.10600
H	2.93200	-35.70300	-0.83300
H	1.43100	-35.76700	-0.64800
O	4.59300	-35.48000	-0.04800
H	5.18800	-35.72000	-0.75900
H	4.66600	-36.19900	0.57900
O	-13.18700	-21.63700	0.26600
H	-13.32200	-22.39700	-0.30000
H	-13.60400	-20.91300	-0.20100
O	-2.05100	-26.45300	-10.72600
H	-1.34800	-26.24800	-10.11100
H	-2.77500	-25.88500	-10.46100
O	-1.22200	-30.70200	-4.60600
H	-1.27800	-30.24500	-5.44500
H	-0.66900	-31.46300	-4.78400
O	2.58900	-34.91300	-3.78100
H	2.33300	-35.31300	-2.95000
H	1.96400	-34.19900	-3.90600
O	6.90000	-32.17300	-1.27400
H	6.34000	-32.10400	-0.50100
H	6.29500	-32.34900	-1.99400
O	-10.03000	-20.74000	0.47800
H	-10.14700	-19.79500	0.37600
H	-10.85100	-21.04200	0.86600
O	-0.14300	-26.01500	-8.67600
H	0.69200	-26.44700	-8.85900
H	-0.30400	-26.18600	-7.74900
O	-0.48800	-29.51100	-7.31100
H	-0.54300	-29.67700	-8.25200
H	-1.11300	-28.80400	-7.15500
O	-15.69900	-34.27500	6.51500
H	-15.05300	-34.78500	7.00400
H	-15.90700	-33.53800	7.09000
O	-3.17300	-27.20700	12.01500
H	-2.30400	-27.03300	11.65300
H	-3.65300	-26.38700	11.89200
O	-7.17800	-40.01100	-7.73200
H	-7.19100	-39.06600	-7.57500
H	-6.28600	-40.19400	-8.02600
O	-9.00500	-20.08700	3.69300
H	-8.10200	-20.05500	4.01000
H	-9.23100	-21.01700	3.70800
O	-7.24900	-21.33400	0.35400
H	-8.20000	-21.33400	0.24100
H	-6.94600	-22.08000	-0.16200

O	-3.36300	-43.41600	-3.47800
H	-4.13000	-43.97100	-3.33200
H	-2.95200	-43.34400	-2.61600
O	-11.35900	-37.12800	10.61300
H	-10.49600	-37.48400	10.82300
H	-11.93300	-37.46700	11.30000
O	8.48600	-15.49800	1.41900
H	9.12400	-15.61700	0.71500
H	8.36800	-14.55000	1.47800
O	5.29500	-15.54500	1.35500
H	4.95700	-15.66100	0.46700
H	6.16400	-15.94600	1.33500
O	1.15300	-13.69100	-2.65500
H	0.59000	-13.78200	-3.42300
H	1.03900	-14.51100	-2.17500
O	5.77700	-14.39100	7.31400
H	5.76600	-14.78600	6.44200
H	5.93900	-15.12500	7.90700
O	-1.30000	-12.19600	0.18300
H	-0.50800	-12.53700	-0.23200
H	-1.06000	-12.08700	1.10400
O	6.73300	-10.02400	-7.34400
H	6.08200	-9.43900	-7.73000
H	7.52800	-9.49400	-7.28400
O	0.65900	-12.93500	-6.94500
H	1.43200	-13.48300	-7.08400
H	1.01200	-12.07100	-6.73300
O	2.49700	-11.60800	6.26200
H	2.20600	-10.70300	6.14600
H	3.13300	-11.56600	6.97600
O	3.42300	-14.23000	2.85200
H	4.01900	-14.45400	2.13800
H	3.48100	-14.97200	3.45400
O	2.99600	-16.77600	-9.31300
H	3.24800	-17.29800	-10.07500
H	2.05800	-16.94000	-9.21000
O	8.04000	-17.77300	-1.50600
H	8.82600	-18.19100	-1.85700
H	8.30400	-16.86800	-1.33700
O	8.21100	-12.33500	-6.80700
H	7.57600	-12.26200	-6.09500
H	7.88100	-11.74600	-7.48500
O	13.44100	-17.83500	-0.60200
H	14.17300	-18.40500	-0.36900
H	12.68500	-18.22300	-0.16200
O	2.71500	-14.87300	-7.11100
H	2.59900	-15.54900	-6.44300
H	2.82500	-15.36000	-7.92700
O	5.05800	-15.41200	-3.40600
H	4.69400	-15.93900	-2.69500
H	5.91500	-15.80200	-3.57900
O	10.96300	-24.12300	3.85700
H	10.63200	-23.25300	3.63300
H	10.36800	-24.43900	4.53700
O	4.69900	-20.38300	-8.41000
H	4.86600	-19.51500	-8.04300
H	4.50900	-20.22100	-9.33400
O	7.45600	-11.92200	3.25700
H	7.15700	-11.01900	3.14800
H	7.11200	-12.18800	4.10900
O	0.90800	-15.47000	-0.71000
H	-0.01700	-15.60300	-0.50200
H	1.15600	-14.69100	-0.21300
O	10.85600	-19.40300	0.93600
H	9.94800	-19.47200	0.64200
H	10.80500	-18.92600	1.76500
O	4.67800	-17.92800	-7.42500
H	4.55800	-17.48200	-6.58700
H	3.98300	-17.57900	-7.98400
O	-1.73100	-15.82700	0.12500
H	-1.60200	-15.57300	1.03900
H	-2.41200	-15.23400	-0.19500
O	4.93000	-17.98400	8.88000
H	4.15400	-17.42500	8.87000
H	5.56900	-17.49900	9.40100
O	12.72200	-21.20200	2.71400
H	12.08000	-20.90800	2.06800
H	12.21800	-21.31600	3.52000
O	10.37600	-17.45500	-6.59700
H	11.28800	-17.22300	-6.77200
H	9.86700	-16.87200	-7.16000
O	3.18500	-15.17600	8.44200
H	3.92600	-14.63700	8.16500
H	2.47900	-14.54900	8.59900
O	8.66000	-13.03300	7.03900
H	9.06900	-13.47500	6.29400
H	7.93800	-13.60800	7.29200
O	5.01400	-18.09600	4.41200
H	5.86000	-17.67200	4.55600
H	4.95300	-18.75200	5.10600
O	8.98300	-12.47300	0.79400
H	8.35400	-12.30800	0.09200
H	8.59600	-12.05900	1.56500
O	12.86200	-11.53700	-1.85100
H	12.17700	-11.15700	-2.40200
H	12.94700	-10.92800	-1.11800
O	8.16300	-20.43500	6.07000
H	7.92000	-19.68100	5.53400
H	8.43900	-20.05700	6.90500
O	10.11700	-13.89100	4.74800
H	10.32900	-13.24800	4.07200
H	10.07000	-14.72500	4.28000
O	7.72000	-21.28000	1.65200
H	6.85800	-20.98100	1.94400
H	7.74600	-21.06300	0.72000
O	4.53600	-12.73500	-1.94100
H	5.00000	-12.45700	-2.73000
H	4.27200	-13.63700	-2.12300
O	1.42900	-13.18800	0.99700
H	1.99900	-12.76000	0.35900
H	2.01900	-13.47000	1.69600
O	3.47300	-16.61300	-5.13200
H	4.17500	-16.16100	-4.66400
H	3.14100	-17.25500	-4.50400
O	1.92100	-18.02200	9.24300
H	1.29100	-17.34000	9.47700
H	1.81500	-18.13400	8.29900
O	-2.78400	-18.35500	0.17000

H	-3.57500	-18.09900	-0.30600
H	-2.29300	-17.54000	0.27500
O	11.23200	-11.01400	2.92000
H	12.05400	-11.41600	2.63900
H	11.22800	-11.11700	3.87200
O	-0.83300	-17.99000	-2.87200
H	-1.63000	-17.87800	-2.35300
H	-1.06000	-17.65200	-3.73800
O	4.99800	-11.46600	5.07600
H	5.06200	-11.00400	5.91200
H	4.28900	-12.09600	5.20500
O	-4.50100	-18.10200	2.60100
H	-4.25000	-17.21700	2.86700
H	-3.96500	-18.28100	1.82900
O	19.32300	-14.67000	-0.73100
H	18.56900	-15.24000	-0.88700
H	19.45700	-14.70400	0.21600
O	4.71200	-9.79900	-5.05600
H	4.57700	-8.95400	-4.62700
H	5.25300	-9.59700	-5.81900
O	1.19900	-23.72000	11.60600
H	0.80600	-24.27300	12.28100
H	2.01600	-23.40900	11.99600
O	-1.83500	-12.28100	-4.48300
H	-1.32200	-11.48700	-4.33200
H	-1.18200	-12.97900	-4.54900
O	-0.02500	-9.16500	-2.40100
H	0.15100	-8.24300	-2.58800
H	0.77000	-9.47900	-1.97100
O	4.11600	-22.30400	9.19800
H	4.85700	-21.70300	9.11800
H	4.34800	-23.04600	8.64000
O	-0.74000	-17.11800	7.47600
H	-0.85500	-18.02000	7.77600
H	-1.59600	-16.87200	7.12400
O	6.33900	-9.17700	9.07400
H	5.89600	-9.96300	8.75700
H	7.00100	-8.98900	8.40900
O	8.43700	-8.89600	7.10300
H	8.41900	-8.07500	6.61100
H	9.35900	-9.02100	7.32800
O	4.89500	-11.38200	7.94500
H	5.42700	-12.14300	7.71500
H	4.36600	-11.67500	8.68700
O	8.06200	-26.27600	3.47400
H	7.71800	-25.38600	3.39800
H	8.43000	-26.46600	2.61100
O	-3.87000	-13.95300	-3.70700
H	-3.03200	-13.54300	-3.92400
H	-3.69200	-14.89300	-3.73700
O	8.02700	-16.28500	-8.01800
H	7.51100	-15.51500	-7.78000
H	7.75400	-16.95900	-7.39500
O	5.61100	-21.44000	-2.96000
H	4.75600	-21.82900	-3.13900
H	6.02400	-21.36100	-3.82000
O	7.14100	-23.90500	1.43900
H	6.63800	-23.94200	0.62600
H	7.43400	-22.99600	1.49900
O	3.38600	-23.82200	0.44600
H	4.25100	-24.01700	0.08600
H	3.45300	-24.05100	1.37300
O	13.00000	-13.53200	-6.08300
H	12.22300	-13.87100	-6.52800
H	13.67900	-13.52700	-6.75800
O	7.81500	-18.41200	-6.19300
H	7.51000	-18.09800	-5.34100
H	8.76800	-18.33400	-6.15000
O	6.26000	-12.25300	-4.54300
H	5.73200	-12.98800	-4.85600
H	5.75900	-11.47600	-4.78900
O	11.23900	-14.05800	0.57300
H	10.58200	-13.36900	0.66200
H	12.04300	-13.66900	0.91700
O	0.99200	-21.59100	-2.18300
H	1.56600	-21.81400	-2.91500
H	1.57900	-21.52200	-1.43000
O	10.05000	-15.70400	-1.37600
H	10.56300	-15.07100	-0.87400
H	10.70000	-16.30400	-1.74200
O	1.31900	-19.56100	-8.16300
H	0.48200	-19.22300	-7.84200
H	1.17900	-19.69600	-9.10000
O	3.29400	-24.71700	-3.45500
H	2.64100	-25.03200	-4.07900
H	3.25200	-25.34000	-2.72900
O	4.40500	-14.43500	-11.54000
H	3.49800	-14.66600	-11.34100
H	4.90200	-15.23200	-11.35200
O	7.31200	-5.72600	-8.62000
H	6.63800	-5.42200	-8.01200
H	7.69900	-4.92300	-8.97000
O	6.52800	-18.22900	0.87000
H	5.61000	-18.00800	0.71200
H	6.94400	-18.15400	0.01100
O	7.31100	-16.83000	-4.00000
H	8.05900	-16.31800	-4.30900
H	7.58000	-17.15200	-3.14000
O	5.58400	-14.33400	-7.01700
H	4.67500	-14.61800	-6.92000
H	5.61400	-13.91400	-7.87700
O	10.81600	-10.72300	-3.24800
H	10.36200	-11.56400	-3.19200
H	10.18500	-10.08700	-2.91100
O	9.75900	-19.79400	-2.01800
H	9.02100	-20.30600	-1.68700
H	10.01200	-20.23400	-2.83000
O	9.25900	-8.88600	-2.31300
H	9.55200	-8.32500	-3.03200
H	9.76900	-8.59300	-1.55800
O	9.86000	-27.01700	-1.42400
H	10.16600	-27.87700	-1.71100
H	10.58600	-26.42500	-1.62400
O	-3.84700	-16.61400	-3.03600
H	-4.04500	-17.20700	-3.76100
H	-4.40300	-16.91800	-2.31800
O	4.13200	-16.72100	-0.91600
H	4.18700	-17.67400	-0.99200

H	3.32200	-16.56500	-0.43200
O	5.28200	-12.79300	-9.67900
H	4.84400	-13.51000	-10.13800
H	6.02300	-12.56600	-10.24000
O	2.07200	-18.10300	-3.15700
H	1.25900	-17.80400	-2.75100
H	2.56800	-18.49800	-2.44000
O	4.22300	-8.10800	-1.22400
H	4.18000	-7.88200	-2.15300
H	5.13900	-8.34100	-1.07500
O	7.66900	-17.63900	5.67400
H	8.02400	-17.41200	6.53400
H	8.31600	-17.30700	5.05200
O	13.39900	-14.51700	-3.62100
H	13.26100	-14.32700	-4.54900
H	13.04300	-13.75600	-3.16400
O	1.56300	-20.53200	2.45900
H	2.20300	-20.67300	1.76100
H	0.73100	-20.81800	2.08300
O	13.42600	-12.45000	1.39000
H	13.86000	-12.54200	2.23900
H	14.13900	-12.47400	0.75200
O	2.33900	-11.23100	-1.42800
H	3.13300	-11.72600	-1.62900
H	1.62800	-11.76000	-1.79100
O	7.15900	-8.85000	2.92400
H	7.21200	-8.28100	3.69100
H	6.29000	-8.67900	2.55900
O	-1.35300	-17.08700	-5.35200
H	-2.28300	-17.14900	-5.13500
H	-1.25700	-17.61000	-6.14800
O	-1.43400	-20.56200	-2.76400
H	-1.17300	-19.64700	-2.87300
H	-0.61200	-21.02900	-2.61400
O	0.03800	-19.14800	4.40600
H	0.71100	-19.35600	3.75700
H	-0.78900	-19.34600	3.96700
O	14.28700	-12.40700	3.92400
H	14.48100	-11.50300	4.16900
H	14.18700	-12.86700	4.75700
O	5.94100	-22.52200	6.42300
H	5.29400	-21.82300	6.32900
H	6.71100	-22.08800	6.79000
O	9.34900	-12.25700	9.49800
H	9.19700	-12.48600	8.58100
H	9.33900	-11.30000	9.51100
O	6.11700	-14.68400	4.48200
H	7.04700	-14.53200	4.30900
H	5.69700	-14.59500	3.62700
O	4.91600	-10.65900	2.25200
H	4.77700	-11.02700	3.12500
H	4.12800	-10.14400	2.08100
O	5.51100	-5.55800	-6.40500
H	5.68600	-6.02900	-5.59100
H	4.82400	-4.93100	-6.17700
O	6.46700	-11.28000	-0.35900
H	6.13200	-11.32000	0.53700
H	5.96700	-11.94600	-0.83200
O	0.44100	-15.87400	5.16600
H	0.30300	-16.60600	5.76700
H	1.35200	-15.96300	4.88500
O	9.40300	-13.93500	-10.08500
H	8.78600	-14.66000	-10.18200
H	8.93400	-13.17400	-10.42900
O	3.06900	-16.16200	4.65500
H	3.77900	-16.80200	4.61300
H	3.02600	-15.91000	5.57700
O	11.05800	-9.22500	7.57500
H	10.99900	-9.90900	6.90800
H	11.46000	-8.48300	7.12300
O	1.78200	-19.17500	6.47400
H	1.24600	-19.62600	7.12600
H	1.19400	-19.04000	5.73100
O	1.19300	-19.71600	-10.96300
H	0.31600	-19.83700	-11.32700
H	1.66800	-20.50800	-11.21500
O	11.06000	-11.26100	5.80700
H	10.17300	-11.59800	5.92900
H	11.63000	-11.99300	6.04400
O	-7.28700	-14.92800	-0.86900
H	-6.60000	-15.24200	-1.45700
H	-7.44200	-14.02400	-1.14200
O	-2.48400	-20.03900	3.55900
H	-2.56800	-20.64700	2.82500
H	-3.30500	-19.54900	3.56000
O	4.59000	-6.60100	2.77300
H	4.51700	-5.79500	3.28500
H	4.14800	-6.40200	1.94800
O	4.37900	-7.36400	-3.71700
H	3.59600	-6.82100	-3.62300
H	5.07800	-6.84600	-3.31700
O	7.68900	-21.55400	-1.07400
H	8.18600	-22.30700	-1.39300
H	6.91700	-21.51800	-1.63900
O	15.54200	-15.85000	0.56800
H	15.53100	-14.96800	0.19800
H	14.67300	-16.20300	0.37400
O	7.05000	-6.70200	4.92900
H	7.76300	-6.06700	4.85600
H	6.55900	-6.60600	4.11300
O	8.77200	-12.79700	-3.50000
H	7.83700	-12.67700	-3.66900
H	8.94900	-13.70000	-3.76400
O	4.27200	-19.41200	-1.34600
H	3.90200	-19.96500	-0.65800
H	4.76800	-20.01700	-1.89900
O	12.15900	-16.95500	-2.96500
H	12.62500	-16.12200	-3.03900
H	12.61000	-17.42100	-2.26000
O	2.10100	-8.95300	5.74900
H	2.80200	-8.31900	5.90200
H	1.63100	-8.61100	4.98900
O	10.22700	-10.61100	-5.96600
H	10.50200	-10.72500	-5.05700
H	9.35700	-11.00900	-6.00500
O	10.13100	-24.58700	1.07400
H	9.21700	-24.35700	1.24300
H	10.56000	-24.50300	1.92600

O	9.90100	-16.39800	3.57400
H	9.24800	-16.15100	2.92000
H	10.27900	-17.21200	3.24100
O	7.19600	-7.43500	-5.06100
H	7.72500	-7.94600	-5.67400
H	7.45900	-7.75100	-4.19700
O	5.95000	-15.65600	10.06100
H	5.27400	-15.75700	10.73100
H	6.77300	-15.81200	10.52500
O	-2.30200	-9.41900	2.68200
H	-1.71700	-10.16500	2.81500
H	-2.50000	-9.10900	3.56600
O	-7.71000	-16.84800	1.40300
H	-7.67200	-17.38200	2.19600
H	-8.21600	-17.37500	0.78400
O	-0.33100	-22.00200	4.89900
H	-0.26700	-21.14000	4.48700
H	-0.39500	-22.61500	4.16600
O	-0.85600	-19.93000	8.54600
H	-0.22900	-20.31200	9.16000
H	-1.71300	-20.14800	8.91200
O	9.54300	-9.52300	10.15200
H	10.08700	-8.91000	9.65700
H	8.96200	-8.96500	10.66800
O	-4.08000	-18.12200	-5.52200
H	-3.78400	-19.03100	-5.57500
H	-4.98100	-18.14300	-5.84400
O	7.82900	-6.40100	1.28300
H	7.70400	-7.20200	0.77400
H	8.48700	-5.90700	0.79500
O	-0.55000	-20.04600	0.46100
H	-1.27100	-19.43300	0.32200
H	0.23900	-19.51100	0.38300
O	9.89300	-8.60000	2.91100
H	8.94600	-8.72000	2.83900
H	10.24100	-9.48800	2.99400
O	2.25300	-22.71100	6.73500
H	2.77600	-22.46500	7.49800
H	2.03400	-21.87900	6.31500
O	-2.35100	-20.44100	-5.39800
H	-2.67900	-21.32100	-5.58000
H	-1.99700	-20.49600	-4.51000
O	6.89700	-8.80500	-0.88800
H	6.75000	-9.73300	-0.70800
H	7.72300	-8.78300	-1.37300
O	0.76300	-20.15300	-5.03000
H	1.10300	-19.62500	-5.75300
H	1.21100	-19.81500	-4.25500
O	10.50100	-7.98200	0.19800
H	11.42500	-8.18800	0.06000
H	10.33200	-8.23100	1.10700
O	3.45800	-21.77000	4.42000
H	3.11700	-22.56900	4.82200
H	2.75200	-21.46900	3.84800
O	4.10900	-24.68500	2.87300
H	4.93200	-25.15900	2.75100
H	4.35300	-23.90600	3.37300
O	2.75000	-21.65600	-6.83100
H	3.47100	-21.27800	-7.33500
H	2.12700	-20.93700	-6.72800
O	6.89300	-20.95100	-5.89500
H	7.11000	-20.06900	-6.19600
H	6.41300	-21.34300	-6.62500
O	1.63300	-17.99300	0.68200
H	1.48800	-17.67900	1.57400
H	1.15600	-17.37400	0.12900
O	3.49300	-21.00300	0.73000
H	3.47900	-21.89800	0.39300
H	4.21000	-20.99500	1.36400
O	5.34600	-20.56900	2.77500
H	5.08700	-19.64700	2.77400
H	4.81400	-20.96600	3.46500
O	10.29000	-7.46400	-4.44600
H	10.46400	-8.02900	-5.19800
H	10.78300	-6.66400	-4.62800
O	-3.89200	-15.45200	3.62100
H	-4.43700	-14.67600	3.75300
H	-3.01400	-15.10500	3.46300
O	2.89700	-22.12100	-4.00100
H	3.01800	-23.04900	-3.80200
H	2.60700	-22.10700	-4.91300
O	5.76600	-24.43600	-0.86500
H	5.78800	-24.37200	-1.81900
H	6.18300	-25.27500	-0.67100
O	16.14400	-14.70000	-2.82700
H	16.58700	-15.44900	-3.22500
H	15.26700	-14.70900	-3.20900
O	11.27800	-8.14700	-7.34600
H	11.97300	-7.52600	-7.12700
H	11.62000	-8.99700	-7.07100
O	-3.10600	-16.32500	6.51400
H	-3.84700	-16.17700	7.10000
H	-3.42600	-16.06400	5.65000
O	13.17700	-10.69700	-5.81700
H	13.70200	-10.35800	-6.54200
H	13.08900	-11.63200	-6.00100
O	4.83900	-6.78400	6.31200
H	5.14800	-7.02900	7.18400
H	5.63600	-6.57900	5.82400
O	-2.66000	-8.43900	-1.45500
H	-1.83400	-8.73800	-1.83500
H	-3.01000	-9.20800	-1.00600
O	2.30800	-9.08600	0.61400
H	1.68600	-9.74900	0.31500
H	2.97900	-9.06000	-0.06800
O	-4.45900	-10.52000	-0.49600
H	-5.29600	-10.94800	-0.67800
H	-4.48700	-10.32700	0.44100
O	2.38100	-20.60800	10.20800
H	2.43000	-19.73200	9.82500
H	2.92300	-21.15000	9.63500
O	12.86600	-10.06400	9.48800
H	12.07600	-10.08100	8.94800
H	12.63000	-9.52500	10.24300
O	12.98600	-13.21500	6.80100
H	13.03600	-13.38000	7.74300
H	12.87700	-14.08200	6.41100
O	1.87000	-10.53600	-5.91000

H	2.01800	-9.85200	-6.56300
H	2.67600	-10.55500	-5.39500
O	10.27600	-17.23100	8.11700
H	11.17700	-17.37100	7.82500
H	10.35100	-17.06600	9.05700
O	4.61100	-19.85500	6.96200
H	3.74100	-19.56100	6.69100
H	4.84700	-19.27100	7.68300
O	14.48800	-10.79500	7.04500
H	14.19600	-10.43200	7.88200
H	13.87500	-11.50800	6.86700
O	15.68700	-13.23000	-0.43400
H	16.52200	-12.79100	-0.27100
H	15.82300	-13.70600	-1.25400
O	12.73700	-15.67200	5.51300
H	13.21200	-15.47900	4.70500
H	11.81300	-15.61600	5.26800
O	-0.37500	-14.50900	-4.90400
H	-0.62100	-15.41300	-4.70600
H	-0.20600	-14.50700	-5.84600
O	7.31500	-23.63700	4.47900
H	7.83300	-22.90100	4.15300
H	6.96000	-23.33000	5.31400
O	-1.10600	-18.77600	-7.40500
H	-1.79900	-18.70200	-8.06100
H	-1.35000	-19.54000	-6.88300
O	7.56600	-27.21700	-0.00100
H	7.84600	-27.88400	0.62500
H	8.35100	-27.02400	-0.51400
O	5.36900	-8.04100	-8.43900
H	4.79700	-7.42200	-8.89200
H	5.94200	-7.49100	-7.90500
O	11.43700	-18.79500	3.74600
H	12.38800	-18.69500	3.76700
H	11.26700	-19.58700	4.25600
O	9.45800	-21.70900	3.93200
H	8.86300	-21.42900	3.23700
H	9.17300	-21.22000	4.70400
O	8.58900	-7.75700	-7.30800
H	9.52200	-7.82300	-7.10400
H	8.50100	-6.92100	-7.76600
O	11.21800	-8.44100	-10.34700
H	11.13900	-8.77500	-9.45400
H	12.03900	-7.94800	-10.34800
O	13.91500	-15.57400	2.86000
H	13.88300	-16.51300	3.04600
H	14.52600	-15.49600	2.12700
O	5.75400	-16.70500	-11.09700
H	6.70000	-16.83400	-11.02800
H	5.37500	-17.44300	-10.62000
O	14.06500	-18.78300	2.90600
H	13.54900	-19.56700	2.71900
H	14.94900	-19.11100	3.07100
O	11.72600	-25.03900	-1.13400
H	11.52800	-25.34800	-0.25000
H	12.33600	-24.31300	-1.00400
O	19.73300	-14.51300	2.00100
H	20.46300	-14.77400	2.56200
H	19.12400	-15.25000	2.04100
O	17.81500	-16.37200	2.59800
H	17.08800	-16.50600	1.99000
H	17.39900	-16.06400	3.40400
O	5.63800	-12.65300	10.36300
H	6.55800	-12.47500	10.55700
H	5.60300	-13.59700	10.21100
O	3.93800	-18.78000	-11.16100
H	3.03100	-19.07600	-11.08500
H	4.21600	-19.07700	-12.02800
O	-5.14400	-17.45900	-0.71200
H	-5.46400	-18.31300	-1.00400
H	-5.69100	-17.24400	0.04400
O	-1.36600	-14.74300	3.00500
H	-1.14700	-13.81100	3.02400
H	-0.95400	-15.10200	3.79100
O	5.55400	-24.23600	-5.00700
H	4.74400	-24.58100	-4.63200
H	6.24300	-24.54500	-4.41800
O	7.48300	-25.15800	-3.38500
H	8.03600	-24.73900	-2.72600
H	7.72000	-26.08500	-3.34200
O	3.50700	-19.17000	12.39600
H	3.40600	-18.22000	12.32700
H	3.28900	-19.49800	11.52300
O	2.70800	-21.92100	-11.02000
H	3.57000	-21.59600	-10.76100
H	2.89300	-22.70800	-11.53300
O	-5.27500	-13.83900	1.25800
H	-5.96900	-14.25300	0.74500
H	-4.52800	-13.79700	0.66100
O	10.86100	-23.04800	-3.87700
H	10.28100	-22.39000	-4.25900
H	10.59500	-23.87000	-4.28800
O	9.31200	-23.64800	-1.67500
H	9.81200	-23.53600	-2.48300
H	9.95500	-23.95200	-1.03500
O	3.37000	-15.74000	11.21200
H	2.88700	-14.94000	11.41900
H	3.45600	-15.72900	10.25900
O	-3.36200	-13.90700	-0.86600
H	-3.38500	-13.80800	-1.81700
H	-3.05200	-13.06000	-0.54400
O	-0.70300	-11.84900	3.24900
H	0.25100	-11.88100	3.18200
H	-0.90200	-12.31000	4.06400
O	-0.11300	-13.27900	5.81600
H	0.70600	-12.78500	5.84000
H	0.16100	-14.19600	5.78800
O	9.64400	-20.88800	-4.82100
H	8.75600	-20.60300	-5.03900
H	10.05900	-21.04900	-5.66800
O	12.99500	-20.86400	-1.10600
H	13.77100	-20.37400	-0.83300
H	12.27000	-20.43800	-0.64800
O	15.43200	-20.15100	-0.04800
H	16.02700	-20.39100	-0.75900
H	15.50500	-20.87000	0.57900
O	8.78800	-11.12400	-10.72600
H	9.49200	-10.91900	-10.11100

H	8.06400	-10.55600	-10.46100
O	9.61800	-15.37300	-4.60600
H	9.56100	-14.91600	-5.44500
H	10.17100	-16.13400	-4.78400
O	13.42800	-19.58400	-3.78100
H	13.17200	-19.98400	-2.95000
H	12.80400	-18.86900	-3.90600
O	17.74000	-16.84400	-1.27400
H	17.18000	-16.77500	-0.50100
H	17.13400	-17.02000	-1.99400
O	10.69700	-10.68500	-8.67600
H	11.53100	-11.11700	-8.85900
H	10.53500	-10.85700	-7.74900
O	10.35100	-14.18200	-7.31100
H	10.29600	-14.34800	-8.25200
H	9.72600	-13.47500	-7.15500
O	-4.85900	-18.94500	6.51500
H	-4.21400	-19.45600	7.00400
H	-5.06800	-18.20900	7.09000
O	7.66600	-11.87700	12.01500
H	8.53500	-11.70300	11.65300
H	7.18700	-11.05800	11.89200
O	3.66100	-24.68100	-7.73200
H	3.64900	-23.73700	-7.57500
H	4.55300	-24.86400	-8.02600
O	7.47600	-28.08700	-3.47800
H	6.70900	-28.64100	-3.33200
H	7.88700	-28.01500	-2.61600
O	-0.52000	-21.79900	10.61300
H	0.34300	-22.15500	10.82300
H	-1.09300	-22.13700	11.30000
O	19.32500	-0.16900	1.41900
H	19.96300	-0.28800	0.71500
H	19.20700	0.78000	1.47800
O	16.13400	-0.21600	1.35500
H	15.79600	-0.33200	0.46700
H	17.00300	-0.61700	1.33500
O	11.99300	1.63800	-2.65500
H	11.42900	1.54700	-3.42300
H	11.87800	0.81800	-2.17500
O	16.61600	0.93800	7.31400
H	16.60500	0.54300	6.44200
H	16.77800	0.20400	7.90700
O	9.53900	3.13300	0.18300
H	10.33100	2.79200	-0.23200
H	9.77900	3.24200	1.10400
O	17.57300	5.30500	-7.34400
H	16.92200	5.89000	-7.73000
H	18.36700	5.83500	-7.28400
O	11.49800	2.39400	-6.94500
H	12.27100	1.84700	-7.08400
H	11.85100	3.25800	-6.73300
O	13.33700	3.72100	6.26200
H	13.04600	4.62600	6.14600
H	13.97300	3.76300	6.97600
O	14.26200	1.09900	2.85200
H	14.85900	0.87500	2.13800
H	14.32000	0.35800	3.45400
O	13.83500	-1.44700	-9.31300
H	14.08800	-1.96900	-10.07500
H	12.89700	-1.61000	-9.21000
O	18.87900	-2.44300	-1.50600
H	19.66600	-2.86100	-1.85700
H	19.14300	-1.53900	-1.33700
O	19.05000	2.99400	-6.80700
H	18.41500	3.06700	-6.09500
H	18.72000	3.58300	-7.48500
O	24.28000	-2.50600	-0.60200
H	25.01300	-3.07600	-0.36900
H	23.52400	-2.89400	-0.16200
O	13.55500	0.45600	-7.11100
H	13.43800	-0.22000	-6.44300
H	13.66500	-0.03100	-7.92700
O	15.89800	-0.08300	-3.40600
H	15.53400	-0.61000	-2.69500
H	16.75400	-0.47300	-3.57900
O	21.80300	-8.79400	3.85700
H	21.47100	-7.92400	3.63300
H	21.20700	-9.11000	4.53700
O	15.53800	-5.05400	-8.41000
H	15.70600	-4.18600	-8.04300
H	15.34800	-4.89100	-9.33400
O	18.29600	3.40800	3.25700
H	17.99600	4.31000	3.14800
H	17.95100	3.14100	4.10900
O	11.74700	-0.14100	-0.71000
H	10.82200	-0.27300	-0.50200
H	11.99600	0.63800	-0.21300
O	21.69600	-4.07400	0.93600
H	20.78700	-4.14300	0.64200
H	21.64400	-3.59700	1.76500
O	15.51700	-2.59800	-7.42500
H	15.39700	-2.15300	-6.58700
H	14.82200	-2.25000	-7.98400
O	13.27800	10.17200	-3.77600
H	13.20000	11.12300	-3.69700
H	12.67400	9.82700	-3.11900
O	15.77000	-2.65500	8.88000
H	14.99300	-2.09600	8.87000
H	16.40900	-2.17000	9.40100
O	23.56200	-5.87300	2.71400
H	22.91900	-5.57900	2.06800
H	23.05700	-5.98700	3.52000
O	21.21600	-2.12600	-6.59700
H	22.12800	-1.89300	-6.77200
H	20.70700	-1.54300	-7.16000
O	14.02400	0.15300	8.44200
H	14.76600	0.69200	8.16500
H	13.31800	0.78000	8.59900
O	19.50000	2.29600	7.03900
H	19.90800	1.85400	6.29400
H	18.77800	1.72200	7.29200
O	15.85300	-2.76600	4.41200
H	16.69900	-2.34300	4.55600
H	15.79300	-3.42300	5.10600
O	19.82300	2.85600	0.79400
H	19.19400	3.02100	0.09200
H	19.43500	3.27100	1.56500

O	23.70200	3.79200	-1.85100
H	23.01700	4.17200	-2.40200
H	23.78600	4.40100	-1.11800
O	20.95700	1.43800	4.74800
H	21.16800	2.08100	4.07200
H	20.91000	0.60400	4.28000
O	18.55900	-5.95000	1.65200
H	17.69800	-5.65200	1.94400
H	18.58600	-5.73300	0.72000
O	15.37500	2.59400	-1.94100
H	15.83900	2.87300	-2.73000
H	15.11100	1.69200	-2.12300
O	12.26800	2.14100	0.99700
H	12.83800	2.57000	0.35900
H	12.85900	1.85900	1.69600
O	14.31200	-1.28400	-5.13200
H	15.01500	-0.83200	-4.66400
H	13.98000	-1.92600	-4.50400
O	12.76000	-2.69300	9.24300
H	12.13000	-2.01100	9.47700
H	12.65400	-2.80400	8.29900
O	22.07100	4.31500	2.92000
H	22.89300	3.91300	2.63900
H	22.06800	4.21200	3.87200
O	15.83700	3.86300	5.07600
H	15.90100	4.32500	5.91200
H	15.12800	3.23300	5.20500
O	30.16200	0.65900	-0.73100
H	29.40800	0.09000	-0.88700
H	30.29700	0.62500	0.21600
O	15.55200	5.53000	-5.05600
H	15.41600	6.37500	-4.62700
H	16.09300	5.73200	-5.81900
O	12.03800	-8.39100	11.60600
H	11.64500	-8.94400	12.28100
H	12.85500	-8.08000	11.99600
O	9.00500	3.04800	-4.48300
H	9.51700	3.84200	-4.33200
H	9.65700	2.35000	-4.54900
O	10.81400	6.16400	-2.40100
H	10.99100	7.08600	-2.58800
H	11.61000	5.85000	-1.97100
O	14.95500	-6.97500	9.19800
H	15.69600	-6.37400	9.11800
H	15.18700	-7.71700	8.64000
O	10.09900	-1.78900	7.47600
H	9.98500	-2.69100	7.77600
H	9.24400	-1.54300	7.12400
O	17.17900	6.15300	9.07400
H	16.73500	5.36600	8.75700
H	17.84000	6.34000	8.40900
O	11.93700	7.68200	3.60000
H	12.48800	7.55400	2.82800
H	11.09800	7.98200	3.25000
O	19.27700	6.43300	7.10300
H	19.25900	7.25400	6.61100
H	20.19900	6.30800	7.32800
O	15.73400	3.94700	7.94500
H	16.26700	3.18600	7.71500
H	15.20500	3.65500	8.68700
O	18.90100	-10.94700	3.47400
H	18.55700	-10.05700	3.39800
H	19.26900	-11.13700	2.61100
O	18.86600	-0.95600	-8.01800
H	18.35100	-0.18500	-7.78000
H	18.59400	-1.62900	-7.39500
O	16.45100	-6.11100	-2.96000
H	15.59500	-6.50000	-3.13900
H	16.86300	-6.03200	-3.82000
O	17.98100	-8.57600	1.43900
H	17.47700	-8.61300	0.62600
H	18.27400	-7.66700	1.49900
O	14.22500	-8.49200	0.44600
H	15.09000	-8.68800	0.08600
H	14.29300	-8.72100	1.37300
O	23.83900	1.79700	-6.08300
H	23.06200	1.45800	-6.52800
H	24.51900	1.80300	-6.75800
O	18.65500	-3.08300	-6.19300
H	18.35000	-2.76900	-5.34100
H	19.60800	-3.00500	-6.15000
O	17.09900	3.07600	-4.54300
H	16.57100	2.34100	-4.85600
H	16.59800	3.85300	-4.78900
O	22.07900	1.27100	0.57300
H	21.42100	1.96100	0.66200
H	22.88300	1.66000	0.91700
O	11.83100	-6.26200	-2.18300
H	12.40600	-6.48500	-2.91500
H	12.41800	-6.19300	-1.43000
O	20.88900	-0.37500	-1.37600
H	21.40200	0.25800	-0.87400
H	21.53900	-0.97500	-1.74200
O	12.15800	-4.23200	-8.16300
H	11.32200	-3.89400	-7.84200
H	12.01900	-4.36700	-9.10000
O	14.13300	-9.38800	-3.45500
H	13.48000	-9.70300	-4.07900
H	14.09100	-10.01000	-2.72900
O	15.24500	0.89400	-11.54000
H	14.33700	0.66300	-11.34100
H	15.74200	0.09800	-11.35200
O	18.15100	9.60400	-8.62000
H	17.47800	9.90700	-8.01200
H	18.53800	10.40700	-8.97000
O	17.36700	-2.90000	0.87000
H	16.44900	-2.67800	0.71200
H	17.78300	-2.82500	0.01100
O	18.15100	-1.50100	-4.00000
H	18.89800	-0.98900	-4.30900
H	18.42000	-1.82200	-3.14000
O	16.42300	0.99500	-7.01700
H	15.51500	0.71100	-6.92000
H	16.45300	1.41500	-7.87700
O	21.65500	4.60600	-3.24800
H	21.20200	3.76500	-3.19200
H	21.02500	5.24200	-2.91100
O	20.59900	-4.46500	-2.01800

H	19.86100	-4.97700	-1.68700
H	20.85100	-4.90500	-2.83000
O	20.09900	6.44300	-2.31300
H	20.39100	7.00400	-3.03200
H	20.60800	6.73600	-1.55800
O	20.70000	-11.68700	-1.42400
H	21.00600	-12.54800	-1.71100
H	21.42500	-11.09600	-1.62400
O	14.97200	-1.39200	-0.91600
H	15.02700	-2.34500	-0.99200
H	14.16100	-1.23600	-0.43200
O	16.12100	2.53600	-9.67900
H	15.68300	1.82000	-10.13800
H	16.86200	2.76400	-10.24000
O	12.91100	-2.77400	-3.15700
H	12.09800	-2.47500	-2.75100
H	13.40700	-3.16900	-2.44000
O	15.06200	7.22100	-1.22400
H	15.02000	7.44800	-2.15300
H	15.97800	6.98800	-1.07500
O	24.23800	0.81200	-3.62100
H	24.10000	1.00200	-4.54900
H	23.88200	1.57400	-3.16400
O	12.40300	-5.20200	2.45900
H	13.04200	-5.34400	1.76100
H	11.57000	-5.48900	2.08300
O	24.26500	2.87900	1.39000
H	24.69900	2.78700	2.23900
H	24.97800	2.85500	0.75200
O	13.17900	4.09800	-1.42800
H	13.97300	3.60300	-1.62900
H	12.46800	3.57000	-1.79100
O	17.99800	6.48000	2.92400
H	18.05200	7.04900	3.69100
H	17.13000	6.65000	2.55900
O	9.48700	-1.75800	-5.35200
H	8.55700	-1.81900	-5.13500
H	9.58300	-2.28000	-6.14800
O	10.87800	-3.81900	4.40600
H	11.55000	-4.02700	3.75700
H	10.05100	-4.01600	3.96700
O	25.12700	2.92200	3.92400
H	25.32000	3.82600	4.16900
H	25.02700	2.46200	4.75700
O	16.78000	-7.19200	6.42300
H	16.13300	-6.49300	6.32900
H	17.55100	-6.75900	6.79000
O	20.18800	3.07200	9.49800
H	20.03700	2.84300	8.58100
H	20.17900	4.02900	9.51100
O	16.95700	0.64600	4.48200
H	17.88600	0.79700	4.30900
H	16.53600	0.73400	3.62700
O	15.75600	4.67100	2.25200
H	15.61700	4.30300	3.12500
H	14.96700	5.18500	2.08100
O	16.35000	9.77200	-6.40500
H	16.52600	9.30000	-5.59100
H	15.66400	10.39800	-6.17700
O	17.30600	4.04900	-0.35900
H	16.97100	4.00900	0.53700
H	16.80700	3.38300	-0.83200
O	11.28100	-0.54400	5.16600
H	11.14300	-1.27600	5.76700
H	12.19100	-0.63300	4.88500
O	20.24200	1.39400	-10.08500
H	19.62500	0.66900	-10.18200
H	19.77400	2.15500	-10.42900
O	13.90900	-0.83200	4.65500
H	14.61900	-1.47300	4.61300
H	13.86600	-0.58100	5.57700
O	21.89800	6.10400	7.57500
H	21.83900	5.42000	6.90800
H	22.29900	6.84600	7.12300
O	12.62200	-3.84600	6.47400
H	12.08500	-4.29700	7.12600
H	12.03300	-3.71100	5.73100
O	10.91700	12.92100	0.36900
H	11.15600	13.55200	1.04700
H	10.07400	12.56900	0.65500
O	12.03200	-4.38700	-10.96300
H	11.15500	-4.50800	-11.32700
H	12.50800	-5.17800	-11.21500
O	21.89900	4.06800	5.80700
H	21.01200	3.73100	5.92900
H	22.46900	3.33700	6.04400
O	8.35600	-4.71000	3.55900
H	8.27100	-5.31800	2.82500
H	7.53400	-4.21900	3.56000
O	15.42900	8.72800	2.77300
H	15.35600	9.53400	3.28500
H	14.98700	8.92800	1.94800
O	15.21800	7.96600	-3.71700
H	14.43500	8.50800	-3.62300
H	15.91800	8.48300	-3.31700
O	18.52800	-6.22500	-1.07400
H	19.02500	-6.97800	-1.39300
H	17.75700	-6.18800	-1.63900
O	26.38100	-0.52100	0.56800
H	26.37000	0.36200	0.19800
H	25.51300	-0.87400	0.37400
O	17.88900	8.62700	4.92900
H	18.60200	9.26200	4.85600
H	17.39800	8.72400	4.11300
O	19.61100	2.53200	-3.50000
H	18.67700	2.65200	-3.66900
H	19.78900	1.63000	-3.76400
O	15.11200	-4.08300	-1.34600
H	14.74100	-4.63600	-0.65800
H	15.60700	-4.68700	-1.89900
O	22.99800	-1.62600	-2.96500
H	23.46400	-0.79300	-3.03900
H	23.44900	-2.09200	-2.26000
O	12.94000	6.37700	5.74900
H	13.64100	7.01000	5.90200
H	12.47000	6.71800	4.98900
O	21.06600	4.71800	-5.96600
H	21.34200	4.60400	-5.05700

H	20.19600	4.32000	-6.00500
O	20.97000	-9.25800	1.07400
H	20.05600	-9.02800	1.24300
H	21.39900	-9.17400	1.92600
O	17.70100	8.96700	-2.32300
H	17.88400	8.14900	-1.86000
H	18.52500	9.18400	-2.76000
O	18.03600	7.89400	-5.06100
H	18.56400	7.38300	-5.67400
H	18.29800	7.57800	-4.19700
O	16.78900	-0.32700	10.06100
H	16.11300	-0.42800	10.73100
H	17.61200	-0.48300	10.52500
O	11.39100	8.94500	-2.30100
H	10.72200	9.43000	-2.78500
H	11.25200	9.19200	-1.38700
O	15.53200	10.98000	4.29700
H	16.08300	11.14300	5.06200
H	15.87200	11.57000	3.62500
O	10.50800	-6.67300	4.89900
H	10.57200	-5.81100	4.48700
H	10.44500	-7.28500	4.16600
O	9.98300	-4.60000	8.54600
H	10.61000	-4.98300	9.16000
H	9.12600	-4.81900	8.91200
O	20.38300	5.80600	10.15200
H	20.92700	6.41900	9.65700
H	19.80100	6.36400	10.66800
O	18.66800	8.92800	1.28300
H	18.54400	8.12700	0.77400
H	19.32600	9.42300	0.79500
O	20.73200	6.72900	2.91100
H	19.78500	6.60900	2.83900
H	21.08000	5.84100	2.99400
O	13.09200	-7.38200	6.73500
H	13.61500	-7.13600	7.49800
H	12.87300	-6.55000	6.31500
O	8.48900	-5.11100	-5.39800
H	8.16000	-5.99200	-5.58000
H	8.84300	-5.16700	-4.51000
O	17.73700	6.52400	-0.88800
H	17.58900	5.59600	-0.70800
H	18.56200	6.54600	-1.37300
O	11.60300	-4.82400	-5.03000
H	11.94200	-4.29600	-5.75300
H	12.05000	-4.48600	-4.25500
O	21.34000	7.34700	0.19800
H	22.26500	7.14100	0.06000
H	21.17200	7.09900	1.10700
O	14.29700	-6.44000	4.42000
H	13.95700	-7.24000	4.82200
H	13.59100	-6.14000	3.84800
O	14.94900	-9.35500	2.87300
H	15.77100	-9.82900	2.75100
H	15.19300	-8.57600	3.37300
O	13.59000	-6.32700	-6.83100
H	14.31000	-5.94900	-7.33500
H	12.96600	-5.60800	-6.72800
O	17.73300	-5.62200	-5.89500
H	17.94900	-4.74000	-6.19600
H	17.25200	-6.01400	-6.62500
O	12.47300	-2.66400	0.68200
H	12.32700	-2.35000	1.57400
H	11.99500	-2.04500	0.12900
O	14.33200	-5.67300	0.73000
H	14.31800	-6.56900	0.39300
H	15.05000	-5.66600	1.36400
O	16.18500	-5.24000	2.77500
H	15.92700	-4.31800	2.77400
H	15.65300	-5.63700	3.46500
O	21.13000	7.86600	-4.44600
H	21.30300	7.30000	-5.19800
H	21.62200	8.66600	-4.62800
O	6.94700	-0.12300	3.62100
H	6.40300	0.65300	3.75300
H	7.82500	0.22400	3.46300
O	13.73600	-6.79200	-4.00100
H	13.85800	-7.72000	-3.80200
H	13.44600	-6.77800	-4.91300
O	16.60500	-9.10600	-0.86500
H	16.62700	-9.04200	-1.81900
H	17.02200	-9.94600	-0.67100
O	26.98300	0.62900	-2.82700
H	27.42700	-0.12000	-3.22500
H	26.10600	0.62000	-3.20900
O	22.11700	7.18200	-7.34600
H	22.81200	7.80300	-7.12700
H	22.45900	6.33200	-7.07100
O	24.01600	4.63200	-5.81700
H	24.54100	4.97100	-6.54200
H	23.92800	3.69700	-6.00100
O	15.67800	8.54600	6.31200
H	15.98700	8.30000	7.18400
H	16.47600	8.75000	5.82400
O	15.70000	10.69900	-1.45800
H	15.26100	10.20700	-0.76500
H	16.45100	10.15700	-1.69900
O	13.14800	6.24300	0.61400
H	12.52600	5.58000	0.31500
H	13.81800	6.26900	-0.06800
O	13.22000	-5.27900	10.20800
H	13.26900	-4.40200	9.82500
H	13.76200	-5.82100	9.63500
O	23.70600	5.26500	9.48800
H	22.91500	5.24800	8.94800
H	23.46900	5.80400	10.24300
O	23.82500	2.11500	6.80100
H	23.87500	1.95000	7.74300
H	23.71700	1.24700	6.41100
O	12.70900	4.79400	-5.91000
H	12.85800	5.47700	-6.56300
H	13.51600	4.77400	-5.39500
O	15.45000	-4.52600	6.96200
H	14.58000	-4.23200	6.69100
H	15.68600	-3.94100	7.68300
O	25.32700	4.53400	7.04500
H	25.03600	4.89700	7.88200
H	24.71400	3.82100	6.86700

O	26.52700	2.10000	-0.43400
H	27.36100	2.53900	-0.27100
H	26.66300	1.62400	-1.25400
O	10.46400	0.82000	-4.90400
H	10.21900	-0.08400	-4.70600
H	10.63400	0.82200	-5.84600
O	18.15400	-8.30800	4.47900
H	18.67200	-7.57100	4.15300
H	17.79900	-8.00100	5.31400
O	9.73400	-3.44700	-7.40500
H	9.04100	-3.37300	-8.06100
H	9.48900	-4.21100	-6.88300
O	18.40500	-11.88800	-0.00100
H	18.68500	-12.55500	0.62500
H	19.19000	-11.69500	-0.51400
O	16.20900	7.28800	-8.43900
H	15.63600	7.90700	-8.89200
H	16.78200	7.83800	-7.90500
O	20.29800	-6.38000	3.93200
H	19.70200	-6.10000	3.23700
H	20.01200	-5.89100	4.70400
O	19.42800	7.57200	-7.30800
H	20.36100	7.50700	-7.10400
H	19.34100	8.40800	-7.76600
O	22.05700	6.88900	-10.34700
H	21.97800	6.55500	-9.45400
H	22.87800	7.38100	-10.34800
O	24.75500	-0.24500	2.86000
H	24.72300	-1.18400	3.04600
H	25.36500	-0.16700	2.12700
O	16.59300	-1.37600	-11.09700
H	17.53900	-1.50500	-11.02800
H	16.21500	-2.11400	-10.62000
O	24.90400	-3.45400	2.90600
H	24.38800	-4.23800	2.71900
H	25.78800	-3.78200	3.07100
O	22.56500	-9.71000	-1.13400
H	22.36800	-10.01800	-0.25000
H	23.17500	-8.98400	-1.00400
O	30.57300	0.81600	2.00100
H	31.30300	0.55500	2.56200
H	29.96300	0.07900	2.04100
O	28.65400	-1.04300	2.59800
H	27.92700	-1.17700	1.99000
H	28.23900	-0.73500	3.40400
O	16.47700	2.67600	10.36300
H	17.39700	2.85400	10.55700
H	16.44200	1.73200	10.21100
O	14.77700	-3.45100	-11.16100
H	13.87100	-3.74700	-11.08500
H	15.05500	-3.74800	-12.02800
O	9.47300	0.58600	3.00500
H	9.69200	1.51800	3.02400
H	9.88600	0.22700	3.79100
O	16.39400	-8.90700	-5.00700
H	15.58400	-9.25200	-4.63200
H	17.08200	-9.21600	-4.41800
O	18.32300	-9.82900	-3.38500
H	18.87600	-9.41000	-2.72600
H	18.56000	-10.75500	-3.34200
O	14.34600	-3.84000	12.39600
H	14.24600	-2.89100	12.32700
H	14.12800	-4.16900	11.52300
O	13.54700	-6.59200	-11.02000
H	14.40900	-6.26600	-10.76100
H	13.73200	-7.37900	-11.53300
O	5.56400	1.49000	1.25800
H	4.87000	1.07600	0.74500
H	6.31100	1.53200	0.66100
O	21.70100	-7.71900	-3.87700
H	21.12100	-7.06100	-4.25900
H	21.43400	-8.54100	-4.28800
O	20.15100	-8.31900	-1.67500
H	20.65100	-8.20700	-2.48300
H	20.79500	-8.62200	-1.03500
O	14.20900	-0.41100	11.21200
H	13.72600	0.38900	11.41900
H	14.29500	-0.40000	10.25900
O	10.72600	2.05000	5.81600
H	11.54600	2.54400	5.84000
H	11.00000	1.13300	5.78800
O	20.48300	-5.55900	-4.82100
H	19.59600	-5.27400	-5.03900
H	20.89900	-5.72000	-5.66800
O	23.83500	-5.53400	-1.10600
H	24.61100	-5.04500	-0.83300
H	23.10900	-5.10900	-0.64800
O	26.27200	-4.82200	-0.04800
H	26.86600	-5.06200	-0.75900
H	26.34400	-5.54100	0.57900
O	19.62700	4.20500	-10.72600
H	20.33100	4.41000	-10.11100
H	18.90400	4.77300	-10.46100
O	20.45700	-0.04400	-4.60600
H	20.40100	0.41300	-5.44500
H	21.01000	-0.80400	-4.78400
O	24.26800	-4.25500	-3.78100
H	24.01100	-4.65500	-2.95000
H	23.64300	-3.54000	-3.90600
O	28.57900	-1.51500	-1.27400
H	28.01900	-1.44600	-0.50100
H	27.97300	-1.69100	-1.99400
O	11.64800	9.91900	0.47800
H	11.53200	10.86300	0.37600
H	10.82800	9.61600	0.86600
O	21.53600	4.64400	-8.67600
H	22.37100	4.21200	-8.85900
H	21.37500	4.47200	-7.74900
O	21.19100	1.14700	-7.31100
H	21.13600	0.98100	-8.25200
H	20.56500	1.85500	-7.15500
O	5.98000	-3.61600	6.51500
H	6.62600	-4.12600	7.00400
H	5.77200	-2.87900	7.09000
O	18.50500	3.45200	12.01500
H	19.37400	3.62600	11.65300
H	18.02600	4.27100	11.89200
O	14.50000	-9.35200	-7.73200

H	14.48800	-8.40800	-7.57500
H	15.39300	-9.53500	-8.02600
O	12.67400	10.57200	3.69300
H	13.57700	10.60400	4.01000
H	12.44800	9.64200	3.70800
O	14.42900	9.32500	0.35400
H	13.47900	9.32500	0.24100
H	14.73300	8.57800	-0.16200
O	18.31600	-12.75800	-3.47800
H	17.54900	-13.31200	-3.33200
H	18.72700	-12.68600	-2.61600
O	10.32000	-6.47000	10.61300
H	11.18300	-6.82600	10.82300
H	9.74600	-6.80800	11.30000
O	30.16500	15.16100	1.41900
H	30.80200	15.04100	0.71500
H	30.04700	16.10900	1.47800
O	26.97400	15.11300	1.35500
H	26.63500	14.99800	0.46700
H	27.84200	14.71300	1.33500
O	22.83200	16.96700	-2.65500
H	22.26900	16.87600	-3.42300
H	22.71700	16.14700	-2.17500
O	27.45500	16.26700	7.31400
H	27.44400	15.87200	6.44200
H	27.61800	15.53400	7.90700
O	20.37900	18.46200	0.18300
H	21.17100	18.12100	-0.23200
H	20.61800	18.57200	1.10400
O	28.41200	20.63400	-7.34400
H	27.76100	21.21900	-7.73000
H	29.20700	21.16500	-7.28400
O	22.33800	17.72300	-6.94500
H	23.11100	17.17600	-7.08400
H	22.69000	18.58700	-6.73300
O	24.17600	19.05100	6.26200
H	23.88500	19.95500	6.14600
H	24.81200	19.09200	6.97600
O	25.10200	16.42800	2.85200
H	25.69800	16.20400	2.13800
H	25.15900	15.68700	3.45400
O	24.67400	13.88200	-9.31300
H	24.92700	13.36000	-10.07500
H	23.73700	13.71900	-9.21000
O	29.71900	12.88600	-1.50600
H	30.50500	12.46800	-1.85700
H	29.98300	13.79000	-1.33700
O	29.89000	18.32300	-6.80700
H	29.25400	18.39700	-6.09500
H	29.55900	18.91200	-7.48500
O	35.12000	12.82300	-0.60200
H	35.85200	12.25300	-0.36900
H	34.36300	12.43600	-0.16200
O	24.39400	15.78500	-7.11100
H	24.27800	15.11000	-6.44300
H	24.50400	15.29800	-7.92700
O	26.73700	15.24600	-3.40600
H	26.37300	14.71900	-2.69500
H	27.59400	14.85600	-3.57900
O	32.64200	6.53500	3.85700
H	32.31000	7.40500	3.63300
H	32.04700	6.21900	4.53700
O	26.37800	10.27500	-8.41000
H	26.54500	11.14300	-8.04300
H	26.18700	10.43800	-9.33400
O	29.13500	18.73700	3.25700
H	28.83500	19.63900	3.14800
H	28.79000	18.47000	4.10900
O	22.58600	15.18800	-0.71000
H	21.66200	15.05600	-0.50200
H	22.83500	15.96800	-0.21300
O	32.53500	11.25500	0.93600
H	31.62700	11.18700	0.64200
H	32.48400	11.73200	1.76500
O	26.35700	12.73100	-7.42500
H	26.23700	13.17700	-6.58700
H	25.66200	13.07900	-7.98400
O	19.94700	14.83100	0.12500
H	20.07700	15.08500	1.03900
H	19.26700	15.42400	-0.19500
O	24.11700	25.50100	-3.77600
H	24.04000	26.45200	-3.69700
H	23.51300	25.15600	-3.11900
O	26.60900	12.67400	8.88000
H	25.83200	13.23300	8.87000
H	27.24800	13.16000	9.40100
O	34.40100	9.45700	2.71400
H	33.75900	9.75000	2.06800
H	33.89700	9.34200	3.52000
O	32.05500	13.20300	-6.59700
H	32.96700	13.43600	-6.77200
H	31.54600	13.78700	-7.16000
O	24.86400	15.48300	8.44200
H	25.60500	16.02100	8.16500
H	24.15700	16.10900	8.59900
O	30.33900	17.62500	7.03900
H	30.74800	17.18400	6.29400
H	29.61700	17.05100	7.29200
O	26.69200	12.56300	4.41200
H	27.53800	12.98700	4.55600
H	26.63200	11.90600	5.10600
O	30.66200	18.18600	0.79400
H	30.03300	18.35000	0.09200
H	30.27500	18.60000	1.56500
O	34.54100	19.12100	-1.85100
H	33.85600	19.50100	-2.40200
H	34.62500	19.73100	-1.11800
O	29.84200	10.22300	6.07000
H	29.59800	10.97700	5.53400
H	30.11800	10.60200	6.90500
O	31.79600	16.76700	4.74800
H	32.00800	17.41000	4.07200
H	31.74900	15.93300	4.28000
O	29.39900	9.37900	1.65200
H	28.53700	9.67700	1.94400
H	29.42500	9.59600	0.72000
O	26.21500	17.92300	-1.94100
H	26.67900	18.20200	-2.73000

H	25.95100	17.02100	-2.12300
O	23.10700	17.47000	0.99700
H	23.67700	17.89900	0.35900
H	23.69800	17.18800	1.69600
O	25.15200	14.04500	-5.13200
H	25.85400	14.49700	-4.66400
H	24.82000	13.40400	-4.50400
O	23.60000	12.63600	9.24300
H	22.97000	13.31800	9.47700
H	23.49300	12.52500	8.29900
O	18.89400	12.30400	0.17000
H	18.10400	12.55900	-0.30600
H	19.38500	13.11800	0.27500
O	32.91100	19.64400	2.92000
H	33.73200	19.24200	2.63900
H	32.90700	19.54100	3.87200
O	20.84600	12.66800	-2.87200
H	20.04900	12.78000	-2.35300
H	20.61800	13.00700	-3.73800
O	26.67600	19.19300	5.07600
H	26.74100	19.65500	5.91200
H	25.96700	18.56200	5.20500
O	17.17800	12.55700	2.60100
H	17.42900	13.44100	2.86700
H	17.71400	12.37700	1.82900
O	41.00100	15.98800	-0.73100
H	40.24800	15.41900	-0.88700
H	41.13600	15.95400	0.21600
O	26.39100	20.85900	-5.05600
H	26.25600	21.70500	-4.62700
H	26.93200	21.06100	-5.81900
O	22.87800	6.93900	11.60600
H	22.48500	6.38500	12.28100
H	23.69400	7.25000	11.99600
O	19.84400	18.37700	-4.48300
H	20.35700	19.17100	-4.33200
H	20.49600	17.68000	-4.54900
O	21.65400	21.49400	-2.40100
H	21.83000	22.41500	-2.58800
H	22.44900	21.17900	-1.97100
O	25.79500	8.35400	9.19800
H	26.53600	8.95500	9.11800
H	26.02600	7.61200	8.64000
O	20.93900	13.54000	7.47600
H	20.82400	12.63900	7.77600
H	20.08300	13.78600	7.12400
O	28.01800	21.48200	9.07400
H	27.57400	20.69500	8.75700
H	28.68000	21.67000	8.40900
O	22.77700	23.01100	3.60000
H	23.32700	22.88300	2.82800
H	21.93800	23.31100	3.25000
O	30.11600	21.76200	7.10300
H	30.09800	22.58300	6.61100
H	31.03800	21.63700	7.32800
O	26.57300	19.27600	7.94500
H	27.10600	18.51500	7.71500
H	26.04500	18.98400	8.68700
O	29.74000	4.38200	3.47400
H	29.39700	5.27300	3.39800
H	30.10800	4.19300	2.61100
O	17.80900	16.70600	-3.70700
H	18.64600	17.11600	-3.92400
H	17.98700	15.76600	-3.73700
O	29.70500	14.37300	-8.01800
H	29.19000	15.14400	-7.78000
H	29.43300	13.70000	-7.39500
O	27.29000	9.21800	-2.96000
H	26.43400	8.82900	-3.13900
H	27.70300	9.29700	-3.82000
O	28.82000	6.75300	1.43900
H	28.31700	6.71600	0.62600
H	29.11300	7.66200	1.49900
O	25.06400	6.83700	0.44600
H	25.93000	6.64100	0.08600
H	25.13200	6.60800	1.37300
O	34.67900	17.12700	-6.08300
H	33.90200	16.78800	-6.52800
H	35.35800	17.13200	-6.75800
O	29.49400	12.24600	-6.19300
H	29.18900	12.56100	-5.34100
H	30.44700	12.32400	-6.15000
O	27.93900	18.40500	-4.54300
H	27.41100	17.67000	-4.85600
H	27.43700	19.18300	-4.78900
O	32.91800	16.60000	0.57300
H	32.26000	17.29000	0.66200
H	33.72200	16.99000	0.91700
O	22.67100	9.06700	-2.18300
H	23.24500	8.84500	-2.91500
H	23.25800	9.13600	-1.43000
O	31.72900	14.95400	-1.37600
H	32.24200	15.58700	-0.87400
H	32.37900	14.35500	-1.74200
O	22.99800	11.09700	-8.16300
H	22.16100	11.43500	-7.84200
H	22.85800	10.96200	-9.10000
O	24.97300	5.94100	-3.45500
H	24.31900	5.62600	-4.07900
H	24.93100	5.31900	-2.72900
O	26.08400	16.22300	-11.54000
H	25.17700	15.99200	-11.34100
H	26.58100	15.42700	-11.35200
O	28.99100	24.93300	-8.62000
H	28.31700	25.23600	-8.01200
H	29.37700	25.73600	-8.97000
O	28.20700	12.42900	0.87000
H	27.28900	12.65100	0.71200
H	28.62300	12.50400	0.01100
O	28.99000	13.82800	-4.00000
H	29.73700	14.34100	-4.30900
H	29.25900	13.50700	-3.14000
O	27.26300	16.32400	-7.01700
H	26.35400	16.04000	-6.92000
H	27.29200	16.74400	-7.87700
O	32.49500	19.93500	-3.24800
H	32.04100	19.09400	-3.19200
H	31.86400	20.57100	-2.91100

O	31.43800	10.86400	-2.01800
H	30.70000	10.35200	-1.68700
H	31.69100	10.42400	-2.83000
O	30.93800	21.77300	-2.31300
H	31.23000	22.33300	-3.03200
H	31.44800	22.06500	-1.55800
O	31.53900	3.64200	-1.42400
H	31.84500	2.78200	-1.71100
H	32.26400	4.23400	-1.62400
O	17.83200	14.04500	-3.03600
H	17.63400	13.45200	-3.76100
H	17.27600	13.74000	-2.31800
O	25.81100	13.93700	-0.91600
H	25.86600	12.98500	-0.99200
H	25.00000	14.09300	-0.43200
O	26.96000	17.86500	-9.67900
H	26.52200	17.14900	-10.13800
H	27.70100	18.09300	-10.24000
O	23.75000	12.55500	-3.15700
H	22.93700	12.85400	-2.75100
H	24.24600	12.16000	-2.44000
O	25.90100	22.55100	-1.22400
H	25.85900	22.77700	-2.15300
H	26.81800	22.31700	-1.07500
O	29.34800	13.01900	5.67400
H	29.70300	13.24600	6.53400
H	29.99500	13.35100	5.05200
O	35.07800	16.14100	-3.62100
H	34.93900	16.33100	-4.54900
H	34.72100	16.90300	-3.16400
O	23.24200	10.12700	2.45900
H	23.88200	9.98600	1.76100
H	22.41000	9.84000	2.08300
O	35.10500	18.20900	1.39000
H	35.53800	18.11600	2.23900
H	35.81800	18.18400	0.75200
O	24.01800	19.42700	-1.42800
H	24.81200	18.93200	-1.62900
H	23.30700	18.89900	-1.79100
O	28.83700	21.80900	2.92400
H	28.89100	22.37800	3.69100
H	27.96900	21.97900	2.55900
O	20.32600	13.57100	-5.35200
H	19.39600	13.51000	-5.13500
H	20.42200	13.04900	-6.14800
O	20.24500	10.09700	-2.76400
H	20.50500	11.01100	-2.87300
H	21.06600	9.62900	-2.61400
O	21.71700	11.51100	4.40600
H	22.39000	11.30200	3.75700
H	20.89000	11.31300	3.96700
O	35.96600	18.25100	3.92400
H	36.16000	19.15600	4.16900
H	35.86600	17.79200	4.75700
O	27.62000	8.13700	6.42300
H	26.97300	8.83600	6.32900
H	28.39000	8.57000	6.79000
O	31.02800	18.40200	9.49800
H	30.87600	18.17300	8.58100
H	31.01800	19.35900	9.51100
O	27.79600	15.97500	4.48200
H	28.72500	16.12600	4.30900
H	27.37500	16.06300	3.62700
O	26.59500	20.00000	2.25200
H	26.45600	19.63200	3.12500
H	25.80700	20.51500	2.08100
O	27.19000	25.10100	-6.40500
H	27.36500	24.63000	-5.59100
H	26.50300	25.72700	-6.17700
O	28.14600	19.37800	-0.35900
H	27.81100	19.33800	0.53700
H	27.64600	18.71300	-0.83200
O	22.12000	14.78500	5.16600
H	21.98200	14.05300	5.76700
H	23.03000	14.69600	4.88500
O	31.08100	16.72400	-10.08500
H	30.46400	15.99800	-10.18200
H	30.61300	17.48500	-10.42900
O	24.74800	14.49700	4.65500
H	25.45800	13.85600	4.61300
H	24.70500	14.74800	5.57700
O	32.73700	21.43400	7.57500
H	32.67800	20.74900	6.90800
H	33.13900	22.17500	7.12300
O	23.46100	11.48300	6.47400
H	22.92500	11.03200	7.12600
H	22.87300	11.61800	5.73100
O	21.75600	28.25000	0.36900
H	21.99600	28.88200	1.04700
H	20.91300	27.89900	0.65500
O	22.87100	10.94200	-10.96300
H	21.99400	10.82100	-11.32700
H	23.34700	10.15100	-11.21500
O	32.73900	19.39700	5.80700
H	31.85100	19.06000	5.92900
H	33.30800	18.66600	6.04400
O	14.39200	15.73000	-0.86900
H	15.07900	15.41600	-1.45700
H	14.23600	16.63400	-1.14200
O	19.19500	10.61900	3.55900
H	19.11100	10.01100	2.82500
H	18.37300	11.11000	3.56000
O	26.26900	24.05800	2.77300
H	26.19600	24.86300	3.28500
H	25.82600	24.25700	1.94800
O	26.05800	23.29500	-3.71700
H	25.27400	23.83700	-3.62300
H	26.75700	23.81200	-3.31700
O	29.36700	9.10400	-1.07400
H	29.86400	8.35100	-1.39300
H	28.59600	9.14100	-1.63900
O	37.22000	14.80800	0.56800
H	37.20900	15.69100	0.19800
H	36.35200	14.45500	0.37400
O	28.72900	23.95600	4.92900
H	29.44100	24.59100	4.85600
H	28.23800	24.05300	4.11300
O	30.45000	17.86100	-3.50000

H	29.51600	17.98200	-3.66900
H	30.62800	16.95900	-3.76400
O	25.95100	11.24600	-1.34600
H	25.58100	10.69300	-0.65800
H	26.44700	10.64200	-1.89900
O	33.83800	13.70300	-2.96500
H	34.30400	14.53600	-3.03900
H	34.28800	13.23800	-2.26000
O	23.78000	21.70600	5.74900
H	24.48100	22.33900	5.90200
H	23.30900	22.04700	4.98900
O	31.90500	20.04800	-5.96600
H	32.18100	19.93300	-5.05700
H	31.03600	19.64900	-6.00500
O	31.80900	6.07100	1.07400
H	30.89600	6.30200	1.24300
H	32.23900	6.15500	1.92600
O	31.58000	14.26000	3.57400
H	30.92700	14.50800	2.92000
H	31.95800	13.44700	3.24100
O	28.54100	24.29600	-2.32300
H	28.72300	23.47800	-1.86000
H	29.36400	24.51300	-2.76000
O	28.87500	23.22300	-5.06100
H	29.40400	22.71200	-5.67400
H	29.13800	22.90700	-4.19700
O	27.62900	15.00200	10.06100
H	26.95300	14.90200	10.73100
H	28.45100	14.84600	10.52500
O	19.37700	21.23900	2.68200
H	19.96200	20.49400	2.81500
H	19.17900	21.55000	3.56600
O	22.23000	24.27500	-2.30100
H	21.56100	24.75900	-2.78500
H	22.09200	24.52100	-1.38700
O	13.96800	13.81000	1.40300
H	14.00700	13.27600	2.19600
H	13.46200	13.28300	0.78400
O	26.37100	26.30900	4.29700
H	26.92300	26.47200	5.06200
H	26.71200	26.89900	3.62500
O	21.34700	8.65600	4.89900
H	21.41200	9.51800	4.48700
H	21.28400	8.04400	4.16600
O	20.82300	10.72900	8.54600
H	21.45000	10.34600	9.16000
H	19.96500	10.51000	8.91200
O	31.22200	21.13500	10.15200
H	31.76600	21.74800	9.65700
H	30.64100	21.69300	10.66800
O	17.59900	12.53600	-5.52200
H	17.89500	11.62700	-5.57500
H	16.69800	12.51500	-5.84400
O	29.50800	24.25700	1.28300
H	29.38300	23.45600	0.77400
H	30.16600	24.75200	0.79500
O	21.12900	10.61200	0.46100
H	20.40700	11.22600	0.32200
H	21.91800	11.14800	0.38300
O	31.57100	22.05800	2.91100
H	30.62400	21.93900	2.83900
H	31.92000	21.17000	2.99400
O	23.93100	7.94700	6.73500
H	24.45500	8.19400	7.49800
H	23.71200	8.77900	6.31500
O	19.32800	10.21800	-5.39800
H	18.99900	9.33800	-5.58000
H	19.68200	10.16300	-4.51000
O	28.57600	21.85300	-0.88800
H	28.42800	20.92500	-0.70800
H	29.40100	21.87500	-1.37300
O	22.44200	10.50500	-5.03000
H	22.78200	11.03400	-5.75300
H	22.88900	10.84300	-4.25500
O	32.17900	22.67600	0.19800
H	33.10400	22.47000	0.06000
H	32.01100	22.42800	1.10700
O	25.13700	8.88900	4.42000
H	24.79600	8.09000	4.82200
H	24.43000	9.18900	3.84800
O	25.78800	5.97400	2.87300
H	26.61100	5.50000	2.75100
H	26.03200	6.75300	3.37300
O	24.42900	9.00300	-6.83100
H	25.15000	9.38100	-7.33500
H	23.80600	9.72100	-6.72800
O	28.57200	9.70700	-5.89500
H	28.78800	10.59000	-6.19600
H	28.09200	9.31600	-6.62500
O	23.31200	12.66600	0.68200
H	23.16600	12.98000	1.57400
H	22.83500	13.28400	0.12900
O	25.17200	9.65600	0.73000
H	25.15800	8.76000	0.39300
H	25.88900	9.66300	1.36400
O	27.02400	10.09000	2.77500
H	26.76600	11.01100	2.77400
H	26.49300	9.69300	3.46500
O	31.96900	23.19500	-4.44600
H	32.14300	22.62900	-5.19800
H	32.46200	23.99500	-4.62800
O	17.78700	15.20600	3.62100
H	17.24200	15.98200	3.75300
H	18.66500	15.55300	3.46300
O	24.57500	8.53700	-4.00100
H	24.69700	7.60900	-3.80200
H	24.28600	8.55100	-4.91300
O	27.44400	6.22300	-0.86500
H	27.46700	6.28700	-1.81900
H	27.86100	5.38300	-0.67100
O	37.82300	15.95800	-2.82700
H	38.26600	15.20900	-3.22500
H	36.94500	15.94900	-3.20900
O	32.95600	22.51100	-7.34600
H	33.65200	23.13200	-7.12700
H	33.29900	21.66100	-7.07100
O	18.57300	14.33300	6.51400
H	17.83100	14.48100	7.10000

H	18.25200	14.59400	5.65000
O	34.85600	19.96100	-5.81700
H	35.38100	20.30000	-6.54200
H	34.76800	19.02600	-6.00100
O	26.51800	23.87500	6.31200
H	26.82600	23.63000	7.18400
H	27.31500	24.07900	5.82400
O	26.54000	26.02900	-1.45800
H	26.10000	25.53600	-0.76500
H	27.29000	25.48600	-1.69900
O	19.01900	22.22000	-1.45500
H	19.84500	21.92000	-1.83500
H	18.66800	21.45000	-1.00600
O	23.98700	21.57200	0.61400
H	23.36500	20.90900	0.31500
H	24.65700	21.59800	-0.06800
O	17.22000	20.13800	-0.49600
H	16.38300	19.71100	-0.67800
H	17.19200	20.33200	0.44100
O	24.05900	10.05100	10.20800
H	24.10800	10.92700	9.82500
H	24.60200	9.50800	9.63500
O	34.54500	20.59500	9.48800
H	33.75500	20.57700	8.94800
H	34.30800	21.13300	10.24300
O	34.66500	17.44400	6.80100
H	34.71500	17.27900	7.74300
H	34.55600	16.57700	6.41100
O	23.54800	20.12300	-5.91000
H	23.69700	20.80600	-6.56300
H	24.35500	20.10300	-5.39500
O	31.95500	13.42700	8.11700
H	32.85600	13.28800	7.82500
H	32.03000	13.59200	9.05700
O	26.28900	10.80400	6.96200
H	25.42000	11.09700	6.69100
H	26.52500	11.38800	7.68300
O	36.16700	19.86400	7.04500
H	35.87500	20.22700	7.88200
H	35.55400	19.15000	6.86700
O	37.36600	17.42900	-0.43400
H	38.20100	17.86800	-0.27100
H	37.50200	16.95300	-1.25400
O	34.41500	14.98700	5.51300
H	34.89100	15.18000	4.70500
H	33.49200	15.04300	5.26800
O	21.30400	16.14900	-4.90400
H	21.05800	15.24500	-4.70600
H	21.47300	16.15100	-5.84600
O	28.99300	7.02200	4.47900
H	29.51100	7.75800	4.15300
H	28.63900	7.32800	5.31400
O	20.57300	11.88200	-7.40500
H	19.88000	11.95700	-8.06100
H	20.32800	11.11800	-6.88300
O	29.24500	3.44200	-0.00100
H	29.52500	2.77400	0.62500
H	30.03000	3.63500	-0.51400
O	27.04800	22.61800	-8.43900
H	26.47500	23.23700	-8.89200
H	27.62100	23.16700	-7.90500
O	33.11500	11.86300	3.74600
H	34.06700	11.96300	3.76700
H	32.94600	11.07100	4.25600
O	31.13700	8.94900	3.93200
H	30.54200	9.22900	3.23700
H	30.85200	9.43800	4.70400
O	30.26800	22.90100	-7.30800
H	31.20100	22.83600	-7.10400
H	30.18000	23.73700	-7.76600
O	32.89600	22.21800	-10.34700
H	32.81800	21.88400	-9.45400
H	33.71700	22.71000	-10.34800
O	35.59400	15.08400	2.86000
H	35.56200	14.14600	3.04600
H	36.20400	15.16200	2.12700
O	27.43300	13.95400	-11.09700
H	28.37800	13.82400	-11.02800
H	27.05400	13.21500	-10.62000
O	35.74400	11.87500	2.90600
H	35.22800	11.09100	2.71900
H	36.62800	11.54700	3.07100
O	33.40500	5.61900	-1.13400
H	33.20700	5.31100	-0.25000
H	34.01500	6.34500	-1.00400
O	41.41200	16.14600	2.00100
H	42.14200	15.88400	2.56200
H	40.80300	15.40800	2.04100
O	39.49300	14.28600	2.59800
H	38.76700	14.15200	1.99000
H	39.07800	14.59400	3.40400
O	27.31600	18.00500	10.36300
H	28.23700	18.18300	10.55700
H	27.28100	17.06100	10.21100
O	25.61700	11.87900	-11.16100
H	24.71000	11.58200	-11.08500
H	25.89400	11.58200	-12.02800
O	16.53500	13.19900	-0.71200
H	16.21500	12.34600	-1.00400
H	15.98800	13.41400	0.04400
O	20.31300	15.91600	3.00500
H	20.53100	16.84700	3.02400
H	20.72500	15.55600	3.79100
O	27.23300	6.42200	-5.00700
H	26.42300	6.07700	-4.63200
H	27.92200	6.11300	-4.41800
O	29.16200	5.50000	-3.38500
H	29.71500	5.92000	-2.72600
H	29.39900	4.57400	-3.34200
O	25.18500	11.48900	12.39600
H	25.08500	12.43800	12.32700
H	24.96800	11.16100	11.52300
O	24.38700	8.73700	-11.02000
H	25.24900	9.06300	-10.76100
H	24.57200	7.95000	-11.53300
O	16.40300	16.82000	1.25800
H	15.71000	16.40500	0.74500
H	17.15100	16.86200	0.66100

O	32.54000	7.61000	-3.87700
H	31.96000	8.26900	-4.25900
H	32.27400	6.78800	-4.28800
O	30.99000	7.01100	-1.67500
H	31.49000	7.12200	-2.48300
H	31.63400	6.70700	-1.03500
O	25.04900	14.91900	11.21200
H	24.56500	15.71900	11.41900
H	25.13500	14.92900	10.25900
O	18.31700	16.75100	-0.86600
H	18.29400	16.85000	-1.81700
H	18.62700	17.59800	-0.54400
O	20.97600	18.80900	3.24900
H	21.93000	18.77700	3.18200
H	20.77700	18.34800	4.06400
O	21.56600	17.37900	5.81600
H	22.38500	17.87400	5.84000
H	21.84000	16.46200	5.78800
O	31.32300	9.77000	-4.82100
H	30.43500	10.05500	-5.03900
H	31.73800	9.61000	-5.66800
O	34.67400	9.79500	-1.10600
H	35.45000	10.28400	-0.83300
H	33.94900	10.22100	-0.64800
O	37.11100	10.50800	-0.04800
H	37.70600	10.26700	-0.75900
H	37.18400	9.78800	0.57900
O	19.33100	24.35100	0.26600
H	19.19600	23.59100	-0.30000
H	18.91400	25.07500	-0.20100
O	30.46700	19.53500	-10.72600
H	31.17000	19.74000	-10.11100
H	29.74300	20.10200	-10.46100
O	31.29700	15.28600	-4.60600
H	31.24000	15.74200	-5.44500
H	31.84900	14.52500	-4.78400
O	35.10700	11.07500	-3.78100
H	34.85100	10.67400	-2.95000
H	34.48200	11.78900	-3.90600
O	39.41800	13.81400	-1.27400
H	38.85800	13.88400	-0.50100
H	38.81300	13.63800	-1.99400
O	22.48800	25.24800	0.47800
H	22.37100	26.19200	0.37600
H	21.66700	24.94500	0.86600
O	32.37500	19.97300	-8.67600
H	33.21000	19.54100	-8.85900
H	32.21400	19.80100	-7.74900
O	32.03000	16.47600	-7.31100
H	31.97500	16.31100	-8.25200
H	31.40500	17.18400	-7.15500
O	16.81900	11.71300	6.51500
H	17.46500	11.20300	7.00400
H	16.61100	12.45000	7.09000
O	29.34500	18.78100	12.01500
H	30.21400	18.95500	11.65300
H	28.86500	19.60000	11.89200
O	25.34000	5.97700	-7.73200
H	25.32800	6.92100	-7.57500
H	26.23200	5.79400	-8.02600
O	23.51300	25.90100	3.69300
H	24.41600	25.93300	4.01000
H	23.28700	24.97100	3.70800
O	25.26900	24.65400	0.35400
H	24.31800	24.65400	0.24100
H	25.57200	23.90700	-0.16200
O	29.15500	2.57100	-3.47800
H	28.38800	2.01700	-3.33200
H	29.56600	2.64300	-2.61600
O	21.15900	8.86000	10.61300
H	22.02200	8.50300	10.82300
H	20.58600	8.52100	11.30000
O	41.00400	30.49000	1.41900
H	41.64200	30.37100	0.71500
H	40.88600	31.43800	1.47800
O	37.81300	30.44300	1.35500
H	37.47500	30.32700	0.46700
H	38.68200	30.04200	1.33500
O	33.67200	32.29600	-2.65500
H	33.10800	32.20500	-3.42300
H	33.55700	31.47600	-2.17500
O	38.29500	31.59600	7.31400
H	38.28400	31.20100	6.44200
H	38.45700	30.86300	7.90700
O	31.21800	33.79100	0.18300
H	32.01000	33.45000	-0.23200
H	31.45800	33.90100	1.10400
O	33.17700	33.05200	-6.94500
H	33.95000	32.50500	-7.08400
H	33.53000	33.91700	-6.73300
O	35.01600	34.38000	6.26200
H	34.72500	35.28400	6.14600
H	35.65100	34.42100	6.97600
O	35.94100	31.75700	2.85200
H	36.53800	31.53300	2.13800
H	35.99900	31.01600	3.45400
O	35.51400	29.21100	-9.31300
H	35.76600	28.69000	-10.07500
H	34.57600	29.04800	-9.21000
O	40.55800	28.21500	-1.50600
H	41.34400	27.79700	-1.85700
H	40.82200	29.12000	-1.33700
O	35.23300	31.11400	-7.11100
H	35.11700	30.43900	-6.44300
H	35.34400	30.62700	-7.92700
O	37.57700	30.57500	-3.40600
H	37.21200	30.04800	-2.69500
H	38.43300	30.18500	-3.57900
O	37.21700	25.60400	-8.41000
H	37.38400	26.47200	-8.04300
H	37.02700	25.76700	-9.33400
O	33.42600	30.51700	-0.71000
H	32.50100	30.38500	-0.50200
H	33.67400	31.29700	-0.21300
O	37.19600	28.06000	-7.42500
H	37.07600	28.50600	-6.58700
H	36.50100	28.40900	-7.98400
O	30.78700	30.16100	0.12500

H	30.91600	30.41400	1.03900
H	30.10600	30.75300	-0.19500
O	37.44800	28.00300	8.88000
H	36.67200	28.56300	8.87000
H	38.08700	28.48900	9.40100
O	35.70300	30.81200	8.44200
H	36.44400	31.35000	8.16500
H	34.99700	31.43800	8.59900
O	37.53200	27.89200	4.41200
H	38.37800	28.31600	4.55600
H	37.47200	27.23500	5.10600
O	40.68200	25.55200	6.07000
H	40.43800	26.30700	5.53400
H	40.95800	25.93100	6.90500
O	40.23800	24.70800	1.65200
H	39.37700	25.00600	1.94400
H	40.26400	24.92500	0.72000
O	37.05400	33.25200	-1.94100
H	37.51800	33.53100	-2.73000
H	36.79000	32.35100	-2.12300
O	33.94700	32.79900	0.99700
H	34.51700	33.22800	0.35900
H	34.53700	32.51800	1.69600
O	35.99100	29.37400	-5.13200
H	36.69400	29.82700	-4.66400
H	35.65900	28.73300	-4.50400
O	34.43900	27.96600	9.24300
H	33.80900	28.64700	9.47700
H	34.33300	27.85400	8.29900
O	29.73400	27.63300	0.17000
H	28.94400	27.88900	-0.30600
H	30.22500	28.44800	0.27500
O	31.68500	27.99700	-2.87200
H	30.88800	28.10900	-2.35300
H	31.45800	28.33600	-3.73800
O	28.01700	27.88600	2.60100
H	28.26800	28.77100	2.86700
H	28.55300	27.70600	1.82900
O	33.71700	22.26800	11.60600
H	33.32400	21.71400	12.28100
H	34.53400	22.57900	11.99600
O	30.68300	33.70600	-4.48300
H	31.19600	34.50000	-4.33200
H	31.33600	33.00900	-4.54900
O	36.63400	23.68400	9.19800
H	37.37500	24.28400	9.11800
H	36.86600	22.94100	8.64000
O	31.77800	28.86900	7.47600
H	31.66400	27.96800	7.77600
H	30.92300	29.11600	7.12400
O	40.58000	19.71200	3.47400
H	40.23600	20.60200	3.39800
H	40.94800	19.52200	2.61100
O	28.64800	32.03500	-3.70700
H	29.48600	32.44500	-3.92400
H	28.82600	31.09500	-3.73700
O	40.54500	29.70300	-8.01800
H	40.02900	30.47300	-7.78000
H	40.27300	29.02900	-7.39500
O	38.12900	24.54700	-2.96000
H	37.27400	24.15800	-3.13900
H	38.54200	24.62700	-3.82000
O	39.65900	22.08200	1.43900
H	39.15600	22.04500	0.62600
H	39.95200	22.99100	1.49900
O	35.90400	22.16600	0.44600
H	36.76900	21.97000	0.08600
H	35.97100	21.93700	1.37300
O	40.33300	27.57500	-6.19300
H	40.02800	27.89000	-5.34100
H	41.28600	27.65300	-6.15000
O	33.51000	24.39600	-2.18300
H	34.08400	24.17400	-2.91500
H	34.09700	24.46500	-1.43000
O	33.83700	26.42700	-8.16300
H	33.00100	26.76400	-7.84200
H	33.69700	26.29200	-9.10000
O	35.81200	21.27000	-3.45500
H	35.15900	20.95500	-4.07900
H	35.77000	20.64800	-2.72900
O	36.92300	31.55200	-11.54000
H	36.01600	31.32100	-11.34100
H	37.42000	30.75600	-11.35200
O	39.04600	27.75900	0.87000
H	38.12800	27.98000	0.71200
H	39.46200	27.83300	0.01100
O	39.82900	29.15800	-4.00000
H	40.57700	29.67000	-4.30900
H	40.09900	28.83600	-3.14000
O	38.10200	31.65400	-7.01700
H	37.19300	31.36900	-6.92000
H	38.13200	32.07300	-7.87700
O	42.27700	26.19300	-2.01800
H	41.54000	25.68100	-1.68700
H	42.53000	25.75300	-2.83000
O	28.67100	29.37400	-3.03600
H	28.47300	28.78100	-3.76100
H	28.11600	29.06900	-2.31800
O	36.65100	29.26600	-0.91600
H	36.70500	28.31400	-0.99200
H	35.84000	29.42200	-0.43200
O	37.80000	33.19400	-9.67900
H	37.36200	32.47800	-10.13800
H	38.54100	33.42200	-10.24000
O	34.59000	27.88400	-3.15700
H	33.77700	28.18400	-2.75100
H	35.08600	27.49000	-2.44000
O	40.18700	28.34800	5.67400
H	40.54200	28.57500	6.53400
H	40.83400	28.68000	5.05200
O	34.08100	25.45600	2.45900
H	34.72100	25.31500	1.76100
H	33.24900	25.16900	2.08300
O	34.85700	34.75600	-1.42800
H	35.65100	34.26100	-1.62900
H	34.14600	34.22800	-1.79100
O	31.16600	28.90000	-5.35200
H	30.23500	28.83900	-5.13500

H	31.26100	28.37800	-6.14800
O	31.08400	25.42600	-2.76400
H	31.34500	26.34000	-2.87300
H	31.90600	24.95800	-2.61400
O	32.55700	26.84000	4.40600
H	33.22900	26.63100	3.75700
H	31.73000	26.64200	3.96700
O	38.45900	23.46600	6.42300
H	37.81200	24.16500	6.32900
H	39.22900	23.90000	6.79000
O	38.63600	31.30400	4.48200
H	39.56500	31.45500	4.30900
H	38.21500	31.39200	3.62700
O	32.95900	30.11400	5.16600
H	32.82100	29.38200	5.76700
H	33.87000	30.02500	4.88500
O	35.58800	29.82600	4.65500
H	36.29800	29.18500	4.61300
H	35.54500	30.07700	5.57700
O	34.30000	26.81300	6.47400
H	33.76400	26.36200	7.12600
H	33.71200	26.94700	5.73100
O	33.71100	26.27100	-10.96300
H	32.83400	26.15000	-11.32700
H	34.18600	25.48000	-11.21500
O	25.23100	31.05900	-0.86900
H	25.91800	30.74500	-1.45700
H	25.07600	31.96300	-1.14200
O	30.03400	25.94800	3.55900
H	29.95000	25.34100	2.82500
H	29.21300	26.43900	3.56000
O	40.20700	24.43300	-1.07400
H	40.70400	23.68000	-1.39300
H	39.43500	24.47000	-1.63900
O	36.79000	26.57500	-1.34600
H	36.42000	26.02200	-0.65800
H	37.28600	25.97100	-1.89900
O	42.41900	29.58900	3.57400
H	41.76600	29.83700	2.92000
H	42.79700	28.77600	3.24100
O	38.46800	30.33100	10.06100
H	37.79200	30.23100	10.73100
H	39.29100	30.17500	10.52500
O	30.21600	36.56900	2.68200
H	30.80100	35.82300	2.81500
H	30.01800	36.87900	3.56600
O	24.80800	29.13900	1.40300
H	24.84600	28.60600	2.19600
H	24.30200	28.61300	0.78400
O	32.18700	23.98600	4.89900
H	32.25100	24.84700	4.48700
H	32.12400	23.37300	4.16600
O	31.66200	26.05800	8.54600
H	32.28900	25.67600	9.16000
H	30.80500	25.83900	8.91200
O	28.43900	27.86600	-5.52200
H	28.73400	26.95700	-5.57500
H	27.53700	27.84400	-5.84400
O	31.96800	25.94100	0.46100
H	31.24700	26.55500	0.32200
H	32.75700	26.47700	0.38300
O	34.77100	23.27700	6.73500
H	35.29400	23.52300	7.49800
H	34.55200	24.10900	6.31500
O	30.16700	25.54700	-5.39800
H	29.83900	24.66700	-5.58000
H	30.52100	25.49200	-4.51000
O	33.28200	25.83400	-5.03000
H	33.62100	26.36300	-5.75300
H	33.72900	26.17200	-4.25500
O	35.97600	24.21800	4.42000
H	35.63600	23.41900	4.82200
H	35.27000	24.51800	3.84800
O	36.62800	21.30300	2.87300
H	37.45000	20.82900	2.75100
H	36.87100	22.08200	3.37300
O	35.26800	24.33200	-6.83100
H	35.98900	24.71000	-7.33500
H	34.64500	25.05100	-6.72800
O	39.41100	25.03600	-5.89500
H	39.62800	25.91900	-6.19600
H	38.93100	24.64500	-6.62500
O	34.15100	27.99500	0.68200
H	34.00600	28.30900	1.57400
H	33.67400	28.61300	0.12900
O	36.01100	24.98500	0.73000
H	35.99700	24.08900	0.39300
H	36.72800	24.99200	1.36400
O	37.86400	25.41900	2.77500
H	37.60500	26.34100	2.77400
H	37.33200	25.02200	3.46500
O	28.62600	30.53500	3.62100
H	28.08100	31.31100	3.75300
H	29.50400	30.88200	3.46300
O	35.41500	23.86700	-4.00100
H	35.53600	22.93800	-3.80200
H	35.12500	23.88000	-4.91300
O	38.28400	21.55200	-0.86500
H	38.30600	21.61600	-1.81900
H	38.70100	20.71200	-0.67100
O	29.41300	29.66200	6.51400
H	28.67100	29.81100	7.10000
H	29.09200	29.92300	5.65000
O	29.85800	37.54900	-1.45500
H	30.68400	37.24900	-1.83500
H	29.50800	36.78000	-1.00600
O	28.05900	35.46800	-0.49600
H	27.22200	35.04000	-0.67800
H	28.03100	35.66100	0.44100
O	34.89900	25.38000	10.20800
H	34.94800	26.25600	9.82500
H	35.44100	24.83700	9.63500
O	34.38800	35.45200	-5.91000
H	34.53700	36.13500	-6.56300
H	35.19400	35.43200	-5.39500
O	37.12900	26.13300	6.96200
H	36.25900	26.42600	6.69100
H	37.36500	26.71700	7.68300

O	32.14300	31.47800	-4.90400
H	31.89800	30.57500	-4.70600
H	32.31300	31.48000	-5.84600
O	39.83300	22.35100	4.47900
H	40.35100	23.08700	4.15300
H	39.47800	22.65700	5.31400
O	31.41300	27.21100	-7.40500
H	30.71900	27.28600	-8.06100
H	31.16800	26.44700	-6.88300
O	40.08400	18.77100	-0.00100
H	40.36400	18.10300	0.62500
H	40.86900	18.96400	-0.51400
O	41.97600	24.27800	3.93200
H	41.38100	24.55900	3.23700
H	41.69100	24.76800	4.70400
O	38.27200	29.28300	-11.09700
H	39.21800	29.15300	-11.02800
H	37.89300	28.54400	-10.62000
O	36.45600	27.20800	-11.16100
H	35.54900	26.91100	-11.08500
H	36.73400	26.91100	-12.02800
O	27.37400	28.52800	-0.71200
H	27.05400	27.67500	-1.00400
H	26.82700	28.74300	0.04400
O	31.15200	31.24500	3.00500
H	31.37100	32.17600	3.02400
H	31.56500	30.88500	3.79100
O	38.07200	21.75200	-5.00700
H	37.26300	21.40600	-4.63200
H	38.76100	21.44200	-4.41800
O	40.00200	20.82900	-3.38500
H	40.55500	21.24900	-2.72600
H	40.23900	19.90300	-3.34200
O	36.02500	26.81800	12.39600
H	35.92400	27.76700	12.32700
H	35.80700	26.49000	11.52300
O	35.22600	24.06700	-11.02000
H	36.08800	24.39200	-10.76100
H	35.41100	23.28000	-11.53300
O	27.24300	32.14900	1.25800
H	26.54900	31.73400	0.74500
H	27.99000	32.19100	0.66100
O	41.83000	22.34000	-1.67500
H	42.33000	22.45200	-2.48300
H	42.47300	22.03600	-1.03500
O	35.88800	30.24800	11.21200
H	35.40500	31.04800	11.41900
H	35.97400	30.25900	10.25900
O	29.15700	32.08100	-0.86600
H	29.13400	32.17900	-1.81700
H	29.46700	32.92700	-0.54400
O	31.81500	34.13800	3.24900
H	32.77000	34.10700	3.18200
H	31.61700	33.67700	4.06400
O	32.40500	32.70800	5.81600
H	33.22500	33.20300	5.84000
H	32.67900	31.79100	5.78800
O	42.16200	25.09900	-4.82100
H	41.27400	25.38400	-5.03900
H	42.57700	24.93900	-5.66800
O	27.65900	27.04200	6.51500
H	28.30500	26.53200	7.00400
H	27.45000	27.77900	7.09000
O	36.17900	21.30600	-7.73200
H	36.16700	22.25000	-7.57500
H	37.07100	21.12300	-8.02600
O	39.99400	17.90000	-3.47800
H	39.22800	17.34600	-3.33200
H	40.40500	17.97300	-2.61600
O	31.99800	24.18900	10.61300
H	32.86200	23.83300	10.82300
H	31.42500	23.85000	11.30000
O	-8.40100	-43.48000	-17.05200
H	-8.47800	-42.52900	-16.97300
H	-9.00500	-43.82600	-16.39400
O	-10.86500	-47.48800	-15.67600
H	-10.68800	-46.56600	-15.86400
H	-10.06900	-47.80200	-15.24700
O	-9.74100	-45.97000	-9.67500
H	-9.19100	-46.09800	-10.44700
H	-10.58000	-45.67100	-10.02500
O	-3.52700	-44.04900	-21.89600
H	-4.20100	-43.74500	-21.28700
H	-3.14100	-43.24600	-22.24500
O	-6.61700	-46.43100	-14.49900
H	-6.65900	-46.20500	-15.42800
H	-5.70100	-46.66400	-14.35100
O	-5.32800	-43.88100	-19.68100
H	-5.15300	-44.35200	-18.86600
H	-6.01500	-43.25400	-19.45300
O	-10.76200	-40.73200	-12.90700
H	-10.52200	-40.10000	-12.22900
H	-11.60500	-41.08300	-12.62000
O	-6.24900	-44.92400	-10.50300
H	-6.32200	-44.11800	-9.99100
H	-6.69200	-44.72500	-11.32800
O	-6.46000	-45.68700	-16.99200
H	-7.24400	-45.14400	-16.89900
H	-5.76100	-45.16900	-16.59300
O	-3.79000	-45.02500	-8.34700
H	-3.07700	-44.39000	-8.41900
H	-4.28000	-44.92900	-9.16300
O	-8.73800	-47.27600	-7.52600
H	-8.03700	-46.64200	-7.37300
H	-9.20900	-46.93400	-8.28700
O	-3.97700	-44.68500	-15.59800
H	-3.79500	-45.50300	-15.13500
H	-3.15400	-44.46800	-16.03600
O	-3.64300	-45.75800	-18.33700
H	-3.11400	-46.26900	-18.95000
H	-3.38000	-46.07400	-17.47200
O	-13.14200	-47.74200	-10.59300
H	-12.55600	-48.48800	-10.46000
H	-13.33900	-47.43200	-9.71000
O	-10.28800	-44.70700	-15.57700
H	-10.95700	-44.22200	-16.06100
H	-10.42600	-44.46000	-14.66300
O	-6.14700	-42.67200	-8.97900

H	-5.59500	-42.50900	-8.21400
H	-5.80700	-42.08200	-9.65100
O	-3.01000	-44.72400	-11.99200
H	-3.13500	-45.52500	-12.50100
H	-2.35200	-44.23000	-12.48100
O	-6.00100	-45.10700	-6.96400
H	-5.69200	-45.35200	-6.09200
H	-5.20300	-44.90200	-7.45200
O	-5.97900	-42.95300	-14.73300
H	-6.41800	-43.44500	-14.04000
H	-5.22800	-43.49600	-14.97400
O	-13.49900	-46.76200	-14.73100
H	-12.67300	-47.06100	-15.11100
H	-13.85000	-47.53100	-14.28200
O	-8.53100	-47.40900	-12.66100
H	-9.15300	-48.07200	-12.96100
H	-7.86100	-47.38300	-13.34400
O	-15.29800	-48.84300	-13.77200
H	-16.13500	-49.27100	-13.95400
H	-15.32600	-48.65000	-12.83500
O	-5.47000	-46.36400	-21.71400
H	-6.04300	-45.74500	-22.16700
H	-4.89700	-45.81400	-21.18000
O	-13.18700	-44.63000	-13.00900
H	-13.32200	-45.39000	-13.57600
H	-13.60400	-43.90600	-13.47700
O	-10.03000	-43.73300	-12.79800
H	-10.14700	-42.78900	-12.90000
H	-10.85100	-44.03600	-12.40900
O	-9.00500	-43.08100	-9.58200
H	-8.10200	-43.04800	-9.26500
H	-9.23100	-44.01100	-9.56700
O	-7.24900	-44.32800	-12.92100
H	-8.20000	-44.32700	-13.03500
H	-6.94600	-45.07400	-13.43700
O	8.48600	-38.49100	-11.85600
H	9.12400	-38.61100	-12.56000
H	8.36800	-37.54300	-11.79800
O	5.29500	-38.53900	-11.92000
H	4.95700	-38.65500	-12.80800
H	6.16400	-38.94000	-11.94000
O	1.15300	-36.68500	-15.93100
H	0.59000	-36.77600	-16.69900
H	1.03900	-37.50500	-15.45000
O	5.77700	-37.38500	-5.96200
H	5.76600	-37.78000	-6.83400
H	5.93900	-38.11900	-5.36800
O	-1.30000	-35.19000	-13.09200
H	-0.50800	-35.53100	-13.50800
H	-1.06000	-35.08100	-12.17200
O	6.73300	-33.01800	-20.61900
H	6.08200	-32.43300	-21.00600
H	7.52800	-32.48700	-20.56000
O	0.65900	-35.92900	-20.22000
H	1.43200	-36.47600	-20.35900
H	1.01200	-35.06500	-20.00900
O	2.49700	-34.60200	-7.01400
H	2.20600	-33.69700	-7.12900
H	3.13300	-34.56000	-6.29900
O	3.42300	-37.22400	-10.42400
H	4.01900	-37.44800	-11.13800
H	3.48100	-37.96500	-9.82100
O	2.99600	-39.77000	-22.58900
H	3.24800	-40.29200	-23.35000
H	2.05800	-39.93300	-22.48600
O	8.04000	-40.76600	-14.78100
H	8.82600	-41.18400	-15.13200
H	8.30400	-39.86200	-14.61300
O	8.21100	-35.32900	-20.08200
H	7.57600	-35.25600	-19.37000
H	7.88100	-34.74000	-20.76000
O	2.71500	-37.86700	-20.38600
H	2.59900	-38.54300	-19.71800
H	2.82500	-38.35400	-21.20300
O	5.05800	-38.40600	-16.68100
H	4.69400	-38.93300	-15.97000
H	5.91500	-38.79600	-16.85500
O	4.69900	-43.37700	-21.68500
H	4.86600	-42.50900	-21.31800
H	4.50900	-43.21400	-22.60900
O	7.45600	-34.91500	-10.01900
H	7.15700	-34.01300	-10.12700
H	7.11200	-35.18200	-9.16700
O	0.90800	-38.46400	-13.98500
H	-0.01700	-38.59600	-13.77700
H	1.15600	-37.68500	-13.48800
O	4.67800	-40.92100	-20.70100
H	4.55800	-40.47600	-19.86200
H	3.98300	-40.57300	-21.25900
O	-1.73100	-38.82100	-13.15100
H	-1.60200	-38.56700	-12.23700
H	-2.41200	-38.22800	-13.47000
O	2.43900	-28.15100	-17.05200
H	2.36100	-27.20000	-16.97300
H	1.83500	-28.49600	-16.39400
O	4.93000	-40.97800	-4.39600
H	4.15400	-40.41900	-4.40500
H	5.56900	-40.49300	-3.87400
O	10.37600	-40.44900	-19.87300
H	11.28800	-40.21600	-20.04800
H	9.86700	-39.86500	-20.43500
O	3.18500	-38.17000	-4.83400
H	3.92600	-37.63100	-5.11000
H	2.47900	-37.54300	-4.67700
O	8.66000	-36.02700	-6.23600
H	9.06900	-36.46800	-6.98100
H	7.93800	-36.60100	-5.98300
O	5.01400	-41.08900	-8.86300
H	5.86000	-40.66600	-8.72000
H	4.95300	-41.74600	-8.16900
O	8.98300	-35.46700	-12.48100
H	8.35400	-35.30200	-13.18400
H	8.59600	-35.05200	-11.71000
O	12.86200	-34.53100	-15.12700
H	12.17700	-34.15100	-15.67700
H	12.94700	-33.92200	-14.39400
O	10.11700	-36.88500	-8.52700
H	10.32900	-36.24200	-9.20400

H	10.07000	-37.71900	-8.99500
O	4.53600	-35.72900	-15.21600
H	5.00000	-35.45000	-16.00600
H	4.27200	-36.63100	-15.39900
O	1.42900	-36.18200	-12.27800
H	1.99900	-35.75300	-12.91700
H	2.01900	-36.46400	-11.58000
O	3.47300	-39.60700	-18.40700
H	4.17500	-39.15500	-17.94000
H	3.14100	-40.24800	-17.77900
O	1.92100	-41.01600	-4.03200
H	1.29100	-40.33400	-3.79900
H	1.81500	-41.12700	-4.97700
O	-2.78400	-41.34900	-13.10600
H	-3.57500	-41.09300	-13.58200
H	-2.29300	-40.53400	-13.00000
O	11.23200	-34.00800	-10.35500
H	12.05400	-34.41000	-10.63700
H	11.22800	-34.11100	-9.40300
O	-0.83300	-40.98400	-16.14700
H	-1.63000	-40.87200	-15.62900
H	-1.06000	-40.64500	-17.01300
O	4.99800	-34.46000	-8.19900
H	5.06200	-33.99800	-7.36300
H	4.28900	-35.09000	-8.07100
O	-4.50100	-41.09500	-10.67400
H	-4.25000	-40.21100	-10.40800
H	-3.96500	-41.27500	-11.44700
O	19.32300	-37.66400	-14.00700
H	18.56900	-38.23300	-14.16200
H	19.45700	-37.69800	-13.05900
O	4.71200	-32.79300	-18.33100
H	4.57700	-31.94800	-17.90300
H	5.25300	-32.59100	-19.09400
O	-1.83500	-35.27500	-17.75800
H	-1.32200	-34.48100	-17.60800
H	-1.18200	-35.97300	-17.82500
O	-0.02500	-32.15900	-15.67600
H	0.15100	-31.23700	-15.86400
H	0.77000	-32.47300	-15.24700
O	-0.74000	-40.11200	-5.80000
H	-0.85500	-41.01400	-5.49900
H	-1.59600	-39.86600	-6.15100
O	6.33900	-32.17000	-4.20100
H	5.89600	-32.95700	-4.51900
H	7.00100	-31.98200	-4.86700
O	1.09800	-30.64100	-9.67500
H	1.64900	-30.76900	-10.44700
H	0.25900	-30.34100	-10.02500
O	8.43700	-31.89000	-6.17300
H	8.41900	-31.06900	-6.66500
H	9.35900	-32.01500	-5.94800
O	4.89500	-34.37600	-5.33100
H	5.42700	-35.13700	-5.56100
H	4.36600	-34.66800	-4.58800
O	-3.87000	-36.94600	-16.98200
H	-3.03200	-36.53700	-17.19900
H	-3.69200	-37.88600	-17.01200
O	8.02700	-39.27900	-21.29400
H	7.51100	-38.50800	-21.05500
H	7.75400	-39.95200	-20.67100
O	13.00000	-36.52600	-19.35900
H	12.22300	-36.86500	-19.80300
H	13.67900	-36.52000	-20.03300
O	7.81500	-41.40600	-19.46800
H	7.51000	-41.09200	-18.61700
H	8.76800	-41.32800	-19.42500
O	6.26000	-35.24700	-17.81900
H	5.73200	-35.98200	-18.13100
H	5.75900	-34.47000	-18.06500
O	11.23900	-37.05200	-12.70200
H	10.58200	-36.36200	-12.61400
H	12.04300	-36.66300	-12.35900
O	0.99200	-44.58500	-15.45800
H	1.56600	-44.80800	-16.19100
H	1.57900	-44.51600	-14.70500
O	10.05000	-38.69800	-14.65200
H	10.56300	-38.06500	-14.14900
H	10.70000	-39.29800	-15.01700
O	1.31900	-42.55500	-21.43800
H	0.48200	-42.21700	-21.11800
H	1.17900	-42.69000	-22.37600
O	4.40500	-37.42900	-24.81500
H	3.49800	-37.66000	-24.61600
H	4.90200	-38.22500	-24.62800
O	7.31200	-28.71900	-21.89600
H	6.63800	-28.41600	-21.28700
H	7.69900	-27.91600	-22.24500
O	6.52800	-41.22300	-12.40600
H	5.61000	-41.00100	-12.56400
H	6.94400	-41.14800	-13.26500
O	7.31100	-39.82400	-17.27500
H	8.05900	-39.31200	-17.58500
H	7.58000	-40.14500	-16.41500
O	5.58400	-37.32800	-20.29300
H	4.67500	-37.61200	-20.19600
H	5.61400	-36.90800	-21.15200
O	10.81600	-33.71700	-16.52400
H	10.36200	-34.55800	-16.46700
H	10.18500	-33.08100	-16.18600
O	9.25900	-31.88000	-15.58900
H	9.55200	-31.31900	-16.30700
H	9.76900	-31.58700	-14.83300
O	-3.84700	-39.60700	-16.31100
H	-4.04500	-40.20000	-17.03600
H	-4.40300	-39.91200	-15.59400
O	4.13200	-39.71500	-14.19200
H	4.18700	-40.66800	-14.26800
H	3.32200	-39.55900	-13.70800
O	5.28200	-35.78700	-22.95400
H	4.84400	-36.50300	-23.41300
H	6.02300	-35.55900	-23.51500
O	2.07200	-41.09700	-16.43300
H	1.25900	-40.79800	-16.02600
H	2.56800	-41.49200	-15.71500
O	4.22300	-31.10100	-14.49900
H	4.18000	-30.87500	-15.42800
H	5.13900	-31.33500	-14.35100

O	7.66900	-40.63300	-7.60100
H	8.02400	-40.40600	-6.74200
H	8.31600	-40.30100	-8.22400
O	13.39900	-37.51100	-16.89600
H	13.26100	-37.32100	-17.82400
H	13.04300	-36.74900	-16.44000
O	1.56300	-43.52500	-10.81700
H	2.20300	-43.66700	-11.51400
H	0.73100	-43.81200	-11.19300
O	13.42600	-35.44400	-11.88500
H	13.86000	-35.53600	-11.03700
H	14.13900	-35.46800	-12.52300
O	2.33900	-34.22500	-14.70300
H	3.13300	-34.72000	-14.90500
H	1.62800	-34.75300	-15.06600
O	7.15900	-31.84300	-10.35200
H	7.21200	-31.27400	-9.58400
H	6.29000	-31.67300	-10.71700
O	-1.35300	-40.08100	-18.62700
H	-2.28300	-40.14200	-18.41000
H	-1.25700	-40.60300	-19.42400
O	-1.43400	-43.55500	-16.03900
H	-1.17300	-42.64100	-16.14800
H	-0.61200	-44.02300	-15.89000
O	0.03800	-42.14200	-8.86900
H	0.71100	-42.35000	-9.51800
H	-0.78900	-42.33900	-9.30900
O	14.28700	-35.40100	-9.35200
H	14.48100	-34.49700	-9.10600
H	14.18700	-35.86000	-8.51800
O	9.34900	-35.25100	-3.77800
H	9.19700	-35.47900	-4.69500
H	9.33900	-34.29300	-3.76500
O	6.11700	-37.67700	-8.79300
H	7.04700	-37.52600	-8.96600
H	5.69700	-37.58900	-9.64800
O	4.91600	-33.65200	-11.02300
H	4.77700	-34.02000	-10.15100
H	4.12800	-33.13800	-11.19500
O	5.51100	-28.55100	-19.68100
H	5.68600	-29.02300	-18.86600
H	4.82400	-27.92500	-19.45300
O	6.46700	-34.27400	-13.63400
H	6.13200	-34.31400	-12.73900
H	5.96700	-34.94000	-14.10700
O	0.44100	-38.86700	-8.10900
H	0.30300	-39.59900	-7.50800
H	1.35200	-38.95600	-8.39100
O	9.40300	-36.92900	-23.36100
H	8.78600	-37.65400	-23.45700
H	8.93400	-36.16800	-23.70400
O	3.06900	-39.15500	-8.62100
H	3.77900	-39.79600	-8.66200
H	3.02600	-38.90400	-7.69800
O	11.05800	-32.21900	-5.70000
H	10.99900	-32.90300	-6.36700
H	11.46000	-31.47700	-6.15200
O	1.78200	-42.16900	-6.80100
H	1.24600	-42.62000	-6.14900
H	1.19400	-42.03400	-7.54400
O	0.07700	-25.40200	-12.90700
H	0.31700	-24.77100	-12.22900
H	-0.76600	-25.75400	-12.62000
O	1.19300	-42.71000	-24.23900
H	0.31600	-42.83100	-24.60200
H	1.66800	-43.50100	-24.49100
O	11.06000	-34.25500	-7.46900
H	10.17300	-34.59200	-7.34600
H	11.63000	-34.98600	-7.23200
O	-7.28700	-37.92200	-14.14400
H	-6.60000	-38.23600	-14.73200
H	-7.44200	-37.01800	-14.41800
O	-2.48400	-43.03300	-9.71600
H	-2.56800	-43.64100	-10.45100
H	-3.30500	-42.54200	-9.71500
O	4.59000	-29.59500	-10.50300
H	4.51700	-28.78900	-9.99100
H	4.14800	-29.39500	-11.32800
O	4.37900	-30.35700	-16.99200
H	3.59600	-29.81500	-16.89900
H	5.07800	-29.84000	-16.59300
O	15.54200	-38.84400	-12.70700
H	15.53100	-37.96100	-13.07800
H	14.67300	-39.19700	-12.90200
O	7.05000	-29.69600	-8.34700
H	7.76300	-29.06100	-8.41900
H	6.55900	-29.59900	-9.16300
O	8.77200	-35.79100	-16.77500
H	7.83700	-35.67100	-16.94500
H	8.94900	-36.69300	-17.03900
O	4.27200	-42.40600	-14.62200
H	3.90200	-42.95900	-13.93400
H	4.76800	-43.01000	-15.17400
O	12.15900	-39.94900	-16.24000
H	12.62500	-39.11600	-16.31400
H	12.61000	-40.41400	-15.53600
O	2.10100	-31.94600	-7.52600
H	2.80200	-31.31300	-7.37300
H	1.63100	-31.60500	-8.28700
O	10.22700	-33.60500	-19.24200
H	10.50200	-33.71900	-18.33200
H	9.35700	-34.00300	-19.28000
O	9.90100	-39.39200	-9.70100
H	9.24800	-39.14400	-10.35600
H	10.27900	-40.20600	-10.03500
O	6.86200	-29.35600	-15.59800
H	7.04400	-30.17400	-15.13500
H	7.68500	-29.13900	-16.03600
O	7.19600	-30.42900	-18.33700
H	7.72500	-30.94000	-18.95000
H	7.45900	-30.74500	-17.47200
O	5.95000	-38.65000	-3.21400
H	5.27400	-38.75100	-2.54400
H	6.77300	-38.80600	-2.75100
O	-2.30200	-32.41300	-10.59300
H	-1.71700	-33.15800	-10.46000
H	-2.50000	-32.10200	-9.71000
O	0.55200	-29.37800	-15.57700

H	-0.11700	-28.89300	-16.06100
H	0.41300	-29.13100	-14.66300
O	-7.71000	-39.84200	-11.87300
H	-7.67200	-40.37600	-11.07900
H	-8.21600	-40.36900	-12.49200
O	4.69200	-27.34300	-8.97900
H	5.24400	-27.18000	-8.21400
H	5.03300	-26.75300	-9.65100
O	-0.33100	-44.99600	-8.37600
H	-0.26700	-44.13400	-8.78900
H	-0.39500	-45.60800	-9.10900
O	-0.85600	-42.92300	-4.72900
H	-0.22900	-43.30600	-4.11500
H	-1.71300	-43.14200	-4.36400
O	9.54300	-32.51700	-3.12400
H	10.08700	-31.90400	-3.61800
H	8.96200	-31.95900	-2.60700
O	-4.08000	-41.11600	-18.79700
H	-3.78400	-42.02500	-18.85100
H	-4.98100	-41.13700	-19.11900
O	7.82900	-29.39500	-11.99200
H	7.70400	-30.19600	-12.50100
H	8.48700	-28.90000	-12.48100
O	-0.55000	-43.04000	-12.81500
H	-1.27100	-42.42700	-12.95400
H	0.23900	-42.50400	-12.89300
O	9.89300	-31.59400	-10.36400
H	8.94600	-31.71400	-10.43700
H	10.24100	-32.48200	-10.28200
O	-2.35100	-43.43400	-18.67300
H	-2.67900	-44.31500	-18.85600
H	-1.99700	-43.49000	-17.78600
O	6.89700	-31.79900	-14.16400
H	6.75000	-32.72700	-13.98300
H	7.72300	-31.77700	-14.64800
O	0.76300	-43.14700	-18.30600
H	1.10300	-42.61900	-19.02800
H	1.21100	-42.80900	-17.53000
O	10.50100	-30.97600	-13.07700
H	11.42500	-31.18200	-13.21500
H	10.33200	-31.22400	-12.16900
O	2.75000	-44.65000	-20.10700
H	3.47100	-44.27200	-20.61000
H	2.12700	-43.93100	-20.00300
O	1.63300	-40.98700	-12.59400
H	1.48800	-40.67300	-11.70100
H	1.15600	-40.36800	-13.14700
O	10.29000	-30.45700	-17.72100
H	10.46400	-31.02300	-18.47400
H	10.78300	-29.65700	-17.90300
O	-3.89200	-38.44600	-9.65500
H	-4.43700	-37.67000	-9.52300
H	-3.01400	-38.09900	-9.81300
O	16.14400	-37.69400	-16.10200
H	16.58700	-38.44300	-16.50000
H	15.26700	-37.70300	-16.48500
O	11.27800	-31.14100	-20.62200
H	11.97300	-30.52000	-20.40300
H	11.62000	-31.99100	-20.34600
O	-3.10600	-39.31900	-6.76200
H	-3.84700	-39.17100	-6.17500
H	-3.42600	-39.05800	-7.62500
O	13.17700	-33.69100	-19.09300
H	13.70200	-33.35200	-19.81800
H	13.08900	-34.62600	-19.27600
O	4.83900	-29.77700	-6.96400
H	5.14800	-30.02300	-6.09200
H	5.63600	-29.57300	-7.45200
O	4.86100	-27.62400	-14.73300
H	4.42100	-28.11600	-14.04000
H	5.61200	-28.16600	-14.97400
O	-2.66000	-31.43200	-14.73100
H	-1.83400	-31.73200	-15.11100
H	-3.01000	-32.20200	-14.28200
O	2.30800	-32.08000	-12.66100
H	1.68600	-32.74300	-12.96100
H	2.97900	-32.05400	-13.34400
O	-4.45900	-33.51400	-13.77200
H	-5.29600	-33.94200	-13.95400
H	-4.48700	-33.32100	-12.83500
O	2.38100	-43.60200	-3.06800
H	2.43000	-42.72500	-3.45000
H	2.92300	-44.14400	-3.64100
O	12.86600	-33.05800	-3.78800
H	12.07600	-33.07500	-4.32700
H	12.63000	-32.51900	-3.03300
O	12.98600	-36.20800	-6.47400
H	13.03600	-36.37300	-5.53300
H	12.87700	-37.07600	-6.86500
O	1.87000	-33.52900	-19.18500
H	2.01800	-32.84600	-19.83900
H	2.67600	-33.54900	-18.67000
O	10.27600	-40.22500	-5.15800
H	11.17700	-40.36500	-5.45000
H	10.35100	-40.06000	-4.21900
O	4.61100	-42.84900	-6.31300
H	3.74100	-42.55500	-6.58500
H	4.84700	-42.26400	-5.59200
O	14.48800	-33.78900	-6.23000
H	14.19600	-33.42600	-5.39400
H	13.87500	-34.50200	-6.40900
O	15.68700	-36.22300	-13.71000
H	16.52200	-35.78400	-13.54600
H	15.82300	-36.69900	-14.52900
O	12.73700	-38.66500	-7.76200
H	13.21200	-38.47300	-8.57000
H	11.81300	-38.60900	-8.00800
O	-0.37500	-37.50300	-18.17900
H	-0.62100	-38.40700	-17.98100
H	-0.20600	-37.50100	-19.12100
O	-1.10600	-41.77000	-20.68000
H	-1.79900	-41.69600	-21.33600
H	-1.35000	-42.53400	-20.15800
O	5.36900	-31.03500	-21.71400
H	4.79700	-30.41600	-22.16700
H	5.94200	-30.48500	-21.18000
O	8.58900	-30.75100	-20.58300
H	9.52200	-30.81600	-20.38000

H	8.50100	-29.91500	-21.04200
O	11.21800	-31.43400	-23.62300
H	11.13900	-31.76800	-22.72900
H	12.03900	-30.94200	-23.62400
O	13.91500	-38.56800	-10.41500
H	13.88300	-39.50700	-10.22900
H	14.52600	-38.49000	-11.14900
O	5.75400	-39.69900	-24.37200
H	6.70000	-39.82800	-24.30400
H	5.37500	-40.43700	-23.89500
O	19.73300	-37.50700	-11.27500
H	20.46300	-37.76800	-10.71400
H	19.12400	-38.24400	-11.23400
O	5.63800	-35.64700	-2.91200
H	6.55800	-35.46900	-2.71800
H	5.60300	-36.59100	-3.06400
O	3.93800	-41.77400	-24.43700
H	3.03100	-42.07000	-24.36100
H	4.21600	-42.07100	-25.30400
O	-5.14400	-40.45300	-13.98700
H	-5.46400	-41.30600	-14.28000
H	-5.69100	-40.23800	-13.23100
O	-1.36600	-37.73700	-10.27000
H	-1.14700	-36.80500	-10.25100
H	-0.95400	-38.09600	-9.48500
O	3.50700	-42.16300	-0.88000
H	3.40600	-41.21400	-0.94900
H	3.28900	-42.49200	-1.75200
O	-5.27500	-36.83300	-12.01800
H	-5.96900	-37.24700	-12.53100
H	-4.52800	-36.79100	-12.61400
O	3.37000	-38.73400	-2.06300
H	2.88700	-37.93400	-1.85700
H	3.45600	-38.72300	-3.01700
O	-3.36200	-36.90100	-14.14100
H	-3.38500	-36.80200	-15.09300
H	-3.05200	-36.05400	-13.81900
O	-0.70300	-34.84300	-10.02600
H	0.25100	-34.87500	-10.09300
H	-0.90200	-35.30400	-9.21100
O	-0.11300	-36.27300	-7.45900
H	0.70600	-35.77900	-7.43600
H	0.16100	-37.19000	-7.48800
O	-2.34800	-29.30100	-13.00900
H	-2.48200	-30.06100	-13.57600
H	-2.76500	-28.57700	-13.47700
O	8.78800	-34.11800	-24.00200
H	9.49200	-33.91300	-23.38600
H	8.06400	-33.55000	-23.73700
O	9.61800	-38.36700	-17.88200
H	9.56100	-37.91000	-18.72100
H	10.17100	-39.12700	-18.05900
O	0.80900	-28.40400	-12.79800
H	0.69200	-27.46000	-12.90000
H	-0.01200	-28.70700	-12.40900
O	10.69700	-33.67900	-21.95200
H	11.53100	-34.11100	-22.13400
H	10.53500	-33.85100	-21.02400
O	10.35100	-37.17600	-20.58600
H	10.29600	-37.34200	-21.52700
H	9.72600	-36.46800	-20.43000
O	-4.85900	-41.93900	-6.76000
H	-4.21400	-42.44900	-6.27100
H	-5.06800	-41.20200	-6.18600
O	7.66600	-34.87100	-1.26100
H	8.53500	-34.69700	-1.62200
H	7.18700	-34.05200	-1.38300
O	1.83500	-27.75100	-9.58200
H	2.73700	-27.71900	-9.26500
H	1.60800	-28.68100	-9.56700
O	3.59000	-28.99800	-12.92100
H	2.63900	-28.99800	-13.03500
H	3.89300	-29.74500	-13.43700
O	-0.52000	-44.79200	-2.66300
H	0.34300	-45.14900	-2.45200
H	-1.09300	-45.13100	-1.97500
O	19.32500	-23.16200	-11.85600
H	19.96300	-23.28200	-12.56000
H	19.20700	-22.21400	-11.79800
O	16.13400	-23.21000	-11.92000
H	15.79600	-23.32500	-12.80800
H	17.00300	-23.61000	-11.94000
O	11.99300	-21.35600	-15.93100
H	11.42900	-21.44700	-16.69900
H	11.87800	-22.17600	-15.45000
O	16.61600	-22.05600	-5.96200
H	16.60500	-22.45100	-6.83400
H	16.77800	-22.78900	-5.36800
O	9.53900	-19.86100	-13.09200
H	10.33100	-20.20200	-13.50800
H	9.77900	-19.75100	-12.17200
O	17.57300	-17.68900	-20.61900
H	16.92200	-17.10400	-21.00600
H	18.36700	-17.15800	-20.56000
O	11.49800	-20.60000	-20.22000
H	12.27100	-21.14700	-20.35900
H	11.85100	-19.73600	-20.00900
O	13.33700	-19.27200	-7.01400
H	13.04600	-18.36800	-7.12900
H	13.97300	-19.23100	-6.29900
O	14.26200	-21.89500	-10.42400
H	14.85900	-22.11900	-11.13800
H	14.32000	-22.63600	-9.82100
O	13.83500	-24.44100	-22.58900
H	14.08800	-24.96300	-23.35000
H	12.89700	-24.60400	-22.48600
O	18.87900	-25.43700	-14.78100
H	19.66600	-25.85500	-15.13200
H	19.14300	-24.53300	-14.61300
O	19.05000	-20.00000	-20.08200
H	18.41500	-19.92600	-19.37000
H	18.72000	-19.41100	-20.76000
O	24.28000	-25.50000	-13.87800
H	25.01300	-26.07000	-13.64400
H	23.52400	-25.88700	-13.43700
O	13.55500	-22.53800	-20.38600
H	13.43800	-23.21300	-19.71800
H	13.66500	-23.02500	-21.20300

O	15.89800	-23.07700	-16.68100
H	15.53400	-23.60400	-15.97000
H	16.75400	-23.46700	-16.85500
O	21.80300	-31.78700	-9.41800
H	21.47100	-30.91800	-9.64200
H	21.20700	-32.10400	-8.73900
O	15.53800	-28.04800	-21.68500
H	15.70600	-27.18000	-21.31800
H	15.34800	-27.88500	-22.60900
O	18.29600	-19.58600	-10.01900
H	17.99600	-18.68400	-10.12700
H	17.95100	-19.85300	-9.16700
O	11.74700	-23.13500	-13.98500
H	10.82200	-23.26700	-13.77700
H	11.99600	-22.35500	-13.48800
O	21.69600	-27.06800	-12.33900
H	20.78700	-27.13600	-12.63300
H	21.64400	-26.59100	-11.51100
O	15.51700	-25.59200	-20.70100
H	15.39700	-25.14600	-19.86200
H	14.82200	-25.24400	-21.25900
O	9.10800	-23.49200	-13.15100
H	9.23700	-23.23800	-12.23700
H	8.42800	-22.89900	-13.47000
O	13.27800	-12.82200	-17.05200
H	13.20000	-11.87100	-16.97300
H	12.67400	-13.16700	-16.39400
O	15.77000	-25.64900	-4.39600
H	14.99300	-25.09000	-4.40500
H	16.40900	-25.16300	-3.87400
O	23.56200	-28.86600	-10.56100
H	22.91900	-28.57300	-11.20700
H	23.05700	-28.98100	-9.75600
O	21.21600	-25.12000	-19.87300
H	22.12800	-24.88700	-20.04800
H	20.70700	-24.53600	-20.43500
O	14.02400	-22.84000	-4.83400
H	14.76600	-22.30200	-5.11000
H	13.31800	-22.21400	-4.67700
O	19.50000	-20.69800	-6.23600
H	19.90800	-21.13900	-6.98100
H	18.77800	-21.27200	-5.98300
O	15.85300	-25.76000	-8.86300
H	16.69900	-25.33600	-8.72000
H	15.79300	-26.41700	-8.16900
O	19.82300	-20.13700	-12.48100
H	19.19400	-19.97300	-13.18400
H	19.43500	-19.72300	-11.71000
O	23.70200	-19.20200	-15.12700
H	23.01700	-18.82200	-15.67700
H	23.78600	-18.59200	-14.39400
O	19.00300	-28.10000	-7.20500
H	18.75900	-27.34600	-7.74200
H	19.27900	-27.72100	-6.37000
O	20.95700	-21.55600	-8.52700
H	21.16800	-20.91300	-9.20400
H	20.91000	-22.39000	-8.99500
O	18.55900	-28.94400	-11.62400
H	17.69800	-28.64600	-11.33200
H	18.58600	-28.72700	-12.55600
O	15.37500	-20.40000	-15.21600
H	15.83900	-20.12100	-16.00600
H	15.11100	-21.30200	-15.39900
O	12.26800	-20.85300	-12.27800
H	12.83800	-20.42400	-12.91700
H	12.85900	-21.13500	-11.58000
O	14.31200	-24.27800	-18.40700
H	15.01500	-23.82600	-17.94000
H	13.98000	-24.91900	-17.77900
O	12.76000	-25.68600	-4.03200
H	12.13000	-25.00500	-3.79900
H	12.65400	-25.79800	-4.97700
O	8.05500	-26.01900	-13.10600
H	7.26500	-25.76400	-13.58200
H	8.54600	-25.20400	-13.00000
O	22.07100	-18.67900	-10.35500
H	22.89300	-19.08100	-10.63700
H	22.06800	-18.78200	-9.40300
O	10.00600	-25.65500	-16.14700
H	9.20900	-25.54300	-15.62900
H	9.77900	-25.31600	-17.01300
O	15.83700	-19.13000	-8.19900
H	15.90100	-18.66800	-7.36300
H	15.12800	-19.76100	-8.07100
O	6.33900	-25.76600	-10.67400
H	6.58900	-24.88200	-10.40800
H	6.87500	-25.94600	-11.44700
O	30.16200	-22.33500	-14.00700
H	29.40800	-22.90400	-14.16200
H	30.29700	-22.36900	-13.05900
O	15.55200	-17.46400	-18.33100
H	15.41600	-16.61800	-17.90300
H	16.09300	-17.26200	-19.09400
O	12.03800	-31.38400	-1.67000
H	11.64500	-31.93800	-0.99500
H	12.85500	-31.07300	-1.27900
O	9.00500	-19.94600	-17.75800
H	9.51700	-19.15200	-17.60800
H	9.65700	-20.64300	-17.82500
O	10.81400	-16.82900	-15.67600
H	10.99100	-15.90800	-15.86400
H	11.61000	-17.14400	-15.24700
O	14.95500	-29.96900	-4.07800
H	15.69600	-29.36800	-4.15700
H	15.18700	-30.71100	-4.63600
O	10.09900	-24.78300	-5.80000
H	9.98500	-25.68400	-5.49900
H	9.24400	-24.53700	-6.15100
O	17.17900	-16.84100	-4.20100
H	16.73500	-17.62800	-4.51900
H	17.84000	-16.65300	-4.86700
O	11.93700	-15.31200	-9.67500
H	12.48800	-15.44000	-10.44700
H	11.09800	-15.01200	-10.02500
O	19.27700	-16.56100	-6.17300
H	19.25900	-15.74000	-6.66500
H	20.19900	-16.68600	-5.94800
O	15.73400	-19.04700	-5.33100

H	16.26700	-19.80800	-5.56100
H	15.20500	-19.33900	-4.58800
O	18.90100	-33.94100	-9.80100
H	18.55700	-33.05000	-9.87700
H	19.26900	-34.13000	-10.66400
O	6.97000	-21.61700	-16.98200
H	7.80700	-21.20700	-17.19900
H	7.14700	-22.55700	-17.01200
O	18.86600	-23.95000	-21.29400
H	18.35100	-23.17900	-21.05500
H	18.59400	-24.62300	-20.67100
O	16.45100	-29.10500	-16.23500
H	15.59500	-29.49400	-16.41500
H	16.86300	-29.02500	-17.09500
O	17.98100	-31.57000	-11.83600
H	17.47700	-31.60700	-12.64900
H	18.27400	-30.66100	-11.77700
O	14.22500	-31.48600	-12.83000
H	15.09000	-31.68200	-13.18900
H	14.29300	-31.71500	-11.90300
O	23.83900	-21.19600	-19.35900
H	23.06200	-21.53500	-19.80300
H	24.51900	-21.19100	-20.03300
O	18.65500	-26.07700	-19.46800
H	18.35000	-25.76200	-18.61700
H	19.60800	-25.99900	-19.42500
O	17.09900	-19.91800	-17.81900
H	16.57100	-20.65300	-18.13100
H	16.59800	-19.14000	-18.06500
O	22.07900	-21.72300	-12.70200
H	21.42100	-21.03300	-12.61400
H	22.88300	-21.33300	-12.35900
O	11.83100	-29.25600	-15.45800
H	12.40600	-29.47800	-16.19100
H	12.41800	-29.18700	-14.70500
O	20.88900	-23.36900	-14.65200
H	21.40200	-22.73600	-14.14900
H	21.53900	-23.96800	-15.01700
O	12.15800	-27.22600	-21.43800
H	11.32200	-26.88800	-21.11800
H	12.01900	-27.36100	-22.37600
O	14.13300	-32.38200	-16.73100
H	13.48000	-32.69700	-17.35500
H	14.09100	-33.00400	-16.00500
O	15.24500	-22.10000	-24.81500
H	14.33700	-22.33100	-24.61600
H	15.74200	-22.89600	-24.62800
O	18.15100	-13.39000	-21.89600
H	17.47800	-13.08700	-21.28700
H	18.53800	-12.58700	-22.24500
O	17.36700	-25.89400	-12.40600
H	16.44900	-25.67200	-12.56400
H	17.78300	-25.81900	-13.26500
O	18.15100	-24.49500	-17.27500
H	18.89800	-23.98200	-17.58500
H	18.42000	-24.81600	-16.41500
O	16.42300	-21.99900	-20.29300
H	15.51500	-22.28300	-20.19600
H	16.45300	-21.57900	-21.15200
O	21.65500	-18.38800	-16.52400
H	21.20200	-19.22900	-16.46700
H	21.02500	-17.75200	-16.18600
O	20.59900	-27.45900	-15.29400
H	19.86100	-27.97100	-14.96300
H	20.85100	-27.89900	-16.10500
O	20.09900	-16.55000	-15.58900
H	20.39100	-15.99000	-16.30700
H	20.60800	-16.25700	-14.83300
O	20.70000	-34.68100	-14.69900
H	21.00600	-35.54100	-14.98700
H	21.42500	-34.08900	-14.90000
O	6.99200	-24.27800	-16.31100
H	6.79500	-24.87100	-17.03600
H	6.43700	-24.58300	-15.59400
O	14.97200	-24.38600	-14.19200
H	15.02700	-25.33800	-14.26800
H	14.16100	-24.23000	-13.70800
O	16.12100	-20.45800	-22.95400
H	15.68300	-21.17400	-23.41300
H	16.86200	-20.23000	-23.51500
O	12.91100	-25.76800	-16.43300
H	12.09800	-25.46900	-16.02600
H	13.40700	-26.16300	-15.71500
O	15.06200	-15.77200	-14.49900
H	15.02000	-15.54600	-15.42800
H	15.97800	-16.00600	-14.35100
O	18.50900	-25.30400	-7.60100
H	18.86300	-25.07700	-6.74200
H	19.15600	-24.97200	-8.22400
O	24.23800	-22.18200	-16.89600
H	24.10000	-21.99200	-17.82400
H	23.88200	-21.42000	-16.44000
O	12.40300	-28.19600	-10.81700
H	13.04200	-28.33700	-11.51400
H	11.57000	-28.48300	-11.19300
O	24.26500	-20.11400	-11.88500
H	24.69900	-20.20700	-11.03700
H	24.97800	-20.13900	-12.52300
O	13.17900	-18.89600	-14.70300
H	13.97300	-19.39100	-14.90500
H	12.46800	-19.42400	-15.06600
O	17.99800	-16.51400	-10.35200
H	18.05200	-15.94500	-9.58400
H	17.13000	-16.34400	-10.71700
O	9.48700	-24.75200	-18.62700
H	8.55700	-24.81300	-18.41000
H	9.58300	-25.27400	-19.42400
O	9.40500	-28.22600	-16.03900
H	9.66600	-27.31200	-16.14800
H	10.22700	-28.69400	-15.89000
O	10.87800	-26.81200	-8.86900
H	11.55000	-27.02100	-9.51800
H	10.05100	-27.01000	-9.30900
O	25.12700	-20.07200	-9.35200
H	25.32000	-19.16700	-9.10600
H	25.02700	-20.53100	-8.51800
O	16.78000	-30.18600	-6.85300
H	16.13300	-29.48700	-6.94600

H	17.55100	-29.75300	-6.48500
O	20.18800	-19.92100	-3.77800
H	20.03700	-20.15000	-4.69500
H	20.17900	-18.96400	-3.76500
O	16.95700	-22.34800	-8.79300
H	17.88600	-22.19700	-8.96600
H	16.53600	-22.26000	-9.64800
O	15.75600	-18.32300	-11.02300
H	15.61700	-18.69100	-10.15100
H	14.96700	-17.80800	-11.19500
O	16.35000	-13.22200	-19.68100
H	16.52600	-13.69300	-18.86600
H	15.66400	-12.59600	-19.45300
O	17.30600	-18.94500	-13.63400
H	16.97100	-18.98500	-12.73900
H	16.80700	-19.61000	-14.10700
O	11.28100	-23.53800	-8.10900
H	11.14300	-24.27000	-7.50800
H	12.19100	-23.62700	-8.39100
O	20.24200	-21.59900	-23.36100
H	19.62500	-22.32500	-23.45700
H	19.77400	-20.83800	-23.70400
O	13.90900	-23.82600	-8.62100
H	14.61900	-24.46700	-8.66200
H	13.86600	-23.57500	-7.69800
O	21.89800	-16.88900	-5.70000
H	21.83900	-17.57400	-6.36700
H	22.29900	-16.14800	-6.15200
O	12.62200	-26.84000	-6.80100
H	12.08500	-27.29100	-6.14900
H	12.03300	-26.70500	-7.54400
O	10.91700	-10.07300	-12.90700
H	11.15600	-9.44100	-12.22900
H	10.07400	-10.42400	-12.62000
O	12.03200	-27.38100	-24.23900
H	11.15500	-27.50200	-24.60200
H	12.50800	-28.17200	-24.49100
O	21.89900	-18.92500	-7.46900
H	21.01200	-19.26300	-7.34600
H	22.46900	-19.65700	-7.23200
O	3.55200	-22.59300	-14.14400
H	4.23900	-22.90700	-14.73200
H	3.39700	-21.68900	-14.41800
O	8.35600	-27.70400	-9.71600
H	8.27100	-28.31100	-10.45100
H	7.53400	-27.21300	-9.71500
O	15.42900	-14.26500	-10.50300
H	15.35600	-13.46000	-9.99100
H	14.98700	-14.06600	-11.32800
O	15.21800	-15.02800	-16.99200
H	14.43500	-14.48600	-16.89900
H	15.91800	-14.51100	-16.59300
O	18.52800	-29.21900	-14.34900
H	19.02500	-29.97200	-14.66900
H	17.75700	-29.18200	-14.91500
O	26.38100	-23.51500	-12.70700
H	26.37000	-22.63200	-13.07800
H	25.51300	-23.86800	-12.90200
O	17.88900	-14.36700	-8.34700
H	18.60200	-13.73200	-8.41900
H	17.39800	-14.27000	-9.16300
O	19.61100	-20.46200	-16.77500
H	18.67700	-20.34100	-16.94500
H	19.78900	-21.36400	-17.03900
O	15.11200	-27.07700	-14.62200
H	14.74100	-27.63000	-13.93400
H	15.60700	-27.68100	-15.17400
O	22.99800	-24.62000	-16.24000
H	23.46400	-23.78700	-16.31400
H	23.44900	-25.08500	-15.53600
O	12.94000	-16.61700	-7.52600
H	13.64100	-15.98400	-7.37300
H	12.47000	-16.27600	-8.28700
O	21.06600	-18.27500	-19.24200
H	21.34200	-18.39000	-18.33200
H	20.19600	-18.67400	-19.28000
O	20.97000	-32.25200	-12.20100
H	20.05600	-32.02100	-12.03300
H	21.39900	-32.16800	-11.35000
O	20.74100	-24.06300	-9.70100
H	20.08800	-23.81500	-10.35600
H	21.11900	-24.87600	-10.03500
O	17.70100	-14.02700	-15.59800
H	17.88400	-14.84500	-15.13500
H	18.52500	-13.81000	-16.03600
O	18.03600	-15.10000	-18.33700
H	18.56400	-15.61100	-18.95000
H	18.29800	-15.41600	-17.47200
O	16.78900	-23.32100	-3.21400
H	16.11300	-23.42100	-2.54400
H	17.61200	-23.47700	-2.75100
O	8.53700	-17.08400	-10.59300
H	9.12300	-17.82900	-10.46000
H	8.34000	-16.77300	-9.71000
O	11.39100	-14.04800	-15.57700
H	10.72200	-13.56400	-16.06100
H	11.25200	-13.80200	-14.66300
O	3.12900	-24.51300	-11.87300
H	3.16700	-25.04700	-11.07900
H	2.62300	-25.03900	-12.49200
O	15.53200	-12.01400	-8.97900
H	16.08300	-11.85100	-8.21400
H	15.87200	-11.42400	-9.65100
O	10.50800	-29.66700	-8.37600
H	10.57200	-28.80500	-8.78900
H	10.44500	-30.27900	-9.10900
O	9.98300	-27.59400	-4.72900
H	10.61000	-27.97700	-4.11500
H	9.12600	-27.81300	-4.36400
O	20.38300	-17.18800	-3.12400
H	20.92700	-16.57500	-3.61800
H	19.80100	-16.63000	-2.60700
O	6.76000	-25.78700	-18.79700
H	7.05500	-26.69600	-18.85100
H	5.85900	-25.80800	-19.11900
O	18.66800	-14.06600	-11.99200
H	18.54400	-14.86700	-12.50100
H	19.32600	-13.57100	-12.48100

O	10.28900	-27.71100	-12.81500
H	9.56800	-27.09700	-12.95400
H	11.07900	-27.17500	-12.89300
O	20.73200	-16.26500	-10.36400
H	19.78500	-16.38400	-10.43700
H	21.08000	-17.15300	-10.28200
O	13.09200	-30.37600	-6.54100
H	13.61500	-30.12900	-5.77800
H	12.87300	-29.54400	-6.96000
O	8.48900	-28.10500	-18.67300
H	8.16000	-28.98500	-18.85600
H	8.84300	-28.16000	-17.78600
O	17.73700	-16.47000	-14.16400
H	17.58900	-17.39800	-13.98300
H	18.56200	-16.44800	-14.64800
O	11.60300	-27.81800	-18.30600
H	11.94200	-27.28900	-19.02800
H	12.05000	-27.48000	-17.53000
O	21.34000	-15.64700	-13.07700
H	22.26500	-15.85300	-13.21500
H	21.17200	-15.89500	-12.16900
O	14.29700	-29.43400	-8.85600
H	13.95700	-30.23300	-8.45300
H	13.59100	-29.13400	-9.42800
O	14.94900	-32.34900	-10.40300
H	15.77100	-32.82300	-10.52500
H	15.19300	-31.57000	-9.90300
O	13.59000	-29.32000	-20.10700
H	14.31000	-28.94200	-20.61000
H	12.96600	-28.60200	-20.00300
O	17.73300	-28.61600	-19.17100
H	17.94900	-27.73300	-19.47200
H	17.25200	-29.00700	-19.90000
O	12.47300	-25.65700	-12.59400
H	12.32700	-25.34300	-11.70100
H	11.99500	-25.03900	-13.14700
O	14.33200	-28.66700	-12.54600
H	14.31800	-29.56300	-12.88200
H	15.05000	-28.66000	-11.91200
O	16.18500	-28.23300	-10.50000
H	15.92700	-27.31200	-10.50200
H	15.65300	-28.63000	-9.81000
O	21.13000	-15.12800	-17.72100
H	21.30300	-15.69400	-18.47400
H	21.62200	-14.32800	-17.90300
O	6.94700	-23.11700	-9.65500
H	6.40300	-22.34100	-9.52300
H	7.82500	-22.77000	-9.81300
O	13.73600	-29.78600	-17.27700
H	13.85800	-30.71400	-17.07800
H	13.44600	-29.77200	-18.18900
O	16.60500	-32.10000	-14.14000
H	16.62700	-32.03600	-15.09500
H	17.02200	-32.94000	-13.94600
O	26.98300	-22.36500	-16.10200
H	27.42700	-23.11400	-16.50000
H	26.10600	-22.37400	-16.48500
O	22.11700	-15.81200	-20.62200
H	22.81200	-15.19100	-20.40300
H	22.45900	-16.66200	-20.34600
O	7.73400	-23.99000	-6.76200
H	6.99200	-23.84200	-6.17500
H	7.41300	-23.72900	-7.62500
O	24.01600	-18.36200	-19.09300
H	24.54100	-18.02300	-19.81800
H	23.92800	-19.29700	-19.27600
O	15.67800	-14.44800	-6.96400
H	15.98700	-14.69300	-6.09200
H	16.47600	-14.24400	-7.45200
O	15.70000	-12.29400	-14.73300
H	15.26100	-12.78700	-14.04000
H	16.45100	-12.83700	-14.97400
O	8.17900	-16.10300	-14.73100
H	9.00500	-16.40300	-15.11100
H	7.82900	-16.87300	-14.28200
O	13.14800	-16.75100	-12.66100
H	12.52600	-17.41400	-12.96100
H	13.81800	-16.72500	-13.34400
O	6.38000	-18.18500	-13.77200
H	5.54400	-18.61200	-13.95400
H	6.35300	-17.99100	-12.83500
O	13.22000	-28.27200	-3.06800
H	13.26900	-27.39600	-3.45000
H	13.76200	-28.81500	-3.64100
O	23.70600	-17.72800	-3.78800
H	22.91500	-17.74600	-4.32700
H	23.46900	-17.19000	-3.03300
O	23.82500	-20.87900	-6.47400
H	23.87500	-21.04400	-5.53300
H	23.71700	-21.74600	-6.86500
O	12.70900	-18.20000	-19.18500
H	12.85800	-17.51700	-19.83900
H	13.51600	-18.22000	-18.67000
O	21.11600	-24.89600	-5.15800
H	22.01700	-25.03500	-5.45000
H	21.19100	-24.73100	-4.21900
O	15.45000	-27.51900	-6.31300
H	14.58000	-27.22600	-6.58500
H	15.68600	-26.93500	-5.59200
O	25.32700	-18.45900	-6.23000
H	25.03600	-18.09600	-5.39400
H	24.71400	-19.17300	-6.40900
O	26.52700	-20.89400	-13.71000
H	27.36100	-20.45500	-13.54600
H	26.66300	-21.37000	-14.52900
O	23.57600	-23.33600	-7.76200
H	24.05100	-23.14300	-8.57000
H	22.65200	-23.28000	-8.00800
O	10.46400	-22.17400	-18.17900
H	10.21900	-23.07700	-17.98100
H	10.63400	-22.17200	-19.12100
O	18.15400	-31.30100	-8.79700
H	18.67200	-30.56500	-9.12200
H	17.79900	-30.99500	-7.96200
O	9.73400	-26.44100	-20.68000
H	9.04100	-26.36600	-21.33600
H	9.48900	-27.20500	-20.15800
O	18.40500	-34.88100	-13.27700

H	18.68500	-35.54900	-12.65000
H	19.19000	-34.68800	-13.78900
O	16.20900	-15.70500	-21.71400
H	15.63600	-15.08600	-22.16700
H	16.78200	-15.15500	-21.18000
O	22.27600	-26.46000	-9.52900
H	23.22800	-26.36000	-9.50800
H	22.10600	-27.25200	-9.02000
O	20.29800	-29.37400	-9.34300
H	19.70200	-29.09400	-10.03900
H	20.01200	-28.88500	-8.57200
O	19.42800	-15.42200	-20.58300
H	20.36100	-15.48700	-20.38000
H	19.34100	-14.58600	-21.04200
O	22.05700	-16.10500	-23.62300
H	21.97800	-16.43900	-22.72900
H	22.87800	-15.61300	-23.62400
O	24.75500	-23.23900	-10.41500
H	24.72300	-24.17700	-10.22900
H	25.36500	-23.16100	-11.14900
O	16.59300	-24.36900	-24.37200
H	17.53900	-24.49900	-24.30400
H	16.21500	-25.10800	-23.89500
O	24.90400	-26.44800	-10.36900
H	24.38800	-27.23200	-10.55600
H	25.78800	-26.77600	-10.20500
O	22.56500	-32.70400	-14.41000
H	22.36800	-33.01200	-13.52500
H	23.17500	-31.97800	-14.28000
O	30.57300	-22.17700	-11.27500
H	31.30300	-22.43900	-10.71400
H	29.96300	-22.91400	-11.23400
O	28.65400	-24.03700	-10.67700
H	27.92700	-24.17100	-11.28500
H	28.23900	-23.72900	-9.87100
O	16.47700	-20.31800	-2.91200
H	17.39700	-20.14000	-2.71800
H	16.44200	-21.26200	-3.06400
O	14.77700	-26.44400	-24.43700
H	13.87100	-26.74100	-24.36100
H	15.05500	-26.74100	-25.30400
O	5.69500	-25.12400	-13.98700
H	5.37600	-25.97700	-14.28000
H	5.14900	-24.90900	-13.23100
O	9.47300	-22.40700	-10.27000
H	9.69200	-21.47600	-10.25100
H	9.88600	-22.76700	-9.48500
O	16.39400	-31.90100	-18.28300
H	15.58400	-32.24600	-17.90700
H	17.08200	-32.21000	-17.69400
O	18.32300	-32.82300	-16.66000
H	18.87600	-32.40300	-16.00100
H	18.56000	-33.74900	-16.61800
O	14.34600	-26.83400	-0.88000
H	14.24600	-25.88500	-0.94900
H	14.12800	-27.16200	-1.75200
O	13.54700	-29.58600	-24.29600
H	14.40900	-29.26000	-24.03600
H	13.73200	-30.37300	-24.80800
O	5.56400	-21.50300	-12.01800
H	4.87000	-21.91800	-12.53100
H	6.31100	-21.46100	-12.61400
O	21.70100	-30.71300	-17.15200
H	21.12100	-30.05400	-17.53400
H	21.43400	-31.53500	-17.56300
O	20.15100	-31.31200	-14.95000
H	20.65100	-31.20100	-15.75900
H	20.79500	-31.61600	-14.31000
O	14.20900	-23.40400	-2.06300
H	13.72600	-22.60400	-1.85700
H	14.29500	-23.39400	-3.01700
O	7.47800	-21.57200	-14.14100
H	7.45500	-21.47300	-15.09300
H	7.78800	-20.72500	-13.81900
O	10.13700	-19.51400	-10.02600
H	11.09100	-19.54600	-10.09300
H	9.93800	-19.97500	-9.21100
O	10.72600	-20.94400	-7.45900
H	11.54600	-20.44900	-7.43600
H	11.00000	-21.86100	-7.48800
O	20.48300	-28.55300	-18.09700
H	19.59600	-28.26800	-18.31500
H	20.89900	-28.71300	-18.94400
O	23.83500	-28.52800	-14.38100
H	24.61100	-28.03900	-14.10900
H	23.10900	-28.10200	-13.92400
O	26.27200	-27.81500	-13.32400
H	26.86600	-28.05600	-14.03400
H	26.34400	-28.53500	-12.69600
O	8.49200	-13.97200	-13.00900
H	8.35700	-14.73200	-13.57600
H	8.07500	-13.24800	-13.47700
O	19.62700	-18.78800	-24.00200
H	20.33100	-18.58300	-23.38600
H	18.90400	-18.22100	-23.73700
O	20.45700	-23.03700	-17.88200
H	20.40100	-22.58000	-18.72100
H	21.01000	-23.79800	-18.05900
O	24.26800	-27.24800	-17.05700
H	24.01100	-27.64900	-16.22600
H	23.64300	-26.53400	-17.18200
O	28.57900	-24.50900	-14.54900
H	28.01900	-24.43900	-13.77600
H	27.97300	-24.68500	-15.26900
O	11.64800	-13.07500	-12.79800
H	11.53200	-12.13100	-12.90000
H	10.82800	-13.37800	-12.40900
O	21.53600	-18.35000	-21.95200
H	22.37100	-18.78200	-22.13400
H	21.37500	-18.52200	-21.02400
O	21.19100	-21.84700	-20.58600
H	21.13600	-22.01200	-21.52700
H	20.56500	-21.13900	-20.43000
O	5.98000	-26.61000	-6.76000
H	6.62600	-27.12000	-6.27100
H	5.77200	-25.87300	-6.18600
O	18.50500	-19.54200	-1.26100
H	19.37400	-19.36800	-1.62200

H	18.02600	-18.72300	-1.38300
O	14.50000	-32.34600	-21.00800
H	14.48800	-31.40200	-20.85100
H	15.39300	-32.52900	-21.30200
O	12.67400	-12.42200	-9.58200
H	13.57700	-12.39000	-9.26500
H	12.44800	-13.35200	-9.56700
O	14.42900	-13.66900	-12.92100
H	13.47900	-13.66900	-13.03500
H	14.73300	-14.41600	-13.43700
O	18.31600	-35.75200	-16.75300
H	17.54900	-36.30600	-16.60800
H	18.72700	-35.68000	-15.89200
O	10.32000	-29.46300	-2.66300
H	11.18300	-29.82000	-2.45200
H	9.74600	-29.80200	-1.97500
O	30.16500	-7.83300	-11.85600
H	30.80200	-7.95200	-12.56000
H	30.04700	-6.88500	-11.79800
O	26.97400	-7.88000	-11.92000
H	26.63500	-7.99600	-12.80800
H	27.84200	-8.28100	-11.94000
O	22.83200	-6.02700	-15.93100
H	22.26900	-6.11800	-16.69900
H	22.71700	-6.84700	-15.45000
O	27.45500	-6.72700	-5.96200
H	27.44400	-7.12200	-6.83400
H	27.61800	-7.46000	-5.36800
O	20.37900	-4.53200	-13.09200
H	21.17100	-4.87300	-13.50800
H	20.61800	-4.42200	-12.17200
O	28.41200	-2.36000	-20.61900
H	27.76100	-1.77400	-21.00600
H	29.20700	-1.82900	-20.56000
O	22.33800	-5.27100	-20.22000
H	23.11100	-5.81800	-20.35900
H	22.69000	-4.40600	-20.00900
O	24.17600	-3.94300	-7.01400
H	23.88500	-3.03900	-7.12900
H	24.81200	-3.90200	-6.29900
O	25.10200	-6.56500	-10.42400
H	25.69800	-6.79000	-11.13800
H	25.15900	-7.30700	-9.82100
O	24.67400	-9.11200	-22.58900
H	24.92700	-9.63300	-23.35000
H	23.73700	-9.27500	-22.48600
O	29.71900	-10.10800	-14.78100
H	30.50500	-10.52600	-15.13200
H	29.98300	-9.20300	-14.61300
O	29.89000	-4.67100	-20.08200
H	29.25400	-4.59700	-19.37000
H	29.55900	-4.08100	-20.76000
O	35.12000	-10.17000	-13.87800
H	35.85200	-10.74100	-13.64400
H	34.36300	-10.55800	-13.43700
O	24.39400	-7.20900	-20.38600
H	24.27800	-7.88400	-19.71800
H	24.50400	-7.69500	-21.20300
O	26.73700	-7.74800	-16.68100
H	26.37300	-8.27500	-15.97000
H	27.59400	-8.13800	-16.85500
O	32.64200	-16.45800	-9.41800
H	32.31000	-15.58900	-9.64200
H	32.04700	-16.77500	-8.73900
O	26.37800	-12.71900	-21.68500
H	26.54500	-11.85100	-21.31800
H	26.18700	-12.55600	-22.60900
O	29.13500	-4.25700	-10.01900
H	28.83500	-3.35500	-10.12700
H	28.79000	-4.52400	-9.16700
O	22.58600	-7.80600	-13.98500
H	21.66200	-7.93800	-13.77700
H	22.83500	-7.02600	-13.48800
O	32.53500	-11.73900	-12.33900
H	31.62700	-11.80700	-12.63300
H	32.48400	-11.26200	-11.51100
O	26.35700	-10.26300	-20.70100
H	26.23700	-9.81700	-19.86200
H	25.66200	-9.91400	-21.25900
O	19.94700	-8.16200	-13.15100
H	20.07700	-7.90900	-12.23700
H	19.26700	-7.57000	-13.47000
O	24.11700	2.50700	-17.05200
H	24.04000	3.45800	-16.97300
H	23.51300	2.16200	-16.39400
O	26.60900	-10.32000	-4.39600
H	25.83200	-9.76000	-4.40500
H	27.24800	-9.83400	-3.87400
O	34.40100	-13.53700	-10.56100
H	33.75900	-13.24400	-11.20700
H	33.89700	-13.65200	-9.75600
O	32.05500	-9.79100	-19.87300
H	32.96700	-9.55800	-20.04800
H	31.54600	-9.20700	-20.43500
O	24.86400	-7.51100	-4.83400
H	25.60500	-6.97300	-5.11000
H	24.15700	-6.88500	-4.67700
O	30.33900	-5.36800	-6.23600
H	30.74800	-5.81000	-6.98100
H	29.61700	-5.94300	-5.98300
O	26.69200	-10.43100	-8.86300
H	27.53800	-10.00700	-8.72000
H	26.63200	-11.08800	-8.16900
O	30.66200	-4.80800	-12.48100
H	30.03300	-4.64400	-13.18400
H	30.27500	-4.39400	-11.71000
O	34.54100	-3.87300	-15.12700
H	33.85600	-3.49300	-15.67700
H	34.62500	-3.26300	-14.39400
O	29.84200	-12.77100	-7.20500
H	29.59800	-12.01600	-7.74200
H	30.11800	-12.39200	-6.37000
O	31.79600	-6.22700	-8.52700
H	32.00800	-5.58400	-9.20400
H	31.74900	-7.06100	-8.99500
O	29.39900	-13.61500	-11.62400
H	28.53700	-13.31700	-11.33200
H	29.42500	-13.39800	-12.55600

O	26.21500	-5.07100	-15.21600
H	26.67900	-4.79200	-16.00600
H	25.95100	-5.97200	-15.39900
O	23.10700	-5.52400	-12.27800
H	23.67700	-5.09500	-12.91700
H	23.69800	-5.80500	-11.58000
O	25.15200	-8.94800	-18.40700
H	25.85400	-8.49600	-17.94000
H	24.82000	-9.59000	-17.77900
O	23.60000	-10.35700	-4.03200
H	22.97000	-9.67600	-3.79900
H	23.49300	-10.46900	-4.97700
O	18.89400	-10.69000	-13.10600
H	18.10400	-10.43400	-13.58200
H	19.38500	-9.87500	-13.00000
O	32.91100	-3.35000	-10.35500
H	33.73200	-3.75200	-10.63700
H	32.90700	-3.45300	-9.40300
O	20.84600	-10.32600	-16.14700
H	20.04900	-10.21400	-15.62900
H	20.61800	-9.98700	-17.01300
O	26.67600	-3.80100	-8.19900
H	26.74100	-3.33900	-7.36300
H	25.96700	-4.43100	-8.07100
O	17.17800	-10.43700	-10.67400
H	17.42900	-9.55200	-10.40800
H	17.71400	-10.61700	-11.44700
O	41.00100	-7.00600	-14.00700
H	40.24800	-7.57500	-14.16200
H	41.13600	-7.03900	-13.05900
O	26.39100	-2.13400	-18.33100
H	26.25600	-1.28900	-17.90300
H	26.93200	-1.93200	-19.09400
O	22.87800	-16.05500	-1.67000
H	22.48500	-16.60900	-0.99500
H	23.69400	-15.74400	-1.27900
O	19.84400	-4.61700	-17.75800
H	20.35700	-3.82300	-17.60800
H	20.49600	-5.31400	-17.82500
O	21.65400	-1.50000	-15.67600
H	21.83000	-0.57800	-15.86400
H	22.44900	-1.81400	-15.24700
O	25.79500	-14.63900	-4.07800
H	26.53600	-14.03900	-4.15700
H	26.02600	-15.38200	-4.63600
O	20.93900	-9.45400	-5.80000
H	20.82400	-10.35500	-5.49900
H	20.08300	-9.20700	-6.15100
O	28.01800	-1.51200	-4.20100
H	27.57400	-2.29800	-4.51900
H	28.68000	-1.32400	-4.86700
O	22.77700	0.01800	-9.67500
H	23.32700	-0.11100	-10.44700
H	21.93800	0.31700	-10.02500
O	30.11600	-1.23200	-6.17300
H	30.09800	-0.41100	-6.66500
H	31.03800	-1.35600	-5.94800
O	26.57300	-3.71700	-5.33100
H	27.10600	-4.47900	-5.56100
H	26.04500	-4.01000	-4.58800
O	29.74000	-18.61100	-9.80100
H	29.39700	-17.72100	-9.87700
H	30.10800	-18.80100	-10.66400
O	17.80900	-6.28800	-16.98200
H	18.64600	-5.87800	-17.19900
H	17.98700	-7.22800	-17.01200
O	29.70500	-8.62000	-21.29400
H	29.19000	-7.85000	-21.05500
H	29.43300	-9.29400	-20.67100
O	27.29000	-13.77500	-16.23500
H	26.43400	-14.16500	-16.41500
H	27.70300	-13.69600	-17.09500
O	28.82000	-16.24100	-11.83600
H	28.31700	-16.27800	-12.64900
H	29.11300	-15.33200	-11.77700
O	25.06400	-16.15700	-12.83000
H	25.93000	-16.35300	-13.18900
H	25.13200	-16.38600	-11.90300
O	34.67900	-5.86700	-19.35900
H	33.90200	-6.20600	-19.80300
H	35.35800	-5.86200	-20.03300
O	29.49400	-10.74800	-19.46800
H	29.18900	-10.43300	-18.61700
H	30.44700	-10.67000	-19.42500
O	27.93900	-4.58900	-17.81900
H	27.41100	-5.32300	-18.13100
H	27.43700	-3.81100	-18.06500
O	32.91800	-6.39400	-12.70200
H	32.26000	-5.70400	-12.61400
H	33.72200	-6.00400	-12.35900
O	22.67100	-13.92700	-15.45800
H	23.24500	-14.14900	-16.19100
H	23.25800	-13.85800	-14.70500
O	31.72900	-8.03900	-14.65200
H	32.24200	-7.40600	-14.14900
H	32.37900	-8.63900	-15.01700
O	22.99800	-11.89600	-21.43800
H	22.16100	-11.55900	-21.11800
H	22.85800	-12.03100	-22.37600
O	24.97300	-17.05300	-16.73100
H	24.31900	-17.36800	-17.35500
H	24.93100	-17.67500	-16.00500
O	26.08400	-6.77100	-24.81500
H	25.17700	-7.00200	-24.61600
H	26.58100	-7.56700	-24.62800
O	28.99100	1.93900	-21.89600
H	28.31700	2.24200	-21.28700
H	29.37700	2.74200	-22.24500
O	28.20700	-10.56400	-12.40600
H	27.28900	-10.34300	-12.56400
H	28.62300	-10.49000	-13.26500
O	28.99000	-9.16500	-17.27500
H	29.73700	-8.65300	-17.58500
H	29.25900	-9.48700	-16.41500
O	27.26300	-6.66900	-20.29300
H	26.35400	-6.95400	-20.19600
H	27.29200	-6.25000	-21.15200
O	32.49500	-3.05900	-16.52400

H	32.04100	-3.90000	-16.46700
H	31.86400	-2.42300	-16.18600
O	31.43800	-12.13000	-15.29400
H	30.70000	-12.64200	-14.96300
H	31.69100	-12.57000	-16.10500
O	30.93800	-1.22100	-15.58900
H	31.23000	-0.66100	-16.30700
H	31.44800	-0.92800	-14.83300
O	31.53900	-19.35200	-14.69900
H	31.84500	-20.21200	-14.98700
H	32.26400	-18.76000	-14.90000
O	17.83200	-8.94900	-16.31100
H	17.63400	-9.54200	-17.03600
H	17.27600	-9.25400	-15.59400
O	25.81100	-9.05700	-14.19200
H	25.86600	-10.00900	-14.26800
H	25.00000	-8.90100	-13.70800
O	26.96000	-5.12900	-22.95400
H	26.52200	-5.84500	-23.41300
H	27.70100	-4.90100	-23.51500
O	23.75000	-10.43900	-16.43300
H	22.93700	-10.13900	-16.02600
H	24.24600	-10.83300	-15.71500
O	25.90100	-0.44300	-14.49900
H	25.85900	-0.21700	-15.42800
H	26.81800	-0.67700	-14.35100
O	29.34800	-9.97500	-7.60100
H	29.70300	-9.74800	-6.74200
H	29.99500	-9.64300	-8.22400
O	35.07800	-6.85300	-16.89600
H	34.93900	-6.66300	-17.82400
H	34.72100	-6.09100	-16.44000
O	23.24200	-12.86700	-10.81700
H	23.88200	-13.00800	-11.51400
H	22.41000	-13.15400	-11.19300
O	35.10500	-4.78500	-11.88500
H	35.53800	-4.87800	-11.03700
H	35.81800	-4.80900	-12.52300
O	24.01800	-3.56700	-14.70300
H	24.81200	-4.06200	-14.90500
H	23.30700	-4.09500	-15.06600
O	28.83700	-1.18500	-10.35200
H	28.89100	-0.61600	-9.58400
H	27.96900	-1.01500	-10.71700
O	20.32600	-9.42300	-18.62700
H	19.39600	-9.48400	-18.41000
H	20.42200	-9.94500	-19.42400
O	20.24500	-12.89700	-16.03900
H	20.50500	-11.98200	-16.14800
H	21.06600	-13.36500	-15.89000
O	21.71700	-11.48300	-8.86900
H	22.39000	-11.69200	-9.51800
H	20.89000	-11.68100	-9.30900
O	35.96600	-4.74300	-9.35200
H	36.16000	-3.83800	-9.10600
H	35.86600	-5.20200	-8.51800
O	27.62000	-14.85700	-6.85300
H	26.97300	-14.15800	-6.94600
H	28.39000	-14.42300	-6.48500
O	31.02800	-4.59200	-3.77800
H	30.87600	-4.82100	-4.69500
H	31.01800	-3.63500	-3.76500
O	27.79600	-7.01900	-8.79300
H	28.72500	-6.86800	-8.96600
H	27.37500	-6.93100	-9.64800
O	26.59500	-2.99400	-11.02300
H	26.45600	-3.36200	-10.15100
H	25.80700	-2.47900	-11.19500
O	27.19000	2.10700	-19.68100
H	27.36500	1.63600	-18.86600
H	26.50300	2.73400	-19.45300
O	28.14600	-3.61600	-13.63400
H	27.81100	-3.65600	-12.73900
H	27.64600	-4.28100	-14.10700
O	22.12000	-8.20900	-8.10900
H	21.98200	-8.94100	-7.50800
H	23.03000	-8.29800	-8.39100
O	31.08100	-6.27000	-23.36100
H	30.46400	-6.99500	-23.45700
H	30.61300	-5.50900	-23.70400
O	24.74800	-8.49700	-8.62100
H	25.45800	-9.13800	-8.66200
H	24.70500	-8.24600	-7.69800
O	32.73700	-1.56000	-5.70000
H	32.67800	-2.24400	-6.36700
H	33.13900	-0.81800	-6.15200
O	23.46100	-11.51000	-6.80100
H	22.92500	-11.96100	-6.14900
H	22.87300	-11.37600	-7.54400
O	21.75600	5.25600	-12.90700
H	21.99600	5.88800	-12.22900
H	20.91300	4.90500	-12.62000
O	22.87100	-12.05200	-24.23900
H	21.99400	-12.17300	-24.60200
H	23.34700	-12.84300	-24.49100
O	32.73900	-3.59600	-7.46900
H	31.85100	-3.93400	-7.34600
H	33.30800	-4.32800	-7.23200
O	14.39200	-7.26400	-14.14400
H	15.07900	-7.57800	-14.73200
H	14.23600	-6.36000	-14.41800
O	19.19500	-12.37500	-9.71600
H	19.11100	-12.98200	-10.45100
H	18.37300	-11.88400	-9.71500
O	26.26900	1.06400	-10.50300
H	26.19600	1.86900	-9.99100
H	25.82600	1.26300	-11.32800
O	26.05800	0.30100	-16.99200
H	25.27400	0.84300	-16.89900
H	26.75700	0.81800	-16.59300
O	29.36700	-13.89000	-14.34900
H	29.86400	-14.64300	-14.66900
H	28.59600	-13.85300	-14.91500
O	37.22000	-8.18500	-12.70700
H	37.20900	-7.30300	-13.07800
H	36.35200	-8.53800	-12.90200
O	28.72900	0.96300	-8.34700
H	29.44100	1.59700	-8.41900

H	28.23800	1.05900	-9.16300
O	30.45000	-5.13200	-16.77500
H	29.51600	-5.01200	-16.94500
H	30.62800	-6.03500	-17.03900
O	25.95100	-11.74800	-14.62200
H	25.58100	-12.30100	-13.93400
H	26.44700	-12.35200	-15.17400
O	33.83800	-9.29100	-16.24000
H	34.30400	-8.45800	-16.31400
H	34.28800	-9.75600	-15.53600
O	23.78000	-1.28800	-7.52600
H	24.48100	-0.65400	-7.37300
H	23.30900	-0.94700	-8.28700
O	31.90500	-2.94600	-19.24200
H	32.18100	-3.06100	-18.33200
H	31.03600	-3.34400	-19.28000
O	31.80900	-16.92300	-12.20100
H	30.89600	-16.69200	-12.03300
H	32.23900	-16.83900	-11.35000
O	31.58000	-8.73400	-9.70100
H	30.92700	-8.48600	-10.35600
H	31.95800	-9.54700	-10.03500
O	28.54100	1.30200	-15.59800
H	28.72300	0.48500	-15.13500
H	29.36400	1.51900	-16.03600
O	28.87500	0.22900	-18.33700
H	29.40400	-0.28200	-18.95000
H	29.13800	-0.08700	-17.47200
O	27.62900	-7.99200	-3.21400
H	26.95300	-8.09200	-2.54400
H	28.45100	-8.14800	-2.75100
O	19.37700	-1.75400	-10.59300
H	19.96200	-2.50000	-10.46000
H	19.17900	-1.44400	-9.71000
O	22.23000	1.28100	-15.57700
H	21.56100	1.76500	-16.06100
H	22.09200	1.52800	-14.66300
O	13.96800	-9.18400	-11.87300
H	14.00700	-9.71700	-11.07900
H	13.46200	-9.71000	-12.49200
O	26.37100	3.31500	-8.97900
H	26.92300	3.47800	-8.21400
H	26.71200	3.90600	-9.65100
O	21.34700	-14.33700	-8.37600
H	21.41200	-13.47600	-8.78900
H	21.28400	-14.95000	-9.10900
O	20.82300	-12.26500	-4.72900
H	21.45000	-12.64700	-4.11500
H	19.96500	-12.48400	-4.36400
O	31.22200	-1.85900	-3.12400
H	31.76600	-1.24600	-3.61800
H	30.64100	-1.30100	-2.60700
O	17.59900	-10.45700	-18.79700
H	17.89500	-11.36600	-18.85100
H	16.69800	-10.47900	-19.11900
O	29.50800	1.26300	-11.99200
H	29.38300	0.46200	-12.50100
H	30.16600	1.75800	-12.48100
O	21.12900	-12.38200	-12.81500
H	20.40700	-11.76800	-12.95400
H	21.91800	-11.84600	-12.89300
O	31.57100	-0.93600	-10.36400
H	30.62400	-1.05500	-10.43700
H	31.92000	-1.82400	-10.28200
O	23.93100	-15.04600	-6.54100
H	24.45500	-14.80000	-5.77800
H	23.71200	-14.21400	-6.96000
O	19.32800	-12.77600	-18.67300
H	18.99900	-13.65600	-18.85600
H	19.68200	-12.83100	-17.78600
O	28.57600	-1.14100	-14.16400
H	28.42800	-2.06900	-13.98300
H	29.40100	-1.11900	-14.64800
O	22.44200	-12.48900	-18.30600
H	22.78200	-11.96000	-19.02800
H	22.88900	-12.15100	-17.53000
O	32.17900	-0.31800	-13.07700
H	33.10400	-0.52300	-13.21500
H	32.01100	-0.56600	-12.16900
O	25.13700	-14.10500	-8.85600
H	24.79600	-14.90400	-8.45300
H	24.43000	-13.80500	-9.42800
O	25.78800	-17.02000	-10.40300
H	26.61100	-17.49400	-10.52500
H	26.03200	-16.24100	-9.90300
O	24.42900	-13.99100	-20.10700
H	25.15000	-13.61300	-20.61000
H	23.80600	-13.27200	-20.00300
O	28.57200	-13.28700	-19.17100
H	28.78800	-12.40400	-19.47200
H	28.09200	-13.67800	-19.90000
O	23.31200	-10.32800	-12.59400
H	23.16600	-10.01400	-11.70100
H	22.83500	-9.71000	-13.14700
O	25.17200	-13.33800	-12.54600
H	25.15800	-14.23400	-12.88200
H	25.88900	-13.33100	-11.91200
O	27.02400	-12.90400	-10.50000
H	26.76600	-11.98200	-10.50200
H	26.49300	-13.30100	-9.81000
O	31.96900	0.20100	-17.72100
H	32.14300	-0.36400	-18.47400
H	32.46200	1.00100	-17.90300
O	17.78700	-7.78800	-9.65500
H	17.24200	-7.01200	-9.52300
H	18.66500	-7.44100	-9.81300
O	24.57500	-14.45600	-17.27700
H	24.69700	-15.38500	-17.07800
H	24.28600	-14.44300	-18.18900
O	27.44400	-16.77100	-14.14000
H	27.46700	-16.70700	-15.09500
H	27.86100	-17.61100	-13.94600
O	37.82300	-7.03500	-16.10200
H	38.26600	-7.78500	-16.50000
H	36.94500	-7.04400	-16.48500
O	32.95600	-0.48200	-20.62200
H	33.65200	0.13800	-20.40300
H	33.29900	-1.33300	-20.34600

O	18.57300	-8.66100	-6.76200
H	17.83100	-8.51200	-6.17500
H	18.25200	-8.40000	-7.62500
O	34.85600	-3.03200	-19.09300
H	35.38100	-2.69400	-19.81800
H	34.76800	-3.96800	-19.27600
O	26.51800	0.88100	-6.96400
H	26.82600	0.63600	-6.09200
H	27.31500	1.08600	-7.45200
O	26.54000	3.03500	-14.73300
H	26.10000	2.54200	-14.04000
H	27.29000	2.49200	-14.97400
O	19.01900	-0.77400	-14.73100
H	19.84500	-1.07400	-15.11100
H	18.66800	-1.54300	-14.28200
O	23.98700	-1.42100	-12.66100
H	23.36500	-2.08400	-12.96100
H	24.65700	-1.39500	-13.34400
O	17.22000	-2.85500	-13.77200
H	16.38300	-3.28300	-13.95400
H	17.19200	-2.66200	-12.83500
O	24.05900	-12.94300	-3.06800
H	24.10800	-12.06700	-3.45000
H	24.60200	-13.48600	-3.64100
O	34.54500	-2.39900	-3.78800
H	33.75500	-2.41700	-4.32700
H	34.30800	-1.86000	-3.03300
O	34.66500	-5.55000	-6.47400
H	34.71500	-5.71500	-5.53300
H	34.55600	-6.41700	-6.86500
O	23.54800	-2.87100	-19.18500
H	23.69700	-2.18800	-19.83900
H	24.35500	-2.89100	-18.67000
O	31.95500	-9.56700	-5.15800
H	32.85600	-9.70600	-5.45000
H	32.03000	-9.40100	-4.21900
O	26.28900	-12.19000	-6.31300
H	25.42000	-11.89700	-6.58500
H	26.52500	-11.60600	-5.59200
O	36.16700	-3.13000	-6.23000
H	35.87500	-2.76700	-5.39400
H	35.55400	-3.84400	-6.40900
O	37.36600	-5.56500	-13.71000
H	38.20100	-5.12600	-13.54600
H	37.50200	-6.04100	-14.52900
O	34.41500	-8.00700	-7.76200
H	34.89100	-7.81400	-8.57000
H	33.49200	-7.95100	-8.00800
O	21.30400	-6.84500	-18.17900
H	21.05800	-7.74800	-17.98100
H	21.47300	-6.84300	-19.12100
O	28.99300	-15.97200	-8.79700
H	29.51100	-15.23600	-9.12200
H	28.63900	-15.66600	-7.96200
O	20.57300	-11.11200	-20.68000
H	19.88000	-11.03700	-21.33600
H	20.32800	-11.87600	-20.15800
O	29.24500	-19.55200	-13.27700
H	29.52500	-20.22000	-12.65000
H	30.03000	-19.35900	-13.78900
O	27.04800	-0.37600	-21.71400
H	26.47500	0.24300	-22.16700
H	27.62100	0.17400	-21.18000
O	33.11500	-11.13000	-9.52900
H	34.06700	-11.03100	-9.50800
H	32.94600	-11.92300	-9.02000
O	31.13700	-14.04500	-9.34300
H	30.54200	-13.76400	-10.03900
H	30.85200	-13.55500	-8.57200
O	30.26800	-0.09300	-20.58300
H	31.20100	-0.15800	-20.38000
H	30.18000	0.74300	-21.04200
O	32.89600	-0.77600	-23.62300
H	32.81800	-1.11000	-22.72900
H	33.71700	-0.28400	-23.62400
O	35.59400	-7.91000	-10.41500
H	35.56200	-8.84800	-10.22900
H	36.20400	-7.83100	-11.14900
O	27.43300	-9.04000	-24.37200
H	28.37800	-9.17000	-24.30400
H	27.05400	-9.77900	-23.89500
O	35.74400	-11.11900	-10.36900
H	35.22800	-11.90300	-10.55600
H	36.62800	-11.44700	-10.20500
O	33.40500	-17.37500	-14.41000
H	33.20700	-17.68300	-13.52500
H	34.01500	-16.64800	-14.28000
O	41.41200	-6.84800	-11.27500
H	42.14200	-7.11000	-10.71400
H	40.80300	-7.58500	-11.23400
O	39.49300	-8.70700	-10.67700
H	38.76700	-8.84200	-11.28500
H	39.07800	-8.40000	-9.87100
O	27.31600	-4.98800	-2.91200
H	28.23700	-4.81100	-2.71800
H	27.28100	-5.93300	-3.06400
O	25.61700	-11.11500	-24.43700
H	24.71000	-11.41200	-24.36100
H	25.89400	-11.41200	-25.30400
O	16.53500	-9.79500	-13.98700
H	16.21500	-10.64800	-14.28000
H	15.98800	-9.58000	-13.23100
O	20.31300	-7.07800	-10.27000
H	20.53100	-6.14700	-10.25100
H	20.72500	-7.43700	-9.48500
O	27.23300	-16.57100	-18.28300
H	26.42300	-16.91700	-17.90700
H	27.92200	-16.88100	-17.69400
O	29.16200	-17.49400	-16.66000
H	29.71500	-17.07400	-16.00100
H	29.39900	-18.42000	-16.61800
O	25.18500	-11.50500	-0.88000
H	25.08500	-10.55600	-0.94900
H	24.96800	-11.83300	-1.75200
O	24.38700	-14.25600	-24.29600
H	25.24900	-13.93100	-24.03600
H	24.57200	-15.04300	-24.80800
O	16.40300	-6.17400	-12.01800

H	15.71000	-6.58900	-12.53100
H	17.15100	-6.13200	-12.61400
O	32.54000	-15.38400	-17.15200
H	31.96000	-14.72500	-17.53400
H	32.27400	-16.20600	-17.56300
O	30.99000	-15.98300	-14.95000
H	31.49000	-15.87100	-15.75900
H	31.63400	-16.28700	-14.31000
O	25.04900	-8.07500	-2.06300
H	24.56500	-7.27500	-1.85700
H	25.13500	-8.06400	-3.01700
O	18.31700	-6.24200	-14.14100
H	18.29400	-6.14400	-15.09300
H	18.62700	-5.39600	-13.81900
O	20.97600	-4.18500	-10.02600
H	21.93000	-4.21600	-10.09300
H	20.77700	-4.64600	-9.21100
O	21.56600	-5.61500	-7.45900
H	22.38500	-5.12000	-7.43600
H	21.84000	-6.53100	-7.48800
O	31.32300	-13.22400	-18.09700
H	30.43500	-12.93900	-18.31500
H	31.73800	-13.38400	-18.94400
O	34.67400	-13.19900	-14.38100
H	35.45000	-12.70900	-14.10900
H	33.94900	-12.77300	-13.92400
O	37.11100	-12.48600	-13.32400
H	37.70600	-12.72700	-14.03400
H	37.18400	-13.20500	-12.69600
O	19.33100	1.35700	-13.00900
H	19.19600	0.59700	-13.57600
H	18.91400	2.08100	-13.47700
O	30.46700	-3.45900	-24.00200
H	31.17000	-3.25400	-23.38600
H	29.74300	-2.89100	-23.73700
O	31.29700	-7.70800	-17.88200
H	31.24000	-7.25100	-18.72100
H	31.84900	-8.46900	-18.05900
O	35.10700	-11.91900	-17.05700
H	34.85100	-12.32000	-16.22600
H	34.48200	-11.20500	-17.18200
O	39.41800	-9.18000	-14.54900
H	38.85800	-9.11000	-13.77600
H	38.81300	-9.35500	-15.26900
O	22.48800	2.25400	-12.79800
H	22.37100	3.19900	-12.90000
H	21.66700	1.95100	-12.40900
O	32.37500	-3.02100	-21.95200
H	33.21000	-3.45300	-22.13400
H	32.21400	-3.19300	-21.02400
O	32.03000	-6.51800	-20.58600
H	31.97500	-6.68300	-21.52700
H	31.40500	-5.81000	-20.43000
O	16.81900	-11.28100	-6.76000
H	17.46500	-11.79100	-6.27100
H	16.61100	-10.54400	-6.18600
O	29.34500	-4.21300	-1.26100
H	30.21400	-4.03900	-1.62200
H	28.86500	-3.39300	-1.38300
O	25.34000	-17.01700	-21.00800
H	25.32800	-16.07300	-20.85100
H	26.23200	-17.20000	-21.30200
O	23.51300	2.90700	-9.58200
H	24.41600	2.93900	-9.26500
H	23.28700	1.97700	-9.56700
O	25.26900	1.66000	-12.92100
H	24.31800	1.66000	-13.03500
H	25.57200	0.91300	-13.43700
O	29.15500	-20.42300	-16.75300
H	28.38800	-20.97700	-16.60800
H	29.56600	-20.35000	-15.89200
O	21.15900	-14.13400	-2.66300
H	22.02200	-14.49000	-2.45200
H	20.58600	-14.47300	-1.97500
O	41.00400	7.49600	-11.85600
H	41.64200	7.37700	-12.56000
H	40.88600	8.44400	-11.79800
O	37.81300	7.44900	-11.92000
H	37.47500	7.33300	-12.80800
H	38.68200	7.04800	-11.94000
O	33.67200	9.30200	-15.93100
H	33.10800	9.21100	-16.69900
H	33.55700	8.48200	-15.45000
O	38.29500	8.60200	-5.96200
H	38.28400	8.20800	-6.83400
H	38.45700	7.86900	-5.36800
O	31.21800	10.79700	-13.09200
H	32.01000	10.45700	-13.50800
H	31.45800	10.90700	-12.17200
O	39.25200	12.96900	-20.61900
H	38.60000	13.55500	-21.00600
H	40.04600	13.50000	-20.56000
O	33.17700	10.05800	-20.22000
H	33.95000	9.51100	-20.35900
H	33.53000	10.92300	-20.00900
O	35.01600	11.38600	-7.01400
H	34.72500	12.29100	-7.12900
H	35.65100	11.42800	-6.29900
O	35.94100	8.76400	-10.42400
H	36.53800	8.53900	-11.13800
H	35.99900	8.02200	-9.82100
O	35.51400	6.21800	-22.58900
H	35.76600	5.69600	-23.35000
H	34.57600	6.05400	-22.48600
O	40.55800	5.22100	-14.78100
H	41.34400	4.80300	-15.13200
H	40.82200	6.12600	-14.61300
O	40.72900	10.65900	-20.08200
H	40.09400	10.73200	-19.37000
H	40.39900	11.24800	-20.76000
O	35.23300	8.12000	-20.38600
H	35.11700	7.44500	-19.71800
H	35.34400	7.63400	-21.20300
O	37.57700	7.58100	-16.68100
H	37.21200	7.05500	-15.97000
H	38.43300	7.19100	-16.85500
O	37.21700	2.61000	-21.68500
H	37.38400	3.47800	-21.31800

H	37.02700	2.77300	-22.60900
O	39.97500	11.07200	-10.01900
H	39.67500	11.97500	-10.12700
H	39.63000	10.80500	-9.16700
O	33.42600	7.52400	-13.98500
H	32.50100	7.39100	-13.77700
H	33.67400	8.30300	-13.48800
O	37.19600	5.06600	-20.70100
H	37.07600	5.51200	-19.86200
H	36.50100	5.41500	-21.25900
O	30.78700	7.16700	-13.15100
H	30.91600	7.42000	-12.23700
H	30.10600	7.76000	-13.47000
O	34.95700	17.83700	-17.05200
H	34.87900	18.78700	-16.97300
H	34.35300	17.49100	-16.39400
O	37.44800	5.00900	-4.39600
H	36.67200	5.56900	-4.40500
H	38.08700	5.49500	-3.87400
O	35.70300	7.81800	-4.83400
H	36.44400	8.35600	-5.11000
H	34.99700	8.44500	-4.67700
O	41.17900	9.96100	-6.23600
H	41.58700	9.51900	-6.98100
H	40.45600	9.38600	-5.98300
O	37.53200	4.89800	-8.86300
H	38.37800	5.32200	-8.72000
H	37.47200	4.24100	-8.16900
O	41.50100	10.52100	-12.48100
H	40.87200	10.68600	-13.18400
H	41.11400	10.93500	-11.71000
O	40.68200	2.55900	-7.20500
H	40.43800	3.31300	-7.74200
H	40.95800	2.93700	-6.37000
O	42.63500	9.10200	-8.52700
H	42.84700	9.74600	-9.20400
H	42.58800	8.26900	-8.99500
O	40.23800	1.71400	-11.62400
H	39.37700	2.01200	-11.33200
H	40.26400	1.93100	-12.55600
O	37.05400	10.25800	-15.21600
H	37.51800	10.53700	-16.00600
H	36.79000	9.35700	-15.39900
O	33.94700	9.80600	-12.27800
H	34.51700	10.23400	-12.91700
H	34.53700	9.52400	-11.58000
O	35.99100	6.38100	-18.40700
H	36.69400	6.83300	-17.94000
H	35.65900	5.73900	-17.77900
O	34.43900	4.97200	-4.03200
H	33.80900	5.65400	-3.79900
H	34.33300	4.86000	-4.97700
O	29.73400	4.63900	-13.10600
H	28.94400	4.89500	-13.58200
H	30.22500	5.45400	-13.00000
O	31.68500	5.00400	-16.14700
H	30.88800	5.11500	-15.62900
H	31.45800	5.34200	-17.01300
O	37.51600	11.52800	-8.19900
H	37.58000	11.99000	-7.36300
H	36.80700	10.89800	-8.07100
O	28.01700	4.89200	-10.67400
H	28.26800	5.77700	-10.40800
H	28.55300	4.71300	-11.44700
O	37.23000	13.19500	-18.33100
H	37.09500	14.04000	-17.90300
H	37.77100	13.39700	-19.09400
O	33.71700	-0.72600	-1.67000
H	33.32400	-1.27900	-0.99500
H	34.53400	-0.41500	-1.27900
O	30.68300	10.71200	-17.75800
H	31.19600	11.50700	-17.60800
H	31.33600	10.01500	-17.82500
O	32.49300	13.82900	-15.67600
H	32.67000	14.75100	-15.86400
H	33.28900	13.51500	-15.24700
O	36.63400	0.69000	-4.07800
H	37.37500	1.29000	-4.15700
H	36.86600	-0.05200	-4.63600
O	31.77800	5.87500	-5.80000
H	31.66400	4.97400	-5.49900
H	30.92300	6.12200	-6.15100
O	38.85700	13.81700	-4.20100
H	38.41400	13.03100	-4.51900
H	39.51900	14.00500	-4.86700
O	33.61600	15.34700	-9.67500
H	34.16700	15.21800	-10.44700
H	32.77700	15.64600	-10.02500
O	40.95500	14.09700	-6.17300
H	40.93700	14.91800	-6.66500
H	41.87700	13.97300	-5.94800
O	37.41300	11.61200	-5.33100
H	37.94600	10.85100	-5.56100
H	36.88400	11.31900	-4.58800
O	40.58000	-3.28200	-9.80100
H	40.23600	-2.39200	-9.87700
H	40.94800	-3.47200	-10.66400
O	28.64800	9.04100	-16.98200
H	29.48600	9.45100	-17.19900
H	28.82600	8.10100	-17.01200
O	40.54500	6.70900	-21.29400
H	40.02900	7.47900	-21.05500
H	40.27300	6.03500	-20.67100
O	38.12900	1.55400	-16.23500
H	37.27400	1.16400	-16.41500
H	38.54200	1.63300	-17.09500
O	39.65900	-0.91200	-11.83600
H	39.15600	-0.94900	-12.64900
H	39.95200	-0.00200	-11.77700
O	35.90400	-0.82800	-12.83000
H	36.76900	-1.02400	-13.18900
H	35.97100	-1.05700	-11.90300
O	40.33300	4.58100	-19.46800
H	40.02800	4.89600	-18.61700
H	41.28600	4.66000	-19.42500
O	38.77800	10.74000	-17.81900
H	38.25000	10.00600	-18.13100
H	38.27700	11.51800	-18.06500

O	33.51000	1.40200	-15.45800
H	34.08400	1.18000	-16.19100
H	34.09700	1.47100	-14.70500
O	33.83700	3.43300	-21.43800
H	33.00100	3.77000	-21.11800
H	33.69700	3.29800	-22.37600
O	35.81200	-1.72300	-16.73100
H	35.15900	-2.03900	-17.35500
H	35.77000	-2.34600	-16.00500
O	36.92300	8.55800	-24.81500
H	36.01600	8.32700	-24.61600
H	37.42000	7.76200	-24.62800
O	39.83000	17.26800	-21.89600
H	39.15600	17.57100	-21.28700
H	40.21700	18.07100	-22.24500
O	39.04600	4.76500	-12.40600
H	38.12800	4.98600	-12.56400
H	39.46200	4.83900	-13.26500
O	39.82900	6.16400	-17.27500
H	40.57700	6.67600	-17.58500
H	40.09900	5.84200	-16.41500
O	38.10200	8.66000	-20.29300
H	37.19300	8.37600	-20.19600
H	38.13200	9.08000	-21.15200
O	42.27700	3.20000	-15.29400
H	41.54000	2.68700	-14.96300
H	42.53000	2.76000	-16.10500
O	41.77700	14.10800	-15.58900
H	42.07000	14.66900	-16.30700
H	42.28700	14.40100	-14.83300
O	28.67100	6.38000	-16.31100
H	28.47300	5.78700	-17.03600
H	28.11600	6.07600	-15.59400
O	36.65100	6.27300	-14.19200
H	36.70500	5.32000	-14.26800
H	35.84000	6.42900	-13.70800
O	37.80000	10.20100	-22.95400
H	37.36200	9.48400	-23.41300
H	38.54100	10.42800	-23.51500
O	34.59000	4.89000	-16.43300
H	33.77700	5.19000	-16.02600
H	35.08600	4.49600	-15.71500
O	36.74100	14.88600	-14.49900
H	36.69800	15.11200	-15.42800
H	37.65700	14.65200	-14.35100
O	40.18700	5.35400	-7.60100
H	40.54200	5.58200	-6.74200
H	40.83400	5.68600	-8.22400
O	34.08100	2.46200	-10.81700
H	34.72100	2.32100	-11.51400
H	33.24900	2.17600	-11.19300
O	34.85700	11.76200	-14.70300
H	35.65100	11.26700	-14.90500
H	34.14600	11.23400	-15.06600
O	39.67700	14.14400	-10.35200
H	39.73000	14.71300	-9.58400
H	38.80800	14.31400	-10.71700
O	31.16600	5.90700	-18.62700
H	30.23500	5.84500	-18.41000
H	31.26100	5.38400	-19.42400
O	31.08400	2.43200	-16.03900
H	31.34500	3.34700	-16.14800
H	31.90600	1.96500	-15.89000
O	32.55700	3.84600	-8.86900
H	33.22900	3.63800	-9.51800
H	31.73000	3.64800	-9.30900
O	38.45900	0.47200	-6.85300
H	37.81200	1.17100	-6.94600
H	39.22900	0.90600	-6.48500
O	41.86700	10.73700	-3.77800
H	41.71600	10.50800	-4.69500
H	41.85800	11.69400	-3.76500
O	38.63600	8.31000	-8.79300
H	39.56500	8.46100	-8.96600
H	38.21500	8.39900	-9.64800
O	37.43400	12.33500	-11.02300
H	37.29600	11.96700	-10.15100
H	36.64600	12.85000	-11.19500
O	38.02900	17.43600	-19.68100
H	38.20400	16.96500	-18.86600
H	37.34300	18.06300	-19.45300
O	38.98500	11.71300	-13.63400
H	38.65000	11.67400	-12.73900
H	38.48500	11.04800	-14.10700
O	32.95900	7.12000	-8.10900
H	32.82100	6.38800	-7.50800
H	33.87000	7.03100	-8.39100
O	41.92100	9.05900	-23.36100
H	41.30400	8.33400	-23.45700
H	41.45300	9.82000	-23.70400
O	35.58800	6.83200	-8.62100
H	36.29800	6.19200	-8.66200
H	35.54500	7.08300	-7.69800
O	34.30000	3.81900	-6.80100
H	33.76400	3.36800	-6.14900
H	33.71200	3.95300	-7.54400
O	32.59500	20.58500	-12.90700
H	32.83500	21.21700	-12.22900
H	31.75200	20.23400	-12.62000
O	33.71100	3.27800	-24.23900
H	32.83400	3.15600	-24.60200
H	34.18600	2.48600	-24.49100
O	25.23100	8.06500	-14.14400
H	25.91800	7.75100	-14.73200
H	25.07600	8.96900	-14.41800
O	30.03400	2.95400	-9.71600
H	29.95000	2.34700	-10.45100
H	29.21300	3.44500	-9.71500
O	37.10800	16.39300	-10.50300
H	37.03500	17.19900	-9.99100
H	36.66600	16.59200	-11.32800
O	36.89700	15.63000	-16.99200
H	36.11400	16.17200	-16.89900
H	37.59600	16.14800	-16.59300
O	40.20700	1.44000	-14.34900
H	40.70400	0.68700	-14.66900
H	39.43500	1.47600	-14.91500
O	39.56800	16.29200	-8.34700

H	40.28100	16.92600	-8.41900
H	39.07700	16.38800	-9.16300
O	41.29000	10.19700	-16.77500
H	40.35500	10.31700	-16.94500
H	41.46700	9.29400	-17.03900
O	36.79000	3.58100	-14.62200
H	36.42000	3.02900	-13.93400
H	37.28600	2.97700	-15.17400
O	34.61900	14.04100	-7.52600
H	35.32000	14.67500	-7.37300
H	34.14900	14.38300	-8.28700
O	42.74500	12.38300	-19.24200
H	43.02100	12.26900	-18.33200
H	41.87500	11.98500	-19.28000
O	42.41900	6.59600	-9.70100
H	41.76600	6.84300	-10.35600
H	42.79700	5.78200	-10.03500
O	39.38000	16.63200	-15.59800
H	39.56200	15.81400	-15.13500
H	40.20300	16.84800	-16.03600
O	39.71400	15.55800	-18.33700
H	40.24300	15.04800	-18.95000
H	39.97700	15.24300	-17.47200
O	38.46800	7.33800	-3.21400
H	37.79200	7.23700	-2.54400
H	39.29100	7.18200	-2.75100
O	30.21600	13.57500	-10.59300
H	30.80100	12.82900	-10.46000
H	30.01800	13.88500	-9.71000
O	33.07000	16.61000	-15.57700
H	32.40100	17.09500	-16.06100
H	32.93100	16.85700	-14.66300
O	24.80800	6.14500	-11.87300
H	24.84600	5.61200	-11.07900
H	24.30200	5.61900	-12.49200
O	37.21000	18.64400	-8.97900
H	37.76200	18.80700	-8.21400
H	37.55100	19.23500	-9.65100
O	32.18700	0.99200	-8.37600
H	32.25100	1.85300	-8.78900
H	32.12400	0.37900	-9.10900
O	31.66200	3.06400	-4.72900
H	32.28900	2.68200	-4.11500
H	30.80500	2.84600	-4.36400
O	42.06100	13.47000	-3.12400
H	42.60500	14.08300	-3.61800
H	41.48000	14.02800	-2.60700
O	28.43900	4.87200	-18.79700
H	28.73400	3.96300	-18.85100
H	27.53700	4.85100	-19.11900
O	40.34700	16.59300	-11.99200
H	40.22200	15.79100	-12.50100
H	41.00500	17.08700	-12.48100
O	31.96800	2.94700	-12.81500
H	31.24700	3.56100	-12.95400
H	32.75700	3.48300	-12.89300
O	42.41100	14.39300	-10.36400
H	41.46400	14.27400	-10.43700
H	42.75900	13.50600	-10.28200
O	34.77100	0.28300	-6.54100
H	35.29400	0.52900	-5.77800
H	34.55200	1.11500	-6.96000
O	30.16700	2.55300	-18.67300
H	29.83900	1.67300	-18.85600
H	30.52100	2.49800	-17.78600
O	39.41500	14.18900	-14.16400
H	39.26800	13.26000	-13.98300
H	40.24100	14.21100	-14.64800
O	33.28200	2.84000	-18.30600
H	33.62100	3.36900	-19.02800
H	33.72900	3.17900	-17.53000
O	35.97600	1.22400	-8.85600
H	35.63600	0.42500	-8.45300
H	35.27000	1.52400	-9.42800
O	36.62800	-1.69100	-10.40300
H	37.45000	-2.16500	-10.52500
H	36.87100	-0.91200	-9.90300
O	35.26800	1.33800	-20.10700
H	35.98900	1.71600	-20.61000
H	34.64500	2.05700	-20.00300
O	39.41100	2.04200	-19.17100
H	39.62800	2.92500	-19.47200
H	38.93100	1.65100	-19.90000
O	34.15100	5.00100	-12.59400
H	34.00600	5.31500	-11.70100
H	33.67400	5.62000	-13.14700
O	36.01100	1.99100	-12.54600
H	35.99700	1.09500	-12.88200
H	36.72800	1.99800	-11.91200
O	37.86400	2.42500	-10.50000
H	37.60500	3.34700	-10.50200
H	37.33200	2.02800	-9.81000
O	28.62600	7.54200	-9.65500
H	28.08100	8.31800	-9.52300
H	29.50400	7.88900	-9.81300
O	35.41500	0.87300	-17.27700
H	35.53600	-0.05600	-17.07800
H	35.12500	0.88700	-18.18900
O	38.28400	-1.44200	-14.14000
H	38.30600	-1.37800	-15.09500
H	38.70100	-2.28100	-13.94600
O	29.41300	6.66900	-6.76200
H	28.67100	6.81700	-6.17500
H	29.09200	6.92900	-7.62500
O	37.35700	16.21000	-6.96400
H	37.66600	15.96500	-6.09200
H	38.15400	16.41500	-7.45200
O	37.37900	18.36400	-14.73300
H	36.93900	17.87200	-14.04000
H	38.13000	17.82100	-14.97400
O	29.85800	14.55500	-14.73100
H	30.68400	14.25500	-15.11100
H	29.50800	13.78600	-14.28200
O	34.82600	13.90800	-12.66100
H	34.20400	13.24500	-12.96100
H	35.49700	13.93400	-13.34400
O	28.05900	12.47400	-13.77200
H	27.22200	12.04600	-13.95400

H	28.03100	12.66700	-12.83500
O	34.89900	2.38600	-3.06800
H	34.94800	3.26200	-3.45000
H	35.44100	1.84400	-3.64100
O	34.38800	12.45800	-19.18500
H	34.53700	13.14200	-19.83900
H	35.19400	12.43900	-18.67000
O	37.12900	3.13900	-6.31300
H	36.25900	3.43200	-6.58500
H	37.36500	3.72300	-5.59200
O	32.14300	8.48500	-18.17900
H	31.89800	7.58100	-17.98100
H	32.31300	8.48600	-19.12100
O	39.83300	-0.64300	-8.79700
H	40.35100	0.09300	-9.12200
H	39.47800	-0.33700	-7.96200
O	31.41300	4.21700	-20.68000
H	30.71900	4.29200	-21.33600
H	31.16800	3.45400	-20.15800
O	40.08400	-4.22300	-13.27700
H	40.36400	-4.89100	-12.65000
H	40.86900	-4.03000	-13.78900
O	37.88700	14.95300	-21.71400
H	37.31500	15.57200	-22.16700
H	38.46100	15.50300	-21.18000
O	41.97600	1.28400	-9.34300
H	41.38100	1.56500	-10.03900
H	41.69100	1.77400	-8.57200
O	41.10700	15.23700	-20.58300
H	42.04000	15.17100	-20.38000
H	41.01900	16.07200	-21.04200
O	38.27200	6.28900	-24.37200
H	39.21800	6.16000	-24.30400
H	37.89300	5.55000	-23.89500
O	38.15600	10.34100	-2.91200
H	39.07600	10.51900	-2.71800
H	38.12100	9.39600	-3.06400
O	36.45600	4.21400	-24.43700
H	35.54900	3.91700	-24.36100
H	36.73400	3.91700	-25.30400
O	27.37400	5.53500	-13.98700
H	27.05400	4.68100	-14.28000
H	26.82700	5.75000	-13.23100
O	31.15200	8.25100	-10.27000
H	31.37100	9.18300	-10.25100
H	31.56500	7.89200	-9.48500
O	38.07200	-1.24200	-18.28300
H	37.26300	-1.58800	-17.90700
H	38.76100	-1.55200	-17.69400
O	40.00200	-2.16400	-16.66000
H	40.55500	-1.74500	-16.00100
H	40.23900	-3.09100	-16.61800
O	36.02500	3.82400	-0.88000
H	35.92400	4.77400	-0.94900
H	35.80700	3.49600	-1.75200
O	35.22600	1.07300	-24.29600
H	36.08800	1.39800	-24.03600
H	35.41100	0.28600	-24.80800
O	27.24300	9.15500	-12.01800
H	26.54900	8.74000	-12.53100
H	27.99000	9.19700	-12.61400
O	41.83000	-0.65400	-14.95000
H	42.33000	-0.54200	-15.75900
H	42.47300	-0.95800	-14.31000
O	35.88800	7.25400	-2.06300
H	35.40500	8.05400	-1.85700
H	35.97400	7.26500	-3.01700
O	29.15700	9.08700	-14.14100
H	29.13400	9.18600	-15.09300
H	29.46700	9.93300	-13.81900
O	31.81500	11.14400	-10.02600
H	32.77000	11.11300	-10.09300
H	31.61700	10.68300	-9.21100
O	32.40500	9.71500	-7.45900
H	33.22500	10.20900	-7.43600
H	32.67900	8.79800	-7.48800
O	42.16200	2.10600	-18.09700
H	41.27400	2.39100	-18.31500
H	42.57700	1.94500	-18.94400
O	30.17000	16.68600	-13.00900
H	30.03600	15.92600	-13.57600
H	29.75400	17.41000	-13.47700
O	41.30600	11.87000	-24.00200
H	42.01000	12.07500	-23.38600
H	40.58200	12.43800	-23.73700
O	42.13600	7.62100	-17.88200
H	42.07900	8.07800	-18.72100
H	42.68900	6.86000	-18.05900
O	33.32700	17.58300	-12.79800
H	33.21000	18.52800	-12.90000
H	32.50600	17.28100	-12.40900
O	42.86900	8.81200	-20.58600
H	42.81400	8.64600	-21.52700
H	42.24400	9.51900	-20.43000
O	27.65900	4.04800	-6.76000
H	28.30500	3.53800	-6.27100
H	27.45000	4.78500	-6.18600
O	40.18400	11.11600	-1.26100
H	41.05300	11.29000	-1.62200
H	39.70500	11.93600	-1.38300
O	36.17900	-1.68800	-21.00800
H	36.16700	-0.74300	-20.85100
H	37.07100	-1.87100	-21.30200
O	34.35300	18.23600	-9.58200
H	35.25500	18.26800	-9.26500
H	34.12600	17.30600	-9.56700
O	36.10800	16.98900	-12.92100
H	35.15800	16.98900	-13.03500
H	36.41100	16.24200	-13.43700
O	39.99400	-5.09300	-16.75300
H	39.22800	-5.64800	-16.60800
H	40.40500	-5.02100	-15.89200
O	31.99800	1.19500	-2.66300
H	32.86200	0.83900	-2.45200
H	31.42500	0.85600	-1.97500
O	42.05700	26.12700	-13.09200
H	42.85000	25.78600	-13.50800
H	42.29700	26.23600	-12.17200

O	41.62600	22.49600	-13.15100
H	41.75500	22.75000	-12.23700
H	40.94600	23.08900	-13.47000
O	40.57300	19.96800	-13.10600
H	39.78300	20.22400	-13.58200
H	41.06400	20.78300	-13.00000
O	42.52500	20.33300	-16.14700
H	41.72800	20.44500	-15.62900
H	42.29700	20.67100	-17.01300
O	38.85700	20.22100	-10.67400
H	39.10800	21.10600	-10.40800
H	39.39300	20.04200	-11.44700
O	41.52300	26.04200	-17.75800
H	42.03500	26.83600	-17.60800
H	42.17500	25.34400	-17.82500
O	42.61800	21.20500	-5.80000
H	42.50300	20.30300	-5.49900
H	41.76200	21.45100	-6.15100
O	39.48800	24.37000	-16.98200
H	40.32500	24.78000	-17.19900
H	39.66600	23.43000	-17.01200
O	39.51000	21.70900	-16.31100
H	39.31300	21.11600	-17.03600
H	38.95500	21.40500	-15.59400
O	42.00500	21.23600	-18.62700
H	41.07500	21.17400	-18.41000
H	42.10100	20.71300	-19.42400
O	41.92300	17.76100	-16.03900
H	42.18400	18.67600	-16.14800
H	42.74500	17.29400	-15.89000
O	36.07000	23.39400	-14.14400
H	36.75700	23.08100	-14.73200
H	35.91500	24.29900	-14.41800
O	40.87400	18.28400	-9.71600
H	40.79000	17.67600	-10.45100
H	40.05200	18.77400	-9.71500
O	41.05500	28.90400	-10.59300
H	41.64100	28.15800	-10.46000
H	40.85800	29.21400	-9.71000
O	35.64700	21.47500	-11.87300
H	35.68600	20.94100	-11.07900
H	35.14100	20.94800	-12.49200
O	39.27800	20.20100	-18.79700
H	39.57300	19.29200	-18.85100
H	38.37700	20.18000	-19.11900
O	41.00700	17.88200	-18.67300
H	40.67800	17.00200	-18.85600
H	41.36100	17.82700	-17.78600
O	39.46500	22.87100	-9.65500
H	38.92100	23.64700	-9.52300
H	40.34300	23.21800	-9.81300
O	40.25200	21.99800	-6.76200
H	39.51000	22.14600	-6.17500
H	39.93100	22.25900	-7.62500
O	40.69800	29.88400	-14.73100
H	41.52300	29.58500	-15.11100
H	40.34700	29.11500	-14.28200
O	38.89800	27.80300	-13.77200
H	38.06200	27.37500	-13.95400
H	38.87100	27.99600	-12.83500
O	42.25200	19.54700	-20.68000
H	41.55900	19.62100	-21.33600
H	42.00700	18.78300	-20.15800
O	38.21300	20.86400	-13.98700
H	37.89400	20.01000	-14.28000
H	37.66700	21.07900	-13.23100
O	41.99100	23.58000	-10.27000
H	42.21000	24.51200	-10.25100
H	42.40400	23.22100	-9.48500
O	38.08200	24.48400	-12.01800
H	37.38900	24.07000	-12.53100
H	38.83000	24.52600	-12.61400
O	39.99600	24.41600	-14.14100
H	39.97300	24.51500	-15.09300
H	40.30600	25.26200	-13.81900
O	38.49800	19.37800	-6.76000
H	39.14400	18.86700	-6.27100
H	38.29000	20.11400	-6.18600
O	13.27800	-35.81600	-30.32700
H	13.20000	-34.86500	-30.24800
H	12.67400	-36.16100	-29.67000
O	10.81400	-39.82300	-28.95100
H	10.99100	-38.90100	-29.13900
H	11.61000	-40.13700	-28.52200
O	11.93700	-38.30500	-22.95000
H	12.48800	-38.43400	-23.72300
H	11.09800	-38.00600	-23.30100
O	15.06200	-38.76600	-27.77500
H	15.02000	-38.54000	-28.70400
H	15.97800	-39.00000	-27.62600
O	16.35000	-36.21600	-32.95600
H	16.52600	-36.68700	-32.14200
H	15.66400	-35.58900	-32.72800
O	10.91700	-33.06700	-26.18200
H	11.15600	-32.43500	-25.50400
H	10.07400	-33.41800	-25.89600
O	15.42900	-37.25900	-23.77800
H	15.35600	-36.45400	-23.26600
H	14.98700	-37.06000	-24.60300
O	15.21800	-38.02200	-30.26800
H	14.43500	-37.48000	-30.17400
H	15.91800	-37.50500	-29.86800
O	17.88900	-37.36000	-21.62200
H	18.60200	-36.72600	-21.69500
H	17.39800	-37.26400	-22.43800
O	12.94000	-39.61100	-20.80200
H	13.64100	-38.97700	-20.64900
H	12.47000	-39.27000	-21.56200
O	17.70100	-37.02100	-28.87400
H	17.88400	-37.83800	-28.41100
H	18.52500	-36.80400	-29.31100
O	18.03600	-38.09400	-31.61200
H	18.56400	-38.60500	-32.22500
H	18.29800	-38.40900	-30.74800
O	8.53700	-40.07700	-23.86900
H	9.12300	-40.82300	-23.73600
H	8.34000	-39.76700	-22.98500
O	11.39100	-37.04200	-28.85200

H	10.72200	-36.55800	-29.33600
H	11.25200	-36.79500	-27.93800
O	15.53200	-35.00800	-22.25400
H	16.08300	-34.84500	-21.48900
H	15.87200	-34.41700	-22.92600
O	18.66800	-37.06000	-25.26800
H	18.54400	-37.86100	-25.77700
H	19.32600	-36.56500	-25.75600
O	15.67800	-37.44200	-20.23900
H	15.98700	-37.68700	-19.36700
H	16.47600	-37.23700	-20.72700
O	15.70000	-35.28800	-28.00900
H	15.26100	-35.78100	-27.31500
H	16.45100	-35.83100	-28.25000
O	8.17900	-39.09700	-28.00600
H	9.00500	-39.39700	-28.38600
H	7.82900	-39.86600	-27.55700
O	13.14800	-39.74400	-25.93700
H	12.52600	-40.40700	-26.23600
H	13.81800	-39.71800	-26.61900
O	6.38000	-41.17800	-27.04700
H	5.54400	-41.60600	-27.22900
H	6.35300	-40.98500	-26.11000
O	8.49200	-36.96600	-26.28500
H	8.35700	-37.72600	-26.85100
H	8.07500	-36.24200	-26.75200
O	11.64800	-36.06900	-26.07300
H	11.53200	-35.12400	-26.17500
H	10.82800	-36.37200	-25.68400
O	12.67400	-35.41600	-22.85700
H	13.57700	-35.38400	-22.54100
H	12.44800	-36.34600	-22.84300
O	14.42900	-36.66300	-26.19600
H	13.47900	-36.66300	-26.31000
H	14.73300	-37.41000	-26.71300
O	30.16500	-30.82700	-25.13200
H	30.80200	-30.94600	-25.83600
H	30.04700	-29.87900	-25.07300
O	26.97400	-30.87400	-25.19600
H	26.63500	-30.99000	-26.08400
H	27.84200	-31.27500	-25.21600
O	22.83200	-29.02100	-29.20600
H	22.26900	-29.11200	-29.97400
H	22.71700	-29.84100	-28.72600
O	27.45500	-29.72100	-19.23700
H	27.44400	-30.11500	-20.10900
H	27.61800	-30.45400	-18.64400
O	20.37900	-27.52500	-26.36800
H	21.17100	-27.86600	-26.78300
H	20.61800	-27.41600	-25.44700
O	22.33800	-28.26500	-33.49600
H	23.11100	-28.81200	-33.63500
H	22.69000	-27.40000	-33.28400
O	24.17600	-26.93700	-20.28900
H	23.88500	-26.03200	-20.40500
H	24.81200	-26.89500	-19.57500
O	25.10200	-29.55900	-23.69900
H	25.69800	-29.78400	-24.41300
H	25.15900	-30.30100	-23.09700
O	29.13500	-27.25100	-23.29400
H	28.83500	-26.34800	-23.40300
H	28.79000	-27.51800	-22.44200
O	22.58600	-30.79900	-27.26100
H	21.66200	-30.93200	-27.05300
H	22.83500	-30.02000	-26.76400
O	19.94700	-31.15600	-26.42600
H	20.07700	-30.90300	-25.51200
H	19.26700	-30.56300	-26.74600
O	24.11700	-20.48600	-30.32700
H	24.04000	-19.53600	-30.24800
H	23.51300	-20.83200	-29.67000
O	26.60900	-33.31400	-17.67100
H	25.83200	-32.75400	-17.68100
H	27.24800	-32.82800	-17.14900
O	24.86400	-30.50500	-18.10900
H	25.60500	-29.96700	-18.38600
H	24.15700	-29.87800	-17.95200
O	30.33900	-28.36200	-19.51200
H	30.74800	-28.80400	-20.25700
H	29.61700	-28.93700	-19.25900
O	26.69200	-33.42500	-22.13900
H	27.53800	-33.00100	-21.99500
H	26.63200	-34.08200	-21.44500
O	30.66200	-27.80200	-25.75700
H	30.03300	-27.63700	-26.45900
H	30.27500	-27.38800	-24.98600
O	31.79600	-29.22100	-21.80300
H	32.00800	-28.57700	-22.47900
H	31.74900	-30.05400	-22.27100
O	26.21500	-28.06500	-28.49200
H	26.67900	-27.78600	-29.28100
H	25.95100	-28.96600	-28.67400
O	23.10700	-28.51700	-25.55400
H	23.67700	-28.08900	-26.19200
H	23.69800	-28.79900	-24.85500
O	23.60000	-33.35100	-17.30800
H	22.97000	-32.66900	-17.07400
H	23.49300	-33.46300	-18.25200
O	18.89400	-33.68400	-26.38100
H	18.10400	-33.42800	-26.85700
H	19.38500	-32.86900	-26.27600
O	32.91100	-26.34300	-23.63100
H	33.73200	-26.74500	-23.91200
H	32.90700	-26.44700	-22.67900
O	20.84600	-33.31900	-29.42300
H	20.04900	-33.20800	-28.90400
H	20.61800	-32.98100	-30.28900
O	26.67600	-26.79500	-21.47400
H	26.74100	-26.33300	-20.63900
H	25.96700	-27.42500	-21.34600
O	17.17800	-33.43100	-23.95000
H	17.42900	-32.54600	-23.68400
H	17.71400	-33.61000	-24.72200
O	26.39100	-25.12800	-31.60700
H	26.25600	-24.28300	-31.17800
H	26.93200	-24.92600	-32.37000
O	19.84400	-27.61100	-31.03400
H	20.35700	-26.81600	-30.88300

H	20.49600	-28.30800	-31.10000
O	21.65400	-24.49400	-28.95100
H	21.83000	-23.57200	-29.13900
H	22.44900	-24.80800	-28.52200
O	20.93900	-32.44800	-19.07500
H	20.82400	-33.34900	-18.77500
H	20.08300	-32.20100	-19.42700
O	28.01800	-24.50600	-17.47700
H	27.57400	-25.29200	-17.79400
H	28.68000	-24.31800	-18.14200
O	22.77700	-22.97600	-22.95000
H	23.32700	-23.10500	-23.72300
H	21.93800	-22.67700	-23.30100
O	30.11600	-24.22600	-19.44800
H	30.09800	-23.40500	-19.94000
H	31.03800	-24.35000	-19.22300
O	26.57300	-26.71100	-18.60600
H	27.10600	-27.47200	-18.83600
H	26.04500	-27.00400	-17.86400
O	17.80900	-29.28200	-30.25800
H	18.64600	-28.87200	-30.47400
H	17.98700	-30.22200	-30.28800
O	27.93900	-27.58300	-31.09400
H	27.41100	-28.31700	-31.40700
H	27.43700	-26.80500	-31.34000
O	32.91800	-29.38800	-25.97700
H	32.26000	-28.69800	-25.88900
H	33.72200	-28.99800	-25.63400
O	22.67100	-36.92100	-28.73300
H	23.24500	-37.14300	-29.46600
H	23.25800	-36.85200	-27.98100
O	28.20700	-33.55800	-25.68100
H	27.28900	-33.33700	-25.83900
H	28.62300	-33.48400	-26.54000
O	30.93800	-24.21500	-28.86400
H	31.23000	-23.65400	-29.58300
H	31.44800	-23.92200	-28.10900
O	17.83200	-31.94300	-29.58700
H	17.63400	-32.53600	-30.31200
H	17.27600	-32.24700	-28.86900
O	25.81100	-32.05000	-27.46700
H	25.86600	-33.00300	-27.54300
H	25.00000	-31.89400	-26.98300
O	23.75000	-33.43300	-29.70800
H	22.93700	-33.13300	-29.30200
H	24.24600	-33.82700	-28.99100
O	25.90100	-23.43700	-27.77500
H	25.85900	-23.21100	-28.70400
H	26.81800	-23.67100	-27.62600
O	29.34800	-32.96800	-20.87700
H	29.70300	-32.74100	-20.01700
H	29.99500	-32.63700	-21.49900
O	23.24200	-35.86100	-24.09200
H	23.88200	-36.00200	-24.79000
H	22.41000	-36.14700	-24.46800
O	24.01800	-26.56100	-27.97900
H	24.81200	-27.05600	-28.18000
H	23.30700	-27.08900	-28.34200
O	28.83700	-24.17900	-23.62700
H	28.89100	-23.61000	-22.86000
H	27.96900	-24.00900	-23.99200
O	20.32600	-32.41600	-31.90300
H	19.39600	-32.47800	-31.68600
H	20.42200	-32.93900	-32.69900
O	20.24500	-35.89100	-29.31500
H	20.50500	-34.97600	-29.42400
H	21.06600	-36.35800	-29.16500
O	21.71700	-34.47700	-22.14500
H	22.39000	-34.68500	-22.79400
H	20.89000	-34.67500	-22.58400
O	35.96600	-27.73700	-22.62700
H	36.16000	-26.83200	-22.38100
H	35.86600	-28.19600	-21.79400
O	31.02800	-27.58600	-17.05300
H	30.87600	-27.81500	-17.97000
H	31.01800	-26.62900	-17.04000
O	27.79600	-30.01300	-22.06900
H	28.72500	-29.86200	-22.24200
H	27.37500	-29.92400	-22.92400
O	26.59500	-25.98800	-24.29900
H	26.45600	-26.35600	-23.42600
H	25.80700	-25.47300	-24.47000
O	27.19000	-20.88700	-32.95600
H	27.36500	-21.35800	-32.14200
H	26.50300	-20.26000	-32.72800
O	28.14600	-26.61000	-26.91000
H	27.81100	-26.64900	-26.01400
H	27.64600	-27.27500	-27.38300
O	22.12000	-31.20300	-21.38500
H	21.98200	-31.93500	-20.78400
H	23.03000	-31.29200	-21.66600
O	24.74800	-31.49100	-21.89600
H	25.45800	-32.13100	-21.93800
H	24.70500	-31.23900	-20.97400
O	32.73700	-24.55400	-18.97600
H	32.67800	-25.23800	-19.64300
H	33.13900	-23.81200	-19.42800
O	23.46100	-34.50400	-20.07700
H	22.92500	-34.95500	-19.42500
H	22.87300	-34.37000	-20.82000
O	21.75600	-17.73800	-26.18200
H	21.99600	-17.10600	-25.50400
H	20.91300	-18.08900	-25.89600
O	32.73900	-26.59000	-20.74400
H	31.85100	-26.92700	-20.62200
H	33.30800	-27.32200	-20.50700
O	14.39200	-30.25800	-27.42000
H	15.07900	-30.57200	-28.00800
H	14.23600	-29.35400	-27.69300
O	19.19500	-35.36900	-22.99100
H	19.11100	-35.97600	-23.72600
H	18.37300	-34.87800	-22.99100
O	26.26900	-21.93000	-23.77800
H	26.19600	-21.12400	-23.26600
H	25.82600	-21.73100	-24.60300
O	26.05800	-22.69300	-30.26800
H	25.27400	-22.15100	-30.17400
H	26.75700	-22.17500	-29.86800

O	28.72900	-22.03100	-21.62200
H	29.44100	-21.39700	-21.69500
H	28.23800	-21.93500	-22.43800
O	23.78000	-24.28200	-20.80200
H	24.48100	-23.64800	-20.64900
H	23.30900	-23.94000	-21.56200
O	31.58000	-31.72700	-22.97700
H	30.92700	-31.48000	-23.63100
H	31.95800	-32.54100	-23.31000
O	28.54100	-21.69100	-28.87400
H	28.72300	-22.50900	-28.41100
H	29.36400	-21.47500	-29.31100
O	28.87500	-22.76500	-31.61200
H	29.40400	-23.27500	-32.22500
H	29.13800	-23.08000	-30.74800
O	27.62900	-30.98500	-16.49000
H	26.95300	-31.08600	-15.82000
H	28.45100	-31.14100	-16.02600
O	19.37700	-24.74800	-23.86900
H	19.96200	-25.49400	-23.73600
H	19.17900	-24.43800	-22.98500
O	22.23000	-21.71300	-28.85200
H	21.56100	-21.22800	-29.33600
H	22.09200	-21.46600	-27.93800
O	13.96800	-32.17800	-25.14800
H	14.00700	-32.71100	-24.35500
H	13.46200	-32.70400	-25.76700
O	26.37100	-19.67900	-22.25400
H	26.92300	-19.51600	-21.48900
H	26.71200	-19.08800	-22.92600
O	21.34700	-37.33100	-21.65200
H	21.41200	-36.47000	-22.06400
H	21.28400	-37.94400	-22.38500
O	20.82300	-35.25900	-18.00500
H	21.45000	-35.64100	-17.39100
H	19.96500	-35.47700	-17.63900
O	31.22200	-24.85300	-16.39900
H	31.76600	-24.24000	-16.89400
H	30.64100	-24.29500	-15.88300
O	17.59900	-33.45100	-32.07300
H	17.89500	-34.36000	-32.12600
H	16.69800	-33.47200	-32.39500
O	29.50800	-21.73000	-25.26800
H	29.38300	-22.53200	-25.77700
H	30.16600	-21.23600	-25.75600
O	21.12900	-35.37600	-26.09000
H	20.40700	-34.76200	-26.22900
H	21.91800	-34.84000	-26.16800
O	31.57100	-23.93000	-23.64000
H	30.62400	-24.04900	-23.71200
H	31.92000	-24.81700	-23.55700
O	19.32800	-35.77000	-31.94900
H	18.99900	-36.65000	-32.13100
H	19.68200	-35.82500	-31.06100
O	28.57600	-24.13400	-27.43900
H	28.42800	-25.06300	-27.25900
H	29.40100	-24.11200	-27.92400
O	32.17900	-23.31100	-26.35300
H	33.10400	-23.51700	-26.49000
H	32.01100	-23.56000	-25.44400
O	23.31200	-33.32200	-25.86900
H	23.16600	-33.00800	-24.97700
H	22.83500	-32.70300	-26.42200
O	17.78700	-30.78100	-22.93000
H	17.24200	-30.00500	-22.79800
H	18.66500	-30.43400	-23.08800
O	18.57300	-31.65400	-20.03700
H	17.83100	-31.50600	-19.45100
H	18.25200	-31.39400	-20.90100
O	26.51800	-22.11300	-20.23900
H	26.82600	-22.35800	-19.36700
H	27.31500	-21.90800	-20.72700
O	26.54000	-19.95900	-28.00900
H	26.10000	-20.45100	-27.31500
H	27.29000	-20.50200	-28.25000
O	19.01900	-23.76800	-28.00600
H	19.84500	-24.06800	-28.38600
H	18.66800	-24.53700	-27.55700
O	23.98700	-24.41500	-25.93700
H	23.36500	-25.07800	-26.23600
H	24.65700	-24.38900	-26.61900
O	17.22000	-25.84900	-27.04700
H	16.38300	-26.27700	-27.22900
H	17.19200	-25.65600	-26.11000
O	24.05900	-35.93700	-16.34300
H	24.10800	-35.06100	-16.72500
H	24.60200	-36.47900	-16.91600
O	34.54500	-25.39300	-17.06300
H	33.75500	-25.41100	-17.60300
H	34.30800	-24.85400	-16.30800
O	34.66500	-28.54400	-19.75000
H	34.71500	-28.70900	-18.80800
H	34.55600	-29.41100	-20.14000
O	23.54800	-25.86500	-32.46100
H	23.69700	-25.18100	-33.11400
H	24.35500	-25.88400	-31.94600
O	31.95500	-32.56100	-18.43400
H	32.85600	-32.70000	-18.72600
H	32.03000	-32.39500	-17.49400
O	26.28900	-35.18400	-19.58900
H	25.42000	-34.89100	-19.86000
H	26.52500	-34.60000	-18.86800
O	36.16700	-26.12400	-19.50600
H	35.87500	-25.76100	-18.66900
H	35.55400	-26.83700	-19.68400
O	34.41500	-31.00100	-21.03800
H	34.89100	-30.80800	-21.84600
H	33.49200	-30.94500	-21.28300
O	21.30400	-29.83800	-31.45500
H	21.05800	-30.74200	-31.25700
H	21.47300	-29.83700	-32.39700
O	27.31600	-27.98200	-16.18800
H	28.23700	-27.80400	-15.99400
H	27.28100	-28.92700	-16.34000
O	16.53500	-32.78800	-27.26200
H	16.21500	-33.64200	-27.55500
H	15.98800	-32.57300	-26.50700
O	20.31300	-30.07200	-23.54600

H	20.53100	-29.14000	-23.52700
H	20.72500	-30.43100	-22.76000
O	25.18500	-34.49900	-14.15500
H	25.08500	-33.54900	-14.22400
H	24.96800	-34.82700	-15.02800
O	16.40300	-29.16800	-25.29300
H	15.71000	-29.58300	-25.80600
H	17.15100	-29.12600	-25.89000
O	25.04900	-31.06900	-15.33900
H	24.56500	-30.26900	-15.13200
H	25.13500	-31.05800	-16.29200
O	18.31700	-29.23600	-27.41700
H	18.29400	-29.13700	-28.36800
H	18.62700	-28.39000	-27.09500
O	20.97600	-27.17900	-23.30200
H	21.93000	-27.21000	-23.36900
H	20.77700	-27.64000	-22.48700
O	21.56600	-28.60800	-20.73500
H	22.38500	-28.11400	-20.71100
H	21.84000	-29.52500	-20.76300
O	19.33100	-21.63700	-26.28500
H	19.19600	-22.39700	-26.85100
H	18.91400	-20.91300	-26.75200
O	22.48800	-20.74000	-26.07300
H	22.37100	-19.79500	-26.17500
H	21.66700	-21.04200	-25.68400
O	16.81900	-34.27500	-20.03600
H	17.46500	-34.78500	-19.54700
H	16.61100	-33.53800	-19.46100
O	29.34500	-27.20700	-14.53600
H	30.21400	-27.03300	-14.89800
H	28.86500	-26.38700	-14.65900
O	23.51300	-20.08700	-22.85700
H	24.41600	-20.05500	-22.54100
H	23.28700	-21.01700	-22.84300
O	25.26900	-21.33400	-26.19600
H	24.31800	-21.33400	-26.31000
H	25.57200	-22.08000	-26.71300
O	21.15900	-37.12800	-15.93800
H	22.02200	-37.48400	-15.72800
H	20.58600	-37.46700	-15.25100
O	41.00400	-15.49800	-25.13200
H	41.64200	-15.61700	-25.83600
H	40.88600	-14.55000	-25.07300
O	37.81300	-15.54500	-25.19600
H	37.47500	-15.66100	-26.08400
H	38.68200	-15.94600	-25.21600
O	33.67200	-13.69100	-29.20600
H	33.10800	-13.78200	-29.97400
H	33.55700	-14.51100	-28.72600
O	38.29500	-14.39100	-19.23700
H	38.28400	-14.78600	-20.10900
H	38.45700	-15.12500	-18.64400
O	31.21800	-12.19600	-26.36800
H	32.01000	-12.53700	-26.78300
H	31.45800	-12.08700	-25.44700
O	33.17700	-12.93500	-33.49600
H	33.95000	-13.48300	-33.63500
H	33.53000	-12.07100	-33.28400
O	35.01600	-11.60800	-20.28900
H	34.72500	-10.70300	-20.40500
H	35.65100	-11.56600	-19.57500
O	35.94100	-14.23000	-23.69900
H	36.53800	-14.45400	-24.41300
H	35.99900	-14.97200	-23.09700
O	39.97500	-11.92200	-23.29400
H	39.67500	-11.01900	-23.40300
H	39.63000	-12.18800	-22.44200
O	33.42600	-15.47000	-27.26100
H	32.50100	-15.60300	-27.05300
H	33.67400	-14.69100	-26.76400
O	30.78700	-15.82700	-26.42600
H	30.91600	-15.57300	-25.51200
H	30.10600	-15.23400	-26.74600
O	34.95700	-5.15700	-30.32700
H	34.87900	-4.20600	-30.24800
H	34.35300	-5.50300	-29.67000
O	37.44800	-17.98400	-17.67100
H	36.67200	-17.42500	-17.68100
H	38.08700	-17.49900	-17.14900
O	35.70300	-15.17600	-18.10900
H	36.44400	-14.63700	-18.38600
H	34.99700	-14.54900	-17.95200
O	41.17900	-13.03300	-19.51200
H	41.58700	-13.47500	-20.25700
H	40.45600	-13.60800	-19.25900
O	37.53200	-18.09600	-22.13900
H	38.37800	-17.67200	-21.99500
H	37.47200	-18.75200	-21.44500
O	41.50100	-12.47300	-25.75700
H	40.87200	-12.30800	-26.45900
H	41.11400	-12.05900	-24.98600
O	40.68200	-20.43500	-20.48000
H	40.43800	-19.68100	-21.01700
H	40.95800	-20.05700	-19.64600
O	42.63500	-13.89100	-21.80300
H	42.84700	-13.24800	-22.47900
H	42.58800	-14.72500	-22.27100
O	37.05400	-12.73500	-28.49200
H	37.51800	-12.45700	-29.28100
H	36.79000	-13.63700	-28.67400
O	33.94700	-13.18800	-25.55400
H	34.51700	-12.76000	-26.19200
H	34.53700	-13.47000	-24.85500
O	34.43900	-18.02200	-17.30800
H	33.80900	-17.34000	-17.07400
H	34.33300	-18.13400	-18.25200
O	29.73400	-18.35500	-26.38100
H	28.94400	-18.09900	-26.85700
H	30.22500	-17.54000	-26.27600
O	31.68500	-17.99000	-29.42300
H	30.88800	-17.87800	-28.90400
H	31.45800	-17.65200	-30.28900
O	37.51600	-11.46600	-21.47400
H	37.58000	-11.00400	-20.63900
H	36.80700	-12.09600	-21.34600
O	28.01700	-18.10200	-23.95000
H	28.26800	-17.21700	-23.68400

H	28.55300	-18.28100	-24.72200
O	37.23000	-9.79900	-31.60700
H	37.09500	-8.95400	-31.17800
H	37.77100	-9.59700	-32.37000
O	33.71700	-23.72000	-14.94500
H	33.32400	-24.27300	-14.27000
H	34.53400	-23.40900	-14.55400
O	30.68300	-12.28100	-31.03400
H	31.19600	-11.48700	-30.88300
H	31.33600	-12.97900	-31.10000
O	32.49300	-9.16500	-28.95100
H	32.67000	-8.24300	-29.13900
H	33.28900	-9.47900	-28.52200
O	36.63400	-22.30400	-17.35300
H	37.37500	-21.70300	-17.43300
H	36.86600	-23.04600	-17.91100
O	31.77800	-17.11800	-19.07500
H	31.66400	-18.02000	-18.77500
H	30.92300	-16.87200	-19.42700
O	38.85700	-9.17700	-17.47700
H	38.41400	-9.96300	-17.79400
H	39.51900	-8.98900	-18.14200
O	33.61600	-7.64700	-22.95000
H	34.16700	-7.77500	-23.72300
H	32.77700	-7.34800	-23.30100
O	40.95500	-8.89600	-19.44800
H	40.93700	-8.07500	-19.94000
H	41.87700	-9.02100	-19.22300
O	37.41300	-11.38200	-18.60600
H	37.94600	-12.14300	-18.83600
H	36.88400	-11.67500	-17.86400
O	28.64800	-13.95300	-30.25800
H	29.48600	-13.54300	-30.47400
H	28.82600	-14.89300	-30.28800
O	38.77800	-12.25300	-31.09400
H	38.25000	-12.98800	-31.40700
H	38.27700	-11.47600	-31.34000
O	33.51000	-21.59100	-28.73300
H	34.08400	-21.81400	-29.46600
H	34.09700	-21.52200	-27.98100
O	39.04600	-18.22900	-25.68100
H	38.12800	-18.00800	-25.83900
H	39.46200	-18.15400	-26.54000
O	41.77700	-8.88600	-28.86400
H	42.07000	-8.32500	-29.58300
H	42.28700	-8.59300	-28.10900
O	28.67100	-16.61400	-29.58700
H	28.47300	-17.20700	-30.31200
H	28.11600	-16.91800	-28.86900
O	36.65100	-16.72100	-27.46700
H	36.70500	-17.67400	-27.54300
H	35.84000	-16.56500	-26.98300
O	34.59000	-18.10300	-29.70800
H	33.77700	-17.80400	-29.30200
H	35.08600	-18.49800	-28.99100
O	36.74100	-8.10800	-27.77500
H	36.69800	-7.88200	-28.70400
H	37.65700	-8.34100	-27.62600
O	40.18700	-17.63900	-20.87700
H	40.54200	-17.41200	-20.01700
H	40.83400	-17.30700	-21.49900
O	34.08100	-20.53200	-24.09200
H	34.72100	-20.67300	-24.79000
H	33.24900	-20.81800	-24.46800
O	34.85700	-11.23100	-27.97900
H	35.65100	-11.72600	-28.18000
H	34.14600	-11.76000	-28.34200
O	39.67700	-8.85000	-23.62700
H	39.73000	-8.28100	-22.86000
H	38.80800	-8.67900	-23.99200
O	31.16600	-17.08700	-31.90300
H	30.23500	-17.14900	-31.68600
H	31.26100	-17.61000	-32.69900
O	31.08400	-20.56200	-29.31500
H	31.34500	-19.64700	-29.42400
H	31.90600	-21.02900	-29.16500
O	32.55700	-19.14800	-22.14500
H	33.22900	-19.35600	-22.79400
H	31.73000	-19.34600	-22.58400
O	38.45900	-22.52200	-20.12800
H	37.81200	-21.82300	-20.22200
H	39.22900	-22.08800	-19.76100
O	41.86700	-12.25700	-17.05300
H	41.71600	-12.48600	-17.97000
H	41.85800	-11.30000	-17.04000
O	38.63600	-14.68400	-22.06900
H	39.56500	-14.53200	-22.24200
H	38.21500	-14.59500	-22.92400
O	37.43400	-10.65900	-24.29900
H	37.29600	-11.02700	-23.42600
H	36.64600	-10.14400	-24.47000
O	38.02900	-5.55800	-32.95600
H	38.20400	-6.02900	-32.14200
H	37.34300	-4.93100	-32.72800
O	38.98500	-11.28000	-26.91000
H	38.65000	-11.32000	-26.01400
H	38.48500	-11.94600	-27.38300
O	32.95900	-15.87400	-21.38500
H	32.82100	-16.60600	-20.78400
H	33.87000	-15.96300	-21.66600
O	35.58800	-16.16200	-21.89600
H	36.29800	-16.80200	-21.93800
H	35.54500	-15.91000	-20.97400
O	34.30000	-19.17500	-20.07700
H	33.76400	-19.62600	-19.42500
H	33.71200	-19.04000	-20.82000
O	32.59500	-2.40900	-26.18200
H	32.83500	-1.77700	-25.50400
H	31.75200	-2.76000	-25.89600
O	25.23100	-14.92800	-27.42000
H	25.91800	-15.24200	-28.00800
H	25.07600	-14.02400	-27.69300
O	30.03400	-20.03900	-22.99100
H	29.95000	-20.64700	-23.72600
H	29.21300	-19.54900	-22.99100
O	37.10800	-6.60100	-23.77800
H	37.03500	-5.79500	-23.26600
H	36.66600	-6.40200	-24.60300

O	36.89700	-7.36400	-30.26800
H	36.11400	-6.82100	-30.17400
H	37.59600	-6.84600	-29.86800
O	39.56800	-6.70200	-21.62200
H	40.28100	-6.06700	-21.69500
H	39.07700	-6.60600	-22.43800
O	34.61900	-8.95300	-20.80200
H	35.32000	-8.31900	-20.64900
H	34.14900	-8.61100	-21.56200
O	42.41900	-16.39800	-22.97700
H	41.76600	-16.15100	-23.63100
H	42.79700	-17.21200	-23.31000
O	39.38000	-6.36200	-28.87400
H	39.56200	-7.18000	-28.41100
H	40.20300	-6.14500	-29.31100
O	39.71400	-7.43500	-31.61200
H	40.24300	-7.94600	-32.22500
H	39.97700	-7.75100	-30.74800
O	38.46800	-15.65600	-16.49000
H	37.79200	-15.75700	-15.82000
H	39.29100	-15.81200	-16.02600
O	30.21600	-9.41900	-23.86900
H	30.80100	-10.16500	-23.73600
H	30.01800	-9.10900	-22.98500
O	33.07000	-6.38400	-28.85200
H	32.40100	-5.89900	-29.33600
H	32.93100	-6.13700	-27.93800
O	24.80800	-16.84800	-25.14800
H	24.84600	-17.38200	-24.35500
H	24.30200	-17.37500	-25.76700
O	37.21000	-4.34900	-22.25400
H	37.76200	-4.18600	-21.48900
H	37.55100	-3.75900	-22.92600
O	32.18700	-22.00200	-21.65200
H	32.25100	-21.14000	-22.06400
H	32.12400	-22.61500	-22.38500
O	31.66200	-19.93000	-18.00500
H	32.28900	-20.31200	-17.39100
H	30.80500	-20.14800	-17.63900
O	42.06100	-9.52300	-16.39900
H	42.60500	-8.91000	-16.89400
H	41.48000	-8.96500	-15.88300
O	28.43900	-18.12200	-32.07300
H	28.73400	-19.03100	-32.12600
H	27.53700	-18.14300	-32.39500
O	40.34700	-6.40100	-25.26800
H	40.22200	-7.20200	-25.77700
H	41.00500	-5.90700	-25.75600
O	31.96800	-20.04600	-26.09000
H	31.24700	-19.43300	-26.22900
H	32.75700	-19.51100	-26.16800
O	42.41100	-8.60000	-23.64000
H	41.46400	-8.72000	-23.71200
H	42.75900	-9.48800	-23.55700
O	34.77100	-22.71100	-19.81600
H	35.29400	-22.46500	-19.05300
H	34.55200	-21.87900	-20.23600
O	30.16700	-20.44100	-31.94900
H	29.83900	-21.32100	-32.13100
H	30.52100	-20.49600	-31.06100
O	39.41500	-8.80500	-27.43900
H	39.26800	-9.73300	-27.25900
H	40.24100	-8.78300	-27.92400
O	35.97600	-21.77000	-22.13100
H	35.63600	-22.56900	-21.72900
H	35.27000	-21.46900	-22.70300
O	36.62800	-24.68500	-23.67800
H	37.45000	-25.15900	-23.80000
H	36.87100	-23.90600	-23.17800
O	34.15100	-17.99300	-25.86900
H	34.00600	-17.67900	-24.97700
H	33.67400	-17.37400	-26.42200
O	36.01100	-21.00300	-25.82100
H	35.99700	-21.89800	-26.15800
H	36.72800	-20.99500	-25.18700
O	37.86400	-20.56900	-23.77600
H	37.60500	-19.64700	-23.77700
H	37.33200	-20.96600	-23.08600
O	28.62600	-15.45200	-22.93000
H	28.08100	-14.67600	-22.79800
H	29.50400	-15.10500	-23.08800
O	29.41300	-16.32500	-20.03700
H	28.67100	-16.17700	-19.45100
H	29.09200	-16.06400	-20.90100
O	37.35700	-6.78400	-20.23900
H	37.66600	-7.02900	-19.36700
H	38.15400	-6.57900	-20.72700
O	37.37900	-4.63000	-28.00900
H	36.93900	-5.12200	-27.31500
H	38.13000	-5.17300	-28.25000
O	29.85800	-8.43900	-28.00600
H	30.68400	-8.73800	-28.38600
H	29.50800	-9.20800	-27.55700
O	34.82600	-9.08600	-25.93700
H	34.20400	-9.74900	-26.23600
H	35.49700	-9.06000	-26.61900
O	28.05900	-10.52000	-27.04700
H	27.22200	-10.94800	-27.22900
H	28.03100	-10.32700	-26.11000
O	34.89900	-20.60800	-16.34300
H	34.94800	-19.73200	-16.72500
H	35.44100	-21.15000	-16.91600
O	34.38800	-10.53600	-32.46100
H	34.53700	-9.85200	-33.11400
H	35.19400	-10.55500	-31.94600
O	37.12900	-19.85500	-19.58900
H	36.25900	-19.56100	-19.86000
H	37.36500	-19.27100	-18.86800
O	32.14300	-14.50900	-31.45500
H	31.89800	-15.41300	-31.25700
H	32.31300	-14.50700	-32.39700
O	39.83300	-23.63700	-22.07200
H	40.35100	-22.90100	-22.39700
H	39.47800	-23.33000	-21.23700
O	41.97600	-21.70900	-22.61900
H	41.38100	-21.42900	-23.31400
H	41.69100	-21.22000	-21.84700
O	38.15600	-12.65300	-16.18800

H	39.07600	-12.47500	-15.99400
H	38.12100	-13.59700	-16.34000
O	27.37400	-17.45900	-27.26200
H	27.05400	-18.31300	-27.55500
H	26.82700	-17.24400	-26.50700
O	31.15200	-14.74300	-23.54600
H	31.37100	-13.81100	-23.52700
H	31.56500	-15.10200	-22.76000
O	36.02500	-19.17000	-14.15500
H	35.92400	-18.22000	-14.22400
H	35.80700	-19.49800	-15.02800
O	27.24300	-13.83900	-25.29300
H	26.54900	-14.25300	-25.80600
H	27.99000	-13.79700	-25.89000
O	35.88800	-15.74000	-15.33900
H	35.40500	-14.94000	-15.13200
H	35.97400	-15.72900	-16.29200
O	29.15700	-13.90700	-27.41700
H	29.13400	-13.80800	-28.36800
H	29.46700	-13.06000	-27.09500
O	31.81500	-11.84900	-23.30200
H	32.77000	-11.88100	-23.36900
H	31.61700	-12.31000	-22.48700
O	32.40500	-13.27900	-20.73500
H	33.22500	-12.78500	-20.71100
H	32.67900	-14.19600	-20.76300
O	30.17000	-6.30700	-26.28500
H	30.03600	-7.06700	-26.85100
H	29.75400	-5.58300	-26.75200
O	33.32700	-5.41000	-26.07300
H	33.21000	-4.46600	-26.17500
H	32.50600	-5.71300	-25.68400
O	27.65900	-18.94500	-20.03600
H	28.30500	-19.45600	-19.54700
H	27.45000	-18.20900	-19.46100
O	40.18400	-11.87700	-14.53600
H	41.05300	-11.70300	-14.89800
H	39.70500	-11.05800	-14.65900
O	34.35300	-4.75800	-22.85700
H	35.25500	-4.72500	-22.54100
H	34.12600	-5.68800	-22.84300
O	36.10800	-6.00500	-26.19600
H	35.15800	-6.00400	-26.31000
H	36.41100	-6.75100	-26.71300
O	31.99800	-21.79900	-15.93800
H	32.86200	-22.15500	-15.72800
H	31.42500	-22.13700	-15.25100
O	42.05700	3.13300	-26.36800
H	42.85000	2.79200	-26.78300
H	42.29700	3.24200	-25.44700
O	41.62600	-0.49800	-26.42600
H	41.75500	-0.24400	-25.51200
H	40.94600	0.09500	-26.74600
O	40.57300	-3.02600	-26.38100
H	39.78300	-2.77000	-26.85700
H	41.06400	-2.21100	-26.27600
O	42.52500	-2.66100	-29.42300
H	41.72800	-2.54900	-28.90400
H	42.29700	-2.32200	-30.28900
O	38.85700	-2.77200	-23.95000
H	39.10800	-1.88800	-23.68400
H	39.39300	-2.95200	-24.72200
O	41.52300	3.04800	-31.03400
H	42.03500	3.84200	-30.88300
H	42.17500	2.35000	-31.10000
O	42.61800	-1.78900	-19.07500
H	42.50300	-2.69100	-18.77500
H	41.76200	-1.54300	-19.42700
O	39.48800	1.37700	-30.25800
H	40.32500	1.78600	-30.47400
H	39.66600	0.43700	-30.28800
O	39.51000	-1.28400	-29.58700
H	39.31300	-1.87700	-30.31200
H	38.95500	-1.58900	-28.86900
O	42.00500	-1.75800	-31.90300
H	41.07500	-1.81900	-31.68600
H	42.10100	-2.28000	-32.69900
O	41.92300	-5.23200	-29.31500
H	42.18400	-4.31800	-29.42400
H	42.74500	-5.70000	-29.16500
O	36.07000	0.40100	-27.42000
H	36.75700	0.08700	-28.00800
H	35.91500	1.30500	-27.69300
O	40.87400	-4.71000	-22.99100
H	40.79000	-5.31800	-23.72600
H	40.05200	-4.21900	-22.99100
O	41.05500	5.91000	-23.86900
H	41.64100	5.16500	-23.73600
H	40.85800	6.22100	-22.98500
O	35.64700	-1.51900	-25.14800
H	35.68600	-2.05300	-24.35500
H	35.14100	-2.04600	-25.76700
O	39.27800	-2.79300	-32.07300
H	39.57300	-3.70200	-32.12600
H	38.37700	-2.81400	-32.39500
O	41.00700	-5.11100	-31.94900
H	40.67800	-5.99200	-32.13100
H	41.36100	-5.16700	-31.06100
O	39.46500	-0.12300	-22.93000
H	38.92100	0.65300	-22.79800
H	40.34300	0.22400	-23.08800
O	40.25200	-0.99600	-20.03700
H	39.51000	-0.84800	-19.45100
H	39.93100	-0.73500	-20.90100
O	40.69800	6.89100	-28.00600
H	41.52300	6.59100	-28.38600
H	40.34700	6.12100	-27.55700
O	38.89800	4.80900	-27.04700
H	38.06200	4.38100	-27.22900
H	38.87100	5.00200	-26.11000
O	38.21300	-2.13000	-27.26200
H	37.89400	-2.98300	-27.55500
H	37.66700	-1.91500	-26.50700
O	41.99100	0.58600	-23.54600
H	42.21000	1.51800	-23.52700
H	42.40400	0.22700	-22.76000
O	38.08200	1.49000	-25.29300
H	37.38900	1.07600	-25.80600

H	38.83000	1.53200	-25.89000
O	39.99600	1.42200	-27.41700
H	39.97300	1.52100	-28.36800
H	40.30600	2.26900	-27.09500
O	41.01000	9.02200	-26.28500
H	40.87500	8.26200	-26.85100
H	40.59300	9.74600	-26.75200
O	38.49800	-3.61600	-20.03600
H	39.14400	-4.12600	-19.54700
H	38.29000	-2.87900	-19.46100
O	-40.75600	-2.50600	25.94900
H	-40.02400	-3.07600	26.18200
H	-41.51200	-2.89400	26.38900
O	-41.47500	-5.87300	29.26500
H	-42.11700	-5.57900	28.61900
H	-41.97900	-5.98700	30.07100
O	-41.33500	3.79200	24.70000
H	-42.02000	4.17200	24.14900
H	-41.25000	4.40100	25.43300
O	-34.87400	0.65900	25.82000
H	-35.62800	0.09000	25.66400
H	-34.74000	0.62500	26.76700
O	-41.19700	1.79700	20.46800
H	-41.97400	1.45800	20.02300
H	-40.51800	1.80300	19.79300
O	-40.79800	0.81200	22.93000
H	-40.93600	1.00200	22.00200
H	-41.15400	1.57400	23.38700
O	-40.77100	2.87900	27.94100
H	-40.33700	2.78700	28.78900
H	-40.05800	2.85500	27.30300
O	-39.90900	2.92200	30.47400
H	-39.71600	3.82600	30.72000
H	-40.00900	2.46200	31.30800
O	-38.65500	-0.52100	27.11900
H	-38.66600	0.36200	26.74900
H	-39.52400	-0.87400	26.92500
O	-42.03800	-1.62600	23.58600
H	-41.57200	-0.79300	23.51200
H	-41.58700	-2.09200	24.29100
O	-38.05300	0.62900	23.72400
H	-37.61000	-0.12000	23.32600
H	-38.93000	0.62000	23.34200
O	-41.02000	4.63200	20.73400
H	-40.49500	4.97100	20.00900
H	-41.10800	3.69700	20.55000
O	-41.21100	2.11500	33.35200
H	-41.16100	1.95000	34.29400
H	-41.32000	1.24700	32.96200
O	-39.70900	4.53400	33.59600
H	-40.00100	4.89700	34.43300
H	-40.32200	3.82100	33.41800
O	-38.51000	2.10000	26.11600
H	-37.67500	2.53900	26.28000
H	-38.37400	1.62400	25.29700
O	-41.46000	-0.34200	32.06400
H	-40.98500	-0.15000	31.25600
H	-42.38400	-0.28600	31.81900
O	-40.28100	-0.24500	29.41100
H	-40.31400	-1.18400	29.59700
H	-39.67100	-0.16700	28.67800
O	-40.13200	-3.45400	29.45700
H	-40.64800	-4.23800	29.27000
H	-39.24800	-3.78200	29.62200
O	-42.47100	-9.71000	25.41700
H	-42.66800	-10.01800	26.30100
H	-41.86100	-8.98400	25.54600
O	-34.46400	0.81600	28.55200
H	-33.73300	0.55500	29.11300
H	-35.07300	0.07900	28.59200
O	-36.38200	-1.04300	29.14900
H	-37.10900	-1.17700	28.54100
H	-36.79700	-0.73500	29.95500
O	-41.20200	-5.53400	25.44500
H	-40.42600	-5.04500	25.71800
H	-41.92700	-5.10900	25.90200
O	-38.76400	-4.82200	26.50300
H	-38.17000	-5.06200	25.79200
H	-38.69200	-5.54100	27.13000
O	-40.76900	-4.25500	22.77000
H	-41.02500	-4.65500	23.60100
H	-41.39300	-3.54000	22.64500
O	-36.45700	-1.51500	25.27700
H	-37.01700	-1.44600	26.05000
H	-37.06300	-1.69100	24.55700
O	-34.87200	15.16100	27.97000
H	-34.23400	15.04100	27.26600
H	-34.99000	16.10900	28.02900
O	-38.06300	15.11300	27.90600
H	-38.40100	14.99800	27.01800
H	-37.19400	14.71300	27.88600
O	-42.20400	16.96700	23.89600
H	-42.76800	16.87600	23.12700
H	-42.31900	16.14700	24.37600
O	-36.62400	20.63400	19.20700
H	-37.27500	21.21900	18.82100
H	-35.83000	21.16500	19.26700
O	-42.69900	17.72300	19.60600
H	-41.92500	17.17600	19.46700
H	-42.34600	18.58700	19.81700
O	-40.36200	13.88200	17.23800
H	-40.10900	13.36000	16.47600
H	-41.29900	13.71900	17.34100
O	-35.31800	12.88600	25.04500
H	-34.53100	12.46800	24.69400
H	-35.05400	13.79000	25.21300
O	-35.14700	18.32300	19.74400
H	-35.78200	18.39700	20.45600
H	-35.47700	18.91200	19.06600
O	-29.91700	12.82300	25.94900
H	-29.18400	12.25300	26.18200
H	-30.67300	12.43600	26.38900
O	-40.64200	15.78500	19.44000
H	-40.75900	15.11000	20.10800
H	-40.53200	15.29800	18.62300
O	-38.29900	15.24600	23.14500
H	-38.66300	14.71900	23.85600
H	-37.44200	14.85600	22.97200

O	-32.39400	6.53500	30.40800
H	-32.72600	7.40500	30.18400
H	-32.99000	6.21900	31.08700
O	-38.65900	10.27500	18.14100
H	-38.49100	11.14300	18.50800
H	-38.84900	10.43800	17.21700
O	-32.50100	11.25500	27.48700
H	-33.41000	11.18700	27.19300
H	-32.55300	11.73200	28.31600
O	-38.68000	12.73100	19.12600
H	-38.80000	13.17700	19.96400
H	-39.37400	13.07900	18.56700
O	-30.63500	9.45700	29.26500
H	-31.27800	9.75000	28.61900
H	-31.14000	9.34200	30.07100
O	-32.98100	13.20300	19.95400
H	-32.06900	13.43600	19.77900
H	-33.49000	13.78700	19.39100
O	-38.34400	12.56300	30.96300
H	-37.49800	12.98700	31.10700
H	-38.40400	11.90600	31.65700
O	-34.37400	18.18600	27.34500
H	-35.00300	18.35000	26.64300
H	-34.76200	18.60000	28.11600
O	-30.49500	19.12100	24.70000
H	-31.18000	19.50100	24.14900
H	-30.41100	19.73100	25.43300
O	-35.19400	10.22300	32.62100
H	-35.43800	10.97700	32.08500
H	-34.91800	10.60200	33.45600
O	-33.24000	16.76700	31.29900
H	-33.02800	17.41000	30.62300
H	-33.28700	15.93300	30.83100
O	-35.63800	9.37900	28.20300
H	-36.49900	9.67700	28.49400
H	-35.61100	9.59600	27.27100
O	-38.82100	17.92300	24.61000
H	-38.35800	18.20200	23.82100
H	-39.08600	17.02100	24.42700
O	-39.88500	14.04500	21.41900
H	-39.18200	14.49700	21.88600
H	-40.21600	13.40400	22.04700
O	-32.12600	19.64400	29.47100
H	-31.30400	19.24200	29.19000
H	-32.12900	19.54100	30.42300
O	-24.03500	15.98800	25.82000
H	-24.78900	15.41900	25.66400
H	-23.90000	15.95400	26.76700
O	-38.64500	20.85900	21.49500
H	-38.78100	21.70500	21.92400
H	-38.10400	21.06100	20.73200
O	-35.29600	4.38200	30.02500
H	-35.63900	5.27300	29.94900
H	-34.92800	4.19300	29.16200
O	-35.33100	14.37300	18.53200
H	-35.84600	15.14400	18.77100
H	-35.60300	13.70000	19.15600
O	-37.74600	9.21800	23.59100
H	-38.60200	8.82900	23.41200
H	-37.33300	9.29700	22.73100
O	-36.21600	6.75300	27.99000
H	-36.72000	6.71600	27.17700
H	-35.92300	7.66200	28.05000
O	-39.97200	6.83700	26.99700
H	-39.10700	6.64100	26.63700
H	-39.90400	6.60800	27.92400
O	-30.35700	17.12700	20.46800
H	-31.13400	16.78800	20.02300
H	-29.67800	17.13200	19.79300
O	-35.54200	12.24600	20.35800
H	-35.84700	12.56100	21.20900
H	-34.58900	12.32400	20.40100
O	-37.09800	18.40500	22.00800
H	-37.62500	17.67000	21.69500
H	-37.59900	19.18300	21.76200
O	-32.11800	16.60000	27.12400
H	-32.77600	17.29000	27.21200
H	-31.31400	16.99000	27.46800
O	-42.36600	9.06700	24.36800
H	-41.79100	8.84500	23.63600
H	-41.77900	9.13600	25.12100
O	-33.30800	14.95400	25.17500
H	-32.79500	15.58700	25.67700
H	-32.65800	14.35500	24.80900
O	-40.06300	5.94100	23.09600
H	-40.71700	5.62600	22.47200
H	-40.10500	5.31900	23.82200
O	-38.95200	16.22300	15.01100
H	-39.86000	15.99200	15.21000
H	-38.45500	15.42700	15.19900
O	-36.04600	24.93300	17.93100
H	-36.71900	25.23600	18.53900
H	-35.65900	25.73600	17.58100
O	-36.83000	12.42900	27.42100
H	-37.74700	12.65100	27.26300
H	-36.41400	12.50400	26.56200
O	-36.04600	13.82800	22.55100
H	-35.29900	14.34100	22.24200
H	-35.77700	13.50700	23.41100
O	-37.77300	16.32400	19.53400
H	-38.68200	16.04000	19.63100
H	-37.74400	16.74400	18.67400
O	-32.54200	19.93500	23.30300
H	-32.99500	19.09400	23.35900
H	-33.17200	20.57100	23.64000
O	-33.59800	10.86400	24.53300
H	-34.33600	10.35200	24.86400
H	-33.34600	10.42400	23.72100
O	-34.09800	21.77300	24.23800
H	-33.80600	22.33300	23.51900
H	-33.58800	22.06500	24.99300
O	-33.49700	3.64200	25.12700
H	-33.19100	2.78200	24.84000
H	-32.77200	4.23400	24.92700
O	-39.22500	13.93700	25.63500
H	-39.17000	12.98500	25.55900
H	-40.03600	14.09300	26.11900
O	-38.07600	17.86500	16.87200

H	-38.51400	17.14900	16.41300
H	-37.33500	18.09300	16.31100
O	-41.28600	12.55500	23.39400
H	-42.09900	12.85400	23.80000
H	-40.79000	12.16000	24.11100
O	-35.68800	13.01900	32.22500
H	-35.33300	13.24600	33.08500
H	-35.04100	13.35100	31.60300
O	-29.95900	16.14100	22.93000
H	-30.09700	16.33100	22.00200
H	-30.31500	16.90300	23.38700
O	-41.79400	10.12700	29.01000
H	-41.15400	9.98600	28.31200
H	-42.62700	9.84000	28.63400
O	-29.93200	18.20900	27.94100
H	-29.49800	18.11600	28.78900
H	-29.21800	18.18400	27.30300
O	-41.01800	19.42700	25.12300
H	-40.22400	18.93200	24.92200
H	-41.72900	18.89900	24.76000
O	-29.07000	18.25100	30.47400
H	-28.87600	19.15600	30.72000
H	-29.17000	17.79200	31.30800
O	-37.41600	8.13700	32.97400
H	-38.06400	8.83600	32.88000
H	-36.64600	8.57000	33.34100
O	-37.84600	25.10100	20.14600
H	-37.67100	24.63000	20.96000
H	-38.53300	25.72700	20.37400
O	-36.89100	19.37800	26.19200
H	-37.22600	19.33800	27.08800
H	-37.39000	18.71300	25.71900
O	-33.95500	16.72400	16.46600
H	-34.57200	15.99800	16.36900
H	-34.42300	17.48500	16.12200
O	-38.97900	23.29500	22.83400
H	-39.76200	23.83700	22.92800
H	-38.27900	23.81200	23.23300
O	-35.66900	9.10400	25.47700
H	-35.17200	8.35100	25.15800
H	-36.44000	9.14100	24.91200
O	-27.81600	14.80800	27.11900
H	-27.82700	15.69100	26.74900
H	-28.68400	14.45500	26.92500
O	-34.58600	17.86100	23.05100
H	-35.52000	17.98200	22.88200
H	-34.40800	16.95900	22.78700
O	-39.08500	11.24600	25.20500
H	-39.45600	10.69300	25.89300
H	-38.58900	10.64200	24.65200
O	-31.19900	13.70300	23.58600
H	-30.73300	14.53600	23.51200
H	-30.74800	13.23800	24.29100
O	-33.13100	20.04800	20.58500
H	-32.85500	19.93300	21.49400
H	-34.00100	19.64900	20.54600
O	-33.22700	6.07100	27.62500
H	-34.14100	6.30200	27.79400
H	-32.79800	6.15500	28.47700
O	-33.45600	14.26000	30.12500
H	-34.10900	14.50800	29.47100
H	-33.07800	13.44700	29.79200
O	-36.49600	24.29600	24.22800
H	-36.31300	23.47800	24.69100
H	-35.67200	24.51300	23.79100
O	-36.16100	23.22300	21.49000
H	-35.63300	22.71200	20.87700
H	-35.89900	22.90700	22.35400
O	-36.46000	21.85300	25.66200
H	-36.60800	20.92500	25.84300
H	-35.63500	21.87500	25.17800
O	-42.59400	10.50500	21.52100
H	-42.25400	11.03400	20.79800
H	-42.14700	10.84300	22.29600
O	-32.85700	22.67600	26.74900
H	-31.93200	22.47000	26.61100
H	-33.02500	22.42800	27.65800
O	-39.90000	8.88900	30.97100
H	-40.24000	8.09000	31.37300
H	-40.60600	9.18900	30.39900
O	-39.24800	5.97400	29.42400
H	-38.42500	5.50000	29.30100
H	-39.00400	6.75300	29.92300
O	-40.60700	9.00300	19.72000
H	-39.88600	9.38100	19.21600
H	-41.23100	9.72100	19.82300
O	-36.46400	9.70700	20.65600
H	-36.24800	10.59000	20.35500
H	-36.94500	9.31600	19.92600
O	-41.72400	12.66600	27.23300
H	-41.87000	12.98000	28.12500
H	-42.20200	13.28400	26.68000
O	-39.86500	9.65600	27.28100
H	-39.87800	8.76000	26.94400
H	-39.14700	9.66300	27.91500
O	-38.01200	10.09000	29.32600
H	-38.27000	11.01100	29.32500
H	-38.54300	9.69300	30.01600
O	-33.06700	23.19500	22.10500
H	-32.89400	22.62900	21.35300
H	-32.57400	23.99500	21.92300
O	-40.46100	8.53700	22.55000
H	-40.33900	7.60900	22.74900
H	-40.75100	8.55100	21.63700
O	-37.59200	6.22300	25.68600
H	-37.57000	6.28700	24.73100
H	-37.17500	5.38300	25.88000
O	-27.21300	15.95800	23.72400
H	-26.77000	15.20900	23.32600
H	-28.09100	15.94900	23.34200
O	-32.08000	22.51100	19.20500
H	-31.38500	23.13200	19.42400
H	-31.73800	21.66100	19.48000
O	-30.18100	19.96100	20.73400
H	-29.65600	20.30000	20.00900
H	-30.26800	19.02600	20.55000
O	-30.37200	17.44400	33.35200
H	-30.32200	17.27900	34.29400

H	-30.48000	16.57700	32.96200
O	-41.48800	20.12300	20.64100
H	-41.33900	20.80600	19.98800
H	-40.68100	20.10300	21.15600
O	-28.87000	19.86400	33.59600
H	-29.16100	20.22700	34.43300
H	-29.48200	19.15000	33.41800
O	-27.67000	17.42900	26.11600
H	-26.83500	17.86800	26.28000
H	-27.53400	16.95300	25.29700
O	-30.62100	14.98700	32.06400
H	-30.14600	15.18000	31.25600
H	-31.54400	15.04300	31.81900
O	-36.04300	7.02200	31.03000
H	-35.52500	7.75800	30.70400
H	-36.39700	7.32800	31.86400
O	-35.79200	3.44200	26.55000
H	-35.51200	2.77400	27.17600
H	-35.00700	3.63500	26.03700
O	-37.98800	22.61800	18.11200
H	-38.56100	23.23700	17.65900
H	-37.41500	23.16700	18.64600
O	-31.92100	11.86300	30.29700
H	-30.96900	11.96300	30.31800
H	-32.09000	11.07100	30.80700
O	-33.89900	8.94900	30.48300
H	-34.49400	9.22900	29.78800
H	-34.18400	9.43800	31.25500
O	-34.76900	22.90100	19.24300
H	-33.83600	22.83600	19.44700
H	-34.85600	23.73700	18.78500
O	-32.14000	22.21800	16.20400
H	-32.21800	21.88400	17.09700
H	-31.31900	22.71000	16.20300
O	-29.44200	15.08400	29.41100
H	-29.47400	14.14600	29.59700
H	-28.83200	15.16200	28.67800
O	-37.60400	13.95400	15.45400
H	-36.65800	13.82400	15.52300
H	-37.98200	13.21500	15.93100
O	-29.29300	11.87500	29.45700
H	-29.80900	11.09100	29.27000
H	-28.40900	11.54700	29.62200
O	-31.63100	5.61900	25.41700
H	-31.82900	5.31100	26.30100
H	-31.02200	6.34500	25.54600
O	-23.62400	16.14600	28.55200
H	-22.89400	15.88400	29.11300
H	-24.23400	15.40800	28.59200
O	-25.54300	14.28600	29.14900
H	-26.27000	14.15200	28.54100
H	-25.95800	14.59400	29.95500
O	-39.41900	11.87900	15.38900
H	-40.32600	11.58200	15.46600
H	-39.14200	11.58200	14.52300
O	-37.80300	6.42200	21.54400
H	-38.61300	6.07700	21.91900
H	-37.11500	6.11300	22.13300
O	-35.87400	5.50000	23.16600
H	-35.32100	5.92000	23.82500
H	-35.63700	4.57400	23.20900
O	-40.65000	8.73700	15.53100
H	-39.78800	9.06300	15.79000
H	-40.46400	7.95000	15.01800
O	-32.49600	7.61000	22.67400
H	-33.07600	8.26900	22.29200
H	-32.76300	6.78800	22.26300
O	-34.04600	7.01100	24.87600
H	-33.54600	7.12200	24.06800
H	-33.40200	6.70700	25.51600
O	-33.71400	9.77000	21.73000
H	-34.60100	10.05500	21.51200
H	-33.29800	9.61000	20.88300
O	-30.36200	9.79500	25.44500
H	-29.58600	10.28400	25.71800
H	-31.08700	10.22100	25.90200
O	-27.92500	10.50800	26.50300
H	-27.33100	10.26700	25.79200
H	-27.85200	9.78800	27.13000
O	-34.56900	19.53500	15.82500
H	-33.86600	19.74000	16.44000
H	-35.29300	20.10200	16.09000
O	-33.74000	15.28600	21.94500
H	-33.79600	15.74200	21.10600
H	-33.18700	14.52500	21.76700
O	-29.92900	11.07500	22.77000
H	-30.18500	10.67400	23.60100
H	-30.55400	11.78900	22.64500
O	-25.61800	13.81400	25.27700
H	-26.17800	13.88400	26.05000
H	-26.22400	13.63800	24.55700
O	-32.66100	19.97300	17.87400
H	-31.82600	19.54100	17.69200
H	-32.82200	19.80100	18.80200
O	-33.00600	16.47600	19.24000
H	-33.06100	16.31100	18.29900
H	-33.63200	17.18400	19.39600
O	-39.69700	5.97700	18.81900
H	-39.70900	6.92100	18.97500
H	-38.80400	5.79400	18.52500
O	-35.88100	2.57100	23.07300
H	-36.64800	2.01700	23.21900
H	-35.47000	2.64300	23.93500
O	-24.03200	30.49000	27.97000
H	-23.39400	30.37100	27.26600
H	-24.15000	31.43800	28.02900
O	-27.22300	30.44300	27.90600
H	-27.56200	30.32700	27.01800
H	-26.35400	30.04200	27.88600
O	-31.36500	32.29600	23.89600
H	-31.92800	32.20500	23.12700
H	-31.47900	31.47600	24.37600
O	-31.85900	33.05200	19.60600
H	-31.08600	32.50500	19.46700
H	-31.50700	33.91700	19.81700
O	-29.52300	29.21100	17.23800
H	-29.27000	28.69000	16.47600
H	-30.46000	29.04800	17.34100

O	-24.47800	28.21500	25.04500
H	-23.69200	27.79700	24.69400
H	-24.21400	29.12000	25.21300
O	-24.30700	33.65200	19.74400
H	-24.94300	33.72600	20.45600
H	-24.63700	34.24200	19.06600
O	-19.07700	28.15300	25.94900
H	-18.34500	27.58200	26.18200
H	-19.83300	27.76500	26.38900
O	-29.80300	31.11400	19.44000
H	-29.91900	30.43900	20.10800
H	-29.69300	30.62700	18.62300
O	-27.46000	30.57500	23.14500
H	-27.82400	30.04800	23.85600
H	-26.60300	30.18500	22.97200
O	-21.55500	21.86500	30.40800
H	-21.88600	22.73400	30.18400
H	-22.15000	21.54800	31.08700
O	-27.81900	25.60400	18.14100
H	-27.65200	26.47200	18.50800
H	-28.00900	25.76700	17.21700
O	-21.66200	26.58400	27.48700
H	-22.57000	26.51600	27.19300
H	-21.71300	27.06100	28.31600
O	-27.84000	28.06000	19.12600
H	-27.96000	28.50600	19.96400
H	-28.53500	28.40900	18.56700
O	-19.79600	24.78600	29.26500
H	-20.43800	25.07900	28.61900
H	-20.30000	24.67100	30.07100
O	-22.14200	28.53200	19.95400
H	-21.23000	28.76500	19.77900
H	-22.65100	29.11600	19.39100
O	-27.50500	27.89200	30.96300
H	-26.65800	28.31600	31.10700
H	-27.56500	27.23500	31.65700
O	-23.53500	33.51500	27.34500
H	-24.16400	33.67900	26.64300
H	-23.92200	33.92900	28.11600
O	-19.65600	34.45000	24.70000
H	-20.34100	34.83000	24.14900
H	-19.57200	35.06000	25.43300
O	-24.35500	25.55200	32.62100
H	-24.59900	26.30700	32.08500
H	-24.07900	25.93100	33.45600
O	-22.40100	32.09600	31.29900
H	-22.18900	32.73900	30.62300
H	-22.44800	31.26200	30.83100
O	-24.79800	24.70800	28.20300
H	-25.66000	25.00600	28.49400
H	-24.77200	24.92500	27.27100
O	-27.98200	33.25200	24.61000
H	-27.51800	33.53100	23.82100
H	-28.24600	32.35100	24.42700
O	-29.04500	29.37400	21.41900
H	-28.34300	29.82700	21.88600
H	-29.37700	28.73300	22.04700
O	-21.28600	34.97300	29.47100
H	-20.46400	34.57100	29.19000
H	-21.29000	34.87000	30.42300
O	-33.35100	27.99700	23.67900
H	-34.14800	28.10900	24.19800
H	-33.57900	28.33600	22.81300
O	-13.19600	31.31700	25.82000
H	-13.94900	30.74800	25.66400
H	-13.06100	31.28400	26.76700
O	-24.45700	19.71200	30.02500
H	-24.80000	20.60200	29.94900
H	-24.08900	19.52200	29.16200
O	-24.49100	29.70300	18.53200
H	-25.00700	30.47300	18.77100
H	-24.76400	29.02900	19.15600
O	-26.90700	24.54700	23.59100
H	-27.76300	24.15800	23.41200
H	-26.49400	24.62700	22.73100
O	-25.37700	22.08200	27.99000
H	-25.88000	22.04500	27.17700
H	-25.08400	22.99100	28.05000
O	-29.13300	22.16600	26.99700
H	-28.26700	21.97000	26.63700
H	-29.06500	21.93700	27.92400
O	-19.51800	32.45600	20.46800
H	-20.29500	32.11700	20.02300
H	-18.83900	32.46100	19.79300
O	-24.70300	27.57500	20.35800
H	-25.00800	27.89000	21.20900
H	-23.75000	27.65300	20.40100
O	-26.25800	33.73400	22.00800
H	-26.78600	33.00000	21.69500
H	-26.75900	34.51200	21.76200
O	-21.27900	31.92900	27.12400
H	-21.93700	32.61900	27.21200
H	-20.47500	32.31900	27.46800
O	-31.52600	24.39600	24.36800
H	-30.95200	24.17400	23.63600
H	-30.93900	24.46500	25.12100
O	-22.46800	30.28400	25.17500
H	-21.95500	30.91700	25.67700
H	-21.81800	29.68400	24.80900
O	-31.19900	26.42700	18.38800
H	-32.03600	26.76400	18.70900
H	-31.33900	26.29200	17.45100
O	-29.22400	21.27000	23.09600
H	-29.87800	20.95500	22.47200
H	-29.26600	20.64800	23.82200
O	-28.11300	31.55200	15.01100
H	-29.02000	31.32100	15.21000
H	-27.61600	30.75600	15.19900
O	-25.99000	27.75900	27.42100
H	-26.90800	27.98000	27.26300
H	-25.57400	27.83300	26.56200
O	-25.20700	29.15800	22.55100
H	-24.46000	29.67000	22.24200
H	-24.93800	28.83600	23.41100
O	-26.93400	31.65400	19.53400
H	-27.84300	31.36900	19.63100
H	-26.90500	32.07300	18.67400
O	-21.70200	35.26400	23.30300

H	-22.15600	34.42300	23.35900
H	-22.33300	35.90000	23.64000
O	-22.75900	26.19300	24.53300
H	-23.49700	25.68100	24.86400
H	-22.50600	25.75300	23.72100
O	-22.65800	18.97100	25.12700
H	-22.35200	18.11100	24.84000
H	-21.93200	19.56300	24.92700
O	-28.38600	29.26600	25.63500
H	-28.33100	28.31400	25.55900
H	-29.19700	29.42200	26.11900
O	-27.23700	33.19400	16.87200
H	-27.67500	32.47800	16.41300
H	-26.49500	33.42200	16.31100
O	-30.44600	27.88400	23.39400
H	-31.26000	28.18400	23.80000
H	-29.95000	27.49000	24.11100
O	-24.84900	28.34800	32.22500
H	-24.49400	28.57500	33.08500
H	-24.20200	28.68000	31.60300
O	-19.11900	31.47000	22.93000
H	-19.25700	31.66000	22.00200
H	-19.47600	32.23200	23.38700
O	-30.95500	25.45600	29.01000
H	-30.31500	25.31500	28.31200
H	-31.78700	25.16900	28.63400
O	-19.09200	33.53800	27.94100
H	-18.65900	33.44500	28.78900
H	-18.37900	33.51400	27.30300
O	-30.17900	34.75600	25.12300
H	-29.38500	34.26100	24.92200
H	-30.89000	34.22800	24.76000
O	-33.87100	28.90000	21.19900
H	-34.80100	28.83900	21.41600
H	-33.77500	28.37800	20.40300
O	-33.95200	25.42600	23.78700
H	-33.69100	26.34000	23.67800
H	-33.13000	24.95800	23.93700
O	-18.23100	33.58000	30.47400
H	-18.03700	34.48500	30.72000
H	-18.33100	33.12100	31.30800
O	-26.57700	23.46600	32.97400
H	-27.22400	24.16500	32.88000
H	-25.80700	23.90000	33.34100
O	-26.05100	34.70700	26.19200
H	-26.38600	34.66700	27.08800
H	-26.55100	34.04200	25.71900
O	-23.11500	32.05300	16.46600
H	-23.73200	31.32800	16.36900
H	-23.58400	32.81400	16.12200
O	-31.32600	26.27100	15.58800
H	-32.20300	26.15000	15.22400
H	-30.85000	25.48000	15.33600
O	-24.82900	24.43300	25.47700
H	-24.33300	23.68000	25.15800
H	-25.60100	24.47000	24.91200
O	-16.97700	30.13800	27.11900
H	-16.98800	31.02000	26.74900
H	-17.84500	29.78500	26.92500
O	-23.74700	33.19100	23.05100
H	-24.68100	33.31100	22.88200
H	-23.56900	32.28800	22.78700
O	-28.24600	26.57500	25.20500
H	-28.61600	26.02200	25.89300
H	-27.75000	25.97100	24.65200
O	-20.35900	29.03200	23.58600
H	-19.89300	29.86500	23.51200
H	-19.90800	28.56700	24.29100
O	-22.29200	35.37700	20.58500
H	-22.01600	35.26200	21.49400
H	-23.16100	34.97900	20.54600
O	-22.38800	21.40000	27.62500
H	-23.30100	21.63100	27.79400
H	-21.95800	21.48400	28.47700
O	-22.61700	29.58900	30.12500
H	-23.27000	29.83700	29.47100
H	-22.23900	28.77600	29.79200
O	-36.59800	27.86600	21.02900
H	-36.30200	26.95700	20.97600
H	-37.49900	27.84400	20.70700
O	-33.06800	25.94100	27.01200
H	-33.79000	26.55500	26.87300
H	-32.27900	26.47700	26.93300
O	-34.86900	25.54700	21.15300
H	-35.19800	24.66700	20.97000
H	-34.51500	25.49200	22.04100
O	-31.75500	25.83400	21.52100
H	-31.41500	26.36300	20.79800
H	-31.30700	26.17200	22.29600
O	-29.06000	24.21800	30.97100
H	-29.40100	23.41900	31.37300
H	-29.76600	24.51800	30.39900
O	-28.40900	21.30300	29.42400
H	-27.58600	20.82900	29.30100
H	-28.16500	22.08200	29.92300
O	-29.76800	24.33200	19.72000
H	-29.04700	24.71000	19.21600
H	-30.39100	25.05100	19.82300
O	-25.62500	25.03600	20.65600
H	-25.40800	25.91900	20.35500
H	-26.10500	24.64500	19.92600
O	-30.88500	27.99500	27.23300
H	-31.03000	28.30900	28.12500
H	-31.36200	28.61300	26.68000
O	-29.02500	24.98500	27.28100
H	-29.03900	24.08900	26.94400
H	-28.30800	24.99200	27.91500
O	-27.17300	25.41900	29.32600
H	-27.43100	26.34100	29.32500
H	-27.70400	25.02200	30.01600
O	-29.62200	23.86700	22.55000
H	-29.50000	22.93800	22.74900
H	-29.91100	23.88000	21.63700
O	-26.75200	21.55200	25.68600
H	-26.73000	21.61600	24.73100
H	-26.33600	20.71200	25.88000
O	-16.37400	31.28800	23.72400
H	-15.93100	30.53800	23.32600

H	-17.25100	31.27900	23.34200
O	-19.34100	35.29100	20.73400
H	-18.81600	35.62900	20.00900
H	-19.42900	34.35500	20.55000
O	-19.53200	32.77300	33.35200
H	-19.48200	32.60800	34.29400
H	-19.64100	31.90600	32.96200
O	-18.03000	35.19300	33.59600
H	-18.32200	35.55600	34.43300
H	-18.64300	34.47900	33.41800
O	-16.83100	32.75800	26.11600
H	-15.99600	33.19700	26.28000
H	-16.69500	32.28200	25.29700
O	-19.78200	30.31600	32.06400
H	-19.30600	30.50900	31.25600
H	-20.70500	30.37200	31.81900
O	-32.89300	31.47800	21.64700
H	-33.13900	30.57500	21.84500
H	-32.72400	31.48000	20.70500
O	-25.20400	22.35100	31.03000
H	-24.68500	23.08700	30.70400
H	-25.55800	22.65700	31.86400
O	-33.62400	27.21100	19.14600
H	-34.31700	27.28600	18.49000
H	-33.86900	26.44700	19.66800
O	-24.95200	18.77100	26.55000
H	-24.67200	18.10300	27.17600
H	-24.16700	18.96400	26.03700
O	-21.08100	27.19300	30.29700
H	-20.13000	27.29200	30.31800
H	-21.25100	26.40000	30.80700
O	-23.06000	24.27800	30.48300
H	-23.65500	24.55900	29.78800
H	-23.34500	24.76800	31.25500
O	-21.30000	37.54700	16.20400
H	-21.37900	37.21300	17.09700
H	-20.47900	38.03900	16.20300
O	-18.60300	30.41300	29.41100
H	-18.63500	29.47500	29.59700
H	-17.99300	30.49200	28.67800
O	-26.76400	29.28300	15.45400
H	-25.81800	29.15300	15.52300
H	-27.14300	28.54400	15.93100
O	-18.45300	27.20400	29.45700
H	-18.96900	26.42000	29.27000
H	-17.56900	26.87600	29.62200
O	-20.79200	20.94800	25.41700
H	-20.99000	20.64000	26.30100
H	-20.18200	21.67500	25.54600
O	-12.78500	31.47500	28.55200
H	-12.05500	31.21300	29.11300
H	-13.39400	30.73800	28.59200
O	-14.70300	29.61600	29.14900
H	-15.43000	29.48100	28.54100
H	-15.11900	29.92300	29.95500
O	-28.58000	27.20800	15.38900
H	-29.48700	26.91100	15.46600
H	-28.30200	26.91100	14.52300
O	-26.96400	21.75200	21.54400
H	-27.77400	21.40600	21.91900
H	-26.27500	21.44200	22.13300
O	-25.03500	20.82900	23.16600
H	-24.48200	21.24900	23.82500
H	-24.79800	19.90300	23.20900
O	-29.81000	24.06700	15.53100
H	-28.94800	24.39200	15.79000
H	-29.62500	23.28000	15.01800
O	-21.65700	22.93900	22.67400
H	-22.23700	23.59800	22.29200
H	-21.92300	22.11700	22.26300
O	-23.20700	22.34000	24.87600
H	-22.70700	22.45200	24.06800
H	-22.56300	22.03600	25.51600
O	-22.87400	25.09900	21.73000
H	-23.76200	25.38400	21.51200
H	-22.45900	24.93900	20.88300
O	-19.52300	25.12400	25.44500
H	-18.74700	25.61400	25.71800
H	-20.24800	25.55000	25.90200
O	-17.08600	25.83700	26.50300
H	-16.49100	25.59600	25.79200
H	-17.01300	25.11700	27.13000
O	-23.73000	34.86400	15.82500
H	-23.02600	35.06900	16.44000
H	-24.45400	35.43200	16.09000
O	-22.90000	30.61500	21.94500
H	-22.95700	31.07200	21.10600
H	-22.34700	29.85400	21.76700
O	-19.09000	26.40400	22.77000
H	-19.34600	26.00300	23.60100
H	-19.71500	27.11800	22.64500
O	-14.77900	29.14300	25.27700
H	-15.33900	29.21300	26.05000
H	-15.38400	28.96800	24.55700
O	-21.82100	35.30200	17.87400
H	-20.98700	34.87000	17.69200
H	-21.98300	35.13000	18.80200
O	-22.16700	31.80500	19.24000
H	-22.22200	31.64000	18.29900
H	-22.79200	32.51300	19.39600
O	-28.85700	21.30600	18.81900
H	-28.86900	22.25000	18.97500
H	-27.96500	21.12300	18.52500
O	-25.04200	17.90000	23.07300
H	-25.80900	17.34600	23.21900
H	-24.63100	17.97300	23.93500
O	-10.71500	37.19400	30.40800
H	-11.04700	38.06300	30.18400
H	-11.31100	36.87800	31.08700
O	-8.95600	40.11500	29.26500
H	-9.59900	40.40800	28.61900
H	-9.46100	40.00000	30.07100
O	-13.61700	35.04100	30.02500
H	-13.96100	35.93100	29.94900
H	-13.24900	34.85100	29.16200
O	-14.53700	37.41100	27.99000
H	-15.04100	37.37400	27.17700
H	-14.24400	38.32100	28.05000

O	-18.29300	37.49500	26.99700
H	-17.42800	37.29900	26.63700
H	-18.22500	37.26600	27.92400
O	-18.38500	36.60000	23.09600
H	-19.03800	36.28400	22.47200
H	-18.42700	35.97700	23.82200
O	-11.81800	34.30000	25.12700
H	-11.51200	33.44000	24.84000
H	-11.09300	34.89200	24.92700
O	-15.73800	38.79500	32.97400
H	-16.38500	39.49400	32.88000
H	-14.96800	39.22900	33.34100
O	-13.99000	39.76300	25.47700
H	-13.49300	39.01000	25.15800
H	-14.76100	39.79900	24.91200
O	-11.54800	36.73000	27.62500
H	-12.46200	36.96000	27.79400
H	-11.11900	36.81300	28.47700
O	-17.56900	36.63200	29.42400
H	-16.74700	36.15800	29.30100
H	-17.32600	37.41100	29.92300
O	-15.91300	36.88100	25.68600
H	-15.89100	36.94500	24.73100
H	-15.49600	36.04200	25.88000
O	-14.36400	37.68000	31.03000
H	-13.84600	38.41600	30.70400
H	-14.71900	37.98600	31.86400
O	-14.11300	34.10000	26.55000
H	-13.83300	33.43200	27.17600
H	-13.32800	34.29300	26.03700
O	-12.22100	39.60700	30.48300
H	-12.81600	39.88800	29.78800
H	-12.50600	40.09700	31.25500
O	-7.61400	42.53400	29.45700
H	-8.13000	41.74900	29.27000
H	-6.73000	42.20500	29.62200
O	-9.95300	36.27800	25.41700
H	-10.15000	35.96900	26.30100
H	-9.34300	37.00400	25.54600
O	-16.12400	37.08100	21.54400
H	-16.93400	36.73500	21.91900
H	-15.43600	36.77100	22.13300
O	-14.19500	36.15900	23.16600
H	-13.64200	36.57800	23.82500
H	-13.95800	35.23200	23.20900
O	-10.81700	38.26900	22.67400
H	-11.39800	38.92700	22.29200
H	-11.08400	37.44600	22.26300
O	-12.36700	37.66900	24.87600
H	-11.86700	37.78100	24.06800
H	-11.72400	37.36500	25.51600
O	-12.03500	40.42900	21.73000
H	-12.92200	40.71400	21.51200
H	-11.62000	40.26800	20.88300
O	-8.68400	40.45300	25.44500
H	-7.90800	40.94300	25.71800
H	-9.40900	40.87900	25.90200
O	-6.24600	41.16600	26.50300
H	-5.65200	40.92600	25.79200
H	-6.17400	40.44700	27.13000
O	-18.01800	36.63500	18.81900
H	-18.03000	37.57900	18.97500
H	-17.12500	36.45200	18.52500
O	-14.20300	33.23000	23.07300
H	-14.96900	32.67500	23.21900
H	-13.79200	33.30200	23.93500
O	-40.75600	-25.50000	12.67300
H	-40.02400	-26.07000	12.90700
H	-41.51200	-25.88700	13.11400
O	-41.47500	-28.86600	15.99000
H	-42.11700	-28.57300	15.34400
H	-41.97900	-28.98100	16.79500
O	-41.33500	-19.20200	11.42400
H	-42.02000	-18.82200	10.87400
H	-41.25000	-18.59200	12.15700
O	-34.87400	-22.33500	12.54400
H	-35.62800	-22.90400	12.38900
H	-34.74000	-22.36900	13.49100
O	-41.19700	-21.19600	7.19200
H	-41.97400	-21.53500	6.74800
H	-40.51800	-21.19100	6.51800
O	-40.79800	-22.18200	9.65500
H	-40.93600	-21.99200	8.72700
H	-41.15400	-21.42000	10.11100
O	-40.77100	-20.11400	14.66600
H	-40.33700	-20.20700	15.51400
H	-40.05800	-20.13900	14.02800
O	-39.90900	-20.07200	17.19900
H	-39.71600	-19.16700	17.44500
H	-40.00900	-20.53100	18.03300
O	-38.65500	-23.51500	13.84400
H	-38.66600	-22.63200	13.47300
H	-39.52400	-23.86800	13.64900
O	-42.03800	-24.62000	10.31100
H	-41.57200	-23.78700	10.23700
H	-41.58700	-25.08500	11.01500
O	-38.05300	-22.36500	10.44900
H	-37.61000	-23.11400	10.05100
H	-38.93000	-22.37400	10.06600
O	-41.02000	-18.36200	7.45800
H	-40.49500	-18.02300	6.73300
H	-41.10800	-19.29700	7.27400
O	-41.33100	-17.72800	22.76300
H	-42.12100	-17.74600	22.22400
H	-41.56700	-17.19000	23.51800
O	-41.21100	-20.87900	20.07700
H	-41.16100	-21.04400	21.01800
H	-41.32000	-21.74600	19.68600
O	-39.70900	-18.45900	20.32100
H	-40.00100	-18.09600	21.15700
H	-40.32200	-19.17300	20.14200
O	-38.51000	-20.89400	12.84100
H	-37.67500	-20.45500	13.00500
H	-38.37400	-21.37000	12.02200
O	-41.46000	-23.33600	18.78900
H	-40.98500	-23.14300	17.98100
H	-42.38400	-23.28000	18.54300
O	-40.28100	-23.23900	16.13600

H	-40.31400	-24.17700	16.32200
H	-39.67100	-23.16100	15.40200
O	-40.13200	-26.44800	16.18200
H	-40.64800	-27.23200	15.99500
H	-39.24800	-26.77600	16.34600
O	-34.46400	-22.17700	15.27600
H	-33.73300	-22.43900	15.83700
H	-35.07300	-22.91400	15.31700
O	-36.38200	-24.03700	15.87400
H	-37.10900	-24.17100	15.26500
H	-36.79700	-23.72900	16.68000
O	-41.20200	-28.52800	12.17000
H	-40.42600	-28.03900	12.44200
H	-41.92700	-28.10200	12.62700
O	-38.76400	-27.81500	13.22700
H	-38.17000	-28.05600	12.51700
H	-38.69200	-28.53500	13.85500
O	-40.76900	-27.24800	9.49400
H	-41.02500	-27.64900	10.32500
H	-41.39300	-26.53400	9.36900
O	-36.45700	-24.50900	12.00200
H	-37.01700	-24.43900	12.77500
H	-37.06300	-24.68500	11.28100
O	-34.87200	-7.83300	14.69500
H	-34.23400	-7.95200	13.99100
H	-34.99000	-6.88500	14.75300
O	-38.06300	-7.88000	14.63100
H	-38.40100	-7.99600	13.74300
H	-37.19400	-8.28100	14.61100
O	-42.20400	-6.02700	10.62000
H	-42.76800	-6.11800	9.85200
H	-42.31900	-6.84700	11.10100
O	-37.58100	-6.72700	20.58900
H	-37.59200	-7.12200	19.71700
H	-37.41900	-7.46000	21.18300
O	-36.62400	-2.36000	5.93200
H	-37.27500	-1.77400	5.54500
H	-35.83000	-1.82900	5.99100
O	-42.69900	-5.27100	6.33100
H	-41.92500	-5.81800	6.19200
H	-42.34600	-4.40600	6.54200
O	-40.86000	-3.94300	19.53700
H	-41.15100	-3.03900	19.42200
H	-40.22400	-3.90200	20.25200
O	-39.93400	-6.56500	16.12700
H	-39.33800	-6.79000	15.41300
H	-39.87700	-7.30700	16.73000
O	-40.36200	-9.11200	3.96200
H	-40.10900	-9.63300	3.20100
H	-41.29900	-9.27500	4.06500
O	-35.31800	-10.10800	11.77000
H	-34.53100	-10.52600	11.41900
H	-35.05400	-9.20300	11.93800
O	-35.14700	-4.67100	6.46900
H	-35.78200	-4.59700	7.18100
H	-35.47700	-4.08100	5.79100
O	-29.91700	-10.17000	12.67300
H	-29.18400	-10.74100	12.90700
H	-30.67300	-10.55800	13.11400
O	-40.64200	-7.20900	6.16500
H	-40.75900	-7.88400	6.83300
H	-40.53200	-7.69500	5.34800
O	-38.29900	-7.74800	9.86900
H	-38.66300	-8.27500	10.58100
H	-37.44200	-8.13800	9.69600
O	-32.39400	-16.45800	17.13300
H	-32.72600	-15.58900	16.90900
H	-32.99000	-16.77500	17.81200
O	-38.65900	-12.71900	4.86600
H	-38.49100	-11.85100	5.23300
H	-38.84900	-12.55600	3.94200
O	-35.90100	-4.25700	16.53200
H	-36.20100	-3.35500	16.42400
H	-36.24600	-4.52400	17.38400
O	-32.50100	-11.73900	14.21200
H	-33.41000	-11.80700	13.91800
H	-32.55300	-11.26200	15.04000
O	-38.68000	-10.26300	5.85000
H	-38.80000	-9.81700	6.68900
H	-39.37400	-9.91400	5.29200
O	-40.91900	2.50700	9.49900
H	-40.99700	3.45800	9.57800
H	-41.52300	2.16200	10.15700
O	-38.42700	-10.32000	22.15500
H	-39.20400	-9.76000	22.14600
H	-37.78800	-9.83400	22.67700
O	-30.63500	-13.53700	15.99000
H	-31.27800	-13.24400	15.34400
H	-31.14000	-13.65200	16.79500
O	-32.98100	-9.79100	6.67800
H	-32.06900	-9.55800	6.50300
H	-33.49000	-9.20700	6.11600
O	-40.17300	-7.51100	21.71700
H	-39.43100	-6.97300	21.44100
H	-40.87900	-6.88500	21.87400
O	-34.69700	-5.36800	20.31400
H	-34.28900	-5.81000	19.57000
H	-35.41900	-5.94300	20.56800
O	-38.34400	-10.43100	17.68800
H	-37.49800	-10.00700	17.83100
H	-38.40400	-11.08800	18.38200
O	-34.37400	-4.80800	14.07000
H	-35.00300	-4.64400	13.36700
H	-34.76200	-4.39400	14.84100
O	-30.49500	-3.87300	11.42400
H	-31.18000	-3.49300	10.87400
H	-30.41100	-3.26300	12.15700
O	-35.19400	-12.77100	19.34600
H	-35.43800	-12.01600	18.80900
H	-34.91800	-12.39200	20.18100
O	-33.24000	-6.22700	18.02400
H	-33.02800	-5.58400	17.34700
H	-33.28700	-7.06100	17.55600
O	-35.63800	-13.61500	14.92700
H	-36.49900	-13.31700	15.21900
H	-35.61100	-13.39800	13.99500
O	-38.82100	-5.07100	11.33500
H	-38.35800	-4.79200	10.54500

H	-39.08600	-5.97200	11.15200
O	-41.92900	-5.52400	14.27300
H	-41.35900	-5.09500	13.63400
H	-41.33800	-5.80500	14.97100
O	-39.88500	-8.94800	8.14400
H	-39.18200	-8.49600	8.61100
H	-40.21600	-9.59000	8.77200
O	-41.43600	-10.35700	22.51900
H	-42.06700	-9.67600	22.75200
H	-41.54300	-10.46900	21.57400
O	-32.12600	-3.35000	16.19600
H	-31.30400	-3.75200	15.91400
H	-32.12900	-3.45300	17.14700
O	-38.36000	-3.80100	18.35200
H	-38.29500	-3.33900	19.18800
H	-39.06900	-4.43100	18.48000
O	-24.03500	-7.00600	12.54400
H	-24.78900	-7.57500	12.38900
H	-23.90000	-7.03900	13.49100
O	-38.64500	-2.13400	8.22000
H	-38.78100	-1.28900	8.64800
H	-38.10400	-1.93200	7.45600
O	-42.15800	-16.05500	24.88100
H	-42.55200	-16.60900	25.55600
H	-41.34200	-15.74400	25.27200
O	-39.24200	-14.63900	22.47300
H	-38.50100	-14.03900	22.39400
H	-39.01000	-15.38200	21.91500
O	-37.01800	-1.51200	22.35000
H	-37.46200	-2.29800	22.03200
H	-36.35700	-1.32400	21.68400
O	-34.92000	-1.23200	20.37800
H	-34.93800	-0.41100	19.88600
H	-33.99800	-1.35600	20.60300
O	-38.46300	-3.71700	21.22000
H	-37.93000	-4.47900	20.99000
H	-38.99100	-4.01000	21.96300
O	-35.29600	-18.61100	16.75000
H	-35.63900	-17.72100	16.67400
H	-34.92800	-18.80100	15.88700
O	-35.33100	-8.62000	5.25700
H	-35.84600	-7.85000	5.49600
H	-35.60300	-9.29400	5.88000
O	-37.74600	-13.77500	10.31600
H	-38.60200	-14.16500	10.13600
H	-37.33300	-13.69600	9.45500
O	-36.21600	-16.24100	14.71500
H	-36.72000	-16.27800	13.90100
H	-35.92300	-15.33200	14.77400
O	-39.97200	-16.15700	13.72100
H	-39.10700	-16.35300	13.36200
H	-39.90400	-16.38600	14.64800
O	-30.35700	-5.86700	7.19200
H	-31.13400	-6.20600	6.74800
H	-29.67800	-5.86200	6.51800
O	-35.54200	-10.74800	7.08300
H	-35.84700	-10.43300	7.93400
H	-34.58900	-10.67000	7.12600
O	-37.09800	-4.58900	8.73200
H	-37.62500	-5.32300	8.42000
H	-37.59900	-3.81100	8.48600
O	-32.11800	-6.39400	13.84900
H	-32.77600	-5.70400	13.93700
H	-31.31400	-6.00400	14.19200
O	-42.36600	-13.92700	11.09300
H	-41.79100	-14.14900	10.36000
H	-41.77900	-13.85800	11.84600
O	-33.30800	-8.03900	11.89900
H	-32.79500	-7.40600	12.40200
H	-32.65800	-8.63900	11.53400
O	-40.06300	-17.05300	9.82000
H	-40.71700	-17.36800	9.19600
H	-40.10500	-17.67500	10.54600
O	-38.95200	-6.77100	1.73600
H	-39.86000	-7.00200	1.93500
H	-38.45500	-7.56700	1.92300
O	-36.04600	1.93900	4.65500
H	-36.71900	2.24200	5.26400
H	-35.65900	2.74200	4.30600
O	-36.83000	-10.56400	14.14500
H	-37.74700	-10.34300	13.98700
H	-36.41400	-10.49000	13.28600
O	-36.04600	-9.16500	9.27500
H	-35.29900	-8.65300	8.96600
H	-35.77700	-9.48700	10.13600
O	-37.77300	-6.66900	6.25800
H	-38.68200	-6.95400	6.35500
H	-37.74400	-6.25000	5.39800
O	-32.54200	-3.05900	10.02700
H	-32.99500	-3.90000	10.08400
H	-33.17200	-2.42300	10.36500
O	-33.59800	-12.13000	11.25700
H	-34.33600	-12.64200	11.58800
H	-33.34600	-12.57000	10.44600
O	-34.09800	-1.22100	10.96200
H	-33.80600	-0.66100	10.24300
H	-33.58800	-0.92800	11.71800
O	-33.49700	-19.35200	11.85200
H	-33.19100	-20.21200	11.56400
H	-32.77200	-18.76000	11.65100
O	-39.22500	-9.05700	12.35900
H	-39.17000	-10.00900	12.28300
H	-40.03600	-8.90100	12.84300
O	-38.07600	-5.12900	3.59700
H	-38.51400	-5.84500	3.13800
H	-37.33500	-4.90100	3.03500
O	-41.28600	-10.43900	10.11800
H	-42.09900	-10.13900	10.52500
H	-40.79000	-10.83300	10.83600
O	-39.13500	-0.44300	12.05200
H	-39.17700	-0.21700	11.12200
H	-38.21900	-0.67700	12.20000
O	-35.68800	-9.97500	18.95000
H	-35.33300	-9.74800	19.80900
H	-35.04100	-9.64300	18.32700
O	-29.95900	-6.85300	9.65500
H	-30.09700	-6.66300	8.72700
H	-30.31500	-6.09100	10.11100

O	-41.79400	-12.86700	15.73400
H	-41.15400	-13.00800	15.03700
H	-42.62700	-13.15400	15.35800
O	-29.93200	-4.78500	14.66600
H	-29.49800	-4.87800	15.51400
H	-29.21800	-4.80900	14.02800
O	-41.01800	-3.56700	11.84800
H	-40.22400	-4.06200	11.64600
H	-41.72900	-4.09500	11.48500
O	-36.19900	-1.18500	16.19900
H	-36.14500	-0.61600	16.96700
H	-37.06700	-1.01500	15.83400
O	-29.07000	-4.74300	17.19900
H	-28.87600	-3.83800	17.44500
H	-29.17000	-5.20200	18.03300
O	-37.41600	-14.85700	19.69800
H	-38.06400	-14.15800	19.60400
H	-36.64600	-14.42300	20.06600
O	-34.00900	-4.59200	22.77300
H	-34.16000	-4.82100	21.85600
H	-34.01800	-3.63500	22.78600
O	-37.24000	-7.01900	17.75800
H	-36.31100	-6.86800	17.58500
H	-37.66100	-6.93100	16.90300
O	-38.44100	-2.99400	15.52800
H	-38.58000	-3.36200	16.40000
H	-39.23000	-2.47900	15.35600
O	-37.84600	2.10700	6.87000
H	-37.67100	1.63600	7.68500
H	-38.53300	2.73400	7.09800
O	-36.89100	-3.61600	12.91600
H	-37.22600	-3.65600	13.81200
H	-37.39000	-4.28100	12.44300
O	-33.95500	-6.27000	3.19000
H	-34.57200	-6.99500	3.09300
H	-34.42300	-5.50900	2.84700
O	-40.28800	-8.49700	17.93000
H	-39.57800	-9.13800	17.88900
H	-40.33100	-8.24600	18.85300
O	-32.29900	-1.56000	20.85100
H	-32.35800	-2.24400	20.18400
H	-31.89700	-0.81800	20.39900
O	-41.57500	-11.51000	19.75000
H	-42.11100	-11.96100	20.40200
H	-42.16300	-11.37600	19.00700
O	-32.29800	-3.59600	19.08200
H	-33.18500	-3.93400	19.20500
H	-31.72800	-4.32800	19.31900
O	-38.76700	1.06400	16.04800
H	-38.84000	1.86900	16.56000
H	-39.21000	1.26300	15.22300
O	-38.97900	0.30100	9.55900
H	-39.76200	0.84300	9.65200
H	-38.27900	0.81800	9.95800
O	-35.66900	-13.89000	12.20200
H	-35.17200	-14.64300	11.88200
H	-36.44000	-13.85300	11.63600
O	-27.81600	-8.18500	13.84400
H	-27.82700	-7.30300	13.47300
H	-28.68400	-8.53800	13.64900
O	-36.30800	0.96300	18.20400
H	-35.59500	1.59700	18.13200
H	-36.79900	1.05900	17.38800
O	-34.58600	-5.13200	9.77600
H	-35.52000	-5.01200	9.60600
H	-34.40800	-6.03500	9.51200
O	-39.08500	-11.74800	11.92900
H	-39.45600	-12.30100	12.61700
H	-38.58900	-12.35200	11.37700
O	-31.19900	-9.29100	10.31100
H	-30.73300	-8.45800	10.23700
H	-30.74800	-9.75600	11.01500
O	-41.25600	-1.28800	19.02500
H	-40.55500	-0.65400	19.17800
H	-41.72700	-0.94700	18.26400
O	-33.13100	-2.94600	7.30900
H	-32.85500	-3.06100	8.21900
H	-34.00100	-3.34400	7.27100
O	-33.22700	-16.92300	14.35000
H	-34.14100	-16.69200	14.51800
H	-32.79800	-16.83900	15.20100
O	-33.45600	-8.73400	16.85000
H	-34.10900	-8.48600	16.19500
H	-33.07800	-9.54700	16.51600
O	-36.49600	1.30200	10.95300
H	-36.31300	0.48500	11.41500
H	-35.67200	1.51900	10.51500
O	-36.16100	0.22900	8.21400
H	-35.63300	-0.28200	7.60100
H	-35.89900	-0.08700	9.07900
O	-37.40800	-7.99200	23.33600
H	-38.08300	-8.09200	24.00700
H	-36.58500	-8.14800	23.80000
O	-38.66500	3.31500	17.57200
H	-38.11300	3.47800	18.33700
H	-38.32500	3.90600	16.90000
O	-33.81400	-1.85900	23.42700
H	-33.27000	-1.24600	22.93300
H	-34.39600	-1.30100	23.94400
O	-35.52800	1.26300	14.55900
H	-35.65300	0.46200	14.05000
H	-34.87100	1.75800	14.07000
O	-33.46500	-0.93600	16.18700
H	-34.41200	-1.05500	16.11400
H	-33.11700	-1.82400	16.26900
O	-41.10500	-15.04600	20.01000
H	-40.58200	-14.80000	20.77300
H	-41.32400	-14.21400	19.59100
O	-36.46000	-1.14100	12.38700
H	-36.60800	-2.06900	12.56800
H	-35.63500	-1.11900	11.90300
O	-42.59400	-12.48900	8.24500
H	-42.25400	-11.96000	7.52300
H	-42.14700	-12.15100	9.02100
O	-32.85700	-0.31800	13.47400
H	-31.93200	-0.52300	13.33600
H	-33.02500	-0.56600	14.38200
O	-39.90000	-14.10500	17.69500

H	-40.24000	-14.90400	18.09700
H	-40.60600	-13.80500	17.12300
O	-39.24800	-17.02000	16.14800
H	-38.42500	-17.49400	16.02600
H	-39.00400	-16.24100	16.64800
O	-40.60700	-13.99100	6.44400
H	-39.88600	-13.61300	5.94100
H	-41.23100	-13.27200	6.54800
O	-36.46400	-13.28700	7.38000
H	-36.24800	-12.40400	7.07900
H	-36.94500	-13.67800	6.65100
O	-41.72400	-10.32800	13.95700
H	-41.87000	-10.01400	14.85000
H	-42.20200	-9.71000	13.40400
O	-39.86500	-13.33800	14.00500
H	-39.87800	-14.23400	13.66900
H	-39.14700	-13.33100	14.63900
O	-38.01200	-12.90400	16.05100
H	-38.27000	-11.98200	16.04900
H	-38.54300	-13.30100	16.74100
O	-33.06700	0.20100	8.83000
H	-32.89400	-0.36400	8.07700
H	-32.57400	1.00100	8.64800
O	-40.46100	-14.45600	9.27400
H	-40.33900	-15.38500	9.47300
H	-40.75100	-14.44300	8.36200
O	-37.59200	-16.77100	12.41100
H	-37.57000	-16.70700	11.45600
H	-37.17500	-17.61100	12.60500
O	-27.21300	-7.03500	10.44900
H	-26.77000	-7.78500	10.05100
H	-28.09100	-7.04400	10.06600
O	-32.08000	-0.48200	5.92900
H	-31.38500	0.13800	6.14800
H	-31.73800	-1.33300	6.20500
O	-30.18100	-3.03200	7.45800
H	-29.65600	-2.69400	6.73300
H	-30.26800	-3.96800	7.27400
O	-38.51900	0.88100	19.58700
H	-38.21000	0.63600	20.45900
H	-37.72100	1.08600	19.09900
O	-38.49700	3.03500	11.81800
H	-38.93600	2.54200	12.51100
H	-37.74600	2.49200	11.57700
O	-41.04900	-1.42100	13.89000
H	-41.67100	-2.08400	13.59000
H	-40.37900	-1.39500	13.20700
O	-40.97700	-12.94300	23.48300
H	-40.92800	-12.06700	23.10100
H	-40.43500	-13.48600	22.91000
O	-30.49100	-2.39900	22.76300
H	-31.28200	-2.41700	22.22400
H	-30.72800	-1.86000	23.51800
O	-30.37200	-5.55000	20.07700
H	-30.32200	-5.71500	21.01800
H	-30.48000	-6.41700	19.68600
O	-41.48800	-2.87100	7.36600
H	-41.33900	-2.18800	6.71200
H	-40.68100	-2.89100	7.88100
O	-33.08100	-9.56700	21.39200
H	-32.18000	-9.70600	21.10100
H	-33.00600	-9.40100	22.33200
O	-38.74700	-12.19000	20.23800
H	-39.61700	-11.89700	19.96600
H	-38.51100	-11.60600	20.95900
O	-28.87000	-3.13000	20.32100
H	-29.16100	-2.76700	21.15700
H	-29.48200	-3.84400	20.14200
O	-27.67000	-5.56500	12.84100
H	-26.83500	-5.12600	13.00500
H	-27.53400	-6.04100	12.02200
O	-30.62100	-8.00700	18.78900
H	-30.14600	-7.81400	17.98100
H	-31.54400	-7.95100	18.54300
O	-36.04300	-15.97200	17.75400
H	-35.52500	-15.23600	17.42900
H	-36.39700	-15.66600	18.58900
O	-35.79200	-19.55200	13.27400
H	-35.51200	-20.22000	13.90000
H	-35.00700	-19.35900	12.76200
O	-37.98800	-0.37600	4.83700
H	-38.56100	0.24300	4.38400
H	-37.41500	0.17400	5.37100
O	-31.92100	-11.13000	17.02200
H	-30.96900	-11.03100	17.04300
H	-32.09000	-11.92300	17.53100
O	-33.89900	-14.04500	17.20800
H	-34.49400	-13.76400	16.51200
H	-34.18400	-13.55500	17.97900
O	-34.76900	-0.09300	5.96800
H	-33.83600	-0.15800	6.17100
H	-34.85600	0.74300	5.50900
O	-32.14000	-0.77600	2.92800
H	-32.21800	-1.11000	3.82200
H	-31.31900	-0.28400	2.92700
O	-29.44200	-7.91000	16.13600
H	-29.47400	-8.84800	16.32200
H	-28.83200	-7.83100	15.40200
O	-37.60400	-9.04000	2.17900
H	-36.65800	-9.17000	2.24700
H	-37.98200	-9.77900	2.65500
O	-29.29300	-11.11900	16.18200
H	-29.80900	-11.90300	15.99500
H	-28.40900	-11.44700	16.34600
O	-31.63100	-17.37500	12.14100
H	-31.82900	-17.68300	13.02600
H	-31.02200	-16.64800	12.27100
O	-23.62400	-6.84800	15.27600
H	-22.89400	-7.11000	15.83700
H	-24.23400	-7.58500	15.31700
O	-25.54300	-8.70700	15.87400
H	-26.27000	-8.84200	15.26500
H	-25.95800	-8.40000	16.68000
O	-37.72000	-4.98800	23.63900
H	-36.80000	-4.81100	23.83300
H	-37.75500	-5.93300	23.48700
O	-39.41900	-11.11500	2.11400
H	-40.32600	-11.41200	2.19000

H	-39.14200	-11.41200	1.24700
O	-37.80300	-16.57100	8.26800
H	-38.61300	-16.91700	8.64400
H	-37.11500	-16.88100	8.85700
O	-35.87400	-17.49400	9.89100
H	-35.32100	-17.07400	10.55000
H	-35.63700	-18.42000	9.93300
O	-39.85100	-11.50500	25.67100
H	-39.95100	-10.55600	25.60200
H	-40.06900	-11.83300	24.79900
O	-40.65000	-14.25600	2.25500
H	-39.78800	-13.93100	2.51500
H	-40.46400	-15.04300	1.74300
O	-32.49600	-15.38400	9.39900
H	-33.07600	-14.72500	9.01700
H	-32.76300	-16.20600	8.98700
O	-34.04600	-15.98300	11.60100
H	-33.54600	-15.87100	10.79200
H	-33.40200	-16.28700	12.24100
O	-39.98800	-8.07500	24.48800
H	-40.47100	-7.27500	24.69400
H	-39.90200	-8.06400	23.53400
O	-33.71400	-13.22400	8.45400
H	-34.60100	-12.93900	8.23600
H	-33.29800	-13.38400	7.60700
O	-30.36200	-13.19900	12.17000
H	-29.58600	-12.70900	12.44200
H	-31.08700	-12.77300	12.62700
O	-27.92500	-12.48600	13.22700
H	-27.33100	-12.72700	12.51700
H	-27.85200	-13.20500	13.85500
O	-34.56900	-3.45900	2.54900
H	-33.86600	-3.25400	3.16500
H	-35.29300	-2.89100	2.81400
O	-33.74000	-7.70800	8.66900
H	-33.79600	-7.25100	7.83000
H	-33.18700	-8.46900	8.49200
O	-29.92900	-11.91900	9.49400
H	-30.18500	-12.32000	10.32500
H	-30.55400	-11.20500	9.36900
O	-25.61800	-9.18000	12.00200
H	-26.17800	-9.11000	12.77500
H	-26.22400	-9.35500	11.28100
O	-32.66100	-3.02100	4.59900
H	-31.82600	-3.45300	4.41700
H	-32.82200	-3.19300	5.52700
O	-33.00600	-6.51800	5.96500
H	-33.06100	-6.68300	5.02300
H	-33.63200	-5.81000	6.12100
O	-35.69200	-4.21300	25.29000
H	-34.82200	-4.03900	24.92900
H	-36.17100	-3.39300	25.16800
O	-39.69700	-17.01700	5.54300
H	-39.70900	-16.07300	5.70000
H	-38.80400	-17.20000	5.24900
O	-41.52300	2.90700	16.96900
H	-40.62000	2.93900	17.28600
H	-41.74900	1.97700	16.98400
O	-39.76800	1.66000	13.63000
H	-40.71800	1.66000	13.51600
H	-39.46400	0.91300	13.11400
O	-35.88100	-20.42300	9.79800
H	-36.64800	-20.97700	9.94300
H	-35.47000	-20.35000	10.65900
O	-24.03200	7.49600	14.69500
H	-23.39400	7.37700	13.99100
H	-24.15000	8.44400	14.75300
O	-27.22300	7.44900	14.63100
H	-27.56200	7.33300	13.74300
H	-26.35400	7.04800	14.61100
O	-31.36500	9.30200	10.62000
H	-31.92800	9.21100	9.85200
H	-31.47900	8.48200	11.10100
O	-26.74100	8.60200	20.58900
H	-26.75300	8.20800	19.71700
H	-26.57900	7.86900	21.18300
O	-33.81800	10.79700	13.45900
H	-33.02600	10.45700	13.04300
H	-33.57900	10.90700	14.37900
O	-25.78500	12.96900	5.93200
H	-26.43600	13.55500	5.54500
H	-24.99000	13.50000	5.99100
O	-31.85900	10.05800	6.33100
H	-31.08600	9.51100	6.19200
H	-31.50700	10.92300	6.54200
O	-30.02100	11.38600	19.53700
H	-30.31200	12.29100	19.42200
H	-29.38500	11.42800	20.25200
O	-29.09500	8.76400	16.12700
H	-28.49900	8.53900	15.41300
H	-29.03800	8.02200	16.73000
O	-29.52300	6.21800	3.96200
H	-29.27000	5.69600	3.20100
H	-30.46000	6.05400	4.06500
O	-24.47800	5.22100	11.77000
H	-23.69200	4.80300	11.41900
H	-24.21400	6.12600	11.93800
O	-24.30700	10.65900	6.46900
H	-24.94300	10.73200	7.18100
H	-24.63700	11.24800	5.79100
O	-19.07700	5.15900	12.67300
H	-18.34500	4.58900	12.90700
H	-19.83300	4.77100	13.11400
O	-29.80300	8.12000	6.16500
H	-29.91900	7.44500	6.83300
H	-29.69300	7.63400	5.34800
O	-27.46000	7.58100	9.86900
H	-27.82400	7.05500	10.58100
H	-26.60300	7.19100	9.69600
O	-21.55500	-1.12900	17.13300
H	-21.88600	-0.26000	16.90900
H	-22.15000	-1.44500	17.81200
O	-27.81900	2.61000	4.86600
H	-27.65200	3.47800	5.23300
H	-28.00900	2.77300	3.94200
O	-25.06200	11.07200	16.53200
H	-25.36200	11.97500	16.42400
H	-25.40700	10.80500	17.38400

O	-31.61000	7.52400	12.56600
H	-32.53500	7.39100	12.77400
H	-31.36200	8.30300	13.06300
O	-21.66200	3.59000	14.21200
H	-22.57000	3.52200	13.91800
H	-21.71300	4.06800	15.04000
O	-27.84000	5.06600	5.85000
H	-27.96000	5.51200	6.68900
H	-28.53500	5.41500	5.29200
O	-34.25000	7.16700	13.40000
H	-34.12000	7.42000	14.31400
H	-34.93000	7.76000	13.08100
O	-30.08000	17.83700	9.49900
H	-30.15700	18.78700	9.57800
H	-30.68300	17.49100	10.15700
O	-27.58800	5.00900	22.15500
H	-28.36500	5.56900	22.14600
H	-26.94900	5.49500	22.67700
O	-19.79600	1.79200	15.99000
H	-20.43800	2.08500	15.34400
H	-20.30000	1.67700	16.79500
O	-22.14200	5.53800	6.67800
H	-21.23000	5.77100	6.50300
H	-22.65100	6.12200	6.11600
O	-29.33300	7.81800	21.71700
H	-28.59200	8.35600	21.44100
H	-30.04000	8.44500	21.87400
O	-23.85800	9.96100	20.31400
H	-23.44900	9.51900	19.57000
H	-24.58000	9.38600	20.56800
O	-27.50500	4.89800	17.68800
H	-26.65800	5.32200	17.83100
H	-27.56500	4.24100	18.38200
O	-23.53500	10.52100	14.07000
H	-24.16400	10.68600	13.36700
H	-23.92200	10.93500	14.84100
O	-19.65600	11.45600	11.42400
H	-20.34100	11.83600	10.87400
H	-19.57200	12.06600	12.15700
O	-24.35500	2.55900	19.34600
H	-24.59900	3.31300	18.80900
H	-24.07900	2.93700	20.18100
O	-22.40100	9.10200	18.02400
H	-22.18900	9.74600	17.34700
H	-22.44800	8.26900	17.55600
O	-24.79800	1.71400	14.92700
H	-25.66000	2.01200	15.21900
H	-24.77200	1.93100	13.99500
O	-27.98200	10.25800	11.33500
H	-27.51800	10.53700	10.54500
H	-28.24600	9.35700	11.15200
O	-31.09000	9.80600	14.27300
H	-30.51900	10.23400	13.63400
H	-30.49900	9.52400	14.97100
O	-29.04500	6.38100	8.14400
H	-28.34300	6.83300	8.61100
H	-29.37700	5.73900	8.77200
O	-30.59700	4.97200	22.51900
H	-31.22700	5.65400	22.75200
H	-30.70300	4.86000	21.57400
O	-35.30300	4.63900	13.44500
H	-36.09300	4.89500	12.96900
H	-34.81100	5.45400	13.55100
O	-21.28600	11.98000	16.19600
H	-20.46400	11.57800	15.91400
H	-21.29000	11.87600	17.14700
O	-33.35100	5.00400	10.40400
H	-34.14800	5.11500	10.92200
H	-33.57900	5.34200	9.53800
O	-27.52100	11.52800	18.35200
H	-27.45600	11.99000	19.18800
H	-28.22900	10.89800	18.48000
O	-37.01900	4.89200	15.87700
H	-36.76800	5.77700	16.14300
H	-36.48300	4.71300	15.10400
O	-13.19600	8.32300	12.54400
H	-13.94900	7.75400	12.38900
H	-13.06100	8.29000	13.49100
O	-27.80600	13.19500	8.22000
H	-27.94100	14.04000	8.64800
H	-27.26500	13.39700	7.45600
O	-31.31900	-0.72600	24.88100
H	-31.71200	-1.27900	25.55600
H	-30.50200	-0.41500	25.27200
O	-34.35300	10.71200	8.79300
H	-33.84000	11.50700	8.94300
H	-33.70100	10.01500	8.72600
O	-32.54300	13.82900	10.87500
H	-32.36700	14.75100	10.68700
H	-31.74800	13.51500	11.30400
O	-28.40200	0.69000	22.47300
H	-27.66100	1.29000	22.39400
H	-28.17000	-0.05200	21.91500
O	-33.25800	5.87500	20.75100
H	-33.37300	4.97400	21.05200
H	-34.11400	6.12200	20.40000
O	-26.17900	13.81700	22.35000
H	-26.62300	13.03100	22.03200
H	-25.51700	14.00500	21.68400
O	-31.42000	15.34700	16.87600
H	-30.86900	15.21800	16.10400
H	-32.25900	15.64600	16.52500
O	-24.08100	14.09700	20.37800
H	-24.09900	14.91800	19.88600
H	-23.15900	13.97300	20.60300
O	-27.62300	11.61200	21.22000
H	-27.09100	10.85100	20.99000
H	-28.15200	11.31900	21.96300
O	-24.45700	-3.28200	16.75000
H	-24.80000	-2.39200	16.67400
H	-24.08900	-3.47200	15.88700
O	-36.38800	9.04100	9.56900
H	-35.55000	9.45100	9.35200
H	-36.21000	8.10100	9.53900
O	-24.49100	6.70900	5.25700
H	-25.00700	7.47900	5.49600
H	-24.76400	6.03500	5.88000
O	-26.90700	1.55400	10.31600

H	-27.76300	1.16400	10.13600
H	-26.49400	1.63300	9.45500
O	-25.37700	-0.91200	14.71500
H	-25.88000	-0.94900	13.90100
H	-25.08400	-0.00200	14.77400
O	-29.13300	-0.82800	13.72100
H	-28.26700	-1.02400	13.36200
H	-29.06500	-1.05700	14.64800
O	-19.51800	9.46200	7.19200
H	-20.29500	9.12300	6.74800
H	-18.83900	9.46700	6.51800
O	-24.70300	4.58100	7.08300
H	-25.00800	4.89600	7.93400
H	-23.75000	4.66000	7.12600
O	-26.25800	10.74000	8.73200
H	-26.78600	10.00600	8.42000
H	-26.75900	11.51800	8.48600
O	-21.27900	8.93500	13.84900
H	-21.93700	9.62500	13.93700
H	-20.47500	9.32500	14.19200
O	-31.52600	1.40200	11.09300
H	-30.95200	1.18000	10.36000
H	-30.93900	1.47100	11.84600
O	-22.46800	7.29000	11.89900
H	-21.95500	7.92300	12.40200
H	-21.81800	6.69000	11.53400
O	-31.19900	3.43300	5.11300
H	-32.03600	3.77000	5.43300
H	-31.33900	3.29800	4.17500
O	-29.22400	-1.72300	9.82000
H	-29.87800	-2.03900	9.19600
H	-29.26600	-2.34600	10.54600
O	-28.11300	8.55800	1.73600
H	-29.02000	8.32700	1.93500
H	-27.61600	7.76200	1.92300
O	-25.20600	17.26800	4.65500
H	-25.88000	17.57100	5.26400
H	-24.82000	18.07100	4.30600
O	-25.99000	4.76500	14.14500
H	-26.90800	4.98600	13.98700
H	-25.57400	4.83900	13.28600
O	-25.20700	6.16400	9.27500
H	-24.46000	6.67600	8.96600
H	-24.93800	5.84200	10.13600
O	-26.93400	8.66000	6.25800
H	-27.84300	8.37600	6.35500
H	-26.90500	9.08000	5.39800
O	-21.70200	12.27000	10.02700
H	-22.15600	11.42900	10.08400
H	-22.33300	12.90700	10.36500
O	-22.75900	3.20000	11.25700
H	-23.49700	2.68700	11.58800
H	-22.50600	2.76000	10.44600
O	-23.25900	14.10800	10.96200
H	-22.96700	14.66900	10.24300
H	-22.74900	14.40100	11.71800
O	-22.65800	-4.02300	11.85200
H	-22.35200	-4.88300	11.56400
H	-21.93200	-3.43100	11.65100
O	-36.36500	6.38000	10.24000
H	-36.56300	5.78700	9.51500
H	-36.92100	6.07600	10.95700
O	-28.38600	6.27300	12.35900
H	-28.33100	5.32000	12.28300
H	-29.19700	6.42900	12.84300
O	-27.23700	10.20100	3.59700
H	-27.67500	9.48400	3.13800
H	-26.49500	10.42800	3.03500
O	-30.44600	4.89000	10.11800
H	-31.26000	5.19000	10.52500
H	-29.95000	4.49600	10.83600
O	-28.29600	14.88600	12.05200
H	-28.33800	15.11200	11.12200
H	-27.37900	14.65200	12.20000
O	-24.84900	5.35400	18.95000
H	-24.49400	5.58200	19.80900
H	-24.20200	5.68600	18.32700
O	-19.11900	8.47600	9.65500
H	-19.25700	8.66600	8.72700
H	-19.47600	9.23800	10.11100
O	-30.95500	2.46200	15.73400
H	-30.31500	2.32100	15.03700
H	-31.78700	2.17600	15.35800
O	-19.09200	10.54400	14.66600
H	-18.65900	10.45200	15.51400
H	-18.37900	10.52000	14.02800
O	-30.17900	11.76200	11.84800
H	-29.38500	11.26700	11.64600
H	-30.89000	11.23400	11.48500
O	-25.35900	14.14400	16.19900
H	-25.30600	14.71300	16.96700
H	-26.22800	14.31400	15.83400
O	-33.87100	5.90700	7.92400
H	-34.80100	5.84500	8.14100
H	-33.77500	5.38400	7.12700
O	-33.95200	2.43200	10.51200
H	-33.69100	3.34700	10.40300
H	-33.13000	1.96500	10.66100
O	-32.48000	3.84600	17.68100
H	-31.80700	3.63800	17.03300
H	-33.30700	3.64800	17.24200
O	-18.23100	10.58600	17.19900
H	-18.03700	11.49100	17.44500
H	-18.33100	10.12700	18.03300
O	-26.57700	0.47200	19.69800
H	-27.22400	1.17100	19.60400
H	-25.80700	0.90600	20.06600
O	-23.16900	10.73700	22.77300
H	-23.32100	10.50800	21.85600
H	-23.17900	11.69400	22.78600
O	-26.40100	8.31000	17.75800
H	-25.47100	8.46100	17.58500
H	-26.82200	8.39900	16.90300
O	-27.60200	12.33500	15.52800
H	-27.74100	11.96700	16.40000
H	-28.39000	12.85000	15.35600
O	-27.00700	17.43600	6.87000
H	-26.83200	16.96500	7.68500

H	-27.69400	18.06300	7.09800
O	-26.05100	11.71300	12.91600
H	-26.38600	11.67400	13.81200
H	-26.55100	11.04800	12.44300
O	-32.07700	7.12000	18.44200
H	-32.21500	6.38800	19.04300
H	-31.16600	7.03100	18.16000
O	-23.11500	9.05900	3.19000
H	-23.73200	8.33400	3.09300
H	-23.58400	9.82000	2.84700
O	-29.44900	6.83200	17.93000
H	-28.73900	6.19200	17.88900
H	-29.49200	7.08300	18.85300
O	-21.46000	13.76900	20.85100
H	-21.51900	13.08500	20.18400
H	-21.05800	14.51100	20.39900
O	-30.73600	3.81900	19.75000
H	-31.27200	3.36800	20.40200
H	-31.32400	3.95300	19.00700
O	-32.44100	20.58500	13.64400
H	-32.20100	21.21700	14.32200
H	-33.28400	20.23400	13.93100
O	-31.32600	3.27800	2.31200
H	-32.20300	3.15600	1.94900
H	-30.85000	2.48600	2.06000
O	-21.45800	11.73300	19.08200
H	-22.34600	11.39500	19.20500
H	-20.88800	11.00100	19.31900
O	-39.80500	8.06500	12.40600
H	-39.11800	7.75100	11.81900
H	-39.96100	8.96900	12.13300
O	-35.00200	2.95400	16.83500
H	-35.08600	2.34700	16.10000
H	-35.82400	3.44500	16.83600
O	-27.92800	16.39300	16.04800
H	-28.00100	17.19900	16.56000
H	-28.37000	16.59200	15.22300
O	-28.13900	15.63000	9.55900
H	-28.92200	16.17200	9.65200
H	-27.44000	16.14800	9.95800
O	-24.82900	1.44000	12.20200
H	-24.33300	0.68700	11.88200
H	-25.60100	1.47600	11.63600
O	-16.97700	7.14400	13.84400
H	-16.98800	8.02600	13.47300
H	-17.84500	6.79100	13.64900
O	-25.46800	16.29200	18.20400
H	-24.75500	16.92600	18.13200
H	-25.95900	16.38800	17.38800
O	-23.74700	10.19700	9.77600
H	-24.68100	10.31700	9.60600
H	-23.56900	9.29400	9.51200
O	-28.24600	3.58100	11.92900
H	-28.61600	3.02900	12.61700
H	-27.75000	2.97700	11.37700
O	-20.35900	6.03900	10.31100
H	-19.89300	6.87100	10.23700
H	-19.90800	5.57300	11.01500
O	-30.41700	14.04100	19.02500
H	-29.71600	14.67500	19.17800
H	-30.88800	14.38300	18.26400
O	-22.29200	12.38300	7.30900
H	-22.01600	12.26900	8.21900
H	-23.16100	11.98500	7.27100
O	-22.38800	-1.59300	14.35000
H	-23.30100	-1.36300	14.51800
H	-21.95800	-1.51000	15.20100
O	-22.61700	6.59600	16.85000
H	-23.27000	6.84300	16.19500
H	-22.23900	5.78200	16.51600
O	-25.65600	16.63200	10.95300
H	-25.47400	15.81400	11.41500
H	-24.83300	16.84800	10.51500
O	-25.32200	15.55800	8.21400
H	-24.79300	15.04800	7.60100
H	-25.05900	15.24300	9.07900
O	-26.56800	7.33800	23.33600
H	-27.24400	7.23700	24.00700
H	-25.74500	7.18200	23.80000
O	-34.82000	13.57500	15.95800
H	-34.23500	12.82900	16.09100
H	-35.01800	13.88500	16.84100
O	-31.96600	16.61000	10.97400
H	-32.63500	17.09500	10.49000
H	-32.10500	16.85700	11.88800
O	-40.22900	6.14500	14.67800
H	-40.19000	5.61200	15.47200
H	-40.73400	5.61900	14.05900
O	-27.82600	18.64400	17.57200
H	-27.27400	18.80700	18.33700
H	-27.48500	19.23500	16.90000
O	-32.84900	0.99200	18.17400
H	-32.78500	1.85300	17.76200
H	-32.91300	0.37900	17.44200
O	-33.37400	3.06400	21.82200
H	-32.74700	2.68200	22.43600
H	-34.23100	2.84600	22.18700
O	-22.97500	13.47000	23.42700
H	-22.43100	14.08300	22.93300
H	-23.55600	14.02800	23.94400
O	-36.59800	4.87200	7.75400
H	-36.30200	3.96300	7.70000
H	-37.49900	4.85100	7.43200
O	-24.68900	16.59300	14.55900
H	-24.81400	15.79100	14.05000
H	-24.03100	17.08700	14.07000
O	-33.06800	2.94700	13.73600
H	-33.79000	3.56100	13.59700
H	-32.27900	3.48300	13.65800
O	-22.62600	14.39300	16.18700
H	-23.57300	14.27400	16.11400
H	-22.27700	13.50600	16.26900
O	-30.26600	0.28300	20.01000
H	-29.74200	0.52900	20.77300
H	-30.48400	1.11500	19.59100
O	-34.86900	2.55300	7.87800
H	-35.19800	1.67300	7.69500
H	-34.51500	2.49800	8.76500

O	-25.62100	14.18900	12.38700
H	-25.76800	13.26000	12.56800
H	-24.79600	14.21100	11.90300
O	-31.75500	2.84000	8.24500
H	-31.41500	3.36900	7.52300
H	-31.30700	3.17900	9.02100
O	-22.01800	15.01200	13.47400
H	-21.09300	14.80600	13.33600
H	-22.18600	14.76300	14.38200
O	-29.06000	1.22400	17.69500
H	-29.40100	0.42500	18.09700
H	-29.76600	1.52400	17.12300
O	-28.40900	-1.69100	16.14800
H	-27.58600	-2.16500	16.02600
H	-28.16500	-0.91200	16.64800
O	-29.76800	1.33800	6.44400
H	-29.04700	1.71600	5.94100
H	-30.39100	2.05700	6.54800
O	-25.62500	2.04200	7.38000
H	-25.40800	2.92500	7.07900
H	-26.10500	1.65100	6.65100
O	-30.88500	5.00100	13.95700
H	-31.03000	5.31500	14.85000
H	-31.36200	5.62000	13.40400
O	-29.02500	1.99100	14.00500
H	-29.03900	1.09500	13.66900
H	-28.30800	1.99800	14.63900
O	-27.17300	2.42500	16.05100
H	-27.43100	3.34700	16.04900
H	-27.70400	2.02800	16.74100
O	-22.22800	15.53000	8.83000
H	-22.05400	14.96500	8.07700
H	-21.73500	16.33000	8.64800
O	-36.41000	7.54200	16.89600
H	-36.95500	8.31800	17.02800
H	-35.53200	7.88900	16.73800
O	-29.62200	0.87300	9.27400
H	-29.50000	-0.05600	9.47300
H	-29.91100	0.88700	8.36200
O	-26.75200	-1.44200	12.41100
H	-26.73000	-1.37800	11.45600
H	-26.33600	-2.28100	12.60500
O	-16.37400	8.29400	10.44900
H	-15.93100	7.54400	10.05100
H	-17.25100	8.28500	10.06600
O	-21.24000	14.84700	5.92900
H	-20.54500	15.46700	6.14800
H	-20.89800	13.99600	6.20500
O	-35.62400	6.66900	19.78900
H	-36.36600	6.81700	20.37600
H	-35.94400	6.92900	18.92600
O	-19.34100	12.29700	7.45800
H	-18.81600	12.63500	6.73300
H	-19.42900	11.36100	7.27400
O	-27.67900	16.21000	19.58700
H	-27.37100	15.96500	20.45900
H	-26.88200	16.41500	19.09900
O	-27.65700	18.36400	11.81800
H	-28.09700	17.87200	12.51100
H	-26.90600	17.82100	11.57700
O	-35.17800	14.55500	11.82000
H	-34.35200	14.25500	11.44000
H	-35.52900	13.78600	12.26900
O	-30.21000	13.90800	13.89000
H	-30.83200	13.24500	13.59000
H	-29.53900	13.93400	13.20700
O	-36.97700	12.47400	12.77900
H	-37.81400	12.04600	12.59700
H	-37.00500	12.66700	13.71600
O	-30.13700	2.38600	23.48300
H	-30.08900	3.26200	23.10100
H	-29.59500	1.84400	22.91000
O	-19.65200	12.93000	22.76300
H	-20.44200	12.91200	22.22400
H	-19.88900	13.46900	23.51800
O	-19.53200	9.77900	20.07700
H	-19.48200	9.61400	21.01800
H	-19.64100	8.91200	19.68600
O	-30.64900	12.45800	7.36600
H	-30.50000	13.14200	6.71200
H	-29.84200	12.43900	7.88100
O	-22.24200	5.76200	21.39200
H	-21.34100	5.62300	21.10100
H	-22.16700	5.92800	22.33200
O	-27.90700	3.13900	20.23800
H	-28.77700	3.43200	19.96600
H	-27.67200	3.72300	20.95900
O	-18.03000	12.19900	20.32100
H	-18.32200	12.56200	21.15700
H	-18.64300	11.48600	20.14200
O	-16.83100	9.76400	12.84100
H	-15.99600	10.20300	13.00500
H	-16.69500	9.28800	12.02200
O	-19.78200	7.32200	18.78900
H	-19.30600	7.51500	17.98100
H	-20.70500	7.37800	18.54300
O	-32.89300	8.48500	8.37200
H	-33.13900	7.58100	8.57000
H	-32.72400	8.48600	7.43000
O	-25.20400	-0.64300	17.75400
H	-24.68500	0.09300	17.42900
H	-25.55800	-0.33700	18.58900
O	-33.62400	4.21700	5.87000
H	-34.31700	4.29200	5.21400
H	-33.86900	3.45400	6.39300
O	-24.95200	-4.22300	13.27400
H	-24.67200	-4.89100	13.90000
H	-24.16700	-4.03000	12.76200
O	-27.14900	14.95300	4.83700
H	-27.72200	15.57200	4.38400
H	-26.57600	15.50300	5.37100
O	-21.08100	4.19900	17.02200
H	-20.13000	4.29900	17.04300
H	-21.25100	3.40700	17.53100
O	-23.06000	1.28400	17.20800
H	-23.65500	1.56500	16.51200
H	-23.34500	1.77400	17.97900
O	-23.92900	15.23700	5.96800

H	-22.99600	15.17100	6.17100
H	-24.01700	16.07200	5.50900
O	-21.30000	14.55300	2.92800
H	-21.37900	14.21900	3.82200
H	-20.47900	15.04500	2.92700
O	-18.60300	7.41900	16.13600
H	-18.63500	6.48100	16.32200
H	-17.99300	7.49800	15.40200
O	-26.76400	6.28900	2.17900
H	-25.81800	6.16000	2.24700
H	-27.14300	5.55000	2.65500
O	-18.45300	4.21100	16.18200
H	-18.96900	3.42600	15.99500
H	-17.56900	3.88200	16.34600
O	-20.79200	-2.04500	12.14100
H	-20.99000	-2.35400	13.02600
H	-20.18200	-1.31900	12.27100
O	-12.78500	8.48100	15.27600
H	-12.05500	8.22000	15.83700
H	-13.39400	7.74400	15.31700
O	-14.70300	6.62200	15.87400
H	-15.43000	6.48700	15.26500
H	-15.11900	6.92900	16.68000
O	-26.88000	10.34100	23.63900
H	-25.96000	10.51900	23.83300
H	-26.91500	9.39600	23.48700
O	-28.58000	4.21400	2.11400
H	-29.48700	3.91700	2.19000
H	-28.30200	3.91700	1.24700
O	-37.66200	5.53500	12.56400
H	-37.98200	4.68100	12.27100
H	-38.20900	5.75000	13.32000
O	-33.88400	8.25100	16.28100
H	-33.66600	9.18300	16.30000
H	-33.47200	7.89200	17.06600
O	-26.96400	-1.24200	8.26800
H	-27.77400	-1.58800	8.64400
H	-26.27500	-1.55200	8.85700
O	-25.03500	-2.16400	9.89100
H	-24.48200	-1.74500	10.55000
H	-24.79800	-3.09100	9.93300
O	-29.01100	3.82400	25.67100
H	-29.11200	4.77400	25.60200
H	-29.22900	3.49600	24.79900
O	-29.81000	1.07300	2.25500
H	-28.94800	1.39800	2.51500
H	-29.62500	0.28600	1.74300
O	-37.79300	9.15500	14.53300
H	-38.48700	8.74000	14.02000
H	-37.04600	9.19700	13.93700
O	-21.65700	-0.05400	9.39900
H	-22.23700	0.60400	9.01700
H	-21.92300	-0.87700	8.98700
O	-23.20700	-0.65400	11.60100
H	-22.70700	-0.54200	10.79200
H	-22.56300	-0.95800	12.24100
O	-29.14800	7.25400	24.48800
H	-29.63200	8.05400	24.69400
H	-29.06200	7.26500	23.53400
O	-35.88000	9.08700	12.41000
H	-35.90300	9.18600	11.45800
H	-35.57000	9.93300	12.73200
O	-33.22100	11.14400	16.52400
H	-32.26700	11.11300	16.45700
H	-33.42000	10.68300	17.34000
O	-32.63100	9.71500	19.09200
H	-31.81200	10.20900	19.11500
H	-32.35700	8.79800	19.06300
O	-22.87400	2.10600	8.45400
H	-23.76200	2.39100	8.23600
H	-22.45900	1.94500	7.60700
O	-19.52300	2.13000	12.17000
H	-18.74700	2.62000	12.44200
H	-20.24800	2.55600	12.62700
O	-17.08600	2.84300	13.22700
H	-16.49100	2.60300	12.51700
H	-17.01300	2.12400	13.85500
O	-34.86600	16.68600	13.54200
H	-35.00100	15.92600	12.97500
H	-35.28300	17.41000	13.07400
O	-23.73000	11.87000	2.54900
H	-23.02600	12.07500	3.16500
H	-24.45400	12.43800	2.81400
O	-22.90000	7.62100	8.66900
H	-22.95700	8.07800	7.83000
H	-22.34700	6.86000	8.49200
O	-19.09000	3.41000	9.49400
H	-19.34600	3.01000	10.32500
H	-19.71500	4.12400	9.36900
O	-14.77900	6.15000	12.00200
H	-15.33900	6.21900	12.77500
H	-15.38400	5.97400	11.28100
O	-31.70900	17.58300	13.75300
H	-31.82600	18.52800	13.65100
H	-32.53000	17.28100	14.14200
O	-21.82100	12.30800	4.59900
H	-20.98700	11.87600	4.41700
H	-21.98300	12.13700	5.52700
O	-22.16700	8.81200	5.96500
H	-22.22200	8.64600	5.02300
H	-22.79200	9.51900	6.12100
O	-37.37800	4.04800	19.79100
H	-36.73200	3.53800	20.28000
H	-37.58600	4.78500	20.36500
O	-24.85200	11.11600	25.29000
H	-23.98300	11.29000	24.92900
H	-25.33200	11.93600	25.16800
O	-28.85700	-1.68800	5.54300
H	-28.86900	-0.74300	5.70000
H	-27.96500	-1.87100	5.24900
O	-30.68400	18.23600	16.96900
H	-29.78100	18.26800	17.28600
H	-30.91000	17.30600	16.98400
O	-28.92800	16.98900	13.63000
H	-29.87900	16.98900	13.51600
H	-28.62500	16.24200	13.11400
O	-25.04200	-5.09300	9.79800
H	-25.80900	-5.64800	9.94300

H	-24.63100	-5.02100	10.65900
O	-33.03800	1.19500	23.88800
H	-32.17500	0.83900	24.09900
H	-33.61100	0.85600	24.57600
O	-13.19300	22.82500	14.69500
H	-12.55500	22.70600	13.99100
H	-13.31100	23.77300	14.75300
O	-16.38400	22.77800	14.63100
H	-16.72200	22.66200	13.74300
H	-15.51500	22.37700	14.61100
O	-20.52500	24.63200	10.62000
H	-21.08900	24.54100	9.85200
H	-20.64000	23.81200	11.10100
O	-15.90200	23.93100	20.58900
H	-15.91300	23.53700	19.71700
H	-15.74000	23.19800	21.18300
O	-22.97900	26.12700	13.45900
H	-22.18700	25.78600	13.04300
H	-22.73900	26.23600	14.37900
O	-14.94500	28.29900	5.93200
H	-15.59700	28.88400	5.54500
H	-14.15100	28.82900	5.99100
O	-21.02000	25.38800	6.33100
H	-20.24700	24.84000	6.19200
H	-20.66700	26.25200	6.54200
O	-19.18100	26.71500	19.53700
H	-19.47200	27.62000	19.42200
H	-18.54600	26.75700	20.25200
O	-18.25600	24.09300	16.12700
H	-17.65900	23.86800	15.41300
H	-18.19800	23.35100	16.73000
O	-18.68300	21.54700	3.96200
H	-18.43100	21.02500	3.20100
H	-19.62100	21.38300	4.06500
O	-13.63900	20.55000	11.77000
H	-12.85300	20.13200	11.41900
H	-13.37500	21.45500	11.93800
O	-13.46800	25.98800	6.46900
H	-14.10300	26.06100	7.18100
H	-13.79800	26.57700	5.79100
O	-8.23800	20.48800	12.67300
H	-7.50500	19.91800	12.90700
H	-8.99400	20.10000	13.11400
O	-18.96400	23.45000	6.16500
H	-19.08000	22.77400	6.83300
H	-18.85300	22.96300	5.34800
O	-16.62000	22.91100	9.86900
H	-16.98500	22.38400	10.58100
H	-15.76400	22.52100	9.69600
O	-10.71500	14.20000	17.13300
H	-11.04700	15.07000	16.90900
H	-11.31100	13.88400	17.81200
O	-16.98000	17.94000	4.86600
H	-16.81200	18.80800	5.23300
H	-17.17000	18.10200	3.94200
O	-14.22200	26.40100	16.53200
H	-14.52200	27.30400	16.42400
H	-14.56700	26.13500	17.38400
O	-20.77100	22.85300	12.56600
H	-21.69600	22.72000	12.77400
H	-20.52300	23.63200	13.06300
O	-10.82300	18.92000	14.21200
H	-11.73100	18.85100	13.91800
H	-10.87400	19.39700	15.04000
O	-17.00100	20.39500	5.85000
H	-17.12100	20.84100	6.68900
H	-17.69600	20.74400	5.29200
O	-23.41000	22.49600	13.40000
H	-23.28100	22.75000	14.31400
H	-24.09000	23.08900	13.08100
O	-19.24000	33.16600	9.49900
H	-19.31800	34.11700	9.57800
H	-19.84400	32.82000	10.15700
O	-16.74800	20.33900	22.15500
H	-17.52500	20.89800	22.14600
H	-16.10900	20.82400	22.67700
O	-8.95600	17.12100	15.99000
H	-9.59900	17.41500	15.34400
H	-9.46100	17.00700	16.79500
O	-11.30200	20.86800	6.67800
H	-10.39100	21.10000	6.50300
H	-11.81100	21.45100	6.11600
O	-18.49400	23.14700	21.71700
H	-17.75200	23.68600	21.44100
H	-19.20000	23.77400	21.87400
O	-13.01800	25.29000	20.31400
H	-12.61000	24.84800	19.57000
H	-13.74100	24.71500	20.56800
O	-16.66500	20.22700	17.68800
H	-15.81900	20.65100	17.83100
H	-16.72500	19.57100	18.38200
O	-12.69500	25.85000	14.07000
H	-13.32500	26.01500	13.36700
H	-13.08300	26.26400	14.84100
O	-8.81700	26.78600	11.42400
H	-9.50100	27.16500	10.87400
H	-8.73200	27.39500	12.15700
O	-13.51500	17.88800	19.34600
H	-13.75900	18.64200	18.80900
H	-13.23900	18.26600	20.18100
O	-11.56100	24.43200	18.02400
H	-11.35000	25.07500	17.34700
H	-11.60800	23.59800	17.55600
O	-13.95900	17.04300	14.92700
H	-14.82000	17.34100	15.21900
H	-13.93300	17.26000	13.99500
O	-17.14300	25.58800	11.33500
H	-16.67900	25.86600	10.54500
H	-17.40700	24.68600	11.15200
O	-20.25000	25.13500	14.27300
H	-19.68000	25.56300	13.63400
H	-19.65900	24.85300	14.97100
O	-18.20600	21.71000	8.14400
H	-17.50300	22.16200	8.61100
H	-18.53800	21.06800	8.77200
O	-19.75800	20.30100	22.51900
H	-20.38800	20.98300	22.75200
H	-19.86400	20.18900	21.57400

O	-24.46300	19.96800	13.44500
H	-25.25300	20.22400	12.96900
H	-23.97200	20.78300	13.55100
O	-10.44700	27.30900	16.19600
H	-9.62500	26.90700	15.91400
H	-10.45100	27.20600	17.14700
O	-22.51200	20.33300	10.40400
H	-23.30900	20.44500	10.92200
H	-22.73900	20.67100	9.53800
O	-16.68100	26.85700	18.35200
H	-16.61700	27.31900	19.18800
H	-17.39000	26.22700	18.48000
O	-26.17900	20.22100	15.87700
H	-25.92900	21.10600	16.14300
H	-25.64300	20.04200	15.10400
O	-2.35600	23.65300	12.54400
H	-3.11000	23.08300	12.38900
H	-2.22100	23.61900	13.49100
O	-16.96600	28.52400	8.22000
H	-17.10200	29.36900	8.64800
H	-16.42500	28.72600	7.45600
O	-20.48000	14.60300	24.88100
H	-20.87300	14.05000	25.55600
H	-19.66300	14.91400	25.27200
O	-23.51300	26.04200	8.79300
H	-23.00100	26.83600	8.94300
H	-22.86100	25.34400	8.72600
O	-21.70400	29.15800	10.87500
H	-21.52700	30.08000	10.68700
H	-20.90800	28.84400	11.30400
O	-17.56300	16.01900	22.47300
H	-16.82200	16.62000	22.39400
H	-17.33100	15.27700	21.91500
O	-22.41900	21.20500	20.75100
H	-22.53300	20.30300	21.05200
H	-23.27400	21.45100	20.40000
O	-15.34000	29.14600	22.35000
H	-15.78300	28.36000	22.03200
H	-14.67800	29.33400	21.68400
O	-20.58100	30.67600	16.87600
H	-20.03000	30.54800	16.10400
H	-21.42000	30.97500	16.52500
O	-13.24100	29.42700	20.37800
H	-13.26000	30.24800	19.88600
H	-12.31900	29.30200	20.60300
O	-16.78400	26.94100	21.22000
H	-16.25100	26.18000	20.99000
H	-17.31300	26.64800	21.96300
O	-13.61700	12.04700	16.75000
H	-13.96100	12.93700	16.67400
H	-13.24900	11.85700	15.88700
O	-25.54900	24.37000	9.56900
H	-24.71100	24.78000	9.35200
H	-25.37100	23.43000	9.53900
O	-13.65200	22.03800	5.25700
H	-14.16700	22.80800	5.49600
H	-13.92400	21.36400	5.88000
O	-16.06700	16.88300	10.31600
H	-16.92300	16.49400	10.13600
H	-15.65500	16.96200	9.45500
O	-14.53700	14.41800	14.71500
H	-15.04100	14.38100	13.90100
H	-14.24400	15.32700	14.77400
O	-18.29300	14.50100	13.72100
H	-17.42800	14.30600	13.36200
H	-18.22500	14.27200	14.64800
O	-8.67900	24.79100	7.19200
H	-9.45600	24.45200	6.74800
H	-8.00000	24.79600	6.51800
O	-13.86300	19.91100	7.08300
H	-14.16800	20.22500	7.93400
H	-12.91000	19.98900	7.12600
O	-15.41900	26.07000	8.73200
H	-15.94700	25.33500	8.42000
H	-15.92000	26.84700	8.48600
O	-10.44000	24.26500	13.84900
H	-11.09700	24.95400	13.93700
H	-9.63600	24.65400	14.19200
O	-20.68700	16.73200	11.09300
H	-20.11300	16.50900	10.36000
H	-20.10000	16.80000	11.84600
O	-11.62900	22.61900	11.89900
H	-11.11600	23.25200	12.40200
H	-10.97900	22.01900	11.53400
O	-20.36000	18.76200	5.11300
H	-21.19600	19.10000	5.43300
H	-20.50000	18.62700	4.17500
O	-18.38500	13.60600	9.82000
H	-19.03800	13.29100	9.19600
H	-18.42700	12.98300	10.54600
O	-17.27400	23.88800	1.73600
H	-18.18100	23.65700	1.93500
H	-16.77600	23.09100	1.92300
O	-14.36700	32.59700	4.65500
H	-15.04100	32.90100	5.26400
H	-13.98000	33.40000	4.30600
O	-15.15100	20.09400	14.14500
H	-16.06900	20.31500	13.98700
H	-14.73500	20.16900	13.28600
O	-14.36700	21.49300	9.27500
H	-13.62000	22.00500	8.96600
H	-14.09800	21.17100	10.13600
O	-16.09500	23.98900	6.25800
H	-17.00400	23.70500	6.35500
H	-16.06500	24.40900	5.39800
O	-10.86300	27.60000	10.02700
H	-11.31700	26.75900	10.08400
H	-11.49300	28.23600	10.36500
O	-11.92000	18.52900	11.25700
H	-12.65700	18.01700	11.58800
H	-11.66700	18.08900	10.44600
O	-12.42000	29.43700	10.96200
H	-12.12700	29.99800	10.24300
H	-11.91000	29.73000	11.71800
O	-11.81800	11.30600	11.85200
H	-11.51200	10.44600	11.56400
H	-11.09300	11.89800	11.65100
O	-25.52600	21.70900	10.24000

H	-25.72300	21.11600	9.51500
H	-26.08100	21.40500	10.95700
O	-17.54600	21.60200	12.35900
H	-17.49100	20.64900	12.28300
H	-18.35700	21.75800	12.84300
O	-16.39700	25.53000	3.59700
H	-16.83500	24.81300	3.13800
H	-15.65600	25.75700	3.03500
O	-19.60700	20.21900	10.11800
H	-20.42000	20.51900	10.52500
H	-19.11100	19.82500	10.83600
O	-17.45600	30.21500	12.05200
H	-17.49900	30.44100	11.12200
H	-16.54000	29.98200	12.20000
O	-14.01000	20.68400	18.95000
H	-13.65500	20.91100	19.80900
H	-13.36200	21.01600	18.32700
O	-8.28000	23.80600	9.65500
H	-8.41800	23.99600	8.72700
H	-8.63600	24.56700	10.11100
O	-20.11600	17.79100	15.73400
H	-19.47600	17.65000	15.03700
H	-20.94800	17.50500	15.35800
O	-8.25300	25.87300	14.66600
H	-7.81900	25.78100	15.51400
H	-7.54000	25.84900	14.02800
O	-19.34000	27.09200	11.84800
H	-18.54600	26.59700	11.64600
H	-20.05000	26.56300	11.48500
O	-14.52000	29.47300	16.19900
H	-14.46600	30.04200	16.96700
H	-15.38900	29.64400	15.83400
O	-23.03100	21.23600	7.92400
H	-23.96200	21.17400	8.14100
H	-22.93600	20.71300	7.12700
O	-23.11300	17.76100	10.51200
H	-22.85200	18.67600	10.40300
H	-22.29100	17.29400	10.66100
O	-21.64000	19.17500	17.68100
H	-20.96800	18.96700	17.03300
H	-22.46700	18.97700	17.24200
O	-7.39100	25.91600	17.19900
H	-7.19800	26.82000	17.44500
H	-7.49100	25.45600	18.03300
O	-15.73800	15.80100	19.69800
H	-16.38500	16.50000	19.60400
H	-14.96800	16.23500	20.06600
O	-12.33000	26.06600	22.77300
H	-12.48100	25.83700	21.85600
H	-12.33900	27.02300	22.78600
O	-15.56100	23.63900	17.75800
H	-14.63200	23.79100	17.58500
H	-15.98200	23.72800	16.90300
O	-16.76200	27.66400	15.52800
H	-16.90100	27.29600	16.40000
H	-17.55100	28.17900	15.35600
O	-16.16800	32.76500	6.87000
H	-15.99200	32.29400	7.68500
H	-16.85400	33.39200	7.09800
O	-15.21200	27.04300	12.91600
H	-15.54700	27.00300	13.81200
H	-15.71200	26.37700	12.44300
O	-21.23800	22.44900	18.44200
H	-21.37600	21.71700	19.04300
H	-20.32700	22.36000	18.16000
O	-12.27600	24.38800	3.19000
H	-12.89300	23.66300	3.09300
H	-12.74400	25.14900	2.84700
O	-18.60900	22.16100	17.93000
H	-17.89900	21.52100	17.88900
H	-18.65200	22.41300	18.85300
O	-10.62100	29.09800	20.85100
H	-10.67900	28.41400	20.18400
H	-10.21900	29.84000	20.39900
O	-19.89600	19.14800	19.75000
H	-20.43300	18.69700	20.40200
H	-20.48500	19.28300	19.00700
O	-21.60200	35.91400	13.64400
H	-21.36200	36.54600	14.32200
H	-22.44500	35.56300	13.93100
O	-20.48600	18.60700	2.31200
H	-21.36300	18.48600	1.94900
H	-20.01000	17.81500	2.06000
O	-10.61900	27.06200	19.08200
H	-11.50600	26.72500	19.20500
H	-10.04900	26.33000	19.31900
O	-28.96600	23.39400	12.40600
H	-28.27900	23.08100	11.81900
H	-29.12100	24.29900	12.13300
O	-24.16200	18.28400	16.83500
H	-24.24700	17.67600	16.10000
H	-24.98400	18.77400	16.83600
O	-17.08900	31.72200	16.04800
H	-17.16200	32.52800	16.56000
H	-17.53100	31.92100	15.22300
O	-17.30000	30.95900	9.55900
H	-18.08300	31.50200	9.65200
H	-16.60000	31.47700	9.95800
O	-13.99000	16.76900	12.20200
H	-13.49300	16.01600	11.88200
H	-14.76100	16.80500	11.63600
O	-6.13700	22.47300	13.84400
H	-6.14800	23.35500	13.47300
H	-7.00500	22.12000	13.64900
O	-14.62900	31.62100	18.20400
H	-13.91600	32.25600	18.13200
H	-15.12000	31.71700	17.38800
O	-12.90700	25.52600	9.77600
H	-13.84100	25.64600	9.60600
H	-12.72900	24.62300	9.51200
O	-17.40700	18.91100	11.92900
H	-17.77700	18.35800	12.61700
H	-16.91100	18.30600	11.37700
O	-9.52000	21.36800	10.31100
H	-9.05400	22.20100	10.23700
H	-9.06900	20.90200	11.01500
O	-19.57800	29.37000	19.02500
H	-18.87700	30.00400	19.17800

H	-20.04800	29.71200	18.26400
O	-11.45200	27.71200	7.30900
H	-11.17600	27.59800	8.21900
H	-12.32200	27.31400	7.27100
O	-11.54800	13.73600	14.35000
H	-12.46200	13.96600	14.51800
H	-11.11900	13.82000	15.20100
O	-11.77800	21.92500	16.85000
H	-12.43000	22.17200	16.19500
H	-11.40000	21.11100	16.51600
O	-14.81700	31.96100	10.95300
H	-14.63500	31.14300	11.41500
H	-13.99400	32.17800	10.51500
O	-14.48300	30.88800	8.21400
H	-13.95400	30.37700	7.60100
H	-14.22000	30.57200	9.07900
O	-15.72900	22.66700	23.33600
H	-16.40500	22.56600	24.00700
H	-14.90600	22.51100	23.80000
O	-23.98100	28.90400	15.95800
H	-23.39600	28.15800	16.09100
H	-24.17800	29.21400	16.84100
O	-21.12700	31.93900	10.97400
H	-21.79600	32.42400	10.49000
H	-21.26600	32.18600	11.88800
O	-29.38900	21.47500	14.67800
H	-29.35100	20.94100	15.47200
H	-29.89500	20.94800	14.05900
O	-16.98700	33.97300	17.57200
H	-16.43500	34.13700	18.33700
H	-16.64600	34.56400	16.90000
O	-22.01000	16.32100	18.17400
H	-21.94600	17.18200	17.76200
H	-22.07300	15.70800	17.44200
O	-22.53500	18.39300	21.82200
H	-21.90800	18.01100	22.43600
H	-23.39200	18.17500	22.18700
O	-12.13500	28.80000	23.42700
H	-11.59100	29.41300	22.93300
H	-12.71700	29.35800	23.94400
O	-25.75800	20.20100	7.75400
H	-25.46300	19.29200	7.70000
H	-26.65900	20.18000	7.43200
O	-13.85000	31.92200	14.55900
H	-13.97500	31.12100	14.05000
H	-13.19200	32.41600	14.07000
O	-22.22900	18.27700	13.73600
H	-22.95000	18.89000	13.59700
H	-21.44000	18.81200	13.65800
O	-11.78600	29.72300	16.18700
H	-12.73300	29.60300	16.11400
H	-11.43800	28.83500	16.26900
O	-19.42600	15.61200	20.01000
H	-18.90300	15.85800	20.77300
H	-19.64500	16.44400	19.59100
O	-24.03000	17.88200	7.87800
H	-24.35800	17.00200	7.69500
H	-23.67600	17.82700	8.76500
O	-14.78200	29.51800	12.38700
H	-14.92900	28.59000	12.56800
H	-13.95600	29.54000	11.90300
O	-20.91500	18.17000	8.24500
H	-20.57600	18.69800	7.52300
H	-20.46800	18.50800	9.02100
O	-11.17800	30.34100	13.47400
H	-10.25300	30.13500	13.33600
H	-11.34700	30.09200	14.38200
O	-18.22100	16.55300	17.69500
H	-18.56100	15.75400	18.09700
H	-18.92700	16.85400	17.12300
O	-17.56900	13.63800	16.14800
H	-16.74700	13.16400	16.02600
H	-17.32600	14.41700	16.64800
O	-18.92900	16.66700	6.44400
H	-18.20800	17.04500	5.94100
H	-19.55200	17.38600	6.54800
O	-14.78500	17.37200	7.38000
H	-14.56900	18.25400	7.07900
H	-15.26600	16.98000	6.65100
O	-20.04500	20.33000	13.95700
H	-20.19100	20.64400	14.85000
H	-20.52300	20.94900	13.40400
O	-18.18600	17.32000	14.00500
H	-18.20000	16.42500	13.66900
H	-17.46900	17.32800	14.63900
O	-16.33300	17.75400	16.05100
H	-16.59100	18.67600	16.04900
H	-16.86500	17.35700	16.74100
O	-11.38900	30.85900	8.83000
H	-11.21500	30.29400	8.07700
H	-10.89600	31.65900	8.64800
O	-25.57100	22.87100	16.89600
H	-26.11600	23.64700	17.02800
H	-24.69300	23.21800	16.73800
O	-18.78200	16.20200	9.27400
H	-18.66100	15.27400	9.47300
H	-19.07200	16.21600	8.36200
O	-15.91300	13.88700	12.41100
H	-15.89100	13.95100	11.45600
H	-15.49600	13.04800	12.60500
O	-5.53500	23.62300	10.44900
H	-5.09200	22.87400	10.05100
H	-6.41200	23.61400	10.06600
O	-10.40100	30.17600	5.92900
H	-9.70600	30.79700	6.14800
H	-10.05900	29.32600	6.20500
O	-24.78400	21.99800	19.78900
H	-25.52600	22.14600	20.37600
H	-25.10500	22.25900	18.92600
O	-8.50200	27.62600	7.45800
H	-7.97700	27.96500	6.73300
H	-8.59000	26.69100	7.27400
O	-16.84000	31.53900	19.58700
H	-16.53100	31.29400	20.45900
H	-16.04200	31.74400	19.09900
O	-16.81800	33.69300	11.81800
H	-17.25700	33.20100	12.51100
H	-16.06700	33.15000	11.57700

O	-24.33900	29.88400	11.82000
H	-23.51300	29.58500	11.44000
H	-24.68900	29.11500	12.26900
O	-19.37000	29.23700	13.89000
H	-19.99200	28.57400	13.59000
H	-18.70000	29.26300	13.20700
O	-26.13800	27.80300	12.77900
H	-26.97500	27.37500	12.59700
H	-26.16500	27.99600	13.71600
O	-19.29800	17.71500	23.48300
H	-19.24900	18.59100	23.10100
H	-18.75600	17.17300	22.91000
O	-8.81300	28.25900	22.76300
H	-9.60300	28.24200	22.22400
H	-9.04900	28.79800	23.51800
O	-8.69300	25.10800	20.07700
H	-8.64300	24.94300	21.01800
H	-8.80100	24.24100	19.68600
O	-19.80900	27.78700	7.36600
H	-19.66000	28.47100	6.71200
H	-19.00200	27.76800	7.88100
O	-11.40200	21.09200	21.39200
H	-10.50100	20.95200	21.10100
H	-11.32800	21.25700	22.33200
O	-17.06800	18.46800	20.23800
H	-17.93800	18.76200	19.96600
H	-16.83200	19.05200	20.95900
O	-7.19100	27.52800	20.32100
H	-7.48200	27.89100	21.15700
H	-7.80400	26.81500	20.14200
O	-5.99100	25.09300	12.84100
H	-5.15700	25.53200	13.00500
H	-5.85500	24.61700	12.02200
O	-8.94200	22.65100	18.78900
H	-8.46700	22.84400	17.98100
H	-9.86600	22.70700	18.54300
O	-22.05400	23.81400	8.37200
H	-22.29900	22.91000	8.57000
H	-21.88400	23.81600	7.43000
O	-14.36400	14.68600	17.75400
H	-13.84600	15.42200	17.42900
H	-14.71900	14.99300	18.58900
O	-22.78400	19.54700	5.87000
H	-23.47700	19.62100	5.21400
H	-23.02900	18.78300	6.39300
O	-14.11300	11.10600	13.27400
H	-13.83300	10.43900	13.90000
H	-13.32800	11.29900	12.76200
O	-16.31000	30.28200	4.83700
H	-16.88200	30.90100	4.38400
H	-15.73600	30.83200	5.37100
O	-10.24200	19.52800	17.02200
H	-9.29000	19.62800	17.04300
H	-10.41200	18.73600	17.53100
O	-12.22100	16.61400	17.20800
H	-12.81600	16.89400	16.51200
H	-12.50600	17.10300	17.97900
O	-13.09000	30.56600	5.96800
H	-12.15700	30.50000	6.17100
H	-13.17800	31.40100	5.50900
O	-10.46100	29.88200	2.92800
H	-10.54000	29.54800	3.82200
H	-9.64000	30.37500	2.92700
O	-7.76300	22.74900	16.13600
H	-7.79600	21.81000	16.32200
H	-7.15300	22.82700	15.40200
O	-15.92500	21.61800	2.17900
H	-14.97900	21.48900	2.24700
H	-16.30300	20.88000	2.65500
O	-7.61400	19.54000	16.18200
H	-8.13000	18.75600	15.99500
H	-6.73000	19.21200	16.34600
O	-9.95300	13.28400	12.14100
H	-10.15000	12.97500	13.02600
H	-9.34300	14.01000	12.27100
O	-1.94600	23.81000	15.27600
H	-1.21500	23.54900	15.83700
H	-2.55500	23.07300	15.31700
O	-3.86400	21.95100	15.87400
H	-4.59100	21.81700	15.26500
H	-4.27900	22.25900	16.68000
O	-16.04100	25.67000	23.63900
H	-15.12100	25.84800	23.83300
H	-16.07600	24.72600	23.48700
O	-17.74100	19.54300	2.11400
H	-18.64700	19.24700	2.19000
H	-17.46300	19.24600	1.24700
O	-26.82300	20.86400	12.56400
H	-27.14300	20.01000	12.27100
H	-27.36900	21.07900	13.32000
O	-23.04500	23.58000	16.28100
H	-22.82600	24.51200	16.30000
H	-22.63200	23.22100	17.06600
O	-16.12400	14.08700	8.26800
H	-16.93400	13.74200	8.64400
H	-15.43600	13.77800	8.85700
O	-14.19500	13.16500	9.89100
H	-13.64200	13.58400	10.55000
H	-13.95800	12.23800	9.93300
O	-18.17200	19.15300	25.67100
H	-18.27200	20.10300	25.60200
H	-18.39000	18.82500	24.79900
O	-18.97100	16.40200	2.25500
H	-18.10900	16.72700	2.51500
H	-18.78600	15.61500	1.74300
O	-26.95400	24.48400	14.53300
H	-27.64800	24.07000	14.02000
H	-26.20700	24.52600	13.93700
O	-10.81700	15.27500	9.39900
H	-11.39800	15.93300	9.01700
H	-11.08400	14.45300	8.98700
O	-12.36700	14.67500	11.60100
H	-11.86700	14.78700	10.79200
H	-11.72400	14.37100	12.24100
O	-18.30900	22.58300	24.48800
H	-18.79200	23.38300	24.69400
H	-18.22300	22.59400	23.53400
O	-25.04000	24.41600	12.41000

H	-25.06300	24.51500	11.45800
H	-24.73000	25.26200	12.73200
O	-22.38200	26.47400	16.52400
H	-21.42700	26.44200	16.45700
H	-22.58000	26.01300	17.34000
O	-21.79200	25.04400	19.09200
H	-20.97200	25.53800	19.11500
H	-21.51800	24.12700	19.06300
O	-12.03500	17.43500	8.45400
H	-12.92200	17.72000	8.23600
H	-11.62000	17.27400	7.60700
O	-8.68400	17.45900	12.17000
H	-7.90800	17.94900	12.44200
H	-9.40900	17.88500	12.62700
O	-6.24600	18.17200	13.22700
H	-5.65200	17.93200	12.51700
H	-6.17400	17.45300	13.85500
O	-24.02700	32.01600	13.54200
H	-24.16100	31.25600	12.97500
H	-24.44300	32.74000	13.07400
O	-12.89100	27.19900	2.54900
H	-12.18700	27.40400	3.16500
H	-13.61400	27.76700	2.81400
O	-12.06100	22.95000	8.66900
H	-12.11700	23.40700	7.83000
H	-11.50800	22.18900	8.49200
O	-8.25000	18.73900	9.49400
H	-8.50700	18.33900	10.32500
H	-8.87500	19.45400	9.36900
O	-3.93900	21.47900	12.00200
H	-4.49900	21.54800	12.77500
H	-4.54500	21.30300	11.28100
O	-20.87000	32.91200	13.75300
H	-20.98600	33.85700	13.65100
H	-21.69000	32.61000	14.14200
O	-10.98200	27.63700	4.59900
H	-10.14700	27.20600	4.41700
H	-11.14400	27.46600	5.52700
O	-11.32700	24.14100	5.96500
H	-11.38300	23.97500	5.02300
H	-11.95300	24.84800	6.12100
O	-26.53800	19.37800	19.79100
H	-25.89200	18.86700	20.28000
H	-26.74600	20.11400	20.36500
O	-14.01300	26.44600	25.29000
H	-13.14400	26.62000	24.92900
H	-14.49200	27.26500	25.16800
O	-18.01800	13.64200	5.54300
H	-18.03000	14.58600	5.70000
H	-17.12500	13.45900	5.24900
O	-19.84400	33.56500	16.96900
H	-18.94100	33.59800	17.28600
H	-20.07000	32.63500	16.98400
O	-18.08900	32.31800	13.63000
H	-19.03900	32.31900	13.51600
H	-17.78600	31.57200	13.11400
O	-14.20300	10.23600	9.79800
H	-14.96900	9.68200	9.94300
H	-13.79200	10.30800	10.65900
O	-22.19800	16.52400	23.88800
H	-21.33500	16.16800	24.09900
H	-22.77200	16.18600	24.57600
O	-2.35400	38.15400	14.69500
H	-1.71600	38.03500	13.99100
H	-2.47100	39.10300	14.75300
O	-5.54500	38.10700	14.63100
H	-5.88300	37.99100	13.74300
H	-4.67600	37.70600	14.61100
O	-9.68600	39.96100	10.62000
H	-10.24900	39.87000	9.85200
H	-9.80100	39.14100	11.10100
O	-5.06300	39.26100	20.58900
H	-5.07400	38.86600	19.71700
H	-4.90100	38.52700	21.18300
O	-10.18000	40.71700	6.33100
H	-9.40700	40.17000	6.19200
H	-9.82800	41.58100	6.54200
O	-7.41600	39.42200	16.12700
H	-6.82000	39.19800	15.41300
H	-7.35900	38.68100	16.73000
O	-7.84400	36.87600	3.96200
H	-7.59100	36.35400	3.20100
H	-8.78100	36.71300	4.06500
O	-2.79900	35.88000	11.77000
H	-2.01300	35.46200	11.41900
H	-2.53600	36.78400	11.93800
O	-2.62800	41.31700	6.46900
H	-3.26400	41.39000	7.18100
H	-2.95900	41.90600	5.79100
O	2.60100	35.81700	12.67300
H	3.33400	35.24700	12.90700
H	1.84500	35.42900	13.11400
O	-8.12400	38.77900	6.16500
H	-8.24100	38.10300	6.83300
H	-8.01400	38.29200	5.34800
O	-5.78100	38.24000	9.86900
H	-6.14500	37.71300	10.58100
H	-4.92400	37.85000	9.69600
O	0.12400	29.52900	17.13300
H	-0.20800	30.39900	16.90900
H	-0.47200	29.21300	17.81200
O	-6.14000	33.26900	4.86600
H	-5.97300	34.13700	5.23300
H	-6.33100	33.43200	3.94200
O	-3.38300	41.73100	16.53200
H	-3.68300	42.63300	16.42400
H	-3.72800	41.46400	17.38400
O	-9.93200	38.18200	12.56600
H	-10.85700	38.05000	12.77400
H	-9.68300	38.96100	13.06300
O	0.01700	34.24900	14.21200
H	-0.89200	34.18000	13.91800
H	-0.03400	34.72600	15.04000
O	-6.16200	35.72500	5.85000
H	-6.28200	36.17000	6.68900
H	-6.85600	36.07300	5.29200
O	-12.57100	37.82500	13.40000
H	-12.44200	38.07900	14.31400

H	-13.25100	38.41800	13.08100
O	-5.90900	35.66800	22.15500
H	-6.68600	36.22700	22.14600
H	-5.27000	36.15300	22.67700
O	1.88300	32.45000	15.99000
H	1.24000	32.74400	15.34400
H	1.37800	32.33600	16.79500
O	-0.46300	36.19700	6.67800
H	0.44900	36.42900	6.50300
H	-0.97200	36.78000	6.11600
O	-7.65400	38.47600	21.71700
H	-6.91300	39.01500	21.44100
H	-8.36100	39.10300	21.87400
O	-2.17900	40.61900	20.31400
H	-1.77100	40.17700	19.57000
H	-2.90100	40.04400	20.56800
O	-5.82600	35.55600	17.68800
H	-4.98000	35.98000	17.83100
H	-5.88600	34.90000	18.38200
O	-1.85600	41.17900	14.07000
H	-2.48500	41.34400	13.36700
H	-2.24300	41.59400	14.84100
O	2.02300	42.11500	11.42400
H	1.33800	42.49500	10.87400
H	2.10700	42.72400	12.15700
O	-2.67600	33.21700	19.34600
H	-2.92000	33.97100	18.80900
H	-2.40000	33.59500	20.18100
O	-0.72200	39.76100	18.02400
H	-0.51000	40.40400	17.34700
H	-0.76900	38.92700	17.55600
O	-3.11900	32.37300	14.92700
H	-3.98100	32.67100	15.21900
H	-3.09300	32.59000	13.99500
O	-6.30300	40.91700	11.33500
H	-5.83900	41.19600	10.54500
H	-6.56800	40.01500	11.15200
O	-9.41100	40.46400	14.27300
H	-8.84100	40.89300	13.63400
H	-8.82000	40.18200	14.97100
O	-7.36600	37.03900	8.14400
H	-6.66400	37.49100	8.61100
H	-7.69800	36.39700	8.77200
O	-8.91800	35.63000	22.51900
H	-9.54800	36.31200	22.75200
H	-9.02500	35.51900	21.57400
O	-13.62400	35.29700	13.44500
H	-14.41400	35.55300	12.96900
H	-13.13300	36.11200	13.55100
O	0.39300	42.63800	16.19600
H	1.21400	42.23600	15.91400
H	0.38900	42.53500	17.14700
O	-11.67200	35.66200	10.40400
H	-12.46900	35.77400	10.92200
H	-11.90000	36.00100	9.53800
O	-5.84200	42.18600	18.35200
H	-5.77700	42.64800	19.18800
H	-6.55100	41.55600	18.48000
O	-15.34000	35.55000	15.87700
H	-15.08900	36.43500	16.14300
H	-14.80400	35.37100	15.10400
O	8.48300	38.98200	12.54400
H	7.73000	38.41300	12.38900
H	8.61800	38.94800	13.49100
O	-9.64000	29.93200	24.88100
H	-10.03400	29.37900	25.55600
H	-8.82400	30.24300	25.27200
O	-6.72300	31.34800	22.47300
H	-5.98300	31.94900	22.39400
H	-6.49200	30.60600	21.91500
O	-11.57900	36.53400	20.75100
H	-11.69400	35.63200	21.05200
H	-12.43500	36.78000	20.40000
O	-5.94500	42.27000	21.22000
H	-5.41200	41.50900	20.99000
H	-6.47300	41.97800	21.96300
O	-2.77800	27.37600	16.75000
H	-3.12100	28.26600	16.67400
H	-2.41000	27.18600	15.88700
O	-14.70900	39.70000	9.56900
H	-13.87200	40.10900	9.35200
H	-14.53100	38.76000	9.53900
O	-2.81300	37.36700	5.25700
H	-3.32800	38.13800	5.49600
H	-3.08500	36.69400	5.88000
O	-5.22800	32.21200	10.31600
H	-6.08400	31.82300	10.13600
H	-4.81500	32.29100	9.45500
O	-3.69800	29.74700	14.71500
H	-4.20200	29.71000	13.90100
H	-3.40500	30.65600	14.77400
O	-7.45400	29.83100	13.72100
H	-6.58900	29.63500	13.36200
H	-7.38600	29.60200	14.64800
O	2.16100	40.12000	7.19200
H	1.38400	39.78100	6.74800
H	2.84000	40.12600	6.51800
O	-3.02400	35.24000	7.08300
H	-3.32900	35.55400	7.93400
H	-2.07100	35.31800	7.12600
O	-4.57900	41.39900	8.73200
H	-5.10700	40.66400	8.42000
H	-5.08100	42.17600	8.48600
O	0.40000	39.59400	13.84900
H	-0.25800	40.28400	13.93700
H	1.20400	39.98300	14.19200
O	-9.84800	32.06100	11.09300
H	-9.27300	31.83800	10.36000
H	-9.26000	32.13000	11.84600
O	-0.79000	37.94800	11.89900
H	-0.27600	38.58100	12.40200
H	-0.14000	37.34800	11.53400
O	-9.52100	34.09100	5.11300
H	-10.35700	34.42900	5.43300
H	-9.66000	33.95600	4.17500
O	-7.54500	28.93500	9.82000
H	-8.19900	28.62000	9.19600
H	-7.58700	28.31300	10.54600

O	-6.43400	39.21700	1.73600
H	-7.34100	38.98600	1.93500
H	-5.93700	38.42100	1.92300
O	-4.31200	35.42300	14.14500
H	-5.22900	35.64500	13.98700
H	-3.89600	35.49800	13.28600
O	-3.52800	36.82200	9.27500
H	-2.78100	37.33400	8.96600
H	-3.25900	36.50100	10.13600
O	-5.25500	39.31800	6.25800
H	-6.16400	39.03400	6.35500
H	-5.22600	39.73800	5.39800
O	-0.02300	42.92900	10.02700
H	-0.47700	42.08800	10.08400
H	-0.65400	43.56500	10.36500
O	-1.08000	33.85800	11.25700
H	-1.81800	33.34600	11.58800
H	-0.82700	33.41800	10.44600
O	-0.97900	26.63600	11.85200
H	-0.67300	25.77500	11.56400
H	-0.25400	27.22700	11.65100
O	-14.68700	37.03900	10.24000
H	-14.88400	36.44600	9.51500
H	-15.24200	36.73400	10.95700
O	-6.70700	36.93100	12.35900
H	-6.65200	35.97800	12.28300
H	-7.51800	37.08700	12.84300
O	-5.55800	40.85900	3.59700
H	-5.99600	40.14300	3.13800
H	-4.81700	41.08700	3.03500
O	-8.76800	35.54900	10.11800
H	-9.58100	35.84800	10.52500
H	-8.27200	35.15400	10.83600
O	-3.17000	36.01300	18.95000
H	-2.81500	36.24000	19.80900
H	-2.52300	36.34500	18.32700
O	2.56000	39.13500	9.65500
H	2.42100	39.32500	8.72700
H	2.20300	39.89700	10.11100
O	-9.27600	33.12100	15.73400
H	-8.63600	32.97900	15.03700
H	-10.10800	32.83400	15.35800
O	2.58600	41.20200	14.66600
H	3.02000	41.11000	15.51400
H	3.30000	41.17800	14.02800
O	-8.50000	42.42100	11.84800
H	-7.70600	41.92600	11.64600
H	-9.21100	41.89300	11.48500
O	-12.19200	36.56500	7.92400
H	-13.12200	36.50400	8.14100
H	-12.09600	36.04300	7.12700
O	-12.27300	33.09100	10.51200
H	-12.01300	34.00500	10.40300
H	-11.45200	32.62300	10.66100
O	-10.80100	34.50400	17.68100
H	-10.12900	34.29600	17.03300
H	-11.62800	34.30700	17.24200
O	3.44800	41.24500	17.19900
H	3.64200	42.14900	17.44500
H	3.34800	40.78500	18.03300
O	-4.89800	31.13100	19.69800
H	-5.54600	31.83000	19.60400
H	-4.12800	31.56400	20.06600
O	-1.49000	41.39500	22.77300
H	-1.64200	41.16600	21.85600
H	-1.50000	42.35200	22.78600
O	-4.72200	38.96900	17.75800
H	-3.79300	39.12000	17.58500
H	-5.14300	39.05700	16.90300
O	-4.37300	42.37200	12.91600
H	-4.70800	42.33200	13.81200
H	-4.87200	41.70600	12.44300
O	-10.39800	37.77900	18.44200
H	-10.53600	37.04700	19.04300
H	-9.48800	37.68900	18.16000
O	-1.43700	39.71700	3.19000
H	-2.05400	38.99200	3.09300
H	-1.90500	40.47800	2.84700
O	-7.77000	37.49100	17.93000
H	-7.06000	36.85000	17.88900
H	-7.81300	37.74200	18.85300
O	0.21900	44.42700	20.85100
H	0.16000	43.74300	20.18400
H	0.62100	45.16900	20.39900
O	-9.05700	34.47700	19.75000
H	-9.59300	34.02600	20.40200
H	-9.64500	34.61200	19.00700
O	-9.64700	33.93600	2.31200
H	-10.52400	33.81500	1.94900
H	-9.17100	33.14500	2.06000
O	0.22100	42.39100	19.08200
H	-0.66700	42.05400	19.20500
H	0.79000	41.66000	19.31900
O	-13.32300	33.61300	16.83500
H	-13.40700	33.00500	16.10000
H	-14.14500	34.10400	16.83600
O	-3.15100	32.09800	12.20200
H	-2.65400	31.34500	11.88200
H	-3.92200	32.13400	11.63600
O	4.70200	37.80200	13.84400
H	4.69100	38.68500	13.47300
H	3.83400	37.44900	13.64900
O	-2.06800	40.85500	9.77600
H	-3.00200	40.97500	9.60600
H	-1.89000	39.95200	9.51200
O	-6.56700	34.24000	11.92900
H	-6.93700	33.68700	12.61700
H	-6.07100	33.63600	11.37700
O	1.32000	36.69700	10.31100
H	1.78600	37.53000	10.23700
H	1.77000	36.23100	11.01500
O	-0.61300	43.04100	7.30900
H	-0.33700	42.92700	8.21900
H	-1.48200	42.64300	7.27100
O	-0.70900	29.06500	14.35000
H	-1.62300	29.29500	14.51800
H	-0.28000	29.14900	15.20100
O	-0.93800	37.25400	16.85000

H	-1.59100	37.50200	16.19500
H	-0.56000	36.44000	16.51600
O	-4.88900	37.99600	23.33600
H	-5.56500	37.89500	24.00700
H	-4.06700	37.84000	23.80000
O	-18.55000	36.80400	14.67800
H	-18.51100	36.27000	15.47200
H	-19.05600	36.27700	14.05900
O	-11.17100	31.65000	18.17400
H	-11.10700	32.51200	17.76200
H	-11.23400	31.03800	17.44200
O	-11.69500	33.72300	21.82200
H	-11.06800	33.34000	22.43600
H	-12.55300	33.50400	22.18700
O	-1.29600	44.12900	23.42700
H	-0.75200	44.74200	22.93300
H	-1.87700	44.68700	23.94400
O	-14.91900	35.53000	7.75400
H	-14.62400	34.62100	7.70000
H	-15.82000	35.50900	7.43200
O	-11.38900	33.60600	13.73600
H	-12.11100	34.21900	13.59700
H	-10.60000	34.14200	13.65800
O	-8.58700	30.94100	20.01000
H	-8.06300	31.18700	20.77300
H	-8.80600	31.77300	19.59100
O	-13.19000	33.21200	7.87800
H	-13.51900	32.33100	7.69500
H	-12.83600	33.15600	8.76500
O	-10.07600	33.49900	8.24500
H	-9.73600	34.02700	7.52300
H	-9.62900	33.83700	9.02100
O	-7.38200	31.88300	17.69500
H	-7.72200	31.08300	18.09700
H	-8.08800	32.18300	17.12300
O	-6.73000	28.96800	16.14800
H	-5.90700	28.49400	16.02600
H	-6.48600	29.74700	16.64800
O	-8.08900	31.99600	6.44400
H	-7.36800	32.37400	5.94100
H	-8.71300	32.71500	6.54800
O	-3.94600	32.70100	7.38000
H	-3.73000	33.58300	7.07900
H	-4.42700	32.30900	6.65100
O	-9.20600	35.65900	13.95700
H	-9.35200	35.97300	14.85000
H	-9.68400	36.27800	13.40400
O	-7.34600	32.65000	14.00500
H	-7.36000	31.75400	13.66900
H	-6.62900	32.65700	14.63900
O	-5.49400	33.08300	16.05100
H	-5.75200	34.00500	16.04900
H	-6.02500	32.68600	16.74100
O	-14.73200	38.20000	16.89600
H	-15.27600	38.97600	17.02800
H	-13.85400	38.54700	16.73800
O	-7.94300	31.53100	9.27400
H	-7.82100	30.60300	9.47300
H	-8.23300	31.54500	8.36200
O	-5.07400	29.21700	12.41100
H	-5.05100	29.28100	11.45600
H	-4.65700	28.37700	12.60500
O	5.30500	38.95200	10.44900
H	5.74800	38.20300	10.05100
H	4.42700	38.94300	10.06600
O	-13.94500	37.32700	19.78900
H	-14.68700	37.47500	20.37600
H	-14.26600	37.58800	18.92600
O	2.33700	42.95500	7.45800
H	2.86300	43.29400	6.73300
H	2.25000	42.02000	7.27400
O	-8.45900	33.04400	23.48300
H	-8.41000	33.92100	23.10100
H	-7.91600	32.50200	22.91000
O	2.02700	43.58800	22.76300
H	1.23600	43.57100	22.22400
H	1.79000	44.12700	23.51800
O	2.14700	40.43800	20.07700
H	2.19600	40.27300	21.01800
H	2.03800	39.57000	19.68600
O	-0.56300	36.42100	21.39200
H	0.33800	36.28100	21.10100
H	-0.48800	36.58600	22.33200
O	-6.22900	33.79700	20.23800
H	-7.09800	34.09100	19.96600
H	-5.99300	34.38100	20.95900
O	3.64800	42.85700	20.32100
H	3.35700	43.22000	21.15700
H	3.03600	42.14400	20.14200
O	4.84800	40.42300	12.84100
H	5.68300	40.86200	13.00500
H	4.98400	39.94700	12.02200
O	1.89700	37.98100	18.78900
H	2.37300	38.17300	17.98100
H	0.97400	38.03700	18.54300
O	-11.21500	39.14300	8.37200
H	-11.46000	38.23900	8.57000
H	-11.04500	39.14500	7.43000
O	-3.52500	30.01500	17.75400
H	-3.00700	30.75200	17.42900
H	-3.87900	30.32200	18.58900
O	-11.94500	34.87600	5.87000
H	-12.63800	34.95000	5.21400
H	-12.19000	34.11200	6.39300
O	-3.27400	26.43500	13.27400
H	-2.99300	25.76800	13.90000
H	-2.48900	26.62800	12.76200
O	0.59700	34.85700	17.02200
H	1.54900	34.95700	17.04300
H	0.42800	34.06500	17.53100
O	-1.38100	31.94300	17.20800
H	-1.97600	32.22300	16.51200
H	-1.66600	32.43200	17.97900
O	0.37800	45.21200	2.92800
H	0.30000	44.87800	3.82200
H	1.19900	45.70400	2.92700
O	3.07600	38.07800	16.13600
H	3.04400	37.13900	16.32200

H	3.68600	38.15600	15.40200
O	-5.08600	36.94700	2.17900
H	-4.14000	36.81800	2.24700
H	-5.46400	36.20900	2.65500
O	3.22600	34.86900	16.18200
H	2.70900	34.08500	15.99500
H	4.11000	34.54100	16.34600
O	0.88700	28.61300	12.14100
H	0.68900	28.30500	13.02600
H	1.49700	29.33900	12.27100
O	8.89400	39.13900	15.27600
H	9.62400	38.87800	15.83700
H	8.28400	38.40200	15.31700
O	6.97500	37.28000	15.87400
H	6.24900	37.14600	15.26500
H	6.56000	37.58800	16.68000
O	-5.20200	40.99900	23.63900
H	-4.28100	41.17700	23.83300
H	-5.23700	40.05500	23.48700
O	-6.90100	34.87200	2.11400
H	-7.80800	34.57600	2.19000
H	-6.62400	34.57500	1.24700
O	-15.98300	36.19300	12.56400
H	-16.30300	35.34000	12.27100
H	-16.53000	36.40800	13.32000
O	-12.20500	38.90900	16.28100
H	-11.98700	39.84100	16.30000
H	-11.79300	38.55000	17.06600
O	-5.28500	29.41600	8.26800
H	-6.09500	29.07100	8.64400
H	-4.59700	29.10700	8.85700
O	-3.35600	28.49400	9.89100
H	-2.80300	28.91300	10.55000
H	-3.11900	27.56700	9.93300
O	-7.33300	34.48300	25.67100
H	-7.43300	35.43200	25.60200
H	-7.55100	34.15400	24.79900
O	-8.13100	31.73100	2.25500
H	-7.26900	32.05700	2.51500
H	-7.94600	30.94400	1.74300
O	0.02200	30.60400	9.39900
H	-0.55800	31.26200	9.01700
H	-0.24500	29.78200	8.98700
O	-1.52800	30.00400	11.60100
H	-1.02800	30.11600	10.79200
H	-0.88400	29.70100	12.24100
O	-7.47000	37.91200	24.48800
H	-7.95300	38.71200	24.69400
H	-7.38300	37.92300	23.53400
O	-10.95200	40.37300	19.09200
H	-10.13300	40.86700	19.11500
H	-10.67900	39.45600	19.06300
O	-1.19600	32.76400	8.45400
H	-2.08300	33.04900	8.23600
H	-0.78000	32.60300	7.60700
O	2.15600	32.78900	12.17000
H	2.93200	33.27800	12.44200
H	1.43100	33.21400	12.62700
O	4.59300	33.50100	13.22700
H	5.18800	33.26100	12.51700
H	4.66600	32.78200	13.85500
O	-2.05100	42.52800	2.54900
H	-1.34800	42.73300	3.16500
H	-2.77500	43.09600	2.81400
O	-1.22200	38.27900	8.66900
H	-1.27800	38.73600	7.83000
H	-0.66900	37.51800	8.49200
O	2.58900	34.06800	9.49400
H	2.33300	33.66800	10.32500
H	1.96400	34.78300	9.36900
O	6.90000	36.80800	12.00200
H	6.34000	36.87700	12.77500
H	6.29500	36.63200	11.28100
O	-0.14300	42.96700	4.59900
H	0.69200	42.53500	4.41700
H	-0.30400	42.79500	5.52700
O	-0.48800	39.47000	5.96500
H	-0.54300	39.30400	5.02300
H	-1.11300	40.17800	6.12100
O	-15.69900	34.70700	19.79100
H	-15.05300	34.19700	20.28000
H	-15.90700	35.44400	20.36500
O	-3.17300	41.77500	25.29000
H	-2.30400	41.94900	24.92900
H	-3.65300	42.59400	25.16800
O	-7.17800	28.97100	5.54300
H	-7.19100	29.91500	5.70000
H	-6.28600	28.78800	5.24900
O	-3.36300	25.56500	9.79800
H	-4.13000	25.01100	9.94300
H	-2.95200	25.63700	10.65900
O	-11.35900	31.85300	23.88800
H	-10.49600	31.49700	24.09900
H	-11.93300	31.51500	24.57600
O	10.96300	44.85800	17.13300
H	10.63200	45.72800	16.90900
H	10.36800	44.54200	17.81200
O	12.72200	47.78000	15.99000
H	12.08000	48.07300	15.34400
H	12.21800	47.66500	16.79500
O	1.19900	45.26200	24.88100
H	0.80600	44.70800	25.55600
H	2.01600	45.57300	25.27200
O	8.06200	42.70500	16.75000
H	7.71800	43.59600	16.67400
H	8.43000	42.51600	15.88700
O	7.14100	45.07600	14.71500
H	6.63800	45.03900	13.90100
H	7.43400	45.98500	14.77400
O	3.38600	45.16000	13.72100
H	4.25100	44.96400	13.36200
H	3.45300	44.93100	14.64800
O	3.29400	44.26400	9.82000
H	2.64100	43.94900	9.19600
H	3.25200	43.64200	10.54600
O	9.86000	41.96500	11.85200
H	10.16600	41.10500	11.56400
H	10.58600	42.55700	11.65100

O	5.94100	46.46000	19.69800
H	5.29400	47.15900	19.60400
H	6.71100	46.89300	20.06600
O	7.68900	47.42700	12.20200
H	8.18600	46.67400	11.88200
H	6.91700	47.46400	11.63600
O	10.13100	44.39400	14.35000
H	9.21700	44.62500	14.51800
H	10.56000	44.47800	15.20100
O	4.10900	44.29700	16.14800
H	4.93200	43.82300	16.02600
H	4.35300	45.07600	16.64800
O	5.76600	44.54600	12.41100
H	5.78800	44.61000	11.45600
H	6.18300	43.70600	12.60500
O	7.31500	45.34500	17.75400
H	7.83300	46.08100	17.42900
H	6.96000	45.65100	18.58900
O	7.56600	41.76500	13.27400
H	7.84600	41.09700	13.90000
H	8.35100	41.95800	12.76200
O	9.45800	47.27200	17.20800
H	8.86300	47.55200	16.51200
H	9.17300	47.76100	17.97900
O	14.06500	50.19800	16.18200
H	13.54900	49.41400	15.99500
H	14.94900	49.87000	16.34600
O	11.72600	43.94200	12.14100
H	11.52800	43.63400	13.02600
H	12.33600	44.66800	12.27100
O	5.55400	44.74500	8.26800
H	4.74400	44.40000	8.64400
H	6.24300	44.43600	8.85700
O	7.48300	43.82300	9.89100
H	8.03600	44.24300	10.55000
H	7.72000	42.89700	9.93300
O	10.86100	45.93300	9.39900
H	10.28100	46.59200	9.01700
H	10.59500	45.11100	8.98700
O	9.31200	45.33400	11.60100
H	9.81200	45.44500	10.79200
H	9.95500	45.03000	12.24100
O	9.64400	48.09300	8.45400
H	8.75600	48.37800	8.23600
H	10.05900	47.93300	7.60700
O	12.99500	48.11800	12.17000
H	13.77100	48.60700	12.44200
H	12.27000	48.54400	12.62700
O	15.43200	48.83100	13.22700
H	16.02700	48.59000	12.51700
H	15.50500	48.11100	13.85500
O	3.66100	44.30000	5.54300
H	3.64900	45.24400	5.70000
H	4.55300	44.11700	5.24900
O	7.47600	40.89400	9.79800
H	6.70900	40.34000	9.94300
H	7.88700	40.96600	10.65900
O	-34.87200	-30.82700	1.41900
H	-34.23400	-30.94600	0.71500
H	-34.99000	-29.87900	1.47800
O	-38.06300	-30.87400	1.35500
H	-38.40100	-30.99000	0.46700
H	-37.19400	-31.27500	1.33500
O	-42.20400	-29.02100	-2.65500
H	-42.76800	-29.11200	-3.42300
H	-42.31900	-29.84100	-2.17500
O	-37.58100	-29.72100	7.31400
H	-37.59200	-30.11500	6.44200
H	-37.41900	-30.45400	7.90700
O	-36.62400	-25.35400	-7.34400
H	-37.27500	-24.76800	-7.73000
H	-35.83000	-24.82300	-7.28400
O	-42.69900	-28.26500	-6.94500
H	-41.92500	-28.81200	-7.08400
H	-42.34600	-27.40000	-6.73300
O	-40.86000	-26.93700	6.26200
H	-41.15100	-26.03200	6.14600
H	-40.22400	-26.89500	6.97600
O	-39.93400	-29.55900	2.85200
H	-39.33800	-29.78400	2.13800
H	-39.87700	-30.30100	3.45400
O	-35.31800	-33.10200	-1.50600
H	-34.53100	-33.52000	-1.85700
H	-35.05400	-32.19700	-1.33700
O	-35.14700	-27.66400	-6.80700
H	-35.78200	-27.59100	-6.09500
H	-35.47700	-27.07500	-7.48500
O	-29.91700	-33.16400	-0.60200
H	-29.18400	-33.73400	-0.36900
H	-30.67300	-33.55200	-0.16200
O	-40.64200	-30.20300	-7.11100
H	-40.75900	-30.87800	-6.44300
H	-40.53200	-30.68900	-7.92700
O	-38.29900	-30.74200	-3.40600
H	-38.66300	-31.26800	-2.69500
H	-37.44200	-31.13200	-3.57900
O	-35.90100	-27.25100	3.25700
H	-36.20100	-26.34800	3.14800
H	-36.24600	-27.51800	4.10900
O	-32.50100	-34.73300	0.93600
H	-33.41000	-34.80100	0.64200
H	-32.55300	-34.25500	1.76500
O	-40.91900	-20.48600	-3.77600
H	-40.99700	-19.53600	-3.69700
H	-41.52300	-20.83200	-3.11900
O	-38.42700	-33.31400	8.88000
H	-39.20400	-32.75400	8.87000
H	-37.78800	-32.82800	9.40100
O	-30.63500	-36.53100	2.71400
H	-31.27800	-36.23800	2.06800
H	-31.14000	-36.64600	3.52000
O	-32.98100	-32.78500	-6.59700
H	-32.06900	-32.55200	-6.77200
H	-33.49000	-32.20100	-7.16000
O	-40.17300	-30.50500	8.44200
H	-39.43100	-29.96700	8.16500
H	-40.87900	-29.87800	8.59900
O	-34.69700	-28.36200	7.03900

H	-34.28900	-28.80400	6.29400
H	-35.41900	-28.93700	7.29200
O	-34.37400	-27.80200	0.79400
H	-35.00300	-27.63700	0.09200
H	-34.76200	-27.38800	1.56500
O	-30.49500	-26.86700	-1.85100
H	-31.18000	-26.48700	-2.40200
H	-30.41100	-26.25700	-1.11800
O	-35.19400	-35.76400	6.07000
H	-35.43800	-35.01000	5.53400
H	-34.91800	-35.38600	6.90500
O	-33.24000	-29.22100	4.74800
H	-33.02800	-28.57700	4.07200
H	-33.28700	-30.05400	4.28000
O	-38.82100	-28.06500	-1.94100
H	-38.35800	-27.78600	-2.73000
H	-39.08600	-28.96600	-2.12300
O	-41.92900	-28.51700	0.99700
H	-41.35900	-28.08900	0.35900
H	-41.33800	-28.79900	1.69600
O	-32.12600	-26.34300	2.92000
H	-31.30400	-26.74500	2.63900
H	-32.12900	-26.44700	3.87200
O	-38.36000	-26.79500	5.07600
H	-38.29500	-26.33300	5.91200
H	-39.06900	-27.42500	5.20500
O	-24.03500	-30.00000	-0.73100
H	-24.78900	-30.56900	-0.88700
H	-23.90000	-30.03300	0.21600
O	-38.64500	-25.12800	-5.05600
H	-38.78100	-24.28300	-4.62700
H	-38.10400	-24.92600	-5.81900
O	-37.01800	-24.50600	9.07400
H	-37.46200	-25.29200	8.75700
H	-36.35700	-24.31800	8.40900
O	-34.92000	-24.22600	7.10300
H	-34.93800	-23.40500	6.61100
H	-33.99800	-24.35000	7.32800
O	-38.46300	-26.71100	7.94500
H	-37.93000	-27.47200	7.71500
H	-38.99100	-27.00400	8.68700
O	-35.33100	-31.61400	-8.01800
H	-35.84600	-30.84400	-7.78000
H	-35.60300	-32.28800	-7.39500
O	-30.35700	-28.86100	-6.08300
H	-31.13400	-29.20000	-6.52800
H	-29.67800	-28.85600	-6.75800
O	-35.54200	-33.74200	-6.19300
H	-35.84700	-33.42700	-5.34100
H	-34.58900	-33.66300	-6.15000
O	-37.09800	-27.58300	-4.54300
H	-37.62500	-28.31700	-4.85600
H	-37.59900	-26.80500	-4.78900
O	-32.11800	-29.38800	0.57300
H	-32.77600	-28.69800	0.66200
H	-31.31400	-28.99800	0.91700
O	-33.30800	-31.03300	-1.37600
H	-32.79500	-30.40000	-0.87400
H	-32.65800	-31.63300	-1.74200
O	-38.95200	-29.76500	-11.54000
H	-39.86000	-29.99600	-11.34100
H	-38.45500	-30.56100	-11.35200
O	-36.04600	-21.05500	-8.62000
H	-36.71900	-20.75200	-8.01200
H	-35.65900	-20.25200	-8.97000
O	-36.83000	-33.55800	0.87000
H	-37.74700	-33.33700	0.71200
H	-36.41400	-33.48400	0.01100
O	-36.04600	-32.15900	-4.00000
H	-35.29900	-31.64700	-4.30900
H	-35.77700	-32.48100	-3.14000
O	-37.77300	-29.66300	-7.01700
H	-38.68200	-29.94700	-6.92000
H	-37.74400	-29.24300	-7.87700
O	-32.54200	-26.05300	-3.24800
H	-32.99500	-26.89300	-3.19200
H	-33.17200	-25.41600	-2.91100
O	-33.59800	-35.12300	-2.01800
H	-34.33600	-35.63600	-1.68700
H	-33.34600	-35.56300	-2.83000
O	-34.09800	-24.21500	-2.31300
H	-33.80600	-23.65400	-3.03200
H	-33.58800	-23.92200	-1.55800
O	-39.22500	-32.05000	-0.91600
H	-39.17000	-33.00300	-0.99200
H	-40.03600	-31.89400	-0.43200
O	-38.07600	-28.12200	-9.67900
H	-38.51400	-28.83900	-10.13800
H	-37.33500	-27.89500	-10.24000
O	-39.13500	-23.43700	-1.22400
H	-39.17700	-23.21100	-2.15300
H	-38.21900	-23.67100	-1.07500
O	-35.68800	-32.96800	5.67400
H	-35.33300	-32.74100	6.53400
H	-35.04100	-32.63700	5.05200
O	-29.95900	-29.84700	-3.62100
H	-30.09700	-29.65600	-4.54900
H	-30.31500	-29.08500	-3.16400
O	-29.93200	-27.77900	1.39000
H	-29.49800	-27.87100	2.23900
H	-29.21800	-27.80300	0.75200
O	-41.01800	-26.56100	-1.42800
H	-40.22400	-27.05600	-1.62900
H	-41.72900	-27.08900	-1.79100
O	-36.19900	-24.17900	2.92400
H	-36.14500	-23.61000	3.69100
H	-37.06700	-24.00900	2.55900
O	-29.07000	-27.73700	3.92400
H	-28.87600	-26.83200	4.16900
H	-29.17000	-28.19600	4.75700
O	-34.00900	-27.58600	9.49800
H	-34.16000	-27.81500	8.58100
H	-34.01800	-26.62900	9.51100
O	-37.24000	-30.01300	4.48200
H	-36.31100	-29.86200	4.30900
H	-37.66100	-29.92400	3.62700
O	-38.44100	-25.98800	2.25200
H	-38.58000	-26.35600	3.12500

H	-39.23000	-25.47300	2.08100
O	-37.84600	-20.88700	-6.40500
H	-37.67100	-21.35800	-5.59100
H	-38.53300	-20.26000	-6.17700
O	-36.89100	-26.61000	-0.35900
H	-37.22600	-26.64900	0.53700
H	-37.39000	-27.27500	-0.83200
O	-33.95500	-29.26400	-10.08500
H	-34.57200	-29.98900	-10.18200
H	-34.42300	-28.50300	-10.42900
O	-40.28800	-31.49100	4.65500
H	-39.57800	-32.13100	4.61300
H	-40.33100	-31.23900	5.57700
O	-32.29900	-24.55400	7.57500
H	-32.35800	-25.23800	6.90800
H	-31.89700	-23.81200	7.12300
O	-32.29800	-26.59000	5.80700
H	-33.18500	-26.92700	5.92900
H	-31.72800	-27.32200	6.04400
O	-38.76700	-21.93000	2.77300
H	-38.84000	-21.12400	3.28500
H	-39.21000	-21.73100	1.94800
O	-38.97900	-22.69300	-3.71700
H	-39.76200	-22.15100	-3.62300
H	-38.27900	-22.17500	-3.31700
O	-27.81600	-31.17900	0.56800
H	-27.82700	-30.29700	0.19800
H	-28.68400	-31.53200	0.37400
O	-36.30800	-22.03100	4.92900
H	-35.59500	-21.39700	4.85600
H	-36.79900	-21.93500	4.11300
O	-34.58600	-28.12600	-3.50000
H	-35.52000	-28.00600	-3.66900
H	-34.40800	-29.02900	-3.76400
O	-31.19900	-32.28400	-2.96500
H	-30.73300	-31.45200	-3.03900
H	-30.74800	-32.75000	-2.26000
O	-41.25600	-24.28200	5.74900
H	-40.55500	-23.64800	5.90200
H	-41.72700	-23.94000	4.98900
O	-33.13100	-25.94000	-5.96600
H	-32.85500	-26.05400	-5.05700
H	-34.00100	-26.33800	-6.00500
O	-33.45600	-31.72700	3.57400
H	-34.10900	-31.48000	2.92000
H	-33.07800	-32.54100	3.24100
O	-36.49600	-21.69100	-2.32300
H	-36.31300	-22.50900	-1.86000
H	-35.67200	-21.47500	-2.76000
O	-36.16100	-22.76500	-5.06100
H	-35.63300	-23.27500	-5.67400
H	-35.89900	-23.08000	-4.19700
O	-37.40800	-30.98500	10.06100
H	-38.08300	-31.08600	10.73100
H	-36.58500	-31.14100	10.52500
O	-38.66500	-19.67900	4.29700
H	-38.11300	-19.51600	5.06200
H	-38.32500	-19.08800	3.62500
O	-33.81400	-24.85300	10.15200
H	-33.27000	-24.24000	9.65700
H	-34.39600	-24.29500	10.66800
O	-35.52800	-21.73000	1.28300
H	-35.65300	-22.53200	0.77400
H	-34.87100	-21.23600	0.79500
O	-33.46500	-23.93000	2.91100
H	-34.41200	-24.04900	2.83900
H	-33.11700	-24.81700	2.99400
O	-36.46000	-24.13400	-0.88800
H	-36.60800	-25.06300	-0.70800
H	-35.63500	-24.11200	-1.37300
O	-32.85700	-23.31100	0.19800
H	-31.93200	-23.51700	0.06000
H	-33.02500	-23.56000	1.10700
O	-33.06700	-22.79300	-4.44600
H	-32.89400	-23.35800	-5.19800
H	-32.57400	-21.99300	-4.62800
O	-27.21300	-30.02900	-2.82700
H	-26.77000	-30.77900	-3.22500
H	-28.09100	-30.03800	-3.20900
O	-32.08000	-23.47600	-7.34600
H	-31.38500	-22.85600	-7.12700
H	-31.73800	-24.32700	-7.07100
O	-30.18100	-26.02600	-5.81700
H	-29.65600	-25.68800	-6.54200
H	-30.26800	-26.96200	-6.00100
O	-38.51900	-22.11300	6.31200
H	-38.21000	-22.35800	7.18400
H	-37.72100	-21.90800	5.82400
O	-38.49700	-19.95900	-1.45800
H	-38.93600	-20.45100	-0.76500
H	-37.74600	-20.50200	-1.69900
O	-41.04900	-24.41500	0.61400
H	-41.67100	-25.07800	0.31500
H	-40.37900	-24.38900	-0.06800
O	-30.49100	-25.39300	9.48800
H	-31.28200	-25.41100	8.94800
H	-30.72800	-24.85400	10.24300
O	-30.37200	-28.54400	6.80100
H	-30.32200	-28.70900	7.74300
H	-30.48000	-29.41100	6.41100
O	-41.48800	-25.86500	-5.91000
H	-41.33900	-25.18100	-6.56300
H	-40.68100	-25.88400	-5.39500
O	-33.08100	-32.56100	8.11700
H	-32.18000	-32.70000	7.82500
H	-33.00600	-32.39500	9.05700
O	-28.87000	-26.12400	7.04500
H	-29.16100	-25.76100	7.88200
H	-29.48200	-26.83700	6.86700
O	-27.67000	-28.55900	-0.43400
H	-26.83500	-28.12000	-0.27100
H	-27.53400	-29.03500	-1.25400
O	-30.62100	-31.00100	5.51300
H	-30.14600	-30.80800	4.70500
H	-31.54400	-30.94500	5.26800
O	-37.98800	-23.37000	-8.43900
H	-38.56100	-22.75100	-8.89200
H	-37.41500	-22.82000	-7.90500

O	-31.92100	-34.12400	3.74600
H	-30.96900	-34.02400	3.76700
H	-32.09000	-34.91600	4.25600
O	-34.76900	-23.08600	-7.30800
H	-33.83600	-23.15200	-7.10400
H	-34.85600	-22.25100	-7.76600
O	-32.14000	-23.77000	-10.34700
H	-32.21800	-24.10400	-9.45400
H	-31.31900	-23.27800	-10.34800
O	-29.44200	-30.90400	2.86000
H	-29.47400	-31.84200	3.04600
H	-28.83200	-30.82500	2.12700
O	-37.60400	-32.03400	-11.09700
H	-36.65800	-32.16300	-11.02800
H	-37.98200	-32.77200	-10.62000
O	-29.29300	-34.11200	2.90600
H	-29.80900	-34.89700	2.71900
H	-28.40900	-34.44100	3.07100
O	-23.62400	-29.84200	2.00100
H	-22.89400	-30.10300	2.56200
H	-24.23400	-30.57900	2.04100
O	-25.54300	-31.70100	2.59800
H	-26.27000	-31.83600	1.99000
H	-25.95800	-31.39300	3.40400
O	-37.72000	-27.98200	10.36300
H	-36.80000	-27.80400	10.55700
H	-37.75500	-28.92700	10.21100
O	-39.98800	-31.06900	11.21200
H	-40.47100	-30.26900	11.41900
H	-39.90200	-31.05800	10.25900
O	-33.71400	-36.21700	-4.82100
H	-34.60100	-35.93200	-5.03900
H	-33.29800	-36.37800	-5.66800
O	-30.36200	-36.19300	-1.10600
H	-29.58600	-35.70300	-0.83300
H	-31.08700	-35.76700	-0.64800
O	-27.92500	-35.48000	-0.04800
H	-27.33100	-35.72000	-0.75900
H	-27.85200	-36.19900	0.57900
O	-34.56900	-26.45300	-10.72600
H	-33.86600	-26.24800	-10.11100
H	-35.29300	-25.88500	-10.46100
O	-33.74000	-30.70200	-4.60600
H	-33.79600	-30.24500	-5.44500
H	-33.18700	-31.46300	-4.78400
O	-29.92900	-34.91300	-3.78100
H	-30.18500	-35.31300	-2.95000
H	-30.55400	-34.19900	-3.90600
O	-25.61800	-32.17300	-1.27400
H	-26.17800	-32.10400	-0.50100
H	-26.22400	-32.34900	-1.99400
O	-32.66100	-26.01500	-8.67600
H	-31.82600	-26.44700	-8.85900
H	-32.82200	-26.18600	-7.74900
O	-33.00600	-29.51100	-7.31100
H	-33.06100	-29.67700	-8.25200
H	-33.63200	-28.80400	-7.15500
O	-35.69200	-27.20700	12.01500
H	-34.82200	-27.03300	11.65300
H	-36.17100	-26.38700	11.89200
O	-41.52300	-20.08700	3.69300
H	-40.62000	-20.05500	4.01000
H	-41.74900	-21.01700	3.70800
O	-39.76800	-21.33400	0.35400
H	-40.71800	-21.33400	0.24100
H	-39.46400	-22.08000	-0.16200
O	-24.03200	-15.49800	1.41900
H	-23.39400	-15.61700	0.71500
H	-24.15000	-14.55000	1.47800
O	-27.22300	-15.54500	1.35500
H	-27.56200	-15.66100	0.46700
H	-26.35400	-15.94600	1.33500
O	-31.36500	-13.69100	-2.65500
H	-31.92800	-13.78200	-3.42300
H	-31.47900	-14.51100	-2.17500
O	-26.74100	-14.39100	7.31400
H	-26.75300	-14.78600	6.44200
H	-26.57900	-15.12500	7.90700
O	-33.81800	-12.19600	0.18300
H	-33.02600	-12.53700	-0.23200
H	-33.57900	-12.08700	1.10400
O	-25.78500	-10.02400	-7.34400
H	-26.43600	-9.43900	-7.73000
H	-24.99000	-9.49400	-7.28400
O	-31.85900	-12.93500	-6.94500
H	-31.08600	-13.48300	-7.08400
H	-31.50700	-12.07100	-6.73300
O	-30.02100	-11.60800	6.26200
H	-30.31200	-10.70300	6.14600
H	-29.38500	-11.56600	6.97600
O	-29.09500	-14.23000	2.85200
H	-28.49900	-14.45400	2.13800
H	-29.03800	-14.97200	3.45400
O	-29.52300	-16.77600	-9.31300
H	-29.27000	-17.29800	-10.07500
H	-30.46000	-16.94000	-9.21000
O	-24.47800	-17.77300	-1.50600
H	-23.69200	-18.19100	-1.85700
H	-24.21400	-16.86800	-1.33700
O	-24.30700	-12.33500	-6.80700
H	-24.94300	-12.26200	-6.09500
H	-24.63700	-11.74600	-7.48500
O	-19.07700	-17.83500	-0.60200
H	-18.34500	-18.40500	-0.36900
H	-19.83300	-18.22300	-0.16200
O	-29.80300	-14.87300	-7.11100
H	-29.91900	-15.54900	-6.44300
H	-29.69300	-15.36000	-7.92700
O	-27.46000	-15.41200	-3.40600
H	-27.82400	-15.93900	-2.69500
H	-26.60300	-15.80200	-3.57900
O	-21.55500	-24.12300	3.85700
H	-21.88600	-23.25300	3.63300
H	-22.15000	-24.43900	4.53700
O	-27.81900	-20.38300	-8.41000
H	-27.65200	-19.51500	-8.04300
H	-28.00900	-20.22100	-9.33400
O	-25.06200	-11.92200	3.25700

H	-25.36200	-11.01900	3.14800
H	-25.40700	-12.18800	4.10900
O	-31.61000	-15.47000	-0.71000
H	-32.53500	-15.60300	-0.50200
H	-31.36200	-14.69100	-0.21300
O	-21.66200	-19.40300	0.93600
H	-22.57000	-19.47200	0.64200
H	-21.71300	-18.92600	1.76500
O	-27.84000	-17.92800	-7.42500
H	-27.96000	-17.48200	-6.58700
H	-28.53500	-17.57900	-7.98400
O	-34.25000	-15.82700	0.12500
H	-34.12000	-15.57300	1.03900
H	-34.93000	-15.23400	-0.19500
O	-30.08000	-5.15700	-3.77600
H	-30.15700	-4.20600	-3.69700
H	-30.68300	-5.50300	-3.11900
O	-27.58800	-17.98400	8.88000
H	-28.36500	-17.42500	8.87000
H	-26.94900	-17.49900	9.40100
O	-19.79600	-21.20200	2.71400
H	-20.43800	-20.90800	2.06800
H	-20.30000	-21.31600	3.52000
O	-22.14200	-17.45500	-6.59700
H	-21.23000	-17.22300	-6.77200
H	-22.65100	-16.87200	-7.16000
O	-29.33300	-15.17600	8.44200
H	-28.59200	-14.63700	8.16500
H	-30.04000	-14.54900	8.59900
O	-23.85800	-13.03300	7.03900
H	-23.44900	-13.47500	6.29400
H	-24.58000	-13.60800	7.29200
O	-27.50500	-18.09600	4.41200
H	-26.65800	-17.67200	4.55600
H	-27.56500	-18.75200	5.10600
O	-23.53500	-12.47300	0.79400
H	-24.16400	-12.30800	0.09200
H	-23.92200	-12.05900	1.56500
O	-19.65600	-11.53700	-1.85100
H	-20.34100	-11.15700	-2.40200
H	-19.57200	-10.92800	-1.11800
O	-24.35500	-20.43500	6.07000
H	-24.59900	-19.68100	5.53400
H	-24.07900	-20.05700	6.90500
O	-22.40100	-13.89100	4.74800
H	-22.18900	-13.24800	4.07200
H	-22.44800	-14.72500	4.28000
O	-24.79800	-21.28000	1.65200
H	-25.66000	-20.98100	1.94400
H	-24.77200	-21.06300	0.72000
O	-27.98200	-12.73500	-1.94100
H	-27.51800	-12.45700	-2.73000
H	-28.24600	-13.63700	-2.12300
O	-31.09000	-13.18800	0.99700
H	-30.51900	-12.76000	0.35900
H	-30.49900	-13.47000	1.69600
O	-29.04500	-16.61300	-5.13200
H	-28.34300	-16.16100	-4.66400
H	-29.37700	-17.25500	-4.50400
O	-30.59700	-18.02200	9.24300
H	-31.22700	-17.34000	9.47700
H	-30.70300	-18.13400	8.29900
O	-35.30300	-18.35500	0.17000
H	-36.09300	-18.09900	-0.30600
H	-34.81100	-17.54000	0.27500
O	-21.28600	-11.01400	2.92000
H	-20.46400	-11.41600	2.63900
H	-21.29000	-11.11700	3.87200
O	-33.35100	-17.99000	-2.87200
H	-34.14800	-17.87800	-2.35300
H	-33.57900	-17.65200	-3.73800
O	-27.52100	-11.46600	5.07600
H	-27.45600	-11.00400	5.91200
H	-28.22900	-12.09600	5.20500
O	-37.01900	-18.10200	2.60100
H	-36.76800	-17.21700	2.86700
H	-36.48300	-18.28100	1.82900
O	-13.19600	-14.67000	-0.73100
H	-13.94900	-15.24000	-0.88700
H	-13.06100	-14.70400	0.21600
O	-27.80600	-9.79900	-5.05600
H	-27.94100	-8.95400	-4.62700
H	-27.26500	-9.59700	-5.81900
O	-31.31900	-23.72000	11.60600
H	-31.71200	-24.27300	12.28100
H	-30.50200	-23.40900	11.99600
O	-34.35300	-12.28100	-4.48300
H	-33.84000	-11.48700	-4.33200
H	-33.70100	-12.97900	-4.54900
O	-32.54300	-9.16500	-2.40100
H	-32.36700	-8.24300	-2.58800
H	-31.74800	-9.47900	-1.97100
O	-28.40200	-22.30400	9.19800
H	-27.66100	-21.70300	9.11800
H	-28.17000	-23.04600	8.64000
O	-33.25800	-17.11800	7.47600
H	-33.37300	-18.02000	7.77600
H	-34.11400	-16.87200	7.12400
O	-26.17900	-9.17700	9.07400
H	-26.62300	-9.96300	8.75700
H	-25.51700	-8.98900	8.40900
O	-31.42000	-7.64700	3.60000
H	-30.86900	-7.77500	2.82800
H	-32.25900	-7.34800	3.25000
O	-24.08100	-8.89600	7.10300
H	-24.09900	-8.07500	6.61100
H	-23.15900	-9.02100	7.32800
O	-27.62300	-11.38200	7.94500
H	-27.09100	-12.14300	7.71500
H	-28.15200	-11.67500	8.68700
O	-24.45700	-26.27600	3.47400
H	-24.80000	-25.38600	3.39800
H	-24.08900	-26.46600	2.61100
O	-36.38800	-13.95300	-3.70700
H	-35.55000	-13.54300	-3.92400
H	-36.21000	-14.89300	-3.73700
O	-24.49100	-16.28500	-8.01800
H	-25.00700	-15.51500	-7.78000

H	-24.76400	-16.95900	-7.39500
O	-26.90700	-21.44000	-2.96000
H	-27.76300	-21.82900	-3.13900
H	-26.49400	-21.36100	-3.82000
O	-25.37700	-23.90500	1.43900
H	-25.88000	-23.94200	0.62600
H	-25.08400	-22.99600	1.49900
O	-29.13300	-23.82200	0.44600
H	-28.26700	-24.01700	0.08600
H	-29.06500	-24.05100	1.37300
O	-19.51800	-13.53200	-6.08300
H	-20.29500	-13.87100	-6.52800
H	-18.83900	-13.52700	-6.75800
O	-24.70300	-18.41200	-6.19300
H	-25.00800	-18.09800	-5.34100
H	-23.75000	-18.33400	-6.15000
O	-26.25800	-12.25300	-4.54300
H	-26.78600	-12.98800	-4.85600
H	-26.75900	-11.47600	-4.78900
O	-21.27900	-14.05800	0.57300
H	-21.93700	-13.36900	0.66200
H	-20.47500	-13.66900	0.91700
O	-31.52600	-21.59100	-2.18300
H	-30.95200	-21.81400	-2.91500
H	-30.93900	-21.52200	-1.43000
O	-22.46800	-15.70400	-1.37600
H	-21.95500	-15.07100	-0.87400
H	-21.81800	-16.30400	-1.74200
O	-31.19900	-19.56100	-8.16300
H	-32.03600	-19.22300	-7.84200
H	-31.33900	-19.69600	-9.10000
O	-29.22400	-24.71700	-3.45500
H	-29.87800	-25.03200	-4.07900
H	-29.26600	-25.34000	-2.72900
O	-28.11300	-14.43500	-11.54000
H	-29.02000	-14.66600	-11.34100
H	-27.61600	-15.23200	-11.35200
O	-25.20600	-5.72600	-8.62000
H	-25.88000	-5.42200	-8.01200
H	-24.82000	-4.92300	-8.97000
O	-25.99000	-18.22900	0.87000
H	-26.90800	-18.00800	0.71200
H	-25.57400	-18.15400	0.01100
O	-25.20700	-16.83000	-4.00000
H	-24.46000	-16.31800	-4.30900
H	-24.93800	-17.15200	-3.14000
O	-26.93400	-14.33400	-7.01700
H	-27.84300	-14.61800	-6.92000
H	-26.90500	-13.91400	-7.87700
O	-21.70200	-10.72300	-3.24800
H	-22.15600	-11.56400	-3.19200
H	-22.33300	-10.08700	-2.91100
O	-22.75900	-19.79400	-2.01800
H	-23.49700	-20.30600	-1.68700
H	-22.50600	-20.23400	-2.83000
O	-23.25900	-8.88600	-2.31300
H	-22.96700	-8.32500	-3.03200
H	-22.74900	-8.59300	-1.55800
O	-22.65800	-27.01700	-1.42400
H	-22.35200	-27.87700	-1.71100
H	-21.93200	-26.42500	-1.62400
O	-36.36500	-16.61400	-3.03600
H	-36.56300	-17.20700	-3.76100
H	-36.92100	-16.91800	-2.31800
O	-28.38600	-16.72100	-0.91600
H	-28.33100	-17.67400	-0.99200
H	-29.19700	-16.56500	-0.43200
O	-27.23700	-12.79300	-9.67900
H	-27.67500	-13.51000	-10.13800
H	-26.49500	-12.56600	-10.24000
O	-30.44600	-18.10300	-3.15700
H	-31.26000	-17.80400	-2.75100
H	-29.95000	-18.49800	-2.44000
O	-28.29600	-8.10800	-1.22400
H	-28.33800	-7.88200	-2.15300
H	-27.37900	-8.34100	-1.07500
O	-24.84900	-17.63900	5.67400
H	-24.49400	-17.41200	6.53400
H	-24.20200	-17.30700	5.05200
O	-19.11900	-14.51700	-3.62100
H	-19.25700	-14.32700	-4.54900
H	-19.47600	-13.75600	-3.16400
O	-30.95500	-20.53200	2.45900
H	-30.31500	-20.67300	1.76100
H	-31.78700	-20.81800	2.08300
O	-19.09200	-12.45000	1.39000
H	-18.65900	-12.54200	2.23900
H	-18.37900	-12.47400	0.75200
O	-30.17900	-11.23100	-1.42800
H	-29.38500	-11.72600	-1.62900
H	-30.89000	-11.76000	-1.79100
O	-25.35900	-8.85000	2.92400
H	-25.30600	-8.28100	3.69100
H	-26.22800	-8.67900	2.55900
O	-33.87100	-17.08700	-5.35200
H	-34.80100	-17.14900	-5.13500
H	-33.77500	-17.61000	-6.14800
O	-33.95200	-20.56200	-2.76400
H	-33.69100	-19.64700	-2.87300
H	-33.13000	-21.02900	-2.61400
O	-32.48000	-19.14800	4.40600
H	-31.80700	-19.35600	3.75700
H	-33.30700	-19.34600	3.96700
O	-18.23100	-12.40700	3.92400
H	-18.03700	-11.50300	4.16900
H	-18.33100	-12.86700	4.75700
O	-26.57700	-22.52200	6.42300
H	-27.22400	-21.82300	6.32900
H	-25.80700	-22.08800	6.79000
O	-23.16900	-12.25700	9.49800
H	-23.32100	-12.48600	8.58100
H	-23.17900	-11.30000	9.51100
O	-26.40100	-14.68400	4.48200
H	-25.47100	-14.53200	4.30900
H	-26.82200	-14.59500	3.62700
O	-27.60200	-10.65900	2.25200
H	-27.74100	-11.02700	3.12500
H	-28.39000	-10.14400	2.08100

O	-27.00700	-5.55800	-6.40500
H	-26.83200	-6.02900	-5.59100
H	-27.69400	-4.93100	-6.17700
O	-26.05100	-11.28000	-0.35900
H	-26.38600	-11.32000	0.53700
H	-26.55100	-11.94600	-0.83200
O	-32.07700	-15.87400	5.16600
H	-32.21500	-16.60600	5.76700
H	-31.16600	-15.96300	4.88500
O	-23.11500	-13.93500	-10.08500
H	-23.73200	-14.66000	-10.18200
H	-23.58400	-13.17400	-10.42900
O	-29.44900	-16.16200	4.65500
H	-28.73900	-16.80200	4.61300
H	-29.49200	-15.91000	5.57700
O	-21.46000	-9.22500	7.57500
H	-21.51900	-9.90900	6.90800
H	-21.05800	-8.48300	7.12300
O	-30.73600	-19.17500	6.47400
H	-31.27200	-19.62600	7.12600
H	-31.32400	-19.04000	5.73100
O	-32.44100	-2.40900	0.36900
H	-32.20100	-1.77700	1.04700
H	-33.28400	-2.76000	0.65500
O	-31.32600	-19.71600	-10.96300
H	-32.20300	-19.83700	-11.32700
H	-30.85000	-20.50800	-11.21500
O	-21.45800	-11.26100	5.80700
H	-22.34600	-11.59800	5.92900
H	-20.88800	-11.99300	6.04400
O	-39.80500	-14.92800	-0.86900
H	-39.11800	-15.24200	-1.45700
H	-39.96100	-14.02400	-1.14200
O	-35.00200	-20.03900	3.55900
H	-35.08600	-20.64700	2.82500
H	-35.82400	-19.54900	3.56000
O	-27.92800	-6.60100	2.77300
H	-28.00100	-5.79500	3.28500
H	-28.37000	-6.40200	1.94800
O	-28.13900	-7.36400	-3.71700
H	-28.92200	-6.82100	-3.62300
H	-27.44000	-6.84600	-3.31700
O	-24.82900	-21.55400	-1.07400
H	-24.33300	-22.30700	-1.39300
H	-25.60100	-21.51800	-1.63900
O	-16.97700	-15.85000	0.56800
H	-16.98800	-14.96800	0.19800
H	-17.84500	-16.20300	0.37400
O	-25.46800	-6.70200	4.92900
H	-24.75500	-6.06700	4.85600
H	-25.95900	-6.60600	4.11300
O	-23.74700	-12.79700	-3.50000
H	-24.68100	-12.67700	-3.66900
H	-23.56900	-13.70000	-3.76400
O	-28.24600	-19.41200	-1.34600
H	-28.61600	-19.96500	-0.65800
H	-27.75000	-20.01700	-1.89900
O	-20.35900	-16.95500	-2.96500
H	-19.89300	-16.12200	-3.03900
H	-19.90800	-17.42100	-2.26000
O	-30.41700	-8.95300	5.74900
H	-29.71600	-8.31900	5.90200
H	-30.88800	-8.61100	4.98900
O	-22.29200	-10.61100	-5.96600
H	-22.01600	-10.72500	-5.05700
H	-23.16100	-11.00900	-6.00500
O	-22.38800	-24.58700	1.07400
H	-23.30100	-24.35700	1.24300
H	-21.95800	-24.50300	1.92600
O	-22.61700	-16.39800	3.57400
H	-23.27000	-16.15100	2.92000
H	-22.23900	-17.21200	3.24100
O	-25.65600	-6.36200	-2.32300
H	-25.47400	-7.18000	-1.86000
H	-24.83300	-6.14500	-2.76000
O	-25.32200	-7.43500	-5.06100
H	-24.79300	-7.94600	-5.67400
H	-25.05900	-7.75100	-4.19700
O	-26.56800	-15.65600	10.06100
H	-27.24400	-15.75700	10.73100
H	-25.74500	-15.81200	10.52500
O	-34.82000	-9.41900	2.68200
H	-34.23500	-10.16500	2.81500
H	-35.01800	-9.10900	3.56600
O	-31.96600	-6.38400	-2.30100
H	-32.63500	-5.89900	-2.78500
H	-32.10500	-6.13700	-1.38700
O	-40.22900	-16.84800	1.40300
H	-40.19000	-17.38200	2.19600
H	-40.73400	-17.37500	0.78400
O	-27.82600	-4.34900	4.29700
H	-27.27400	-4.18600	5.06200
H	-27.48500	-3.75900	3.62500
O	-32.84900	-22.00200	4.89900
H	-32.78500	-21.14000	4.48700
H	-32.91300	-22.61500	4.16600
O	-33.37400	-19.93000	8.54600
H	-32.74700	-20.31200	9.16000
H	-34.23100	-20.14800	8.91200
O	-22.97500	-9.52300	10.15200
H	-22.43100	-8.91000	9.65700
H	-23.55600	-8.96500	10.66800
O	-36.59800	-18.12200	-5.52200
H	-36.30200	-19.03100	-5.57500
H	-37.49900	-18.14300	-5.84400
O	-24.68900	-6.40100	1.28300
H	-24.81400	-7.20200	0.77400
H	-24.03100	-5.90700	0.79500
O	-33.06800	-20.04600	0.46100
H	-33.79000	-19.43300	0.32200
H	-32.27900	-19.51100	0.38300
O	-22.62600	-8.60000	2.91100
H	-23.57300	-8.72000	2.83900
H	-22.27700	-9.48800	2.99400
O	-30.26600	-22.71100	6.73500
H	-29.74200	-22.46500	7.49800
H	-30.48400	-21.87900	6.31500
O	-34.86900	-20.44100	-5.39800

H	-35.19800	-21.32100	-5.58000
H	-34.51500	-20.49600	-4.51000
O	-25.62100	-8.80500	-0.88800
H	-25.76800	-9.73300	-0.70800
H	-24.79600	-8.78300	-1.37300
O	-31.75500	-20.15300	-5.03000
H	-31.41500	-19.62500	-5.75300
H	-31.30700	-19.81500	-4.25500
O	-22.01800	-7.98200	0.19800
H	-21.09300	-8.18800	0.06000
H	-22.18600	-8.23100	1.10700
O	-29.06000	-21.77000	4.42000
H	-29.40100	-22.56900	4.82200
H	-29.76600	-21.46900	3.84800
O	-28.40900	-24.68500	2.87300
H	-27.58600	-25.15900	2.75100
H	-28.16500	-23.90600	3.37300
O	-29.76800	-21.65600	-6.83100
H	-29.04700	-21.27800	-7.33500
H	-30.39100	-20.93700	-6.72800
O	-25.62500	-20.95100	-5.89500
H	-25.40800	-20.06900	-6.19600
H	-26.10500	-21.34300	-6.62500
O	-30.88500	-17.99300	0.68200
H	-31.03000	-17.67900	1.57400
H	-31.36200	-17.37400	0.12900
O	-29.02500	-21.00300	0.73000
H	-29.03900	-21.89800	0.39300
H	-28.30800	-20.99500	1.36400
O	-27.17300	-20.56900	2.77500
H	-27.43100	-19.64700	2.77400
H	-27.70400	-20.96600	3.46500
O	-22.22800	-7.46400	-4.44600
H	-22.05400	-8.02900	-5.19800
H	-21.73500	-6.66400	-4.62800
O	-36.41000	-15.45200	3.62100
H	-36.95500	-14.67600	3.75300
H	-35.53200	-15.10500	3.46300
O	-29.62200	-22.12100	-4.00100
H	-29.50000	-23.04900	-3.80200
H	-29.91100	-22.10700	-4.91300
O	-26.75200	-24.43600	-0.86500
H	-26.73000	-24.37200	-1.81900
H	-26.33600	-25.27500	-0.67100
O	-16.37400	-14.70000	-2.82700
H	-15.93100	-15.44900	-3.22500
H	-17.25100	-14.70900	-3.20900
O	-21.24000	-8.14700	-7.34600
H	-20.54500	-7.52600	-7.12700
H	-20.89800	-8.99700	-7.07100
O	-35.62400	-16.32500	6.51400
H	-36.36600	-16.17700	7.10000
H	-35.94400	-16.06400	5.65000
O	-19.34100	-10.69700	-5.81700
H	-18.81600	-10.35800	-6.54200
H	-19.42900	-11.63200	-6.00100
O	-27.67900	-6.78400	6.31200
H	-27.37100	-7.02900	7.18400
H	-26.88200	-6.57900	5.82400
O	-27.65700	-4.63000	-1.45800
H	-28.09700	-5.12200	-0.76500
H	-26.90600	-5.17300	-1.69900
O	-35.17800	-8.43900	-1.45500
H	-34.35200	-8.73800	-1.83500
H	-35.52900	-9.20800	-1.00600
O	-30.21000	-9.08600	0.61400
H	-30.83200	-9.74900	0.31500
H	-29.53900	-9.06000	-0.06800
O	-36.97700	-10.52000	-0.49600
H	-37.81400	-10.94800	-0.67800
H	-37.00500	-10.32700	0.44100
O	-30.13700	-20.60800	10.20800
H	-30.08900	-19.73200	9.82500
H	-29.59500	-21.15000	9.63500
O	-19.65200	-10.06400	9.48800
H	-20.44200	-10.08100	8.94800
H	-19.88900	-9.52500	10.24300
O	-19.53200	-13.21500	6.80100
H	-19.48200	-13.38000	7.74300
H	-19.64100	-14.08200	6.41100
O	-30.64900	-10.53600	-5.91000
H	-30.50000	-9.85200	-6.56300
H	-29.84200	-10.55500	-5.39500
O	-22.24200	-17.23100	8.11700
H	-21.34100	-17.37100	7.82500
H	-22.16700	-17.06600	9.05700
O	-27.90700	-19.85500	6.96200
H	-28.77700	-19.56100	6.69100
H	-27.67200	-19.27100	7.68300
O	-18.03000	-10.79500	7.04500
H	-18.32200	-10.43200	7.88200
H	-18.64300	-11.50800	6.86700
O	-16.83100	-13.23000	-0.43400
H	-15.99600	-12.79100	-0.27100
H	-16.69500	-13.70600	-1.25400
O	-19.78200	-15.67200	5.51300
H	-19.30600	-15.47900	4.70500
H	-20.70500	-15.61600	5.26800
O	-32.89300	-14.50900	-4.90400
H	-33.13900	-15.41300	-4.70600
H	-32.72400	-14.50700	-5.84600
O	-25.20400	-23.63700	4.47900
H	-24.68500	-22.90100	4.15300
H	-25.55800	-23.33000	5.31400
O	-33.62400	-18.77600	-7.40500
H	-34.31700	-18.70200	-8.06100
H	-33.86900	-19.54000	-6.88300
O	-24.95200	-27.21700	-0.00100
H	-24.67200	-27.88400	0.62500
H	-24.16700	-27.02400	-0.51400
O	-27.14900	-8.04100	-8.43900
H	-27.72200	-7.42200	-8.89200
H	-26.57600	-7.49100	-7.90500
O	-21.08100	-18.79500	3.74600
H	-20.13000	-18.69500	3.76700
H	-21.25100	-19.58700	4.25600
O	-23.06000	-21.70900	3.93200
H	-23.65500	-21.42900	3.23700

H	-23.34500	-21.22000	4.70400
O	-23.92900	-7.75700	-7.30800
H	-22.99600	-7.82300	-7.10400
H	-24.01700	-6.92100	-7.76600
O	-21.30000	-8.44100	-10.34700
H	-21.37900	-8.77500	-9.45400
H	-20.47900	-7.94800	-10.34800
O	-18.60300	-15.57400	2.86000
H	-18.63500	-16.51300	3.04600
H	-17.99300	-15.49600	2.12700
O	-26.76400	-16.70500	-11.09700
H	-25.81800	-16.83400	-11.02800
H	-27.14300	-17.44300	-10.62000
O	-18.45300	-18.78300	2.90600
H	-18.96900	-19.56700	2.71900
H	-17.56900	-19.11100	3.07100
O	-20.79200	-25.03900	-1.13400
H	-20.99000	-25.34800	-0.25000
H	-20.18200	-24.31300	-1.00400
O	-12.78500	-14.51300	2.00100
H	-12.05500	-14.77400	2.56200
H	-13.39400	-15.25000	2.04100
O	-14.70300	-16.37200	2.59800
H	-15.43000	-16.50600	1.99000
H	-15.11900	-16.06400	3.40400
O	-26.88000	-12.65300	10.36300
H	-25.96000	-12.47500	10.55700
H	-26.91500	-13.59700	10.21100
O	-28.58000	-18.78000	-11.16100
H	-29.48700	-19.07600	-11.08500
H	-28.30200	-19.07700	-12.02800
O	-37.66200	-17.45900	-0.71200
H	-37.98200	-18.31300	-1.00400
H	-38.20900	-17.24400	0.04400
O	-33.88400	-14.74300	3.00500
H	-33.66600	-13.81100	3.02400
H	-33.47200	-15.10200	3.79100
O	-26.96400	-24.23600	-5.00700
H	-27.77400	-24.58100	-4.63200
H	-26.27500	-24.54500	-4.41800
O	-25.03500	-25.15800	-3.38500
H	-24.48200	-24.73900	-2.72600
H	-24.79800	-26.08500	-3.34200
O	-29.01100	-19.17000	12.39600
H	-29.11200	-18.22000	12.32700
H	-29.22900	-19.49800	11.52300
O	-29.81000	-21.92100	-11.02000
H	-28.94800	-21.59600	-10.76100
H	-29.62500	-22.70800	-11.53300
O	-37.79300	-13.83900	1.25800
H	-38.48700	-14.25300	0.74500
H	-37.04600	-13.79700	0.66100
O	-21.65700	-23.04800	-3.87700
H	-22.23700	-22.39000	-4.25900
H	-21.92300	-23.87000	-4.28800
O	-23.20700	-23.64800	-1.67500
H	-22.70700	-23.53600	-2.48300
H	-22.56300	-23.95200	-1.03500
O	-29.14800	-15.74000	11.21200
H	-29.63200	-14.94000	11.41900
H	-29.06200	-15.72900	10.25900
O	-35.88000	-13.90700	-0.86600
H	-35.90300	-13.80800	-1.81700
H	-35.57000	-13.06000	-0.54400
O	-33.22100	-11.84900	3.24900
H	-32.26700	-11.88100	3.18200
H	-33.42000	-12.31000	4.06400
O	-32.63100	-13.27900	5.81600
H	-31.81200	-12.78500	5.84000
H	-32.35700	-14.19600	5.78800
O	-22.87400	-20.88800	-4.82100
H	-23.76200	-20.60300	-5.03900
H	-22.45900	-21.04900	-5.66800
O	-19.52300	-20.86400	-1.10600
H	-18.74700	-20.37400	-0.83300
H	-20.24800	-20.43800	-0.64800
O	-17.08600	-20.15100	-0.04800
H	-16.49100	-20.39100	-0.75900
H	-17.01300	-20.87000	0.57900
O	-34.86600	-6.30700	0.26600
H	-35.00100	-7.06700	-0.30000
H	-35.28300	-5.58300	-0.20100
O	-23.73000	-11.12400	-10.72600
H	-23.02600	-10.91900	-10.11100
H	-24.45400	-10.55600	-10.46100
O	-22.90000	-15.37300	-4.60600
H	-22.95700	-14.91600	-5.44500
H	-22.34700	-16.13400	-4.78400
O	-19.09000	-19.58400	-3.78100
H	-19.34600	-19.98400	-2.95000
H	-19.71500	-18.86900	-3.90600
O	-14.77900	-16.84400	-1.27400
H	-15.33900	-16.77500	-0.50100
H	-15.38400	-17.02000	-1.99400
O	-31.70900	-5.41000	0.47800
H	-31.82600	-4.46600	0.37600
H	-32.53000	-5.71300	0.86600
O	-21.82100	-10.68500	-8.67600
H	-20.98700	-11.11700	-8.85900
H	-21.98300	-10.85700	-7.74900
O	-22.16700	-14.18200	-7.31100
H	-22.22200	-14.34800	-8.25200
H	-22.79200	-13.47500	-7.15500
O	-37.37800	-18.94500	6.51500
H	-36.73200	-19.45600	7.00400
H	-37.58600	-18.20900	7.09000
O	-24.85200	-11.87700	12.01500
H	-23.98300	-11.70300	11.65300
H	-25.33200	-11.05800	11.89200
O	-28.85700	-24.68100	-7.73200
H	-28.86900	-23.73700	-7.57500
H	-27.96500	-24.86400	-8.02600
O	-30.68400	-4.75800	3.69300
H	-29.78100	-4.72500	4.01000
H	-30.91000	-5.68800	3.70800
O	-28.92800	-6.00500	0.35400
H	-29.87900	-6.00400	0.24100
H	-28.62500	-6.75100	-0.16200

O	-25.04200	-28.08700	-3.47800
H	-25.80900	-28.64100	-3.33200
H	-24.63100	-28.01500	-2.61600
O	-33.03800	-21.79900	10.61300
H	-32.17500	-22.15500	10.82300
H	-33.61100	-22.13700	11.30000
O	-13.19300	-0.16900	1.41900
H	-12.55500	-0.28800	0.71500
H	-13.31100	0.78000	1.47800
O	-16.38400	-0.21600	1.35500
H	-16.72200	-0.33200	0.46700
H	-15.51500	-0.61700	1.33500
O	-20.52500	1.63800	-2.65500
H	-21.08900	1.54700	-3.42300
H	-20.64000	0.81800	-2.17500
O	-15.90200	0.93800	7.31400
H	-15.91300	0.54300	6.44200
H	-15.74000	0.20400	7.90700
O	-22.97900	3.13300	0.18300
H	-22.18700	2.79200	-0.23200
H	-22.73900	3.24200	1.10400
O	-14.94500	5.30500	-7.34400
H	-15.59700	5.89000	-7.73000
H	-14.15100	5.83500	-7.28400
O	-21.02000	2.39400	-6.94500
H	-20.24700	1.84700	-7.08400
H	-20.66700	3.25800	-6.73300
O	-19.18100	3.72100	6.26200
H	-19.47200	4.62600	6.14600
H	-18.54600	3.76300	6.97600
O	-18.25600	1.09900	2.85200
H	-17.65900	0.87500	2.13800
H	-18.19800	0.35800	3.45400
O	-13.63900	-2.44300	-1.50600
H	-12.85300	-2.86100	-1.85700
H	-13.37500	-1.53900	-1.33700
O	-13.46800	2.99400	-6.80700
H	-14.10300	3.06700	-6.09500
H	-13.79800	3.58300	-7.48500
O	-8.23800	-2.50600	-0.60200
H	-7.50500	-3.07600	-0.36900
H	-8.99400	-2.89400	-0.16200
O	-16.62000	-0.08300	-3.40600
H	-16.98500	-0.61000	-2.69500
H	-15.76400	-0.47300	-3.57900
O	-10.71500	-8.79400	3.85700
H	-11.04700	-7.92400	3.63300
H	-11.31100	-9.11000	4.53700
O	-16.98000	-5.05400	-8.41000
H	-16.81200	-4.18600	-8.04300
H	-17.17000	-4.89100	-9.33400
O	-14.22200	3.40800	3.25700
H	-14.52200	4.31000	3.14800
H	-14.56700	3.14100	4.10900
O	-20.77100	-0.14100	-0.71000
H	-21.69600	-0.27300	-0.50200
H	-20.52300	0.63800	-0.21300
O	-10.82300	-4.07400	0.93600
H	-11.73100	-4.14300	0.64200
H	-10.87400	-3.59700	1.76500
O	-17.00100	-2.59800	-7.42500
H	-17.12100	-2.15300	-6.58700
H	-17.69600	-2.25000	-7.98400
O	-19.24000	10.17200	-3.77600
H	-19.31800	11.12300	-3.69700
H	-19.84400	9.82700	-3.11900
O	-16.74800	-2.65500	8.88000
H	-17.52500	-2.09600	8.87000
H	-16.10900	-2.17000	9.40100
O	-8.95600	-5.87300	2.71400
H	-9.59900	-5.57900	2.06800
H	-9.46100	-5.98700	3.52000
O	-11.30200	-2.12600	-6.59700
H	-10.39100	-1.89300	-6.77200
H	-11.81100	-1.54300	-7.16000
O	-18.49400	0.15300	8.44200
H	-17.75200	0.69200	8.16500
H	-19.20000	0.78000	8.59900
O	-13.01800	2.29600	7.03900
H	-12.61000	1.85400	6.29400
H	-13.74100	1.72200	7.29200
O	-16.66500	-2.76600	4.41200
H	-15.81900	-2.34300	4.55600
H	-16.72500	-3.42300	5.10600
O	-13.51500	-5.10600	6.07000
H	-13.75900	-4.35200	5.53400
H	-13.23900	-4.72800	6.90500
O	-11.56100	1.43800	4.74800
H	-11.35000	2.08100	4.07200
H	-11.60800	0.60400	4.28000
O	-13.95900	-5.95000	1.65200
H	-14.82000	-5.65200	1.94400
H	-13.93300	-5.73300	0.72000
O	-17.14300	2.59400	-1.94100
H	-16.67900	2.87300	-2.73000
H	-17.40700	1.69200	-2.12300
O	-20.25000	2.14100	0.99700
H	-19.68000	2.57000	0.35900
H	-19.65900	1.85900	1.69600
O	-18.20600	-1.28400	-5.13200
H	-17.50300	-0.83200	-4.66400
H	-18.53800	-1.92600	-4.50400
O	-19.75800	-2.69300	9.24300
H	-20.38800	-2.01100	9.47700
H	-19.86400	-2.80400	8.29900
O	-24.46300	-3.02600	0.17000
H	-25.25300	-2.77000	-0.30600
H	-23.97200	-2.21100	0.27500
O	-22.51200	-2.66100	-2.87200
H	-23.30900	-2.54900	-2.35300
H	-22.73900	-2.32200	-3.73800
O	-16.68100	3.86300	5.07600
H	-16.61700	4.32500	5.91200
H	-17.39000	3.23300	5.20500
O	-16.96600	5.53000	-5.05600
H	-17.10200	6.37500	-4.62700
H	-16.42500	5.73200	-5.81900
O	-20.48000	-8.39100	11.60600

H	-20.87300	-8.94400	12.28100
H	-19.66300	-8.08000	11.99600
O	-23.51300	3.04800	-4.48300
H	-23.00100	3.84200	-4.33200
H	-22.86100	2.35000	-4.54900
O	-21.70400	6.16400	-2.40100
H	-21.52700	7.08600	-2.58800
H	-20.90800	5.85000	-1.97100
O	-17.56300	-6.97500	9.19800
H	-16.82200	-6.37400	9.11800
H	-17.33100	-7.71700	8.64000
O	-22.41900	-1.78900	7.47600
H	-22.53300	-2.69100	7.77600
H	-23.27400	-1.54300	7.12400
O	-15.34000	6.15300	9.07400
H	-15.78300	5.36600	8.75700
H	-14.67800	6.34000	8.40900
O	-20.58100	7.68200	3.60000
H	-20.03000	7.55400	2.82800
H	-21.42000	7.98200	3.25000
O	-13.24100	6.43300	7.10300
H	-13.26000	7.25400	6.61100
H	-12.31900	6.30800	7.32800
O	-16.78400	3.94700	7.94500
H	-16.25100	3.18600	7.71500
H	-17.31300	3.65500	8.68700
O	-13.61700	-10.94700	3.47400
H	-13.96100	-10.05700	3.39800
H	-13.24900	-11.13700	2.61100
O	-25.54900	1.37700	-3.70700
H	-24.71100	1.78600	-3.92400
H	-25.37100	0.43700	-3.73700
O	-13.65200	-0.95600	-8.01800
H	-14.16700	-0.18500	-7.78000
H	-13.92400	-1.62900	-7.39500
O	-16.06700	-6.11100	-2.96000
H	-16.92300	-6.50000	-3.13900
H	-15.65500	-6.03200	-3.82000
O	-14.53700	-8.57600	1.43900
H	-15.04100	-8.61300	0.62600
H	-14.24400	-7.66700	1.49900
O	-18.29300	-8.49200	0.44600
H	-17.42800	-8.68800	0.08600
H	-18.22500	-8.72100	1.37300
O	-13.86300	-3.08300	-6.19300
H	-14.16800	-2.76900	-5.34100
H	-12.91000	-3.00500	-6.15000
O	-15.41900	3.07600	-4.54300
H	-15.94700	2.34100	-4.85600
H	-15.92000	3.85300	-4.78900
O	-10.44000	1.27100	0.57300
H	-11.09700	1.96100	0.66200
H	-9.63600	1.66000	0.91700
O	-20.68700	-6.26200	-2.18300
H	-20.11300	-6.48500	-2.91500
H	-20.10000	-6.19300	-1.43000
O	-11.62900	-0.37500	-1.37600
H	-11.11600	0.25800	-0.87400
H	-10.97900	-0.97500	-1.74200
O	-20.36000	-4.23200	-8.16300
H	-21.19600	-3.89400	-7.84200
H	-20.50000	-4.36700	-9.10000
O	-18.38500	-9.38800	-3.45500
H	-19.03800	-9.70300	-4.07900
H	-18.42700	-10.01000	-2.72900
O	-14.36700	9.60400	-8.62000
H	-15.04100	9.90700	-8.01200
H	-13.98000	10.40700	-8.97000
O	-15.15100	-2.90000	0.87000
H	-16.06900	-2.67800	0.71200
H	-14.73500	-2.82500	0.01100
O	-14.36700	-1.50100	-4.00000
H	-13.62000	-0.98900	-4.30900
H	-14.09800	-1.82200	-3.14000
O	-16.09500	0.99500	-7.01700
H	-17.00400	0.71100	-6.92000
H	-16.06500	1.41500	-7.87700
O	-11.92000	-4.46500	-2.01800
H	-12.65700	-4.97700	-1.68700
H	-11.66700	-4.90500	-2.83000
O	-11.81800	-11.68700	-1.42400
H	-11.51200	-12.54800	-1.71100
H	-11.09300	-11.09600	-1.62400
O	-25.52600	-1.28400	-3.03600
H	-25.72300	-1.87700	-3.76100
H	-26.08100	-1.58900	-2.31800
O	-17.54600	-1.39200	-0.91600
H	-17.49100	-2.34500	-0.99200
H	-18.35700	-1.23600	-0.43200
O	-16.39700	2.53600	-9.67900
H	-16.83500	1.82000	-10.13800
H	-15.65600	2.76400	-10.24000
O	-19.60700	-2.77400	-3.15700
H	-20.42000	-2.47500	-2.75100
H	-19.11100	-3.16900	-2.44000
O	-17.45600	7.22100	-1.22400
H	-17.49900	7.44800	-2.15300
H	-16.54000	6.98800	-1.07500
O	-14.01000	-2.31000	5.67400
H	-13.65500	-2.08300	6.53400
H	-13.36200	-1.97800	5.05200
O	-20.11600	-5.20200	2.45900
H	-19.47600	-5.34400	1.76100
H	-20.94800	-5.48900	2.08300
O	-19.34000	4.09800	-1.42800
H	-18.54600	3.60300	-1.62900
H	-20.05000	3.57000	-1.79100
O	-14.52000	6.48000	2.92400
H	-14.46600	7.04900	3.69100
H	-15.38900	6.65000	2.55900
O	-23.03100	-1.75800	-5.35200
H	-23.96200	-1.81900	-5.13500
H	-22.93600	-2.28000	-6.14800
O	-23.11300	-5.23200	-2.76400
H	-22.85200	-4.31800	-2.87300
H	-22.29100	-5.70000	-2.61400
O	-21.64000	-3.81900	4.40600
H	-20.96800	-4.02700	3.75700

H	-22.46700	-4.01600	3.96700
O	-15.73800	-7.19200	6.42300
H	-16.38500	-6.49300	6.32900
H	-14.96800	-6.75900	6.79000
O	-12.33000	3.07200	9.49800
H	-12.48100	2.84300	8.58100
H	-12.33900	4.02900	9.51100
O	-15.56100	0.64600	4.48200
H	-14.63200	0.79700	4.30900
H	-15.98200	0.73400	3.62700
O	-16.76200	4.67100	2.25200
H	-16.90100	4.30300	3.12500
H	-17.55100	5.18500	2.08100
O	-16.16800	9.77200	-6.40500
H	-15.99200	9.30000	-5.59100
H	-16.85400	10.39800	-6.17700
O	-15.21200	4.04900	-0.35900
H	-15.54700	4.00900	0.53700
H	-15.71200	3.38300	-0.83200
O	-12.27600	1.39400	-10.08500
H	-12.89300	0.66900	-10.18200
H	-12.74400	2.15500	-10.42900
O	-18.60900	-0.83200	4.65500
H	-17.89900	-1.47300	4.61300
H	-18.65200	-0.58100	5.57700
O	-10.62100	6.10400	7.57500
H	-10.67900	5.42000	6.90800
H	-10.21900	6.84600	7.12300
O	-19.89600	-3.84600	6.47400
H	-20.43300	-4.29700	7.12600
H	-20.48500	-3.71100	5.73100
O	-21.60200	12.92100	0.36900
H	-21.36200	13.55200	1.04700
H	-22.44500	12.56900	0.65500
O	-28.96600	0.40100	-0.86900
H	-28.27900	0.08700	-1.45700
H	-29.12100	1.30500	-1.14200
O	-24.16200	-4.71000	3.55900
H	-24.24700	-5.31800	2.82500
H	-24.98400	-4.21900	3.56000
O	-17.08900	8.72800	2.77300
H	-17.16200	9.53400	3.28500
H	-17.53100	8.92800	1.94800
O	-17.30000	7.96600	-3.71700
H	-18.08300	8.50800	-3.62300
H	-16.60000	8.48300	-3.31700
O	-13.99000	-6.22500	-1.07400
H	-13.49300	-6.97800	-1.39300
H	-14.76100	-6.18800	-1.63900
O	-12.90700	2.53200	-3.50000
H	-13.84100	2.65200	-3.66900
H	-12.72900	1.63000	-3.76400
O	-17.40700	-4.08300	-1.34600
H	-17.77700	-4.63600	-0.65800
H	-16.91100	-4.68700	-1.89900
O	-9.52000	-1.62600	-2.96500
H	-9.05400	-0.79300	-3.03900
H	-9.06900	-2.09200	-2.26000
O	-19.57800	6.37700	5.74900
H	-18.87700	7.01000	5.90200
H	-20.04800	6.71800	4.98900
O	-11.45200	4.71800	-5.96600
H	-11.17600	4.60400	-5.05700
H	-12.32200	4.32000	-6.00500
O	-11.54800	-9.25800	1.07400
H	-12.46200	-9.02800	1.24300
H	-11.11900	-9.17400	1.92600
O	-11.77800	-1.06900	3.57400
H	-12.43000	-0.82100	2.92000
H	-11.40000	-1.88300	3.24100
O	-14.81700	8.96700	-2.32300
H	-14.63500	8.14900	-1.86000
H	-13.99400	9.18400	-2.76000
O	-14.48300	7.89400	-5.06100
H	-13.95400	7.38300	-5.67400
H	-14.22000	7.57800	-4.19700
O	-15.72900	-0.32700	10.06100
H	-16.40500	-0.42800	10.73100
H	-14.90600	-0.48300	10.52500
O	-23.98100	5.91000	2.68200
H	-23.39600	5.16500	2.81500
H	-24.17800	6.22100	3.56600
O	-21.12700	8.94500	-2.30100
H	-21.79600	9.43000	-2.78500
H	-21.26600	9.19200	-1.38700
O	-29.38900	-1.51900	1.40300
H	-29.35100	-2.05300	2.19600
H	-29.89500	-2.04600	0.78400
O	-16.98700	10.98000	4.29700
H	-16.43500	11.14300	5.06200
H	-16.64600	11.57000	3.62500
O	-22.01000	-6.67300	4.89900
H	-21.94600	-5.81100	4.48700
H	-22.07300	-7.28500	4.16600
O	-22.53500	-4.60000	8.54600
H	-21.90800	-4.98300	9.16000
H	-23.39200	-4.81900	8.91200
O	-12.13500	5.80600	10.15200
H	-11.59100	6.41900	9.65700
H	-12.71700	6.36400	10.66800
O	-25.75800	-2.79300	-5.52200
H	-25.46300	-3.70200	-5.57500
H	-26.65900	-2.81400	-5.84400
O	-22.22900	-4.71700	0.46100
H	-22.95000	-4.10400	0.32200
H	-21.44000	-4.18100	0.38300
O	-19.42600	-7.38200	6.73500
H	-18.90300	-7.13600	7.49800
H	-19.64500	-6.55000	6.31500
O	-24.03000	-5.11100	-5.39800
H	-24.35800	-5.99200	-5.58000
H	-23.67600	-5.16700	-4.51000
O	-20.91500	-4.82400	-5.03000
H	-20.57600	-4.29600	-5.75300
H	-20.46800	-4.48600	-4.25500
O	-18.22100	-6.44000	4.42000
H	-18.56100	-7.24000	4.82200
H	-18.92700	-6.14000	3.84800

O	-17.56900	-9.35500	2.87300
H	-16.74700	-9.82900	2.75100
H	-17.32600	-8.57600	3.37300
O	-18.92900	-6.32700	-6.83100
H	-18.20800	-5.94900	-7.33500
H	-19.55200	-5.60800	-6.72800
O	-14.78500	-5.62200	-5.89500
H	-14.56900	-4.74000	-6.19600
H	-15.26600	-6.01400	-6.62500
O	-20.04500	-2.66400	0.68200
H	-20.19100	-2.35000	1.57400
H	-20.52300	-2.04500	0.12900
O	-18.18600	-5.67300	0.73000
H	-18.20000	-6.56900	0.39300
H	-17.46900	-5.66600	1.36400
O	-16.33300	-5.24000	2.77500
H	-16.59100	-4.31800	2.77400
H	-16.86500	-5.63700	3.46500
O	-18.78200	-6.79200	-4.00100
H	-18.66100	-7.72000	-3.80200
H	-19.07200	-6.77800	-4.91300
O	-15.91300	-9.10600	-0.86500
H	-15.89100	-9.04200	-1.81900
H	-15.49600	-9.94600	-0.67100
O	-10.40100	7.18200	-7.34600
H	-9.70600	7.80300	-7.12700
H	-10.05900	6.33200	-7.07100
O	-16.84000	8.54600	6.31200
H	-16.53100	8.30000	7.18400
H	-16.04200	8.75000	5.82400
O	-16.81800	10.69900	-1.45800
H	-17.25700	10.20700	-0.76500
H	-16.06700	10.15700	-1.69900
O	-24.33900	6.89100	-1.45500
H	-23.51300	6.59100	-1.83500
H	-24.68900	6.12100	-1.00600
O	-19.37000	6.24300	0.61400
H	-19.99200	5.58000	0.31500
H	-18.70000	6.26900	-0.06800
O	-26.13800	4.80900	-0.49600
H	-26.97500	4.38100	-0.67800
H	-26.16500	5.00200	0.44100
O	-19.29800	-5.27900	10.20800
H	-19.24900	-4.40200	9.82500
H	-18.75600	-5.82100	9.63500
O	-8.81300	5.26500	9.48800
H	-9.60300	5.24800	8.94800
H	-9.04900	5.80400	10.24300
O	-8.69300	2.11500	6.80100
H	-8.64300	1.95000	7.74300
H	-8.80100	1.24700	6.41100
O	-19.80900	4.79400	-5.91000
H	-19.66000	5.47700	-6.56300
H	-19.00200	4.77400	-5.39500
O	-11.40200	-1.90200	8.11700
H	-10.50100	-2.04200	7.82500
H	-11.32800	-1.73700	9.05700
O	-17.06800	-4.52600	6.96200
H	-17.93800	-4.23200	6.69100
H	-16.83200	-3.94100	7.68300
O	-8.94200	-0.34200	5.51300
H	-8.46700	-0.15000	4.70500
H	-9.86600	-0.28600	5.26800
O	-22.05400	0.82000	-4.90400
H	-22.29900	-0.08400	-4.70600
H	-21.88400	0.82200	-5.84600
O	-14.36400	-8.30800	4.47900
H	-13.84600	-7.57100	4.15300
H	-14.71900	-8.00100	5.31400
O	-22.78400	-3.44700	-7.40500
H	-23.47700	-3.37300	-8.06100
H	-23.02900	-4.21100	-6.88300
O	-14.11300	-11.88800	-0.00100
H	-13.83300	-12.55500	0.62500
H	-13.32800	-11.69500	-0.51400
O	-16.31000	7.28800	-8.43900
H	-16.88200	7.90700	-8.89200
H	-15.73600	7.83800	-7.90500
O	-10.24200	-3.46600	3.74600
H	-9.29000	-3.36600	3.76700
H	-10.41200	-4.25800	4.25600
O	-12.22100	-6.38000	3.93200
H	-12.81600	-6.10000	3.23700
H	-12.50600	-5.89100	4.70400
O	-13.09000	7.57200	-7.30800
H	-12.15700	7.50700	-7.10400
H	-13.17800	8.40800	-7.76600
O	-10.46100	6.88900	-10.34700
H	-10.54000	6.55500	-9.45400
H	-9.64000	7.38100	-10.34800
O	-7.76300	-0.24500	2.86000
H	-7.79600	-1.18400	3.04600
H	-7.15300	-0.16700	2.12700
O	-15.92500	-1.37600	-11.09700
H	-14.97900	-1.50500	-11.02800
H	-16.30300	-2.11400	-10.62000
O	-7.61400	-3.45400	2.90600
H	-8.13000	-4.23800	2.71900
H	-6.73000	-3.78200	3.07100
O	-9.95300	-9.71000	-1.13400
H	-10.15000	-10.01800	-0.25000
H	-9.34300	-8.98400	-1.00400
O	-16.04100	2.67600	10.36300
H	-15.12100	2.85400	10.55700
H	-16.07600	1.73200	10.21100
O	-26.82300	-2.13000	-0.71200
H	-27.14300	-2.98300	-1.00400
H	-27.36900	-1.91500	0.04400
O	-16.12400	-8.90700	-5.00700
H	-16.93400	-9.25200	-4.63200
H	-15.43600	-9.21600	-4.41800
O	-14.19500	-9.82900	-3.38500
H	-13.64200	-9.41000	-2.72600
H	-13.95800	-10.75500	-3.34200
O	-18.17200	-3.84000	12.39600
H	-18.27200	-2.89100	12.32700
H	-18.39000	-4.16900	11.52300
O	-18.97100	-6.59200	-11.02000

H	-18.10900	-6.26600	-10.76100
H	-18.78600	-7.37900	-11.53300
O	-26.95400	1.49000	1.25800
H	-27.64800	1.07600	0.74500
H	-26.20700	1.53200	0.66100
O	-10.81700	-7.71900	-3.87700
H	-11.39800	-7.06100	-4.25900
H	-11.08400	-8.54100	-4.28800
O	-12.36700	-8.31900	-1.67500
H	-11.86700	-8.20700	-2.48300
H	-11.72400	-8.62200	-1.03500
O	-18.30900	-0.41100	11.21200
H	-18.79200	0.38900	11.41900
H	-18.22300	-0.40000	10.25900
O	-25.04000	1.42200	-0.86600
H	-25.06300	1.52100	-1.81700
H	-24.73000	2.26900	-0.54400
O	-22.38200	3.48000	3.24900
H	-21.42700	3.44800	3.18200
H	-22.58000	3.01900	4.06400
O	-21.79200	2.05000	5.81600
H	-20.97200	2.54400	5.84000
H	-21.51800	1.13300	5.78800
O	-12.03500	-5.55900	-4.82100
H	-12.92200	-5.27400	-5.03900
H	-11.62000	-5.72000	-5.66800
O	-8.68400	-5.53400	-1.10600
H	-7.90800	-5.04500	-0.83300
H	-9.40900	-5.10900	-0.64800
O	-6.24600	-4.82200	-0.04800
H	-5.65200	-5.06200	-0.75900
H	-6.17400	-5.54100	0.57900
O	-24.02700	9.02200	0.26600
H	-24.16100	8.26200	-0.30000
H	-24.44300	9.74600	-0.20100
O	-12.89100	4.20500	-10.72600
H	-12.18700	4.41000	-10.11100
H	-13.61400	4.77300	-10.46100
O	-12.06100	-0.04400	-4.60600
H	-12.11700	0.41300	-5.44500
H	-11.50800	-0.80400	-4.78400
O	-8.25000	-4.25500	-3.78100
H	-8.50700	-4.65500	-2.95000
H	-8.87500	-3.54000	-3.90600
O	-20.87000	9.91900	0.47800
H	-20.98600	10.86300	0.37600
H	-21.69000	9.61600	0.86600
O	-10.98200	4.64400	-8.67600
H	-10.14700	4.21200	-8.85900
H	-11.14400	4.47200	-7.74900
O	-11.32700	1.14700	-7.31100
H	-11.38300	0.98100	-8.25200
H	-11.95300	1.85500	-7.15500
O	-26.53800	-3.61600	6.51500
H	-25.89200	-4.12600	7.00400
H	-26.74600	-2.87900	7.09000
O	-14.01300	3.45200	12.01500
H	-13.14400	3.62600	11.65300
H	-14.49200	4.27100	11.89200
O	-18.01800	-9.35200	-7.73200
H	-18.03000	-8.40800	-7.57500
H	-17.12500	-9.53500	-8.02600
O	-19.84400	10.57200	3.69300
H	-18.94100	10.60400	4.01000
H	-20.07000	9.64200	3.70800
O	-18.08900	9.32500	0.35400
H	-19.03900	9.32500	0.24100
H	-17.78600	8.57800	-0.16200
O	-14.20300	-12.75800	-3.47800
H	-14.96900	-13.31200	-3.33200
H	-13.79200	-12.68600	-2.61600
O	-22.19800	-6.47000	10.61300
H	-21.33500	-6.82600	10.82300
H	-22.77200	-6.80800	11.30000
O	-2.35400	15.16100	1.41900
H	-1.71600	15.04100	0.71500
H	-2.47100	16.10900	1.47800
O	-5.54500	15.11300	1.35500
H	-5.88300	14.99800	0.46700
H	-4.67600	14.71300	1.33500
O	-9.68600	16.96700	-2.65500
H	-10.24900	16.87600	-3.42300
H	-9.80100	16.14700	-2.17500
O	-5.06300	16.26700	7.31400
H	-5.07400	15.87200	6.44200
H	-4.90100	15.53400	7.90700
O	-12.13900	18.46200	0.18300
H	-11.34700	18.12100	-0.23200
H	-11.90000	18.57200	1.10400
O	-4.10600	20.63400	-7.34400
H	-4.75700	21.21900	-7.73000
H	-3.31200	21.16500	-7.28400
O	-10.18000	17.72300	-6.94500
H	-9.40700	17.17600	-7.08400
H	-9.82800	18.58700	-6.73300
O	-8.34200	19.05100	6.26200
H	-8.63300	19.95500	6.14600
H	-7.70600	19.09200	6.97600
O	-7.41600	16.42800	2.85200
H	-6.82000	16.20400	2.13800
H	-7.35900	15.68700	3.45400
O	-7.84400	13.88200	-9.31300
H	-7.59100	13.36000	-10.07500
H	-8.78100	13.71900	-9.21000
O	-2.79900	12.88600	-1.50600
H	-2.01300	12.46800	-1.85700
H	-2.53600	13.79000	-1.33700
O	-2.62800	18.32300	-6.80700
H	-3.26400	18.39700	-6.09500
H	-2.95900	18.91200	-7.48500
O	-8.12400	15.78500	-7.11100
H	-8.24100	15.11000	-6.44300
H	-8.01400	15.29800	-7.92700
O	-5.78100	15.24600	-3.40600
H	-6.14500	14.71900	-2.69500
H	-4.92400	14.85600	-3.57900
O	-6.14000	10.27500	-8.41000
H	-5.97300	11.14300	-8.04300

H	-6.33100	10.43800	-9.33400
O	-3.38300	18.73700	3.25700
H	-3.68300	19.63900	3.14800
H	-3.72800	18.47000	4.10900
O	-9.93200	15.18800	-0.71000
H	-10.85700	15.05600	-0.50200
H	-9.68300	15.96800	-0.21300
O	-6.16200	12.73100	-7.42500
H	-6.28200	13.17700	-6.58700
H	-6.85600	13.07900	-7.98400
O	-12.57100	14.83100	0.12500
H	-12.44200	15.08500	1.03900
H	-13.25100	15.42400	-0.19500
O	-8.40100	25.50100	-3.77600
H	-8.47800	26.45200	-3.69700
H	-9.00500	25.15600	-3.11900
O	-5.90900	12.67400	8.88000
H	-6.68600	13.23300	8.87000
H	-5.27000	13.16000	9.40100
O	-0.46300	13.20300	-6.59700
H	0.44900	13.43600	-6.77200
H	-0.97200	13.78700	-7.16000
O	-7.65400	15.48300	8.44200
H	-6.91300	16.02100	8.16500
H	-8.36100	16.10900	8.59900
O	-2.17900	17.62500	7.03900
H	-1.77100	17.18400	6.29400
H	-2.90100	17.05100	7.29200
O	-5.82600	12.56300	4.41200
H	-4.98000	12.98700	4.55600
H	-5.88600	11.90600	5.10600
O	-1.85600	18.18600	0.79400
H	-2.48500	18.35000	0.09200
H	-2.24300	18.60000	1.56500
O	2.02300	19.12100	-1.85100
H	1.33800	19.50100	-2.40200
H	2.10700	19.73100	-1.11800
O	-0.72200	16.76700	4.74800
H	-0.51000	17.41000	4.07200
H	-0.76900	15.93300	4.28000
O	-6.30300	17.92300	-1.94100
H	-5.83900	18.20200	-2.73000
H	-6.56800	17.02100	-2.12300
O	-9.41100	17.47000	0.99700
H	-8.84100	17.89900	0.35900
H	-8.82000	17.18800	1.69600
O	-7.36600	14.04500	-5.13200
H	-6.66400	14.49700	-4.66400
H	-7.69800	13.40400	-4.50400
O	-8.91800	12.63600	9.24300
H	-9.54800	13.31800	9.47700
H	-9.02500	12.52500	8.29900
O	-13.62400	12.30400	0.17000
H	-14.41400	12.55900	-0.30600
H	-13.13300	13.11800	0.27500
O	0.39300	19.64400	2.92000
H	1.21400	19.24200	2.63900
H	0.38900	19.54100	3.87200
O	-11.67200	12.66800	-2.87200
H	-12.46900	12.78000	-2.35300
H	-11.90000	13.00700	-3.73800
O	-5.84200	19.19300	5.07600
H	-5.77700	19.65500	5.91200
H	-6.55100	18.56200	5.20500
O	-15.34000	12.55700	2.60100
H	-15.08900	13.44100	2.86700
H	-14.80400	12.37700	1.82900
O	8.48300	15.98800	-0.73100
H	7.73000	15.41900	-0.88700
H	8.61800	15.95400	0.21600
O	-6.12700	20.85900	-5.05600
H	-6.26200	21.70500	-4.62700
H	-5.58600	21.06100	-5.81900
O	-9.64000	6.93900	11.60600
H	-10.03400	6.38500	12.28100
H	-8.82400	7.25000	11.99600
O	-12.67400	18.37700	-4.48300
H	-12.16200	19.17100	-4.33200
H	-12.02200	17.68000	-4.54900
O	-10.86500	21.49400	-2.40100
H	-10.68800	22.41500	-2.58800
H	-10.06900	21.17900	-1.97100
O	-11.57900	13.54000	7.47600
H	-11.69400	12.63900	7.77600
H	-12.43500	13.78600	7.12400
O	-4.50000	21.48200	9.07400
H	-4.94400	20.69500	8.75700
H	-3.83800	21.67000	8.40900
O	-9.74100	23.01100	3.60000
H	-9.19100	22.88300	2.82800
H	-10.58000	23.31100	3.25000
O	-2.40200	21.76200	7.10300
H	-2.42000	22.58300	6.61100
H	-1.48000	21.63700	7.32800
O	-5.94500	19.27600	7.94500
H	-5.41200	18.51500	7.71500
H	-6.47300	18.98400	8.68700
O	-14.70900	16.70600	-3.70700
H	-13.87200	17.11600	-3.92400
H	-14.53100	15.76600	-3.73700
O	-2.81300	14.37300	-8.01800
H	-3.32800	15.14400	-7.78000
H	-3.08500	13.70000	-7.39500
O	2.16100	17.12700	-6.08300
H	1.38400	16.78800	-6.52800
H	2.84000	17.13200	-6.75800
O	-3.02400	12.24600	-6.19300
H	-3.32900	12.56100	-5.34100
H	-2.07100	12.32400	-6.15000
O	-4.57900	18.40500	-4.54300
H	-5.10700	17.67000	-4.85600
H	-5.08100	19.18300	-4.78900
O	0.40000	16.60000	0.57300
H	-0.25800	17.29000	0.66200
H	1.20400	16.99000	0.91700
O	-0.79000	14.95400	-1.37600
H	-0.27600	15.58700	-0.87400
H	-0.14000	14.35500	-1.74200

O	-9.52100	11.09700	-8.16300
H	-10.35700	11.43500	-7.84200
H	-9.66000	10.96200	-9.10000
O	-6.43400	16.22300	-11.54000
H	-7.34100	15.99200	-11.34100
H	-5.93700	15.42700	-11.35200
O	-3.52700	24.93300	-8.62000
H	-4.20100	25.23600	-8.01200
H	-3.14100	25.73600	-8.97000
O	-4.31200	12.42900	0.87000
H	-5.22900	12.65100	0.71200
H	-3.89600	12.50400	0.01100
O	-3.52800	13.82800	-4.00000
H	-2.78100	14.34100	-4.30900
H	-3.25900	13.50700	-3.14000
O	-5.25500	16.32400	-7.01700
H	-6.16400	16.04000	-6.92000
H	-5.22600	16.74400	-7.87700
O	-0.02300	19.93500	-3.24800
H	-0.47700	19.09400	-3.19200
H	-0.65400	20.57100	-2.91100
O	-1.58000	21.77300	-2.31300
H	-1.28800	22.33300	-3.03200
H	-1.07000	22.06500	-1.55800
O	-14.68700	14.04500	-3.03600
H	-14.88400	13.45200	-3.76100
H	-15.24200	13.74000	-2.31800
O	-6.70700	13.93700	-0.91600
H	-6.65200	12.98500	-0.99200
H	-7.51800	14.09300	-0.43200
O	-5.55800	17.86500	-9.67900
H	-5.99600	17.14900	-10.13800
H	-4.81700	18.09300	-10.24000
O	-8.76800	12.55500	-3.15700
H	-9.58100	12.85400	-2.75100
H	-8.27200	12.16000	-2.44000
O	-6.61700	22.55100	-1.22400
H	-6.65900	22.77700	-2.15300
H	-5.70100	22.31700	-1.07500
O	-3.17000	13.01900	5.67400
H	-2.81500	13.24600	6.53400
H	-2.52300	13.35100	5.05200
O	2.56000	16.14100	-3.62100
H	2.42100	16.33100	-4.54900
H	2.20300	16.90300	-3.16400
O	2.58600	18.20900	1.39000
H	3.02000	18.11600	2.23900
H	3.30000	18.18400	0.75200
O	-8.50000	19.42700	-1.42800
H	-7.70600	18.93200	-1.62900
H	-9.21100	18.89900	-1.79100
O	-3.68100	21.80900	2.92400
H	-3.62700	22.37800	3.69100
H	-4.54900	21.97900	2.55900
O	-12.19200	13.57100	-5.35200
H	-13.12200	13.51000	-5.13500
H	-12.09600	13.04900	-6.14800
O	-12.27300	10.09700	-2.76400
H	-12.01300	11.01100	-2.87300
H	-11.45200	9.62900	-2.61400
O	3.44800	18.25100	3.92400
H	3.64200	19.15600	4.16900
H	3.34800	17.79200	4.75700
O	-1.49000	18.40200	9.49800
H	-1.64200	18.17300	8.58100
H	-1.50000	19.35900	9.51100
O	-4.72200	15.97500	4.48200
H	-3.79300	16.12600	4.30900
H	-5.14300	16.06300	3.62700
O	-5.92300	20.00000	2.25200
H	-6.06200	19.63200	3.12500
H	-6.71200	20.51500	2.08100
O	-5.32800	25.10100	-6.40500
H	-5.15300	24.63000	-5.59100
H	-6.01500	25.72700	-6.17700
O	-4.37300	19.37800	-0.35900
H	-4.70800	19.33800	0.53700
H	-4.87200	18.71300	-0.83200
O	-10.39800	14.78500	5.16600
H	-10.53600	14.05300	5.76700
H	-9.48800	14.69600	4.88500
O	-1.43700	16.72400	-10.08500
H	-2.05400	15.99800	-10.18200
H	-1.90500	17.48500	-10.42900
O	-7.77000	14.49700	4.65500
H	-7.06000	13.85600	4.61300
H	-7.81300	14.74800	5.57700
O	0.21900	21.43400	7.57500
H	0.16000	20.74900	6.90800
H	0.62100	22.17500	7.12300
O	-10.76200	28.25000	0.36900
H	-10.52200	28.88200	1.04700
H	-11.60500	27.89900	0.65500
O	-9.64700	10.94200	-10.96300
H	-10.52400	10.82100	-11.32700
H	-9.17100	10.15100	-11.21500
O	0.22100	19.39700	5.80700
H	-0.66700	19.06000	5.92900
H	0.79000	18.66600	6.04400
O	-18.12700	15.73000	-0.86900
H	-17.43900	15.41600	-1.45700
H	-18.28200	16.63400	-1.14200
O	-6.24900	24.05800	2.77300
H	-6.32200	24.86300	3.28500
H	-6.69200	24.25700	1.94800
O	-6.46000	23.29500	-3.71700
H	-7.24400	23.83700	-3.62300
H	-5.76100	23.81200	-3.31700
O	4.70200	14.80800	0.56800
H	4.69100	15.69100	0.19800
H	3.83400	14.45500	0.37400
O	-3.79000	23.95600	4.92900
H	-3.07700	24.59100	4.85600
H	-4.28100	24.05300	4.11300
O	-2.06800	17.86100	-3.50000
H	-3.00200	17.98200	-3.66900
H	-1.89000	16.95900	-3.76400
O	-6.56700	11.24600	-1.34600

H	-6.93700	10.69300	-0.65800
H	-6.07100	10.64200	-1.89900
O	-8.73800	21.70600	5.74900
H	-8.03700	22.33900	5.90200
H	-9.20900	22.04700	4.98900
O	-0.61300	20.04800	-5.96600
H	-0.33700	19.93300	-5.05700
H	-1.48200	19.64900	-6.00500
O	-0.93800	14.26000	3.57400
H	-1.59100	14.50800	2.92000
H	-0.56000	13.44700	3.24100
O	-3.97700	24.29600	-2.32300
H	-3.79500	23.47800	-1.86000
H	-3.15400	24.51300	-2.76000
O	-3.64300	23.22300	-5.06100
H	-3.11400	22.71200	-5.67400
H	-3.38000	22.90700	-4.19700
O	-4.88900	15.00200	10.06100
H	-5.56500	14.90200	10.73100
H	-4.06700	14.84600	10.52500
O	-13.14200	21.23900	2.68200
H	-12.55600	20.49400	2.81500
H	-13.33900	21.55000	3.56600
O	-10.28800	24.27500	-2.30100
H	-10.95700	24.75900	-2.78500
H	-10.42600	24.52100	-1.38700
O	-18.55000	13.81000	1.40300
H	-18.51100	13.27600	2.19600
H	-19.05600	13.28300	0.78400
O	-6.14700	26.30900	4.29700
H	-5.59500	26.47200	5.06200
H	-5.80700	26.89900	3.62500
O	-11.69500	10.72900	8.54600
H	-11.06800	10.34600	9.16000
H	-12.55300	10.51000	8.91200
O	-1.29600	21.13500	10.15200
H	-0.75200	21.74800	9.65700
H	-1.87700	21.69300	10.66800
O	-14.91900	12.53600	-5.52200
H	-14.62400	11.62700	-5.57500
H	-15.82000	12.51500	-5.84400
O	-3.01000	24.25700	1.28300
H	-3.13500	23.45600	0.77400
H	-2.35200	24.75200	0.79500
O	-0.94700	22.05800	2.91100
H	-1.89400	21.93900	2.83900
H	-0.59900	21.17000	2.99400
O	-13.19000	10.21800	-5.39800
H	-13.51900	9.33800	-5.58000
H	-12.83600	10.16300	-4.51000
O	-3.94200	21.85300	-0.88800
H	-4.09000	20.92500	-0.70800
H	-3.11700	21.87500	-1.37300
O	-10.07600	10.50500	-5.03000
H	-9.73600	11.03400	-5.75300
H	-9.62900	10.84300	-4.25500
O	-0.33900	22.67600	0.19800
H	0.58600	22.47000	0.06000
H	-0.50700	22.42800	1.10700
O	-8.08900	9.00300	-6.83100
H	-7.36800	9.38100	-7.33500
H	-8.71300	9.72100	-6.72800
O	-3.94600	9.70700	-5.89500
H	-3.73000	10.59000	-6.19600
H	-4.42700	9.31600	-6.62500
O	-9.20600	12.66600	0.68200
H	-9.35200	12.98000	1.57400
H	-9.68400	13.28400	0.12900
O	-0.54900	23.19500	-4.44600
H	-0.37600	22.62900	-5.19800
H	-0.05600	23.99500	-4.62800
O	-14.73200	15.20600	3.62100
H	-15.27600	15.98200	3.75300
H	-13.85400	15.55300	3.46300
O	5.30500	15.95800	-2.82700
H	5.74800	15.20900	-3.22500
H	4.42700	15.94900	-3.20900
O	0.43800	22.51100	-7.34600
H	1.13300	23.13200	-7.12700
H	0.78100	21.66100	-7.07100
O	-13.94500	14.33300	6.51400
H	-14.68700	14.48100	7.10000
H	-14.26600	14.59400	5.65000
O	2.33700	19.96100	-5.81700
H	2.86300	20.30000	-6.54200
H	2.25000	19.02600	-6.00100
O	-6.00100	23.87500	6.31200
H	-5.69200	23.63000	7.18400
H	-5.20300	24.07900	5.82400
O	-5.97900	26.02900	-1.45800
H	-6.41800	25.53600	-0.76500
H	-5.22800	25.48600	-1.69900
O	-13.49900	22.22000	-1.45500
H	-12.67300	21.92000	-1.83500
H	-13.85000	21.45000	-1.00600
O	-8.53100	21.57200	0.61400
H	-9.15300	20.90900	0.31500
H	-7.86100	21.59800	-0.06800
O	-15.29800	20.13800	-0.49600
H	-16.13500	19.71100	-0.67800
H	-15.32600	20.33200	0.44100
O	-8.45900	10.05100	10.20800
H	-8.41000	10.92700	9.82500
H	-7.91600	9.50800	9.63500
O	2.02700	20.59500	9.48800
H	1.23600	20.57700	8.94800
H	1.79000	21.13300	10.24300
O	2.14700	17.44400	6.80100
H	2.19600	17.27900	7.74300
H	2.03800	16.57700	6.41100
O	-8.97000	20.12300	-5.91000
H	-8.82100	20.80600	-6.56300
H	-8.16300	20.10300	-5.39500
O	-0.56300	13.42700	8.11700
H	0.33800	13.28800	7.82500
H	-0.48800	13.59200	9.05700
O	3.64800	19.86400	7.04500
H	3.35700	20.22700	7.88200

H	3.03600	19.15000	6.86700
O	4.84800	17.42900	-0.43400
H	5.68300	17.86800	-0.27100
H	4.98400	16.95300	-1.25400
O	1.89700	14.98700	5.51300
H	2.37300	15.18000	4.70500
H	0.97400	15.04300	5.26800
O	-11.21500	16.14900	-4.90400
H	-11.46000	15.24500	-4.70600
H	-11.04500	16.15100	-5.84600
O	-11.94500	11.88200	-7.40500
H	-12.63800	11.95700	-8.06100
H	-12.19000	11.11800	-6.88300
O	-5.47000	22.61800	-8.43900
H	-6.04300	23.23700	-8.89200
H	-4.89700	23.16700	-7.90500
O	-2.25100	22.90100	-7.30800
H	-1.31700	22.83600	-7.10400
H	-2.33800	23.73700	-7.76600
O	0.37800	22.21800	-10.34700
H	0.30000	21.88400	-9.45400
H	1.19900	22.71000	-10.34800
O	3.07600	15.08400	2.86000
H	3.04400	14.14600	3.04600
H	3.68600	15.16200	2.12700
O	-5.08600	13.95400	-11.09700
H	-4.14000	13.82400	-11.02800
H	-5.46400	13.21500	-10.62000
O	0.88700	5.61900	-1.13400
H	0.68900	5.31100	-0.25000
H	1.49700	6.34500	-1.00400
O	8.89400	16.14600	2.00100
H	9.62400	15.88400	2.56200
H	8.28400	15.40800	2.04100
O	6.97500	14.28600	2.59800
H	6.24900	14.15200	1.99000
H	6.56000	14.59400	3.40400
O	-5.20200	18.00500	10.36300
H	-4.28100	18.18300	10.55700
H	-5.23700	17.06100	10.21100
O	-6.90100	11.87900	-11.16100
H	-7.80800	11.58200	-11.08500
H	-6.62400	11.58200	-12.02800
O	-15.98300	13.19900	-0.71200
H	-16.30300	12.34600	-1.00400
H	-16.53000	13.41400	0.04400
O	-12.20500	15.91600	3.00500
H	-11.98700	16.84700	3.02400
H	-11.79300	15.55600	3.79100
O	-7.33300	11.48900	12.39600
H	-7.43300	12.43800	12.32700
H	-7.55100	11.16100	11.52300
O	-8.13100	8.73700	-11.02000
H	-7.26900	9.06300	-10.76100
H	-7.94600	7.95000	-11.53300
O	-16.11500	16.82000	1.25800
H	-16.80800	16.40500	0.74500
H	-15.36700	16.86200	0.66100
O	0.02200	7.61000	-3.87700
H	-0.55800	8.26900	-4.25900
H	-0.24500	6.78800	-4.28800
O	-7.47000	14.91900	11.21200
H	-7.95300	15.71900	11.41900
H	-7.38300	14.92900	10.25900
O	-14.20100	16.75100	-0.86600
H	-14.22400	16.85000	-1.81700
H	-13.89100	17.59800	-0.54400
O	-11.54200	18.80900	3.24900
H	-10.58800	18.77700	3.18200
H	-11.74100	18.34800	4.06400
O	-10.95200	17.37900	5.81600
H	-10.13300	17.87400	5.84000
H	-10.67900	16.46200	5.78800
O	-1.19600	9.77000	-4.82100
H	-2.08300	10.05500	-5.03900
H	-0.78000	9.61000	-5.66800
O	-13.18700	24.35100	0.26600
H	-13.32200	23.59100	-0.30000
H	-13.60400	25.07500	-0.20100
O	-2.05100	19.53500	-10.72600
H	-1.34800	19.74000	-10.11100
H	-2.77500	20.10200	-10.46100
O	-1.22200	15.28600	-4.60600
H	-1.27800	15.74200	-5.44500
H	-0.66900	14.52500	-4.78400
O	6.90000	13.81400	-1.27400
H	6.34000	13.88400	-0.50100
H	6.29500	13.63800	-1.99400
O	-10.03000	25.24800	0.47800
H	-10.14700	26.19200	0.37600
H	-10.85100	24.94500	0.86600
O	-0.14300	19.97300	-8.67600
H	0.69200	19.54100	-8.85900
H	-0.30400	19.80100	-7.74900
O	-0.48800	16.47600	-7.31100
H	-0.54300	16.31100	-8.25200
H	-1.11300	17.18400	-7.15500
O	-15.69900	11.71300	6.51500
H	-15.05300	11.20300	7.00400
H	-15.90700	12.45000	7.09000
O	-3.17300	18.78100	12.01500
H	-2.30400	18.95500	11.65300
H	-3.65300	19.60000	11.89200
O	-9.00500	25.90100	3.69300
H	-8.10200	25.93300	4.01000
H	-9.23100	24.97100	3.70800
O	-7.24900	24.65400	0.35400
H	-8.20000	24.65400	0.24100
H	-6.94600	23.90700	-0.16200
O	-11.35900	8.86000	10.61300
H	-10.49600	8.50300	10.82300
H	-11.93300	8.52100	11.30000
O	8.48600	30.49000	1.41900
H	9.12400	30.37100	0.71500
H	8.36800	31.43800	1.47800
O	5.29500	30.44300	1.35500
H	4.95700	30.32700	0.46700
H	6.16400	30.04200	1.33500

O	1.15300	32.29600	-2.65500
H	0.59000	32.20500	-3.42300
H	1.03900	31.47600	-2.17500
O	5.77700	31.59600	7.31400
H	5.76600	31.20100	6.44200
H	5.93900	30.86300	7.90700
O	-1.30000	33.79100	0.18300
H	-0.50800	33.45000	-0.23200
H	-1.06000	33.90100	1.10400
O	6.73300	35.96300	-7.34400
H	6.08200	36.54900	-7.73000
H	7.52800	36.49400	-7.28400
O	0.65900	33.05200	-6.94500
H	1.43200	32.50500	-7.08400
H	1.01200	33.91700	-6.73300
O	2.49700	34.38000	6.26200
H	2.20600	35.28400	6.14600
H	3.13300	34.42100	6.97600
O	3.42300	31.75700	2.85200
H	4.01900	31.53300	2.13800
H	3.48100	31.01600	3.45400
O	2.99600	29.21100	-9.31300
H	3.24800	28.69000	-10.07500
H	2.05800	29.04800	-9.21000
O	8.04000	28.21500	-1.50600
H	8.82600	27.79700	-1.85700
H	8.30400	29.12000	-1.33700
O	8.21100	33.65200	-6.80700
H	7.57600	33.72600	-6.09500
H	7.88100	34.24200	-7.48500
O	13.44100	28.15300	-0.60200
H	14.17300	27.58200	-0.36900
H	12.68500	27.76500	-0.16200
O	2.71500	31.11400	-7.11100
H	2.59900	30.43900	-6.44300
H	2.82500	30.62700	-7.92700
O	5.05800	30.57500	-3.40600
H	4.69400	30.04800	-2.69500
H	5.91500	30.18500	-3.57900
O	10.96300	21.86500	3.85700
H	10.63200	22.73400	3.63300
H	10.36800	21.54800	4.53700
O	4.69900	25.60400	-8.41000
H	4.86600	26.47200	-8.04300
H	4.50900	25.76700	-9.33400
O	7.45600	34.06600	3.25700
H	7.15700	34.96800	3.14800
H	7.11200	33.79900	4.10900
O	0.90800	30.51700	-0.71000
H	-0.01700	30.38500	-0.50200
H	1.15600	31.29700	-0.21300
O	10.85600	26.58400	0.93600
H	9.94800	26.51600	0.64200
H	10.80500	27.06100	1.76500
O	4.67800	28.06000	-7.42500
H	4.55800	28.50600	-6.58700
H	3.98300	28.40900	-7.98400
O	-1.73100	30.16100	0.12500
H	-1.60200	30.41400	1.03900
H	-2.41200	30.75300	-0.19500
O	2.43900	40.83000	-3.77600
H	2.36100	41.78100	-3.69700
H	1.83500	40.48500	-3.11900
O	4.93000	28.00300	8.88000
H	4.15400	28.56300	8.87000
H	5.56900	28.48900	9.40100
O	12.72200	24.78600	2.71400
H	12.08000	25.07900	2.06800
H	12.21800	24.67100	3.52000
O	10.37600	28.53200	-6.59700
H	11.28800	28.76500	-6.77200
H	9.86700	29.11600	-7.16000
O	3.18500	30.81200	8.44200
H	3.92600	31.35000	8.16500
H	2.47900	31.43800	8.59900
O	8.66000	32.95500	7.03900
H	9.06900	32.51300	6.29400
H	7.93800	32.38000	7.29200
O	5.01400	27.89200	4.41200
H	5.86000	28.31600	4.55600
H	4.95300	27.23500	5.10600
O	8.98300	33.51500	0.79400
H	8.35400	33.67900	0.09200
H	8.59600	33.92900	1.56500
O	12.86200	34.45000	-1.85100
H	12.17700	34.83000	-2.40200
H	12.94700	35.06000	-1.11800
O	8.16300	25.55200	6.07000
H	7.92000	26.30700	5.53400
H	8.43900	25.93100	6.90500
O	10.11700	32.09600	4.74800
H	10.32900	32.73900	4.07200
H	10.07000	31.26200	4.28000
O	7.72000	24.70800	1.65200
H	6.85800	25.00600	1.94400
H	7.74600	24.92500	0.72000
O	4.53600	33.25200	-1.94100
H	5.00000	33.53100	-2.73000
H	4.27200	32.35100	-2.12300
O	1.42900	32.79900	0.99700
H	1.99900	33.22800	0.35900
H	2.01900	32.51800	1.69600
O	3.47300	29.37400	-5.13200
H	4.17500	29.82700	-4.66400
H	3.14100	28.73300	-4.50400
O	1.92100	27.96600	9.24300
H	1.29100	28.64700	9.47700
H	1.81500	27.85400	8.29900
O	-2.78400	27.63300	0.17000
H	-3.57500	27.88900	-0.30600
H	-2.29300	28.44800	0.27500
O	11.23200	34.97300	2.92000
H	12.05400	34.57100	2.63900
H	11.22800	34.87000	3.87200
O	-0.83300	27.99700	-2.87200
H	-1.63000	28.10900	-2.35300
H	-1.06000	28.33600	-3.73800
O	4.99800	34.52200	5.07600

H	5.06200	34.98400	5.91200
H	4.28900	33.89200	5.20500
O	-4.50100	27.88600	2.60100
H	-4.25000	28.77100	2.86700
H	-3.96500	27.70600	1.82900
O	19.32300	31.31700	-0.73100
H	18.56900	30.74800	-0.88700
H	19.45700	31.28400	0.21600
O	4.71200	36.18900	-5.05600
H	4.57700	37.03400	-4.62700
H	5.25300	36.39100	-5.81900
O	1.19900	22.26800	11.60600
H	0.80600	21.71400	12.28100
H	2.01600	22.57900	11.99600
O	-1.83500	33.70600	-4.48300
H	-1.32200	34.50000	-4.33200
H	-1.18200	33.00900	-4.54900
O	-0.02500	36.82300	-2.40100
H	0.15100	37.74500	-2.58800
H	0.77000	36.50900	-1.97100
O	4.11600	23.68400	9.19800
H	4.85700	24.28400	9.11800
H	4.34800	22.94100	8.64000
O	-0.74000	28.86900	7.47600
H	-0.85500	27.96800	7.77600
H	-1.59600	29.11600	7.12400
O	6.33900	36.81100	9.07400
H	5.89600	36.02500	8.75700
H	7.00100	36.99900	8.40900
O	1.09800	38.34100	3.60000
H	1.64900	38.21200	2.82800
H	0.25900	38.64000	3.25000
O	8.43700	37.09100	7.10300
H	8.41900	37.91200	6.61100
H	9.35900	36.96700	7.32800
O	4.89500	34.60600	7.94500
H	5.42700	33.84400	7.71500
H	4.36600	34.31300	8.68700
O	8.06200	19.71200	3.47400
H	7.71800	20.60200	3.39800
H	8.43000	19.52200	2.61100
O	-3.87000	32.03500	-3.70700
H	-3.03200	32.44500	-3.92400
H	-3.69200	31.09500	-3.73700
O	8.02700	29.70300	-8.01800
H	7.51100	30.47300	-7.78000
H	7.75400	29.02900	-7.39500
O	5.61100	24.54700	-2.96000
H	4.75600	24.15800	-3.13900
H	6.02400	24.62700	-3.82000
O	7.14100	22.08200	1.43900
H	6.63800	22.04500	0.62600
H	7.43400	22.99100	1.49900
O	3.38600	22.16600	0.44600
H	4.25100	21.97000	0.08600
H	3.45300	21.93700	1.37300
O	13.00000	32.45600	-6.08300
H	12.22300	32.11700	-6.52800
H	13.67900	32.46100	-6.75800
O	7.81500	27.57500	-6.19300
H	7.51000	27.89000	-5.34100
H	8.76800	27.65300	-6.15000
O	6.26000	33.73400	-4.54300
H	5.73200	33.00000	-4.85600
H	5.75900	34.51200	-4.78900
O	11.23900	31.92900	0.57300
H	10.58200	32.61900	0.66200
H	12.04300	32.31900	0.91700
O	0.99200	24.39600	-2.18300
H	1.56600	24.17400	-2.91500
H	1.57900	24.46500	-1.43000
O	10.05000	30.28400	-1.37600
H	10.56300	30.91700	-0.87400
H	10.70000	29.68400	-1.74200
O	1.31900	26.42700	-8.16300
H	0.48200	26.76400	-7.84200
H	1.17900	26.29200	-9.10000
O	3.29400	21.27000	-3.45500
H	2.64100	20.95500	-4.07900
H	3.25200	20.64800	-2.72900
O	4.40500	31.55200	-11.54000
H	3.49800	31.32100	-11.34100
H	4.90200	30.75600	-11.35200
O	7.31200	40.26200	-8.62000
H	6.63800	40.56500	-8.01200
H	7.69900	41.06500	-8.97000
O	6.52800	27.75900	0.87000
H	5.61000	27.98000	0.71200
H	6.94400	27.83300	0.01100
O	7.31100	29.15800	-4.00000
H	8.05900	29.67000	-4.30900
H	7.58000	28.83600	-3.14000
O	5.58400	31.65400	-7.01700
H	4.67500	31.36900	-6.92000
H	5.61400	32.07300	-7.87700
O	10.81600	35.26400	-3.24800
H	10.36200	34.42300	-3.19200
H	10.18500	35.90000	-2.91100
O	9.75900	26.19300	-2.01800
H	9.02100	25.68100	-1.68700
H	10.01200	25.75300	-2.83000
O	9.25900	37.10200	-2.31300
H	9.55200	37.66200	-3.03200
H	9.76900	37.39500	-1.55800
O	9.86000	18.97100	-1.42400
H	10.16600	18.11100	-1.71100
H	10.58600	19.56300	-1.62400
O	-3.84700	29.37400	-3.03600
H	-4.04500	28.78100	-3.76100
H	-4.40300	29.06900	-2.31800
O	4.13200	29.26600	-0.91600
H	4.18700	28.31400	-0.99200
H	3.32200	29.42200	-0.43200
O	5.28200	33.19400	-9.67900
H	4.84400	32.47800	-10.13800
H	6.02300	33.42200	-10.24000
O	2.07200	27.88400	-3.15700
H	1.25900	28.18400	-2.75100

H	2.56800	27.49000	-2.44000
O	4.22300	37.88000	-1.22400
H	4.18000	38.10600	-2.15300
H	5.13900	37.64600	-1.07500
O	7.66900	28.34800	5.67400
H	8.02400	28.57500	6.53400
H	8.31600	28.68000	5.05200
O	13.39900	31.47000	-3.62100
H	13.26100	31.66000	-4.54900
H	13.04300	32.23200	-3.16400
O	1.56300	25.45600	2.45900
H	2.20300	25.31500	1.76100
H	0.73100	25.16900	2.08300
O	13.42600	33.53800	1.39000
H	13.86000	33.44500	2.23900
H	14.13900	33.51400	0.75200
O	2.33900	34.75600	-1.42800
H	3.13300	34.26100	-1.62900
H	1.62800	34.22800	-1.79100
O	7.15900	37.13800	2.92400
H	7.21200	37.70700	3.69100
H	6.29000	37.30800	2.55900
O	-1.35300	28.90000	-5.35200
H	-2.28300	28.83900	-5.13500
H	-1.25700	28.37800	-6.14800
O	-1.43400	25.42600	-2.76400
H	-1.17300	26.34000	-2.87300
H	-0.61200	24.95800	-2.61400
O	0.03800	26.84000	4.40600
H	0.71100	26.63100	3.75700
H	-0.78900	26.64200	3.96700
O	14.28700	33.58000	3.92400
H	14.48100	34.48500	4.16900
H	14.18700	33.12100	4.75700
O	5.94100	23.46600	6.42300
H	5.29400	24.16500	6.32900
H	6.71100	23.90000	6.79000
O	9.34900	33.73100	9.49800
H	9.19700	33.50200	8.58100
H	9.33900	34.68800	9.51100
O	6.11700	31.30400	4.48200
H	7.04700	31.45500	4.30900
H	5.69700	31.39200	3.62700
O	4.91600	35.32900	2.25200
H	4.77700	34.96100	3.12500
H	4.12800	35.84400	2.08100
O	5.51100	40.43000	-6.40500
H	5.68600	39.95900	-5.59100
H	4.82400	41.05700	-6.17700
O	6.46700	34.70700	-0.35900
H	6.13200	34.66700	0.53700
H	5.96700	34.04200	-0.83200
O	0.44100	30.11400	5.16600
H	0.30300	29.38200	5.76700
H	1.35200	30.02500	4.88500
O	9.40300	32.05300	-10.08500
H	8.78600	31.32800	-10.18200
H	8.93400	32.81400	-10.42900
O	3.06900	29.82600	4.65500
H	3.77900	29.18500	4.61300
H	3.02600	30.07700	5.57700
O	11.05800	36.76300	7.57500
H	10.99900	36.07900	6.90800
H	11.46000	37.50500	7.12300
O	1.78200	26.81300	6.47400
H	1.24600	26.36200	7.12600
H	1.19400	26.94700	5.73100
O	0.07700	43.57900	0.36900
H	0.31700	44.21100	1.04700
H	-0.76600	43.22800	0.65500
O	1.19300	26.27100	-10.96300
H	0.31600	26.15000	-11.32700
H	1.66800	25.48000	-11.21500
O	11.06000	34.72700	5.80700
H	10.17300	34.38900	5.92900
H	11.63000	33.99500	6.04400
O	-7.28700	31.05900	-0.86900
H	-6.60000	30.74500	-1.45700
H	-7.44200	31.96300	-1.14200
O	-2.48400	25.94800	3.55900
H	-2.56800	25.34100	2.82500
H	-3.30500	26.43900	3.56000
O	4.59000	39.38700	2.77300
H	4.51700	40.19200	3.28500
H	4.14800	39.58600	1.94800
O	4.37900	38.62400	-3.71700
H	3.59600	39.16600	-3.62300
H	5.07800	39.14100	-3.31700
O	7.68900	24.43300	-1.07400
H	8.18600	23.68000	-1.39300
H	6.91700	24.47000	-1.63900
O	15.54200	30.13800	0.56800
H	15.53100	31.02000	0.19800
H	14.67300	29.78500	0.37400
O	7.05000	39.28600	4.92900
H	7.76300	39.92000	4.85600
H	6.55900	39.38200	4.11300
O	8.77200	33.19100	-3.50000
H	7.83700	33.31100	-3.66900
H	8.94900	32.28800	-3.76400
O	4.27200	26.57500	-1.34600
H	3.90200	26.02200	-0.65800
H	4.76800	25.97100	-1.89900
O	12.15900	29.03200	-2.96500
H	12.62500	29.86500	-3.03900
H	12.61000	28.56700	-2.26000
O	2.10100	37.03500	5.74900
H	2.80200	37.66900	5.90200
H	1.63100	37.37600	4.98900
O	10.22700	35.37700	-5.96600
H	10.50200	35.26200	-5.05700
H	9.35700	34.97900	-6.00500
O	10.13100	21.40000	1.07400
H	9.21700	21.63100	1.24300
H	10.56000	21.48400	1.92600
O	9.90100	29.58900	3.57400
H	9.24800	29.83700	2.92000
H	10.27900	28.77600	3.24100

O	6.86200	39.62500	-2.32300
H	7.04400	38.80800	-1.86000
H	7.68500	39.84200	-2.76000
O	7.19600	38.55200	-5.06100
H	7.72500	38.04100	-5.67400
H	7.45900	38.23600	-4.19700
O	5.95000	30.33100	10.06100
H	5.27400	30.23100	10.73100
H	6.77300	30.17500	10.52500
O	-2.30200	36.56900	2.68200
H	-1.71700	35.82300	2.81500
H	-2.50000	36.87900	3.56600
O	0.55200	39.60400	-2.30100
H	-0.11700	40.08800	-2.78500
H	0.41300	39.85100	-1.38700
O	-7.71000	29.13900	1.40300
H	-7.67200	28.60600	2.19600
H	-8.21600	28.61300	0.78400
O	4.69200	41.63800	4.29700
H	5.24400	41.80100	5.06200
H	5.03300	42.22900	3.62500
O	-0.33100	23.98600	4.89900
H	-0.26700	24.84700	4.48700
H	-0.39500	23.37300	4.16600
O	-0.85600	26.05800	8.54600
H	-0.22900	25.67600	9.16000
H	-1.71300	25.83900	8.91200
O	9.54300	36.46400	10.15200
H	10.08700	37.07700	9.65700
H	8.96200	37.02200	10.66800
O	-4.08000	27.86600	-5.52200
H	-3.78400	26.95700	-5.57500
H	-4.98100	27.84400	-5.84400
O	7.82900	39.58600	1.28300
H	7.70400	38.78500	0.77400
H	8.48700	40.08100	0.79500
O	-0.55000	25.94100	0.46100
H	-1.27100	26.55500	0.32200
H	0.23900	26.47700	0.38300
O	9.89300	37.38700	2.91100
H	8.94600	37.26800	2.83900
H	10.24100	36.49900	2.99400
O	2.25300	23.27700	6.73500
H	2.77600	23.52300	7.49800
H	2.03400	24.10900	6.31500
O	-2.35100	25.54700	-5.39800
H	-2.67900	24.66700	-5.58000
H	-1.99700	25.49200	-4.51000
O	6.89700	37.18200	-0.88800
H	6.75000	36.25400	-0.70800
H	7.72300	37.20400	-1.37300
O	0.76300	25.83400	-5.03000
H	1.10300	26.36300	-5.75300
H	1.21100	26.17200	-4.25500
O	10.50100	38.00500	0.19800
H	11.42500	37.80000	0.06000
H	10.33200	37.75700	1.10700
O	3.45800	24.21800	4.42000
H	3.11700	23.41900	4.82200
H	2.75200	24.51800	3.84800
O	4.10900	21.30300	2.87300
H	4.93200	20.82900	2.75100
H	4.35300	22.08200	3.37300
O	2.75000	24.33200	-6.83100
H	3.47100	24.71000	-7.33500
H	2.12700	25.05100	-6.72800
O	6.89300	25.03600	-5.89500
H	7.11000	25.91900	-6.19600
H	6.41300	24.64500	-6.62500
O	1.63300	27.99500	0.68200
H	1.48800	28.30900	1.57400
H	1.15600	28.61300	0.12900
O	3.49300	24.98500	0.73000
H	3.47900	24.08900	0.39300
H	4.21000	24.99200	1.36400
O	5.34600	25.41900	2.77500
H	5.08700	26.34100	2.77400
H	4.81400	25.02200	3.46500
O	10.29000	38.52400	-4.44600
H	10.46400	37.95900	-5.19800
H	10.78300	39.32400	-4.62800
O	-3.89200	30.53500	3.62100
H	-4.43700	31.31100	3.75300
H	-3.01400	30.88200	3.46300
O	2.89700	23.86700	-4.00100
H	3.01800	22.93800	-3.80200
H	2.60700	23.88000	-4.91300
O	5.76600	21.55200	-0.86500
H	5.78800	21.61600	-1.81900
H	6.18300	20.71200	-0.67100
O	16.14400	31.28800	-2.82700
H	16.58700	30.53800	-3.22500
H	15.26700	31.27900	-3.20900
O	11.27800	37.84100	-7.34600
H	11.97300	38.46100	-7.12700
H	11.62000	36.99000	-7.07100
O	-3.10600	29.66200	6.51400
H	-3.84700	29.81100	7.10000
H	-3.42600	29.92300	5.65000
O	13.17700	35.29100	-5.81700
H	13.70200	35.62900	-6.54200
H	13.08900	34.35500	-6.00100
O	4.83900	39.20400	6.31200
H	5.14800	38.95900	7.18400
H	5.63600	39.40900	5.82400
O	4.86100	41.35800	-1.45800
H	4.42100	40.86500	-0.76500
H	5.61200	40.81500	-1.69900
O	-2.66000	37.54900	-1.45500
H	-1.83400	37.24900	-1.83500
H	-3.01000	36.78000	-1.00600
O	2.30800	36.90200	0.61400
H	1.68600	36.23900	0.31500
H	2.97900	36.92800	-0.06800
O	-4.45900	35.46800	-0.49600
H	-5.29600	35.04000	-0.67800
H	-4.48700	35.66100	0.44100
O	2.38100	25.38000	10.20800

H	2.43000	26.25600	9.82500
H	2.92300	24.83700	9.63500
O	12.86600	35.92400	9.48800
H	12.07600	35.90600	8.94800
H	12.63000	36.46300	10.24300
O	12.98600	32.77300	6.80100
H	13.03600	32.60800	7.74300
H	12.87700	31.90600	6.41100
O	1.87000	35.45200	-5.91000
H	2.01800	36.13500	-6.56300
H	2.67600	35.43200	-5.39500
O	10.27600	28.75600	8.11700
H	11.17700	28.61700	7.82500
H	10.35100	28.92200	9.05700
O	4.61100	26.13300	6.96200
H	3.74100	26.42600	6.69100
H	4.84700	26.71700	7.68300
O	14.48800	35.19300	7.04500
H	14.19600	35.55600	7.88200
H	13.87500	34.47900	6.86700
O	15.68700	32.75800	-0.43400
H	16.52200	33.19700	-0.27100
H	15.82300	32.28200	-1.25400
O	12.73700	30.31600	5.51300
H	13.21200	30.50900	4.70500
H	11.81300	30.37200	5.26800
O	-0.37500	31.47800	-4.90400
H	-0.62100	30.57500	-4.70600
H	-0.20600	31.48000	-5.84600
O	7.31500	22.35100	4.47900
H	7.83300	23.08700	4.15300
H	6.96000	22.65700	5.31400
O	-1.10600	27.21100	-7.40500
H	-1.79900	27.28600	-8.06100
H	-1.35000	26.44700	-6.88300
O	7.56600	18.77100	-0.00100
H	7.84600	18.10300	0.62500
H	8.35100	18.96400	-0.51400
O	5.36900	37.94700	-8.43900
H	4.79700	38.56600	-8.89200
H	5.94200	38.49700	-7.90500
O	11.43700	27.19300	3.74600
H	12.38800	27.29200	3.76700
H	11.26700	26.40000	4.25600
O	9.45800	24.27800	3.93200
H	8.86300	24.55900	3.23700
H	9.17300	24.76800	4.70400
O	8.58900	38.23000	-7.30800
H	9.52200	38.16500	-7.10400
H	8.50100	39.06600	-7.76600
O	11.21800	37.54700	-10.34700
H	11.13900	37.21300	-9.45400
H	12.03900	38.03900	-10.34800
O	13.91500	30.41300	2.86000
H	13.88300	29.47500	3.04600
H	14.52600	30.49200	2.12700
O	5.75400	29.28300	-11.09700
H	6.70000	29.15300	-11.02800
H	5.37500	28.54400	-10.62000
O	14.06500	27.20400	2.90600
H	13.54900	26.42000	2.71900
H	14.94900	26.87600	3.07100
O	11.72600	20.94800	-1.13400
H	11.52800	20.64000	-0.25000
H	12.33600	21.67500	-1.00400
O	19.73300	31.47500	2.00100
H	20.46300	31.21300	2.56200
H	19.12400	30.73800	2.04100
O	17.81500	29.61600	2.59800
H	17.08800	29.48100	1.99000
H	17.39900	29.92300	3.40400
O	5.63800	33.33500	10.36300
H	6.55800	33.51200	10.55700
H	5.60300	32.39000	10.21100
O	3.93800	27.20800	-11.16100
H	3.03100	26.91100	-11.08500
H	4.21600	26.91100	-12.02800
O	-5.14400	28.52800	-0.71200
H	-5.46400	27.67500	-1.00400
H	-5.69100	28.74300	0.04400
O	-1.36600	31.24500	3.00500
H	-1.14700	32.17600	3.02400
H	-0.95400	30.88500	3.79100
O	5.55400	21.75200	-5.00700
H	4.74400	21.40600	-4.63200
H	6.24300	21.44200	-4.41800
O	7.48300	20.82900	-3.38500
H	8.03600	21.24900	-2.72600
H	7.72000	19.90300	-3.34200
O	3.50700	26.81800	12.39600
H	3.40600	27.76700	12.32700
H	3.28900	26.49000	11.52300
O	2.70800	24.06700	-11.02000
H	3.57000	24.39200	-10.76100
H	2.89300	23.28000	-11.53300
O	-5.27500	32.14900	1.25800
H	-5.96900	31.73400	0.74500
H	-4.52800	32.19100	0.66100
O	10.86100	22.93900	-3.87700
H	10.28100	23.59800	-4.25900
H	10.59500	22.11700	-4.28800
O	9.31200	22.34000	-1.67500
H	9.81200	22.45200	-2.48300
H	9.95500	22.03600	-1.03500
O	3.37000	30.24800	11.21200
H	2.88700	31.04800	11.41900
H	3.45600	30.25900	10.25900
O	-3.36200	32.08100	-0.86600
H	-3.38500	32.17900	-1.81700
H	-3.05200	32.92700	-0.54400
O	-0.70300	34.13800	3.24900
H	0.25100	34.10700	3.18200
H	-0.90200	33.67700	4.06400
O	-0.11300	32.70800	5.81600
H	0.70600	33.20300	5.84000
H	0.16100	31.79100	5.78800
O	9.64400	25.09900	-4.82100
H	8.75600	25.38400	-5.03900

H	10.05900	24.93900	-5.66800
O	12.99500	25.12400	-1.10600
H	13.77100	25.61400	-0.83300
H	12.27000	25.55000	-0.64800
O	15.43200	25.83700	-0.04800
H	16.02700	25.59600	-0.75900
H	15.50500	25.11700	0.57900
O	-2.34800	39.68000	0.26600
H	-2.48200	38.92000	-0.30000
H	-2.76500	40.40400	-0.20100
O	8.78800	34.86400	-10.72600
H	9.49200	35.06900	-10.11100
H	8.06400	35.43200	-10.46100
O	9.61800	30.61500	-4.60600
H	9.56100	31.07200	-5.44500
H	10.17100	29.85400	-4.78400
O	13.42800	26.40400	-3.78100
H	13.17200	26.00300	-2.95000
H	12.80400	27.11800	-3.90600
O	17.74000	29.14300	-1.27400
H	17.18000	29.21300	-0.50100
H	17.13400	28.96800	-1.99400
O	0.80900	40.57700	0.47800
H	0.69200	41.52200	0.37600
H	-0.01200	40.27400	0.86600
O	10.69700	35.30200	-8.67600
H	11.53100	34.87000	-8.85900
H	10.53500	35.13000	-7.74900
O	10.35100	31.80500	-7.31100
H	10.29600	31.64000	-8.25200
H	9.72600	32.51300	-7.15500
O	-4.85900	27.04200	6.51500
H	-4.21400	26.53200	7.00400
H	-5.06800	27.77900	7.09000
O	7.66600	34.11000	12.01500
H	8.53500	34.28400	11.65300
H	7.18700	34.93000	11.89200
O	3.66100	21.30600	-7.73200
H	3.64900	22.25000	-7.57500
H	4.55300	21.12300	-8.02600
O	1.83500	41.23000	3.69300
H	2.73700	41.26200	4.01000
H	1.60800	40.30000	3.70800
O	3.59000	39.98300	0.35400
H	2.63900	39.98300	0.24100
H	3.89300	39.23600	-0.16200
O	7.47600	17.90000	-3.47800
H	6.70900	17.34600	-3.33200
H	7.88700	17.97300	-2.61600
O	-0.52000	24.18900	10.61300
H	0.34300	23.83300	10.82300
H	-1.09300	23.85000	11.30000
O	19.32500	45.81900	1.41900
H	19.96300	45.70000	0.71500
H	19.20700	46.76700	1.47800
O	16.13400	45.77200	1.35500
H	15.79600	45.65600	0.46700
H	17.00300	45.37100	1.33500
O	11.99300	47.62500	-2.65500
H	11.42900	47.53400	-3.42300
H	11.87800	46.80500	-2.17500
O	16.61600	46.92500	7.31400
H	16.60500	46.53100	6.44200
H	16.77800	46.19200	7.90700
O	11.49800	48.38100	-6.94500
H	12.27100	47.83400	-7.08400
H	11.85100	49.24600	-6.73300
O	14.26200	47.08700	2.85200
H	14.85900	46.86200	2.13800
H	14.32000	46.34500	3.45400
O	13.83500	44.54100	-9.31300
H	14.08800	44.01900	-10.07500
H	12.89700	44.37700	-9.21000
O	18.87900	43.54400	-1.50600
H	19.66600	43.12600	-1.85700
H	19.14300	44.44900	-1.33700
O	24.28000	43.48200	-0.60200
H	25.01300	42.91200	-0.36900
H	23.52400	43.09400	-0.16200
O	13.55500	46.44300	-7.11100
H	13.43800	45.76800	-6.44300
H	13.66500	45.95700	-7.92700
O	15.89800	45.90400	-3.40600
H	15.53400	45.37800	-2.69500
H	16.75400	45.51400	-3.57900
O	21.80300	37.19400	3.85700
H	21.47100	38.06300	3.63300
H	21.20700	36.87800	4.53700
O	15.53800	40.93300	-8.41000
H	15.70600	41.80100	-8.04300
H	15.34800	41.09600	-9.33400
O	11.74700	45.84700	-0.71000
H	10.82200	45.71400	-0.50200
H	11.99600	46.62600	-0.21300
O	21.69600	41.91300	0.93600
H	20.78700	41.84500	0.64200
H	21.64400	42.39100	1.76500
O	15.51700	43.38900	-7.42500
H	15.39700	43.83500	-6.58700
H	14.82200	43.73800	-7.98400
O	9.10800	45.49000	0.12500
H	9.23700	45.74300	1.03900
H	8.42800	46.08300	-0.19500
O	15.77000	43.33200	8.88000
H	14.99300	43.89200	8.87000
H	16.40900	43.81800	9.40100
O	23.56200	40.11500	2.71400
H	22.91900	40.40800	2.06800
H	23.05700	40.00000	3.52000
O	21.21600	43.86100	-6.59700
H	22.12800	44.09400	-6.77200
H	20.70700	44.44500	-7.16000
O	14.02400	46.14100	8.44200
H	14.76600	46.67900	8.16500
H	13.31800	46.76800	8.59900
O	15.85300	43.22100	4.41200
H	16.69900	43.64500	4.55600
H	15.79300	42.56400	5.10600

O	19.00300	40.88200	6.07000
H	18.75900	41.63600	5.53400
H	19.27900	41.26000	6.90500
O	18.55900	40.03700	1.65200
H	17.69800	40.33500	1.94400
H	18.58600	40.25400	0.72000
O	15.37500	48.58100	-1.94100
H	15.83900	48.86000	-2.73000
H	15.11100	47.68000	-2.12300
O	12.26800	48.12900	0.99700
H	12.83800	48.55700	0.35900
H	12.85900	47.84700	1.69600
O	14.31200	44.70400	-5.13200
H	15.01500	45.15600	-4.66400
H	13.98000	44.06200	-4.50400
O	12.76000	43.29500	9.24300
H	12.13000	43.97600	9.47700
H	12.65400	43.18300	8.29900
O	8.05500	42.96200	0.17000
H	7.26500	43.21800	-0.30600
H	8.54600	43.77700	0.27500
O	10.00600	43.32700	-2.87200
H	9.20900	43.43800	-2.35300
H	9.77900	43.66500	-3.73800
O	6.33900	43.21500	2.60100
H	6.58900	44.10000	2.86700
H	6.87500	43.03600	1.82900
O	12.03800	37.59700	11.60600
H	11.64500	37.04400	12.28100
H	12.85500	37.90800	11.99600
O	14.95500	39.01300	9.19800
H	15.69600	39.61300	9.11800
H	15.18700	38.27000	8.64000
O	10.09900	44.19800	7.47600
H	9.98500	43.29700	7.77600
H	9.24400	44.44500	7.12400
O	18.90100	35.04100	3.47400
H	18.55700	35.93100	3.39800
H	19.26900	34.85100	2.61100
O	6.97000	47.36400	-3.70700
H	7.80700	47.77400	-3.92400
H	7.14700	46.42400	-3.73700
O	18.86600	45.03200	-8.01800
H	18.35100	45.80200	-7.78000
H	18.59400	44.35800	-7.39500
O	16.45100	39.87700	-2.96000
H	15.59500	39.48700	-3.13900
H	16.86300	39.95600	-3.82000
O	17.98100	37.41100	1.43900
H	17.47700	37.37400	0.62600
H	18.27400	38.32100	1.49900
O	14.22500	37.49500	0.44600
H	15.09000	37.29900	0.08600
H	14.29300	37.26600	1.37300
O	18.65500	42.90400	-6.19300
H	18.35000	43.21900	-5.34100
H	19.60800	42.98300	-6.15000
O	11.83100	39.72500	-2.18300
H	12.40600	39.50300	-2.91500
H	12.41800	39.79400	-1.43000
O	12.15800	41.75600	-8.16300
H	11.32200	42.09300	-7.84200
H	12.01900	41.62100	-9.10000
O	14.13300	36.60000	-3.45500
H	13.48000	36.28400	-4.07900
H	14.09100	35.97700	-2.72900
O	15.24500	46.88100	-11.54000
H	14.33700	46.65000	-11.34100
H	15.74200	46.08500	-11.35200
O	17.36700	43.08800	0.87000
H	16.44900	43.30900	0.71200
H	17.78300	43.16200	0.01100
O	18.15100	44.48700	-4.00000
H	18.89800	44.99900	-4.30900
H	18.42000	44.16500	-3.14000
O	16.42300	46.98300	-7.01700
H	15.51500	46.69900	-6.92000
H	16.45300	47.40300	-7.87700
O	20.59900	41.52300	-2.01800
H	19.86100	41.01000	-1.68700
H	20.85100	41.08300	-2.83000
O	20.70000	34.30000	-1.42400
H	21.00600	33.44000	-1.71100
H	21.42500	34.89200	-1.62400
O	6.99200	44.70300	-3.03600
H	6.79500	44.11000	-3.76100
H	6.43700	44.39800	-2.31800
O	14.97200	44.59600	-0.91600
H	15.02700	43.64300	-0.99200
H	14.16100	44.75200	-0.43200
O	16.12100	48.52400	-9.67900
H	15.68300	47.80700	-10.13800
H	16.86200	48.75100	-10.24000
O	12.91100	43.21300	-3.15700
H	12.09800	43.51300	-2.75100
H	13.40700	42.81900	-2.44000
O	18.50900	43.67700	5.67400
H	18.86300	43.90500	6.53400
H	19.15600	44.00900	5.05200
O	12.40300	40.78500	2.45900
H	13.04200	40.64400	1.76100
H	11.57000	40.49900	2.08300
O	13.17900	50.08500	-1.42800
H	13.97300	49.59000	-1.62900
H	12.46800	49.55700	-1.79100
O	9.48700	44.23000	-5.35200
H	8.55700	44.16800	-5.13500
H	9.58300	43.70700	-6.14800
O	9.40500	40.75500	-2.76400
H	9.66600	41.67000	-2.87300
H	10.22700	40.28800	-2.61400
O	10.87800	42.16900	4.40600
H	11.55000	41.96100	3.75700
H	10.05100	41.97100	3.96700
O	16.78000	38.79500	6.42300
H	16.13300	39.49400	6.32900
H	17.55100	39.22900	6.79000
O	16.95700	46.63300	4.48200

H	17.88600	46.78400	4.30900
H	16.53600	46.72200	3.62700
O	11.28100	45.44300	5.16600
H	11.14300	44.71100	5.76700
H	12.19100	45.35400	4.88500
O	13.90900	45.15500	4.65500
H	14.61900	44.51500	4.61300
H	13.86600	45.40600	5.57700
O	12.62200	42.14200	6.47400
H	12.08500	41.69100	7.12600
H	12.03300	42.27600	5.73100
O	12.03200	41.60100	-10.96300
H	11.15500	41.47900	-11.32700
H	12.50800	40.80900	-11.21500
O	8.35600	41.27700	3.55900
H	8.27100	40.67000	2.82500
H	7.53400	41.76800	3.56000
O	18.52800	39.76300	-1.07400
H	19.02500	39.01000	-1.39300
H	17.75700	39.79900	-1.63900
O	15.11200	41.90400	-1.34600
H	14.74100	41.35200	-0.65800
H	15.60700	41.30000	-1.89900
O	20.97000	36.73000	1.07400
H	20.05600	36.96000	1.24300
H	21.39900	36.81300	1.92600
O	20.74100	44.91900	3.57400
H	20.08800	45.16600	2.92000
H	21.11900	44.10500	3.24100
O	16.78900	45.66100	10.06100
H	16.11300	45.56000	10.73100
H	17.61200	45.50500	10.52500
O	3.12900	44.46800	1.40300
H	3.16700	43.93500	2.19600
H	2.62300	43.94200	0.78400
O	10.50800	39.31500	4.89900
H	10.57200	40.17600	4.48700
H	10.44500	38.70200	4.16600
O	9.98300	41.38700	8.54600
H	10.61000	41.00500	9.16000
H	9.12600	41.16900	8.91200
O	6.76000	43.19500	-5.52200
H	7.05500	42.28600	-5.57500
H	5.85900	43.17400	-5.84400
O	10.28900	41.27000	0.46100
H	9.56800	41.88400	0.32200
H	11.07900	41.80600	0.38300
O	13.09200	38.60600	6.73500
H	13.61500	38.85200	7.49800
H	12.87300	39.43800	6.31500
O	8.48900	40.87600	-5.39800
H	8.16000	39.99600	-5.58000
H	8.84300	40.82100	-4.51000
O	11.60300	41.16300	-5.03000
H	11.94200	41.69200	-5.75300
H	12.05000	41.50200	-4.25500
O	14.29700	39.54700	4.42000
H	13.95700	38.74800	4.82200
H	13.59100	39.84700	3.84800
O	14.94900	36.63200	2.87300
H	15.77100	36.15800	2.75100
H	15.19300	37.41100	3.37300
O	13.59000	39.66100	-6.83100
H	14.31000	40.03900	-7.33500
H	12.96600	40.38000	-6.72800
O	17.73300	40.36500	-5.89500
H	17.94900	41.24800	-6.19600
H	17.25200	39.97400	-6.62500
O	12.47300	43.32400	0.68200
H	12.32700	43.63800	1.57400
H	11.99500	43.94300	0.12900
O	14.33200	40.31400	0.73000
H	14.31800	39.41800	0.39300
H	15.05000	40.32100	1.36400
O	16.18500	40.74800	2.77500
H	15.92700	41.67000	2.77400
H	15.65300	40.35100	3.46500
O	6.94700	45.86500	3.62100
H	6.40300	46.64100	3.75300
H	7.82500	46.21200	3.46300
O	13.73600	39.19600	-4.00100
H	13.85800	38.26700	-3.80200
H	13.44600	39.21000	-4.91300
O	16.60500	36.88100	-0.86500
H	16.62700	36.94500	-1.81900
H	17.02200	36.04200	-0.67100
O	7.73400	44.99200	6.51400
H	6.99200	45.14000	7.10000
H	7.41300	45.25200	5.65000
O	13.22000	40.70900	10.20800
H	13.26900	41.58500	9.82500
H	13.76200	40.16700	9.63500
O	21.11600	44.08500	8.11700
H	22.01700	43.94600	7.82500
H	21.19100	44.25100	9.05700
O	15.45000	41.46200	6.96200
H	14.58000	41.75500	6.69100
H	15.68600	42.04600	7.68300
O	10.46400	46.80800	-4.90400
H	10.21900	45.90400	-4.70600
H	10.63400	46.80900	-5.84600
O	18.15400	37.68000	4.47900
H	18.67200	38.41600	4.15300
H	17.79900	37.98600	5.31400
O	9.73400	42.54000	-7.40500
H	9.04100	42.61500	-8.06100
H	9.48900	41.77700	-6.88300
O	18.40500	34.10000	-0.00100
H	18.68500	33.43200	0.62500
H	19.19000	34.29300	-0.51400
O	22.27600	42.52200	3.74600
H	23.22800	42.62200	3.76700
H	22.10600	41.73000	4.25600
O	20.29800	39.60700	3.93200
H	19.70200	39.88800	3.23700
H	20.01200	40.09700	4.70400
O	16.59300	44.61200	-11.09700
H	17.53900	44.48300	-11.02800

H	16.21500	43.87300	-10.62000
O	24.90400	42.53400	2.90600
H	24.38800	41.74900	2.71900
H	25.78800	42.20500	3.07100
O	22.56500	36.27800	-1.13400
H	22.36800	35.96900	-0.25000
H	23.17500	37.00400	-1.00400
O	14.77700	42.53700	-11.16100
H	13.87100	42.24000	-11.08500
H	15.05500	42.24000	-12.02800
O	5.69500	43.85800	-0.71200
H	5.37600	43.00400	-1.00400
H	5.14900	44.07300	0.04400
O	9.47300	46.57400	3.00500
H	9.69200	47.50600	3.02400
H	9.88600	46.21500	3.79100
O	16.39400	37.08100	-5.00700
H	15.58400	36.73500	-4.63200
H	17.08200	36.77100	-4.41800
O	18.32300	36.15900	-3.38500
H	18.87600	36.57800	-2.72600
H	18.56000	35.23200	-3.34200
O	14.34600	42.14700	12.39600
H	14.24600	43.09700	12.32700
H	14.12800	41.81900	11.52300
O	13.54700	39.39600	-11.02000
H	14.40900	39.72100	-10.76100
H	13.73200	38.60900	-11.53300
O	21.70100	38.26900	-3.87700
H	21.12100	38.92700	-4.25900
H	21.43400	37.44600	-4.28800
O	20.15100	37.66900	-1.67500
H	20.65100	37.78100	-2.48300
H	20.79500	37.36500	-1.03500
O	14.20900	45.57700	11.21200
H	13.72600	46.37700	11.41900
H	14.29500	45.58800	10.25900
O	10.72600	48.03700	5.81600
H	11.54600	48.53200	5.84000
H	11.00000	47.12100	5.78800
O	20.48300	40.42900	-4.82100
H	19.59600	40.71400	-5.03900
H	20.89900	40.26800	-5.66800
O	23.83500	40.45300	-1.10600
H	24.61100	40.94300	-0.83300
H	23.10900	40.87900	-0.64800
O	26.27200	41.16600	-0.04800
H	26.86600	40.92600	-0.75900
H	26.34400	40.44700	0.57900
O	24.26800	41.73300	-3.78100
H	24.01100	41.33300	-2.95000
H	23.64300	42.44700	-3.90600
O	5.98000	42.37100	6.51500
H	6.62600	41.86100	7.00400
H	5.77200	43.10800	7.09000
O	14.50000	36.63500	-7.73200
H	14.48800	37.57900	-7.57500
H	15.39300	36.45200	-8.02600
O	18.31600	33.23000	-3.47800
H	17.54900	32.67500	-3.33200
H	18.72700	33.30200	-2.61600
O	10.32000	39.51800	10.61300
H	11.18300	39.16200	10.82300
H	9.74600	39.17900	11.30000
O	-24.03200	-38.49100	-11.85600
H	-23.39400	-38.61100	-12.56000
H	-24.15000	-37.54300	-11.79800
O	-27.22300	-38.53900	-11.92000
H	-27.56200	-38.65500	-12.80800
H	-26.35400	-38.94000	-11.94000
O	-31.36500	-36.68500	-15.93100
H	-31.92800	-36.77600	-16.69900
H	-31.47900	-37.50500	-15.45000
O	-26.74100	-37.38500	-5.96200
H	-26.75300	-37.78000	-6.83400
H	-26.57900	-38.11900	-5.36800
O	-33.81800	-35.19000	-13.09200
H	-33.02600	-35.53100	-13.50800
H	-33.57900	-35.08100	-12.17200
O	-25.78500	-33.01800	-20.61900
H	-26.43600	-32.43300	-21.00600
H	-24.99000	-32.48700	-20.56000
O	-30.02100	-34.60200	-7.01400
H	-30.31200	-33.69700	-7.12900
H	-29.38500	-34.56000	-6.29900
O	-29.09500	-37.22400	-10.42400
H	-28.49900	-37.44800	-11.13800
H	-29.03800	-37.96500	-9.82100
O	-24.47800	-40.76600	-14.78100
H	-23.69200	-41.18400	-15.13200
H	-24.21400	-39.86200	-14.61300
O	-24.30700	-35.32900	-20.08200
H	-24.94300	-35.25600	-19.37000
H	-24.63700	-34.74000	-20.76000
O	-19.07700	-40.82900	-13.87800
H	-18.34500	-41.39900	-13.64400
H	-19.83300	-41.21700	-13.43700
O	-27.46000	-38.40600	-16.68100
H	-27.82400	-38.93300	-15.97000
H	-26.60300	-38.79600	-16.85500
O	-25.06200	-34.91500	-10.01900
H	-25.36200	-34.01300	-10.12700
H	-25.40700	-35.18200	-9.16700
O	-21.66200	-42.39700	-12.33900
H	-22.57000	-42.46600	-12.63300
H	-21.71300	-41.92000	-11.51100
O	-30.08000	-28.15100	-17.05200
H	-30.15700	-27.20000	-16.97300
H	-30.68300	-28.49600	-16.39400
O	-27.58800	-40.97800	-4.39600
H	-28.36500	-40.41900	-4.40500
H	-26.94900	-40.49300	-3.87400
O	-19.79600	-44.19600	-10.56100
H	-20.43800	-43.90200	-11.20700
H	-20.30000	-44.31000	-9.75600
O	-29.33300	-38.17000	-4.83400
H	-28.59200	-37.63100	-5.11000
H	-30.04000	-37.54300	-4.67700

O	-23.85800	-36.02700	-6.23600
H	-23.44900	-36.46800	-6.98100
H	-24.58000	-36.60100	-5.98300
O	-23.53500	-35.46700	-12.48100
H	-24.16400	-35.30200	-13.18400
H	-23.92200	-35.05200	-11.71000
O	-19.65600	-34.53100	-15.12700
H	-20.34100	-34.15100	-15.67700
H	-19.57200	-33.92200	-14.39400
O	-24.35500	-43.42900	-7.20500
H	-24.59900	-42.67500	-7.74200
H	-24.07900	-43.05100	-6.37000
O	-22.40100	-36.88500	-8.52700
H	-22.18900	-36.24200	-9.20400
H	-22.44800	-37.71900	-8.99500
O	-27.98200	-35.72900	-15.21600
H	-27.51800	-35.45000	-16.00600
H	-28.24600	-36.63100	-15.39900
O	-31.09000	-36.18200	-12.27800
H	-30.51900	-35.75300	-12.91700
H	-30.49900	-36.46400	-11.58000
O	-21.28600	-34.00800	-10.35500
H	-20.46400	-34.41000	-10.63700
H	-21.29000	-34.11100	-9.40300
O	-27.52100	-34.46000	-8.19900
H	-27.45600	-33.99800	-7.36300
H	-28.22900	-35.09000	-8.07100
O	-13.19600	-37.66400	-14.00700
H	-13.94900	-38.23300	-14.16200
H	-13.06100	-37.69800	-13.05900
O	-27.80600	-32.79300	-18.33100
H	-27.94100	-31.94800	-17.90300
H	-27.26500	-32.59100	-19.09400
O	-34.35300	-35.27500	-17.75800
H	-33.84000	-34.48100	-17.60800
H	-33.70100	-35.97300	-17.82500
O	-32.54300	-32.15900	-15.67600
H	-32.36700	-31.23700	-15.86400
H	-31.74800	-32.47300	-15.24700
O	-26.17900	-32.17000	-4.20100
H	-26.62300	-32.95700	-4.51900
H	-25.51700	-31.98200	-4.86700
O	-31.42000	-30.64100	-9.67500
H	-30.86900	-30.76900	-10.44700
H	-32.25900	-30.34100	-10.02500
O	-24.08100	-31.89000	-6.17300
H	-24.09900	-31.06900	-6.66500
H	-23.15900	-32.01500	-5.94800
O	-27.62300	-34.37600	-5.33100
H	-27.09100	-35.13700	-5.56100
H	-28.15200	-34.66800	-4.58800
O	-19.51800	-36.52600	-19.35900
H	-20.29500	-36.86500	-19.80300
H	-18.83900	-36.52000	-20.03300
O	-26.25800	-35.24700	-17.81900
H	-26.78600	-35.98200	-18.13100
H	-26.75900	-34.47000	-18.06500
O	-21.27900	-37.05200	-12.70200
H	-21.93700	-36.36200	-12.61400
H	-20.47500	-36.66300	-12.35900
O	-22.46800	-38.69800	-14.65200
H	-21.95500	-38.06500	-14.14900
H	-21.81800	-39.29800	-15.01700
O	-25.20600	-28.71900	-21.89600
H	-25.88000	-28.41600	-21.28700
H	-24.82000	-27.91600	-22.24500
O	-25.99000	-41.22300	-12.40600
H	-26.90800	-41.00100	-12.56400
H	-25.57400	-41.14800	-13.26500
O	-25.20700	-39.82400	-17.27500
H	-24.46000	-39.31200	-17.58500
H	-24.93800	-40.14500	-16.41500
O	-21.70200	-33.71700	-16.52400
H	-22.15600	-34.55800	-16.46700
H	-22.33300	-33.08100	-16.18600
O	-22.75900	-42.78800	-15.29400
H	-23.49700	-43.30000	-14.96300
H	-22.50600	-43.22800	-16.10500
O	-23.25900	-31.88000	-15.58900
H	-22.96700	-31.31900	-16.30700
H	-22.74900	-31.58700	-14.83300
O	-28.38600	-39.71500	-14.19200
H	-28.33100	-40.66800	-14.26800
H	-29.19700	-39.55900	-13.70800
O	-28.29600	-31.10100	-14.49900
H	-28.33800	-30.87500	-15.42800
H	-27.37900	-31.33500	-14.35100
O	-24.84900	-40.63300	-7.60100
H	-24.49400	-40.40600	-6.74200
H	-24.20200	-40.30100	-8.22400
O	-19.11900	-37.51100	-16.89600
H	-19.25700	-37.32100	-17.82400
H	-19.47600	-36.74900	-16.44000
O	-19.09200	-35.44400	-11.88500
H	-18.65900	-35.53600	-11.03700
H	-18.37900	-35.46800	-12.52300
O	-30.17900	-34.22500	-14.70300
H	-29.38500	-34.72000	-14.90500
H	-30.89000	-34.75300	-15.06600
O	-25.35900	-31.84300	-10.35200
H	-25.30600	-31.27400	-9.58400
H	-26.22800	-31.67300	-10.71700
O	-18.23100	-35.40100	-9.35200
H	-18.03700	-34.49700	-9.10600
H	-18.33100	-35.86000	-8.51800
O	-23.16900	-35.25100	-3.77800
H	-23.32100	-35.47900	-4.69500
H	-23.17900	-34.29300	-3.76500
O	-26.40100	-37.67700	-8.79300
H	-25.47100	-37.52600	-8.96600
H	-26.82200	-37.58900	-9.64800
O	-27.60200	-33.65200	-11.02300
H	-27.74100	-34.02000	-10.15100
H	-28.39000	-33.13800	-11.19500
O	-27.00700	-28.55100	-19.68100
H	-26.83200	-29.02300	-18.86600
H	-27.69400	-27.92500	-19.45300
O	-26.05100	-34.27400	-13.63400

H	-26.38600	-34.31400	-12.73900
H	-26.55100	-34.94000	-14.10700
O	-29.44900	-39.15500	-8.62100
H	-28.73900	-39.79600	-8.66200
H	-29.49200	-38.90400	-7.69800
O	-21.46000	-32.21900	-5.70000
H	-21.51900	-32.90300	-6.36700
H	-21.05800	-31.47700	-6.15200
O	-32.44100	-25.40200	-12.90700
H	-32.20100	-24.77100	-12.22900
H	-33.28400	-25.75400	-12.62000
O	-21.45800	-34.25500	-7.46900
H	-22.34600	-34.59200	-7.34600
H	-20.88800	-34.98600	-7.23200
O	-27.92800	-29.59500	-10.50300
H	-28.00100	-28.78900	-9.99100
H	-28.37000	-29.39500	-11.32800
O	-28.13900	-30.35700	-16.99200
H	-28.92200	-29.81500	-16.89900
H	-27.44000	-29.84000	-16.59300
O	-16.97700	-38.84400	-12.70700
H	-16.98800	-37.96100	-13.07800
H	-17.84500	-39.19700	-12.90200
O	-25.46800	-29.69600	-8.34700
H	-24.75500	-29.06100	-8.41900
H	-25.95900	-29.59900	-9.16300
O	-23.74700	-35.79100	-16.77500
H	-24.68100	-35.67100	-16.94500
H	-23.56900	-36.69300	-17.03900
O	-20.35900	-39.94900	-16.24000
H	-19.89300	-39.11600	-16.31400
H	-19.90800	-40.41400	-15.53600
O	-30.41700	-31.94600	-7.52600
H	-29.71600	-31.31300	-7.37300
H	-30.88800	-31.60500	-8.28700
O	-22.29200	-33.60500	-19.24200
H	-22.01600	-33.71900	-18.33200
H	-23.16100	-34.00300	-19.28000
O	-22.61700	-39.39200	-9.70100
H	-23.27000	-39.14400	-10.35600
H	-22.23900	-40.20600	-10.03500
O	-25.65600	-29.35600	-15.59800
H	-25.47400	-30.17400	-15.13500
H	-24.83300	-29.13900	-16.03600
O	-25.32200	-30.42900	-18.33700
H	-24.79300	-30.94000	-18.95000
H	-25.05900	-30.74500	-17.47200
O	-26.56800	-38.65000	-3.21400
H	-27.24400	-38.75100	-2.54400
H	-25.74500	-38.80600	-2.75100
O	-34.82000	-32.41300	-10.59300
H	-34.23500	-33.15800	-10.46000
H	-35.01800	-32.10200	-9.71000
O	-31.96600	-29.37800	-15.57700
H	-32.63500	-28.89300	-16.06100
H	-32.10500	-29.13100	-14.66300
O	-27.82600	-27.34300	-8.97900
H	-27.27400	-27.18000	-8.21400
H	-27.48500	-26.75300	-9.65100
O	-22.97500	-32.51700	-3.12400
H	-22.43100	-31.90400	-3.61800
H	-23.55600	-31.95900	-2.60700
O	-24.68900	-29.39500	-11.99200
H	-24.81400	-30.19600	-12.50100
H	-24.03100	-28.90000	-12.48100
O	-22.62600	-31.59400	-10.36400
H	-23.57300	-31.71400	-10.43700
H	-22.27700	-32.48200	-10.28200
O	-25.62100	-31.79900	-14.16400
H	-25.76800	-32.72700	-13.98300
H	-24.79600	-31.77700	-14.64800
O	-22.01800	-30.97600	-13.07700
H	-21.09300	-31.18200	-13.21500
H	-22.18600	-31.22400	-12.16900
O	-22.22800	-30.45700	-17.72100
H	-22.05400	-31.02300	-18.47400
H	-21.73500	-29.65700	-17.90300
O	-16.37400	-37.69400	-16.10200
H	-15.93100	-38.44300	-16.50000
H	-17.25100	-37.70300	-16.48500
O	-21.24000	-31.14100	-20.62200
H	-20.54500	-30.52000	-20.40300
H	-20.89800	-31.99100	-20.34600
O	-19.34100	-33.69100	-19.09300
H	-18.81600	-33.35200	-19.81800
H	-19.42900	-34.62600	-19.27600
O	-27.67900	-29.77700	-6.96400
H	-27.37100	-30.02300	-6.09200
H	-26.88200	-29.57300	-7.45200
O	-27.65700	-27.62400	-14.73300
H	-28.09700	-28.11600	-14.04000
H	-26.90600	-28.16600	-14.97400
O	-35.17800	-31.43200	-14.73100
H	-34.35200	-31.73200	-15.11100
H	-35.52900	-32.20200	-14.28200
O	-30.21000	-32.08000	-12.66100
H	-30.83200	-32.74300	-12.96100
H	-29.53900	-32.05400	-13.34400
O	-36.97700	-33.51400	-13.77200
H	-37.81400	-33.94200	-13.95400
H	-37.00500	-33.32100	-12.83500
O	-19.65200	-33.05800	-3.78800
H	-20.44200	-33.07500	-4.32700
H	-19.88900	-32.51900	-3.03300
O	-19.53200	-36.20800	-6.47400
H	-19.48200	-36.37300	-5.53300
H	-19.64100	-37.07600	-6.86500
O	-30.64900	-33.52900	-19.18500
H	-30.50000	-32.84600	-19.83900
H	-29.84200	-33.54900	-18.67000
O	-22.24200	-40.22500	-5.15800
H	-21.34100	-40.36500	-5.45000
H	-22.16700	-40.06000	-4.21900
O	-18.03000	-33.78900	-6.23000
H	-18.32200	-33.42600	-5.39400
H	-18.64300	-34.50200	-6.40900
O	-16.83100	-36.22300	-13.71000
H	-15.99600	-35.78400	-13.54600

H	-16.69500	-36.69900	-14.52900
O	-19.78200	-38.66500	-7.76200
H	-19.30600	-38.47300	-8.57000
H	-20.70500	-38.60900	-8.00800
O	-27.14900	-31.03500	-21.71400
H	-27.72200	-30.41600	-22.16700
H	-26.57600	-30.48500	-21.18000
O	-21.08100	-41.78900	-9.52900
H	-20.13000	-41.68900	-9.50800
H	-21.25100	-42.58100	-9.02000
O	-23.92900	-30.75100	-20.58300
H	-22.99600	-30.81600	-20.38000
H	-24.01700	-29.91500	-21.04200
O	-21.30000	-31.43400	-23.62300
H	-21.37900	-31.76800	-22.72900
H	-20.47900	-30.94200	-23.62400
O	-18.60300	-38.56800	-10.41500
H	-18.63500	-39.50700	-10.22900
H	-17.99300	-38.49000	-11.14900
O	-18.45300	-41.77700	-10.36900
H	-18.96900	-42.56100	-10.55600
H	-17.56900	-42.10500	-10.20500
O	-12.78500	-37.50700	-11.27500
H	-12.05500	-37.76800	-10.71400
H	-13.39400	-38.24400	-11.23400
O	-14.70300	-39.36600	-10.67700
H	-15.43000	-39.50000	-11.28500
H	-15.11900	-39.05800	-9.87100
O	-26.88000	-35.64700	-2.91200
H	-25.96000	-35.46900	-2.71800
H	-26.91500	-36.59100	-3.06400
O	-29.14800	-38.73400	-2.06300
H	-29.63200	-37.93400	-1.85700
H	-29.06200	-38.72300	-3.01700
O	-33.22100	-34.84300	-10.02600
H	-32.26700	-34.87500	-10.09300
H	-33.42000	-35.30400	-9.21100
O	-32.63100	-36.27300	-7.45900
H	-31.81200	-35.77900	-7.43600
H	-32.35700	-37.19000	-7.48800
O	-19.52300	-43.85700	-14.38100
H	-18.74700	-43.36800	-14.10900
H	-20.24800	-43.43200	-13.92400
O	-17.08600	-43.14500	-13.32400
H	-16.49100	-43.38500	-14.03400
H	-17.01300	-43.86400	-12.69600
O	-34.86600	-29.30100	-13.00900
H	-35.00100	-30.06100	-13.57600
H	-35.28300	-28.57700	-13.47700
O	-22.90000	-38.36700	-17.88200
H	-22.95700	-37.91000	-18.72100
H	-22.34700	-39.12700	-18.05900
O	-19.09000	-42.57800	-17.05700
H	-19.34600	-42.97800	-16.22600
H	-19.71500	-41.86300	-17.18200
O	-14.77900	-39.83800	-14.54900
H	-15.33900	-39.76900	-13.77600
H	-15.38400	-40.01400	-15.26900
O	-31.70900	-28.40400	-12.79800
H	-31.82600	-27.46000	-12.90000
H	-32.53000	-28.70700	-12.40900
O	-21.82100	-33.67900	-21.95200
H	-20.98700	-34.11100	-22.13400
H	-21.98300	-33.85100	-21.02400
O	-22.16700	-37.17600	-20.58600
H	-22.22200	-37.34200	-21.52700
H	-22.79200	-36.46800	-20.43000
O	-24.85200	-34.87100	-1.26100
H	-23.98300	-34.69700	-1.62200
H	-25.33200	-34.05200	-1.38300
O	-30.68400	-27.75100	-9.58200
H	-29.78100	-27.71900	-9.26500
H	-30.91000	-28.68100	-9.56700
O	-28.92800	-28.99800	-12.92100
H	-29.87900	-28.99800	-13.03500
H	-28.62500	-29.74500	-13.43700
O	-13.19300	-23.16200	-11.85600
H	-12.55500	-23.28200	-12.56000
H	-13.31100	-22.21400	-11.79800
O	-16.38400	-23.21000	-11.92000
H	-16.72200	-23.32500	-12.80800
H	-15.51500	-23.61000	-11.94000
O	-20.52500	-21.35600	-15.93100
H	-21.08900	-21.44700	-16.69900
H	-20.64000	-22.17600	-15.45000
O	-15.90200	-22.05600	-5.96200
H	-15.91300	-22.45100	-6.83400
H	-15.74000	-22.78900	-5.36800
O	-22.97900	-19.86100	-13.09200
H	-22.18700	-20.20200	-13.50800
H	-22.73900	-19.75100	-12.17200
O	-14.94500	-17.68900	-20.61900
H	-15.59700	-17.10400	-21.00600
H	-14.15100	-17.15800	-20.56000
O	-21.02000	-20.60000	-20.22000
H	-20.24700	-21.14700	-20.35900
H	-20.66700	-19.73600	-20.00900
O	-19.18100	-19.27200	-7.01400
H	-19.47200	-18.36800	-7.12900
H	-18.54600	-19.23100	-6.29900
O	-18.25600	-21.89500	-10.42400
H	-17.65900	-22.11900	-11.13800
H	-18.19800	-22.63600	-9.82100
O	-18.68300	-24.44100	-22.58900
H	-18.43100	-24.96300	-23.35000
H	-19.62100	-24.60400	-22.48600
O	-13.63900	-25.43700	-14.78100
H	-12.85300	-25.85500	-15.13200
H	-13.37500	-24.53300	-14.61300
O	-13.46800	-20.00000	-20.08200
H	-14.10300	-19.92600	-19.37000
H	-13.79800	-19.41100	-20.76000
O	-8.23800	-25.50000	-13.87800
H	-7.50500	-26.07000	-13.64400
H	-8.99400	-25.88700	-13.43700
O	-18.96400	-22.53800	-20.38600
H	-19.08000	-23.21300	-19.71800
H	-18.85300	-23.02500	-21.20300

O	-16.62000	-23.07700	-16.68100
H	-16.98500	-23.60400	-15.97000
H	-15.76400	-23.46700	-16.85500
O	-10.71500	-31.78700	-9.41800
H	-11.04700	-30.91800	-9.64200
H	-11.31100	-32.10400	-8.73900
O	-16.98000	-28.04800	-21.68500
H	-16.81200	-27.18000	-21.31800
H	-17.17000	-27.88500	-22.60900
O	-14.22200	-19.58600	-10.01900
H	-14.52200	-18.68400	-10.12700
H	-14.56700	-19.85300	-9.16700
O	-20.77100	-23.13500	-13.98500
H	-21.69600	-23.26700	-13.77700
H	-20.52300	-22.35500	-13.48800
O	-10.82300	-27.06800	-12.33900
H	-11.73100	-27.13600	-12.63300
H	-10.87400	-26.59100	-11.51100
O	-17.00100	-25.59200	-20.70100
H	-17.12100	-25.14600	-19.86200
H	-17.69600	-25.24400	-21.25900
O	-23.41000	-23.49200	-13.15100
H	-23.28100	-23.23800	-12.23700
H	-24.09000	-22.89900	-13.47000
O	-19.24000	-12.82200	-17.05200
H	-19.31800	-11.87100	-16.97300
H	-19.84400	-13.16700	-16.39400
O	-16.74800	-25.64900	-4.39600
H	-17.52500	-25.09000	-4.40500
H	-16.10900	-25.16300	-3.87400
O	-8.95600	-28.86600	-10.56100
H	-9.59900	-28.57300	-11.20700
H	-9.46100	-28.98100	-9.75600
O	-11.30200	-25.12000	-19.87300
H	-10.39100	-24.88700	-20.04800
H	-11.81100	-24.53600	-20.43500
O	-18.49400	-22.84000	-4.83400
H	-17.75200	-22.30200	-5.11000
H	-19.20000	-22.21400	-4.67700
O	-13.01800	-20.69800	-6.23600
H	-12.61000	-21.13900	-6.98100
H	-13.74100	-21.27200	-5.98300
O	-16.66500	-25.76000	-8.86300
H	-15.81900	-25.33600	-8.72000
H	-16.72500	-26.41700	-8.16900
O	-12.69500	-20.13700	-12.48100
H	-13.32500	-19.97300	-13.18400
H	-13.08300	-19.72300	-11.71000
O	-8.81700	-19.20200	-15.12700
H	-9.50100	-18.82200	-15.67700
H	-8.73200	-18.59200	-14.39400
O	-13.51500	-28.10000	-7.20500
H	-13.75900	-27.34600	-7.74200
H	-13.23900	-27.72100	-6.37000
O	-11.56100	-21.55600	-8.52700
H	-11.35000	-20.91300	-9.20400
H	-11.60800	-22.39000	-8.99500
O	-13.95900	-28.94400	-11.62400
H	-14.82000	-28.64600	-11.33200
H	-13.93300	-28.72700	-12.55600
O	-17.14300	-20.40000	-15.21600
H	-16.67900	-20.12100	-16.00600
H	-17.40700	-21.30200	-15.39900
O	-20.25000	-20.85300	-12.27800
H	-19.68000	-20.42400	-12.91700
H	-19.65900	-21.13500	-11.58000
O	-18.20600	-24.27800	-18.40700
H	-17.50300	-23.82600	-17.94000
H	-18.53800	-24.91900	-17.77900
O	-19.75800	-25.68600	-4.03200
H	-20.38800	-25.00500	-3.79900
H	-19.86400	-25.79800	-4.97700
O	-24.46300	-26.01900	-13.10600
H	-25.25300	-25.76400	-13.58200
H	-23.97200	-25.20400	-13.00000
O	-10.44700	-18.67900	-10.35500
H	-9.62500	-19.08100	-10.63700
H	-10.45100	-18.78200	-9.40300
O	-22.51200	-25.65500	-16.14700
H	-23.30900	-25.54300	-15.62900
H	-22.73900	-25.31600	-17.01300
O	-16.68100	-19.13000	-8.19900
H	-16.61700	-18.66800	-7.36300
H	-17.39000	-19.76100	-8.07100
O	-26.17900	-25.76600	-10.67400
H	-25.92900	-24.88200	-10.40800
H	-25.64300	-25.94600	-11.44700
O	-2.35600	-22.33500	-14.00700
H	-3.11000	-22.90400	-14.16200
H	-2.22100	-22.36900	-13.05900
O	-16.96600	-17.46400	-18.33100
H	-17.10200	-16.61800	-17.90300
H	-16.42500	-17.26200	-19.09400
O	-20.48000	-31.38400	-1.67000
H	-20.87300	-31.93800	-0.99500
H	-19.66300	-31.07300	-1.27900
O	-23.51300	-19.94600	-17.75800
H	-23.00100	-19.15200	-17.60800
H	-22.86100	-20.64300	-17.82500
O	-21.70400	-16.82900	-15.67600
H	-21.52700	-15.90800	-15.86400
H	-20.90800	-17.14400	-15.24700
O	-17.56300	-29.96900	-4.07800
H	-16.82200	-29.36800	-4.15700
H	-17.33100	-30.71100	-4.63600
O	-22.41900	-24.78300	-5.80000
H	-22.53300	-25.68400	-5.49900
H	-23.27400	-24.53700	-6.15100
O	-15.34000	-16.84100	-4.20100
H	-15.78300	-17.62800	-4.51900
H	-14.67800	-16.65300	-4.86700
O	-20.58100	-15.31200	-9.67500
H	-20.03000	-15.44000	-10.44700
H	-21.42000	-15.01200	-10.02500
O	-13.24100	-16.56100	-6.17300
H	-13.26000	-15.74000	-6.66500
H	-12.31900	-16.68600	-5.94800
O	-16.78400	-19.04700	-5.33100

H	-16.25100	-19.80800	-5.56100
H	-17.31300	-19.33900	-4.58800
O	-13.61700	-33.94100	-9.80100
H	-13.96100	-33.05000	-9.87700
H	-13.24900	-34.13000	-10.66400
O	-25.54900	-21.61700	-16.98200
H	-24.71100	-21.20700	-17.19900
H	-25.37100	-22.55700	-17.01200
O	-13.65200	-23.95000	-21.29400
H	-14.16700	-23.17900	-21.05500
H	-13.92400	-24.62300	-20.67100
O	-16.06700	-29.10500	-16.23500
H	-16.92300	-29.49400	-16.41500
H	-15.65500	-29.02500	-17.09500
O	-14.53700	-31.57000	-11.83600
H	-15.04100	-31.60700	-12.64900
H	-14.24400	-30.66100	-11.77700
O	-18.29300	-31.48600	-12.83000
H	-17.42800	-31.68200	-13.18900
H	-18.22500	-31.71500	-11.90300
O	-8.67900	-21.19600	-19.35900
H	-9.45600	-21.53500	-19.80300
H	-8.00000	-21.19100	-20.03300
O	-13.86300	-26.07700	-19.46800
H	-14.16800	-25.76200	-18.61700
H	-12.91000	-25.99900	-19.42500
O	-15.41900	-19.91800	-17.81900
H	-15.94700	-20.65300	-18.13100
H	-15.92000	-19.14000	-18.06500
O	-10.44000	-21.72300	-12.70200
H	-11.09700	-21.03300	-12.61400
H	-9.63600	-21.33300	-12.35900
O	-20.68700	-29.25600	-15.45800
H	-20.11300	-29.47800	-16.19100
H	-20.10000	-29.18700	-14.70500
O	-11.62900	-23.36900	-14.65200
H	-11.11600	-22.73600	-14.14900
H	-10.97900	-23.96800	-15.01700
O	-20.36000	-27.22600	-21.43800
H	-21.19600	-26.88800	-21.11800
H	-20.50000	-27.36100	-22.37600
O	-18.38500	-32.38200	-16.73100
H	-19.03800	-32.69700	-17.35500
H	-18.42700	-33.00400	-16.00500
O	-17.27400	-22.10000	-24.81500
H	-18.18100	-22.33100	-24.61600
H	-16.77600	-22.89600	-24.62800
O	-14.36700	-13.39000	-21.89600
H	-15.04100	-13.08700	-21.28700
H	-13.98000	-12.58700	-22.24500
O	-15.15100	-25.89400	-12.40600
H	-16.06900	-25.67200	-12.56400
H	-14.73500	-25.81900	-13.26500
O	-14.36700	-24.49500	-17.27500
H	-13.62000	-23.98200	-17.58500
H	-14.09800	-24.81600	-16.41500
O	-16.09500	-21.99900	-20.29300
H	-17.00400	-22.28300	-20.19600
H	-16.06500	-21.57900	-21.15200
O	-10.86300	-18.38800	-16.52400
H	-11.31700	-19.22900	-16.46700
H	-11.49300	-17.75200	-16.18600
O	-11.92000	-27.45900	-15.29400
H	-12.65700	-27.97100	-14.96300
H	-11.66700	-27.89900	-16.10500
O	-12.42000	-16.55000	-15.58900
H	-12.12700	-15.99000	-16.30700
H	-11.91000	-16.25700	-14.83300
O	-11.81800	-34.68100	-14.69900
H	-11.51200	-35.54100	-14.98700
H	-11.09300	-34.08900	-14.90000
O	-25.52600	-24.27800	-16.31100
H	-25.72300	-24.87100	-17.03600
H	-26.08100	-24.58300	-15.59400
O	-17.54600	-24.38600	-14.19200
H	-17.49100	-25.33800	-14.26800
H	-18.35700	-24.23000	-13.70800
O	-16.39700	-20.45800	-22.95400
H	-16.83500	-21.17400	-23.41300
H	-15.65600	-20.23000	-23.51500
O	-19.60700	-25.76800	-16.43300
H	-20.42000	-25.46900	-16.02600
H	-19.11100	-26.16300	-15.71500
O	-17.45600	-15.77200	-14.49900
H	-17.49900	-15.54600	-15.42800
H	-16.54000	-16.00600	-14.35100
O	-14.01000	-25.30400	-7.60100
H	-13.65500	-25.07700	-6.74200
H	-13.36200	-24.97200	-8.22400
O	-8.28000	-22.18200	-16.89600
H	-8.41800	-21.99200	-17.82400
H	-8.63600	-21.42000	-16.44000
O	-20.11600	-28.19600	-10.81700
H	-19.47600	-28.33700	-11.51400
H	-20.94800	-28.48300	-11.19300
O	-8.25300	-20.11400	-11.88500
H	-7.81900	-20.20700	-11.03700
H	-7.54000	-20.13900	-12.52300
O	-19.34000	-18.89600	-14.70300
H	-18.54600	-19.39100	-14.90500
H	-20.05000	-19.42400	-15.06600
O	-14.52000	-16.51400	-10.35200
H	-14.46600	-15.94500	-9.58400
H	-15.38900	-16.34400	-10.71700
O	-23.03100	-24.75200	-18.62700
H	-23.96200	-24.81300	-18.41000
H	-22.93600	-25.27400	-19.42400
O	-23.11300	-28.22600	-16.03900
H	-22.85200	-27.31200	-16.14800
H	-22.29100	-28.69400	-15.89000
O	-21.64000	-26.81200	-8.86900
H	-20.96800	-27.02100	-9.51800
H	-22.46700	-27.01000	-9.30900
O	-7.39100	-20.07200	-9.35200
H	-7.19800	-19.16700	-9.10600
H	-7.49100	-20.53100	-8.51800
O	-15.73800	-30.18600	-6.85300
H	-16.38500	-29.48700	-6.94600

H	-14.96800	-29.75300	-6.48500
O	-12.33000	-19.92100	-3.77800
H	-12.48100	-20.15000	-4.69500
H	-12.33900	-18.96400	-3.76500
O	-15.56100	-22.34800	-8.79300
H	-14.63200	-22.19700	-8.96600
H	-15.98200	-22.26000	-9.64800
O	-16.76200	-18.32300	-11.02300
H	-16.90100	-18.69100	-10.15100
H	-17.55100	-17.80800	-11.19500
O	-16.16800	-13.22200	-19.68100
H	-15.99200	-13.69300	-18.86600
H	-16.85400	-12.59600	-19.45300
O	-15.21200	-18.94500	-13.63400
H	-15.54700	-18.98500	-12.73900
H	-15.71200	-19.61000	-14.10700
O	-21.23800	-23.53800	-8.10900
H	-21.37600	-24.27000	-7.50800
H	-20.32700	-23.62700	-8.39100
O	-12.27600	-21.59900	-23.36100
H	-12.89300	-22.32500	-23.45700
H	-12.74400	-20.83800	-23.70400
O	-18.60900	-23.82600	-8.62100
H	-17.89900	-24.46700	-8.66200
H	-18.65200	-23.57500	-7.69800
O	-10.62100	-16.88900	-5.70000
H	-10.67900	-17.57400	-6.36700
H	-10.21900	-16.14800	-6.15200
O	-19.89600	-26.84000	-6.80100
H	-20.43300	-27.29100	-6.14900
H	-20.48500	-26.70500	-7.54400
O	-21.60200	-10.07300	-12.90700
H	-21.36200	-9.44100	-12.22900
H	-22.44500	-10.42400	-12.62000
O	-20.48600	-27.38100	-24.23900
H	-21.36300	-27.50200	-24.60200
H	-20.01000	-28.17200	-24.49100
O	-10.61900	-18.92500	-7.46900
H	-11.50600	-19.26300	-7.34600
H	-10.04900	-19.65700	-7.23200
O	-28.96600	-22.59300	-14.14400
H	-28.27900	-22.90700	-14.73200
H	-29.12100	-21.68900	-14.41800
O	-24.16200	-27.70400	-9.71600
H	-24.24700	-28.31100	-10.45100
H	-24.98400	-27.21300	-9.71500
O	-17.08900	-14.26500	-10.50300
H	-17.16200	-13.46000	-9.99100
H	-17.53100	-14.06600	-11.32800
O	-17.30000	-15.02800	-16.99200
H	-18.08300	-14.48600	-16.89900
H	-16.60000	-14.51100	-16.59300
O	-13.99000	-29.21900	-14.34900
H	-13.49300	-29.97200	-14.66900
H	-14.76100	-29.18200	-14.91500
O	-6.13700	-23.51500	-12.70700
H	-6.14800	-22.63200	-13.07800
H	-7.00500	-23.86800	-12.90200
O	-14.62900	-14.36700	-8.34700
H	-13.91600	-13.73200	-8.41900
H	-15.12000	-14.27000	-9.16300
O	-12.90700	-20.46200	-16.77500
H	-13.84100	-20.34100	-16.94500
H	-12.72900	-21.36400	-17.03900
O	-17.40700	-27.07700	-14.62200
H	-17.77700	-27.63000	-13.93400
H	-16.91100	-27.68100	-15.17400
O	-9.52000	-24.62000	-16.24000
H	-9.05400	-23.78700	-16.31400
H	-9.06900	-25.08500	-15.53600
O	-19.57800	-16.61700	-7.52600
H	-18.87700	-15.98400	-7.37300
H	-20.04800	-16.27600	-8.28700
O	-11.45200	-18.27500	-19.24200
H	-11.17600	-18.39000	-18.33200
H	-12.32200	-18.67400	-19.28000
O	-11.54800	-32.25200	-12.20100
H	-12.46200	-32.02100	-12.03300
H	-11.11900	-32.16800	-11.35000
O	-11.77800	-24.06300	-9.70100
H	-12.43000	-23.81500	-10.35600
H	-11.40000	-24.87600	-10.03500
O	-14.81700	-14.02700	-15.59800
H	-14.63500	-14.84500	-15.13500
H	-13.99400	-13.81000	-16.03600
O	-14.48300	-15.10000	-18.33700
H	-13.95400	-15.61100	-18.95000
H	-14.22000	-15.41600	-17.47200
O	-15.72900	-23.32100	-3.21400
H	-16.40500	-23.42100	-2.54400
H	-14.90600	-23.47700	-2.75100
O	-23.98100	-17.08400	-10.59300
H	-23.39600	-17.82900	-10.46000
H	-24.17800	-16.77300	-9.71000
O	-21.12700	-14.04800	-15.57700
H	-21.79600	-13.56400	-16.06100
H	-21.26600	-13.80200	-14.66300
O	-29.38900	-24.51300	-11.87300
H	-29.35100	-25.04700	-11.07900
H	-29.89500	-25.03900	-12.49200
O	-16.98700	-12.01400	-8.97900
H	-16.43500	-11.85100	-8.21400
H	-16.64600	-11.42400	-9.65100
O	-22.01000	-29.66700	-8.37600
H	-21.94600	-28.80500	-8.78900
H	-22.07300	-30.27900	-9.10900
O	-22.53500	-27.59400	-4.72900
H	-21.90800	-27.97700	-4.11500
H	-23.39200	-27.81300	-4.36400
O	-12.13500	-17.18800	-3.12400
H	-11.59100	-16.57500	-3.61800
H	-12.71700	-16.63000	-2.60700
O	-25.75800	-25.78700	-18.79700
H	-25.46300	-26.69600	-18.85100
H	-26.65900	-25.80800	-19.11900
O	-13.85000	-14.06600	-11.99200
H	-13.97500	-14.86700	-12.50100
H	-13.19200	-13.57100	-12.48100

O	-22.22900	-27.71100	-12.81500
H	-22.95000	-27.09700	-12.95400
H	-21.44000	-27.17500	-12.89300
O	-11.78600	-16.26500	-10.36400
H	-12.73300	-16.38400	-10.43700
H	-11.43800	-17.15300	-10.28200
O	-19.42600	-30.37600	-6.54100
H	-18.90300	-30.12900	-5.77800
H	-19.64500	-29.54400	-6.96000
O	-24.03000	-28.10500	-18.67300
H	-24.35800	-28.98500	-18.85600
H	-23.67600	-28.16000	-17.78600
O	-14.78200	-16.47000	-14.16400
H	-14.92900	-17.39800	-13.98300
H	-13.95600	-16.44800	-14.64800
O	-20.91500	-27.81800	-18.30600
H	-20.57600	-27.28900	-19.02800
H	-20.46800	-27.48000	-17.53000
O	-11.17800	-15.64700	-13.07700
H	-10.25300	-15.85300	-13.21500
H	-11.34700	-15.89500	-12.16900
O	-18.22100	-29.43400	-8.85600
H	-18.56100	-30.23300	-8.45300
H	-18.92700	-29.13400	-9.42800
O	-17.56900	-32.34900	-10.40300
H	-16.74700	-32.82300	-10.52500
H	-17.32600	-31.57000	-9.90300
O	-18.92900	-29.32000	-20.10700
H	-18.20800	-28.94200	-20.61000
H	-19.55200	-28.60200	-20.00300
O	-14.78500	-28.61600	-19.17100
H	-14.56900	-27.73300	-19.47200
H	-15.26600	-29.00700	-19.90000
O	-20.04500	-25.65700	-12.59400
H	-20.19100	-25.34300	-11.70100
H	-20.52300	-25.03900	-13.14700
O	-18.18600	-28.66700	-12.54600
H	-18.20000	-29.56300	-12.88200
H	-17.46900	-28.66000	-11.91200
O	-16.33300	-28.23300	-10.50000
H	-16.59100	-27.31200	-10.50200
H	-16.86500	-28.63000	-9.81000
O	-11.38900	-15.12800	-17.72100
H	-11.21500	-15.69400	-18.47400
H	-10.89600	-14.32800	-17.90300
O	-25.57100	-23.11700	-9.65500
H	-26.11600	-22.34100	-9.52300
H	-24.69300	-22.77000	-9.81300
O	-18.78200	-29.78600	-17.27700
H	-18.66100	-30.71400	-17.07800
H	-19.07200	-29.77200	-18.18900
O	-15.91300	-32.10000	-14.14000
H	-15.89100	-32.03600	-15.09500
H	-15.49600	-32.94000	-13.94600
O	-5.53500	-22.36500	-16.10200
H	-5.09200	-23.11400	-16.50000
H	-6.41200	-22.37400	-16.48500
O	-10.40100	-15.81200	-20.62200
H	-9.70600	-15.19100	-20.40300
H	-10.05900	-16.66200	-20.34600
O	-24.78400	-23.99000	-6.76200
H	-25.52600	-23.84200	-6.17500
H	-25.10500	-23.72900	-7.62500
O	-8.50200	-18.36200	-19.09300
H	-7.97700	-18.02300	-19.81800
H	-8.59000	-19.29700	-19.27600
O	-16.84000	-14.44800	-6.96400
H	-16.53100	-14.69300	-6.09200
H	-16.04200	-14.24400	-7.45200
O	-16.81800	-12.29400	-14.73300
H	-17.25700	-12.78700	-14.04000
H	-16.06700	-12.83700	-14.97400
O	-24.33900	-16.10300	-14.73100
H	-23.51300	-16.40300	-15.11100
H	-24.68900	-16.87300	-14.28200
O	-19.37000	-16.75100	-12.66100
H	-19.99200	-17.41400	-12.96100
H	-18.70000	-16.72500	-13.34400
O	-26.13800	-18.18500	-13.77200
H	-26.97500	-18.61200	-13.95400
H	-26.16500	-17.99100	-12.83500
O	-19.29800	-28.27200	-3.06800
H	-19.24900	-27.39600	-3.45000
H	-18.75600	-28.81500	-3.64100
O	-8.81300	-17.72800	-3.78800
H	-9.60300	-17.74600	-4.32700
H	-9.04900	-17.19000	-3.03300
O	-8.69300	-20.87900	-6.47400
H	-8.64300	-21.04400	-5.53300
H	-8.80100	-21.74600	-6.86500
O	-19.80900	-18.20000	-19.18500
H	-19.66000	-17.51700	-19.83900
H	-19.00200	-18.22000	-18.67000
O	-11.40200	-24.89600	-5.15800
H	-10.50100	-25.03500	-5.45000
H	-11.32800	-24.73100	-4.21900
O	-17.06800	-27.51900	-6.31300
H	-17.93800	-27.22600	-6.58500
H	-16.83200	-26.93500	-5.59200
O	-7.19100	-18.45900	-6.23000
H	-7.48200	-18.09600	-5.39400
H	-7.80400	-19.17300	-6.40900
O	-5.99100	-20.89400	-13.71000
H	-5.15700	-20.45500	-13.54600
H	-5.85500	-21.37000	-14.52900
O	-8.94200	-23.33600	-7.76200
H	-8.46700	-23.14300	-8.57000
H	-9.86600	-23.28000	-8.00800
O	-22.05400	-22.17400	-18.17900
H	-22.29900	-23.07700	-17.98100
H	-21.88400	-22.17200	-19.12100
O	-14.36400	-31.30100	-8.79700
H	-13.84600	-30.56500	-9.12200
H	-14.71900	-30.99500	-7.96200
O	-22.78400	-26.44100	-20.68000
H	-23.47700	-26.36600	-21.33600
H	-23.02900	-27.20500	-20.15800
O	-14.11300	-34.88100	-13.27700

H	-13.83300	-35.54900	-12.65000
H	-13.32800	-34.68800	-13.78900
O	-16.31000	-15.70500	-21.71400
H	-16.88200	-15.08600	-22.16700
H	-15.73600	-15.15500	-21.18000
O	-10.24200	-26.46000	-9.52900
H	-9.29000	-26.36000	-9.50800
H	-10.41200	-27.25200	-9.02000
O	-12.22100	-29.37400	-9.34300
H	-12.81600	-29.09400	-10.03900
H	-12.50600	-28.88500	-8.57200
O	-13.09000	-15.42200	-20.58300
H	-12.15700	-15.48700	-20.38000
H	-13.17800	-14.58600	-21.04200
O	-10.46100	-16.10500	-23.62300
H	-10.54000	-16.43900	-22.72900
H	-9.64000	-15.61300	-23.62400
O	-7.76300	-23.23900	-10.41500
H	-7.79600	-24.17700	-10.22900
H	-7.15300	-23.16100	-11.14900
O	-15.92500	-24.36900	-24.37200
H	-14.97900	-24.49900	-24.30400
H	-16.30300	-25.10800	-23.89500
O	-7.61400	-26.44800	-10.36900
H	-8.13000	-27.23200	-10.55600
H	-6.73000	-26.77600	-10.20500
O	-9.95300	-32.70400	-14.41000
H	-10.15000	-33.01200	-13.52500
H	-9.34300	-31.97800	-14.28000
O	-1.94600	-22.17700	-11.27500
H	-1.21500	-22.43900	-10.71400
H	-2.55500	-22.91400	-11.23400
O	-3.86400	-24.03700	-10.67700
H	-4.59100	-24.17100	-11.28500
H	-4.27900	-23.72900	-9.87100
O	-16.04100	-20.31800	-2.91200
H	-15.12100	-20.14000	-2.71800
H	-16.07600	-21.26200	-3.06400
O	-17.74100	-26.44400	-24.43700
H	-18.64700	-26.74100	-24.36100
H	-17.46300	-26.74100	-25.30400
O	-26.82300	-25.12400	-13.98700
H	-27.14300	-25.97700	-14.28000
H	-27.36900	-24.90900	-13.23100
O	-23.04500	-22.40700	-10.27000
H	-22.82600	-21.47600	-10.25100
H	-22.63200	-22.76700	-9.48500
O	-16.12400	-31.90100	-18.28300
H	-16.93400	-32.24600	-17.90700
H	-15.43600	-32.21000	-17.69400
O	-14.19500	-32.82300	-16.66000
H	-13.64200	-32.40300	-16.00100
H	-13.95800	-33.74900	-16.61800
O	-18.17200	-26.83400	-0.88000
H	-18.27200	-25.88500	-0.94900
H	-18.39000	-27.16200	-1.75200
O	-18.97100	-29.58600	-24.29600
H	-18.10900	-29.26000	-24.03600
H	-18.78600	-30.37300	-24.80800
O	-26.95400	-21.50300	-12.01800
H	-27.64800	-21.91800	-12.53100
H	-26.20700	-21.46100	-12.61400
O	-10.81700	-30.71300	-17.15200
H	-11.39800	-30.05400	-17.53400
H	-11.08400	-31.53500	-17.56300
O	-12.36700	-31.31200	-14.95000
H	-11.86700	-31.20100	-15.75900
H	-11.72400	-31.61600	-14.31000
O	-18.30900	-23.40400	-2.06300
H	-18.79200	-22.60400	-1.85700
H	-18.22300	-23.39400	-3.01700
O	-25.04000	-21.57200	-14.14100
H	-25.06300	-21.47300	-15.09300
H	-24.73000	-20.72500	-13.81900
O	-22.38200	-19.51400	-10.02600
H	-21.42700	-19.54600	-10.09300
H	-22.58000	-19.97500	-9.21100
O	-21.79200	-20.94400	-7.45900
H	-20.97200	-20.44900	-7.43600
H	-21.51800	-21.86100	-7.48800
O	-12.03500	-28.55300	-18.09700
H	-12.92200	-28.26800	-18.31500
H	-11.62000	-28.71300	-18.94400
O	-8.68400	-28.52800	-14.38100
H	-7.90800	-28.03900	-14.10900
H	-9.40900	-28.10200	-13.92400
O	-6.24600	-27.81500	-13.32400
H	-5.65200	-28.05600	-14.03400
H	-6.17400	-28.53500	-12.69600
O	-24.02700	-13.97200	-13.00900
H	-24.16100	-14.73200	-13.57600
H	-24.44300	-13.24800	-13.47700
O	-12.89100	-18.78800	-24.00200
H	-12.18700	-18.58300	-23.38600
H	-13.61400	-18.22100	-23.73700
O	-12.06100	-23.03700	-17.88200
H	-12.11700	-22.58000	-18.72100
H	-11.50800	-23.79800	-18.05900
O	-8.25000	-27.24800	-17.05700
H	-8.50700	-27.64900	-16.22600
H	-8.87500	-26.53400	-17.18200
O	-3.93900	-24.50900	-14.54900
H	-4.49900	-24.43900	-13.77600
H	-4.54500	-24.68500	-15.26900
O	-20.87000	-13.07500	-12.79800
H	-20.98600	-12.13100	-12.90000
H	-21.69000	-13.37800	-12.40900
O	-10.98200	-18.35000	-21.95200
H	-10.14700	-18.78200	-22.13400
H	-11.14400	-18.52200	-21.02400
O	-11.32700	-21.84700	-20.58600
H	-11.38300	-22.01200	-21.52700
H	-11.95300	-21.13900	-20.43000
O	-26.53800	-26.61000	-6.76000
H	-25.89200	-27.12000	-6.27100
H	-26.74600	-25.87300	-6.18600
O	-14.01300	-19.54200	-1.26100
H	-13.14400	-19.36800	-1.62200

H	-14.49200	-18.72300	-1.38300
O	-18.01800	-32.34600	-21.00800
H	-18.03000	-31.40200	-20.85100
H	-17.12500	-32.52900	-21.30200
O	-19.84400	-12.42200	-9.58200
H	-18.94100	-12.39000	-9.26500
H	-20.07000	-13.35200	-9.56700
O	-18.08900	-13.66900	-12.92100
H	-19.03900	-13.66900	-13.03500
H	-17.78600	-14.41600	-13.43700
O	-14.20300	-35.75200	-16.75300
H	-14.96900	-36.30600	-16.60800
H	-13.79200	-35.68000	-15.89200
O	-22.19800	-29.46300	-2.66300
H	-21.33500	-29.82000	-2.45200
H	-22.77200	-29.80200	-1.97500
O	-2.35400	-7.83300	-11.85600
H	-1.71600	-7.95200	-12.56000
H	-2.47100	-6.88500	-11.79800
O	-5.54500	-7.88000	-11.92000
H	-5.88300	-7.99600	-12.80800
H	-4.67600	-8.28100	-11.94000
O	-9.68600	-6.02700	-15.93100
H	-10.24900	-6.11800	-16.69900
H	-9.80100	-6.84700	-15.45000
O	-5.06300	-6.72700	-5.96200
H	-5.07400	-7.12200	-6.83400
H	-4.90100	-7.46000	-5.36800
O	-12.13900	-4.53200	-13.09200
H	-11.34700	-4.87300	-13.50800
H	-11.90000	-4.42200	-12.17200
O	-4.10600	-2.36000	-20.61900
H	-4.75700	-1.77400	-21.00600
H	-3.31200	-1.82900	-20.56000
O	-8.34200	-3.94300	-7.01400
H	-8.63300	-3.03900	-7.12900
H	-7.70600	-3.90200	-6.29900
O	-7.41600	-6.56500	-10.42400
H	-6.82000	-6.79000	-11.13800
H	-7.35900	-7.30700	-9.82100
O	-7.84400	-9.11200	-22.58900
H	-7.59100	-9.63300	-23.35000
H	-8.78100	-9.27500	-22.48600
O	-2.79900	-10.10800	-14.78100
H	-2.01300	-10.52600	-15.13200
H	-2.53600	-9.20300	-14.61300
O	2.60100	-10.17000	-13.87800
H	3.33400	-10.74100	-13.64400
H	1.84500	-10.55800	-13.43700
O	-5.78100	-7.74800	-16.68100
H	-6.14500	-8.27500	-15.97000
H	-4.92400	-8.13800	-16.85500
O	0.12400	-16.45800	-9.41800
H	-0.20800	-15.58900	-9.64200
H	-0.47200	-16.77500	-8.73900
O	-6.14000	-12.71900	-21.68500
H	-5.97300	-11.85100	-21.31800
H	-6.33100	-12.55600	-22.60900
O	-3.38300	-4.25700	-10.01900
H	-3.68300	-3.35500	-10.12700
H	-3.72800	-4.52400	-9.16700
O	-9.93200	-7.80600	-13.98500
H	-10.85700	-7.93800	-13.77700
H	-9.68300	-7.02600	-13.48800
O	0.01700	-11.73900	-12.33900
H	-0.89200	-11.80700	-12.63300
H	-0.03400	-11.26200	-11.51100
O	-6.16200	-10.26300	-20.70100
H	-6.28200	-9.81700	-19.86200
H	-6.85600	-9.91400	-21.25900
O	-12.57100	-8.16200	-13.15100
H	-12.44200	-7.90900	-12.23700
H	-13.25100	-7.57000	-13.47000
O	-8.40100	2.50700	-17.05200
H	-8.47800	3.45800	-16.97300
H	-9.00500	2.16200	-16.39400
O	-5.90900	-10.32000	-4.39600
H	-6.68600	-9.76000	-4.40500
H	-5.27000	-9.83400	-3.87400
O	1.88300	-13.53700	-10.56100
H	1.24000	-13.24400	-11.20700
H	1.37800	-13.65200	-9.75600
O	-0.46300	-9.79100	-19.87300
H	0.44900	-9.55800	-20.04800
H	-0.97200	-9.20700	-20.43500
O	-7.65400	-7.51100	-4.83400
H	-6.91300	-6.97300	-5.11000
H	-8.36100	-6.88500	-4.67700
O	-2.17900	-5.36800	-6.23600
H	-1.77100	-5.81000	-6.98100
H	-2.90100	-5.94300	-5.98300
O	-5.82600	-10.43100	-8.86300
H	-4.98000	-10.00700	-8.72000
H	-5.88600	-11.08800	-8.16900
O	-1.85600	-4.80800	-12.48100
H	-2.48500	-4.64400	-13.18400
H	-2.24300	-4.39400	-11.71000
O	2.02300	-3.87300	-15.12700
H	1.33800	-3.49300	-15.67700
H	2.10700	-3.26300	-14.39400
O	-2.67600	-12.77100	-7.20500
H	-2.92000	-12.01600	-7.74200
H	-2.40000	-12.39200	-6.37000
O	-0.72200	-6.22700	-8.52700
H	-0.51000	-5.58400	-9.20400
H	-0.76900	-7.06100	-8.99500
O	-3.11900	-13.61500	-11.62400
H	-3.98100	-13.31700	-11.33200
H	-3.09300	-13.39800	-12.55600
O	-6.30300	-5.07100	-15.21600
H	-5.83900	-4.79200	-16.00600
H	-6.56800	-5.97200	-15.39900
O	-9.41100	-5.52400	-12.27800
H	-8.84100	-5.09500	-12.91700
H	-8.82000	-5.80500	-11.58000
O	-7.36600	-8.94800	-18.40700
H	-6.66400	-8.49600	-17.94000
H	-7.69800	-9.59000	-17.77900

O	-8.91800	-10.35700	-4.03200
H	-9.54800	-9.67600	-3.79900
H	-9.02500	-10.46900	-4.97700
O	-13.62400	-10.69000	-13.10600
H	-14.41400	-10.43400	-13.58200
H	-13.13300	-9.87500	-13.00000
O	0.39300	-3.35000	-10.35500
H	1.21400	-3.75200	-10.63700
H	0.38900	-3.45300	-9.40300
O	-11.67200	-10.32600	-16.14700
H	-12.46900	-10.21400	-15.62900
H	-11.90000	-9.98700	-17.01300
O	-5.84200	-3.80100	-8.19900
H	-5.77700	-3.33900	-7.36300
H	-6.55100	-4.43100	-8.07100
O	-15.34000	-10.43700	-10.67400
H	-15.08900	-9.55200	-10.40800
H	-14.80400	-10.61700	-11.44700
O	8.48300	-7.00600	-14.00700
H	7.73000	-7.57500	-14.16200
H	8.61800	-7.03900	-13.05900
O	-9.64000	-16.05500	-1.67000
H	-10.03400	-16.60900	-0.99500
H	-8.82400	-15.74400	-1.27900
O	-12.67400	-4.61700	-17.75800
H	-12.16200	-3.82300	-17.60800
H	-12.02200	-5.31400	-17.82500
O	-10.86500	-1.50000	-15.67600
H	-10.68800	-0.57800	-15.86400
H	-10.06900	-1.81400	-15.24700
O	-6.72300	-14.63900	-4.07800
H	-5.98300	-14.03900	-4.15700
H	-6.49200	-15.38200	-4.63600
O	-11.57900	-9.45400	-5.80000
H	-11.69400	-10.35500	-5.49900
H	-12.43500	-9.20700	-6.15100
O	-9.74100	0.01800	-9.67500
H	-9.19100	-0.11100	-10.44700
H	-10.58000	0.31700	-10.02500
O	-5.94500	-3.71700	-5.33100
H	-5.41200	-4.47900	-5.56100
H	-6.47300	-4.01000	-4.58800
O	-2.77800	-18.61100	-9.80100
H	-3.12100	-17.72100	-9.87700
H	-2.41000	-18.80100	-10.66400
O	-14.70900	-6.28800	-16.98200
H	-13.87200	-5.87800	-17.19900
H	-14.53100	-7.22800	-17.01200
O	-5.22800	-13.77500	-16.23500
H	-6.08400	-14.16500	-16.41500
H	-4.81500	-13.69600	-17.09500
O	-3.69800	-16.24100	-11.83600
H	-4.20200	-16.27800	-12.64900
H	-3.40500	-15.33200	-11.77700
O	-7.45400	-16.15700	-12.83000
H	-6.58900	-16.35300	-13.18900
H	-7.38600	-16.38600	-11.90300
O	2.16100	-5.86700	-19.35900
H	1.38400	-6.20600	-19.80300
H	2.84000	-5.86200	-20.03300
O	-3.02400	-10.74800	-19.46800
H	-3.32900	-10.43300	-18.61700
H	-2.07100	-10.67000	-19.42500
O	0.40000	-6.39400	-12.70200
H	-0.25800	-5.70400	-12.61400
H	1.20400	-6.00400	-12.35900
O	-9.84800	-13.92700	-15.45800
H	-9.27300	-14.14900	-16.19100
H	-9.26000	-13.85800	-14.70500
O	-0.79000	-8.03900	-14.65200
H	-0.27600	-7.40600	-14.14900
H	-0.14000	-8.63900	-15.01700
O	-9.52100	-11.89600	-21.43800
H	-10.35700	-11.55900	-21.11800
H	-9.66000	-12.03100	-22.37600
O	-7.54500	-17.05300	-16.73100
H	-8.19900	-17.36800	-17.35500
H	-7.58700	-17.67500	-16.00500
O	-3.52700	1.93900	-21.89600
H	-4.20100	2.24200	-21.28700
H	-3.14100	2.74200	-22.24500
O	-4.31200	-10.56400	-12.40600
H	-5.22900	-10.34300	-12.56400
H	-3.89600	-10.49000	-13.26500
O	-3.52800	-9.16500	-17.27500
H	-2.78100	-8.65300	-17.58500
H	-3.25900	-9.48700	-16.41500
O	-0.02300	-3.05900	-16.52400
H	-0.47700	-3.90000	-16.46700
H	-0.65400	-2.42300	-16.18600
O	-1.08000	-12.13000	-15.29400
H	-1.81800	-12.64200	-14.96300
H	-0.82700	-12.57000	-16.10500
O	-1.58000	-1.22100	-15.58900
H	-1.28800	-0.66100	-16.30700
H	-1.07000	-0.92800	-14.83300
O	-0.97900	-19.35200	-14.69900
H	-0.67300	-20.21200	-14.98700
H	-0.25400	-18.76000	-14.90000
O	-14.68700	-8.94900	-16.31100
H	-14.88400	-9.54200	-17.03600
H	-15.24200	-9.25400	-15.59400
O	-6.70700	-9.05700	-14.19200
H	-6.65200	-10.00900	-14.26800
H	-7.51800	-8.90100	-13.70800
O	-8.76800	-10.43900	-16.43300
H	-9.58100	-10.13900	-16.02600
H	-8.27200	-10.83300	-15.71500
O	-6.61700	-0.44300	-14.49900
H	-6.65900	-0.21700	-15.42800
H	-5.70100	-0.67700	-14.35100
O	-3.17000	-9.97500	-7.60100
H	-2.81500	-9.74800	-6.74200
H	-2.52300	-9.64300	-8.22400
O	2.56000	-6.85300	-16.89600
H	2.42100	-6.66300	-17.82400
H	2.20300	-6.09100	-16.44000
O	-9.27600	-12.86700	-10.81700

H	-8.63600	-13.00800	-11.51400
H	-10.10800	-13.15400	-11.19300
O	2.58600	-4.78500	-11.88500
H	3.02000	-4.87800	-11.03700
H	3.30000	-4.80900	-12.52300
O	-8.50000	-3.56700	-14.70300
H	-7.70600	-4.06200	-14.90500
H	-9.21100	-4.09500	-15.06600
O	-3.68100	-1.18500	-10.35200
H	-3.62700	-0.61600	-9.58400
H	-4.54900	-1.01500	-10.71700
O	-12.19200	-9.42300	-18.62700
H	-13.12200	-9.48400	-18.41000
H	-12.09600	-9.94500	-19.42400
O	-12.27300	-12.89700	-16.03900
H	-12.01300	-11.98200	-16.14800
H	-11.45200	-13.36500	-15.89000
O	-10.80100	-11.48300	-8.86900
H	-10.12900	-11.69200	-9.51800
H	-11.62800	-11.68100	-9.30900
O	3.44800	-4.74300	-9.35200
H	3.64200	-3.83800	-9.10600
H	3.34800	-5.20200	-8.51800
O	-4.89800	-14.85700	-6.85300
H	-5.54600	-14.15800	-6.94600
H	-4.12800	-14.42300	-6.48500
O	-4.72200	-7.01900	-8.79300
H	-3.79300	-6.86800	-8.96600
H	-5.14300	-6.93100	-9.64800
O	-5.92300	-2.99400	-11.02300
H	-6.06200	-3.36200	-10.15100
H	-6.71200	-2.47900	-11.19500
O	-5.32800	2.10700	-19.68100
H	-5.15300	1.63600	-18.86600
H	-6.01500	2.73400	-19.45300
O	-4.37300	-3.61600	-13.63400
H	-4.70800	-3.65600	-12.73900
H	-4.87200	-4.28100	-14.10700
O	-10.39800	-8.20900	-8.10900
H	-10.53600	-8.94100	-7.50800
H	-9.48800	-8.29800	-8.39100
O	-7.77000	-8.49700	-8.62100
H	-7.06000	-9.13800	-8.66200
H	-7.81300	-8.24600	-7.69800
O	-9.05700	-11.51000	-6.80100
H	-9.59300	-11.96100	-6.14900
H	-9.64500	-11.37600	-7.54400
O	-10.76200	5.25600	-12.90700
H	-10.52200	5.88800	-12.22900
H	-11.60500	4.90500	-12.62000
O	-9.64700	-12.05200	-24.23900
H	-10.52400	-12.17300	-24.60200
H	-9.17100	-12.84300	-24.49100
O	0.22100	-3.59600	-7.46900
H	-0.66700	-3.93400	-7.34600
H	0.79000	-4.32800	-7.23200
O	-18.12700	-7.26400	-14.14400
H	-17.43900	-7.57800	-14.73200
H	-18.28200	-6.36000	-14.41800
O	-13.32300	-12.37500	-9.71600
H	-13.40700	-12.98200	-10.45100
H	-14.14500	-11.88400	-9.71500
O	-6.24900	1.06400	-10.50300
H	-6.32200	1.86900	-9.99100
H	-6.69200	1.26300	-11.32800
O	-6.46000	0.30100	-16.99200
H	-7.24400	0.84300	-16.89900
H	-5.76100	0.81800	-16.59300
O	-3.15100	-13.89000	-14.34900
H	-2.65400	-14.64300	-14.66900
H	-3.92200	-13.85300	-14.91500
O	4.70200	-8.18500	-12.70700
H	4.69100	-7.30300	-13.07800
H	3.83400	-8.53800	-12.90200
O	-2.06800	-5.13200	-16.77500
H	-3.00200	-5.01200	-16.94500
H	-1.89000	-6.03500	-17.03900
O	-6.56700	-11.74800	-14.62200
H	-6.93700	-12.30100	-13.93400
H	-6.07100	-12.35200	-15.17400
O	1.32000	-9.29100	-16.24000
H	1.78600	-8.45800	-16.31400
H	1.77000	-9.75600	-15.53600
O	-8.73800	-1.28800	-7.52600
H	-8.03700	-0.65400	-7.37300
H	-9.20900	-0.94700	-8.28700
O	-0.61300	-2.94600	-19.24200
H	-0.33700	-3.06100	-18.33200
H	-1.48200	-3.34400	-19.28000
O	-0.70900	-16.92300	-12.20100
H	-1.62300	-16.69200	-12.03300
H	-0.28000	-16.83900	-11.35000
O	-0.93800	-8.73400	-9.70100
H	-1.59100	-8.48600	-10.35600
H	-0.56000	-9.54700	-10.03500
O	-3.97700	1.30200	-15.59800
H	-3.79500	0.48500	-15.13500
H	-3.15400	1.51900	-16.03600
O	-3.64300	0.22900	-18.33700
H	-3.11400	-0.28200	-18.95000
H	-3.38000	-0.08700	-17.47200
O	-4.88900	-7.99200	-3.21400
H	-5.56500	-8.09200	-2.54400
H	-4.06700	-8.14800	-2.75100
O	-13.14200	-1.75400	-10.59300
H	-12.55600	-2.50000	-10.46000
H	-13.33900	-1.44400	-9.71000
O	-10.28800	1.28100	-15.57700
H	-10.95700	1.76500	-16.06100
H	-10.42600	1.52800	-14.66300
O	-18.55000	-9.18400	-11.87300
H	-18.51100	-9.71700	-11.07900
H	-19.05600	-9.71000	-12.49200
O	-6.14700	3.31500	-8.97900
H	-5.59500	3.47800	-8.21400
H	-5.80700	3.90600	-9.65100
O	-11.17100	-14.33700	-8.37600
H	-11.10700	-13.47600	-8.78900

H	-11.23400	-14.95000	-9.10900
O	-11.69500	-12.26500	-4.72900
H	-11.06800	-12.64700	-4.11500
H	-12.55300	-12.48400	-4.36400
O	-14.91900	-10.45700	-18.79700
H	-14.62400	-11.36600	-18.85100
H	-15.82000	-10.47900	-19.11900
O	-3.01000	1.26300	-11.99200
H	-3.13500	0.46200	-12.50100
H	-2.35200	1.75800	-12.48100
O	-11.38900	-12.38200	-12.81500
H	-12.11100	-11.76800	-12.95400
H	-10.60000	-11.84600	-12.89300
O	-0.94700	-0.93600	-10.36400
H	-1.89400	-1.05500	-10.43700
H	-0.59900	-1.82400	-10.28200
O	-8.58700	-15.04600	-6.54100
H	-8.06300	-14.80000	-5.77800
H	-8.80600	-14.21400	-6.96000
O	-13.19000	-12.77600	-18.67300
H	-13.51900	-13.65600	-18.85600
H	-12.83600	-12.83100	-17.78600
O	-3.94200	-1.14100	-14.16400
H	-4.09000	-2.06900	-13.98300
H	-3.11700	-1.11900	-14.64800
O	-10.07600	-12.48900	-18.30600
H	-9.73600	-11.96000	-19.02800
H	-9.62900	-12.15100	-17.53000
O	-0.33900	-0.31800	-13.07700
H	0.58600	-0.52300	-13.21500
H	-0.50700	-0.56600	-12.16900
O	-7.38200	-14.10500	-8.85600
H	-7.72200	-14.90400	-8.45300
H	-8.08800	-13.80500	-9.42800
O	-6.73000	-17.02000	-10.40300
H	-5.90700	-17.49400	-10.52500
H	-6.48600	-16.24100	-9.90300
O	-8.08900	-13.99100	-20.10700
H	-7.36800	-13.61300	-20.61000
H	-8.71300	-13.27200	-20.00300
O	-3.94600	-13.28700	-19.17100
H	-3.73000	-12.40400	-19.47200
H	-4.42700	-13.67800	-19.90000
O	-9.20600	-10.32800	-12.59400
H	-9.35200	-10.01400	-11.70100
H	-9.68400	-9.71000	-13.14700
O	-7.34600	-13.33800	-12.54600
H	-7.36000	-14.23400	-12.88200
H	-6.62900	-13.33100	-11.91200
O	-5.49400	-12.90400	-10.50000
H	-5.75200	-11.98200	-10.50200
H	-6.02500	-13.30100	-9.81000
O	-0.54900	0.20100	-17.72100
H	-0.37600	-0.36400	-18.47400
H	-0.05600	1.00100	-17.90300
O	-14.73200	-7.78800	-9.65500
H	-15.27600	-7.01200	-9.52300
H	-13.85400	-7.44100	-9.81300
O	-7.94300	-14.45600	-17.27700
H	-7.82100	-15.38500	-17.07800
H	-8.23300	-14.44300	-18.18900
O	-5.07400	-16.77100	-14.14000
H	-5.05100	-16.70700	-15.09500
H	-4.65700	-17.61100	-13.94600
O	5.30500	-7.03500	-16.10200
H	5.74800	-7.78500	-16.50000
H	4.42700	-7.04400	-16.48500
O	0.43800	-0.48200	-20.62200
H	1.13300	0.13800	-20.40300
H	0.78100	-1.33300	-20.34600
O	-13.94500	-8.66100	-6.76200
H	-14.68700	-8.51200	-6.17500
H	-14.26600	-8.40000	-7.62500
O	2.33700	-3.03200	-19.09300
H	2.86300	-2.69400	-19.81800
H	2.25000	-3.96800	-19.27600
O	-5.97900	3.03500	-14.73300
H	-6.41800	2.54200	-14.04000
H	-5.22800	2.49200	-14.97400
O	-13.49900	-0.77400	-14.73100
H	-12.67300	-1.07400	-15.11100
H	-13.85000	-1.54300	-14.28200
O	-8.53100	-1.42100	-12.66100
H	-9.15300	-2.08400	-12.96100
H	-7.86100	-1.39500	-13.34400
O	-15.29800	-2.85500	-13.77200
H	-16.13500	-3.28300	-13.95400
H	-15.32600	-2.66200	-12.83500
O	-8.45900	-12.94300	-3.06800
H	-8.41000	-12.06700	-3.45000
H	-7.91600	-13.48600	-3.64100
O	2.14700	-5.55000	-6.47400
H	2.19600	-5.71500	-5.53300
H	2.03800	-6.41700	-6.86500
O	-0.56300	-9.56700	-5.15800
H	0.33800	-9.70600	-5.45000
H	-0.48800	-9.40100	-4.21900
O	-6.22900	-12.19000	-6.31300
H	-7.09800	-11.89700	-6.58500
H	-5.99300	-11.60600	-5.59200
O	4.84800	-5.56500	-13.71000
H	5.68300	-5.12600	-13.54600
H	4.98400	-6.04100	-14.52900
O	1.89700	-8.00700	-7.76200
H	2.37300	-7.81400	-8.57000
H	0.97400	-7.95100	-8.00800
O	-11.21500	-6.84500	-18.17900
H	-11.46000	-7.74800	-17.98100
H	-11.04500	-6.84300	-19.12100
O	-3.52500	-15.97200	-8.79700
H	-3.00700	-15.23600	-9.12200
H	-3.87900	-15.66600	-7.96200
O	-11.94500	-11.11200	-20.68000
H	-12.63800	-11.03700	-21.33600
H	-12.19000	-11.87600	-20.15800
O	-3.27400	-19.55200	-13.27700
H	-2.99300	-20.22000	-12.65000
H	-2.48900	-19.35900	-13.78900

O	-5.47000	-0.37600	-21.71400
H	-6.04300	0.24300	-22.16700
H	-4.89700	0.17400	-21.18000
O	0.59700	-11.13000	-9.52900
H	1.54900	-11.03100	-9.50800
H	0.42800	-11.92300	-9.02000
O	-1.38100	-14.04500	-9.34300
H	-1.97600	-13.76400	-10.03900
H	-1.66600	-13.55500	-8.57200
O	-2.25100	-0.09300	-20.58300
H	-1.31700	-0.15800	-20.38000
H	-2.33800	0.74300	-21.04200
O	0.37800	-0.77600	-23.62300
H	0.30000	-1.11000	-22.72900
H	1.19900	-0.28400	-23.62400
O	3.07600	-7.91000	-10.41500
H	3.04400	-8.84800	-10.22900
H	3.68600	-7.83100	-11.14900
O	3.22600	-11.11900	-10.36900
H	2.70900	-11.90300	-10.55600
H	4.11000	-11.44700	-10.20500
O	0.88700	-17.37500	-14.41000
H	0.68900	-17.68300	-13.52500
H	1.49700	-16.64800	-14.28000
O	8.89400	-6.84800	-11.27500
H	9.62400	-7.11000	-10.71400
H	8.28400	-7.58500	-11.23400
O	6.97500	-8.70700	-10.67700
H	6.24900	-8.84200	-11.28500
H	6.56000	-8.40000	-9.87100
O	-5.20200	-4.98800	-2.91200
H	-4.28100	-4.81100	-2.71800
H	-5.23700	-5.93300	-3.06400
O	-6.90100	-11.11500	-24.43700
H	-7.80800	-11.41200	-24.36100
H	-6.62400	-11.41200	-25.30400
O	-15.98300	-9.79500	-13.98700
H	-16.30300	-10.64800	-14.28000
H	-16.53000	-9.58000	-13.23100
O	-12.20500	-7.07800	-10.27000
H	-11.98700	-6.14700	-10.25100
H	-11.79300	-7.43700	-9.48500
O	-5.28500	-16.57100	-18.28300
H	-6.09500	-16.91700	-17.90700
H	-4.59700	-16.88100	-17.69400
O	-3.35600	-17.49400	-16.66000
H	-2.80300	-17.07400	-16.00100
H	-3.11900	-18.42000	-16.61800
O	-7.33300	-11.50500	-0.88000
H	-7.43300	-10.55600	-0.94900
H	-7.55100	-11.83300	-1.75200
O	-8.13100	-14.25600	-24.29600
H	-7.26900	-13.93100	-24.03600
H	-7.94600	-15.04300	-24.80800
O	-16.11500	-6.17400	-12.01800
H	-16.80800	-6.58900	-12.53100
H	-15.36700	-6.13200	-12.61400
O	0.02200	-15.38400	-17.15200
H	-0.55800	-14.72500	-17.53400
H	-0.24500	-16.20600	-17.56300
O	-1.52800	-15.98300	-14.95000
H	-1.02800	-15.87100	-15.75900
H	-0.88400	-16.28700	-14.31000
O	-7.47000	-8.07500	-2.06300
H	-7.95300	-7.27500	-1.85700
H	-7.38300	-8.06400	-3.01700
O	-14.20100	-6.24200	-14.14100
H	-14.22400	-6.14400	-15.09300
H	-13.89100	-5.39600	-13.81900
O	-11.54200	-4.18500	-10.02600
H	-10.58800	-4.21600	-10.09300
H	-11.74100	-4.64600	-9.21100
O	-10.95200	-5.61500	-7.45900
H	-10.13300	-5.12000	-7.43600
H	-10.67900	-6.53100	-7.48800
O	-1.19600	-13.22400	-18.09700
H	-2.08300	-12.93900	-18.31500
H	-0.78000	-13.38400	-18.94400
O	2.15600	-13.19900	-14.38100
H	2.93200	-12.70900	-14.10900
H	1.43100	-12.77300	-13.92400
O	4.59300	-12.48600	-13.32400
H	5.18800	-12.72700	-14.03400
H	4.66600	-13.20500	-12.69600
O	-13.18700	1.35700	-13.00900
H	-13.32200	0.59700	-13.57600
H	-13.60400	2.08100	-13.47700
O	-2.05100	-3.45900	-24.00200
H	-1.34800	-3.25400	-23.38600
H	-2.77500	-2.89100	-23.73700
O	-1.22200	-7.70800	-17.88200
H	-1.27800	-7.25100	-18.72100
H	-0.66900	-8.46900	-18.05900
O	2.58900	-11.91900	-17.05700
H	2.33300	-12.32000	-16.22600
H	1.96400	-11.20500	-17.18200
O	6.90000	-9.18000	-14.54900
H	6.34000	-9.11000	-13.77600
H	6.29500	-9.35500	-15.26900
O	-10.03000	2.25400	-12.79800
H	-10.14700	3.19900	-12.90000
H	-10.85100	1.95100	-12.40900
O	-0.14300	-3.02100	-21.95200
H	0.69200	-3.45300	-22.13400
H	-0.30400	-3.19300	-21.02400
O	-0.48800	-6.51800	-20.58600
H	-0.54300	-6.68300	-21.52700
H	-1.11300	-5.81000	-20.43000
O	-15.69900	-11.28100	-6.76000
H	-15.05300	-11.79100	-6.27100
H	-15.90700	-10.54400	-6.18600
O	-7.17800	-17.01700	-21.00800
H	-7.19100	-16.07300	-20.85100
H	-6.28600	-17.20000	-21.30200
O	-9.00500	2.90700	-9.58200
H	-8.10200	2.93900	-9.26500
H	-9.23100	1.97700	-9.56700
O	-7.24900	1.66000	-12.92100

H	-8.20000	1.66000	-13.03500
H	-6.94600	0.91300	-13.43700
O	-3.36300	-20.42300	-16.75300
H	-4.13000	-20.97700	-16.60800
H	-2.95200	-20.35000	-15.89200
O	-11.35900	-14.13400	-2.66300
H	-10.49600	-14.49000	-2.45200
H	-11.93300	-14.47300	-1.97500
O	8.48600	7.49600	-11.85600
H	9.12400	7.37700	-12.56000
H	8.36800	8.44400	-11.79800
O	5.29500	7.44900	-11.92000
H	4.95700	7.33300	-12.80800
H	6.16400	7.04800	-11.94000
O	1.15300	9.30200	-15.93100
H	0.59000	9.21100	-16.69900
H	1.03900	8.48200	-15.45000
O	5.77700	8.60200	-5.96200
H	5.76600	8.20800	-6.83400
H	5.93900	7.86900	-5.36800
O	-1.30000	10.79700	-13.09200
H	-0.50800	10.45700	-13.50800
H	-1.06000	10.90700	-12.17200
O	6.73300	12.96900	-20.61900
H	6.08200	13.55500	-21.00600
H	7.52800	13.50000	-20.56000
O	0.65900	10.05800	-20.22000
H	1.43200	9.51100	-20.35900
H	1.01200	10.92300	-20.00900
O	2.49700	11.38600	-7.01400
H	2.20600	12.29100	-7.12900
H	3.13300	11.42800	-6.29900
O	3.42300	8.76400	-10.42400
H	4.01900	8.53900	-11.13800
H	3.48100	8.02200	-9.82100
O	2.99600	6.21800	-22.58900
H	3.24800	5.69600	-23.35000
H	2.05800	6.05400	-22.48600
O	8.04000	5.22100	-14.78100
H	8.82600	4.80300	-15.13200
H	8.30400	6.12600	-14.61300
O	8.21100	10.65900	-20.08200
H	7.57600	10.73200	-19.37000
H	7.88100	11.24800	-20.76000
O	13.44100	5.15900	-13.87800
H	14.17300	4.58900	-13.64400
H	12.68500	4.77100	-13.43700
O	2.71500	8.12000	-20.38600
H	2.59900	7.44500	-19.71800
H	2.82500	7.63400	-21.20300
O	5.05800	7.58100	-16.68100
H	4.69400	7.05500	-15.97000
H	5.91500	7.19100	-16.85500
O	10.96300	-1.12900	-9.41800
H	10.63200	-0.26000	-9.64200
H	10.36800	-1.44500	-8.73900
O	4.69900	2.61000	-21.68500
H	4.86600	3.47800	-21.31800
H	4.50900	2.77300	-22.60900
O	7.45600	11.07200	-10.01900
H	7.15700	11.97500	-10.12700
H	7.11200	10.80500	-9.16700
O	0.90800	7.52400	-13.98500
H	-0.01700	7.39100	-13.77700
H	1.15600	8.30300	-13.48800
O	10.85600	3.59000	-12.33900
H	9.94800	3.52200	-12.63300
H	10.80500	4.06800	-11.51100
O	4.67800	5.06600	-20.70100
H	4.55800	5.51200	-19.86200
H	3.98300	5.41500	-21.25900
O	-1.73100	7.16700	-13.15100
H	-1.60200	7.42000	-12.23700
H	-2.41200	7.76000	-13.47000
O	2.43900	17.83700	-17.05200
H	2.36100	18.78700	-16.97300
H	1.83500	17.49100	-16.39400
O	4.93000	5.00900	-4.39600
H	4.15400	5.56900	-4.40500
H	5.56900	5.49500	-3.87400
O	12.72200	1.79200	-10.56100
H	12.08000	2.08500	-11.20700
H	12.21800	1.67700	-9.75600
O	10.37600	5.53800	-19.87300
H	11.28800	5.77100	-20.04800
H	9.86700	6.12200	-20.43500
O	3.18500	7.81800	-4.83400
H	3.92600	8.35600	-5.11000
H	2.47900	8.44500	-4.67700
O	8.66000	9.96100	-6.23600
H	9.06900	9.51900	-6.98100
H	7.93800	9.38600	-5.98300
O	5.01400	4.89800	-8.86300
H	5.86000	5.32200	-8.72000
H	4.95300	4.24100	-8.16900
O	8.98300	10.52100	-12.48100
H	8.35400	10.68600	-13.18400
H	8.59600	10.93500	-11.71000
O	12.86200	11.45600	-15.12700
H	12.17700	11.83600	-15.67700
H	12.94700	12.06600	-14.39400
O	8.16300	2.55900	-7.20500
H	7.92000	3.31300	-7.74200
H	8.43900	2.93700	-6.37000
O	10.11700	9.10200	-8.52700
H	10.32900	9.74600	-9.20400
H	10.07000	8.26900	-8.99500
O	7.72000	1.71400	-11.62400
H	6.85800	2.01200	-11.33200
H	7.74600	1.93100	-12.55600
O	4.53600	10.25800	-15.21600
H	5.00000	10.53700	-16.00600
H	4.27200	9.35700	-15.39900
O	1.42900	9.80600	-12.27800
H	1.99900	10.23400	-12.91700
H	2.01900	9.52400	-11.58000
O	3.47300	6.38100	-18.40700
H	4.17500	6.83300	-17.94000

H	3.14100	5.73900	-17.77900
O	-2.78400	4.63900	-13.10600
H	-3.57500	4.89500	-13.58200
H	-2.29300	5.45400	-13.00000
O	11.23200	11.98000	-10.35500
H	12.05400	11.57800	-10.63700
H	11.22800	11.87600	-9.40300
O	-0.83300	5.00400	-16.14700
H	-1.63000	5.11500	-15.62900
H	-1.06000	5.34200	-17.01300
O	4.99800	11.52800	-8.19900
H	5.06200	11.99000	-7.36300
H	4.28900	10.89800	-8.07100
O	-4.50100	4.89200	-10.67400
H	-4.25000	5.77700	-10.40800
H	-3.96500	4.71300	-11.44700
O	19.32300	8.32300	-14.00700
H	18.56900	7.75400	-14.16200
H	19.45700	8.29000	-13.05900
O	4.71200	13.19500	-18.33100
H	4.57700	14.04000	-17.90300
H	5.25300	13.39700	-19.09400
O	-1.83500	10.71200	-17.75800
H	-1.32200	11.50700	-17.60800
H	-1.18200	10.01500	-17.82500
O	-0.02500	13.82900	-15.67600
H	0.15100	14.75100	-15.86400
H	0.77000	13.51500	-15.24700
O	6.33900	13.81700	-4.20100
H	5.89600	13.03100	-4.51900
H	7.00100	14.00500	-4.86700
O	1.09800	15.34700	-9.67500
H	1.64900	15.21800	-10.44700
H	0.25900	15.64600	-10.02500
O	8.43700	14.09700	-6.17300
H	8.41900	14.91800	-6.66500
H	9.35900	13.97300	-5.94800
O	4.89500	11.61200	-5.33100
H	5.42700	10.85100	-5.56100
H	4.36600	11.31900	-4.58800
O	8.06200	-3.28200	-9.80100
H	7.71800	-2.39200	-9.87700
H	8.43000	-3.47200	-10.66400
O	-3.87000	9.04100	-16.98200
H	-3.03200	9.45100	-17.19900
H	-3.69200	8.10100	-17.01200
O	8.02700	6.70900	-21.29400
H	7.51100	7.47900	-21.05500
H	7.75400	6.03500	-20.67100
O	5.61100	1.55400	-16.23500
H	4.75600	1.16400	-16.41500
H	6.02400	1.63300	-17.09500
O	7.14100	-0.91200	-11.83600
H	6.63800	-0.94900	-12.64900
H	7.43400	-0.00200	-11.77700
O	3.38600	-0.82800	-12.83000
H	4.25100	-1.02400	-13.18900
H	3.45300	-1.05700	-11.90300
O	13.00000	9.46200	-19.35900
H	12.22300	9.12300	-19.80300
H	13.67900	9.46700	-20.03300
O	7.81500	4.58100	-19.46800
H	7.51000	4.89600	-18.61700
H	8.76800	4.66000	-19.42500
O	6.26000	10.74000	-17.81900
H	5.73200	10.00600	-18.13100
H	5.75900	11.51800	-18.06500
O	11.23900	8.93500	-12.70200
H	10.58200	9.62500	-12.61400
H	12.04300	9.32500	-12.35900
O	0.99200	1.40200	-15.45800
H	1.56600	1.18000	-16.19100
H	1.57900	1.47100	-14.70500
O	10.05000	7.29000	-14.65200
H	10.56300	7.92300	-14.14900
H	10.70000	6.69000	-15.01700
O	1.31900	3.43300	-21.43800
H	0.48200	3.77000	-21.11800
H	1.17900	3.29800	-22.37600
O	3.29400	-1.72300	-16.73100
H	2.64100	-2.03900	-17.35500
H	3.25200	-2.34600	-16.00500
O	4.40500	8.55800	-24.81500
H	3.49800	8.32700	-24.61600
H	4.90200	7.76200	-24.62800
O	7.31200	17.26800	-21.89600
H	6.63800	17.57100	-21.28700
H	7.69900	18.07100	-22.24500
O	6.52800	4.76500	-12.40600
H	5.61000	4.98600	-12.56400
H	6.94400	4.83900	-13.26500
O	7.31100	6.16400	-17.27500
H	8.05900	6.67600	-17.58500
H	7.58000	5.84200	-16.41500
O	5.58400	8.66000	-20.29300
H	4.67500	8.37600	-20.19600
H	5.61400	9.08000	-21.15200
O	10.81600	12.27000	-16.52400
H	10.36200	11.42900	-16.46700
H	10.18500	12.90700	-16.18600
O	9.75900	3.20000	-15.29400
H	9.02100	2.68700	-14.96300
H	10.01200	2.76000	-16.10500
O	9.25900	14.10800	-15.58900
H	9.55200	14.66900	-16.30700
H	9.76900	14.40100	-14.83300
O	9.86000	-4.02300	-14.69900
H	10.16600	-4.88300	-14.98700
H	10.58600	-3.43100	-14.90000
O	-3.84700	6.38000	-16.31100
H	-4.04500	5.78700	-17.03600
H	-4.40300	6.07600	-15.59400
O	4.13200	6.27300	-14.19200
H	4.18700	5.32000	-14.26800
H	3.32200	6.42900	-13.70800
O	5.28200	10.20100	-22.95400
H	4.84400	9.48400	-23.41300
H	6.02300	10.42800	-23.51500

O	2.07200	4.89000	-16.43300
H	1.25900	5.19000	-16.02600
H	2.56800	4.49600	-15.71500
O	4.22300	14.88600	-14.49900
H	4.18000	15.11200	-15.42800
H	5.13900	14.65200	-14.35100
O	7.66900	5.35400	-7.60100
H	8.02400	5.58200	-6.74200
H	8.31600	5.68600	-8.22400
O	13.39900	8.47600	-16.89600
H	13.26100	8.66600	-17.82400
H	13.04300	9.23800	-16.44000
O	1.56300	2.46200	-10.81700
H	2.20300	2.32100	-11.51400
H	0.73100	2.17600	-11.19300
O	13.42600	10.54400	-11.88500
H	13.86000	10.45200	-11.03700
H	14.13900	10.52000	-12.52300
O	2.33900	11.76200	-14.70300
H	3.13300	11.26700	-14.90500
H	1.62800	11.23400	-15.06600
O	7.15900	14.14400	-10.35200
H	7.21200	14.71300	-9.58400
H	6.29000	14.31400	-10.71700
O	-1.35300	5.90700	-18.62700
H	-2.28300	5.84500	-18.41000
H	-1.25700	5.38400	-19.42400
O	-1.43400	2.43200	-16.03900
H	-1.17300	3.34700	-16.14800
H	-0.61200	1.96500	-15.89000
O	14.28700	10.58600	-9.35200
H	14.48100	11.49100	-9.10600
H	14.18700	10.12700	-8.51800
O	9.34900	10.73700	-3.77800
H	9.19700	10.50800	-4.69500
H	9.33900	11.69400	-3.76500
O	6.11700	8.31000	-8.79300
H	7.04700	8.46100	-8.96600
H	5.69700	8.39900	-9.64800
O	4.91600	12.33500	-11.02300
H	4.77700	11.96700	-10.15100
H	4.12800	12.85000	-11.19500
O	5.51100	17.43600	-19.68100
H	5.68600	16.96500	-18.86600
H	4.82400	18.06300	-19.45300
O	6.46700	11.71300	-13.63400
H	6.13200	11.67400	-12.73900
H	5.96700	11.04800	-14.10700
O	0.44100	7.12000	-8.10900
H	0.30300	6.38800	-7.50800
H	1.35200	7.03100	-8.39100
O	9.40300	9.05900	-23.36100
H	8.78600	8.33400	-23.45700
H	8.93400	9.82000	-23.70400
O	3.06900	6.83200	-8.62100
H	3.77900	6.19200	-8.66200
H	3.02600	7.08300	-7.69800
O	11.05800	13.76900	-5.70000
H	10.99900	13.08500	-6.36700
H	11.46000	14.51100	-6.15200
O	0.07700	20.58500	-12.90700
H	0.31700	21.21700	-12.22900
H	-0.76600	20.23400	-12.62000
O	1.19300	3.27800	-24.23900
H	0.31600	3.15600	-24.60200
H	1.66800	2.48600	-24.49100
O	11.06000	11.73300	-7.46900
H	10.17300	11.39500	-7.34600
H	11.63000	11.00100	-7.23200
O	-7.28700	8.06500	-14.14400
H	-6.60000	7.75100	-14.73200
H	-7.44200	8.96900	-14.41800
O	4.59000	16.39300	-10.50300
H	4.51700	17.19900	-9.99100
H	4.14800	16.59200	-11.32800
O	4.37900	15.63000	-16.99200
H	3.59600	16.17200	-16.89900
H	5.07800	16.14800	-16.59300
O	7.68900	1.44000	-14.34900
H	8.18600	0.68700	-14.66900
H	6.91700	1.47600	-14.91500
O	15.54200	7.14400	-12.70700
H	15.53100	8.02600	-13.07800
H	14.67300	6.79100	-12.90200
O	7.05000	16.29200	-8.34700
H	7.76300	16.92600	-8.41900
H	6.55900	16.38800	-9.16300
O	8.77200	10.19700	-16.77500
H	7.83700	10.31700	-16.94500
H	8.94900	9.29400	-17.03900
O	4.27200	3.58100	-14.62200
H	3.90200	3.02900	-13.93400
H	4.76800	2.97700	-15.17400
O	12.15900	6.03900	-16.24000
H	12.62500	6.87100	-16.31400
H	12.61000	5.57300	-15.53600
O	2.10100	14.04100	-7.52600
H	2.80200	14.67500	-7.37300
H	1.63100	14.38300	-8.28700
O	10.22700	12.38300	-19.24200
H	10.50200	12.26900	-18.33200
H	9.35700	11.98500	-19.28000
O	10.13100	-1.59300	-12.20100
H	9.21700	-1.36300	-12.03300
H	10.56000	-1.51000	-11.35000
O	9.90100	6.59600	-9.70100
H	9.24800	6.84300	-10.35600
H	10.27900	5.78200	-10.03500
O	6.86200	16.63200	-15.59800
H	7.04400	15.81400	-15.13500
H	7.68500	16.84800	-16.03600
O	7.19600	15.55800	-18.33700
H	7.72500	15.04800	-18.95000
H	7.45900	15.24300	-17.47200
O	-2.30200	13.57500	-10.59300
H	-1.71700	12.82900	-10.46000
H	-2.50000	13.88500	-9.71000
O	0.55200	16.61000	-15.57700

H	-0.11700	17.09500	-16.06100
H	0.41300	16.85700	-14.66300
O	-7.71000	6.14500	-11.87300
H	-7.67200	5.61200	-11.07900
H	-8.21600	5.61900	-12.49200
O	4.69200	18.64400	-8.97900
H	5.24400	18.80700	-8.21400
H	5.03300	19.23500	-9.65100
O	9.54300	13.47000	-3.12400
H	10.08700	14.08300	-3.61800
H	8.96200	14.02800	-2.60700
O	-4.08000	4.87200	-18.79700
H	-3.78400	3.96300	-18.85100
H	-4.98100	4.85100	-19.11900
O	7.82900	16.59300	-11.99200
H	7.70400	15.79100	-12.50100
H	8.48700	17.08700	-12.48100
O	-0.55000	2.94700	-12.81500
H	-1.27100	3.56100	-12.95400
H	0.23900	3.48300	-12.89300
O	9.89300	14.39300	-10.36400
H	8.94600	14.27400	-10.43700
H	10.24100	13.50600	-10.28200
O	-2.35100	2.55300	-18.67300
H	-2.67900	1.67300	-18.85600
H	-1.99700	2.49800	-17.78600
O	6.89700	14.18900	-14.16400
H	6.75000	13.26000	-13.98300
H	7.72300	14.21100	-14.64800
O	0.76300	2.84000	-18.30600
H	1.10300	3.36900	-19.02800
H	1.21100	3.17900	-17.53000
O	10.50100	15.01200	-13.07700
H	11.42500	14.80600	-13.21500
H	10.33200	14.76300	-12.16900
O	3.45800	1.22400	-8.85600
H	3.11700	0.42500	-8.45300
H	2.75200	1.52400	-9.42800
O	4.10900	-1.69100	-10.40300
H	4.93200	-2.16500	-10.52500
H	4.35300	-0.91200	-9.90300
O	2.75000	1.33800	-20.10700
H	3.47100	1.71600	-20.61000
H	2.12700	2.05700	-20.00300
O	6.89300	2.04200	-19.17100
H	7.11000	2.92500	-19.47200
H	6.41300	1.65100	-19.90000
O	1.63300	5.00100	-12.59400
H	1.48800	5.31500	-11.70100
H	1.15600	5.62000	-13.14700
O	3.49300	1.99100	-12.54600
H	3.47900	1.09500	-12.88200
H	4.21000	1.99800	-11.91200
O	5.34600	2.42500	-10.50000
H	5.08700	3.34700	-10.50200
H	4.81400	2.02800	-9.81000
O	10.29000	15.53000	-17.72100
H	10.46400	14.96500	-18.47400
H	10.78300	16.33000	-17.90300
O	-3.89200	7.54200	-9.65500
H	-4.43700	8.31800	-9.52300
H	-3.01400	7.88900	-9.81300
O	2.89700	0.87300	-17.27700
H	3.01800	-0.05600	-17.07800
H	2.60700	0.88700	-18.18900
O	5.76600	-1.44200	-14.14000
H	5.78800	-1.37800	-15.09500
H	6.18300	-2.28100	-13.94600
O	16.14400	8.29400	-16.10200
H	16.58700	7.54400	-16.50000
H	15.26700	8.28500	-16.48500
O	11.27800	14.84700	-20.62200
H	11.97300	15.46700	-20.40300
H	11.62000	13.99600	-20.34600
O	13.17700	12.29700	-19.09300
H	13.70200	12.63500	-19.81800
H	13.08900	11.36100	-19.27600
O	4.83900	16.21000	-6.96400
H	5.14800	15.96500	-6.09200
H	5.63600	16.41500	-7.45200
O	4.86100	18.36400	-14.73300
H	4.42100	17.87200	-14.04000
H	5.61200	17.82100	-14.97400
O	-2.66000	14.55500	-14.73100
H	-1.83400	14.25500	-15.11100
H	-3.01000	13.78600	-14.28200
O	2.30800	13.90800	-12.66100
H	1.68600	13.24500	-12.96100
H	2.97900	13.93400	-13.34400
O	-4.45900	12.47400	-13.77200
H	-5.29600	12.04600	-13.95400
H	-4.48700	12.66700	-12.83500
O	12.86600	12.93000	-3.78800
H	12.07600	12.91200	-4.32700
H	12.63000	13.46900	-3.03300
O	12.98600	9.77900	-6.47400
H	13.03600	9.61400	-5.53300
H	12.87700	8.91200	-6.86500
O	1.87000	12.45800	-19.18500
H	2.01800	13.14200	-19.83900
H	2.67600	12.43900	-18.67000
O	10.27600	5.76200	-5.15800
H	11.17700	5.62300	-5.45000
H	10.35100	5.92800	-4.21900
O	14.48800	12.19900	-6.23000
H	14.19600	12.56200	-5.39400
H	13.87500	11.48600	-6.40900
O	15.68700	9.76400	-13.71000
H	16.52200	10.20300	-13.54600
H	15.82300	9.28800	-14.52900
O	12.73700	7.32200	-7.76200
H	13.21200	7.51500	-8.57000
H	11.81300	7.37800	-8.00800
O	-0.37500	8.48500	-18.17900
H	-0.62100	7.58100	-17.98100
H	-0.20600	8.48600	-19.12100
O	-1.10600	4.21700	-20.68000
H	-1.79900	4.29200	-21.33600

H	-1.35000	3.45400	-20.15800
O	7.56600	-4.22300	-13.27700
H	7.84600	-4.89100	-12.65000
H	8.35100	-4.03000	-13.78900
O	5.36900	14.95300	-21.71400
H	4.79700	15.57200	-22.16700
H	5.94200	15.50300	-21.18000
O	11.43700	4.19900	-9.52900
H	12.38800	4.29900	-9.50800
H	11.26700	3.40700	-9.02000
O	9.45800	1.28400	-9.34300
H	8.86300	1.56500	-10.03900
H	9.17300	1.77400	-8.57200
O	8.58900	15.23700	-20.58300
H	9.52200	15.17100	-20.38000
H	8.50100	16.07200	-21.04200
O	11.21800	14.55300	-23.62300
H	11.13900	14.21900	-22.72900
H	12.03900	15.04500	-23.62400
O	13.91500	7.41900	-10.41500
H	13.88300	6.48100	-10.22900
H	14.52600	7.49800	-11.14900
O	5.75400	6.28900	-24.37200
H	6.70000	6.16000	-24.30400
H	5.37500	5.55000	-23.89500
O	14.06500	4.21100	-10.36900
H	13.54900	3.42600	-10.55600
H	14.94900	3.88200	-10.20500
O	11.72600	-2.04500	-14.41000
H	11.52800	-2.35400	-13.52500
H	12.33600	-1.31900	-14.28000
O	19.73300	8.48100	-11.27500
H	20.46300	8.22000	-10.71400
H	19.12400	7.74400	-11.23400
O	17.81500	6.62200	-10.67700
H	17.08800	6.48700	-11.28500
H	17.39900	6.92900	-9.87100
O	3.93800	4.21400	-24.43700
H	3.03100	3.91700	-24.36100
H	4.21600	3.91700	-25.30400
O	-5.14400	5.53500	-13.98700
H	-5.46400	4.68100	-14.28000
H	-5.69100	5.75000	-13.23100
O	-1.36600	8.25100	-10.27000
H	-1.14700	9.18300	-10.25100
H	-0.95400	7.89200	-9.48500
O	5.55400	-1.24200	-18.28300
H	4.74400	-1.58800	-17.90700
H	6.24300	-1.55200	-17.69400
O	7.48300	-2.16400	-16.66000
H	8.03600	-1.74500	-16.00100
H	7.72000	-3.09100	-16.61800
O	3.50700	3.82400	-0.88000
H	3.40600	4.77400	-0.94900
H	3.28900	3.49600	-1.75200
O	2.70800	1.07300	-24.29600
H	3.57000	1.39800	-24.03600
H	2.89300	0.28600	-24.80800
O	-5.27500	9.15500	-12.01800
H	-5.96900	8.74000	-12.53100
H	-4.52800	9.19700	-12.61400
O	10.86100	-0.05400	-17.15200
H	10.28100	0.60400	-17.53400
H	10.59500	-0.87700	-17.56300
O	9.31200	-0.65400	-14.95000
H	9.81200	-0.54200	-15.75900
H	9.95500	-0.95800	-14.31000
O	-3.36200	9.08700	-14.14100
H	-3.38500	9.18600	-15.09300
H	-3.05200	9.93300	-13.81900
O	-0.70300	11.14400	-10.02600
H	0.25100	11.11300	-10.09300
H	-0.90200	10.68300	-9.21100
O	-0.11300	9.71500	-7.45900
H	0.70600	10.20900	-7.43600
H	0.16100	8.79800	-7.48800
O	9.64400	2.10600	-18.09700
H	8.75600	2.39100	-18.31500
H	10.05900	1.94500	-18.94400
O	12.99500	2.13000	-14.38100
H	13.77100	2.62000	-14.10900
H	12.27000	2.55600	-13.92400
O	15.43200	2.84300	-13.32400
H	16.02700	2.60300	-14.03400
H	15.50500	2.12400	-12.69600
O	-2.34800	16.68600	-13.00900
H	-2.48200	15.92600	-13.57600
H	-2.76500	17.41000	-13.47700
O	8.78800	11.87000	-24.00200
H	9.49200	12.07500	-23.38600
H	8.06400	12.43800	-23.73700
O	9.61800	7.62100	-17.88200
H	9.56100	8.07800	-18.72100
H	10.17100	6.86000	-18.05900
O	13.42800	3.41000	-17.05700
H	13.17200	3.01000	-16.22600
H	12.80400	4.12400	-17.18200
O	17.74000	6.15000	-14.54900
H	17.18000	6.21900	-13.77600
H	17.13400	5.97400	-15.26900
O	0.80900	17.58300	-12.79800
H	0.69200	18.52800	-12.90000
H	-0.01200	17.28100	-12.40900
O	10.69700	12.30800	-21.95200
H	11.53100	11.87600	-22.13400
H	10.53500	12.13700	-21.02400
O	10.35100	8.81200	-20.58600
H	10.29600	8.64600	-21.52700
H	9.72600	9.51900	-20.43000
O	3.66100	-1.68800	-21.00800
H	3.64900	-0.74300	-20.85100
H	4.55300	-1.87100	-21.30200
O	1.83500	18.23600	-9.58200
H	2.73700	18.26800	-9.26500
H	1.60800	17.30600	-9.56700
O	3.59000	16.98900	-12.92100
H	2.63900	16.98900	-13.03500
H	3.89300	16.24200	-13.43700

O	7.47600	-5.09300	-16.75300
H	6.70900	-5.64800	-16.60800
H	7.88700	-5.02100	-15.89200
O	19.32500	22.82500	-11.85600
H	19.96300	22.70600	-12.56000
H	19.20700	23.77300	-11.79800
O	16.13400	22.77800	-11.92000
H	15.79600	22.66200	-12.80800
H	17.00300	22.37700	-11.94000
O	11.99300	24.63200	-15.93100
H	11.42900	24.54100	-16.69900
H	11.87800	23.81200	-15.45000
O	16.61600	23.93100	-5.96200
H	16.60500	23.53700	-6.83400
H	16.77800	23.19800	-5.36800
O	9.53900	26.12700	-13.09200
H	10.33100	25.78600	-13.50800
H	9.77900	26.23600	-12.17200
O	17.57300	28.29900	-20.61900
H	16.92200	28.88400	-21.00600
H	18.36700	28.82900	-20.56000
O	11.49800	25.38800	-20.22000
H	12.27100	24.84000	-20.35900
H	11.85100	26.25200	-20.00900
O	13.33700	26.71500	-7.01400
H	13.04600	27.62000	-7.12900
H	13.97300	26.75700	-6.29900
O	14.26200	24.09300	-10.42400
H	14.85900	23.86800	-11.13800
H	14.32000	23.35100	-9.82100
O	13.83500	21.54700	-22.58900
H	14.08800	21.02500	-23.35000
H	12.89700	21.38300	-22.48600
O	18.87900	20.55000	-14.78100
H	19.66600	20.13200	-15.13200
H	19.14300	21.45500	-14.61300
O	19.05000	25.98800	-20.08200
H	18.41500	26.06100	-19.37000
H	18.72000	26.57700	-20.76000
O	24.28000	20.48800	-13.87800
H	25.01300	19.91800	-13.64400
H	23.52400	20.10000	-13.43700
O	13.55500	23.45000	-20.38600
H	13.43800	22.77400	-19.71800
H	13.66500	22.96300	-21.20300
O	15.89800	22.91100	-16.68100
H	15.53400	22.38400	-15.97000
H	16.75400	22.52100	-16.85500
O	21.80300	14.20000	-9.41800
H	21.47100	15.07000	-9.64200
H	21.20700	13.88400	-8.73900
O	15.53800	17.94000	-21.68500
H	15.70600	18.80800	-21.31800
H	15.34800	18.10200	-22.60900
O	18.29600	26.40100	-10.01900
H	17.99600	27.30400	-10.12700
H	17.95100	26.13500	-9.16700
O	11.74700	22.85300	-13.98500
H	10.82200	22.72000	-13.77700
H	11.99600	23.63200	-13.48800
O	21.69600	18.92000	-12.33900
H	20.78700	18.85100	-12.63300
H	21.64400	19.39700	-11.51100
O	15.51700	20.39500	-20.70100
H	15.39700	20.84100	-19.86200
H	14.82200	20.74400	-21.25900
O	9.10800	22.49600	-13.15100
H	9.23700	22.75000	-12.23700
H	8.42800	23.08900	-13.47000
O	13.27800	33.16600	-17.05200
H	13.20000	34.11700	-16.97300
H	12.67400	32.82000	-16.39400
O	15.77000	20.33900	-4.39600
H	14.99300	20.89800	-4.40500
H	16.40900	20.82400	-3.87400
O	23.56200	17.12100	-10.56100
H	22.91900	17.41500	-11.20700
H	23.05700	17.00700	-9.75600
O	21.21600	20.86800	-19.87300
H	22.12800	21.10000	-20.04800
H	20.70700	21.45100	-20.43500
O	14.02400	23.14700	-4.83400
H	14.76600	23.68600	-5.11000
H	13.31800	23.77400	-4.67700
O	19.50000	25.29000	-6.23600
H	19.90800	24.84800	-6.98100
H	18.77800	24.71500	-5.98300
O	15.85300	20.22700	-8.86300
H	16.69900	20.65100	-8.72000
H	15.79300	19.57100	-8.16900
O	19.82300	25.85000	-12.48100
H	19.19400	26.01500	-13.18400
H	19.43500	26.26400	-11.71000
O	23.70200	26.78600	-15.12700
H	23.01700	27.16500	-15.67700
H	23.78600	27.39500	-14.39400
O	19.00300	17.88800	-7.20500
H	18.75900	18.64200	-7.74200
H	19.27900	18.26600	-6.37000
O	20.95700	24.43200	-8.52700
H	21.16800	25.07500	-9.20400
H	20.91000	23.59800	-8.99500
O	18.55900	17.04300	-11.62400
H	17.69800	17.34100	-11.33200
H	18.58600	17.26000	-12.55600
O	15.37500	25.58800	-15.21600
H	15.83900	25.86600	-16.00600
H	15.11100	24.68600	-15.39900
O	12.26800	25.13500	-12.27800
H	12.83800	25.56300	-12.91700
H	12.85900	24.85300	-11.58000
O	14.31200	21.71000	-18.40700
H	15.01500	22.16200	-17.94000
H	13.98000	21.06800	-17.77900
O	12.76000	20.30100	-4.03200
H	12.13000	20.98300	-3.79900
H	12.65400	20.18900	-4.97700
O	8.05500	19.96800	-13.10600

H	7.26500	20.22400	-13.58200
H	8.54600	20.78300	-13.00000
O	22.07100	27.30900	-10.35500
H	22.89300	26.90700	-10.63700
H	22.06800	27.20600	-9.40300
O	10.00600	20.33300	-16.14700
H	9.20900	20.44500	-15.62900
H	9.77900	20.67100	-17.01300
O	15.83700	26.85700	-8.19900
H	15.90100	27.31900	-7.36300
H	15.12800	26.22700	-8.07100
O	6.33900	20.22100	-10.67400
H	6.58900	21.10600	-10.40800
H	6.87500	20.04200	-11.44700
O	30.16200	23.65300	-14.00700
H	29.40800	23.08300	-14.16200
H	30.29700	23.61900	-13.05900
O	15.55200	28.52400	-18.33100
H	15.41600	29.36900	-17.90300
H	16.09300	28.72600	-19.09400
O	12.03800	14.60300	-1.67000
H	11.64500	14.05000	-0.99500
H	12.85500	14.91400	-1.27900
O	9.00500	26.04200	-17.75800
H	9.51700	26.83600	-17.60800
H	9.65700	25.34400	-17.82500
O	10.81400	29.15800	-15.67600
H	10.99100	30.08000	-15.86400
H	11.61000	28.84400	-15.24700
O	14.95500	16.01900	-4.07800
H	15.69600	16.62000	-4.15700
H	15.18700	15.27700	-4.63600
O	10.09900	21.20500	-5.80000
H	9.98500	20.30300	-5.49900
H	9.24400	21.45100	-6.15100
O	17.17900	29.14600	-4.20100
H	16.73500	28.36000	-4.51900
H	17.84000	29.33400	-4.86700
O	11.93700	30.67600	-9.67500
H	12.48800	30.54800	-10.44700
H	11.09800	30.97500	-10.02500
O	19.27700	29.42700	-6.17300
H	19.25900	30.24800	-6.66500
H	20.19900	29.30200	-5.94800
O	15.73400	26.94100	-5.33100
H	16.26700	26.18000	-5.56100
H	15.20500	26.64800	-4.58800
O	18.90100	12.04700	-9.80100
H	18.55700	12.93700	-9.87700
H	19.26900	11.85700	-10.66400
O	6.97000	24.37000	-16.98200
H	7.80700	24.78000	-17.19900
H	7.14700	23.43000	-17.01200
O	18.86600	22.03800	-21.29400
H	18.35100	22.80800	-21.05500
H	18.59400	21.36400	-20.67100
O	16.45100	16.88300	-16.23500
H	15.59500	16.49400	-16.41500
H	16.86300	16.96200	-17.09500
O	17.98100	14.41800	-11.83600
H	17.47700	14.38100	-12.64900
H	18.27400	15.32700	-11.77700
O	14.22500	14.50100	-12.83000
H	15.09000	14.30600	-13.18900
H	14.29300	14.27200	-11.90300
O	23.83900	24.79100	-19.35900
H	23.06200	24.45200	-19.80300
H	24.51900	24.79600	-20.03300
O	18.65500	19.91100	-19.46800
H	18.35000	20.22500	-18.61700
H	19.60800	19.98900	-19.42500
O	17.09900	26.07000	-17.81900
H	16.57100	25.33500	-18.13100
H	16.59800	26.84700	-18.06500
O	22.07900	24.26500	-12.70200
H	21.42100	24.95400	-12.61400
H	22.88300	24.65400	-12.35900
O	11.83100	16.73200	-15.45800
H	12.40600	16.50900	-16.19100
H	12.41800	16.80000	-14.70500
O	20.88900	22.61900	-14.65200
H	21.40200	23.25200	-14.14900
H	21.53900	22.01900	-15.01700
O	12.15800	18.76200	-21.43800
H	11.32200	19.10000	-21.11800
H	12.01900	18.62700	-22.37600
O	14.13300	13.60600	-16.73100
H	13.48000	13.29100	-17.35500
H	14.09100	12.98300	-16.00500
O	15.24500	23.88800	-24.81500
H	14.33700	23.65700	-24.61600
H	15.74200	23.09100	-24.62800
O	18.15100	32.59700	-21.89600
H	17.47800	32.90100	-21.28700
H	18.53800	33.40000	-22.24500
O	17.36700	20.09400	-12.40600
H	16.44900	20.31500	-12.56400
H	17.78300	20.16900	-13.26500
O	18.15100	21.49300	-17.27500
H	18.89800	22.00500	-17.58500
H	18.42000	21.17100	-16.41500
O	16.42300	23.98900	-20.29300
H	15.51500	23.70500	-20.19600
H	16.45300	24.40900	-21.15200
O	21.65500	27.60000	-16.52400
H	21.20200	26.75900	-16.46700
H	21.02500	28.23600	-16.18600
O	20.59900	18.52900	-15.29400
H	19.86100	18.01700	-14.96300
H	20.85100	18.08900	-16.10500
O	20.09900	29.43700	-15.58900
H	20.39100	29.99800	-16.30700
H	20.60800	29.73000	-14.83300
O	20.70000	11.30600	-14.69900
H	21.00600	10.44600	-14.98700
H	21.42500	11.89800	-14.90000
O	6.99200	21.70900	-16.31100
H	6.79500	21.11600	-17.03600

H	6.43700	21.40500	-15.59400
O	14.97200	21.60200	-14.19200
H	15.02700	20.64900	-14.26800
H	14.16100	21.75800	-13.70800
O	16.12100	25.53000	-22.95400
H	15.68300	24.81300	-23.41300
H	16.86200	25.75700	-23.51500
O	12.91100	20.21900	-16.43300
H	12.09800	20.51900	-16.02600
H	13.40700	19.82500	-15.71500
O	15.06200	30.21500	-14.49900
H	15.02000	30.44100	-15.42800
H	15.97800	29.98200	-14.35100
O	18.50900	20.68400	-7.60100
H	18.86300	20.91100	-6.74200
H	19.15600	21.01600	-8.22400
O	24.23800	23.80600	-16.89600
H	24.10000	23.99600	-17.82400
H	23.88200	24.56700	-16.44000
O	12.40300	17.79100	-10.81700
H	13.04200	17.65000	-11.51400
H	11.57000	17.50500	-11.19300
O	24.26500	25.87300	-11.88500
H	24.69900	25.78100	-11.03700
H	24.97800	25.84900	-12.52300
O	13.17900	27.09200	-14.70300
H	13.97300	26.59700	-14.90500
H	12.46800	26.56300	-15.06600
O	17.99800	29.47300	-10.35200
H	18.05200	30.04200	-9.58400
H	17.13000	29.64400	-10.71700
O	9.48700	21.23600	-18.62700
H	8.55700	21.17400	-18.41000
H	9.58300	20.71300	-19.42400
O	9.40500	17.76100	-16.03900
H	9.66600	18.67600	-16.14800
H	10.22700	17.29400	-15.89000
O	10.87800	19.17500	-8.86900
H	11.55000	18.96700	-9.51800
H	10.05100	18.97700	-9.30900
O	25.12700	25.91600	-9.35200
H	25.32000	26.82000	-9.10600
H	25.02700	25.45600	-8.51800
O	16.78000	15.80100	-6.85300
H	16.13300	16.50000	-6.94600
H	17.55100	16.23500	-6.48500
O	20.18800	26.06600	-3.77800
H	20.03700	25.83700	-4.69500
H	20.17900	27.02300	-3.76500
O	16.95700	23.63900	-8.79300
H	17.88600	23.79100	-8.96600
H	16.53600	23.72800	-9.64800
O	15.75600	27.66400	-11.02300
H	15.61700	27.29600	-10.15100
H	14.96700	28.17900	-11.19500
O	16.35000	32.76500	-19.68100
H	16.52600	32.29400	-18.86600
H	15.66400	33.39200	-19.45300
O	17.30600	27.04300	-13.63400
H	16.97100	27.00300	-12.73900
H	16.80700	26.37700	-14.10700
O	11.28100	22.44900	-8.10900
H	11.14300	21.71700	-7.50800
H	12.19100	22.36000	-8.39100
O	20.24200	24.38800	-23.36100
H	19.62500	23.66300	-23.45700
H	19.77400	25.14900	-23.70400
O	13.90900	22.16100	-8.62100
H	14.61900	21.52100	-8.66200
H	13.86600	22.41300	-7.69800
O	21.89800	29.09800	-5.70000
H	21.83900	28.41400	-6.36700
H	22.29900	29.84000	-6.15200
O	12.62200	19.14800	-6.80100
H	12.08500	18.69700	-6.14900
H	12.03300	19.28300	-7.54400
O	10.91700	35.91400	-12.90700
H	11.15600	36.54600	-12.22900
H	10.07400	35.56300	-12.62000
O	12.03200	18.60700	-24.23900
H	11.15500	18.48600	-24.60200
H	12.50800	17.81500	-24.49100
O	21.89900	27.06200	-7.46900
H	21.01200	26.72500	-7.34600
H	22.46900	26.33000	-7.23200
O	3.55200	23.39400	-14.14400
H	4.23900	23.08100	-14.73200
H	3.39700	24.29900	-14.41800
O	8.35600	18.28400	-9.71600
H	8.27100	17.67600	-10.45100
H	7.53400	18.77400	-9.71500
O	15.42900	31.72200	-10.50300
H	15.35600	32.52800	-9.99100
H	14.98700	31.92100	-11.32800
O	15.21800	30.95900	-16.99200
H	14.43500	31.50200	-16.89900
H	15.91800	31.47700	-16.59300
O	18.52800	16.76900	-14.34900
H	19.02500	16.01600	-14.66900
H	17.75700	16.80500	-14.91500
O	26.38100	22.47300	-12.70700
H	26.37000	23.35500	-13.07800
H	25.51300	22.12000	-12.90200
O	17.88900	31.62100	-8.34700
H	18.60200	32.25600	-8.41900
H	17.39800	31.71700	-9.16300
O	19.61100	25.52600	-16.77500
H	18.67700	25.64600	-16.94500
H	19.78900	24.62300	-17.03900
O	15.11200	18.91100	-14.62200
H	14.74100	18.35800	-13.93400
H	15.60700	18.30600	-15.17400
O	22.99800	21.36800	-16.24000
H	23.46400	22.20100	-16.31400
H	23.44900	20.90200	-15.53600
O	12.94000	29.37000	-7.52600
H	13.64100	30.00400	-7.37300
H	12.47000	29.71200	-8.28700

O	21.06600	27.71200	-19.24200
H	21.34200	27.59800	-18.33200
H	20.19600	27.31400	-19.28000
O	20.97000	13.73600	-12.20100
H	20.05600	13.96600	-12.03300
H	21.39900	13.82000	-11.35000
O	20.74100	21.92500	-9.70100
H	20.08800	22.17200	-10.35600
H	21.11900	21.11100	-10.03500
O	17.70100	31.96100	-15.59800
H	17.88400	31.14300	-15.13500
H	18.52500	32.17800	-16.03600
O	18.03600	30.88800	-18.33700
H	18.56400	30.37700	-18.95000
H	18.29800	30.57200	-17.47200
O	16.78900	22.66700	-3.21400
H	16.11300	22.56600	-2.54400
H	17.61200	22.51100	-2.75100
O	8.53700	28.90400	-10.59300
H	9.12300	28.15800	-10.46000
H	8.34000	29.21400	-9.71000
O	11.39100	31.93900	-15.57700
H	10.72200	32.42400	-16.06100
H	11.25200	32.18600	-14.66300
O	3.12900	21.47500	-11.87300
H	3.16700	20.94100	-11.07900
H	2.62300	20.94800	-12.49200
O	15.53200	33.97300	-8.97900
H	16.08300	34.13700	-8.21400
H	15.87200	34.56400	-9.65100
O	10.50800	16.32100	-8.37600
H	10.57200	17.18200	-8.78900
H	10.44500	15.70800	-9.10900
O	9.98300	18.39300	-4.72900
H	10.61000	18.01100	-4.11500
H	9.12600	18.17500	-4.36400
O	20.38300	28.80000	-3.12400
H	20.92700	29.41300	-3.61800
H	19.80100	29.35800	-2.60700
O	6.76000	20.20100	-18.79700
H	7.05500	19.29200	-18.85100
H	5.85900	20.18000	-19.11900
O	18.66800	31.92200	-11.99200
H	18.54400	31.12100	-12.50100
H	19.32600	32.41600	-12.48100
O	10.28900	18.27700	-12.81500
H	9.56800	18.89000	-12.95400
H	11.07900	18.81200	-12.89300
O	20.73200	29.72300	-10.36400
H	19.78500	29.60300	-10.43700
H	21.08000	28.83500	-10.28200
O	13.09200	15.61200	-6.54100
H	13.61500	15.85800	-5.77800
H	12.87300	16.44400	-6.96000
O	8.48900	17.88200	-18.67300
H	8.16000	17.00200	-18.85600
H	8.84300	17.82700	-17.78600
O	17.73700	29.51800	-14.16400
H	17.58900	28.59000	-13.98300
H	18.56200	29.54000	-14.64800
O	11.60300	18.17000	-18.30600
H	11.94200	18.69800	-19.02800
H	12.05000	18.50800	-17.53000
O	21.34000	30.34100	-13.07700
H	22.26500	30.13500	-13.21500
H	21.17200	30.09200	-12.16900
O	14.29700	16.55300	-8.85600
H	13.95700	15.75400	-8.45300
H	13.59100	16.85400	-9.42800
O	14.94900	13.63800	-10.40300
H	15.77100	13.16400	-10.52500
H	15.19300	14.41700	-9.90300
O	13.59000	16.66700	-20.10700
H	14.31000	17.04500	-20.61000
H	12.96600	17.38600	-20.00300
O	17.73300	17.37200	-19.17100
H	17.94900	18.25400	-19.47200
H	17.25200	16.98000	-19.90000
O	12.47300	20.33000	-12.59400
H	12.32700	20.64400	-11.70100
H	11.99500	20.94900	-13.14700
O	14.33200	17.32000	-12.54600
H	14.31800	16.42500	-12.88200
H	15.05000	17.32800	-11.91200
O	16.18500	17.75400	-10.50000
H	15.92700	18.67600	-10.50200
H	15.65300	17.35700	-9.81000
O	21.13000	30.85900	-17.72100
H	21.30300	30.29400	-18.47400
H	21.62200	31.65900	-17.90300
O	6.94700	22.87100	-9.65500
H	6.40300	23.64700	-9.52300
H	7.82500	23.21800	-9.81300
O	13.73600	16.20200	-17.27700
H	13.85800	15.27400	-17.07800
H	13.44600	16.21600	-18.18900
O	16.60500	13.88700	-14.14000
H	16.62700	13.95100	-15.09500
H	17.02200	13.04800	-13.94600
O	26.98300	23.62300	-16.10200
H	27.42700	22.87400	-16.50000
H	26.10600	23.61400	-16.48500
O	22.11700	30.17600	-20.62200
H	22.81200	30.79700	-20.40300
H	22.45900	29.32600	-20.34600
O	7.73400	21.99800	-6.76200
H	6.99200	22.14600	-6.17500
H	7.41300	22.25900	-7.62500
O	24.01600	27.62600	-19.09300
H	24.54100	27.96500	-19.81800
H	23.92800	26.69100	-19.27600
O	15.67800	31.53900	-6.96400
H	15.98700	31.29400	-6.09200
H	16.47600	31.74400	-7.45200
O	15.70000	33.69300	-14.73300
H	15.26100	33.20100	-14.04000
H	16.45100	33.15000	-14.97400
O	8.17900	29.88400	-14.73100

H	9.00500	29.58500	-15.11100
H	7.82900	29.11500	-14.28200
O	13.14800	29.23700	-12.66100
H	12.52600	28.57400	-12.96100
H	13.81800	29.26300	-13.34400
O	6.38000	27.80300	-13.77200
H	5.54400	27.37500	-13.95400
H	6.35300	27.99600	-12.83500
O	13.22000	17.71500	-3.06800
H	13.26900	18.59100	-3.45000
H	13.76200	17.17300	-3.64100
O	23.70600	28.25900	-3.78800
H	22.91500	28.24200	-4.32700
H	23.46900	28.79800	-3.03300
O	23.82500	25.10800	-6.47400
H	23.87500	24.94300	-5.53300
H	23.71700	24.24100	-6.86500
O	12.70900	27.78700	-19.18500
H	12.85800	28.47100	-19.83900
H	13.51600	27.76800	-18.67000
O	21.11600	21.09200	-5.15800
H	22.01700	20.95200	-5.45000
H	21.19100	21.25700	-4.21900
O	15.45000	18.46800	-6.31300
H	14.58000	18.76200	-6.58500
H	15.68600	19.05200	-5.59200
O	25.32700	27.52800	-6.23000
H	25.03600	27.89100	-5.39400
H	24.71400	26.81500	-6.40900
O	26.52700	25.09300	-13.71000
H	27.36100	25.53200	-13.54600
H	26.66300	24.61700	-14.52900
O	23.57600	22.65100	-7.76200
H	24.05100	22.84400	-8.57000
H	22.65200	22.70700	-8.00800
O	10.46400	23.81400	-18.17900
H	10.21900	22.91000	-17.98100
H	10.63400	23.81600	-19.12100
O	18.15400	14.68600	-8.79700
H	18.67200	15.42200	-9.12200
H	17.79900	14.99300	-7.96200
O	9.73400	19.54700	-20.68000
H	9.04100	19.62100	-21.33600
H	9.48900	18.78300	-20.15800
O	18.40500	11.10600	-13.27700
H	18.68500	10.43900	-12.65000
H	19.19000	11.29900	-13.78900
O	16.20900	30.28200	-21.71400
H	15.63600	30.90100	-22.16700
H	16.78200	30.83200	-21.18000
O	22.27600	19.52800	-9.52900
H	23.22800	19.62800	-9.50800
H	22.10600	18.73600	-9.02000
O	20.29800	16.61400	-9.34300
H	19.70200	16.89400	-10.03900
H	20.01200	17.10300	-8.57200
O	19.42800	30.56600	-20.58300
H	20.36100	30.50000	-20.38000
H	19.34100	31.40100	-21.04200
O	22.05700	29.88200	-23.62300
H	21.97800	29.54800	-22.72900
H	22.87800	30.37500	-23.62400
O	24.75500	22.74900	-10.41500
H	24.72300	21.81000	-10.22900
H	25.36500	22.82700	-11.14900
O	16.59300	21.61800	-24.37200
H	17.53900	21.48900	-24.30400
H	16.21500	20.88000	-23.89500
O	24.90400	19.54000	-10.36900
H	24.38800	18.75600	-10.55600
H	25.78800	19.21200	-10.20500
O	22.56500	13.28400	-14.41000
H	22.36800	12.97500	-13.52500
H	23.17500	14.01000	-14.28000
O	30.57300	23.81000	-11.27500
H	31.30300	23.54900	-10.71400
H	29.96300	23.07300	-11.23400
O	28.65400	21.95100	-10.67700
H	27.92700	21.81700	-11.28500
H	28.23900	22.25900	-9.87100
O	16.47700	25.67000	-2.91200
H	17.39700	25.84800	-2.71800
H	16.44200	24.72600	-3.06400
O	14.77700	19.54300	-24.43700
H	13.87100	19.24700	-24.36100
H	15.05500	19.24600	-25.30400
O	5.69500	20.86400	-13.98700
H	5.37600	20.01000	-14.28000
H	5.14900	21.07900	-13.23100
O	9.47300	23.58000	-10.27000
H	9.69200	24.51200	-10.25100
H	9.88600	23.22100	-9.48500
O	16.39400	14.08700	-18.28300
H	15.58400	13.74200	-17.90700
H	17.08200	13.77800	-17.69400
O	18.32300	13.16500	-16.66000
H	18.87600	13.58400	-16.00100
H	18.56000	12.23800	-16.61800
O	14.34600	19.15300	-0.88000
H	14.24600	20.10300	-0.94900
H	14.12800	18.82500	-1.75200
O	13.54700	16.40200	-24.29600
H	14.40900	16.72700	-24.03600
H	13.73200	15.61500	-24.80800
O	5.56400	24.48400	-12.01800
H	4.87000	24.07000	-12.53100
H	6.31100	24.52600	-12.61400
O	21.70100	15.27500	-17.15200
H	21.12100	15.93300	-17.53400
H	21.43400	14.45300	-17.56300
O	20.15100	14.67500	-14.95000
H	20.65100	14.78700	-15.75900
H	20.79500	14.37100	-14.31000
O	14.20900	22.58300	-2.06300
H	13.72600	23.38300	-1.85700
H	14.29500	22.59400	-3.01700
O	7.47800	24.41600	-14.14100
H	7.45500	24.51500	-15.09300

H	7.78800	25.26200	-13.81900
O	10.13700	26.47400	-10.02600
H	11.09100	26.44200	-10.09300
H	9.93800	26.01300	-9.21100
O	10.72600	25.04400	-7.45900
H	11.54600	25.53800	-7.43600
H	11.00000	24.12700	-7.48800
O	20.48300	17.43500	-18.09700
H	19.59600	17.72000	-18.31500
H	20.89900	17.27400	-18.94400
O	23.83500	17.45900	-14.38100
H	24.61100	17.94900	-14.10900
H	23.10900	17.88500	-13.92400
O	26.27200	18.17200	-13.32400
H	26.86600	17.93200	-14.03400
H	26.34400	17.45300	-12.69600
O	8.49200	32.01600	-13.00900
H	8.35700	31.25600	-13.57600
H	8.07500	32.74000	-13.47700
O	19.62700	27.19900	-24.00200
H	20.33100	27.40400	-23.38600
H	18.90400	27.76700	-23.73700
O	20.45700	22.95000	-17.88200
H	20.40100	23.40700	-18.72100
H	21.01000	22.18900	-18.05900
O	24.26800	18.73900	-17.05700
H	24.01100	18.33900	-16.22600
H	23.64300	19.45400	-17.18200
O	28.57900	21.47900	-14.54900
H	28.01900	21.54800	-13.77600
H	27.97300	21.30300	-15.26900
O	11.64800	32.91200	-12.79800
H	11.53200	33.85700	-12.90000
H	10.82800	32.61000	-12.40900
O	21.53600	27.63700	-21.95200
H	22.37100	27.20600	-22.13400
H	21.37500	27.46600	-21.02400
O	21.19100	24.14100	-20.58600
H	21.13600	23.97500	-21.52700
H	20.56500	24.84800	-20.43000
O	5.98000	19.37800	-6.76000
H	6.62600	18.86700	-6.27100
H	5.77200	20.11400	-6.18600
O	18.50500	26.44600	-1.26100
H	19.37400	26.62000	-1.62200
H	18.02600	27.26500	-1.38300
O	14.50000	13.64200	-21.00800
H	14.48800	14.58600	-20.85100
H	15.39300	13.45900	-21.30200
O	12.67400	33.56500	-9.58200
H	13.57700	33.59800	-9.26500
H	12.44800	32.63500	-9.56700
O	14.42900	32.31800	-12.92100
H	13.47900	32.31900	-13.03500
H	14.73300	31.57200	-13.43700
O	18.31600	10.23600	-16.75300
H	17.54900	9.68200	-16.60800
H	18.72700	10.30800	-15.89200
O	10.32000	16.52400	-2.66300
H	11.18300	16.16800	-2.45200
H	9.74600	16.18600	-1.97500
O	30.16500	38.15400	-11.85600
H	30.80200	38.03500	-12.56000
H	30.04700	39.10300	-11.79800
O	26.97400	38.10700	-11.92000
H	26.63500	37.99100	-12.80800
H	27.84200	37.70600	-11.94000
O	22.83200	39.96100	-15.93100
H	22.26900	39.87000	-16.69900
H	22.71700	39.14100	-15.45000
O	27.45500	39.26100	-5.96200
H	27.44400	38.86600	-6.83400
H	27.61800	38.52700	-5.36800
O	20.37900	41.45600	-13.09200
H	21.17100	41.11500	-13.50800
H	20.61800	41.56500	-12.17200
O	24.17600	42.04400	-7.01400
H	23.88500	42.94900	-7.12900
H	24.81200	42.08600	-6.29900
O	25.10200	39.42200	-10.42400
H	25.69800	39.19800	-11.13800
H	25.15900	38.68100	-9.82100
O	29.71900	35.88000	-14.78100
H	30.50500	35.46200	-15.13200
H	29.98300	36.78400	-14.61300
O	35.12000	35.81700	-13.87800
H	35.85200	35.24700	-13.64400
H	34.36300	35.42900	-13.43700
O	26.73700	38.24000	-16.68100
H	26.37300	37.71300	-15.97000
H	27.59400	37.85000	-16.85500
O	32.64200	29.52900	-9.41800
H	32.31000	30.39900	-9.64200
H	32.04700	29.21300	-8.73900
O	22.58600	38.18200	-13.98500
H	21.66200	38.05000	-13.77700
H	22.83500	38.96100	-13.48800
O	32.53500	34.24900	-12.33900
H	31.62700	34.18000	-12.63300
H	32.48400	34.72600	-11.51100
O	26.35700	35.72500	-20.70100
H	26.23700	36.17000	-19.86200
H	25.66200	36.07300	-21.25900
O	19.94700	37.82500	-13.15100
H	20.07700	38.07900	-12.23700
H	19.26700	38.41800	-13.47000
O	26.60900	35.66800	-4.39600
H	25.83200	36.22700	-4.40500
H	27.24800	36.15300	-3.87400
O	34.40100	32.45000	-10.56100
H	33.75900	32.74400	-11.20700
H	33.89700	32.33600	-9.75600
O	24.86400	38.47600	-4.83400
H	25.60500	39.01500	-5.11000
H	24.15700	39.10300	-4.67700
O	26.69200	35.55600	-8.86300
H	27.53800	35.98000	-8.72000
H	26.63200	34.90000	-8.16900

O	29.84200	33.21700	-7.20500
H	29.59800	33.97100	-7.74200
H	30.11800	33.59500	-6.37000
O	29.39900	32.37300	-11.62400
H	28.53700	32.67100	-11.33200
H	29.42500	32.59000	-12.55600
O	26.21500	40.91700	-15.21600
H	26.67900	41.19600	-16.00600
H	25.95100	40.01500	-15.39900
O	23.10700	40.46400	-12.27800
H	23.67700	40.89300	-12.91700
H	23.69800	40.18200	-11.58000
O	25.15200	37.03900	-18.40700
H	25.85400	37.49100	-17.94000
H	24.82000	36.39700	-17.77900
O	23.60000	35.63000	-4.03200
H	22.97000	36.31200	-3.79900
H	23.49300	35.51900	-4.97700
O	18.89400	35.29700	-13.10600
H	18.10400	35.55300	-13.58200
H	19.38500	36.11200	-13.00000
O	20.84600	35.66200	-16.14700
H	20.04900	35.77400	-15.62900
H	20.61800	36.00100	-17.01300
O	17.17800	35.55000	-10.67400
H	17.42900	36.43500	-10.40800
H	17.71400	35.37100	-11.44700
O	22.87800	29.93200	-1.67000
H	22.48500	29.37900	-0.99500
H	23.69400	30.24300	-1.27900
O	19.84400	41.37100	-17.75800
H	20.35700	42.16500	-17.60800
H	20.49600	40.67300	-17.82500
O	25.79500	31.34800	-4.07800
H	26.53600	31.94900	-4.15700
H	26.02600	30.60600	-4.63600
O	20.93900	36.53400	-5.80000
H	20.82400	35.63200	-5.49900
H	20.08300	36.78000	-6.15100
O	29.74000	27.37600	-9.80100
H	29.39700	28.26600	-9.87700
H	30.10800	27.18600	-10.66400
O	17.80900	39.70000	-16.98200
H	18.64600	40.10900	-17.19900
H	17.98700	38.76000	-17.01200
O	27.29000	32.21200	-16.23500
H	26.43400	31.82300	-16.41500
H	27.70300	32.29100	-17.09500
O	28.82000	29.74700	-11.83600
H	28.31700	29.71000	-12.64900
H	29.11300	30.65600	-11.77700
O	25.06400	29.83100	-12.83000
H	25.93000	29.63500	-13.18900
H	25.13200	29.60200	-11.90300
O	29.49400	35.24000	-19.46800
H	29.18900	35.55400	-18.61700
H	30.44700	35.31800	-19.42500
O	22.67100	32.06100	-15.45800
H	23.24500	31.83800	-16.19100
H	23.25800	32.13000	-14.70500
O	22.99800	34.09100	-21.43800
H	22.16100	34.42900	-21.11800
H	22.85800	33.95600	-22.37600
O	24.97300	28.93500	-16.73100
H	24.31900	28.62000	-17.35500
H	24.93100	28.31300	-16.00500
O	28.20700	35.42300	-12.40600
H	27.28900	35.64500	-12.56400
H	28.62300	35.49800	-13.26500
O	28.99000	36.82200	-17.27500
H	29.73700	37.33400	-17.58500
H	29.25900	36.50100	-16.41500
O	31.43800	33.85800	-15.29400
H	30.70000	33.34600	-14.96300
H	31.69100	33.41800	-16.10500
O	31.53900	26.63600	-14.69900
H	31.84500	25.77500	-14.98700
H	32.26400	27.22700	-14.90000
O	17.83200	37.03900	-16.31100
H	17.63400	36.44600	-17.03600
H	17.27600	36.73400	-15.59400
O	25.81100	36.93100	-14.19200
H	25.86600	35.97800	-14.26800
H	25.00000	37.08700	-13.70800
O	23.75000	35.54900	-16.43300
H	22.93700	35.84800	-16.02600
H	24.24600	35.15400	-15.71500
O	29.34800	36.01300	-7.60100
H	29.70300	36.24000	-6.74200
H	29.99500	36.34500	-8.22400
O	23.24200	33.12100	-10.81700
H	23.88200	32.97900	-11.51400
H	22.41000	32.83400	-11.19300
O	24.01800	42.42100	-14.70300
H	24.81200	41.92600	-14.90500
H	23.30700	41.89300	-15.06600
O	20.32600	36.56500	-18.62700
H	19.39600	36.50400	-18.41000
H	20.42200	36.04300	-19.42400
O	20.24500	33.09100	-16.03900
H	20.50500	34.00500	-16.14800
H	21.06600	32.62300	-15.89000
O	21.71700	34.50400	-8.86900
H	22.39000	34.29600	-9.51800
H	20.89000	34.30700	-9.30900
O	27.62000	31.13100	-6.85300
H	26.97300	31.83000	-6.94600
H	28.39000	31.56400	-6.48500
O	27.79600	38.96900	-8.79300
H	28.72500	39.12000	-8.96600
H	27.37500	39.05700	-9.64800
O	22.12000	37.77900	-8.10900
H	21.98200	37.04700	-7.50800
H	23.03000	37.68900	-8.39100
O	24.74800	37.49100	-8.62100
H	25.45800	36.85000	-8.66200
H	24.70500	37.74200	-7.69800
O	23.46100	34.47700	-6.80100

H	22.92500	34.02600	-6.14900
H	22.87300	34.61200	-7.54400
O	14.39200	38.72400	-14.14400
H	15.07900	38.41000	-14.73200
H	14.23600	39.62800	-14.41800
O	19.19500	33.61300	-9.71600
H	19.11100	33.00500	-10.45100
H	18.37300	34.10400	-9.71500
O	29.36700	32.09800	-14.34900
H	29.86400	31.34500	-14.66900
H	28.59600	32.13400	-14.91500
O	25.95100	34.24000	-14.62200
H	25.58100	33.68700	-13.93400
H	26.44700	33.63600	-15.17400
O	31.80900	29.06500	-12.20100
H	30.89600	29.29500	-12.03300
H	32.23900	29.14900	-11.35000
O	31.58000	37.25400	-9.70100
H	30.92700	37.50200	-10.35600
H	31.95800	36.44000	-10.03500
O	27.62900	37.99600	-3.21400
H	26.95300	37.89500	-2.54400
H	28.45100	37.84000	-2.75100
O	19.37700	44.23300	-10.59300
H	19.96200	43.48800	-10.46000
H	19.17900	44.54400	-9.71000
O	13.96800	36.80400	-11.87300
H	14.00700	36.27000	-11.07900
H	13.46200	36.27700	-12.49200
O	21.34700	31.65000	-8.37600
H	21.41200	32.51200	-8.78900
H	21.28400	31.03800	-9.10900
O	20.82300	33.72300	-4.72900
H	21.45000	33.34000	-4.11500
H	19.96500	33.50400	-4.36400
O	17.59900	35.53000	-18.79700
H	17.89500	34.62100	-18.85100
H	16.69800	35.50900	-19.11900
O	21.12900	33.60600	-12.81500
H	20.40700	34.21900	-12.95400
H	21.91800	34.14200	-12.89300
O	23.93100	30.94100	-6.54100
H	24.45500	31.18700	-5.77800
H	23.71200	31.77300	-6.96000
O	19.32800	33.21200	-18.67300
H	18.99900	32.33100	-18.85600
H	19.68200	33.15600	-17.78600
O	22.44200	33.49900	-18.30600
H	22.78200	34.02700	-19.02800
H	22.88900	33.83700	-17.53000
O	25.13700	31.88300	-8.85600
H	24.79600	31.08300	-8.45300
H	24.43000	32.18300	-9.42800
O	25.78800	28.96800	-10.40300
H	26.61100	28.49400	-10.52500
H	26.03200	29.74700	-9.90300
O	24.42900	31.99600	-20.10700
H	25.15000	32.37400	-20.61000
H	23.80600	32.71500	-20.00300
O	28.57200	32.70100	-19.17100
H	28.78800	33.58300	-19.47200
H	28.09200	32.30900	-19.90000
O	23.31200	35.65900	-12.59400
H	23.16600	35.97300	-11.70100
H	22.83500	36.27800	-13.14700
O	25.17200	32.65000	-12.54600
H	25.15800	31.75400	-12.88200
H	25.88900	32.65700	-11.91200
O	27.02400	33.08300	-10.50000
H	26.76600	34.00500	-10.50200
H	26.49300	32.68600	-9.81000
O	17.78700	38.20000	-9.65500
H	17.24200	38.97600	-9.52300
H	18.66500	38.54700	-9.81300
O	24.57500	31.53100	-17.27700
H	24.69700	30.60300	-17.07800
H	24.28600	31.54500	-18.18900
O	27.44400	29.21700	-14.14000
H	27.46700	29.28100	-15.09500
H	27.86100	28.37700	-13.94600
O	18.57300	37.32700	-6.76200
H	17.83100	37.47500	-6.17500
H	18.25200	37.58800	-7.62500
O	19.01900	45.21300	-14.73100
H	19.84500	44.91400	-15.11100
H	18.66800	44.44400	-14.28200
O	17.22000	43.13200	-13.77200
H	16.38300	42.70400	-13.95400
H	17.19200	43.32500	-12.83500
O	24.05900	33.04400	-3.06800
H	24.10800	33.92100	-3.45000
H	24.60200	32.50200	-3.64100
O	31.95500	36.42100	-5.15800
H	32.85600	36.28100	-5.45000
H	32.03000	36.58600	-4.21900
O	26.28900	33.79700	-6.31300
H	25.42000	34.09100	-6.58500
H	26.52500	34.38100	-5.59200
O	21.30400	39.14300	-18.17900
H	21.05800	38.23900	-17.98100
H	21.47300	39.14500	-19.12100
O	28.99300	30.01500	-8.79700
H	29.51100	30.75200	-9.12200
H	28.63900	30.32200	-7.96200
O	20.57300	34.87600	-20.68000
H	19.88000	34.95000	-21.33600
H	20.32800	34.11200	-20.15800
O	29.24500	26.43500	-13.27700
H	29.52500	25.76800	-12.65000
H	30.03000	26.62800	-13.78900
O	33.11500	34.85700	-9.52900
H	34.06700	34.95700	-9.50800
H	32.94600	34.06500	-9.02000
O	31.13700	31.94300	-9.34300
H	30.54200	32.22300	-10.03900
H	30.85200	32.43200	-8.57200
O	35.74400	34.86900	-10.36900
H	35.22800	34.08500	-10.55600

H	36.62800	34.54100	-10.20500
O	33.40500	28.61300	-14.41000
H	33.20700	28.30500	-13.52500
H	34.01500	29.33900	-14.28000
O	16.53500	36.19300	-13.98700
H	16.21500	35.34000	-14.28000
H	15.98800	36.40800	-13.23100
O	20.31300	38.90900	-10.27000
H	20.53100	39.84100	-10.25100
H	20.72500	38.55000	-9.48500
O	27.23300	29.41600	-18.28300
H	26.42300	29.07100	-17.90700
H	27.92200	29.10700	-17.69400
O	29.16200	28.49400	-16.66000
H	29.71500	28.91300	-16.00100
H	29.39900	27.56700	-16.61800
O	25.18500	34.48300	-0.88000
H	25.08500	35.43200	-0.94900
H	24.96800	34.15400	-1.75200
O	16.40300	39.81300	-12.01800
H	15.71000	39.39900	-12.53100
H	17.15100	39.85500	-12.61400
O	32.54000	30.60400	-17.15200
H	31.96000	31.26200	-17.53400
H	32.27400	29.78200	-17.56300
O	30.99000	30.00400	-14.95000
H	31.49000	30.11600	-15.75900
H	31.63400	29.70100	-14.31000
O	25.04900	37.91200	-2.06300
H	24.56500	38.71200	-1.85700
H	25.13500	37.92300	-3.01700
O	18.31700	39.74500	-14.14100
H	18.29400	39.84400	-15.09300
H	18.62700	40.59200	-13.81900
O	20.97600	41.80300	-10.02600
H	21.93000	41.77100	-10.09300
H	20.77700	41.34200	-9.21100
O	21.56600	40.37300	-7.45900
H	22.38500	40.86700	-7.43600
H	21.84000	39.45600	-7.48800
O	31.32300	32.76400	-18.09700
H	30.43500	33.04900	-18.31500
H	31.73800	32.60300	-18.94400
O	34.67400	32.78900	-14.38100
H	35.45000	33.27800	-14.10900
H	33.94900	33.21400	-13.92400
O	37.11100	33.50100	-13.32400
H	37.70600	33.26100	-14.03400
H	37.18400	32.78200	-12.69600
O	35.10700	34.06800	-17.05700
H	34.85100	33.66800	-16.22600
H	34.48200	34.78300	-17.18200
O	16.81900	34.70700	-6.76000
H	17.46500	34.19700	-6.27100
H	16.61100	35.44400	-6.18600
O	25.34000	28.97100	-21.00800
H	25.32800	29.91500	-20.85100
H	26.23200	28.78800	-21.30200
O	29.15500	25.56500	-16.75300
H	28.38800	25.01100	-16.60800
H	29.56600	25.63700	-15.89200
O	21.15900	31.85300	-2.66300
H	22.02200	31.49700	-2.45200
H	20.58600	31.51500	-1.97500
O	-19.18100	-42.26600	-20.28900
H	-19.47200	-41.36200	-20.40500
H	-18.54600	-42.22500	-19.57500
O	-18.49400	-45.83400	-18.10900
H	-17.75200	-45.29600	-18.38600
H	-19.20000	-45.20800	-17.95200
O	-13.01800	-43.69100	-19.51200
H	-12.61000	-44.13300	-20.25700
H	-13.74100	-44.26600	-19.25900
O	-10.44700	-41.67300	-23.63100
H	-9.62500	-42.07500	-23.91200
H	-10.45100	-41.77600	-22.67900
O	-15.34000	-39.83500	-17.47700
H	-15.78300	-40.62100	-17.79400
H	-14.67800	-39.64700	-18.14200
O	-13.24100	-39.55500	-19.44800
H	-13.26000	-38.73400	-19.94000
H	-12.31900	-39.67900	-19.22300
O	-16.78400	-42.04000	-18.60600
H	-16.25100	-42.80100	-18.83600
H	-17.31300	-42.33300	-17.86400
O	-7.39100	-43.06600	-22.62700
H	-7.19800	-42.16100	-22.38100
H	-7.49100	-43.52500	-21.79400
O	-12.33000	-42.91500	-17.05300
H	-12.48100	-43.14400	-17.97000
H	-12.33900	-41.95800	-17.04000
O	-10.62100	-39.88300	-18.97600
H	-10.67900	-40.56700	-19.64300
H	-10.21900	-39.14100	-19.42800
O	-10.61900	-41.91900	-20.74400
H	-11.50600	-42.25700	-20.62200
H	-10.04900	-42.65100	-20.50700
O	-14.62900	-37.36000	-21.62200
H	-13.91600	-36.72600	-21.69500
H	-15.12000	-37.26400	-22.43800
O	-19.57800	-39.61100	-20.80200
H	-18.87700	-38.97700	-20.64900
H	-20.04800	-39.27000	-21.56200
O	-15.72900	-46.31500	-16.49000
H	-16.40500	-46.41500	-15.82000
H	-14.90600	-46.47100	-16.02600
O	-16.98700	-35.00800	-22.25400
H	-16.43500	-34.84500	-21.48900
H	-16.64600	-34.41700	-22.92600
O	-12.13500	-40.18200	-16.39900
H	-11.59100	-39.56900	-16.89400
H	-12.71700	-39.62400	-15.88300
O	-11.78600	-39.25900	-23.64000
H	-12.73300	-39.37800	-23.71200
H	-11.43800	-40.14700	-23.55700
O	-16.84000	-37.44200	-20.23900
H	-16.53100	-37.68700	-19.36700
H	-16.04200	-37.23700	-20.72700

O	-8.81300	-40.72200	-17.06300
H	-9.60300	-40.74000	-17.60300
H	-9.04900	-40.18300	-16.30800
O	-8.69300	-43.87300	-19.75000
H	-8.64300	-44.03800	-18.80800
H	-8.80100	-44.74000	-20.14000
O	-11.40200	-47.89000	-18.43400
H	-10.50100	-48.02900	-18.72600
H	-11.32800	-47.72400	-17.49400
O	-7.19100	-41.45300	-19.50600
H	-7.48200	-41.09000	-18.66900
H	-7.80400	-42.16600	-19.68400
O	-8.94200	-46.33000	-21.03800
H	-8.46700	-46.13700	-21.84600
H	-9.86600	-46.27400	-21.28300
O	-1.94600	-45.17100	-24.55000
H	-1.21500	-45.43300	-23.98900
H	-2.55500	-45.90800	-24.51000
O	-16.04100	-43.31100	-16.18800
H	-15.12100	-43.13400	-15.99400
H	-16.07600	-44.25600	-16.34000
O	-18.30900	-46.39800	-15.33900
H	-18.79200	-45.59800	-15.13200
H	-18.22300	-46.38700	-16.29200
O	-14.01300	-42.53600	-14.53600
H	-13.14400	-42.36200	-14.89800
H	-14.49200	-41.71600	-14.65900
O	-19.84400	-35.41600	-22.85700
H	-18.94100	-35.38400	-22.54100
H	-20.07000	-36.34600	-22.84300
O	-2.35400	-30.82700	-25.13200
H	-1.71600	-30.94600	-25.83600
H	-2.47100	-29.87900	-25.07300
O	-5.54500	-30.87400	-25.19600
H	-5.88300	-30.99000	-26.08400
H	-4.67600	-31.27500	-25.21600
O	-9.68600	-29.02100	-29.20600
H	-10.24900	-29.11200	-29.97400
H	-9.80100	-29.84100	-28.72600
O	-5.06300	-29.72100	-19.23700
H	-5.07400	-30.11500	-20.10900
H	-4.90100	-30.45400	-18.64400
O	-12.13900	-27.52500	-26.36800
H	-11.34700	-27.86600	-26.78300
H	-11.90000	-27.41600	-25.44700
O	-4.10600	-25.35400	-33.89500
H	-4.75700	-24.76800	-34.28100
H	-3.31200	-24.82300	-33.83500
O	-8.34200	-26.93700	-20.28900
H	-8.63300	-26.03200	-20.40500
H	-7.70600	-26.89500	-19.57500
O	-7.41600	-29.55900	-23.69900
H	-6.82000	-29.78400	-24.41300
H	-7.35900	-30.30100	-23.09700
O	-2.79900	-33.10200	-28.05700
H	-2.01300	-33.52000	-28.40800
H	-2.53600	-32.19700	-27.88800
O	-2.62800	-27.66400	-33.35800
H	-3.26400	-27.59100	-32.64600
H	-2.95900	-27.07500	-34.03600
O	2.60100	-33.16400	-27.15300
H	3.33400	-33.73400	-26.91900
H	1.84500	-33.55200	-26.71300
O	-5.78100	-30.74200	-29.95700
H	-6.14500	-31.26800	-29.24600
H	-4.92400	-31.13200	-30.13000
O	0.12400	-39.45200	-22.69400
H	-0.20800	-38.58300	-22.91800
H	-0.47200	-39.76800	-22.01400
O	-3.38300	-27.25100	-23.29400
H	-3.68300	-26.34800	-23.40300
H	-3.72800	-27.51800	-22.44200
O	-9.93200	-30.79900	-27.26100
H	-10.85700	-30.93200	-27.05300
H	-9.68300	-30.02000	-26.76400
O	0.01700	-34.73300	-25.61400
H	-0.89200	-34.80100	-25.90900
H	-0.03400	-34.25500	-24.78600
O	-12.57100	-31.15600	-26.42600
H	-12.44200	-30.90300	-25.51200
H	-13.25100	-30.56300	-26.74600
O	-8.40100	-20.48600	-30.32700
H	-8.47800	-19.53600	-30.24800
H	-9.00500	-20.83200	-29.67000
O	-5.90900	-33.31400	-17.67100
H	-6.68600	-32.75400	-17.68100
H	-5.27000	-32.82800	-17.14900
O	1.88300	-36.53100	-23.83700
H	1.24000	-36.23800	-24.48300
H	1.37800	-36.64600	-23.03100
O	-7.65400	-30.50500	-18.10900
H	-6.91300	-29.96700	-18.38600
H	-8.36100	-29.87800	-17.95200
O	-2.17900	-28.36200	-19.51200
H	-1.77100	-28.80400	-20.25700
H	-2.90100	-28.93700	-19.25900
O	-5.82600	-33.42500	-22.13900
H	-4.98000	-33.00100	-21.99500
H	-5.88600	-34.08200	-21.44500
O	-1.85600	-27.80200	-25.75700
H	-2.48500	-27.63700	-26.45900
H	-2.24300	-27.38800	-24.98600
O	2.02300	-26.86700	-28.40200
H	1.33800	-26.48700	-28.95300
H	2.10700	-26.25700	-27.66900
O	-2.67600	-35.76400	-20.48000
H	-2.92000	-35.01000	-21.01700
H	-2.40000	-35.38600	-19.64600
O	-0.72200	-29.22100	-21.80300
H	-0.51000	-28.57700	-22.47900
H	-0.76900	-30.05400	-22.27100
O	-3.11900	-36.60900	-24.89900
H	-3.98100	-36.31100	-24.60700
H	-3.09300	-36.39200	-25.83100
O	-6.30300	-28.06500	-28.49200
H	-5.83900	-27.78600	-29.28100
H	-6.56800	-28.96600	-28.67400
O	-9.41100	-28.51700	-25.55400

H	-8.84100	-28.08900	-26.19200
H	-8.82000	-28.79900	-24.85500
O	-8.91800	-33.35100	-17.30800
H	-9.54800	-32.66900	-17.07400
H	-9.02500	-33.46300	-18.25200
O	-13.62400	-33.68400	-26.38100
H	-14.41400	-33.42800	-26.85700
H	-13.13300	-32.86900	-26.27600
O	0.39300	-26.34300	-23.63100
H	1.21400	-26.74500	-23.91200
H	0.38900	-26.44700	-22.67900
O	-5.84200	-26.79500	-21.47400
H	-5.77700	-26.33300	-20.63900
H	-6.55100	-27.42500	-21.34600
O	-15.34000	-33.43100	-23.95000
H	-15.08900	-32.54600	-23.68400
H	-14.80400	-33.61000	-24.72200
O	8.48300	-30.00000	-27.28200
H	7.73000	-30.56900	-27.43800
H	8.61800	-30.03300	-26.33500
O	-6.12700	-25.12800	-31.60700
H	-6.26200	-24.28300	-31.17800
H	-5.58600	-24.92600	-32.37000
O	-9.64000	-39.04900	-14.94500
H	-10.03400	-39.60200	-14.27000
H	-8.82400	-38.73800	-14.55400
O	-12.67400	-27.61100	-31.03400
H	-12.16200	-26.81600	-30.88300
H	-12.02200	-28.30800	-31.10000
O	-10.86500	-24.49400	-28.95100
H	-10.68800	-23.57200	-29.13900
H	-10.06900	-24.80800	-28.52200
O	-6.72300	-37.63300	-17.35300
H	-5.98300	-37.03300	-17.43300
H	-6.49200	-38.37500	-17.91100
O	-11.57900	-32.44800	-19.07500
H	-11.69400	-33.34900	-18.77500
H	-12.43500	-32.20100	-19.42700
O	-4.50000	-24.50600	-17.47700
H	-4.94400	-25.29200	-17.79400
H	-3.83800	-24.31800	-18.14200
O	-9.74100	-22.97600	-22.95000
H	-9.19100	-23.10500	-23.72300
H	-10.58000	-22.67700	-23.30100
O	-2.40200	-24.22600	-19.44800
H	-2.42000	-23.40500	-19.94000
H	-1.48000	-24.35000	-19.22300
O	-5.94500	-26.71100	-18.60600
H	-5.41200	-27.47200	-18.83600
H	-6.47300	-27.00400	-17.86400
O	-2.77800	-41.60500	-23.07700
H	-3.12100	-40.71500	-23.15300
H	-2.41000	-41.79500	-23.94000
O	-3.69800	-39.23500	-25.11200
H	-4.20200	-39.27200	-25.92500
H	-3.40500	-38.32500	-25.05200
O	-7.45400	-39.15100	-26.10500
H	-6.58900	-39.34600	-26.46500
H	-7.38600	-39.38000	-25.17800
O	2.16100	-28.86100	-32.63400
H	1.38400	-29.20000	-33.07900
H	2.84000	-28.85600	-33.30900
O	-4.57900	-27.58300	-31.09400
H	-5.10700	-28.31700	-31.40700
H	-5.08100	-26.80500	-31.34000
O	0.40000	-29.38800	-25.97700
H	-0.25800	-28.69800	-25.88900
H	1.20400	-28.99800	-25.63400
O	-0.79000	-31.03300	-27.92700
H	-0.27600	-30.40000	-27.42500
H	-0.14000	-31.63300	-28.29300
O	-3.52700	-21.05500	-35.17100
H	-4.20100	-20.75200	-34.56200
H	-3.14100	-20.25200	-35.52000
O	-4.31200	-33.55800	-25.68100
H	-5.22900	-33.33700	-25.83900
H	-3.89600	-33.48400	-26.54000
O	-3.52800	-32.15900	-30.55100
H	-2.78100	-31.64700	-30.86000
H	-3.25900	-32.48100	-29.69100
O	-0.02300	-26.05300	-29.79900
H	-0.47700	-26.89300	-29.74300
H	-0.65400	-25.41600	-29.46200
O	-1.08000	-35.12300	-28.56900
H	-1.81800	-35.63600	-28.23800
H	-0.82700	-35.56300	-29.38100
O	-1.58000	-24.21500	-28.86400
H	-1.28800	-23.65400	-29.58300
H	-1.07000	-23.92200	-28.10900
O	-6.70700	-32.05000	-27.46700
H	-6.65200	-33.00300	-27.54300
H	-7.51800	-31.89400	-26.98300
O	-6.61700	-23.43700	-27.77500
H	-6.65900	-23.21100	-28.70400
H	-5.70100	-23.67100	-27.62600
O	-3.17000	-32.96800	-20.87700
H	-2.81500	-32.74100	-20.01700
H	-2.52300	-32.63700	-21.49900
O	2.56000	-29.84700	-30.17200
H	2.42100	-29.65600	-31.10000
H	2.20300	-29.08500	-29.71500
O	-9.27600	-35.86100	-24.09200
H	-8.63600	-36.00200	-24.79000
H	-10.10800	-36.14700	-24.46800
O	2.58600	-27.77900	-25.16100
H	3.02000	-27.87100	-24.31200
H	3.30000	-27.80300	-25.79900
O	-8.50000	-26.56100	-27.97900
H	-7.70600	-27.05600	-28.18000
H	-9.21100	-27.08900	-28.34200
O	-3.68100	-24.17900	-23.62700
H	-3.62700	-23.61000	-22.86000
H	-4.54900	-24.00900	-23.99200
O	-10.80100	-34.47700	-22.14500
H	-10.12900	-34.68500	-22.79400
H	-11.62800	-34.67500	-22.58400
O	3.44800	-27.73700	-22.62700
H	3.64200	-26.83200	-22.38100

H	3.34800	-28.19600	-21.79400
O	-4.89800	-37.85100	-20.12800
H	-5.54600	-37.15200	-20.22200
H	-4.12800	-37.41700	-19.76100
O	-1.49000	-27.58600	-17.05300
H	-1.64200	-27.81500	-17.97000
H	-1.50000	-26.62900	-17.04000
O	-4.72200	-30.01300	-22.06900
H	-3.79300	-29.86200	-22.24200
H	-5.14300	-29.92400	-22.92400
O	-5.92300	-25.98800	-24.29900
H	-6.06200	-26.35600	-23.42600
H	-6.71200	-25.47300	-24.47000
O	-5.32800	-20.88700	-32.95600
H	-5.15300	-21.35800	-32.14200
H	-6.01500	-20.26000	-32.72800
O	-4.37300	-26.61000	-26.91000
H	-4.70800	-26.64900	-26.01400
H	-4.87200	-27.27500	-27.38300
O	-10.39800	-31.20300	-21.38500
H	-10.53600	-31.93500	-20.78400
H	-9.48800	-31.29200	-21.66600
O	-7.77000	-31.49100	-21.89600
H	-7.06000	-32.13100	-21.93800
H	-7.81300	-31.23900	-20.97400
O	0.21900	-24.55400	-18.97600
H	0.16000	-25.23800	-19.64300
H	0.62100	-23.81200	-19.42800
O	-9.05700	-34.50400	-20.07700
H	-9.59300	-34.95500	-19.42500
H	-9.64500	-34.37000	-20.82000
O	-10.76200	-17.73800	-26.18200
H	-10.52200	-17.10600	-25.50400
H	-11.60500	-18.08900	-25.89600
O	0.22100	-26.59000	-20.74400
H	-0.66700	-26.92700	-20.62200
H	0.79000	-27.32200	-20.50700
O	-13.32300	-35.36900	-22.99100
H	-13.40700	-35.97600	-23.72600
H	-14.14500	-34.87800	-22.99100
O	-6.24900	-21.93000	-23.77800
H	-6.32200	-21.12400	-23.26600
H	-6.69200	-21.73100	-24.60300
O	-6.46000	-22.69300	-30.26800
H	-7.24400	-22.15100	-30.17400
H	-5.76100	-22.17500	-29.86800
O	-3.15100	-36.88300	-27.62400
H	-2.65400	-37.63600	-27.94400
H	-3.92200	-36.84700	-28.19000
O	4.70200	-31.17900	-25.98300
H	4.69100	-30.29700	-26.35300
H	3.83400	-31.53200	-26.17700
O	-3.79000	-22.03100	-21.62200
H	-3.07700	-21.39700	-21.69500
H	-4.28000	-21.93500	-22.43800
O	-2.06800	-28.12600	-30.05100
H	-3.00200	-28.00600	-30.22000
H	-1.89000	-29.02900	-30.31500
O	-6.56700	-34.74200	-27.89700
H	-6.93700	-35.29400	-27.20900
H	-6.07100	-35.34600	-28.45000
O	1.32000	-32.28400	-29.51600
H	1.78600	-31.45200	-29.59000
H	1.77000	-32.75000	-28.81100
O	-8.73800	-24.28200	-20.80200
H	-8.03700	-23.64800	-20.64900
H	-9.20900	-23.94000	-21.56200
O	-0.61300	-25.94000	-32.51700
H	-0.33700	-26.05400	-31.60800
H	-1.48200	-26.33800	-32.55600
O	-0.70900	-39.91600	-25.47700
H	-1.62300	-39.68600	-25.30800
H	-0.28000	-39.83200	-24.62500
O	-0.93800	-31.72700	-22.97700
H	-1.59100	-31.48000	-23.63100
H	-0.56000	-32.54100	-23.31000
O	-3.97700	-21.69100	-28.87400
H	-3.79500	-22.50900	-28.41100
H	-3.15400	-21.47500	-29.31100
O	-3.64300	-22.76500	-31.61200
H	-3.11400	-23.27500	-32.22500
H	-3.38000	-23.08000	-30.74800
O	-4.88900	-30.98500	-16.49000
H	-5.56500	-31.08600	-15.82000
H	-4.06700	-31.14100	-16.02600
O	-13.14200	-24.74800	-23.86900
H	-12.55600	-25.49400	-23.73600
H	-13.33900	-24.43800	-22.98500
O	-10.28800	-21.71300	-28.85200
H	-10.95700	-21.22800	-29.33600
H	-10.42600	-21.46600	-27.93800
O	-18.55000	-32.17800	-25.14800
H	-18.51100	-32.71100	-24.35500
H	-19.05600	-32.70400	-25.76700
O	-6.14700	-19.67900	-22.25400
H	-5.59500	-19.51600	-21.48900
H	-5.80700	-19.08800	-22.92600
O	-11.17100	-37.33100	-21.65200
H	-11.10700	-36.47000	-22.06400
H	-11.23400	-37.94400	-22.38500
O	-11.69500	-35.25900	-18.00500
H	-11.06800	-35.64100	-17.39100
H	-12.55300	-35.47700	-17.63900
O	-1.29600	-24.85300	-16.39900
H	-0.75200	-24.24000	-16.89400
H	-1.87700	-24.29500	-15.88300
O	-3.01000	-21.73000	-25.26800
H	-3.13500	-22.53200	-25.77700
H	-2.35200	-21.23600	-25.75600
O	-11.38900	-35.37600	-26.09000
H	-12.11100	-34.76200	-26.22900
H	-10.60000	-34.84000	-26.16800
O	-0.94700	-23.93000	-23.64000
H	-1.89400	-24.04900	-23.71200
H	-0.59900	-24.81700	-23.55700
O	-8.58700	-38.04000	-19.81600
H	-8.06300	-37.79400	-19.05300
H	-8.80600	-37.20800	-20.23600

O	-3.94200	-24.13400	-27.43900
H	-4.09000	-25.06300	-27.25900
H	-3.11700	-24.11200	-27.92400
O	-0.33900	-23.31100	-26.35300
H	0.58600	-23.51700	-26.49000
H	-0.50700	-23.56000	-25.44400
O	-7.38200	-37.09900	-22.13100
H	-7.72200	-37.89800	-21.72900
H	-8.08800	-36.79900	-22.70300
O	-6.73000	-40.01400	-23.67800
H	-5.90700	-40.48800	-23.80000
H	-6.48600	-39.23500	-23.17800
O	-9.20600	-33.32200	-25.86900
H	-9.35200	-33.00800	-24.97700
H	-9.68400	-32.70300	-26.42200
O	-7.34600	-36.33200	-25.82100
H	-7.36000	-37.22800	-26.15800
H	-6.62900	-36.32500	-25.18700
O	-5.49400	-35.89800	-23.77600
H	-5.75200	-34.97600	-23.77700
H	-6.02500	-36.29500	-23.08600
O	-0.54900	-22.79300	-30.99700
H	-0.37600	-23.35800	-31.74900
H	-0.05600	-21.99300	-31.17900
O	-14.73200	-30.78100	-22.93000
H	-15.27600	-30.00500	-22.79800
H	-13.85400	-30.43400	-23.08800
O	5.30500	-30.02900	-29.37800
H	5.74800	-30.77900	-29.77600
H	4.42700	-30.03800	-29.76000
O	0.43800	-23.47600	-33.89700
H	1.13300	-22.85600	-33.67800
H	0.78100	-24.32700	-33.62200
O	-13.94500	-31.65400	-20.03700
H	-14.68700	-31.50600	-19.45100
H	-14.26600	-31.39400	-20.90100
O	2.33700	-26.02600	-32.36800
H	2.86300	-25.68800	-33.09300
H	2.25000	-26.96200	-32.55200
O	-6.00100	-22.11300	-20.23900
H	-5.69200	-22.35800	-19.36700
H	-5.20300	-21.90800	-20.72700
O	-5.97900	-19.95900	-28.00900
H	-6.41800	-20.45100	-27.31500
H	-5.22800	-20.50200	-28.25000
O	-13.49900	-23.76800	-28.00600
H	-12.67300	-24.06800	-28.38600
H	-13.85000	-24.53700	-27.55700
O	-8.53100	-24.41500	-25.93700
H	-9.15300	-25.07800	-26.23600
H	-7.86100	-24.38900	-26.61900
O	-15.29800	-25.84900	-27.04700
H	-16.13500	-26.27700	-27.22900
H	-15.32600	-25.65600	-26.11000
O	-8.45900	-35.93700	-16.34300
H	-8.41000	-35.06100	-16.72500
H	-7.91600	-36.47900	-16.91600
O	2.02700	-25.39300	-17.06300
H	1.23600	-25.41100	-17.60300
H	1.79000	-24.85400	-16.30800
O	2.14700	-28.54400	-19.75000
H	2.19600	-28.70900	-18.80800
H	2.03800	-29.41100	-20.14000
O	-8.97000	-25.86500	-32.46100
H	-8.82100	-25.18100	-33.11400
H	-8.16300	-25.88400	-31.94600
O	-0.56300	-32.56100	-18.43400
H	0.33800	-32.70000	-18.72600
H	-0.48800	-32.39500	-17.49400
O	-6.22900	-35.18400	-19.58900
H	-7.09800	-34.89100	-19.86000
H	-5.99300	-34.60000	-18.86800
O	3.64800	-26.12400	-19.50600
H	3.35700	-25.76100	-18.66900
H	3.03600	-26.83700	-19.68400
O	4.84800	-28.55900	-26.98500
H	5.68300	-28.12000	-26.82200
H	4.98400	-29.03500	-27.80500
O	1.89700	-31.00100	-21.03800
H	2.37300	-30.80800	-21.84600
H	0.97400	-30.94500	-21.28300
O	-3.52500	-38.96600	-22.07200
H	-3.00700	-38.23000	-22.39700
H	-3.87900	-38.66000	-21.23700
O	-3.27400	-42.54600	-26.55200
H	-2.99300	-43.21400	-25.92600
H	-2.48900	-42.35300	-27.06500
O	-5.47000	-23.37000	-34.98900
H	-6.04300	-22.75100	-35.44200
H	-4.89700	-22.82000	-34.45600
O	0.59700	-34.12400	-22.80500
H	1.54900	-34.02400	-22.78400
H	0.42800	-34.91600	-22.29500
O	-1.38100	-37.03900	-22.61900
H	-1.97600	-36.75800	-23.31400
H	-1.66600	-36.54900	-21.84700
O	-2.25100	-23.08600	-33.85900
H	-1.31700	-23.15200	-33.65500
H	-2.33800	-22.25100	-34.31700
O	0.37800	-23.77000	-36.89800
H	0.30000	-24.10400	-36.00500
H	1.19900	-23.27800	-36.89900
O	3.07600	-30.90400	-23.69100
H	3.04400	-31.84200	-23.50500
H	3.68600	-30.82500	-24.42400
O	3.22600	-34.11200	-23.64500
H	2.70900	-34.89700	-23.83200
H	4.11000	-34.44100	-23.48000
O	0.88700	-40.36800	-27.68500
H	0.68900	-40.67700	-26.80100
H	1.49700	-39.64200	-27.55500
O	8.89400	-29.84200	-24.55000
H	9.62400	-30.10300	-23.98900
H	8.28400	-30.57900	-24.51000
O	6.97500	-31.70100	-23.95300
H	6.24900	-31.83600	-24.56100
H	6.56000	-31.39300	-23.14700
O	-5.20200	-27.98200	-16.18800

H	-4.28100	-27.80400	-15.99400
H	-5.23700	-28.92700	-16.34000
O	-12.20500	-30.07200	-23.54600
H	-11.98700	-29.14000	-23.52700
H	-11.79300	-30.43100	-22.76000
O	-7.33300	-34.49900	-14.15500
H	-7.43300	-33.54900	-14.22400
H	-7.55100	-34.82700	-15.02800
O	-16.11500	-29.16800	-25.29300
H	-16.80800	-29.58300	-25.80600
H	-15.36700	-29.12600	-25.89000
O	-1.52800	-38.97700	-28.22600
H	-1.02800	-38.86500	-29.03400
H	-0.88400	-39.28100	-27.58500
O	-7.47000	-31.06900	-15.33900
H	-7.95300	-30.26900	-15.13200
H	-7.38300	-31.05800	-16.29200
O	-14.20100	-29.23600	-27.41700
H	-14.22400	-29.13700	-28.36800
H	-13.89100	-28.39000	-27.09500
O	-11.54200	-27.17900	-23.30200
H	-10.58800	-27.21000	-23.36900
H	-11.74100	-27.64000	-22.48700
O	-10.95200	-28.60800	-20.73500
H	-10.13300	-28.11400	-20.71100
H	-10.67900	-29.52500	-20.76300
O	2.15600	-36.19300	-27.65700
H	2.93200	-35.70300	-27.38400
H	1.43100	-35.76700	-27.19900
O	4.59300	-35.48000	-26.59900
H	5.18800	-35.72000	-27.31000
H	4.66600	-36.19900	-25.97200
O	-13.18700	-21.63700	-26.28500
H	-13.32200	-22.39700	-26.85100
H	-13.60400	-20.91300	-26.75200
O	-1.22200	-30.70200	-31.15700
H	-1.27800	-30.24500	-31.99600
H	-0.66900	-31.46300	-31.33500
O	2.58900	-34.91300	-30.33200
H	2.33300	-35.31300	-29.50100
H	1.96400	-34.19900	-30.45700
O	6.90000	-32.17300	-27.82500
H	6.34000	-32.10400	-27.05200
H	6.29500	-32.34900	-28.54500
O	-10.03000	-20.74000	-26.07300
H	-10.14700	-19.79500	-26.17500
H	-10.85100	-21.04200	-25.68400
O	-0.14300	-26.01500	-35.22700
H	0.69200	-26.44700	-35.41000
H	-0.30400	-26.18600	-34.30000
O	-0.48800	-29.51100	-33.86200
H	-0.54300	-29.67700	-34.80300
H	-1.11300	-28.80400	-33.70600
O	-15.69900	-34.27500	-20.03600
H	-15.05300	-34.78500	-19.54700
H	-15.90700	-33.53800	-19.46100
O	-3.17300	-27.20700	-14.53600
H	-2.30400	-27.03300	-14.89800
H	-3.65300	-26.38700	-14.65900
O	-9.00500	-20.08700	-22.85700
H	-8.10200	-20.05500	-22.54100
H	-9.23100	-21.01700	-22.84300
O	-7.24900	-21.33400	-26.19600
H	-8.20000	-21.33400	-26.31000
H	-6.94600	-22.08000	-26.71300
O	-11.35900	-37.12800	-15.93800
H	-10.49600	-37.48400	-15.72800
H	-11.93300	-37.46700	-15.25100
O	8.48600	-15.49800	-25.13200
H	9.12400	-15.61700	-25.83600
H	8.36800	-14.55000	-25.07300
O	5.29500	-15.54500	-25.19600
H	4.95700	-15.66100	-26.08400
H	6.16400	-15.94600	-25.21600
O	1.15300	-13.69100	-29.20600
H	0.59000	-13.78200	-29.97400
H	1.03900	-14.51100	-28.72600
O	5.77700	-14.39100	-19.23700
H	5.76600	-14.78600	-20.10900
H	5.93900	-15.12500	-18.64400
O	-1.30000	-12.19600	-26.36800
H	-0.50800	-12.53700	-26.78300
H	-1.06000	-12.08700	-25.44700
O	6.73300	-10.02400	-33.89500
H	6.08200	-9.43900	-34.28100
H	7.52800	-9.49400	-33.83500
O	0.65900	-12.93500	-33.49600
H	1.43200	-13.48300	-33.63500
H	1.01200	-12.07100	-33.28400
O	2.49700	-11.60800	-20.28900
H	2.20600	-10.70300	-20.40500
H	3.13300	-11.56600	-19.57500
O	3.42300	-14.23000	-23.69900
H	4.01900	-14.45400	-24.41300
H	3.48100	-14.97200	-23.09700
O	2.99600	-16.77600	-35.86400
H	3.24800	-17.29800	-36.62600
H	2.05800	-16.94000	-35.76100
O	8.04000	-17.77300	-28.05700
H	8.82600	-18.19100	-28.40800
H	8.30400	-16.86800	-27.88800
O	8.21100	-12.33500	-33.35800
H	7.57600	-12.26200	-32.64600
H	7.88100	-11.74600	-34.03600
O	13.44100	-17.83500	-27.15300
H	14.17300	-18.40500	-26.91900
H	12.68500	-18.22300	-26.71300
O	2.71500	-14.87300	-33.66200
H	2.59900	-15.54900	-32.99300
H	2.82500	-15.36000	-34.47800
O	5.05800	-15.41200	-29.95700
H	4.69400	-15.93900	-29.24600
H	5.91500	-15.80200	-30.13000
O	10.96300	-24.12300	-22.69400
H	10.63200	-23.25300	-22.91800
H	10.36800	-24.43900	-22.01400
O	4.69900	-20.38300	-34.96100
H	4.86600	-19.51500	-34.59400

H	4.50900	-20.22100	-35.88500
O	7.45600	-11.92200	-23.29400
H	7.15700	-11.01900	-23.40300
H	7.11200	-12.18800	-22.44200
O	0.90800	-15.47000	-27.26100
H	-0.01700	-15.60300	-27.05300
H	1.15600	-14.69100	-26.76400
O	10.85600	-19.40300	-25.61400
H	9.94800	-19.47200	-25.90900
H	10.80500	-18.92600	-24.78600
O	4.67800	-17.92800	-33.97600
H	4.55800	-17.48200	-33.13800
H	3.98300	-17.57900	-34.53500
O	-1.73100	-15.82700	-26.42600
H	-1.60200	-15.57300	-25.51200
H	-2.41200	-15.23400	-26.74600
O	2.43900	-5.15700	-30.32700
H	2.36100	-4.20600	-30.24800
H	1.83500	-5.50300	-29.67000
O	4.93000	-17.98400	-17.67100
H	4.15400	-17.42500	-17.68100
H	5.56900	-17.49900	-17.14900
O	12.72200	-21.20200	-23.83700
H	12.08000	-20.90800	-24.48300
H	12.21800	-21.31600	-23.03100
O	10.37600	-17.45500	-33.14800
H	11.28800	-17.22300	-33.32300
H	9.86700	-16.87200	-33.71100
O	3.18500	-15.17600	-18.10900
H	3.92600	-14.63700	-18.38600
H	2.47900	-14.54900	-17.95200
O	8.66000	-13.03300	-19.51200
H	9.06900	-13.47500	-20.25700
H	7.93800	-13.60800	-19.25900
O	5.01400	-18.09600	-22.13900
H	5.86000	-17.67200	-21.99500
H	4.95300	-18.75200	-21.44500
O	8.98300	-12.47300	-25.75700
H	8.35400	-12.30800	-26.45900
H	8.59600	-12.05900	-24.98600
O	12.86200	-11.53700	-28.40200
H	12.17700	-11.15700	-28.95300
H	12.94700	-10.92800	-27.66900
O	8.16300	-20.43500	-20.48000
H	7.92000	-19.68100	-21.01700
H	8.43900	-20.05700	-19.64600
O	10.11700	-13.89100	-21.80300
H	10.32900	-13.24800	-22.47900
H	10.07000	-14.72500	-22.27100
O	7.72000	-21.28000	-24.89900
H	6.85800	-20.98100	-24.60700
H	7.74600	-21.06300	-25.83100
O	4.53600	-12.73500	-28.49200
H	5.00000	-12.45700	-29.28100
H	4.27200	-13.63700	-28.67400
O	1.42900	-13.18800	-25.55400
H	1.99900	-12.76000	-26.19200
H	2.01900	-13.47000	-24.85500
O	3.47300	-16.61300	-31.68300
H	4.17500	-16.16100	-31.21500
H	3.14100	-17.25500	-31.05500
O	1.92100	-18.02200	-17.30800
H	1.29100	-17.34000	-17.07400
H	1.81500	-18.13400	-18.25200
O	-2.78400	-18.35500	-26.38100
H	-3.57500	-18.09900	-26.85700
H	-2.29300	-17.54000	-26.27600
O	11.23200	-11.01400	-23.63100
H	12.05400	-11.41600	-23.91200
H	11.22800	-11.11700	-22.67900
O	-0.83300	-17.99000	-29.42300
H	-1.63000	-17.87800	-28.90400
H	-1.06000	-17.65200	-30.28900
O	4.99800	-11.46600	-21.47400
H	5.06200	-11.00400	-20.63900
H	4.28900	-12.09600	-21.34600
O	-4.50100	-18.10200	-23.95000
H	-4.25000	-17.21700	-23.68400
H	-3.96500	-18.28100	-24.72200
O	19.32300	-14.67000	-27.28200
H	18.56900	-15.24000	-27.43800
H	19.45700	-14.70400	-26.33500
O	4.71200	-9.79900	-31.60700
H	4.57700	-8.95400	-31.17800
H	5.25300	-9.59700	-32.37000
O	1.19900	-23.72000	-14.94500
H	0.80600	-24.27300	-14.27000
H	2.01600	-23.40900	-14.55400
O	-1.83500	-12.28100	-31.03400
H	-1.32200	-11.48700	-30.88300
H	-1.18200	-12.97900	-31.10000
O	-0.02500	-9.16500	-28.95100
H	0.15100	-8.24300	-29.13900
H	0.77000	-9.47900	-28.52200
O	4.11600	-22.30400	-17.35300
H	4.85700	-21.70300	-17.43300
H	4.34800	-23.04600	-17.91100
O	-0.74000	-17.11800	-19.07500
H	-0.85500	-18.02000	-18.77500
H	-1.59600	-16.87200	-19.42700
O	1.09800	-7.64700	-22.95000
H	1.64900	-7.77500	-23.72300
H	0.25900	-7.34800	-23.30100
O	4.89500	-11.38200	-18.60600
H	5.42700	-12.14300	-18.83600
H	4.36600	-11.67500	-17.86400
O	8.06200	-26.27600	-23.07700
H	7.71800	-25.38600	-23.15300
H	8.43000	-26.46600	-23.94000
O	-3.87000	-13.95300	-30.25800
H	-3.03200	-13.54300	-30.47400
H	-3.69200	-14.89300	-30.28800
O	8.02700	-16.28500	-34.56900
H	7.51100	-15.51500	-34.33100
H	7.75400	-16.95900	-33.94600
O	5.61100	-21.44000	-29.51100
H	4.75600	-21.82900	-29.69000
H	6.02400	-21.36100	-30.37100

O	7.14100	-23.90500	-25.11200
H	6.63800	-23.94200	-25.92500
H	7.43400	-22.99600	-25.05200
O	3.38600	-23.82200	-26.10500
H	4.25100	-24.01700	-26.46500
H	3.45300	-24.05100	-25.17800
O	13.00000	-13.53200	-32.63400
H	12.22300	-13.87100	-33.07900
H	13.67900	-13.52700	-33.30900
O	7.81500	-18.41200	-32.74300
H	7.51000	-18.09800	-31.89200
H	8.76800	-18.33400	-32.70100
O	6.26000	-12.25300	-31.09400
H	5.73200	-12.98800	-31.40700
H	5.75900	-11.47600	-31.34000
O	11.23900	-14.05800	-25.97700
H	10.58200	-13.36900	-25.88900
H	12.04300	-13.66900	-25.63400
O	0.99200	-21.59100	-28.73300
H	1.56600	-21.81400	-29.46600
H	1.57900	-21.52200	-27.98100
O	10.05000	-15.70400	-27.92700
H	10.56300	-15.07100	-27.42500
H	10.70000	-16.30400	-28.29300
O	1.31900	-19.56100	-34.71400
H	0.48200	-19.22300	-34.39300
H	1.17900	-19.69600	-35.65100
O	3.29400	-24.71700	-30.00600
H	2.64100	-25.03200	-30.63000
H	3.25200	-25.34000	-29.28000
O	4.40500	-14.43500	-38.09100
H	3.49800	-14.66600	-37.89200
H	4.90200	-15.23200	-37.90300
O	7.31200	-5.72600	-35.17100
H	6.63800	-5.42200	-34.56200
H	7.69900	-4.92300	-35.52000
O	6.52800	-18.22900	-25.68100
H	5.61000	-18.00800	-25.83900
H	6.94400	-18.15400	-26.54000
O	7.31100	-16.83000	-30.55100
H	8.05900	-16.31800	-30.86000
H	7.58000	-17.15200	-29.69100
O	5.58400	-14.33400	-33.56800
H	4.67500	-14.61800	-33.47100
H	5.61400	-13.91400	-34.42800
O	10.81600	-10.72300	-29.79900
H	10.36200	-11.56400	-29.74300
H	10.18500	-10.08700	-29.46200
O	9.75900	-19.79400	-28.56900
H	9.02100	-20.30600	-28.23800
H	10.01200	-20.23400	-29.38100
O	9.25900	-8.88600	-28.86400
H	9.55200	-8.32500	-29.58300
H	9.76900	-8.59300	-28.10900
O	9.86000	-27.01700	-27.97500
H	10.16600	-27.87700	-28.26200
H	10.58600	-26.42500	-28.17500
O	-3.84700	-16.61400	-29.58700
H	-4.04500	-17.20700	-30.31200
H	-4.40300	-16.91800	-28.86900
O	4.13200	-16.72100	-27.46700
H	4.18700	-17.67400	-27.54300
H	3.32200	-16.56500	-26.98300
O	5.28200	-12.79300	-36.22900
H	4.84400	-13.51000	-36.68900
H	6.02300	-12.56600	-36.79100
O	2.07200	-18.10300	-29.70800
H	1.25900	-17.80400	-29.30200
H	2.56800	-18.49800	-28.99100
O	4.22300	-8.10800	-27.77500
H	4.18000	-7.88200	-28.70400
H	5.13900	-8.34100	-27.62600
O	7.66900	-17.63900	-20.87700
H	8.02400	-17.41200	-20.01700
H	8.31600	-17.30700	-21.49900
O	13.39900	-14.51700	-30.17200
H	13.26100	-14.32700	-31.10000
H	13.04300	-13.75600	-29.71500
O	1.56300	-20.53200	-24.09200
H	2.20300	-20.67300	-24.79000
H	0.73100	-20.81800	-24.46800
O	13.42600	-12.45000	-25.16100
H	13.86000	-12.54200	-24.31200
H	14.13900	-12.47400	-25.79900
O	2.33900	-11.23100	-27.97900
H	3.13300	-11.72600	-28.18000
H	1.62800	-11.76000	-28.34200
O	7.15900	-8.85000	-23.62700
H	7.21200	-8.28100	-22.86000
H	6.29000	-8.67900	-23.99200
O	-1.35300	-17.08700	-31.90300
H	-2.28300	-17.14900	-31.68600
H	-1.25700	-17.61000	-32.69900
O	-1.43400	-20.56200	-29.31500
H	-1.17300	-19.64700	-29.42400
H	-0.61200	-21.02900	-29.16500
O	0.03800	-19.14800	-22.14500
H	0.71100	-19.35600	-22.79400
H	-0.78900	-19.34600	-22.58400
O	14.28700	-12.40700	-22.62700
H	14.48100	-11.50300	-22.38100
H	14.18700	-12.86700	-21.79400
O	5.94100	-22.52200	-20.12800
H	5.29400	-21.82300	-20.22200
H	6.71100	-22.08800	-19.76100
O	9.34900	-12.25700	-17.05300
H	9.19700	-12.48600	-17.97000
H	9.33900	-11.30000	-17.04000
O	6.11700	-14.68400	-22.06900
H	7.04700	-14.53200	-22.24200
H	5.69700	-14.59500	-22.92400
O	4.91600	-10.65900	-24.29900
H	4.77700	-11.02700	-23.42600
H	4.12800	-10.14400	-24.47000
O	5.51100	-5.55800	-32.95600
H	5.68600	-6.02900	-32.14200
H	4.82400	-4.93100	-32.72800
O	6.46700	-11.28000	-26.91000

H	6.13200	-11.32000	-26.01400
H	5.96700	-11.94600	-27.38300
O	0.44100	-15.87400	-21.38500
H	0.30300	-16.60600	-20.78400
H	1.35200	-15.96300	-21.66600
O	9.40300	-13.93500	-36.63600
H	8.78600	-14.66000	-36.73300
H	8.93400	-13.17400	-36.98000
O	3.06900	-16.16200	-21.89600
H	3.77900	-16.80200	-21.93800
H	3.02600	-15.91000	-20.97400
O	11.05800	-9.22500	-18.97600
H	10.99900	-9.90900	-19.64300
H	11.46000	-8.48300	-19.42800
O	1.78200	-19.17500	-20.07700
H	1.24600	-19.62600	-19.42500
H	1.19400	-19.04000	-20.82000
O	0.07700	-2.40900	-26.18200
H	0.31700	-1.77700	-25.50400
H	-0.76600	-2.76000	-25.89600
O	1.19300	-19.71600	-37.51400
H	0.31600	-19.83700	-37.87800
H	1.66800	-20.50800	-37.76600
O	11.06000	-11.26100	-20.74400
H	10.17300	-11.59800	-20.62200
H	11.63000	-11.99300	-20.50700
O	-7.28700	-14.92800	-27.42000
H	-6.60000	-15.24200	-28.00800
H	-7.44200	-14.02400	-27.69300
O	-2.48400	-20.03900	-22.99100
H	-2.56800	-20.64700	-23.72600
H	-3.30500	-19.54900	-22.99100
O	4.59000	-6.60100	-23.77800
H	4.51700	-5.79500	-23.26600
H	4.14800	-6.40200	-24.60300
O	4.37900	-7.36400	-30.26800
H	3.59600	-6.82100	-30.17400
H	5.07800	-6.84600	-29.86800
O	7.68900	-21.55400	-27.62400
H	8.18600	-22.30700	-27.94400
H	6.91700	-21.51800	-28.19000
O	15.54200	-15.85000	-25.98300
H	15.53100	-14.96800	-26.35300
H	14.67300	-16.20300	-26.17700
O	8.77200	-12.79700	-30.05100
H	7.83700	-12.67700	-30.22000
H	8.94900	-13.70000	-30.31500
O	4.27200	-19.41200	-27.89700
H	3.90200	-19.96500	-27.20900
H	4.76800	-20.01700	-28.45000
O	12.15900	-16.95500	-29.51600
H	12.62500	-16.12200	-29.59000
H	12.61000	-17.42100	-28.81100
O	2.10100	-8.95300	-20.80200
H	2.80200	-8.31900	-20.64900
H	1.63100	-8.61100	-21.56200
O	10.22700	-10.61100	-32.51700
H	10.50200	-10.72500	-31.60800
H	9.35700	-11.00900	-32.55600
O	10.13100	-24.58700	-25.47700
H	9.21700	-24.35700	-25.30800
H	10.56000	-24.50300	-24.62500
O	9.90100	-16.39800	-22.97700
H	9.24800	-16.15100	-23.63100
H	10.27900	-17.21200	-23.31000
O	6.86200	-6.36200	-28.87400
H	7.04400	-7.18000	-28.41100
H	7.68500	-6.14500	-29.31100
O	7.19600	-7.43500	-31.61200
H	7.72500	-7.94600	-32.22500
H	7.45900	-7.75100	-30.74800
O	5.95000	-15.65600	-16.49000
H	5.27400	-15.75700	-15.82000
H	6.77300	-15.81200	-16.02600
O	0.55200	-6.38400	-28.85200
H	-0.11700	-5.89900	-29.33600
H	0.41300	-6.13700	-27.93800
O	-7.71000	-16.84800	-25.14800
H	-7.67200	-17.38200	-24.35500
H	-8.21600	-17.37500	-25.76700
O	4.69200	-4.34900	-22.25400
H	5.24400	-4.18600	-21.48900
H	5.03300	-3.75900	-22.92600
O	-0.33100	-22.00200	-21.65200
H	-0.26700	-21.14000	-22.06400
H	-0.39500	-22.61500	-22.38500
O	-0.85600	-19.93000	-18.00500
H	-0.22900	-20.31200	-17.39100
H	-1.71300	-20.14800	-17.63900
O	9.54300	-9.52300	-16.39900
H	10.08700	-8.91000	-16.89400
H	8.96200	-8.96500	-15.88300
O	-4.08000	-18.12200	-32.07300
H	-3.78400	-19.03100	-32.12600
H	-4.98100	-18.14300	-32.39500
O	7.82900	-6.40100	-25.26800
H	7.70400	-7.20200	-25.77700
H	8.48700	-5.90700	-25.75600
O	-0.55000	-20.04600	-26.09000
H	-1.27100	-19.43300	-26.22900
H	0.23900	-19.51100	-26.16800
O	9.89300	-8.60000	-23.64000
H	8.94600	-8.72000	-23.71200
H	10.24100	-9.48800	-23.55700
O	2.25300	-22.71100	-19.81600
H	2.77600	-22.46500	-19.05300
H	2.03400	-21.87900	-20.23600
O	-2.35100	-20.44100	-31.94900
H	-2.67900	-21.32100	-32.13100
H	-1.99700	-20.49600	-31.06100
O	6.89700	-8.80500	-27.43900
H	6.75000	-9.73300	-27.25900
H	7.72300	-8.78300	-27.92400
O	0.76300	-20.15300	-31.58100
H	1.10300	-19.62500	-32.30300
H	1.21100	-19.81500	-30.80500
O	10.50100	-7.98200	-26.35300
H	11.42500	-8.18800	-26.49000

H	10.33200	-8.23100	-25.44400
O	3.45800	-21.77000	-22.13100
H	3.11700	-22.56900	-21.72900
H	2.75200	-21.46900	-22.70300
O	4.10900	-24.68500	-23.67800
H	4.93200	-25.15900	-23.80000
H	4.35300	-23.90600	-23.17800
O	2.75000	-21.65600	-33.38200
H	3.47100	-21.27800	-33.88600
H	2.12700	-20.93700	-33.27900
O	6.89300	-20.95100	-32.44600
H	7.11000	-20.06900	-32.74700
H	6.41300	-21.34300	-33.17600
O	1.63300	-17.99300	-25.86900
H	1.48800	-17.67900	-24.97700
H	1.15600	-17.37400	-26.42200
O	3.49300	-21.00300	-25.82100
H	3.47900	-21.89800	-26.15800
H	4.21000	-20.99500	-25.18700
O	5.34600	-20.56900	-23.77600
H	5.08700	-19.64700	-23.77700
H	4.81400	-20.96600	-23.08600
O	10.29000	-7.46400	-30.99700
H	10.46400	-8.02900	-31.74900
H	10.78300	-6.66400	-31.17900
O	-3.89200	-15.45200	-22.93000
H	-4.43700	-14.67600	-22.79800
H	-3.01400	-15.10500	-23.08800
O	2.89700	-22.12100	-30.55200
H	3.01800	-23.04900	-30.35300
H	2.60700	-22.10700	-31.46400
O	5.76600	-24.43600	-27.41600
H	5.78800	-24.37200	-28.37000
H	6.18300	-25.27500	-27.22200
O	16.14400	-14.70000	-29.37800
H	16.58700	-15.44900	-29.77600
H	15.26700	-14.70900	-29.76000
O	11.27800	-8.14700	-33.89700
H	11.97300	-7.52600	-33.67800
H	11.62000	-8.99700	-33.62200
O	-3.10600	-16.32500	-20.03700
H	-3.84700	-16.17700	-19.45100
H	-3.42600	-16.06400	-20.90100
O	13.17700	-10.69700	-32.36800
H	13.70200	-10.35800	-33.09300
H	13.08900	-11.63200	-32.55200
O	4.86100	-4.63000	-28.00900
H	4.42100	-5.12200	-27.31500
H	5.61200	-5.17300	-28.25000
O	-2.66000	-8.43900	-28.00600
H	-1.83400	-8.73800	-28.38600
H	-3.01000	-9.20800	-27.55700
O	2.30800	-9.08600	-25.93700
H	1.68600	-9.74900	-26.23600
H	2.97900	-9.06000	-26.61900
O	-4.45900	-10.52000	-27.04700
H	-5.29600	-10.94800	-27.22900
H	-4.48700	-10.32700	-26.11000
O	2.38100	-20.60800	-16.34300
H	2.43000	-19.73200	-16.72500
H	2.92300	-21.15000	-16.91600
O	12.86600	-10.06400	-17.06300
H	12.07600	-10.08100	-17.60300
H	12.63000	-9.52500	-16.30800
O	12.98600	-13.21500	-19.75000
H	13.03600	-13.38000	-18.80800
H	12.87700	-14.08200	-20.14000
O	1.87000	-10.53600	-32.46100
H	2.01800	-9.85200	-33.11400
H	2.67600	-10.55500	-31.94600
O	10.27600	-17.23100	-18.43400
H	11.17700	-17.37100	-18.72600
H	10.35100	-17.06600	-17.49400
O	4.61100	-19.85500	-19.58900
H	3.74100	-19.56100	-19.86000
H	4.84700	-19.27100	-18.86800
O	14.48800	-10.79500	-19.50600
H	14.19600	-10.43200	-18.66900
H	13.87500	-11.50800	-19.68400
O	15.68700	-13.23000	-26.98500
H	16.52200	-12.79100	-26.82200
H	15.82300	-13.70600	-27.80500
O	12.73700	-15.67200	-21.03800
H	13.21200	-15.47900	-21.84600
H	11.81300	-15.61600	-21.28300
O	-0.37500	-14.50900	-31.45500
H	-0.62100	-15.41300	-31.25700
H	-0.20600	-14.50700	-32.39700
O	7.31500	-23.63700	-22.07200
H	7.83300	-22.90100	-22.39700
H	6.96000	-23.33000	-21.23700
O	-1.10600	-18.77600	-33.95600
H	-1.79900	-18.70200	-34.61200
H	-1.35000	-19.54000	-33.43400
O	7.56600	-27.21700	-26.55200
H	7.84600	-27.88400	-25.92600
H	8.35100	-27.02400	-27.06500
O	5.36900	-8.04100	-34.98900
H	4.79700	-7.42200	-35.44200
H	5.94200	-7.49100	-34.45600
O	11.43700	-18.79500	-22.80500
H	12.38800	-18.69500	-22.78400
H	11.26700	-19.58700	-22.29500
O	9.45800	-21.70900	-22.61900
H	8.86300	-21.42900	-23.31400
H	9.17300	-21.22000	-21.84700
O	8.58900	-7.75700	-33.85900
H	9.52200	-7.82300	-33.65500
H	8.50100	-6.92100	-34.31700
O	11.21800	-8.44100	-36.89800
H	11.13900	-8.77500	-36.00500
H	12.03900	-7.94800	-36.89900
O	13.91500	-15.57400	-23.69100
H	13.88300	-16.51300	-23.50500
H	14.52600	-15.49600	-24.42400
O	5.75400	-16.70500	-37.64800
H	6.70000	-16.83400	-37.57900
H	5.37500	-17.44300	-37.17100

O	14.06500	-18.78300	-23.64500
H	13.54900	-19.56700	-23.83200
H	14.94900	-19.11100	-23.48000
O	11.72600	-25.03900	-27.68500
H	11.52800	-25.34800	-26.80100
H	12.33600	-24.31300	-27.55500
O	19.73300	-14.51300	-24.55000
H	20.46300	-14.77400	-23.98900
H	19.12400	-15.25000	-24.51000
O	17.81500	-16.37200	-23.95300
H	17.08800	-16.50600	-24.56100
H	17.39900	-16.06400	-23.14700
O	5.63800	-12.65300	-16.18800
H	6.55800	-12.47500	-15.99400
H	5.60300	-13.59700	-16.34000
O	3.93800	-18.78000	-37.71200
H	3.03100	-19.07600	-37.63600
H	4.21600	-19.07700	-38.57900
O	-5.14400	-17.45900	-27.26200
H	-5.46400	-18.31300	-27.55500
H	-5.69100	-17.24400	-26.50700
O	-1.36600	-14.74300	-23.54600
H	-1.14700	-13.81100	-23.52700
H	-0.95400	-15.10200	-22.76000
O	5.55400	-24.23600	-31.55800
H	4.74400	-24.58100	-31.18200
H	6.24300	-24.54500	-30.96900
O	7.48300	-25.15800	-29.93600
H	8.03600	-24.73900	-29.27700
H	7.72000	-26.08500	-29.89300
O	3.50700	-19.17000	-14.15500
H	3.40600	-18.22000	-14.22400
H	3.28900	-19.49800	-15.02800
O	2.70800	-21.92100	-37.57100
H	3.57000	-21.59600	-37.31200
H	2.89300	-22.70800	-38.08400
O	-5.27500	-13.83900	-25.29300
H	-5.96900	-14.25300	-25.80600
H	-4.52800	-13.79700	-25.89000
O	10.86100	-23.04800	-30.42800
H	10.28100	-22.39000	-30.81000
H	10.59500	-23.87000	-30.83900
O	9.31200	-23.64800	-28.22600
H	9.81200	-23.53600	-29.03400
H	9.95500	-23.95200	-27.58500
O	3.37000	-15.74000	-15.33900
H	2.88700	-14.94000	-15.13200
H	3.45600	-15.72900	-16.29200
O	-3.36200	-13.90700	-27.41700
H	-3.38500	-13.80800	-28.36800
H	-3.05200	-13.06000	-27.09500
O	-0.70300	-11.84900	-23.30200
H	0.25100	-11.88100	-23.36900
H	-0.90200	-12.31000	-22.48700
O	-0.11300	-13.27900	-20.73500
H	0.70600	-12.78500	-20.71100
H	0.16100	-14.19600	-20.76300
O	9.64400	-20.88800	-31.37200
H	8.75600	-20.60300	-31.59000
H	10.05900	-21.04900	-32.21900
O	12.99500	-20.86400	-27.65700
H	13.77100	-20.37400	-27.38400
H	12.27000	-20.43800	-27.19900
O	15.43200	-20.15100	-26.59900
H	16.02700	-20.39100	-27.31000
H	15.50500	-20.87000	-25.97200
O	-2.34800	-6.30700	-26.28500
H	-2.48200	-7.06700	-26.85100
H	-2.76500	-5.58300	-26.75200
O	8.78800	-11.12400	-37.27700
H	9.49200	-10.91900	-36.66100
H	8.06400	-10.55600	-37.01200
O	9.61800	-15.37300	-31.15700
H	9.56100	-14.91600	-31.99600
H	10.17100	-16.13400	-31.33500
O	13.42800	-19.58400	-30.33200
H	13.17200	-19.98400	-29.50100
H	12.80400	-18.86900	-30.45700
O	17.74000	-16.84400	-27.82500
H	17.18000	-16.77500	-27.05200
H	17.13400	-17.02000	-28.54500
O	0.80900	-5.41000	-26.07300
H	0.69200	-4.46600	-26.17500
H	-0.01200	-5.71300	-25.68400
O	10.69700	-10.68500	-35.22700
H	11.53100	-11.11700	-35.41000
H	10.53500	-10.85700	-34.30000
O	10.35100	-14.18200	-33.86200
H	10.29600	-14.34800	-34.80300
H	9.72600	-13.47500	-33.70600
O	-4.85900	-18.94500	-20.03600
H	-4.21400	-19.45600	-19.54700
H	-5.06800	-18.20900	-19.46100
O	7.66600	-11.87700	-14.53600
H	8.53500	-11.70300	-14.89800
H	7.18700	-11.05800	-14.65900
O	3.66100	-24.68100	-34.28300
H	3.64900	-23.73700	-34.12600
H	4.55300	-24.86400	-34.57700
O	1.83500	-4.75800	-22.85700
H	2.73700	-4.72500	-22.54100
H	1.60800	-5.68800	-22.84300
O	3.59000	-6.00500	-26.19600
H	2.63900	-6.00400	-26.31000
H	3.89300	-6.75100	-26.71300
O	7.47600	-28.08700	-30.02900
H	6.70900	-28.64100	-29.88300
H	7.88700	-28.01500	-29.16700
O	-0.52000	-21.79900	-15.93800
H	0.34300	-22.15500	-15.72800
H	-1.09300	-22.13700	-15.25100
O	19.32500	-0.16900	-25.13200
H	19.96300	-0.28800	-25.83600
H	19.20700	0.78000	-25.07300
O	16.13400	-0.21600	-25.19600
H	15.79600	-0.33200	-26.08400
H	17.00300	-0.61700	-25.21600
O	11.99300	1.63800	-29.20600

H	11.42900	1.54700	-29.97400
H	11.87800	0.81800	-28.72600
O	16.61600	0.93800	-19.23700
H	16.60500	0.54300	-20.10900
H	16.77800	0.20400	-18.64400
O	9.53900	3.13300	-26.36800
H	10.33100	2.79200	-26.78300
H	9.77900	3.24200	-25.44700
O	17.57300	5.30500	-33.89500
H	16.92200	5.89000	-34.28100
H	18.36700	5.83500	-33.83500
O	11.49800	2.39400	-33.49600
H	12.27100	1.84700	-33.63500
H	11.85100	3.25800	-33.28400
O	13.33700	3.72100	-20.28900
H	13.04600	4.62600	-20.40500
H	13.97300	3.76300	-19.57500
O	14.26200	1.09900	-23.69900
H	14.85900	0.87500	-24.41300
H	14.32000	0.35800	-23.09700
O	13.83500	-1.44700	-35.86400
H	14.08800	-1.96900	-36.62600
H	12.89700	-1.61000	-35.76100
O	18.87900	-2.44300	-28.05700
H	19.66600	-2.86100	-28.40800
H	19.14300	-1.53900	-27.88800
O	19.05000	2.99400	-33.35800
H	18.41500	3.06700	-32.64600
H	18.72000	3.58300	-34.03600
O	24.28000	-2.50600	-27.15300
H	25.01300	-3.07600	-26.91900
H	23.52400	-2.89400	-26.71300
O	13.55500	0.45600	-33.66200
H	13.43800	-0.22000	-32.99300
H	13.66500	-0.03100	-34.47800
O	15.89800	-0.08300	-29.95700
H	15.53400	-0.61000	-29.24600
H	16.75400	-0.47300	-30.13000
O	21.80300	-8.79400	-22.69400
H	21.47100	-7.92400	-22.91800
H	21.20700	-9.11000	-22.01400
O	15.53800	-5.05400	-34.96100
H	15.70600	-4.18600	-34.59400
H	15.34800	-4.89100	-35.88500
O	18.29600	3.40800	-23.29400
H	17.99600	4.31000	-23.40300
H	17.95100	3.14100	-22.44200
O	11.74700	-0.14100	-27.26100
H	10.82200	-0.27300	-27.05300
H	11.99600	0.63800	-26.76400
O	21.69600	-4.07400	-25.61400
H	20.78700	-4.14300	-25.90900
H	21.64400	-3.59700	-24.78600
O	15.51700	-2.59800	-33.97600
H	15.39700	-2.15300	-33.13800
H	14.82200	-2.25000	-34.53500
O	9.10800	-0.49800	-26.42600
H	9.23700	-0.24400	-25.51200
H	8.42800	0.09500	-26.74600
O	13.27800	10.17200	-30.32700
H	13.20000	11.12300	-30.24800
H	12.67400	9.82700	-29.67000
O	15.77000	-2.65500	-17.67100
H	14.99300	-2.09600	-17.68100
H	16.40900	-2.17000	-17.14900
O	23.56200	-5.87300	-23.83700
H	22.91900	-5.57900	-24.48300
H	23.05700	-5.98700	-23.03100
O	21.21600	-2.12600	-33.14800
H	22.12800	-1.89300	-33.32300
H	20.70700	-1.54300	-33.71100
O	14.02400	0.15300	-18.10900
H	14.76600	0.69200	-18.38600
H	13.31800	0.78000	-17.95200
O	19.50000	2.29600	-19.51200
H	19.90800	1.85400	-20.25700
H	18.77800	1.72200	-19.25900
O	15.85300	-2.76600	-22.13900
H	16.69900	-2.34300	-21.99500
H	15.79300	-3.42300	-21.44500
O	19.82300	2.85600	-25.75700
H	19.19400	3.02100	-26.45900
H	19.43500	3.27100	-24.98600
O	23.70200	3.79200	-28.40200
H	23.01700	4.17200	-28.95300
H	23.78600	4.40100	-27.66900
O	19.00300	-5.10600	-20.48000
H	18.75900	-4.35200	-21.01700
H	19.27900	-4.72800	-19.64600
O	20.95700	1.43800	-21.80300
H	21.16800	2.08100	-22.47900
H	20.91000	0.60400	-22.27100
O	18.55900	-5.95000	-24.89900
H	17.69800	-5.65200	-24.60700
H	18.58600	-5.73300	-25.83100
O	15.37500	2.59400	-28.49200
H	15.83900	2.87300	-29.28100
H	15.11100	1.69200	-28.67400
O	12.26800	2.14100	-25.55400
H	12.83800	2.57000	-26.19200
H	12.85900	1.85900	-24.85500
O	14.31200	-1.28400	-31.68300
H	15.01500	-0.83200	-31.21500
H	13.98000	-1.92600	-31.05500
O	12.76000	-2.69300	-17.30800
H	12.13000	-2.01100	-17.07400
H	12.65400	-2.80400	-18.25200
O	8.05500	-3.02600	-26.38100
H	7.26500	-2.77000	-26.85700
H	8.54600	-2.21100	-26.27600
O	22.07100	4.31500	-23.63100
H	22.89300	3.91300	-23.91200
H	22.06800	4.21200	-22.67900
O	10.00600	-2.66100	-29.42300
H	9.20900	-2.54900	-28.90400
H	9.77900	-2.32200	-30.28900
O	15.83700	3.86300	-21.47400
H	15.90100	4.32500	-20.63900

H	15.12800	3.23300	-21.34600
O	6.33900	-2.77200	-23.95000
H	6.58900	-1.88800	-23.68400
H	6.87500	-2.95200	-24.72200
O	30.16200	0.65900	-27.28200
H	29.40800	0.09000	-27.43800
H	30.29700	0.62500	-26.33500
O	15.55200	5.53000	-31.60700
H	15.41600	6.37500	-31.17800
H	16.09300	5.73200	-32.37000
O	12.03800	-8.39100	-14.94500
H	11.64500	-8.94400	-14.27000
H	12.85500	-8.08000	-14.55400
O	9.00500	3.04800	-31.03400
H	9.51700	3.84200	-30.88300
H	9.65700	2.35000	-31.10000
O	10.81400	6.16400	-28.95100
H	10.99100	7.08600	-29.13900
H	11.61000	5.85000	-28.52200
O	14.95500	-6.97500	-17.35300
H	15.69600	-6.37400	-17.43300
H	15.18700	-7.71700	-17.91100
O	10.09900	-1.78900	-19.07500
H	9.98500	-2.69100	-18.77500
H	9.24400	-1.54300	-19.42700
O	17.17900	6.15300	-17.47700
H	16.73500	5.36600	-17.79400
H	17.84000	6.34000	-18.14200
O	11.93700	7.68200	-22.95000
H	12.48800	7.55400	-23.72300
H	11.09800	7.98200	-23.30100
O	19.27700	6.43300	-19.44800
H	19.25900	7.25400	-19.94000
H	20.19900	6.30800	-19.22300
O	15.73400	3.94700	-18.60600
H	16.26700	3.18600	-18.83600
H	15.20500	3.65500	-17.86400
O	18.90100	-10.94700	-23.07700
H	18.55700	-10.05700	-23.15300
H	19.26900	-11.13700	-23.94000
O	6.97000	1.37700	-30.25800
H	7.80700	1.78600	-30.47400
H	7.14700	0.43700	-30.28800
O	18.86600	-0.95600	-34.56900
H	18.35100	-0.18500	-34.33100
H	18.59400	-1.62900	-33.94600
O	16.45100	-6.11100	-29.51100
H	15.59500	-6.50000	-29.69000
H	16.86300	-6.03200	-30.37100
O	17.98100	-8.57600	-25.11200
H	17.47700	-8.61300	-25.92500
H	18.27400	-7.66700	-25.05200
O	14.22500	-8.49200	-26.10500
H	15.09000	-8.68800	-26.46500
H	14.29300	-8.72100	-25.17800
O	23.83900	1.79700	-32.63400
H	23.06200	1.45800	-33.07900
H	24.51900	1.80300	-33.30900
O	18.65500	-3.08300	-32.74300
H	18.35000	-2.76900	-31.89200
H	19.60800	-3.00500	-32.70100
O	17.09900	3.07600	-31.09400
H	16.57100	2.34100	-31.40700
H	16.59800	3.85300	-31.34000
O	22.07900	1.27100	-25.97700
H	21.42100	1.96100	-25.88900
H	22.88300	1.66000	-25.63400
O	11.83100	-6.26200	-28.73300
H	12.40600	-6.48500	-29.46600
H	12.41800	-6.19300	-27.98100
O	20.88900	-0.37500	-27.92700
H	21.40200	0.25800	-27.42500
H	21.53900	-0.97500	-28.29300
O	12.15800	-4.23200	-34.71400
H	11.32200	-3.89400	-34.39300
H	12.01900	-4.36700	-35.65100
O	14.13300	-9.38800	-30.00600
H	13.48000	-9.70300	-30.63000
H	14.09100	-10.01000	-29.28000
O	15.24500	0.89400	-38.09100
H	14.33700	0.66300	-37.89200
H	15.74200	0.09800	-37.90300
O	18.15100	9.60400	-35.17100
H	17.47800	9.90700	-34.56200
H	18.53800	10.40700	-35.52000
O	17.36700	-2.90000	-25.68100
H	16.44900	-2.67800	-25.83900
H	17.78300	-2.82500	-26.54000
O	18.15100	-1.50100	-30.55100
H	18.89800	-0.98900	-30.86000
H	18.42000	-1.82200	-29.69100
O	16.42300	0.99500	-33.56800
H	15.51500	0.71100	-33.47100
H	16.45300	1.41500	-34.42800
O	21.65500	4.60600	-29.79900
H	21.20200	3.76500	-29.74300
H	21.02500	5.24200	-29.46200
O	20.59900	-4.46500	-28.56900
H	19.86100	-4.97700	-28.23800
H	20.85100	-4.90500	-29.38100
O	20.09900	6.44300	-28.86400
H	20.39100	7.00400	-29.58300
H	20.60800	6.73600	-28.10900
O	20.70000	-11.68700	-27.97500
H	21.00600	-12.54800	-28.26200
H	21.42500	-11.09600	-28.17500
O	6.99200	-1.28400	-29.58700
H	6.79500	-1.87700	-30.31200
H	6.43700	-1.58900	-28.86900
O	14.97200	-1.39200	-27.46700
H	15.02700	-2.34500	-27.54300
H	14.16100	-1.23600	-26.98300
O	16.12100	2.53600	-36.22900
H	15.68300	1.82000	-36.68900
H	16.86200	2.76400	-36.79100
O	12.91100	-2.77400	-29.70800
H	12.09800	-2.47500	-29.30200
H	13.40700	-3.16900	-28.99100

O	15.06200	7.22100	-27.77500
H	15.02000	7.44800	-28.70400
H	15.97800	6.98800	-27.62600
O	18.50900	-2.31000	-20.87700
H	18.86300	-2.08300	-20.01700
H	19.15600	-1.97800	-21.49900
O	24.23800	0.81200	-30.17200
H	24.10000	1.00200	-31.10000
H	23.88200	1.57400	-29.71500
O	12.40300	-5.20200	-24.09200
H	13.04200	-5.34400	-24.79000
H	11.57000	-5.48900	-24.46800
O	24.26500	2.87900	-25.16100
H	24.69900	2.78700	-24.31200
H	24.97800	2.85500	-25.79900
O	13.17900	4.09800	-27.97900
H	13.97300	3.60300	-28.18000
H	12.46800	3.57000	-28.34200
O	17.99800	6.48000	-23.62700
H	18.05200	7.04900	-22.86000
H	17.13000	6.65000	-23.99200
O	9.48700	-1.75800	-31.90300
H	8.55700	-1.81900	-31.68600
H	9.58300	-2.28000	-32.69900
O	9.40500	-5.23200	-29.31500
H	9.66600	-4.31800	-29.42400
H	10.22700	-5.70000	-29.16500
O	10.87800	-3.81900	-22.14500
H	11.55000	-4.02700	-22.79400
H	10.05100	-4.01600	-22.58400
O	25.12700	2.92200	-22.62700
H	25.32000	3.82600	-22.38100
H	25.02700	2.46200	-21.79400
O	16.78000	-7.19200	-20.12800
H	16.13300	-6.49300	-20.22200
H	17.55100	-6.75900	-19.76100
O	20.18800	3.07200	-17.05300
H	20.03700	2.84300	-17.97000
H	20.17900	4.02900	-17.04000
O	16.95700	0.64600	-22.06900
H	17.88600	0.79700	-22.24200
H	16.53600	0.73400	-22.92400
O	15.75600	4.67100	-24.29900
H	15.61700	4.30300	-23.42600
H	14.96700	5.18500	-24.47000
O	16.35000	9.77200	-32.95600
H	16.52600	9.30000	-32.14200
H	15.66400	10.39800	-32.72800
O	17.30600	4.04900	-26.91000
H	16.97100	4.00900	-26.01400
H	16.80700	3.38300	-27.38300
O	11.28100	-0.54400	-21.38500
H	11.14300	-1.27600	-20.78400
H	12.19100	-0.63300	-21.66600
O	20.24200	1.39400	-36.63600
H	19.62500	0.66900	-36.73300
H	19.77400	2.15500	-36.98000
O	13.90900	-0.83200	-21.89600
H	14.61900	-1.47300	-21.93800
H	13.86600	-0.58100	-20.97400
O	21.89800	6.10400	-18.97600
H	21.83900	5.42000	-19.64300
H	22.29900	6.84600	-19.42800
O	12.62200	-3.84600	-20.07700
H	12.08500	-4.29700	-19.42500
H	12.03300	-3.71100	-20.82000
O	10.91700	12.92100	-26.18200
H	11.15600	13.55200	-25.50400
H	10.07400	12.56900	-25.89600
O	12.03200	-4.38700	-37.51400
H	11.15500	-4.50800	-37.87800
H	12.50800	-5.17800	-37.76600
O	21.89900	4.06800	-20.74400
H	21.01200	3.73100	-20.62200
H	22.46900	3.33700	-20.50700
O	3.55200	0.40100	-27.42000
H	4.23900	0.08700	-28.00800
H	3.39700	1.30500	-27.69300
O	8.35600	-4.71000	-22.99100
H	8.27100	-5.31800	-23.72600
H	7.53400	-4.21900	-22.99100
O	15.42900	8.72800	-23.77800
H	15.35600	9.53400	-23.26600
H	14.98700	8.92800	-24.60300
O	15.21800	7.96600	-30.26800
H	14.43500	8.50800	-30.17400
H	15.91800	8.48300	-29.86800
O	18.52800	-6.22500	-27.62400
H	19.02500	-6.97800	-27.94400
H	17.75700	-6.18800	-28.19000
O	26.38100	-0.52100	-25.98300
H	26.37000	0.36200	-26.35300
H	25.51300	-0.87400	-26.17700
O	17.88900	8.62700	-21.62200
H	18.60200	9.26200	-21.69500
H	17.39800	8.72400	-22.43800
O	19.61100	2.53200	-30.05100
H	18.67700	2.65200	-30.22000
H	19.78900	1.63000	-30.31500
O	15.11200	-4.08300	-27.89700
H	14.74100	-4.63600	-27.20900
H	15.60700	-4.68700	-28.45000
O	22.99800	-1.62600	-29.51600
H	23.46400	-0.79300	-29.59000
H	23.44900	-2.09200	-28.81100
O	12.94000	6.37700	-20.80200
H	13.64100	7.01000	-20.64900
H	12.47000	6.71800	-21.56200
O	21.06600	4.71800	-32.51700
H	21.34200	4.60400	-31.60800
H	20.19600	4.32000	-32.55600
O	20.97000	-9.25800	-25.47700
H	20.05600	-9.02800	-25.30800
H	21.39900	-9.17400	-24.62500
O	20.74100	-1.06900	-22.97700
H	20.08800	-0.82100	-23.63100
H	21.11900	-1.88300	-23.31000
O	17.70100	8.96700	-28.87400

H	17.88400	8.14900	-28.41100
H	18.52500	9.18400	-29.31100
O	18.03600	7.89400	-31.61200
H	18.56400	7.38300	-32.22500
H	18.29800	7.57800	-30.74800
O	16.78900	-0.32700	-16.49000
H	16.11300	-0.42800	-15.82000
H	17.61200	-0.48300	-16.02600
O	8.53700	5.91000	-23.86900
H	9.12300	5.16500	-23.73600
H	8.34000	6.22100	-22.98500
O	11.39100	8.94500	-28.85200
H	10.72200	9.43000	-29.33600
H	11.25200	9.19200	-27.93800
O	3.12900	-1.51900	-25.14800
H	3.16700	-2.05300	-24.35500
H	2.62300	-2.04600	-25.76700
O	15.53200	10.98000	-22.25400
H	16.08300	11.14300	-21.48900
H	15.87200	11.57000	-22.92600
O	10.50800	-6.67300	-21.65200
H	10.57200	-5.81100	-22.06400
H	10.44500	-7.28500	-22.38500
O	9.98300	-4.60000	-18.00500
H	10.61000	-4.98300	-17.39100
H	9.12600	-4.81900	-17.63900
O	20.38300	5.80600	-16.39900
H	20.92700	6.41900	-16.89400
H	19.80100	6.36400	-15.88300
O	6.76000	-2.79300	-32.07300
H	7.05500	-3.70200	-32.12600
H	5.85900	-2.81400	-32.39500
O	18.66800	8.92800	-25.26800
H	18.54400	8.12700	-25.77700
H	19.32600	9.42300	-25.75600
O	10.28900	-4.71700	-26.09000
H	9.56800	-4.10400	-26.22900
H	11.07900	-4.18100	-26.16800
O	20.73200	6.72900	-23.64000
H	19.78500	6.60900	-23.71200
H	21.08000	5.84100	-23.55700
O	13.09200	-7.38200	-19.81600
H	13.61500	-7.13600	-19.05300
H	12.87300	-6.55000	-20.23600
O	8.48900	-5.11100	-31.94900
H	8.16000	-5.99200	-32.13100
H	8.84300	-5.16700	-31.06100
O	17.73700	6.52400	-27.43900
H	17.58900	5.59600	-27.25900
H	18.56200	6.54600	-27.92400
O	11.60300	-4.82400	-31.58100
H	11.94200	-4.29600	-32.30300
H	12.05000	-4.48600	-30.80500
O	21.34000	7.34700	-26.35300
H	22.26500	7.14100	-26.49000
H	21.17200	7.09900	-25.44400
O	14.29700	-6.44000	-22.13100
H	13.95700	-7.24000	-21.72900
H	13.59100	-6.14000	-22.70300
O	14.94900	-9.35500	-23.67800
H	15.77100	-9.82900	-23.80000
H	15.19300	-8.57600	-23.17800
O	13.59000	-6.32700	-33.38200
H	14.31000	-5.94900	-33.88600
H	12.96600	-5.60800	-33.27900
O	17.73300	-5.62200	-32.44600
H	17.94900	-4.74000	-32.74700
H	17.25200	-6.01400	-33.17600
O	12.47300	-2.66400	-25.86900
H	12.32700	-2.35000	-24.97700
H	11.99500	-2.04500	-26.42200
O	14.33200	-5.67300	-25.82100
H	14.31800	-6.56900	-26.15800
H	15.05000	-5.66600	-25.18700
O	16.18500	-5.24000	-23.77600
H	15.92700	-4.31800	-23.77700
H	15.65300	-5.63700	-23.08600
O	21.13000	7.86600	-30.99700
H	21.30300	7.30000	-31.74900
H	21.62200	8.66600	-31.17900
O	6.94700	-0.12300	-22.93000
H	6.40300	0.65300	-22.79800
H	7.82500	0.22400	-23.08800
O	13.73600	-6.79200	-30.55200
H	13.85800	-7.72000	-30.35300
H	13.44600	-6.77800	-31.46400
O	16.60500	-9.10600	-27.41600
H	16.62700	-9.04200	-28.37000
H	17.02200	-9.94600	-27.22200
O	26.98300	0.62900	-29.37800
H	27.42700	-0.12000	-29.77600
H	26.10600	0.62000	-29.76000
O	22.11700	7.18200	-33.89700
H	22.81200	7.80300	-33.67800
H	22.45900	6.33200	-33.62200
O	7.73400	-0.99600	-20.03700
H	6.99200	-0.84800	-19.45100
H	7.41300	-0.73500	-20.90100
O	24.01600	4.63200	-32.36800
H	24.54100	4.97100	-33.09300
H	23.92800	3.69700	-32.55200
O	15.67800	8.54600	-20.23900
H	15.98700	8.30000	-19.36700
H	16.47600	8.75000	-20.72700
O	15.70000	10.69900	-28.00900
H	15.26100	10.20700	-27.31500
H	16.45100	10.15700	-28.25000
O	8.17900	6.89100	-28.00600
H	9.00500	6.59100	-28.38600
H	7.82900	6.12100	-27.55700
O	13.14800	6.24300	-25.93700
H	12.52600	5.58000	-26.23600
H	13.81800	6.26900	-26.61900
O	6.38000	4.80900	-27.04700
H	5.54400	4.38100	-27.22900
H	6.35300	5.00200	-26.11000
O	13.22000	-5.27900	-16.34300
H	13.26900	-4.40200	-16.72500

H	13.76200	-5.82100	-16.91600
O	23.70600	5.26500	-17.06300
H	22.91500	5.24800	-17.60300
H	23.46900	5.80400	-16.30800
O	23.82500	2.11500	-19.75000
H	23.87500	1.95000	-18.80800
H	23.71700	1.24700	-20.14000
O	12.70900	4.79400	-32.46100
H	12.85800	5.47700	-33.11400
H	13.51600	4.77400	-31.94600
O	21.11600	-1.90200	-18.43400
H	22.01700	-2.04200	-18.72600
H	21.19100	-1.73700	-17.49400
O	15.45000	-4.52600	-19.58900
H	14.58000	-4.23200	-19.86000
H	15.68600	-3.94100	-18.86800
O	25.32700	4.53400	-19.50600
H	25.03600	4.89700	-18.66900
H	24.71400	3.82100	-19.68400
O	26.52700	2.10000	-26.98500
H	27.36100	2.53900	-26.82200
H	26.66300	1.62400	-27.80500
O	23.57600	-0.34200	-21.03800
H	24.05100	-0.15000	-21.84600
H	22.65200	-0.28600	-21.28300
O	10.46400	0.82000	-31.45500
H	10.21900	-0.08400	-31.25700
H	10.63400	0.82200	-32.39700
O	18.15400	-8.30800	-22.07200
H	18.67200	-7.57100	-22.39700
H	17.79900	-8.00100	-21.23700
O	9.73400	-3.44700	-33.95600
H	9.04100	-3.37300	-34.61200
H	9.48900	-4.21100	-33.43400
O	18.40500	-11.88800	-26.55200
H	18.68500	-12.55500	-25.92600
H	19.19000	-11.69500	-27.06500
O	16.20900	7.28800	-34.98900
H	15.63600	7.90700	-35.44200
H	16.78200	7.83800	-34.45600
O	22.27600	-3.46600	-22.80500
H	23.22800	-3.36600	-22.78400
H	22.10600	-4.25800	-22.29500
O	20.29800	-6.38000	-22.61900
H	19.70200	-6.10000	-23.31400
H	20.01200	-5.89100	-21.84700
O	19.42800	7.57200	-33.85900
H	20.36100	7.50700	-33.65500
H	19.34100	8.40800	-34.31700
O	22.05700	6.88900	-36.89800
H	21.97800	6.55500	-36.00500
H	22.87800	7.38100	-36.89900
O	24.75500	-0.24500	-23.69100
H	24.72300	-1.18400	-23.50500
H	25.36500	-0.16700	-24.42400
O	16.59300	-1.37600	-37.64800
H	17.53900	-1.50500	-37.57900
H	16.21500	-2.11400	-37.17100
O	24.90400	-3.45400	-23.64500
H	24.38800	-4.23800	-23.83200
H	25.78800	-3.78200	-23.48000
O	22.56500	-9.71000	-27.68500
H	22.36800	-10.01800	-26.80100
H	23.17500	-8.98400	-27.55500
O	30.57300	0.81600	-24.55000
H	31.30300	0.55500	-23.98900
H	29.96300	0.07900	-24.51000
O	28.65400	-1.04300	-23.95300
H	27.92700	-1.17700	-24.56100
H	28.23900	-0.73500	-23.14700
O	16.47700	2.67600	-16.18800
H	17.39700	2.85400	-15.99400
H	16.44200	1.73200	-16.34000
O	14.77700	-3.45100	-37.71200
H	13.87100	-3.74700	-37.63600
H	15.05500	-3.74800	-38.57900
O	5.69500	-2.13000	-27.26200
H	5.37600	-2.98300	-27.55500
H	5.14900	-1.91500	-26.50700
O	9.47300	0.58600	-23.54600
H	9.69200	1.51800	-23.52700
H	9.88600	0.22700	-22.76000
O	16.39400	-8.90700	-31.55800
H	15.58400	-9.25200	-31.18200
H	17.08200	-9.21600	-30.96900
O	18.32300	-9.82900	-29.93600
H	18.87600	-9.41000	-29.27700
H	18.56000	-10.75500	-29.89300
O	14.34600	-3.84000	-14.15500
H	14.24600	-2.89100	-14.22400
H	14.12800	-4.16900	-15.02800
O	13.54700	-6.59200	-37.57100
H	14.40900	-6.26600	-37.31200
H	13.73200	-7.37900	-38.08400
O	5.56400	1.49000	-25.29300
H	4.87000	1.07600	-25.80600
H	6.31100	1.53200	-25.89000
O	21.70100	-7.71900	-30.42800
H	21.12100	-7.06100	-30.81000
H	21.43400	-8.54100	-30.83900
O	20.15100	-8.31900	-28.22600
H	20.65100	-8.20700	-29.03400
H	20.79500	-8.62200	-27.58500
O	14.20900	-0.41100	-15.33900
H	13.72600	0.38900	-15.13200
H	14.29500	-0.40000	-16.29200
O	7.47800	1.42200	-27.41700
H	7.45500	1.52100	-28.36800
H	7.78800	2.26900	-27.09500
O	10.13700	3.48000	-23.30200
H	11.09100	3.44800	-23.36900
H	9.93800	3.01900	-22.48700
O	10.72600	2.05000	-20.73500
H	11.54600	2.54400	-20.71100
H	11.00000	1.13300	-20.76300
O	20.48300	-5.55900	-31.37200
H	19.59600	-5.27400	-31.59000
H	20.89900	-5.72000	-32.21900

O	23.83500	-5.53400	-27.65700
H	24.61100	-5.04500	-27.38400
H	23.10900	-5.10900	-27.19900
O	26.27200	-4.82200	-26.59900
H	26.86600	-5.06200	-27.31000
H	26.34400	-5.54100	-25.97200
O	8.49200	9.02200	-26.28500
H	8.35700	8.26200	-26.85100
H	8.07500	9.74600	-26.75200
O	19.62700	4.20500	-37.27700
H	20.33100	4.41000	-36.66100
H	18.90400	4.77300	-37.01200
O	20.45700	-0.04400	-31.15700
H	20.40100	0.41300	-31.99600
H	21.01000	-0.80400	-31.33500
O	24.26800	-4.25500	-30.33200
H	24.01100	-4.65500	-29.50100
H	23.64300	-3.54000	-30.45700
O	28.57900	-1.51500	-27.82500
H	28.01900	-1.44600	-27.05200
H	27.97300	-1.69100	-28.54500
O	11.64800	9.91900	-26.07300
H	11.53200	10.86300	-26.17500
H	10.82800	9.61600	-25.68400
O	21.53600	4.64400	-35.22700
H	22.37100	4.21200	-35.41000
H	21.37500	4.47200	-34.30000
O	21.19100	1.14700	-33.86200
H	21.13600	0.98100	-34.80300
H	20.56500	1.85500	-33.70600
O	18.50500	3.45200	-14.53600
H	19.37400	3.62600	-14.89800
H	18.02600	4.27100	-14.65900
O	14.50000	-9.35200	-34.28300
H	14.48800	-8.40800	-34.12600
H	15.39300	-9.53500	-34.57700
O	12.67400	10.57200	-22.85700
H	13.57700	10.60400	-22.54100
H	12.44800	9.64200	-22.84300
O	14.42900	9.32500	-26.19600
H	13.47900	9.32500	-26.31000
H	14.73300	8.57800	-26.71300
O	18.31600	-12.75800	-30.02900
H	17.54900	-13.31200	-29.88300
H	18.72700	-12.68600	-29.16700
O	10.32000	-6.47000	-15.93800
H	11.18300	-6.82600	-15.72800
H	9.74600	-6.80800	-15.25100
O	30.16500	15.16100	-25.13200
H	30.80200	15.04100	-25.83600
H	30.04700	16.10900	-25.07300
O	26.97400	15.11300	-25.19600
H	26.63500	14.99800	-26.08400
H	27.84200	14.71300	-25.21600
O	22.83200	16.96700	-29.20600
H	22.26900	16.87600	-29.97400
H	22.71700	16.14700	-28.72600
O	27.45500	16.26700	-19.23700
H	27.44400	15.87200	-20.10900
H	27.61800	15.53400	-18.64400
O	20.37900	18.46200	-26.36800
H	21.17100	18.12100	-26.78300
H	20.61800	18.57200	-25.44700
O	24.17600	19.05100	-20.28900
H	23.88500	19.95500	-20.40500
H	24.81200	19.09200	-19.57500
O	25.10200	16.42800	-23.69900
H	25.69800	16.20400	-24.41300
H	25.15900	15.68700	-23.09700
O	29.71900	12.88600	-28.05700
H	30.50500	12.46800	-28.40800
H	29.98300	13.79000	-27.88800
O	35.12000	12.82300	-27.15300
H	35.85200	12.25300	-26.91900
H	34.36300	12.43600	-26.71300
O	26.73700	15.24600	-29.95700
H	26.37300	14.71900	-29.24600
H	27.59400	14.85600	-30.13000
O	32.64200	6.53500	-22.69400
H	32.31000	7.40500	-22.91800
H	32.04700	6.21900	-22.01400
O	29.13500	18.73700	-23.29400
H	28.83500	19.63900	-23.40300
H	28.79000	18.47000	-22.44200
O	22.58600	15.18800	-27.26100
H	21.66200	15.05600	-27.05300
H	22.83500	15.96800	-26.76400
O	32.53500	11.25500	-25.61400
H	31.62700	11.18700	-25.90900
H	32.48400	11.73200	-24.78600
O	26.35700	12.73100	-33.97600
H	26.23700	13.17700	-33.13800
H	25.66200	13.07900	-34.53500
O	19.94700	14.83100	-26.42600
H	20.07700	15.08500	-25.51200
H	19.26700	15.42400	-26.74600
O	26.60900	12.67400	-17.67100
H	25.83200	13.23300	-17.68100
H	27.24800	13.16000	-17.14900
O	34.40100	9.45700	-23.83700
H	33.75900	9.75000	-24.48300
H	33.89700	9.34200	-23.03100
O	24.86400	15.48300	-18.10900
H	25.60500	16.02100	-18.38600
H	24.15700	16.10900	-17.95200
O	30.33900	17.62500	-19.51200
H	30.74800	17.18400	-20.25700
H	29.61700	17.05100	-19.25900
O	26.69200	12.56300	-22.13900
H	27.53800	12.98700	-21.99500
H	26.63200	11.90600	-21.44500
O	30.66200	18.18600	-25.75700
H	30.03300	18.35000	-26.45900
H	30.27500	18.60000	-24.98600
O	29.84200	10.22300	-20.48000
H	29.59800	10.97700	-21.01700
H	30.11800	10.60200	-19.64600
O	31.79600	16.76700	-21.80300

H	32.00800	17.41000	-22.47900
H	31.74900	15.93300	-22.27100
O	29.39900	9.37900	-24.89900
H	28.53700	9.67700	-24.60700
H	29.42500	9.59600	-25.83100
O	26.21500	17.92300	-28.49200
H	26.67900	18.20200	-29.28100
H	25.95100	17.02100	-28.67400
O	23.10700	17.47000	-25.55400
H	23.67700	17.89900	-26.19200
H	23.69800	17.18800	-24.85500
O	25.15200	14.04500	-31.68300
H	25.85400	14.49700	-31.21500
H	24.82000	13.40400	-31.05500
O	23.60000	12.63600	-17.30800
H	22.97000	13.31800	-17.07400
H	23.49300	12.52500	-18.25200
O	18.89400	12.30400	-26.38100
H	18.10400	12.55900	-26.85700
H	19.38500	13.11800	-26.27600
O	32.91100	19.64400	-23.63100
H	33.73200	19.24200	-23.91200
H	32.90700	19.54100	-22.67900
O	20.84600	12.66800	-29.42300
H	20.04900	12.78000	-28.90400
H	20.61800	13.00700	-30.28900
O	26.67600	19.19300	-21.47400
H	26.74100	19.65500	-20.63900
H	25.96700	18.56200	-21.34600
O	17.17800	12.55700	-23.95000
H	17.42900	13.44100	-23.68400
H	17.71400	12.37700	-24.72200
O	22.87800	6.93900	-14.94500
H	22.48500	6.38500	-14.27000
H	23.69400	7.25000	-14.55400
O	19.84400	18.37700	-31.03400
H	20.35700	19.17100	-30.88300
H	20.49600	17.68000	-31.10000
O	21.65400	21.49400	-28.95100
H	21.83000	22.41500	-29.13900
H	22.44900	21.17900	-28.52200
O	25.79500	8.35400	-17.35300
H	26.53600	8.95500	-17.43300
H	26.02600	7.61200	-17.91100
O	20.93900	13.54000	-19.07500
H	20.82400	12.63900	-18.77500
H	20.08300	13.78600	-19.42700
O	28.01800	21.48200	-17.47700
H	27.57400	20.69500	-17.79400
H	28.68000	21.67000	-18.14200
O	22.77700	23.01100	-22.95000
H	23.32700	22.88300	-23.72300
H	21.93800	23.31100	-23.30100
O	30.11600	21.76200	-19.44800
H	30.09800	22.58300	-19.94000
H	31.03800	21.63700	-19.22300
O	26.57300	19.27600	-18.60600
H	27.10600	18.51500	-18.83600
H	26.04500	18.98400	-17.86400
O	29.74000	4.38200	-23.07700
H	29.39700	5.27300	-23.15300
H	30.10800	4.19300	-23.94000
O	17.80900	16.70600	-30.25800
H	18.64600	17.11600	-30.47400
H	17.98700	15.76600	-30.28800
O	27.29000	9.21800	-29.51100
H	26.43400	8.82900	-29.69000
H	27.70300	9.29700	-30.37100
O	28.82000	6.75300	-25.11200
H	28.31700	6.71600	-25.92500
H	29.11300	7.66200	-25.05200
O	25.06400	6.83700	-26.10500
H	25.93000	6.64100	-26.46500
H	25.13200	6.60800	-25.17800
O	29.49400	12.24600	-32.74300
H	29.18900	12.56100	-31.89200
H	30.44700	12.32400	-32.70100
O	32.91800	16.60000	-25.97700
H	32.26000	17.29000	-25.88900
H	33.72200	16.99000	-25.63400
O	22.67100	9.06700	-28.73300
H	23.24500	8.84500	-29.46600
H	23.25800	9.13600	-27.98100
O	31.72900	14.95400	-27.92700
H	32.24200	15.58700	-27.42500
H	32.37900	14.35500	-28.29300
O	22.99800	11.09700	-34.71400
H	22.16100	11.43500	-34.39300
H	22.85800	10.96200	-35.65100
O	24.97300	5.94100	-30.00600
H	24.31900	5.62600	-30.63000
H	24.93100	5.31900	-29.28000
O	28.20700	12.42900	-25.68100
H	27.28900	12.65100	-25.83900
H	28.62300	12.50400	-26.54000
O	28.99000	13.82800	-30.55100
H	29.73700	14.34100	-30.86000
H	29.25900	13.50700	-29.69100
O	31.43800	10.86400	-28.56900
H	30.70000	10.35200	-28.23800
H	31.69100	10.42400	-29.38100
O	31.53900	3.64200	-27.97500
H	31.84500	2.78200	-28.26200
H	32.26400	4.23400	-28.17500
O	17.83200	14.04500	-29.58700
H	17.63400	13.45200	-30.31200
H	17.27600	13.74000	-28.86900
O	25.81100	13.93700	-27.46700
H	25.86600	12.98500	-27.54300
H	25.00000	14.09300	-26.98300
O	23.75000	12.55500	-29.70800
H	22.93700	12.85400	-29.30200
H	24.24600	12.16000	-28.99100
O	25.90100	22.55100	-27.77500
H	25.85900	22.77700	-28.70400
H	26.81800	22.31700	-27.62600
O	29.34800	13.01900	-20.87700
H	29.70300	13.24600	-20.01700

H	29.99500	13.35100	-21.49900
O	23.24200	10.12700	-24.09200
H	23.88200	9.98600	-24.79000
H	22.41000	9.84000	-24.46800
O	35.10500	18.20900	-25.16100
H	35.53800	18.11600	-24.31200
H	35.81800	18.18400	-25.79900
O	24.01800	19.42700	-27.97900
H	24.81200	18.93200	-28.18000
H	23.30700	18.89900	-28.34200
O	28.83700	21.80900	-23.62700
H	28.89100	22.37800	-22.86000
H	27.96900	21.97900	-23.99200
O	20.32600	13.57100	-31.90300
H	19.39600	13.51000	-31.68600
H	20.42200	13.04900	-32.69900
O	20.24500	10.09700	-29.31500
H	20.50500	11.01100	-29.42400
H	21.06600	9.62900	-29.16500
O	21.71700	11.51100	-22.14500
H	22.39000	11.30200	-22.79400
H	20.89000	11.31300	-22.58400
O	35.96600	18.25100	-22.62700
H	36.16000	19.15600	-22.38100
H	35.86600	17.79200	-21.79400
O	27.62000	8.13700	-20.12800
H	26.97300	8.83600	-20.22200
H	28.39000	8.57000	-19.76100
O	31.02800	18.40200	-17.05300
H	30.87600	18.17300	-17.97000
H	31.01800	19.35900	-17.04000
O	27.79600	15.97500	-22.06900
H	28.72500	16.12600	-22.24200
H	27.37500	16.06300	-22.92400
O	26.59500	20.00000	-24.29900
H	26.45600	19.63200	-23.42600
H	25.80700	20.51500	-24.47000
O	28.14600	19.37800	-26.91000
H	27.81100	19.33800	-26.01400
H	27.64600	18.71300	-27.38300
O	22.12000	14.78500	-21.38500
H	21.98200	14.05300	-20.78400
H	23.03000	14.69600	-21.66600
O	24.74800	14.49700	-21.89600
H	25.45800	13.85600	-21.93800
H	24.70500	14.74800	-20.97400
O	32.73700	21.43400	-18.97600
H	32.67800	20.74900	-19.64300
H	33.13900	22.17500	-19.42800
O	23.46100	11.48300	-20.07700
H	22.92500	11.03200	-19.42500
H	22.87300	11.61800	-20.82000
O	21.75600	28.25000	-26.18200
H	21.99600	28.88200	-25.50400
H	20.91300	27.89900	-25.89600
O	32.73900	19.39700	-20.74400
H	31.85100	19.06000	-20.62200
H	33.30800	18.66600	-20.50700
O	14.39200	15.73000	-27.42000
H	15.07900	15.41600	-28.00800
H	14.23600	16.63400	-27.69300
O	19.19500	10.61900	-22.99100
H	19.11100	10.01100	-23.72600
H	18.37300	11.11000	-22.99100
O	26.26900	24.05800	-23.77800
H	26.19600	24.86300	-23.26600
H	25.82600	24.25700	-24.60300
O	29.36700	9.10400	-27.62400
H	29.86400	8.35100	-27.94400
H	28.59600	9.14100	-28.19000
O	37.22000	14.80800	-25.98300
H	37.20900	15.69100	-26.35300
H	36.35200	14.45500	-26.17700
O	28.72900	23.95600	-21.62200
H	29.44100	24.59100	-21.69500
H	28.23800	24.05300	-22.43800
O	25.95100	11.24600	-27.89700
H	25.58100	10.69300	-27.20900
H	26.44700	10.64200	-28.45000
O	33.83800	13.70300	-29.51600
H	34.30400	14.53600	-29.59000
H	34.28800	13.23800	-28.81100
O	23.78000	21.70600	-20.80200
H	24.48100	22.33900	-20.64900
H	23.30900	22.04700	-21.56200
O	31.80900	6.07100	-25.47700
H	30.89600	6.30200	-25.30800
H	32.23900	6.15500	-24.62500
O	31.58000	14.26000	-22.97700
H	30.92700	14.50800	-23.63100
H	31.95800	13.44700	-23.31000
O	27.62900	15.00200	-16.49000
H	26.95300	14.90200	-15.82000
H	28.45100	14.84600	-16.02600
O	19.37700	21.23900	-23.86900
H	19.96200	20.49400	-23.73600
H	19.17900	21.55000	-22.98500
O	22.23000	24.27500	-28.85200
H	21.56100	24.75900	-29.33600
H	22.09200	24.52100	-27.93800
O	13.96800	13.81000	-25.14800
H	14.00700	13.27600	-24.35500
H	13.46200	13.28300	-25.76700
O	26.37100	26.30900	-22.25400
H	26.92300	26.47200	-21.48900
H	26.71200	26.89900	-22.92600
O	21.34700	8.65600	-21.65200
H	21.41200	9.51800	-22.06400
H	21.28400	8.04400	-22.38500
O	20.82300	10.72900	-18.00500
H	21.45000	10.34600	-17.39100
H	19.96500	10.51000	-17.63900
O	31.22200	21.13500	-16.39900
H	31.76600	21.74800	-16.89400
H	30.64100	21.69300	-15.88300
O	17.59900	12.53600	-32.07300
H	17.89500	11.62700	-32.12600
H	16.69800	12.51500	-32.39500

O	29.50800	24.25700	-25.26800
H	29.38300	23.45600	-25.77700
H	30.16600	24.75200	-25.75600
O	21.12900	10.61200	-26.09000
H	20.40700	11.22600	-26.22900
H	21.91800	11.14800	-26.16800
O	31.57100	22.05800	-23.64000
H	30.62400	21.93900	-23.71200
H	31.92000	21.17000	-23.55700
O	23.93100	7.94700	-19.81600
H	24.45500	8.19400	-19.05300
H	23.71200	8.77900	-20.23600
O	19.32800	10.21800	-31.94900
H	18.99900	9.33800	-32.13100
H	19.68200	10.16300	-31.06100
O	28.57600	21.85300	-27.43900
H	28.42800	20.92500	-27.25900
H	29.40100	21.87500	-27.92400
O	22.44200	10.50500	-31.58100
H	22.78200	11.03400	-32.30300
H	22.88900	10.84300	-30.80500
O	25.13700	8.88900	-22.13100
H	24.79600	8.09000	-21.72900
H	24.43000	9.18900	-22.70300
O	25.78800	5.97400	-23.67800
H	26.61100	5.50000	-23.80000
H	26.03200	6.75300	-23.17800
O	24.42900	9.00300	-33.38200
H	25.15000	9.38100	-33.88600
H	23.80600	9.72100	-33.27900
O	28.57200	9.70700	-32.44600
H	28.78800	10.59000	-32.74700
H	28.09200	9.31600	-33.17600
O	23.31200	12.66600	-25.86900
H	23.16600	12.98000	-24.97700
H	22.83500	13.28400	-26.42200
O	25.17200	9.65600	-25.82100
H	25.15800	8.76000	-26.15800
H	25.88900	9.66300	-25.18700
O	27.02400	10.09000	-23.77600
H	26.76600	11.01100	-23.77700
H	26.49300	9.69300	-23.08600
O	17.78700	15.20600	-22.93000
H	17.24200	15.98200	-22.79800
H	18.66500	15.55300	-23.08800
O	24.57500	8.53700	-30.55200
H	24.69700	7.60900	-30.35300
H	24.28600	8.55100	-31.46400
O	27.44400	6.22300	-27.41600
H	27.46700	6.28700	-28.37000
H	27.86100	5.38300	-27.22200
O	18.57300	14.33300	-20.03700
H	17.83100	14.48100	-19.45100
H	18.25200	14.59400	-20.90100
O	26.51800	23.87500	-20.23900
H	26.82600	23.63000	-19.36700
H	27.31500	24.07900	-20.72700
O	19.01900	22.22000	-28.00600
H	19.84500	21.92000	-28.38600
H	18.66800	21.45000	-27.55700
O	23.98700	21.57200	-25.93700
H	23.36500	20.90900	-26.23600
H	24.65700	21.59800	-26.61900
O	17.22000	20.13800	-27.04700
H	16.38300	19.71100	-27.22900
H	17.19200	20.33200	-26.11000
O	24.05900	10.05100	-16.34300
H	24.10800	10.92700	-16.72500
H	24.60200	9.50800	-16.91600
O	34.54500	20.59500	-17.06300
H	33.75500	20.57700	-17.60300
H	34.30800	21.13300	-16.30800
O	34.66500	17.44400	-19.75000
H	34.71500	17.27900	-18.80800
H	34.55600	16.57700	-20.14000
O	31.95500	13.42700	-18.43400
H	32.85600	13.28800	-18.72600
H	32.03000	13.59200	-17.49400
O	26.28900	10.80400	-19.58900
H	25.42000	11.09700	-19.86000
H	26.52500	11.38800	-18.86800
O	36.16700	19.86400	-19.50600
H	35.87500	20.22700	-18.66900
H	35.55400	19.15000	-19.68400
O	34.41500	14.98700	-21.03800
H	34.89100	15.18000	-21.84600
H	33.49200	15.04300	-21.28300
O	21.30400	16.14900	-31.45500
H	21.05800	15.24500	-31.25700
H	21.47300	16.15100	-32.39700
O	28.99300	7.02200	-22.07200
H	29.51100	7.75800	-22.39700
H	28.63900	7.32800	-21.23700
O	20.57300	11.88200	-33.95600
H	19.88000	11.95700	-34.61200
H	20.32800	11.11800	-33.43400
O	29.24500	3.44200	-26.55200
H	29.52500	2.77400	-25.92600
H	30.03000	3.63500	-27.06500
O	33.11500	11.86300	-22.80500
H	34.06700	11.96300	-22.78400
H	32.94600	11.07100	-22.29500
O	31.13700	8.94900	-22.61900
H	30.54200	9.22900	-23.31400
H	30.85200	9.43800	-21.84700
O	35.59400	15.08400	-23.69100
H	35.56200	14.14600	-23.50500
H	36.20400	15.16200	-24.42400
O	35.74400	11.87500	-23.64500
H	35.22800	11.09100	-23.83200
H	36.62800	11.54700	-23.48000
O	33.40500	5.61900	-27.68500
H	33.20700	5.31100	-26.80100
H	34.01500	6.34500	-27.55500
O	41.41200	16.14600	-24.55000
H	42.14200	15.88400	-23.98900
H	40.80300	15.40800	-24.51000
O	39.49300	14.28600	-23.95300

H	38.76700	14.15200	-24.56100
H	39.07800	14.59400	-23.14700
O	27.31600	18.00500	-16.18800
H	28.23700	18.18300	-15.99400
H	27.28100	17.06100	-16.34000
O	16.53500	13.19900	-27.26200
H	16.21500	12.34600	-27.55500
H	15.98800	13.41400	-26.50700
O	20.31300	15.91600	-23.54600
H	20.53100	16.84700	-23.52700
H	20.72500	15.55600	-22.76000
O	27.23300	6.42200	-31.55800
H	26.42300	6.07700	-31.18200
H	27.92200	6.11300	-30.96900
O	29.16200	5.50000	-29.93600
H	29.71500	5.92000	-29.27700
H	29.39900	4.57400	-29.89300
O	25.18500	11.48900	-14.15500
H	25.08500	12.43800	-14.22400
H	24.96800	11.16100	-15.02800
O	16.40300	16.82000	-25.29300
H	15.71000	16.40500	-25.80600
H	17.15100	16.86200	-25.89000
O	32.54000	7.61000	-30.42800
H	31.96000	8.26900	-30.81000
H	32.27400	6.78800	-30.83900
O	30.99000	7.01100	-28.22600
H	31.49000	7.12200	-29.03400
H	31.63400	6.70700	-27.58500
O	25.04900	14.91900	-15.33900
H	24.56500	15.71900	-15.13200
H	25.13500	14.92900	-16.29200
O	18.31700	16.75100	-27.41700
H	18.29400	16.85000	-28.36800
H	18.62700	17.59800	-27.09500
O	20.97600	18.80900	-23.30200
H	21.93000	18.77700	-23.36900
H	20.77700	18.34800	-22.48700
O	21.56600	17.37900	-20.73500
H	22.38500	17.87400	-20.71100
H	21.84000	16.46200	-20.76300
O	31.32300	9.77000	-31.37200
H	30.43500	10.05500	-31.59000
H	31.73800	9.61000	-32.21900
O	34.67400	9.79500	-27.65700
H	35.45000	10.28400	-27.38400
H	33.94900	10.22100	-27.19900
O	37.11100	10.50800	-26.59900
H	37.70600	10.26700	-27.31000
H	37.18400	9.78800	-25.97200
O	19.33100	24.35100	-26.28500
H	19.19600	23.59100	-26.85100
H	18.91400	25.07500	-26.75200
O	35.10700	11.07500	-30.33200
H	34.85100	10.67400	-29.50100
H	34.48200	11.78900	-30.45700
O	39.41800	13.81400	-27.82500
H	38.85800	13.88400	-27.05200
H	38.81300	13.63800	-28.54500
O	22.48800	25.24800	-26.07300
H	22.37100	26.19200	-26.17500
H	21.66700	24.94500	-25.68400
O	16.81900	11.71300	-20.03600
H	17.46500	11.20300	-19.54700
H	16.61100	12.45000	-19.46100
O	29.34500	18.78100	-14.53600
H	30.21400	18.95500	-14.89800
H	28.86500	19.60000	-14.65900
O	25.34000	5.97700	-34.28300
H	25.32800	6.92100	-34.12600
H	26.23200	5.79400	-34.57700
O	23.51300	25.90100	-22.85700
H	24.41600	25.93300	-22.54100
H	23.28700	24.97100	-22.84300
O	25.26900	24.65400	-26.19600
H	24.31800	24.65400	-26.31000
H	25.57200	23.90700	-26.71300
O	29.15500	2.57100	-30.02900
H	28.38800	2.01700	-29.88300
H	29.56600	2.64300	-29.16700
O	21.15900	8.86000	-15.93800
H	22.02200	8.50300	-15.72800
H	20.58600	8.52100	-15.25100
O	37.44800	28.00300	-17.67100
H	36.67200	28.56300	-17.68100
H	38.08700	28.48900	-17.14900
O	35.70300	30.81200	-18.10900
H	36.44400	31.35000	-18.38600
H	34.99700	31.43800	-17.95200
O	34.43900	27.96600	-17.30800
H	33.80900	28.64700	-17.07400
H	34.33300	27.85400	-18.25200
O	28.01700	27.88600	-23.95000
H	28.26800	28.77100	-23.68400
H	28.55300	27.70600	-24.72200
O	33.71700	22.26800	-14.94500
H	33.32400	21.71400	-14.27000
H	34.53400	22.57900	-14.55400
O	36.63400	23.68400	-17.35300
H	37.37500	24.28400	-17.43300
H	36.86600	22.94100	-17.91100
O	31.77800	28.86900	-19.07500
H	31.66400	27.96800	-18.77500
H	30.92300	29.11600	-19.42700
O	40.58000	19.71200	-23.07700
H	40.23600	20.60200	-23.15300
H	40.94800	19.52200	-23.94000
O	32.55700	26.84000	-22.14500
H	33.22900	26.63100	-22.79400
H	31.73000	26.64200	-22.58400
O	38.45900	23.46600	-20.12800
H	37.81200	24.16500	-20.22200
H	39.22900	23.90000	-19.76100
O	34.30000	26.81300	-20.07700
H	33.76400	26.36200	-19.42500
H	33.71200	26.94700	-20.82000
O	30.03400	25.94800	-22.99100
H	29.95000	25.34100	-23.72600

H	29.21300	26.43900	-22.99100
O	38.46800	30.33100	-16.49000
H	37.79200	30.23100	-15.82000
H	39.29100	30.17500	-16.02600
O	24.80800	29.13900	-25.14800
H	24.84600	28.60600	-24.35500
H	24.30200	28.61300	-25.76700
O	32.18700	23.98600	-21.65200
H	32.25100	24.84700	-22.06400
H	32.12400	23.37300	-22.38500
O	31.66200	26.05800	-18.00500
H	32.28900	25.67600	-17.39100
H	30.80500	25.83900	-17.63900
O	34.77100	23.27700	-19.81600
H	35.29400	23.52300	-19.05300
H	34.55200	24.10900	-20.23600
O	35.97600	24.21800	-22.13100
H	35.63600	23.41900	-21.72900
H	35.27000	24.51800	-22.70300
O	36.62800	21.30300	-23.67800
H	37.45000	20.82900	-23.80000
H	36.87100	22.08200	-23.17800
O	29.41300	29.66200	-20.03700
H	28.67100	29.81100	-19.45100
H	29.09200	29.92300	-20.90100
O	34.89900	25.38000	-16.34300
H	34.94800	26.25600	-16.72500
H	35.44100	24.83700	-16.91600
O	37.12900	26.13300	-19.58900
H	36.25900	26.42600	-19.86000
H	37.36500	26.71700	-18.86800
O	39.83300	22.35100	-22.07200
H	40.35100	23.08700	-22.39700
H	39.47800	22.65700	-21.23700
O	36.02500	26.81800	-14.15500
H	35.92400	27.76700	-14.22400
H	35.80700	26.49000	-15.02800
O	35.88800	30.24800	-15.33900
H	35.40500	31.04800	-15.13200
H	35.97400	30.25900	-16.29200
O	27.65900	27.04200	-20.03600
H	28.30500	26.53200	-19.54700
H	27.45000	27.77900	-19.46100
O	31.99800	24.18900	-15.93800
H	32.86200	23.83300	-15.72800
H	31.42500	23.85000	-15.25100
O	2.49700	-34.60200	-33.56500
H	2.20600	-33.69700	-33.68000
H	3.13300	-34.56000	-32.85000
O	3.18500	-38.17000	-31.38500
H	3.92600	-37.63100	-31.66100
H	2.47900	-37.54300	-31.22700
O	8.66000	-36.02700	-32.78700
H	9.06900	-36.46800	-33.53200
H	7.93800	-36.60100	-32.53400
O	11.23200	-34.00800	-36.90600
H	12.05400	-34.41000	-37.18800
H	11.22800	-34.11100	-35.95400
O	6.33900	-32.17000	-30.75200
H	5.89600	-32.95700	-31.07000
H	7.00100	-31.98200	-31.41800
O	8.43700	-31.89000	-32.72400
H	8.41900	-31.06900	-33.21600
H	9.35900	-32.01500	-32.49900
O	4.89500	-34.37600	-31.88200
H	5.42700	-35.13700	-32.11200
H	4.36600	-34.66800	-31.13900
O	14.28700	-35.40100	-35.90300
H	14.48100	-34.49700	-35.65700
H	14.18700	-35.86000	-35.06900
O	9.34900	-35.25100	-30.32900
H	9.19700	-35.47900	-31.24600
H	9.33900	-34.29300	-30.31600
O	11.05800	-32.21900	-32.25100
H	10.99900	-32.90300	-32.91800
H	11.46000	-31.47700	-32.70300
O	11.06000	-34.25500	-34.02000
H	10.17300	-34.59200	-33.89700
H	11.63000	-34.98600	-33.78300
O	7.05000	-29.69600	-34.89800
H	7.76300	-29.06100	-34.97000
H	6.55900	-29.59900	-35.71400
O	2.10100	-31.94600	-34.07700
H	2.80200	-31.31300	-33.92400
H	1.63100	-31.60500	-34.83800
O	5.95000	-38.65000	-29.76500
H	5.27400	-38.75100	-29.09500
H	6.77300	-38.80600	-29.30200
O	4.69200	-27.34300	-35.53000
H	5.24400	-27.18000	-34.76500
H	5.03300	-26.75300	-36.20200
O	9.54300	-32.51700	-29.67500
H	10.08700	-31.90400	-30.16900
H	8.96200	-31.95900	-29.15800
O	9.89300	-31.59400	-36.91500
H	8.94600	-31.71400	-36.98700
H	10.24100	-32.48200	-36.83300
O	4.83900	-29.77700	-33.51500
H	5.14800	-30.02300	-32.64200
H	5.63600	-29.57300	-34.00300
O	12.86600	-33.05800	-30.33900
H	12.07600	-33.07500	-30.87800
H	12.63000	-32.51900	-29.58400
O	12.98600	-36.20800	-33.02500
H	13.03600	-36.37300	-32.08400
H	12.87700	-37.07600	-33.41600
O	10.27600	-40.22500	-31.70900
H	11.17700	-40.36500	-32.00100
H	10.35100	-40.06000	-30.77000
O	14.48800	-33.78900	-32.78100
H	14.19600	-33.42600	-31.94500
H	13.87500	-34.50200	-32.96000
O	12.73700	-38.66500	-34.31300
H	13.21200	-38.47300	-35.12100
H	11.81300	-38.60900	-34.55900
O	5.63800	-35.64700	-29.46300
H	6.55800	-35.46900	-29.26900
H	5.60300	-36.59100	-29.61500

O	3.50700	-42.16300	-27.43100
H	3.40600	-41.21400	-27.50000
H	3.28900	-42.49200	-28.30300
O	3.37000	-38.73400	-28.61400
H	2.88700	-37.93400	-28.40800
H	3.45600	-38.72300	-29.56700
O	7.66600	-34.87100	-27.81200
H	8.53500	-34.69700	-28.17300
H	7.18700	-34.05200	-27.93400
O	1.83500	-27.75100	-36.13300
H	2.73700	-27.71900	-35.81600
H	1.60800	-28.68100	-36.11800
O	19.32500	-23.16200	-38.40700
H	19.96300	-23.28200	-39.11100
H	19.20700	-22.21400	-38.34900
O	16.13400	-23.21000	-38.47100
H	15.79600	-23.32500	-39.35900
H	17.00300	-23.61000	-38.49100
O	11.99300	-21.35600	-42.48200
H	11.42900	-21.44700	-43.25000
H	11.87800	-22.17600	-42.00100
O	16.61600	-22.05600	-32.51300
H	16.60500	-22.45100	-33.38500
H	16.77800	-22.78900	-31.91900
O	9.53900	-19.86100	-39.64300
H	10.33100	-20.20200	-40.05800
H	9.77900	-19.75100	-38.72300
O	13.33700	-19.27200	-33.56500
H	13.04600	-18.36800	-33.68000
H	13.97300	-19.23100	-32.85000
O	14.26200	-21.89500	-36.97500
H	14.85900	-22.11900	-37.68900
H	14.32000	-22.63600	-36.37200
O	18.29600	-19.58600	-36.57000
H	17.99600	-18.68400	-36.67800
H	17.95100	-19.85300	-35.71800
O	11.74700	-23.13500	-40.53600
H	10.82200	-23.26700	-40.32800
H	11.99600	-22.35500	-40.03900
O	9.10800	-23.49200	-39.70200
H	9.23700	-23.23800	-38.78800
H	8.42800	-22.89900	-40.02100
O	13.27800	-12.82200	-43.60300
H	13.20000	-11.87100	-43.52400
H	12.67400	-13.16700	-42.94500
O	15.77000	-25.64900	-30.94600
H	14.99300	-25.09000	-30.95600
H	16.40900	-25.16300	-30.42500
O	14.02400	-22.84000	-31.38500
H	14.76600	-22.30200	-31.66100
H	13.31800	-22.21400	-31.22700
O	19.50000	-20.69800	-32.78700
H	19.90800	-21.13900	-33.53200
H	18.77800	-21.27200	-32.53400
O	15.85300	-25.76000	-35.41400
H	16.69900	-25.33600	-35.27100
H	15.79300	-26.41700	-34.72000
O	19.82300	-20.13700	-39.03200
H	19.19400	-19.97300	-39.73500
H	19.43500	-19.72300	-38.26100
O	19.00300	-28.10000	-33.75600
H	18.75900	-27.34600	-34.29300
H	19.27900	-27.72100	-32.92100
O	20.95700	-21.55600	-35.07800
H	21.16800	-20.91300	-35.75500
H	20.91000	-22.39000	-35.54600
O	15.37500	-20.40000	-41.76700
H	15.83900	-20.12100	-42.55700
H	15.11100	-21.30200	-41.95000
O	12.26800	-20.85300	-38.82900
H	12.83800	-20.42400	-39.46700
H	12.85900	-21.13500	-38.13100
O	12.76000	-25.68600	-30.58300
H	12.13000	-25.00500	-30.35000
H	12.65400	-25.79800	-31.52800
O	8.05500	-26.01900	-39.65700
H	7.26500	-25.76400	-40.13300
H	8.54600	-25.20400	-39.55100
O	22.07100	-18.67900	-36.90600
H	22.89300	-19.08100	-37.18800
H	22.06800	-18.78200	-35.95400
O	15.83700	-19.13000	-34.75000
H	15.90100	-18.66800	-33.91400
H	15.12800	-19.76100	-34.62200
O	6.33900	-25.76600	-37.22500
H	6.58900	-24.88200	-36.95900
H	6.87500	-25.94600	-37.99800
O	15.55200	-17.46400	-44.88200
H	15.41600	-16.61800	-44.45400
H	16.09300	-17.26200	-45.64500
O	12.03800	-31.38400	-28.22100
H	11.64500	-31.93800	-27.54600
H	12.85500	-31.07300	-27.83000
O	9.00500	-19.94600	-44.30900
H	9.51700	-19.15200	-44.15900
H	9.65700	-20.64300	-44.37600
O	10.81400	-16.82900	-42.22700
H	10.99100	-15.90800	-42.41500
H	11.61000	-17.14400	-41.79800
O	14.95500	-29.96900	-30.62900
H	15.69600	-29.36800	-30.70800
H	15.18700	-30.71100	-31.18700
O	10.09900	-24.78300	-32.35100
H	9.98500	-25.68400	-32.05000
H	9.24400	-24.53700	-32.70200
O	17.17900	-16.84100	-30.75200
H	16.73500	-17.62800	-31.07000
H	17.84000	-16.65300	-31.41800
O	11.93700	-15.31200	-36.22600
H	12.48800	-15.44000	-36.99800
H	11.09800	-15.01200	-36.57600
O	19.27700	-16.56100	-32.72400
H	19.25900	-15.74000	-33.21600
H	20.19900	-16.68600	-32.49900
O	15.73400	-19.04700	-31.88200
H	16.26700	-19.80800	-32.11200
H	15.20500	-19.33900	-31.13900
O	17.09900	-19.91800	-44.37000

H	16.57100	-20.65300	-44.68200
H	16.59800	-19.14000	-44.61600
O	22.07900	-21.72300	-39.25300
H	21.42100	-21.03300	-39.16500
H	22.88300	-21.33300	-38.91000
O	17.36700	-25.89400	-38.95700
H	16.44900	-25.67200	-39.11500
H	17.78300	-25.81900	-39.81600
O	20.09900	-16.55000	-42.14000
H	20.39100	-15.99000	-42.85800
H	20.60800	-16.25700	-41.38400
O	14.97200	-24.38600	-40.74300
H	15.02700	-25.33800	-40.81800
H	14.16100	-24.23000	-40.25900
O	15.06200	-15.77200	-41.05000
H	15.02000	-15.54600	-41.97900
H	15.97800	-16.00600	-40.90200
O	18.50900	-25.30400	-34.15200
H	18.86300	-25.07700	-33.29300
H	19.15600	-24.97200	-34.77400
O	12.40300	-28.19600	-37.36800
H	13.04200	-28.33700	-38.06500
H	11.57000	-28.48300	-37.74400
O	13.17900	-18.89600	-41.25400
H	13.97300	-19.39100	-41.45600
H	12.46800	-19.42400	-41.61700
O	17.99800	-16.51400	-36.90300
H	18.05200	-15.94500	-36.13500
H	17.13000	-16.34400	-37.26700
O	10.87800	-26.81200	-35.42000
H	11.55000	-27.02100	-36.06900
H	10.05100	-27.01000	-35.86000
O	25.12700	-20.07200	-35.90300
H	25.32000	-19.16700	-35.65700
H	25.02700	-20.53100	-35.06900
O	16.78000	-30.18600	-33.40400
H	16.13300	-29.48700	-33.49700
H	17.55100	-29.75300	-33.03600
O	20.18800	-19.92100	-30.32900
H	20.03700	-20.15000	-31.24600
H	20.17900	-18.96400	-30.31600
O	16.95700	-22.34800	-35.34400
H	17.88600	-22.19700	-35.51700
H	16.53600	-22.26000	-36.19900
O	15.75600	-18.32300	-37.57400
H	15.61700	-18.69100	-36.70200
H	14.96700	-17.80800	-37.74600
O	16.35000	-13.22200	-46.23200
H	16.52600	-13.69300	-45.41700
H	15.66400	-12.59600	-46.00400
O	17.30600	-18.94500	-40.18500
H	16.97100	-18.98500	-39.29000
H	16.80700	-19.61000	-40.65800
O	11.28100	-23.53800	-34.66000
H	11.14300	-24.27000	-34.05900
H	12.19100	-23.62700	-34.94200
O	13.90900	-23.82600	-35.17200
H	14.61900	-24.46700	-35.21300
H	13.86600	-23.57500	-34.24900
O	21.89800	-16.88900	-32.25100
H	21.83900	-17.57400	-32.91800
H	22.29900	-16.14800	-32.70300
O	12.62200	-26.84000	-33.35200
H	12.08500	-27.29100	-32.70000
H	12.03300	-26.70500	-34.09500
O	10.91700	-10.07300	-39.45800
H	11.15600	-9.44100	-38.78000
H	10.07400	-10.42400	-39.17100
O	21.89900	-18.92500	-34.02000
H	21.01200	-19.26300	-33.89700
H	22.46900	-19.65700	-33.78300
O	8.35600	-27.70400	-36.26700
H	8.27100	-28.31100	-37.00200
H	7.53400	-27.21300	-36.26600
O	15.42900	-14.26500	-37.05400
H	15.35600	-13.46000	-36.54200
H	14.98700	-14.06600	-37.87900
O	15.21800	-15.02800	-43.54300
H	14.43500	-14.48600	-43.45000
H	15.91800	-14.51100	-43.14400
O	17.88900	-14.36700	-34.89800
H	18.60200	-13.73200	-34.97000
H	17.39800	-14.27000	-35.71400
O	12.94000	-16.61700	-34.07700
H	13.64100	-15.98400	-33.92400
H	12.47000	-16.27600	-34.83800
O	20.74100	-24.06300	-36.25200
H	20.08800	-23.81500	-36.90700
H	21.11900	-24.87600	-36.58600
O	17.70100	-14.02700	-42.14900
H	17.88400	-14.84500	-41.68600
H	18.52500	-13.81000	-42.58700
O	18.03600	-15.10000	-44.88800
H	18.56400	-15.61100	-45.50100
H	18.29800	-15.41600	-44.02300
O	16.78900	-23.32100	-29.76500
H	16.11300	-23.42100	-29.09500
H	17.61200	-23.47700	-29.30200
O	8.53700	-17.08400	-37.14400
H	9.12300	-17.82900	-37.01100
H	8.34000	-16.77300	-36.26000
O	11.39100	-14.04800	-42.12800
H	10.72200	-13.56400	-42.61100
H	11.25200	-13.80200	-41.21400
O	3.12900	-24.51300	-38.42400
H	3.16700	-25.04700	-37.63000
H	2.62300	-25.03900	-39.04300
O	15.53200	-12.01400	-35.53000
H	16.08300	-11.85100	-34.76500
H	15.87200	-11.42400	-36.20200
O	10.50800	-29.66700	-34.92700
H	10.57200	-28.80500	-35.34000
H	10.44500	-30.27900	-35.66000
O	9.98300	-27.59400	-31.28000
H	10.61000	-27.97700	-30.66600
H	9.12600	-27.81300	-30.91500
O	20.38300	-17.18800	-29.67500
H	20.92700	-16.57500	-30.16900

H	19.80100	-16.63000	-29.15800
O	18.66800	-14.06600	-38.54300
H	18.54400	-14.86700	-39.05200
H	19.32600	-13.57100	-39.03200
O	10.28900	-27.71100	-39.36600
H	9.56800	-27.09700	-39.50500
H	11.07900	-27.17500	-39.44400
O	20.73200	-16.26500	-36.91500
H	19.78500	-16.38400	-36.98700
H	21.08000	-17.15300	-36.83300
O	13.09200	-30.37600	-33.09100
H	13.61500	-30.12900	-32.32900
H	12.87300	-29.54400	-33.51100
O	17.73700	-16.47000	-40.71500
H	17.58900	-17.39800	-40.53400
H	18.56200	-16.44800	-41.19900
O	21.34000	-15.64700	-39.62800
H	22.26500	-15.85300	-39.76600
H	21.17200	-15.89500	-38.71900
O	14.29700	-29.43400	-35.40700
H	13.95700	-30.23300	-35.00400
H	13.59100	-29.13400	-35.97900
O	14.94900	-32.34900	-36.95400
H	15.77100	-32.82300	-37.07600
H	15.19300	-31.57000	-36.45400
O	12.47300	-25.65700	-39.14500
H	12.32700	-25.34300	-38.25200
H	11.99500	-25.03900	-39.69700
O	14.33200	-28.66700	-39.09600
H	14.31800	-29.56300	-39.43300
H	15.05000	-28.66000	-38.46300
O	16.18500	-28.23300	-37.05100
H	15.92700	-27.31200	-37.05300
H	15.65300	-28.63000	-36.36100
O	6.94700	-23.11700	-36.20600
H	6.40300	-22.34100	-36.07400
H	7.82500	-22.77000	-36.36400
O	7.73400	-23.99000	-33.31300
H	6.99200	-23.84200	-32.72600
H	7.41300	-23.72900	-34.17600
O	15.67800	-14.44800	-33.51500
H	15.98700	-14.69300	-32.64200
H	16.47600	-14.24400	-34.00300
O	15.70000	-12.29400	-41.28400
H	15.26100	-12.78700	-40.59100
H	16.45100	-12.83700	-41.52500
O	8.17900	-16.10300	-41.28200
H	9.00500	-16.40300	-41.66200
H	7.82900	-16.87300	-40.83300
O	13.14800	-16.75100	-39.21200
H	12.52600	-17.41400	-39.51200
H	13.81800	-16.72500	-39.89500
O	6.38000	-18.18500	-40.32300
H	5.54400	-18.61200	-40.50400
H	6.35300	-17.99100	-39.38600
O	13.22000	-28.27200	-29.61900
H	13.26900	-27.39600	-30.00100
H	13.76200	-28.81500	-30.19200
O	23.70600	-17.72800	-30.33900
H	22.91500	-17.74600	-30.87800
H	23.46900	-17.19000	-29.58400
O	23.82500	-20.87900	-33.02500
H	23.87500	-21.04400	-32.08400
H	23.71700	-21.74600	-33.41600
O	12.70900	-18.20000	-45.73600
H	12.85800	-17.51700	-46.38900
H	13.51600	-18.22000	-45.22100
O	21.11600	-24.89600	-31.70900
H	22.01700	-25.03500	-32.00100
H	21.19100	-24.73100	-30.77000
O	15.45000	-27.51900	-32.86400
H	14.58000	-27.22600	-33.13600
H	15.68600	-26.93500	-32.14300
O	25.32700	-18.45900	-32.78100
H	25.03600	-18.09600	-31.94500
H	24.71400	-19.17300	-32.96000
O	23.57600	-23.33600	-34.31300
H	24.05100	-23.14300	-35.12100
H	22.65200	-23.28000	-34.55900
O	18.15400	-31.30100	-35.34800
H	18.67200	-30.56500	-35.67300
H	17.79900	-30.99500	-34.51300
O	20.29800	-29.37400	-35.89400
H	19.70200	-29.09400	-36.59000
H	20.01200	-28.88500	-35.12300
O	16.47700	-20.31800	-29.46300
H	17.39700	-20.14000	-29.26900
H	16.44200	-21.26200	-29.61500
O	9.47300	-22.40700	-36.82100
H	9.69200	-21.47600	-36.80200
H	9.88600	-22.76700	-36.03600
O	14.34600	-26.83400	-27.43100
H	14.24600	-25.88500	-27.50000
H	14.12800	-27.16200	-28.30300
O	5.56400	-21.50300	-38.56900
H	4.87000	-21.91800	-39.08200
H	6.31100	-21.46100	-39.16500
O	14.20900	-23.40400	-28.61400
H	13.72600	-22.60400	-28.40800
H	14.29500	-23.39400	-29.56700
O	7.47800	-21.57200	-40.69200
H	7.45500	-21.47300	-41.64400
H	7.78800	-20.72500	-40.37000
O	10.13700	-19.51400	-36.57700
H	11.09100	-19.54600	-36.64400
H	9.93800	-19.97500	-35.76200
O	10.72600	-20.94400	-34.01000
H	11.54600	-20.44900	-33.98700
H	11.00000	-21.86100	-34.03900
O	8.49200	-13.97200	-39.56000
H	8.35700	-14.73200	-40.12700
H	8.07500	-13.24800	-40.02700
O	11.64800	-13.07500	-39.34900
H	11.53200	-12.13100	-39.45100
H	10.82800	-13.37800	-38.96000
O	5.98000	-26.61000	-33.31100
H	6.62600	-27.12000	-32.82200
H	5.77200	-25.87300	-32.73700

O	18.50500	-19.54200	-27.81200
H	19.37400	-19.36800	-28.17300
H	18.02600	-18.72300	-27.93400
O	12.67400	-12.42200	-36.13300
H	13.57700	-12.39000	-35.81600
H	12.44800	-13.35200	-36.11800
O	14.42900	-13.66900	-39.47200
H	13.47900	-13.66900	-39.58500
H	14.73300	-14.41600	-39.98800
O	10.32000	-29.46300	-29.21400
H	11.18300	-29.82000	-29.00300
H	9.74600	-29.80200	-28.52600
O	30.16500	-7.83300	-38.40700
H	30.80200	-7.95200	-39.11100
H	30.04700	-6.88500	-38.34900
O	26.97400	-7.88000	-38.47100
H	26.63500	-7.99600	-39.35900
H	27.84200	-8.28100	-38.49100
O	22.83200	-6.02700	-42.48200
H	22.26900	-6.11800	-43.25000
H	22.71700	-6.84700	-42.00100
O	27.45500	-6.72700	-32.51300
H	27.44400	-7.12200	-33.38500
H	27.61800	-7.46000	-31.91900
O	20.37900	-4.53200	-39.64300
H	21.17100	-4.87300	-40.05800
H	20.61800	-4.42200	-38.72300
O	24.17600	-3.94300	-33.56500
H	23.88500	-3.03900	-33.68000
H	24.81200	-3.90200	-32.85000
O	25.10200	-6.56500	-36.97500
H	25.69800	-6.79000	-37.68900
H	25.15900	-7.30700	-36.37200
O	29.13500	-4.25700	-36.57000
H	28.83500	-3.35500	-36.67800
H	28.79000	-4.52400	-35.71800
O	22.58600	-7.80600	-40.53600
H	21.66200	-7.93800	-40.32800
H	22.83500	-7.02600	-40.03900
O	19.94700	-8.16200	-39.70200
H	20.07700	-7.90900	-38.78800
H	19.26700	-7.57000	-40.02100
O	26.60900	-10.32000	-30.94600
H	25.83200	-9.76000	-30.95600
H	27.24800	-9.83400	-30.42500
O	24.86400	-7.51100	-31.38500
H	25.60500	-6.97300	-31.66100
H	24.15700	-6.88500	-31.22700
O	30.33900	-5.36800	-32.78700
H	30.74800	-5.81000	-33.53200
H	29.61700	-5.94300	-32.53400
O	26.69200	-10.43100	-35.41400
H	27.53800	-10.00700	-35.27100
H	26.63200	-11.08800	-34.72000
O	30.66200	-4.80800	-39.03200
H	30.03300	-4.64400	-39.73500
H	30.27500	-4.39400	-38.26100
O	29.84200	-12.77100	-33.75600
H	29.59800	-12.01600	-34.29300
H	30.11800	-12.39200	-32.92100
O	31.79600	-6.22700	-35.07800
H	32.00800	-5.58400	-35.75500
H	31.74900	-7.06100	-35.54600
O	26.21500	-5.07100	-41.76700
H	26.67900	-4.79200	-42.55700
H	25.95100	-5.97200	-41.95000
O	23.10700	-5.52400	-38.82900
H	23.67700	-5.09500	-39.46700
H	23.69800	-5.80500	-38.13100
O	23.60000	-10.35700	-30.58300
H	22.97000	-9.67600	-30.35000
H	23.49300	-10.46900	-31.52800
O	18.89400	-10.69000	-39.65700
H	18.10400	-10.43400	-40.13300
H	19.38500	-9.87500	-39.55100
O	32.91100	-3.35000	-36.90600
H	33.73200	-3.75200	-37.18800
H	32.90700	-3.45300	-35.95400
O	20.84600	-10.32600	-42.69800
H	20.04900	-10.21400	-42.18000
H	20.61800	-9.98700	-43.56400
O	26.67600	-3.80100	-34.75000
H	26.74100	-3.33900	-33.91400
H	25.96700	-4.43100	-34.62200
O	17.17800	-10.43700	-37.22500
H	17.42900	-9.55200	-36.95900
H	17.71400	-10.61700	-37.99800
O	22.87800	-16.05500	-28.22100
H	22.48500	-16.60900	-27.54600
H	23.69400	-15.74400	-27.83000
O	19.84400	-4.61700	-44.30900
H	20.35700	-3.82300	-44.15900
H	20.49600	-5.31400	-44.37600
O	21.65400	-1.50000	-42.22700
H	21.83000	-0.57800	-42.41500
H	22.44900	-1.81400	-41.79800
O	25.79500	-14.63900	-30.62900
H	26.53600	-14.03900	-30.70800
H	26.02600	-15.38200	-31.18700
O	20.93900	-9.45400	-32.35100
H	20.82400	-10.35500	-32.05000
H	20.08300	-9.20700	-32.70200
O	28.01800	-1.51200	-30.75200
H	27.57400	-2.29800	-31.07000
H	28.68000	-1.32400	-31.41800
O	22.77700	0.01800	-36.22600
H	23.32700	-0.11100	-36.99800
H	21.93800	0.31700	-36.57600
O	30.11600	-1.23200	-32.72400
H	30.09800	-0.41100	-33.21600
H	31.03800	-1.35600	-32.49900
O	26.57300	-3.71700	-31.88200
H	27.10600	-4.47900	-32.11200
H	26.04500	-4.01000	-31.13900
O	17.80900	-6.28800	-43.53300
H	18.64600	-5.87800	-43.75000
H	17.98700	-7.22800	-43.56300
O	32.91800	-6.39400	-39.25300

H	32.26000	-5.70400	-39.16500
H	33.72200	-6.00400	-38.91000
O	22.67100	-13.92700	-42.00900
H	23.24500	-14.14900	-42.74200
H	23.25800	-13.85800	-41.25600
O	28.20700	-10.56400	-38.95700
H	27.28900	-10.34300	-39.11500
H	28.62300	-10.49000	-39.81600
O	17.83200	-8.94900	-42.86200
H	17.63400	-9.54200	-43.58700
H	17.27600	-9.25400	-42.14500
O	25.81100	-9.05700	-40.74300
H	25.86600	-10.00900	-40.81800
H	25.00000	-8.90100	-40.25900
O	23.75000	-10.43900	-42.98400
H	22.93700	-10.13900	-42.57700
H	24.24600	-10.83300	-42.26600
O	25.90100	-0.44300	-41.05000
H	25.85900	-0.21700	-41.97900
H	26.81800	-0.67700	-40.90200
O	29.34800	-9.97500	-34.15200
H	29.70300	-9.74800	-33.29300
H	29.99500	-9.64300	-34.77400
O	23.24200	-12.86700	-37.36800
H	23.88200	-13.00800	-38.06500
H	22.41000	-13.15400	-37.74400
O	24.01800	-3.56700	-41.25400
H	24.81200	-4.06200	-41.45600
H	23.30700	-4.09500	-41.61700
O	28.83700	-1.18500	-36.90300
H	28.89100	-0.61600	-36.13500
H	27.96900	-1.01500	-37.26700
O	20.32600	-9.42300	-45.17800
H	19.39600	-9.48400	-44.96100
H	20.42200	-9.94500	-45.97400
O	20.24500	-12.89700	-42.59000
H	20.50500	-11.98200	-42.69900
H	21.06600	-13.36500	-42.44100
O	21.71700	-11.48300	-35.42000
H	22.39000	-11.69200	-36.06900
H	20.89000	-11.68100	-35.86000
O	35.96600	-4.74300	-35.90300
H	36.16000	-3.83800	-35.65700
H	35.86600	-5.20200	-35.06900
O	27.62000	-14.85700	-33.40400
H	26.97300	-14.15800	-33.49700
H	28.39000	-14.42300	-33.03600
O	31.02800	-4.59200	-30.32900
H	30.87600	-4.82100	-31.24600
H	31.01800	-3.63500	-30.31600
O	27.79600	-7.01900	-35.34400
H	28.72500	-6.86800	-35.51700
H	27.37500	-6.93100	-36.19900
O	26.59500	-2.99400	-37.57400
H	26.45600	-3.36200	-36.70200
H	25.80700	-2.47900	-37.74600
O	28.14600	-3.61600	-40.18500
H	27.81100	-3.65600	-39.29000
H	27.64600	-4.28100	-40.65800
O	22.12000	-8.20900	-34.66000
H	21.98200	-8.94100	-34.05900
H	23.03000	-8.29800	-34.94200
O	24.74800	-8.49700	-35.17200
H	25.45800	-9.13800	-35.21300
H	24.70500	-8.24600	-34.24900
O	32.73700	-1.56000	-32.25100
H	32.67800	-2.24400	-32.91800
H	33.13900	-0.81800	-32.70300
O	23.46100	-11.51000	-33.35200
H	22.92500	-11.96100	-32.70000
H	22.87300	-11.37600	-34.09500
O	21.75600	5.25600	-39.45800
H	21.99600	5.88800	-38.78000
H	20.91300	4.90500	-39.17100
O	32.73900	-3.59600	-34.02000
H	31.85100	-3.93400	-33.89700
H	33.30800	-4.32800	-33.78300
O	14.39200	-7.26400	-40.69500
H	15.07900	-7.57800	-41.28300
H	14.23600	-6.36000	-40.96900
O	19.19500	-12.37500	-36.26700
H	19.11100	-12.98200	-37.00200
H	18.37300	-11.88400	-36.26600
O	26.26900	1.06400	-37.05400
H	26.19600	1.86900	-36.54200
H	25.82600	1.26300	-37.87900
O	28.72900	0.96300	-34.89800
H	29.44100	1.59700	-34.97000
H	28.23800	1.05900	-35.71400
O	23.78000	-1.28800	-34.07700
H	24.48100	-0.65400	-33.92400
H	23.30900	-0.94700	-34.83800
O	31.58000	-8.73400	-36.25200
H	30.92700	-8.48600	-36.90700
H	31.95800	-9.54700	-36.58600
O	27.62900	-7.99200	-29.76500
H	26.95300	-8.09200	-29.09500
H	28.45100	-8.14800	-29.30200
O	19.37700	-1.75400	-37.14400
H	19.96200	-2.50000	-37.01100
H	19.17900	-1.44400	-36.26000
O	22.23000	1.28100	-42.12800
H	21.56100	1.76500	-42.61100
H	22.09200	1.52800	-41.21400
O	13.96800	-9.18400	-38.42400
H	14.00700	-9.71700	-37.63000
H	13.46200	-9.71000	-39.04300
O	26.37100	3.31500	-35.53000
H	26.92300	3.47800	-34.76500
H	26.71200	3.90600	-36.20200
O	21.34700	-14.33700	-34.92700
H	21.41200	-13.47600	-35.34000
H	21.28400	-14.95000	-35.66000
O	20.82300	-12.26500	-31.28000
H	21.45000	-12.64700	-30.66600
H	19.96500	-12.48400	-30.91500
O	31.22200	-1.85900	-29.67500
H	31.76600	-1.24600	-30.16900

H	30.64100	-1.30100	-29.15800
O	17.59900	-10.45700	-45.34800
H	17.89500	-11.36600	-45.40200
H	16.69800	-10.47900	-45.67000
O	29.50800	1.26300	-38.54300
H	29.38300	0.46200	-39.05200
H	30.16600	1.75800	-39.03200
O	21.12900	-12.38200	-39.36600
H	20.40700	-11.76800	-39.50500
H	21.91800	-11.84600	-39.44400
O	31.57100	-0.93600	-36.91500
H	30.62400	-1.05500	-36.98700
H	31.92000	-1.82400	-36.83300
O	23.93100	-15.04600	-33.09100
H	24.45500	-14.80000	-32.32900
H	23.71200	-14.21400	-33.51100
O	19.32800	-12.77600	-45.22400
H	18.99900	-13.65600	-45.40700
H	19.68200	-12.83100	-44.33700
O	28.57600	-1.14100	-40.71500
H	28.42800	-2.06900	-40.53400
H	29.40100	-1.11900	-41.19900
O	25.13700	-14.10500	-35.40700
H	24.79600	-14.90400	-35.00400
H	24.43000	-13.80500	-35.97900
O	25.78800	-17.02000	-36.95400
H	26.61100	-17.49400	-37.07600
H	26.03200	-16.24100	-36.45400
O	23.31200	-10.32800	-39.14500
H	23.16600	-10.01400	-38.25200
H	22.83500	-9.71000	-39.69700
O	25.17200	-13.33800	-39.09600
H	25.15800	-14.23400	-39.43300
H	25.88900	-13.33100	-38.46300
O	27.02400	-12.90400	-37.05100
H	26.76600	-11.98200	-37.05300
H	26.49300	-13.30100	-36.36100
O	17.78700	-7.78800	-36.20600
H	17.24200	-7.01200	-36.07400
H	18.66500	-7.44100	-36.36400
O	18.57300	-8.66100	-33.31300
H	17.83100	-8.51200	-32.72600
H	18.25200	-8.40000	-34.17600
O	26.51800	0.88100	-33.51500
H	26.82600	0.63600	-32.64200
H	27.31500	1.08600	-34.00300
O	19.01900	-0.77400	-41.28200
H	19.84500	-1.07400	-41.66200
H	18.66800	-1.54300	-40.83300
O	23.98700	-1.42100	-39.21200
H	23.36500	-2.08400	-39.51200
H	24.65700	-1.39500	-39.89500
O	17.22000	-2.85500	-40.32300
H	16.38300	-3.28300	-40.50400
H	17.19200	-2.66200	-39.38600
O	24.05900	-12.94300	-29.61900
H	24.10800	-12.06700	-30.00100
H	24.60200	-13.48600	-30.19200
O	34.54500	-2.39900	-30.33900
H	33.75500	-2.41700	-30.87800
H	34.30800	-1.86000	-29.58400
O	34.66500	-5.55000	-33.02500
H	34.71500	-5.71500	-32.08400
H	34.55600	-6.41700	-33.41600
O	31.95500	-9.56700	-31.70900
H	32.85600	-9.70600	-32.00100
H	32.03000	-9.40100	-30.77000
O	26.28900	-12.19000	-32.86400
H	25.42000	-11.89700	-33.13600
H	26.52500	-11.60600	-32.14300
O	36.16700	-3.13000	-32.78100
H	35.87500	-2.76700	-31.94500
H	35.55400	-3.84400	-32.96000
O	34.41500	-8.00700	-34.31300
H	34.89100	-7.81400	-35.12100
H	33.49200	-7.95100	-34.55900
O	21.30400	-6.84500	-44.73000
H	21.05800	-7.74800	-44.53200
H	21.47300	-6.84300	-45.67200
O	28.99300	-15.97200	-35.34800
H	29.51100	-15.23600	-35.67300
H	28.63900	-15.66600	-34.51300
O	31.13700	-14.04500	-35.89400
H	30.54200	-13.76400	-36.59000
H	30.85200	-13.55500	-35.12300
O	27.31600	-4.98800	-29.46300
H	28.23700	-4.81100	-29.26900
H	27.28100	-5.93300	-29.61500
O	16.53500	-9.79500	-40.53800
H	16.21500	-10.64800	-40.83100
H	15.98800	-9.58000	-39.78200
O	20.31300	-7.07800	-36.82100
H	20.53100	-6.14700	-36.80200
H	20.72500	-7.43700	-36.03600
O	25.18500	-11.50500	-27.43100
H	25.08500	-10.55600	-27.50000
H	24.96800	-11.83300	-28.30300
O	16.40300	-6.17400	-38.56900
H	15.71000	-6.58900	-39.08200
H	17.15100	-6.13200	-39.16500
O	25.04900	-8.07500	-28.61400
H	24.56500	-7.27500	-28.40800
H	25.13500	-8.06400	-29.56700
O	18.31700	-6.24200	-40.69200
H	18.29400	-6.14400	-41.64400
H	18.62700	-5.39600	-40.37000
O	20.97600	-4.18500	-36.57700
H	21.93000	-4.21600	-36.64400
H	20.77700	-4.64600	-35.76200
O	21.56600	-5.61500	-34.01000
H	22.38500	-5.12000	-33.98700
H	21.84000	-6.53100	-34.03900
O	19.33100	1.35700	-39.56000
H	19.19600	0.59700	-40.12700
H	18.91400	2.08100	-40.02700
O	22.48800	2.25400	-39.34900
H	22.37100	3.19900	-39.45100
H	21.66700	1.95100	-38.96000

O	16.81900	-11.28100	-33.31100
H	17.46500	-11.79100	-32.82200
H	16.61100	-10.54400	-32.73700
O	29.34500	-4.21300	-27.81200
H	30.21400	-4.03900	-28.17300
H	28.86500	-3.39300	-27.93400
O	23.51300	2.90700	-36.13300
H	24.41600	2.93900	-35.81600
H	23.28700	1.97700	-36.11800
O	25.26900	1.66000	-39.47200
H	24.31800	1.66000	-39.58500
H	25.57200	0.91300	-39.98800
O	21.15900	-14.13400	-29.21400
H	22.02200	-14.49000	-29.00300
H	20.58600	-14.47300	-28.52600
O	37.44800	5.00900	-30.94600
H	36.67200	5.56900	-30.95600
H	38.08700	5.49500	-30.42500
O	35.70300	7.81800	-31.38500
H	36.44400	8.35600	-31.66100
H	34.99700	8.44500	-31.22700
O	34.43900	4.97200	-30.58300
H	33.80900	5.65400	-30.35000
H	34.33300	4.86000	-31.52800
O	28.01700	4.89200	-37.22500
H	28.26800	5.77700	-36.95900
H	28.55300	4.71300	-37.99800
O	33.71700	-0.72600	-28.22100
H	33.32400	-1.27900	-27.54600
H	34.53400	-0.41500	-27.83000
O	36.63400	0.69000	-30.62900
H	37.37500	1.29000	-30.70800
H	36.86600	-0.05200	-31.18700
O	31.77800	5.87500	-32.35100
H	31.66400	4.97400	-32.05000
H	30.92300	6.12200	-32.70200
O	32.55700	3.84600	-35.42000
H	33.22900	3.63800	-36.06900
H	31.73000	3.64800	-35.86000
O	38.45900	0.47200	-33.40400
H	37.81200	1.17100	-33.49700
H	39.22900	0.90600	-33.03600
O	34.30000	3.81900	-33.35200
H	33.76400	3.36800	-32.70000
H	33.71200	3.95300	-34.09500
O	30.03400	2.95400	-36.26700
H	29.95000	2.34700	-37.00200
H	29.21300	3.44500	-36.26600
O	38.46800	7.33800	-29.76500
H	37.79200	7.23700	-29.09500
H	39.29100	7.18200	-29.30200
O	24.80800	6.14500	-38.42400
H	24.84600	5.61200	-37.63000
H	24.30200	5.61900	-39.04300
O	32.18700	0.99200	-34.92700
H	32.25100	1.85300	-35.34000
H	32.12400	0.37900	-35.66000
O	31.66200	3.06400	-31.28000
H	32.28900	2.68200	-30.66600
H	30.80500	2.84600	-30.91500
O	34.77100	0.28300	-33.09100
H	35.29400	0.52900	-32.32900
H	34.55200	1.11500	-33.51100
O	35.97600	1.22400	-35.40700
H	35.63600	0.42500	-35.00400
H	35.27000	1.52400	-35.97900
O	36.62800	-1.69100	-36.95400
H	37.45000	-2.16500	-37.07600
H	36.87100	-0.91200	-36.45400
O	29.41300	6.66900	-33.31300
H	28.67100	6.81700	-32.72600
H	29.09200	6.92900	-34.17600
O	34.89900	2.38600	-29.61900
H	34.94800	3.26200	-30.00100
H	35.44100	1.84400	-30.19200
O	37.12900	3.13900	-32.86400
H	36.25900	3.43200	-33.13600
H	37.36500	3.72300	-32.14300
O	39.83300	-0.64300	-35.34800
H	40.35100	0.09300	-35.67300
H	39.47800	-0.33700	-34.51300
O	38.15600	10.34100	-29.46300
H	39.07600	10.51900	-29.26900
H	38.12100	9.39600	-29.61500
O	36.02500	3.82400	-27.43100
H	35.92400	4.77400	-27.50000
H	35.80700	3.49600	-28.30300
O	35.88800	7.25400	-28.61400
H	35.40500	8.05400	-28.40800
H	35.97400	7.26500	-29.56700
O	27.65900	4.04800	-33.31100
H	28.30500	3.53800	-32.82200
H	27.45000	4.78500	-32.73700
O	40.18400	11.11600	-27.81200
H	41.05300	11.29000	-28.17300
H	39.70500	11.93600	-27.93400
O	31.99800	1.19500	-29.21400
H	32.86200	0.83900	-29.00300
H	31.42500	0.85600	-28.52600
O	-40.75600	20.48800	12.67300
H	-40.02400	19.91800	12.90700
H	-41.51200	20.10000	13.11400
O	-41.47500	17.12100	15.99000
H	-42.11700	17.41500	15.34400
H	-41.97900	17.00700	16.79500
O	-41.33500	26.78600	11.42400
H	-42.02000	27.16500	10.87400
H	-41.25000	27.39500	12.15700
O	-34.87400	23.65300	12.54400
H	-35.62800	23.08300	12.38900
H	-34.74000	23.61900	13.49100
O	-41.19700	24.79100	7.19200
H	-41.97400	24.45200	6.74800
H	-40.51800	24.79600	6.51800
O	-40.79800	23.80600	9.65500
H	-40.93600	23.99600	8.72700
H	-41.15400	24.56700	10.11100
O	-40.77100	25.87300	14.66600

H	-40.33700	25.78100	15.51400
H	-40.05800	25.84900	14.02800
O	-39.90900	25.91600	17.19900
H	-39.71600	26.82000	17.44500
H	-40.00900	25.45600	18.03300
O	-38.65500	22.47300	13.84400
H	-38.66600	23.35500	13.47300
H	-39.52400	22.12000	13.64900
O	-42.03800	21.36800	10.31100
H	-41.57200	22.20100	10.23700
H	-41.58700	20.90200	11.01500
O	-38.05300	23.62300	10.44900
H	-37.61000	22.87400	10.05100
H	-38.93000	23.61400	10.06600
O	-41.02000	27.62600	7.45800
H	-40.49500	27.96500	6.73300
H	-41.10800	26.69100	7.27400
O	-41.21100	25.10800	20.07700
H	-41.16100	24.94300	21.01800
H	-41.32000	24.24100	19.68600
O	-39.70900	27.52800	20.32100
H	-40.00100	27.89100	21.15700
H	-40.32200	26.81500	20.14200
O	-38.51000	25.09300	12.84100
H	-37.67500	25.53200	13.00500
H	-38.37400	24.61700	12.02200
O	-41.46000	22.65100	18.78900
H	-40.98500	22.84400	17.98100
H	-42.38400	22.70700	18.54300
O	-40.28100	22.74900	16.13600
H	-40.31400	21.81000	16.32200
H	-39.67100	22.82700	15.40200
O	-40.13200	19.54000	16.18200
H	-40.64800	18.75600	15.99500
H	-39.24800	19.21200	16.34600
O	-42.47100	13.28400	12.14100
H	-42.66800	12.97500	13.02600
H	-41.86100	14.01000	12.27100
O	-34.46400	23.81000	15.27600
H	-33.73300	23.54900	15.83700
H	-35.07300	23.07300	15.31700
O	-36.38200	21.95100	15.87400
H	-37.10900	21.81700	15.26500
H	-36.79700	22.25900	16.68000
O	-41.20200	17.45900	12.17000
H	-40.42600	17.94900	12.44200
H	-41.92700	17.88500	12.62700
O	-38.76400	18.17200	13.22700
H	-38.17000	17.93200	12.51700
H	-38.69200	17.45300	13.85500
O	-40.76900	18.73900	9.49400
H	-41.02500	18.33900	10.32500
H	-41.39300	19.45400	9.36900
O	-36.45700	21.47900	12.00200
H	-37.01700	21.54800	12.77500
H	-37.06300	21.30300	11.28100
O	-32.39400	29.52900	17.13300
H	-32.72600	30.39900	16.90900
H	-32.99000	29.21300	17.81200
O	-30.63500	32.45000	15.99000
H	-31.27800	32.74400	15.34400
H	-31.14000	32.33600	16.79500
O	-35.29600	27.37600	16.75000
H	-35.63900	28.26600	16.67400
H	-34.92800	27.18600	15.88700
O	-36.21600	29.74700	14.71500
H	-36.72000	29.71000	13.90100
H	-35.92300	30.65600	14.77400
O	-39.97200	29.83100	13.72100
H	-39.10700	29.63500	13.36200
H	-39.90400	29.60200	14.64800
O	-40.06300	28.93500	9.82000
H	-40.71700	28.62000	9.19600
H	-40.10500	28.31300	10.54600
O	-33.49700	26.63600	11.85200
H	-33.19100	25.77500	11.56400
H	-32.77200	27.22700	11.65100
O	-37.41600	31.13100	19.69800
H	-38.06400	31.83000	19.60400
H	-36.64600	31.56400	20.06600
O	-35.66900	32.09800	12.20200
H	-35.17200	31.34500	11.88200
H	-36.44000	32.13400	11.63600
O	-33.22700	29.06500	14.35000
H	-34.14100	29.29500	14.51800
H	-32.79800	29.14900	15.20100
O	-39.24800	28.96800	16.14800
H	-38.42500	28.49400	16.02600
H	-39.00400	29.74700	16.64800
O	-37.59200	29.21700	12.41100
H	-37.57000	29.28100	11.45600
H	-37.17500	28.37700	12.60500
O	-36.04300	30.01500	17.75400
H	-35.52500	30.75200	17.42900
H	-36.39700	30.32200	18.58900
O	-35.79200	26.43500	13.27400
H	-35.51200	25.76800	13.90000
H	-35.00700	26.62800	12.76200
O	-33.89900	31.94300	17.20800
H	-34.49400	32.22300	16.51200
H	-34.18400	32.43200	17.97900
O	-29.29300	34.86900	16.18200
H	-29.80900	34.08500	15.99500
H	-28.40900	34.54100	16.34600
O	-31.63100	28.61300	12.14100
H	-31.82900	28.30500	13.02600
H	-31.02200	29.33900	12.27100
O	-37.80300	29.41600	8.26800
H	-38.61300	29.07100	8.64400
H	-37.11500	29.10700	8.85700
O	-35.87400	28.49400	9.89100
H	-35.32100	28.91300	10.55000
H	-35.63700	27.56700	9.93300
O	-32.49600	30.60400	9.39900
H	-33.07600	31.26200	9.01700
H	-32.76300	29.78200	8.98700
O	-34.04600	30.00400	11.60100
H	-33.54600	30.11600	10.79200

H	-33.40200	29.70100	12.24100
O	-33.71400	32.76400	8.45400
H	-34.60100	33.04900	8.23600
H	-33.29800	32.60300	7.60700
O	-30.36200	32.78900	12.17000
H	-29.58600	33.27800	12.44200
H	-31.08700	33.21400	12.62700
O	-27.92500	33.50100	13.22700
H	-27.33100	33.26100	12.51700
H	-27.85200	32.78200	13.85500
O	-39.69700	28.97100	5.54300
H	-39.70900	29.91500	5.70000
H	-38.80400	28.78800	5.24900
O	-35.88100	25.56500	9.79800
H	-36.64800	25.01100	9.94300
H	-35.47000	25.63700	10.65900
O	-40.75600	-2.50600	-0.60200
H	-40.02400	-3.07600	-0.36900
H	-41.51200	-2.89400	-0.16200
O	-41.47500	-5.87300	2.71400
H	-42.11700	-5.57900	2.06800
H	-41.97900	-5.98700	3.52000
O	-41.33500	3.79200	-1.85100
H	-42.02000	4.17200	-2.40200
H	-41.25000	4.40100	-1.11800
O	-34.87400	0.65900	-0.73100
H	-35.62800	0.09000	-0.88700
H	-34.74000	0.62500	0.21600
O	-41.19700	1.79700	-6.08300
H	-41.97400	1.45800	-6.52800
H	-40.51800	1.80300	-6.75800
O	-40.79800	0.81200	-3.62100
H	-40.93600	1.00200	-4.54900
H	-41.15400	1.57400	-3.16400
O	-40.77100	2.87900	1.39000
H	-40.33700	2.78700	2.23900
H	-40.05800	2.85500	0.75200
O	-39.90900	2.92200	3.92400
H	-39.71600	3.82600	4.16900
H	-40.00900	2.46200	4.75700
O	-38.65500	-0.52100	0.56800
H	-38.66600	0.36200	0.19800
H	-39.52400	-0.87400	0.37400
O	-42.03800	-1.62600	-2.96500
H	-41.57200	-0.79300	-3.03900
H	-41.58700	-2.09200	-2.26000
O	-38.05300	0.62900	-2.82700
H	-37.61000	-0.12000	-3.22500
H	-38.93000	0.62000	-3.20900
O	-41.02000	4.63200	-5.81700
H	-40.49500	4.97100	-6.54200
H	-41.10800	3.69700	-6.00100
O	-41.33100	5.26500	9.48800
H	-42.12100	5.24800	8.94800
H	-41.56700	5.80400	10.24300
O	-41.21100	2.11500	6.80100
H	-41.16100	1.95000	7.74300
H	-41.32000	1.24700	6.41100
O	-39.70900	4.53400	7.04500
H	-40.00100	4.89700	7.88200
H	-40.32200	3.82100	6.86700
O	-38.51000	2.10000	-0.43400
H	-37.67500	2.53900	-0.27100
H	-38.37400	1.62400	-1.25400
O	-41.46000	-0.34200	5.51300
H	-40.98500	-0.15000	4.70500
H	-42.38400	-0.28600	5.26800
O	-40.28100	-0.24500	2.86000
H	-40.31400	-1.18400	3.04600
H	-39.67100	-0.16700	2.12700
O	-40.13200	-3.45400	2.90600
H	-40.64800	-4.23800	2.71900
H	-39.24800	-3.78200	3.07100
O	-42.47100	-9.71000	-1.13400
H	-42.66800	-10.01800	-0.25000
H	-41.86100	-8.98400	-1.00400
O	-34.46400	0.81600	2.00100
H	-33.73300	0.55500	2.56200
H	-35.07300	0.07900	2.04100
O	-36.38200	-1.04300	2.59800
H	-37.10900	-1.17700	1.99000
H	-36.79700	-0.73500	3.40400
O	-41.20200	-5.53400	-1.10600
H	-40.42600	-5.04500	-0.83300
H	-41.92700	-5.10900	-0.64800
O	-38.76400	-4.82200	-0.04800
H	-38.17000	-5.06200	-0.75900
H	-38.69200	-5.54100	0.57900
O	-40.76900	-4.25500	-3.78100
H	-41.02500	-4.65500	-2.95000
H	-41.39300	-3.54000	-3.90600
O	-36.45700	-1.51500	-1.27400
H	-37.01700	-1.44600	-0.50100
H	-37.06300	-1.69100	-1.99400
O	-34.87200	15.16100	1.41900
H	-34.23400	15.04100	0.71500
H	-34.99000	16.10900	1.47800
O	-38.06300	15.11300	1.35500
H	-38.40100	14.99800	0.46700
H	-37.19400	14.71300	1.33500
O	-42.20400	16.96700	-2.65500
H	-42.76800	16.87600	-3.42300
H	-42.31900	16.14700	-2.17500
O	-37.58100	16.26700	7.31400
H	-37.59200	15.87200	6.44200
H	-37.41900	15.53400	7.90700
O	-36.62400	20.63400	-7.34400
H	-37.27500	21.21900	-7.73000
H	-35.83000	21.16500	-7.28400
O	-42.69900	17.72300	-6.94500
H	-41.92500	17.17600	-7.08400
H	-42.34600	18.58700	-6.73300
O	-40.86000	19.05100	6.26200
H	-41.15100	19.95500	6.14600
H	-40.22400	19.09200	6.97600
O	-39.93400	16.42800	2.85200
H	-39.33800	16.20400	2.13800
H	-39.87700	15.68700	3.45400

O	-40.36200	13.88200	-9.31300
H	-40.10900	13.36000	-10.07500
H	-41.29900	13.71900	-9.21000
O	-35.31800	12.88600	-1.50600
H	-34.53100	12.46800	-1.85700
H	-35.05400	13.79000	-1.33700
O	-35.14700	18.32300	-6.80700
H	-35.78200	18.39700	-6.09500
H	-35.47700	18.91200	-7.48500
O	-29.91700	12.82300	-0.60200
H	-29.18400	12.25300	-0.36900
H	-30.67300	12.43600	-0.16200
O	-40.64200	15.78500	-7.11100
H	-40.75900	15.11000	-6.44300
H	-40.53200	15.29800	-7.92700
O	-38.29900	15.24600	-3.40600
H	-38.66300	14.71900	-2.69500
H	-37.44200	14.85600	-3.57900
O	-32.39400	6.53500	3.85700
H	-32.72600	7.40500	3.63300
H	-32.99000	6.21900	4.53700
O	-38.65900	10.27500	-8.41000
H	-38.49100	11.14300	-8.04300
H	-38.84900	10.43800	-9.33400
O	-35.90100	18.73700	3.25700
H	-36.20100	19.63900	3.14800
H	-36.24600	18.47000	4.10900
O	-32.50100	11.25500	0.93600
H	-33.41000	11.18700	0.64200
H	-32.55300	11.73200	1.76500
O	-38.68000	12.73100	-7.42500
H	-38.80000	13.17700	-6.58700
H	-39.37400	13.07900	-7.98400
O	-40.91900	25.50100	-3.77600
H	-40.99700	26.45200	-3.69700
H	-41.52300	25.15600	-3.11900
O	-38.42700	12.67400	8.88000
H	-39.20400	13.23300	8.87000
H	-37.78800	13.16000	9.40100
O	-30.63500	9.45700	2.71400
H	-31.27800	9.75000	2.06800
H	-31.14000	9.34200	3.52000
O	-32.98100	13.20300	-6.59700
H	-32.06900	13.43600	-6.77200
H	-33.49000	13.78700	-7.16000
O	-40.17300	15.48300	8.44200
H	-39.43100	16.02100	8.16500
H	-40.87900	16.10900	8.59900
O	-34.69700	17.62500	7.03900
H	-34.28900	17.18400	6.29400
H	-35.41900	17.05100	7.29200
O	-38.34400	12.56300	4.41200
H	-37.49800	12.98700	4.55600
H	-38.40400	11.90600	5.10600
O	-34.37400	18.18600	0.79400
H	-35.00300	18.35000	0.09200
H	-34.76200	18.60000	1.56500
O	-30.49500	19.12100	-1.85100
H	-31.18000	19.50100	-2.40200
H	-30.41100	19.73100	-1.11800
O	-35.19400	10.22300	6.07000
H	-35.43800	10.97700	5.53400
H	-34.91800	10.60200	6.90500
O	-33.24000	16.76700	4.74800
H	-33.02800	17.41000	4.07200
H	-33.28700	15.93300	4.28000
O	-35.63800	9.37900	1.65200
H	-36.49900	9.67700	1.94400
H	-35.61100	9.59600	0.72000
O	-38.82100	17.92300	-1.94100
H	-38.35800	18.20200	-2.73000
H	-39.08600	17.02100	-2.12300
O	-41.92900	17.47000	0.99700
H	-41.35900	17.89900	0.35900
H	-41.33800	17.18800	1.69600
O	-39.88500	14.04500	-5.13200
H	-39.18200	14.49700	-4.66400
H	-40.21600	13.40400	-4.50400
O	-41.43600	12.63600	9.24300
H	-42.06700	13.31800	9.47700
H	-41.54300	12.52500	8.29900
O	-32.12600	19.64400	2.92000
H	-31.30400	19.24200	2.63900
H	-32.12900	19.54100	3.87200
O	-38.36000	19.19300	5.07600
H	-38.29500	19.65500	5.91200
H	-39.06900	18.56200	5.20500
O	-24.03500	15.98800	-0.73100
H	-24.78900	15.41900	-0.88700
H	-23.90000	15.95400	0.21600
O	-38.64500	20.85900	-5.05600
H	-38.78100	21.70500	-4.62700
H	-38.10400	21.06100	-5.81900
O	-42.15800	6.93900	11.60600
H	-42.55200	6.38500	12.28100
H	-41.34200	7.25000	11.99600
O	-39.24200	8.35400	9.19800
H	-38.50100	8.95500	9.11800
H	-39.01000	7.61200	8.64000
O	-37.01800	21.48200	9.07400
H	-37.46200	20.69500	8.75700
H	-36.35700	21.67000	8.40900
O	-34.92000	21.76200	7.10300
H	-34.93800	22.58300	6.61100
H	-33.99800	21.63700	7.32800
O	-38.46300	19.27600	7.94500
H	-37.93000	18.51500	7.71500
H	-38.99100	18.98400	8.68700
O	-35.29600	4.38200	3.47400
H	-35.63900	5.27300	3.39800
H	-34.92800	4.19300	2.61100
O	-35.33100	14.37300	-8.01800
H	-35.84600	15.14400	-7.78000
H	-35.60300	13.70000	-7.39500
O	-37.74600	9.21800	-2.96000
H	-38.60200	8.82900	-3.13900
H	-37.33300	9.29700	-3.82000
O	-36.21600	6.75300	1.43900

H	-36.72000	6.71600	0.62600
H	-35.92300	7.66200	1.49900
O	-39.97200	6.83700	0.44600
H	-39.10700	6.64100	0.08600
H	-39.90400	6.60800	1.37300
O	-30.35700	17.12700	-6.08300
H	-31.13400	16.78800	-6.52800
H	-29.67800	17.13200	-6.75800
O	-35.54200	12.24600	-6.19300
H	-35.84700	12.56100	-5.34100
H	-34.58900	12.32400	-6.15000
O	-37.09800	18.40500	-4.54300
H	-37.62500	17.67000	-4.85600
H	-37.59900	19.18300	-4.78900
O	-32.11800	16.60000	0.57300
H	-32.77600	17.29000	0.66200
H	-31.31400	16.99000	0.91700
O	-42.36600	9.06700	-2.18300
H	-41.79100	8.84500	-2.91500
H	-41.77900	9.13600	-1.43000
O	-33.30800	14.95400	-1.37600
H	-32.79500	15.58700	-0.87400
H	-32.65800	14.35500	-1.74200
O	-40.06300	5.94100	-3.45500
H	-40.71700	5.62600	-4.07900
H	-40.10500	5.31900	-2.72900
O	-38.95200	16.22300	-11.54000
H	-39.86000	15.99200	-11.34100
H	-38.45500	15.42700	-11.35200
O	-36.04600	24.93300	-8.62000
H	-36.71900	25.23600	-8.01200
H	-35.65900	25.73600	-8.97000
O	-36.83000	12.42900	0.87000
H	-37.74700	12.65100	0.71200
H	-36.41400	12.50400	0.01100
O	-36.04600	13.82800	-4.00000
H	-35.29900	14.34100	-4.30900
H	-35.77700	13.50700	-3.14000
O	-37.77300	16.32400	-7.01700
H	-38.68200	16.04000	-6.92000
H	-37.74400	16.74400	-7.87700
O	-32.54200	19.93500	-3.24800
H	-32.99500	19.09400	-3.19200
H	-33.17200	20.57100	-2.91100
O	-33.59800	10.86400	-2.01800
H	-34.33600	10.35200	-1.68700
H	-33.34600	10.42400	-2.83000
O	-34.09800	21.77300	-2.31300
H	-33.80600	22.33300	-3.03200
H	-33.58800	22.06500	-1.55800
O	-33.49700	3.64200	-1.42400
H	-33.19100	2.78200	-1.71100
H	-32.77200	4.23400	-1.62400
O	-39.22500	13.93700	-0.91600
H	-39.17000	12.98500	-0.99200
H	-40.03600	14.09300	-0.43200
O	-38.07600	17.86500	-9.67900
H	-38.51400	17.14900	-10.13800
H	-37.33500	18.09300	-10.24000
O	-41.28600	12.55500	-3.15700
H	-42.09900	12.85400	-2.75100
H	-40.79000	12.16000	-2.44000
O	-39.13500	22.55100	-1.22400
H	-39.17700	22.77700	-2.15300
H	-38.21900	22.31700	-1.07500
O	-35.68800	13.01900	5.67400
H	-35.33300	13.24600	6.53400
H	-35.04100	13.35100	5.05200
O	-29.95900	16.14100	-3.62100
H	-30.09700	16.33100	-4.54900
H	-30.31500	16.90300	-3.16400
O	-41.79400	10.12700	2.45900
H	-41.15400	9.98600	1.76100
H	-42.62700	9.84000	2.08300
O	-29.93200	18.20900	1.39000
H	-29.49800	18.11600	2.23900
H	-29.21800	18.18400	0.75200
O	-41.01800	19.42700	-1.42800
H	-40.22400	18.93200	-1.62900
H	-41.72900	18.89900	-1.79100
O	-36.19900	21.80900	2.92400
H	-36.14500	22.37800	3.69100
H	-37.06700	21.97900	2.55900
O	-29.07000	18.25100	3.92400
H	-28.87600	19.15600	4.16900
H	-29.17000	17.79200	4.75700
O	-37.41600	8.13700	6.42300
H	-38.06400	8.83600	6.32900
H	-36.64600	8.57000	6.79000
O	-34.00900	18.40200	9.49800
H	-34.16000	18.17300	8.58100
H	-34.01800	19.35900	9.51100
O	-37.24000	15.97500	4.48200
H	-36.31100	16.12600	4.30900
H	-37.66100	16.06300	3.62700
O	-38.44100	20.00000	2.25200
H	-38.58000	19.63200	3.12500
H	-39.23000	20.51500	2.08100
O	-37.84600	25.10100	-6.40500
H	-37.67100	24.63000	-5.59100
H	-38.53300	25.72700	-6.17700
O	-36.89100	19.37800	-0.35900
H	-37.22600	19.33800	0.53700
H	-37.39000	18.71300	-0.83200
O	-33.95500	16.72400	-10.08500
H	-34.57200	15.99800	-10.18200
H	-34.42300	17.48500	-10.42900
O	-40.28800	14.49700	4.65500
H	-39.57800	13.85600	4.61300
H	-40.33100	14.74800	5.57700
O	-32.29900	21.43400	7.57500
H	-32.35800	20.74900	6.90800
H	-31.89700	22.17500	7.12300
O	-41.57500	11.48300	6.47400
H	-42.11100	11.03200	7.12600
H	-42.16300	11.61800	5.73100
O	-32.29800	19.39700	5.80700
H	-33.18500	19.06000	5.92900

H	-31.72800	18.66600	6.04400
O	-38.76700	24.05800	2.77300
H	-38.84000	24.86300	3.28500
H	-39.21000	24.25700	1.94800
O	-38.97900	23.29500	-3.71700
H	-39.76200	23.83700	-3.62300
H	-38.27900	23.81200	-3.31700
O	-35.66900	9.10400	-1.07400
H	-35.17200	8.35100	-1.39300
H	-36.44000	9.14100	-1.63900
O	-27.81600	14.80800	0.56800
H	-27.82700	15.69100	0.19800
H	-28.68400	14.45500	0.37400
O	-36.30800	23.95600	4.92900
H	-35.59500	24.59100	4.85600
H	-36.79900	24.05300	4.11300
O	-34.58600	17.86100	-3.50000
H	-35.52000	17.98200	-3.66900
H	-34.40800	16.95900	-3.76400
O	-39.08500	11.24600	-1.34600
H	-39.45600	10.69300	-0.65800
H	-38.58900	10.64200	-1.89900
O	-31.19900	13.70300	-2.96500
H	-30.73300	14.53600	-3.03900
H	-30.74800	13.23800	-2.26000
O	-41.25600	21.70600	5.74900
H	-40.55500	22.33900	5.90200
H	-41.72700	22.04700	4.98900
O	-33.13100	20.04800	-5.96600
H	-32.85500	19.93300	-5.05700
H	-34.00100	19.64900	-6.00500
O	-33.22700	6.07100	1.07400
H	-34.14100	6.30200	1.24300
H	-32.79800	6.15500	1.92600
O	-33.45600	14.26000	3.57400
H	-34.10900	14.50800	2.92000
H	-33.07800	13.44700	3.24100
O	-36.49600	24.29600	-2.32300
H	-36.31300	23.47800	-1.86000
H	-35.67200	24.51300	-2.76000
O	-36.16100	23.22300	-5.06100
H	-35.63300	22.71200	-5.67400
H	-35.89900	22.90700	-4.19700
O	-37.40800	15.00200	10.06100
H	-38.08300	14.90200	10.73100
H	-36.58500	14.84600	10.52500
O	-38.66500	26.30900	4.29700
H	-38.11300	26.47200	5.06200
H	-38.32500	26.89900	3.62500
O	-33.81400	21.13500	10.15200
H	-33.27000	21.74800	9.65700
H	-34.39600	21.69300	10.66800
O	-35.52800	24.25700	1.28300
H	-35.65300	23.45600	0.77400
H	-34.87100	24.75200	0.79500
O	-33.46500	22.05800	2.91100
H	-34.41200	21.93900	2.83900
H	-33.11700	21.17000	2.99400
O	-41.10500	7.94700	6.73500
H	-40.58200	8.19400	7.49800
H	-41.32400	8.77900	6.31500
O	-36.46000	21.85300	-0.88800
H	-36.60800	20.92500	-0.70800
H	-35.63500	21.87500	-1.37300
O	-42.59400	10.50500	-5.03000
H	-42.25400	11.03400	-5.75300
H	-42.14700	10.84300	-4.25500
O	-32.85700	22.67600	0.19800
H	-31.93200	22.47000	0.06000
H	-33.02500	22.42800	1.10700
O	-39.90000	8.88900	4.42000
H	-40.24000	8.09000	4.82200
H	-40.60600	9.18900	3.84800
O	-39.24800	5.97400	2.87300
H	-38.42500	5.50000	2.75100
H	-39.00400	6.75300	3.37300
O	-40.60700	9.00300	-6.83100
H	-39.88600	9.38100	-7.33500
H	-41.23100	9.72100	-6.72800
O	-36.46400	9.70700	-5.89500
H	-36.24800	10.59000	-6.19600
H	-36.94500	9.31600	-6.62500
O	-41.72400	12.66600	0.68200
H	-41.87000	12.98000	1.57400
H	-42.20200	13.28400	0.12900
O	-39.86500	9.65600	0.73000
H	-39.87800	8.76000	0.39300
H	-39.14700	9.66300	1.36400
O	-38.01200	10.09000	2.77500
H	-38.27000	11.01100	2.77400
H	-38.54300	9.69300	3.46500
O	-33.06700	23.19500	-4.44600
H	-32.89400	22.62900	-5.19800
H	-32.57400	23.99500	-4.62800
O	-40.46100	8.53700	-4.00100
H	-40.33900	7.60900	-3.80200
H	-40.75100	8.55100	-4.91300
O	-37.59200	6.22300	-0.86500
H	-37.57000	6.28700	-1.81900
H	-37.17500	5.38300	-0.67100
O	-27.21300	15.95800	-2.82700
H	-26.77000	15.20900	-3.22500
H	-28.09100	15.94900	-3.20900
O	-32.08000	22.51100	-7.34600
H	-31.38500	23.13200	-7.12700
H	-31.73800	21.66100	-7.07100
O	-30.18100	19.96100	-5.81700
H	-29.65600	20.30000	-6.54200
H	-30.26800	19.02600	-6.00100
O	-38.51900	23.87500	6.31200
H	-38.21000	23.63000	7.18400
H	-37.72100	24.07900	5.82400
O	-38.49700	26.02900	-1.45800
H	-38.93600	25.53600	-0.76500
H	-37.74600	25.48600	-1.69900
O	-41.04900	21.57200	0.61400
H	-41.67100	20.90900	0.31500
H	-40.37900	21.59800	-0.06800

O	-40.97700	10.05100	10.20800
H	-40.92800	10.92700	9.82500
H	-40.43500	9.50800	9.63500
O	-30.49100	20.59500	9.48800
H	-31.28200	20.57700	8.94800
H	-30.72800	21.13300	10.24300
O	-30.37200	17.44400	6.80100
H	-30.32200	17.27900	7.74300
H	-30.48000	16.57700	6.41100
O	-41.48800	20.12300	-5.91000
H	-41.33900	20.80600	-6.56300
H	-40.68100	20.10300	-5.39500
O	-33.08100	13.42700	8.11700
H	-32.18000	13.28800	7.82500
H	-33.00600	13.59200	9.05700
O	-38.74700	10.80400	6.96200
H	-39.61700	11.09700	6.69100
H	-38.51100	11.38800	7.68300
O	-28.87000	19.86400	7.04500
H	-29.16100	20.22700	7.88200
H	-29.48200	19.15000	6.86700
O	-27.67000	17.42900	-0.43400
H	-26.83500	17.86800	-0.27100
H	-27.53400	16.95300	-1.25400
O	-30.62100	14.98700	5.51300
H	-30.14600	15.18000	4.70500
H	-31.54400	15.04300	5.26800
O	-36.04300	7.02200	4.47900
H	-35.52500	7.75800	4.15300
H	-36.39700	7.32800	5.31400
O	-35.79200	3.44200	-0.00100
H	-35.51200	2.77400	0.62500
H	-35.00700	3.63500	-0.51400
O	-37.98800	22.61800	-8.43900
H	-38.56100	23.23700	-8.89200
H	-37.41500	23.16700	-7.90500
O	-31.92100	11.86300	3.74600
H	-30.96900	11.96300	3.76700
H	-32.09000	11.07100	4.25600
O	-33.89900	8.94900	3.93200
H	-34.49400	9.22900	3.23700
H	-34.18400	9.43800	4.70400
O	-34.76900	22.90100	-7.30800
H	-33.83600	22.83600	-7.10400
H	-34.85600	23.73700	-7.76600
O	-32.14000	22.21800	-10.34700
H	-32.21800	21.88400	-9.45400
H	-31.31900	22.71000	-10.34800
O	-29.44200	15.08400	2.86000
H	-29.47400	14.14600	3.04600
H	-28.83200	15.16200	2.12700
O	-37.60400	13.95400	-11.09700
H	-36.65800	13.82400	-11.02800
H	-37.98200	13.21500	-10.62000
O	-29.29300	11.87500	2.90600
H	-29.80900	11.09100	2.71900
H	-28.40900	11.54700	3.07100
O	-31.63100	5.61900	-1.13400
H	-31.82900	5.31100	-0.25000
H	-31.02200	6.34500	-1.00400
O	-23.62400	16.14600	2.00100
H	-22.89400	15.88400	2.56200
H	-24.23400	15.40800	2.04100
O	-25.54300	14.28600	2.59800
H	-26.27000	14.15200	1.99000
H	-25.95800	14.59400	3.40400
O	-37.72000	18.00500	10.36300
H	-36.80000	18.18300	10.55700
H	-37.75500	17.06100	10.21100
O	-39.41900	11.87900	-11.16100
H	-40.32600	11.58200	-11.08500
H	-39.14200	11.58200	-12.02800
O	-37.80300	6.42200	-5.00700
H	-38.61300	6.07700	-4.63200
H	-37.11500	6.11300	-4.41800
O	-35.87400	5.50000	-3.38500
H	-35.32100	5.92000	-2.72600
H	-35.63700	4.57400	-3.34200
O	-39.85100	11.48900	12.39600
H	-39.95100	12.43800	12.32700
H	-40.06900	11.16100	11.52300
O	-40.65000	8.73700	-11.02000
H	-39.78800	9.06300	-10.76100
H	-40.46400	7.95000	-11.53300
O	-32.49600	7.61000	-3.87700
H	-33.07600	8.26900	-4.25900
H	-32.76300	6.78800	-4.28800
O	-34.04600	7.01100	-1.67500
H	-33.54600	7.12200	-2.48300
H	-33.40200	6.70700	-1.03500
O	-39.98800	14.91900	11.21200
H	-40.47100	15.71900	11.41900
H	-39.90200	14.92900	10.25900
O	-33.71400	9.77000	-4.82100
H	-34.60100	10.05500	-5.03900
H	-33.29800	9.61000	-5.66800
O	-30.36200	9.79500	-1.10600
H	-29.58600	10.28400	-0.83300
H	-31.08700	10.22100	-0.64800
O	-27.92500	10.50800	-0.04800
H	-27.33100	10.26700	-0.75900
H	-27.85200	9.78800	0.57900
O	-34.56900	19.53500	-10.72600
H	-33.86600	19.74000	-10.11100
H	-35.29300	20.10200	-10.46100
O	-33.74000	15.28600	-4.60600
H	-33.79600	15.74200	-5.44500
H	-33.18700	14.52500	-4.78400
O	-29.92900	11.07500	-3.78100
H	-30.18500	10.67400	-2.95000
H	-30.55400	11.78900	-3.90600
O	-25.61800	13.81400	-1.27400
H	-26.17800	13.88400	-0.50100
H	-26.22400	13.63800	-1.99400
O	-32.66100	19.97300	-8.67600
H	-31.82600	19.54100	-8.85900
H	-32.82200	19.80100	-7.74900
O	-33.00600	16.47600	-7.31100

H	-33.06100	16.31100	-8.25200
H	-33.63200	17.18400	-7.15500
O	-35.69200	18.78100	12.01500
H	-34.82200	18.95500	11.65300
H	-36.17100	19.60000	11.89200
O	-39.69700	5.97700	-7.73200
H	-39.70900	6.92100	-7.57500
H	-38.80400	5.79400	-8.02600
O	-41.52300	25.90100	3.69300
H	-40.62000	25.93300	4.01000
H	-41.74900	24.97100	3.70800
O	-39.76800	24.65400	0.35400
H	-40.71800	24.65400	0.24100
H	-39.46400	23.90700	-0.16200
O	-35.88100	2.57100	-3.47800
H	-36.64800	2.01700	-3.33200
H	-35.47000	2.64300	-2.61600
O	-24.03200	30.49000	1.41900
H	-23.39400	30.37100	0.71500
H	-24.15000	31.43800	1.47800
O	-27.22300	30.44300	1.35500
H	-27.56200	30.32700	0.46700
H	-26.35400	30.04200	1.33500
O	-31.36500	32.29600	-2.65500
H	-31.92800	32.20500	-3.42300
H	-31.47900	31.47600	-2.17500
O	-26.74100	31.59600	7.31400
H	-26.75300	31.20100	6.44200
H	-26.57900	30.86300	7.90700
O	-31.85900	33.05200	-6.94500
H	-31.08600	32.50500	-7.08400
H	-31.50700	33.91700	-6.73300
O	-29.09500	31.75700	2.85200
H	-28.49900	31.53300	2.13800
H	-29.03800	31.01600	3.45400
O	-29.52300	29.21100	-9.31300
H	-29.27000	28.69000	-10.07500
H	-30.46000	29.04800	-9.21000
O	-24.47800	28.21500	-1.50600
H	-23.69200	27.79700	-1.85700
H	-24.21400	29.12000	-1.33700
O	-24.30700	33.65200	-6.80700
H	-24.94300	33.72600	-6.09500
H	-24.63700	34.24200	-7.48500
O	-19.07700	28.15300	-0.60200
H	-18.34500	27.58200	-0.36900
H	-19.83300	27.76500	-0.16200
O	-29.80300	31.11400	-7.11100
H	-29.91900	30.43900	-6.44300
H	-29.69300	30.62700	-7.92700
O	-27.46000	30.57500	-3.40600
H	-27.82400	30.04800	-2.69500
H	-26.60300	30.18500	-3.57900
O	-21.55500	21.86500	3.85700
H	-21.88600	22.73400	3.63300
H	-22.15000	21.54800	4.53700
O	-27.81900	25.60400	-8.41000
H	-27.65200	26.47200	-8.04300
H	-28.00900	25.76700	-9.33400
O	-25.06200	34.06600	3.25700
H	-25.36200	34.96800	3.14800
H	-25.40700	33.79900	4.10900
O	-31.61000	30.51700	-0.71000
H	-32.53500	30.38500	-0.50200
H	-31.36200	31.29700	-0.21300
O	-21.66200	26.58400	0.93600
H	-22.57000	26.51600	0.64200
H	-21.71300	27.06100	1.76500
O	-27.84000	28.06000	-7.42500
H	-27.96000	28.50600	-6.58700
H	-28.53500	28.40900	-7.98400
O	-34.25000	30.16100	0.12500
H	-34.12000	30.41400	1.03900
H	-34.93000	30.75300	-0.19500
O	-27.58800	28.00300	8.88000
H	-28.36500	28.56300	8.87000
H	-26.94900	28.48900	9.40100
O	-19.79600	24.78600	2.71400
H	-20.43800	25.07900	2.06800
H	-20.30000	24.67100	3.52000
O	-22.14200	28.53200	-6.59700
H	-21.23000	28.76500	-6.77200
H	-22.65100	29.11600	-7.16000
O	-29.33300	30.81200	8.44200
H	-28.59200	31.35000	8.16500
H	-30.04000	31.43800	8.59900
O	-23.85800	32.95500	7.03900
H	-23.44900	32.51300	6.29400
H	-24.58000	32.38000	7.29200
O	-27.50500	27.89200	4.41200
H	-26.65800	28.31600	4.55600
H	-27.56500	27.23500	5.10600
O	-23.53500	33.51500	0.79400
H	-24.16400	33.67900	0.09200
H	-23.92200	33.92900	1.56500
O	-19.65600	34.45000	-1.85100
H	-20.34100	34.83000	-2.40200
H	-19.57200	35.06000	-1.11800
O	-24.35500	25.55200	6.07000
H	-24.59900	26.30700	5.53400
H	-24.07900	25.93100	6.90500
O	-22.40100	32.09600	4.74800
H	-22.18900	32.73900	4.07200
H	-22.44800	31.26200	4.28000
O	-24.79800	24.70800	1.65200
H	-25.66000	25.00600	1.94400
H	-24.77200	24.92500	0.72000
O	-27.98200	33.25200	-1.94100
H	-27.51800	33.53100	-2.73000
H	-28.24600	32.35100	-2.12300
O	-31.09000	32.79900	0.99700
H	-30.51900	33.22800	0.35900
H	-30.49900	32.51800	1.69600
O	-29.04500	29.37400	-5.13200
H	-28.34300	29.82700	-4.66400
H	-29.37700	28.73300	-4.50400
O	-30.59700	27.96600	9.24300
H	-31.22700	28.64700	9.47700

H	-30.70300	27.85400	8.29900
O	-35.30300	27.63300	0.17000
H	-36.09300	27.88900	-0.30600
H	-34.81100	28.44800	0.27500
O	-21.28600	34.97300	2.92000
H	-20.46400	34.57100	2.63900
H	-21.29000	34.87000	3.87200
O	-33.35100	27.99700	-2.87200
H	-34.14800	28.10900	-2.35300
H	-33.57900	28.33600	-3.73800
O	-27.52100	34.52200	5.07600
H	-27.45600	34.98400	5.91200
H	-28.22900	33.89200	5.20500
O	-37.01900	27.88600	2.60100
H	-36.76800	28.77100	2.86700
H	-36.48300	27.70600	1.82900
O	-13.19600	31.31700	-0.73100
H	-13.94900	30.74800	-0.88700
H	-13.06100	31.28400	0.21600
O	-31.31900	22.26800	11.60600
H	-31.71200	21.71400	12.28100
H	-30.50200	22.57900	11.99600
O	-28.40200	23.68400	9.19800
H	-27.66100	24.28400	9.11800
H	-28.17000	22.94100	8.64000
O	-33.25800	28.86900	7.47600
H	-33.37300	27.96800	7.77600
H	-34.11400	29.11600	7.12400
O	-27.62300	34.60600	7.94500
H	-27.09100	33.84400	7.71500
H	-28.15200	34.31300	8.68700
O	-24.45700	19.71200	3.47400
H	-24.80000	20.60200	3.39800
H	-24.08900	19.52200	2.61100
O	-36.38800	32.03500	-3.70700
H	-35.55000	32.44500	-3.92400
H	-36.21000	31.09500	-3.73700
O	-24.49100	29.70300	-8.01800
H	-25.00700	30.47300	-7.78000
H	-24.76400	29.02900	-7.39500
O	-26.90700	24.54700	-2.96000
H	-27.76300	24.15800	-3.13900
H	-26.49400	24.62700	-3.82000
O	-25.37700	22.08200	1.43900
H	-25.88000	22.04500	0.62600
H	-25.08400	22.99100	1.49900
O	-29.13300	22.16600	0.44600
H	-28.26700	21.97000	0.08600
H	-29.06500	21.93700	1.37300
O	-19.51800	32.45600	-6.08300
H	-20.29500	32.11700	-6.52800
H	-18.83900	32.46100	-6.75800
O	-24.70300	27.57500	-6.19300
H	-25.00800	27.89000	-5.34100
H	-23.75000	27.65300	-6.15000
O	-26.25800	33.73400	-4.54300
H	-26.78600	33.00000	-4.85600
H	-26.75900	34.51200	-4.78900
O	-21.27900	31.92900	0.57300
H	-21.93700	32.61900	0.66200
H	-20.47500	32.31900	0.91700
O	-31.52600	24.39600	-2.18300
H	-30.95200	24.17400	-2.91500
H	-30.93900	24.46500	-1.43000
O	-22.46800	30.28400	-1.37600
H	-21.95500	30.91700	-0.87400
H	-21.81800	29.68400	-1.74200
O	-31.19900	26.42700	-8.16300
H	-32.03600	26.76400	-7.84200
H	-31.33900	26.29200	-9.10000
O	-29.22400	21.27000	-3.45500
H	-29.87800	20.95500	-4.07900
H	-29.26600	20.64800	-2.72900
O	-28.11300	31.55200	-11.54000
H	-29.02000	31.32100	-11.34100
H	-27.61600	30.75600	-11.35200
O	-25.99000	27.75900	0.87000
H	-26.90800	27.98000	0.71200
H	-25.57400	27.83300	0.01100
O	-25.20700	29.15800	-4.00000
H	-24.46000	29.67000	-4.30900
H	-24.93800	28.83600	-3.14000
O	-26.93400	31.65400	-7.01700
H	-27.84300	31.36900	-6.92000
H	-26.90500	32.07300	-7.87700
O	-21.70200	35.26400	-3.24800
H	-22.15600	34.42300	-3.19200
H	-22.33300	35.90000	-2.91100
O	-22.75900	26.19300	-2.01800
H	-23.49700	25.68100	-1.68700
H	-22.50600	25.75300	-2.83000
O	-22.65800	18.97100	-1.42400
H	-22.35200	18.11100	-1.71100
H	-21.93200	19.56300	-1.62400
O	-36.36500	29.37400	-3.03600
H	-36.56300	28.78100	-3.76100
H	-36.92100	29.06900	-2.31800
O	-28.38600	29.26600	-0.91600
H	-28.33100	28.31400	-0.99200
H	-29.19700	29.42200	-0.43200
O	-27.23700	33.19400	-9.67900
H	-27.67500	32.47800	-10.13800
H	-26.49500	33.42200	-10.24000
O	-30.44600	27.88400	-3.15700
H	-31.26000	28.18400	-2.75100
H	-29.95000	27.49000	-2.44000
O	-24.84900	28.34800	5.67400
H	-24.49400	28.57500	6.53400
H	-24.20200	28.68000	5.05200
O	-19.11900	31.47000	-3.62100
H	-19.25700	31.66000	-4.54900
H	-19.47600	32.23200	-3.16400
O	-30.95500	25.45600	2.45900
H	-30.31500	25.31500	1.76100
H	-31.78700	25.16900	2.08300
O	-19.09200	33.53800	1.39000
H	-18.65900	33.44500	2.23900
H	-18.37900	33.51400	0.75200

O	-30.17900	34.75600	-1.42800
H	-29.38500	34.26100	-1.62900
H	-30.89000	34.22800	-1.79100
O	-33.87100	28.90000	-5.35200
H	-34.80100	28.83900	-5.13500
H	-33.77500	28.37800	-6.14800
O	-33.95200	25.42600	-2.76400
H	-33.69100	26.34000	-2.87300
H	-33.13000	24.95800	-2.61400
O	-32.48000	26.84000	4.40600
H	-31.80700	26.63100	3.75700
H	-33.30700	26.64200	3.96700
O	-18.23100	33.58000	3.92400
H	-18.03700	34.48500	4.16900
H	-18.33100	33.12100	4.75700
O	-26.57700	23.46600	6.42300
H	-27.22400	24.16500	6.32900
H	-25.80700	23.90000	6.79000
O	-23.16900	33.73100	9.49800
H	-23.32100	33.50200	8.58100
H	-23.17900	34.68800	9.51100
O	-26.40100	31.30400	4.48200
H	-25.47100	31.45500	4.30900
H	-26.82200	31.39200	3.62700
O	-26.05100	34.70700	-0.35900
H	-26.38600	34.66700	0.53700
H	-26.55100	34.04200	-0.83200
O	-32.07700	30.11400	5.16600
H	-32.21500	29.38200	5.76700
H	-31.16600	30.02500	4.88500
O	-23.11500	32.05300	-10.08500
H	-23.73200	31.32800	-10.18200
H	-23.58400	32.81400	-10.42900
O	-29.44900	29.82600	4.65500
H	-28.73900	29.18500	4.61300
H	-29.49200	30.07700	5.57700
O	-21.46000	36.76300	7.57500
H	-21.51900	36.07900	6.90800
H	-21.05800	37.50500	7.12300
O	-30.73600	26.81300	6.47400
H	-31.27200	26.36200	7.12600
H	-31.32400	26.94700	5.73100
O	-31.32600	26.27100	-10.96300
H	-32.20300	26.15000	-11.32700
H	-30.85000	25.48000	-11.21500
O	-21.45800	34.72700	5.80700
H	-22.34600	34.38900	5.92900
H	-20.88800	33.99500	6.04400
O	-35.00200	25.94800	3.55900
H	-35.08600	25.34100	2.82500
H	-35.82400	26.43900	3.56000
O	-24.82900	24.43300	-1.07400
H	-24.33300	23.68000	-1.39300
H	-25.60100	24.47000	-1.63900
O	-16.97700	30.13800	0.56800
H	-16.98800	31.02000	0.19800
H	-17.84500	29.78500	0.37400
O	-23.74700	33.19100	-3.50000
H	-24.68100	33.31100	-3.66900
H	-23.56900	32.28800	-3.76400
O	-28.24600	26.57500	-1.34600
H	-28.61600	26.02200	-0.65800
H	-27.75000	25.97100	-1.89900
O	-20.35900	29.03200	-2.96500
H	-19.89300	29.86500	-3.03900
H	-19.90800	28.56700	-2.26000
O	-22.29200	35.37700	-5.96600
H	-22.01600	35.26200	-5.05700
H	-23.16100	34.97900	-6.00500
O	-22.38800	21.40000	1.07400
H	-23.30100	21.63100	1.24300
H	-21.95800	21.48400	1.92600
O	-22.61700	29.58900	3.57400
H	-23.27000	29.83700	2.92000
H	-22.23900	28.77600	3.24100
O	-26.56800	30.33100	10.06100
H	-27.24400	30.23100	10.73100
H	-25.74500	30.17500	10.52500
O	-40.22900	29.13900	1.40300
H	-40.19000	28.60600	2.19600
H	-40.73400	28.61300	0.78400
O	-32.84900	23.98600	4.89900
H	-32.78500	24.84700	4.48700
H	-32.91300	23.37300	4.16600
O	-33.37400	26.05800	8.54600
H	-32.74700	25.67600	9.16000
H	-34.23100	25.83900	8.91200
O	-22.97500	36.46400	10.15200
H	-22.43100	37.07700	9.65700
H	-23.55600	37.02200	10.66800
O	-36.59800	27.86600	-5.52200
H	-36.30200	26.95700	-5.57500
H	-37.49900	27.84400	-5.84400
O	-33.06800	25.94100	0.46100
H	-33.79000	26.55500	0.32200
H	-32.27900	26.47700	0.38300
O	-30.26600	23.27700	6.73500
H	-29.74200	23.52300	7.49800
H	-30.48400	24.10900	6.31500
O	-34.86900	25.54700	-5.39800
H	-35.19800	24.66700	-5.58000
H	-34.51500	25.49200	-4.51000
O	-31.75500	25.83400	-5.03000
H	-31.41500	26.36300	-5.75300
H	-31.30700	26.17200	-4.25500
O	-29.06000	24.21800	4.42000
H	-29.40100	23.41900	4.82200
H	-29.76600	24.51800	3.84800
O	-28.40900	21.30300	2.87300
H	-27.58600	20.82900	2.75100
H	-28.16500	22.08200	3.37300
O	-29.76800	24.33200	-6.83100
H	-29.04700	24.71000	-7.33500
H	-30.39100	25.05100	-6.72800
O	-25.62500	25.03600	-5.89500
H	-25.40800	25.91900	-6.19600
H	-26.10500	24.64500	-6.62500
O	-30.88500	27.99500	0.68200

H	-31.03000	28.30900	1.57400
H	-31.36200	28.61300	0.12900
O	-29.02500	24.98500	0.73000
H	-29.03900	24.08900	0.39300
H	-28.30800	24.99200	1.36400
O	-27.17300	25.41900	2.77500
H	-27.43100	26.34100	2.77400
H	-27.70400	25.02200	3.46500
O	-36.41000	30.53500	3.62100
H	-36.95500	31.31100	3.75300
H	-35.53200	30.88200	3.46300
O	-29.62200	23.86700	-4.00100
H	-29.50000	22.93800	-3.80200
H	-29.91100	23.88000	-4.91300
O	-26.75200	21.55200	-0.86500
H	-26.73000	21.61600	-1.81900
H	-26.33600	20.71200	-0.67100
O	-16.37400	31.28800	-2.82700
H	-15.93100	30.53800	-3.22500
H	-17.25100	31.27900	-3.20900
O	-35.62400	29.66200	6.51400
H	-36.36600	29.81100	7.10000
H	-35.94400	29.92300	5.65000
O	-19.34100	35.29100	-5.81700
H	-18.81600	35.62900	-6.54200
H	-19.42900	34.35500	-6.00100
O	-30.13700	25.38000	10.20800
H	-30.08900	26.25600	9.82500
H	-29.59500	24.83700	9.63500
O	-19.65200	35.92400	9.48800
H	-20.44200	35.90600	8.94800
H	-19.88900	36.46300	10.24300
O	-19.53200	32.77300	6.80100
H	-19.48200	32.60800	7.74300
H	-19.64100	31.90600	6.41100
O	-22.24200	28.75600	8.11700
H	-21.34100	28.61700	7.82500
H	-22.16700	28.92200	9.05700
O	-27.90700	26.13300	6.96200
H	-28.77700	26.42600	6.69100
H	-27.67200	26.71700	7.68300
O	-18.03000	35.19300	7.04500
H	-18.32200	35.55600	7.88200
H	-18.64300	34.47900	6.86700
O	-16.83100	32.75800	-0.43400
H	-15.99600	33.19700	-0.27100
H	-16.69500	32.28200	-1.25400
O	-19.78200	30.31600	5.51300
H	-19.30600	30.50900	4.70500
H	-20.70500	30.37200	5.26800
O	-32.89300	31.47800	-4.90400
H	-33.13900	30.57500	-4.70600
H	-32.72400	31.48000	-5.84600
O	-25.20400	22.35100	4.47900
H	-24.68500	23.08700	4.15300
H	-25.55800	22.65700	5.31400
O	-33.62400	27.21100	-7.40500
H	-34.31700	27.28600	-8.06100
H	-33.86900	26.44700	-6.88300
O	-24.95200	18.77100	-0.00100
H	-24.67200	18.10300	0.62500
H	-24.16700	18.96400	-0.51400
O	-21.08100	27.19300	3.74600
H	-20.13000	27.29200	3.76700
H	-21.25100	26.40000	4.25600
O	-23.06000	24.27800	3.93200
H	-23.65500	24.55900	3.23700
H	-23.34500	24.76800	4.70400
O	-21.30000	37.54700	-10.34700
H	-21.37900	37.21300	-9.45400
H	-20.47900	38.03900	-10.34800
O	-18.60300	30.41300	2.86000
H	-18.63500	29.47500	3.04600
H	-17.99300	30.49200	2.12700
O	-26.76400	29.28300	-11.09700
H	-25.81800	29.15300	-11.02800
H	-27.14300	28.54400	-10.62000
O	-18.45300	27.20400	2.90600
H	-18.96900	26.42000	2.71900
H	-17.56900	26.87600	3.07100
O	-20.79200	20.94800	-1.13400
H	-20.99000	20.64000	-0.25000
H	-20.18200	21.67500	-1.00400
O	-12.78500	31.47500	2.00100
H	-12.05500	31.21300	2.56200
H	-13.39400	30.73800	2.04100
O	-14.70300	29.61600	2.59800
H	-15.43000	29.48100	1.99000
H	-15.11900	29.92300	3.40400
O	-26.88000	33.33500	10.36300
H	-25.96000	33.51200	10.55700
H	-26.91500	32.39000	10.21100
O	-28.58000	27.20800	-11.16100
H	-29.48700	26.91100	-11.08500
H	-28.30200	26.91100	-12.02800
O	-37.66200	28.52800	-0.71200
H	-37.98200	27.67500	-1.00400
H	-38.20900	28.74300	0.04400
O	-33.88400	31.24500	3.00500
H	-33.66600	32.17600	3.02400
H	-33.47200	30.88500	3.79100
O	-26.96400	21.75200	-5.00700
H	-27.77400	21.40600	-4.63200
H	-26.27500	21.44200	-4.41800
O	-25.03500	20.82900	-3.38500
H	-24.48200	21.24900	-2.72600
H	-24.79800	19.90300	-3.34200
O	-29.01100	26.81800	12.39600
H	-29.11200	27.76700	12.32700
H	-29.22900	26.49000	11.52300
O	-29.81000	24.06700	-11.02000
H	-28.94800	24.39200	-10.76100
H	-29.62500	23.28000	-11.53300
O	-21.65700	22.93900	-3.87700
H	-22.23700	23.59800	-4.25900
H	-21.92300	22.11700	-4.28800
O	-23.20700	22.34000	-1.67500
H	-22.70700	22.45200	-2.48300

H	-22.56300	22.03600	-1.03500
O	-29.14800	30.24800	11.21200
H	-29.63200	31.04800	11.41900
H	-29.06200	30.25900	10.25900
O	-32.63100	32.70800	5.81600
H	-31.81200	33.20300	5.84000
H	-32.35700	31.79100	5.78800
O	-22.87400	25.09900	-4.82100
H	-23.76200	25.38400	-5.03900
H	-22.45900	24.93900	-5.66800
O	-19.52300	25.12400	-1.10600
H	-18.74700	25.61400	-0.83300
H	-20.24800	25.55000	-0.64800
O	-17.08600	25.83700	-0.04800
H	-16.49100	25.59600	-0.75900
H	-17.01300	25.11700	0.57900
O	-23.73000	34.86400	-10.72600
H	-23.02600	35.06900	-10.11100
H	-24.45400	35.43200	-10.46100
O	-22.90000	30.61500	-4.60600
H	-22.95700	31.07200	-5.44500
H	-22.34700	29.85400	-4.78400
O	-19.09000	26.40400	-3.78100
H	-19.34600	26.00300	-2.95000
H	-19.71500	27.11800	-3.90600
O	-14.77900	29.14300	-1.27400
H	-15.33900	29.21300	-0.50100
H	-15.38400	28.96800	-1.99400
O	-21.82100	35.30200	-8.67600
H	-20.98700	34.87000	-8.85900
H	-21.98300	35.13000	-7.74900
O	-22.16700	31.80500	-7.31100
H	-22.22200	31.64000	-8.25200
H	-22.79200	32.51300	-7.15500
O	-37.37800	27.04200	6.51500
H	-36.73200	26.53200	7.00400
H	-37.58600	27.77900	7.09000
O	-24.85200	34.11000	12.01500
H	-23.98300	34.28400	11.65300
H	-25.33200	34.93000	11.89200
O	-28.85700	21.30600	-7.73200
H	-28.86900	22.25000	-7.57500
H	-27.96500	21.12300	-8.02600
O	-25.04200	17.90000	-3.47800
H	-25.80900	17.34600	-3.33200
H	-24.63100	17.97300	-2.61600
O	-33.03800	24.18900	10.61300
H	-32.17500	23.83300	10.82300
H	-33.61100	23.85000	11.30000
O	-10.71500	37.19400	3.85700
H	-11.04700	38.06300	3.63300
H	-11.31100	36.87800	4.53700
O	-8.95600	40.11500	2.71400
H	-9.59900	40.40800	2.06800
H	-9.46100	40.00000	3.52000
O	-20.48000	37.59700	11.60600
H	-20.87300	37.04400	12.28100
H	-19.66300	37.90800	11.99600
O	-13.61700	35.04100	3.47400
H	-13.96100	35.93100	3.39800
H	-13.24900	34.85100	2.61100
O	-14.53700	37.41100	1.43900
H	-15.04100	37.37400	0.62600
H	-14.24400	38.32100	1.49900
O	-18.29300	37.49500	0.44600
H	-17.42800	37.29900	0.08600
H	-18.22500	37.26600	1.37300
O	-18.38500	36.60000	-3.45500
H	-19.03800	36.28400	-4.07900
H	-18.42700	35.97700	-2.72900
O	-11.81800	34.30000	-1.42400
H	-11.51200	33.44000	-1.71100
H	-11.09300	34.89200	-1.62400
O	-15.73800	38.79500	6.42300
H	-16.38500	39.49400	6.32900
H	-14.96800	39.22900	6.79000
O	-13.99000	39.76300	-1.07400
H	-13.49300	39.01000	-1.39300
H	-14.76100	39.79900	-1.63900
O	-11.54800	36.73000	1.07400
H	-12.46200	36.96000	1.24300
H	-11.11900	36.81300	1.92600
O	-17.56900	36.63200	2.87300
H	-16.74700	36.15800	2.75100
H	-17.32600	37.41100	3.37300
O	-15.91300	36.88100	-0.86500
H	-15.89100	36.94500	-1.81900
H	-15.49600	36.04200	-0.67100
O	-14.36400	37.68000	4.47900
H	-13.84600	38.41600	4.15300
H	-14.71900	37.98600	5.31400
O	-14.11300	34.10000	-0.00100
H	-13.83300	33.43200	0.62500
H	-13.32800	34.29300	-0.51400
O	-12.22100	39.60700	3.93200
H	-12.81600	39.88800	3.23700
H	-12.50600	40.09700	4.70400
O	-7.61400	42.53400	2.90600
H	-8.13000	41.74900	2.71900
H	-6.73000	42.20500	3.07100
O	-9.95300	36.27800	-1.13400
H	-10.15000	35.96900	-0.25000
H	-9.34300	37.00400	-1.00400
O	-16.12400	37.08100	-5.00700
H	-16.93400	36.73500	-4.63200
H	-15.43600	36.77100	-4.41800
O	-14.19500	36.15900	-3.38500
H	-13.64200	36.57800	-2.72600
H	-13.95800	35.23200	-3.34200
O	-10.81700	38.26900	-3.87700
H	-11.39800	38.92700	-4.25900
H	-11.08400	37.44600	-4.28800
O	-12.36700	37.66900	-1.67500
H	-11.86700	37.78100	-2.48300
H	-11.72400	37.36500	-1.03500
O	-12.03500	40.42900	-4.82100
H	-12.92200	40.71400	-5.03900
H	-11.62000	40.26800	-5.66800

O	-8.68400	40.45300	-1.10600
H	-7.90800	40.94300	-0.83300
H	-9.40900	40.87900	-0.64800
O	-6.24600	41.16600	-0.04800
H	-5.65200	40.92600	-0.75900
H	-6.17400	40.44700	0.57900
O	-18.01800	36.63500	-7.73200
H	-18.03000	37.57900	-7.57500
H	-17.12500	36.45200	-8.02600
O	-14.20300	33.23000	-3.47800
H	-14.96900	32.67500	-3.33200
H	-13.79200	33.30200	-2.61600
O	-40.75600	-25.50000	-13.87800
H	-40.02400	-26.07000	-13.64400
H	-41.51200	-25.88700	-13.43700
O	-41.47500	-28.86600	-10.56100
H	-42.11700	-28.57300	-11.20700
H	-41.97900	-28.98100	-9.75600
O	-41.33500	-19.20200	-15.12700
H	-42.02000	-18.82200	-15.67700
H	-41.25000	-18.59200	-14.39400
O	-34.87400	-22.33500	-14.00700
H	-35.62800	-22.90400	-14.16200
H	-34.74000	-22.36900	-13.05900
O	-41.19700	-21.19600	-19.35900
H	-41.97400	-21.53500	-19.80300
H	-40.51800	-21.19100	-20.03300
O	-40.79800	-22.18200	-16.89600
H	-40.93600	-21.99200	-17.82400
H	-41.15400	-21.42000	-16.44000
O	-40.77100	-20.11400	-11.88500
H	-40.33700	-20.20700	-11.03700
H	-40.05800	-20.13900	-12.52300
O	-39.90900	-20.07200	-9.35200
H	-39.71600	-19.16700	-9.10600
H	-40.00900	-20.53100	-8.51800
O	-38.65500	-23.51500	-12.70700
H	-38.66600	-22.63200	-13.07800
H	-39.52400	-23.86800	-12.90200
O	-42.03800	-24.62000	-16.24000
H	-41.57200	-23.78700	-16.31400
H	-41.58700	-25.08500	-15.53600
O	-38.05300	-22.36500	-16.10200
H	-37.61000	-23.11400	-16.50000
H	-38.93000	-22.37400	-16.48500
O	-41.02000	-18.36200	-19.09300
H	-40.49500	-18.02300	-19.81800
H	-41.10800	-19.29700	-19.27600
O	-41.33100	-17.72800	-3.78800
H	-42.12100	-17.74600	-4.32700
H	-41.56700	-17.19000	-3.03300
O	-41.21100	-20.87900	-6.47400
H	-41.16100	-21.04400	-5.53300
H	-41.32000	-21.74600	-6.86500
O	-39.70900	-18.45900	-6.23000
H	-40.00100	-18.09600	-5.39400
H	-40.32200	-19.17300	-6.40900
O	-38.51000	-20.89400	-13.71000
H	-37.67500	-20.45500	-13.54600
H	-38.37400	-21.37000	-14.52900
O	-41.46000	-23.33600	-7.76200
H	-40.98500	-23.14300	-8.57000
H	-42.38400	-23.28000	-8.00800
O	-40.28100	-23.23900	-10.41500
H	-40.31400	-24.17700	-10.22900
H	-39.67100	-23.16100	-11.14900
O	-40.13200	-26.44800	-10.36900
H	-40.64800	-27.23200	-10.55600
H	-39.24800	-26.77600	-10.20500
O	-34.46400	-22.17700	-11.27500
H	-33.73300	-22.43900	-10.71400
H	-35.07300	-22.91400	-11.23400
O	-36.38200	-24.03700	-10.67700
H	-37.10900	-24.17100	-11.28500
H	-36.79700	-23.72900	-9.87100
O	-41.20200	-28.52800	-14.38100
H	-40.42600	-28.03900	-14.10900
H	-41.92700	-28.10200	-13.92400
O	-38.76400	-27.81500	-13.32400
H	-38.17000	-28.05600	-14.03400
H	-38.69200	-28.53500	-12.69600
O	-40.76900	-27.24800	-17.05700
H	-41.02500	-27.64900	-16.22600
H	-41.39300	-26.53400	-17.18200
O	-36.45700	-24.50900	-14.54900
H	-37.01700	-24.43900	-13.77600
H	-37.06300	-24.68500	-15.26900
O	-34.87200	-7.83300	-11.85600
H	-34.23400	-7.95200	-12.56000
H	-34.99000	-6.88500	-11.79800
O	-38.06300	-7.88000	-11.92000
H	-38.40100	-7.99600	-12.80800
H	-37.19400	-8.28100	-11.94000
O	-42.20400	-6.02700	-15.93100
H	-42.76800	-6.11800	-16.69900
H	-42.31900	-6.84700	-15.45000
O	-37.58100	-6.72700	-5.96200
H	-37.59200	-7.12200	-6.83400
H	-37.41900	-7.46000	-5.36800
O	-36.62400	-2.36000	-20.61900
H	-37.27500	-1.77400	-21.00600
H	-35.83000	-1.82900	-20.56000
O	-42.69900	-5.27100	-20.22000
H	-41.92500	-5.81800	-20.35900
H	-42.34600	-4.40600	-20.00900
O	-40.86000	-3.94300	-7.01400
H	-41.15100	-3.03900	-7.12900
H	-40.22400	-3.90200	-6.29900
O	-39.93400	-6.56500	-10.42400
H	-39.33800	-6.79000	-11.13800
H	-39.87700	-7.30700	-9.82100
O	-40.36200	-9.11200	-22.58900
H	-40.10900	-9.63300	-23.35000
H	-41.29900	-9.27500	-22.48600
O	-35.31800	-10.10800	-14.78100
H	-34.53100	-10.52600	-15.13200
H	-35.05400	-9.20300	-14.61300
O	-35.14700	-4.67100	-20.08200

H	-35.78200	-4.59700	-19.37000
H	-35.47700	-4.08100	-20.76000
O	-29.91700	-10.17000	-13.87800
H	-29.18400	-10.74100	-13.64400
H	-30.67300	-10.55800	-13.43700
O	-40.64200	-7.20900	-20.38600
H	-40.75900	-7.88400	-19.71800
H	-40.53200	-7.69500	-21.20300
O	-38.29900	-7.74800	-16.68100
H	-38.66300	-8.27500	-15.97000
H	-37.44200	-8.13800	-16.85500
O	-32.39400	-16.45800	-9.41800
H	-32.72600	-15.58900	-9.64200
H	-32.99000	-16.77500	-8.73900
O	-38.65900	-12.71900	-21.68500
H	-38.49100	-11.85100	-21.31800
H	-38.84900	-12.55600	-22.60900
O	-35.90100	-4.25700	-10.01900
H	-36.20100	-3.35500	-10.12700
H	-36.24600	-4.52400	-9.16700
O	-32.50100	-11.73900	-12.33900
H	-33.41000	-11.80700	-12.63300
H	-32.55300	-11.26200	-11.51100
O	-38.68000	-10.26300	-20.70100
H	-38.80000	-9.81700	-19.86200
H	-39.37400	-9.91400	-21.25900
O	-40.91900	2.50700	-17.05200
H	-40.99700	3.45800	-16.97300
H	-41.52300	2.16200	-16.39400
O	-38.42700	-10.32000	-4.39600
H	-39.20400	-9.76000	-4.40500
H	-37.78800	-9.83400	-3.87400
O	-30.63500	-13.53700	-10.56100
H	-31.27800	-13.24400	-11.20700
H	-31.14000	-13.65200	-9.75600
O	-32.98100	-9.79100	-19.87300
H	-32.06900	-9.55800	-20.04800
H	-33.49000	-9.20700	-20.43500
O	-40.17300	-7.51100	-4.83400
H	-39.43100	-6.97300	-5.11000
H	-40.87900	-6.88500	-4.67700
O	-34.69700	-5.36800	-6.23600
H	-34.28900	-5.81000	-6.98100
H	-35.41900	-5.94300	-5.98300
O	-38.34400	-10.43100	-8.86300
H	-37.49800	-10.00700	-8.72000
H	-38.40400	-11.08800	-8.16900
O	-34.37400	-4.80800	-12.48100
H	-35.00300	-4.64400	-13.18400
H	-34.76200	-4.39400	-11.71000
O	-30.49500	-3.87300	-15.12700
H	-31.18000	-3.49300	-15.67700
H	-30.41100	-3.26300	-14.39400
O	-35.19400	-12.77100	-7.20500
H	-35.43800	-12.01600	-7.74200
H	-34.91800	-12.39200	-6.37000
O	-33.24000	-6.22700	-8.52700
H	-33.02800	-5.58400	-9.20400
H	-33.28700	-7.06100	-8.99500
O	-35.63800	-13.61500	-11.62400
H	-36.49900	-13.31700	-11.33200
H	-35.61100	-13.39800	-12.55600
O	-38.82100	-5.07100	-15.21600
H	-38.35800	-4.79200	-16.00600
H	-39.08600	-5.97200	-15.39900
O	-41.92900	-5.52400	-12.27800
H	-41.35900	-5.09500	-12.91700
H	-41.33800	-5.80500	-11.58000
O	-39.88500	-8.94800	-18.40700
H	-39.18200	-8.49600	-17.94000
H	-40.21600	-9.59000	-17.77900
O	-41.43600	-10.35700	-4.03200
H	-42.06700	-9.67600	-3.79900
H	-41.54300	-10.46900	-4.97700
O	-32.12600	-3.35000	-10.35500
H	-31.30400	-3.75200	-10.63700
H	-32.12900	-3.45300	-9.40300
O	-38.36000	-3.80100	-8.19900
H	-38.29500	-3.33900	-7.36300
H	-39.06900	-4.43100	-8.07100
O	-24.03500	-7.00600	-14.00700
H	-24.78900	-7.57500	-14.16200
H	-23.90000	-7.03900	-13.05900
O	-38.64500	-2.13400	-18.33100
H	-38.78100	-1.28900	-17.90300
H	-38.10400	-1.93200	-19.09400
O	-42.15800	-16.05500	-1.67000
H	-42.55200	-16.60900	-0.99500
H	-41.34200	-15.74400	-1.27900
O	-39.24200	-14.63900	-4.07800
H	-38.50100	-14.03900	-4.15700
H	-39.01000	-15.38200	-4.63600
O	-37.01800	-1.51200	-4.20100
H	-37.46200	-2.29800	-4.51900
H	-36.35700	-1.32400	-4.86700
O	-34.92000	-1.23200	-6.17300
H	-34.93800	-0.41100	-6.66500
H	-33.99800	-1.35600	-5.94800
O	-38.46300	-3.71700	-5.33100
H	-37.93000	-4.47900	-5.56100
H	-38.99100	-4.01000	-4.58800
O	-35.29600	-18.61100	-9.80100
H	-35.63900	-17.72100	-9.87700
H	-34.92800	-18.80100	-10.66400
O	-35.33100	-8.62000	-21.29400
H	-35.84600	-7.85000	-21.05500
H	-35.60300	-9.29400	-20.67100
O	-37.74600	-13.77500	-16.23500
H	-38.60200	-14.16500	-16.41500
H	-37.33300	-13.69600	-17.09500
O	-36.21600	-16.24100	-11.83600
H	-36.72000	-16.27800	-12.64900
H	-35.92300	-15.33200	-11.77700
O	-39.97200	-16.15700	-12.83000
H	-39.10700	-16.35300	-13.18900
H	-39.90400	-16.38600	-11.90300
O	-30.35700	-5.86700	-19.35900
H	-31.13400	-6.20600	-19.80300

H	-29.67800	-5.86200	-20.03300
O	-35.54200	-10.74800	-19.46800
H	-35.84700	-10.43300	-18.61700
H	-34.58900	-10.67000	-19.42500
O	-37.09800	-4.58900	-17.81900
H	-37.62500	-5.32300	-18.13100
H	-37.59900	-3.81100	-18.06500
O	-32.11800	-6.39400	-12.70200
H	-32.77600	-5.70400	-12.61400
H	-31.31400	-6.00400	-12.35900
O	-42.36600	-13.92700	-15.45800
H	-41.79100	-14.14900	-16.19100
H	-41.77900	-13.85800	-14.70500
O	-33.30800	-8.03900	-14.65200
H	-32.79500	-7.40600	-14.14900
H	-32.65800	-8.63900	-15.01700
O	-40.06300	-17.05300	-16.73100
H	-40.71700	-17.36800	-17.35500
H	-40.10500	-17.67500	-16.00500
O	-38.95200	-6.77100	-24.81500
H	-39.86000	-7.00200	-24.61600
H	-38.45500	-7.56700	-24.62800
O	-36.04600	1.93900	-21.89600
H	-36.71900	2.24200	-21.28700
H	-35.65900	2.74200	-22.24500
O	-36.83000	-10.56400	-12.40600
H	-37.74700	-10.34300	-12.56400
H	-36.41400	-10.49000	-13.26500
O	-36.04600	-9.16500	-17.27500
H	-35.29900	-8.65300	-17.58500
H	-35.77700	-9.48700	-16.41500
O	-37.77300	-6.66900	-20.29300
H	-38.68200	-6.95400	-20.19600
H	-37.74400	-6.25000	-21.15200
O	-32.54200	-3.05900	-16.52400
H	-32.99500	-3.90000	-16.46700
H	-33.17200	-2.42300	-16.18600
O	-33.59800	-12.13000	-15.29400
H	-34.33600	-12.64200	-14.96300
H	-33.34600	-12.57000	-16.10500
O	-34.09800	-1.22100	-15.58900
H	-33.80600	-0.66100	-16.30700
H	-33.58800	-0.92800	-14.83300
O	-33.49700	-19.35200	-14.69900
H	-33.19100	-20.21200	-14.98700
H	-32.77200	-18.76000	-14.90000
O	-39.22500	-9.05700	-14.19200
H	-39.17000	-10.00900	-14.26800
H	-40.03600	-8.90100	-13.70800
O	-38.07600	-5.12900	-22.95400
H	-38.51400	-5.84500	-23.41300
H	-37.33500	-4.90100	-23.51500
O	-41.28600	-10.43900	-16.43300
H	-42.09900	-10.13900	-16.02600
H	-40.79000	-10.83300	-15.71500
O	-39.13500	-0.44300	-14.49900
H	-39.17700	-0.21700	-15.42800
H	-38.21900	-0.67700	-14.35100
O	-35.68800	-9.97500	-7.60100
H	-35.33300	-9.74800	-6.74200
H	-35.04100	-9.64300	-8.22400
O	-29.95900	-6.85300	-16.89600
H	-30.09700	-6.66300	-17.82400
H	-30.31500	-6.09100	-16.44000
O	-41.79400	-12.86700	-10.81700
H	-41.15400	-13.00800	-11.51400
H	-42.62700	-13.15400	-11.19300
O	-29.93200	-4.78500	-11.88500
H	-29.49800	-4.87800	-11.03700
H	-29.21800	-4.80900	-12.52300
O	-41.01800	-3.56700	-14.70300
H	-40.22400	-4.06200	-14.90500
H	-41.72900	-4.09500	-15.06600
O	-36.19900	-1.18500	-10.35200
H	-36.14500	-0.61600	-9.58400
H	-37.06700	-1.01500	-10.71700
O	-29.07000	-4.74300	-9.35200
H	-28.87600	-3.83800	-9.10600
H	-29.17000	-5.20200	-8.51800
O	-37.41600	-14.85700	-6.85300
H	-38.06400	-14.15800	-6.94600
H	-36.64600	-14.42300	-6.48500
O	-34.00900	-4.59200	-3.77800
H	-34.16000	-4.82100	-4.69500
H	-34.01800	-3.63500	-3.76500
O	-37.24000	-7.01900	-8.79300
H	-36.31100	-6.86800	-8.96600
H	-37.66100	-6.93100	-9.64800
O	-38.44100	-2.99400	-11.02300
H	-38.58000	-3.36200	-10.15100
H	-39.23000	-2.47900	-11.19500
O	-37.84600	2.10700	-19.68100
H	-37.67100	1.63600	-18.86600
H	-38.53300	2.73400	-19.45300
O	-36.89100	-3.61600	-13.63400
H	-37.22600	-3.65600	-12.73900
H	-37.39000	-4.28100	-14.10700
O	-33.95500	-6.27000	-23.36100
H	-34.57200	-6.99500	-23.45700
H	-34.42300	-5.50900	-23.70400
O	-40.28800	-8.49700	-8.62100
H	-39.57800	-9.13800	-8.66200
H	-40.33100	-8.24600	-7.69800
O	-32.29900	-1.56000	-5.70000
H	-32.35800	-2.24400	-6.36700
H	-31.89700	-0.81800	-6.15200
O	-41.57500	-11.51000	-6.80100
H	-42.11100	-11.96100	-6.14900
H	-42.16300	-11.37600	-7.54400
O	-32.29800	-3.59600	-7.46900
H	-33.18500	-3.93400	-7.34600
H	-31.72800	-4.32800	-7.23200
O	-38.76700	1.06400	-10.50300
H	-38.84000	1.86900	-9.99100
H	-39.21000	1.26300	-11.32800
O	-38.97900	0.30100	-16.99200
H	-39.76200	0.84300	-16.89900
H	-38.27900	0.81800	-16.59300

O	-35.66900	-13.89000	-14.34900
H	-35.17200	-14.64300	-14.66900
H	-36.44000	-13.85300	-14.91500
O	-27.81600	-8.18500	-12.70700
H	-27.82700	-7.30300	-13.07800
H	-28.68400	-8.53800	-12.90200
O	-36.30800	0.96300	-8.34700
H	-35.59500	1.59700	-8.41900
H	-36.79900	1.05900	-9.16300
O	-34.58600	-5.13200	-16.77500
H	-35.52000	-5.01200	-16.94500
H	-34.40800	-6.03500	-17.03900
O	-39.08500	-11.74800	-14.62200
H	-39.45600	-12.30100	-13.93400
H	-38.58900	-12.35200	-15.17400
O	-31.19900	-9.29100	-16.24000
H	-30.73300	-8.45800	-16.31400
H	-30.74800	-9.75600	-15.53600
O	-41.25600	-1.28800	-7.52600
H	-40.55500	-0.65400	-7.37300
H	-41.72700	-0.94700	-8.28700
O	-33.13100	-2.94600	-19.24200
H	-32.85500	-3.06100	-18.33200
H	-34.00100	-3.34400	-19.28000
O	-33.22700	-16.92300	-12.20100
H	-34.14100	-16.69200	-12.03300
H	-32.79800	-16.83900	-11.35000
O	-33.45600	-8.73400	-9.70100
H	-34.10900	-8.48600	-10.35600
H	-33.07800	-9.54700	-10.03500
O	-36.49600	1.30200	-15.59800
H	-36.31300	0.48500	-15.13500
H	-35.67200	1.51900	-16.03600
O	-36.16100	0.22900	-18.33700
H	-35.63300	-0.28200	-18.95000
H	-35.89900	-0.08700	-17.47200
O	-37.40800	-7.99200	-3.21400
H	-38.08300	-8.09200	-2.54400
H	-36.58500	-8.14800	-2.75100
O	-38.66500	3.31500	-8.97900
H	-38.11300	3.47800	-8.21400
H	-38.32500	3.90600	-9.65100
O	-33.81400	-1.85900	-3.12400
H	-33.27000	-1.24600	-3.61800
H	-34.39600	-1.30100	-2.60700
O	-35.52800	1.26300	-11.99200
H	-35.65300	0.46200	-12.50100
H	-34.87100	1.75800	-12.48100
O	-33.46500	-0.93600	-10.36400
H	-34.41200	-1.05500	-10.43700
H	-33.11700	-1.82400	-10.28200
O	-41.10500	-15.04600	-6.54100
H	-40.58200	-14.80000	-5.77800
H	-41.32400	-14.21400	-6.96000
O	-36.46000	-1.14100	-14.16400
H	-36.60800	-2.06900	-13.98300
H	-35.63500	-1.11900	-14.64800
O	-42.59400	-12.48900	-18.30600
H	-42.25400	-11.96000	-19.02800
H	-42.14700	-12.15100	-17.53000
O	-32.85700	-0.31800	-13.07700
H	-31.93200	-0.52300	-13.21500
H	-33.02500	-0.56600	-12.16900
O	-39.90000	-14.10500	-8.85600
H	-40.24000	-14.90400	-8.45300
H	-40.60600	-13.80500	-9.42800
O	-39.24800	-17.02000	-10.40300
H	-38.42500	-17.49400	-10.52500
H	-39.00400	-16.24100	-9.90300
O	-40.60700	-13.99100	-20.10700
H	-39.88600	-13.61300	-20.61000
H	-41.23100	-13.27200	-20.00300
O	-36.46400	-13.28700	-19.17100
H	-36.24800	-12.40400	-19.47200
H	-36.94500	-13.67800	-19.90000
O	-41.72400	-10.32800	-12.59400
H	-41.87000	-10.01400	-11.70100
H	-42.20200	-9.71000	-13.14700
O	-39.86500	-13.33800	-12.54600
H	-39.87800	-14.23400	-12.88200
H	-39.14700	-13.33100	-11.91200
O	-38.01200	-12.90400	-10.50000
H	-38.27000	-11.98200	-10.50200
H	-38.54300	-13.30100	-9.81000
O	-33.06700	0.20100	-17.72100
H	-32.89400	-0.36400	-18.47400
H	-32.57400	1.00100	-17.90300
O	-40.46100	-14.45600	-17.27700
H	-40.33900	-15.38500	-17.07800
H	-40.75100	-14.44300	-18.18900
O	-37.59200	-16.77100	-14.14000
H	-37.57000	-16.70700	-15.09500
H	-37.17500	-17.61100	-13.94600
O	-27.21300	-7.03500	-16.10200
H	-26.77000	-7.78500	-16.50000
H	-28.09100	-7.04400	-16.48500
O	-32.08000	-0.48200	-20.62200
H	-31.38500	0.13800	-20.40300
H	-31.73800	-1.33300	-20.34600
O	-30.18100	-3.03200	-19.09300
H	-29.65600	-2.69400	-19.81800
H	-30.26800	-3.96800	-19.27600
O	-38.51900	0.88100	-6.96400
H	-38.21000	0.63600	-6.09200
H	-37.72100	1.08600	-7.45200
O	-38.49700	3.03500	-14.73300
H	-38.93600	2.54200	-14.04000
H	-37.74600	2.49200	-14.97400
O	-41.04900	-1.42100	-12.66100
H	-41.67100	-2.08400	-12.96100
H	-40.37900	-1.39500	-13.34400
O	-40.97700	-12.94300	-3.06800
H	-40.92800	-12.06700	-3.45000
H	-40.43500	-13.48600	-3.64100
O	-30.49100	-2.39900	-3.78800
H	-31.28200	-2.41700	-4.32700
H	-30.72800	-1.86000	-3.03300
O	-30.37200	-5.55000	-6.47400

H	-30.32200	-5.71500	-5.53300
H	-30.48000	-6.41700	-6.86500
O	-41.48800	-2.87100	-19.18500
H	-41.33900	-2.18800	-19.83900
H	-40.68100	-2.89100	-18.67000
O	-33.08100	-9.56700	-5.15800
H	-32.18000	-9.70600	-5.45000
H	-33.00600	-9.40100	-4.21900
O	-38.74700	-12.19000	-6.31300
H	-39.61700	-11.89700	-6.58500
H	-38.51100	-11.60600	-5.59200
O	-28.87000	-3.13000	-6.23000
H	-29.16100	-2.76700	-5.39400
H	-29.48200	-3.84400	-6.40900
O	-27.67000	-5.56500	-13.71000
H	-26.83500	-5.12600	-13.54600
H	-27.53400	-6.04100	-14.52900
O	-30.62100	-8.00700	-7.76200
H	-30.14600	-7.81400	-8.57000
H	-31.54400	-7.95100	-8.00800
O	-36.04300	-15.97200	-8.79700
H	-35.52500	-15.23600	-9.12200
H	-36.39700	-15.66600	-7.96200
O	-35.79200	-19.55200	-13.27700
H	-35.51200	-20.22000	-12.65000
H	-35.00700	-19.35900	-13.78900
O	-37.98800	-0.37600	-21.71400
H	-38.56100	0.24300	-22.16700
H	-37.41500	0.17400	-21.18000
O	-31.92100	-11.13000	-9.52900
H	-30.96900	-11.03100	-9.50800
H	-32.09000	-11.92300	-9.02000
O	-33.89900	-14.04500	-9.34300
H	-34.49400	-13.76400	-10.03900
H	-34.18400	-13.55500	-8.57200
O	-34.76900	-0.09300	-20.58300
H	-33.83600	-0.15800	-20.38000
H	-34.85600	0.74300	-21.04200
O	-32.14000	-0.77600	-23.62300
H	-32.21800	-1.11000	-22.72900
H	-31.31900	-0.28400	-23.62400
O	-29.44200	-7.91000	-10.41500
H	-29.47400	-8.84800	-10.22900
H	-28.83200	-7.83100	-11.14900
O	-37.60400	-9.04000	-24.37200
H	-36.65800	-9.17000	-24.30400
H	-37.98200	-9.77900	-23.89500
O	-29.29300	-11.11900	-10.36900
H	-29.80900	-11.90300	-10.55600
H	-28.40900	-11.44700	-10.20500
O	-31.63100	-17.37500	-14.41000
H	-31.82900	-17.68300	-13.52500
H	-31.02200	-16.64800	-14.28000
O	-23.62400	-6.84800	-11.27500
H	-22.89400	-7.11000	-10.71400
H	-24.23400	-7.58500	-11.23400
O	-25.54300	-8.70700	-10.67700
H	-26.27000	-8.84200	-11.28500
H	-25.95800	-8.40000	-9.87100
O	-37.72000	-4.98800	-2.91200
H	-36.80000	-4.81100	-2.71800
H	-37.75500	-5.93300	-3.06400
O	-39.41900	-11.11500	-24.43700
H	-40.32600	-11.41200	-24.36100
H	-39.14200	-11.41200	-25.30400
O	-37.80300	-16.57100	-18.28300
H	-38.61300	-16.91700	-17.90700
H	-37.11500	-16.88100	-17.69400
O	-35.87400	-17.49400	-16.66000
H	-35.32100	-17.07400	-16.00100
H	-35.63700	-18.42000	-16.61800
O	-39.85100	-11.50500	-0.88000
H	-39.95100	-10.55600	-0.94900
H	-40.06900	-11.83300	-1.75200
O	-40.65000	-14.25600	-24.29600
H	-39.78800	-13.93100	-24.03600
H	-40.46400	-15.04300	-24.80800
O	-32.49600	-15.38400	-17.15200
H	-33.07600	-14.72500	-17.53400
H	-32.76300	-16.20600	-17.56300
O	-34.04600	-15.98300	-14.95000
H	-33.54600	-15.87100	-15.75900
H	-33.40200	-16.28700	-14.31000
O	-39.98800	-8.07500	-2.06300
H	-40.47100	-7.27500	-1.85700
H	-39.90200	-8.06400	-3.01700
O	-33.71400	-13.22400	-18.09700
H	-34.60100	-12.93900	-18.31500
H	-33.29800	-13.38400	-18.94400
O	-30.36200	-13.19900	-14.38100
H	-29.58600	-12.70900	-14.10900
H	-31.08700	-12.77300	-13.92400
O	-27.92500	-12.48600	-13.32400
H	-27.33100	-12.72700	-14.03400
H	-27.85200	-13.20500	-12.69600
O	-34.56900	-3.45900	-24.00200
H	-33.86600	-3.25400	-23.38600
H	-35.29300	-2.89100	-23.73700
O	-33.74000	-7.70800	-17.88200
H	-33.79600	-7.25100	-18.72100
H	-33.18700	-8.46900	-18.05900
O	-29.92900	-11.91900	-17.05700
H	-30.18500	-12.32000	-16.22600
H	-30.55400	-11.20500	-17.18200
O	-25.61800	-9.18000	-14.54900
H	-26.17800	-9.11000	-13.77600
H	-26.22400	-9.35500	-15.26900
O	-32.66100	-3.02100	-21.95200
H	-31.82600	-3.45300	-22.13400
H	-32.82200	-3.19300	-21.02400
O	-33.00600	-6.51800	-20.58600
H	-33.06100	-6.68300	-21.52700
H	-33.63200	-5.81000	-20.43000
O	-35.69200	-4.21300	-1.26100
H	-34.82200	-4.03900	-1.62200
H	-36.17100	-3.39300	-1.38300
O	-39.69700	-17.01700	-21.00800
H	-39.70900	-16.07300	-20.85100

H	-38.80400	-17.20000	-21.30200
O	-41.52300	2.90700	-9.58200
H	-40.62000	2.93900	-9.26500
H	-41.74900	1.97700	-9.56700
O	-39.76800	1.66000	-12.92100
H	-40.71800	1.66000	-13.03500
H	-39.46400	0.91300	-13.43700
O	-35.88100	-20.42300	-16.75300
H	-36.64800	-20.97700	-16.60800
H	-35.47000	-20.35000	-15.89200
O	-24.03200	7.49600	-11.85600
H	-23.39400	7.37700	-12.56000
H	-24.15000	8.44400	-11.79800
O	-27.22300	7.44900	-11.92000
H	-27.56200	7.33300	-12.80800
H	-26.35400	7.04800	-11.94000
O	-31.36500	9.30200	-15.93100
H	-31.92800	9.21100	-16.69900
H	-31.47900	8.48200	-15.45000
O	-26.74100	8.60200	-5.96200
H	-26.75300	8.20800	-6.83400
H	-26.57900	7.86900	-5.36800
O	-33.81800	10.79700	-13.09200
H	-33.02600	10.45700	-13.50800
H	-33.57900	10.90700	-12.17200
O	-25.78500	12.96900	-20.61900
H	-26.43600	13.55500	-21.00600
H	-24.99000	13.50000	-20.56000
O	-31.85900	10.05800	-20.22000
H	-31.08600	9.51100	-20.35900
H	-31.50700	10.92300	-20.00900
O	-30.02100	11.38600	-7.01400
H	-30.31200	12.29100	-7.12900
H	-29.38500	11.42800	-6.29900
O	-29.09500	8.76400	-10.42400
H	-28.49900	8.53900	-11.13800
H	-29.03800	8.02200	-9.82100
O	-29.52300	6.21800	-22.58900
H	-29.27000	5.69600	-23.35000
H	-30.46000	6.05400	-22.48600
O	-24.47800	5.22100	-14.78100
H	-23.69200	4.80300	-15.13200
H	-24.21400	6.12600	-14.61300
O	-24.30700	10.65900	-20.08200
H	-24.94300	10.73200	-19.37000
H	-24.63700	11.24800	-20.76000
O	-19.07700	5.15900	-13.87800
H	-18.34500	4.58900	-13.64400
H	-19.83300	4.77100	-13.43700
O	-29.80300	8.12000	-20.38600
H	-29.91900	7.44500	-19.71800
H	-29.69300	7.63400	-21.20300
O	-27.46000	7.58100	-16.68100
H	-27.82400	7.05500	-15.97000
H	-26.60300	7.19100	-16.85500
O	-27.81900	2.61000	-21.68500
H	-27.65200	3.47800	-21.31800
H	-28.00900	2.77300	-22.60900
O	-25.06200	11.07200	-10.01900
H	-25.36200	11.97500	-10.12700
H	-25.40700	10.80500	-9.16700
O	-31.61000	7.52400	-13.98500
H	-32.53500	7.39100	-13.77700
H	-31.36200	8.30300	-13.48800
O	-21.66200	3.59000	-12.33900
H	-22.57000	3.52200	-12.63300
H	-21.71300	4.06800	-11.51100
O	-27.84000	5.06600	-20.70100
H	-27.96000	5.51200	-19.86200
H	-28.53500	5.41500	-21.25900
O	-34.25000	7.16700	-13.15100
H	-34.12000	7.42000	-12.23700
H	-34.93000	7.76000	-13.47000
O	-30.08000	17.83700	-17.05200
H	-30.15700	18.78700	-16.97300
H	-30.68300	17.49100	-16.39400
O	-27.58800	5.00900	-4.39600
H	-28.36500	5.56900	-4.40500
H	-26.94900	5.49500	-3.87400
O	-22.14200	5.53800	-19.87300
H	-21.23000	5.77100	-20.04800
H	-22.65100	6.12200	-20.43500
O	-29.33300	7.81800	-4.83400
H	-28.59200	8.35600	-5.11000
H	-30.04000	8.44500	-4.67700
O	-23.85800	9.96100	-6.23600
H	-23.44900	9.51900	-6.98100
H	-24.58000	9.38600	-5.98300
O	-27.50500	4.89800	-8.86300
H	-26.65800	5.32200	-8.72000
H	-27.56500	4.24100	-8.16900
O	-23.53500	10.52100	-12.48100
H	-24.16400	10.68600	-13.18400
H	-23.92200	10.93500	-11.71000
O	-19.65600	11.45600	-15.12700
H	-20.34100	11.83600	-15.67700
H	-19.57200	12.06600	-14.39400
O	-24.35500	2.55900	-7.20500
H	-24.59900	3.31300	-7.74200
H	-24.07900	2.93700	-6.37000
O	-22.40100	9.10200	-8.52700
H	-22.18900	9.74600	-9.20400
H	-22.44800	8.26900	-8.99500
O	-24.79800	1.71400	-11.62400
H	-25.66000	2.01200	-11.33200
H	-24.77200	1.93100	-12.55600
O	-27.98200	10.25800	-15.21600
H	-27.51800	10.53700	-16.00600
H	-28.24600	9.35700	-15.39900
O	-31.09000	9.80600	-12.27800
H	-30.51900	10.23400	-12.91700
H	-30.49900	9.52400	-11.58000
O	-29.04500	6.38100	-18.40700
H	-28.34300	6.83300	-17.94000
H	-29.37700	5.73900	-17.77900
O	-30.59700	4.97200	-4.03200
H	-31.22700	5.65400	-3.79900
H	-30.70300	4.86000	-4.97700

O	-35.30300	4.63900	-13.10600
H	-36.09300	4.89500	-13.58200
H	-34.81100	5.45400	-13.00000
O	-21.28600	11.98000	-10.35500
H	-20.46400	11.57800	-10.63700
H	-21.29000	11.87600	-9.40300
O	-33.35100	5.00400	-16.14700
H	-34.14800	5.11500	-15.62900
H	-33.57900	5.34200	-17.01300
O	-27.52100	11.52800	-8.19900
H	-27.45600	11.99000	-7.36300
H	-28.22900	10.89800	-8.07100
O	-37.01900	4.89200	-10.67400
H	-36.76800	5.77700	-10.40800
H	-36.48300	4.71300	-11.44700
O	-13.19600	8.32300	-14.00700
H	-13.94900	7.75400	-14.16200
H	-13.06100	8.29000	-13.05900
O	-27.80600	13.19500	-18.33100
H	-27.94100	14.04000	-17.90300
H	-27.26500	13.39700	-19.09400
O	-31.31900	-0.72600	-1.67000
H	-31.71200	-1.27900	-0.99500
H	-30.50200	-0.41500	-1.27900
O	-34.35300	10.71200	-17.75800
H	-33.84000	11.50700	-17.60800
H	-33.70100	10.01500	-17.82500
O	-32.54300	13.82900	-15.67600
H	-32.36700	14.75100	-15.86400
H	-31.74800	13.51500	-15.24700
O	-28.40200	0.69000	-4.07800
H	-27.66100	1.29000	-4.15700
H	-28.17000	-0.05200	-4.63600
O	-33.25800	5.87500	-5.80000
H	-33.37300	4.97400	-5.49900
H	-34.11400	6.12200	-6.15100
O	-26.17900	13.81700	-4.20100
H	-26.62300	13.03100	-4.51900
H	-25.51700	14.00500	-4.86700
O	-31.42000	15.34700	-9.67500
H	-30.86900	15.21800	-10.44700
H	-32.25900	15.64600	-10.02600
O	-24.08100	14.09700	-6.17300
H	-24.09900	14.91800	-6.66500
H	-23.15900	13.97300	-5.94800
O	-27.62300	11.61200	-5.33100
H	-27.09100	10.85100	-5.56100
H	-28.15200	11.31900	-4.58800
O	-24.45700	-3.28200	-9.80100
H	-24.80000	-2.39200	-9.87700
H	-24.08900	-3.47200	-10.66400
O	-36.38800	9.04100	-16.98200
H	-35.55000	9.45100	-17.19900
H	-36.21000	8.10100	-17.01200
O	-24.49100	6.70900	-21.29400
H	-25.00700	7.47900	-21.05500
H	-24.76400	6.03500	-20.67100
O	-26.90700	1.55400	-16.23500
H	-27.76300	1.16400	-16.41500
H	-26.49400	1.63300	-17.09500
O	-25.37700	-0.91200	-11.83600
H	-25.88000	-0.94900	-12.64900
H	-25.08400	-0.00200	-11.77700
O	-29.13300	-0.82800	-12.83000
H	-28.26700	-1.02400	-13.18900
H	-29.06500	-1.05700	-11.90300
O	-19.51800	9.46200	-19.35900
H	-20.29500	9.12300	-19.80300
H	-18.83900	9.46700	-20.03300
O	-24.70300	4.58100	-19.46800
H	-25.00800	4.89600	-18.61700
H	-23.75000	4.66000	-19.42500
O	-26.25800	10.74000	-17.81900
H	-26.78600	10.00600	-18.13100
H	-26.75900	11.51800	-18.06500
O	-21.27900	8.93500	-12.70200
H	-21.93700	9.62500	-12.61400
H	-20.47500	9.32500	-12.35900
O	-31.52600	1.40200	-15.45800
H	-30.95200	1.18000	-16.19100
H	-30.93900	1.47100	-14.70500
O	-22.46800	7.29000	-14.65200
H	-21.95500	7.92300	-14.14900
H	-21.81800	6.69000	-15.01700
O	-31.19900	3.43300	-21.43800
H	-32.03600	3.77000	-21.11800
H	-31.33900	3.29800	-22.37600
O	-29.22400	-1.72300	-16.73100
H	-29.87800	-2.03900	-17.35500
H	-29.26600	-2.34600	-16.00500
O	-28.11300	8.55800	-24.81500
H	-29.02000	8.32700	-24.61600
H	-27.61600	7.76200	-24.62800
O	-25.20600	17.26800	-21.89600
H	-25.88000	17.57100	-21.28700
H	-24.82000	18.07100	-22.24500
O	-25.99000	4.76500	-12.40600
H	-26.90800	4.98600	-12.56400
H	-25.57400	4.83900	-13.26500
O	-25.20700	6.16400	-17.27500
H	-24.46000	6.67600	-17.58500
H	-24.93800	5.84200	-16.41500
O	-26.93400	8.66000	-20.29300
H	-27.84300	8.37600	-20.19600
H	-26.90500	9.08000	-21.15200
O	-21.70200	12.27000	-16.52400
H	-22.15600	11.42900	-16.46700
H	-22.33300	12.90700	-16.18600
O	-22.75900	3.20000	-15.29400
H	-23.49700	2.68700	-14.96300
H	-22.50600	2.76000	-16.10500
O	-23.25900	14.10800	-15.58900
H	-22.96700	14.66900	-16.30700
H	-22.74900	14.40100	-14.83300
O	-22.65800	-4.02300	-14.69900
H	-22.35200	-4.88300	-14.98700
H	-21.93200	-3.43100	-14.90000
O	-36.36500	6.38000	-16.31100

H	-36.56300	5.78700	-17.03600
H	-36.92100	6.07600	-15.59400
O	-28.38600	6.27300	-14.19200
H	-28.33100	5.32000	-14.26800
H	-29.19700	6.42900	-13.70800
O	-27.23700	10.20100	-22.95400
H	-27.67500	9.48400	-23.41300
H	-26.49500	10.42800	-23.51500
O	-30.44600	4.89000	-16.43300
H	-31.26000	5.19000	-16.02600
H	-29.95000	4.49600	-15.71500
O	-28.29600	14.88600	-14.49900
H	-28.33800	15.11200	-15.42800
H	-27.37900	14.65200	-14.35100
O	-24.84900	5.35400	-7.60100
H	-24.49400	5.58200	-6.74200
H	-24.20200	5.68600	-8.22400
O	-19.11900	8.47600	-16.89600
H	-19.25700	8.66600	-17.82400
H	-19.47600	9.23800	-16.44000
O	-30.95500	2.46200	-10.81700
H	-30.31500	2.32100	-11.51400
H	-31.78700	2.17600	-11.19300
O	-19.09200	10.54400	-11.88500
H	-18.65900	10.45200	-11.03700
H	-18.37900	10.52000	-12.52300
O	-30.17900	11.76200	-14.70300
H	-29.38500	11.26700	-14.90500
H	-30.89000	11.23400	-15.06600
O	-25.35900	14.14400	-10.35200
H	-25.30600	14.71300	-9.58400
H	-26.22800	14.31400	-10.71700
O	-33.87100	5.90700	-18.62700
H	-34.80100	5.84500	-18.41000
H	-33.77500	5.38400	-19.42400
O	-33.95200	2.43200	-16.03900
H	-33.69100	3.34700	-16.14800
H	-33.13000	1.96500	-15.89000
O	-32.48000	3.84600	-8.86900
H	-31.80700	3.63800	-9.51800
H	-33.30700	3.64800	-9.30900
O	-18.23100	10.58600	-9.35200
H	-18.03700	11.49100	-9.10600
H	-18.33100	10.12700	-8.51800
O	-26.57700	0.47200	-6.85300
H	-27.22400	1.17100	-6.94600
H	-25.80700	0.90600	-6.48500
O	-23.16900	10.73700	-3.77800
H	-23.32100	10.50800	-4.69500
H	-23.17900	11.69400	-3.76500
O	-26.40100	8.31000	-8.79300
H	-25.47100	8.46100	-8.96600
H	-26.82200	8.39900	-9.64800
O	-27.60200	12.33500	-11.02300
H	-27.74100	11.96700	-10.15100
H	-28.39000	12.85000	-11.19500
O	-27.00700	17.43600	-19.68100
H	-26.83200	16.96500	-18.86600
H	-27.69400	18.06300	-19.45300
O	-26.05100	11.71300	-13.63400
H	-26.38600	11.67400	-12.73900
H	-26.55100	11.04800	-14.10700
O	-32.07700	7.12000	-8.10900
H	-32.21500	6.38800	-7.50800
H	-31.16600	7.03100	-8.39100
O	-23.11500	9.05900	-23.36100
H	-23.73200	8.33400	-23.45700
H	-23.58400	9.82000	-23.70400
O	-29.44900	6.83200	-8.62100
H	-28.73900	6.19200	-8.66200
H	-29.49200	7.08300	-7.69800
O	-21.46000	13.76900	-5.70000
H	-21.51900	13.08500	-6.36700
H	-21.05800	14.51100	-6.15200
O	-30.73600	3.81900	-6.80100
H	-31.27200	3.36800	-6.14900
H	-31.32400	3.95300	-7.54400
O	-32.44100	20.58500	-12.90700
H	-32.20100	21.21700	-12.22900
H	-33.28400	20.23400	-12.62000
O	-31.32600	3.27800	-24.23900
H	-32.20300	3.15600	-24.60200
H	-30.85000	2.48600	-24.49100
O	-21.45800	11.73300	-7.46900
H	-22.34600	11.39500	-7.34600
H	-20.88800	11.00100	-7.23200
O	-39.80500	8.06500	-14.14400
H	-39.11800	7.75100	-14.73200
H	-39.96100	8.96900	-14.41800
O	-35.00200	2.95400	-9.71600
H	-35.08600	2.34700	-10.45100
H	-35.82400	3.44500	-9.71500
O	-27.92800	16.39300	-10.50300
H	-28.00100	17.19900	-9.99100
H	-28.37000	16.59200	-11.32800
O	-28.13900	15.63000	-16.99200
H	-28.92200	16.17200	-16.89900
H	-27.44000	16.14800	-16.59300
O	-24.82900	1.44000	-14.34900
H	-24.33300	0.68700	-14.66900
H	-25.60100	1.47600	-14.91500
O	-16.97700	7.14400	-12.70700
H	-16.98800	8.02600	-13.07800
H	-17.84500	6.79100	-12.90200
O	-25.46800	16.29200	-8.34700
H	-24.75500	16.92600	-8.41900
H	-25.95900	16.38800	-9.16300
O	-23.74700	10.19700	-16.77500
H	-24.68100	10.31700	-16.94500
H	-23.56900	9.29400	-17.03900
O	-28.24600	3.58100	-14.62200
H	-28.61600	3.02900	-13.93400
H	-27.75000	2.97700	-15.17400
O	-20.35900	6.03900	-16.24000
H	-19.89300	6.87100	-16.31400
H	-19.90800	5.57300	-15.53600
O	-30.41700	14.04100	-7.52600
H	-29.71600	14.67500	-7.37300

H	-30.88800	14.38300	-8.28700
O	-22.29200	12.38300	-19.24200
H	-22.01600	12.26900	-18.33200
H	-23.16100	11.98500	-19.28000
O	-22.61700	6.59600	-9.70100
H	-23.27000	6.84300	-10.35600
H	-22.23900	5.78200	-10.03500
O	-25.65600	16.63200	-15.59800
H	-25.47400	15.81400	-15.13500
H	-24.83300	16.84800	-16.03600
O	-25.32200	15.55800	-18.33700
H	-24.79300	15.04800	-18.95000
H	-25.05900	15.24300	-17.47200
O	-26.56800	7.33800	-3.21400
H	-27.24400	7.23700	-2.54400
H	-25.74500	7.18200	-2.75100
O	-34.82000	13.57500	-10.59300
H	-34.23500	12.82900	-10.46000
H	-35.01800	13.88500	-9.71000
O	-31.96600	16.61000	-15.57700
H	-32.63500	17.09500	-16.06100
H	-32.10500	16.85700	-14.66300
O	-40.22900	6.14500	-11.87300
H	-40.19000	5.61200	-11.07900
H	-40.73400	5.61900	-12.49200
O	-27.82600	18.64400	-8.97900
H	-27.27400	18.80700	-8.21400
H	-27.48500	19.23500	-9.65100
O	-32.84900	0.99200	-8.37600
H	-32.78500	1.85300	-8.78900
H	-32.91300	0.37900	-9.10900
O	-33.37400	3.06400	-4.72900
H	-32.74700	2.68200	-4.11500
H	-34.23100	2.84600	-4.36400
O	-22.97500	13.47000	-3.12400
H	-22.43100	14.08300	-3.61800
H	-23.55600	14.02800	-2.60700
O	-36.59800	4.87200	-18.79700
H	-36.30200	3.96300	-18.85100
H	-37.49900	4.85100	-19.11900
O	-24.68900	16.59300	-11.99200
H	-24.81400	15.79100	-12.50100
H	-24.03100	17.08700	-12.48100
O	-33.06800	2.94700	-12.81500
H	-33.79000	3.56100	-12.95400
H	-32.27900	3.48300	-12.89300
O	-22.62600	14.39300	-10.36400
H	-23.57300	14.27400	-10.43700
H	-22.27700	13.50600	-10.28200
O	-30.26600	0.28300	-6.54100
H	-29.74200	0.52900	-5.77800
H	-30.48400	1.11500	-6.96000
O	-34.86900	2.55300	-18.67300
H	-35.19800	1.67300	-18.85600
H	-34.51500	2.49800	-17.78600
O	-25.62100	14.18900	-14.16400
H	-25.76800	13.26000	-13.98300
H	-24.79600	14.21100	-14.64800
O	-31.75500	2.84000	-18.30600
H	-31.41500	3.36900	-19.02800
H	-31.30700	3.17900	-17.53000
O	-22.01800	15.01200	-13.07700
H	-21.09300	14.80600	-13.21500
H	-22.18600	14.76300	-12.16900
O	-29.06000	1.22400	-8.85600
H	-29.40100	0.42500	-8.45300
H	-29.76600	1.52400	-9.42800
O	-28.40900	-1.69100	-10.40300
H	-27.58600	-2.16500	-10.52500
H	-28.16500	-0.91200	-9.90300
O	-29.76800	1.33800	-20.10700
H	-29.04700	1.71600	-20.61000
H	-30.39100	2.05700	-20.00300
O	-25.62500	2.04200	-19.17100
H	-25.40800	2.92500	-19.47200
H	-26.10500	1.65100	-19.90000
O	-30.88500	5.00100	-12.59400
H	-31.03000	5.31500	-11.70100
H	-31.36200	5.62000	-13.14700
O	-29.02500	1.99100	-12.54600
H	-29.03900	1.09500	-12.88200
H	-28.30800	1.99800	-11.91200
O	-27.17300	2.42500	-10.50000
H	-27.43100	3.34700	-10.50200
H	-27.70400	2.02800	-9.81000
O	-22.22800	15.53000	-17.72100
H	-22.05400	14.96500	-18.47400
H	-21.73500	16.33000	-17.90300
O	-36.41000	7.54200	-9.65500
H	-36.95500	8.31800	-9.52300
H	-35.53200	7.88900	-9.81300
O	-29.62200	0.87300	-17.27700
H	-29.50000	-0.05600	-17.07800
H	-29.91100	0.88700	-18.18900
O	-26.75200	-1.44200	-14.14000
H	-26.73000	-1.37800	-15.09500
H	-26.33600	-2.28100	-13.94600
O	-16.37400	8.29400	-16.10200
H	-15.93100	7.54400	-16.50000
H	-17.25100	8.28500	-16.48500
O	-21.24000	14.84700	-20.62200
H	-20.54500	15.46700	-20.40300
H	-20.89800	13.99600	-20.34600
O	-35.62400	6.66900	-6.76200
H	-36.36600	6.81700	-6.17500
H	-35.94400	6.92900	-7.62500
O	-19.34100	12.29700	-19.09300
H	-18.81600	12.63500	-19.81800
H	-19.42900	11.36100	-19.27600
O	-27.67900	16.21000	-6.96400
H	-27.37100	15.96500	-6.09200
H	-26.88200	16.41500	-7.45200
O	-27.65700	18.36400	-14.73300
H	-28.09700	17.87200	-14.04000
H	-26.90600	17.82100	-14.97400
O	-35.17800	14.55500	-14.73100
H	-34.35200	14.25500	-15.11100
H	-35.52900	13.78600	-14.28200

O	-30.21000	13.90800	-12.66100
H	-30.83200	13.24500	-12.96100
H	-29.53900	13.93400	-13.34400
O	-36.97700	12.47400	-13.77200
H	-37.81400	12.04600	-13.95400
H	-37.00500	12.66700	-12.83500
O	-30.13700	2.38600	-3.06800
H	-30.08900	3.26200	-3.45000
H	-29.59500	1.84400	-3.64100
O	-19.65200	12.93000	-3.78800
H	-20.44200	12.91200	-4.32700
H	-19.88900	13.46900	-3.03300
O	-19.53200	9.77900	-6.47400
H	-19.48200	9.61400	-5.53300
H	-19.64100	8.91200	-6.86500
O	-30.64900	12.45800	-19.18500
H	-30.50000	13.14200	-19.83900
H	-29.84200	12.43900	-18.67000
O	-22.24200	5.76200	-5.15800
H	-21.34100	5.62300	-5.45000
H	-22.16700	5.92800	-4.21900
O	-27.90700	3.13900	-6.31300
H	-28.77700	3.43200	-6.58500
H	-27.67200	3.72300	-5.59200
O	-18.03000	12.19900	-6.23000
H	-18.32200	12.56200	-5.39400
H	-18.64300	11.48600	-6.40900
O	-16.83100	9.76400	-13.71000
H	-15.99600	10.20300	-13.54600
H	-16.69500	9.28800	-14.52900
O	-19.78200	7.32200	-7.76200
H	-19.30600	7.51500	-8.57000
H	-20.70500	7.37800	-8.00800
O	-32.89300	8.48500	-18.17900
H	-33.13900	7.58100	-17.98100
H	-32.72400	8.48600	-19.12100
O	-25.20400	-0.64300	-8.79700
H	-24.68500	0.09300	-9.12200
H	-25.55800	-0.33700	-7.96200
O	-33.62400	4.21700	-20.68000
H	-34.31700	4.29200	-21.33600
H	-33.86900	3.45400	-20.15800
O	-24.95200	-4.22300	-13.27700
H	-24.67200	-4.89100	-12.65000
H	-24.16700	-4.03000	-13.78900
O	-27.14900	14.95300	-21.71400
H	-27.72200	15.57200	-22.16700
H	-26.57600	15.50300	-21.18000
O	-21.08100	4.19900	-9.52900
H	-20.13000	4.29900	-9.50800
H	-21.25100	3.40700	-9.02000
O	-23.06000	1.28400	-9.34300
H	-23.65500	1.56500	-10.03900
H	-23.34500	1.77400	-8.57200
O	-23.92900	15.23700	-20.58300
H	-22.99600	15.17100	-20.38000
H	-24.01700	16.07200	-21.04200
O	-21.30000	14.55300	-23.62300
H	-21.37900	14.21900	-22.72900
H	-20.47900	15.04500	-23.62400
O	-18.60300	7.41900	-10.41500
H	-18.63500	6.48100	-10.22900
H	-17.99300	7.49800	-11.14900
O	-26.76400	6.28900	-24.37200
H	-25.81800	6.16000	-24.30400
H	-27.14300	5.55000	-23.89500
O	-18.45300	4.21100	-10.36900
H	-18.96900	3.42600	-10.55600
H	-17.56900	3.88200	-10.20500
O	-20.79200	-2.04500	-14.41000
H	-20.99000	-2.35400	-13.52500
H	-20.18200	-1.31900	-14.28000
O	-12.78500	8.48100	-11.27500
H	-12.05500	8.22000	-10.71400
H	-13.39400	7.74400	-11.23400
O	-14.70300	6.62200	-10.67700
H	-15.43000	6.48700	-11.28500
H	-15.11900	6.92900	-9.87100
O	-26.88000	10.34100	-2.91200
H	-25.96000	10.51900	-2.71800
H	-26.91500	9.39600	-3.06400
O	-28.58000	4.21400	-24.43700
H	-29.48700	3.91700	-24.36100
H	-28.30200	3.91700	-25.30400
O	-37.66200	5.53500	-13.98700
H	-37.98200	4.68100	-14.28000
H	-38.20900	5.75000	-13.23100
O	-33.88400	8.25100	-10.27000
H	-33.66600	9.18300	-10.25100
H	-33.47200	7.89200	-9.48500
O	-26.96400	-1.24200	-18.28300
H	-27.77400	-1.58800	-17.90700
H	-26.27500	-1.55200	-17.69400
O	-25.03500	-2.16400	-16.66000
H	-24.48200	-1.74500	-16.00100
H	-24.79800	-3.09100	-16.61800
O	-29.01100	3.82400	-0.88000
H	-29.11200	4.77400	-0.94900
H	-29.22900	3.49600	-1.75200
O	-29.81000	1.07300	-24.29600
H	-28.94800	1.39800	-24.03600
H	-29.62500	0.28600	-24.80800
O	-37.79300	9.15500	-12.01800
H	-38.48700	8.74000	-12.53100
H	-37.04600	9.19700	-12.61400
O	-21.65700	-0.05400	-17.15200
H	-22.23700	0.60400	-17.53400
H	-21.92300	-0.87700	-17.56300
O	-23.20700	-0.65400	-14.95000
H	-22.70700	-0.54200	-15.75900
H	-22.56300	-0.95800	-14.31000
O	-29.14800	7.25400	-2.06300
H	-29.63200	8.05400	-1.85700
H	-29.06200	7.26500	-3.01700
O	-35.88000	9.08700	-14.14100
H	-35.90300	9.18600	-15.09300
H	-35.57000	9.93300	-13.81900
O	-33.22100	11.14400	-10.02600

H	-32.26700	11.11300	-10.09300
H	-33.42000	10.68300	-9.21100
O	-32.63100	9.71500	-7.45900
H	-31.81200	10.20900	-7.43600
H	-32.35700	8.79800	-7.48800
O	-22.87400	2.10600	-18.09700
H	-23.76200	2.39100	-18.31500
H	-22.45900	1.94500	-18.94400
O	-19.52300	2.13000	-14.38100
H	-18.74700	2.62000	-14.10900
H	-20.24800	2.55600	-13.92400
O	-17.08600	2.84300	-13.32400
H	-16.49100	2.60300	-14.03400
H	-17.01300	2.12400	-12.69600
O	-34.86600	16.68600	-13.00900
H	-35.00100	15.92600	-13.57600
H	-35.28300	17.41000	-13.47700
O	-23.73000	11.87000	-24.00200
H	-23.02600	12.07500	-23.38600
H	-24.45400	12.43800	-23.73700
O	-22.90000	7.62100	-17.88200
H	-22.95700	8.07800	-18.72100
H	-22.34700	6.86000	-18.05900
O	-19.09000	3.41000	-17.05700
H	-19.34600	3.01000	-16.22600
H	-19.71500	4.12400	-17.18200
O	-14.77900	6.15000	-14.54900
H	-15.33900	6.21900	-13.77600
H	-15.38400	5.97400	-15.26900
O	-31.70900	17.58300	-12.79800
H	-31.82600	18.52800	-12.90000
H	-32.53000	17.28100	-12.40900
O	-21.82100	12.30800	-21.95200
H	-20.98700	11.87600	-22.13400
H	-21.98300	12.13700	-21.02400
O	-22.16700	8.81200	-20.58600
H	-22.22200	8.64600	-21.52700
H	-22.79200	9.51900	-20.43000
O	-37.37800	4.04800	-6.76000
H	-36.73200	3.53800	-6.27100
H	-37.58600	4.78500	-6.18600
O	-24.85200	11.11600	-1.26100
H	-23.98300	11.29000	-1.62200
H	-25.33200	11.93600	-1.38300
O	-28.85700	-1.68800	-21.00800
H	-28.86900	-0.74300	-20.85100
H	-27.96500	-1.87100	-21.30200
O	-30.68400	18.23600	-9.58200
H	-29.78100	18.26800	-9.26500
H	-30.91000	17.30600	-9.56700
O	-28.92800	16.98900	-12.92100
H	-29.87900	16.98900	-13.03500
H	-28.62500	16.24200	-13.43700
O	-25.04200	-5.09300	-16.75300
H	-25.80900	-5.64800	-16.60800
H	-24.63100	-5.02100	-15.89200
O	-33.03800	1.19500	-2.66300
H	-32.17500	0.83900	-2.45200
H	-33.61100	0.85600	-1.97500
O	-13.19300	22.82500	-11.85600
H	-12.55500	22.70600	-12.56000
H	-13.31100	23.77300	-11.79800
O	-16.38400	22.77800	-11.92000
H	-16.72200	22.66200	-12.80800
H	-15.51500	22.37700	-11.94000
O	-20.52500	24.63200	-15.93100
H	-21.08900	24.54100	-16.69900
H	-20.64000	23.81200	-15.45000
O	-15.90200	23.93100	-5.96200
H	-15.91300	23.53700	-6.83400
H	-15.74000	23.19800	-5.36800
O	-22.97900	26.12700	-13.09200
H	-22.18700	25.78600	-13.50800
H	-22.73900	26.23600	-12.17200
O	-14.94500	28.29900	-20.61900
H	-15.59700	28.88400	-21.00600
H	-14.15100	28.82900	-20.56000
O	-21.02000	25.38800	-20.22000
H	-20.24700	24.84000	-20.35900
H	-20.66700	26.25200	-20.00900
O	-19.18100	26.71500	-7.01400
H	-19.47200	27.62000	-7.12900
H	-18.54600	26.75700	-6.29900
O	-18.25600	24.09300	-10.42400
H	-17.65900	23.86800	-11.13800
H	-18.19800	23.35100	-9.82100
O	-18.68300	21.54700	-22.58900
H	-18.43100	21.02500	-23.35000
H	-19.62100	21.38300	-22.48600
O	-13.63900	20.55000	-14.78100
H	-12.85300	20.13200	-15.13200
H	-13.37500	21.45500	-14.61300
O	-13.46800	25.98800	-20.08200
H	-14.10300	26.06100	-19.37000
H	-13.79800	26.57700	-20.76000
O	-8.23800	20.48800	-13.87800
H	-7.50500	19.91800	-13.64400
H	-8.99400	20.10000	-13.43700
O	-18.96400	23.45000	-20.38600
H	-19.08000	22.77400	-19.71800
H	-18.85300	22.96300	-21.20300
O	-16.62000	22.91100	-16.68100
H	-16.98500	22.38400	-15.97000
H	-15.76400	22.52100	-16.85500
O	-10.71500	14.20000	-9.41800
H	-11.04700	15.07000	-9.64200
H	-11.31100	13.88400	-8.73900
O	-16.98000	17.94000	-21.68500
H	-16.81200	18.80800	-21.31800
H	-17.17000	18.10200	-22.60900
O	-14.22200	26.40100	-10.01900
H	-14.52200	27.30400	-10.12700
H	-14.56700	26.13500	-9.16700
O	-20.77100	22.85300	-13.98500
H	-21.69600	22.72000	-13.77700
H	-20.52300	23.63200	-13.48800
O	-10.82300	18.92000	-12.33900
H	-11.73100	18.85100	-12.63300

H	-10.87400	19.39700	-11.51100
O	-17.00100	20.39500	-20.70100
H	-17.12100	20.84100	-19.86200
H	-17.69600	20.74400	-21.25900
O	-23.41000	22.49600	-13.15100
H	-23.28100	22.75000	-12.23700
H	-24.09000	23.08900	-13.47000
O	-19.24000	33.16600	-17.05200
H	-19.31800	34.11700	-16.97300
H	-19.84400	32.82000	-16.39400
O	-16.74800	20.33900	-4.39600
H	-17.52500	20.89800	-4.40500
H	-16.10900	20.82400	-3.87400
O	-8.95600	17.12100	-10.56100
H	-9.59900	17.41500	-11.20700
H	-9.46100	17.00700	-9.75600
O	-11.30200	20.86800	-19.87300
H	-10.39100	21.10000	-20.04800
H	-11.81100	21.45100	-20.43500
O	-18.49400	23.14700	-4.83400
H	-17.75200	23.68600	-5.11000
H	-19.20000	23.77400	-4.67700
O	-13.01800	25.29000	-6.23600
H	-12.61000	24.84800	-6.98100
H	-13.74100	24.71500	-5.98300
O	-16.66500	20.22700	-8.86300
H	-15.81900	20.65100	-8.72000
H	-16.72500	19.57100	-8.16900
O	-12.69500	25.85000	-12.48100
H	-13.32500	26.01500	-13.18400
H	-13.08300	26.26400	-11.71000
O	-8.81700	26.78600	-15.12700
H	-9.50100	27.16500	-15.67700
H	-8.73200	27.39500	-14.39400
O	-13.51500	17.88800	-7.20500
H	-13.75900	18.64200	-7.74200
H	-13.23900	18.26600	-6.37000
O	-11.56100	24.43200	-8.52700
H	-11.35000	25.07500	-9.20400
H	-11.60800	23.59800	-8.99500
O	-13.95900	17.04300	-11.62400
H	-14.82000	17.34100	-11.33200
H	-13.93300	17.26000	-12.55600
O	-17.14300	25.58800	-15.21600
H	-16.67900	25.86600	-16.00600
H	-17.40700	24.68600	-15.39900
O	-20.25000	25.13500	-12.27800
H	-19.68000	25.56300	-12.91700
H	-19.65900	24.85300	-11.58000
O	-18.20600	21.71000	-18.40700
H	-17.50300	22.16200	-17.94000
H	-18.53800	21.06800	-17.77900
O	-19.75800	20.30100	-4.03200
H	-20.38800	20.98300	-3.79900
H	-19.86400	20.18900	-4.97700
O	-24.46300	19.96800	-13.10600
H	-25.25300	20.22400	-13.58200
H	-23.97200	20.78300	-13.00000
O	-10.44700	27.30900	-10.35500
H	-9.62500	26.90700	-10.63700
H	-10.45100	27.20600	-9.40300
O	-22.51200	20.33300	-16.14700
H	-23.30900	20.44500	-15.62900
H	-22.73900	20.67100	-17.01300
O	-16.68100	26.85700	-8.19900
H	-16.61700	27.31900	-7.36300
H	-17.39000	26.22700	-8.07100
O	-26.17900	20.22100	-10.67400
H	-25.92900	21.10600	-10.40800
H	-25.64300	20.04200	-11.44700
O	-2.35600	23.65300	-14.00700
H	-3.11000	23.08300	-14.16200
H	-2.22100	23.61900	-13.05900
O	-16.96600	28.52400	-18.33100
H	-17.10200	29.36900	-17.90300
H	-16.42500	28.72600	-19.09400
O	-20.48000	14.60300	-1.67000
H	-20.87300	14.05000	-0.99500
H	-19.66300	14.91400	-1.27900
O	-23.51300	26.04200	-17.75800
H	-23.00100	26.83600	-17.60800
H	-22.86100	25.34400	-17.82500
O	-21.70400	29.15800	-15.67600
H	-21.52700	30.08000	-15.86400
H	-20.90800	28.84400	-15.24700
O	-17.56300	16.01900	-4.07800
H	-16.82200	16.62000	-4.15700
H	-17.33100	15.27700	-4.63600
O	-22.41900	21.20500	-5.80000
H	-22.53300	20.30300	-5.49900
H	-23.27400	21.45100	-6.15100
O	-15.34000	29.14600	-4.20100
H	-15.78300	28.36000	-4.51900
H	-14.67800	29.33400	-4.86700
O	-20.58100	30.67600	-9.67500
H	-20.03000	30.54800	-10.44700
H	-21.42000	30.97500	-10.02600
O	-13.24100	29.42700	-6.17300
H	-13.26000	30.24800	-6.66500
H	-12.31900	29.30200	-5.94800
O	-16.78400	26.94100	-5.33100
H	-16.25100	26.18000	-5.56100
H	-17.31300	26.64800	-4.58800
O	-13.61700	12.04700	-9.80100
H	-13.96100	12.93700	-9.87700
H	-13.24900	11.85700	-10.66400
O	-25.54900	24.37000	-16.98200
H	-24.71100	24.78000	-17.19900
H	-25.37100	23.43000	-17.01200
O	-13.65200	22.03800	-21.29400
H	-14.16700	22.80800	-21.05500
H	-13.92400	21.36400	-20.67100
O	-16.06700	16.88300	-16.23500
H	-16.92300	16.49400	-16.41500
H	-15.65500	16.96200	-17.09500
O	-14.53700	14.41800	-11.83600
H	-15.04100	14.38100	-12.64900
H	-14.24400	15.32700	-11.77700

O	-18.29300	14.50100	-12.83000
H	-17.42800	14.30600	-13.18900
H	-18.22500	14.27200	-11.90300
O	-8.67900	24.79100	-19.35900
H	-9.45600	24.45200	-19.80300
H	-8.00000	24.79600	-20.03300
O	-13.86300	19.91100	-19.46800
H	-14.16800	20.22500	-18.61700
H	-12.91000	19.98900	-19.42500
O	-15.41900	26.07000	-17.81900
H	-15.94700	25.33500	-18.13100
H	-15.92000	26.84700	-18.06500
O	-10.44000	24.26500	-12.70200
H	-11.09700	24.95400	-12.61400
H	-9.63600	24.65400	-12.35900
O	-20.68700	16.73200	-15.45800
H	-20.11300	16.50900	-16.19100
H	-20.10000	16.80000	-14.70500
O	-11.62900	22.61900	-14.65200
H	-11.11600	23.25200	-14.14900
H	-10.97900	22.01900	-15.01700
O	-20.36000	18.76200	-21.43800
H	-21.19600	19.10000	-21.11800
H	-20.50000	18.62700	-22.37600
O	-18.38500	13.60600	-16.73100
H	-19.03800	13.29100	-17.35500
H	-18.42700	12.98300	-16.00500
O	-17.27400	23.88800	-24.81500
H	-18.18100	23.65700	-24.61600
H	-16.77600	23.09100	-24.62800
O	-14.36700	32.59700	-21.89600
H	-15.04100	32.90100	-21.28700
H	-13.98000	33.40000	-22.24500
O	-15.15100	20.09400	-12.40600
H	-16.06900	20.31500	-12.56400
H	-14.73500	20.16900	-13.26500
O	-14.36700	21.49300	-17.27500
H	-13.62000	22.00500	-17.58500
H	-14.09800	21.17100	-16.41500
O	-16.09500	23.98900	-20.29300
H	-17.00400	23.70500	-20.19600
H	-16.06500	24.40900	-21.15200
O	-10.86300	27.60000	-16.52400
H	-11.31700	26.75900	-16.46700
H	-11.49300	28.23600	-16.18600
O	-11.92000	18.52900	-15.29400
H	-12.65700	18.01700	-14.96300
H	-11.66700	18.08900	-16.10500
O	-12.42000	29.43700	-15.58900
H	-12.12700	29.99800	-16.30700
H	-11.91000	29.73000	-14.83300
O	-11.81800	11.30600	-14.69900
H	-11.51200	10.44600	-14.98700
H	-11.09300	11.89800	-14.90000
O	-25.52600	21.70900	-16.31100
H	-25.72300	21.11600	-17.03600
H	-26.08100	21.40500	-15.59400
O	-17.54600	21.60200	-14.19200
H	-17.49100	20.64900	-14.26800
H	-18.35700	21.75800	-13.70800
O	-16.39700	25.53000	-22.95400
H	-16.83500	24.81300	-23.41300
H	-15.65600	25.75700	-23.51500
O	-19.60700	20.21900	-16.43300
H	-20.42000	20.51900	-16.02600
H	-19.11100	19.82500	-15.71500
O	-17.45600	30.21500	-14.49900
H	-17.49900	30.44100	-15.42800
H	-16.54000	29.98200	-14.35100
O	-14.01000	20.68400	-7.60100
H	-13.65500	20.91100	-6.74200
H	-13.36200	21.01600	-8.22400
O	-8.28000	23.80600	-16.89600
H	-8.41800	23.99600	-17.82400
H	-8.63600	24.56700	-16.44000
O	-20.11600	17.79100	-10.81700
H	-19.47600	17.65000	-11.51400
H	-20.94800	17.50500	-11.19300
O	-8.25300	25.87300	-11.88500
H	-7.81900	25.78100	-11.03700
H	-7.54000	25.84900	-12.52300
O	-19.34000	27.09200	-14.70300
H	-18.54600	26.59700	-14.90500
H	-20.05000	26.56300	-15.06600
O	-14.52000	29.47300	-10.35200
H	-14.46600	30.04200	-9.58400
H	-15.38900	29.64400	-10.71700
O	-23.03100	21.23600	-18.62700
H	-23.96200	21.17400	-18.41000
H	-22.93600	20.71300	-19.42400
O	-23.11300	17.76100	-16.03900
H	-22.85200	18.67600	-16.14800
H	-22.29100	17.29400	-15.89000
O	-21.64000	19.17500	-8.86900
H	-20.96800	18.96700	-9.51800
H	-22.46700	18.97700	-9.30900
O	-7.39100	25.91600	-9.35200
H	-7.19800	26.82000	-9.10600
H	-7.49100	25.45600	-8.51800
O	-15.73800	15.80100	-6.85300
H	-16.38500	16.50000	-6.94600
H	-14.96800	16.23500	-6.48500
O	-12.33000	26.06600	-3.77800
H	-12.48100	25.83700	-4.69500
H	-12.33900	27.02300	-3.76500
O	-15.56100	23.63900	-8.79300
H	-14.63200	23.79100	-8.96600
H	-15.98200	23.72800	-9.64800
O	-16.76200	27.66400	-11.02300
H	-16.90100	27.29600	-10.15100
H	-17.55100	28.17900	-11.19500
O	-16.16800	32.76500	-19.68100
H	-15.99200	32.29400	-18.86600
H	-16.85400	33.39200	-19.45300
O	-15.21200	27.04300	-13.63400
H	-15.54700	27.00300	-12.73900
H	-15.71200	26.37700	-14.10700
O	-21.23800	22.44900	-8.10900

H	-21.37600	21.71700	-7.50800
H	-20.32700	22.36000	-8.39100
O	-12.27600	24.38800	-23.36100
H	-12.89300	23.66300	-23.45700
H	-12.74400	25.14900	-23.70400
O	-18.60900	22.16100	-8.62100
H	-17.89900	21.52100	-8.66200
H	-18.65200	22.41300	-7.69800
O	-10.62100	29.09800	-5.70000
H	-10.67900	28.41400	-6.36700
H	-10.21900	29.84000	-6.15200
O	-19.89600	19.14800	-6.80100
H	-20.43300	18.69700	-6.14900
H	-20.48500	19.28300	-7.54400
O	-21.60200	35.91400	-12.90700
H	-21.36200	36.54600	-12.22900
H	-22.44500	35.56300	-12.62000
O	-20.48600	18.60700	-24.23900
H	-21.36300	18.48600	-24.60200
H	-20.01000	17.81500	-24.49100
O	-10.61900	27.06200	-7.46900
H	-11.50600	26.72500	-7.34600
H	-10.04900	26.33000	-7.23200
O	-28.96600	23.39400	-14.14400
H	-28.27900	23.08100	-14.73200
H	-29.12100	24.29900	-14.41800
O	-24.16200	18.28400	-9.71600
H	-24.24700	17.67600	-10.45100
H	-24.98400	18.77400	-9.71500
O	-17.08900	31.72200	-10.50300
H	-17.16200	32.52800	-9.99100
H	-17.53100	31.92100	-11.32800
O	-17.30000	30.95900	-16.99200
H	-18.08300	31.50200	-16.89900
H	-16.60000	31.47700	-16.59300
O	-13.99000	16.76900	-14.34900
H	-13.49300	16.01600	-14.66900
H	-14.76100	16.80500	-14.91500
O	-6.13700	22.47300	-12.70700
H	-6.14800	23.35500	-13.07800
H	-7.00500	22.12000	-12.90200
O	-14.62900	31.62100	-8.34700
H	-13.91600	32.25600	-8.41900
H	-15.12000	31.71700	-9.16300
O	-12.90700	25.52600	-16.77500
H	-13.84100	25.64600	-16.94500
H	-12.72900	24.62300	-17.03900
O	-17.40700	18.91100	-14.62200
H	-17.77700	18.35800	-13.93400
H	-16.91100	18.30600	-15.17400
O	-9.52000	21.36800	-16.24000
H	-9.05400	22.20100	-16.31400
H	-9.06900	20.90200	-15.53600
O	-19.57800	29.37000	-7.52600
H	-18.87700	30.00400	-7.37300
H	-20.04800	29.71200	-8.28700
O	-11.45200	27.71200	-19.24200
H	-11.17600	27.59800	-18.33200
H	-12.32200	27.31400	-19.28000
O	-11.54800	13.73600	-12.20100
H	-12.46200	13.96600	-12.03300
H	-11.11900	13.82000	-11.35000
O	-11.77800	21.92500	-9.70100
H	-12.43000	22.17200	-10.35600
H	-11.40000	21.11100	-10.03500
O	-14.81700	31.96100	-15.59800
H	-14.63500	31.14300	-15.13500
H	-13.99400	32.17800	-16.03600
O	-14.48300	30.88800	-18.33700
H	-13.95400	30.37700	-18.95000
H	-14.22000	30.57200	-17.47200
O	-15.72900	22.66700	-3.21400
H	-16.40500	22.56600	-2.54400
H	-14.90600	22.51100	-2.75100
O	-23.98100	28.90400	-10.59300
H	-23.39600	28.15800	-10.46000
H	-24.17800	29.21400	-9.71000
O	-21.12700	31.93900	-15.57700
H	-21.79600	32.42400	-16.06100
H	-21.26600	32.18600	-14.66300
O	-29.38900	21.47500	-11.87300
H	-29.35100	20.94100	-11.07900
H	-29.89500	20.94800	-12.49200
O	-16.98700	33.97300	-8.97900
H	-16.43500	34.13700	-8.21400
H	-16.64600	34.56400	-9.65100
O	-22.01000	16.32100	-8.37600
H	-21.94600	17.18200	-8.78900
H	-22.07300	15.70800	-9.10900
O	-22.53500	18.39300	-4.72900
H	-21.90800	18.01100	-4.11500
H	-23.39200	18.17500	-4.36400
O	-12.13500	28.80000	-3.12400
H	-11.59100	29.41300	-3.61800
H	-12.71700	29.35800	-2.60700
O	-25.75800	20.20100	-18.79700
H	-25.46300	19.29200	-18.85100
H	-26.65900	20.18000	-19.11900
O	-13.85000	31.92200	-11.99200
H	-13.97500	31.12100	-12.50100
H	-13.19200	32.41600	-12.48100
O	-22.22900	18.27700	-12.81500
H	-22.95000	18.89000	-12.95400
H	-21.44000	18.81200	-12.89300
O	-11.78600	29.72300	-10.36400
H	-12.73300	29.60300	-10.43700
H	-11.43800	28.83500	-10.28200
O	-19.42600	15.61200	-6.54100
H	-18.90300	15.85800	-5.77800
H	-19.64500	16.44400	-6.96000
O	-24.03000	17.88200	-18.67300
H	-24.35800	17.00200	-18.85600
H	-23.67600	17.82700	-17.78600
O	-14.78200	29.51800	-14.16400
H	-14.92900	28.59000	-13.98300
H	-13.95600	29.54000	-14.64800
O	-20.91500	18.17000	-18.30600
H	-20.57600	18.69800	-19.02800

H	-20.46800	18.50800	-17.53000
O	-11.17800	30.34100	-13.07700
H	-10.25300	30.13500	-13.21500
H	-11.34700	30.09200	-12.16900
O	-18.22100	16.55300	-8.85600
H	-18.56100	15.75400	-8.45300
H	-18.92700	16.85400	-9.42800
O	-17.56900	13.63800	-10.40300
H	-16.74700	13.16400	-10.52500
H	-17.32600	14.41700	-9.90300
O	-18.92900	16.66700	-20.10700
H	-18.20800	17.04500	-20.61000
H	-19.55200	17.38600	-20.00300
O	-14.78500	17.37200	-19.17100
H	-14.56900	18.25400	-19.47200
H	-15.26600	16.98000	-19.90000
O	-20.04500	20.33000	-12.59400
H	-20.19100	20.64400	-11.70100
H	-20.52300	20.94900	-13.14700
O	-18.18600	17.32000	-12.54600
H	-18.20000	16.42500	-12.88200
H	-17.46900	17.32800	-11.91200
O	-16.33300	17.75400	-10.50000
H	-16.59100	18.67600	-10.50200
H	-16.86500	17.35700	-9.81000
O	-11.38900	30.85900	-17.72100
H	-11.21500	30.29400	-18.47400
H	-10.89600	31.65900	-17.90300
O	-25.57100	22.87100	-9.65500
H	-26.11600	23.64700	-9.52300
H	-24.69300	23.21800	-9.81300
O	-18.78200	16.20200	-17.27700
H	-18.66100	15.27400	-17.07800
H	-19.07200	16.21600	-18.18900
O	-15.91300	13.88700	-14.14000
H	-15.89100	13.95100	-15.09500
H	-15.49600	13.04800	-13.94600
O	-5.53500	23.62300	-16.10200
H	-5.09200	22.87400	-16.50000
H	-6.41200	23.61400	-16.48500
O	-10.40100	30.17600	-20.62200
H	-9.70600	30.79700	-20.40300
H	-10.05900	29.32600	-20.34600
O	-24.78400	21.99800	-6.76200
H	-25.52600	22.14600	-6.17500
H	-25.10500	22.25900	-7.62500
O	-8.50200	27.62600	-19.09300
H	-7.97700	27.96500	-19.81800
H	-8.59000	26.69100	-19.27600
O	-16.84000	31.53900	-6.96400
H	-16.53100	31.29400	-6.09200
H	-16.04200	31.74400	-7.45200
O	-16.81800	33.69300	-14.73300
H	-17.25700	33.20100	-14.04000
H	-16.06700	33.15000	-14.97400
O	-24.33900	29.88400	-14.73100
H	-23.51300	29.58500	-15.11100
H	-24.68900	29.11500	-14.28200
O	-19.37000	29.23700	-12.66100
H	-19.99200	28.57400	-12.96100
H	-18.70000	29.26300	-13.34400
O	-26.13800	27.80300	-13.77200
H	-26.97500	27.37500	-13.95400
H	-26.16500	27.99600	-12.83500
O	-19.29800	17.71500	-3.06800
H	-19.24900	18.59100	-3.45000
H	-18.75600	17.17300	-3.64100
O	-8.81300	28.25900	-3.78800
H	-9.60300	28.24200	-4.32700
H	-9.04900	28.79800	-3.03300
O	-8.69300	25.10800	-6.47400
H	-8.64300	24.94300	-5.53300
H	-8.80100	24.24100	-6.86500
O	-19.80900	27.78700	-19.18500
H	-19.66000	28.47100	-19.83900
H	-19.00200	27.76800	-18.67000
O	-11.40200	21.09200	-5.15800
H	-10.50100	20.95200	-5.45000
H	-11.32800	21.25700	-4.21900
O	-17.06800	18.46800	-6.31300
H	-17.93800	18.76200	-6.58500
H	-16.83200	19.05200	-5.59200
O	-7.19100	27.52800	-6.23000
H	-7.48200	27.89100	-5.39400
H	-7.80400	26.81500	-6.40900
O	-5.99100	25.09300	-13.71000
H	-5.15700	25.53200	-13.54600
H	-5.85500	24.61700	-14.52900
O	-8.94200	22.65100	-7.76200
H	-8.46700	22.84400	-8.57000
H	-9.86600	22.70700	-8.00800
O	-22.05400	23.81400	-18.17900
H	-22.29900	22.91000	-17.98100
H	-21.88400	23.81600	-19.12100
O	-14.36400	14.68600	-8.79700
H	-13.84600	15.42200	-9.12200
H	-14.71900	14.99300	-7.96200
O	-22.78400	19.54700	-20.68000
H	-23.47700	19.62100	-21.33600
H	-23.02900	18.78300	-20.15800
O	-14.11300	11.10600	-13.27700
H	-13.83300	10.43900	-12.65000
H	-13.32800	11.29900	-13.78900
O	-16.31000	30.28200	-21.71400
H	-16.88200	30.90100	-22.16700
H	-15.73600	30.83200	-21.18000
O	-10.24200	19.52800	-9.52900
H	-9.29000	19.62800	-9.50800
H	-10.41200	18.73600	-9.02000
O	-12.22100	16.61400	-9.34300
H	-12.81600	16.89400	-10.03900
H	-12.50600	17.10300	-8.57200
O	-13.09000	30.56600	-20.58300
H	-12.15700	30.50000	-20.38000
H	-13.17800	31.40100	-21.04200
O	-10.46100	29.88200	-23.62300
H	-10.54000	29.54800	-22.72900
H	-9.64000	30.37500	-23.62400

O	-7.76300	22.74900	-10.41500
H	-7.79600	21.81000	-10.22900
H	-7.15300	22.82700	-11.14900
O	-15.92500	21.61800	-24.37200
H	-14.97900	21.48900	-24.30400
H	-16.30300	20.88000	-23.89500
O	-7.61400	19.54000	-10.36900
H	-8.13000	18.75600	-10.55600
H	-6.73000	19.21200	-10.20500
O	-9.95300	13.28400	-14.41000
H	-10.15000	12.97500	-13.52500
H	-9.34300	14.01000	-14.28000
O	-1.94600	23.81000	-11.27500
H	-1.21500	23.54900	-10.71400
H	-2.55500	23.07300	-11.23400
O	-3.86400	21.95100	-10.67700
H	-4.59100	21.81700	-11.28500
H	-4.27900	22.25900	-9.87100
O	-16.04100	25.67000	-2.91200
H	-15.12100	25.84800	-2.71800
H	-16.07600	24.72600	-3.06400
O	-17.74100	19.54300	-24.43700
H	-18.64700	19.24700	-24.36100
H	-17.46300	19.24600	-25.30400
O	-26.82300	20.86400	-13.98700
H	-27.14300	20.01000	-14.28000
H	-27.36900	21.07900	-13.23100
O	-23.04500	23.58000	-10.27000
H	-22.82600	24.51200	-10.25100
H	-22.63200	23.22100	-9.48500
O	-16.12400	14.08700	-18.28300
H	-16.93400	13.74200	-17.90700
H	-15.43600	13.77800	-17.69400
O	-14.19500	13.16500	-16.66000
H	-13.64200	13.58400	-16.00100
H	-13.95800	12.23800	-16.61800
O	-18.17200	19.15300	-0.88000
H	-18.27200	20.10300	-0.94900
H	-18.39000	18.82500	-1.75200
O	-18.97100	16.40200	-24.29600
H	-18.10900	16.72700	-24.03600
H	-18.78600	15.61500	-24.80800
O	-26.95400	24.48400	-12.01800
H	-27.64800	24.07000	-12.53100
H	-26.20700	24.52600	-12.61400
O	-10.81700	15.27500	-17.15200
H	-11.39800	15.93300	-17.53400
H	-11.08400	14.45300	-17.56300
O	-12.36700	14.67500	-14.95000
H	-11.86700	14.78700	-15.75900
H	-11.72400	14.37100	-14.31000
O	-18.30900	22.58300	-2.06300
H	-18.79200	23.38300	-1.85700
H	-18.22300	22.59400	-3.01700
O	-25.04000	24.41600	-14.14100
H	-25.06300	24.51500	-15.09300
H	-24.73000	25.26200	-13.81900
O	-22.38200	26.47400	-10.02600
H	-21.42700	26.44200	-10.09300
H	-22.58000	26.01300	-9.21100
O	-21.79200	25.04400	-7.45900
H	-20.97200	25.53800	-7.43600
H	-21.51800	24.12700	-7.48800
O	-12.03500	17.43500	-18.09700
H	-12.92200	17.72000	-18.31500
H	-11.62000	17.27400	-18.94400
O	-8.68400	17.45900	-14.38100
H	-7.90800	17.94900	-14.10900
H	-9.40900	17.88500	-13.92400
O	-6.24600	18.17200	-13.32400
H	-5.65200	17.93200	-14.03400
H	-6.17400	17.45300	-12.69600
O	-24.02700	32.01600	-13.00900
H	-24.16100	31.25600	-13.57600
H	-24.44300	32.74000	-13.47700
O	-12.89100	27.19900	-24.00200
H	-12.18700	27.40400	-23.38600
H	-13.61400	27.76700	-23.73700
O	-12.06100	22.95000	-17.88200
H	-12.11700	23.40700	-18.72100
H	-11.50800	22.18900	-18.05900
O	-8.25000	18.73900	-17.05700
H	-8.50700	18.33900	-16.22600
H	-8.87500	19.45400	-17.18200
O	-3.93900	21.47900	-14.54900
H	-4.49900	21.54800	-13.77600
H	-4.54500	21.30300	-15.26900
O	-20.87000	32.91200	-12.79800
H	-20.98600	33.85700	-12.90000
H	-21.69000	32.61000	-12.40900
O	-10.98200	27.63700	-21.95200
H	-10.14700	27.20600	-22.13400
H	-11.14400	27.46600	-21.02400
O	-11.32700	24.14100	-20.58600
H	-11.38300	23.97500	-21.52700
H	-11.95300	24.84800	-20.43000
O	-26.53800	19.37800	-6.76000
H	-25.89200	18.86700	-6.27100
H	-26.74600	20.11400	-6.18600
O	-14.01300	26.44600	-1.26100
H	-13.14400	26.62000	-1.62200
H	-14.49200	27.26500	-1.38300
O	-18.01800	13.64200	-21.00800
H	-18.03000	14.58600	-20.85100
H	-17.12500	13.45900	-21.30200
O	-19.84400	33.56500	-9.58200
H	-18.94100	33.59800	-9.26500
H	-20.07000	32.63500	-9.56700
O	-18.08900	32.31800	-12.92100
H	-19.03900	32.31900	-13.03500
H	-17.78600	31.57200	-13.43700
O	-14.20300	10.23600	-16.75300
H	-14.96900	9.68200	-16.60800
H	-13.79200	10.30800	-15.89200
O	-22.19800	16.52400	-2.66300
H	-21.33500	16.16800	-2.45200
H	-22.77200	16.18600	-1.97500
O	-2.35400	38.15400	-11.85600

H	-1.71600	38.03500	-12.56000
H	-2.47100	39.10300	-11.79800
O	-5.54500	38.10700	-11.92000
H	-5.88300	37.99100	-12.80800
H	-4.67600	37.70600	-11.94000
O	-9.68600	39.96100	-15.93100
H	-10.24900	39.87000	-16.69900
H	-9.80100	39.14100	-15.45000
O	-5.06300	39.26100	-5.96200
H	-5.07400	38.86600	-6.83400
H	-4.90100	38.52700	-5.36800
O	-10.18000	40.71700	-20.22000
H	-9.40700	40.17000	-20.35900
H	-9.82800	41.58100	-20.00900
O	-7.41600	39.42200	-10.42400
H	-6.82000	39.19800	-11.13800
H	-7.35900	38.68100	-9.82100
O	-7.84400	36.87600	-22.58900
H	-7.59100	36.35400	-23.35000
H	-8.78100	36.71300	-22.48600
O	-2.79900	35.88000	-14.78100
H	-2.01300	35.46200	-15.13200
H	-2.53600	36.78400	-14.61300
O	-2.62800	41.31700	-20.08200
H	-3.26400	41.39000	-19.37000
H	-2.95900	41.90600	-20.76000
O	2.60100	35.81700	-13.87800
H	3.33400	35.24700	-13.64400
H	1.84500	35.42900	-13.43700
O	-8.12400	38.77900	-20.38600
H	-8.24100	38.10300	-19.71800
H	-8.01400	38.29200	-21.20300
O	-5.78100	38.24000	-16.68100
H	-6.14500	37.71300	-15.97000
H	-4.92400	37.85000	-16.85500
O	0.12400	29.52900	-9.41800
H	-0.20800	30.39900	-9.64200
H	-0.47200	29.21300	-8.73900
O	-6.14000	33.26900	-21.68500
H	-5.97300	34.13700	-21.31800
H	-6.33100	33.43200	-22.60900
O	-3.38300	41.73100	-10.01900
H	-3.68300	42.63300	-10.12700
H	-3.72800	41.46400	-9.16700
O	-9.93200	38.18200	-13.98500
H	-10.85700	38.05000	-13.77700
H	-9.68300	38.96100	-13.48800
O	0.01700	34.24900	-12.33900
H	-0.89200	34.18000	-12.63300
H	-0.03400	34.72600	-11.51100
O	-6.16200	35.72500	-20.70100
H	-6.28200	36.17000	-19.86200
H	-6.85600	36.07300	-21.25900
O	-12.57100	37.82500	-13.15100
H	-12.44200	38.07900	-12.23700
H	-13.25100	38.41800	-13.47000
O	-5.90900	35.66800	-4.39600
H	-6.68600	36.22700	-4.40500
H	-5.27000	36.15300	-3.87400
O	1.88300	32.45000	-10.56100
H	1.24000	32.74400	-11.20700
H	1.37800	32.33600	-9.75600
O	-0.46300	36.19700	-19.87300
H	0.44900	36.42900	-20.04800
H	-0.97200	36.78000	-20.43500
O	-7.65400	38.47600	-4.83400
H	-6.91300	39.01500	-5.11000
H	-8.36100	39.10300	-4.67700
O	-2.17900	40.61900	-6.23600
H	-1.77100	40.17700	-6.98100
H	-2.90100	40.04400	-5.98300
O	-5.82600	35.55600	-8.86300
H	-4.98000	35.98000	-8.72000
H	-5.88600	34.90000	-8.16900
O	-1.85600	41.17900	-12.48100
H	-2.48500	41.34400	-13.18400
H	-2.24300	41.59300	-11.71000
O	2.02300	42.11500	-15.12700
H	1.33800	42.49500	-15.67700
H	2.10700	42.72400	-14.39400
O	-2.67600	33.21700	-7.20500
H	-2.92000	33.97100	-7.74200
H	-2.40000	33.59500	-6.37000
O	-0.72200	39.76100	-8.52700
H	-0.51000	40.40400	-9.20400
H	-0.76900	38.92700	-8.99500
O	-3.11900	32.37300	-11.62400
H	-3.98100	32.67100	-11.33200
H	-3.09300	32.59000	-12.55600
O	-6.30300	40.91700	-15.21600
H	-5.83900	41.19600	-16.00600
H	-6.56800	40.01500	-15.39900
O	-9.41100	40.46400	-12.27800
H	-8.84100	40.89300	-12.91700
H	-8.82000	40.18200	-11.58000
O	-7.36600	37.03900	-18.40700
H	-6.66400	37.49100	-17.94000
H	-7.69800	36.39700	-17.77900
O	-8.91800	35.63000	-4.03200
H	-9.54800	36.31200	-3.79900
H	-9.02500	35.51900	-4.97700
O	-13.62400	35.29700	-13.10600
H	-14.41400	35.55300	-13.58200
H	-13.13300	36.11200	-13.00000
O	0.39300	42.63800	-10.35500
H	1.21400	42.23600	-10.63700
H	0.38900	42.53500	-9.40300
O	-11.67200	35.66200	-16.14700
H	-12.46900	35.77400	-15.62900
H	-11.90000	36.00100	-17.01300
O	-5.84200	42.18600	-8.19900
H	-5.77700	42.64800	-7.36300
H	-6.55100	41.55600	-8.07100
O	-15.34000	35.55000	-10.67400
H	-15.08900	36.43500	-10.40800
H	-14.80400	35.37100	-11.44700
O	8.48300	38.98200	-14.00700
H	7.73000	38.41300	-14.16200

H	8.61800	38.94800	-13.05900
O	-9.64000	29.93200	-1.67000
H	-10.03400	29.37900	-0.99500
H	-8.82400	30.24300	-1.27900
O	-6.72300	31.34800	-4.07800
H	-5.98300	31.94900	-4.15700
H	-6.49200	30.60600	-4.63600
O	-11.57900	36.53400	-5.80000
H	-11.69400	35.63200	-5.49900
H	-12.43500	36.78000	-6.15100
O	-5.94500	42.27000	-5.33100
H	-5.41200	41.50900	-5.56100
H	-6.47300	41.97800	-4.58800
O	-2.77800	27.37600	-9.80100
H	-3.12100	28.26600	-9.87700
H	-2.41000	27.18600	-10.66400
O	-14.70900	39.70000	-16.98200
H	-13.87200	40.10900	-17.19900
H	-14.53100	38.76000	-17.01200
O	-2.81300	37.36700	-21.29400
H	-3.32800	38.13800	-21.05500
H	-3.08500	36.69400	-20.67100
O	-5.22800	32.21200	-16.23500
H	-6.08400	31.82300	-16.41500
H	-4.81500	32.29100	-17.09500
O	-3.69800	29.74700	-11.83600
H	-4.20200	29.71000	-12.64900
H	-3.40500	30.65600	-11.77700
O	-7.45400	29.83100	-12.83000
H	-6.58900	29.63500	-13.18900
H	-7.38600	29.60200	-11.90300
O	2.16100	40.12000	-19.35900
H	1.38400	39.78100	-19.80300
H	2.84000	40.12600	-20.03300
O	-3.02400	35.24000	-19.46800
H	-3.32900	35.55400	-18.61700
H	-2.07100	35.31800	-19.42500
O	-4.57900	41.39900	-17.81900
H	-5.10700	40.66400	-18.13100
H	-5.08100	42.17600	-18.06500
O	0.40000	39.59400	-12.70200
H	-0.25800	40.28400	-12.61400
H	1.20400	39.98300	-12.35900
O	-9.84800	32.06100	-15.45800
H	-9.27300	31.83800	-16.19100
H	-9.26000	32.13000	-14.70500
O	-0.79000	37.94800	-14.65200
H	-0.27600	38.58100	-14.14900
H	-0.14000	37.34800	-15.01700
O	-9.52100	34.09100	-21.43800
H	-10.35700	34.42900	-21.11800
H	-9.66000	33.95600	-22.37600
O	-7.54500	28.93500	-16.73100
H	-8.19900	28.62000	-17.35500
H	-7.58700	28.31300	-16.00500
O	-6.43400	39.21700	-24.81500
H	-7.34100	38.98600	-24.61600
H	-5.93700	38.42100	-24.62800
O	-4.31200	35.42300	-12.40600
H	-5.22900	35.64500	-12.56400
H	-3.89600	35.49800	-13.26500
O	-3.52800	36.82200	-17.27500
H	-2.78100	37.33400	-17.58500
H	-3.25900	36.50100	-16.41500
O	-5.25500	39.31800	-20.29300
H	-6.16400	39.03400	-20.19600
H	-5.22600	39.73800	-21.15200
O	-0.02300	42.92900	-16.52400
H	-0.47700	42.08800	-16.46700
H	-0.65400	43.56500	-16.18600
O	-1.08000	33.85800	-15.29400
H	-1.81800	33.34600	-14.96300
H	-0.82700	33.41800	-16.10500
O	-0.97900	26.63600	-14.69900
H	-0.67300	25.77500	-14.98700
H	-0.25400	27.22700	-14.90000
O	-14.68700	37.03900	-16.31100
H	-14.88400	36.44600	-17.03600
H	-15.24200	36.73400	-15.59400
O	-6.70700	36.93100	-14.19200
H	-6.65200	35.97800	-14.26800
H	-7.51800	37.08700	-13.70800
O	-5.55800	40.85900	-22.95400
H	-5.99600	40.14300	-23.41300
H	-4.81700	41.08700	-23.51500
O	-8.76800	35.54900	-16.43300
H	-9.58100	35.84800	-16.02600
H	-8.27200	35.15400	-15.71500
O	-3.17000	36.01300	-7.60100
H	-2.81500	36.24000	-6.74200
H	-2.52300	36.34500	-8.22400
O	2.56000	39.13500	-16.89600
H	2.42100	39.32500	-17.82400
H	2.20300	39.89700	-16.44000
O	-9.27600	33.12100	-10.81700
H	-8.63600	32.97900	-11.51400
H	-10.10800	32.83400	-11.19300
O	2.58600	41.20200	-11.88500
H	3.02000	41.11000	-11.03700
H	3.30000	41.17800	-12.52300
O	-8.50000	42.42100	-14.70300
H	-7.70600	41.92600	-14.90500
H	-9.21100	41.89300	-15.06600
O	-12.19200	36.56500	-18.62700
H	-13.12200	36.50400	-18.41000
H	-12.09600	36.04300	-19.42400
O	-12.27300	33.09100	-16.03900
H	-12.01300	34.00500	-16.14800
H	-11.45200	32.62300	-15.89000
O	-10.80100	34.50400	-8.86900
H	-10.12900	34.29600	-9.51800
H	-11.62800	34.30700	-9.30900
O	3.44800	41.24500	-9.35200
H	3.64200	42.14900	-9.10600
H	3.34800	40.78500	-8.51800
O	-4.89800	31.13100	-6.85300
H	-5.54600	31.83000	-6.94600
H	-4.12800	31.56400	-6.48500

O	-1.49000	41.39500	-3.77800
H	-1.64200	41.16600	-4.69500
H	-1.50000	42.35200	-3.76500
O	-4.72200	38.96900	-8.79300
H	-3.79300	39.12000	-8.96600
H	-5.14300	39.05700	-9.64800
O	-4.37300	42.37200	-13.63400
H	-4.70800	42.33200	-12.73900
H	-4.87200	41.70600	-14.10700
O	-10.39800	37.77900	-8.10900
H	-10.53600	37.04700	-7.50800
H	-9.48800	37.68900	-8.39100
O	-1.43700	39.71700	-23.36100
H	-2.05400	38.99200	-23.45700
H	-1.90500	40.47800	-23.70400
O	-7.77000	37.49100	-8.62100
H	-7.06000	36.85000	-8.66200
H	-7.81300	37.74200	-7.69800
O	0.21900	44.42700	-5.70000
H	0.16000	43.74300	-6.36700
H	0.62100	45.16900	-6.15200
O	-9.05700	34.47700	-6.80100
H	-9.59300	34.02600	-6.14900
H	-9.64500	34.61200	-7.54400
O	-9.64700	33.93600	-24.23900
H	-10.52400	33.81500	-24.60200
H	-9.17100	33.14500	-24.49100
O	0.22100	42.39100	-7.46900
H	-0.66700	42.05400	-7.34600
H	0.79000	41.66000	-7.23200
O	-13.32300	33.61300	-9.71600
H	-13.40700	33.00500	-10.45100
H	-14.14500	34.10400	-9.71500
O	-3.15100	32.09800	-14.34900
H	-2.65400	31.34500	-14.66900
H	-3.92200	32.13400	-14.91500
O	4.70200	37.80200	-12.70700
H	4.69100	38.68500	-13.07800
H	3.83400	37.44900	-12.90200
O	-2.06800	40.85500	-16.77500
H	-3.00200	40.97500	-16.94500
H	-1.89000	39.95200	-17.03900
O	-6.56700	34.24000	-14.62200
H	-6.93700	33.68700	-13.93400
H	-6.07100	33.63600	-15.17400
O	1.32000	36.69700	-16.24000
H	1.78600	37.53000	-16.31400
H	1.77000	36.23100	-15.53600
O	-0.61300	43.04100	-19.24200
H	-0.33700	42.92700	-18.33200
H	-1.48200	42.64300	-19.28000
O	-0.70900	29.06500	-12.20100
H	-1.62300	29.29500	-12.03300
H	-0.28000	29.14900	-11.35000
O	-0.93800	37.25400	-9.70100
H	-1.59100	37.50200	-10.35600
H	-0.56000	36.44000	-10.03500
O	-4.88900	37.99600	-3.21400
H	-5.56500	37.89500	-2.54400
H	-4.06700	37.84000	-2.75100
O	-18.55000	36.80400	-11.87300
H	-18.51100	36.27000	-11.07900
H	-19.05600	36.27700	-12.49200
O	-11.17100	31.65000	-8.37600
H	-11.10700	32.51200	-8.78900
H	-11.23400	31.03800	-9.10900
O	-11.69500	33.72300	-4.72900
H	-11.06800	33.34000	-4.11500
H	-12.55300	33.50400	-4.36400
O	-1.29600	44.12900	-3.12400
H	-0.75200	44.74200	-3.61800
H	-1.87700	44.68700	-2.60700
O	-14.91900	35.53000	-18.79700
H	-14.62400	34.62100	-18.85100
H	-15.82000	35.50900	-19.11900
O	-11.38900	33.60600	-12.81500
H	-12.11100	34.21900	-12.95400
H	-10.60000	34.14200	-12.89300
O	-8.58700	30.94100	-6.54100
H	-8.06300	31.18700	-5.77800
H	-8.80600	31.77300	-6.96000
O	-13.19000	33.21200	-18.67300
H	-13.51900	32.33100	-18.85600
H	-12.83600	33.15600	-17.78600
O	-10.07600	33.49900	-18.30600
H	-9.73600	34.02700	-19.02800
H	-9.62900	33.83700	-17.53000
O	-7.38200	31.88300	-8.85600
H	-7.72200	31.08300	-8.45300
H	-8.08800	32.18300	-9.42800
O	-6.73000	28.96800	-10.40300
H	-5.90700	28.49400	-10.52500
H	-6.48600	29.74700	-9.90300
O	-8.08900	31.99600	-20.10700
H	-7.36800	32.37400	-20.61000
H	-8.71300	32.71500	-20.00300
O	-3.94600	32.70100	-19.17100
H	-3.73000	33.58300	-19.47200
H	-4.42700	32.30900	-19.90000
O	-9.20600	35.65900	-12.59400
H	-9.35200	35.97300	-11.70100
H	-9.68400	36.27800	-13.14700
O	-7.34600	32.65000	-12.54600
H	-7.36000	31.75400	-12.88200
H	-6.62900	32.65700	-11.91200
O	-5.49400	33.08300	-10.50000
H	-5.75200	34.00500	-10.50200
H	-6.02500	32.68600	-9.81000
O	-14.73200	38.20000	-9.65500
H	-15.27600	38.97600	-9.52300
H	-13.85400	38.54700	-9.81300
O	-7.94300	31.53100	-17.27700
H	-7.82100	30.60300	-17.07800
H	-8.23300	31.54500	-18.18900
O	-5.07400	29.21700	-14.14000
H	-5.05100	29.28100	-15.09500
H	-4.65700	28.37700	-13.94600
O	5.30500	38.95200	-16.10200

H	5.74800	38.20300	-16.50000
H	4.42700	38.94300	-16.48500
O	-13.94500	37.32700	-6.76200
H	-14.68700	37.47500	-6.17500
H	-14.26600	37.58800	-7.62500
O	2.33700	42.95500	-19.09300
H	2.86300	43.29400	-19.81800
H	2.25000	42.02000	-19.27600
O	-8.45900	33.04400	-3.06800
H	-8.41000	33.92100	-3.45000
H	-7.91600	32.50200	-3.64100
O	2.02700	43.58800	-3.78800
H	1.23600	43.57100	-4.32700
H	1.79000	44.12700	-3.03300
O	2.14700	40.43800	-6.47400
H	2.19600	40.27300	-5.53300
H	2.03800	39.57000	-6.86500
O	-0.56300	36.42100	-5.15800
H	0.33800	36.28100	-5.45000
H	-0.48800	36.58600	-4.21900
O	-6.22900	33.79700	-6.31300
H	-7.09800	34.09100	-6.58500
H	-5.99300	34.38100	-5.59200
O	3.64800	42.85700	-6.23000
H	3.35700	43.22000	-5.39400
H	3.03600	42.14400	-6.40900
O	4.84800	40.42300	-13.71000
H	5.68300	40.86200	-13.54600
H	4.98400	39.94700	-14.52900
O	1.89700	37.98100	-7.76200
H	2.37300	38.17300	-8.57000
H	0.97400	38.03700	-8.00800
O	-11.21500	39.14300	-18.17900
H	-11.46000	38.23900	-17.98100
H	-11.04500	39.14500	-19.12100
O	-3.52500	30.01500	-8.79700
H	-3.00700	30.75200	-9.12200
H	-3.87900	30.32200	-7.96200
O	-11.94500	34.87600	-20.68000
H	-12.63800	34.95000	-21.33600
H	-12.19000	34.11200	-20.15800
O	-3.27400	26.43500	-13.27700
H	-2.99300	25.76800	-12.65000
H	-2.48900	26.62800	-13.78900
O	0.59700	34.85700	-9.52900
H	1.54900	34.95700	-9.50800
H	0.42800	34.06500	-9.02000
O	-1.38100	31.94300	-9.34300
H	-1.97600	32.22300	-10.03900
H	-1.66600	32.43200	-8.57200
O	0.37800	45.21200	-23.62300
H	0.30000	44.87800	-22.72900
H	1.19900	45.70400	-23.62400
O	3.07600	38.07800	-10.41500
H	3.04400	37.13900	-10.22900
H	3.68600	38.15600	-11.14900
O	-5.08600	36.94700	-24.37200
H	-4.14000	36.81800	-24.30400
H	-5.46400	36.20900	-23.89500
O	3.22600	34.86900	-10.36900
H	2.70900	34.08500	-10.55600
H	4.11000	34.54100	-10.20500
O	0.88700	28.61300	-14.41000
H	0.68900	28.30500	-13.52500
H	1.49700	29.33900	-14.28000
O	8.89400	39.13900	-11.27500
H	9.62400	38.87800	-10.71400
H	8.28400	38.40200	-11.23400
O	6.97500	37.28000	-10.67700
H	6.24900	37.14600	-11.28500
H	6.56000	37.58800	-9.87100
O	-5.20200	40.99900	-2.91200
H	-4.28100	41.17700	-2.71800
H	-5.23700	40.05500	-3.06400
O	-6.90100	34.87200	-24.43700
H	-7.80800	34.57600	-24.36100
H	-6.62400	34.57500	-25.30400
O	-15.98300	36.19300	-13.98700
H	-16.30300	35.34000	-14.28000
H	-16.53000	36.40800	-13.23100
O	-12.20500	38.90900	-10.27000
H	-11.98700	39.84100	-10.25100
H	-11.79300	38.55000	-9.48500
O	-5.28500	29.41600	-18.28300
H	-6.09500	29.07100	-17.90700
H	-4.59700	29.10700	-17.69400
O	-3.35600	28.49400	-16.66000
H	-2.80300	28.91300	-16.00100
H	-3.11900	27.56700	-16.61800
O	-7.33300	34.48300	-0.88000
H	-7.43300	35.43200	-0.94900
H	-7.55100	34.15400	-1.75200
O	-8.13100	31.73100	-24.29600
H	-7.26900	32.05700	-24.03600
H	-7.94600	30.94400	-24.80800
O	0.02200	30.60400	-17.15200
H	-0.55800	31.26200	-17.53400
H	-0.24500	29.78200	-17.56300
O	-1.52800	30.00400	-14.95000
H	-1.02800	30.11600	-15.75900
H	-0.88400	29.70100	-14.31000
O	-7.47000	37.91200	-2.06300
H	-7.95300	38.71200	-1.85700
H	-7.38300	37.92300	-3.01700
O	-10.95200	40.37300	-7.45900
H	-10.13300	40.86700	-7.43600
H	-10.67900	39.45600	-7.48800
O	-1.19600	32.76400	-18.09700
H	-2.08300	33.04900	-18.31500
H	-0.78000	32.60300	-18.94400
O	2.15600	32.78900	-14.38100
H	2.93200	33.27800	-14.10900
H	1.43100	33.21400	-13.92400
O	4.59300	33.50100	-13.32400
H	5.18800	33.26100	-14.03400
H	4.66600	32.78200	-12.69600
O	-2.05100	42.52800	-24.00200
H	-1.34800	42.73300	-23.38600

H	-2.77500	43.09600	-23.73700
O	-1.22200	38.27900	-17.88200
H	-1.27800	38.73600	-18.72100
H	-0.66900	37.51800	-18.05900
O	2.58900	34.06800	-17.05700
H	2.33300	33.66800	-16.22600
H	1.96400	34.78300	-17.18200
O	6.90000	36.80800	-14.54900
H	6.34000	36.87700	-13.77600
H	6.29500	36.63200	-15.26900
O	-0.14300	42.96700	-21.95200
H	0.69200	42.53500	-22.13400
H	-0.30400	42.79500	-21.02400
O	-0.48800	39.47000	-20.58600
H	-0.54300	39.30400	-21.52700
H	-1.11300	40.17800	-20.43000
O	-15.69900	34.70700	-6.76000
H	-15.05300	34.19700	-6.27100
H	-15.90700	35.44400	-6.18600
O	-3.17300	41.77500	-1.26100
H	-2.30400	41.94900	-1.62200
H	-3.65300	42.59400	-1.38300
O	-7.17800	28.97100	-21.00800
H	-7.19100	29.91500	-20.85100
H	-6.28600	28.78800	-21.30200
O	-3.36300	25.56500	-16.75300
H	-4.13000	25.01100	-16.60800
H	-2.95200	25.63700	-15.89200
O	-11.35900	31.85300	-2.66300
H	-10.49600	31.49700	-2.45200
H	-11.93300	31.51500	-1.97500
O	10.96300	44.85800	-9.41800
H	10.63200	45.72800	-9.64200
H	10.36800	44.54200	-8.73900
O	12.72200	47.78000	-10.56100
H	12.08000	48.07300	-11.20700
H	12.21800	47.66500	-9.75600
O	1.19900	45.26200	-1.67000
H	0.80600	44.70800	-0.99500
H	2.01600	45.57300	-1.27900
O	8.06200	42.70500	-9.80100
H	7.71800	43.59600	-9.87700
H	8.43000	42.51600	-10.66400
O	7.14100	45.07600	-11.83600
H	6.63800	45.03900	-12.64900
H	7.43400	45.98500	-11.77700
O	3.38600	45.16000	-12.83000
H	4.25100	44.96400	-13.18900
H	3.45300	44.93100	-11.90300
O	3.29400	44.26400	-16.73100
H	2.64100	43.94900	-17.35500
H	3.25200	43.64200	-16.00500
O	9.86000	41.96500	-14.69900
H	10.16600	41.10500	-14.98700
H	10.58600	42.55700	-14.90000
O	5.94100	46.46000	-6.85300
H	5.29400	47.15900	-6.94600
H	6.71100	46.89300	-6.48500
O	7.68900	47.42700	-14.34900
H	8.18600	46.67400	-14.66900
H	6.91700	47.46400	-14.91500
O	10.13100	44.39400	-12.20100
H	9.21700	44.62500	-12.03300
H	10.56000	44.47800	-11.35000
O	4.10900	44.29700	-10.40300
H	4.93200	43.82300	-10.52500
H	4.35300	45.07600	-9.90300
O	5.76600	44.54600	-14.14000
H	5.78800	44.61000	-15.09500
H	6.18300	43.70600	-13.94600
O	7.31500	45.34500	-8.79700
H	7.83300	46.08100	-9.12200
H	6.96000	45.65100	-7.96200
O	7.56600	41.76500	-13.27700
H	7.84600	41.09700	-12.65000
H	8.35100	41.95800	-13.78900
O	9.45800	47.27200	-9.34300
H	8.86300	47.55200	-10.03900
H	9.17300	47.76100	-8.57200
O	14.06500	50.19800	-10.36900
H	13.54900	49.41400	-10.55600
H	14.94900	49.87000	-10.20500
O	11.72600	43.94200	-14.41000
H	11.52800	43.63400	-13.52500
H	12.33600	44.66800	-14.28000
O	5.55400	44.74500	-18.28300
H	4.74400	44.40000	-17.90700
H	6.24300	44.43600	-17.69400
O	7.48300	43.82300	-16.66000
H	8.03600	44.24300	-16.00100
H	7.72000	42.89700	-16.61800
O	10.86100	45.93300	-17.15200
H	10.28100	46.59200	-17.53400
H	10.59500	45.11100	-17.56300
O	9.31200	45.33400	-14.95000
H	9.81200	45.44500	-15.75900
H	9.95500	45.03000	-14.31000
O	9.64400	48.09300	-18.09700
H	8.75600	48.37800	-18.31500
H	10.05900	47.93300	-18.94400
O	12.99500	48.11800	-14.38100
H	13.77100	48.60700	-14.10900
H	12.27000	48.54400	-13.92400
O	15.43200	48.83100	-13.32400
H	16.02700	48.59000	-14.03400
H	15.50500	48.11100	-12.69600
O	3.66100	44.30000	-21.00800
H	3.64900	45.24400	-20.85100
H	4.55300	44.11700	-21.30200
O	7.47600	40.89400	-16.75300
H	6.70900	40.34000	-16.60800
H	7.88700	40.96600	-15.89200
O	-40.86000	-26.93700	-20.28900
H	-41.15100	-26.03200	-20.40500
H	-40.22400	-26.89500	-19.57500
O	-40.17300	-30.50500	-18.10900
H	-39.43100	-29.96700	-18.38600
H	-40.87900	-29.87800	-17.95200

O	-34.69700	-28.36200	-19.51200
H	-34.28900	-28.80400	-20.25700
H	-35.41900	-28.93700	-19.25900
O	-32.12600	-26.34300	-23.63100
H	-31.30400	-26.74500	-23.91200
H	-32.12900	-26.44700	-22.67900
O	-37.01800	-24.50600	-17.47700
H	-37.46200	-25.29200	-17.79400
H	-36.35700	-24.31800	-18.14200
O	-34.92000	-24.22600	-19.44800
H	-34.93800	-23.40500	-19.94000
H	-33.99800	-24.35000	-19.22300
O	-38.46300	-26.71100	-18.60600
H	-37.93000	-27.47200	-18.83600
H	-38.99100	-27.00400	-17.86400
O	-29.07000	-27.73700	-22.62700
H	-28.87600	-26.83200	-22.38100
H	-29.17000	-28.19600	-21.79400
O	-34.00900	-27.58600	-17.05300
H	-34.16000	-27.81500	-17.97000
H	-34.01800	-26.62900	-17.04000
O	-32.29900	-24.55400	-18.97600
H	-32.35800	-25.23800	-19.64300
H	-31.89700	-23.81200	-19.42800
O	-32.29800	-26.59000	-20.74400
H	-33.18500	-26.92700	-20.62200
H	-31.72800	-27.32200	-20.50700
O	-36.30800	-22.03100	-21.62200
H	-35.59500	-21.39700	-21.69500
H	-36.79900	-21.93500	-22.43800
O	-41.25600	-24.28200	-20.80200
H	-40.55500	-23.64800	-20.64900
H	-41.72700	-23.94000	-21.56200
O	-37.40800	-30.98500	-16.49000
H	-38.08300	-31.08600	-15.82000
H	-36.58500	-31.14100	-16.02600
O	-38.66500	-19.67900	-22.25400
H	-38.11300	-19.51600	-21.48900
H	-38.32500	-19.08800	-22.92600
O	-33.81400	-24.85300	-16.39900
H	-33.27000	-24.24000	-16.89400
H	-34.39600	-24.29500	-15.88300
O	-33.46500	-23.93000	-23.64000
H	-34.41200	-24.04900	-23.71200
H	-33.11700	-24.81700	-23.55700
O	-38.51900	-22.11300	-20.23900
H	-38.21000	-22.35800	-19.36700
H	-37.72100	-21.90800	-20.72700
O	-30.49100	-25.39300	-17.06300
H	-31.28200	-25.41100	-17.60300
H	-30.72800	-24.85400	-16.30800
O	-30.37200	-28.54400	-19.75000
H	-30.32200	-28.70900	-18.80800
H	-30.48000	-29.41100	-20.14000
O	-33.08100	-32.56100	-18.43400
H	-32.18000	-32.70000	-18.72600
H	-33.00600	-32.39500	-17.49400
O	-28.87000	-26.12400	-19.50600
H	-29.16100	-25.76100	-18.66900
H	-29.48200	-26.83700	-19.68400
O	-30.62100	-31.00100	-21.03800
H	-30.14600	-30.80800	-21.84600
H	-31.54400	-30.94500	-21.28300
O	-23.62400	-29.84200	-24.55000
H	-22.89400	-30.10300	-23.98900
H	-24.23400	-30.57900	-24.51000
O	-37.72000	-27.98200	-16.18800
H	-36.80000	-27.80400	-15.99400
H	-37.75500	-28.92700	-16.34000
O	-39.98800	-31.06900	-15.33900
H	-40.47100	-30.26900	-15.13200
H	-39.90200	-31.05800	-16.29200
O	-35.69200	-27.20700	-14.53600
H	-34.82200	-27.03300	-14.89800
H	-36.17100	-26.38700	-14.65900
O	-41.52300	-20.08700	-22.85700
H	-40.62000	-20.05500	-22.54100
H	-41.74900	-21.01700	-22.84300
O	-24.03200	-15.49800	-25.13200
H	-23.39400	-15.61700	-25.83600
H	-24.15000	-14.55000	-25.07300
O	-27.22300	-15.54500	-25.19600
H	-27.56200	-15.66100	-26.08400
H	-26.35400	-15.94600	-25.21600
O	-31.36500	-13.69100	-29.20600
H	-31.92800	-13.78200	-29.97400
H	-31.47900	-14.51100	-28.72600
O	-26.74100	-14.39100	-19.23700
H	-26.75300	-14.78600	-20.10900
H	-26.57900	-15.12500	-18.64400
O	-33.81800	-12.19600	-26.36800
H	-33.02600	-12.53700	-26.78300
H	-33.57900	-12.08700	-25.44700
O	-25.78500	-10.02400	-33.89500
H	-26.43600	-9.43900	-34.28100
H	-24.99000	-9.49400	-33.83500
O	-30.02100	-11.60800	-20.28900
H	-30.31200	-10.70300	-20.40500
H	-29.38500	-11.56600	-19.57500
O	-29.09500	-14.23000	-23.69900
H	-28.49900	-14.45400	-24.41300
H	-29.03800	-14.97200	-23.09700
O	-24.47800	-17.77300	-28.05700
H	-23.69200	-18.19100	-28.40800
H	-24.21400	-16.86800	-27.88800
O	-24.30700	-12.33500	-33.35800
H	-24.94300	-12.26200	-32.64600
H	-24.63700	-11.74600	-34.03600
O	-19.07700	-17.83500	-27.15300
H	-18.34500	-18.40500	-26.91900
H	-19.83300	-18.22300	-26.71300
O	-27.46000	-15.41200	-29.95700
H	-27.82400	-15.93900	-29.24600
H	-26.60300	-15.80200	-30.13000
O	-21.55500	-24.12300	-22.69400
H	-21.88600	-23.25300	-22.91800
H	-22.15000	-24.43900	-22.01400
O	-25.06200	-11.92200	-23.29400

```
H        -25.36200      -11.01900      -23.40300
H        -25.40700      -12.18800      -22.44200
O        -31.61000      -15.47000      -27.26100
H        -32.53500      -15.60300      -27.05300
H        -31.36200      -14.69100      -26.76400
O        -21.66200      -19.40300      -25.61400
H        -22.57000      -19.47200      -25.90900
H        -21.71300      -18.92600      -24.78600
O        -34.25000      -15.82700      -26.42600
H        -34.12000      -15.57300      -25.51200
H        -34.93000      -15.23400      -26.74600
O        -30.08000       -5.15700      -30.32700
H        -30.15700       -4.20600      -30.24800
H        -30.68300       -5.50300      -29.67000
O        -27.58800      -17.98400      -17.67100
H        -28.36500      -17.42500      -17.68100
H        -26.94900      -17.49900      -17.14900
O        -19.79600      -21.20200      -23.83700
H        -20.43800      -20.90800      -24.48300
H        -20.30000      -21.31600      -23.03100
O        -29.33300      -15.17600      -18.10900
H        -28.59200      -14.63700      -18.38600
H        -30.04000      -14.54900      -17.95200
O        -23.85800      -13.03300      -19.51200
H        -23.44900      -13.47500      -20.25700
H        -24.58000      -13.60800      -19.25900
O        -27.50500      -18.09600      -22.13900
H        -26.65800      -17.67200      -21.99500
H        -27.56500      -18.75200      -21.44500
O        -23.53500      -12.47300      -25.75700
H        -24.16400      -12.30800      -26.45900
H        -23.92200      -12.05900      -24.98600
O        -19.65600      -11.53700      -28.40200
H        -20.34100      -11.15700      -28.95300
H        -19.57200      -10.92800      -27.66900
O        -24.35500      -20.43500      -20.48000
H        -24.59900      -19.68100      -21.01700
H        -24.07900      -20.05700      -19.64600
O        -22.40100      -13.89100      -21.80300
H        -22.18900      -13.24800      -22.47900
H        -22.44800      -14.72500      -22.27100
O        -24.79800      -21.28000      -24.89900
H        -25.66000      -20.98100      -24.60700
H        -24.77200      -21.06300      -25.83100
O        -27.98200      -12.73500      -28.49200
H        -27.51800      -12.45700      -29.28100
H        -28.24600      -13.63700      -28.67400
O        -31.09000      -13.18800      -25.55400
H        -30.51900      -12.76000      -26.19200
H        -30.49900      -13.47000      -24.85500
O        -30.59700      -18.02200      -17.30800
H        -31.22700      -17.34000      -17.07400
H        -30.70300      -18.13400      -18.25200
O        -35.30300      -18.35500      -26.38100
H        -36.09300      -18.09900      -26.85700
H        -34.81100      -17.54000      -26.27600
O        -21.28600      -11.01400      -23.63100
H        -20.46400      -11.41600      -23.91200
H        -21.29000      -11.11700      -22.67900
O        -27.52100      -11.46600      -21.47400
H        -27.45600      -11.00400      -20.63900
H        -28.22900      -12.09600      -21.34600
O        -37.01900      -18.10200      -23.95000
H        -36.76800      -17.21700      -23.68400
H        -36.48300      -18.28100      -24.72200
O        -13.19600      -14.67000      -27.28200
H        -13.94900      -15.24000      -27.43800
H        -13.06100      -14.70400      -26.33500
O        -27.80600       -9.79900      -31.60700
H        -27.94100       -8.95400      -31.17800
H        -27.26500       -9.59700      -32.37000
O        -31.31900      -23.72000      -14.94500
H        -31.71200      -24.27300      -14.27000
H        -30.50200      -23.40900      -14.55400
O        -34.35300      -12.28100      -31.03400
H        -33.84000      -11.48700      -30.88300
H        -33.70100      -12.97900      -31.10000
O        -32.54300       -9.16500      -28.95100
H        -32.36700       -8.24300      -29.13900
H        -31.74800       -9.47900      -28.52200
O        -28.40200      -22.30400      -17.35300
H        -27.66100      -21.70300      -17.43300
H        -28.17000      -23.04600      -17.91100
O        -33.25800      -17.11800      -19.07500
H        -33.37300      -18.02000      -18.77500
H        -34.11400      -16.87200      -19.42700
O        -26.17900       -9.17700      -17.47700
H        -26.62300       -9.96300      -17.79400
H        -25.51700       -8.98900      -18.14200
O        -31.42000       -7.64700      -22.95000
H        -30.86900       -7.77500      -23.72300
H        -32.25900       -7.34800      -23.30100
O        -24.08100       -8.89600      -19.44800
H        -24.09900       -8.07500      -19.94000
H        -23.15900       -9.02100      -19.22300
O        -27.62300      -11.38200      -18.60600
H        -27.09100      -12.14300      -18.83600
H        -28.15200      -11.67500      -17.86400
O        -24.45700      -26.27600      -23.07700
H        -24.80000      -25.38600      -23.15300
H        -24.08900      -26.46600      -23.94000
O        -25.37700      -23.90500      -25.11200
H        -25.88000      -23.94200      -25.92500
H        -25.08400      -22.99600      -25.05200
O        -29.13300      -23.82200      -26.10500
H        -28.26700      -24.01700      -26.46500
H        -29.06500      -24.05100      -25.17800
O        -19.51800      -13.53200      -32.63400
H        -20.29500      -13.87100      -33.07900
H        -18.83900      -13.52700      -33.30900
O        -26.25800      -12.25300      -31.09400
H        -26.78600      -12.98800      -31.40700
H        -26.75900      -11.47600      -31.34000
O        -21.27900      -14.05800      -25.97700
H        -21.93700      -13.36900      -25.88900
H        -20.47500      -13.66900      -25.63400
O        -22.46800      -15.70400      -27.92700
H        -21.95500      -15.07100      -27.42500
```

H	-21.81800	-16.30400	-28.29300
O	-25.20600	-5.72600	-35.17100
H	-25.88000	-5.42200	-34.56200
H	-24.82000	-4.92300	-35.52000
O	-25.99000	-18.22900	-25.68100
H	-26.90800	-18.00800	-25.83900
H	-25.57400	-18.15400	-26.54000
O	-25.20700	-16.83000	-30.55100
H	-24.46000	-16.31800	-30.86000
H	-24.93800	-17.15200	-29.69100
O	-21.70200	-10.72300	-29.79900
H	-22.15600	-11.56400	-29.74300
H	-22.33300	-10.08700	-29.46200
O	-22.75900	-19.79400	-28.56900
H	-23.49700	-20.30600	-28.23800
H	-22.50600	-20.23400	-29.38100
O	-23.25900	-8.88600	-28.86400
H	-22.96700	-8.32500	-29.58300
H	-22.74900	-8.59300	-28.10900
O	-28.38600	-16.72100	-27.46700
H	-28.33100	-17.67400	-27.54300
H	-29.19700	-16.56500	-26.98300
O	-28.29600	-8.10800	-27.77500
H	-28.33800	-7.88200	-28.70400
H	-27.37900	-8.34100	-27.62600
O	-24.84900	-17.63900	-20.87700
H	-24.49400	-17.41200	-20.01700
H	-24.20200	-17.30700	-21.49900
O	-19.11900	-14.51700	-30.17200
H	-19.25700	-14.32700	-31.10000
H	-19.47600	-13.75600	-29.71500
O	-30.95500	-20.53200	-24.09200
H	-30.31500	-20.67300	-24.79000
H	-31.78700	-20.81800	-24.46800
O	-19.09200	-12.45000	-25.16100
H	-18.65900	-12.54200	-24.31200
H	-18.37900	-12.47400	-25.79900
O	-30.17900	-11.23100	-27.97900
H	-29.38500	-11.72600	-28.18000
H	-30.89000	-11.76000	-28.34200
O	-25.35900	-8.85000	-23.62700
H	-25.30600	-8.28100	-22.86000
H	-26.22800	-8.67900	-23.99200
O	-32.48000	-19.14800	-22.14500
H	-31.80700	-19.35600	-22.79400
H	-33.30700	-19.34600	-22.58400
O	-18.23100	-12.40700	-22.62700
H	-18.03700	-11.50300	-22.38100
H	-18.33100	-12.86700	-21.79400
O	-26.57700	-22.52200	-20.12800
H	-27.22400	-21.82300	-20.22200
H	-25.80700	-22.08800	-19.76100
O	-23.16900	-12.25700	-17.05300
H	-23.32100	-12.48600	-17.97000
H	-23.17900	-11.30000	-17.04000
O	-26.40100	-14.68400	-22.06900
H	-25.47100	-14.53200	-22.24200
H	-26.82200	-14.59500	-22.92400
O	-27.60200	-10.65900	-24.29900
H	-27.74100	-11.02700	-23.42600
H	-28.39000	-10.14400	-24.47000
O	-27.00700	-5.55800	-32.95600
H	-26.83200	-6.02900	-32.14200
H	-27.69400	-4.93100	-32.72800
O	-26.05100	-11.28000	-26.91000
H	-26.38600	-11.32000	-26.01400
H	-26.55100	-11.94600	-27.38300
O	-32.07700	-15.87400	-21.38500
H	-32.21500	-16.60600	-20.78400
H	-31.16600	-15.96300	-21.66600
O	-29.44900	-16.16200	-21.89600
H	-28.73900	-16.80200	-21.93800
H	-29.49200	-15.91000	-20.97400
O	-21.46000	-9.22500	-18.97600
H	-21.51900	-9.90900	-19.64300
H	-21.05800	-8.48300	-19.42800
O	-30.73600	-19.17500	-20.07700
H	-31.27200	-19.62600	-19.42500
H	-31.32400	-19.04000	-20.82000
O	-32.44100	-2.40900	-26.18200
H	-32.20100	-1.77700	-25.50400
H	-33.28400	-2.76000	-25.89600
O	-21.45800	-11.26100	-20.74400
H	-22.34600	-11.59800	-20.62200
H	-20.88800	-11.99300	-20.50700
O	-35.00200	-20.03900	-22.99100
H	-35.08600	-20.64700	-23.72600
H	-35.82400	-19.54900	-22.99100
O	-27.92800	-6.60100	-23.77800
H	-28.00100	-5.79500	-23.26600
H	-28.37000	-6.40200	-24.60300
O	-28.13900	-7.36400	-30.26800
H	-28.92200	-6.82100	-30.17400
H	-27.44000	-6.84600	-29.86800
O	-24.82900	-21.55400	-27.62400
H	-24.33300	-22.30700	-27.94400
H	-25.60100	-21.51800	-28.19000
O	-16.97700	-15.85000	-25.98300
H	-16.98800	-14.96800	-26.35300
H	-17.84500	-16.20300	-26.17700
O	-25.46800	-6.70200	-21.62200
H	-24.75500	-6.06700	-21.69500
H	-25.95900	-6.60600	-22.43800
O	-23.74700	-12.79700	-30.05100
H	-24.68100	-12.67700	-30.22000
H	-23.56900	-13.70000	-30.31500
O	-28.24600	-19.41200	-27.89700
H	-28.61600	-19.96500	-27.20900
H	-27.75000	-20.01700	-28.45000
O	-20.35900	-16.95500	-29.51600
H	-19.89300	-16.12200	-29.59000
H	-19.90800	-17.42100	-28.81100
O	-30.41700	-8.95300	-20.80200
H	-29.71600	-8.31900	-20.64900
H	-30.88800	-8.61100	-21.56200
O	-22.29200	-10.61100	-32.51700
H	-22.01600	-10.72500	-31.60800
H	-23.16100	-11.00900	-32.55600

O	-22.38800	-24.58700	-25.47700
H	-23.30100	-24.35700	-25.30800
H	-21.95800	-24.50300	-24.62500
O	-22.61700	-16.39800	-22.97700
H	-23.27000	-16.15100	-23.63100
H	-22.23900	-17.21200	-23.31000
O	-25.65600	-6.36200	-28.87400
H	-25.47400	-7.18000	-28.41100
H	-24.83300	-6.14500	-29.31100
O	-25.32200	-7.43500	-31.61200
H	-24.79300	-7.94600	-32.22500
H	-25.05900	-7.75100	-30.74800
O	-26.56800	-15.65600	-16.49000
H	-27.24400	-15.75700	-15.82000
H	-25.74500	-15.81200	-16.02600
O	-34.82000	-9.41900	-23.86900
H	-34.23500	-10.16500	-23.73600
H	-35.01800	-9.10900	-22.98500
O	-31.96600	-6.38400	-28.85200
H	-32.63500	-5.89900	-29.33600
H	-32.10500	-6.13700	-27.93800
O	-40.22900	-16.84800	-25.14800
H	-40.19000	-17.38200	-24.35500
H	-40.73400	-17.37500	-25.76700
O	-27.82600	-4.34900	-22.25400
H	-27.27400	-4.18600	-21.48900
H	-27.48500	-3.75900	-22.92600
O	-32.84900	-22.00200	-21.65200
H	-32.78500	-21.14000	-22.06400
H	-32.91300	-22.61500	-22.38500
O	-33.37400	-19.93000	-18.00500
H	-32.74700	-20.31200	-17.39100
H	-34.23100	-20.14800	-17.63900
O	-22.97500	-9.52300	-16.39900
H	-22.43100	-8.91000	-16.89400
H	-23.55600	-8.96500	-15.88300
O	-24.68900	-6.40100	-25.26800
H	-24.81400	-7.20200	-25.77700
H	-24.03100	-5.90700	-25.75600
O	-33.06800	-20.04600	-26.09000
H	-33.79000	-19.43300	-26.22900
H	-32.27900	-19.51100	-26.16800
O	-22.62600	-8.60000	-23.64000
H	-23.57300	-8.72000	-23.71200
H	-22.27700	-9.48800	-23.55700
O	-30.26600	-22.71100	-19.81600
H	-29.74200	-22.46500	-19.05300
H	-30.48400	-21.87900	-20.23600
O	-25.62100	-8.80500	-27.43900
H	-25.76800	-9.73300	-27.25900
H	-24.79600	-8.78300	-27.92400
O	-22.01800	-7.98200	-26.35300
H	-21.09300	-8.18800	-26.49000
H	-22.18600	-8.23100	-25.44400
O	-29.06000	-21.77000	-22.13100
H	-29.40100	-22.56900	-21.72900
H	-29.76600	-21.46900	-22.70300
O	-28.40900	-24.68500	-23.67800
H	-27.58600	-25.15900	-23.80000
H	-28.16500	-23.90600	-23.17800
O	-30.88500	-17.99300	-25.86900
H	-31.03000	-17.67900	-24.97700
H	-31.36200	-17.37400	-26.42200
O	-29.02500	-21.00300	-25.82100
H	-29.03900	-21.89800	-26.15800
H	-28.30800	-20.99500	-25.18700
O	-27.17300	-20.56900	-23.77600
H	-27.43100	-19.64700	-23.77700
H	-27.70400	-20.96600	-23.08600
O	-22.22800	-7.46400	-30.99700
H	-22.05400	-8.02900	-31.74900
H	-21.73500	-6.66400	-31.17900
O	-36.41000	-15.45200	-22.93000
H	-36.95500	-14.67600	-22.79800
H	-35.53200	-15.10500	-23.08800
O	-16.37400	-14.70000	-29.37800
H	-15.93100	-15.44900	-29.77600
H	-17.25100	-14.70900	-29.76000
O	-21.24000	-8.14700	-33.89700
H	-20.54500	-7.52600	-33.67800
H	-20.89800	-8.99700	-33.62200
O	-35.62400	-16.32500	-20.03700
H	-36.36600	-16.17700	-19.45100
H	-35.94400	-16.06400	-20.90100
O	-19.34100	-10.69700	-32.36800
H	-18.81600	-10.35800	-33.09300
H	-19.42900	-11.63200	-32.55200
O	-27.67900	-6.78400	-20.23900
H	-27.37100	-7.02900	-19.36700
H	-26.88200	-6.57900	-20.72700
O	-27.65700	-4.63000	-28.00900
H	-28.09700	-5.12200	-27.31500
H	-26.90600	-5.17300	-28.25000
O	-35.17800	-8.43900	-28.00600
H	-34.35200	-8.73800	-28.38600
H	-35.52900	-9.20800	-27.55700
O	-30.21000	-9.08600	-25.93700
H	-30.83200	-9.74900	-26.23600
H	-29.53900	-9.06000	-26.61900
O	-36.97700	-10.52000	-27.04700
H	-37.81400	-10.94800	-27.22900
H	-37.00500	-10.32700	-26.11000
O	-30.13700	-20.60800	-16.34300
H	-30.08900	-19.73200	-16.72500
H	-29.59500	-21.15000	-16.91600
O	-19.65200	-10.06400	-17.06300
H	-20.44200	-10.08100	-17.60300
H	-19.88900	-9.52500	-16.30800
O	-19.53200	-13.21500	-19.75000
H	-19.48200	-13.38000	-18.80800
H	-19.64100	-14.08200	-20.14000
O	-30.64900	-10.53600	-32.46100
H	-30.50000	-9.85200	-33.11400
H	-29.84200	-10.55500	-31.94600
O	-22.24200	-17.23100	-18.43400
H	-21.34100	-17.37100	-18.72600
H	-22.16700	-17.06600	-17.49400
O	-27.90700	-19.85500	-19.58900

H	-28.77700	-19.56100	-19.86000
H	-27.67200	-19.27100	-18.86800
O	-18.03000	-10.79500	-19.50600
H	-18.32200	-10.43200	-18.66900
H	-18.64300	-11.50800	-19.68400
O	-16.83100	-13.23000	-26.98500
H	-15.99600	-12.79100	-26.82200
H	-16.69500	-13.70600	-27.80500
O	-19.78200	-15.67200	-21.03800
H	-19.30600	-15.47900	-21.84600
H	-20.70500	-15.61600	-21.28300
O	-25.20400	-23.63700	-22.07200
H	-24.68500	-22.90100	-22.39700
H	-25.55800	-23.33000	-21.23700
O	-24.95200	-27.21700	-26.55200
H	-24.67200	-27.88400	-25.92600
H	-24.16700	-27.02400	-27.06500
O	-27.14900	-8.04100	-34.98900
H	-27.72200	-7.42200	-35.44200
H	-26.57600	-7.49100	-34.45600
O	-21.08100	-18.79500	-22.80500
H	-20.13000	-18.69500	-22.78400
H	-21.25100	-19.58700	-22.29500
O	-23.06000	-21.70900	-22.61900
H	-23.65500	-21.42900	-23.31400
H	-23.34500	-21.22000	-21.84700
O	-23.92900	-7.75700	-33.85900
H	-22.99600	-7.82300	-33.65500
H	-24.01700	-6.92100	-34.31700
O	-21.30000	-8.44100	-36.89800
H	-21.37900	-8.77500	-36.00500
H	-20.47900	-7.94800	-36.89900
O	-18.60300	-15.57400	-23.69100
H	-18.63500	-16.51300	-23.50500
H	-17.99300	-15.49600	-24.42400
O	-18.45300	-18.78300	-23.64500
H	-18.96900	-19.56700	-23.83200
H	-17.56900	-19.11100	-23.48000
O	-20.79200	-25.03900	-27.68500
H	-20.99000	-25.34800	-26.80100
H	-20.18200	-24.31300	-27.55500
O	-12.78500	-14.51300	-24.55000
H	-12.05500	-14.77400	-23.98900
H	-13.39400	-15.25000	-24.51000
O	-14.70300	-16.37200	-23.95300
H	-15.43000	-16.50600	-24.56100
H	-15.11900	-16.06400	-23.14700
O	-26.88000	-12.65300	-16.18800
H	-25.96000	-12.47500	-15.99400
H	-26.91500	-13.59700	-16.34000
O	-33.88400	-14.74300	-23.54600
H	-33.66600	-13.81100	-23.52700
H	-33.47200	-15.10200	-22.76000
O	-29.01100	-19.17000	-14.15500
H	-29.11200	-18.22000	-14.22400
H	-29.22900	-19.49800	-15.02800
O	-37.79300	-13.83900	-25.29300
H	-38.48700	-14.25300	-25.80600
H	-37.04600	-13.79700	-25.89000
O	-23.20700	-23.64800	-28.22600
H	-22.70700	-23.53600	-29.03400
H	-22.56300	-23.95200	-27.58500
O	-29.14800	-15.74000	-15.33900
H	-29.63200	-14.94000	-15.13200
H	-29.06200	-15.72900	-16.29200
O	-35.88000	-13.90700	-27.41700
H	-35.90300	-13.80800	-28.36800
H	-35.57000	-13.06000	-27.09500
O	-33.22100	-11.84900	-23.30200
H	-32.26700	-11.88100	-23.36900
H	-33.42000	-12.31000	-22.48700
O	-32.63100	-13.27900	-20.73500
H	-31.81200	-12.78500	-20.71100
H	-32.35700	-14.19600	-20.76300
O	-19.52300	-20.86400	-27.65700
H	-18.74700	-20.37400	-27.38400
H	-20.24800	-20.43800	-27.19900
O	-17.08600	-20.15100	-26.59900
H	-16.49100	-20.39100	-27.31000
H	-17.01300	-20.87000	-25.97200
O	-34.86600	-6.30700	-26.28500
H	-35.00100	-7.06700	-26.85100
H	-35.28300	-5.58300	-26.75200
O	-22.90000	-15.37300	-31.15700
H	-22.95700	-14.91600	-31.99600
H	-22.34700	-16.13400	-31.33500
O	-19.09000	-19.58400	-30.33200
H	-19.34600	-19.98400	-29.50100
H	-19.71500	-18.86900	-30.45700
O	-14.77900	-16.84400	-27.82500
H	-15.33900	-16.77500	-27.05200
H	-15.38400	-17.02000	-28.54500
O	-31.70900	-5.41000	-26.07300
H	-31.82600	-4.46600	-26.17500
H	-32.53000	-5.71300	-25.68400
O	-21.82100	-10.68500	-35.22700
H	-20.98700	-11.11700	-35.41000
H	-21.98300	-10.85700	-34.30000
O	-22.16700	-14.18200	-33.86200
H	-22.22200	-14.34800	-34.80300
H	-22.79200	-13.47500	-33.70600
O	-37.37800	-18.94500	-20.03600
H	-36.73200	-19.45600	-19.54700
H	-37.58600	-18.20900	-19.46100
O	-24.85200	-11.87700	-14.53600
H	-23.98300	-11.70300	-14.89800
H	-25.33200	-11.05800	-14.65900
O	-30.68400	-4.75800	-22.85700
H	-29.78100	-4.72500	-22.54100
H	-30.91000	-5.68800	-22.84300
O	-28.92800	-6.00500	-26.19600
H	-29.87900	-6.00400	-26.31000
H	-28.62500	-6.75100	-26.71300
O	-33.03800	-21.79900	-15.93800
H	-32.17500	-22.15500	-15.72800
H	-33.61100	-22.13700	-15.25100
O	-13.19300	-0.16900	-25.13200
H	-12.55500	-0.28800	-25.83600

H	-13.31100	0.78000	-25.07300
O	-16.38400	-0.21600	-25.19600
H	-16.72200	-0.33200	-26.08400
H	-15.51500	-0.61700	-25.21600
O	-20.52500	1.63800	-29.20600
H	-21.08900	1.54700	-29.97400
H	-20.64000	0.81800	-28.72600
O	-15.90200	0.93800	-19.23700
H	-15.91300	0.54300	-20.10900
H	-15.74000	0.20400	-18.64400
O	-22.97900	3.13300	-26.36800
H	-22.18700	2.79200	-26.78300
H	-22.73900	3.24200	-25.44700
O	-14.94500	5.30500	-33.89500
H	-15.59700	5.89000	-34.28100
H	-14.15100	5.83500	-33.83500
O	-21.02000	2.39400	-33.49600
H	-20.24700	1.84700	-33.63500
H	-20.66700	3.25800	-33.28400
O	-19.18100	3.72100	-20.28900
H	-19.47200	4.62600	-20.40500
H	-18.54600	3.76300	-19.57500
O	-18.25600	1.09900	-23.69900
H	-17.65900	0.87500	-24.41300
H	-18.19800	0.35800	-23.09700
O	-18.68300	-1.44700	-35.86400
H	-18.43100	-1.96900	-36.62600
H	-19.62100	-1.61000	-35.76100
O	-13.63900	-2.44300	-28.05700
H	-12.85300	-2.86100	-28.40800
H	-13.37500	-1.53900	-27.88800
O	-13.46800	2.99400	-33.35800
H	-14.10300	3.06700	-32.64600
H	-13.79800	3.58300	-34.03600
O	-8.23800	-2.50600	-27.15300
H	-7.50500	-3.07600	-26.91900
H	-8.99400	-2.89400	-26.71300
O	-18.96400	0.45600	-33.66200
H	-19.08000	-0.22000	-32.99300
H	-18.85300	-0.03100	-34.47800
O	-16.62000	-0.08300	-29.95700
H	-16.98500	-0.61000	-29.24600
H	-15.76400	-0.47300	-30.13000
O	-10.71500	-8.79400	-22.69400
H	-11.04700	-7.92400	-22.91800
H	-11.31100	-9.11000	-22.01400
O	-16.98000	-5.05400	-34.96100
H	-16.81200	-4.18600	-34.59400
H	-17.17000	-4.89100	-35.88500
O	-14.22200	3.40800	-23.29400
H	-14.52200	4.31000	-23.40300
H	-14.56700	3.14100	-22.44200
O	-20.77100	-0.14100	-27.26100
H	-21.69600	-0.27300	-27.05300
H	-20.52300	0.63800	-26.76400
O	-10.82300	-4.07400	-25.61400
H	-11.73100	-4.14300	-25.90900
H	-10.87400	-3.59700	-24.78600
O	-17.00100	-2.59800	-33.97600
H	-17.12100	-2.15300	-33.13800
H	-17.69600	-2.25000	-34.53500
O	-23.41000	-0.49800	-26.42600
H	-23.28100	-0.24400	-25.51200
H	-24.09000	0.09500	-26.74600
O	-19.24000	10.17200	-30.32700
H	-19.31800	11.12300	-30.24800
H	-19.84400	9.82700	-29.67000
O	-16.74800	-2.65500	-17.67100
H	-17.52500	-2.09600	-17.68100
H	-16.10900	-2.17000	-17.14900
O	-8.95600	-5.87300	-23.83700
H	-9.59900	-5.57900	-24.48300
H	-9.46100	-5.98700	-23.03100
O	-11.30200	-2.12600	-33.14800
H	-10.39100	-1.89300	-33.32300
H	-11.81100	-1.54300	-33.71100
O	-18.49400	0.15300	-18.10900
H	-17.75200	0.69200	-18.38600
H	-19.20000	0.78000	-17.95200
O	-13.01800	2.29600	-19.51200
H	-12.61000	1.85400	-20.25700
H	-13.74100	1.72200	-19.25900
O	-16.66500	-2.76600	-22.13900
H	-15.81900	-2.34300	-21.99500
H	-16.72500	-3.42300	-21.44500
O	-12.69500	2.85600	-25.75700
H	-13.32500	3.02100	-26.45900
H	-13.08300	3.27100	-24.98600
O	-8.81700	3.79200	-28.40200
H	-9.50100	4.17200	-28.95300
H	-8.73200	4.40100	-27.66900
O	-13.51500	-5.10600	-20.48000
H	-13.75900	-4.35200	-21.01700
H	-13.23900	-4.72800	-19.64600
O	-11.56100	1.43800	-21.80300
H	-11.35000	2.08100	-22.47900
H	-11.60800	0.60400	-22.27100
O	-13.95900	-5.95000	-24.89900
H	-14.82000	-5.65200	-24.60700
H	-13.93300	-5.73300	-25.83100
O	-17.14300	2.59400	-28.49200
H	-16.67900	2.87300	-29.28100
H	-17.40700	1.69200	-28.67400
O	-20.25000	2.14100	-25.55400
H	-19.68000	2.57000	-26.19200
H	-19.65900	1.85900	-24.85500
O	-18.20600	-1.28400	-31.68300
H	-17.50300	-0.83200	-31.21500
H	-18.53800	-1.92600	-31.05500
O	-19.75800	-2.69300	-17.30800
H	-20.38800	-2.01100	-17.07400
H	-19.86400	-2.80400	-18.25200
O	-24.46300	-3.02600	-26.38100
H	-25.25300	-2.77000	-26.85700
H	-23.97200	-2.21100	-26.27600
O	-10.44700	4.31500	-23.63100
H	-9.62500	3.91300	-23.91200
H	-10.45100	4.21200	-22.67900

O	-22.51200	-2.66100	-29.42300
H	-23.30900	-2.54900	-28.90400
H	-22.73900	-2.32200	-30.28900
O	-16.68100	3.86300	-21.47400
H	-16.61700	4.32500	-20.63900
H	-17.39000	3.23300	-21.34600
O	-26.17900	-2.77200	-23.95000
H	-25.92900	-1.88800	-23.68400
H	-25.64300	-2.95200	-24.72200
O	-2.35600	0.65900	-27.28200
H	-3.11000	0.09000	-27.43800
H	-2.22100	0.62500	-26.33500
O	-16.96600	5.53000	-31.60700
H	-17.10200	6.37500	-31.17800
H	-16.42500	5.73200	-32.37000
O	-20.48000	-8.39100	-14.94500
H	-20.87300	-8.94400	-14.27000
H	-19.66300	-8.08000	-14.55400
O	-23.51300	3.04800	-31.03400
H	-23.00100	3.84200	-30.88300
H	-22.86100	2.35000	-31.10000
O	-21.70400	6.16400	-28.95100
H	-21.52700	7.08600	-29.13900
H	-20.90800	5.85000	-28.52200
O	-17.56300	-6.97500	-17.35300
H	-16.82200	-6.37400	-17.43300
H	-17.33100	-7.71700	-17.91100
O	-22.41900	-1.78900	-19.07500
H	-22.53300	-2.69100	-18.77500
H	-23.27400	-1.54300	-19.42700
O	-15.34000	6.15300	-17.47700
H	-15.78300	5.36600	-17.79400
H	-14.67800	6.34000	-18.14200
O	-20.58100	7.68200	-22.95000
H	-20.03000	7.55400	-23.72300
H	-21.42000	7.98200	-23.30100
O	-13.24100	6.43300	-19.44800
H	-13.26000	7.25400	-19.94000
H	-12.31900	6.30800	-19.22300
O	-16.78400	3.94700	-18.60600
H	-16.25100	3.18600	-18.83600
H	-17.31300	3.65500	-17.86400
O	-13.61700	-10.94700	-23.07700
H	-13.96100	-10.05700	-23.15300
H	-13.24900	-11.13700	-23.94000
O	-25.54900	1.37700	-30.25800
H	-24.71100	1.78600	-30.47400
H	-25.37100	0.43700	-30.28800
O	-13.65200	-0.95600	-34.56900
H	-14.16700	-0.18500	-34.33100
H	-13.92400	-1.62900	-33.94600
O	-16.06700	-6.11100	-29.51100
H	-16.92300	-6.50000	-29.69000
H	-15.65500	-6.03200	-30.37100
O	-14.53700	-8.57600	-25.11200
H	-15.04100	-8.61300	-25.92500
H	-14.24400	-7.66700	-25.05200
O	-18.29300	-8.49200	-26.10500
H	-17.42800	-8.68800	-26.46500
H	-18.22500	-8.72100	-25.17800
O	-8.67900	1.79700	-32.63400
H	-9.45600	1.45800	-33.07900
H	-8.00000	1.80300	-33.30900
O	-13.86300	-3.08300	-32.74300
H	-14.16800	-2.76900	-31.89200
H	-12.91000	-3.00500	-32.70100
O	-15.41900	3.07600	-31.09400
H	-15.94700	2.34100	-31.40700
H	-15.92000	3.85300	-31.34000
O	-10.44000	1.27100	-25.97700
H	-11.09700	1.96100	-25.88900
H	-9.63600	1.66000	-25.63400
O	-20.68700	-6.26200	-28.73300
H	-20.11300	-6.48500	-29.46600
H	-20.10000	-6.19300	-27.98100
O	-11.62900	-0.37500	-27.92700
H	-11.11600	0.25800	-27.42500
H	-10.97900	-0.97500	-28.29300
O	-20.36000	-4.23200	-34.71400
H	-21.19600	-3.89400	-34.39300
H	-20.50000	-4.36700	-35.65100
O	-18.38500	-9.38800	-30.00600
H	-19.03800	-9.70300	-30.63000
H	-18.42700	-10.01000	-29.28000
O	-17.27400	0.89400	-38.09100
H	-18.18100	0.66300	-37.89200
H	-16.77600	0.09800	-37.90300
O	-14.36700	9.60400	-35.17100
H	-15.04100	9.90700	-34.56200
H	-13.98000	10.40700	-35.52000
O	-15.15100	-2.90000	-25.68100
H	-16.06900	-2.67800	-25.83900
H	-14.73500	-2.82500	-26.54000
O	-14.36700	-1.50100	-30.55100
H	-13.62000	-0.98900	-30.86000
H	-14.09800	-1.82200	-29.69100
O	-16.09500	0.99500	-33.56800
H	-17.00400	0.71100	-33.47100
H	-16.06500	1.41500	-34.42800
O	-10.86300	4.60600	-29.79900
H	-11.31700	3.76500	-29.74300
H	-11.49300	5.24200	-29.46200
O	-11.92000	-4.46500	-28.56900
H	-12.65700	-4.97700	-28.23800
H	-11.66700	-4.90500	-29.38100
O	-12.42000	6.44300	-28.86400
H	-12.12700	7.00400	-29.58300
H	-11.91000	6.73600	-28.10900
O	-11.81800	-11.68700	-27.97500
H	-11.51200	-12.54800	-28.26200
H	-11.09300	-11.09600	-28.17500
O	-25.52600	-1.28400	-29.58700
H	-25.72300	-1.87700	-30.31200
H	-26.08100	-1.58900	-28.86900
O	-17.54600	-1.39200	-27.46700
H	-17.49100	-2.34500	-27.54300
H	-18.35700	-1.23600	-26.98300
O	-16.39700	2.53600	-36.22900

H	-16.83500	1.82000	-36.68900
H	-15.65600	2.76400	-36.79100
O	-19.60700	-2.77400	-29.70800
H	-20.42000	-2.47500	-29.30200
H	-19.11100	-3.16900	-28.99100
O	-17.45600	7.22100	-27.77500
H	-17.49900	7.44800	-28.70400
H	-16.54000	6.98800	-27.62600
O	-14.01000	-2.31000	-20.87700
H	-13.65500	-2.08300	-20.01700
H	-13.36200	-1.97800	-21.49900
O	-8.28000	0.81200	-30.17200
H	-8.41800	1.00200	-31.10000
H	-8.63600	1.57400	-29.71500
O	-20.11600	-5.20200	-24.09200
H	-19.47600	-5.34400	-24.79000
H	-20.94800	-5.48900	-24.46800
O	-8.25300	2.87900	-25.16100
H	-7.81900	2.78700	-24.31200
H	-7.54000	2.85500	-25.79900
O	-19.34000	4.09800	-27.97900
H	-18.54600	3.60300	-28.18000
H	-20.05000	3.57000	-28.34200
O	-14.52000	6.48000	-23.62700
H	-14.46600	7.04900	-22.86000
H	-15.38900	6.65000	-23.99200
O	-23.03100	-1.75800	-31.90300
H	-23.96200	-1.81900	-31.68600
H	-22.93600	-2.28000	-32.69900
O	-23.11300	-5.23200	-29.31500
H	-22.85200	-4.31800	-29.42400
H	-22.29100	-5.70000	-29.16500
O	-21.64000	-3.81900	-22.14500
H	-20.96800	-4.02700	-22.79400
H	-22.46700	-4.01600	-22.58400
O	-7.39100	2.92200	-22.62700
H	-7.19800	3.82600	-22.38100
H	-7.49100	2.46200	-21.79400
O	-15.73800	-7.19200	-20.12800
H	-16.38500	-6.49300	-20.22200
H	-14.96800	-6.75900	-19.76100
O	-12.33000	3.07200	-17.05300
H	-12.48100	2.84300	-17.97000
H	-12.33900	4.02900	-17.04000
O	-15.56100	0.64600	-22.06900
H	-14.63200	0.79700	-22.24200
H	-15.98200	0.73400	-22.92400
O	-16.76200	4.67100	-24.29900
H	-16.90100	4.30300	-23.42600
H	-17.55100	5.18500	-24.47000
O	-16.16800	9.77200	-32.95600
H	-15.99200	9.30000	-32.14200
H	-16.85400	10.39800	-32.72800
O	-15.21200	4.04900	-26.91000
H	-15.54700	4.00900	-26.01400
H	-15.71200	3.38300	-27.38300
O	-21.23800	-0.54400	-21.38500
H	-21.37600	-1.27600	-20.78400
H	-20.32700	-0.63300	-21.66600
O	-12.27600	1.39400	-36.63600
H	-12.89300	0.66900	-36.73300
H	-12.74400	2.15500	-36.98000
O	-18.60900	-0.83200	-21.89600
H	-17.89900	-1.47300	-21.93800
H	-18.65200	-0.58100	-20.97400
O	-10.62100	6.10400	-18.97600
H	-10.67900	5.42000	-19.64300
H	-10.21900	6.84600	-19.42800
O	-19.89600	-3.84600	-20.07700
H	-20.43300	-4.29700	-19.42500
H	-20.48500	-3.71100	-20.82000
O	-21.60200	12.92100	-26.18200
H	-21.36200	13.55200	-25.50400
H	-22.44500	12.56900	-25.89600
O	-20.48600	-4.38700	-37.51400
H	-21.36300	-4.50800	-37.87800
H	-20.01000	-5.17800	-37.76600
O	-10.61900	4.06800	-20.74400
H	-11.50600	3.73100	-20.62200
H	-10.04900	3.33700	-20.50700
O	-28.96600	0.40100	-27.42000
H	-28.27900	0.08700	-28.00800
H	-29.12100	1.30500	-27.69300
O	-24.16200	-4.71000	-22.99100
H	-24.24700	-5.31800	-23.72600
H	-24.98400	-4.21900	-22.99100
O	-17.08900	8.72800	-23.77800
H	-17.16200	9.53400	-23.26600
H	-17.53100	8.92800	-24.60300
O	-17.30000	7.96600	-30.26800
H	-18.08300	8.50800	-30.17400
H	-16.60000	8.48300	-29.86800
O	-13.99000	-6.22500	-27.62400
H	-13.49300	-6.97800	-27.94400
H	-14.76100	-6.18800	-28.19000
O	-6.13700	-0.52100	-25.98300
H	-6.14800	0.36200	-26.35300
H	-7.00500	-0.87400	-26.17700
O	-14.62900	8.62700	-21.62200
H	-13.91600	9.26200	-21.69500
H	-15.12000	8.72400	-22.43800
O	-12.90700	2.53200	-30.05100
H	-13.84100	2.65200	-30.22000
H	-12.72900	1.63000	-30.31500
O	-17.40700	-4.08300	-27.89700
H	-17.77700	-4.63600	-27.20900
H	-16.91100	-4.68700	-28.45000
O	-9.52000	-1.62600	-29.51600
H	-9.05400	-0.79300	-29.59000
H	-9.06900	-2.09200	-28.81100
O	-19.57800	6.37700	-20.80200
H	-18.87700	7.01000	-20.64900
H	-20.04800	6.71800	-21.56200
O	-11.45200	4.71800	-32.51700
H	-11.17600	4.60400	-31.60800
H	-12.32200	4.32000	-32.55600
O	-11.54800	-9.25800	-25.47700
H	-12.46200	-9.02800	-25.30800

H	-11.11900	-9.17400	-24.62500
O	-11.77800	-1.06900	-22.97700
H	-12.43000	-0.82100	-23.63100
H	-11.40000	-1.88300	-23.31000
O	-14.81700	8.96700	-28.87400
H	-14.63500	8.14900	-28.41100
H	-13.99400	9.18400	-29.31100
O	-14.48300	7.89400	-31.61200
H	-13.95400	7.38300	-32.22500
H	-14.22000	7.57800	-30.74800
O	-15.72900	-0.32700	-16.49000
H	-16.40500	-0.42800	-15.82000
H	-14.90600	-0.48300	-16.02600
O	-23.98100	5.91000	-23.86900
H	-23.39600	5.16500	-23.73600
H	-24.17800	6.22100	-22.98500
O	-21.12700	8.94500	-28.85200
H	-21.79600	9.43000	-29.33600
H	-21.26600	9.19200	-27.93800
O	-29.38900	-1.51900	-25.14800
H	-29.35100	-2.05300	-24.35500
H	-29.89500	-2.04600	-25.76700
O	-16.98700	10.98000	-22.25400
H	-16.43500	11.14300	-21.48900
H	-16.64600	11.57000	-22.92600
O	-22.01000	-6.67300	-21.65200
H	-21.94600	-5.81100	-22.06400
H	-22.07300	-7.28500	-22.38500
O	-22.53500	-4.60000	-18.00500
H	-21.90800	-4.98300	-17.39100
H	-23.39200	-4.81900	-17.63900
O	-12.13500	5.80600	-16.39900
H	-11.59100	6.41900	-16.89400
H	-12.71700	6.36400	-15.88300
O	-25.75800	-2.79300	-32.07300
H	-25.46300	-3.70200	-32.12600
H	-26.65900	-2.81400	-32.39500
O	-13.85000	8.92800	-25.26800
H	-13.97500	8.12700	-25.77700
H	-13.19200	9.42300	-25.75600
O	-22.22900	-4.71700	-26.09000
H	-22.95000	-4.10400	-26.22900
H	-21.44000	-4.18100	-26.16800
O	-11.78600	6.72900	-23.64000
H	-12.73300	6.60900	-23.71200
H	-11.43800	5.84100	-23.55700
O	-19.42600	-7.38200	-19.81600
H	-18.90300	-7.13600	-19.05300
H	-19.64500	-6.55000	-20.23600
O	-24.03000	-5.11100	-31.94900
H	-24.35800	-5.99200	-32.13100
H	-23.67600	-5.16700	-31.06100
O	-14.78200	6.52400	-27.43900
H	-14.92900	5.59600	-27.25900
H	-13.95600	6.54600	-27.92400
O	-20.91500	-4.82400	-31.58100
H	-20.57600	-4.29600	-32.30300
H	-20.46800	-4.48600	-30.80500
O	-11.17800	7.34700	-26.35300
H	-10.25300	7.14100	-26.49000
H	-11.34700	7.09900	-25.44400
O	-18.22100	-6.44000	-22.13100
H	-18.56100	-7.24000	-21.72900
H	-18.92700	-6.14000	-22.70300
O	-17.56900	-9.35500	-23.67800
H	-16.74700	-9.82900	-23.80000
H	-17.32600	-8.57600	-23.17800
O	-18.92900	-6.32700	-33.38200
H	-18.20800	-5.94900	-33.88600
H	-19.55200	-5.60800	-33.27900
O	-14.78500	-5.62200	-32.44600
H	-14.56900	-4.74000	-32.74700
H	-15.26600	-6.01400	-33.17600
O	-20.04500	-2.66400	-25.86900
H	-20.19100	-2.35000	-24.97700
H	-20.52300	-2.04500	-26.42200
O	-18.18600	-5.67300	-25.82100
H	-18.20000	-6.56900	-26.15800
H	-17.46900	-5.66600	-25.18700
O	-16.33300	-5.24000	-23.77600
H	-16.59100	-4.31800	-23.77700
H	-16.86500	-5.63700	-23.08600
O	-11.38900	7.86600	-30.99700
H	-11.21500	7.30000	-31.74900
H	-10.89600	8.66600	-31.17900
O	-25.57100	-0.12300	-22.93000
H	-26.11600	0.65300	-22.79800
H	-24.69300	0.22400	-23.08800
O	-18.78200	-6.79200	-30.55200
H	-18.66100	-7.72000	-30.35300
H	-19.07200	-6.77800	-31.46400
O	-15.91300	-9.10600	-27.41600
H	-15.89100	-9.04200	-28.37000
H	-15.49600	-9.94600	-27.22200
O	-5.53500	0.62900	-29.37800
H	-5.09200	-0.12000	-29.77600
H	-6.41200	0.62000	-29.76000
O	-10.40100	7.18200	-33.89700
H	-9.70600	7.80300	-33.67800
H	-10.05900	6.33200	-33.62200
O	-24.78400	-0.99600	-20.03700
H	-25.52600	-0.84800	-19.45100
H	-25.10500	-0.73500	-20.90100
O	-8.50200	4.63200	-32.36800
H	-7.97700	4.97100	-33.09300
H	-8.59000	3.69700	-32.55200
O	-16.84000	8.54600	-20.23900
H	-16.53100	8.30000	-19.36700
H	-16.04200	8.75000	-20.72700
O	-16.81800	10.69900	-28.00900
H	-17.25700	10.20700	-27.31500
H	-16.06700	10.15700	-28.25000
O	-24.33900	6.89100	-28.00600
H	-23.51300	6.59100	-28.38600
H	-24.68900	6.12100	-27.55700
O	-19.37000	6.24300	-25.93700
H	-19.99200	5.58000	-26.23600
H	-18.70000	6.26900	-26.61900

O	-26.13800	4.80900	-27.04700
H	-26.97500	4.38100	-27.22900
H	-26.16500	5.00200	-26.11000
O	-19.29800	-5.27900	-16.34300
H	-19.24900	-4.40200	-16.72500
H	-18.75600	-5.82100	-16.91600
O	-8.81300	5.26500	-17.06300
H	-9.60300	5.24800	-17.60300
H	-9.04900	5.80400	-16.30800
O	-8.69300	2.11500	-19.75000
H	-8.64300	1.95000	-18.80800
H	-8.80100	1.24700	-20.14000
O	-19.80900	4.79400	-32.46100
H	-19.66000	5.47700	-33.11400
H	-19.00200	4.77400	-31.94600
O	-11.40200	-1.90200	-18.43400
H	-10.50100	-2.04200	-18.72600
H	-11.32800	-1.73700	-17.49400
O	-17.06800	-4.52600	-19.58900
H	-17.93800	-4.23200	-19.86000
H	-16.83200	-3.94100	-18.86800
O	-7.19100	4.53400	-19.50600
H	-7.48200	4.89700	-18.66900
H	-7.80400	3.82100	-19.68400
O	-5.99100	2.10000	-26.98500
H	-5.15700	2.53900	-26.82200
H	-5.85500	1.62400	-27.80500
O	-8.94200	-0.34200	-21.03800
H	-8.46700	-0.15000	-21.84600
H	-9.86600	-0.28600	-21.28300
O	-22.05400	0.82000	-31.45500
H	-22.29900	-0.08400	-31.25700
H	-21.88400	0.82200	-32.39700
O	-14.36400	-8.30800	-22.07200
H	-13.84600	-7.57100	-22.39700
H	-14.71900	-8.00100	-21.23700
O	-22.78400	-3.44700	-33.95600
H	-23.47700	-3.37300	-34.61200
H	-23.02900	-4.21100	-33.43400
O	-14.11300	-11.88800	-26.55200
H	-13.83300	-12.55500	-25.92600
H	-13.32800	-11.69500	-27.06500
O	-16.31000	7.28800	-34.98900
H	-16.88200	7.90700	-35.44200
H	-15.73600	7.83800	-34.45600
O	-10.24200	-3.46600	-22.80500
H	-9.29000	-3.36600	-22.78400
H	-10.41200	-4.25800	-22.29500
O	-12.22100	-6.38000	-22.61900
H	-12.81600	-6.10000	-23.31400
H	-12.50600	-5.89100	-21.84700
O	-13.09000	7.57200	-33.85900
H	-12.15700	7.50700	-33.65500
H	-13.17800	8.40800	-34.31700
O	-10.46100	6.88900	-36.89800
H	-10.54000	6.55500	-36.00500
H	-9.64000	7.38100	-36.89900
O	-7.76300	-0.24500	-23.69100
H	-7.79600	-1.18400	-23.50500
H	-7.15300	-0.16700	-24.42400
O	-15.92500	-1.37600	-37.64800
H	-14.97900	-1.50500	-37.57900
H	-16.30300	-2.11400	-37.17100
O	-7.61400	-3.45400	-23.64500
H	-8.13000	-4.23800	-23.83200
H	-6.73000	-3.78200	-23.48000
O	-9.95300	-9.71000	-27.68500
H	-10.15000	-10.01800	-26.80100
H	-9.34300	-8.98400	-27.55500
O	-1.94600	0.81600	-24.55000
H	-1.21500	0.55500	-23.98900
H	-2.55500	0.07900	-24.51000
O	-3.86400	-1.04300	-23.95300
H	-4.59100	-1.17700	-24.56100
H	-4.27900	-0.73500	-23.14700
O	-16.04100	2.67600	-16.18800
H	-15.12100	2.85400	-15.99400
H	-16.07600	1.73200	-16.34000
O	-17.74100	-3.45100	-37.71200
H	-18.64700	-3.74700	-37.63600
H	-17.46300	-3.74800	-38.57900
O	-26.82300	-2.13000	-27.26200
H	-27.14300	-2.98300	-27.55500
H	-27.36900	-1.91500	-26.50700
O	-23.04500	0.58600	-23.54600
H	-22.82600	1.51800	-23.52700
H	-22.63200	0.22700	-22.76000
O	-16.12400	-8.90700	-31.55800
H	-16.93400	-9.25200	-31.18200
H	-15.43600	-9.21600	-30.96900
O	-14.19500	-9.82900	-29.93600
H	-13.64200	-9.41000	-29.27700
H	-13.95800	-10.75500	-29.89300
O	-18.17200	-3.84000	-14.15500
H	-18.27200	-2.89100	-14.22400
H	-18.39000	-4.16900	-15.02800
O	-18.97100	-6.59200	-37.57100
H	-18.10900	-6.26600	-37.31200
H	-18.78600	-7.37900	-38.08400
O	-26.95400	1.49000	-25.29300
H	-27.64800	1.07600	-25.80600
H	-26.20700	1.53200	-25.89000
O	-10.81700	-7.71900	-30.42800
H	-11.39800	-7.06100	-30.81000
H	-11.08400	-8.54100	-30.83900
O	-12.36700	-8.31900	-28.22600
H	-11.86700	-8.20700	-29.03400
H	-11.72400	-8.62200	-27.58500
O	-18.30900	-0.41100	-15.33900
H	-18.79200	0.38900	-15.13200
H	-18.22300	-0.40000	-16.29200
O	-25.04000	1.42200	-27.41700
H	-25.06300	1.52100	-28.36800
H	-24.73000	2.26900	-27.09500
O	-22.38200	3.48000	-23.30200
H	-21.42700	3.44800	-23.36900
H	-22.58000	3.01900	-22.48700
O	-21.79200	2.05000	-20.73500

H	-20.97200	2.54400	-20.71100
H	-21.51800	1.13300	-20.76300
O	-12.03500	-5.55900	-31.37200
H	-12.92200	-5.27400	-31.59000
H	-11.62000	-5.72000	-32.21900
O	-8.68400	-5.53400	-27.65700
H	-7.90800	-5.04500	-27.38400
H	-9.40900	-5.10900	-27.19900
O	-6.24600	-4.82200	-26.59900
H	-5.65200	-5.06200	-27.31000
H	-6.17400	-5.54100	-25.97200
O	-24.02700	9.02200	-26.28500
H	-24.16100	8.26200	-26.85100
H	-24.44300	9.74600	-26.75200
O	-12.89100	4.20500	-37.27700
H	-12.18700	4.41000	-36.66100
H	-13.61400	4.77300	-37.01200
O	-12.06100	-0.04400	-31.15700
H	-12.11700	0.41300	-31.99600
H	-11.50800	-0.80400	-31.33500
O	-8.25000	-4.25500	-30.33200
H	-8.50700	-4.65500	-29.50100
H	-8.87500	-3.54000	-30.45700
O	-3.93900	-1.51500	-27.82500
H	-4.49900	-1.44600	-27.05200
H	-4.54500	-1.69100	-28.54500
O	-20.87000	9.91900	-26.07300
H	-20.98600	10.86300	-26.17500
H	-21.69000	9.61600	-25.68400
O	-10.98200	4.64400	-35.22700
H	-10.14700	4.21200	-35.41000
H	-11.14400	4.47200	-34.30000
O	-11.32700	1.14700	-33.86200
H	-11.38300	0.98100	-34.80300
H	-11.95300	1.85500	-33.70600
O	-26.53800	-3.61600	-20.03600
H	-25.89200	-4.12600	-19.54700
H	-26.74600	-2.87900	-19.46100
O	-14.01300	3.45200	-14.53600
H	-13.14400	3.62600	-14.89800
H	-14.49200	4.27100	-14.65900
O	-18.01800	-9.35200	-34.28300
H	-18.03000	-8.40800	-34.12600
H	-17.12500	-9.53500	-34.57700
O	-19.84400	10.57200	-22.85700
H	-18.94100	10.60400	-22.54100
H	-20.07000	9.64200	-22.84300
O	-18.08900	9.32500	-26.19600
H	-19.03900	9.32500	-26.31000
H	-17.78600	8.57800	-26.71300
O	-14.20300	-12.75800	-30.02900
H	-14.96900	-13.31200	-29.88300
H	-13.79200	-12.68600	-29.16700
O	-22.19800	-6.47000	-15.93800
H	-21.33500	-6.82600	-15.72800
H	-22.77200	-6.80800	-15.25100
O	-2.35400	15.16100	-25.13200
H	-1.71600	15.04100	-25.83600
H	-2.47100	16.10900	-25.07300
O	-5.54500	15.11300	-25.19600
H	-5.88300	14.99800	-26.08400
H	-4.67600	14.71300	-25.21600
O	-9.68600	16.96700	-29.20600
H	-10.24900	16.87600	-29.97400
H	-9.80100	16.14700	-28.72600
O	-5.06300	16.26700	-19.23700
H	-5.07400	15.87200	-20.10900
H	-4.90100	15.53400	-18.64400
O	-12.13900	18.46200	-26.36800
H	-11.34700	18.12100	-26.78300
H	-11.90000	18.57200	-25.44700
O	-4.10600	20.63400	-33.89500
H	-4.75700	21.21900	-34.28100
H	-3.31200	21.16500	-33.83500
O	-10.18000	17.72300	-33.49600
H	-9.40700	17.17600	-33.63500
H	-9.82800	18.58700	-33.28400
O	-8.34200	19.05100	-20.28900
H	-8.63300	19.95500	-20.40500
H	-7.70600	19.09200	-19.57500
O	-7.41600	16.42800	-23.69900
H	-6.82000	16.20400	-24.41300
H	-7.35900	15.68700	-23.09700
O	-7.84400	13.88200	-35.86400
H	-7.59100	13.36000	-36.62600
H	-8.78100	13.71900	-35.76100
O	-2.79900	12.88600	-28.05700
H	-2.01300	12.46800	-28.40800
H	-2.53600	13.79000	-27.88800
O	-2.62800	18.32300	-33.35800
H	-3.26400	18.39700	-32.64600
H	-2.95900	18.91200	-34.03600
O	2.60100	12.82300	-27.15300
H	3.33400	12.25300	-26.91900
H	1.84500	12.43600	-26.71300
O	-8.12400	15.78500	-33.66200
H	-8.24100	15.11000	-32.99300
H	-8.01400	15.29800	-34.47800
O	-5.78100	15.24600	-29.95700
H	-6.14500	14.71900	-29.24600
H	-4.92400	14.85600	-30.13000
O	0.12400	6.53500	-22.69400
H	-0.20800	7.40500	-22.91800
H	-0.47200	6.21900	-22.01400
O	-6.14000	10.27500	-34.96100
H	-5.97300	11.14300	-34.59400
H	-6.33100	10.43800	-35.88500
O	-3.38300	18.73700	-23.29400
H	-3.68300	19.63900	-23.40300
H	-3.72800	18.47000	-22.44200
O	-9.93200	15.18800	-27.26100
H	-10.85700	15.05600	-27.05300
H	-9.68300	15.96800	-26.76400
O	0.01700	11.25500	-25.61400
H	-0.89200	11.18700	-25.90900
H	-0.03400	11.73200	-24.78600
O	-6.16200	12.73100	-33.97600
H	-6.28200	13.17700	-33.13800

H	-6.85600	13.07900	-34.53500
O	-12.57100	14.83100	-26.42600
H	-12.44200	15.08500	-25.51200
H	-13.25100	15.42400	-26.74600
O	-8.40100	25.50100	-30.32700
H	-8.47800	26.45200	-30.24800
H	-9.00500	25.15600	-29.67000
O	-5.90900	12.67400	-17.67100
H	-6.68600	13.23300	-17.68100
H	-5.27000	13.16000	-17.14900
O	1.88300	9.45700	-23.83700
H	1.24000	9.75000	-24.48300
H	1.37800	9.34200	-23.03100
O	-0.46300	13.20300	-33.14800
H	0.44900	13.43600	-33.32300
H	-0.97200	13.78700	-33.71100
O	-7.65400	15.48300	-18.10900
H	-6.91300	16.02100	-18.38600
H	-8.36100	16.10900	-17.95200
O	-2.17900	17.62500	-19.51200
H	-1.77100	17.18400	-20.25700
H	-2.90100	17.05100	-19.25900
O	-5.82600	12.56300	-22.13900
H	-4.98000	12.98700	-21.99500
H	-5.88600	11.90600	-21.44500
O	-1.85600	18.18600	-25.75700
H	-2.48500	18.35000	-26.45900
H	-2.24300	18.60000	-24.98600
O	2.02300	19.12100	-28.40200
H	1.33800	19.50100	-28.95300
H	2.10700	19.73100	-27.66900
O	-2.67600	10.22300	-20.48000
H	-2.92000	10.97700	-21.01700
H	-2.40000	10.60200	-19.64600
O	-0.72200	16.76700	-21.80300
H	-0.51000	17.41000	-22.47900
H	-0.76900	15.93300	-22.27100
O	-3.11900	9.37900	-24.89900
H	-3.98100	9.67700	-24.60700
H	-3.09300	9.59600	-25.83100
O	-6.30300	17.92300	-28.49200
H	-5.83900	18.20200	-29.28100
H	-6.56800	17.02100	-28.67400
O	-9.41100	17.47000	-25.55400
H	-8.84100	17.89900	-26.19200
H	-8.82000	17.18800	-24.85500
O	-7.36600	14.04500	-31.68300
H	-6.66400	14.49700	-31.21500
H	-7.69800	13.40400	-31.05500
O	-8.91800	12.63600	-17.30800
H	-9.54800	13.31800	-17.07400
H	-9.02500	12.52500	-18.25200
O	-13.62400	12.30400	-26.38100
H	-14.41400	12.55900	-26.85700
H	-13.13300	13.11800	-26.27600
O	0.39300	19.64400	-23.63100
H	1.21400	19.24200	-23.91200
H	0.38900	19.54100	-22.67900
O	-11.67200	12.66800	-29.42300
H	-12.46900	12.78000	-28.90400
H	-11.90000	13.00700	-30.28900
O	-5.84200	19.19300	-21.47400
H	-5.77700	19.65500	-20.63900
H	-6.55100	18.56200	-21.34600
O	-15.34000	12.55700	-23.95000
H	-15.08900	13.44100	-23.68400
H	-14.80400	12.37700	-24.72200
O	8.48300	15.98800	-27.28200
H	7.73000	15.41900	-27.43800
H	8.61800	15.95400	-26.33500
O	-6.12700	20.85900	-31.60700
H	-6.26200	21.70500	-31.17800
H	-5.58600	21.06100	-32.37000
O	-9.64000	6.93900	-14.94500
H	-10.03400	6.38500	-14.27000
H	-8.82400	7.25000	-14.55400
O	-12.67400	18.37700	-31.03400
H	-12.16200	19.17100	-30.88300
H	-12.02200	17.68000	-31.10000
O	-10.86500	21.49400	-28.95100
H	-10.68800	22.41500	-29.13900
H	-10.06900	21.17900	-28.52200
O	-6.72300	8.35400	-17.35300
H	-5.98300	8.95500	-17.43300
H	-6.49200	7.61200	-17.91100
O	-11.57900	13.54000	-19.07500
H	-11.69400	12.63900	-18.77500
H	-12.43500	13.78600	-19.42700
O	-4.50000	21.48200	-17.47700
H	-4.94400	20.69500	-17.79400
H	-3.83800	21.67000	-18.14200
O	-9.74100	23.01100	-22.95000
H	-9.19100	22.88300	-23.72300
H	-10.58000	23.31100	-23.30100
O	-2.40200	21.76200	-19.44800
H	-2.42000	22.58300	-19.94000
H	-1.48000	21.63700	-19.22300
O	-5.94500	19.27600	-18.60600
H	-5.41200	18.51500	-18.83600
H	-6.47300	18.98400	-17.86400
O	-2.77800	4.38200	-23.07700
H	-3.12100	5.27300	-23.15300
H	-2.41000	4.19300	-23.94000
O	-14.70900	16.70600	-30.25800
H	-13.87200	17.11600	-30.47400
H	-14.53100	15.76600	-30.28800
O	-2.81300	14.37300	-34.56900
H	-3.32800	15.14400	-34.33100
H	-3.08500	13.70000	-33.94600
O	-5.22800	9.21800	-29.51100
H	-6.08400	8.82900	-29.69000
H	-4.81500	9.29700	-30.37100
O	-3.69800	6.75300	-25.11200
H	-4.20200	6.71600	-25.92500
H	-3.40500	7.66200	-25.05200
O	-7.45400	6.83700	-26.10500
H	-6.58900	6.64100	-26.46500
H	-7.38600	6.60800	-25.17800

O	2.16100	17.12700	-32.63400
H	1.38400	16.78800	-33.07900
H	2.84000	17.13200	-33.30900
O	-3.02400	12.24600	-32.74300
H	-3.32900	12.56100	-31.89200
H	-2.07100	12.32400	-32.70100
O	-4.57900	18.40500	-31.09400
H	-5.10700	17.67000	-31.40700
H	-5.08100	19.18300	-31.34000
O	0.40000	16.60000	-25.97700
H	-0.25800	17.29000	-25.88900
H	1.20400	16.99000	-25.63400
O	-9.84800	9.06700	-28.73300
H	-9.27300	8.84500	-29.46600
H	-9.26000	9.13600	-27.98100
O	-0.79000	14.95400	-27.92700
H	-0.27600	15.58700	-27.42500
H	-0.14000	14.35500	-28.29300
O	-9.52100	11.09700	-34.71400
H	-10.35700	11.43500	-34.39300
H	-9.66000	10.96200	-35.65100
O	-7.54500	5.94100	-30.00600
H	-8.19900	5.62600	-30.63000
H	-7.58700	5.31900	-29.28000
O	-6.43400	16.22300	-38.09100
H	-7.34100	15.99200	-37.89200
H	-5.93700	15.42700	-37.90300
O	-3.52700	24.93300	-35.17100
H	-4.20100	25.23600	-34.56200
H	-3.14100	25.73600	-35.52000
O	-4.31200	12.42900	-25.68100
H	-5.22900	12.65100	-25.83900
H	-3.89600	12.50400	-26.54000
O	-3.52800	13.82800	-30.55100
H	-2.78100	14.34100	-30.86000
H	-3.25900	13.50700	-29.69100
O	-5.25500	16.32400	-33.56800
H	-6.16400	16.04000	-33.47100
H	-5.22600	16.74400	-34.42800
O	-0.02300	19.93500	-29.79900
H	-0.47700	19.09400	-29.74300
H	-0.65400	20.57100	-29.46200
O	-1.08000	10.86400	-28.56900
H	-1.81800	10.35200	-28.23800
H	-0.82700	10.42400	-29.38100
O	-1.58000	21.77300	-28.86400
H	-1.28800	22.33300	-29.58300
H	-1.07000	22.06500	-28.10900
O	-0.97900	3.64200	-27.97500
H	-0.67300	2.78200	-28.26200
H	-0.25400	4.23400	-28.17500
O	-14.68700	14.04500	-29.58700
H	-14.88400	13.45200	-30.31200
H	-15.24200	13.74000	-28.86900
O	-6.70700	13.93700	-27.46700
H	-6.65200	12.98500	-27.54300
H	-7.51800	14.09300	-26.98300
O	-5.55800	17.86500	-36.22900
H	-5.99600	17.14900	-36.68900
H	-4.81700	18.09300	-36.79100
O	-8.76800	12.55500	-29.70800
H	-9.58100	12.85400	-29.30200
H	-8.27200	12.16000	-28.99100
O	-6.61700	22.55100	-27.77500
H	-6.65900	22.77700	-28.70400
H	-5.70100	22.31700	-27.62600
O	-3.17000	13.01900	-20.87700
H	-2.81500	13.24600	-20.01700
H	-2.52300	13.35100	-21.49900
O	2.56000	16.14100	-30.17200
H	2.42100	16.33100	-31.10000
H	2.20300	16.90300	-29.71500
O	-9.27600	10.12700	-24.09200
H	-8.63600	9.98600	-24.79000
H	-10.10800	9.84000	-24.46800
O	2.58600	18.20900	-25.16100
H	3.02000	18.11600	-24.31200
H	3.30000	18.18400	-25.79900
O	-8.50000	19.42700	-27.97900
H	-7.70600	18.93200	-28.18000
H	-9.21100	18.89900	-28.34200
O	-3.68100	21.80900	-23.62700
H	-3.62700	22.37800	-22.86000
H	-4.54900	21.97900	-23.99200
O	-12.19200	13.57100	-31.90300
H	-13.12200	13.51000	-31.68600
H	-12.09600	13.04900	-32.69900
O	-12.27300	10.09700	-29.31500
H	-12.01300	11.01100	-29.42400
H	-11.45200	9.62900	-29.16500
O	-10.80100	11.51100	-22.14500
H	-10.12900	11.30200	-22.79400
H	-11.62800	11.31300	-22.58400
O	3.44800	18.25100	-22.62700
H	3.64200	19.15600	-22.38100
H	3.34800	17.79200	-21.79400
O	-4.89800	8.13700	-20.12800
H	-5.54600	8.83600	-20.22200
H	-4.12800	8.57000	-19.76100
O	-1.49000	18.40200	-17.05300
H	-1.64200	18.17300	-17.97000
H	-1.50000	19.35900	-17.04000
O	-4.72200	15.97500	-22.06900
H	-3.79300	16.12600	-22.24200
H	-5.14300	16.06300	-22.92400
O	-5.92300	20.00000	-24.29900
H	-6.06200	19.63200	-23.42600
H	-6.71200	20.51500	-24.47000
O	-5.32800	25.10100	-32.95600
H	-5.15300	24.63000	-32.14200
H	-6.01500	25.72700	-32.72800
O	-4.37300	19.37800	-26.91000
H	-4.70800	19.33800	-26.01400
H	-4.87200	18.71300	-27.38300
O	-10.39800	14.78500	-21.38500
H	-10.53600	14.05300	-20.78400
H	-9.48800	14.69600	-21.66600
O	-1.43700	16.72400	-36.63600

H	-2.05400	15.99800	-36.73300
H	-1.90500	17.48500	-36.98000
O	-7.77000	14.49700	-21.89600
H	-7.06000	13.85600	-21.93800
H	-7.81300	14.74800	-20.97400
O	0.21900	21.43400	-18.97600
H	0.16000	20.74900	-19.64300
H	0.62100	22.17500	-19.42800
O	-9.05700	11.48300	-20.07700
H	-9.59300	11.03200	-19.42500
H	-9.64500	11.61800	-20.82000
O	-10.76200	28.25000	-26.18200
H	-10.52200	28.88200	-25.50400
H	-11.60500	27.89900	-25.89600
O	-9.64700	10.94200	-37.51400
H	-10.52400	10.82100	-37.87800
H	-9.17100	10.15100	-37.76600
O	0.22100	19.39700	-20.74400
H	-0.66700	19.06000	-20.62200
H	0.79000	18.66600	-20.50700
O	-18.12700	15.73000	-27.42000
H	-17.43900	15.41600	-28.00800
H	-18.28200	16.63400	-27.69300
O	-13.32300	10.61900	-22.99100
H	-13.40700	10.01100	-23.72600
H	-14.14500	11.11000	-22.99100
O	-6.24900	24.05800	-23.77800
H	-6.32200	24.86300	-23.26600
H	-6.69200	24.25700	-24.60300
O	-6.46000	23.29500	-30.26800
H	-7.24400	23.83700	-30.17400
H	-5.76100	23.81200	-29.86800
O	-3.15100	9.10400	-27.62400
H	-2.65400	8.35100	-27.94400
H	-3.92200	9.14100	-28.19000
O	4.70200	14.80800	-25.98300
H	4.69100	15.69100	-26.35300
H	3.83400	14.45500	-26.17700
O	-3.79000	23.95600	-21.62200
H	-3.07700	24.59100	-21.69500
H	-4.28000	24.05300	-22.43800
O	-2.06800	17.86100	-30.05100
H	-3.00200	17.98200	-30.22000
H	-1.89000	16.95900	-30.31500
O	-6.56700	11.24600	-27.89700
H	-6.93700	10.69300	-27.20900
H	-6.07100	10.64200	-28.45000
O	1.32000	13.70300	-29.51600
H	1.78600	14.53600	-29.59000
H	1.77000	13.23800	-28.81100
O	-8.73800	21.70600	-20.80200
H	-8.03700	22.33900	-20.64900
H	-9.20900	22.04700	-21.56200
O	-0.61300	20.04800	-32.51700
H	-0.33700	19.93300	-31.60800
H	-1.48200	19.64900	-32.55600
O	-0.70900	6.07100	-25.47700
H	-1.62300	6.30200	-25.30800
H	-0.28000	6.15500	-24.62500
O	-0.93800	14.26000	-22.97700
H	-1.59100	14.50800	-23.63100
H	-0.56000	13.44700	-23.31000
O	-3.97700	24.29600	-28.87400
H	-3.79500	23.47800	-28.41100
H	-3.15400	24.51300	-29.31100
O	-3.64300	23.22300	-31.61200
H	-3.11400	22.71200	-32.22500
H	-3.38000	22.90700	-30.74800
O	-4.88900	15.00200	-16.49000
H	-5.56500	14.90200	-15.82000
H	-4.06700	14.84600	-16.02600
O	-13.14200	21.23900	-23.86900
H	-12.55600	20.49400	-23.73600
H	-13.33900	21.55000	-22.98500
O	-10.28800	24.27500	-28.85200
H	-10.95700	24.75900	-29.33600
H	-10.42600	24.52100	-27.93800
O	-18.55000	13.81000	-25.14800
H	-18.51100	13.27600	-24.35500
H	-19.05600	13.28300	-25.76700
O	-6.14700	26.30900	-22.25400
H	-5.59500	26.47200	-21.48900
H	-5.80700	26.89900	-22.92600
O	-11.17100	8.65600	-21.65200
H	-11.10700	9.51800	-22.06400
H	-11.23400	8.04400	-22.38500
O	-11.69500	10.72900	-18.00500
H	-11.06800	10.34600	-17.39100
H	-12.55300	10.51000	-17.63900
O	-1.29600	21.13500	-16.39900
H	-0.75200	21.74800	-16.89400
H	-1.87700	21.69300	-15.88300
O	-14.91900	12.53600	-32.07300
H	-14.62400	11.62700	-32.12600
H	-15.82000	12.51500	-32.39500
O	-3.01000	24.25700	-25.26800
H	-3.13500	23.45600	-25.77700
H	-2.35200	24.75200	-25.75600
O	-11.38900	10.61200	-26.09000
H	-12.11100	11.22600	-26.22900
H	-10.60000	11.14800	-26.16800
O	-0.94700	22.05800	-23.64000
H	-1.89400	21.93900	-23.71200
H	-0.59900	21.17000	-23.55700
O	-8.58700	7.94700	-19.81600
H	-8.06300	8.19400	-19.05300
H	-8.80600	8.77900	-20.23600
O	-13.19000	10.21800	-31.94900
H	-13.51900	9.33800	-32.13100
H	-12.83600	10.16300	-31.06100
O	-3.94200	21.85300	-27.43900
H	-4.09000	20.92500	-27.25900
H	-3.11700	21.87500	-27.92400
O	-10.07600	10.50500	-31.58100
H	-9.73600	11.03400	-32.30300
H	-9.62900	10.84300	-30.80500
O	-0.33900	22.67600	-26.35300
H	0.58600	22.47000	-26.49000

H	-0.50700	22.42800	-25.44400
O	-7.38200	8.88900	-22.13100
H	-7.72200	8.09000	-21.72900
H	-8.08800	9.18900	-22.70300
O	-6.73000	5.97400	-23.67800
H	-5.90700	5.50000	-23.80000
H	-6.48600	6.75300	-23.17800
O	-8.08900	9.00300	-33.38200
H	-7.36800	9.38100	-33.88600
H	-8.71300	9.72100	-33.27900
O	-3.94600	9.70700	-32.44600
H	-3.73000	10.59000	-32.74700
H	-4.42700	9.31600	-33.17600
O	-9.20600	12.66600	-25.86900
H	-9.35200	12.98000	-24.97700
H	-9.68400	13.28400	-26.42200
O	-7.34600	9.65600	-25.82100
H	-7.36000	8.76000	-26.15800
H	-6.62900	9.66300	-25.18700
O	-5.49400	10.09000	-23.77600
H	-5.75200	11.01100	-23.77700
H	-6.02500	9.69300	-23.08600
O	-0.54900	23.19500	-30.99700
H	-0.37600	22.62900	-31.74900
H	-0.05600	23.99500	-31.17900
O	-14.73200	15.20600	-22.93000
H	-15.27600	15.98200	-22.79800
H	-13.85400	15.55300	-23.08800
O	-7.94300	8.53700	-30.55200
H	-7.82100	7.60900	-30.35300
H	-8.23300	8.55100	-31.46400
O	-5.07400	6.22300	-27.41600
H	-5.05100	6.28700	-28.37000
H	-4.65700	5.38300	-27.22200
O	5.30500	15.95800	-29.37800
H	5.74800	15.20900	-29.77600
H	4.42700	15.94900	-29.76000
O	0.43800	22.51100	-33.89700
H	1.13300	23.13200	-33.67800
H	0.78100	21.66100	-33.62200
O	-13.94500	14.33300	-20.03700
H	-14.68700	14.48100	-19.45100
H	-14.26600	14.59400	-20.90100
O	2.33700	19.96100	-32.36800
H	2.86300	20.30000	-33.09300
H	2.25000	19.02600	-32.55200
O	-6.00100	23.87500	-20.23900
H	-5.69200	23.63000	-19.36700
H	-5.20300	24.07900	-20.72700
O	-5.97900	26.02900	-28.00900
H	-6.41800	25.53600	-27.31500
H	-5.22800	25.48600	-28.25000
O	-13.49900	22.22000	-28.00600
H	-12.67300	21.92000	-28.38600
H	-13.85000	21.45000	-27.55700
O	-8.53100	21.57200	-25.93700
H	-9.15300	20.90900	-26.23600
H	-7.86100	21.59800	-26.61900
O	-15.29800	20.13800	-27.04700
H	-16.13500	19.71100	-27.22900
H	-15.32600	20.33200	-26.11000
O	-8.45900	10.05100	-16.34300
H	-8.41000	10.92700	-16.72500
H	-7.91600	9.50800	-16.91600
O	2.02700	20.59500	-17.06300
H	1.23600	20.57700	-17.60300
H	1.79000	21.13300	-16.30800
O	2.14700	17.44400	-19.75000
H	2.19600	17.27900	-18.80800
H	2.03800	16.57700	-20.14000
O	-8.97000	20.12300	-32.46100
H	-8.82100	20.80600	-33.11400
H	-8.16300	20.10300	-31.94600
O	-0.56300	13.42700	-18.43400
H	0.33800	13.28800	-18.72600
H	-0.48800	13.59200	-17.49400
O	-6.22900	10.80400	-19.58900
H	-7.09800	11.09700	-19.86000
H	-5.99300	11.38800	-18.86800
O	3.64800	19.86400	-19.50600
H	3.35700	20.22700	-18.66900
H	3.03600	19.15000	-19.68400
O	4.84800	17.42900	-26.98500
H	5.68300	17.86800	-26.82200
H	4.98400	16.95300	-27.80500
O	1.89700	14.98700	-21.03800
H	2.37300	15.18000	-21.84600
H	0.97400	15.04300	-21.28300
O	-11.21500	16.14900	-31.45500
H	-11.46000	15.24500	-31.25700
H	-11.04500	16.15100	-32.39700
O	-3.52500	7.02200	-22.07200
H	-3.00700	7.75800	-22.39700
H	-3.87900	7.32800	-21.23700
O	-11.94500	11.88200	-33.95600
H	-12.63800	11.95700	-34.61200
H	-12.19000	11.11800	-33.43400
O	-3.27400	3.44200	-26.55200
H	-2.99300	2.77400	-25.92600
H	-2.48900	3.63500	-27.06500
O	-5.47000	22.61800	-34.98900
H	-6.04300	23.23700	-35.44200
H	-4.89700	23.16700	-34.45600
O	0.59700	11.86300	-22.80500
H	1.54900	11.96300	-22.78400
H	0.42800	11.07100	-22.29500
O	-1.38100	8.94900	-22.61900
H	-1.97600	9.22900	-23.31400
H	-1.66600	9.43800	-21.84700
O	-2.25100	22.90100	-33.85900
H	-1.31700	22.83600	-33.65500
H	-2.33800	23.73700	-34.31700
O	0.37800	22.21800	-36.89800
H	0.30000	21.88400	-36.00500
H	1.19900	22.71000	-36.89900
O	3.07600	15.08400	-23.69100
H	3.04400	14.14600	-23.50500
H	3.68600	15.16200	-24.42400

O	-5.08600	13.95400	-37.64800
H	-4.14000	13.82400	-37.57900
H	-5.46400	13.21500	-37.17100
O	3.22600	11.87500	-23.64500
H	2.70900	11.09100	-23.83200
H	4.11000	11.54700	-23.48000
O	0.88700	5.61900	-27.68500
H	0.68900	5.31100	-26.80100
H	1.49700	6.34500	-27.55500
O	8.89400	16.14600	-24.55000
H	9.62400	15.88400	-23.98900
H	8.28400	15.40800	-24.51000
O	6.97500	14.28600	-23.95300
H	6.24900	14.15200	-24.56100
H	6.56000	14.59400	-23.14700
O	-5.20200	18.00500	-16.18800
H	-4.28100	18.18300	-15.99400
H	-5.23700	17.06100	-16.34000
O	-6.90100	11.87900	-37.71200
H	-7.80800	11.58200	-37.63600
H	-6.62400	11.58200	-38.57900
O	-15.98300	13.19900	-27.26200
H	-16.30300	12.34600	-27.55500
H	-16.53000	13.41400	-26.50700
O	-12.20500	15.91600	-23.54600
H	-11.98700	16.84700	-23.52700
H	-11.79300	15.55600	-22.76000
O	-5.28500	6.42200	-31.55800
H	-6.09500	6.07700	-31.18200
H	-4.59700	6.11300	-30.96900
O	-3.35600	5.50000	-29.93600
H	-2.80300	5.92000	-29.27700
H	-3.11900	4.57400	-29.89300
O	-7.33300	11.48900	-14.15500
H	-7.43300	12.43800	-14.22400
H	-7.55100	11.16100	-15.02800
O	-8.13100	8.73700	-37.57100
H	-7.26900	9.06300	-37.31200
H	-7.94600	7.95000	-38.08400
O	-16.11500	16.82000	-25.29300
H	-16.80800	16.40500	-25.80600
H	-15.36700	16.86200	-25.89000
O	0.02200	7.61000	-30.42800
H	-0.55800	8.26900	-30.81000
H	-0.24500	6.78800	-30.83900
O	-1.52800	7.01100	-28.22600
H	-1.02800	7.12200	-29.03400
H	-0.88400	6.70700	-27.58500
O	-7.47000	14.91900	-15.33900
H	-7.95300	15.71900	-15.13200
H	-7.38300	14.92900	-16.29200
O	-14.20100	16.75100	-27.41700
H	-14.22400	16.85000	-28.36800
H	-13.89100	17.59800	-27.09500
O	-11.54200	18.80900	-23.30200
H	-10.58800	18.77700	-23.36900
H	-11.74100	18.34800	-22.48700
O	-10.95200	17.37900	-20.73500
H	-10.13300	17.87400	-20.71100
H	-10.67900	16.46200	-20.76300
O	-1.19600	9.77000	-31.37200
H	-2.08300	10.05500	-31.59000
H	-0.78000	9.61000	-32.21900
O	2.15600	9.79500	-27.65700
H	2.93200	10.28400	-27.38400
H	1.43100	10.22100	-27.19900
O	4.59300	10.50800	-26.59900
H	5.18800	10.26700	-27.31000
H	4.66600	9.78800	-25.97200
O	-13.18700	24.35100	-26.28500
H	-13.32200	23.59100	-26.85100
H	-13.60400	25.07500	-26.75200
O	-2.05100	19.53500	-37.27700
H	-1.34800	19.74000	-36.66100
H	-2.77500	20.10200	-37.01200
O	-1.22200	15.28600	-31.15700
H	-1.27800	15.74200	-31.99600
H	-0.66900	14.52500	-31.33500
O	2.58900	11.07500	-30.33200
H	2.33300	10.67400	-29.50100
H	1.96400	11.78900	-30.45700
O	6.90000	13.81400	-27.82500
H	6.34000	13.88400	-27.05200
H	6.29500	13.63800	-28.54500
O	-10.03000	25.24800	-26.07300
H	-10.14700	26.19200	-26.17500
H	-10.85100	24.94500	-25.68400
O	-0.14300	19.97300	-35.22700
H	0.69200	19.54100	-35.41000
H	-0.30400	19.80100	-34.30000
O	-0.48800	16.47600	-33.86200
H	-0.54300	16.31100	-34.80300
H	-1.11300	17.18400	-33.70600
O	-15.69900	11.71300	-20.03600
H	-15.05300	11.20300	-19.54700
H	-15.90700	12.45000	-19.46100
O	-3.17300	18.78100	-14.53600
H	-2.30400	18.95500	-14.89800
H	-3.65300	19.60000	-14.65900
O	-7.17800	5.97700	-34.28300
H	-7.19100	6.92100	-34.12600
H	-6.28600	5.79400	-34.57700
O	-9.00500	25.90100	-22.85700
H	-8.10200	25.93300	-22.54100
H	-9.23100	24.97100	-22.84300
O	-7.24900	24.65400	-26.19600
H	-8.20000	24.65400	-26.31000
H	-6.94600	23.90700	-26.71300
O	-3.36300	2.57100	-30.02900
H	-4.13000	2.01700	-29.88300
H	-2.95200	2.64300	-29.16700
O	-11.35900	8.86000	-15.93800
H	-10.49600	8.50300	-15.72800
H	-11.93300	8.52100	-15.25100
O	8.48600	30.49000	-25.13200
H	9.12400	30.37100	-25.83600
H	8.36800	31.43800	-25.07300
O	5.29500	30.44300	-25.19600

H	4.95700	30.32700	-26.08400
H	6.16400	30.04200	-25.21600
O	1.15300	32.29600	-29.20600
H	0.59000	32.20500	-29.97400
H	1.03900	31.47600	-28.72600
O	5.77700	31.59600	-19.23700
H	5.76600	31.20100	-20.10900
H	5.93900	30.86300	-18.64400
O	-1.30000	33.79100	-26.36800
H	-0.50800	33.45000	-26.78300
H	-1.06000	33.90100	-25.44700
O	2.49700	34.38000	-20.28900
H	2.20600	35.28400	-20.40500
H	3.13300	34.42100	-19.57500
O	3.42300	31.75700	-23.69900
H	4.01900	31.53300	-24.41300
H	3.48100	31.01600	-23.09700
O	8.04000	28.21500	-28.05700
H	8.82600	27.79700	-28.40800
H	8.30400	29.12000	-27.88800
O	13.44100	28.15300	-27.15300
H	14.17300	27.58200	-26.91900
H	12.68500	27.76500	-26.71300
O	5.05800	30.57500	-29.95700
H	4.69400	30.04800	-29.24600
H	5.91500	30.18500	-30.13000
O	10.96300	21.86500	-22.69400
H	10.63200	22.73400	-22.91800
H	10.36800	21.54800	-22.01400
O	7.45600	34.06600	-23.29400
H	7.15700	34.96800	-23.40300
H	7.11200	33.79900	-22.44200
O	0.90800	30.51700	-27.26100
H	-0.01700	30.38500	-27.05300
H	1.15600	31.29700	-26.76400
O	10.85600	26.58400	-25.61400
H	9.94800	26.51600	-25.90900
H	10.80500	27.06100	-24.78600
O	4.67800	28.06000	-33.97600
H	4.55800	28.50600	-33.13800
H	3.98300	28.40900	-34.53500
O	-1.73100	30.16100	-26.42600
H	-1.60200	30.41400	-25.51200
H	-2.41200	30.75300	-26.74600
O	4.93000	28.00300	-17.67100
H	4.15400	28.56300	-17.68100
H	5.56900	28.48900	-17.14900
O	12.72200	24.78600	-23.83700
H	12.08000	25.07900	-24.48300
H	12.21800	24.67100	-23.03100
O	3.18500	30.81200	-18.10900
H	3.92600	31.35000	-18.38600
H	2.47900	31.43800	-17.95200
O	8.66000	32.95500	-19.51200
H	9.06900	32.51300	-20.25700
H	7.93800	32.38000	-19.25900
O	5.01400	27.89200	-22.13900
H	5.86000	28.31600	-21.99500
H	4.95300	27.23500	-21.44500
O	8.98300	33.51500	-25.75700
H	8.35400	33.67900	-26.45900
H	8.59600	33.92900	-24.98600
O	8.16300	25.55200	-20.48000
H	7.92000	26.30700	-21.01700
H	8.43900	25.93100	-19.64600
O	10.11700	32.09600	-21.80300
H	10.32900	32.73900	-22.47900
H	10.07000	31.26200	-22.27100
O	7.72000	24.70800	-24.89900
H	6.85800	25.00600	-24.60700
H	7.74600	24.92500	-25.83100
O	4.53600	33.25200	-28.49200
H	5.00000	33.53100	-29.28100
H	4.27200	32.35100	-28.67400
O	1.42900	32.79900	-25.55400
H	1.99900	33.22800	-26.19200
H	2.01900	32.51800	-24.85500
O	3.47300	29.37400	-31.68300
H	4.17500	29.82700	-31.21500
H	3.14100	28.73300	-31.05500
O	1.92100	27.96600	-17.30800
H	1.29100	28.64700	-17.07400
H	1.81500	27.85400	-18.25200
O	-2.78400	27.63300	-26.38100
H	-3.57500	27.88900	-26.85700
H	-2.29300	28.44800	-26.27600
O	11.23200	34.97300	-23.63100
H	12.05400	34.57100	-23.91200
H	11.22800	34.87000	-22.67900
O	-0.83300	27.99700	-29.42300
H	-1.63000	28.10900	-28.90400
H	-1.06000	28.33600	-30.28900
O	4.99800	34.52200	-21.47400
H	5.06200	34.98400	-20.63900
H	4.28900	33.89200	-21.34600
O	-4.50100	27.88600	-23.95000
H	-4.25000	28.77100	-23.68400
H	-3.96500	27.70600	-24.72200
O	1.19900	22.26800	-14.94500
H	0.80600	21.71400	-14.27000
H	2.01600	22.57900	-14.55400
O	-1.83500	33.70600	-31.03400
H	-1.32200	34.50000	-30.88300
H	-1.18200	33.00900	-31.10000
O	-0.02500	36.82300	-28.95100
H	0.15100	37.74500	-29.13900
H	0.77000	36.50900	-28.52200
O	4.11600	23.68400	-17.35300
H	4.85700	24.28400	-17.43300
H	4.34800	22.94100	-17.91100
O	-0.74000	28.86900	-19.07500
H	-0.85500	27.96800	-18.77500
H	-1.59600	29.11600	-19.42700
O	6.33900	36.81100	-17.47700
H	5.89600	36.02500	-17.79400
H	7.00100	36.99900	-18.14200
O	1.09800	38.34100	-22.95000
H	1.64900	38.21200	-23.72300

H	0.25900	38.64000	-23.30100
O	8.43700	37.09100	-19.44800
H	8.41900	37.91200	-19.94000
H	9.35900	36.96700	-19.22300
O	4.89500	34.60600	-18.60600
H	5.42700	33.84400	-18.83600
H	4.36600	34.31300	-17.86400
O	8.06200	19.71200	-23.07700
H	7.71800	20.60200	-23.15300
H	8.43000	19.52200	-23.94000
O	-3.87000	32.03500	-30.25800
H	-3.03200	32.44500	-30.47400
H	-3.69200	31.09500	-30.28800
O	5.61100	24.54700	-29.51100
H	4.75600	24.15800	-29.69000
H	6.02400	24.62700	-30.37100
O	7.14100	22.08200	-25.11200
H	6.63800	22.04500	-25.92500
H	7.43400	22.99100	-25.05200
O	3.38600	22.16600	-26.10500
H	4.25100	21.97000	-26.46500
H	3.45300	21.93700	-25.17800
O	7.81500	27.57500	-32.74300
H	7.51000	27.89000	-31.89200
H	8.76800	27.65300	-32.70100
O	11.23900	31.92900	-25.97700
H	10.58200	32.61900	-25.88900
H	12.04300	32.31900	-25.63400
O	0.99200	24.39600	-28.73300
H	1.56600	24.17400	-29.46600
H	1.57900	24.46500	-27.98100
O	10.05000	30.28400	-27.92700
H	10.56300	30.91700	-27.42500
H	10.70000	29.68400	-28.29300
O	1.31900	26.42700	-34.71400
H	0.48200	26.76400	-34.39300
H	1.17900	26.29200	-35.65100
O	3.29400	21.27000	-30.00600
H	2.64100	20.95500	-30.63000
H	3.25200	20.64800	-29.28000
O	6.52800	27.75900	-25.68100
H	5.61000	27.98000	-25.83900
H	6.94400	27.83300	-26.54000
O	7.31100	29.15800	-30.55100
H	8.05900	29.67000	-30.86000
H	7.58000	28.83600	-29.69100
O	9.75900	26.19300	-28.56900
H	9.02100	25.68100	-28.23800
H	10.01200	25.75300	-29.38100
O	9.86000	18.97100	-27.97500
H	10.16600	18.11100	-28.26200
H	10.58600	19.56300	-28.17500
O	-3.84700	29.37400	-29.58700
H	-4.04500	28.78100	-30.31200
H	-4.40300	29.06900	-28.86900
O	4.13200	29.26600	-27.46700
H	4.18700	28.31400	-27.54300
H	3.32200	29.42200	-26.98300
O	2.07200	27.88400	-29.70800
H	1.25900	28.18400	-29.30200
H	2.56800	27.49000	-28.99100
O	4.22300	37.88000	-27.77500
H	4.18000	38.10600	-28.70400
H	5.13900	37.64600	-27.62600
O	7.66900	28.34800	-20.87700
H	8.02400	28.57500	-20.01700
H	8.31600	28.68000	-21.49900
O	1.56300	25.45600	-24.09200
H	2.20300	25.31500	-24.79000
H	0.73100	25.16900	-24.46800
O	13.42600	33.53800	-25.16100
H	13.86000	33.44500	-24.31200
H	14.13900	33.51400	-25.79900
O	2.33900	34.75600	-27.97900
H	3.13300	34.26100	-28.18000
H	1.62800	34.22800	-28.34200
O	7.15900	37.13800	-23.62700
H	7.21200	37.70700	-22.86000
H	6.29000	37.30800	-23.99200
O	-1.35300	28.90000	-31.90300
H	-2.28300	28.83900	-31.68600
H	-1.25700	28.37800	-32.69900
O	-1.43400	25.42600	-29.31500
H	-1.17300	26.34000	-29.42400
H	-0.61200	24.95800	-29.16500
O	0.03800	26.84000	-22.14500
H	0.71100	26.63100	-22.79400
H	-0.78900	26.64200	-22.58400
O	14.28700	33.58000	-22.62700
H	14.48100	34.48500	-22.38100
H	14.18700	33.12100	-21.79400
O	5.94100	23.46600	-20.12800
H	5.29400	24.16500	-20.22200
H	6.71100	23.90000	-19.76100
O	9.34900	33.73100	-17.05300
H	9.19700	33.50200	-17.97000
H	9.33900	34.68800	-17.04000
O	6.11700	31.30400	-22.06900
H	7.04700	31.45500	-22.24200
H	5.69700	31.39200	-22.92400
O	4.91600	35.32900	-24.29900
H	4.77700	34.96100	-23.42600
H	4.12800	35.84400	-24.47000
O	6.46700	34.70700	-26.91000
H	6.13200	34.66700	-26.01400
H	5.96700	34.04200	-27.38300
O	0.44100	30.11400	-21.38500
H	0.30300	29.38200	-20.78400
H	1.35200	30.02500	-21.66600
O	3.06900	29.82600	-21.89600
H	3.77900	29.18500	-21.93800
H	3.02600	30.07700	-20.97400
O	11.05800	36.76300	-18.97600
H	10.99900	36.07900	-19.64300
H	11.46000	37.50500	-19.42800
O	1.78200	26.81300	-20.07700
H	1.24600	26.36200	-19.42500
H	1.19400	26.94700	-20.82000

O	0.07700	43.57900	-26.18200
H	0.31700	44.21100	-25.50400
H	-0.76600	43.22800	-25.89600
O	11.06000	34.72700	-20.74400
H	10.17300	34.38900	-20.62200
H	11.63000	33.99500	-20.50700
O	-7.28700	31.05900	-27.42000
H	-6.60000	30.74500	-28.00800
H	-7.44200	31.96300	-27.69300
O	-2.48400	25.94800	-22.99100
H	-2.56800	25.34100	-23.72600
H	-3.30500	26.43900	-22.99100
O	4.59000	39.38700	-23.77800
H	4.51700	40.19200	-23.26600
H	4.14800	39.58600	-24.60300
O	7.68900	24.43300	-27.62400
H	8.18600	23.68000	-27.94400
H	6.91700	24.47000	-28.19000
O	15.54200	30.13800	-25.98300
H	15.53100	31.02000	-26.35300
H	14.67300	29.78500	-26.17700
O	7.05000	39.28600	-21.62200
H	7.76300	39.92000	-21.69500
H	6.55900	39.38200	-22.43800
O	4.27200	26.57500	-27.89700
H	3.90200	26.02200	-27.20900
H	4.76800	25.97100	-28.45000
O	12.15900	29.03200	-29.51600
H	12.62500	29.86500	-29.59000
H	12.61000	28.56700	-28.81100
O	2.10100	37.03500	-20.80200
H	2.80200	37.66900	-20.64900
H	1.63100	37.37600	-21.56200
O	10.13100	21.40000	-25.47700
H	9.21700	21.63100	-25.30800
H	10.56000	21.48400	-24.62500
O	9.90100	29.58900	-22.97700
H	9.24800	29.83700	-23.63100
H	10.27900	28.77600	-23.31000
O	5.95000	30.33100	-16.49000
H	5.27400	30.23100	-15.82000
H	6.77300	30.17500	-16.02600
O	-2.30200	36.56900	-23.86900
H	-1.71700	35.82300	-23.73600
H	-2.50000	36.87900	-22.98500
O	0.55200	39.60400	-28.85200
H	-0.11700	40.08800	-29.33600
H	0.41300	39.85100	-27.93800
O	-7.71000	29.13900	-25.14800
H	-7.67200	28.60600	-24.35500
H	-8.21600	28.61300	-25.76700
O	4.69200	41.63800	-22.25400
H	5.24400	41.80100	-21.48900
H	5.03300	42.22900	-22.92600
O	-0.33100	23.98600	-21.65200
H	-0.26700	24.84700	-22.06400
H	-0.39500	23.37300	-22.38500
O	-0.85600	26.05800	-18.00500
H	-0.22900	25.67600	-17.39100
H	-1.71300	25.83900	-17.63900
O	9.54300	36.46400	-16.39900
H	10.08700	37.07700	-16.89400
H	8.96200	37.02200	-15.88300
O	-4.08000	27.86600	-32.07300
H	-3.78400	26.95700	-32.12600
H	-4.98100	27.84400	-32.39500
O	7.82900	39.58600	-25.26800
H	7.70400	38.78500	-25.77700
H	8.48700	40.08100	-25.75600
O	-0.55000	25.94100	-26.09000
H	-1.27100	26.55500	-26.22900
H	0.23900	26.47700	-26.16800
O	9.89300	37.38700	-23.64000
H	8.94600	37.26800	-23.71200
H	10.24100	36.49900	-23.55700
O	2.25300	23.27700	-19.81600
H	2.77600	23.52300	-19.05300
H	2.03400	24.10900	-20.23600
O	-2.35100	25.54700	-31.94900
H	-2.67900	24.66700	-32.13100
H	-1.99700	25.49200	-31.06100
O	6.89700	37.18200	-27.43900
H	6.75000	36.25400	-27.25900
H	7.72300	37.20400	-27.92400
O	0.76300	25.83400	-31.58100
H	1.10300	26.36300	-32.30300
H	1.21100	26.17200	-30.80500
O	3.45800	24.21800	-22.13100
H	3.11700	23.41900	-21.72900
H	2.75200	24.51800	-22.70300
O	4.10900	21.30300	-23.67800
H	4.93200	20.82900	-23.80000
H	4.35300	22.08200	-23.17800
O	2.75000	24.33200	-33.38200
H	3.47100	24.71000	-33.88600
H	2.12700	25.05100	-33.27900
O	6.89300	25.03600	-32.44600
H	7.11000	25.91900	-32.74700
H	6.41300	24.64500	-33.17600
O	1.63300	27.99500	-25.86900
H	1.48800	28.30900	-24.97700
H	1.15600	28.61300	-26.42200
O	3.49300	24.98500	-25.82100
H	3.47900	24.08900	-26.15800
H	4.21000	24.99200	-25.18700
O	5.34600	25.41900	-23.77600
H	5.08700	26.34100	-23.77700
H	4.81400	25.02200	-23.08600
O	-3.89200	30.53500	-22.93000
H	-4.43700	31.31100	-22.79800
H	-3.01400	30.88200	-23.08800
O	2.89700	23.86700	-30.55200
H	3.01800	22.93800	-30.35300
H	2.60700	23.88000	-31.46400
O	5.76600	21.55200	-27.41600
H	5.78800	21.61600	-28.37000
H	6.18300	20.71200	-27.22200
O	-3.10600	29.66200	-20.03700

H	-3.84700	29.81100	-19.45100
H	-3.42600	29.92300	-20.90100
O	4.83900	39.20400	-20.23900
H	5.14800	38.95900	-19.36700
H	5.63600	39.40900	-20.72700
O	-2.66000	37.54900	-28.00600
H	-1.83400	37.24900	-28.38600
H	-3.01000	36.78000	-27.55700
O	2.30800	36.90200	-25.93700
H	1.68600	36.23900	-26.23600
H	2.97900	36.92800	-26.61900
O	-4.45900	35.46800	-27.04700
H	-5.29600	35.04000	-27.22900
H	-4.48700	35.66100	-26.11000
O	2.38100	25.38000	-16.34300
H	2.43000	26.25600	-16.72500
H	2.92300	24.83700	-16.91600
O	12.86600	35.92400	-17.06300
H	12.07600	35.90600	-17.60300
H	12.63000	36.46300	-16.30800
O	12.98600	32.77300	-19.75000
H	13.03600	32.60800	-18.80800
H	12.87700	31.90600	-20.14000
O	10.27600	28.75600	-18.43400
H	11.17700	28.61700	-18.72600
H	10.35100	28.92200	-17.49400
O	4.61100	26.13300	-19.58900
H	3.74100	26.42600	-19.86000
H	4.84700	26.71700	-18.86800
O	14.48800	35.19300	-19.50600
H	14.19600	35.55600	-18.66900
H	13.87500	34.47900	-19.68400
O	12.73700	30.31600	-21.03800
H	13.21200	30.50900	-21.84600
H	11.81300	30.37200	-21.28300
O	-0.37500	31.47800	-31.45500
H	-0.62100	30.57500	-31.25700
H	-0.20600	31.48000	-32.39700
O	7.31500	22.35100	-22.07200
H	7.83300	23.08700	-22.39700
H	6.96000	22.65700	-21.23700
O	-1.10600	27.21100	-33.95600
H	-1.79900	27.28600	-34.61200
H	-1.35000	26.44700	-33.43400
O	7.56600	18.77100	-26.55200
H	7.84600	18.10300	-25.92600
H	8.35100	18.96400	-27.06500
O	11.43700	27.19300	-22.80500
H	12.38800	27.29200	-22.78400
H	11.26700	26.40000	-22.29500
O	9.45800	24.27800	-22.61900
H	8.86300	24.55900	-23.31400
H	9.17300	24.76800	-21.84700
O	13.91500	30.41300	-23.69100
H	13.88300	29.47500	-23.50500
H	14.52600	30.49200	-24.42400
O	14.06500	27.20400	-23.64500
H	13.54900	26.42000	-23.83200
H	14.94900	26.87600	-23.48000
O	11.72600	20.94800	-27.68500
H	11.52800	20.64000	-26.80100
H	12.33600	21.67500	-27.55500
O	19.73300	31.47500	-24.55000
H	20.46300	31.21300	-23.98900
H	19.12400	30.73800	-24.51000
O	17.81500	29.61600	-23.95300
H	17.08800	29.48100	-24.56100
H	17.39900	29.92300	-23.14700
O	5.63800	33.33500	-16.18800
H	6.55800	33.51200	-15.99400
H	5.60300	32.39000	-16.34000
O	-5.14400	28.52800	-27.26200
H	-5.46400	27.67500	-27.55500
H	-5.69100	28.74300	-26.50700
O	-1.36600	31.24500	-23.54600
H	-1.14700	32.17600	-23.52700
H	-0.95400	30.88500	-22.76000
O	5.55400	21.75200	-31.55800
H	4.74400	21.40600	-31.18200
H	6.24300	21.44200	-30.96900
O	7.48300	20.82900	-29.93600
H	8.03600	21.24900	-29.27700
H	7.72000	19.90300	-29.89300
O	3.50700	26.81800	-14.15500
H	3.40600	27.76700	-14.22400
H	3.28900	26.49000	-15.02800
O	-5.27500	32.14900	-25.29300
H	-5.96900	31.73400	-25.80600
H	-4.52800	32.19100	-25.89000
O	10.86100	22.93900	-30.42800
H	10.28100	23.59800	-30.81000
H	10.59500	22.11700	-30.83900
O	9.31200	22.34000	-28.22600
H	9.81200	22.45200	-29.03400
H	9.95500	22.03600	-27.58500
O	3.37000	30.24800	-15.33900
H	2.88700	31.04800	-15.13200
H	3.45600	30.25900	-16.29200
O	-3.36200	32.08100	-27.41700
H	-3.38500	32.17900	-28.36800
H	-3.05200	32.92700	-27.09500
O	-0.70300	34.13800	-23.30200
H	0.25100	34.10700	-23.36900
H	-0.90200	33.67700	-22.48700
O	-0.11300	32.70800	-20.73500
H	0.70600	33.20300	-20.71100
H	0.16100	31.79100	-20.76300
O	9.64400	25.09900	-31.37200
H	8.75600	25.38400	-31.59000
H	10.05900	24.93900	-32.21900
O	12.99500	25.12400	-27.65700
H	13.77100	25.61400	-27.38400
H	12.27000	25.55000	-27.19900
O	15.43200	25.83700	-26.59900
H	16.02700	25.59600	-27.31000
H	15.50500	25.11700	-25.97200
O	-2.34800	39.68000	-26.28500
H	-2.48200	38.92000	-26.85100

H	-2.76500	40.40400	-26.75200
O	13.42800	26.40400	-30.33200
H	13.17200	26.00300	-29.50100
H	12.80400	27.11800	-30.45700
O	17.74000	29.14300	-27.82500
H	17.18000	29.21300	-27.05200
H	17.13400	28.96800	-28.54500
O	0.80900	40.57700	-26.07300
H	0.69200	41.52200	-26.17500
H	-0.01200	40.27400	-25.68400
O	-4.85900	27.04200	-20.03600
H	-4.21400	26.53200	-19.54700
H	-5.06800	27.77900	-19.46100
O	7.66600	34.11000	-14.53600
H	8.53500	34.28400	-14.89800
H	7.18700	34.93000	-14.65900
O	3.66100	21.30600	-34.28300
H	3.64900	22.25000	-34.12600
H	4.55300	21.12300	-34.57700
O	1.83500	41.23000	-22.85700
H	2.73700	41.26200	-22.54100
H	1.60800	40.30000	-22.84300
O	3.59000	39.98300	-26.19600
H	2.63900	39.98300	-26.31000
H	3.89300	39.23600	-26.71300
O	7.47600	17.90000	-30.02900
H	6.70900	17.34600	-29.88300
H	7.88700	17.97300	-29.16700
O	-0.52000	24.18900	-15.93800
H	0.34300	23.83300	-15.72800
H	-1.09300	23.85000	-15.25100
O	15.77000	43.33200	-17.67100
H	14.99300	43.89200	-17.68100
H	16.40900	43.81800	-17.14900
O	14.02400	46.14100	-18.10900
H	14.76600	46.67900	-18.38600
H	13.31800	46.76800	-17.95200
O	12.76000	43.29500	-17.30800
H	12.13000	43.97600	-17.07400
H	12.65400	43.18300	-18.25200
O	6.33900	43.21500	-23.95000
H	6.58900	44.10000	-23.68400
H	6.87500	43.03600	-24.72200
O	12.03800	37.59700	-14.94500
H	11.64500	37.04400	-14.27000
H	12.85500	37.90800	-14.55400
O	14.95500	39.01300	-17.35300
H	15.69600	39.61300	-17.43300
H	15.18700	38.27000	-17.91100
O	10.09900	44.19800	-19.07500
H	9.98500	43.29700	-18.77500
H	9.24400	44.44500	-19.42700
O	18.90100	35.04100	-23.07700
H	18.55700	35.93100	-23.15300
H	19.26900	34.85100	-23.94000
O	10.87800	42.16900	-22.14500
H	11.55000	41.96100	-22.79400
H	10.05100	41.97100	-22.58400
O	16.78000	38.79500	-20.12800
H	16.13300	39.49400	-20.22200
H	17.55100	39.22900	-19.76100
O	12.62200	42.14200	-20.07700
H	12.08500	41.69100	-19.42500
H	12.03300	42.27600	-20.82000
O	8.35600	41.27700	-22.99100
H	8.27100	40.67000	-23.72600
H	7.53400	41.76800	-22.99100
O	16.78900	45.66100	-16.49000
H	16.11300	45.56000	-15.82000
H	17.61200	45.50500	-16.02600
O	3.12900	44.46800	-25.14800
H	3.16700	43.93500	-24.35500
H	2.62300	43.94200	-25.76700
O	10.50800	39.31500	-21.65200
H	10.57200	40.17600	-22.06400
H	10.44500	38.70200	-22.38500
O	9.98300	41.38700	-18.00500
H	10.61000	41.00500	-17.39100
H	9.12600	41.16900	-17.63900
O	13.09200	38.60600	-19.81600
H	13.61500	38.85200	-19.05300
H	12.87300	39.43800	-20.23600
O	14.29700	39.54700	-22.13100
H	13.95700	38.74800	-21.72900
H	13.59100	39.84700	-22.70300
O	14.94900	36.63200	-23.67800
H	15.77100	36.15800	-23.80000
H	15.19300	37.41100	-23.17800
O	7.73400	44.99200	-20.03700
H	6.99200	45.14000	-19.45100
H	7.41300	45.25200	-20.90100
O	13.22000	40.70900	-16.34300
H	13.26900	41.58500	-16.72500
H	13.76200	40.16700	-16.91600
O	15.45000	41.46200	-19.58900
H	14.58000	41.75500	-19.86000
H	15.68600	42.04600	-18.86800
O	18.15400	37.68000	-22.07200
H	18.67200	38.41600	-22.39700
H	17.79900	37.98600	-21.23700
O	14.34600	42.14700	-14.15500
H	14.24600	43.09700	-14.22400
H	14.12800	41.81900	-15.02800
O	14.20900	45.57700	-15.33900
H	13.72600	46.37700	-15.13200
H	14.29500	45.58800	-16.29200
O	5.98000	42.37100	-20.03600
H	6.62600	41.86100	-19.54700
H	5.77200	43.10800	-19.46100
O	10.32000	39.51800	-15.93800
H	11.18300	39.16200	-15.72800
H	9.74600	39.17900	-15.25100
O	-19.18100	-19.27200	-33.56500
H	-19.47200	-18.36800	-33.68000
H	-18.54600	-19.23100	-32.85000
O	-18.49400	-22.84000	-31.38500
H	-17.75200	-22.30200	-31.66100
H	-19.20000	-22.21400	-31.22700

O	-13.01800	-20.69800	-32.78700
H	-12.61000	-21.13900	-33.53200
H	-13.74100	-21.27200	-32.53400
O	-10.44700	-18.67900	-36.90600
H	-9.62500	-19.08100	-37.18800
H	-10.45100	-18.78200	-35.95400
O	-15.34000	-16.84100	-30.75200
H	-15.78300	-17.62800	-31.07000
H	-14.67800	-16.65300	-31.41800
O	-13.24100	-16.56100	-32.72400
H	-13.26000	-15.74000	-33.21600
H	-12.31900	-16.68600	-32.49900
O	-16.78400	-19.04700	-31.88200
H	-16.25100	-19.80800	-32.11200
H	-17.31300	-19.33900	-31.13900
O	-7.39100	-20.07200	-35.90300
H	-7.19800	-19.16700	-35.65700
H	-7.49100	-20.53100	-35.06900
O	-12.33000	-19.92100	-30.32900
H	-12.48100	-20.15000	-31.24600
H	-12.33900	-18.96400	-30.31600
O	-10.62100	-16.88900	-32.25100
H	-10.67900	-17.57400	-32.91800
H	-10.21900	-16.14800	-32.70300
O	-10.61900	-18.92500	-34.02000
H	-11.50600	-19.26300	-33.89700
H	-10.04900	-19.65700	-33.78300
O	-14.62900	-14.36700	-34.89800
H	-13.91600	-13.73200	-34.97000
H	-15.12000	-14.27000	-35.71400
O	-19.57800	-16.61700	-34.07700
H	-18.87700	-15.98400	-33.92400
H	-20.04800	-16.27600	-34.83800
O	-15.72900	-23.32100	-29.76500
H	-16.40500	-23.42100	-29.09500
H	-14.90600	-23.47700	-29.30200
O	-16.98700	-12.01400	-35.53000
H	-16.43500	-11.85100	-34.76500
H	-16.64600	-11.42400	-36.20200
O	-12.13500	-17.18800	-29.67500
H	-11.59100	-16.57500	-30.16900
H	-12.71700	-16.63000	-29.15800
O	-11.78600	-16.26500	-36.91500
H	-12.73300	-16.38400	-36.98700
H	-11.43800	-17.15300	-36.83300
O	-16.84000	-14.44800	-33.51500
H	-16.53100	-14.69300	-32.64200
H	-16.04200	-14.24400	-34.00300
O	-8.81300	-17.72800	-30.33900
H	-9.60300	-17.74600	-30.87800
H	-9.04900	-17.19000	-29.58400
O	-8.69300	-20.87900	-33.02500
H	-8.64300	-21.04400	-32.08400
H	-8.80100	-21.74600	-33.41600
O	-11.40200	-24.89600	-31.70900
H	-10.50100	-25.03500	-32.00100
H	-11.32800	-24.73100	-30.77000
O	-7.19100	-18.45900	-32.78100
H	-7.48200	-18.09600	-31.94500
H	-7.80400	-19.17300	-32.96000
O	-8.94200	-23.33600	-34.31300
H	-8.46700	-23.14300	-35.12100
H	-9.86600	-23.28000	-34.55900
O	-1.94600	-22.17700	-37.82500
H	-1.21500	-22.43900	-37.26500
H	-2.55500	-22.91400	-37.78500
O	-16.04100	-20.31800	-29.46300
H	-15.12100	-20.14000	-29.26900
H	-16.07600	-21.26200	-29.61500
O	-18.17200	-26.83400	-27.43100
H	-18.27200	-25.88500	-27.50000
H	-18.39000	-27.16200	-28.30300
O	-18.30900	-23.40400	-28.61400
H	-18.79200	-22.60400	-28.40800
H	-18.22300	-23.39400	-29.56700
O	-14.01300	-19.54200	-27.81200
H	-13.14400	-19.36800	-28.17300
H	-14.49200	-18.72300	-27.93400
O	-19.84400	-12.42200	-36.13300
H	-18.94100	-12.39000	-35.81600
H	-20.07000	-13.35200	-36.11800
O	-2.35400	-7.83300	-38.40700
H	-1.71600	-7.95200	-39.11100
H	-2.47100	-6.88500	-38.34900
O	-5.54500	-7.88000	-38.47100
H	-5.88300	-7.99600	-39.35900
H	-4.67600	-8.28100	-38.49100
O	-9.68600	-6.02700	-42.48200
H	-10.24900	-6.11800	-43.25000
H	-9.80100	-6.84700	-42.00100
O	-5.06300	-6.72700	-32.51300
H	-5.07400	-7.12200	-33.38500
H	-4.90100	-7.46000	-31.91900
O	-12.13900	-4.53200	-39.64300
H	-11.34700	-4.87300	-40.05800
H	-11.90000	-4.42200	-38.72300
O	-4.10600	-2.36000	-47.17000
H	-4.75700	-1.77400	-47.55700
H	-3.31200	-1.82900	-47.11100
O	-8.34200	-3.94300	-33.56500
H	-8.63300	-3.03900	-33.68000
H	-7.70600	-3.90200	-32.85000
O	-7.41600	-6.56500	-36.97500
H	-6.82000	-6.79000	-37.68900
H	-7.35900	-7.30700	-36.37200
O	-2.79900	-10.10800	-41.33200
H	-2.01300	-10.52600	-41.68300
H	-2.53600	-9.20300	-41.16400
O	-2.62800	-4.67100	-46.63300
H	-3.26400	-4.59700	-45.92100
H	-2.95900	-4.08100	-47.31100
O	2.60100	-10.17000	-40.42900
H	3.33400	-10.74100	-40.19500
H	1.84500	-10.55800	-39.98800
O	-5.78100	-7.74800	-43.23200
H	-6.14500	-8.27500	-42.52100
H	-4.92400	-8.13800	-43.40600
O	0.12400	-16.45800	-35.96900

H	-0.20800	-15.58900	-36.19300
H	-0.47200	-16.77500	-35.29000
O	-3.38300	-4.25700	-36.57000
H	-3.68300	-3.35500	-36.67800
H	-3.72800	-4.52400	-35.71800
O	-9.93200	-7.80600	-40.53600
H	-10.85700	-7.93800	-40.32800
H	-9.68300	-7.02600	-40.03900
O	0.01700	-11.73900	-38.89000
H	-0.89200	-11.80700	-39.18400
H	-0.03400	-11.26200	-38.06200
O	-12.57100	-8.16200	-39.70200
H	-12.44200	-7.90900	-38.78800
H	-13.25100	-7.57000	-40.02100
O	-8.40100	2.50700	-43.60300
H	-8.47800	3.45800	-43.52400
H	-9.00500	2.16200	-42.94500
O	-5.90900	-10.32000	-30.94600
H	-6.68600	-9.76000	-30.95600
H	-5.27000	-9.83400	-30.42500
O	1.88300	-13.53700	-37.11200
H	1.24000	-13.24400	-37.75800
H	1.37800	-13.65200	-36.30700
O	-7.65400	-7.51100	-31.38500
H	-6.91300	-6.97300	-31.66100
H	-8.36100	-6.88500	-31.22700
O	-2.17900	-5.36800	-32.78700
H	-1.77100	-5.81000	-33.53200
H	-2.90100	-5.94300	-32.53400
O	-5.82600	-10.43100	-35.41400
H	-4.98000	-10.00700	-35.27100
H	-5.88600	-11.08800	-34.72000
O	-1.85600	-4.80800	-39.03200
H	-2.48500	-4.64400	-39.73500
H	-2.24300	-4.39400	-38.26100
O	2.02300	-3.87300	-41.67800
H	1.33800	-3.49300	-42.22800
H	2.10700	-3.26300	-40.94500
O	-2.67600	-12.77100	-33.75600
H	-2.92000	-12.01600	-34.29300
H	-2.40000	-12.39200	-32.92100
O	-0.72200	-6.22700	-35.07800
H	-0.51000	-5.58400	-35.75500
H	-0.76900	-7.06100	-35.54600
O	-3.11900	-13.61500	-38.17500
H	-3.98100	-13.31700	-37.88300
H	-3.09300	-13.39800	-39.10600
O	-6.30300	-5.07100	-41.76700
H	-5.83900	-4.79200	-42.55700
H	-6.56800	-5.97200	-41.95000
O	-9.41100	-5.52400	-38.82900
H	-8.84100	-5.09500	-39.46700
H	-8.82000	-5.80500	-38.13100
O	-8.91800	-10.35700	-30.58300
H	-9.54800	-9.67600	-30.35000
H	-9.02500	-10.46900	-31.52800
O	-13.62400	-10.69000	-39.65700
H	-14.41400	-10.43400	-40.13300
H	-13.13300	-9.87500	-39.55100
O	0.39300	-3.35000	-36.90600
H	1.21400	-3.75200	-37.18800
H	0.38900	-3.45300	-35.95400
O	-5.84200	-3.80100	-34.75000
H	-5.77700	-3.33900	-33.91400
H	-6.55100	-4.43100	-34.62200
O	-15.34000	-10.43700	-37.22500
H	-15.08900	-9.55200	-36.95900
H	-14.80400	-10.61700	-37.99800
O	8.48300	-7.00600	-40.55800
H	7.73000	-7.57500	-40.71300
H	8.61800	-7.03900	-39.61000
O	-6.12700	-2.13400	-44.88200
H	-6.26200	-1.28900	-44.45400
H	-5.58600	-1.93200	-45.64500
O	-9.64000	-16.05500	-28.22100
H	-10.03400	-16.60900	-27.54600
H	-8.82400	-15.74400	-27.83000
O	-12.67400	-4.61700	-44.30900
H	-12.16200	-3.82300	-44.15900
H	-12.02200	-5.31400	-44.37600
O	-10.86500	-1.50000	-42.22700
H	-10.68800	-0.57800	-42.41500
H	-10.06900	-1.81400	-41.79800
O	-6.72300	-14.63900	-30.62900
H	-5.98300	-14.03900	-30.70800
H	-6.49200	-15.38200	-31.18700
O	-11.57900	-9.45400	-32.35100
H	-11.69400	-10.35500	-32.05000
H	-12.43500	-9.20700	-32.70200
O	-4.50000	-1.51200	-30.75200
H	-4.94400	-2.29800	-31.07000
H	-3.83800	-1.32400	-31.41800
O	-9.74100	0.01800	-36.22600
H	-9.19100	-0.11100	-36.99800
H	-10.58000	0.31700	-36.57600
O	-2.40200	-1.23200	-32.72400
H	-2.42000	-0.41100	-33.21600
H	-1.48000	-1.35600	-32.49900
O	-5.94500	-3.71700	-31.88200
H	-5.41200	-4.47900	-32.11200
H	-6.47300	-4.01000	-31.13900
O	-2.77800	-18.61100	-36.35200
H	-3.12100	-17.72100	-36.42800
H	-2.41000	-18.80100	-37.21500
O	-3.69800	-16.24100	-38.38700
H	-4.20200	-16.27800	-39.20000
H	-3.40500	-15.33200	-38.32800
O	-7.45400	-16.15700	-39.38100
H	-6.58900	-16.35300	-39.74000
H	-7.38600	-16.38600	-38.45400
O	2.16100	-5.86700	-45.91000
H	1.38400	-6.20600	-46.35400
H	2.84000	-5.86200	-46.58400
O	-4.57900	-4.58900	-44.37000
H	-5.10700	-5.32300	-44.68200
H	-5.08100	-3.81100	-44.61600
O	0.40000	-6.39400	-39.25300
H	-0.25800	-5.70400	-39.16500

H	1.20400	-6.00400	-38.91000
O	-0.79000	-8.03900	-41.20300
H	-0.27600	-7.40600	-40.70000
H	-0.14000	-8.63900	-41.56800
O	-3.52700	1.93900	-48.44600
H	-4.20100	2.24200	-47.83800
H	-3.14100	2.74200	-48.79600
O	-4.31200	-10.56400	-38.95700
H	-5.22900	-10.34300	-39.11500
H	-3.89600	-10.49000	-39.81600
O	-3.52800	-9.16500	-43.82600
H	-2.78100	-8.65300	-44.13600
H	-3.25900	-9.48700	-42.96600
O	-0.02300	-3.05900	-43.07500
H	-0.47700	-3.90000	-43.01800
H	-0.65400	-2.42300	-42.73700
O	-1.08000	-12.13000	-41.84500
H	-1.81800	-12.64200	-41.51400
H	-0.82700	-12.57000	-42.65600
O	-1.58000	-1.22100	-42.14000
H	-1.28800	-0.66100	-42.85800
H	-1.07000	-0.92800	-41.38400
O	-6.70700	-9.05700	-40.74300
H	-6.65200	-10.00900	-40.81900
H	-7.51800	-8.90100	-40.25900
O	-6.61700	-0.44300	-41.05000
H	-6.65900	-0.21700	-41.97900
H	-5.70100	-0.67700	-40.90200
O	-3.17000	-9.97500	-34.15200
H	-2.81500	-9.74800	-33.29300
H	-2.52300	-9.64300	-34.77400
O	2.56000	-6.85300	-43.44700
H	2.42100	-6.66300	-44.37500
H	2.20300	-6.09100	-42.99000
O	-9.27600	-12.86700	-37.36800
H	-8.63600	-13.00800	-38.06500
H	-10.10800	-13.15400	-37.74400
O	2.58600	-4.78500	-38.43600
H	3.02000	-4.87800	-37.58800
H	3.30000	-4.80900	-39.07400
O	-8.50000	-3.56700	-41.25400
H	-7.70600	-4.06200	-41.45600
H	-9.21100	-4.09500	-41.61700
O	-3.68100	-1.18500	-36.90300
H	-3.62700	-0.61600	-36.13500
H	-4.54900	-1.01500	-37.26700
O	-10.80100	-11.48300	-35.42000
H	-10.12900	-11.69200	-36.06900
H	-11.62800	-11.68100	-35.86000
O	3.44800	-4.74300	-35.90300
H	3.64200	-3.83800	-35.65700
H	3.34800	-5.20200	-35.06900
O	-4.89800	-14.85700	-33.40400
H	-5.54600	-14.15800	-33.49700
H	-4.12800	-14.42300	-33.03600
O	-1.49000	-4.59200	-30.32900
H	-1.64200	-4.82100	-31.24600
H	-1.50000	-3.63500	-30.31600
O	-4.72200	-7.01900	-35.34400
H	-3.79300	-6.86800	-35.51700
H	-5.14300	-6.93100	-36.19900
O	-5.92300	-2.99400	-37.57400
H	-6.06200	-3.36200	-36.70200
H	-6.71200	-2.47900	-37.74600
O	-5.32800	2.10700	-46.23200
H	-5.15300	1.63600	-45.41700
H	-6.01500	2.73400	-46.00400
O	-4.37300	-3.61600	-40.18500
H	-4.70800	-3.65600	-39.29000
H	-4.87200	-4.28100	-40.65800
O	-10.39800	-8.20900	-34.66000
H	-10.53600	-8.94100	-34.05900
H	-9.48800	-8.29800	-34.94200
O	-7.77000	-8.49700	-35.17200
H	-7.06000	-9.13800	-35.21300
H	-7.81300	-8.24600	-34.24900
O	0.21900	-1.56000	-32.25100
H	0.16000	-2.24400	-32.91800
H	0.62100	-0.81800	-32.70300
O	-9.05700	-11.51000	-33.35200
H	-9.59300	-11.96100	-32.70000
H	-9.64500	-11.37600	-34.09500
O	-10.76200	5.25600	-39.45800
H	-10.52200	5.88800	-38.78000
H	-11.60500	4.90500	-39.17100
O	0.22100	-3.59600	-34.02000
H	-0.66700	-3.93400	-33.89700
H	0.79000	-4.32800	-33.78300
O	-13.32300	-12.37500	-36.26700
H	-13.40700	-12.98200	-37.00200
H	-14.14500	-11.88400	-36.26600
O	-6.24900	1.06400	-37.05400
H	-6.32200	1.86900	-36.54200
H	-6.69200	1.26300	-37.87900
O	-6.46000	0.30100	-43.54300
H	-7.24400	0.84300	-43.45000
H	-5.76100	0.81800	-43.14400
O	-3.15100	-13.89000	-40.90000
H	-2.65400	-14.64300	-41.22000
H	-3.92200	-13.85300	-41.46600
O	4.70200	-8.18500	-39.25800
H	4.69100	-7.30300	-39.62900
H	3.83400	-8.53800	-39.45300
O	-3.79000	0.96300	-34.89800
H	-3.07700	1.59700	-34.97000
H	-4.28000	1.05900	-35.71400
O	-2.06800	-5.13200	-43.32600
H	-3.00200	-5.01200	-43.49600
H	-1.89000	-6.03500	-43.59000
O	-6.56700	-11.74800	-41.17300
H	-6.93700	-12.30100	-40.48500
H	-6.07100	-12.35200	-41.72500
O	1.32000	-9.29100	-42.79100
H	1.78600	-8.45800	-42.86500
H	1.77000	-9.75600	-42.08700
O	-8.73800	-1.28800	-34.07700
H	-8.03700	-0.65400	-33.92400
H	-9.20900	-0.94700	-34.83800

O	-0.61300	-2.94600	-45.79200
H	-0.33700	-3.06100	-44.88300
H	-1.48200	-3.34400	-45.83100
O	-0.70900	-16.92300	-38.75200
H	-1.62300	-16.69200	-38.58400
H	-0.28000	-16.83900	-37.90100
O	-0.93800	-8.73400	-36.25200
H	-1.59100	-8.48600	-36.90700
H	-0.56000	-9.54700	-36.58600
O	-3.97700	1.30200	-42.14900
H	-3.79500	0.48500	-41.68600
H	-3.15400	1.51900	-42.58700
O	-3.64300	0.22900	-44.88800
H	-3.11400	-0.28200	-45.50100
H	-3.38000	-0.08700	-44.02300
O	-4.88900	-7.99200	-29.76500
H	-5.56500	-8.09200	-29.09500
H	-4.06700	-8.14800	-29.30200
O	-13.14200	-1.75400	-37.14400
H	-12.55600	-2.50000	-37.01100
H	-13.33900	-1.44400	-36.26000
O	-10.28800	1.28100	-42.12800
H	-10.95700	1.76500	-42.61100
H	-10.42600	1.52800	-41.21400
O	-18.55000	-9.18400	-38.42400
H	-18.51100	-9.71700	-37.63000
H	-19.05600	-9.71000	-39.04300
O	-6.14700	3.31500	-35.53000
H	-5.59500	3.47800	-34.76500
H	-5.80700	3.90600	-36.20200
O	-11.17100	-14.33700	-34.92700
H	-11.10700	-13.47600	-35.34000
H	-11.23400	-14.95000	-35.66000
O	-11.69500	-12.26500	-31.28000
H	-11.06800	-12.64700	-30.66600
H	-12.55300	-12.48400	-30.91500
O	-1.29600	-1.85900	-29.67500
H	-0.75200	-1.24600	-30.16900
H	-1.87700	-1.30100	-29.15800
O	-3.01000	1.26300	-38.54300
H	-3.13500	0.46200	-39.05200
H	-2.35200	1.75800	-39.03200
O	-11.38900	-12.38200	-39.36600
H	-12.11100	-11.76800	-39.50500
H	-10.60000	-11.84600	-39.44400
O	-0.94700	-0.93600	-36.91500
H	-1.89400	-1.05500	-36.98700
H	-0.59900	-1.82400	-36.83300
O	-8.58700	-15.04600	-33.09100
H	-8.06300	-14.80000	-32.32900
H	-8.80600	-14.21400	-33.51100
O	-3.94200	-1.14100	-40.71500
H	-4.09000	-2.06900	-40.53400
H	-3.11700	-1.11900	-41.19900
O	-0.33900	-0.31800	-39.62800
H	0.58600	-0.52300	-39.76600
H	-0.50700	-0.56600	-38.71900
O	-7.38200	-14.10500	-35.40700
H	-7.72200	-14.90400	-35.00400
H	-8.08800	-13.80500	-35.97900
O	-6.73000	-17.02000	-36.95400
H	-5.90700	-17.49400	-37.07600
H	-6.48600	-16.24100	-36.45400
O	-9.20600	-10.32800	-39.14500
H	-9.35200	-10.01400	-38.25200
H	-9.68400	-9.71000	-39.69700
O	-7.34600	-13.33800	-39.09600
H	-7.36000	-14.23400	-39.43300
H	-6.62900	-13.33100	-38.46300
O	-5.49400	-12.90400	-37.05100
H	-5.75200	-11.98200	-37.05300
H	-6.02500	-13.30100	-36.36100
O	-0.54900	0.20100	-44.27200
H	-0.37600	-0.36400	-45.02500
H	-0.05600	1.00100	-44.45400
O	-14.73200	-7.78800	-36.20600
H	-15.27600	-7.01200	-36.07400
H	-13.85400	-7.44100	-36.36400
O	5.30500	-7.03500	-42.65300
H	5.74800	-7.78500	-43.05100
H	4.42700	-7.04400	-43.03600
O	0.43800	-0.48200	-47.17200
H	1.13300	0.13800	-46.95400
H	0.78100	-1.33300	-46.89700
O	-13.94500	-8.66100	-33.31300
H	-14.68700	-8.51200	-32.72600
H	-14.26600	-8.40000	-34.17600
O	2.33700	-3.03200	-45.64400
H	2.86300	-2.69400	-46.36900
H	2.25000	-3.96800	-45.82700
O	-6.00100	0.88100	-33.51500
H	-5.69200	0.63600	-32.64200
H	-5.20300	1.08600	-34.00300
O	-5.97900	3.03500	-41.28400
H	-6.41800	2.54200	-40.59100
H	-5.22800	2.49200	-41.52500
O	-13.49900	-0.77400	-41.28200
H	-12.67300	-1.07400	-41.66200
H	-13.85000	-1.54300	-40.83300
O	-8.53100	-1.42100	-39.21200
H	-9.15300	-2.08400	-39.51200
H	-7.86100	-1.39500	-39.89500
O	-15.29800	-2.85500	-40.32300
H	-16.13500	-3.28300	-40.50400
H	-15.32600	-2.66200	-39.38600
O	-8.45900	-12.94300	-29.61900
H	-8.41000	-12.06700	-30.00100
H	-7.91600	-13.48600	-30.19200
O	2.02700	-2.39900	-30.33900
H	1.23600	-2.41700	-30.87800
H	1.79000	-1.86000	-29.58400
O	2.14700	-5.55000	-33.02500
H	2.19600	-5.71500	-32.08400
H	2.03800	-6.41700	-33.41600
O	-8.97000	-2.87100	-45.73600
H	-8.82100	-2.18800	-46.38900
H	-8.16300	-2.89100	-45.22100
O	-0.56300	-9.56700	-31.70900

H	0.33800	-9.70600	-32.00100
H	-0.48800	-9.40100	-30.77000
O	-6.22900	-12.19000	-32.86400
H	-7.09800	-11.89700	-33.13600
H	-5.99300	-11.60600	-32.14300
O	3.64800	-3.13000	-32.78100
H	3.35700	-2.76700	-31.94500
H	3.03600	-3.84400	-32.96000
O	4.84800	-5.56500	-40.26100
H	5.68300	-5.12600	-40.09700
H	4.98400	-6.04100	-41.08000
O	1.89700	-8.00700	-34.31300
H	2.37300	-7.81400	-35.12100
H	0.97400	-7.95100	-34.55900
O	-3.52500	-15.97200	-35.34800
H	-3.00700	-15.23600	-35.67300
H	-3.87900	-15.66600	-34.51300
O	-3.27400	-19.55200	-39.82700
H	-2.99300	-20.22000	-39.20100
H	-2.48900	-19.35900	-40.34000
O	-5.47000	-0.37600	-48.26500
H	-6.04300	0.24300	-48.71800
H	-4.89700	0.17400	-47.73100
O	0.59700	-11.13000	-36.08000
H	1.54900	-11.03100	-36.05900
H	0.42800	-11.92300	-35.57100
O	-1.38100	-14.04500	-35.89400
H	-1.97600	-13.76400	-36.59000
H	-1.66600	-13.55500	-35.12300
O	-2.25100	-0.09300	-47.13400
H	-1.31700	-0.15800	-46.93100
H	-2.33800	0.74300	-47.59300
O	0.37800	-0.77600	-50.17400
H	0.30000	-1.11000	-49.28000
H	1.19900	-0.28400	-50.17500
O	3.07600	-7.91000	-36.96600
H	3.04400	-8.84800	-36.78000
H	3.68600	-7.83100	-37.69900
O	3.22600	-11.11900	-36.92000
H	2.70900	-11.90300	-37.10700
H	4.11000	-11.44700	-36.75600
O	0.88700	-17.37500	-40.96100
H	0.68900	-17.68300	-40.07600
H	1.49700	-16.64800	-40.83100
O	8.89400	-6.84800	-37.82500
H	9.62400	-7.11000	-37.26500
H	8.28400	-7.58500	-37.78500
O	6.97500	-8.70700	-37.22800
H	6.24900	-8.84200	-37.83600
H	6.56000	-8.40000	-36.42200
O	-5.20200	-4.98800	-29.46300
H	-4.28100	-4.81100	-29.26900
H	-5.23700	-5.93300	-29.61500
O	-12.20500	-7.07800	-36.82100
H	-11.98700	-6.14700	-36.80200
H	-11.79300	-7.43700	-36.03600
O	-7.33300	-11.50500	-27.43100
H	-7.43300	-10.55600	-27.50000
H	-7.55100	-11.83300	-28.30300
O	-16.11500	-6.17400	-38.56900
H	-16.80800	-6.58900	-39.08200
H	-15.36700	-6.13200	-39.16500
O	-1.52800	-15.98300	-41.50100
H	-1.02800	-15.87100	-42.31000
H	-0.88400	-16.28700	-40.86100
O	-7.47000	-8.07500	-28.61400
H	-7.95300	-7.27500	-28.40800
H	-7.38300	-8.06400	-29.56700
O	-14.20100	-6.24200	-40.69200
H	-14.22400	-6.14400	-41.64400
H	-13.89100	-5.39600	-40.37000
O	-11.54200	-4.18500	-36.57700
H	-10.58800	-4.21600	-36.64400
H	-11.74100	-4.64600	-35.76200
O	-10.95200	-5.61500	-34.01000
H	-10.13300	-5.12000	-33.98700
H	-10.67900	-6.53100	-34.03900
O	2.15600	-13.19900	-40.93200
H	2.93200	-12.70900	-40.66000
H	1.43100	-12.77300	-40.47500
O	4.59300	-12.48600	-39.87500
H	5.18800	-12.72700	-40.58500
H	4.66600	-13.20500	-39.24700
O	-13.18700	1.35700	-39.56000
H	-13.32200	0.59700	-40.12700
H	-13.60400	2.08100	-40.02700
O	-1.22200	-7.70800	-44.43200
H	-1.27800	-7.25100	-45.27200
H	-0.66900	-8.46900	-44.61000
O	2.58900	-11.91900	-43.60700
H	2.33300	-12.32000	-42.77700
H	1.96400	-11.20500	-43.73200
O	6.90000	-9.18000	-41.10000
H	6.34000	-9.11000	-40.32700
H	6.29500	-9.35500	-41.82000
O	-10.03000	2.25400	-39.34900
H	-10.14700	3.19900	-39.45100
H	-10.85100	1.95100	-38.96000
O	-0.14300	-3.02100	-48.50300
H	0.69200	-3.45300	-48.68500
H	-0.30400	-3.19300	-47.57500
O	-0.48800	-6.51800	-47.13700
H	-0.54300	-6.68300	-48.07800
H	-1.11300	-5.81000	-46.98100
O	-15.69900	-11.28100	-33.31100
H	-15.05300	-11.79100	-32.82200
H	-15.90700	-10.54400	-32.73700
O	-3.17300	-4.21300	-27.81200
H	-2.30400	-4.03900	-28.17300
H	-3.65300	-3.39300	-27.93400
O	-9.00500	2.90700	-36.13300
H	-8.10200	2.93900	-35.81600
H	-9.23100	1.97700	-36.11800
O	-7.24900	1.66000	-39.47200
H	-8.20000	1.66000	-39.58500
H	-6.94600	0.91300	-39.98800
O	-11.35900	-14.13400	-29.21400
H	-10.49600	-14.49000	-29.00300

H	-11.93300	-14.47300	-28.52600
O	8.48600	7.49600	-38.40700
H	9.12400	7.37700	-39.11100
H	8.36800	8.44400	-38.34900
O	5.29500	7.44900	-38.47100
H	4.95700	7.33300	-39.35900
H	6.16400	7.04800	-38.49100
O	1.15300	9.30200	-42.48200
H	0.59000	9.21100	-43.25000
H	1.03900	8.48200	-42.00100
O	5.77700	8.60200	-32.51300
H	5.76600	8.20800	-33.38500
H	5.93900	7.86900	-31.91900
O	-1.30000	10.79700	-39.64300
H	-0.50800	10.45700	-40.05800
H	-1.06000	10.90700	-38.72300
O	2.49700	11.38600	-33.56500
H	2.20600	12.29100	-33.68000
H	3.13300	11.42800	-32.85000
O	3.42300	8.76400	-36.97500
H	4.01900	8.53900	-37.68900
H	3.48100	8.02200	-36.37200
O	8.04000	5.22100	-41.33200
H	8.82600	4.80300	-41.68300
H	8.30400	6.12600	-41.16400
O	13.44100	5.15900	-40.42900
H	14.17300	4.58900	-40.19500
H	12.68500	4.77100	-39.98800
O	5.05800	7.58100	-43.23200
H	4.69400	7.05500	-42.52100
H	5.91500	7.19100	-43.40600
O	10.96300	-1.12900	-35.96900
H	10.63200	-0.26000	-36.19300
H	10.36800	-1.44500	-35.29000
O	7.45600	11.07200	-36.57000
H	7.15700	11.97500	-36.67800
H	7.11200	10.80500	-35.71800
O	0.90800	7.52400	-40.53600
H	-0.01700	7.39100	-40.32800
H	1.15600	8.30300	-40.03900
O	10.85600	3.59000	-38.89000
H	9.94800	3.52200	-39.18400
H	10.80500	4.06800	-38.06200
O	4.67800	5.06600	-47.25200
H	4.55800	5.51200	-46.41300
H	3.98300	5.41500	-47.81000
O	-1.73100	7.16700	-39.70200
H	-1.60200	7.42000	-38.78800
H	-2.41200	7.76000	-40.02100
O	4.93000	5.00900	-30.94600
H	4.15400	5.56900	-30.95600
H	5.56900	5.49500	-30.42500
O	12.72200	1.79200	-37.11200
H	12.08000	2.08500	-37.75800
H	12.21800	1.67700	-36.30700
O	3.18500	7.81800	-31.38500
H	3.92600	8.35600	-31.66100
H	2.47900	8.44500	-31.22700
O	8.66000	9.96100	-32.78700
H	9.06900	9.51900	-33.53200
H	7.93800	9.38600	-32.53400
O	5.01400	4.89800	-35.41400
H	5.86000	5.32200	-35.27100
H	4.95300	4.24100	-34.72000
O	8.98300	10.52100	-39.03200
H	8.35400	10.68600	-39.73500
H	8.59600	10.93500	-38.26100
O	8.16300	2.55900	-33.75600
H	7.92000	3.31300	-34.29300
H	8.43900	2.93700	-32.92100
O	10.11700	9.10200	-35.07800
H	10.32900	9.74600	-35.75500
H	10.07000	8.26900	-35.54600
O	7.72000	1.71400	-38.17500
H	6.85800	2.01200	-37.88300
H	7.74600	1.93100	-39.10600
O	4.53600	10.25800	-41.76700
H	5.00000	10.53700	-42.55700
H	4.27200	9.35700	-41.95000
O	1.42900	9.80600	-38.82900
H	1.99900	10.23400	-39.46700
H	2.01900	9.52400	-38.13100
O	3.47300	6.38100	-44.95800
H	4.17500	6.83300	-44.49100
H	3.14100	5.73900	-44.33000
O	1.92100	4.97200	-30.58300
H	1.29100	5.65400	-30.35000
H	1.81500	4.86000	-31.52800
O	-2.78400	4.63900	-39.65700
H	-3.57500	4.89500	-40.13300
H	-2.29300	5.45400	-39.55100
O	11.23200	11.98000	-36.90600
H	12.05400	11.57800	-37.18800
H	11.22800	11.87600	-35.95400
O	-0.83300	5.00400	-42.69800
H	-1.63000	5.11500	-42.18000
H	-1.06000	5.34200	-43.56400
O	4.99800	11.52800	-34.75000
H	5.06200	11.99000	-33.91400
H	4.28900	10.89800	-34.62200
O	-4.50100	4.89200	-37.22500
H	-4.25000	5.77700	-36.95900
H	-3.96500	4.71300	-37.99800
O	1.19900	-0.72600	-28.22100
H	0.80600	-1.27900	-27.54600
H	2.01600	-0.41500	-27.83000
O	-1.83500	10.71200	-44.30900
H	-1.32200	11.50700	-44.15900
H	-1.18200	10.01500	-44.37600
O	-0.02500	13.82900	-42.22700
H	0.15100	14.75100	-42.41500
H	0.77000	13.51500	-41.79800
O	4.11600	0.69000	-30.62900
H	4.85700	1.29000	-30.70800
H	4.34800	-0.05200	-31.18700
O	-0.74000	5.87500	-32.35100
H	-0.85500	4.97400	-32.05000
H	-1.59600	6.12200	-32.70200

O	6.33900	13.81700	-30.75200
H	5.89600	13.03100	-31.07000
H	7.00100	14.00500	-31.41800
O	1.09800	15.34700	-36.22600
H	1.64900	15.21800	-36.99800
H	0.25900	15.64600	-36.57600
O	8.43700	14.09700	-32.72400
H	8.41900	14.91800	-33.21600
H	9.35900	13.97300	-32.49900
O	4.89500	11.61200	-31.88200
H	5.42700	10.85100	-32.11200
H	4.36600	11.31900	-31.13900
O	8.06200	-3.28200	-36.35200
H	7.71800	-2.39200	-36.42800
H	8.43000	-3.47200	-37.21500
O	-3.87000	9.04100	-43.53300
H	-3.03200	9.45100	-43.75000
H	-3.69200	8.10100	-43.56300
O	5.61100	1.55400	-42.78600
H	4.75600	1.16400	-42.96600
H	6.02400	1.63300	-43.64600
O	7.14100	-0.91200	-38.38700
H	6.63800	-0.94900	-39.20000
H	7.43400	-0.00200	-38.32800
O	3.38600	-0.82800	-39.38100
H	4.25100	-1.02400	-39.74000
H	3.45300	-1.05700	-38.45400
O	7.81500	4.58100	-46.01900
H	7.51000	4.89600	-45.16800
H	8.76800	4.66000	-45.97600
O	11.23900	8.93500	-39.25300
H	10.58200	9.62500	-39.16500
H	12.04300	9.32500	-38.91000
O	0.99200	1.40200	-42.00900
H	1.56600	1.18000	-42.74200
H	1.57900	1.47100	-41.25600
O	10.05000	7.29000	-41.20300
H	10.56300	7.92300	-40.70000
H	10.70000	6.69000	-41.56800
O	1.31900	3.43300	-47.98900
H	0.48200	3.77000	-47.66800
H	1.17900	3.29800	-48.92700
O	3.29400	-1.72300	-43.28100
H	2.64100	-2.03900	-43.90600
H	3.25200	-2.34600	-42.55500
O	6.52800	4.76500	-38.95700
H	5.61000	4.98600	-39.11500
H	6.94400	4.83900	-39.81600
O	7.31100	6.16400	-43.82600
H	8.05900	6.67600	-44.13600
H	7.58000	5.84200	-42.96600
O	9.75900	3.20000	-41.84500
H	9.02100	2.68700	-41.51400
H	10.01200	2.76000	-42.65600
O	9.86000	-4.02300	-41.25000
H	10.16600	-4.88300	-41.53800
H	10.58600	-3.43100	-41.45000
O	-3.84700	6.38000	-42.86200
H	-4.04500	5.78700	-43.58700
H	-4.40300	6.07600	-42.14500
O	4.13200	6.27300	-40.74300
H	4.18700	5.32000	-40.81900
H	3.32200	6.42900	-40.25900
O	2.07200	4.89000	-42.98400
H	1.25900	5.19000	-42.57700
H	2.56800	4.49600	-42.26600
O	4.22300	14.88600	-41.05000
H	4.18000	15.11200	-41.97900
H	5.13900	14.65200	-40.90200
O	7.66900	5.35400	-34.15200
H	8.02400	5.58200	-33.29300
H	8.31600	5.68600	-34.77400
O	1.56300	2.46200	-37.36800
H	2.20300	2.32100	-38.06500
H	0.73100	2.17600	-37.74400
O	13.42600	10.54400	-38.43600
H	13.86000	10.45200	-37.58800
H	14.13900	10.52000	-39.07400
O	2.33900	11.76200	-41.25400
H	3.13300	11.26700	-41.45600
H	1.62800	11.23400	-41.61700
O	7.15900	14.14400	-36.90300
H	7.21200	14.71300	-36.13500
H	6.29000	14.31400	-37.26700
O	-1.35300	5.90700	-45.17800
H	-2.28300	5.84500	-44.96100
H	-1.25700	5.38400	-45.97400
O	-1.43400	2.43200	-42.59000
H	-1.17300	3.34700	-42.69900
H	-0.61200	1.96500	-42.44100
O	0.03800	3.84600	-35.42000
H	0.71100	3.63800	-36.06900
H	-0.78900	3.64800	-35.86000
O	14.28700	10.58600	-35.90300
H	14.48100	11.49100	-35.65700
H	14.18700	10.12700	-35.06900
O	5.94100	0.47200	-33.40400
H	5.29400	1.17100	-33.49700
H	6.71100	0.90600	-33.03600
O	9.34900	10.73700	-30.32900
H	9.19700	10.50800	-31.24600
H	9.33900	11.69400	-30.31600
O	6.11700	8.31000	-35.34400
H	7.04700	8.46100	-35.51700
H	5.69700	8.39900	-36.19900
O	4.91600	12.33500	-37.57400
H	4.77700	11.96700	-36.70200
H	4.12800	12.85000	-37.74600
O	6.46700	11.71300	-40.18500
H	6.13200	11.67400	-39.29000
H	5.96700	11.04800	-40.65800
O	0.44100	7.12000	-34.66000
H	0.30300	6.38800	-34.05900
H	1.35200	7.03100	-34.94200
O	3.06900	6.83200	-35.17200
H	3.77900	6.19200	-35.21300
H	3.02600	7.08300	-34.24900
O	11.05800	13.76900	-32.25100

H	10.99900	13.08500	-32.91800
H	11.46000	14.51100	-32.70300
O	1.78200	3.81900	-33.35200
H	1.24600	3.36800	-32.70000
H	1.19400	3.95300	-34.09500
O	0.07700	20.58500	-39.45800
H	0.31700	21.21700	-38.78000
H	-0.76600	20.23400	-39.17100
O	11.06000	11.73300	-34.02000
H	10.17300	11.39500	-33.89700
H	11.63000	11.00100	-33.78300
O	-7.28700	8.06500	-40.69500
H	-6.60000	7.75100	-41.28300
H	-7.44200	8.96900	-40.96900
O	-2.48400	2.95400	-36.26700
H	-2.56800	2.34700	-37.00200
H	-3.30500	3.44500	-36.26600
O	4.59000	16.39300	-37.05400
H	4.51700	17.19900	-36.54200
H	4.14800	16.59200	-37.87900
O	7.68900	1.44000	-40.90000
H	8.18600	0.68700	-41.22000
H	6.91700	1.47600	-41.46600
O	15.54200	7.14400	-39.25800
H	15.53100	8.02600	-39.62900
H	14.67300	6.79100	-39.45300
O	7.05000	16.29200	-34.89800
H	7.76300	16.92600	-34.97000
H	6.55900	16.38800	-35.71400
O	4.27200	3.58100	-41.17300
H	3.90200	3.02900	-40.48500
H	4.76800	2.97700	-41.72500
O	12.15900	6.03900	-42.79100
H	12.62500	6.87100	-42.86500
H	12.61000	5.57300	-42.08700
O	2.10100	14.04100	-34.07700
H	2.80200	14.67500	-33.92400
H	1.63100	14.38300	-34.83800
O	10.13100	-1.59300	-38.75200
H	9.21700	-1.36300	-38.58400
H	10.56000	-1.51000	-37.90100
O	9.90100	6.59600	-36.25200
H	9.24800	6.84300	-36.90700
H	10.27900	5.78200	-36.58600
O	5.95000	7.33800	-29.76500
H	5.27400	7.23700	-29.09500
H	6.77300	7.18200	-29.30200
O	-2.30200	13.57500	-37.14400
H	-1.71700	12.82900	-37.01100
H	-2.50000	13.88500	-36.26000
O	0.55200	16.61000	-42.12800
H	-0.11700	17.09500	-42.61100
H	0.41300	16.85700	-41.21400
O	-7.71000	6.14500	-38.42400
H	-7.67200	5.61200	-37.63000
H	-8.21600	5.61900	-39.04300
O	4.69200	18.64400	-35.53000
H	5.24400	18.80700	-34.76500
H	5.03300	19.23500	-36.20200
O	-0.33100	0.99200	-34.92700
H	-0.26700	1.85300	-35.34000
H	-0.39500	0.37900	-35.66000
O	-0.85600	3.06400	-31.28000
H	-0.22900	2.68200	-30.66600
H	-1.71300	2.84600	-30.91500
O	9.54300	13.47000	-29.67500
H	10.08700	14.08300	-30.16900
H	8.96200	14.02800	-29.15800
O	-4.08000	4.87200	-45.34800
H	-3.78400	3.96300	-45.40200
H	-4.98100	4.85100	-45.67000
O	7.82900	16.59300	-38.54300
H	7.70400	15.79100	-39.05200
H	8.48700	17.08700	-39.03200
O	-0.55000	2.94700	-39.36600
H	-1.27100	3.56100	-39.50500
H	0.23900	3.48300	-39.44400
O	9.89300	14.39300	-36.91500
H	8.94600	14.27400	-36.98700
H	10.24100	13.50600	-36.83300
O	2.25300	0.28300	-33.09100
H	2.77600	0.52900	-32.32900
H	2.03400	1.11500	-33.51100
O	-2.35100	2.55300	-45.22400
H	-2.67900	1.67300	-45.40700
H	-1.99700	2.49800	-44.33700
O	6.89700	14.18900	-40.71500
H	6.75000	13.26000	-40.53400
H	7.72300	14.21100	-41.19900
O	0.76300	2.84000	-44.85700
H	1.10300	3.36900	-45.57900
H	1.21100	3.17900	-44.08100
O	3.45800	1.22400	-35.40700
H	3.11700	0.42500	-35.00400
H	2.75200	1.52400	-35.97900
O	4.10900	-1.69100	-36.95400
H	4.93200	-2.16500	-37.07600
H	4.35300	-0.91200	-36.45400
O	2.75000	1.33800	-46.65700
H	3.47100	1.71600	-47.16100
H	2.12700	2.05700	-46.55400
O	6.89300	2.04200	-45.72200
H	7.11000	2.92500	-46.02300
H	6.41300	1.65100	-46.45100
O	1.63300	5.00100	-39.14500
H	1.48800	5.31500	-38.25200
H	1.15600	5.62000	-39.69700
O	3.49300	1.99100	-39.09600
H	3.47900	1.09500	-39.43300
H	4.21000	1.99800	-38.46300
O	5.34600	2.42500	-37.05100
H	5.08700	3.34700	-37.05300
H	4.81400	2.02800	-36.36100
O	-3.89200	7.54200	-36.20600
H	-4.43700	8.31800	-36.07400
H	-3.01400	7.88900	-36.36400
O	2.89700	0.87300	-43.82800
H	3.01800	-0.05600	-43.62900

H	2.60700	0.88700	-44.74000
O	5.76600	-1.44200	-40.69100
H	5.78800	-1.37800	-41.64600
H	6.18300	-2.28100	-40.49700
O	-3.10600	6.66900	-33.31300
H	-3.84700	6.81700	-32.72600
H	-3.42600	6.92900	-34.17600
O	4.83900	16.21000	-33.51500
H	5.14800	15.96500	-32.64200
H	5.63600	16.41500	-34.00300
O	-2.66000	14.55500	-41.28200
H	-1.83400	14.25500	-41.66200
H	-3.01000	13.78600	-40.83300
O	2.30800	13.90800	-39.21200
H	1.68600	13.24500	-39.51200
H	2.97900	13.93400	-39.89500
O	-4.45900	12.47400	-40.32300
H	-5.29600	12.04600	-40.50400
H	-4.48700	12.66700	-39.38600
O	2.38100	2.38600	-29.61900
H	2.43000	3.26200	-30.00100
H	2.92300	1.84400	-30.19200
O	12.86600	12.93000	-30.33900
H	12.07600	12.91200	-30.87800
H	12.63000	13.46900	-29.58400
O	12.98600	9.77900	-33.02500
H	13.03600	9.61400	-32.08400
H	12.87700	8.91200	-33.41600
O	10.27600	5.76200	-31.70900
H	11.17700	5.62300	-32.00100
H	10.35100	5.92800	-30.77000
O	4.61100	3.13900	-32.86400
H	3.74100	3.43200	-33.13600
H	4.84700	3.72300	-32.14300
O	14.48800	12.19900	-32.78100
H	14.19600	12.56200	-31.94500
H	13.87500	11.48600	-32.96000
O	12.73700	7.32200	-34.31300
H	13.21200	7.51500	-35.12100
H	11.81300	7.37800	-34.55900
O	-0.37500	8.48500	-44.73000
H	-0.62100	7.58100	-44.53200
H	-0.20600	8.48600	-45.67200
O	7.31500	-0.64300	-35.34800
H	7.83300	0.09300	-35.67300
H	6.96000	-0.33700	-34.51300
O	-1.10600	4.21700	-47.23100
H	-1.79900	4.29200	-47.88700
H	-1.35000	3.45400	-46.70900
O	7.56600	-4.22300	-39.82700
H	7.84600	-4.89100	-39.20100
H	8.35100	-4.03000	-40.34000
O	11.43700	4.19900	-36.08000
H	12.38800	4.29900	-36.05900
H	11.26700	3.40700	-35.57100
O	9.45800	1.28400	-35.89400
H	8.86300	1.56500	-36.59000
H	9.17300	1.77400	-35.12300
O	13.91500	7.41900	-36.96600
H	13.88300	6.48100	-36.78000
H	14.52600	7.49800	-37.69900
O	14.06500	4.21100	-36.92000
H	13.54900	3.42600	-37.10700
H	14.94900	3.88200	-36.75600
O	11.72600	-2.04500	-40.96100
H	11.52800	-2.35400	-40.07600
H	12.33600	-1.31900	-40.83100
O	19.73300	8.48100	-37.82500
H	20.46300	8.22000	-37.26500
H	19.12400	7.74400	-37.78500
O	17.81500	6.62200	-37.22800
H	17.08800	6.48700	-37.83600
H	17.39900	6.92900	-36.42200
O	5.63800	10.34100	-29.46300
H	6.55800	10.51900	-29.26900
H	5.60300	9.39600	-29.61500
O	-5.14400	5.53500	-40.53800
H	-5.46400	4.68100	-40.83100
H	-5.69100	5.75000	-39.78200
O	-1.36600	8.25100	-36.82100
H	-1.14700	9.18300	-36.80200
H	-0.95400	7.89200	-36.03600
O	5.55400	-1.24200	-44.83300
H	4.74400	-1.58800	-44.45800
H	6.24300	-1.55200	-44.24500
O	7.48300	-2.16400	-43.21100
H	8.03600	-1.74500	-42.55200
H	7.72000	-3.09100	-43.16900
O	3.50700	3.82400	-27.43100
H	3.40600	4.77400	-27.50000
H	3.28900	3.49600	-28.30300
O	-5.27500	9.15500	-38.56900
H	-5.96900	8.74000	-39.08200
H	-4.52800	9.19700	-39.16500
O	10.86100	-0.05400	-43.70300
H	10.28100	0.60400	-44.08500
H	10.59500	-0.87700	-44.11400
O	9.31200	-0.65400	-41.50100
H	9.81200	-0.54200	-42.31000
H	9.95500	-0.95800	-40.86100
O	3.37000	7.25400	-28.61400
H	2.88700	8.05400	-28.40800
H	3.45600	7.26500	-29.56700
O	-3.36200	9.08700	-40.69200
H	-3.38500	9.18600	-41.64400
H	-3.05200	9.93300	-40.37000
O	-0.70300	11.14400	-36.57700
H	0.25100	11.11300	-36.64400
H	-0.90200	10.68300	-35.76200
O	-0.11300	9.71500	-34.01000
H	0.70600	10.20900	-33.98700
H	0.16100	8.79800	-34.03900
O	9.64400	2.10600	-44.64800
H	8.75600	2.39100	-44.86500
H	10.05900	1.94500	-45.49500
O	12.99500	2.13000	-40.93200
H	13.77100	2.62000	-40.66000
H	12.27000	2.55600	-40.47500

O	15.43200	2.84300	-39.87500
H	16.02700	2.60300	-40.58500
H	15.50500	2.12400	-39.24700
O	-2.34800	16.68600	-39.56000
H	-2.48200	15.92600	-40.12700
H	-2.76500	17.41000	-40.02700
O	13.42800	3.41000	-43.60700
H	13.17200	3.01000	-42.77700
H	12.80400	4.12400	-43.73200
O	17.74000	6.15000	-41.10000
H	17.18000	6.21900	-40.32700
H	17.13400	5.97400	-41.82000
O	0.80900	17.58300	-39.34900
H	0.69200	18.52800	-39.45100
H	-0.01200	17.28100	-38.96000
O	-4.85900	4.04800	-33.31100
H	-4.21400	3.53800	-32.82200
H	-5.06800	4.78500	-32.73700
O	7.66600	11.11600	-27.81200
H	8.53500	11.29000	-28.17300
H	7.18700	11.93600	-27.93400
O	3.66100	-1.68800	-47.55900
H	3.64900	-0.74300	-47.40200
H	4.55300	-1.87100	-47.85300
O	1.83500	18.23600	-36.13300
H	2.73700	18.26800	-35.81600
H	1.60800	17.30600	-36.11800
O	3.59000	16.98900	-39.47200
H	2.63900	16.98900	-39.58500
H	3.89300	16.24200	-39.98800
O	7.47600	-5.09300	-43.30400
H	6.70900	-5.64800	-43.15800
H	7.88700	-5.02100	-42.44300
O	-0.52000	1.19500	-29.21400
H	0.34300	0.83900	-29.00300
H	-1.09300	0.85600	-28.52600
O	15.77000	20.33900	-30.94600
H	14.99300	20.89800	-30.95600
H	16.40900	20.82400	-30.42500
O	14.02400	23.14700	-31.38500
H	14.76600	23.68600	-31.66100
H	13.31800	23.77400	-31.22700
O	12.76000	20.30100	-30.58300
H	12.13000	20.98300	-30.35000
H	12.65400	20.18900	-31.52800
O	6.33900	20.22100	-37.22500
H	6.58900	21.10600	-36.95900
H	6.87500	20.04200	-37.99800
O	12.03800	14.60300	-28.22100
H	11.64500	14.05000	-27.54600
H	12.85500	14.91400	-27.83000
O	14.95500	16.01900	-30.62900
H	15.69600	16.62000	-30.70800
H	15.18700	15.27700	-31.18700
O	10.09900	21.20500	-32.35100
H	9.98500	20.30300	-32.05000
H	9.24400	21.45100	-32.70200
O	18.90100	12.04700	-36.35200
H	18.55700	12.93700	-36.42800
H	19.26900	11.85700	-37.21500
O	10.87800	19.17500	-35.42000
H	11.55000	18.96700	-36.06900
H	10.05100	18.97700	-35.86000
O	16.78000	15.80100	-33.40400
H	16.13300	16.50000	-33.49700
H	17.55100	16.23500	-33.03600
O	12.62200	19.14800	-33.35200
H	12.08500	18.69700	-32.70000
H	12.03300	19.28300	-34.09500
O	8.35600	18.28400	-36.26700
H	8.27100	17.67600	-37.00200
H	7.53400	18.77400	-36.26600
O	16.78900	22.66700	-29.76500
H	16.11300	22.56600	-29.09500
H	17.61200	22.51100	-29.30200
O	3.12900	21.47500	-38.42400
H	3.16700	20.94100	-37.63000
H	2.62300	20.94800	-39.04300
O	10.50800	16.32100	-34.92700
H	10.57200	17.18200	-35.34000
H	10.44500	15.70800	-35.66000
O	9.98300	18.39300	-31.28000
H	10.61000	18.01100	-30.66600
H	9.12600	18.17500	-30.91500
O	13.09200	15.61200	-33.09100
H	13.61500	15.85800	-32.32900
H	12.87300	16.44400	-33.51100
O	14.29700	16.55300	-35.40700
H	13.95700	15.75400	-35.00400
H	13.59100	16.85400	-35.97900
O	14.94900	13.63800	-36.95400
H	15.77100	13.16400	-37.07600
H	15.19300	14.41700	-36.45400
O	7.73400	21.99800	-33.31300
H	6.99200	22.14600	-32.72600
H	7.41300	22.25900	-34.17600
O	13.22000	17.71500	-29.61900
H	13.26900	18.59100	-30.00100
H	13.76200	17.17300	-30.19200
O	15.45000	18.46800	-32.86400
H	14.58000	18.76200	-33.13600
H	15.68600	19.05200	-32.14300
O	18.15400	14.68600	-35.34800
H	18.67200	15.42200	-35.67300
H	17.79900	14.99300	-34.51300
O	16.47700	25.67000	-29.46300
H	17.39700	25.84800	-29.26900
H	16.44200	24.72600	-29.61500
O	14.34600	19.15300	-27.43100
H	14.24600	20.10300	-27.50000
H	14.12800	18.82500	-28.30300
O	14.20900	22.58300	-28.61400
H	13.72600	23.38300	-28.40800
H	14.29500	22.59400	-29.56700
O	5.98000	19.37800	-33.31100
H	6.62600	18.86700	-32.82200
H	5.77200	20.11400	-32.73700
O	18.50500	26.44600	-27.81200

H	19.37400	26.62000	-28.17300
H	18.02600	27.26500	-27.93400
O	10.32000	16.52400	-29.21400
H	11.18300	16.16800	-29.00300
H	9.74600	16.18600	-28.52600
O	2.49700	-11.60800	-46.84000
H	2.20600	-10.70300	-46.95500
H	3.13300	-11.56600	-46.12600
O	3.18500	-15.17600	-44.66000
H	3.92600	-14.63700	-44.93700
H	2.47900	-14.54900	-44.50300
O	8.66000	-13.03300	-46.06300
H	9.06900	-13.47500	-46.80700
H	7.93800	-13.60800	-45.80900
O	11.23200	-11.01400	-50.18100
H	12.05400	-11.41600	-50.46300
H	11.22800	-11.11700	-49.23000
O	6.33900	-9.17700	-44.02700
H	5.89600	-9.96300	-44.34500
H	7.00100	-8.98900	-44.69300
O	8.43700	-8.89600	-45.99900
H	8.41900	-8.07500	-46.49100
H	9.35900	-9.02100	-45.77400
O	4.89500	-11.38200	-45.15700
H	5.42700	-12.14300	-45.38700
H	4.36600	-11.67500	-44.41500
O	14.28700	-12.40700	-49.17800
H	14.48100	-11.50300	-48.93200
H	14.18700	-12.86700	-48.34400
O	9.34900	-12.25700	-43.60400
H	9.19700	-12.48600	-44.52100
H	9.33900	-11.30000	-43.59100
O	11.05800	-9.22500	-45.52700
H	10.99900	-9.90900	-46.19400
H	11.46000	-8.48300	-45.97900
O	11.06000	-11.26100	-47.29500
H	10.17300	-11.59800	-47.17300
H	11.63000	-11.99300	-47.05800
O	7.05000	-6.70200	-48.17300
H	7.76300	-6.06700	-48.24600
H	6.55900	-6.60600	-48.98900
O	2.10100	-8.95300	-47.35300
H	2.80200	-8.31900	-47.20000
H	1.63100	-8.61100	-48.11300
O	5.95000	-15.65600	-43.04100
H	5.27400	-15.75700	-42.37100
H	6.77300	-15.81200	-42.57700
O	4.69200	-4.34900	-48.80500
H	5.24400	-4.18600	-48.04000
H	5.03300	-3.75900	-49.47700
O	9.54300	-9.52300	-42.95000
H	10.08700	-8.91000	-43.44500
H	8.96200	-8.96500	-42.43400
O	9.89300	-8.60000	-50.19100
H	8.94600	-8.72000	-50.26300
H	10.24100	-9.48800	-50.10800
O	4.83900	-6.78400	-46.79000
H	5.14800	-7.02900	-45.91800
H	5.63600	-6.57900	-47.27800
O	12.86600	-10.06400	-43.61400
H	12.07600	-10.08100	-44.15400
H	12.63000	-9.52500	-42.85900
O	12.98600	-13.21500	-46.30100
H	13.03600	-13.38000	-45.35900
H	12.87700	-14.08200	-46.69100
O	10.27600	-17.23100	-44.98500
H	11.17700	-17.37100	-45.27600
H	10.35100	-17.06600	-44.04500
O	14.48800	-10.79500	-46.05700
H	14.19600	-10.43200	-45.22000
H	13.87500	-11.50800	-46.23500
O	12.73700	-15.67200	-47.58900
H	13.21200	-15.47900	-48.39700
H	11.81300	-15.61600	-47.83400
O	5.63800	-12.65300	-42.73900
H	6.55800	-12.47500	-42.54500
H	5.60300	-13.59700	-42.89100
O	3.50700	-19.17000	-40.70600
H	3.40600	-18.22000	-40.77500
H	3.28900	-19.49800	-41.57900
O	3.37000	-15.74000	-41.89000
H	2.88700	-14.94000	-41.68300
H	3.45600	-15.72900	-42.84300
O	7.66600	-11.87700	-41.08700
H	8.53500	-11.70300	-41.44900
H	7.18700	-11.05800	-41.21000
O	1.83500	-4.75800	-49.40800
H	2.73700	-4.72500	-49.09200
H	1.60800	-5.68800	-49.39400
O	15.77000	-2.65500	-44.22200
H	14.99300	-2.09600	-44.23200
H	16.40900	-2.17000	-43.70000
O	14.02400	0.15300	-44.66000
H	14.76600	0.69200	-44.93700
H	13.31800	0.78000	-44.50300
O	12.76000	-2.69300	-43.85900
H	12.13000	-2.01100	-43.62500
H	12.65400	-2.80400	-44.80300
O	6.33900	-2.77200	-50.50000
H	6.58900	-1.88800	-50.23500
H	6.87500	-2.95200	-51.27300
O	12.03800	-8.39100	-41.49600
H	11.64500	-8.94400	-40.82100
H	12.85500	-8.08000	-41.10500
O	14.95500	-6.97500	-43.90400
H	15.69600	-6.37400	-43.98400
H	15.18700	-7.71700	-44.46200
O	10.09900	-1.78900	-45.62600
H	9.98500	-2.69100	-45.32600
H	9.24400	-1.54300	-45.97800
O	10.87800	-3.81900	-48.69600
H	11.55000	-4.02700	-49.34500
H	10.05100	-4.01600	-49.13500
O	16.78000	-7.19200	-46.67900
H	16.13300	-6.49300	-46.77300
H	17.55100	-6.75900	-46.31200
O	12.62200	-3.84600	-46.62800
H	12.08500	-4.29700	-45.97600

H	12.03300	-3.71100	-47.37100
O	8.35600	-4.71000	-49.54200
H	8.27100	-5.31800	-50.27700
H	7.53400	-4.21900	-49.54100
O	16.78900	-0.32700	-43.04100
H	16.11300	-0.42800	-42.37100
H	17.61200	-0.48300	-42.57700
O	3.12900	-1.51900	-51.69900
H	3.16700	-2.05300	-50.90500
H	2.62300	-2.04600	-52.31800
O	10.50800	-6.67300	-48.20300
H	10.57200	-5.81100	-48.61500
H	10.44500	-7.28500	-48.93600
O	9.98300	-4.60000	-44.55600
H	10.61000	-4.98300	-43.94200
H	9.12600	-4.81900	-44.19000
O	13.09200	-7.38200	-46.36700
H	13.61500	-7.13600	-45.60400
H	12.87300	-6.55000	-46.78700
O	14.29700	-6.44000	-48.68200
H	13.95700	-7.24000	-48.28000
H	13.59100	-6.14000	-49.25400
O	14.94900	-9.35500	-50.22900
H	15.77100	-9.82900	-50.35100
H	15.19300	-8.57600	-49.72900
O	7.73400	-0.99600	-46.58800
H	6.99200	-0.84800	-46.00200
H	7.41300	-0.73500	-47.45200
O	13.22000	-5.27900	-42.89400
H	13.26900	-4.40200	-43.27600
H	13.76200	-5.82100	-43.46700
O	15.45000	-4.52600	-46.14000
H	14.58000	-4.23200	-46.41100
H	15.68600	-3.94100	-45.41900
O	18.15400	-8.30800	-48.62300
H	18.67200	-7.57100	-48.94800
H	17.79900	-8.00100	-47.78800
O	16.47700	2.67600	-42.73900
H	17.39700	2.85400	-42.54500
H	16.44200	1.73200	-42.89100
O	14.34600	-3.84000	-40.70600
H	14.24600	-2.89100	-40.77500
H	14.12800	-4.16900	-41.57900
O	14.20900	-0.41100	-41.89000
H	13.72600	0.38900	-41.68300
H	14.29500	-0.40000	-42.84300
O	5.98000	-3.61600	-46.58700
H	6.62600	-4.12600	-46.09800
H	5.77200	-2.87900	-46.01200
O	18.50500	3.45200	-41.08700
H	19.37400	3.62600	-41.44900
H	18.02600	4.27100	-41.21000
O	10.32000	-6.47000	-42.48900
H	11.18300	-6.82600	-42.27900
H	9.74600	-6.80800	-41.80200
O	-40.75600	20.48800	-13.87800
H	-40.02400	19.91800	-13.64400
H	-41.51200	20.10000	-13.43700
O	-41.47500	17.12100	-10.56100
H	-42.11700	17.41500	-11.20700
H	-41.97900	17.00700	-9.75600
O	-41.33500	26.78600	-15.12700
H	-42.02000	27.16500	-15.67700
H	-41.25000	27.39500	-14.39400
O	-34.87400	23.65300	-14.00700
H	-35.62800	23.08300	-14.16200
H	-34.74000	23.61900	-13.06000
O	-41.19700	24.79100	-19.35900
H	-41.97400	24.45200	-19.80300
H	-40.51800	24.79600	-20.03300
O	-40.79800	23.80600	-16.89600
H	-40.93600	23.99600	-17.82400
H	-41.15400	24.56700	-16.44000
O	-40.77100	25.87300	-11.88500
H	-40.33700	25.78100	-11.03700
H	-40.05800	25.84900	-12.52300
O	-39.90900	25.91600	-9.35200
H	-39.71600	26.82000	-9.10600
H	-40.00900	25.45600	-8.51800
O	-38.65500	22.47300	-12.70700
H	-38.66600	23.35500	-13.07800
H	-39.52400	22.12000	-12.90200
O	-42.03800	21.36800	-16.24000
H	-41.57200	22.20100	-16.31400
H	-41.58700	20.90200	-15.53600
O	-38.05300	23.62300	-16.10200
H	-37.61000	22.87400	-16.50000
H	-38.93000	23.61400	-16.48500
O	-41.02000	27.62600	-19.09300
H	-40.49500	27.96500	-19.81800
H	-41.10800	26.69100	-19.27600
O	-41.33100	28.25900	-3.78800
H	-42.12100	28.24200	-4.32700
H	-41.56700	28.79800	-3.03300
O	-41.21100	25.10800	-6.47400
H	-41.16100	24.94300	-5.53300
H	-41.32000	24.24100	-6.86500
O	-39.70900	27.52800	-6.23000
H	-40.00100	27.89100	-5.39400
H	-40.32200	26.81500	-6.40900
O	-38.51000	25.09300	-13.71000
H	-37.67500	25.53200	-13.54600
H	-38.37400	24.61700	-14.52900
O	-41.46000	22.65100	-7.76200
H	-40.98500	22.84400	-8.57000
H	-42.38400	22.70700	-8.00800
O	-40.28100	22.74900	-10.41500
H	-40.31400	21.81000	-10.22900
H	-39.67100	22.82700	-11.14900
O	-40.13200	19.54000	-10.36900
H	-40.64800	18.75600	-10.55600
H	-39.24800	19.21200	-10.20500
O	-42.47100	13.28400	-14.41000
H	-42.66800	12.97500	-13.52500
H	-41.86100	14.01000	-14.28000
O	-34.46400	23.81000	-11.27500
H	-33.73300	23.54900	-10.71400
H	-35.07300	23.07300	-11.23400

O	-36.38200	21.95100	-10.67700
H	-37.10900	21.81700	-11.28500
H	-36.79700	22.25900	-9.87100
O	-41.20200	17.45900	-14.38100
H	-40.42600	17.94900	-14.10900
H	-41.92700	17.88500	-13.92400
O	-38.76400	18.17200	-13.32400
H	-38.17000	17.93200	-14.03400
H	-38.69200	17.45300	-12.69600
O	-40.76900	18.73900	-17.05700
H	-41.02500	18.33900	-16.22600
H	-41.39300	19.45400	-17.18200
O	-36.45700	21.47900	-14.54900
H	-37.01700	21.54800	-13.77600
H	-37.06300	21.30300	-15.26900
O	-32.39400	29.52900	-9.41800
H	-32.72600	30.39900	-9.64200
H	-32.99000	29.21300	-8.73900
O	-30.63500	32.45000	-10.56100
H	-31.27800	32.74400	-11.20700
H	-31.14000	32.33600	-9.75600
O	-42.15800	29.93200	-1.67000
H	-42.55200	29.37900	-0.99500
H	-41.34200	30.24300	-1.27900
O	-35.29600	27.37600	-9.80100
H	-35.63900	28.26600	-9.87700
H	-34.92800	27.18600	-10.66400
O	-36.21600	29.74700	-11.83600
H	-36.72000	29.71000	-12.64900
H	-35.92300	30.65600	-11.77700
O	-39.97200	29.83100	-12.83000
H	-39.10700	29.63500	-13.18900
H	-39.90400	29.60200	-11.90300
O	-40.06300	28.93500	-16.73100
H	-40.71700	28.62000	-17.35500
H	-40.10500	28.31300	-16.00500
O	-33.49700	26.63600	-14.69900
H	-33.19100	25.77500	-14.98700
H	-32.77200	27.22700	-14.90000
O	-37.41600	31.13100	-6.85300
H	-38.06400	31.83000	-6.94600
H	-36.64600	31.56400	-6.48500
O	-35.66900	32.09800	-14.34900
H	-35.17200	31.34500	-14.66900
H	-36.44000	32.13400	-14.91500
O	-33.22700	29.06500	-12.20100
H	-34.14100	29.29500	-12.03300
H	-32.79800	29.14900	-11.35000
O	-39.24800	28.96800	-10.40300
H	-38.42500	28.49400	-10.52500
H	-39.00400	29.74700	-9.90300
O	-37.59200	29.21700	-14.14000
H	-37.57000	29.28100	-15.09500
H	-37.17500	28.37700	-13.94600
O	-36.04300	30.01500	-8.79700
H	-35.52500	30.75200	-9.12200
H	-36.39700	30.32200	-7.96200
O	-35.79200	26.43500	-13.27700
H	-35.51200	25.76800	-12.65000
H	-35.00700	26.62800	-13.78900
O	-33.89900	31.94300	-9.34300
H	-34.49400	32.22300	-10.03900
H	-34.18400	32.43200	-8.57200
O	-29.29300	34.86900	-10.36900
H	-29.80900	34.08500	-10.55600
H	-28.40900	34.54100	-10.20500
O	-31.63100	28.61300	-14.41000
H	-31.82900	28.30500	-13.52500
H	-31.02200	29.33900	-14.28000
O	-37.80300	29.41600	-18.28300
H	-38.61300	29.07100	-17.90700
H	-37.11500	29.10700	-17.69400
O	-35.87400	28.49400	-16.66000
H	-35.32100	28.91300	-16.00100
H	-35.63700	27.56700	-16.61800
O	-32.49600	30.60400	-17.15200
H	-33.07600	31.26200	-17.53400
H	-32.76300	29.78200	-17.56300
O	-34.04600	30.00400	-14.95000
H	-33.54600	30.11600	-15.75900
H	-33.40200	29.70100	-14.31000
O	-33.71400	32.76400	-18.09700
H	-34.60100	33.04900	-18.31500
H	-33.29800	32.60300	-18.94400
O	-30.36200	32.78900	-14.38100
H	-29.58600	33.27800	-14.10900
H	-31.08700	33.21400	-13.92400
O	-27.92500	33.50100	-13.32400
H	-27.33100	33.26100	-14.03400
H	-27.85200	32.78200	-12.69600
O	-35.88100	25.56500	-16.75300
H	-36.64800	25.01100	-16.60800
H	-35.47000	25.63700	-15.89200
O	-40.75600	-2.50600	-27.15300
H	-40.02400	-3.07600	-26.91900
H	-41.51200	-2.89400	-26.71300
O	-41.47500	-5.87300	-23.83700
H	-42.11700	-5.57900	-24.48300
H	-41.97900	-5.98700	-23.03100
O	-41.33500	3.79200	-28.40200
H	-42.02000	4.17200	-28.95300
H	-41.25000	4.40100	-27.66900
O	-34.87400	0.65900	-27.28200
H	-35.62800	0.09000	-27.43800
H	-34.74000	0.62500	-26.33500
O	-41.19700	1.79700	-32.63400
H	-41.97400	1.45800	-33.07900
H	-40.51800	1.80300	-33.30900
O	-40.79800	0.81200	-30.17200
H	-40.93600	1.00200	-31.10000
H	-41.15400	1.57400	-29.71500
O	-40.77100	2.87900	-25.16100
H	-40.33700	2.78700	-24.31200
H	-40.05800	2.85500	-25.79900
O	-39.90900	2.92200	-22.62700
H	-39.71600	3.82600	-22.38100
H	-40.00900	2.46200	-21.79400
O	-38.65500	-0.52100	-25.98300

H	-38.66600	0.36200	-26.35300
H	-39.52400	-0.87400	-26.17700
O	-42.03800	-1.62600	-29.51600
H	-41.57200	-0.79300	-29.59000
H	-41.58700	-2.09200	-28.81100
O	-38.05300	0.62900	-29.37800
H	-37.61000	-0.12000	-29.77600
H	-38.93000	0.62000	-29.76000
O	-41.02000	4.63200	-32.36800
H	-40.49500	4.97100	-33.09300
H	-41.10800	3.69700	-32.55200
O	-41.33100	5.26500	-17.06300
H	-42.12100	5.24800	-17.60300
H	-41.56700	5.80400	-16.30800
O	-41.21100	2.11500	-19.75000
H	-41.16100	1.95000	-18.80800
H	-41.32000	1.24700	-20.14000
O	-39.70900	4.53400	-19.50600
H	-40.00100	4.89700	-18.66900
H	-40.32200	3.82100	-19.68400
O	-38.51000	2.10000	-26.98500
H	-37.67500	2.53900	-26.82200
H	-38.37400	1.62400	-27.80500
O	-41.46000	-0.34200	-21.03800
H	-40.98500	-0.15000	-21.84600
H	-42.38400	-0.28600	-21.28300
O	-40.28100	-0.24500	-23.69100
H	-40.31400	-1.18400	-23.50500
H	-39.67100	-0.16700	-24.42400
O	-40.13200	-3.45400	-23.64500
H	-40.64800	-4.23800	-23.83200
H	-39.24800	-3.78200	-23.48000
O	-42.47100	-9.71000	-27.68500
H	-42.66800	-10.01800	-26.80100
H	-41.86100	-8.98400	-27.55500
O	-34.46400	0.81600	-24.55000
H	-33.73300	0.55500	-23.98900
H	-35.07300	0.07900	-24.51000
O	-36.38200	-1.04300	-23.95300
H	-37.10900	-1.17700	-24.56100
H	-36.79700	-0.73500	-23.14700
O	-41.20200	-5.53400	-27.65700
H	-40.42600	-5.04500	-27.38400
H	-41.92700	-5.10900	-27.19900
O	-38.76400	-4.82200	-26.59900
H	-38.17000	-5.06200	-27.31000
H	-38.69200	-5.54100	-25.97200
O	-40.76900	-4.25500	-30.33200
H	-41.02500	-4.65500	-29.50100
H	-41.39300	-3.54000	-30.45700
O	-36.45700	-1.51500	-27.82500
H	-37.01700	-1.44600	-27.05200
H	-37.06300	-1.69100	-28.54500
O	-34.87200	15.16100	-25.13200
H	-34.23400	15.04100	-25.83600
H	-34.99000	16.10900	-25.07300
O	-38.06300	15.11300	-25.19600
H	-38.40100	14.99800	-26.08400
H	-37.19400	14.71300	-25.21600
O	-37.58100	16.26700	-19.23700
H	-37.59200	15.87200	-20.10900
H	-37.41900	15.53400	-18.64400
O	-40.86000	19.05100	-20.28900
H	-41.15100	19.95500	-20.40500
H	-40.22400	19.09200	-19.57500
O	-39.93400	16.42800	-23.69900
H	-39.33800	16.20400	-24.41300
H	-39.87700	15.68700	-23.09700
O	-35.31800	12.88600	-28.05700
H	-34.53100	12.46800	-28.40800
H	-35.05400	13.79000	-27.88800
O	-29.91700	12.82300	-27.15300
H	-29.18400	12.25300	-26.91900
H	-30.67300	12.43600	-26.71300
O	-32.39400	6.53500	-22.69400
H	-32.72600	7.40500	-22.91800
H	-32.99000	6.21900	-22.01400
O	-35.90100	18.73700	-23.29400
H	-36.20100	19.63900	-23.40300
H	-36.24600	18.47000	-22.44200
O	-32.50100	11.25500	-25.61400
H	-33.41000	11.18700	-25.90900
H	-32.55300	11.73200	-24.78600
O	-38.42700	12.67400	-17.67100
H	-39.20400	13.23300	-17.68100
H	-37.78800	13.16000	-17.14900
O	-30.63500	9.45700	-23.83700
H	-31.27800	9.75000	-24.48300
H	-31.14000	9.34200	-23.03100
O	-32.98100	13.20300	-33.14800
H	-32.06900	13.43600	-33.32300
H	-33.49000	13.78700	-33.71100
O	-40.17300	15.48300	-18.10900
H	-39.43100	16.02100	-18.38600
H	-40.87900	16.10900	-17.95200
O	-34.69700	17.62500	-19.51200
H	-34.28900	17.18400	-20.25700
H	-35.41900	17.05100	-19.25900
O	-38.34400	12.56300	-22.13900
H	-37.49800	12.98700	-21.99500
H	-38.40400	11.90600	-21.44500
O	-34.37400	18.18600	-25.75700
H	-35.00300	18.35000	-26.45900
H	-34.76200	18.60000	-24.98600
O	-30.49500	19.12100	-28.40200
H	-31.18000	19.50100	-28.95300
H	-30.41100	19.73100	-27.66900
O	-35.19400	10.22300	-20.48000
H	-35.43800	10.97700	-21.01700
H	-34.91800	10.60200	-19.64600
O	-33.24000	16.76700	-21.80300
H	-33.02800	17.41000	-22.47900
H	-33.28700	15.93300	-22.27100
O	-35.63800	9.37900	-24.89900
H	-36.49900	9.67700	-24.60700
H	-35.61100	9.59600	-25.83100
O	-41.43600	12.63600	-17.30800
H	-42.06700	13.31800	-17.07400

H	-41.54300	12.52500	-18.25200
O	-32.12600	19.64400	-23.63100
H	-31.30400	19.24200	-23.91200
H	-32.12900	19.54100	-22.67900
O	-38.36000	19.19300	-21.47400
H	-38.29500	19.65500	-20.63900
H	-39.06900	18.56200	-21.34600
O	-24.03500	15.98800	-27.28200
H	-24.78900	15.41900	-27.43800
H	-23.90000	15.95400	-26.33500
O	-42.15800	6.93900	-14.94500
H	-42.55200	6.38500	-14.27000
H	-41.34200	7.25000	-14.55400
O	-39.24200	8.35400	-17.35300
H	-38.50100	8.95500	-17.43300
H	-39.01000	7.61200	-17.91100
O	-37.01800	21.48200	-17.47700
H	-37.46200	20.69500	-17.79400
H	-36.35700	21.67000	-18.14200
O	-34.92000	21.76200	-19.44800
H	-34.93800	22.58300	-19.94000
H	-33.99800	21.63700	-19.22300
O	-38.46300	19.27600	-18.60600
H	-37.93000	18.51500	-18.83600
H	-38.99100	18.98400	-17.86400
O	-35.29600	4.38200	-23.07700
H	-35.63900	5.27300	-23.15300
H	-34.92800	4.19300	-23.94000
O	-37.74600	9.21800	-29.51100
H	-38.60200	8.82900	-29.69000
H	-37.33300	9.29700	-30.37100
O	-36.21600	6.75300	-25.11200
H	-36.72000	6.71600	-25.92500
H	-35.92300	7.66200	-25.05200
O	-39.97200	6.83700	-26.10500
H	-39.10700	6.64100	-26.46500
H	-39.90400	6.60800	-25.17800
O	-30.35700	17.12700	-32.63400
H	-31.13400	16.78800	-33.07900
H	-29.67800	17.13200	-33.30900
O	-35.54200	12.24600	-32.74300
H	-35.84700	12.56100	-31.89200
H	-34.58900	12.32400	-32.70100
O	-32.11800	16.60000	-25.97700
H	-32.77600	17.29000	-25.88900
H	-31.31400	16.99000	-25.63400
O	-42.36600	9.06700	-28.73300
H	-41.79100	8.84500	-29.46600
H	-41.77900	9.13600	-27.98100
O	-33.30800	14.95400	-27.92700
H	-32.79500	15.58700	-27.42500
H	-32.65800	14.35500	-28.29300
O	-40.06300	5.94100	-30.00600
H	-40.71700	5.62600	-30.63000
H	-40.10500	5.31900	-29.28000
O	-36.83000	12.42900	-25.68100
H	-37.74700	12.65100	-25.83900
H	-36.41400	12.50400	-26.54000
O	-36.04600	13.82800	-30.55100
H	-35.29900	14.34100	-30.86000
H	-35.77700	13.50700	-29.69100
O	-32.54200	19.93500	-29.79900
H	-32.99500	19.09400	-29.74300
H	-33.17200	20.57100	-29.46200
O	-33.59800	10.86400	-28.56900
H	-34.33600	10.35200	-28.23800
H	-33.34600	10.42400	-29.38100
O	-33.49700	3.64200	-27.97500
H	-33.19100	2.78200	-28.26200
H	-32.77200	4.23400	-28.17500
O	-39.22500	13.93700	-27.46700
H	-39.17000	12.98500	-27.54300
H	-40.03600	14.09300	-26.98300
O	-35.68800	13.01900	-20.87700
H	-35.33300	13.24600	-20.01700
H	-35.04100	13.35100	-21.49900
O	-29.95900	16.14100	-30.17200
H	-30.09700	16.33100	-31.10000
H	-30.31500	16.90300	-29.71500
O	-41.79400	10.12700	-24.09200
H	-41.15400	9.98600	-24.79000
H	-42.62700	9.84000	-24.46800
O	-29.93200	18.20900	-25.16100
H	-29.49800	18.11600	-24.31200
H	-29.21800	18.18400	-25.79900
O	-36.19900	21.80900	-23.62700
H	-36.14500	22.37800	-22.86000
H	-37.06700	21.97900	-23.99200
O	-29.07000	18.25100	-22.62700
H	-28.87600	19.15600	-22.38100
H	-29.17000	17.79200	-21.79400
O	-37.41600	8.13700	-20.12800
H	-38.06400	8.83600	-20.22200
H	-36.64600	8.57000	-19.76100
O	-34.00900	18.40200	-17.05300
H	-34.16000	18.17300	-17.97000
H	-34.01800	19.35900	-17.04000
O	-37.24000	15.97500	-22.06900
H	-36.31100	16.12600	-22.24200
H	-37.66100	16.06300	-22.92400
O	-38.44100	20.00000	-24.29900
H	-38.58000	19.63200	-23.42600
H	-39.23000	20.51500	-24.47000
O	-36.89100	19.37800	-26.91000
H	-37.22600	19.33800	-26.01400
H	-37.39000	18.71300	-27.38300
O	-40.28800	14.49700	-21.89600
H	-39.57800	13.85600	-21.93800
H	-40.33100	14.74800	-20.97400
O	-32.29900	21.43400	-18.97600
H	-32.35800	20.74900	-19.64300
H	-31.89700	22.17500	-19.42800
O	-41.57500	11.48300	-20.07700
H	-42.11100	11.03200	-19.42500
H	-42.16300	11.61800	-20.82000
O	-32.29800	19.39700	-20.74400
H	-33.18500	19.06000	-20.62200
H	-31.72800	18.66600	-20.50700

O	-35.66900	9.10400	-27.62400
H	-35.17200	8.35100	-27.94400
H	-36.44000	9.14100	-28.19000
O	-27.81600	14.80800	-25.98300
H	-27.82700	15.69100	-26.35300
H	-28.68400	14.45500	-26.17700
O	-36.30800	23.95600	-21.62200
H	-35.59500	24.59100	-21.69500
H	-36.79900	24.05300	-22.43800
O	-34.58600	17.86100	-30.05100
H	-35.52000	17.98200	-30.22000
H	-34.40800	16.95900	-30.31500
O	-39.08500	11.24600	-27.89700
H	-39.45600	10.69300	-27.20900
H	-38.58900	10.64200	-28.45000
O	-31.19900	13.70300	-29.51600
H	-30.73300	14.53600	-29.59000
H	-30.74800	13.23800	-28.81100
O	-41.25600	21.70600	-20.80200
H	-40.55500	22.33900	-20.64900
H	-41.72700	22.04700	-21.56200
O	-33.22700	6.07100	-25.47700
H	-34.14100	6.30200	-25.30800
H	-32.79800	6.15500	-24.62500
O	-33.45600	14.26000	-22.97700
H	-34.10900	14.50800	-23.63100
H	-33.07800	13.44700	-23.31000
O	-37.40800	15.00200	-16.49000
H	-38.08300	14.90200	-15.82000
H	-36.58500	14.84600	-16.02600
O	-38.66500	26.30900	-22.25400
H	-38.11300	26.47200	-21.48900
H	-38.32500	26.89900	-22.92600
O	-33.81400	21.13500	-16.39900
H	-33.27000	21.74800	-16.89400
H	-34.39600	21.69300	-15.88300
O	-35.52800	24.25700	-25.26800
H	-35.65300	23.45600	-25.77700
H	-34.87100	24.75200	-25.75600
O	-33.46500	22.05800	-23.64000
H	-34.41200	21.93900	-23.71200
H	-33.11700	21.17000	-23.55700
O	-41.10500	7.94700	-19.81600
H	-40.58200	8.19400	-19.05300
H	-41.32400	8.77900	-20.23600
O	-36.46000	21.85300	-27.43900
H	-36.60800	20.92500	-27.25900
H	-35.63500	21.87500	-27.92400
O	-32.85700	22.67600	-26.35300
H	-31.93200	22.47000	-26.49000
H	-33.02500	22.42800	-25.44400
O	-39.90000	8.88900	-22.13100
H	-40.24000	8.09000	-21.72900
H	-40.60600	9.18900	-22.70300
O	-39.24800	5.97400	-23.67800
H	-38.42500	5.50000	-23.80000
H	-39.00400	6.75300	-23.17800
O	-36.46400	9.70700	-32.44600
H	-36.24800	10.59000	-32.74700
H	-36.94500	9.31600	-33.17600
O	-41.72400	12.66600	-25.86900
H	-41.87000	12.98000	-24.97700
H	-42.20200	13.28400	-26.42200
O	-39.86500	9.65600	-25.82100
H	-39.87800	8.76000	-26.15800
H	-39.14700	9.66300	-25.18700
O	-38.01200	10.09000	-23.77600
H	-38.27000	11.01100	-23.77700
H	-38.54300	9.69300	-23.08600
O	-40.46100	8.53700	-30.55200
H	-40.33900	7.60900	-30.35300
H	-40.75100	8.55100	-31.46400
O	-37.59200	6.22300	-27.41600
H	-37.57000	6.28700	-28.37000
H	-37.17500	5.38300	-27.22200
O	-27.21300	15.95800	-29.37800
H	-26.77000	15.20900	-29.77600
H	-28.09100	15.94900	-29.76000
O	-30.18100	19.96100	-32.36800
H	-29.65600	20.30000	-33.09300
H	-30.26800	19.02600	-32.55200
O	-38.51900	23.87500	-20.23900
H	-38.21000	23.63000	-19.36700
H	-37.72100	24.07900	-20.72700
O	-40.97700	10.05100	-16.34300
H	-40.92800	10.92700	-16.72500
H	-40.43500	9.50800	-16.91600
O	-30.49100	20.59500	-17.06300
H	-31.28200	20.57700	-17.60300
H	-30.72800	21.13300	-16.30800
O	-30.37200	17.44400	-19.75000
H	-30.32200	17.27900	-18.80800
H	-30.48000	16.57700	-20.14000
O	-33.08100	13.42700	-18.43400
H	-32.18000	13.28800	-18.72600
H	-33.00600	13.59200	-17.49400
O	-38.74700	10.80400	-19.58900
H	-39.61700	11.09700	-19.86000
H	-38.51100	11.38800	-18.86800
O	-28.87000	19.86400	-19.50600
H	-29.16100	20.22700	-18.66900
H	-29.48200	19.15000	-19.68400
O	-27.67000	17.42900	-26.98500
H	-26.83500	17.86800	-26.82200
H	-27.53400	16.95300	-27.80500
O	-30.62100	14.98700	-21.03800
H	-30.14600	15.18000	-21.84600
H	-31.54400	15.04300	-21.28300
O	-36.04300	7.02200	-22.07200
H	-35.52500	7.75800	-22.39700
H	-36.39700	7.32800	-21.23700
O	-35.79200	3.44200	-26.55200
H	-35.51200	2.77400	-25.92600
H	-35.00700	3.63500	-27.06500
O	-31.92100	11.86300	-22.80500
H	-30.96900	11.96300	-22.78400
H	-32.09000	11.07100	-22.29500
O	-33.89900	8.94900	-22.61900

H	-34.49400	9.22900	-23.31400
H	-34.18400	9.43800	-21.84700
O	-29.44200	15.08400	-23.69100
H	-29.47400	14.14600	-23.50500
H	-28.83200	15.16200	-24.42400
O	-29.29300	11.87500	-23.64500
H	-29.80900	11.09100	-23.83200
H	-28.40900	11.54700	-23.48000
O	-31.63100	5.61900	-27.68500
H	-31.82900	5.31100	-26.80100
H	-31.02200	6.34500	-27.55500
O	-23.62400	16.14600	-24.55000
H	-22.89400	15.88400	-23.98900
H	-24.23400	15.40800	-24.51000
O	-25.54300	14.28600	-23.95300
H	-26.27000	14.15200	-24.56100
H	-25.95800	14.59400	-23.14700
O	-37.72000	18.00500	-16.18800
H	-36.80000	18.18300	-15.99400
H	-37.75500	17.06100	-16.34000
O	-37.80300	6.42200	-31.55800
H	-38.61300	6.07700	-31.18200
H	-37.11500	6.11300	-30.96900
O	-35.87400	5.50000	-29.93600
H	-35.32100	5.92000	-29.27700
H	-35.63700	4.57400	-29.89300
O	-39.85100	11.48900	-14.15500
H	-39.95100	12.43800	-14.22400
H	-40.06900	11.16100	-15.02800
O	-32.49600	7.61000	-30.42800
H	-33.07600	8.26900	-30.81000
H	-32.76300	6.78800	-30.83900
O	-34.04600	7.01100	-28.22600
H	-33.54600	7.12200	-29.03400
H	-33.40200	6.70700	-27.58500
O	-39.98800	14.91900	-15.33900
H	-40.47100	15.71900	-15.13200
H	-39.90200	14.92900	-16.29200
O	-33.71400	9.77000	-31.37200
H	-34.60100	10.05500	-31.59000
H	-33.29800	9.61000	-32.21900
O	-30.36200	9.79500	-27.65700
H	-29.58600	10.28400	-27.38400
H	-31.08700	10.22100	-27.19900
O	-27.92500	10.50800	-26.59900
H	-27.33100	10.26700	-27.31000
H	-27.85200	9.78800	-25.97200
O	-33.74000	15.28600	-31.15700
H	-33.79600	15.74200	-31.99600
H	-33.18700	14.52500	-31.33500
O	-29.92900	11.07500	-30.33200
H	-30.18500	10.67400	-29.50100
H	-30.55400	11.78900	-30.45700
O	-25.61800	13.81400	-27.82500
H	-26.17800	13.88400	-27.05200
H	-26.22400	13.63800	-28.54500
O	-35.69200	18.78100	-14.53600
H	-34.82200	18.95500	-14.89800
H	-36.17100	19.60000	-14.65900
O	-35.88100	2.57100	-30.02900
H	-36.64800	2.01700	-29.88300
H	-35.47000	2.64300	-29.16700
O	-24.03200	30.49000	-25.13200
H	-23.39400	30.37100	-25.83600
H	-24.15000	31.43800	-25.07300
O	-27.22300	30.44300	-25.19600
H	-27.56200	30.32700	-26.08400
H	-26.35400	30.04200	-25.21600
O	-26.74100	31.59600	-19.23700
H	-26.75300	31.20100	-20.10900
H	-26.57900	30.86300	-18.64400
O	-29.09500	31.75700	-23.69900
H	-28.49900	31.53300	-24.41300
H	-29.03800	31.01600	-23.09700
O	-24.47800	28.21500	-28.05700
H	-23.69200	27.79700	-28.40800
H	-24.21400	29.12000	-27.88800
O	-19.07700	28.15300	-27.15300
H	-18.34500	27.58200	-26.91900
H	-19.83300	27.76500	-26.71300
O	-21.55500	21.86500	-22.69400
H	-21.88600	22.73400	-22.91800
H	-22.15000	21.54800	-22.01400
O	-25.06200	34.06600	-23.29400
H	-25.36200	34.96800	-23.40300
H	-25.40700	33.79900	-22.44200
O	-21.66200	26.58400	-25.61400
H	-22.57000	26.51600	-25.90900
H	-21.71300	27.06100	-24.78600
O	-27.58800	28.00300	-17.67100
H	-28.36500	28.56300	-17.68100
H	-26.94900	28.48900	-17.14900
O	-19.79600	24.78600	-23.83700
H	-20.43800	25.07900	-24.48300
H	-20.30000	24.67100	-23.03100
O	-22.14200	28.53200	-33.14800
H	-21.23000	28.76500	-33.32300
H	-22.65100	29.11600	-33.71100
O	-29.33300	30.81200	-18.10900
H	-28.59200	31.35000	-18.38600
H	-30.04000	31.43800	-17.95200
O	-23.85800	32.95500	-19.51200
H	-23.44900	32.51300	-20.25700
H	-24.58000	32.38000	-19.25900
O	-27.50500	27.89200	-22.13900
H	-26.65800	28.31600	-21.99500
H	-27.56500	27.23500	-21.44500
O	-23.53500	33.51500	-25.75700
H	-24.16400	33.67900	-26.45900
H	-23.92200	33.92900	-24.98600
O	-19.65600	34.45000	-28.40200
H	-20.34100	34.83000	-28.95300
H	-19.57200	35.06000	-27.66900
O	-24.35500	25.55200	-20.48000
H	-24.59900	26.30700	-21.01700
H	-24.07900	25.93100	-19.64600
O	-22.40100	32.09600	-21.80300
H	-22.18900	32.73900	-22.47900

H	-22.44800	31.26200	-22.27100
O	-24.79800	24.70800	-24.89900
H	-25.66000	25.00600	-24.60700
H	-24.77200	24.92500	-25.83100
O	-30.59700	27.96600	-17.30800
H	-31.22700	28.64700	-17.07400
H	-30.70300	27.85400	-18.25200
O	-21.28600	34.97300	-23.63100
H	-20.46400	34.57100	-23.91200
H	-21.29000	34.87000	-22.67900
O	-27.52100	34.52200	-21.47400
H	-27.45600	34.98400	-20.63900
H	-28.22900	33.89200	-21.34600
O	-13.19600	31.31700	-27.28200
H	-13.94900	30.74800	-27.43800
H	-13.06100	31.28400	-26.33500
O	-31.31900	22.26800	-14.94500
H	-31.71200	21.71400	-14.27000
H	-30.50200	22.57900	-14.55400
O	-28.40200	23.68400	-17.35300
H	-27.66100	24.28400	-17.43300
H	-28.17000	22.94100	-17.91100
O	-33.25800	28.86900	-19.07500
H	-33.37300	27.96800	-18.77500
H	-34.11400	29.11600	-19.42700
O	-27.62300	34.60600	-18.60600
H	-27.09100	33.84400	-18.83600
H	-28.15200	34.31300	-17.86400
O	-24.45700	19.71200	-23.07700
H	-24.80000	20.60200	-23.15300
H	-24.08900	19.52200	-23.94000
O	-26.90700	24.54700	-29.51100
H	-27.76300	24.15800	-29.69000
H	-26.49400	24.62700	-30.37100
O	-25.37700	22.08200	-25.11200
H	-25.88000	22.04500	-25.92500
H	-25.08400	22.99100	-25.05200
O	-29.13300	22.16600	-26.10500
H	-28.26700	21.97000	-26.46500
H	-29.06500	21.93700	-25.17800
O	-19.51800	32.45600	-32.63400
H	-20.29500	32.11700	-33.07900
H	-18.83900	32.46100	-33.30900
O	-24.70300	27.57500	-32.74300
H	-25.00800	27.89000	-31.89200
H	-23.75000	27.65300	-32.70100
O	-21.27900	31.92900	-25.97700
H	-21.93700	32.61900	-25.88900
H	-20.47500	32.31900	-25.63400
O	-31.52600	24.39600	-28.73300
H	-30.95200	24.17400	-29.46600
H	-30.93900	24.46500	-27.98100
O	-22.46800	30.28400	-27.92700
H	-21.95500	30.91700	-27.42500
H	-21.81800	29.68400	-28.29300
O	-29.22400	21.27000	-30.00600
H	-29.87800	20.95500	-30.63000
H	-29.26600	20.64800	-29.28000
O	-25.99000	27.75900	-25.68100
H	-26.90800	27.98000	-25.83900
H	-25.57400	27.83300	-26.54000
O	-25.20700	29.15800	-30.55100
H	-24.46000	29.67000	-30.86000
H	-24.93800	28.83600	-29.69100
O	-21.70200	35.26400	-29.79900
H	-22.15600	34.42300	-29.74300
H	-22.33300	35.90000	-29.46200
O	-22.75900	26.19300	-28.56900
H	-23.49700	25.68100	-28.23800
H	-22.50600	25.75300	-29.38100
O	-22.65800	18.97100	-27.97500
H	-22.35200	18.11100	-28.26200
H	-21.93200	19.56300	-28.17500
O	-28.38600	29.26600	-27.46700
H	-28.33100	28.31400	-27.54300
H	-29.19700	29.42200	-26.98300
O	-24.84900	28.34800	-20.87700
H	-24.49400	28.57500	-20.01700
H	-24.20200	28.68000	-21.49900
O	-19.11900	31.47000	-30.17200
H	-19.25700	31.66000	-31.10000
H	-19.47600	32.23200	-29.71500
O	-30.95500	25.45600	-24.09200
H	-30.31500	25.31500	-24.79000
H	-31.78700	25.16900	-24.46800
O	-19.09200	33.53800	-25.16100
H	-18.65900	33.44500	-24.31200
H	-18.37900	33.51400	-25.79900
O	-32.48000	26.84000	-22.14500
H	-31.80700	26.63100	-22.79400
H	-33.30700	26.64200	-22.58400
O	-18.23100	33.58000	-22.62700
H	-18.03700	34.48500	-22.38100
H	-18.33100	33.12100	-21.79400
O	-26.57700	23.46600	-20.12800
H	-27.22400	24.16500	-20.22200
H	-25.80700	23.90000	-19.76100
O	-23.16900	33.73100	-17.05300
H	-23.32100	33.50200	-17.97000
H	-23.17900	34.68800	-17.04000
O	-26.40100	31.30400	-22.06900
H	-25.47100	31.45500	-22.24200
H	-26.82200	31.39200	-22.92400
O	-26.05100	34.70700	-26.91000
H	-26.38600	34.66700	-26.01400
H	-26.55100	34.04200	-27.38300
O	-32.07700	30.11400	-21.38500
H	-32.21500	29.38200	-20.78400
H	-31.16600	30.02500	-21.66600
O	-29.44900	29.82600	-21.89600
H	-28.73900	29.18500	-21.93800
H	-29.49200	30.07700	-20.97400
O	-21.46000	36.76300	-18.97600
H	-21.51900	36.07900	-19.64300
H	-21.05800	37.50500	-19.42800
O	-30.73600	26.81300	-20.07700
H	-31.27200	26.36200	-19.42500
H	-31.32400	26.94700	-20.82000

O	-21.45800	34.72700	-20.74400
H	-22.34600	34.38900	-20.62200
H	-20.88800	33.99500	-20.50700
O	-35.00200	25.94800	-22.99100
H	-35.08600	25.34100	-23.72600
H	-35.82400	26.43900	-22.99100
O	-24.82900	24.43300	-27.62400
H	-24.33300	23.68000	-27.94400
H	-25.60100	24.47000	-28.19000
O	-16.97700	30.13800	-25.98300
H	-16.98800	31.02000	-26.35300
H	-17.84500	29.78500	-26.17700
O	-23.74700	33.19100	-30.05100
H	-24.68100	33.31100	-30.22000
H	-23.56900	32.28800	-30.31500
O	-28.24600	26.57500	-27.89700
H	-28.61600	26.02200	-27.20900
H	-27.75000	25.97100	-28.45000
O	-20.35900	29.03200	-29.51600
H	-19.89300	29.86500	-29.59000
H	-19.90800	28.56700	-28.81100
O	-22.38800	21.40000	-25.47700
H	-23.30100	21.63100	-25.30800
H	-21.95800	21.48400	-24.62500
O	-22.61700	29.58900	-22.97700
H	-23.27000	29.83700	-23.63100
H	-22.23900	28.77600	-23.31000
O	-26.56800	30.33100	-16.49000
H	-27.24400	30.23100	-15.82000
H	-25.74500	30.17500	-16.02600
O	-32.84900	23.98600	-21.65200
H	-32.78500	24.84700	-22.06400
H	-32.91300	23.37300	-22.38500
O	-33.37400	26.05800	-18.00500
H	-32.74700	25.67600	-17.39100
H	-34.23100	25.83900	-17.63900
O	-22.97500	36.46400	-16.39900
H	-22.43100	37.07700	-16.89400
H	-23.55600	37.02200	-15.88300
O	-33.06800	25.94100	-26.09000
H	-33.79000	26.55500	-26.22900
H	-32.27900	26.47700	-26.16800
O	-30.26600	23.27700	-19.81600
H	-29.74200	23.52300	-19.05300
H	-30.48400	24.10900	-20.23600
O	-29.06000	24.21800	-22.13100
H	-29.40100	23.41900	-21.72900
H	-29.76600	24.51800	-22.70300
O	-28.40900	21.30300	-23.67800
H	-27.58600	20.82900	-23.80000
H	-28.16500	22.08200	-23.17800
O	-25.62500	25.03600	-32.44600
H	-25.40800	25.91900	-32.74700
H	-26.10500	24.64500	-33.17600
O	-30.88500	27.99500	-25.86900
H	-31.03000	28.30900	-24.97700
H	-31.36200	28.61300	-26.42200
O	-29.02500	24.98500	-25.82100
H	-29.03900	24.08900	-26.15800
H	-28.30800	24.99200	-25.18700
O	-27.17300	25.41900	-23.77600
H	-27.43100	26.34100	-23.77700
H	-27.70400	25.02200	-23.08600
O	-29.62200	23.86700	-30.55200
H	-29.50000	22.93800	-30.35300
H	-29.91100	23.88000	-31.46400
O	-26.75200	21.55200	-27.41600
H	-26.73000	21.61600	-28.37000
H	-26.33600	20.71200	-27.22200
O	-16.37400	31.28800	-29.37800
H	-15.93100	30.53800	-29.77600
H	-17.25100	31.27900	-29.76000
O	-35.62400	29.66200	-20.03700
H	-36.36600	29.81100	-19.45100
H	-35.94400	29.92300	-20.90100
O	-19.34100	35.29100	-32.36800
H	-18.81600	35.62900	-33.09300
H	-19.42900	34.35500	-32.55200
O	-30.13700	25.38000	-16.34300
H	-30.08900	26.25600	-16.72500
H	-29.59500	24.83700	-16.91600
O	-19.65200	35.92400	-17.06300
H	-20.44200	35.90600	-17.60300
H	-19.88900	36.46300	-16.30800
O	-19.53200	32.77300	-19.75000
H	-19.48200	32.60800	-18.80800
H	-19.64100	31.90600	-20.14000
O	-22.24200	28.75600	-18.43400
H	-21.34100	28.61700	-18.72600
H	-22.16700	28.92200	-17.49400
O	-27.90700	26.13300	-19.58900
H	-28.77700	26.42600	-19.86000
H	-27.67200	26.71700	-18.86800
O	-18.03000	35.19300	-19.50600
H	-18.32200	35.55600	-18.66900
H	-18.64300	34.47900	-19.68400
O	-16.83100	32.75800	-26.98500
H	-15.99600	33.19700	-26.82200
H	-16.69500	32.28200	-27.80500
O	-19.78200	30.31600	-21.03800
H	-19.30600	30.50900	-21.84600
H	-20.70500	30.37200	-21.28300
O	-25.20400	22.35100	-22.07200
H	-24.68500	23.08700	-22.39700
H	-25.55800	22.65700	-21.23700
O	-24.95200	18.77100	-26.55200
H	-24.67200	18.10300	-25.92600
H	-24.16700	18.96400	-27.06500
O	-21.08100	27.19300	-22.80500
H	-20.13000	27.29200	-22.78400
H	-21.25100	26.40000	-22.29500
O	-23.06000	24.27800	-22.61900
H	-23.65500	24.55900	-23.31400
H	-23.34500	24.76800	-21.84700
O	-18.60300	30.41300	-23.69100
H	-18.63500	29.47500	-23.50500
H	-17.99300	30.49200	-24.42400
O	-18.45300	27.20400	-23.64500

H	-18.96900	26.42000	-23.83200
H	-17.56900	26.87600	-23.48000
O	-20.79200	20.94800	-27.68500
H	-20.99000	20.64000	-26.80100
H	-20.18200	21.67500	-27.55500
O	-12.78500	31.47500	-24.55000
H	-12.05500	31.21300	-23.98900
H	-13.39400	30.73800	-24.51000
O	-14.70300	29.61600	-23.95300
H	-15.43000	29.48100	-24.56100
H	-15.11900	29.92300	-23.14700
O	-26.88000	33.33500	-16.18800
H	-25.96000	33.51200	-15.99400
H	-26.91500	32.39000	-16.34000
O	-33.88400	31.24500	-23.54600
H	-33.66600	32.17600	-23.52700
H	-33.47200	30.88500	-22.76000
O	-26.96400	21.75200	-31.55800
H	-27.77400	21.40600	-31.18200
H	-26.27500	21.44200	-30.96900
O	-25.03500	20.82900	-29.93600
H	-24.48200	21.24900	-29.27700
H	-24.79800	19.90300	-29.89300
O	-29.01100	26.81800	-14.15500
H	-29.11200	27.76700	-14.22400
H	-29.22900	26.49000	-15.02800
O	-21.65700	22.93900	-30.42800
H	-22.23700	23.59800	-30.81000
H	-21.92300	22.11700	-30.83900
O	-23.20700	22.34000	-28.22600
H	-22.70700	22.45200	-29.03400
H	-22.56300	22.03600	-27.58500
O	-29.14800	30.24800	-15.33900
H	-29.63200	31.04800	-15.13200
H	-29.06200	30.25900	-16.29200
O	-32.63100	32.70800	-20.73500
H	-31.81200	33.20300	-20.71100
H	-32.35700	31.79100	-20.76300
O	-22.87400	25.09900	-31.37200
H	-23.76200	25.38400	-31.59000
H	-22.45900	24.93900	-32.21900
O	-19.52300	25.12400	-27.65700
H	-18.74700	25.61400	-27.38400
H	-20.24800	25.55000	-27.19900
O	-17.08600	25.83700	-26.59900
H	-16.49100	25.59600	-27.31000
H	-17.01300	25.11700	-25.97200
O	-22.90000	30.61500	-31.15700
H	-22.95700	31.07200	-31.99600
H	-22.34700	29.85400	-31.33500
O	-19.09000	26.40400	-30.33200
H	-19.34600	26.00300	-29.50100
H	-19.71500	27.11800	-30.45700
O	-14.77900	29.14300	-27.82500
H	-15.33900	29.21300	-27.05200
H	-15.38400	28.96800	-28.54500
O	-37.37800	27.04200	-20.03600
H	-36.73200	26.53200	-19.54700
H	-37.58600	27.77900	-19.46100
O	-24.85200	34.11000	-14.53600
H	-23.98300	34.28400	-14.89800
H	-25.33200	34.93000	-14.65900
O	-25.04200	17.90000	-30.02900
H	-25.80900	17.34600	-29.88300
H	-24.63100	17.97300	-29.16700
O	-33.03800	24.18900	-15.93800
H	-32.17500	23.83300	-15.72800
H	-33.61100	23.85000	-15.25100
O	-10.71500	37.19400	-22.69400
H	-11.04700	38.06300	-22.91800
H	-11.31100	36.87800	-22.01400
O	-8.95600	40.11500	-23.83700
H	-9.59900	40.40800	-24.48300
H	-9.46100	40.00000	-23.03100
O	-20.48000	37.59700	-14.94500
H	-20.87300	37.04400	-14.27000
H	-19.66300	37.90800	-14.55400
O	-13.61700	35.04100	-23.07700
H	-13.96100	35.93100	-23.15300
H	-13.24900	34.85100	-23.94000
O	-14.53700	37.41100	-25.11200
H	-15.04100	37.37400	-25.92500
H	-14.24400	38.32100	-25.05200
O	-18.29300	37.49500	-26.10500
H	-17.42800	37.29900	-26.46500
H	-18.22500	37.26600	-25.17800
O	-18.38500	36.60000	-30.00600
H	-19.03800	36.28400	-30.63000
H	-18.42700	35.97700	-29.28000
O	-11.81800	34.30000	-27.97500
H	-11.51200	33.44000	-28.26200
H	-11.09300	34.89200	-28.17500
O	-15.73800	38.79500	-20.12800
H	-16.38500	39.49400	-20.22200
H	-14.96800	39.22900	-19.76100
O	-13.99000	39.76300	-27.62400
H	-13.49300	39.01000	-27.94400
H	-14.76100	39.79900	-28.19000
O	-11.54800	36.73000	-25.47700
H	-12.46200	36.96000	-25.30800
H	-11.11900	36.81300	-24.62500
O	-17.56900	36.63200	-23.67800
H	-16.74700	36.15800	-23.80000
H	-17.32600	37.41100	-23.17800
O	-15.91300	36.88100	-27.41600
H	-15.89100	36.94500	-28.37000
H	-15.49600	36.04200	-27.22200
O	-14.36400	37.68000	-22.07200
H	-13.84600	38.41600	-22.39700
H	-14.71900	37.98600	-21.23700
O	-14.11300	34.10000	-26.55200
H	-13.83300	33.43200	-25.92600
H	-13.32800	34.29300	-27.06500
O	-12.22100	39.60700	-22.61900
H	-12.81600	39.88800	-23.31400
H	-12.50600	40.09700	-21.84700
O	-7.61400	42.53400	-23.64500
H	-8.13000	41.74900	-23.83200

H	-6.73000	42.20500	-23.48000
O	-9.95300	36.27800	-27.68500
H	-10.15000	35.96900	-26.80100
H	-9.34300	37.00400	-27.55500
O	-16.12400	37.08100	-31.55800
H	-16.93400	36.73500	-31.18200
H	-15.43600	36.77100	-30.96900
O	-14.19500	36.15900	-29.93600
H	-13.64200	36.57800	-29.27700
H	-13.95800	35.23200	-29.89300
O	-10.81700	38.26900	-30.42800
H	-11.39800	38.92700	-30.81000
H	-11.08400	37.44600	-30.83900
O	-12.36700	37.66900	-28.22600
H	-11.86700	37.78100	-29.03400
H	-11.72400	37.36500	-27.58500
O	-12.03500	40.42900	-31.37200
H	-12.92200	40.71400	-31.59000
H	-11.62000	40.26800	-32.21900
O	-8.68400	40.45300	-27.65700
H	-7.90800	40.94300	-27.38400
H	-9.40900	40.87900	-27.19900
O	-6.24600	41.16600	-26.59900
H	-5.65200	40.92600	-27.31000
H	-6.17400	40.44700	-25.97200
O	-14.20300	33.23000	-30.02900
H	-14.96900	32.67500	-29.88300
H	-13.79200	33.30200	-29.16700
O	-40.86000	-3.94300	-33.56500
H	-41.15100	-3.03900	-33.68000
H	-40.22400	-3.90200	-32.85000
O	-40.17300	-7.51100	-31.38500
H	-39.43100	-6.97300	-31.66100
H	-40.87900	-6.88500	-31.22700
O	-34.69700	-5.36800	-32.78700
H	-34.28900	-5.81000	-33.53200
H	-35.41900	-5.94300	-32.53400
O	-32.12600	-3.35000	-36.90600
H	-31.30400	-3.75200	-37.18800
H	-32.12900	-3.45300	-35.95400
O	-37.01800	-1.51200	-30.75200
H	-37.46200	-2.29800	-31.07000
H	-36.35700	-1.32400	-31.41800
O	-34.92000	-1.23200	-32.72400
H	-34.93800	-0.41100	-33.21600
H	-33.99800	-1.35600	-32.49900
O	-38.46300	-3.71700	-31.88200
H	-37.93000	-4.47900	-32.11200
H	-38.99100	-4.01000	-31.13900
O	-29.07000	-4.74300	-35.90300
H	-28.87600	-3.83800	-35.65700
H	-29.17000	-5.20200	-35.06900
O	-34.00900	-4.59200	-30.32900
H	-34.16000	-4.82100	-31.24600
H	-34.01800	-3.63500	-30.31600
O	-32.29900	-1.56000	-32.25100
H	-32.35800	-2.24400	-32.91800
H	-31.89700	-0.81800	-32.70300
O	-32.29800	-3.59600	-34.02000
H	-33.18500	-3.93400	-33.89700
H	-31.72800	-4.32800	-33.78300
O	-36.30800	0.96300	-34.89800
H	-35.59500	1.59700	-34.97000
H	-36.79900	1.05900	-35.71400
O	-41.25600	-1.28800	-34.07700
H	-40.55500	-0.65400	-33.92400
H	-41.72700	-0.94700	-34.83800
O	-37.40800	-7.99200	-29.76500
H	-38.08300	-8.09200	-29.09500
H	-36.58500	-8.14800	-29.30200
O	-38.66500	3.31500	-35.53000
H	-38.11300	3.47800	-34.76500
H	-38.32500	3.90600	-36.20200
O	-33.81400	-1.85900	-29.67500
H	-33.27000	-1.24600	-30.16900
H	-34.39600	-1.30100	-29.15800
O	-33.46500	-0.93600	-36.91500
H	-34.41200	-1.05500	-36.98700
H	-33.11700	-1.82400	-36.83300
O	-38.51900	0.88100	-33.51500
H	-38.21000	0.63600	-32.64200
H	-37.72100	1.08600	-34.00300
O	-30.49100	-2.39900	-30.33900
H	-31.28200	-2.41700	-30.87800
H	-30.72800	-1.86000	-29.58400
O	-30.37200	-5.55000	-33.02500
H	-30.32200	-5.71500	-32.08400
H	-30.48000	-6.41700	-33.41600
O	-33.08100	-9.56700	-31.70900
H	-32.18000	-9.70600	-32.00100
H	-33.00600	-9.40100	-30.77000
O	-28.87000	-3.13000	-32.78100
H	-29.16100	-2.76700	-31.94500
H	-29.48200	-3.84400	-32.96000
O	-30.62100	-8.00700	-34.31300
H	-30.14600	-7.81400	-35.12100
H	-31.54400	-7.95100	-34.55900
O	-23.62400	-6.84800	-37.82500
H	-22.89400	-7.11000	-37.26500
H	-24.23400	-7.58500	-37.78500
O	-37.72000	-4.98800	-29.46300
H	-36.80000	-4.81100	-29.26900
H	-37.75500	-5.93300	-29.61500
O	-39.85100	-11.50500	-27.43100
H	-39.95100	-10.55600	-27.50000
H	-40.06900	-11.83300	-28.30300
O	-39.98800	-8.07500	-28.61400
H	-40.47100	-7.27500	-28.40800
H	-39.90200	-8.06400	-29.56700
O	-35.69200	-4.21300	-27.81200
H	-34.82200	-4.03900	-28.17300
H	-36.17100	-3.39300	-27.93400
O	-24.03200	7.49600	-38.40700
H	-23.39400	7.37700	-39.11100
H	-24.15000	8.44400	-38.34900
O	-27.22300	7.44900	-38.47100
H	-27.56200	7.33300	-39.35900
H	-26.35400	7.04800	-38.49100

O	-26.74100	8.60200	-32.51300
H	-26.75300	8.20800	-33.38500
H	-26.57900	7.86900	-31.91900
O	-30.02100	11.38600	-33.56500
H	-30.31200	12.29100	-33.68000
H	-29.38500	11.42800	-32.85000
O	-29.09500	8.76400	-36.97500
H	-28.49900	8.53900	-37.68900
H	-29.03800	8.02200	-36.37200
O	-24.47800	5.22100	-41.33200
H	-23.69200	4.80300	-41.68300
H	-24.21400	6.12600	-41.16400
O	-19.07700	5.15900	-40.42900
H	-18.34500	4.58900	-40.19500
H	-19.83300	4.77100	-39.98800
O	-21.55500	-1.12900	-35.96900
H	-21.88600	-0.26000	-36.19300
H	-22.15000	-1.44500	-35.29000
O	-25.06200	11.07200	-36.57000
H	-25.36200	11.97500	-36.67800
H	-25.40700	10.80500	-35.71800
O	-21.66200	3.59000	-38.89000
H	-22.57000	3.52200	-39.18400
H	-21.71300	4.06800	-38.06200
O	-27.58800	5.00900	-30.94600
H	-28.36500	5.56900	-30.95600
H	-26.94900	5.49500	-30.42500
O	-19.79600	1.79200	-37.11200
H	-20.43800	2.08500	-37.75800
H	-20.30000	1.67700	-36.30700
O	-29.33300	7.81800	-31.38500
H	-28.59200	8.35600	-31.66100
H	-30.04000	8.44500	-31.22700
O	-23.85800	9.96100	-32.78700
H	-23.44900	9.51900	-33.53200
H	-24.58000	9.38600	-32.53400
O	-27.50500	4.89800	-35.41400
H	-26.65800	5.32200	-35.27100
H	-27.56500	4.24100	-34.72000
O	-23.53500	10.52100	-39.03200
H	-24.16400	10.68600	-39.73500
H	-23.92200	10.93500	-38.26100
O	-19.65600	11.45600	-41.67800
H	-20.34100	11.83600	-42.22800
H	-19.57200	12.06600	-40.94500
O	-24.35500	2.55900	-33.75600
H	-24.59900	3.31300	-34.29300
H	-24.07900	2.93700	-32.92100
O	-22.40100	9.10200	-35.07800
H	-22.18900	9.74600	-35.75500
H	-22.44800	8.26900	-35.54600
O	-24.79800	1.71400	-38.17500
H	-25.66000	2.01200	-37.88300
H	-24.77200	1.93100	-39.10600
O	-30.59700	4.97200	-30.58300
H	-31.22700	5.65400	-30.35000
H	-30.70300	4.86000	-31.52800
O	-21.28600	11.98000	-36.90600
H	-20.46400	11.57800	-37.18800
H	-21.29000	11.87600	-35.95400
O	-27.52100	11.52800	-34.75000
H	-27.45600	11.99000	-33.91400
H	-28.22900	10.89800	-34.62200
O	-13.19600	8.32300	-40.55800
H	-13.94900	7.75400	-40.71300
H	-13.06100	8.29000	-39.61000
O	-31.31900	-0.72600	-28.22100
H	-31.71200	-1.27900	-27.54600
H	-30.50200	-0.41500	-27.83000
O	-28.40200	0.69000	-30.62900
H	-27.66100	1.29000	-30.70800
H	-28.17000	-0.05200	-31.18700
O	-33.25800	5.87500	-32.35100
H	-33.37300	4.97400	-32.05000
H	-34.11400	6.12200	-32.70200
O	-26.17900	13.81700	-30.75200
H	-26.62300	13.03100	-31.07000
H	-25.51700	14.00500	-31.41800
O	-24.08100	14.09700	-32.72400
H	-24.09900	14.91800	-33.21600
H	-23.15900	13.97300	-32.49900
O	-27.62300	11.61200	-31.88200
H	-27.09100	10.85100	-32.11200
H	-28.15200	11.31900	-31.13900
O	-24.45700	-3.28200	-36.35200
H	-24.80000	-2.39200	-36.42800
H	-24.08900	-3.47200	-37.21500
O	-25.37700	-0.91200	-38.38700
H	-25.88000	-0.94900	-39.20000
H	-25.08400	-0.00200	-38.32800
O	-29.13300	-0.82800	-39.38100
H	-28.26700	-1.02400	-39.74000
H	-29.06500	-1.05700	-38.45400
O	-19.51800	9.46200	-45.91000
H	-20.29500	9.12300	-46.35400
H	-18.83900	9.46700	-46.58400
O	-21.27900	8.93500	-39.25300
H	-21.93700	9.62500	-39.16500
H	-20.47500	9.32500	-38.91000
O	-22.46800	7.29000	-41.20300
H	-21.95500	7.92300	-40.70000
H	-21.81800	6.69000	-41.56800
O	-25.99000	4.76500	-38.95700
H	-26.90800	4.98600	-39.11500
H	-25.57400	4.83900	-39.81600
O	-25.20700	6.16400	-43.82600
H	-24.46000	6.67600	-44.13600
H	-24.93800	5.84200	-42.96600
O	-21.70200	12.27000	-43.07500
H	-22.15600	11.42900	-43.01800
H	-22.33300	12.90700	-42.73700
O	-22.75900	3.20000	-41.84500
H	-23.49700	2.68700	-41.51400
H	-22.50600	2.76000	-42.65600
O	-28.38600	6.27300	-40.74300
H	-28.33100	5.32000	-40.81900
H	-29.19700	6.42900	-40.25900
O	-24.84900	5.35400	-34.15200

H	-24.49400	5.58200	-33.29300
H	-24.20200	5.68600	-34.77400
O	-19.11900	8.47600	-43.44700
H	-19.25700	8.66600	-44.37500
H	-19.47600	9.23800	-42.99000
O	-30.95500	2.46200	-37.36800
H	-30.31500	2.32100	-38.06500
H	-31.78700	2.17600	-37.74400
O	-19.09200	10.54400	-38.43600
H	-18.65900	10.45200	-37.58800
H	-18.37900	10.52000	-39.07400
O	-25.35900	14.14400	-36.90300
H	-25.30600	14.71300	-36.13500
H	-26.22800	14.31400	-37.26700
O	-32.48000	3.84600	-35.42000
H	-31.80700	3.63800	-36.06900
H	-33.30700	3.64800	-35.86000
O	-18.23100	10.58600	-35.90300
H	-18.03700	11.49100	-35.65700
H	-18.33100	10.12700	-35.06900
O	-26.57700	0.47200	-33.40400
H	-27.22400	1.17100	-33.49700
H	-25.80700	0.90600	-33.03600
O	-23.16900	10.73700	-30.32900
H	-23.32100	10.50800	-31.24600
H	-23.17900	11.69400	-30.31600
O	-26.40100	8.31000	-35.34400
H	-25.47100	8.46100	-35.51700
H	-26.82200	8.39900	-36.19900
O	-27.60200	12.33500	-37.57400
H	-27.74100	11.96700	-36.70200
H	-28.39000	12.85000	-37.74600
O	-26.05100	11.71300	-40.18500
H	-26.38600	11.67400	-39.29000
H	-26.55100	11.04800	-40.65800
O	-32.07700	7.12000	-34.66000
H	-32.21500	6.38800	-34.05900
H	-31.16600	7.03100	-34.94200
O	-29.44900	6.83200	-35.17200
H	-28.73900	6.19200	-35.21300
H	-29.49200	7.08300	-34.24900
O	-21.46000	13.76900	-32.25100
H	-21.51900	13.08500	-32.91800
H	-21.05800	14.51100	-32.70300
O	-30.73600	3.81900	-33.35200
H	-31.27200	3.36800	-32.70000
H	-31.32400	3.95300	-34.09500
O	-21.45800	11.73300	-34.02000
H	-22.34600	11.39500	-33.89700
H	-20.88800	11.00100	-33.78300
O	-35.00200	2.95400	-36.26700
H	-35.08600	2.34700	-37.00200
H	-35.82400	3.44500	-36.26600
O	-24.82900	1.44000	-40.90000
H	-24.33300	0.68700	-41.22000
H	-25.60100	1.47600	-41.46600
O	-16.97700	7.14400	-39.25800
H	-16.98800	8.02600	-39.62900
H	-17.84500	6.79100	-39.45300
O	-25.46800	16.29200	-34.89800
H	-24.75500	16.92600	-34.97000
H	-25.95900	16.38800	-35.71400
O	-23.74700	10.19700	-43.32600
H	-24.68100	10.31700	-43.49600
H	-23.56900	9.29400	-43.59000
O	-28.24600	3.58100	-41.17300
H	-28.61600	3.02900	-40.48500
H	-27.75000	2.97700	-41.72500
O	-20.35900	6.03900	-42.79100
H	-19.89300	6.87100	-42.86500
H	-19.90800	5.57300	-42.08700
O	-30.41700	14.04100	-34.07700
H	-29.71600	14.67500	-33.92400
H	-30.88800	14.38300	-34.83800
O	-22.38800	-1.59300	-38.75200
H	-23.30100	-1.36300	-38.58400
H	-21.95800	-1.51000	-37.90100
O	-22.61700	6.59600	-36.25200
H	-23.27000	6.84300	-36.90700
H	-22.23900	5.78200	-36.58600
O	-26.56800	7.33800	-29.76500
H	-27.24400	7.23700	-29.09500
H	-25.74500	7.18200	-29.30200
O	-27.82600	18.64400	-35.53000
H	-27.27400	18.80700	-34.76500
H	-27.48500	19.23500	-36.20200
O	-32.84900	0.99200	-34.92700
H	-32.78500	1.85300	-35.34000
H	-32.91300	0.37900	-35.66000
O	-33.37400	3.06400	-31.28000
H	-32.74700	2.68200	-30.66600
H	-34.23100	2.84600	-30.91500
O	-22.97500	13.47000	-29.67500
H	-22.43100	14.08300	-30.16900
H	-23.55600	14.02800	-29.15800
O	-24.68900	16.59300	-38.54300
H	-24.81400	15.79100	-39.05200
H	-24.03100	17.08700	-39.03200
O	-33.06800	2.94700	-39.36600
H	-33.79000	3.56100	-39.50500
H	-32.27900	3.48300	-39.44400
O	-22.62600	14.39300	-36.91500
H	-23.57300	14.27400	-36.98700
H	-22.27700	13.50600	-36.83300
O	-30.26600	0.28300	-33.09100
H	-29.74200	0.52900	-32.32900
H	-30.48400	1.11500	-33.51100
O	-25.62100	14.18900	-40.71500
H	-25.76800	13.26000	-40.53400
H	-24.79600	14.21100	-41.19900
O	-22.01800	15.01200	-39.62800
H	-21.09300	14.80600	-39.76600
H	-22.18600	14.76300	-38.71900
O	-29.06000	1.22400	-35.40700
H	-29.40100	0.42500	-35.00400
H	-29.76600	1.52400	-35.97900
O	-28.40900	-1.69100	-36.95400
H	-27.58600	-2.16500	-37.07600

H	-28.16500	-0.91200	-36.45400
O	-30.88500	5.00100	-39.14500
H	-31.03000	5.31500	-38.25200
H	-31.36200	5.62000	-39.69700
O	-29.02500	1.99100	-39.09600
H	-29.03900	1.09500	-39.43300
H	-28.30800	1.99800	-38.46300
O	-27.17300	2.42500	-37.05100
H	-27.43100	3.34700	-37.05300
H	-27.70400	2.02800	-36.36100
O	-16.37400	8.29400	-42.65300
H	-15.93100	7.54400	-43.05100
H	-17.25100	8.28500	-43.03600
O	-35.62400	6.66900	-33.31300
H	-36.36600	6.81700	-32.72600
H	-35.94400	6.92900	-34.17600
O	-19.34100	12.29700	-45.64400
H	-18.81600	12.63500	-46.36900
H	-19.42900	11.36100	-45.82700
O	-27.67900	16.21000	-33.51500
H	-27.37100	15.96500	-32.64200
H	-26.88200	16.41500	-34.00300
O	-30.13700	2.38600	-29.61900
H	-30.08900	3.26200	-30.00100
H	-29.59500	1.84400	-30.19200
O	-19.65200	12.93000	-30.33900
H	-20.44200	12.91200	-30.87800
H	-19.88900	13.46900	-29.58400
O	-19.53200	9.77900	-33.02500
H	-19.48200	9.61400	-32.08400
H	-19.64100	8.91200	-33.41600
O	-22.24200	5.76200	-31.70900
H	-21.34100	5.62300	-32.00100
H	-22.16700	5.92800	-30.77000
O	-27.90700	3.13900	-32.86400
H	-28.77700	3.43200	-33.13600
H	-27.67200	3.72300	-32.14300
O	-18.03000	12.19900	-32.78100
H	-18.32200	12.56200	-31.94500
H	-18.64300	11.48600	-32.96000
O	-16.83100	9.76400	-40.26100
H	-15.99600	10.20300	-40.09700
H	-16.69500	9.28800	-41.08000
O	-19.78200	7.32200	-34.31300
H	-19.30600	7.51500	-35.12100
H	-20.70500	7.37800	-34.55900
O	-25.20400	-0.64300	-35.34800
H	-24.68500	0.09300	-35.67300
H	-25.55800	-0.33700	-34.51300
O	-24.95200	-4.22300	-39.82700
H	-24.67200	-4.89100	-39.20100
H	-24.16700	-4.03000	-40.34000
O	-21.08100	4.19900	-36.08000
H	-20.13000	4.29900	-36.05900
H	-21.25100	3.40700	-35.57100
O	-23.06000	1.28400	-35.89400
H	-23.65500	1.56500	-36.59000
H	-23.34500	1.77400	-35.12300
O	-18.60300	7.41900	-36.96600
H	-18.63500	6.48100	-36.78000
H	-17.99300	7.49800	-37.69900
O	-18.45300	4.21100	-36.92000
H	-18.96900	3.42600	-37.10700
H	-17.56900	3.88200	-36.75600
O	-20.79200	-2.04500	-40.96100
H	-20.99000	-2.35400	-40.07600
H	-20.18200	-1.31900	-40.83100
O	-12.78500	8.48100	-37.82500
H	-12.05500	8.22000	-37.26500
H	-13.39400	7.74400	-37.78500
O	-14.70300	6.62200	-37.22800
H	-15.43000	6.48700	-37.83600
H	-15.11900	6.92900	-36.42200
O	-26.88000	10.34100	-29.46300
H	-25.96000	10.51900	-29.26900
H	-26.91500	9.39600	-29.61500
O	-33.88400	8.25100	-36.82100
H	-33.66600	9.18300	-36.80200
H	-33.47200	7.89200	-36.03600
O	-29.01100	3.82400	-27.43100
H	-29.11200	4.77400	-27.50000
H	-29.22900	3.49600	-28.30300
O	-23.20700	-0.65400	-41.50100
H	-22.70700	-0.54200	-42.31000
H	-22.56300	-0.95800	-40.86100
O	-29.14800	7.25400	-28.61400
H	-29.63200	8.05400	-28.40800
H	-29.06200	7.26500	-29.56700
O	-32.63100	9.71500	-34.01000
H	-31.81200	10.20900	-33.98700
H	-32.35700	8.79800	-34.03900
O	-19.52300	2.13000	-40.93200
H	-18.74700	2.62000	-40.66000
H	-20.24800	2.55600	-40.47500
O	-17.08600	2.84300	-39.87500
H	-16.49100	2.60300	-40.58500
H	-17.01300	2.12400	-39.24700
O	-22.90000	7.62100	-44.43200
H	-22.95700	8.07800	-45.27200
H	-22.34700	6.86000	-44.61000
O	-19.09000	3.41000	-43.60700
H	-19.34600	3.01000	-42.77700
H	-19.71500	4.12400	-43.73200
O	-14.77900	6.15000	-41.10000
H	-15.33900	6.21900	-40.32700
H	-15.38400	5.97400	-41.82000
O	-37.37800	4.04800	-33.31100
H	-36.73200	3.53800	-32.82200
H	-37.58600	4.78500	-32.73700
O	-24.85200	11.11600	-27.81200
H	-23.98300	11.29000	-28.17300
H	-25.33200	11.93600	-27.93400
O	-33.03800	1.19500	-29.21400
H	-32.17500	0.83900	-29.00300
H	-33.61100	0.85600	-28.52600
O	-13.19300	22.82500	-38.40700
H	-12.55500	22.70600	-39.11100
H	-13.31100	23.77300	-38.34900

O	-16.38400	22.77800	-38.47100
H	-16.72200	22.66200	-39.35900
H	-15.51500	22.37700	-38.49100
O	-15.90200	23.93100	-32.51300
H	-15.91300	23.53700	-33.38500
H	-15.74000	23.19800	-31.91900
O	-19.18100	26.71500	-33.56500
H	-19.47200	27.62000	-33.68000
H	-18.54600	26.75700	-32.85000
O	-18.25600	24.09300	-36.97500
H	-17.65900	23.86800	-37.68900
H	-18.19800	23.35100	-36.37200
O	-13.63900	20.55000	-41.33200
H	-12.85300	20.13200	-41.68300
H	-13.37500	21.45500	-41.16400
O	-8.23800	20.48800	-40.42900
H	-7.50500	19.91800	-40.19500
H	-8.99400	20.10000	-39.98800
O	-10.71500	14.20000	-35.96900
H	-11.04700	15.07000	-36.19300
H	-11.31100	13.88400	-35.29000
O	-14.22200	26.40100	-36.57000
H	-14.52200	27.30400	-36.67800
H	-14.56700	26.13500	-35.71800
O	-10.82300	18.92000	-38.89000
H	-11.73100	18.85100	-39.18400
H	-10.87400	19.39700	-38.06200
O	-16.74800	20.33900	-30.94600
H	-17.52500	20.89800	-30.95600
H	-16.10900	20.82400	-30.42500
O	-8.95600	17.12100	-37.11200
H	-9.59900	17.41500	-37.75800
H	-9.46100	17.00700	-36.30700
O	-18.49400	23.14700	-31.38500
H	-17.75200	23.68600	-31.66100
H	-19.20000	23.77400	-31.22700
O	-13.01800	25.29000	-32.78700
H	-12.61000	24.84800	-33.53200
H	-13.74100	24.71500	-32.53400
O	-16.66500	20.22700	-35.41400
H	-15.81900	20.65100	-35.27100
H	-16.72500	19.57100	-34.72000
O	-12.69500	25.85000	-39.03200
H	-13.32500	26.01500	-39.73500
H	-13.08300	26.26400	-38.26100
O	-13.51500	17.88800	-33.75600
H	-13.75900	18.64200	-34.29300
H	-13.23900	18.26600	-32.92100
O	-11.56100	24.43200	-35.07800
H	-11.35000	25.07500	-35.75500
H	-11.60800	23.59800	-35.54600
O	-13.95900	17.04300	-38.17500
H	-14.82000	17.34100	-37.88300
H	-13.93300	17.26000	-39.10600
O	-19.75800	20.30100	-30.58300
H	-20.38800	20.98300	-30.35000
H	-19.86400	20.18900	-31.52800
O	-10.44700	27.30900	-36.90600
H	-9.62500	26.90700	-37.18800
H	-10.45100	27.20600	-35.95400
O	-16.68100	26.85700	-34.75000
H	-16.61700	27.31900	-33.91400
H	-17.39000	26.22700	-34.62200
O	-20.48000	14.60300	-28.22100
H	-20.87300	14.05000	-27.54600
H	-19.66300	14.91400	-27.83000
O	-17.56300	16.01900	-30.62900
H	-16.82200	16.62000	-30.70800
H	-17.33100	15.27700	-31.18700
O	-22.41900	21.20500	-32.35100
H	-22.53300	20.30300	-32.05000
H	-23.27400	21.45100	-32.70200
O	-15.34000	29.14600	-30.75200
H	-15.78300	28.36000	-31.07000
H	-14.67800	29.33400	-31.41800
O	-13.24100	29.42700	-32.72400
H	-13.26000	30.24800	-33.21600
H	-12.31900	29.30200	-32.49900
O	-16.78400	26.94100	-31.88200
H	-16.25100	26.18000	-32.11200
H	-17.31300	26.64800	-31.13900
O	-13.61700	12.04700	-36.35200
H	-13.96100	12.93700	-36.42800
H	-13.24900	11.85700	-37.21500
O	-16.06700	16.88300	-42.78600
H	-16.92300	16.49400	-42.96600
H	-15.65500	16.96200	-43.64600
O	-14.53700	14.41800	-38.38700
H	-15.04100	14.38100	-39.20000
H	-14.24400	15.32700	-38.32800
O	-18.29300	14.50100	-39.38100
H	-17.42800	14.30600	-39.74000
H	-18.22500	14.27200	-38.45400
O	-13.86300	19.91100	-46.01900
H	-14.16800	20.22500	-45.16800
H	-12.91000	19.98900	-45.97600
O	-10.44000	24.26500	-39.25300
H	-11.09700	24.95400	-39.16500
H	-9.63600	24.65400	-38.91000
O	-20.68700	16.73200	-42.00900
H	-20.11300	16.50900	-42.74200
H	-20.10000	16.80000	-41.25600
O	-11.62900	22.61900	-41.20300
H	-11.11600	23.25200	-40.70000
H	-10.97900	22.01900	-41.56800
O	-18.38500	13.60600	-43.28100
H	-19.03800	13.29100	-43.90600
H	-18.42700	12.98300	-42.55500
O	-15.15100	20.09400	-38.95700
H	-16.06900	20.31500	-39.11500
H	-14.73500	20.16900	-39.81600
O	-14.36700	21.49300	-43.82600
H	-13.62000	22.00500	-44.13600
H	-14.09800	21.17100	-42.96600
O	-11.92000	18.52900	-41.84500
H	-12.65700	18.01700	-41.51400
H	-11.66700	18.08900	-42.65600
O	-11.81800	11.30600	-41.25000

H	-11.51200	10.44600	-41.53800
H	-11.09300	11.89800	-41.45000
O	-17.54600	21.60200	-40.74300
H	-17.49100	20.64900	-40.81900
H	-18.35700	21.75800	-40.25900
O	-14.01000	20.68400	-34.15200
H	-13.65500	20.91100	-33.29300
H	-13.36200	21.01600	-34.77400
O	-20.11600	17.79100	-37.36800
H	-19.47600	17.65000	-38.06500
H	-20.94800	17.50500	-37.74400
O	-8.25300	25.87300	-38.43600
H	-7.81900	25.78100	-37.58800
H	-7.54000	25.84900	-39.07400
O	-14.52000	29.47300	-36.90300
H	-14.46600	30.04200	-36.13500
H	-15.38900	29.64400	-37.26700
O	-21.64000	19.17500	-35.42000
H	-20.96800	18.96700	-36.06900
H	-22.46700	18.97700	-35.86000
O	-7.39100	25.91600	-35.90300
H	-7.19800	26.82000	-35.65700
H	-7.49100	25.45600	-35.06900
O	-15.73800	15.80100	-33.40400
H	-16.38500	16.50000	-33.49700
H	-14.96800	16.23500	-33.03600
O	-12.33000	26.06600	-30.32900
H	-12.48100	25.83700	-31.24600
H	-12.33900	27.02300	-30.31600
O	-15.56100	23.63900	-35.34400
H	-14.63200	23.79100	-35.51700
H	-15.98200	23.72800	-36.19900
O	-16.76200	27.66400	-37.57400
H	-16.90100	27.29600	-36.70200
H	-17.55100	28.17900	-37.74600
O	-15.21200	27.04300	-40.18500
H	-15.54700	27.00300	-39.29000
H	-15.71200	26.37700	-40.65800
O	-21.23800	22.44900	-34.66000
H	-21.37600	21.71700	-34.05900
H	-20.32700	22.36000	-34.94200
O	-18.60900	22.16100	-35.17200
H	-17.89900	21.52100	-35.21300
H	-18.65200	22.41300	-34.24900
O	-10.62100	29.09800	-32.25100
H	-10.67900	28.41400	-32.91800
H	-10.21900	29.84000	-32.70300
O	-19.89600	19.14800	-33.35200
H	-20.43300	18.69700	-32.70000
H	-20.48500	19.28300	-34.09500
O	-10.61900	27.06200	-34.02000
H	-11.50600	26.72500	-33.89700
H	-10.04900	26.33000	-33.78300
O	-24.16200	18.28400	-36.26700
H	-24.24700	17.67600	-37.00200
H	-24.98400	18.77400	-36.26600
O	-13.99000	16.76900	-40.90000
H	-13.49300	16.01600	-41.22000
H	-14.76100	16.80500	-41.46600
O	-6.13700	22.47300	-39.25800
H	-6.14800	23.35500	-39.62900
H	-7.00500	22.12000	-39.45300
O	-14.62900	31.62100	-34.89800
H	-13.91600	32.25600	-34.97000
H	-15.12000	31.71700	-35.71400
O	-17.40700	18.91100	-41.17300
H	-17.77700	18.35800	-40.48500
H	-16.91100	18.30600	-41.72500
O	-9.52000	21.36800	-42.79100
H	-9.05400	22.20100	-42.86500
H	-9.06900	20.90200	-42.08700
O	-19.57800	29.37000	-34.07700
H	-18.87700	30.00400	-33.92400
H	-20.04800	29.71200	-34.83800
O	-11.54800	13.73600	-38.75200
H	-12.46200	13.96600	-38.58400
H	-11.11900	13.82000	-37.90100
O	-11.77800	21.92500	-36.25200
H	-12.43000	22.17200	-36.90700
H	-11.40000	21.11100	-36.58600
O	-15.72900	22.66700	-29.76500
H	-16.40500	22.56600	-29.09500
H	-14.90600	22.51100	-29.30200
O	-16.98700	33.97300	-35.53000
H	-16.43500	34.13700	-34.76500
H	-16.64600	34.56400	-36.20200
O	-22.01000	16.32100	-34.92700
H	-21.94600	17.18200	-35.34000
H	-22.07300	15.70800	-35.66000
O	-22.53500	18.39300	-31.28000
H	-21.90800	18.01100	-30.66600
H	-23.39200	18.17500	-30.91500
O	-12.13500	28.80000	-29.67500
H	-11.59100	29.41300	-30.16900
H	-12.71700	29.35800	-29.15800
O	-13.85000	31.92200	-38.54300
H	-13.97500	31.12100	-39.05200
H	-13.19200	32.41600	-39.03200
O	-22.22900	18.27700	-39.36600
H	-22.95000	18.89000	-39.50500
H	-21.44000	18.81200	-39.44400
O	-11.78600	29.72300	-36.91500
H	-12.73300	29.60300	-36.98700
H	-11.43800	28.83500	-36.83300
O	-19.42600	15.61200	-33.09100
H	-18.90300	15.85800	-32.32900
H	-19.64500	16.44400	-33.51100
O	-14.78200	29.51800	-40.71500
H	-14.92900	28.59000	-40.53400
H	-13.95600	29.54000	-41.19900
O	-18.22100	16.55300	-35.40700
H	-18.56100	15.75400	-35.00400
H	-18.92700	16.85400	-35.97900
O	-17.56900	13.63800	-36.95400
H	-16.74700	13.16400	-37.07600
H	-17.32600	14.41700	-36.45400
O	-14.78500	17.37200	-45.72200
H	-14.56900	18.25400	-46.02300

H	-15.26600	16.98000	-46.45100
O	-20.04500	20.33000	-39.14500
H	-20.19100	20.64400	-38.25200
H	-20.52300	20.94900	-39.69700
O	-18.18600	17.32000	-39.09600
H	-18.20000	16.42500	-39.43300
H	-17.46900	17.32800	-38.46300
O	-16.33300	17.75400	-37.05100
H	-16.59100	18.67600	-37.05300
H	-16.86500	17.35700	-36.36100
O	-18.78200	16.20200	-43.82800
H	-18.66100	15.27400	-43.62900
H	-19.07200	16.21600	-44.74000
O	-15.91300	13.88700	-40.69100
H	-15.89100	13.95100	-41.64600
H	-15.49600	13.04800	-40.49700
O	-24.78400	21.99800	-33.31300
H	-25.52600	22.14600	-32.72600
H	-25.10500	22.25900	-34.17600
O	-16.84000	31.53900	-33.51500
H	-16.53100	31.29400	-32.64200
H	-16.04200	31.74400	-34.00300
O	-19.29800	17.71500	-29.61900
H	-19.24900	18.59100	-30.00100
H	-18.75600	17.17300	-30.19200
O	-8.81300	28.25900	-30.33900
H	-9.60300	28.24200	-30.87800
H	-9.04900	28.79800	-29.58400
O	-8.69300	25.10800	-33.02500
H	-8.64300	24.94300	-32.08400
H	-8.80100	24.24100	-33.41600
O	-11.40200	21.09200	-31.70900
H	-10.50100	20.95200	-32.00100
H	-11.32800	21.25700	-30.77000
O	-17.06800	18.46800	-32.86400
H	-17.93800	18.76200	-33.13600
H	-16.83200	19.05200	-32.14300
O	-7.19100	27.52800	-32.78100
H	-7.48200	27.89100	-31.94500
H	-7.80400	26.81500	-32.96000
O	-8.94200	22.65100	-34.31300
H	-8.46700	22.84400	-35.12100
H	-9.86600	22.70700	-34.55900
O	-14.36400	14.68600	-35.34800
H	-13.84600	15.42200	-35.67300
H	-14.71900	14.99300	-34.51300
O	-14.11300	11.10600	-39.82700
H	-13.83300	10.43900	-39.20100
H	-13.32800	11.29900	-40.34000
O	-10.24200	19.52800	-36.08000
H	-9.29000	19.62800	-36.05900
H	-10.41200	18.73600	-35.57100
O	-12.22100	16.61400	-35.89400
H	-12.81600	16.89400	-36.59000
H	-12.50600	17.10300	-35.12300
O	-7.76300	22.74900	-36.96600
H	-7.79600	21.81000	-36.78000
H	-7.15300	22.82700	-37.69900
O	-7.61400	19.54000	-36.92000
H	-8.13000	18.75600	-37.10700
H	-6.73000	19.21200	-36.75600
O	-9.95300	13.28400	-40.96100
H	-10.15000	12.97500	-40.07600
H	-9.34300	14.01000	-40.83100
O	-1.94600	23.81000	-37.82500
H	-1.21500	23.54900	-37.26500
H	-2.55500	23.07300	-37.78500
O	-3.86400	21.95100	-37.22800
H	-4.59100	21.81700	-37.83600
H	-4.27900	22.25900	-36.42200
O	-16.04100	25.67000	-29.46300
H	-15.12100	25.84800	-29.26900
H	-16.07600	24.72600	-29.61500
O	-23.04500	23.58000	-36.82100
H	-22.82600	24.51200	-36.80200
H	-22.63200	23.22100	-36.03600
O	-16.12400	14.08700	-44.83300
H	-16.93400	13.74200	-44.45800
H	-15.43600	13.77800	-44.24500
O	-14.19500	13.16500	-43.21100
H	-13.64200	13.58400	-42.55200
H	-13.95800	12.23800	-43.16900
O	-18.17200	19.15300	-27.43100
H	-18.27200	20.10300	-27.50000
H	-18.39000	18.82500	-28.30300
O	-10.81700	15.27500	-43.70300
H	-11.39800	15.93300	-44.08500
H	-11.08400	14.45300	-44.11400
O	-12.36700	14.67500	-41.50100
H	-11.86700	14.78700	-42.31000
H	-11.72400	14.37100	-40.86100
O	-18.30900	22.58300	-28.61400
H	-18.79200	23.38300	-28.40800
H	-18.22300	22.59400	-29.56700
O	-21.79200	25.04400	-34.01000
H	-20.97200	25.53800	-33.98700
H	-21.51800	24.12700	-34.03900
O	-12.03500	17.43500	-44.64800
H	-12.92200	17.72000	-44.86500
H	-11.62000	17.27400	-45.49500
O	-8.68400	17.45900	-40.93200
H	-7.90800	17.94900	-40.66000
H	-9.40900	17.88500	-40.47500
O	-6.24600	18.17200	-39.87500
H	-5.65200	17.93200	-40.58500
H	-6.17400	17.45300	-39.24700
O	-8.25000	18.73900	-43.60700
H	-8.50700	18.33900	-42.77700
H	-8.87500	19.45400	-43.73200
O	-3.93900	21.47900	-41.10000
H	-4.49900	21.54800	-40.32700
H	-4.54500	21.30300	-41.82000
O	-26.53800	19.37800	-33.31100
H	-25.89200	18.86700	-32.82200
H	-26.74600	20.11400	-32.73700
O	-14.01300	26.44600	-27.81200
H	-13.14400	26.62000	-28.17300
H	-14.49200	27.26500	-27.93400

O	-14.20300	10.23600	-43.30400
H	-14.96900	9.68200	-43.15800
H	-13.79200	10.30800	-42.44300
O	-22.19800	16.52400	-29.21400
H	-21.33500	16.16800	-29.00300
H	-22.77200	16.18600	-28.52600
O	-5.90900	35.66800	-30.94600
H	-6.68600	36.22700	-30.95600
H	-5.27000	36.15300	-30.42500
O	-7.65400	38.47600	-31.38500
H	-6.91300	39.01500	-31.66100
H	-8.36100	39.10300	-31.22700
O	-8.91800	35.63000	-30.58300
H	-9.54800	36.31200	-30.35000
H	-9.02500	35.51900	-31.52800
O	-9.64000	29.93200	-28.22100
H	-10.03400	29.37900	-27.54600
H	-8.82400	30.24300	-27.83000
O	-6.72300	31.34800	-30.62900
H	-5.98300	31.94900	-30.70800
H	-6.49200	30.60600	-31.18700
O	-11.57900	36.53400	-32.35100
H	-11.69400	35.63200	-32.05000
H	-12.43500	36.78000	-32.70200
O	-2.77800	27.37600	-36.35200
H	-3.12100	28.26600	-36.42800
H	-2.41000	27.18600	-37.21500
O	-10.80100	34.50400	-35.42000
H	-10.12900	34.29600	-36.06900
H	-11.62800	34.30700	-35.86000
O	-4.89800	31.13100	-33.40400
H	-5.54600	31.83000	-33.49700
H	-4.12800	31.56400	-33.03600
O	-9.05700	34.47700	-33.35200
H	-9.59300	34.02600	-32.70000
H	-9.64500	34.61200	-34.09500
O	-13.32300	33.61300	-36.26700
H	-13.40700	33.00500	-37.00200
H	-14.14500	34.10400	-36.26600
O	-4.88900	37.99600	-29.76500
H	-5.56500	37.89500	-29.09500
H	-4.06700	37.84000	-29.30200
O	-11.17100	31.65000	-34.92700
H	-11.10700	32.51200	-35.34000
H	-11.23400	31.03800	-35.66000
O	-11.69500	33.72300	-31.28000
H	-11.06800	33.34000	-30.66600
H	-12.55300	33.50400	-30.91500
O	-8.58700	30.94100	-33.09100
H	-8.06300	31.18700	-32.32900
H	-8.80600	31.77300	-33.51100
O	-7.38200	31.88300	-35.40700
H	-7.72200	31.08300	-35.00400
H	-8.08800	32.18300	-35.97900
O	-6.73000	28.96800	-36.95400
H	-5.90700	28.49400	-37.07600
H	-6.48600	29.74700	-36.45400
O	-13.94500	37.32700	-33.31300
H	-14.68700	37.47500	-32.72600
H	-14.26600	37.58800	-34.17600
O	-8.45900	33.04400	-29.61900
H	-8.41000	33.92100	-30.00100
H	-7.91600	32.50200	-30.19200
O	-6.22900	33.79700	-32.86400
H	-7.09800	34.09100	-33.13600
H	-5.99300	34.38100	-32.14300
O	-3.52500	30.01500	-35.34800
H	-3.00700	30.75200	-35.67300
H	-3.87900	30.32200	-34.51300
O	-5.20200	40.99900	-29.46300
H	-4.28100	41.17700	-29.26900
H	-5.23700	40.05500	-29.61500
O	-7.33300	34.48300	-27.43100
H	-7.43300	35.43200	-27.50000
H	-7.55100	34.15400	-28.30300
O	-7.47000	37.91200	-28.61400
H	-7.95300	38.71200	-28.40800
H	-7.38300	37.92300	-29.56700
O	-15.69900	34.70700	-33.31100
H	-15.05300	34.19700	-32.82200
H	-15.90700	35.44400	-32.73700
O	-3.17300	41.77500	-27.81200
H	-2.30400	41.94900	-28.17300
H	-3.65300	42.59400	-27.93400
O	-11.35900	31.85300	-29.21400
H	-10.49600	31.49700	-29.00300
H	-11.93300	31.51500	-28.52600
O	-19.18100	3.72100	-46.84000
H	-19.47200	4.62600	-46.95500
H	-18.54600	3.76300	-46.12600
O	-18.49400	0.15300	-44.66000
H	-17.75200	0.69200	-44.93700
H	-19.20000	0.78000	-44.50300
O	-13.01800	2.29600	-46.06300
H	-12.61000	1.85400	-46.80700
H	-13.74100	1.72200	-45.80900
O	-10.44700	4.31500	-50.18100
H	-9.62500	3.91300	-50.46300
H	-10.45100	4.21200	-49.23000
O	-15.34000	6.15300	-44.02700
H	-15.78300	5.36600	-44.34500
H	-14.67800	6.34000	-44.69300
O	-13.24100	6.43300	-45.99900
H	-13.26000	7.25400	-46.49100
H	-12.31900	6.30800	-45.77400
O	-16.78400	3.94700	-45.15700
H	-16.25100	3.18600	-45.38700
H	-17.31300	3.65500	-44.41500
O	-7.39100	2.92200	-49.17800
H	-7.19800	3.82600	-48.93200
H	-7.49100	2.46200	-48.34400
O	-12.33000	3.07200	-43.60400
H	-12.48100	2.84300	-44.52100
H	-12.33900	4.02900	-43.59100
O	-10.62100	6.10400	-45.52700
H	-10.67900	5.42000	-46.19400
H	-10.21900	6.84600	-45.97900
O	-10.61900	4.06800	-47.29500

H	-11.50600	3.73100	-47.17300
H	-10.04900	3.33700	-47.05800
O	-14.62900	8.62700	-48.17300
H	-13.91600	9.26200	-48.24600
H	-15.12000	8.72400	-48.98900
O	-19.57800	6.37700	-47.35300
H	-18.87700	7.01000	-47.20000
H	-20.04800	6.71800	-48.11300
O	-15.72900	-0.32700	-43.04100
H	-16.40500	-0.42800	-42.37100
H	-14.90600	-0.48300	-42.57700
O	-16.98700	10.98000	-48.80500
H	-16.43500	11.14300	-48.04000
H	-16.64600	11.57000	-49.47700
O	-12.13500	5.80600	-42.95000
H	-11.59100	6.41900	-43.44500
H	-12.71700	6.36400	-42.43400
O	-11.78600	6.72900	-50.19100
H	-12.73300	6.60900	-50.26300
H	-11.43800	5.84100	-50.10800
O	-16.84000	8.54600	-46.79000
H	-16.53100	8.30000	-45.91800
H	-16.04200	8.75000	-47.27800
O	-8.81300	5.26500	-43.61400
H	-9.60300	5.24800	-44.15400
H	-9.04900	5.80400	-42.85900
O	-8.69300	2.11500	-46.30100
H	-8.64300	1.95000	-45.35900
H	-8.80100	1.24700	-46.69100
O	-11.40200	-1.90200	-44.98500
H	-10.50100	-2.04200	-45.27600
H	-11.32800	-1.73700	-44.04500
O	-7.19100	4.53400	-46.05700
H	-7.48200	4.89700	-45.22000
H	-7.80400	3.82100	-46.23500
O	-8.94200	-0.34200	-47.58900
H	-8.46700	-0.15000	-48.39700
H	-9.86600	-0.28600	-47.83400
O	-1.94600	0.81600	-51.10100
H	-1.21500	0.55500	-50.54000
H	-2.55500	0.07900	-51.06100
O	-16.04100	2.67600	-42.73900
H	-15.12100	2.85400	-42.54500
H	-16.07600	1.73200	-42.89100
O	-18.17200	-3.84000	-40.70600
H	-18.27200	-2.89100	-40.77500
H	-18.39000	-4.16900	-41.57900
O	-18.30900	-0.41100	-41.89000
H	-18.79200	0.38900	-41.68300
H	-18.22300	-0.40000	-42.84300
O	-14.01300	3.45200	-41.08700
H	-13.14400	3.62600	-41.44900
H	-14.49200	4.27100	-41.21000
O	-5.90900	12.67400	-44.22200
H	-6.68600	13.23300	-44.23200
H	-5.27000	13.16000	-43.70000
O	-7.65400	15.48300	-44.66000
H	-6.91300	16.02100	-44.93700
H	-8.36100	16.10900	-44.50300
O	-8.91800	12.63600	-43.85900
H	-9.54800	13.31800	-43.62500
H	-9.02500	12.52500	-44.80300
O	-9.64000	6.93900	-41.49600
H	-10.03400	6.38500	-40.82100
H	-8.82400	7.25000	-41.10500
O	-6.72300	8.35400	-43.90400
H	-5.98300	8.95500	-43.98400
H	-6.49200	7.61200	-44.46200
O	-11.57900	13.54000	-45.62600
H	-11.69400	12.63900	-45.32600
H	-12.43500	13.78600	-45.97800
O	-2.77800	4.38200	-49.62800
H	-3.12100	5.27300	-49.70400
H	-2.41000	4.19300	-50.49100
O	-10.80100	11.51100	-48.69600
H	-10.12900	11.30200	-49.34500
H	-11.62800	11.31300	-49.13500
O	-4.89800	8.13700	-46.67900
H	-5.54600	8.83600	-46.77300
H	-4.12800	8.57000	-46.31200
O	-9.05700	11.48300	-46.62800
H	-9.59300	11.03200	-45.97600
H	-9.64500	11.61800	-47.37100
O	-13.32300	10.61900	-49.54200
H	-13.40700	10.01100	-50.27700
H	-14.14500	11.11000	-49.54100
O	-4.88900	15.00200	-43.04100
H	-5.56500	14.90200	-42.37100
H	-4.06700	14.84600	-42.57700
O	-11.17100	8.65600	-48.20300
H	-11.10700	9.51800	-48.61500
H	-11.23400	8.04400	-48.93600
O	-11.69500	10.72900	-44.55600
H	-11.06800	10.34600	-43.94200
H	-12.55300	10.51000	-44.19000
O	-8.58700	7.94700	-46.36700
H	-8.06300	8.19400	-45.60400
H	-8.80600	8.77900	-46.78700
O	-7.38200	8.88900	-48.68200
H	-7.72200	8.09000	-48.28000
H	-8.08800	9.18900	-49.25400
O	-6.73000	5.97400	-50.22900
H	-5.90700	5.50000	-50.35100
H	-6.48600	6.75300	-49.72900
O	-13.94500	14.33300	-46.58800
H	-14.68700	14.48100	-46.00200
H	-14.26600	14.59400	-47.45200
O	-8.45900	10.05100	-42.89400
H	-8.41000	10.92700	-43.27600
H	-7.91600	9.50800	-43.46700
O	-6.22900	10.80400	-46.14000
H	-7.09800	11.09700	-46.41100
H	-5.99300	11.38800	-45.41900
O	-3.52500	7.02200	-48.62300
H	-3.00700	7.75800	-48.94800
H	-3.87900	7.32800	-47.78800
O	-5.20200	18.00500	-42.73900
H	-4.28100	18.18300	-42.54500

H	-5.23700	17.06100	-42.89100
O	-7.33300	11.48900	-40.70600
H	-7.43300	12.43800	-40.77500
H	-7.55100	11.16100	-41.57900
O	-7.47000	14.91900	-41.89000
H	-7.95300	15.71900	-41.68300
H	-7.38300	14.92900	-42.84300
O	-15.69900	11.71300	-46.58700
H	-15.05300	11.20300	-46.09800
H	-15.90700	12.45000	-46.01200
O	-3.17300	18.78100	-41.08700
H	-2.30400	18.95500	-41.44900
H	-3.65300	19.60000	-41.21000
O	-11.35900	8.86000	-42.48900
H	-10.49600	8.50300	-42.27900
H	-11.93300	8.52100	-41.80200

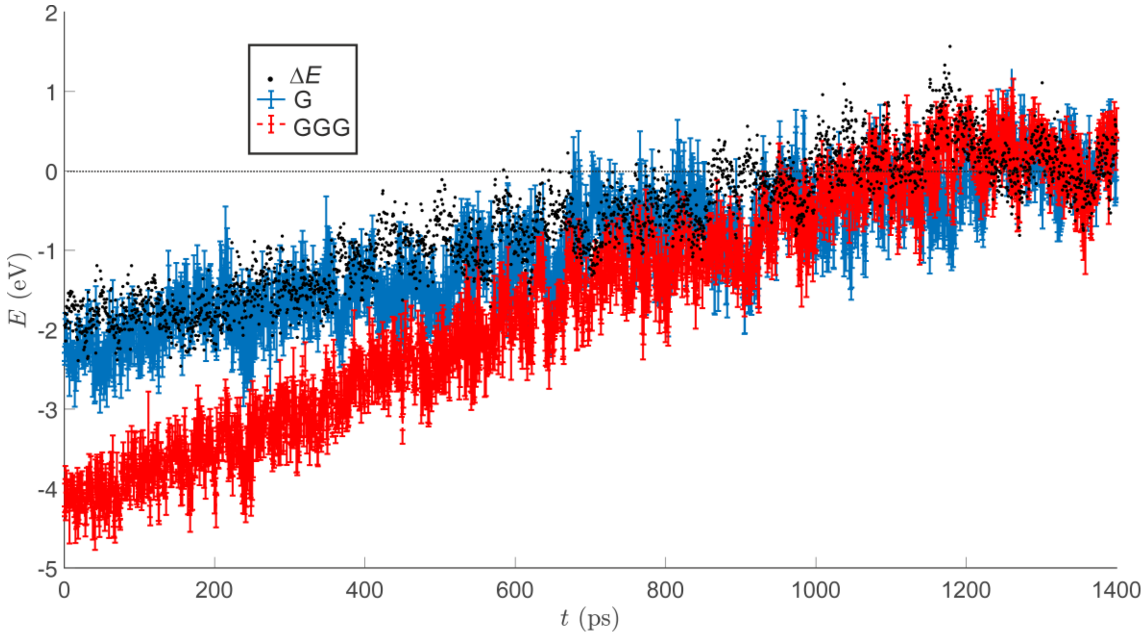

Figure S1. HOMO energy (eV) of 5′-GGG nucleobase stack (red dashed line) and G-3′ nucleobase (blue line) together with their difference (Δ*E*, black dots) for the 5′-GGGTG-3′ double strand immersed in a box consisting of ca. 15000 water molecules and including counterions. Average values over three simulations and are reported. The hole is initially (t = 0) localized on G. Statistical errors are reported as vertical bars.
